# Machine learning enabling prediction of the bond dissociation enthalpy of hypervalent iodine from SMILES

**DOI:** 10.1038/s41598-021-99369-8

**Published:** 2021-10-12

**Authors:** Masaya Nakajima, Tetsuhiro Nemoto

**Affiliations:** Graduate School of Pharmaceutical Sciences, Chiba University, Chiba, Japan

**Keywords:** Cheminformatics, Computational science, Energy

## Abstract

Machine learning to create models on the basis of big data enables predictions from new input data. Many tasks formerly performed by humans can now be achieved by machine learning algorithms in various fields, including scientific areas. Hypervalent iodine compounds (HVIs) have long been applied as useful reactive molecules. The bond dissociation enthalpy (BDE) value is an important indicator of reactivity and stability. Experimentally measuring the BDE value of HVIs is difficult, however, and the value has been estimated by quantum calculations, especially density functional theory (DFT) calculations. Although DFT calculations can access the BDE value with high accuracy, the process is highly time-consuming. Thus, we aimed to reduce the time for predicting the BDE by applying machine learning. We calculated the BDE of more than 1000 HVIs using DFT calculations, and performed machine learning. Converting SMILES strings to Avalon fingerprints and learning using a traditional Elastic Net made it possible to predict the BDE value with high accuracy. Furthermore, an applicability domain search revealed that the learning model could accurately predict the BDE even for uncovered inputs that were not completely included in the training data.

## Introduction

Organic chemistry enables the synthesis of various molecules by continuously breaking and forming molecular bonds. Bond dissociation enthalpy (BDE) is an indicator of the strength of a chemical bond and is an essential consideration in the design of chemical reactions and reactive molecules. Heat energy or light energy in adequate quantities can be used to break the chemical bond homolytically. Therefore, BDE is a commonly estimated on the basis of thermal measurement^1^, kinetics^2^, and electricity^3,4^. In recent years, advances in the development of computers, quantum chemistry, and density functional theory (DFT) calculations, have provided remarkably more accurate methodologies for predicting BDE^5–7^. In silico methods can be used to estimate the BDE, even for pinpoint chemical bonds of complicated molecules and imaginary molecules, enabling the design of reactive molecules and estimating the stability of functional molecules before they are synthesised. Even with advanced computer technology, however, the calculation costs of the DFT method remain enormous. The calculation time exponentially increases by the total number of electrons in a molecule. Therefore, obtaining the BDE values of hundreds or thousands of molecules at once by quantum computations remains challenging.

Hypervalent iodine (HVI), which bears over eight valence electrons on iodine, is a reactive molecule used as an oxidant or an alkylating agent in organic synthesis^8–15^. Heterolytic or homolytic cleavage of a weak, three-center four-electron (3c–4e) bond of HVI progresses the chemical reaction. Therefore, the BDE of the 3c–4e bond of HVI is an essential parameter that has been calculated by the DFT method on demand^16–19^. We previously reported the BDE value of 3c–4e bonds in various HVIs on the basis of DFT calculations^19^. We first determined the optimal functional and basis sets for reproducing the 3c–4e bond in silico and calculated a BDE value of 206 HVIs. While this database is helpful for chemists, it is still necessary to calculate the BDE for HVIs that are not available in the database.

**Figure 1:**
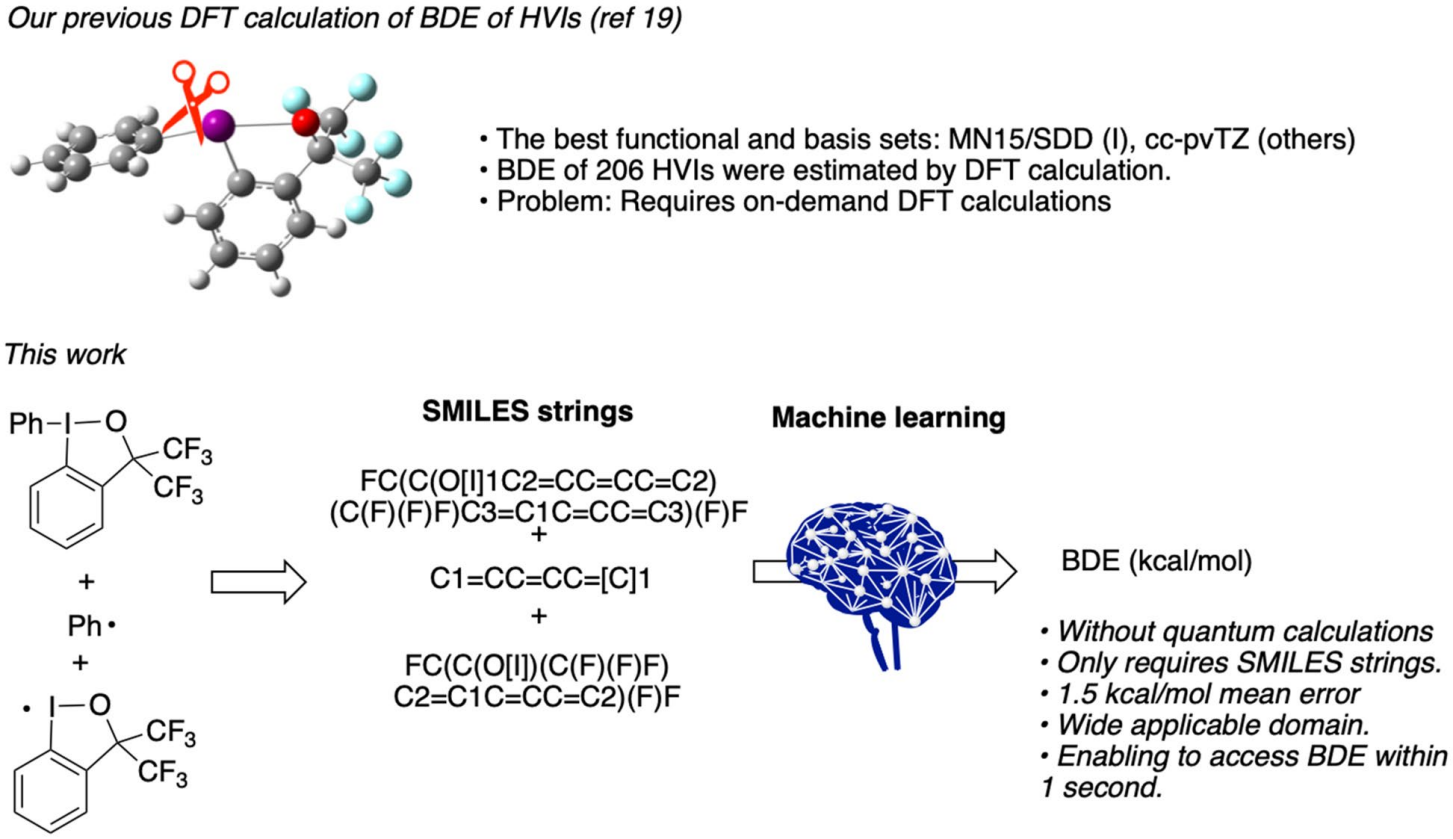
BDE calculations of HVIs using the DFT method (previous work) and machine learning (this work).

Machine learning is currently attracting attention worldwide, and analysing and learning from a population using statistical methods enables immediate prediction of the results from new inputs. In the field of organic chemistry, machine learning is applied for predicting synthetic pathways and reactivity, and optimising reaction conditions^20–26^. Yu and co-workers reported the prediction model of BDE of carbonyl groups with machine learning in 2020^27^. Their important model accurately predicts the BDE value of the C=O bond on the basis of the bond length and bond angle of the relevant site as inputs. Three-dimensional molecular information is required for the input data, however, and thus time-consuming DFT calculations are inescapable. We considered that an ideal and highly useful method of BDE prediction for chemists should not require quantum computations to prepare the input data. Therefore, we decided to use only structural formula information, such as SMILES strings, to predict the BDE value of HVIs by machine learning (Fig. 1).

## Methods

We first performed DFT calculations to increase the sizes of the data set populations. The DFT calculations were performed using Gaussian16 with MN15^28^ functional and SDD^29,30^ (for I and Se) and cc-pvTZ^31^ (for the others) basis sets^19^. Structure optimizations were carried out with an ultrafine grid at 298.15 K in gas phase. Harmonic vibrational frequencies were computed at the same level of theory to confirm no imaginary vibration was observed for the optimised structure. BDE was calculated from the enthalpy (*H*) of each species at 298 K according to the following formula:

$$BDE=y={H}_{radical A}^{298}+ {H}_{radical B}^{298}- {H}_{AB}^{298}$$

**Figure 2:**
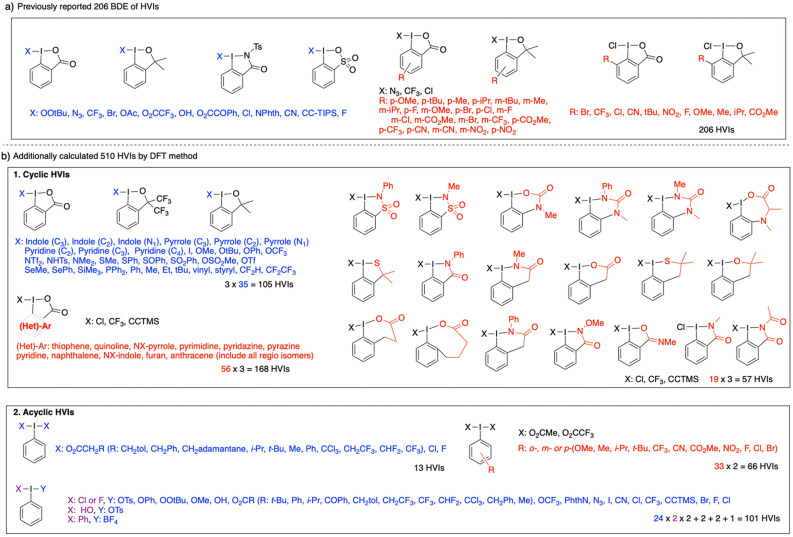
Structures of 716 HVIs for the training and test data sets: **(a)** previously calculated cyclic HVIs, **(b)** additionally calculated 510 HVIs.

In addition to the BDE data of 206 HVIs, which we reported previously, we newly calculated 510 HVIs by DFT calculations (Fig. 2). Various combinations of iodine-containing backbones and leaving groups were calculated to increase the diversity of the data sets. A total of 330 cyclic HVIs were calculated: 105 molecules with 35 types of leaving groups and three common HVI skeletons for cyclic HVIs, and 225 molecules with 75 types of cyclic HVI skeletons and 3 common leaving groups. In addition, 167 acyclic HVIs were calculated: 13 types of symmetric HVIs, 101 types of asymmetric HVIs, and 66 types of HVIs with 33 HVI skeletons and 2 leaving groups. The 716 types of HVIs were randomly divided into 75% training and 25% test data sets. The training data set was first subjected to a grid search by k-partition cross-validation in each machine learning iterative process to optimise the hyperparameters (see supplementary information for details). For machine learning, three types of structural formulas were converted to SMILES: HVI (neutral), leaving group (radical), and HVI skeleton (radical). Then, fingerprints were generated using an RDkit (version 2019.09.3)^32^: Morgan^33^ (Circular, r = 2, 3 or 4), Topological (RDKFingerprint)^32^, MACCS^34^, and Avalon^35^. In each fingerprint, learning from the training data set was performed with optimised hyperparameters using Elastic Net (EN)^36^, support vector (SVR)^37^, Neural Network (NN)^38^, Random Forest (RF)^39^, and LightGBM (LGBM)^40^. The accuracy of the BDE prediction was evaluated by comparison with the test data set. Mean absolute error (MAE) and coefficient of determination (R^2^) were used to evaluate the prediction accuracy of the BDE.

$$\mathrm{MAE}=\frac{1}{n}\sum_{i=1}^{n}|{yi}_{DFT}-{yi}_{ML}|$$

$${R}^{2}=1-\frac{\sum_{i=1}^{n}{({yi}_{DFT}-{yi}_{ML})}^{2}}{\sum_{i=1}^{n}{({yi}_{DFT}-{yi}_{average\_DFT})}^{2}}$$

The training and testing were performed 10 times (random state = 0–9), and accuracy was evaluated by the average.

## Results and discussion

**Figure 3:**
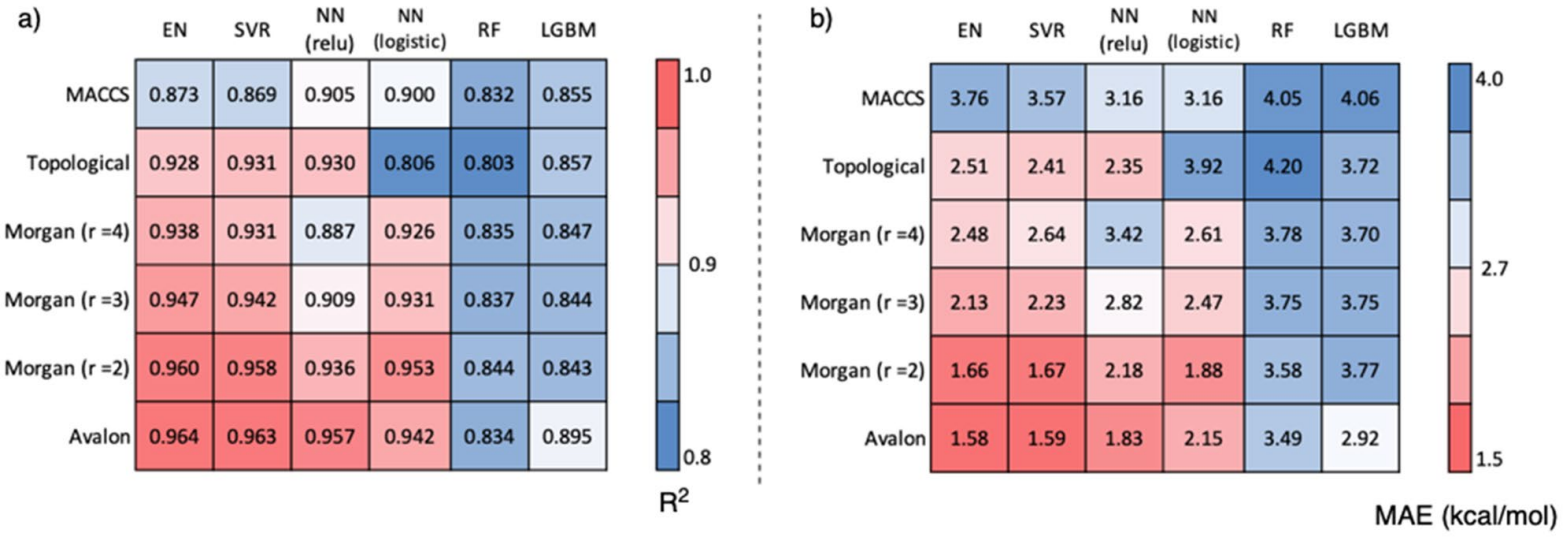
Heat map of accuracy of the prediction by various machine learning algorithm and fingerprints.

As a result of the Grid search, we used both the "relu" and "logistic" evaluation functions for NN (see supplementary information for the detailed grid search results). The Avalon fingerprint, which features various factors such as atoms, bonds, and ring information, enables highly accurate prediction with an R^2^ = 0.964 (Fig. 3a) and MAE = 1.58 kcal/mol (Fig. 3b) by EN, which was the best score. SVR and NNs also gave high scores. In the Morgan fingerprint, which considers each atom's neighbourhood, the increasing number of recognised atoms gave a lower accuracy, and r = 2 (recognising first and second neighbour atoms) with the EN method giving the highest accuracy, similar to Avalon. The Topological fingerprint, which considers atoms and bond types, gave a high R^2^ of 0.931 and a small MAE of 2.41 kcal/mol using the SVR method; however, it was inferior to the Avalon and Morgan fingerprints. The MACCS fingerprint, which counts 166 specific substructures, yielded the worst results. Although it gave an R^2^ of 0.905 and an MAE of 3.16 kcal/mol by the NN (relu) method, the errors were small and acceptable. EN and SVR tended to give good results except for the MACCS fingerprint; on the other hand, RF and LGBM, which are decision-tree learning models, predicted BDE with low accuracy in all fingerprints.

**Figure 4:**
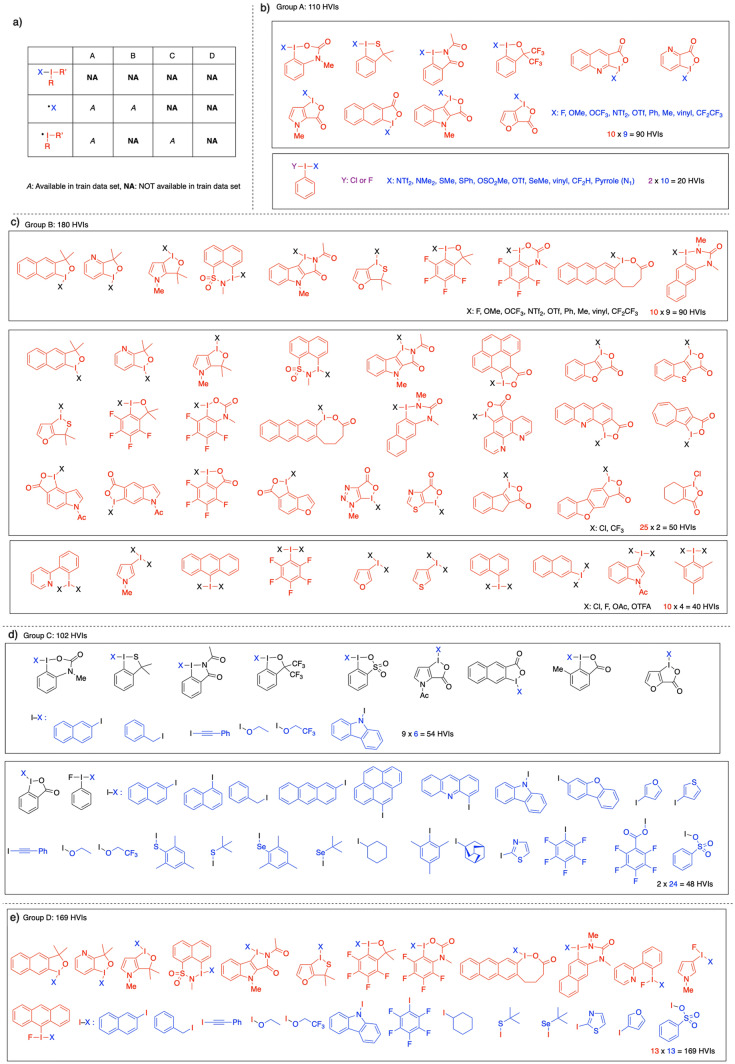
Structures of newly calculated 561 HVIs for AD: **(a)** table for the explanation of group A–D, **(b)** 110 HVIs in group A, **(c)** 180 HVIs in group B, **(d)** 102 HVIs in group C, **(e)** 169 HVIs in group D.

Next, we investigated the applicability domain (AD) of these machine learning models^41^. Verifying the AD of the learning model is essential for examining the overfitting of training and the applicable range of uncovered inputs. For the AD search, the BDE of 561 HVIs was newly calculated by DFT calculations and classified into four groups: group A in which the leaving group and the HVI skeleton were individually included in the training data, group B in which the leaving group was included and the HVI skeleton was not included in the training data, group C in which the leaving group was not included and HVI skeleton was included in the training data, and group D in which neither the leaving group nor the HVI skeleton was included in the training data (Fig. 4). All HVIs shown in Fig. 2 were used as training data, and learning by the decision tree, which was an inappropriate learning model, was excluded.

**Figure 5:**
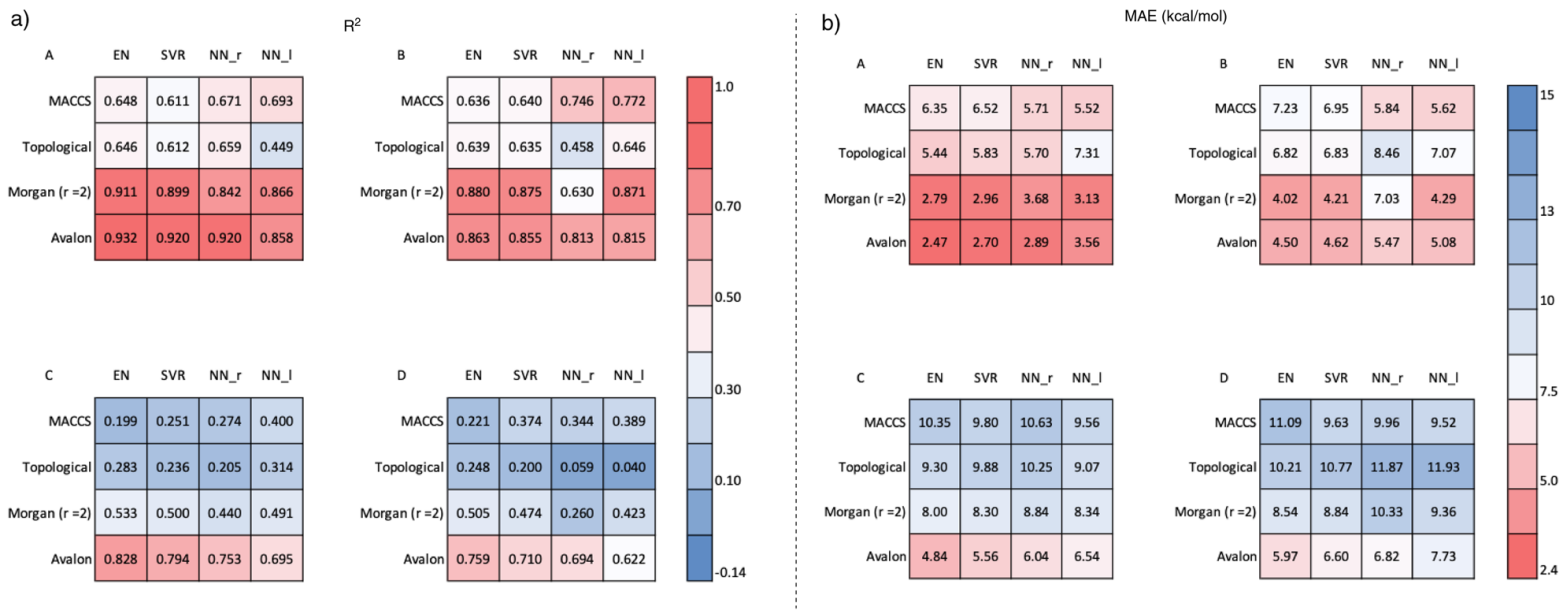
**(a)** Heat maps of the result (R^2^) of AD. **(b)** Heat maps of the result (MAE) of AD.

**Figure 6:**
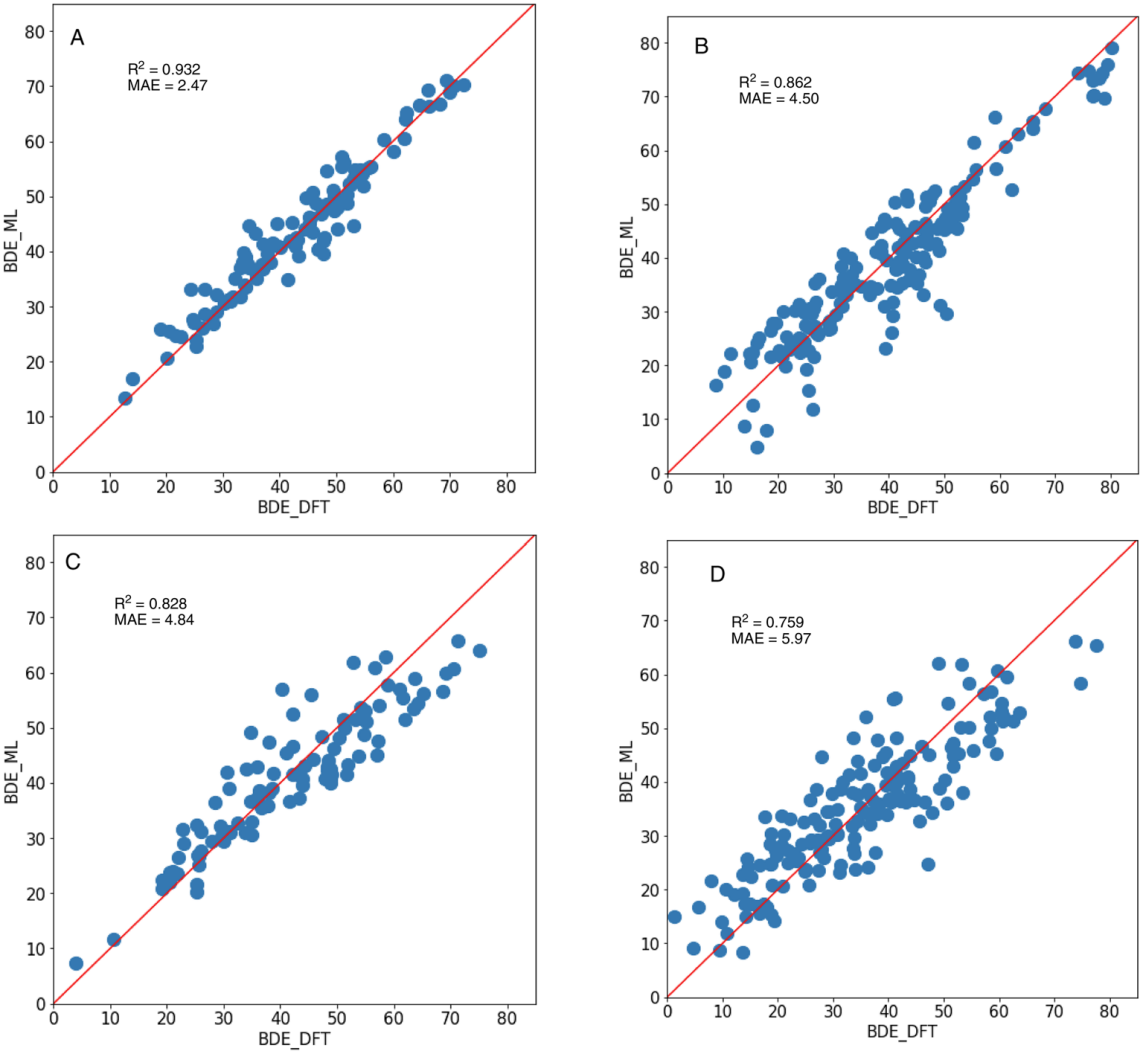
Correlation plots of the prediction by EN with Avalon fingerprint.

The investigation of AD with group A (Fig. 5aA,bA) demonstrated that the Avalon fingerprint maintained high accuracy, that is, R^2^ = 0.932 and MAE = 2.47 kcal/mol with the EN method (Fig. 6A). SVR and NN_r also gave R^2^ = 0.920, 0.920 and MAE = 2.70, 2.89 kcal/mol, respectively. The Morgan (r = 2) fingerprint had a slightly lower accuracy with R^2^ = 0.911 and MAE = 2.79 kcal/mol with the EN method. On the other hand, in the Topological and MACCS fingerprints, the R^2^ value was lower than 0.7 and the minimum MAE was 5.44 kcal/mol (Topological, EN), indicating a significant decrease in accuracy from the test data in Fig. 3. Therefore, overfitting of the training data occurred in the Topological and MACCS fingerprint. With the molecules of group B (Fig. 5aB,bB), which contains new HVI skeletons, the accuracy was slightly decreased but the R^2^ value of the Avalon (Fig. 6B) and Morgan (r = 2) fingerprints maintained a high accuracy of 0.880 and 0.863, respectively. In group C (Fig. 5aC,bC), which contains new leaving groups, the Avalon fingerprint could still predict with adequate accuracy with R^2^ = 0.828 with the EN method (Fig. 6C). The Morgan (r = 2) fingerprint predicted the BDE value with R^2^ = 0.532 and MAE = 8.00 kcal/mol, which are much lower than the values in groups A and B, indicating that prediction with the uncovered leaving groups was not applicable. We considered that because HVI skeletons contain R–I–R' bonds, the Morgan fingerprint could well recognise the pattern of the structure; however, the leaving groups were difficult to learn accurately because of the divergent structures. Finally, we verified the AD of group D (Fig. 5aD,bD), a completely new data set, and revealed that the Avalon fingerprint predicted the BDE value with R^2^ = 0.759 and MAE = 5.97 kcal/mol (Fig. 6D). Because the Avalon fingerprint considers a larger variety of features and/or generates the fingerprint with a larger number of bits than MACCS, topological or Morgan, it was possible to appropriately evaluate the similarity of molecules and predict uncovered data with higher accuracy than other fingerprints.

We finally compared the computation time of the DFT method and machine learning method to calculate the BDE value of 561 HVIs of group A-D. In our computational environment, the DFT method required 4272 days (time converted to per core), i.e., 12 years; on the other hand, machine learning completed the 561 predictions from SMILES strings within 3 s, an overwhelming difference in speed.

## Conclusions

We constructed a BDE prediction model for HVIs from SMILES strings using machine learning, which does not require quantum computations for input data. Avalon fingerprint generation and Elastic Net machine learning made it possible to predict BDE with high accuracy and an MAE of 1.58 kcal/mol. This model exhibited a high applicable range that can be predicted with an MAE of 5.97 kcal/mol, even for completely uncovered inputs. With this model, it is possible to access the predicted value of BDE for HVIs at a remarkable speed compared with modern quantum calculations. We anticipate that machine learning will be carried out by many organic chemists to facilitate the molecular design and reaction design of HVI.

## Supplementary Information

Supplementary Information.

## Data Availability

Computational details including the results of grid search, geometry and energy of HVIs by DFT, and the list of SMILES and the value of BDE_DFT_ are provided in Supplementary Information.
