# Supplementary material for: Machine learning enabling prediction of the bond dissociation enthalpy of hypervalent iodine from SMILES
Source: Sci Rep. 2021 Oct 12;11:20207. doi: 10.1038/s41598-021-99369-8 (PMC8511102; doi:10.1038/s41598-021-99369-8)
Supplement: Supplementary file 1 — Supplementary Information. [file 41598_2021_99369_MOESM1_ESM.pdf]

Supplementary Material

Machine Learning Enabling Prediction of the Bond Dissociation Enthalpy of Hypervalent Iodine from SMILES

Masaya Nakajima*, Tetsuhiro Nemoto*

Graduate School of Pharmaceutical Sciences, Chiba University
e-mail: m.nakajima@chiba-u.jp (MN), tnemoto@faculty.chiba-u.jp (TN)

Table of Contents

1. Computational details………………………………………………………………………….. 3
2. Result of grid search…………………………………………………………..…………….. 6
3. Results of analysis using RMSE…………………………………….………………..…………….. 83
4. List of SMILES and BDE_{DFT} …………………………………………………………….……84
5. Required time for DFT calculations of HVIs in Fig4. ……………………………………………..126
6. Cartesian Coordinates and Energies ……………………………………………………..………137

1. Computational details

We first performed DFT calculations to increase the sizes of the data set populations. The DFT calculations were performed using Gaussian16 with MN15 functional and SDD (for I and Se) and cc-pvTZ (for the others) basis sets. Structure optimizations were carried out with an ultrafine grid at 298.15 K in gas phase. Harmonic vibrational frequencies were computed at the same level of theory to confirm no imaginary vibration was observed for the optimised structure. BDE was calculated from the enthalpy (H) of each species at 298 K according to the following formula:

$$BDE = y = H_{radical\ A}^{298} + H_{radical\ B}^{298} - H_{AB}^{298}$$

The 716 types of HVIs were randomly divided into 75% training and 25% test data sets. The training data set was first subjected to a grid search by k-partition cross-validation in each machine learning iterative process to optimise the hyperparameters (see supplementary information for details). For machine learning, three types of structural formulas were converted to SMILES: HVI (neutral), leaving group (radical), and HVI skeleton (radical). Then, fingerprints were generated using an RDkit (version 2019.09.3): Morgan (Circular, r = 2, 3 or 4), Topological (RDKFingerprint), MACCS, and Avalon. In each fingerprint, learning from the training data set was performed with optimised hyperparameters using Elastic Net (EN), support vector (SVR), Neural Network (NN), Random Forest (RF), and LightGBM (LGBM). The accuracy of the BDE prediction was evaluated by comparison with the test data set. Mean absolute error (MAE) and coefficient of determination (R^2) were used to evaluate the prediction accuracy of the BDE.

$$\text{MAE} = \frac{1}{n}\sum_{i=1}^{n} |yi_{DFT} - yi_{ML}|$$

$$R^2 = 1 - \frac{\sum_{i=1}^{n}(yi_{DFT} - yi_{ML})^2}{\sum_{i=1}^{n}(yi_{DFT} - yi_{average\_DFT})^2}$$

The training and testing were performed 10 times (random state = 0-9), and accuracy was evaluated by the average.

The optimised hyperparameters for each machine learning algorithms are below:

Optimised hyperparameters of EN
ElasticNet(alpha= i, l1_ratio= j, max_iter=100000)
Fingerprint: i j
Avalon (bit =1024): 0.001 0.6
Avalon (bit = 2048): 0.001 0.4
Morgan (r = 2, bit = 1024): 0.001 0.6
Morgan (r = 2, bit = 2048): 0.001 0.4
Morgan (r = 3, bit = 1024): 0.001 1.0
Morgan (r = 3, bit = 2048): 0.001 0.6
Morgan (r = 4, bit = 1024): 0.001 0.8
Morgan (r = 4, bit = 2048): 0.001 0.6
Topological: 0.01 0.2
MACCS: 0.01 1.0

Optimised hyperparameters of RF
RandomForestRegressor(n_estimators= i)
Fingerprint: i

Avalon (bit =1024): 500
Avalon (bit = 2048): 1000
Morgan (r = 2, bit = 1024): 2000
Morgan (r = 2, bit = 2048): 1000
Morgan (r = 3, bit = 1024): 1000
Morgan (r = 3, bit = 2048): 2000
Morgan (r = 4, bit = 1024): 500
Morgan (r = 4, bit = 2048): 1000
Topological: 500
MACCS: 500

Optimised hyperparameters of NN (relu)

```
MLPRegressor(activation= “relu”, alpha=a, batch_size=batch, beta_1=0.9,
        beta_2=0.999, early_stopping=False, epsilon=1e-08,
        hidden_layer_sizes=hid, learning_rate='constant',
        learning_rate_init=0.001, max_iter=100000, momentum=0.9,
        n_iter_no_change=10, nesterovs_momentum=True, power_t=0.5,
        random_state=1, shuffle=True, solver='adam', tol=0.0001,
        validation_fraction=0.1, verbose=False, warm_start=False)
```

Fingerprint: (hid) a batch
Avalon (bit =1024): (50,) 0.001 50
Avalon (bit = 2048): (50,) 0.01 50
Morgan (r = 2, bit = 1024): (200,) 0.01 50
Morgan (r = 2, bit = 2048): (50, 50) 0.001 50
Morgan (r = 3, bit = 1024): (200,) 0.01 50
Morgan (r = 3, bit = 2048): (50,) 0.01 50
Morgan (r = 4, bit = 1024): (100, 200) 0.01 50
Morgan (r = 4, bit = 2048): (50,) 0.01 50
Topological: (50,) 0.0001 100
MACCS: (100,) 0.001 100

Optimised hyperparameters of NN (logistic)

```
MLPRegressor(activation=” logistic”, alpha=a, batch_size=batch, beta_1=0.9,
        beta_2=0.999, early_stopping=False, epsilon=1e-08,
        hidden_layer_sizes=hid, learning_rate='constant',
        learning_rate_init=0.001, max_iter=100000, momentum=0.9,
        n_iter_no_change=10, nesterovs_momentum=True, power_t=0.5,
        random_state=1, shuffle=True, solver='adam', tol=0.0001,
        validation_fraction=0.1, verbose=False, warm_start=False)
```

Fingerprint: (hid) a batch
Avalon (bit =1024): (200,) 0.0001 50
Avalon (bit = 2048): (200,) 0.0001 50
Morgan (r = 2, bit = 1024): (200,) 0.01 50
Morgan (r = 2, bit = 2048): (200,) 0.01 50
Morgan (r = 3, bit = 1024): (200,) 0.01 50
Morgan (r = 3, bit = 2048): (200,) 0.01 50
Morgan (r = 4, bit = 1024): (200, 200) 0.01 50
Morgan (r = 4, bit = 2048): (100,) 0.01 50
Topological: (200, 200) 0.0001 500
MACCS: (200,) 0.0001 200

Optimised hyperparameters of LGBM
lgb.LGBMRegressor(boosting_type = "gbdt", num_leaves = j,max_depth = 0)
Fingerprint: j
Avalon (bit =1024): 10
Avalon (bit = 2048): 10
Morgan (r = 2, bit = 1024): 50
Morgan (r = 2, bit = 2048): 50
Morgan (r = 3, bit = 1024): 50
Morgan (r = 3, bit = 2048): 50
Morgan (r = 4, bit = 1024): 10
Morgan (r = 4, bit = 2048): 50
Topological: 10
MACCS: 50

Optimised hyperparameters of SVR
SVR(C= c_num, kernel = ker, epsilon = e, gamma = r,degree = 3, coef0=1)
Fingerprint: ker c_num r e
Avalon (bit =1024): linear 2 5 0.1
Avalon (bit = 2048): linear 3 1 0.3
Morgan (r = 2, bit = 1024): linear 4 1 0.3
Morgan (r = 2, bit = 2048): linear 4 2 0.3
Morgan (r = 3, bit = 1024): linear 4 1 0.3
Morgan (r = 3, bit = 2048): linear 4 2 0.1
Morgan (r = 4, bit = 1024): linear 4 1 0.1
Morgan (r = 4, bit = 2048): linear 4 1 0.1
Topological: linear 2 5 0.1
MACCS: linear 4 1 0.9

All the results of grid search are listed in next section.

2. Result of grid search

Grid search of Elastic Net, Avalon (1024)
ElasticNet(alpha= i, l1_ratio= j, max_iter=100000)
i j : accuracy of prediction using cross validation, accuracy of prediction using test data
0.001 0.0 : 0.9492206814385513, 0.965622621670798
0.001 0.2 : 0.95069294974515, 0.9660707528939749
0.001 0.4 : 0.951291521495224, 0.9664414195667099
0.001 0.6 : 0.95155352439016, 0.9646045756866058
0.001 0.8 : 0.9494247407525961, 0.9622507766262822
0.001 1.0 : 0.9394165393979144, 0.9582072007084996
0.01 0.0 : 0.9374509435293599, 0.9569428967711846
0.01 0.2 : 0.938674161453679, 0.9564125954066365
0.01 0.4 : 0.9383448528814341, 0.9561353256581122
0.01 0.6 : 0.9382465952053088, 0.9557375065051864
0.01 0.8 : 0.9378007559162047, 0.9539215199297691
0.01 1.0 : 0.9322602101646718, 0.9457087086878035
0.1 0.0 : 0.8726575629244536, 0.8898311893386924
0.1 0.2 : 0.863091550778365, 0.8813108049845005
0.1 0.4 : 0.8567381226134302, 0.8760654456907615
0.1 0.6 : 0.852344245268547, 0.87505639385109
0.1 0.8 : 0.8503962455839627, 0.8773192662562947
0.1 1.0 : 0.8545198056288484, 0.87122064276594

Grid search of Random Forest, Avalon (1024)
RandomForestRegressor(n_estimators= i)
i: accuracy of prediction using cross validation, accuracy of prediction using test data
100 : 0.7872392704078174, 0.8363601532242905
500 : 0.7878301976043577, 0.8378173067117023
1000 : 0.787055932193015, 0.836051199113846
2000 : 0.7869089153995766, 0.8360637239888055

Grid search of NeuralNetwork, Avalon (1024)
MLPRegressor(activation=act, alpha=a, batch_size=batch, beta_1=0.9,
beta_2=0.999, early_stopping=False, epsilon=1e-08,
hidden_layer_sizes=hid, learning_rate='constant',
learning_rate_init=0.001, max_iter=100000, momentum=0.9,
n_iter_no_change=10, nesterovs_momentum=True,
power_t=0.5,
random_state=1, shuffle=True, solver='adam', tol=0.0001,
validation_fraction=0.1, verbose=False, warm_start=False)
act (hid) a batch: accuracy of prediction using cross validation, accuracy of prediction using test data
relu (200,) 0.0001 500 : 0.9331321564916385, 0.9620923423579254
relu (200,) 0.0001 200 : 0.9315462081821607, 0.96930887404449
relu (200,) 0.0001 100 : 0.9346214129953985, 0.9681554478553779
relu (200,) 0.0001 50 : 0.9359959562543576, 0.9701727898079706
relu (200,) 0.001 500 : 0.9320428145619948, 0.9619420433051797
relu (200,) 0.001 200 : 0.9318581214938797, 0.9668656702728221
relu (200,) 0.001 100 : 0.9341230202548975, 0.9682121590650477
relu (200,) 0.001 50 : 0.935266314753974, 0.970225525705651
relu (200,) 0.01 500 : 0.9327722313333957, 0.9619931176387911
relu (200,) 0.01 200 : 0.9318812440390769, 0.9691365053908245
relu (200,) 0.01 100 : 0.9344530092458999, 0.9681972115662775
relu (200,) 0.01 50 : 0.9344141579518279, 0.970443816114845
relu (200, 200) 0.0001 500 : 0.9221139337554256, 0.9648042943601438
relu (200, 200) 0.0001 200 : 0.9226867909321597, 0.9624771684220249
relu (200, 200) 0.0001 100 : 0.9224723890652428, 0.9621146821911236
relu (200, 200) 0.0001 50 : 0.9246516614049695, 0.9620001488506718
relu (200, 200) 0.001 500 : 0.9218240903009608, 0.9649702045710327
relu (200, 200) 0.001 200 : 0.9222546355413851, 0.9632497433997016
relu (200, 200) 0.001 100 : 0.922537267396701, 0.9620799263451829
relu (200, 200) 0.001 50 : 0.9248354130731871, 0.9628629433253305
relu (200, 200) 0.01 500 : 0.9201626289342204, 0.9643909937450671
relu (200, 200) 0.01 200 : 0.9210223668555064, 0.9641978398102864
relu (200, 200) 0.01 100 : 0.9205660165163569, 0.9615838416612137
relu (200, 200) 0.01 50 : 0.9238672719322416, 0.9622355230351181
relu (100,) 0.0001 500 : 0.9351376905757242, 0.9667733847906086
relu (100,) 0.0001 200 : 0.9342190046612714, 0.9707455502486992
relu (100,) 0.0001 100 : 0.9360436908736179, 0.9693678785400588
relu (100,) 0.0001 50 : 0.93611414434446, 0.9697313726372842
relu (100,) 0.001 500 : 0.9351417880538486, 0.9639530560803062
relu (100,) 0.001 200 : 0.9342952474493285, 0.9707084867028175
relu (100,) 0.001 100 : 0.9360567605907553, 0.9693817376725684
relu (100,) 0.001 50 : 0.9362444687386955, 0.9694412852277712
relu (100,) 0.01 500 : 0.9352531381425587, 0.9639039644197194
relu (100,) 0.01 200 : 0.9344522105355273, 0.9706672760101553
relu (100,) 0.01 100 : 0.9362489591194141, 0.9693525775017052
relu (100,) 0.01 50 : 0.9364942295838652, 0.9688945895163711
relu (100, 100) 0.0001 500 : 0.9203399210251202, 0.9647465194082654
relu (100, 100) 0.0001 200 : 0.9231584847992433, 0.9616335065575117
relu (100, 100) 0.0001 100 : 0.9296573540875872, 0.9647135804899699
relu (100, 100) 0.0001 50 : 0.9263977212081971, 0.9647949484479788
relu (100, 100) 0.001 500 : 0.9201594744875328, 0.9654576423333437
relu (100, 100) 0.001 200 : 0.924585440621495, 0.9615087699086652
relu (100, 100) 0.001 100 : 0.9280915964261947, 0.962737502195356
relu (100, 100) 0.001 50 : 0.9238527832918741, 0.9607372504577055
relu (100, 100) 0.01 500 : 0.920156166875785, 0.9633609492972004
relu (100, 100) 0.01 200 : 0.9250713292423038, 0.9618054423201339
relu (100, 100) 0.01 100 : 0.9289251969922642, 0.9631440583619255
relu (100, 100) 0.01 50 : 0.9275927122069347, 0.9616393598448576
relu (100, 200) 0.0001 500 : 0.9162878643684842, 0.9642704107021587
relu (100, 200) 0.0001 200 : 0.9260397504661491, 0.9641676974089024
relu (100, 200) 0.0001 100 : 0.9228484372716597, 0.9653734667282879
relu (100, 200) 0.0001 50 : 0.9236308292137723, 0.9644775821910152
relu (100, 200) 0.001 500 : 0.9167254852958248, 0.9648161840276986
relu (100, 200) 0.001 200 : 0.925362435817488, 0.9628962406544315
relu (100, 200) 0.001 100 : 0.9207093106375254, 0.9649744854467548
relu (100, 200) 0.001 50 : 0.9251683571135392, 0.9623570047065537
relu (100, 200) 0.01 500 : 0.9149317301286717, 0.964966592027985
relu (100, 200) 0.01 200 : 0.923667383689927, 0.9648151042125153
relu (100, 200) 0.01 100 : 0.9200665410159556, 0.9656868483698346
relu (100, 200) 0.01 50 : 0.9255280219421073, 0.9606024937120755
relu (50,) 0.0001 500 : 0.9346539862701471, 0.9623974198270464
relu (50,) 0.0001 200 : 0.9340358781186282, 0.9690171902318527
relu (50,) 0.0001 100 : 0.9354999717503215, 0.968407859748955
relu (50,) 0.0001 50 : 0.9377715302387983, 0.9688125220598868
relu (50,) 0.001 500 : 0.9345744766550708, 0.9624112432173125
relu (50,) 0.001 200 : 0.9344006717077651, 0.969021283756785
relu (50,) 0.001 100 : 0.935487856982365, 0.968435048600343
relu (50,) 0.001 50 : 0.9378863796092626, 0.9673652320569974
relu (50,) 0.01 500 : 0.9347493372489051, 0.9624015135412662

relu (50,) 0.01 200 : 0.9343067960729711, 0.9690803284343936
relu (50,) 0.01 100 : 0.9362267756178808, 0.9684438248874433
relu (50,) 0.01 50 : 0.9377009316308804, 0.9673212011489594
relu (50, 50) 0.0001 500 : 0.9213598953203942,
0.9557159751537738
relu (50, 50) 0.0001 200 : 0.9291262500508347,
0.9593657208571064
relu (50, 50) 0.0001 100 : 0.930588016347435, 0.9613041072249776
relu (50, 50) 0.0001 50 : 0.9346301621200566, 0.9614598092443791
relu (50, 50) 0.001 500 : 0.9239533440970256, 0.9645107276638034
relu (50, 50) 0.001 200 : 0.9309795249163308, 0.9601280072724452
relu (50, 50) 0.001 100 : 0.9268950140838493, 0.9626485848816972
relu (50, 50) 0.001 50 : 0.9333217560696612, 0.9622675195357769
relu (50, 50) 0.01 500 : 0.924692215920168, 0.9622838201092353
relu (50, 50) 0.01 200 : 0.9300508695371577, 0.9627702301918125
relu (50, 50) 0.01 100 : 0.9286638722394625, 0.9580704047416441
relu (50, 50) 0.01 50 : 0.9299255267218063, 0.9643451327135373
relu (50, 100) 0.0001 500 : 0.925603187073665,
0.9641310867470595
relu (50, 100) 0.0001 200 : 0.9299997727678276,
0.9616367285101902
relu (50, 100) 0.0001 100 : 0.9282114846883314,
0.9600367165686621
relu (50, 100) 0.0001 50 : 0.9322748449183917,
0.9683849589917225
relu (50, 100) 0.001 500 : 0.9267897079518239,
0.9613861106092202
relu (50, 100) 0.001 200 : 0.9275454958339899,
0.9585877110272867
relu (50, 100) 0.001 100 : 0.9286725781077013,
0.9588340462121961
relu (50, 100) 0.001 50 : 0.9298121185230542, 0.9684765749080635
relu (50, 100) 0.01 500 : 0.9257459840104822, 0.9637972409942819
relu (50, 100) 0.01 200 : 0.9283271275685507, 0.9636569078202056
relu (50, 100) 0.01 100 : 0.9263281294007019, 0.9600041674209019
relu (50, 100) 0.01 50 : 0.9296772376379598, 0.9669305497586184
relu (50, 200) 0.0001 500 : 0.9200637499127804,
0.9668003914817264
relu (50, 200) 0.0001 200 : 0.9279473872427866,
0.9660573939379469
relu (50, 200) 0.0001 100 : 0.9302871580288807,
0.9601180325355579
relu (50, 200) 0.0001 50 : 0.9290797315628392,
0.9654535992019753
relu (50, 200) 0.001 500 : 0.9217274950447862,
0.9649775171363957
relu (50, 200) 0.001 200 : 0.9249205802635447,
0.9680880017912187
relu (50, 200) 0.001 100 : 0.9311859864603667,
0.9647461481809337
relu (50, 200) 0.001 50 : 0.9304721601145713, 0.9682828313163037
relu (50, 200) 0.01 500 : 0.92131317414958, 0.9537800024332497
relu (50, 200) 0.01 200 : 0.9284058093736505, 0.9673972966730343
relu (50, 200) 0.01 100 : 0.9297656084865646, 0.9646697398858385
relu (50, 200) 0.01 50 : 0.9305927858135654, 0.968509372951265
tanh (200,) 0.0001 500 : 0.8894746163147313, -
0.013001292546483745
tanh (200,) 0.0001 200 : 0.8932488248831646, 0.9420173451113552
tanh (200,) 0.0001 100 : 0.8919767764640729, 0.940556138726507
tanh (200,) 0.0001 50 : 0.9012021856224054, 0.9449069383991623
tanh (200,) 0.001 500 : 0.88852945649882, -0.013001463165789984
tanh (200,) 0.001 200 : 0.8919181064856861, 0.9405822832416781
tanh (200,) 0.001 100 : 0.8951815000346007, 0.945394496367892
tanh (200,) 0.001 50 : 0.9018440779834563, 0.9488639070395467
tanh (200,) 0.01 500 : 0.8879260509928176, -0.013003243004274578
tanh (200,) 0.01 200 : 0.8898195654661791, 0.9366055330498829
tanh (200,) 0.01 100 : 0.8883022636737925, 0.9429021814656143
tanh (200,) 0.01 50 : 0.889403219806997, 0.9484230981392318
tanh (200, 200) 0.0001 500 : 0.16702495829924788, -
0.021245937413543192
tanh (200, 200) 0.0001 200 : -0.008952251875581841,
0.9419128898499268
tanh (200, 200) 0.0001 100 : 0.36691811564552346, -
0.017317189946182365
tanh (200, 200) 0.0001 50 : 0.9117577062893322,
0.9555401449170515
tanh (200, 200) 0.001 500 : 0.1677729204132361, -
0.021246046652947825
tanh (200, 200) 0.001 200 : -0.008952377295175794,
0.945340178287444
tanh (200, 200) 0.001 100 : 0.36460392714573575, -
0.017317219816037177
tanh (200, 200) 0.001 50 : 0.9150589570462879,
0.9477496211333049
tanh (200, 200) 0.01 500 : 0.17095382228126635, -
0.02124713879849871
tanh (200, 200) 0.01 200 : -0.008953657116666491,
0.9506961875631356
tanh (200, 200) 0.01 100 : 0.36855698782095864, -
0.017317966146415564
tanh (200, 200) 0.01 50 : 0.9117318511636385, 0.9482397243070921
tanh (100,) 0.0001 500 : 0.8873238880416453, 0.9318303153521522
tanh (100,) 0.0001 200 : 0.8950723145369889, 0.9368120665949564
tanh (100,) 0.0001 100 : 0.8961913939001823, 0.9453595255474642
tanh (100,) 0.0001 50 : 0.8948600143668285, 0.9363817069001497
tanh (100,) 0.001 500 : 0.8855018674441666, 0.9417585760668211
tanh (100,) 0.001 200 : 0.8912097624796858, 0.9332660819813456
tanh (100,) 0.001 100 : 0.8954517398877107, 0.937673509289842
tanh (100,) 0.001 50 : 0.8948324182932312, 0.9425043152430091
tanh (100,) 0.01 500 : 0.8901056957697933, 0.9397829365401842
tanh (100,) 0.01 200 : 0.8899440619377492, 0.9406355401187015
tanh (100,) 0.01 100 : 0.8926451209282844, 0.9397706732643819
tanh (100,) 0.01 50 : 0.8907507750856926, 0.944846893142246
tanh (100, 100) 0.0001 500 : 0.8683616776556562, -
0.015059031672665979
tanh (100, 100) 0.0001 200 : -0.010041802099251295,
0.9293389320647885
tanh (100, 100) 0.0001 100 : 0.7138338437083502,
0.9371877348057819
tanh (100, 100) 0.0001 50 : 0.891301154607073,
0.9481305798291868
tanh (100, 100) 0.001 500 : 0.8721615580438616, -
0.015059134637798843
tanh (100, 100) 0.001 200 : -0.010041878109644076,
0.9288706714192144
tanh (100, 100) 0.001 100 : 0.7182427263157897,
0.9299483957401421
tanh (100, 100) 0.001 50 : 0.8994615409297481,
0.9447480288386201
tanh (100, 100) 0.01 500 : 0.8700496766712991, -
0.015060059334008002
tanh (100, 100) 0.01 200 : -0.010043172804344325,
0.927181929209034
tanh (100, 100) 0.01 100 : 0.711417220095416, 0.9383483980551237
tanh (100, 100) 0.01 50 : 0.8821077344633347, 0.9462097077791588
tanh (100, 200) 0.0001 500 : 0.8643960301202466,
0.9192352002780891
tanh (100, 200) 0.0001 200 : 0.6917965179933888,
0.9396734613899138
tanh (100, 200) 0.0001 100 : 0.8952586318012555,
0.9143570984895909
tanh (100, 200) 0.0001 50 : 0.8817298529626072,
0.9235191870149909
tanh (100, 200) 0.001 500 : 0.8659323280626415,
0.9284159562576839
tanh (100, 200) 0.001 200 : 0.6993760208703923,
0.9416270733995354
tanh (100, 200) 0.001 100 : 0.8909459518269183,
0.9176211233774377
tanh (100, 200) 0.001 50 : 0.8886869516533645,
0.9374765906751836
tanh (100, 200) 0.01 500 : 0.8710054896063262,
0.9245601745553911
tanh (100, 200) 0.01 200 : 0.6969115330890462,
0.9404145485658655
tanh (100, 200) 0.01 100 : 0.8909992676674976,
0.9128379602534071
tanh (100, 200) 0.01 50 : 0.8894654893145019, 0.9420993208064914

tanh (50,) 0.0001 500 : 0.8829457683390929, 0.9323659486808192
tanh (50,) 0.0001 200 : 0.6958883722963355, 0.9278354841080767
tanh (50,) 0.0001 100 : 0.8935849256455832, 0.9298655641383008
tanh (50,) 0.0001 50 : 0.8885401802964074, 0.9249337560536728
tanh (50,) 0.001 500 : 0.881786975125651, 0.9322646512957178
tanh (50,) 0.001 200 : 0.6962158850386396, 0.9345805567941854
tanh (50,) 0.001 100 : 0.8899077657944623, 0.9340512632529705
tanh (50,) 0.001 50 : 0.8900919121191686, 0.9383288877689301
tanh (50,) 0.01 500 : 0.8821916358193362, 0.9345286463948507
tanh (50,) 0.01 200 : 0.6962986672205687, 0.9216107813739061
tanh (50,) 0.01 100 : 0.8796463044866245, 0.9418486085088464
tanh (50,) 0.01 50 : 0.8875213972392852, 0.9353661260158317
tanh (50, 50) 0.0001 500 : -0.00883420420947716, -0.02679394698598192
tanh (50, 50) 0.0001 200 : -0.008012121264820404, -0.018022306896520623
tanh (50, 50) 0.0001 100 : -0.008901438943121898, -0.019491383968032405
tanh (50, 50) 0.0001 50 : -0.008674632981902741, -0.01756904851719243
tanh (50, 50) 0.001 500 : -0.00883422540746226, -0.02679419159087315
tanh (50, 50) 0.001 200 : -0.008012243120786211, -0.018022398908907444
tanh (50, 50) 0.001 100 : -0.00890154718014129, -0.019491591437363454
tanh (50, 50) 0.001 50 : -0.00867478247388842, -0.01756936920383456
tanh (50, 50) 0.01 500 : -0.008834623432082633, -0.026796655453912077
tanh (50, 50) 0.01 200 : -0.008013398063671939, -0.018023326850643562
tanh (50, 50) 0.01 100 : -0.008902642167946917, -0.019493663523526772
tanh (50, 50) 0.01 50 : -0.008676339563326385, -0.01757257017343461
tanh (50, 100) 0.0001 500 : 0.16221568810643508, -0.015300688690939257
tanh (50, 100) 0.0001 200 : -0.009360670399730075, -0.017918490568820555
tanh (50, 100) 0.0001 100 : -0.008536312255063683, -0.017085337860819116
tanh (50, 100) 0.0001 50 : -0.008932006015546801, -0.017274126398010514
tanh (50, 100) 0.001 500 : 0.15651930195795488, -0.015300792266617957
tanh (50, 100) 0.001 200 : -0.009360821263410913, -0.01791854994035913
tanh (50, 100) 0.001 100 : -0.008536482055512584, -0.017085458245306606
tanh (50, 100) 0.001 50 : -0.008932203584738918, -0.017274274528501943
tanh (50, 100) 0.01 500 : -0.008883226796350608, -0.015302152564911298
tanh (50, 100) 0.01 200 : -0.009362120141635667, -0.01791915769911867
tanh (50, 100) 0.01 100 : -0.008537828012230752, -0.017086714387938073
tanh (50, 100) 0.01 50 : -0.008933937644987644, -0.01727593020503604
tanh (50, 200) 0.0001 500 : 0.32568919560768145, -0.020631802590440618
tanh (50, 200) 0.0001 200 : -0.0083572700456235, 0.9115524718597438
tanh (50, 200) 0.0001 100 : -0.009526535895320487, -0.017179297332939214
tanh (50, 200) 0.0001 50 : 0.16082932236393188, -0.018197244914776256
tanh (50, 200) 0.001 500 : 0.3280135697762399, -0.020631932925199603
tanh (50, 200) 0.001 200 : -0.00835738321470183, 0.9324123051944875
tanh (50, 200) 0.001 100 : -0.009526624958996787, -0.017179370480345346
tanh (50, 200) 0.001 50 : 0.1625507834224053, -0.018196883871881786
tanh (50, 200) 0.01 500 : 0.32739703236763384, -0.02063325775076974
tanh (50, 200) 0.01 200 : -0.00835871173548437, 0.9087075726015686
tanh (50, 200) 0.01 100 : -0.009527889639394216, -0.01718029080360961
tanh (50, 200) 0.01 50 : 0.1660357660457963, -0.018197216445960507
logistic (200,) 0.0001 500 : 0.8969627003490253, 0.930075860366737
logistic (200,) 0.0001 200 : 0.9033623698490505, 0.9304515458739296
logistic (200,) 0.0001 100 : 0.9002412936836744, 0.9271726752941842
logistic (200,) 0.0001 50 : 0.9063810513117767, 0.9341608883638772
logistic (200,) 0.001 500 : 0.8970484604614161, 0.9303451887645853
logistic (200,) 0.001 200 : 0.9010881834970779, 0.9302742726797293
logistic (200,) 0.001 100 : 0.8981083004164937, 0.9307720323147834
logistic (200,) 0.001 50 : 0.9033947867025145, 0.93234291732124
logistic (200,) 0.01 500 : 0.8901045645195239, 0.9313483945800809
logistic (200,) 0.01 200 : 0.8978448999283011, 0.9332091634257702
logistic (200,) 0.01 100 : 0.8997923837212671, 0.9349685883089237
logistic (200,) 0.01 50 : 0.9045443486224981, 0.9348799518771451
logistic (200, 200) 0.0001 500 : 0.8764532207436828, -0.02145814459958517
logistic (200, 200) 0.0001 200 : 0.3553953638238375, 0.9391185670151992
logistic (200, 200) 0.0001 100 : 0.7203675205742096, 0.9316754960676896
logistic (200, 200) 0.0001 50 : 0.8998601811930935, 0.9476203343713392
logistic (200, 200) 0.001 500 : 0.8815021986908789, -0.02145580094198607
logistic (200, 200) 0.001 200 : 0.35924079125961916, 0.9303519026221452
logistic (200, 200) 0.001 100 : 0.7182268729709473, 0.9393633289046616
logistic (200, 200) 0.001 50 : 0.8992836295562492, 0.9442248996697075
logistic (200, 200) 0.01 500 : 0.8787462817569596, -0.021424826007402142
logistic (200, 200) 0.01 200 : 0.3650885673862584, 0.9243776964288531
logistic (200, 200) 0.01 100 : 0.8908193460411115, 0.9363309681783927
logistic (200, 200) 0.01 50 : 0.898694124539003, 0.9470640410540615
logistic (100,) 0.0001 500 : 0.8890332758785743, 0.9284800713201293
logistic (100,) 0.0001 200 : 0.8966259866077099, 0.9351467317312513
logistic (100,) 0.0001 100 : 0.8946304045426002, 0.9349086273948399
logistic (100,) 0.0001 50 : 0.896116117001226, 0.9288643514816576
logistic (100,) 0.001 500 : 0.8885740597273474, 0.9302188934516615
logistic (100,) 0.001 200 : 0.8908940969602416, 0.9315055085675717
logistic (100,) 0.001 100 : 0.8910464575825096, 0.9361470863700673
logistic (100,) 0.001 50 : 0.8947529527355966, 0.9356924303827714
logistic (100,) 0.01 500 : 0.8853691137964695, 0.930893282631714
logistic (100,) 0.01 200 : 0.888862947917465, 0.931823565783028
logistic (100,) 0.01 100 : 0.8943253008408654, 0.94091989410674
logistic (100,) 0.01 50 : 0.8975820424585829, 0.9376640893642092
logistic (100, 100) 0.0001 500 : 0.8799975208245308, -0.01918924514150655
logistic (100, 100) 0.0001 200 : 0.5289426409161211,

0.9387891556751908
logistic (100, 100) 0.0001 100 : 0.7258267383546109, 0.9347252959545751
logistic (100, 100) 0.0001 50 : 0.90606701690857, 0.9389791933774071
logistic (100, 100) 0.001 500 : 0.8786836572886182, -0.019189718084142937
logistic (100, 100) 0.001 200 : 0.5272680211989926, 0.9290687592578555
logistic (100, 100) 0.001 100 : 0.7214200880391183, 0.9390831214604854
logistic (100, 100) 0.001 50 : 0.9041177812084176, 0.9403483837476221
logistic (100, 100) 0.01 500 : 0.8801291762653524, -0.019193160599217052
logistic (100, 100) 0.01 200 : 0.7035146052559157, 0.9332747528325129
logistic (100, 100) 0.01 100 : 0.703097236561061, 0.9362490448994276
logistic (100, 100) 0.01 50 : 0.8901044599852881, 0.9392713018496
logistic (100, 200) 0.0001 500 : -0.008795593169225091, -0.02671376928465108
logistic (100, 200) 0.0001 200 : -0.008878057569554532, 0.9348223031185168
logistic (100, 200) 0.0001 100 : -0.008855252375279132, -0.017867037842141276
logistic (100, 200) 0.0001 50 : 0.5378643274128377, -0.016670410340153374
logistic (100, 200) 0.001 500 : -0.008795694288665733, -0.026714102811966844
logistic (100, 200) 0.001 200 : -0.008878561263601936, 0.9370228547040934
logistic (100, 200) 0.001 100 : -0.008855715196993508, -0.01786719980456475
logistic (100, 200) 0.001 50 : 0.5411551510709272, -0.016667116965635387
logistic (100, 200) 0.01 500 : -0.008796678219404264, -0.02671755484039684
logistic (100, 200) 0.01 200 : -0.008883244763023202, 0.9290527221969399
logistic (100, 200) 0.01 100 : -0.008859867281789979, -0.01786874431478558
logistic (100, 200) 0.01 50 : 0.5398010513037048, -0.016624856513310693
logistic (50,) 0.0001 500 : 0.8881846738700976, 0.932737689009276
logistic (50,) 0.0001 200 : 0.8922874283243674, 0.930425361657433
logistic (50,) 0.0001 100 : 0.8957971679339292, 0.9318565727478881
logistic (50,) 0.0001 50 : 0.8981943331092955, 0.9377405451571492
logistic (50,) 0.001 500 : 0.8857315475348075, 0.9323416752369172
logistic (50,) 0.001 200 : 0.8934574611036485, 0.9292755283088256
logistic (50,) 0.001 100 : 0.8861013239043016, 0.9305717905450133
logistic (50,) 0.001 50 : 0.8941146078299396, 0.9319970330116756
logistic (50,) 0.01 500 : 0.884818734258582, 0.9331033615293172
logistic (50,) 0.01 200 : 0.8896906631234959, 0.930386480037964
logistic (50,) 0.01 100 : 0.8923250263701938, 0.9392680547868932
logistic (50,) 0.01 50 : 0.8969170953353398, 0.9392395411912818
logistic (50, 50) 0.0001 500 : 0.8874525019461614, 0.9424719002783548
logistic (50, 50) 0.0001 200 : 0.8927194246931064, 0.929743707213234
logistic (50, 50) 0.0001 100 : 0.8973620244993711, 0.943050056737006
logistic (50, 50) 0.0001 50 : 0.9028044920514701, 0.9380162219031258
logistic (50, 50) 0.001 500 : 0.8816751355605721, 0.9458243788947146
logistic (50, 50) 0.001 200 : 0.8928553347946678, 0.9280572757171893
logistic (50, 50) 0.001 100 : 0.9005298958281752, 0.9366700493384812
logistic (50, 50) 0.001 50 : 0.9009496483642392, 0.9368843150762354
logistic (50, 50) 0.01 500 : 0.8808488712310382, 0.9479713731995296
logistic (50, 50) 0.01 200 : 0.8920089855895104, 0.9376731975567953
logistic (50, 50) 0.01 100 : 0.8883273677446331, 0.953683485535923
logistic (50, 50) 0.01 50 : 0.896234987133359, 0.9492315932005153
logistic (50, 100) 0.0001 500 : -0.008793313263237313, -0.021677080514300284
logistic (50, 100) 0.0001 200 : -0.010729468376152119, -0.017613357860019496
logistic (50, 100) 0.0001 100 : -0.009149891827183333, -0.019120008619367912
logistic (50, 100) 0.0001 50 : -0.00854807626305818, -0.017343095788494356
logistic (50, 100) 0.001 500 : -0.008793421350857944, -0.02167667369840598
logistic (50, 100) 0.001 200 : -0.01072998282975086, -0.017613373912252817
logistic (50, 100) 0.001 100 : -0.009150362323473438, -0.019120214404964697
logistic (50, 100) 0.001 50 : -0.008548659087220623, -0.017343524924232634
logistic (50, 100) 0.01 500 : -0.008794419227463334, -0.021672381548496045
logistic (50, 100) 0.01 200 : -0.010734752830857675, -0.017613618379830553
logistic (50, 100) 0.01 100 : -0.00915480264450399, -0.01912248733672639
logistic (50, 100) 0.01 50 : -0.008554197587507018, -0.0173481613853832
logistic (50, 200) 0.0001 500 : -0.008849416457961645, -0.02207223235665401
logistic (50, 200) 0.0001 200 : -0.010207757525259842, -0.01780264808590437
logistic (50, 200) 0.0001 100 : -0.008772756056151198, -0.019482026795997864
logistic (50, 200) 0.0001 50 : -0.009190159016139666, -0.01718534684607831
logistic (50, 200) 0.001 500 : -0.008849508269990158, -0.022072532038403425
logistic (50, 200) 0.001 200 : -0.010208199822731245, -0.017802792638031972
logistic (50, 200) 0.001 100 : -0.008773223212888004, -0.019482344253556327
logistic (50, 200) 0.001 50 : -0.009190821872075317, -0.017185755831935934
logistic (50, 200) 0.01 500 : -0.00885041038980967, -0.02207581740427944
logistic (50, 200) 0.01 200 : -0.010212187250254478, -0.01780427169427945
logistic (50, 200) 0.01 100 : -0.008777595676233796, -0.019485665339277203
logistic (50, 200) 0.01 50 : -0.009197169923776727, -0.0171901958224856

Grid search of LightGBM, Avalon (1024)
lgb.LGBMRegressor(boosting_type = "gbdt", num_leaves = j,max_depth = 0)
j: accuracy of prediction using cross validation, accuracy of prediction using test data
10 : 0.8456905457934851, 0.8979688900356251
50 : 0.8444858526715558, 0.8962838339778385
100 : 0.8444858526715558, 0.8962838339778385
150 : 0.8444858526715558, 0.8962838339778385

Grid search of SVR, Avalon(1024)
SVR(C= c_num, kernel = ker, epsilon = e, gamma = r,degree = 3, coef0=1)
Ker c_num r e: accuracy of prediction using cross validation, accuracy of prediction using test data
linear 1 1 0.1 : 0.9374698933811179, 0.9631017341290798
linear 1 1 0.3 : 0.9373802804840003, 0.9629184847520086
linear 1 1 0.5 : 0.9366889923090648, 0.9630003232638848
linear 1 1 0.7 : 0.9356722332350864, 0.9619789739754074

linear 1 1 0.9 : 0.9343665580046003, 0.96164860496472
linear 1 2 0.1 : 0.9374698933811179, 0.9631017341290798
linear 1 2 0.3 : 0.9373802804840003, 0.9629184847520086
linear 1 2 0.5 : 0.9366889923090648, 0.9630003232638848
linear 1 2 0.7 : 0.9356722332350864, 0.9619789739754074
linear 1 2 0.9 : 0.9343665580046003, 0.96164860496472
linear 1 3 0.1 : 0.9374698933811179, 0.9631017341290798
linear 1 3 0.3 : 0.9373802804840003, 0.9629184847520086
linear 1 3 0.5 : 0.9366889923090648, 0.9630003232638848
linear 1 3 0.7 : 0.9356722332350864, 0.9619789739754074
linear 1 3 0.9 : 0.9343665580046003, 0.96164860496472
linear 1 4 0.1 : 0.9374698933811179, 0.9631017341290798
linear 1 4 0.3 : 0.9373802804840003, 0.9629184847520086
linear 1 4 0.5 : 0.9366889923090648, 0.9630003232638848
linear 1 4 0.7 : 0.9356722332350864, 0.9619789739754074
linear 1 4 0.9 : 0.9343665580046003, 0.96164860496472
linear 1 5 0.1 : 0.9374698933811179, 0.9631017341290798
linear 1 5 0.3 : 0.9373802804840003, 0.9629184847520086
linear 1 5 0.5 : 0.9366889923090648, 0.9630003232638848
linear 1 5 0.7 : 0.9356722332350864, 0.9619789739754074
linear 1 5 0.9 : 0.9343665580046003, 0.96164860496472
linear 1 6 0.1 : 0.9374698933811179, 0.9631017341290798
linear 1 6 0.3 : 0.9373802804840003, 0.9629184847520086
linear 1 6 0.5 : 0.9366889923090648, 0.9630003232638848
linear 1 6 0.7 : 0.9356722332350864, 0.9619789739754074
linear 1 6 0.9 : 0.9343665580046003, 0.96164860496472
linear 2 1 0.1 : 0.9479807749917553, 0.9626240863200177
linear 2 1 0.3 : 0.9475725118670075, 0.965035683023164
linear 2 1 0.5 : 0.9467755885572092, 0.9647974528992019
linear 2 1 0.7 : 0.9451196462997341, 0.9639050440548786
linear 2 1 0.9 : 0.9431685078308517, 0.9636397540175632
linear 2 2 0.1 : 0.9479807749917553, 0.9626240863200177
linear 2 2 0.3 : 0.9475725118670075, 0.965035683023164
linear 2 2 0.5 : 0.9467755885572092, 0.9647974528992019
linear 2 2 0.7 : 0.9451196462997341, 0.9639050440548786
linear 2 2 0.9 : 0.9431685078308517, 0.9636397540175632
linear 2 3 0.1 : 0.9479807749917553, 0.9626240863200177
linear 2 3 0.3 : 0.9475725118670075, 0.965035683023164
linear 2 3 0.5 : 0.9467755885572092, 0.9647974528992019
linear 2 3 0.7 : 0.9451196462997341, 0.9639050440548786
linear 2 3 0.9 : 0.9431685078308517, 0.9636397540175632
linear 2 4 0.1 : 0.9479807749917553, 0.9626240863200177
linear 2 4 0.3 : 0.9475725118670075, 0.965035683023164
linear 2 4 0.5 : 0.9467755885572092, 0.9647974528992019
linear 2 4 0.7 : 0.9451196462997341, 0.9639050440548786
linear 2 4 0.9 : 0.9431685078308517, 0.9636397540175632
linear 2 5 0.1 : 0.9479807749917553, 0.9626240863200177
linear 2 5 0.3 : 0.9475725118670075, 0.965035683023164
linear 2 5 0.5 : 0.9467755885572092, 0.9647974528992019
linear 2 5 0.7 : 0.9451196462997341, 0.9639050440548786
linear 2 5 0.9 : 0.9431685078308517, 0.9636397540175632
linear 2 6 0.1 : 0.9479807749917553, 0.9626240863200177
linear 2 6 0.3 : 0.9475725118670075, 0.965035683023164
linear 2 6 0.5 : 0.9467755885572092, 0.9647974528992019
linear 2 6 0.7 : 0.9451196462997341, 0.9639050440548786
linear 2 6 0.9 : 0.9431685078308517, 0.9636397540175632
linear 3 1 0.1 : 0.9476135303174791, 0.962039423238684
linear 3 1 0.3 : 0.9477714090584307, 0.9643953399476858
linear 3 1 0.5 : 0.9472569653646501, 0.9639677794602726
linear 3 1 0.7 : 0.9459327248945375, 0.9629605313723555
linear 3 1 0.9 : 0.944181218317015, 0.9625679297248523
linear 3 2 0.1 : 0.9476135303174791, 0.962039423238684
linear 3 2 0.3 : 0.9477714090584307, 0.9643953399476858
linear 3 2 0.5 : 0.9472569653646501, 0.9639677794602726
linear 3 2 0.7 : 0.9459327248945375, 0.9629605313723555
linear 3 2 0.9 : 0.944181218317015, 0.9625679297248523
linear 3 3 0.1 : 0.9476135303174791, 0.962039423238684
linear 3 3 0.3 : 0.9477714090584307, 0.9643953399476858
linear 3 3 0.5 : 0.9472569653646501, 0.9639677794602726
linear 3 3 0.7 : 0.9459327248945375, 0.9629605313723555
linear 3 3 0.9 : 0.944181218317015, 0.9625679297248523
linear 3 4 0.1 : 0.9476135303174791, 0.962039423238684
linear 3 4 0.3 : 0.9477714090584307, 0.9643953399476858
linear 3 4 0.5 : 0.9472569653646501, 0.9639677794602726
linear 3 4 0.7 : 0.9459327248945375, 0.9629605313723555
linear 3 4 0.9 : 0.944181218317015, 0.9625679297248523
linear 3 5 0.1 : 0.9476135303174791, 0.962039423238684
linear 3 5 0.3 : 0.9477714090584307, 0.9643953399476858
linear 3 5 0.5 : 0.9472569653646501, 0.9639677794602726
linear 3 5 0.7 : 0.9459327248945375, 0.9629605313723555
linear 3 5 0.9 : 0.944181218317015, 0.9625679297248523
linear 3 6 0.1 : 0.9476135303174791, 0.962039423238684
linear 3 6 0.3 : 0.9477714090584307, 0.9643953399476858
linear 3 6 0.5 : 0.9472569653646501, 0.9639677794602726
linear 3 6 0.7 : 0.9459327248945375, 0.9629605313723555
linear 3 6 0.9 : 0.944181218317015, 0.9625679297248523
linear 4 1 0.1 : 0.9471780405870914, 0.9608843852196651
linear 4 1 0.3 : 0.9478575415935138, 0.9632048683759306
linear 4 1 0.5 : 0.9472968760311866, 0.9632709901074884
linear 4 1 0.7 : 0.9464452222231193, 0.9626037095704864
linear 4 1 0.9 : 0.9447939408586231, 0.962188562153640l
linear 4 2 0.1 : 0.9471780405870914, 0.9608843852196651
linear 4 2 0.3 : 0.9478575415935138, 0.9632048683759306
linear 4 2 0.5 : 0.9472968760311866, 0.9632709901074884
linear 4 2 0.7 : 0.9464452222231193, 0.9626037095704864
linear 4 2 0.9 : 0.9447939408586231, 0.962188562153640l
linear 4 3 0.1 : 0.9471780405870914, 0.9608843852196651
linear 4 3 0.3 : 0.9478575415935138, 0.9632048683759306
linear 4 3 0.5 : 0.9472968760311866, 0.9632709901074884
linear 4 3 0.7 : 0.9464452222231193, 0.9626037095704864
linear 4 3 0.9 : 0.9447939408586231, 0.962188562153640l
linear 4 4 0.1 : 0.9471780405870914, 0.9608843852196651
linear 4 4 0.3 : 0.9478575415935138, 0.9632048683759306
linear 4 4 0.5 : 0.9472968760311866, 0.9632709901074884
linear 4 4 0.7 : 0.9464452222231193, 0.9626037095704864
linear 4 4 0.9 : 0.9447939408586231, 0.962188562153640l
linear 4 5 0.1 : 0.9471780405870914, 0.9608843852196651
linear 4 5 0.3 : 0.9478575415935138, 0.9632048683759306
linear 4 5 0.5 : 0.9472968760311866, 0.9632709901074884
linear 4 5 0.7 : 0.9464452222231193, 0.9626037095704864
linear 4 5 0.9 : 0.9447939408586231, 0.962188562153640l
linear 4 6 0.1 : 0.9471780405870914, 0.9608843852196651
linear 4 6 0.3 : 0.9478575415935138, 0.9632048683759306
linear 4 6 0.5 : 0.9472968760311866, 0.9632709901074884
linear 4 6 0.7 : 0.9464452222231193, 0.9626037095704864
linear 4 6 0.9 : 0.9447939408586231, 0.962188562153640l
poly 1 1 0.1 : 0.886650048627027, 0.9280903323810419
poly 1 1 0.3 : 0.8847146704227932, 0.9257008219605919
poly 1 1 0.5 : 0.8825703243562358, 0.923069035030572
poly 1 1 0.7 : 0.8800821270565902, 0.9205167409576858
poly 1 1 0.9 : 0.8773469157017523, 0.9175902518232948
poly 1 2 0.1 : 0.8864173528365498, 0.9278467890411933
poly 1 2 0.3 : 0.8844826008661701, 0.9254624912134969
poly 1 2 0.5 : 0.8823412458709814, 0.922832647164117
poly 1 2 0.7 : 0.8798546988136723, 0.9202753693682655
poly 1 2 0.9 : 0.8771251330586365, 0.9173499124006858
poly 1 3 0.1 : 0.8863397070129739, 0.9277643315255703
poly 1 3 0.3 : 0.8844041583382728, 0.9253830229768895
poly 1 3 0.5 : 0.8822638177216291, 0.9227529888981628
poly 1 3 0.7 : 0.8797787077940271, 0.9201947351589461
poly 1 3 0.9 : 0.8770505055142988, 0.9172692995869651
poly 1 4 0.1 : 0.8863005126612975, 0.9277240652167988
poly 1 4 0.3 : 0.8843650478043171, 0.9253423647435254
poly 1 4 0.5 : 0.8822254681913604, 0.9227137451502208
poly 1 4 0.7 : 0.8797401403253866, 0.9201536070536741
poly 1 4 0.9 : 0.8770130679516855, 0.9172286037463084
poly 1 5 0.1 : 0.8862768223855493, 0.9276994245439937
poly 1 5 0.3 : 0.8843417110317626, 0.9253187296567033
poly 1 5 0.5 : 0.8822019979953846, 0.9226892065775621
poly 1 5 0.7 : 0.8797171408542749, 0.9201288580404248
poly 1 5 0.9 : 0.8769903848657676, 0.9172043995746115
poly 1 6 0.1 : 0.8862610785122698, 0.9276834065868159
poly 1 6 0.3 : 0.8843259806684791, 0.9253028370414745
poly 1 6 0.5 : 0.8821862946789143, 0.9226730739512464
poly 1 6 0.7 : 0.879702058520123, 0.9201125341801019
poly 1 6 0.9 : 0.8769754005430432, 0.9171879313996377
poly 2 1 0.1 : 0.8866503276742014, 0.928089602198918
poly 2 1 0.3 : 0.8847146703338673, 0.9257008220134099

poly 2 1 0.5 : 0.8825703244510565, 0.9230690350761429
poly 2 1 0.7 : 0.8800821270297995, 0.9205167409135993
poly 2 1 0.9 : 0.8773469157017523, 0.9175902518232948
poly 2 2 0.1 : 0.8864173528373647, 0.9278467879228478
poly 2 2 0.3 : 0.8844825403789895, 0.9254624915064111
poly 2 2 0.5 : 0.8823412464763745, 0.9228326474013487
poly 2 2 0.7 : 0.8798546992026388, 0.9202753690180614
poly 2 2 0.9 : 0.8771251330586365, 0.9173499124006858
poly 2 3 0.1 : 0.8863396352607698, 0.9277643311541439
poly 2 3 0.3 : 0.8844046065877571, 0.925383661306614
poly 2 3 0.5 : 0.8822638170175271, 0.9227529879654837
poly 2 3 0.7 : 0.8797787082791692, 0.9201947365960057
poly 2 3 0.9 : 0.8770505055142988, 0.9172692995869651
poly 2 4 0.1 : 0.8863005016239193, 0.9277240689722552
poly 2 4 0.3 : 0.8843652468485352, 0.9253422420355948
poly 2 4 0.5 : 0.8822256117550712, 0.9227124551026343
poly 2 4 0.7 : 0.8797401363610918, 0.9201538213986886
poly 2 4 0.9 : 0.8770130679516855, 0.9172286037463084
poly 2 5 0.1 : 0.8862764422641979, 0.9276994221036001
poly 2 5 0.3 : 0.8843416893814527, 0.9253185959695128
poly 2 5 0.5 : 0.8822019040613064, 0.9226891950378139
poly 2 5 0.7 : 0.8797170832117491, 0.9201288632937259
poly 2 5 0.9 : 0.8769903848657676, 0.9172043995746115
poly 2 6 0.1 : 0.8862610752417799, 0.9276833910713778
poly 2 6 0.3 : 0.8843261414278206, 0.9253029760792705
poly 2 6 0.5 : 0.8821863214927699, 0.9226731624086715
poly 2 6 0.7 : 0.8797019982520128, 0.9201128296546186
poly 2 6 0.9 : 0.8769754005430432, 0.9171879313996377
poly 3 1 0.1 : 0.8866498757217312, 0.928089602254738
poly 3 1 0.3 : 0.884714820873574, 0.9257008220812382
poly 3 1 0.5 : 0.8825703244474858, 0.9230690350615249
poly 3 1 0.7 : 0.880082127076121, 0.9205167408860363
poly 3 1 0.9 : 0.8773469157017523, 0.9175902518232948
poly 3 2 0.1 : 0.8864175209237342, 0.927846787653283
poly 3 2 0.3 : 0.8844820342454668, 0.9254624911703007
poly 3 2 0.5 : 0.8823411823005918, 0.922833313465838
poly 3 2 0.7 : 0.8798547249384832, 0.9202753685547878
poly 3 2 0.9 : 0.8771251330586365, 0.9173499124006858
poly 3 3 0.1 : 0.8863397753702873, 0.9277643346444063
poly 3 3 0.3 : 0.8844046044700397, 0.925383021925356
poly 3 3 0.5 : 0.8822637834836579, 0.922752900607315
poly 3 3 0.7 : 0.8797787065704197, 0.9201947382191764
poly 3 3 0.9 : 0.8770505055142988, 0.9172692995869651
poly 3 4 0.1 : 0.886300539491935, 0.9277240732815473
poly 3 4 0.3 : 0.8843654727759175, 0.925342244275588
poly 3 4 0.5 : 0.8822256146118714, 0.9227130261965187
poly 3 4 0.7 : 0.8797401361912242, 0.9201536099406025
poly 3 4 0.9 : 0.8770130679516855, 0.9172286037463084
poly 3 5 0.1 : 0.8862768210074302, 0.9276994280758023
poly 3 5 0.3 : 0.8843416601673306, 0.925318724941901
poly 3 5 0.5 : 0.8822020672909012, 0.9226892640870883
poly 3 5 0.7 : 0.8797170914115995, 0.9201287861871545
poly 3 5 0.9 : 0.8769903848657676, 0.9172043995746115
poly 3 6 0.1 : 0.8862610837394971, 0.9276832040224968
poly 3 6 0.3 : 0.8843262988946273, 0.9253026046298097
poly 3 6 0.5 : 0.882186328892492, 0.9226733137967436
poly 3 6 0.7 : 0.8797019376862514, 0.9201126652067683
poly 3 6 0.9 : 0.8769754005430432, 0.9171879313996377
poly 4 1 0.1 : 0.886649875783824, 0.9280896023109542
poly 4 1 0.3 : 0.884714670382351, 0.9257008221546896
poly 4 1 0.5 : 0.8825703244288258, 0.9230690351110316
poly 4 1 0.7 : 0.88008212704502, 0.9205167409350667
poly 4 1 0.9 : 0.8773469157017523, 0.9175902518232948
poly 4 2 0.1 : 0.8864175218329846, 0.9278467882956067
poly 4 2 0.3 : 0.8844824516491074, 0.9254624908060898
poly 4 2 0.5 : 0.8823411831658434, 0.9228327266035318
poly 4 2 0.7 : 0.8798547242780763, 0.9202753686332394
poly 4 2 0.9 : 0.8771251330586365, 0.9173499124006858
poly 4 3 0.1 : 0.8863397745000536, 0.9277643379643417
poly 4 3 0.3 : 0.8844044349026283, 0.9253830222981357
poly 4 3 0.5 : 0.8822637834361, 0.9227529897182317
poly 4 3 0.7 : 0.879778706322415, 0.9201947384406526
poly 4 3 0.9 : 0.8770505055142988, 0.9172692995869651
poly 4 4 0.1 : 0.8863005383992647, 0.9277240722586846
poly 4 4 0.3 : 0.8843654723918186, 0.9253422465034937
poly 4 4 0.5 : 0.8822256125573051, 0.9227125403247883
poly 4 4 0.7 : 0.8797401428446883, 0.9201536052806609
poly 4 4 0.9 : 0.8770130679516855, 0.9172286037463084
poly 4 5 0.1 : 0.8862765616887603, 0.9276994319992014
poly 4 5 0.3 : 0.8843416724902893, 0.9253187134496628
poly 4 5 0.5 : 0.8822019919147366, 0.9226892616657837
poly 4 5 0.7 : 0.8797171057186743, 0.9201287690913351
poly 4 5 0.9 : 0.8769903848657676, 0.9172043995746115
poly 4 6 0.1 : 0.8862610526379877, 0.9276832053651684
poly 4 6 0.3 : 0.884326289513767, 0.9253026216514342
poly 4 6 0.5 : 0.8821863307427673, 0.9226731101156456
poly 4 6 0.7 : 0.8797019199008614, 0.9201131292058463
poly 4 6 0.9 : 0.8769754005430432, 0.9171879313996377
rbf 1 1 0.1 : -0.0271567423927405, 0.0019704960719335585
rbf 1 1 0.3 : -0.026902671788778233, 0.0019393978370866316
rbf 1 1 0.5 : -0.026298119077819492, 0.0018698145106782471
rbf 1 1 0.7 : -0.025504034144234744, 0.0018231274495678074
rbf 1 1 0.9 : -0.024246404503582796, 0.0017924358088995396
rbf 1 2 0.1 : -0.027295558230127213, 0.00171091422194091 8
rbf 1 2 0.3 : -0.027041459382604938, 0.0016796425588375685
rbf 1 2 0.5 : -0.02643673946388252, 0.0016097080107493333
rbf 1 2 0.7 : -0.025642479790816353, 0.0015627719442478094
rbf 1 2 0.9 : -0.02438467459616338, 0.001531929198712145 3
rbf 1 3 0.1 : -0.027344796844514317, 0.0016189318132325825
rbf 1 3 0.3 : -0.027090693459380068, 0.0015875977127414176
rbf 1 3 0.5 : -0.026485950536202552, 0.0015175363508230788
rbf 1 3 0.7 : -0.025691643074056268, 0.0014705228353729671
rbf 1 3 0.9 : -0.02443376321063253, 0.001439631871763436
rbf 1 4 0.1 : -0.02736303064144612, 0.001585017508719977
rbf 1 4 0.3 : -0.027108925587570852, 0.0015536604491313
rbf 1 4 0.5 : -0.026504174233752488, 0.0014835524560019575
rbf 1 4 0.7 : -0.025709849216974855, 0.0014365104655633676
rbf 1 4 0.9 : -0.02445194189199129, 0.0014056017735355475
rbf 1 5 0.1 : -0.02736975582763721, 0.0015725297764574986
rbf 1 5 0.3 : -0.027115650159853066, 0.0015411642707647832
rbf 1 5 0.5 : -0.026510895704705106, 0.0014710391231262943
rbf 1 5 0.7 : -0.02571656423007358, 0.0014239866574204951
rbf 1 5 0.9 : -0.0244586468026176, 0.0013930714435314018
rbf 1 6 0.1 : -0.027372232237990346, 0.0015679342561879528
rbf 1 6 0.3 : -0.027118126344361525, 0.0015365656433479202
rbf 1 6 0.5 : -0.02651337074829927, 0.0014664341849192075
rbf 1 6 0.7 : -0.02571903689795687, 0.0014193778655784417
rbf 1 6 0.9 : -0.0244611157540092, 0.0013884602524311118
rbf 2 1 0.1 : -0.02430452734364108, 0.003863932386467206
rbf 2 1 0.3 : -0.02361765083516154, 0.003789503682499973
rbf 2 1 0.5 : -0.022938319576051126, 0.00369088851520738
rbf 2 1 0.7 : -0.022131744716131684, 0.0035733100904136172
rbf 2 1 0.9 : -0.021386266972559166, 0.0034487680240528062
rbf 2 2 0.1 : -0.024573138403217155, 0.0033483477837934483
rbf 2 2 0.3 : -0.02388584576254127, 0.00327333345174019
rbf 2 2 0.5 : -0.023206340516166612, 0.003174000140304334
rbf 2 2 0.7 : -0.0223993576925281, 0.0030556409293641673
rbf 2 2 0.9 : -0.021653813600814465, 0.0029303833954859027
rbf 2 3 0.1 : -0.02467025711535964, 0.003164490641378137
rbf 2 3 0.3 : -0.023982790880726988, 0.0030892617763296215
rbf 2 3 0.5 : -0.023303129763695328, 0.0029896680375417484
rbf 2 3 0.7 : -0.022496016390010885, 0.0028710255266476414
rbf 2 3 0.9 : -0.021750368457717384, 0.0027454927999788215
rbf 2 4 0.1 : -0.0247064819048199, 0.00309653354563999
rbf 2 4 0.3 : -0.024018951796450104, 0.0030212257924226993
rbf 2 4 0.5 : -0.02333923323506948, 0.002921536290107607
rbf 2 4 0.7 : -0.022532071876055104, 0.0028027896049956924
rbf 2 4 0.9 : -0.02178638559513586, 0.002677155680013032 5
rbf 2 5 0.1 : -0.02471987772497388, 0.003071488049894544
rbf 2 5 0.3 : -0.02403232411870828, 0.002996151275602932
rbf 2 5 0.5 : -0.023352584424538803, 0.0028964265441717707
rbf 2 5 0.7 : -0.022545405412725074, 0.0027776415358518047
rbf 2 5 0.9 : -0.021799705023669125, 0.002651970382423041
rbf 2 6 0.1 : -0.02472481518269167, 0.003062268165494597
rbf 2 6 0.3 : -0.02403725293203296, 0.0029869207149478827
rbf 2 6 0.5 : -0.023357505463544027, 0.0028871830234515894
rbf 2 6 0.7 : -0.02255031995762784, 0.002768383916813555
rbf 2 6 0.9 : -0.021804614378476427, 0.0026426990678072304

rbf 3 1 0.1 : -0.021280940918885615, 0.005379286676760242
rbf 3 1 0.3 : -0.020525650230682445, 0.005273551366978602
rbf 3 1 0.5 : -0.019627485712932113, 0.005126951626314158
rbf 3 1 0.7 : -0.01857821730545981, 0.004878435547638227
rbf 3 1 0.9 : -0.017490614907993085, 0.004563857271125515
rbf 3 2 0.1 : -0.02167113283787785, 0.0046099595817780115
rbf 3 2 0.3 : -0.020915680643981062, 0.004503377278135878
rbf 3 2 0.5 : -0.020017516561184844, 0.004355667780743033
rbf 3 2 0.7 : -0.01896757847092916, 0.0041054062484305875
rbf 3 2 0.9 : -0.01788175195035211, 0.003788820507529489
rbf 3 3 0.1 : -0.021814574024874033, 0.004333621260951159
rbf 3 3 0.3 : -0.0210589479664145, 0.00422672328458451
rbf 3 3 0.5 : -0.020160566672544622, 0.004078602611596427
rbf 3 3 0.7 : -0.019110468360924095, 0.0038277001372009067
rbf 3 3 0.9 : -0.018024409398519393, 0.0035103804592004906
rbf 3 4 0.1 : -0.021868465684303385, 0.004231229905944378
rbf 3 4 0.3 : -0.02111275359437208, 0.00412421583891065
rbf 3 4 0.5 : -0.020214333064836422, 0.003975943956476935
rbf 3 4 0.7 : -0.01916416392941054, 0.0037248058082285374
rbf 3 4 0.9 : -0.01807807251463971, 0.0034072162434407582
rbf 3 5 0.1 : -0.021888447667828447, 0.00419345987009101
rbf 3 5 0.3 : -0.021132714651726347, 0.0040864030960451325
rbf 3 5 0.5 : -0.02023426896371929, 0.003938075587236911
rbf 3 5 0.7 : -0.019184073773413246, 0.0036868507402334494
rbf 3 5 0.9 : -0.01809795612186238, 0.0033691618905268284
rbf 3 6 0.1 : -0.021895806847168454, 0.004179551187057928
rbf 3 6 0.3 : -0.021140079089145124, 0.004072478701982574
rbf 3 6 0.5 : -0.020241624145849534, 0.003924130729378139
rbf 3 6 0.7 : -0.019191419370485942, 0.0036728739876908945
rbf 3 6 0.9 : -0.0181052920669591, 0.003355148613110037
rbf 4 1 0.1 : -0.017173240106091205, 0.006188476047663483
rbf 4 1 0.3 : -0.01612068795172521, 0.005753926552080402
rbf 4 1 0.5 : -0.01508451337444856, 0.005291709705528724
rbf 4 1 0.7 : -0.014132770500157755, 0.004806933527087942
rbf 4 1 0.9 : -0.013197406625466312, 0.004194855356970506
rbf 4 2 0.1 : -0.017713344259532172, 0.005177477034288391
rbf 4 2 0.3 : -0.016667749565430046, 0.004747678520872323
rbf 4 2 0.5 : -0.015635372975517692, 0.004290867568271128
rbf 4 2 0.7 : -0.014685929854931912, 0.003812184929373519
rbf 4 2 0.9 : -0.013749763178919561, 0.003209408018349569
rbf 4 3 0.1 : -0.017901593004907214, 0.0048070744719979075
rbf 4 3 0.3 : -0.01685569576389643, 0.004376156687377342
rbf 4 3 0.5 : -0.015822992947557048, 0.003918260462506207
rbf 4 3 0.7 : -0.014874534642023063, 0.0034385293098090353
rbf 4 3 0.9 : -0.013941458584895728, 0.002834533746102208
rbf 4 4 0.1 : -0.01797286534068303, 0.004669498936802019
rbf 4 4 0.3 : -0.016926856689581715, 0.004238169525361712
rbf 4 4 0.5 : -0.015894070169248355, 0.0037798741303153305
rbf 4 4 0.7 : -0.014945429182849756, 0.003299757389715885
rbf 4 4 0.9 : -0.014012300430930913, 0.0026953135164263786
rbf 4 5 0.1 : -0.017999362971459522, 0.004618705719458438
rbf 4 5 0.3 : -0.01695331333399479, 0.004187224880220497
rbf 4 5 0.5 : -0.015920485136163932, 0.0037287826398606505
rbf 4 5 0.7 : -0.014971783805436489, 0.0032485240506039403
rbf 4 5 0.9 : -0.014038639654525587, 0.0026439152540294053
rbf 4 6 0.1 : -0.018009148549408226, 0.004599995292968284
rbf 4 6 0.3 : -0.016963083833896775, 0.004168458746566572
rbf 4 6 0.5 : -0.01593024030376462, 0.0037099624848436763
rbf 4 6 0.7 : -0.014981524539690882, 0.0032296517123908686
rbf 4 6 0.9 : -0.014048366957466585, 0.002624982243935703
sigmoid 1 1 0.1 : -0.02785157494475108, -0.0004817589182650739
sigmoid 1 1 0.3 : -0.028038637425086766, -0.0001527348652718974
sigmoid 1 1 0.5 : -0.029327682929847977, -8.39384078961114e-05
sigmoid 1 1 0.7 : -0.027370781055785543, -0.0001527348652718974
sigmoid 1 1 0.9 : -0.02742593928234638, -0.0003516451204561566
sigmoid 1 2 0.1 : -0.02785157494475108, -0.0004817589182650739
sigmoid 1 2 0.3 : -0.028038637425086766, -0.0001527348652718974
sigmoid 1 2 0.5 : -0.029327682929847977, -8.39384078961114e-05
sigmoid 1 2 0.7 : -0.027370781055785543, -0.0001527348652718974
sigmoid 1 2 0.9 : -0.02742593928234638, -0.0003516451204561566
sigmoid 1 3 0.1 : -0.02785157494475108, -0.0004817589182650739
sigmoid 1 3 0.3 : -0.028038637425086766, -0.0001527348652718974
sigmoid 1 3 0.5 : -0.029327682929847977, -8.39384078961114e-05
sigmoid 1 3 0.7 : -0.027370781055785543, -0.0001527348652718974
sigmoid 1 3 0.9 : -0.02742593928234638, -0.0003516451204561566
sigmoid 1 4 0.1 : -0.02785157494475108, -0.0004817589182650739
sigmoid 1 4 0.3 : -0.028038637425086766, -0.0001527348652718974
sigmoid 1 4 0.5 : -0.029327682929847977, -8.39384078961114e-05
sigmoid 1 4 0.7 : -0.027370781055785543, -0.0001527348652718974
sigmoid 1 4 0.9 : -0.02742593928234638, -0.0003516451204561566
sigmoid 1 5 0.1 : -0.02785157494475108, -0.0004817589182650739
sigmoid 1 5 0.3 : -0.028038637425086766, -0.0001527348652718974
sigmoid 1 5 0.5 : -0.029327682929847977, -8.39384078961114e-05
sigmoid 1 5 0.7 : -0.027370781055785543, -0.0001527348652718974
sigmoid 1 5 0.9 : -0.02742593928234638, -0.0003516451204561566
sigmoid 1 6 0.1 : -0.02785157494475108, -0.0004817589182650739
sigmoid 1 6 0.3 : -0.028038637425086766, -0.0001527348652718974
sigmoid 1 6 0.5 : -0.029327682929847977, -8.39384078961114e-05
sigmoid 1 6 0.7 : -0.027370781055785543, -0.0001527348652718974
sigmoid 1 6 0.9 : -0.02742593928234638, -0.0003516451204561566
sigmoid 2 1 0.1 : -0.02785157494475108, -0.0004817589182650739
sigmoid 2 1 0.3 : -0.028038637425086766, -0.0001527348652718974
sigmoid 2 1 0.5 : -0.029327682929847977, -8.39384078961114e-05
sigmoid 2 1 0.7 : -0.027370781055785543, -0.0001527348652718974
sigmoid 2 1 0.9 : -0.02742593928234638, -0.0003516451204561566
sigmoid 2 2 0.1 : -0.02785157494475108, -0.0004817589182650739
sigmoid 2 2 0.3 : -0.028038637425086766, -0.0001527348652718974
sigmoid 2 2 0.5 : -0.029327682929847977, -8.39384078961114e-05
sigmoid 2 2 0.7 : -0.027370781055785543, -0.0001527348652718974
sigmoid 2 2 0.9 : -0.02742593928234638, -0.0003516451204561566
sigmoid 2 3 0.1 : -0.02785157494475108, -0.0004817589182650739
sigmoid 2 3 0.3 : -0.028038637425086766, -0.0001527348652718974
sigmoid 2 3 0.5 : -0.029327682929847977, -8.39384078961114e-05
sigmoid 2 3 0.7 : -0.027370781055785543, -0.0001527348652718974
sigmoid 2 3 0.9 : -0.02742593928234638, -0.0003516451204561566
sigmoid 2 4 0.1 : -0.02785157494475108, -0.0004817589182650739
sigmoid 2 4 0.3 : -0.028038637425086766, -0.0001527348652718974
sigmoid 2 4 0.5 : -0.029327682929847977, -8.39384078961114e-05
sigmoid 2 4 0.7 : -0.027370781055785543, -0.0001527348652718974
sigmoid 2 4 0.9 : -0.02742593928234638, -0.0003516451204561566
sigmoid 2 5 0.1 : -0.02785157494475108, -0.0004817589182650739
sigmoid 2 5 0.3 : -0.028038637425086766, -0.0001527348652718974
sigmoid 2 5 0.5 : -0.029327682929847977, -8.39384078961114e-05
sigmoid 2 5 0.7 : -0.027370781055785543, -0.0001527348652718974
sigmoid 2 5 0.9 : -0.02742593928234638, -0.0003516451204561566
sigmoid 2 6 0.1 : -0.02785157494475108, -0.0004817589182650739
sigmoid 2 6 0.3 : -0.028038637425086766, -0.0001527348652718974
sigmoid 2 6 0.5 : -0.029327682929847977, -8.39384078961114e-05
sigmoid 2 6 0.7 : -0.027370781055785543, -0.0001527348652718974
sigmoid 2 6 0.9 : -0.02742593928234638, -0.0003516451204561566
sigmoid 3 1 0.1 : -0.02785157494475108, -0.0004817589182650739
sigmoid 3 1 0.3 : -0.028038637425086766, -0.0001527348652718974
sigmoid 3 1 0.5 : -0.029327682929847977, -8.39384078961114e-05
sigmoid 3 1 0.7 : -0.027370781055785543, -0.0001527348652718974
sigmoid 3 1 0.9 : -0.02742593928234638, -0.0003516451204561566
sigmoid 3 2 0.1 : -0.02785157494475108, -0.0004817589182650739
sigmoid 3 2 0.3 : -0.028038637425086766, -0.0001527348652718974
sigmoid 3 2 0.5 : -0.029327682929847977, -8.39384078961114e-05
sigmoid 3 2 0.7 : -0.027370781055785543, -0.0001527348652718974
sigmoid 3 2 0.9 : -0.02742593928234638, -0.0003516451204561566
sigmoid 3 3 0.1 : -0.02785157494475108, -0.0004817589182650739
sigmoid 3 3 0.3 : -0.028038637425086766, -0.0001527348652718974
sigmoid 3 3 0.5 : -0.029327682929847977, -8.39384078961114e-05
sigmoid 3 3 0.7 : -0.027370781055785543, -0.0001527348652718974
sigmoid 3 3 0.9 : -0.02742593928234638, -0.0003516451204561566
sigmoid 3 4 0.1 : -0.02785157494475108, -0.0004817589182650739
sigmoid 3 4 0.3 : -0.028038637425086766, -0.0001527348652718974
sigmoid 3 4 0.5 : -0.029327682929847977, -8.39384078961114e-05
sigmoid 3 4 0.7 : -0.027370781055785543, -0.0001527348652718974
sigmoid 3 4 0.9 : -0.02742593928234638, -0.0003516451204561566
sigmoid 3 5 0.1 : -0.02785157494475108, -0.0004817589182650739
sigmoid 3 5 0.3 : -0.028038637425086766, -0.0001527348652718974
sigmoid 3 5 0.5 : -0.029327682929847977, -8.39384078961114e-05
sigmoid 3 5 0.7 : -0.027370781055785543, -0.0001527348652718974
sigmoid 3 5 0.9 : -0.02742593928234638, -0.0003516451204561566
sigmoid 3 6 0.1 : -0.02785157494475108, -0.0004817589182650739
sigmoid 3 6 0.3 : -0.028038637425086766, -0.0001527348652718974
sigmoid 3 6 0.5 : -0.029327682929847977, -8.39384078961114e-05

sigmoid 3 6 0.7 : -0.027370781055785543, -0.0001527348652718974
sigmoid 3 6 0.9 : -0.02742593928234638, -0.0003516451204561566
sigmoid 4 1 0.1 : -0.02785157494475108, -0.0004817589182650739
sigmoid 4 1 0.3 : -0.028038637425086766, -0.0001527348652718974
sigmoid 4 1 0.5 : -0.029327682929847977, -8.39384078961114e-05
sigmoid 4 1 0.7 : -0.027370781055785543, -0.0001527348652718974
sigmoid 4 1 0.9 : -0.02742593928234638, -0.0003516451204561566
sigmoid 4 2 0.1 : -0.02785157494475108, -0.0004817589182650739
sigmoid 4 2 0.3 : -0.028038637425086766, -0.0001527348652718974
sigmoid 4 2 0.5 : -0.029327682929847977, -8.39384078961114e-05
sigmoid 4 2 0.7 : -0.027370781055785543, -0.0001527348652718974
sigmoid 4 2 0.9 : -0.02742593928234638, -0.0003516451204561566
sigmoid 4 3 0.1 : -0.02785157494475108, -0.0004817589182650739
sigmoid 4 3 0.3 : -0.028038637425086766, -0.0001527348652718974
sigmoid 4 3 0.5 : -0.029327682929847977, -8.39384078961114e-05
sigmoid 4 3 0.7 : -0.027370781055785543, -0.0001527348652718974
sigmoid 4 3 0.9 : -0.02742593928234638, -0.0003516451204561566
sigmoid 4 4 0.1 : -0.02785157494475108, -0.0004817589182650739
sigmoid 4 4 0.3 : -0.028038637425086766, -0.0001527348652718974
sigmoid 4 4 0.5 : -0.029327682929847977, -8.39384078961114e-05
sigmoid 4 4 0.7 : -0.027370781055785543, -0.0001527348652718974
sigmoid 4 4 0.9 : -0.02742593928234638, -0.0003516451204561566
sigmoid 4 5 0.1 : -0.02785157494475108, -0.0004817589182650739
sigmoid 4 5 0.3 : -0.028038637425086766, -0.0001527348652718974
sigmoid 4 5 0.5 : -0.029327682929847977, -8.39384078961114e-05
sigmoid 4 5 0.7 : -0.027370781055785543, -0.0001527348652718974
sigmoid 4 5 0.9 : -0.02742593928234638, -0.0003516451204561566
sigmoid 4 6 0.1 : -0.02785157494475108, -0.0004817589182650739
sigmoid 4 6 0.3 : -0.028038637425086766, -0.0001527348652718974
sigmoid 4 6 0.5 : -0.029327682929847977, -8.39384078961114e-05
sigmoid 4 6 0.7 : -0.027370781055785543, -0.0001527348652718974
sigmoid 4 6 0.9 : -0.02742593928234638, -0.0003516451204561566

Grid search of ElasticNet, Avalon (2048)
ElasticNet(alpha= i, l1_ratio= j, max_iter=100000)
i j : accuracy of prediction using cross validation, accuracy of prediction using test data
0.001 0.0 : 0.943284809567363, 0.9745070278848094
0.001 0.2 : 0.9431922224782706, 0.97606449222926
0.001 0.4 : 0.94339419186211, 0.9753673993661592
0.001 0.6 : 0.9424578623072127, 0.9745999243786544
0.001 0.8 : 0.9422761297101785, 0.9742123606104042
0.001 1.0 : 0.9311147330438294, 0.9727502904204273
0.01 0.0 : 0.9349736688404608, 0.9663073925346086
0.01 0.2 : 0.9364103328461886, 0.9662932119211088
0.01 0.4 : 0.9368294481680222, 0.9661867039265146
0.01 0.6 : 0.9356458366160828, 0.9665555339314782
0.01 0.8 : 0.9342008995887048, 0.9681760757767877
0.01 1.0 : 0.9265924851202069, 0.9696154390652002
0.1 0.0 : 0.8779730033360724, 0.9043426531956555
0.1 0.2 : 0.8670230713112241, 0.8932795716792649
0.1 0.4 : 0.8598450753580671, 0.8870848870831783
0.1 0.6 : 0.8559463726630161, 0.8841374468459793
0.1 0.8 : 0.8540418362730131, 0.8872467059560429
0.1 1.0 : 0.8493289236685072, 0.8900174111787528

Grid search of RandomForest, Avalon (2048)
RandomForestRegressor(n_estimators= i)
i: accuracy of prediction using cross validation, accuracy of prediction using test data
100 1.0 : 0.775430602242894, 0.8175090411285557
500 1.0 : 0.7656022411068906, 0.820264686529149
1000 1.0 : 0.7672847041365436, 0.8220815905202781
2000 1.0 : 0.7670979952504459, 0.8229766378914631

Grid search of NeuralNetwork, Avalon (2048)
MLPRegressor(activation=act, alpha=a, batch_size=batch, beta_1=0.9,
beta_2=0.999, early_stopping=False, epsilon=1e-08,
hidden_layer_sizes=hid, learning_rate='constant',
learning_rate_init=0.001, max_iter=100000, momentum=0.9,
n_iter_no_change=10, nesterovs_momentum=True,
power_t=0.5,
random_state=1, shuffle=True, solver='adam', tol=0.0001,
validation_fraction=0.1, verbose=False, warm_start=False)

act (hid) a batch: accuracy of prediction using cross validation, accuracy of prediction using test data

relu (200,) 0.0001 500 : 0.9239391244949002, 0.965916765060451
relu (200,) 0.0001 200 : 0.9272919483639143, 0.9687744209348198
relu (200,) 0.0001 100 : 0.9271907994599111, 0.9665497803847027
relu (200,) 0.0001 50 : 0.9296853232974177, 0.9678764078107066
relu (200,) 0.001 500 : 0.9239042344534351, 0.9659379352902789
relu (200,) 0.001 200 : 0.9270034849439888, 0.9686735677335196
relu (200,) 0.001 100 : 0.9267243365298056, 0.966488832877798
relu (200,) 0.001 50 : 0.9293678407831779, 0.9676643459005252
relu (200,) 0.01 500 : 0.9237656357581134, 0.9660390569291195
relu (200,) 0.01 200 : 0.9276584385266089, 0.9688369176329231
relu (200,) 0.01 100 : 0.927251794675246, 0.9664649839310333
relu (200,) 0.01 50 : 0.9293767625969422, 0.9678967007136591
relu (200, 200) 0.0001 500 : 0.9147182139826684, 0.9658868303004431
relu (200, 200) 0.0001 200 : 0.918519457340387, 0.9662764384469466
relu (200, 200) 0.0001 100 : 0.9220319447185856, 0.9667369758535517
relu (200, 200) 0.0001 50 : 0.9221157953781036, 0.9679256974476653
relu (200, 200) 0.001 500 : 0.9139164768748838, 0.9621572352697635
relu (200, 200) 0.001 200 : 0.9212449916901099, 0.9681193522694128
relu (200, 200) 0.001 100 : 0.9179604056429689, 0.9661612058038135
relu (200, 200) 0.001 50 : 0.9205485776705172, 0.9660500082735894
relu (200, 200) 0.01 500 : 0.9164788139648973, 0.9641797198255828
relu (200, 200) 0.01 200 : 0.9202872516693562, 0.9659931695171359
relu (200, 200) 0.01 100 : 0.921407327325604, 0.9666209582085051
relu (200, 200) 0.01 50 : 0.9207854688053587, 0.9656318657465937
relu (100,) 0.0001 500 : 0.9230731845927844, 0.9679137449816343
relu (100,) 0.0001 200 : 0.9274711273904785, 0.9677689494836105
relu (100,) 0.0001 100 : 0.9281227562786662, 0.968847472822286
relu (100,) 0.0001 50 : 0.9301946357858484, 0.9681246565918522
relu (100,) 0.001 500 : 0.9234485787066335, 0.9680681731633403
relu (100,) 0.001 200 : 0.9275814621418185, 0.9679500110777358
relu (100,) 0.001 100 : 0.9279294659997387, 0.9688798099726235
relu (100,) 0.001 50 : 0.9297572118512708, 0.969037370539658
relu (100,) 0.01 500 : 0.9232407223576932, 0.9682349170266371
relu (100,) 0.01 200 : 0.927796120397827, 0.9679345688395663
relu (100,) 0.01 100 : 0.9286689292723281, 0.9691981388670168
relu (100,) 0.01 50 : 0.9306442424687198, 0.9693508186230575
relu (100, 100) 0.0001 500 : 0.9181697729080829, 0.9665379388599281
relu (100, 100) 0.0001 200 : 0.9257484425527348, 0.9648460518770479
relu (100, 100) 0.0001 100 : 0.9264096197834197, 0.9629345981196283
relu (100, 100) 0.0001 50 : 0.9245192664699209, 0.9691671667476728
relu (100, 100) 0.001 500 : 0.9198705692407831, 0.9652257281768711
relu (100, 100) 0.001 200 : 0.926816784530148, 0.9665186252965007
relu (100, 100) 0.001 100 : 0.9270639316830442, 0.9628018105217865
relu (100, 100) 0.001 50 : 0.92299306915466, 0.9661430699541327
relu (100, 100) 0.01 500 : 0.9172127005100881, 0.9662522367337607
relu (100, 100) 0.01 200 : 0.9255582175766737, 0.9656098655604601
relu (100, 100) 0.01 100 : 0.9251641857048252, 0.962810518554175
relu (100, 100) 0.01 50 : 0.922853205067194, 0.9670284234866522
relu (100, 200) 0.0001 500 : 0.914592708100112, 0.9625138996429868
relu (100, 200) 0.0001 200 : 0.9221907112039112, 0.9667993090542257

relu (100, 200) 0.0001 100 : 0.9162733817984036, 0.9687695420817419
relu (100, 200) 0.0001 50 : 0.921245259984296, 0.9680154560906713
relu (100, 200) 0.001 500 : 0.9140435323476698, 0.9615407319035949
relu (100, 200) 0.001 200 : 0.9234759255406345, 0.9665112141804306
relu (100, 200) 0.001 100 : 0.9155156023102254, 0.966037974810965
relu (100, 200) 0.001 50 : 0.9223461029936841, 0.9642140200536303
relu (100, 200) 0.01 500 : 0.9138788272074603, 0.9655826575143973
relu (100, 200) 0.01 200 : 0.922870337700824, 0.9673925285840131
relu (100, 200) 0.01 100 : 0.9158430265974846, 0.9676889607307141
relu (100, 200) 0.01 50 : 0.9224833063577688, 0.9646541035411111
relu (50,) 0.0001 500 : 0.9238349081742079, 0.9656955313492607
relu (50,) 0.0001 200 : 0.9276730850349724, 0.9672914154064117
relu (50,) 0.0001 100 : 0.9294966439014816, 0.9680555139592691
relu (50,) 0.0001 50 : 0.9311807084473493, 0.9686012322731795
relu (50,) 0.001 500 : 0.9238078173053704, 0.9656991572088685
relu (50,) 0.001 200 : 0.927683216466775, 0.9672909591841311
relu (50,) 0.001 100 : 0.9297005872726334, 0.9680689988083742
relu (50,) 0.001 50 : 0.9312364348747737, 0.9685281689897948
relu (50,) 0.01 500 : 0.9238512347377241, 0.9657447344754135
relu (50,) 0.01 200 : 0.9276665285453143, 0.9675148342615649
relu (50,) 0.01 100 : 0.9302131613640421, 0.968157809297019
relu (50,) 0.01 50 : 0.9318477545656, 0.9689552538617968
relu (50, 50) 0.0001 500 : 0.9163072333359199, 0.9665724719995268
relu (50, 50) 0.0001 200 : 0.9263947643950765, 0.9652807133038507
relu (50, 50) 0.0001 100 : 0.9232012923612032, 0.965614457178032
relu (50, 50) 0.0001 50 : 0.9279982296477659, 0.9628701837607255
relu (50, 50) 0.001 500 : 0.9178877928895715, 0.9661068934568351
relu (50, 50) 0.001 200 : 0.9248434715577929, 0.9641684904205802
relu (50, 50) 0.001 100 : 0.9245133762256673, 0.966298802865762
relu (50, 50) 0.001 50 : 0.9269267773306413, 0.9630118880680603
relu (50, 50) 0.01 500 : 0.9169094393719106, 0.9657404576087286
relu (50, 50) 0.01 200 : 0.9240275010172722, 0.9655285600225252
relu (50, 50) 0.01 100 : 0.9242414590799093, 0.9644841660104748
relu (50, 50) 0.01 50 : 0.9256872364918687, 0.9644755140856636
relu (50, 100) 0.0001 500 : 0.9171755214237003, 0.9675835742186378
relu (50, 100) 0.0001 200 : 0.9229158785183825, 0.9621889157628853
relu (50, 100) 0.0001 100 : 0.9228105571248809, 0.9648183031378468
relu (50, 100) 0.0001 50 : 0.9250392668018133, 0.9669881801234695
relu (50, 100) 0.001 500 : 0.9186286129987995, 0.9649925937068533
relu (50, 100) 0.001 200 : 0.9222129784010639, 0.962673144235374
relu (50, 100) 0.001 100 : 0.9260164919084051, 0.9666682310217279
relu (50, 100) 0.001 50 : 0.9261082494152006, 0.9650664058936741
relu (50, 100) 0.01 500 : 0.9195833602833083, 0.9655809416167783
relu (50, 100) 0.01 200 : 0.9195676629339594, 0.9630314227382469
relu (50, 100) 0.01 100 : 0.9264217728891234, 0.9648068515897911
relu (50, 100) 0.01 50 : 0.926506148124403, 0.9665534334946522
relu (50, 200) 0.0001 500 : 0.9201610134762574, 0.9616109562434662
relu (50, 200) 0.0001 200 : 0.9231158862985399, 0.9600721322041288
relu (50, 200) 0.0001 100 : 0.9259222735912254, 0.9657502468529249
relu (50, 200) 0.0001 50 : 0.9263345367101701, 0.9654406958790926
relu (50, 200) 0.001 500 : 0.9181574727610124, 0.9624207416709781
relu (50, 200) 0.001 200 : 0.9218326082300848, 0.9622478101940921
relu (50, 200) 0.001 100 : 0.9249177874742672, 0.9665724366630721
relu (50, 200) 0.001 50 : 0.9268901482063724, 0.9668300085971726
relu (50, 200) 0.01 500 : 0.9171536151990758, 0.9556651985440472
relu (50, 200) 0.01 200 : 0.9245565522083019, 0.959695263342676
relu (50, 200) 0.01 100 : 0.9255525040654314, 0.9647504364511795
relu (50, 200) 0.01 50 : 0.928666888327134, 0.965040013540988
tanh (200,) 0.0001 500 : 0.8991621861869211, 0.928369564992775
tanh (200,) 0.0001 200 : 0.9016278259275394, 0.9313225424242471
tanh (200,) 0.0001 100 : 0.9014456113818751, 0.9390599903147743
tanh (200,) 0.0001 50 : 0.904397129928979, 0.9438941107287648
tanh (200,) 0.001 500 : 0.8975462732427001, 0.9360751211770432
tanh (200,) 0.001 200 : 0.9004309003100228, 0.9331129010474192
tanh (200,) 0.001 100 : 0.8988919386143776, 0.9381182910778553
tanh (200,) 0.001 50 : 0.9033587292319398, 0.9451178201905384
tanh (200,) 0.01 500 : 0.8972081852049486, 0.9334710368249617
tanh (200,) 0.01 200 : 0.8993320544724355, 0.9373883103878835
tanh (200,) 0.01 100 : 0.8923412407725554, 0.9414547738219838
tanh (200,) 0.01 50 : 0.8999740124436831, 0.9482968205741307
tanh (200, 200) 0.0001 500 : 0.9045239926188839, -0.022311481330170357
tanh (200, 200) 0.0001 200 : 0.3521610645018977, 0.9312919307315506
tanh (200, 200) 0.0001 100 : 0.9140746190631569, 0.9289113738746712
tanh (200, 200) 0.0001 50 : 0.9163115515288307, 0.9257356144246065
tanh (200, 200) 0.001 500 : 0.8968933126997977, -0.022311639103443692
tanh (200, 200) 0.001 200 : 0.3548156739807454, 0.9309713392634393
tanh (200, 200) 0.001 100 : 0.9142322357324971, 0.9295778204012053
tanh (200, 200) 0.001 50 : 0.9192640439117886, 0.9311854192080626
tanh (200, 200) 0.01 500 : 0.9018956099497343, -0.022313185013246528
tanh (200, 200) 0.01 200 : 0.3580099783374484, 0.932975914097483
tanh (200, 200) 0.01 100 : 0.9169527652835783, 0.9436316667923288
tanh (200, 200) 0.01 50 : 0.9147488978263179, 0.9363676097977294
tanh (100,) 0.0001 500 : 0.8912719098232673, 0.940105414665728
tanh (100,) 0.0001 200 : 0.5199686221465415, 0.9425084923505391
tanh (100,) 0.0001 100 : 0.8938275321201976, 0.9478683387560896
tanh (100,) 0.0001 50 : 0.89592018136324, 0.9499875662995402
tanh (100,) 0.001 500 : 0.8875628531651708, 0.9372635111452031
tanh (100,) 0.001 200 : 0.5218502523115253, 0.939319201871374
tanh (100,) 0.001 100 : 0.8934123745079541, 0.945784536958904
tanh (100,) 0.001 50 : 0.894246168868684, 0.9427203819586071
tanh (100,) 0.01 500 : 0.884652070236165, 0.9297730428867954
tanh (100,) 0.01 200 : 0.7046364246755249, 0.9421383220373419
tanh (100,) 0.01 100 : 0.8877287002783977, 0.948363255825757
tanh (100,) 0.01 50 : 0.8955702199353665, 0.9490968402696158
tanh (100, 100) 0.0001 500 : 0.873601200845725, 0.925485225351363
tanh (100, 100) 0.0001 200 : 0.6934888862683477, 0.9443086074083504
tanh (100, 100) 0.0001 100 : 0.8975898664498759, 0.9226552420315273
tanh (100, 100) 0.0001 50 : 0.9004514947997058, 0.9380478749471763
tanh (100, 100) 0.001 500 : 0.8786569945387173, 0.930578498865073
tanh (100, 100) 0.001 200 : 0.6999497472927211, 0.9418777672058275
tanh (100, 100) 0.001 100 : 0.8950914633860203, 0.9204133614613118
tanh (100, 100) 0.001 50 : 0.8780117013745663, 0.9278569170141807
tanh (100, 100) 0.01 500 : 0.8837294707933608, 0.9267635736440312
tanh (100, 100) 0.01 200 : 0.7031256588994261, 0.9397755090290499
tanh (100, 100) 0.01 100 : 0.8930810024249698,

0.9362978515804384
tanh (100, 100) 0.01 50 : 0.8935369195015287, 0.94899920332832
tanh (100, 200) 0.0001 500 : 0.8816186322108278, -0.02175771995487663
tanh (100, 200) 0.0001 200 : 0.8840517323456789, 0.9047126092389544
tanh (100, 200) 0.0001 100 : 0.8796405472679398, 0.9254752933148758
tanh (100, 200) 0.0001 50 : 0.8929059453088606, 0.9397381368684028
tanh (100, 200) 0.001 500 : 0.8817458872829219, -0.021758411959486246
tanh (100, 200) 0.001 200 : 0.8778484353666108, 0.9068931700720347
tanh (100, 200) 0.001 100 : 0.8923626906189159, 0.9304877002663008
tanh (100, 200) 0.001 50 : 0.8923211580145312, 0.9401277623651252
tanh (100, 200) 0.01 500 : 0.8775493567813715, -0.021765256397018318
tanh (100, 200) 0.01 200 : 0.8810188205905651, 0.9058432332999602
tanh (100, 200) 0.01 100 : 0.8819407616328379, 0.9312064971629205
tanh (100, 200) 0.01 50 : 0.8867711467490684, 0.9310547593069453
tanh (50,) 0.0001 500 : 0.891666163771454, -0.019768777963990125
tanh (50,) 0.0001 200 : 0.6994555791765158, 0.9288025881126468
tanh (50,) 0.0001 100 : 0.8892084477952084, 0.9235481830237152
tanh (50,) 0.0001 50 : 0.8797930908892149, 0.9361584860525457
tanh (50,) 0.001 500 : 0.8851974259400667, -0.019768729167797 26
tanh (50,) 0.001 200 : 0.706100440334542, 0.9302676738522462
tanh (50,) 0.001 100 : 0.8821029369246387, 0.9350463523544779
tanh (50,) 0.001 50 : 0.8863770689431361, 0.9413058851245153
tanh (50,) 0.01 500 : 0.8787710859321859, 0.9260881835586351
tanh (50,) 0.01 200 : 0.7033453225017562, 0.9269306707845061
tanh (50,) 0.01 100 : 0.8823396574480518, 0.9308226989870187
tanh (50,) 0.01 50 : 0.878099087148032, 0.9444283559770986
tanh (50, 50) 0.0001 500 : 0.6788049333211533, 0.9099293190553224
tanh (50, 50) 0.0001 200 : 0.5250038739953414, 0.8972713545445878
tanh (50, 50) 0.0001 100 : 0.8794617786860162, 0.917592645453542
tanh (50, 50) 0.0001 50 : 0.8725155609677195, 0.926781671210144
tanh (50, 50) 0.001 500 : 0.6745285980295403, 0.8507454884576046
tanh (50, 50) 0.001 200 : 0.6885955274697231, 0.9061749077447002
tanh (50, 50) 0.001 100 : 0.8721006808171492, 0.9102006941958632
tanh (50, 50) 0.001 50 : 0.8770736563137354, 0.9126069750128449
tanh (50, 50) 0.01 500 : 0.6908822482909385, 0.8893675037837212
tanh (50, 50) 0.01 200 : 0.6792335548543805, 0.9059016544475318
tanh (50, 50) 0.01 100 : 0.8750706051868894, 0.9190552493941794
tanh (50, 50) 0.01 50 : 0.8745853823085298, 0.9022894498441668
tanh (50, 100) 0.0001 500 : 0.16661951534240244, -0.02021022915484383
tanh (50, 100) 0.0001 200 : -0.009024165288577236, -0.01805144988881957
tanh (50, 100) 0.0001 100 : 0.3353484658303503, -0.01707461376953212
tanh (50, 100) 0.0001 50 : 0.5150621733651815, 0.9112162581802334
tanh (50, 100) 0.001 500 : 0.16613839591116364, -0.020210416585588975
tanh (50, 100) 0.001 200 : -0.009024243213992023, -0.018051519942702487
tanh (50, 100) 0.001 100 : 0.3448631371816081, -0.01707474907200801
tanh (50, 100) 0.001 50 : 0.3371316325182048, 0.9186086181832767
tanh (50, 100) 0.01 500 : 0.1685839588210632, -0.020212285525399665
tanh (50, 100) 0.01 200 : -0.009025321958184885, -0.01805221779048538
tanh (50, 100) 0.01 100 : -0.008299734274662329, -0.017076180556054865
tanh (50, 100) 0.01 50 : 0.323450426994944, -0.01797596957992642
tanh (50, 200) 0.0001 500 : 0.16185036565522287, -0.01499421745707541
tanh (50, 200) 0.0001 200 : -0.010376831808190401, -0.016465712601599014
tanh (50, 200) 0.0001 100 : 0.16202546881982222, 0.9044489174356496
tanh (50, 200) 0.0001 50 : 0.5231751305894126, 0.8566240002337574
tanh (50, 200) 0.001 500 : 0.16066298963414113, -0.014994332185208181
tanh (50, 200) 0.001 200 : -0.010377049797717763, -0.016465765105734942
tanh (50, 200) 0.001 100 : 0.16467257121195322, 0.9276493171509319
tanh (50, 200) 0.001 50 : 0.5212505780244993, 0.9020622438909939
tanh (50, 200) 0.01 500 : 0.15592255594025697, -0.014995536908506857
tanh (50, 200) 0.01 200 : -0.0103793171439448, -0.01646623092697208
tanh (50, 200) 0.01 100 : 0.16433521949169363, -0.015126331731186493
tanh (50, 200) 0.01 50 : 0.3437397467753432, 0.8772925693132485
logistic (200,) 0.0001 500 : 0.8993718321632667, 0.933631358344002
logistic (200,) 0.0001 200 : 0.9079767482358042, 0.9314198040820159
logistic (200,) 0.0001 100 : 0.9107778106912299, 0.9349433286258124
logistic (200,) 0.0001 50 : 0.9133058438608319, 0.9389572982916751
logistic (200,) 0.001 500 : 0.8982473768619421, 0.9373383733254484
logistic (200,) 0.001 200 : 0.9061837201597751, 0.9336237963560248
logistic (200,) 0.001 100 : 0.9066560440620896, 0.9396057294622755
logistic (200,) 0.001 50 : 0.9109881247842561, 0.9415257502390274
logistic (200,) 0.01 500 : 0.8922802757089764, 0.9453824485190824
logistic (200,) 0.01 200 : 0.903722212445351, 0.9417653553452101
logistic (200,) 0.01 100 : 0.9022549240505665, 0.945085592029564
logistic (200,) 0.01 50 : 0.9069386736708853, 0.9483991317466163
logistic (200, 200) 0.0001 500 : 0.8779770881099518, 0.9199627520099402
logistic (200, 200) 0.0001 200 : 0.7164973948938342, 0.9279486272404496
logistic (200, 200) 0.0001 100 : 0.9059156062688078, 0.9365980320986053
logistic (200, 200) 0.0001 50 : 0.9036201584508664, 0.9331399026525424
logistic (200, 200) 0.001 500 : 0.870608405475046, 0.93665419531493
logistic (200, 200) 0.001 200 : 0.7151022655624232, 0.9234760699895722
logistic (200, 200) 0.001 100 : 0.8913332920055206, 0.9408026564486838
logistic (200, 200) 0.001 50 : 0.893024694624884, 0.9427194013534141
logistic (200, 200) 0.01 500 : 0.8623444104884868, 0.9323768336996241
logistic (200, 200) 0.01 200 : 0.7135422084337054, 0.930793056700131
logistic (200, 200) 0.01 100 : 0.8924705803177566, 0.9434742665411775
logistic (200, 200) 0.01 50 : 0.901855496001675, 0.9482040714044648
logistic (100,) 0.0001 500 : 0.8938028434935937, 0.9328665290688418
logistic (100,) 0.0001 200 : 0.9014548983907925, 0.9333960904719266
logistic (100,) 0.0001 100 : 0.9008428568070892, 0.9380661692641727
logistic (100,) 0.0001 50 : 0.9011052610402871, 0.9401330440431903
logistic (100,) 0.001 500 : 0.8930077932219925, 0.9346815102041989

logistic (100,) 0.001 200 : 0.89787678570616, 0.9349197112546982
logistic (100,) 0.001 100 : 0.9007183176509003,
0.9377574802933802
logistic (100,) 0.001 50 : 0.897902047065745, 0.9411942301897813
logistic (100,) 0.01 500 : 0.8867118306253993, 0.9428584937954908
logistic (100,) 0.01 200 : 0.8929530754178149, 0.9434487205226681
logistic (100,) 0.01 100 : 0.8960278155496635, 0.9464023327403811
logistic (100,) 0.01 50 : 0.8986795532665864, 0.9418023628408758
logistic (100, 100) 0.0001 500 : 0.8873942276916786,
0.9206533477172877
logistic (100, 100) 0.0001 200 : 0.8894953087163919,
0.9366504287276151
logistic (100, 100) 0.0001 100 : 0.8895877857103075,
0.9362138540960547
logistic (100, 100) 0.0001 50 : 0.906419185636893,
0.9456735956661856
logistic (100, 100) 0.001 500 : 0.8799934348894922,
0.9243465407271985
logistic (100, 100) 0.001 200 : 0.8979400578882608,
0.9327698455736022
logistic (100, 100) 0.001 100 : 0.8976600917395798,
0.9355610759186144
logistic (100, 100) 0.001 50 : 0.9022290847494328,
0.9471965493905811
logistic (100, 100) 0.01 500 : 0.8853286388106152,
0.9440168489255483
logistic (100, 100) 0.01 200 : 0.5291164309441714,
0.9312700608563528
logistic (100, 100) 0.01 100 : 0.894382718542271,
0.9425156330821612
logistic (100, 100) 0.01 50 : 0.8946809196200413,
0.9558238484795583
logistic (100, 200) 0.0001 500 : 0.34460472221090843, -
0.025398257262529755
logistic (100, 200) 0.0001 200 : -0.007988027532425912,
0.9200198717047084
logistic (100, 200) 0.0001 100 : 0.16989238010247587, -
0.018340480132115422
logistic (100, 200) 0.0001 50 : 0.16153568020295506, -
0.016952891876917953
logistic (100, 200) 0.001 500 : 0.34657615565743516, -
0.025398654735290016
logistic (100, 200) 0.001 200 : -0.007988609537088465,
0.9105604464427925
logistic (100, 200) 0.001 100 : 0.1658562039152776, -
0.01834078840260367
logistic (100, 200) 0.001 50 : 0.1631295577207228, -
0.01696073051829683
logistic (100, 200) 0.01 500 : 0.17545896375693643, -
0.025402650241429736
logistic (100, 200) 0.01 200 : -0.007993604020882606, -
0.017867570952819323
logistic (100, 200) 0.01 100 : -0.009269662063241712, -
0.018343916474934607
logistic (100, 200) 0.01 50 : 0.156827329732324, -
0.01696565078547363
logistic (50,) 0.0001 500 : 0.8882245374211749,
0.9325073708369939
logistic (50,) 0.0001 200 : 0.8939078786266117,
0.9372683893907909
logistic (50,) 0.0001 100 : 0.8958246920280931,
0.9311580045075131
logistic (50,) 0.0001 50 : 0.8987242553250561, 0.9386651814039622
logistic (50,) 0.001 500 : 0.8898787965732685, 0.9337836340368604
logistic (50,) 0.001 200 : 0.8905973633024548, 0.9334672044942771
logistic (50,) 0.001 100 : 0.8892464535991461, 0.938999991800707
logistic (50,) 0.001 50 : 0.8962336409739928, 0.9495733235384948
logistic (50,) 0.01 500 : 0.8836653423454865, 0.934504201915279
logistic (50,) 0.01 200 : 0.892002464272284, 0.9429744374732297
logistic (50,) 0.01 100 : 0.8952690363757598, 0.9477387339573833
logistic (50,) 0.01 50 : 0.9029164899306019, 0.9493983871495162
logistic (50, 50) 0.0001 500 : 0.8876653969087936,
0.9176046878900033
logistic (50, 50) 0.0001 200 : 0.8953539597109472,
0.9251673559424386
logistic (50, 50) 0.0001 100 : 0.8823980201222957,
0.9353216316052136
logistic (50, 50) 0.0001 50 : 0.895497188149166,
0.9479802419002604
logistic (50, 50) 0.001 500 : 0.880028777417986,
0.9299198881991688
logistic (50, 50) 0.001 200 : 0.8965056998239487,
0.9348614870141906
logistic (50, 50) 0.001 100 : 0.8942883234103904,
0.9385669109118109
logistic (50, 50) 0.001 50 : 0.9035136266575314,
0.9368723067266721
logistic (50, 50) 0.01 500 : 0.8706131920002121,
0.9441541811028072
logistic (50, 50) 0.01 200 : 0.8924347664111568,
0.9425354761574726
logistic (50, 50) 0.01 100 : 0.894027085999527,
0.9486896836050519
logistic (50, 50) 0.01 50 : 0.8958724750056378, 0.949494073887905
logistic (50, 100) 0.0001 500 : 0.8482044810039978,
0.9123916204668335
logistic (50, 100) 0.0001 200 : 0.6835798240690114,
0.8990746279324767
logistic (50, 100) 0.0001 100 : 0.8621987579502897,
0.9230367270769388
logistic (50, 100) 0.0001 50 : 0.866988602524273,
0.9297219755058371
logistic (50, 100) 0.001 500 : 0.8527598709364955,
0.9116327147473529
logistic (50, 100) 0.001 200 : 0.6692087309953483,
0.903767003635751
logistic (50, 100) 0.001 100 : 0.8728906438473525,
0.9186720089321898
logistic (50, 100) 0.001 50 : 0.8832111932600071,
0.922720895465477
logistic (50, 100) 0.01 500 : 0.8557260176105366, -
0.02156720316909788
logistic (50, 100) 0.01 200 : 0.33970614397470567,
0.9167638805873207
logistic (50, 100) 0.01 100 : 0.8836168359181457,
0.9332458156037051
logistic (50, 100) 0.01 50 : 0.8852081009112982,
0.9517245281554972
logistic (50, 200) 0.0001 500 : -0.008850160831529497, -
0.022754605410156614
logistic (50, 200) 0.0001 200 : -0.00887034488378644, -
0.01718541686858055
logistic (50, 200) 0.0001 100 : -0.008992595412193705, -
0.018950828226613536
logistic (50, 200) 0.0001 50 : -0.008423927478183702, -
0.018048268168758153
logistic (50, 200) 0.001 500 : -0.008850269423563572, -
0.02275499132495251
logistic (50, 200) 0.001 200 : -0.00887080249834855, -
0.01718554457635446
logistic (50, 200) 0.001 100 : -0.008993234938570271, -
0.018951132064306098
logistic (50, 200) 0.001 50 : -0.008425040053925947, -
0.018048717929039615
logistic (50, 200) 0.01 500 : -0.008851575229721709, -
0.022759253066926277
logistic (50, 200) 0.01 200 : -0.008874940716274926, -
0.0171868833596307
logistic (50, 200) 0.01 100 : -0.008999687540393797, -
0.018954237862316425
logistic (50, 200) 0.01 50 : -0.008437627745627552, -
0.0180533434103487

Grid search of LightGBM, Avalon (2048)
lgb.LGBMRegressor(boosting_type = "gbdt", num_leaves = j,max_depth = 0)
j: accuracy of prediction using cross validation, accuracy of prediction using test data

10 : 0.8492300529046304, 0.9043889465398468
50 : 0.8395687283424719, 0.9192600160657611
100 : 0.8395687283424719, 0.9192600160657611
150 : 0.8395687283424719, 0.9192600160657611

Grid search of SVR, Avalon(2048)
kernel, C, gammma, epsilon
SVR(C= c_num, kernel = ker, epsilon = e, gamma = r,degree = 3, coef0=1)
Ker c_num r e: accuracy of prediction using cross validation, accuracy of prediction using test data
linear 1 1 0.1 : 0.9360696484571296, 0.9716078905798607
linear 1 1 0.3 : 0.936105991301303, 0.9725447158753703
linear 1 1 0.5 : 0.9357022610555175, 0.9722141902376856
linear 1 1 0.7 : 0.9347184355224132, 0.9716324589666623
linear 1 1 0.9 : 0.9332591504255634, 0.970578000089712
linear 1 2 0.1 : 0.9360696484571296, 0.9716078905798607
linear 1 2 0.3 : 0.936105991301303, 0.9725447158753703
linear 1 2 0.5 : 0.9357022610555175, 0.9722141902376856
linear 1 2 0.7 : 0.9347184355224132, 0.9716324589666623
linear 1 2 0.9 : 0.9332591504255634, 0.970578000089712
linear 1 3 0.1 : 0.9360696484571296, 0.9716078905798607
linear 1 3 0.3 : 0.936105991301303, 0.9725447158753703
linear 1 3 0.5 : 0.9357022610555175, 0.9722141902376856
linear 1 3 0.7 : 0.9347184355224132, 0.9716324589666623
linear 1 3 0.9 : 0.9332591504255634, 0.970578000089712
linear 1 4 0.1 : 0.9360696484571296, 0.9716078905798607
linear 1 4 0.3 : 0.936105991301303, 0.9725447158753703
linear 1 4 0.5 : 0.9357022610555175, 0.9722141902376856
linear 1 4 0.7 : 0.9347184355224132, 0.9716324589666623
linear 1 4 0.9 : 0.9332591504255634, 0.970578000089712
linear 1 5 0.1 : 0.9360696484571296, 0.9716078905798607
linear 1 5 0.3 : 0.936105991301303, 0.9725447158753703
linear 1 5 0.5 : 0.9357022610555175, 0.9722141902376856
linear 1 5 0.7 : 0.9347184355224132, 0.9716324589666623
linear 1 5 0.9 : 0.9332591504255634, 0.970578000089712
linear 1 6 0.1 : 0.9360696484571296, 0.9716078905798607
linear 1 6 0.3 : 0.936105991301303, 0.9725447158753703
linear 1 6 0.5 : 0.9357022610555175, 0.9722141902376856
linear 1 6 0.7 : 0.9347184355224132, 0.9716324589666623
linear 1 6 0.9 : 0.9332591504255634, 0.970578000089712
linear 2 1 0.1 : 0.9428257362899457, 0.9733816442243594
linear 2 1 0.3 : 0.9432932948497175, 0.9742675400371666
linear 2 1 0.5 : 0.9423946003100887, 0.973880627113739
linear 2 1 0.7 : 0.9410525108833285, 0.974027974523664
linear 2 1 0.9 : 0.9394229211960058, 0.9725156097729755
linear 2 2 0.1 : 0.9428257362899457, 0.9733816442243594
linear 2 2 0.3 : 0.9432932948497175, 0.9742675400371666
linear 2 2 0.5 : 0.9423946003100887, 0.973880627113739
linear 2 2 0.7 : 0.9410525108833285, 0.974027974523664
linear 2 2 0.9 : 0.9394229211960058, 0.9725156097729755
linear 2 3 0.1 : 0.9428257362899457, 0.9733816442243594
linear 2 3 0.3 : 0.9432932948497175, 0.9742675400371666
linear 2 3 0.5 : 0.9423946003100887, 0.973880627113739
linear 2 3 0.7 : 0.9410525108833285, 0.974027974523664
linear 2 3 0.9 : 0.9394229211960058, 0.9725156097729755
linear 2 4 0.1 : 0.9428257362899457, 0.9733816442243594
linear 2 4 0.3 : 0.9432932948497175, 0.9742675400371666
linear 2 4 0.5 : 0.9423946003100887, 0.973880627113739
linear 2 4 0.7 : 0.9410525108833285, 0.974027974523664
linear 2 4 0.9 : 0.9394229211960058, 0.9725156097729755
linear 2 5 0.1 : 0.9428257362899457, 0.9733816442243594
linear 2 5 0.3 : 0.9432932948497175, 0.9742675400371666
linear 2 5 0.5 : 0.9423946003100887, 0.973880627113739
linear 2 5 0.7 : 0.9410525108833285, 0.974027974523664
linear 2 5 0.9 : 0.9394229211960058, 0.9725156097729755
linear 2 6 0.1 : 0.9428257362899457, 0.9733816442243594
linear 2 6 0.3 : 0.9432932948497175, 0.9742675400371666
linear 2 6 0.5 : 0.9423946003100887, 0.973880627113739
linear 2 6 0.7 : 0.9410525108833285, 0.974027974523664
linear 2 6 0.9 : 0.9394229211960058, 0.9725156097729755
linear 3 1 0.1 : 0.9431746520249404, 0.9732664658521786
linear 3 1 0.3 : 0.9437138242803964, 0.9743482876428643
linear 3 1 0.5 : 0.9430046392997955, 0.9734792235677683
linear 3 1 0.7 : 0.9417367800270998, 0.9730094979156861
linear 3 1 0.9 : 0.9399292954853745, 0.9714167441436147
linear 3 2 0.1 : 0.9431746520249404, 0.9732664658521786
linear 3 2 0.3 : 0.9437138242803964, 0.9743482876428643
linear 3 2 0.5 : 0.9430046392997955, 0.9734792235677683
linear 3 2 0.7 : 0.9417367800270998, 0.9730094979156861
linear 3 2 0.9 : 0.9399292954853745, 0.9714167441436147
linear 3 3 0.1 : 0.9431746520249404, 0.9732664658521786
linear 3 3 0.3 : 0.9437138242803964, 0.9743482876428643
linear 3 3 0.5 : 0.9430046392997955, 0.9734792235677683
linear 3 3 0.7 : 0.9417367800270998, 0.9730094979156861
linear 3 3 0.9 : 0.9399292954853745, 0.9714167441436147
linear 3 4 0.1 : 0.9431746520249404, 0.9732664658521786
linear 3 4 0.3 : 0.9437138242803964, 0.9743482876428643
linear 3 4 0.5 : 0.9430046392997955, 0.9734792235677683
linear 3 4 0.7 : 0.9417367800270998, 0.9730094979156861
linear 3 4 0.9 : 0.9399292954853745, 0.9714167441436147
linear 3 5 0.1 : 0.9431746520249404, 0.9732664658521786
linear 3 5 0.3 : 0.9437138242803964, 0.9743482876428643
linear 3 5 0.5 : 0.9430046392997955, 0.9734792235677683
linear 3 5 0.7 : 0.9417367800270998, 0.9730094979156861
linear 3 5 0.9 : 0.9399292954853745, 0.9714167441436147
linear 3 6 0.1 : 0.9431746520249404, 0.9732664658521786
linear 3 6 0.3 : 0.9437138242803964, 0.9743482876428643
linear 3 6 0.5 : 0.9430046392997955, 0.9734792235677683
linear 3 6 0.7 : 0.9417367800270998, 0.9730094979156861
linear 3 6 0.9 : 0.9399292954853745, 0.9714167441436147
linear 4 1 0.1 : 0.942189353345692, 0.9732137107306217
linear 4 1 0.3 : 0.942948825483478, 0.9745268066865317
linear 4 1 0.5 : 0.9424484057041067, 0.9734800652786395
linear 4 1 0.7 : 0.9413726500329554, 0.9727467461789768
linear 4 1 0.9 : 0.93962345892601, 0.9711282486246055
linear 4 2 0.1 : 0.942189353345692, 0.9732137107306217
linear 4 2 0.3 : 0.942948825483478, 0.9745268066865317
linear 4 2 0.5 : 0.9424484057041067, 0.9734800652786395
linear 4 2 0.7 : 0.9413726500329554, 0.9727467461789768
linear 4 2 0.9 : 0.93962345892601, 0.9711282486246055
linear 4 3 0.1 : 0.942189353345692, 0.9732137107306217
linear 4 3 0.3 : 0.942948825483478, 0.9745268066865317
linear 4 3 0.5 : 0.9424484057041067, 0.9734800652786395
linear 4 3 0.7 : 0.9413726500329554, 0.9727467461789768
linear 4 3 0.9 : 0.93962345892601, 0.9711282486246055
linear 4 4 0.1 : 0.942189353345692, 0.9732137107306217
linear 4 4 0.3 : 0.942948825483478, 0.9745268066865317
linear 4 4 0.5 : 0.9424484057041067, 0.9734800652786395
linear 4 4 0.7 : 0.9413726500329554, 0.9727467461789768
linear 4 4 0.9 : 0.93962345892601, 0.9711282486246055
linear 4 5 0.1 : 0.942189353345692, 0.9732137107306217
linear 4 5 0.3 : 0.942948825483478, 0.9745268066865317
linear 4 5 0.5 : 0.9424484057041067, 0.9734800652786395
linear 4 5 0.7 : 0.9413726500329554, 0.9727467461789768
linear 4 5 0.9 : 0.93962345892601, 0.9711282486246055
linear 4 6 0.1 : 0.942189353345692, 0.9732137107306217
linear 4 6 0.3 : 0.942948825483478, 0.9745268066865317
linear 4 6 0.5 : 0.9424484057041067, 0.9734800652786395
linear 4 6 0.7 : 0.9413726500329554, 0.9727467461789768
linear 4 6 0.9 : 0.93962345892601, 0.9711282486246055
poly 1 1 0.1 : 0.8834629470578992, 0.9293691844732405
poly 1 1 0.3 : 0.8812504714321298, 0.9269885646360989
poly 1 1 0.5 : 0.8787033044421542, 0.9245385125951628
poly 1 1 0.7 : 0.876104884857298, 0.9218956042705791
poly 1 1 0.9 : 0.8731917785641565, 0.9190241760649875
poly 1 2 0.1 : 0.8832220945267165, 0.9291136018868043
poly 1 2 0.3 : 0.8810113542500962, 0.926731849309957
poly 1 2 0.5 : 0.8784914342794317, 0.9242786056859094
poly 1 2 0.7 : 0.8758692694919674, 0.9216366905827564
poly 1 2 0.9 : 0.8729584741339547, 0.9187603238541608
poly 1 3 0.1 : 0.883141441088806, 0.9290274831500698
poly 1 3 0.3 : 0.8809312354205379, 0.9266455677458489
poly 1 3 0.5 : 0.8784115117358429, 0.9241912952669679
poly 1 3 0.7 : 0.8757899273419973, 0.9215495942300785
poly 1 3 0.9 : 0.87288045378955, 0.9186709103608008
poly 1 4 0.1 : 0.8831007246388536, 0.9289846506720918
poly 1 4 0.3 : 0.8808909987486044, 0.9266021932259222

poly 1 4 0.5 : 0.8783714811896518, 0.9241473793674713
poly 1 4 0.7 : 0.8757509101682036, 0.9215060752642287
poly 1 4 0.9 : 0.8728406044684155, 0.9186267612092692
poly 1 5 0.1 : 0.8830766872399212, 0.9289589545929816
poly 1 5 0.3 : 0.8808671059501532, 0.9265762030255448
poly 1 5 0.5 : 0.8783474053564756, 0.9241210275503036
poly 1 5 0.7 : 0.8757270039302935, 0.9214795204609996
poly 1 5 0.9 : 0.872817496651128, 0.9185995702771107
poly 1 6 0.1 : 0.8830598512162633, 0.928941423122018
poly 1 6 0.3 : 0.880850633509952, 0.9265586604581793
poly 1 6 0.5 : 0.8783314331241737, 0.9241032612911798
poly 1 6 0.7 : 0.8757108920041633, 0.9214620315023243
poly 1 6 0.9 : 0.8728012183490182, 0.9185822447245493
poly 2 1 0.1 : 0.8834629277777001, 0.9293691843721057
poly 2 1 0.3 : 0.881250491755217, 0.9269885645900213
poly 2 1 0.5 : 0.8787303304141793, 0.9245385126219885
poly 2 1 0.7 : 0.8761047093733758, 0.9218956042837564
poly 2 1 0.9 : 0.8731917785641565, 0.9190241760649875
poly 2 2 0.1 : 0.8832220947145561, 0.9291136015972522
poly 2 2 0.3 : 0.8810115669972634, 0.9267317690428554
poly 2 2 0.5 : 0.8784913569585806, 0.924278678275006
poly 2 2 0.7 : 0.8758692694965831, 0.921636689750208
poly 2 2 0.9 : 0.8729584741339547, 0.9187603238541608
poly 2 3 0.1 : 0.8831414417337772, 0.9290274844923747
poly 2 3 0.3 : 0.8809313960332513, 0.926645565626311
poly 2 3 0.5 : 0.8784116492312183, 0.9241912968375344
poly 2 3 0.7 : 0.8757901447061336, 0.9215495957618041
poly 2 3 0.9 : 0.87288045378955, 0.9186709103608008
poly 2 4 0.1 : 0.8831005912653008, 0.9289846508824028
poly 2 4 0.3 : 0.8808910938718928, 0.9266022530758999
poly 2 4 0.5 : 0.8783715630256621, 0.9241473691259436
poly 2 4 0.7 : 0.8757510239203921, 0.9215060709203354
poly 2 4 0.9 : 0.8728406044684155, 0.9186267612092692
poly 2 5 0.1 : 0.8830764744139017, 0.9289589546363008
poly 2 5 0.3 : 0.8808669668834936, 0.9265761217376598
poly 2 5 0.5 : 0.8783473630254882, 0.9241210830814806
poly 2 5 0.7 : 0.8757270056159528, 0.9214795124760609
poly 2 5 0.9 : 0.872817496651128, 0.9185995702771107
poly 2 6 0.1 : 0.8830596786203879, 0.9289414226410363
poly 2 6 0.3 : 0.8808506052246393, 0.9265582714795808
poly 2 6 0.5 : 0.8783314415615926, 0.924103463972849
poly 2 6 0.7 : 0.8757109395684864, 0.9214619432723528
poly 2 6 0.9 : 0.8728012183490182, 0.9185822447245493
poly 3 1 0.1 : 0.883462893692478, 0.9293691844540636
poly 3 1 0.3 : 0.8812504917537158, 0.9269885646532237
poly 3 1 0.5 : 0.8787303303704844, 0.9245385124887798
poly 3 1 0.7 : 0.8761047093972085, 0.9218956042132971
poly 3 1 0.9 : 0.8731917785641565, 0.9190241760649875
poly 3 2 0.1 : 0.8832220944365913, 0.9291136016790678
poly 3 2 0.3 : 0.881011487960633, 0.9267318507730405
poly 3 2 0.5 : 0.8784913572825207, 0.9242786055581278
poly 3 2 0.7 : 0.8758692695608035, 0.9216366894878236
poly 3 2 0.9 : 0.8729584741339547, 0.9187603238541608
poly 3 3 0.1 : 0.8831414415722826, 0.9290274841094076
poly 3 3 0.3 : 0.8809311518088638, 0.9266455654642526
poly 3 3 0.5 : 0.8784116498318454, 0.9241912931077997
poly 3 3 0.7 : 0.8757899646265228, 0.9215495929076264
poly 3 3 0.9 : 0.87288045378955, 0.9186709103608008
poly 3 4 0.1 : 0.883100789079158, 0.9289846651774395
poly 3 4 0.3 : 0.8808911900868456, 0.9266022463310054
poly 3 4 0.5 : 0.8783714394582077, 0.9241472354672549
poly 3 4 0.7 : 0.8757508891939784, 0.9215055923154215
poly 3 4 0.9 : 0.8728406044684155, 0.9186267612092692
poly 3 5 0.1 : 0.8830763306630762, 0.9289589541467053
poly 3 5 0.3 : 0.8808670499795281, 0.926576218554094
poly 3 5 0.5 : 0.8783476407247359, 0.9241214595228954
poly 3 5 0.7 : 0.8757267985888418, 0.9214796229754654
poly 3 5 0.9 : 0.872817496651128, 0.9185995702771107
poly 3 6 0.1 : 0.8830596921018301, 0.9289418232578647
poly 3 6 0.3 : 0.8808507239078753, 0.9265588308459147
poly 3 6 0.5 : 0.8783313670378611, 0.9241031868812035
poly 3 6 0.7 : 0.8757112462998677, 0.9214620227644397
poly 3 6 0.9 : 0.8728012183490182, 0.9185822447245493
poly 4 1 0.1 : 0.8834628937708887, 0.9293691844191865
poly 4 1 0.3 : 0.8812504916294589, 0.9269885648321353
poly 4 1 0.5 : 0.8787303304239286, 0.9245385126252468
poly 4 1 0.7 : 0.876104709467991, 0.921895604293211
poly 4 1 0.9 : 0.8731917785641565, 0.9190241760649875
poly 4 2 0.1 : 0.8832217632292266, 0.9291135340427508
poly 4 2 0.3 : 0.8810114872380627, 0.9267318498194942
poly 4 2 0.5 : 0.8784913581378323, 0.9242786060100848
poly 4 2 0.7 : 0.8758692692753975, 0.9216366909648296
poly 4 2 0.9 : 0.8729584741339547, 0.9187603238541608
poly 4 3 0.1 : 0.8831414418091036, 0.929027484376433
poly 4 3 0.3 : 0.8809311499703831, 0.926645570899959
poly 4 3 0.5 : 0.8784113981913804, 0.9241912923201215
poly 4 3 0.7 : 0.8757899625911415, 0.9215495957964763
poly 4 3 0.9 : 0.87288045378955, 0.9186709103608008
poly 4 4 0.1 : 0.8831007928240519, 0.9289846672905608
poly 4 4 0.3 : 0.8808911917868942, 0.9266022409983508
poly 4 4 0.5 : 0.8783714388990373, 0.9241472349873276
poly 4 4 0.7 : 0.875750888441002, 0.9215056041307019
poly 4 4 0.9 : 0.8728406044684155, 0.9186267612092692
poly 4 5 0.1 : 0.883076214327436, 0.9289589681730837
poly 4 5 0.3 : 0.880866853537257, 0.9265762214482695
poly 4 5 0.5 : 0.8783473801021753, 0.9241211614065693
poly 4 5 0.7 : 0.8757267920228632, 0.9214795852589038
poly 4 5 0.9 : 0.872817496651128, 0.9185995702771107
poly 4 6 0.1 : 0.8830596800420049, 0.9289418340488913
poly 4 6 0.3 : 0.8808506817218401, 0.9265585045227152
poly 4 6 0.5 : 0.8783313605356229, 0.9241031979946436
poly 4 6 0.7 : 0.8757112279699207, 0.9214620379559295
poly 4 6 0.9 : 0.8728012183490182, 0.9185822447245493
rbf 1 1 0.1 : -0.02734012689590899, 0.0016210057574772563
rbf 1 1 0.3 : -0.02708604064992981, 0.0015896772952309624
rbf 1 1 0.5 : -0.026481396148583202, 0.0015196270054786964
rbf 1 1 0.7 : -0.02568712790327332, 0.0014726538480190898
rbf 1 1 0.9 : -0.024429215434216677, 0.0014417839873385185
rbf 1 2 0.1 : -0.027372215022877056, 0.0015679390323573017
rbf 1 2 0.3 : -0.02711810912591024, 0.0015365704528821356
rbf 1 2 0.5 : -0.0265133536054059, 0.001466439062220326
rbf 1 2 0.7 : -0.025719019812105247, 0.0014193827953242755
rbf 1 2 0.9 : -0.024461098674682092, 0.0013884652134702469
rbf 1 3 0.1 : -0.02737360214988751, 0.00156539259929811
rbf 1 3 0.3 : -0.027119496131317568, 0.0015340222684231408
rbf 1 3 0.5 : -0.026514739904656536, 0.001463887320569679
rbf 1 3 0.7 : -0.025720404740877934, 0.001416828870442477
rbf 1 3 0.9 : -0.0244624815418657, 0.001385909930675222
rbf 1 4 0.1 : -0.027373670458769796, 0.0015652660198993695
rbf 1 4 0.3 : -0.02711956443403034, 0.001533895603255564
rbf 1 4 0.5 : -0.026514808175630344, 0.0014637604812011151
rbf 1 4 0.7 : -0.025720472946108998, 0.0014167019246953405
rbf 1 4 0.9 : -0.02446254964461252, 0.0013857829186980641
rbf 1 5 0.1 : -0.027373673855745694, 0.0015652597191427864
rbf 1 5 0.3 : -0.027119567830696932, 0.001533889298238389
rbf 1 5 0.5 : -0.026514811570730858, 0.0014637541675301957
rbf 1 5 0.7 : -0.02572047633795056, 0.0014166956057404256
rbf 1 5 0.9 : -0.0244625530313586, 0.0013857765964531144
rbf 1 6 0.1 : -0.027373674024847406, 0.001565259405455044
rbf 1 6 0.3 : -0.027119567999783056, 0.001533888984338927
rbf 1 6 0.5 : -0.02651481173973922, 0.0014637538531997452
rbf 1 6 0.7 : -0.025720476506796965, 0.0014166952911468522
rbf 1 6 0.9 : -0.024462553199951255, 0.001385776281695894
rbf 2 1 0.1 : -0.024661064942118217, 0.0031687499963555243
rbf 2 1 0.3 : -0.02397356550621419, 0.003093529329623479
rbf 2 1 0.5 : -0.02329362173544185, 0.002993953071836475
rbf 2 1 0.7 : -0.022486488096949798, 0.0028753291743740528
rbf 2 1 0.9 : -0.02174082400175803, 0.002749805930603366
rbf 2 2 0.1 : -0.02472478082762648, 0.0030622780659786963
rbf 2 2 0.3 : -0.024037218530813354, 0.002986930726203707
rbf 2 2 0.5 : -0.023357470765286336, 0.0028871931717452393
rbf 2 2 0.7 : -0.022550285188141304, 0.002768394214054948
rbf 2 2 0.9 : -0.02180457954346173, 0.0026427095086566377
rbf 2 3 0.1 : -0.024727547597971133, 0.0030571681916378157
rbf 2 3 0.3 : -0.024039980566746656, 0.002981814837868746
rbf 2 3 0.5 : -0.023360228798467907, 0.0028820699803637773
rbf 2 3 0.7 : -0.022553039700888667, 0.002763263078342404
rbf 2 3 0.9 : -0.02180733125110308, 0.0026375706566402757

rbf 2 4 0.1 : -0.024727683866720352, 0.0030569141878380446
rbf 2 4 0.3 : -0.024040116597695206, 0.002981560539366157
rbf 2 4 0.5 : -0.023360364615945928, 0.0028818153241161326
rbf 2 4 0.7 : -0.02255317533997898, 0.0027630080329298368
rbf 2 4 0.9 : -0.0218074667477969, 0.0026373152331801153
rbf 2 5 0.1 : -0.024727690643361732, 0.0030569015443161485
rbf 2 5 0.3 : -0.02404012336248833, 0.002981547881204638
rbf 2 5 0.5 : -0.023360371370084863, 0.0028818026481831627
rbf 2 5 0.7 : -0.02255318208521877, 0.002762995337664886 4
rbf 2 5 0.9 : -0.021807473485925665, 0.0026373025191354094
rbf 2 6 0.1 : -0.02472769098070362, 0.0030569009148500026
rbf 2 6 0.3 : -0.024040123699240246, 0.002981547251009631
rbf 2 6 0.5 : -0.02336037170630627, 0.002881802017103751 7
rbf 2 6 0.7 : -0.02255318242099693, 0.00276299470562324 5
rbf 2 6 0.9 : -0.02180747382134953, 0.0026373018861589603
rbf 3 1 0.1 : -0.021800297117375144, 0.004339967166779424
rbf 3 1 0.3 : -0.021044621339336756, 0.004233086058879176
rbf 3 1 0.5 : -0.020146183559048625, 0.004084989938599404
rbf 3 1 0.7 : -0.0190960870279576, 0.003834125086157658
rbf 3 1 0.9 : -0.018009981457142877, 0.003516852306964746
rbf 3 2 0.1 : -0.021895747942134225, 0.0041795667779518375
rbf 3 2 0.3 : -0.021140055706706917, 0.0040724944556859155
rbf 3 2 0.5 : -0.020241603817343322, 0.003924146696415831
rbf 3 2 0.7 : -0.019191366460229897, 0.003672890297110487
rbf 3 2 0.9 : -0.018105238858803087, 0.003355165307716068
rbf 3 3 0.1 : -0.021899901222314666, 0.004171856556901443
rbf 3 3 0.3 : -0.021144185399004735, 0.004064775384723052
rbf 3 3 0.5 : -0.02024569618750589, 0.00391641609706872
rbf 3 3 0.7 : -0.019195486110171744, 0.0036651417198586556
rbf 3 3 0.9 : -0.018109314602109405, 0.003347396149577686
rbf 3 4 0.1 : -0.02190009184731432, 0.004171473301388784
rbf 3 4 0.3 : -0.02114435962015433, 0.00406439169553241
rbf 3 4 0.5 : -0.020245899303841595, 0.003916031843005929
rbf 3 4 0.7 : -0.0191956889640156, 0.0036647565853812347
rbf 3 4 0.9 : -0.01810955605717459, 0.003347010006882089 6
rbf 3 5 0.1 : -0.02190010197125445, 0.004171454224290216
rbf 3 5 0.3 : -0.02114436973354694, 0.004064372596890298
rbf 3 5 0.5 : -0.02024590940455382, 0.003916012716303041
rbf 3 5 0.7 : -0.01919569905159806, 0.003664737414943331
rbf 3 5 0.9 : -0.01810956613153909, 0.0033469907863600268
rbf 3 6 0.1 : -0.021900102475223802, 0.004171453274525394
rbf 3 6 0.3 : -0.021144370236991162, 0.004064371646053333
rbf 3 6 0.5 : -0.020245909907366234, 0.003916011764069416
rbf 3 6 0.7 : -0.019195699553756596, 0.0036647364605330024
rbf 3 6 0.9 : -0.018109566633038997, 0.0033469898294566924
rbf 4 1 0.1 : -0.01788233062048792, 0.004815781407965569
rbf 4 1 0.3 : -0.01683635529967149, 0.004384920217560428 5
rbf 4 1 0.5 : -0.015803581472293528, 0.003927056164325982
rbf 4 1 0.7 : -0.01485362341218004, 0.003447381450586206 6
rbf 4 1 0.9 : -0.013919698763231115, 0.0028433866173177558
rbf 4 2 0.1 : -0.01800907724107965, 0.004600017893109154
rbf 4 2 0.3 : -0.016963012072308593, 0.004168481921317135
rbf 4 2 0.5 : -0.015930138453113595, 0.003709986217609429
rbf 4 2 0.7 : -0.014981499588414859, 0.0032296759869370195
rbf 4 2 0.9 : -0.014048240410161238, 0.002625006840530575
rbf 4 3 0.1 : -0.018014568308683376, 0.0045896428445703075
rbf 4 3 0.3 : -0.016968495252184114, 0.004158075495992897
rbf 4 3 0.5 : -0.015935632546558366, 0.003699549364227561
rbf 4 3 0.7 : -0.014986976171372035, 0.003219209738112072
rbf 4 3 0.9 : -0.014053754381146578, 0.0026145067203419137
rbf 4 4 0.1 : -0.01801483869873275, 0.004589127170768759
rbf 4 4 0.3 : -0.01696876522988222, 0.00415755828448372
rbf 4 4 0.5 : -0.015935912798805064, 0.003699030661541647
rbf 4 4 0.7 : -0.014987209766038267, 0.0032186895949884997
rbf 4 4 0.9 : -0.014054023275422956, 0.0026139849044519714
rbf 4 5 0.1 : -0.018014852144656635, 0.004589101502762594
rbf 4 5 0.3 : -0.016968778655155912, 0.004157532540089992
rbf 4 5 0.5 : -0.015935926203082394, 0.0036990048430722844
rbf 4 5 0.7 : -0.014987202040169479, 0.003218663704962932
rbf 4 5 0.9 : -0.014054036642195645, 0.0026139589312444977
rbf 4 6 0.1 : -0.018014852813991577, 0.004589100224868248
rbf 4 6 0.3 : -0.01696877932346208, 0.0041575312583939095
rbf 4 6 0.5 : -0.015935926870342287, 0.003699003557689484
rbf 4 6 0.7 : -0.014987202706447178, 0.003218662416018536
rbf 4 6 0.9 : -0.014054037307558498, 0.0026139576381594143
sigmoid 1 1 0.1 : -0.02785157494475108, -0.0004817589182650739
sigmoid 1 1 0.3 : -0.028038637425086766, -0.00015273486527189 74
sigmoid 1 1 0.5 : -0.029327682929847977, -8.39384078961114e-05
sigmoid 1 1 0.7 : -0.027370781055785543, -0.00015273486527189 74
sigmoid 1 1 0.9 : -0.02742593928234638, -0.0003516451204561566
sigmoid 1 2 0.1 : -0.02785157494475108, -0.0004817589182650739
sigmoid 1 2 0.3 : -0.028038637425086766, -0.0001527348652718974
sigmoid 1 2 0.5 : -0.029327682929847977, -8.39384078961114e-05
sigmoid 1 2 0.7 : -0.027370781055785543, -0.0001527348652718974
sigmoid 1 2 0.9 : -0.02742593928234638, -0.0003516451204561566
sigmoid 1 3 0.1 : -0.02785157494475108, -0.0004817589182650739
sigmoid 1 3 0.3 : -0.028038637425086766, -0.0001527348652718974
sigmoid 1 3 0.5 : -0.029327682929847977, -8.39384078961114e-05
sigmoid 1 3 0.7 : -0.027370781055785543, -0.0001527348652718974
sigmoid 1 3 0.9 : -0.02742593928234638, -0.0003516451204561566
sigmoid 1 4 0.1 : -0.02785157494475108, -0.0004817589182650739
sigmoid 1 4 0.3 : -0.028038637425086766, -0.0001527348652718974
sigmoid 1 4 0.5 : -0.029327682929847977, -8.39384078961114e-05
sigmoid 1 4 0.7 : -0.027370781055785543, -0.0001527348652718974
sigmoid 1 4 0.9 : -0.02742593928234638, -0.0003516451204561566
sigmoid 1 5 0.1 : -0.02785157494475108, -0.0004817589182650739
sigmoid 1 5 0.3 : -0.028038637425086766, -0.0001527348652718974
sigmoid 1 5 0.5 : -0.029327682929847977, -8.39384078961114e-05
sigmoid 1 5 0.7 : -0.027370781055785543, -0.0001527348652718974
sigmoid 1 5 0.9 : -0.02742593928234638, -0.0003516451204561566
sigmoid 1 6 0.1 : -0.02785157494475108, -0.0004817589182650739
sigmoid 1 6 0.3 : -0.028038637425086766, -0.0001527348652718974
sigmoid 1 6 0.5 : -0.029327682929847977, -8.39384078961114e-05
sigmoid 1 6 0.7 : -0.027370781055785543, -0.0001527348652718974
sigmoid 1 6 0.9 : -0.02742593928234638, -0.0003516451204561566
sigmoid 2 1 0.1 : -0.02785157494475108, -0.0004817589182650739
sigmoid 2 1 0.3 : -0.028038637425086766, -0.0001527348652718974
sigmoid 2 1 0.5 : -0.029327682929847977, -8.39384078961114e-05
sigmoid 2 1 0.7 : -0.027370781055785543, -0.0001527348652718974
sigmoid 2 1 0.9 : -0.02742593928234638, -0.0003516451204561566
sigmoid 2 2 0.1 : -0.02785157494475108, -0.0004817589182650739
sigmoid 2 2 0.3 : -0.028038637425086766, -0.0001527348652718974
sigmoid 2 2 0.5 : -0.029327682929847977, -8.39384078961114e-05
sigmoid 2 2 0.7 : -0.027370781055785543, -0.0001527348652718974
sigmoid 2 2 0.9 : -0.02742593928234638, -0.0003516451204561566
sigmoid 2 3 0.1 : -0.02785157494475108, -0.0004817589182650739
sigmoid 2 3 0.3 : -0.028038637425086766, -0.0001527348652718974
sigmoid 2 3 0.5 : -0.029327682929847977, -8.39384078961114e-05
sigmoid 2 3 0.7 : -0.027370781055785543, -0.0001527348652718974
sigmoid 2 3 0.9 : -0.02742593928234638, -0.0003516451204561566
sigmoid 2 4 0.1 : -0.02785157494475108, -0.0004817589182650739
sigmoid 2 4 0.3 : -0.028038637425086766, -0.0001527348652718974
sigmoid 2 4 0.5 : -0.029327682929847977, -8.39384078961114e-05
sigmoid 2 4 0.7 : -0.027370781055785543, -0.0001527348652718974
sigmoid 2 4 0.9 : -0.02742593928234638, -0.0003516451204561566
sigmoid 2 5 0.1 : -0.02785157494475108, -0.0004817589182650739
sigmoid 2 5 0.3 : -0.028038637425086766, -0.0001527348652718974
sigmoid 2 5 0.5 : -0.029327682929847977, -8.39384078961114e-05
sigmoid 2 5 0.7 : -0.027370781055785543, -0.0001527348652718974
sigmoid 2 5 0.9 : -0.02742593928234638, -0.0003516451204561566
sigmoid 2 6 0.1 : -0.02785157494475108, -0.0004817589182650739
sigmoid 2 6 0.3 : -0.028038637425086766, -0.0001527348652718974
sigmoid 2 6 0.5 : -0.029327682929847977, -8.39384078961114e-05
sigmoid 2 6 0.7 : -0.027370781055785543, -0.0001527348652718974
sigmoid 2 6 0.9 : -0.02742593928234638, -0.0003516451204561566
sigmoid 3 1 0.1 : -0.02785157494475108, -0.0004817589182650739
sigmoid 3 1 0.3 : -0.028038637425086766, -0.0001527348652718974
sigmoid 3 1 0.5 : -0.029327682929847977, -8.39384078961114e-05
sigmoid 3 1 0.7 : -0.027370781055785543, -0.0001527348652718974
sigmoid 3 1 0.9 : -0.02742593928234638, -0.0003516451204561566
sigmoid 3 2 0.1 : -0.02785157494475108, -0.0004817589182650739
sigmoid 3 2 0.3 : -0.028038637425086766, -0.0001527348652718974
sigmoid 3 2 0.5 : -0.029327682929847977, -8.39384078961114e-05
sigmoid 3 2 0.7 : -0.027370781055785543, -0.0001527348652718974
sigmoid 3 2 0.9 : -0.02742593928234638, -0.0003516451204561566
sigmoid 3 3 0.1 : -0.02785157494475108, -0.0004817589182650739
sigmoid 3 3 0.3 : -0.028038637425086766, -0.0001527348652718974
sigmoid 3 3 0.5 : -0.029327682929847977, -8.39384078961114e-05

sigmoid 3 3 0.7 : -0.027370781055785543, -0.0001527348652718974
sigmoid 3 3 0.9 : -0.02742593928234638, -0.0003516451204561566
sigmoid 3 4 0.1 : -0.02785157494475108, -0.0004817589182650739
sigmoid 3 4 0.3 : -0.028038637425086766, -0.0001527348652718974
sigmoid 3 4 0.5 : -0.029327682929847977, -8.39384078961114e-05
sigmoid 3 4 0.7 : -0.027370781055785543, -0.0001527348652718974
sigmoid 3 4 0.9 : -0.02742593928234638, -0.0003516451204561566
sigmoid 3 5 0.1 : -0.02785157494475108, -0.0004817589182650739
sigmoid 3 5 0.3 : -0.028038637425086766, -0.0001527348652718974
sigmoid 3 5 0.5 : -0.029327682929847977, -8.39384078961114e-05
sigmoid 3 5 0.7 : -0.027370781055785543, -0.0001527348652718974
sigmoid 3 5 0.9 : -0.02742593928234638, -0.0003516451204561566
sigmoid 3 6 0.1 : -0.02785157494475108, -0.0004817589182650739
sigmoid 3 6 0.3 : -0.028038637425086766, -0.0001527348652718974
sigmoid 3 6 0.5 : -0.029327682929847977, -8.39384078961114e-05
sigmoid 3 6 0.7 : -0.027370781055785543, -0.0001527348652718974
sigmoid 3 6 0.9 : -0.02742593928234638, -0.0003516451204561566
sigmoid 4 1 0.1 : -0.02785157494475108, -0.0004817589182650739
sigmoid 4 1 0.3 : -0.028038637425086766, -0.0001527348652718974
sigmoid 4 1 0.5 : -0.029327682929847977, -8.39384078961114e-05
sigmoid 4 1 0.7 : -0.027370781055785543, -0.0001527348652718974
sigmoid 4 1 0.9 : -0.02742593928234638, -0.0003516451204561566
sigmoid 4 2 0.1 : -0.02785157494475108, -0.0004817589182650739
sigmoid 4 2 0.3 : -0.028038637425086766, -0.0001527348652718974
sigmoid 4 2 0.5 : -0.029327682929847977, -8.39384078961114e-05
sigmoid 4 2 0.7 : -0.027370781055785543, -0.0001527348652718974
sigmoid 4 2 0.9 : -0.02742593928234638, -0.0003516451204561566
sigmoid 4 3 0.1 : -0.02785157494475108, -0.0004817589182650739
sigmoid 4 3 0.3 : -0.028038637425086766, -0.0001527348652718974
sigmoid 4 3 0.5 : -0.029327682929847977, -8.39384078961114e-05
sigmoid 4 3 0.7 : -0.027370781055785543, -0.0001527348652718974
sigmoid 4 3 0.9 : -0.02742593928234638, -0.0003516451204561566
sigmoid 4 4 0.1 : -0.02785157494475108, -0.0004817589182650739
sigmoid 4 4 0.3 : -0.028038637425086766, -0.0001527348652718974
sigmoid 4 4 0.5 : -0.029327682929847977, -8.39384078961114e-05
sigmoid 4 4 0.7 : -0.027370781055785543, -0.0001527348652718974
sigmoid 4 4 0.9 : -0.02742593928234638, -0.0003516451204561566
sigmoid 4 5 0.1 : -0.02785157494475108, -0.0004817589182650739
sigmoid 4 5 0.3 : -0.028038637425086766, -0.0001527348652718974
sigmoid 4 5 0.5 : -0.029327682929847977, -8.39384078961114e-05
sigmoid 4 5 0.7 : -0.027370781055785543, -0.0001527348652718974
sigmoid 4 5 0.9 : -0.02742593928234638, -0.0003516451204561566
sigmoid 4 6 0.1 : -0.02785157494475108, -0.0004817589182650739
sigmoid 4 6 0.3 : -0.028038637425086766, -0.0001527348652718974
sigmoid 4 6 0.5 : -0.029327682929847977, -8.39384078961114e-05
sigmoid 4 6 0.7 : -0.027370781055785543, -0.0001527348652718974
sigmoid 4 6 0.9 : -0.02742593928234638, -0.0003516451204561566

Grid search of ElasticNet, Morgan(r=2)
ElasticNet(alpha= i, l1_ratio= j, max_iter=100000)
i j : accuracy of prediction using cross validation, accuracy of prediction using test data
0.001 0.0 : 0.9160705933904765, 0.9700572881992293
0.001 0.2 : 0.9173587795161244, 0.971353262425651
0.001 0.4 : 0.9189330921949408, 0.9721624273807347
0.001 0.6 : 0.9192190662390572, 0.9719770212017161
0.001 0.8 : 0.917377994898884, 0.9702322699020097
0.001 1.0 : 0.8984123864831115, 0.9590281829850684
0.01 0.0 : 0.8974168684548012, 0.9458014091780501
0.01 0.2 : 0.8989427047763267, 0.9472313459218923
0.01 0.4 : 0.8992380660399112, 0.9485092214883422
0.01 0.6 : 0.8997872339416624, 0.9499568410172539
0.01 0.8 : 0.9033151989377176, 0.9509134049861393
0.01 1.0 : 0.8939616561721688, 0.9459196972613684
0.1 0.0 : 0.7899599209271045, 0.8223441109416121
0.1 0.2 : 0.7803097318024743, 0.8082758617144656
0.1 0.4 : 0.7741092750701325, 0.7999083763561817
0.1 0.6 : 0.7735094310735464, 0.7977650407622581
0.1 0.8 : 0.778592794362416, 0.8051856670759326
0.1 1.0 : 0.7934563544935482, 0.8255593170597142

Grid search of RandomForest, Morgan(r=2)
RandomForestRegressor(n_estimators= i)
i: accuracy of prediction using cross validation, accuracy of prediction using test data
100 0.0 : 0.7908643522397354, 0.8646789658500024
500 0.0 : 0.7922670318169652, 0.8668155844610588
1000 0.0 : 0.7926574040198422, 0.8693943801654519
2000 0.0 : 0.7929485217585815, 0.8653415963878927

Grid search of NeuralNetwork, Morgan(r=2)
MLPRegressor(activation=act, alpha=a, batch_size=batch, beta_1=0.9,
beta_2=0.999, early_stopping=False, epsilon=1e-08,
hidden_layer_sizes=hid, learning_rate='constant',
learning_rate_init=0.001, max_iter=100000, momentum=0.9,
n_iter_no_change=10, nesterovs_momentum=True,
power_t=0.5,
random_state=1, shuffle=True, solver='adam', tol=0.0001,
validation_fraction=0.1, verbose=False, warm_start=False)
act (hid) a batch: accuracy of prediction using cross validation, accuracy of prediction using test data
relu (200,) 0.0001 500 : 0.8459872988348239, 0.9545066202629713
relu (200,) 0.0001 200 : 0.8709890423081831, 0.9513321388198434
relu (200,) 0.0001 100 : 0.8721958148985465, 0.9554836832558058
relu (200,) 0.0001 50 : 0.878297069037351, 0.9546116938096965
relu (200,) 0.001 500 : 0.8459987951445094, 0.9545388853575868
relu (200,) 0.001 200 : 0.8709833884016357, 0.9513899229080606
relu (200,) 0.001 100 : 0.8722634425467453, 0.9555913791926168
relu (200,) 0.001 50 : 0.8782857166634696, 0.9578290070088513
relu (200,) 0.01 500 : 0.8463553425981738, 0.9549171783119933
relu (200,) 0.01 200 : 0.8719225913130796, 0.9519927130562814
relu (200,) 0.01 100 : 0.8739601082269864, 0.9563638946154137
relu (200,) 0.01 50 : 0.8806878101649656, 0.9588368508455797
relu (200, 200) 0.0001 500 : 0.8394267834510346, 0.9407684159629601
relu (200, 200) 0.0001 200 : 0.8665671931811767, 0.9409242714465922
relu (200, 200) 0.0001 100 : 0.8714912227951818, 0.9471034096714703
relu (200, 200) 0.0001 50 : 0.8762875177292957, 0.9482090253468884
relu (200, 200) 0.001 500 : 0.8396439947186953, 0.9421314044059166
relu (200, 200) 0.001 200 : 0.8652979052364017, 0.9417776656035576
relu (200, 200) 0.001 100 : 0.8708371268227761, 0.9458897942950167
relu (200, 200) 0.001 50 : 0.8756506427049778, 0.948696102330001
relu (200, 200) 0.01 500 : 0.8399872653546396, 0.9406536698301341
relu (200, 200) 0.01 200 : 0.866248263508939, 0.9402360010743342
relu (200, 200) 0.01 100 : 0.8719815293027932, 0.9462277930499021
relu (200, 200) 0.01 50 : 0.8769659224009084, 0.9493889526179355
relu (100,) 0.0001 500 : 0.845206333937792, 0.9532243792242922
relu (100,) 0.0001 200 : 0.8739994233052876, 0.9506059579526001
relu (100,) 0.0001 100 : 0.8719498596534644, 0.9559075388577549
relu (100,) 0.0001 50 : 0.876025341624754, 0.9572101530426559
relu (100,) 0.001 500 : 0.8452188057793834, 0.9533523267355063
relu (100,) 0.001 200 : 0.8739654690262094, 0.950588769129914
relu (100,) 0.001 100 : 0.8721172376927877, 0.9549101661442836
relu (100,) 0.001 50 : 0.8760265092245708, 0.9577788592899091
relu (100,) 0.01 500 : 0.8453826636133396, 0.9527089124857115
relu (100,) 0.01 200 : 0.8746454755865946, 0.9511070485212818
relu (100,) 0.01 100 : 0.8733273460443065, 0.9565930463795698
relu (100,) 0.01 50 : 0.8779116862394851, 0.9593958796117785
relu (100, 100) 0.0001 500 : 0.8422484470103095, 0.9499024972521921
relu (100, 100) 0.0001 200 : 0.8700195635496331, 0.9439043233313178
relu (100, 100) 0.0001 100 : 0.8745384898815033, 0.9518059809370163
relu (100, 100) 0.0001 50 : 0.8762970606634634, 0.9525177975115852
relu (100, 100) 0.001 500 : 0.8412209066444557, 0.9505026251048196

relu (100, 100) 0.001 200 : 0.8693867258837482, 0.9426391941665264
relu (100, 100) 0.001 100 : 0.8756202995537763, 0.94892999636464
relu (100, 100) 0.001 50 : 0.8771539546172514, 0.9503738697943084
relu (100, 100) 0.01 500 : 0.8406323213090594, 0.9481064100016767
relu (100, 100) 0.01 200 : 0.8680311846179876, 0.9431650266255575
relu (100, 100) 0.01 100 : 0.8742068046849389, 0.9503044663589539
relu (100, 100) 0.01 50 : 0.8788537156754481, 0.9532643532661036
relu (100, 200) 0.0001 500 : 0.8416309082456863, 0.9422691661060993
relu (100, 200) 0.0001 200 : 0.8698272926674105, 0.9383716184274721
relu (100, 200) 0.0001 100 : 0.8722255925020514, 0.940112137886183
relu (100, 200) 0.0001 50 : 0.8800458004553379, 0.944075667789115
relu (100, 200) 0.001 500 : 0.8434492797809761, 0.947670636165142
relu (100, 200) 0.001 200 : 0.8686803293416098, 0.9392915937323185
relu (100, 200) 0.001 100 : 0.8720494670268837, 0.9440381644666864
relu (100, 200) 0.001 50 : 0.8784495980147135, 0.9452310880353809
relu (100, 200) 0.01 500 : 0.8431359224750861, 0.9446691296261904
relu (100, 200) 0.01 200 : 0.867102864756718, 0.9386135648680162
relu (100, 200) 0.01 100 : 0.8727420465770426, 0.9457510833900888
relu (100, 200) 0.01 50 : 0.8794097992550635, 0.9458912316134579
relu (50,) 0.0001 500 : 0.8443900935413657, 0.9548231459589047
relu (50,) 0.0001 200 : 0.8710393594858914, 0.9529295805123673
relu (50,) 0.0001 100 : 0.8711143245603775, 0.9556584580164887
relu (50,) 0.0001 50 : 0.8751626979669342, 0.9579997139917216
relu (50,) 0.001 500 : 0.8443993364597387, 0.9548315018501128
relu (50,) 0.001 200 : 0.8712103968275585, 0.9529800815960343
relu (50,) 0.001 100 : 0.8711804832111156, 0.9557349054580481
relu (50,) 0.001 50 : 0.8752478188318783, 0.9577537900727014
relu (50,) 0.01 500 : 0.8445477981390697, 0.9549952152245921
relu (50,) 0.01 200 : 0.8719455844753409, 0.9535504116935958
relu (50,) 0.01 100 : 0.8720589641380384, 0.9565523935923622
relu (50,) 0.01 50 : 0.8767101542241968, 0.9588261030586276
relu (50, 50) 0.0001 500 : 0.8418601723823184, 0.942736911586521
relu (50, 50) 0.0001 200 : 0.8734620950293983, 0.9408805402226513
relu (50, 50) 0.0001 100 : 0.8748836124127717, 0.9494260004521535
relu (50, 50) 0.0001 50 : 0.881119900377762, 0.9527567096111073
relu (50, 50) 0.001 500 : 0.8437842603362598, 0.9476132845641119
relu (50, 50) 0.001 200 : 0.8733162024105472, 0.9410929525189644
relu (50, 50) 0.001 100 : 0.8758143435014579, 0.949665869337311
relu (50, 50) 0.001 50 : 0.879263181143336, 0.9523506995754477
relu (50, 50) 0.01 500 : 0.8429238678219878, 0.9480372014281622
relu (50, 50) 0.01 200 : 0.8730244828230669, 0.9418327373837514
relu (50, 50) 0.01 100 : 0.8765402728139884, 0.949261990325986
relu (50, 50) 0.01 50 : 0.878368028422214, 0.9525726168434159
relu (50, 100) 0.0001 500 : 0.8427042953955141, 0.9439018584783191
relu (50, 100) 0.0001 200 : 0.8702115832705173, 0.9431814221932389
relu (50, 100) 0.0001 100 : 0.8707901648090111, 0.9467767140615635
relu (50, 100) 0.0001 50 : 0.8758916859244865, 0.948977278990874
relu (50, 100) 0.001 500 : 0.8423351460672854, 0.9446861821636141
relu (50, 100) 0.001 200 : 0.8714444945634817, 0.9465174624846919
relu (50, 100) 0.001 100 : 0.8707283946281604, 0.9463526024460602
relu (50, 100) 0.001 50 : 0.8748871766400932, 0.9510400836281269
relu (50, 100) 0.01 500 : 0.8391979470507985, 0.9456105222203766
relu (50, 100) 0.01 200 : 0.8716131086470431, 0.9451673400890789
relu (50, 100) 0.01 100 : 0.8713463249189101, 0.9466049103422987
relu (50, 100) 0.01 50 : 0.8734079937044633, 0.951115933152515
relu (50, 200) 0.0001 500 : 0.8428784495780371, 0.9364621830621607
relu (50, 200) 0.0001 200 : 0.8685501709118345, 0.9353972118429715
relu (50, 200) 0.0001 100 : 0.8710580048398077, 0.9392670456873283
relu (50, 200) 0.0001 50 : 0.8783219938700162, 0.9463403806331485
relu (50, 200) 0.001 500 : 0.8443331072521942, 0.9368225153228797
relu (50, 200) 0.001 200 : 0.8707134842258174, 0.9356196438584193
relu (50, 200) 0.001 100 : 0.8722471022282562, 0.9397028044042107
relu (50, 200) 0.001 50 : 0.8796851644778483, 0.9433778656939709
relu (50, 200) 0.01 500 : 0.8438719130793167, 0.9374136498424803
relu (50, 200) 0.01 200 : 0.8718479222018933, 0.9368430437288547
relu (50, 200) 0.01 100 : 0.8720241907761009, 0.9406886944446404
relu (50, 200) 0.01 50 : 0.8792075960561828, 0.949364594291547
tanh (200,) 0.0001 500 : 0.8470436999290627, 0.9237899240068588
tanh (200,) 0.0001 200 : 0.874197505876882, 0.9209092991210702
tanh (200,) 0.0001 100 : 0.8741859198592479, 0.9278203020347873
tanh (200,) 0.0001 50 : 0.8797378477452495, 0.931093671020693
tanh (200,) 0.001 500 : 0.8475031386240687, 0.9252339834182529
tanh (200,) 0.001 200 : 0.8754329321145473, 0.9217949952286109
tanh (200,) 0.001 100 : 0.8755520456919189, 0.9288817106470308
tanh (200,) 0.001 50 : 0.8807148732631574, 0.9315327062676747
tanh (200,) 0.01 500 : 0.8517537584010707, 0.9340911888300627
tanh (200,) 0.01 200 : 0.8825220570775686, 0.9365364582946643
tanh (200,) 0.01 100 : 0.8821453821589254, 0.9362514623625753
tanh (200,) 0.01 50 : 0.8859737001046899, 0.9377892039223666
tanh (200, 200) 0.0001 500 : 0.8453198499283087, 0.9286770863663214
tanh (200, 200) 0.0001 200 : 0.8779839991450927, 0.9234315026010826
tanh (200, 200) 0.0001 100 : 0.8799578166628688, 0.9286201681214867
tanh (200, 200) 0.0001 50 : 0.8897688772693579, 0.932205087406055
tanh (200, 200) 0.001 500 : 0.8461638004049062, 0.9291438602290091
tanh (200, 200) 0.001 200 : 0.8775550392144877, 0.9238328186804918
tanh (200, 200) 0.001 100 : 0.88049861520144, 0.9289490492379022
tanh (200, 200) 0.001 50 : 0.8914007882163739, 0.9329054436733561
tanh (200, 200) 0.01 500 : 0.8535102455637054, 0.9322620523192812
tanh (200, 200) 0.01 200 : 0.8838017724918228, 0.9276004528485751
tanh (200, 200) 0.01 100 : 0.8855845072483767, 0.9317510208213735
tanh (200, 200) 0.01 50 : 0.892540594188058, 0.9381655116668122
tanh (100,) 0.0001 500 : 0.8348785841392669, 0.9181724289124819
tanh (100,) 0.0001 200 : 0.864901797466073, 0.9200251485540686
tanh (100,) 0.0001 100 : 0.8686057345789436, 0.9235181798368185
tanh (100,) 0.0001 50 : 0.8757259089001144, 0.9254959507309847
tanh (100,) 0.001 500 : 0.8358613108141718, 0.9195846559232508
tanh (100,) 0.001 200 : 0.8667844263678868, 0.9215509450866419
tanh (100,) 0.001 100 : 0.8704723148877391, 0.9247473127180412
tanh (100,) 0.001 50 : 0.8771209166285103, 0.9273733799482551
tanh (100,) 0.01 500 : 0.8442075376246546, 0.9273965894753429
tanh (100,) 0.01 200 : 0.8761315998074274, 0.9326505851183701
tanh (100,) 0.01 100 : 0.8775556180774968, 0.9316585851185256
tanh (100,) 0.01 50 : 0.8836099085987261, 0.933182171714448
tanh (100, 100) 0.0001 500 : 0.8396501779490839, 0.9298796946352456
tanh (100, 100) 0.0001 200 : 0.8666278748386599, 0.927963701561504

tanh (100, 100) 0.0001 100 : 0.875261885177309, 0.9346276601177594
tanh (100, 100) 0.0001 50 : 0.883868149236952, 0.9403095294405449
tanh (100, 100) 0.001 500 : 0.840681915284026, 0.9304318623257196
tanh (100, 100) 0.001 200 : 0.8675950897284956, 0.9284556760588485
tanh (100, 100) 0.001 100 : 0.8755646293106718, 0.9397665819471156
tanh (100, 100) 0.001 50 : 0.88650660758685, 0.9396021715137759
tanh (100, 100) 0.01 500 : 0.8515208612814813, 0.9349387201900954
tanh (100, 100) 0.01 200 : 0.8729516385903686, 0.9325899459005839
tanh (100, 100) 0.01 100 : 0.8811478205433236, 0.9415093536199446
tanh (100, 100) 0.01 50 : 0.8879744196048478, 0.9438075990402814
tanh (100, 200) 0.0001 500 : 0.8397637102992735, 0.9249676055893522
tanh (100, 200) 0.0001 200 : 0.879350131580358, 0.9272368998085806
tanh (100, 200) 0.0001 100 : 0.8796137466204799, 0.9303677181329016
tanh (100, 200) 0.0001 50 : 0.8850355419967763, 0.9344852066782986
tanh (100, 200) 0.001 500 : 0.8402031001747211, 0.9252051426782051
tanh (100, 200) 0.001 200 : 0.8798441793050114, 0.9276091263652538
tanh (100, 200) 0.001 100 : 0.8788677507525625, 0.9309369351265676
tanh (100, 200) 0.001 50 : 0.8860894083991031, 0.9346166848038848
tanh (100, 200) 0.01 500 : 0.8442937408729334, 0.92714937919504
tanh (100, 200) 0.01 200 : 0.8836027141243299, 0.9306392184208254
tanh (100, 200) 0.01 100 : 0.8828823519230898, 0.9329236923354851
tanh (100, 200) 0.01 50 : 0.8877781893537519, 0.9363423301566343
tanh (50,) 0.0001 500 : 0.8236365290506082, 0.9154934425259403
tanh (50,) 0.0001 200 : 0.8531617158230475, 0.9158104685947348
tanh (50,) 0.0001 100 : 0.8615940421030249, 0.9168021960150337
tanh (50,) 0.0001 50 : 0.8704171384053769, 0.9193729739752443
tanh (50,) 0.001 500 : 0.8257633449010001, 0.9174841114412889
tanh (50,) 0.001 200 : 0.8563496315721515, 0.9209171562226801
tanh (50,) 0.001 100 : 0.8604252461191028, 0.9194254276198326
tanh (50,) 0.001 50 : 0.8734119128811756, 0.9213236692996762
tanh (50,) 0.01 500 : 0.840050079608577, 0.9248389186623952
tanh (50,) 0.01 200 : 0.8718463099859772, 0.9230411378786912
tanh (50,) 0.01 100 : 0.8737458733951019, 0.9275389960963029
tanh (50,) 0.01 50 : 0.8829707532483948, 0.9269647060678232
tanh (50, 50) 0.0001 500 : 0.8353119636175121, 0.9261275454747895
tanh (50, 50) 0.0001 200 : 0.8721879162166282, 0.9238513115003789
tanh (50, 50) 0.0001 100 : 0.87695079790966, 0.9250918369671027
tanh (50, 50) 0.0001 50 : 0.8826934754401984, 0.9315005617319871
tanh (50, 50) 0.001 500 : 0.8376936849312528, 0.9247243505060251
tanh (50, 50) 0.001 200 : 0.8720583416089862, 0.9258138800571039
tanh (50, 50) 0.001 100 : 0.8774608771288914, 0.9311295368076195
tanh (50, 50) 0.001 50 : 0.8828268593404234, 0.936663449907424
tanh (50, 50) 0.01 500 : 0.8531329454901542, 0.9341819036522377
tanh (50, 50) 0.01 200 : 0.8778461265570909, 0.9383749320337976
tanh (50, 50) 0.01 100 : 0.8857681950288876, 0.9372929105941457
tanh (50, 50) 0.01 50 : 0.8907541580995945, 0.9437056644394173
tanh (50, 100) 0.0001 500 : 0.8402497550642334, 0.9303064557559383
tanh (50, 100) 0.0001 200 : 0.8749822901944209, 0.9256342167821968
tanh (50, 100) 0.0001 100 : 0.8798089099725607, 0.9279681156851824
tanh (50, 100) 0.0001 50 : 0.8824578710009565, 0.9370843651287987
tanh (50, 100) 0.001 500 : 0.8410131319687235, 0.9313378775094158
tanh (50, 100) 0.001 200 : 0.8759532448933438, 0.9274185358571989
tanh (50, 100) 0.001 100 : 0.8800370777124857, 0.9306668183321828
tanh (50, 100) 0.001 50 : 0.8833516140994144, 0.9371781930897619
tanh (50, 100) 0.01 500 : 0.8475061743138319, 0.9338045189796836
tanh (50, 100) 0.01 200 : 0.880729525283852, 0.9272014898189749
tanh (50, 100) 0.01 100 : 0.883224836577152, 0.9343226029983506
tanh (50, 100) 0.01 50 : 0.886833144645139, 0.9411696903946951
tanh (50, 200) 0.0001 500 : 0.8491184520770805, 0.9255273472337553
tanh (50, 200) 0.0001 200 : 0.8836943896734784, 0.9247841756681868
tanh (50, 200) 0.0001 100 : 0.8824507867619522, 0.930878591462104
tanh (50, 200) 0.0001 50 : 0.8883161964053643, 0.9306196756178773
tanh (50, 200) 0.001 500 : 0.8492886797803679, 0.9257534193260533
tanh (50, 200) 0.001 200 : 0.8839631456938237, 0.9250092502908625
tanh (50, 200) 0.001 100 : 0.8827432837499192, 0.930936822080692
tanh (50, 200) 0.001 50 : 0.8884746828702934, 0.9296671778217696
tanh (50, 200) 0.01 500 : 0.8509463520081741, 0.9278038677129324
tanh (50, 200) 0.01 200 : 0.885775740621239, 0.9270929525185065
tanh (50, 200) 0.01 100 : 0.8836820705739287, 0.9302146417851668
tanh (50, 200) 0.01 50 : 0.8891728220896425, 0.930394729285828
logistic (200,) 0.0001 500 : 0.8499667189715987, 0.9313135343368196
logistic (200,) 0.0001 200 : 0.8820094575296569, 0.9251061152291273
logistic (200,) 0.0001 100 : 0.8824557462372763, 0.9324120825050273
logistic (200,) 0.0001 50 : 0.8876105376196037, 0.9375280933066374
logistic (200,) 0.001 500 : 0.851565382344582, 0.9337889171645681
logistic (200,) 0.001 200 : 0.8846943233190568, 0.9273881376754326
logistic (200,) 0.001 100 : 0.8861007184270215, 0.9408244240620651
logistic (200,) 0.001 50 : 0.8914031607620425, 0.9443760629569146
logistic (200,) 0.01 500 : 0.86512467928268, 0.9488897241983909
logistic (200,) 0.01 200 : 0.9001664487649647, 0.9529844894702477
logistic (200,) 0.01 100 : 0.9014561973020069, 0.9548305408372171
logistic (200,) 0.01 50 : 0.9033621922065619, 0.958137949760708
logistic (200, 200) 0.0001 500 : 0.8434407233436836, 0.9222747477780278
logistic (200, 200) 0.0001 200 : 0.8749108920788558, 0.9102746531894557
logistic (200, 200) 0.0001 100 : 0.8784603720966444, 0.9235091556793769
logistic (200, 200) 0.0001 50 : 0.8870405873720605, 0.925090107024462
logistic (200, 200) 0.001 500 : 0.8519596664773325, 0.9233533231383549
logistic (200, 200) 0.001 200 : 0.8813883292845498, 0.9169050894590137
logistic (200, 200) 0.001 100 : 0.8851736296621612, 0.9286490342639276
logistic (200, 200) 0.001 50 : 0.8924680987440103, 0.9310346017248018
logistic (200, 200) 0.01 500 : 0.8737379835540654, 0.9302741643338014
logistic (200, 200) 0.01 200 : 0.8842910464581036, 0.9284335920864241
logistic (200, 200) 0.01 100 : 0.8894332441068794, 0.9332467817811598
logistic (200, 200) 0.01 50 : 0.8942969393904516, 0.9401533553914809
logistic (100,) 0.0001 500 : 0.8425440673519196, 0.9236363398880812

logistic (100,) 0.0001 200 : 0.8757766791128077, 0.9200133766308123
logistic (100,) 0.0001 100 : 0.8758277295679967, 0.9268483356044972
logistic (100,) 0.0001 50 : 0.8818164590220903, 0.9289066195925101
logistic (100,) 0.001 500 : 0.8461630258391409, 0.9267560662704761
logistic (100,) 0.001 200 : 0.8804828909860112, 0.9244681371075366
logistic (100,) 0.001 100 : 0.8812338933113864, 0.9308227574522392
logistic (100,) 0.001 50 : 0.8860608598539826, 0.9335843649177183
logistic (100,) 0.01 500 : 0.8685365221863892, 0.9408676715060303
logistic (100,) 0.01 200 : 0.8951531164511956, 0.9422799319135999
logistic (100,) 0.01 100 : 0.8966880716459544, 0.9468541688633116
logistic (100,) 0.01 50 : 0.8998181476608984, 0.9519151223602798
logistic (100, 100) 0.0001 500 : 0.8422641988671474, 0.9159697820905592
logistic (100, 100) 0.0001 200 : 0.8648461359365622, 0.9128615829346067
logistic (100, 100) 0.0001 100 : 0.8740762122585315, 0.9204466634666156
logistic (100, 100) 0.0001 50 : 0.8816060565099517, 0.9253417057447378
logistic (100, 100) 0.001 500 : 0.8516736410160476, 0.9173048078693614
logistic (100, 100) 0.001 200 : 0.8738872228644684, 0.9176046909559313
logistic (100, 100) 0.001 100 : 0.8820099526012635, 0.9237131575633442
logistic (100, 100) 0.001 50 : 0.8876980906687647, 0.9287130213787244
logistic (100, 100) 0.01 500 : 0.8632013866585704, 0.9242720154781712
logistic (100, 100) 0.01 200 : 0.8700296532025431, 0.9222631632932116
logistic (100, 100) 0.01 100 : 0.8785895648981257, 0.9209216749916312
logistic (100, 100) 0.01 50 : 0.8847442280633462, 0.9392281701228639
logistic (100, 200) 0.0001 500 : 0.8415109864990177, 0.9196830110044871
logistic (100, 200) 0.0001 200 : 0.8670637456583237, 0.9112862368890258
logistic (100, 200) 0.0001 100 : 0.8741739205876522, 0.9224226565798997
logistic (100, 200) 0.0001 50 : 0.8777560731884236, 0.9262010749314321
logistic (100, 200) 0.001 500 : 0.8465496583761715, 0.9241581717753061
logistic (100, 200) 0.001 200 : 0.8718059860002653, 0.9157879611010437
logistic (100, 200) 0.001 100 : 0.8757654459970524, 0.925179382513235
logistic (100, 200) 0.001 50 : 0.8782561068371072, 0.93161317960257
logistic (100, 200) 0.01 500 : 0.8648530505092845, 0.9290835841227222
logistic (100, 200) 0.01 200 : 0.8731798322313097, 0.9282320083433869
logistic (100, 200) 0.01 100 : 0.8792486409441379, 0.9344130642540522
logistic (100, 200) 0.01 50 : 0.8804932997157252, 0.9350716824982792
logistic (50,) 0.0001 500 : 0.8426570616348179, 0.9195759725668582
logistic (50,) 0.0001 200 : 0.8749597472199614, 0.9186822779835897
logistic (50,) 0.0001 100 : 0.8742164378964702, 0.9228733236834675
logistic (50,) 0.0001 50 : 0.8819016482810766, 0.9235720367212791
logistic (50,) 0.001 500 : 0.8493205687919186, 0.9247256553019776
logistic (50,) 0.001 200 : 0.8816976147570126, 0.9256215911877883
logistic (50,) 0.001 100 : 0.8823873986560491, 0.9307007961017354
logistic (50,) 0.001 50 : 0.8872467425944531, 0.9308414867752651
logistic (50,) 0.01 500 : 0.8749582628778407, 0.9348379047716039
logistic (50,) 0.01 200 : 0.8923520676748368, 0.9437610109467426
logistic (50,) 0.01 100 : 0.8953012090111485, 0.9417823141430384
logistic (50,) 0.01 50 : 0.8977240758005888, 0.9489189236185854
logistic (50, 50) 0.0001 500 : 0.8378687958792929, 0.9151403735701581
logistic (50, 50) 0.0001 200 : 0.8664914978648282, 0.9127659527502364
logistic (50, 50) 0.0001 100 : 0.8748640932994481, 0.9307077279319267
logistic (50, 50) 0.0001 50 : 0.8828882079024343, 0.9387106902292771
logistic (50, 50) 0.001 500 : 0.8492519976546248, 0.9246106727764912
logistic (50, 50) 0.001 200 : 0.8724806605742998, 0.9246616705929455
logistic (50, 50) 0.001 100 : 0.8818442001665122, 0.9345596438898848
logistic (50, 50) 0.001 50 : 0.8866232340227151, 0.9439793607005845
logistic (50, 50) 0.01 500 : 0.8435684632484474, 0.9249102254610715
logistic (50, 50) 0.01 200 : 0.8706013158692226, 0.9211873899165393
logistic (50, 50) 0.01 100 : 0.8750491048795592, 0.9238638673987831
logistic (50, 50) 0.01 50 : 0.8771232946682271, 0.9413946033620411
logistic (50, 100) 0.0001 500 : 0.8430688040821657, 0.9204948213789697
logistic (50, 100) 0.0001 200 : 0.8681869880424251, 0.9077528144778604
logistic (50, 100) 0.0001 100 : 0.8717927346778813, 0.9242410170551826
logistic (50, 100) 0.0001 50 : 0.8813929703133075, 0.9323835951920615
logistic (50, 100) 0.001 500 : 0.8503888502923582, 0.9304781816435481
logistic (50, 100) 0.001 200 : 0.8726022821043152, 0.9150610221826168
logistic (50, 100) 0.001 100 : 0.8773063853163737, 0.9280364062054974
logistic (50, 100) 0.001 50 : 0.8829925404564347, 0.932828651350078
logistic (50, 100) 0.01 500 : 0.8694315577628059, 0.9217743686593067
logistic (50, 100) 0.01 200 : 0.876416819990245, 0.9281933086909475
logistic (50, 100) 0.01 100 : 0.8821377941236619, 0.9339268780407264
logistic (50, 100) 0.01 50 : 0.8871693651316649, 0.9342431613690673
logistic (50, 200) 0.0001 500 : 0.836115066085352, 0.9225032593686799
logistic (50, 200) 0.0001 200 : 0.8728285356602352, 0.9132905013691183
logistic (50, 200) 0.0001 100 : 0.8751954976928946, 0.9222909079415472
logistic (50, 200) 0.0001 50 : 0.8820909503298431, 0.9265351968257716
logistic (50, 200) 0.001 500 : 0.8401420110884329, 0.9248105341346475
logistic (50, 200) 0.001 200 : 0.8769431838437866, 0.9169435450023637
logistic (50, 200) 0.001 100 : 0.878054297617329, 0.925645994415905
logistic (50, 200) 0.001 50 : 0.8858390662374728, 0.9298413152766214
logistic (50, 200) 0.01 500 : 0.8612068065008582, 0.9355234797418257
logistic (50, 200) 0.01 200 : 0.8837382615657674, 0.92862965614949

logistic (50, 200) 0.01 100 : 0.8859474066177171, 0.9373247619888715
logistic (50, 200) 0.01 50 : 0.8897673672765002, 0.9407089832770841

Grid search of LightGBM, Morgan(r=2)
lgb.LGBMRegressor(boosting_type = "gbdt", num_leaves = j,max_depth = 0)
j: accuracy of prediction using cross validation, accuracy of prediction using test data
10 : 0.7720376159165153, 0.8469212634342757
50 : 0.7761068925899223, 0.8605984651488636
100 : 0.7761068925899223, 0.8605984651488636
150 : 0.7761068925899223, 0.8605984651488636

Grid search of SVR, Morgan(r=2, 1024)
SVR(C= c_num, kernel = ker, epsilon = e, gamma = r,degree = 3, coef0=1)
Ker c_num r e: accuracy of prediction using cross validation, accuracy of prediction using test data
kernel, C, gammma, epsilon
linear 1 1 0.1 3 : 0.8801000143858, 0.9633137591712483
linear 1 1 0.1 4 : 0.8801000143858, 0.9633137591712483
linear 1 1 0.1 5 : 0.8801000143858, 0.9633137591712483
linear 1 1 0.1 6 : 0.8801000143858, 0.9633137591712483
linear 1 1 0.1 7 : 0.8801000143858, 0.9633137591712483
linear 1 1 0.1 8 : 0.8801000143858, 0.9633137591712483
linear 1 1 0.1 9 : 0.8801000143858, 0.9633137591712483
linear 1 1 0.3 3 : 0.8803979122407402, 0.9633178301452152
linear 1 1 0.3 4 : 0.8803979122407402, 0.9633178301452152
linear 1 1 0.3 5 : 0.8803979122407402, 0.9633178301452152
linear 1 1 0.3 6 : 0.8803979122407402, 0.9633178301452152
linear 1 1 0.3 7 : 0.8803979122407402, 0.9633178301452152
linear 1 1 0.3 8 : 0.8803979122407402, 0.9633178301452152
linear 1 1 0.3 9 : 0.8803979122407402, 0.9633178301452152
linear 1 1 0.5 3 : 0.8797813751434311, 0.9631321912792431
linear 1 1 0.5 4 : 0.8797813751434311, 0.9631321912792431
linear 1 1 0.5 5 : 0.8797813751434311, 0.9631321912792431
linear 1 1 0.5 6 : 0.8797813751434311, 0.9631321912792431
linear 1 1 0.5 7 : 0.8797813751434311, 0.9631321912792431
linear 1 1 0.5 8 : 0.8797813751434311, 0.9631321912792431
linear 1 1 0.5 9 : 0.8797813751434311, 0.9631321912792431
linear 1 1 0.7 3 : 0.8784832714203713, 0.9623144443982917
linear 1 1 0.7 4 : 0.8784832714203713, 0.9623144443982917
linear 1 1 0.7 5 : 0.8784832714203713, 0.9623144443982917
linear 1 1 0.7 6 : 0.8784832714203713, 0.9623144443982917
linear 1 1 0.7 7 : 0.8784832714203713, 0.9623144443982917
linear 1 1 0.7 8 : 0.8784832714203713, 0.9623144443982917
linear 1 1 0.7 9 : 0.8784832714203713, 0.9623144443982917
linear 1 1 0.9 3 : 0.878211207610421, 0.9610778179781068
linear 1 1 0.9 4 : 0.878211207610421, 0.9610778179781068
linear 1 1 0.9 5 : 0.878211207610421, 0.9610778179781068
linear 1 1 0.9 6 : 0.878211207610421, 0.9610778179781068
linear 1 1 0.9 7 : 0.878211207610421, 0.9610778179781068
linear 1 1 0.9 8 : 0.878211207610421, 0.9610778179781068
linear 1 1 0.9 9 : 0.878211207610421, 0.9610778179781068
linear 1 2 0.1 3 : 0.8801000143858, 0.9633137591712483
linear 1 2 0.1 4 : 0.8801000143858, 0.9633137591712483
linear 1 2 0.1 5 : 0.8801000143858, 0.9633137591712483
linear 1 2 0.1 6 : 0.8801000143858, 0.9633137591712483
linear 1 2 0.1 7 : 0.8801000143858, 0.9633137591712483
linear 1 2 0.1 8 : 0.8801000143858, 0.9633137591712483
linear 1 2 0.1 9 : 0.8801000143858, 0.9633137591712483
linear 1 2 0.3 3 : 0.8803979122407402, 0.9633178301452152
linear 1 2 0.3 4 : 0.8803979122407402, 0.9633178301452152
linear 1 2 0.3 5 : 0.8803979122407402, 0.9633178301452152
linear 1 2 0.3 6 : 0.8803979122407402, 0.9633178301452152
linear 1 2 0.3 7 : 0.8803979122407402, 0.9633178301452152
linear 1 2 0.3 8 : 0.8803979122407402, 0.9633178301452152
linear 1 2 0.3 9 : 0.8803979122407402, 0.9633178301452152
linear 1 2 0.5 3 : 0.8797813751434311, 0.9631321912792431
linear 1 2 0.5 4 : 0.8797813751434311, 0.9631321912792431
linear 1 2 0.5 5 : 0.8797813751434311, 0.9631321912792431
linear 1 2 0.5 6 : 0.8797813751434311, 0.9631321912792431
linear 1 2 0.5 7 : 0.8797813751434311, 0.9631321912792431
linear 1 2 0.5 8 : 0.8797813751434311, 0.9631321912792431
linear 1 2 0.5 9 : 0.8797813751434311, 0.9631321912792431
linear 1 2 0.7 3 : 0.8784832714203713, 0.9623144443982917
linear 1 2 0.7 4 : 0.8784832714203713, 0.9623144443982917
linear 1 2 0.7 5 : 0.8784832714203713, 0.9623144443982917
linear 1 2 0.7 6 : 0.8784832714203713, 0.9623144443982917
linear 1 2 0.7 7 : 0.8784832714203713, 0.9623144443982917
linear 1 2 0.7 8 : 0.8784832714203713, 0.9623144443982917
linear 1 2 0.7 9 : 0.8784832714203713, 0.9623144443982917
linear 1 2 0.9 3 : 0.878211207610421, 0.9610778179781068
linear 1 2 0.9 4 : 0.878211207610421, 0.9610778179781068
linear 1 2 0.9 5 : 0.878211207610421, 0.9610778179781068
linear 1 2 0.9 6 : 0.878211207610421, 0.9610778179781068
linear 1 2 0.9 7 : 0.878211207610421, 0.9610778179781068
linear 1 2 0.9 8 : 0.878211207610421, 0.9610778179781068
linear 1 2 0.9 9 : 0.878211207610421, 0.9610778179781068
linear 1 3 0.1 3 : 0.8801000143858, 0.9633137591712483
linear 1 3 0.1 4 : 0.8801000143858, 0.9633137591712483
linear 1 3 0.1 5 : 0.8801000143858, 0.9633137591712483
linear 1 3 0.1 6 : 0.8801000143858, 0.9633137591712483
linear 1 3 0.1 7 : 0.8801000143858, 0.9633137591712483
linear 1 3 0.1 8 : 0.8801000143858, 0.9633137591712483
linear 1 3 0.1 9 : 0.8801000143858, 0.9633137591712483
linear 1 3 0.3 3 : 0.8803979122407402, 0.9633178301452152
linear 1 3 0.3 4 : 0.8803979122407402, 0.9633178301452152
linear 1 3 0.3 5 : 0.8803979122407402, 0.9633178301452152
linear 1 3 0.3 6 : 0.8803979122407402, 0.9633178301452152
linear 1 3 0.3 7 : 0.8803979122407402, 0.9633178301452152
linear 1 3 0.3 8 : 0.8803979122407402, 0.9633178301452152
linear 1 3 0.3 9 : 0.8803979122407402, 0.9633178301452152
linear 1 3 0.5 3 : 0.8797813751434311, 0.9631321912792431
linear 1 3 0.5 4 : 0.8797813751434311, 0.9631321912792431
linear 1 3 0.5 5 : 0.8797813751434311, 0.9631321912792431
linear 1 3 0.5 6 : 0.8797813751434311, 0.9631321912792431
linear 1 3 0.5 7 : 0.8797813751434311, 0.9631321912792431
linear 1 3 0.5 8 : 0.8797813751434311, 0.9631321912792431
linear 1 3 0.5 9 : 0.8797813751434311, 0.9631321912792431
linear 1 3 0.7 3 : 0.8784832714203713, 0.9623144443982917
linear 1 3 0.7 4 : 0.8784832714203713, 0.9623144443982917
linear 1 3 0.7 5 : 0.8784832714203713, 0.9623144443982917
linear 1 3 0.7 6 : 0.8784832714203713, 0.9623144443982917
linear 1 3 0.7 7 : 0.8784832714203713, 0.9623144443982917
linear 1 3 0.7 8 : 0.8784832714203713, 0.9623144443982917
linear 1 3 0.7 9 : 0.8784832714203713, 0.9623144443982917
linear 1 3 0.9 3 : 0.878211207610421, 0.9610778179781068
linear 1 3 0.9 4 : 0.878211207610421, 0.9610778179781068
linear 1 3 0.9 5 : 0.878211207610421, 0.9610778179781068
linear 1 3 0.9 6 : 0.878211207610421, 0.9610778179781068
linear 1 3 0.9 7 : 0.878211207610421, 0.9610778179781068
linear 1 3 0.9 8 : 0.878211207610421, 0.9610778179781068
linear 1 3 0.9 9 : 0.878211207610421, 0.9610778179781068
linear 1 4 0.1 3 : 0.8801000143858, 0.9633137591712483
linear 1 4 0.1 4 : 0.8801000143858, 0.9633137591712483
linear 1 4 0.1 5 : 0.8801000143858, 0.9633137591712483
linear 1 4 0.1 6 : 0.8801000143858, 0.9633137591712483
linear 1 4 0.1 7 : 0.8801000143858, 0.9633137591712483
linear 1 4 0.1 8 : 0.8801000143858, 0.9633137591712483
linear 1 4 0.1 9 : 0.8801000143858, 0.9633137591712483
linear 1 4 0.3 3 : 0.8803979122407402, 0.9633178301452152
linear 1 4 0.3 4 : 0.8803979122407402, 0.9633178301452152
linear 1 4 0.3 5 : 0.8803979122407402, 0.9633178301452152
linear 1 4 0.3 6 : 0.8803979122407402, 0.9633178301452152
linear 1 4 0.3 7 : 0.8803979122407402, 0.9633178301452152
linear 1 4 0.3 8 : 0.8803979122407402, 0.9633178301452152
linear 1 4 0.3 9 : 0.8803979122407402, 0.9633178301452152
linear 1 4 0.5 3 : 0.8797813751434311, 0.9631321912792431
linear 1 4 0.5 4 : 0.8797813751434311, 0.9631321912792431
linear 1 4 0.5 5 : 0.8797813751434311, 0.9631321912792431
linear 1 4 0.5 6 : 0.8797813751434311, 0.9631321912792431
linear 1 4 0.5 7 : 0.8797813751434311, 0.9631321912792431
linear 1 4 0.5 8 : 0.8797813751434311, 0.9631321912792431
linear 1 4 0.5 9 : 0.8797813751434311, 0.9631321912792431
linear 1 4 0.7 3 : 0.8784832714203713, 0.9623144443982917

linear 1 4 0.7 4 : 0.8784832714203713, 0.9623144443982917
linear 1 4 0.7 5 : 0.8784832714203713, 0.9623144443982917
linear 1 4 0.7 6 : 0.8784832714203713, 0.9623144443982917
linear 1 4 0.7 7 : 0.8784832714203713, 0.9623144443982917
linear 1 4 0.7 8 : 0.8784832714203713, 0.9623144443982917
linear 1 4 0.7 9 : 0.8784832714203713, 0.9623144443982917
linear 1 4 0.9 3 : 0.878211207610421, 0.9610778179781068
linear 1 4 0.9 4 : 0.878211207610421, 0.9610778179781068
linear 1 4 0.9 5 : 0.878211207610421, 0.9610778179781068
linear 1 4 0.9 6 : 0.878211207610421, 0.9610778179781068
linear 1 4 0.9 7 : 0.878211207610421, 0.9610778179781068
linear 1 4 0.9 8 : 0.878211207610421, 0.9610778179781068
linear 1 4 0.9 9 : 0.878211207610421, 0.9610778179781068
linear 1 5 0.1 3 : 0.8801000143858, 0.9633137591712483
linear 1 5 0.1 4 : 0.8801000143858, 0.9633137591712483
linear 1 5 0.1 5 : 0.8801000143858, 0.9633137591712483
linear 1 5 0.1 6 : 0.8801000143858, 0.9633137591712483
linear 1 5 0.1 7 : 0.8801000143858, 0.9633137591712483
linear 1 5 0.1 8 : 0.8801000143858, 0.9633137591712483
linear 1 5 0.1 9 : 0.8801000143858, 0.9633137591712483
linear 1 5 0.3 3 : 0.8803979122407402, 0.9633178301452152
linear 1 5 0.3 4 : 0.8803979122407402, 0.9633178301452152
linear 1 5 0.3 5 : 0.8803979122407402, 0.9633178301452152
linear 1 5 0.3 6 : 0.8803979122407402, 0.9633178301452152
linear 1 5 0.3 7 : 0.8803979122407402, 0.9633178301452152
linear 1 5 0.3 8 : 0.8803979122407402, 0.9633178301452152
linear 1 5 0.3 9 : 0.8803979122407402, 0.9633178301452152
linear 1 5 0.5 3 : 0.8797813751434311, 0.9631321912792431
linear 1 5 0.5 4 : 0.8797813751434311, 0.9631321912792431
linear 1 5 0.5 5 : 0.8797813751434311, 0.9631321912792431
linear 1 5 0.5 6 : 0.8797813751434311, 0.9631321912792431
linear 1 5 0.5 7 : 0.8797813751434311, 0.9631321912792431
linear 1 5 0.5 8 : 0.8797813751434311, 0.9631321912792431
linear 1 5 0.5 9 : 0.8797813751434311, 0.9631321912792431
linear 1 5 0.7 3 : 0.8784832714203713, 0.9623144443982917
linear 1 5 0.7 4 : 0.8784832714203713, 0.9623144443982917
linear 1 5 0.7 5 : 0.8784832714203713, 0.9623144443982917
linear 1 5 0.7 6 : 0.8784832714203713, 0.9623144443982917
linear 1 5 0.7 7 : 0.8784832714203713, 0.9623144443982917
linear 1 5 0.7 8 : 0.8784832714203713, 0.9623144443982917
linear 1 5 0.7 9 : 0.8784832714203713, 0.9623144443982917
linear 1 5 0.9 3 : 0.878211207610421, 0.9610778179781068
linear 1 5 0.9 4 : 0.878211207610421, 0.9610778179781068
linear 1 5 0.9 5 : 0.878211207610421, 0.9610778179781068
linear 1 5 0.9 6 : 0.878211207610421, 0.9610778179781068
linear 1 5 0.9 7 : 0.878211207610421, 0.9610778179781068
linear 1 5 0.9 8 : 0.878211207610421, 0.9610778179781068
linear 1 5 0.9 9 : 0.878211207610421, 0.9610778179781068
linear 1 6 0.1 3 : 0.8801000143858, 0.9633137591712483
linear 1 6 0.1 4 : 0.8801000143858, 0.9633137591712483
linear 1 6 0.1 5 : 0.8801000143858, 0.9633137591712483
linear 1 6 0.1 6 : 0.8801000143858, 0.9633137591712483
linear 1 6 0.1 7 : 0.8801000143858, 0.9633137591712483
linear 1 6 0.1 8 : 0.8801000143858, 0.9633137591712483
linear 1 6 0.1 9 : 0.8801000143858, 0.9633137591712483
linear 1 6 0.3 3 : 0.8803979122407402, 0.9633178301452152
linear 1 6 0.3 4 : 0.8803979122407402, 0.9633178301452152
linear 1 6 0.3 5 : 0.8803979122407402, 0.9633178301452152
linear 1 6 0.3 6 : 0.8803979122407402, 0.9633178301452152
linear 1 6 0.3 7 : 0.8803979122407402, 0.9633178301452152
linear 1 6 0.3 8 : 0.8803979122407402, 0.9633178301452152
linear 1 6 0.3 9 : 0.8803979122407402, 0.9633178301452152
linear 1 6 0.5 3 : 0.8797813751434311, 0.9631321912792431
linear 1 6 0.5 4 : 0.8797813751434311, 0.9631321912792431
linear 1 6 0.5 5 : 0.8797813751434311, 0.9631321912792431
linear 1 6 0.5 6 : 0.8797813751434311, 0.9631321912792431
linear 1 6 0.5 7 : 0.8797813751434311, 0.9631321912792431
linear 1 6 0.5 8 : 0.8797813751434311, 0.9631321912792431
linear 1 6 0.5 9 : 0.8797813751434311, 0.9631321912792431
linear 1 6 0.7 3 : 0.8784832714203713, 0.9623144443982917
linear 1 6 0.7 4 : 0.8784832714203713, 0.9623144443982917
linear 1 6 0.7 5 : 0.8784832714203713, 0.9623144443982917
linear 1 6 0.7 6 : 0.8784832714203713, 0.9623144443982917
linear 1 6 0.7 7 : 0.8784832714203713, 0.9623144443982917
linear 1 6 0.7 8 : 0.8784832714203713, 0.9623144443982917
linear 1 6 0.7 9 : 0.8784832714203713, 0.9623144443982917
linear 1 6 0.9 3 : 0.878211207610421, 0.9610778179781068
linear 1 6 0.9 4 : 0.878211207610421, 0.9610778179781068
linear 1 6 0.9 5 : 0.878211207610421, 0.9610778179781068
linear 1 6 0.9 6 : 0.878211207610421, 0.9610778179781068
linear 1 6 0.9 7 : 0.878211207610421, 0.9610778179781068
linear 1 6 0.9 8 : 0.878211207610421, 0.9610778179781068
linear 1 6 0.9 9 : 0.878211207610421, 0.9610778179781068
linear 2 1 0.1 3 : 0.9070412034008541, 0.9702769207981706
linear 2 1 0.1 4 : 0.9070412034008541, 0.9702769207981706
linear 2 1 0.1 5 : 0.9070412034008541, 0.9702769207981706
linear 2 1 0.1 6 : 0.9070412034008541, 0.9702769207981706
linear 2 1 0.1 7 : 0.9070412034008541, 0.9702769207981706
linear 2 1 0.1 8 : 0.9070412034008541, 0.9702769207981706
linear 2 1 0.1 9 : 0.9070412034008541, 0.9702769207981706
linear 2 1 0.3 3 : 0.9074193172874254, 0.9701151690213567
linear 2 1 0.3 4 : 0.9074193172874254, 0.9701151690213567
linear 2 1 0.3 5 : 0.9074193172874254, 0.9701151690213567
linear 2 1 0.3 6 : 0.9074193172874254, 0.9701151690213567
linear 2 1 0.3 7 : 0.9074193172874254, 0.9701151690213567
linear 2 1 0.3 8 : 0.9074193172874254, 0.9701151690213567
linear 2 1 0.3 9 : 0.9074193172874254, 0.9701151690213567
linear 2 1 0.5 3 : 0.9072887848616572, 0.9692256182209725
linear 2 1 0.5 4 : 0.9072887848616572, 0.9692256182209725
linear 2 1 0.5 5 : 0.9072887848616572, 0.9692256182209725
linear 2 1 0.5 6 : 0.9072887848616572, 0.9692256182209725
linear 2 1 0.5 7 : 0.9072887848616572, 0.9692256182209725
linear 2 1 0.5 8 : 0.9072887848616572, 0.9692256182209725
linear 2 1 0.5 9 : 0.9072887848616572, 0.9692256182209725
linear 2 1 0.7 3 : 0.9069068614210171, 0.9683557068464161
linear 2 1 0.7 4 : 0.9069068614210171, 0.9683557068464161
linear 2 1 0.7 5 : 0.9069068614210171, 0.9683557068464161
linear 2 1 0.7 6 : 0.9069068614210171, 0.9683557068464161
linear 2 1 0.7 7 : 0.9069068614210171, 0.9683557068464161
linear 2 1 0.7 8 : 0.9069068614210171, 0.9683557068464161
linear 2 1 0.7 9 : 0.9069068614210171, 0.9683557068464161
linear 2 1 0.9 3 : 0.9056483473780481, 0.9668855554697882
linear 2 1 0.9 4 : 0.9056483473780481, 0.9668855554697882
linear 2 1 0.9 5 : 0.9056483473780481, 0.9668855554697882
linear 2 1 0.9 6 : 0.9056483473780481, 0.9668855554697882
linear 2 1 0.9 7 : 0.9056483473780481, 0.9668855554697882
linear 2 1 0.9 8 : 0.9056483473780481, 0.9668855554697882
linear 2 1 0.9 9 : 0.9056483473780481, 0.9668855554697882
linear 2 2 0.1 3 : 0.9070412034008541, 0.9702769207981706
linear 2 2 0.1 4 : 0.9070412034008541, 0.9702769207981706
linear 2 2 0.1 5 : 0.9070412034008541, 0.9702769207981706
linear 2 2 0.1 6 : 0.9070412034008541, 0.9702769207981706
linear 2 2 0.1 7 : 0.9070412034008541, 0.9702769207981706
linear 2 2 0.1 8 : 0.9070412034008541, 0.9702769207981706
linear 2 2 0.1 9 : 0.9070412034008541, 0.9702769207981706
linear 2 2 0.3 3 : 0.9074193172874254, 0.9701151690213567
linear 2 2 0.3 4 : 0.9074193172874254, 0.9701151690213567
linear 2 2 0.3 5 : 0.9074193172874254, 0.9701151690213567
linear 2 2 0.3 6 : 0.9074193172874254, 0.9701151690213567
linear 2 2 0.3 7 : 0.9074193172874254, 0.9701151690213567
linear 2 2 0.3 8 : 0.9074193172874254, 0.9701151690213567
linear 2 2 0.3 9 : 0.9074193172874254, 0.9701151690213567
linear 2 2 0.5 3 : 0.9072887848616572, 0.9692256182209725
linear 2 2 0.5 4 : 0.9072887848616572, 0.9692256182209725
linear 2 2 0.5 5 : 0.9072887848616572, 0.9692256182209725
linear 2 2 0.5 6 : 0.9072887848616572, 0.9692256182209725
linear 2 2 0.5 7 : 0.9072887848616572, 0.9692256182209725
linear 2 2 0.5 8 : 0.9072887848616572, 0.9692256182209725
linear 2 2 0.5 9 : 0.9072887848616572, 0.9692256182209725
linear 2 2 0.7 3 : 0.9069068614210171, 0.9683557068464161
linear 2 2 0.7 4 : 0.9069068614210171, 0.9683557068464161
linear 2 2 0.7 5 : 0.9069068614210171, 0.9683557068464161
linear 2 2 0.7 6 : 0.9069068614210171, 0.9683557068464161
linear 2 2 0.7 7 : 0.9069068614210171, 0.9683557068464161
linear 2 2 0.7 8 : 0.9069068614210171, 0.9683557068464161
linear 2 2 0.7 9 : 0.9069068614210171, 0.9683557068464161
linear 2 2 0.9 3 : 0.9056483473780481, 0.9668855554697882
linear 2 2 0.9 4 : 0.9056483473780481, 0.9668855554697882

linear 2 2 0.9 5 : 0.9056483473780481, 0.9668855554697882
linear 2 2 0.9 6 : 0.9056483473780481, 0.9668855554697882
linear 2 2 0.9 7 : 0.9056483473780481, 0.9668855554697882
linear 2 2 0.9 8 : 0.9056483473780481, 0.9668855554697882
linear 2 2 0.9 9 : 0.9056483473780481, 0.9668855554697882
linear 2 3 0.1 3 : 0.9070412034008541, 0.9702769207981706
linear 2 3 0.1 4 : 0.9070412034008541, 0.9702769207981706
linear 2 3 0.1 5 : 0.9070412034008541, 0.9702769207981706
linear 2 3 0.1 6 : 0.9070412034008541, 0.9702769207981706
linear 2 3 0.1 7 : 0.9070412034008541, 0.9702769207981706
linear 2 3 0.1 8 : 0.9070412034008541, 0.9702769207981706
linear 2 3 0.1 9 : 0.9070412034008541, 0.9702769207981706
linear 2 3 0.3 3 : 0.9074193172874254, 0.9701151690213567
linear 2 3 0.3 4 : 0.9074193172874254, 0.9701151690213567
linear 2 3 0.3 5 : 0.9074193172874254, 0.9701151690213567
linear 2 3 0.3 6 : 0.9074193172874254, 0.9701151690213567
linear 2 3 0.3 7 : 0.9074193172874254, 0.9701151690213567
linear 2 3 0.3 8 : 0.9074193172874254, 0.9701151690213567
linear 2 3 0.3 9 : 0.9074193172874254, 0.9701151690213567
linear 2 3 0.5 3 : 0.9072887848616572, 0.9692256182209725
linear 2 3 0.5 4 : 0.9072887848616572, 0.9692256182209725
linear 2 3 0.5 5 : 0.9072887848616572, 0.9692256182209725
linear 2 3 0.5 6 : 0.9072887848616572, 0.9692256182209725
linear 2 3 0.5 7 : 0.9072887848616572, 0.9692256182209725
linear 2 3 0.5 8 : 0.9072887848616572, 0.9692256182209725
linear 2 3 0.5 9 : 0.9072887848616572, 0.9692256182209725
linear 2 3 0.7 3 : 0.9069068614210171, 0.9683557068464161
linear 2 3 0.7 4 : 0.9069068614210171, 0.9683557068464161
linear 2 3 0.7 5 : 0.9069068614210171, 0.9683557068464161
linear 2 3 0.7 6 : 0.9069068614210171, 0.9683557068464161
linear 2 3 0.7 7 : 0.9069068614210171, 0.9683557068464161
linear 2 3 0.7 8 : 0.9069068614210171, 0.9683557068464161
linear 2 3 0.7 9 : 0.9069068614210171, 0.9683557068464161
linear 2 3 0.9 3 : 0.9056483473780481, 0.9668855554697882
linear 2 3 0.9 4 : 0.9056483473780481, 0.9668855554697882
linear 2 3 0.9 5 : 0.9056483473780481, 0.9668855554697882
linear 2 3 0.9 6 : 0.9056483473780481, 0.9668855554697882
linear 2 3 0.9 7 : 0.9056483473780481, 0.9668855554697882
linear 2 3 0.9 8 : 0.9056483473780481, 0.9668855554697882
linear 2 3 0.9 9 : 0.9056483473780481, 0.9668855554697882
linear 2 4 0.1 3 : 0.9070412034008541, 0.9702769207981706
linear 2 4 0.1 4 : 0.9070412034008541, 0.9702769207981706
linear 2 4 0.1 5 : 0.9070412034008541, 0.9702769207981706
linear 2 4 0.1 6 : 0.9070412034008541, 0.9702769207981706
linear 2 4 0.1 7 : 0.9070412034008541, 0.9702769207981706
linear 2 4 0.1 8 : 0.9070412034008541, 0.9702769207981706
linear 2 4 0.1 9 : 0.9070412034008541, 0.9702769207981706
linear 2 4 0.3 3 : 0.9074193172874254, 0.9701151690213567
linear 2 4 0.3 4 : 0.9074193172874254, 0.9701151690213567
linear 2 4 0.3 5 : 0.9074193172874254, 0.9701151690213567
linear 2 4 0.3 6 : 0.9074193172874254, 0.9701151690213567
linear 2 4 0.3 7 : 0.9074193172874254, 0.9701151690213567
linear 2 4 0.3 8 : 0.9074193172874254, 0.9701151690213567
linear 2 4 0.3 9 : 0.9074193172874254, 0.9701151690213567
linear 2 4 0.5 3 : 0.9072887848616572, 0.9692256182209725
linear 2 4 0.5 4 : 0.9072887848616572, 0.9692256182209725
linear 2 4 0.5 5 : 0.9072887848616572, 0.9692256182209725
linear 2 4 0.5 6 : 0.9072887848616572, 0.9692256182209725
linear 2 4 0.5 7 : 0.9072887848616572, 0.9692256182209725
linear 2 4 0.5 8 : 0.9072887848616572, 0.9692256182209725
linear 2 4 0.5 9 : 0.9072887848616572, 0.9692256182209725
linear 2 4 0.7 3 : 0.9069068614210171, 0.9683557068464161
linear 2 4 0.7 4 : 0.9069068614210171, 0.9683557068464161
linear 2 4 0.7 5 : 0.9069068614210171, 0.9683557068464161
linear 2 4 0.7 6 : 0.9069068614210171, 0.9683557068464161
linear 2 4 0.7 7 : 0.9069068614210171, 0.9683557068464161
linear 2 4 0.7 8 : 0.9069068614210171, 0.9683557068464161
linear 2 4 0.7 9 : 0.9069068614210171, 0.9683557068464161
linear 2 4 0.9 3 : 0.9056483473780481, 0.9668855554697882
linear 2 4 0.9 4 : 0.9056483473780481, 0.9668855554697882
linear 2 4 0.9 5 : 0.9056483473780481, 0.9668855554697882
linear 2 4 0.9 6 : 0.9056483473780481, 0.9668855554697882
linear 2 4 0.9 7 : 0.9056483473780481, 0.9668855554697882
linear 2 4 0.9 8 : 0.9056483473780481, 0.9668855554697882
linear 2 4 0.9 9 : 0.9056483473780481, 0.9668855554697882
linear 2 5 0.1 3 : 0.9070412034008541, 0.9702769207981706
linear 2 5 0.1 4 : 0.9070412034008541, 0.9702769207981706
linear 2 5 0.1 5 : 0.9070412034008541, 0.9702769207981706
linear 2 5 0.1 6 : 0.9070412034008541, 0.9702769207981706
linear 2 5 0.1 7 : 0.9070412034008541, 0.9702769207981706
linear 2 5 0.1 8 : 0.9070412034008541, 0.9702769207981706
linear 2 5 0.1 9 : 0.9070412034008541, 0.9702769207981706
linear 2 5 0.3 3 : 0.9074193172874254, 0.9701151690213567
linear 2 5 0.3 4 : 0.9074193172874254, 0.9701151690213567
linear 2 5 0.3 5 : 0.9074193172874254, 0.9701151690213567
linear 2 5 0.3 6 : 0.9074193172874254, 0.9701151690213567
linear 2 5 0.3 7 : 0.9074193172874254, 0.9701151690213567
linear 2 5 0.3 8 : 0.9074193172874254, 0.9701151690213567
linear 2 5 0.3 9 : 0.9074193172874254, 0.9701151690213567
linear 2 5 0.5 3 : 0.9072887848616572, 0.9692256182209725
linear 2 5 0.5 4 : 0.9072887848616572, 0.9692256182209725
linear 2 5 0.5 5 : 0.9072887848616572, 0.9692256182209725
linear 2 5 0.5 6 : 0.9072887848616572, 0.9692256182209725
linear 2 5 0.5 7 : 0.9072887848616572, 0.9692256182209725
linear 2 5 0.5 8 : 0.9072887848616572, 0.9692256182209725
linear 2 5 0.5 9 : 0.9072887848616572, 0.9692256182209725
linear 2 5 0.7 3 : 0.9069068614210171, 0.9683557068464161
linear 2 5 0.7 4 : 0.9069068614210171, 0.9683557068464161
linear 2 5 0.7 5 : 0.9069068614210171, 0.9683557068464161
linear 2 5 0.7 6 : 0.9069068614210171, 0.9683557068464161
linear 2 5 0.7 7 : 0.9069068614210171, 0.9683557068464161
linear 2 5 0.7 8 : 0.9069068614210171, 0.9683557068464161
linear 2 5 0.7 9 : 0.9069068614210171, 0.9683557068464161
linear 2 5 0.9 3 : 0.9056483473780481, 0.9668855554697882
linear 2 5 0.9 4 : 0.9056483473780481, 0.9668855554697882
linear 2 5 0.9 5 : 0.9056483473780481, 0.9668855554697882
linear 2 5 0.9 6 : 0.9056483473780481, 0.9668855554697882
linear 2 5 0.9 7 : 0.9056483473780481, 0.9668855554697882
linear 2 5 0.9 8 : 0.9056483473780481, 0.9668855554697882
linear 2 5 0.9 9 : 0.9056483473780481, 0.9668855554697882
linear 2 6 0.1 3 : 0.9070412034008541, 0.9702769207981706
linear 2 6 0.1 4 : 0.9070412034008541, 0.9702769207981706
linear 2 6 0.1 5 : 0.9070412034008541, 0.9702769207981706
linear 2 6 0.1 6 : 0.9070412034008541, 0.9702769207981706
linear 2 6 0.1 7 : 0.9070412034008541, 0.9702769207981706
linear 2 6 0.1 8 : 0.9070412034008541, 0.9702769207981706
linear 2 6 0.1 9 : 0.9070412034008541, 0.9702769207981706
linear 2 6 0.3 3 : 0.9074193172874254, 0.9701151690213567
linear 2 6 0.3 4 : 0.9074193172874254, 0.9701151690213567
linear 2 6 0.3 5 : 0.9074193172874254, 0.9701151690213567
linear 2 6 0.3 6 : 0.9074193172874254, 0.9701151690213567
linear 2 6 0.3 7 : 0.9074193172874254, 0.9701151690213567
linear 2 6 0.3 8 : 0.9074193172874254, 0.9701151690213567
linear 2 6 0.3 9 : 0.9074193172874254, 0.9701151690213567
linear 2 6 0.5 3 : 0.9072887848616572, 0.9692256182209725
linear 2 6 0.5 4 : 0.9072887848616572, 0.9692256182209725
linear 2 6 0.5 5 : 0.9072887848616572, 0.9692256182209725
linear 2 6 0.5 6 : 0.9072887848616572, 0.9692256182209725
linear 2 6 0.5 7 : 0.9072887848616572, 0.9692256182209725
linear 2 6 0.5 8 : 0.9072887848616572, 0.9692256182209725
linear 2 6 0.5 9 : 0.9072887848616572, 0.9692256182209725
linear 2 6 0.7 3 : 0.9069068614210171, 0.9683557068464161
linear 2 6 0.7 4 : 0.9069068614210171, 0.9683557068464161
linear 2 6 0.7 5 : 0.9069068614210171, 0.9683557068464161
linear 2 6 0.7 6 : 0.9069068614210171, 0.9683557068464161
linear 2 6 0.7 7 : 0.9069068614210171, 0.9683557068464161
linear 2 6 0.7 8 : 0.9069068614210171, 0.9683557068464161
linear 2 6 0.7 9 : 0.9069068614210171, 0.9683557068464161
linear 2 6 0.9 3 : 0.9056483473780481, 0.9668855554697882
linear 2 6 0.9 4 : 0.9056483473780481, 0.9668855554697882
linear 2 6 0.9 5 : 0.9056483473780481, 0.9668855554697882
linear 2 6 0.9 6 : 0.9056483473780481, 0.9668855554697882
linear 2 6 0.9 7 : 0.9056483473780481, 0.9668855554697882
linear 2 6 0.9 8 : 0.9056483473780481, 0.9668855554697882
linear 2 6 0.9 9 : 0.9056483473780481, 0.9668855554697882
linear 3 1 0.1 3 : 0.9137097602122657, 0.9687209734721898
linear 3 1 0.1 4 : 0.9137097602122657, 0.9687209734721898
linear 3 1 0.1 5 : 0.9137097602122657, 0.9687209734721898

linear 3 1 0.1 6 : 0.9137097602122657, 0.9687209734721898
linear 3 1 0.1 7 : 0.9137097602122657, 0.9687209734721898
linear 3 1 0.1 8 : 0.9137097602122657, 0.9687209734721898
linear 3 1 0.1 9 : 0.9137097602122657, 0.9687209734721898
linear 3 1 0.3 3 : 0.9145027780615266, 0.9684280114215711
linear 3 1 0.3 4 : 0.9145027780615266, 0.9684280114215711
linear 3 1 0.3 5 : 0.9145027780615266, 0.9684280114215711
linear 3 1 0.3 6 : 0.9145027780615266, 0.9684280114215711
linear 3 1 0.3 7 : 0.9145027780615266, 0.9684280114215711
linear 3 1 0.3 8 : 0.9145027780615266, 0.9684280114215711
linear 3 1 0.3 9 : 0.9145027780615266, 0.9684280114215711
linear 3 1 0.5 3 : 0.9145260563061983, 0.9680204421671282
linear 3 1 0.5 4 : 0.9145260563061983, 0.9680204421671282
linear 3 1 0.5 5 : 0.9145260563061983, 0.9680204421671282
linear 3 1 0.5 6 : 0.9145260563061983, 0.9680204421671282
linear 3 1 0.5 7 : 0.9145260563061983, 0.9680204421671282
linear 3 1 0.5 8 : 0.9145260563061983, 0.9680204421671282
linear 3 1 0.5 9 : 0.9145260563061983, 0.9680204421671282
linear 3 1 0.7 3 : 0.9133757896301257, 0.9676312873604451
linear 3 1 0.7 4 : 0.9133757896301257, 0.9676312873604451
linear 3 1 0.7 5 : 0.9133757896301257, 0.9676312873604451
linear 3 1 0.7 6 : 0.9133757896301257, 0.9676312873604451
linear 3 1 0.7 7 : 0.9133757896301257, 0.9676312873604451
linear 3 1 0.7 8 : 0.9133757896301257, 0.9676312873604451
linear 3 1 0.7 9 : 0.9133757896301257, 0.9676312873604451
linear 3 1 0.9 3 : 0.9118723998238563, 0.9661912163179954
linear 3 1 0.9 4 : 0.9118723998238563, 0.9661912163179954
linear 3 1 0.9 5 : 0.9118723998238563, 0.9661912163179954
linear 3 1 0.9 6 : 0.9118723998238563, 0.9661912163179954
linear 3 1 0.9 7 : 0.9118723998238563, 0.9661912163179954
linear 3 1 0.9 8 : 0.9118723998238563, 0.9661912163179954
linear 3 1 0.9 9 : 0.9118723998238563, 0.9661912163179954
linear 3 2 0.1 3 : 0.9137097602122657, 0.9687209734721898
linear 3 2 0.1 4 : 0.9137097602122657, 0.9687209734721898
linear 3 2 0.1 5 : 0.9137097602122657, 0.9687209734721898
linear 3 2 0.1 6 : 0.9137097602122657, 0.9687209734721898
linear 3 2 0.1 7 : 0.9137097602122657, 0.9687209734721898
linear 3 2 0.1 8 : 0.9137097602122657, 0.9687209734721898
linear 3 2 0.1 9 : 0.9137097602122657, 0.9687209734721898
linear 3 2 0.3 3 : 0.9145027780615266, 0.9684280114215711
linear 3 2 0.3 4 : 0.9145027780615266, 0.9684280114215711
linear 3 2 0.3 5 : 0.9145027780615266, 0.9684280114215711
linear 3 2 0.3 6 : 0.9145027780615266, 0.9684280114215711
linear 3 2 0.3 7 : 0.9145027780615266, 0.9684280114215711
linear 3 2 0.3 8 : 0.9145027780615266, 0.9684280114215711
linear 3 2 0.3 9 : 0.9145027780615266, 0.9684280114215711
linear 3 2 0.5 3 : 0.9145260563061983, 0.9680204421671282
linear 3 2 0.5 4 : 0.9145260563061983, 0.9680204421671282
linear 3 2 0.5 5 : 0.9145260563061983, 0.9680204421671282
linear 3 2 0.5 6 : 0.9145260563061983, 0.9680204421671282
linear 3 2 0.5 7 : 0.9145260563061983, 0.9680204421671282
linear 3 2 0.5 8 : 0.9145260563061983, 0.9680204421671282
linear 3 2 0.5 9 : 0.9145260563061983, 0.9680204421671282
linear 3 2 0.7 3 : 0.9133757896301257, 0.9676312873604451
linear 3 2 0.7 4 : 0.9133757896301257, 0.9676312873604451
linear 3 2 0.7 5 : 0.9133757896301257, 0.9676312873604451
linear 3 2 0.7 6 : 0.9133757896301257, 0.9676312873604451
linear 3 2 0.7 7 : 0.9133757896301257, 0.9676312873604451
linear 3 2 0.7 8 : 0.9133757896301257, 0.9676312873604451
linear 3 2 0.7 9 : 0.9133757896301257, 0.9676312873604451
linear 3 2 0.9 3 : 0.9118723998238563, 0.9661912163179954
linear 3 2 0.9 4 : 0.9118723998238563, 0.9661912163179954
linear 3 2 0.9 5 : 0.9118723998238563, 0.9661912163179954
linear 3 2 0.9 6 : 0.9118723998238563, 0.9661912163179954
linear 3 2 0.9 7 : 0.9118723998238563, 0.9661912163179954
linear 3 2 0.9 8 : 0.9118723998238563, 0.9661912163179954
linear 3 2 0.9 9 : 0.9118723998238563, 0.9661912163179954
linear 3 3 0.1 3 : 0.9137097602122657, 0.9687209734721898
linear 3 3 0.1 4 : 0.9137097602122657, 0.9687209734721898
linear 3 3 0.1 5 : 0.9137097602122657, 0.9687209734721898
linear 3 3 0.1 6 : 0.9137097602122657, 0.9687209734721898
linear 3 3 0.1 7 : 0.9137097602122657, 0.9687209734721898
linear 3 3 0.1 8 : 0.9137097602122657, 0.9687209734721898
linear 3 3 0.1 9 : 0.9137097602122657, 0.9687209734721898
linear 3 3 0.3 3 : 0.9145027780615266, 0.9684280114215711
linear 3 3 0.3 4 : 0.9145027780615266, 0.9684280114215711
linear 3 3 0.3 5 : 0.9145027780615266, 0.9684280114215711
linear 3 3 0.3 6 : 0.9145027780615266, 0.9684280114215711
linear 3 3 0.3 7 : 0.9145027780615266, 0.9684280114215711
linear 3 3 0.3 8 : 0.9145027780615266, 0.9684280114215711
linear 3 3 0.3 9 : 0.9145027780615266, 0.9684280114215711
linear 3 3 0.5 3 : 0.9145260563061983, 0.9680204421671282
linear 3 3 0.5 4 : 0.9145260563061983, 0.9680204421671282
linear 3 3 0.5 5 : 0.9145260563061983, 0.9680204421671282
linear 3 3 0.5 6 : 0.9145260563061983, 0.9680204421671282
linear 3 3 0.5 7 : 0.9145260563061983, 0.9680204421671282
linear 3 3 0.5 8 : 0.9145260563061983, 0.9680204421671282
linear 3 3 0.5 9 : 0.9145260563061983, 0.9680204421671282
linear 3 3 0.7 3 : 0.9133757896301257, 0.9676312873604451
linear 3 3 0.7 4 : 0.9133757896301257, 0.9676312873604451
linear 3 3 0.7 5 : 0.9133757896301257, 0.9676312873604451
linear 3 3 0.7 6 : 0.9133757896301257, 0.9676312873604451
linear 3 3 0.7 7 : 0.9133757896301257, 0.9676312873604451
linear 3 3 0.7 8 : 0.9133757896301257, 0.9676312873604451
linear 3 3 0.7 9 : 0.9133757896301257, 0.9676312873604451
linear 3 3 0.9 3 : 0.9118723998238563, 0.9661912163179954
linear 3 3 0.9 4 : 0.9118723998238563, 0.9661912163179954
linear 3 3 0.9 5 : 0.9118723998238563, 0.9661912163179954
linear 3 3 0.9 6 : 0.9118723998238563, 0.9661912163179954
linear 3 3 0.9 7 : 0.9118723998238563, 0.9661912163179954
linear 3 3 0.9 8 : 0.9118723998238563, 0.9661912163179954
linear 3 3 0.9 9 : 0.9118723998238563, 0.9661912163179954
linear 3 4 0.1 3 : 0.9137097602122657, 0.9687209734721898
linear 3 4 0.1 4 : 0.9137097602122657, 0.9687209734721898
linear 3 4 0.1 5 : 0.9137097602122657, 0.9687209734721898
linear 3 4 0.1 6 : 0.9137097602122657, 0.9687209734721898
linear 3 4 0.1 7 : 0.9137097602122657, 0.9687209734721898
linear 3 4 0.1 8 : 0.9137097602122657, 0.9687209734721898
linear 3 4 0.1 9 : 0.9137097602122657, 0.9687209734721898
linear 3 4 0.3 3 : 0.9145027780615266, 0.9684280114215711
linear 3 4 0.3 4 : 0.9145027780615266, 0.9684280114215711
linear 3 4 0.3 5 : 0.9145027780615266, 0.9684280114215711
linear 3 4 0.3 6 : 0.9145027780615266, 0.9684280114215711
linear 3 4 0.3 7 : 0.9145027780615266, 0.9684280114215711
linear 3 4 0.3 8 : 0.9145027780615266, 0.9684280114215711
linear 3 4 0.3 9 : 0.9145027780615266, 0.9684280114215711
linear 3 4 0.5 3 : 0.9145260563061983, 0.9680204421671282
linear 3 4 0.5 4 : 0.9145260563061983, 0.9680204421671282
linear 3 4 0.5 5 : 0.9145260563061983, 0.9680204421671282
linear 3 4 0.5 6 : 0.9145260563061983, 0.9680204421671282
linear 3 4 0.5 7 : 0.9145260563061983, 0.9680204421671282
linear 3 4 0.5 8 : 0.9145260563061983, 0.9680204421671282
linear 3 4 0.5 9 : 0.9145260563061983, 0.9680204421671282
linear 3 4 0.7 3 : 0.9133757896301257, 0.9676312873604451
linear 3 4 0.7 4 : 0.9133757896301257, 0.9676312873604451
linear 3 4 0.7 5 : 0.9133757896301257, 0.9676312873604451
linear 3 4 0.7 6 : 0.9133757896301257, 0.9676312873604451
linear 3 4 0.7 7 : 0.9133757896301257, 0.9676312873604451
linear 3 4 0.7 8 : 0.9133757896301257, 0.9676312873604451
linear 3 4 0.7 9 : 0.9133757896301257, 0.9676312873604451
linear 3 4 0.9 3 : 0.9118723998238563, 0.9661912163179954
linear 3 4 0.9 4 : 0.9118723998238563, 0.9661912163179954
linear 3 4 0.9 5 : 0.9118723998238563, 0.9661912163179954
linear 3 4 0.9 6 : 0.9118723998238563, 0.9661912163179954
linear 3 4 0.9 7 : 0.9118723998238563, 0.9661912163179954
linear 3 4 0.9 8 : 0.9118723998238563, 0.9661912163179954
linear 3 4 0.9 9 : 0.9118723998238563, 0.9661912163179954
linear 3 5 0.1 3 : 0.9137097602122657, 0.9687209734721898
linear 3 5 0.1 4 : 0.9137097602122657, 0.9687209734721898
linear 3 5 0.1 5 : 0.9137097602122657, 0.9687209734721898
linear 3 5 0.1 6 : 0.9137097602122657, 0.9687209734721898
linear 3 5 0.1 7 : 0.9137097602122657, 0.9687209734721898
linear 3 5 0.1 8 : 0.9137097602122657, 0.9687209734721898
linear 3 5 0.1 9 : 0.9137097602122657, 0.9687209734721898
linear 3 5 0.3 3 : 0.9145027780615266, 0.9684280114215711
linear 3 5 0.3 4 : 0.9145027780615266, 0.9684280114215711
linear 3 5 0.3 5 : 0.9145027780615266, 0.9684280114215711
linear 3 5 0.3 6 : 0.9145027780615266, 0.9684280114215711

linear 3 5 0.3 7 : 0.9145027780615266, 0.9684280114215711
linear 3 5 0.3 8 : 0.9145027780615266, 0.9684280114215711
linear 3 5 0.3 9 : 0.9145027780615266, 0.9684280114215711
linear 3 5 0.5 3 : 0.9145260563061983, 0.9680204421671282
linear 3 5 0.5 4 : 0.9145260563061983, 0.9680204421671282
linear 3 5 0.5 5 : 0.9145260563061983, 0.9680204421671282
linear 3 5 0.5 6 : 0.9145260563061983, 0.9680204421671282
linear 3 5 0.5 7 : 0.9145260563061983, 0.9680204421671282
linear 3 5 0.5 8 : 0.9145260563061983, 0.9680204421671282
linear 3 5 0.5 9 : 0.9145260563061983, 0.9680204421671282
linear 3 5 0.7 3 : 0.9133757896301257, 0.9676312873604451
linear 3 5 0.7 4 : 0.9133757896301257, 0.9676312873604451
linear 3 5 0.7 5 : 0.9133757896301257, 0.9676312873604451
linear 3 5 0.7 6 : 0.9133757896301257, 0.9676312873604451
linear 3 5 0.7 7 : 0.9133757896301257, 0.9676312873604451
linear 3 5 0.7 8 : 0.9133757896301257, 0.9676312873604451
linear 3 5 0.7 9 : 0.9133757896301257, 0.9676312873604451
linear 3 5 0.9 3 : 0.9118723998238563, 0.9661912163179954
linear 3 5 0.9 4 : 0.9118723998238563, 0.9661912163179954
linear 3 5 0.9 5 : 0.9118723998238563, 0.9661912163179954
linear 3 5 0.9 6 : 0.9118723998238563, 0.9661912163179954
linear 3 5 0.9 7 : 0.9118723998238563, 0.9661912163179954
linear 3 5 0.9 8 : 0.9118723998238563, 0.9661912163179954
linear 3 5 0.9 9 : 0.9118723998238563, 0.9661912163179954
linear 3 6 0.1 3 : 0.9137097602122657, 0.9687209734721898
linear 3 6 0.1 4 : 0.9137097602122657, 0.9687209734721898
linear 3 6 0.1 5 : 0.9137097602122657, 0.9687209734721898
linear 3 6 0.1 6 : 0.9137097602122657, 0.9687209734721898
linear 3 6 0.1 7 : 0.9137097602122657, 0.9687209734721898
linear 3 6 0.1 8 : 0.9137097602122657, 0.9687209734721898
linear 3 6 0.1 9 : 0.9137097602122657, 0.9687209734721898
linear 3 6 0.3 3 : 0.9145027780615266, 0.9684280114215711
linear 3 6 0.3 4 : 0.9145027780615266, 0.9684280114215711
linear 3 6 0.3 5 : 0.9145027780615266, 0.9684280114215711
linear 3 6 0.3 6 : 0.9145027780615266, 0.9684280114215711
linear 3 6 0.3 7 : 0.9145027780615266, 0.9684280114215711
linear 3 6 0.3 8 : 0.9145027780615266, 0.9684280114215711
linear 3 6 0.3 9 : 0.9145027780615266, 0.9684280114215711
linear 3 6 0.5 3 : 0.9145260563061983, 0.9680204421671282
linear 3 6 0.5 4 : 0.9145260563061983, 0.9680204421671282
linear 3 6 0.5 5 : 0.9145260563061983, 0.9680204421671282
linear 3 6 0.5 6 : 0.9145260563061983, 0.9680204421671282
linear 3 6 0.5 7 : 0.9145260563061983, 0.9680204421671282
linear 3 6 0.5 8 : 0.9145260563061983, 0.9680204421671282
linear 3 6 0.5 9 : 0.9145260563061983, 0.9680204421671282
linear 3 6 0.7 3 : 0.9133757896301257, 0.9676312873604451
linear 3 6 0.7 4 : 0.9133757896301257, 0.9676312873604451
linear 3 6 0.7 5 : 0.9133757896301257, 0.9676312873604451
linear 3 6 0.7 6 : 0.9133757896301257, 0.9676312873604451
linear 3 6 0.7 7 : 0.9133757896301257, 0.9676312873604451
linear 3 6 0.7 8 : 0.9133757896301257, 0.9676312873604451
linear 3 6 0.7 9 : 0.9133757896301257, 0.9676312873604451
linear 3 6 0.9 3 : 0.9118723998238563, 0.9661912163179954
linear 3 6 0.9 4 : 0.9118723998238563, 0.9661912163179954
linear 3 6 0.9 5 : 0.9118723998238563, 0.9661912163179954
linear 3 6 0.9 6 : 0.9118723998238563, 0.9661912163179954
linear 3 6 0.9 7 : 0.9118723998238563, 0.9661912163179954
linear 3 6 0.9 8 : 0.9118723998238563, 0.9661912163179954
linear 3 6 0.9 9 : 0.9118723998238563, 0.9661912163179954
linear 4 1 0.1 3 : 0.9152615278662795, 0.9691228879070022
linear 4 1 0.1 4 : 0.9152615278662795, 0.9691228879070022
linear 4 1 0.1 5 : 0.9152615278662795, 0.9691228879070022
linear 4 1 0.1 6 : 0.9152615278662795, 0.9691228879070022
linear 4 1 0.1 7 : 0.9152615278662795, 0.9691228879070022
linear 4 1 0.1 8 : 0.9152615278662795, 0.9691228879070022
linear 4 1 0.1 9 : 0.9152615278662795, 0.9691228879070022
linear 4 1 0.3 3 : 0.9162491081824273, 0.9686238467681585
linear 4 1 0.3 4 : 0.9162491081824273, 0.9686238467681585
linear 4 1 0.3 5 : 0.9162491081824273, 0.9686238467681585
linear 4 1 0.3 6 : 0.9162491081824273, 0.9686238467681585
linear 4 1 0.3 7 : 0.9162491081824273, 0.9686238467681585
linear 4 1 0.3 8 : 0.9162491081824273, 0.9686238467681585
linear 4 1 0.3 9 : 0.9162491081824273, 0.9686238467681585
linear 4 1 0.5 3 : 0.9157407333698314, 0.9679663499048227
linear 4 1 0.5 4 : 0.9157407333698314, 0.9679663499048227
linear 4 1 0.5 5 : 0.9157407333698314, 0.9679663499048227
linear 4 1 0.5 6 : 0.9157407333698314, 0.9679663499048227
linear 4 1 0.5 7 : 0.9157407333698314, 0.9679663499048227
linear 4 1 0.5 8 : 0.9157407333698314, 0.9679663499048227
linear 4 1 0.5 9 : 0.9157407333698314, 0.9679663499048227
linear 4 1 0.7 3 : 0.9143388524212265, 0.9676115359646995
linear 4 1 0.7 4 : 0.9143388524212265, 0.9676115359646995
linear 4 1 0.7 5 : 0.9143388524212265, 0.9676115359646995
linear 4 1 0.7 6 : 0.9143388524212265, 0.9676115359646995
linear 4 1 0.7 7 : 0.9143388524212265, 0.9676115359646995
linear 4 1 0.7 8 : 0.9143388524212265, 0.9676115359646995
linear 4 1 0.7 9 : 0.9143388524212265, 0.9676115359646995
linear 4 1 0.9 3 : 0.9128701866130754, 0.9662503509461882
linear 4 1 0.9 4 : 0.9128701866130754, 0.9662503509461882
linear 4 1 0.9 5 : 0.9128701866130754, 0.9662503509461882
linear 4 1 0.9 6 : 0.9128701866130754, 0.9662503509461882
linear 4 1 0.9 7 : 0.9128701866130754, 0.9662503509461882
linear 4 1 0.9 8 : 0.9128701866130754, 0.9662503509461882
linear 4 1 0.9 9 : 0.9128701866130754, 0.9662503509461882
linear 4 2 0.1 3 : 0.9152615278662795, 0.9691228879070022
linear 4 2 0.1 4 : 0.9152615278662795, 0.9691228879070022
linear 4 2 0.1 5 : 0.9152615278662795, 0.9691228879070022
linear 4 2 0.1 6 : 0.9152615278662795, 0.9691228879070022
linear 4 2 0.1 7 : 0.9152615278662795, 0.9691228879070022
linear 4 2 0.1 8 : 0.9152615278662795, 0.9691228879070022
linear 4 2 0.1 9 : 0.9152615278662795, 0.9691228879070022
linear 4 2 0.3 3 : 0.9162491081824273, 0.9686238467681585
linear 4 2 0.3 4 : 0.9162491081824273, 0.9686238467681585
linear 4 2 0.3 5 : 0.9162491081824273, 0.9686238467681585
linear 4 2 0.3 6 : 0.9162491081824273, 0.9686238467681585
linear 4 2 0.3 7 : 0.9162491081824273, 0.9686238467681585
linear 4 2 0.3 8 : 0.9162491081824273, 0.9686238467681585
linear 4 2 0.3 9 : 0.9162491081824273, 0.9686238467681585
linear 4 2 0.5 3 : 0.9157407333698314, 0.9679663499048227
linear 4 2 0.5 4 : 0.9157407333698314, 0.9679663499048227
linear 4 2 0.5 5 : 0.9157407333698314, 0.9679663499048227
linear 4 2 0.5 6 : 0.9157407333698314, 0.9679663499048227
linear 4 2 0.5 7 : 0.9157407333698314, 0.9679663499048227
linear 4 2 0.5 8 : 0.9157407333698314, 0.9679663499048227
linear 4 2 0.5 9 : 0.9157407333698314, 0.9679663499048227
linear 4 2 0.7 3 : 0.9143388524212265, 0.9676115359646995
linear 4 2 0.7 4 : 0.9143388524212265, 0.9676115359646995
linear 4 2 0.7 5 : 0.9143388524212265, 0.9676115359646995
linear 4 2 0.7 6 : 0.9143388524212265, 0.9676115359646995
linear 4 2 0.7 7 : 0.9143388524212265, 0.9676115359646995
linear 4 2 0.7 8 : 0.9143388524212265, 0.9676115359646995
linear 4 2 0.7 9 : 0.9143388524212265, 0.9676115359646995
linear 4 2 0.9 3 : 0.9128701866130754, 0.9662503509461882
linear 4 2 0.9 4 : 0.9128701866130754, 0.9662503509461882
linear 4 2 0.9 5 : 0.9128701866130754, 0.9662503509461882
linear 4 2 0.9 6 : 0.9128701866130754, 0.9662503509461882
linear 4 2 0.9 7 : 0.9128701866130754, 0.9662503509461882
linear 4 2 0.9 8 : 0.9128701866130754, 0.9662503509461882
linear 4 2 0.9 9 : 0.9128701866130754, 0.9662503509461882
linear 4 3 0.1 3 : 0.9152615278662795, 0.9691228879070022
linear 4 3 0.1 4 : 0.9152615278662795, 0.9691228879070022
linear 4 3 0.1 5 : 0.9152615278662795, 0.9691228879070022
linear 4 3 0.1 6 : 0.9152615278662795, 0.9691228879070022
linear 4 3 0.1 7 : 0.9152615278662795, 0.9691228879070022
linear 4 3 0.1 8 : 0.9152615278662795, 0.9691228879070022
linear 4 3 0.1 9 : 0.9152615278662795, 0.9691228879070022
linear 4 3 0.3 3 : 0.9162491081824273, 0.9686238467681585
linear 4 3 0.3 4 : 0.9162491081824273, 0.9686238467681585
linear 4 3 0.3 5 : 0.9162491081824273, 0.9686238467681585
linear 4 3 0.3 6 : 0.9162491081824273, 0.9686238467681585
linear 4 3 0.3 7 : 0.9162491081824273, 0.9686238467681585
linear 4 3 0.3 8 : 0.9162491081824273, 0.9686238467681585
linear 4 3 0.3 9 : 0.9162491081824273, 0.9686238467681585
linear 4 3 0.5 3 : 0.9157407333698314, 0.9679663499048227
linear 4 3 0.5 4 : 0.9157407333698314, 0.9679663499048227
linear 4 3 0.5 5 : 0.9157407333698314, 0.9679663499048227
linear 4 3 0.5 6 : 0.9157407333698314, 0.9679663499048227
linear 4 3 0.5 7 : 0.9157407333698314, 0.9679663499048227

linear 4 3 0.5 8 : 0.9157407333698314, 0.9679663499048227
linear 4 3 0.5 9 : 0.9157407333698314, 0.9679663499048227
linear 4 3 0.7 3 : 0.9143388524212265, 0.9676115359646995
linear 4 3 0.7 4 : 0.9143388524212265, 0.9676115359646995
linear 4 3 0.7 5 : 0.9143388524212265, 0.9676115359646995
linear 4 3 0.7 6 : 0.9143388524212265, 0.9676115359646995
linear 4 3 0.7 7 : 0.9143388524212265, 0.9676115359646995
linear 4 3 0.7 8 : 0.9143388524212265, 0.9676115359646995
linear 4 3 0.7 9 : 0.9143388524212265, 0.9676115359646995
linear 4 3 0.9 3 : 0.9128701866130754, 0.9662503509461882
linear 4 3 0.9 4 : 0.9128701866130754, 0.9662503509461882
linear 4 3 0.9 5 : 0.9128701866130754, 0.9662503509461882
linear 4 3 0.9 6 : 0.9128701866130754, 0.9662503509461882
linear 4 3 0.9 7 : 0.9128701866130754, 0.9662503509461882
linear 4 3 0.9 8 : 0.9128701866130754, 0.9662503509461882
linear 4 3 0.9 9 : 0.9128701866130754, 0.9662503509461882
linear 4 4 0.1 3 : 0.9152615278662795, 0.9691228879070022
linear 4 4 0.1 4 : 0.9152615278662795, 0.9691228879070022
linear 4 4 0.1 5 : 0.9152615278662795, 0.9691228879070022
linear 4 4 0.1 6 : 0.9152615278662795, 0.9691228879070022
linear 4 4 0.1 7 : 0.9152615278662795, 0.9691228879070022
linear 4 4 0.1 8 : 0.9152615278662795, 0.9691228879070022
linear 4 4 0.1 9 : 0.9152615278662795, 0.9691228879070022
linear 4 4 0.3 3 : 0.9162491081824273, 0.9686238467681585
linear 4 4 0.3 4 : 0.9162491081824273, 0.9686238467681585
linear 4 4 0.3 5 : 0.9162491081824273, 0.9686238467681585
linear 4 4 0.3 6 : 0.9162491081824273, 0.9686238467681585
linear 4 4 0.3 7 : 0.9162491081824273, 0.9686238467681585
linear 4 4 0.3 8 : 0.9162491081824273, 0.9686238467681585
linear 4 4 0.3 9 : 0.9162491081824273, 0.9686238467681585
linear 4 4 0.5 3 : 0.9157407333698314, 0.9679663499048227
linear 4 4 0.5 4 : 0.9157407333698314, 0.9679663499048227
linear 4 4 0.5 5 : 0.9157407333698314, 0.9679663499048227
linear 4 4 0.5 6 : 0.9157407333698314, 0.9679663499048227
linear 4 4 0.5 7 : 0.9157407333698314, 0.9679663499048227
linear 4 4 0.5 8 : 0.9157407333698314, 0.9679663499048227
linear 4 4 0.5 9 : 0.9157407333698314, 0.9679663499048227
linear 4 4 0.7 3 : 0.9143388524212265, 0.9676115359646995
linear 4 4 0.7 4 : 0.9143388524212265, 0.9676115359646995
linear 4 4 0.7 5 : 0.9143388524212265, 0.9676115359646995
linear 4 4 0.7 6 : 0.9143388524212265, 0.9676115359646995
linear 4 4 0.7 7 : 0.9143388524212265, 0.9676115359646995
linear 4 4 0.7 8 : 0.9143388524212265, 0.9676115359646995
linear 4 4 0.7 9 : 0.9143388524212265, 0.9676115359646995
linear 4 4 0.9 3 : 0.9128701866130754, 0.9662503509461882
linear 4 4 0.9 4 : 0.9128701866130754, 0.9662503509461882
linear 4 4 0.9 5 : 0.9128701866130754, 0.9662503509461882
linear 4 4 0.9 6 : 0.9128701866130754, 0.9662503509461882
linear 4 4 0.9 7 : 0.9128701866130754, 0.9662503509461882
linear 4 4 0.9 8 : 0.9128701866130754, 0.9662503509461882
linear 4 4 0.9 9 : 0.9128701866130754, 0.9662503509461882
linear 4 5 0.1 3 : 0.9152615278662795, 0.9691228879070022
linear 4 5 0.1 4 : 0.9152615278662795, 0.9691228879070022
linear 4 5 0.1 5 : 0.9152615278662795, 0.9691228879070022
linear 4 5 0.1 6 : 0.9152615278662795, 0.9691228879070022
linear 4 5 0.1 7 : 0.9152615278662795, 0.9691228879070022
linear 4 5 0.1 8 : 0.9152615278662795, 0.9691228879070022
linear 4 5 0.1 9 : 0.9152615278662795, 0.9691228879070022
linear 4 5 0.3 3 : 0.9162491081824273, 0.9686238467681585
linear 4 5 0.3 4 : 0.9162491081824273, 0.9686238467681585
linear 4 5 0.3 5 : 0.9162491081824273, 0.9686238467681585
linear 4 5 0.3 6 : 0.9162491081824273, 0.9686238467681585
linear 4 5 0.3 7 : 0.9162491081824273, 0.9686238467681585
linear 4 5 0.3 8 : 0.9162491081824273, 0.9686238467681585
linear 4 5 0.3 9 : 0.9162491081824273, 0.9686238467681585
linear 4 5 0.5 3 : 0.9157407333698314, 0.9679663499048227
linear 4 5 0.5 4 : 0.9157407333698314, 0.9679663499048227
linear 4 5 0.5 5 : 0.9157407333698314, 0.9679663499048227
linear 4 5 0.5 6 : 0.9157407333698314, 0.9679663499048227
linear 4 5 0.5 7 : 0.9157407333698314, 0.9679663499048227
linear 4 5 0.5 8 : 0.9157407333698314, 0.9679663499048227
linear 4 5 0.5 9 : 0.9157407333698314, 0.9679663499048227
linear 4 5 0.7 3 : 0.9143388524212265, 0.9676115359646995
linear 4 5 0.7 4 : 0.9143388524212265, 0.9676115359646995
linear 4 5 0.7 5 : 0.9143388524212265, 0.9676115359646995
linear 4 5 0.7 6 : 0.9143388524212265, 0.9676115359646995
linear 4 5 0.7 7 : 0.9143388524212265, 0.9676115359646995
linear 4 5 0.7 8 : 0.9143388524212265, 0.9676115359646995
linear 4 5 0.7 9 : 0.9143388524212265, 0.9676115359646995
linear 4 5 0.9 3 : 0.9128701866130754, 0.9662503509461882
linear 4 5 0.9 4 : 0.9128701866130754, 0.9662503509461882
linear 4 5 0.9 5 : 0.9128701866130754, 0.9662503509461882
linear 4 5 0.9 6 : 0.9128701866130754, 0.9662503509461882
linear 4 5 0.9 7 : 0.9128701866130754, 0.9662503509461882
linear 4 5 0.9 8 : 0.9128701866130754, 0.9662503509461882
linear 4 5 0.9 9 : 0.9128701866130754, 0.9662503509461882
linear 4 6 0.1 3 : 0.9152615278662795, 0.9691228879070022
linear 4 6 0.1 4 : 0.9152615278662795, 0.9691228879070022
linear 4 6 0.1 5 : 0.9152615278662795, 0.9691228879070022
linear 4 6 0.1 6 : 0.9152615278662795, 0.9691228879070022
linear 4 6 0.1 7 : 0.9152615278662795, 0.9691228879070022
linear 4 6 0.1 8 : 0.9152615278662795, 0.9691228879070022
linear 4 6 0.1 9 : 0.9152615278662795, 0.9691228879070022
linear 4 6 0.3 3 : 0.9162491081824273, 0.9686238467681585
linear 4 6 0.3 4 : 0.9162491081824273, 0.9686238467681585
linear 4 6 0.3 5 : 0.9162491081824273, 0.9686238467681585
linear 4 6 0.3 6 : 0.9162491081824273, 0.9686238467681585
linear 4 6 0.3 7 : 0.9162491081824273, 0.9686238467681585
linear 4 6 0.3 8 : 0.9162491081824273, 0.9686238467681585
linear 4 6 0.3 9 : 0.9162491081824273, 0.9686238467681585
linear 4 6 0.5 3 : 0.9157407333698314, 0.9679663499048227
linear 4 6 0.5 4 : 0.9157407333698314, 0.9679663499048227
linear 4 6 0.5 5 : 0.9157407333698314, 0.9679663499048227
linear 4 6 0.5 6 : 0.9157407333698314, 0.9679663499048227
linear 4 6 0.5 7 : 0.9157407333698314, 0.9679663499048227
linear 4 6 0.5 8 : 0.9157407333698314, 0.9679663499048227
linear 4 6 0.5 9 : 0.9157407333698314, 0.9679663499048227
linear 4 6 0.7 3 : 0.9143388524212265, 0.9676115359646995
linear 4 6 0.7 4 : 0.9143388524212265, 0.9676115359646995
linear 4 6 0.7 5 : 0.9143388524212265, 0.9676115359646995
linear 4 6 0.7 6 : 0.9143388524212265, 0.9676115359646995
linear 4 6 0.7 7 : 0.9143388524212265, 0.9676115359646995
linear 4 6 0.7 8 : 0.9143388524212265, 0.9676115359646995
linear 4 6 0.7 9 : 0.9143388524212265, 0.9676115359646995
linear 4 6 0.9 3 : 0.9128701866130754, 0.9662503509461882
linear 4 6 0.9 4 : 0.9128701866130754, 0.9662503509461882
linear 4 6 0.9 5 : 0.9128701866130754, 0.9662503509461882
linear 4 6 0.9 6 : 0.9128701866130754, 0.9662503509461882
linear 4 6 0.9 7 : 0.9128701866130754, 0.9662503509461882
linear 4 6 0.9 8 : 0.9128701866130754, 0.9662503509461882
linear 4 6 0.9 9 : 0.9128701866130754, 0.9662503509461882
poly 1 1 0.1 : 0.8228328264834444, 0.883665363143038
poly 1 1 0.3 : 0.8203096429228672, 0.8807473208763442
poly 1 1 0.5 : 0.8176193803045985, 0.8776112815561298
poly 1 1 0.7 : 0.8147722131651104, 0.8743338721675376
poly 1 1 0.9 : 0.8117803295372916, 0.8709897537925801
poly 1 2 0.1 : 0.8210610209395288, 0.8819117390574821
poly 1 2 0.3 : 0.8185210606792716, 0.8789893792278282
poly 1 2 0.5 : 0.8158261116196492, 0.8758557289813872
poly 1 2 0.7 : 0.8129718392227371, 0.8725783773368763
poly 1 2 0.9 : 0.8099811778498307, 0.8692234654989871
poly 1 3 0.1 : 0.8204553914680679, 0.8813123933106576
poly 1 3 0.3 : 0.8179115679636899, 0.8783883279879495
poly 1 3 0.5 : 0.8152146298059085, 0.8752564826214033
poly 1 3 0.7 : 0.8123584019932484, 0.8719764402115965
poly 1 3 0.9 : 0.8093680922014833, 0.8686190917092562
poly 1 4 0.1 : 0.8201502708649947, 0.8810115361919942
poly 1 4 0.3 : 0.8176037531838991, 0.8780840990508693
poly 1 4 0.5 : 0.8149068094926417, 0.8749533099465882
poly 1 4 0.7 : 0.812048640548346, 0.8716721872309463
poly 1 4 0.9 : 0.809058900509567, 0.868315285347399
poly 1 5 0.1 : 0.819966291961423, 0.8808298111939923
poly 1 5 0.3 : 0.8174185760810836, 0.8779002244623597
poly 1 5 0.5 : 0.81472159218931, 0.8747715045802233
poly 1 5 0.7 : 0.8118626132926863, 0.8714905350509933
poly 1 5 0.9 : 0.8088723222837089, 0.8681309680471796
poly 1 6 0.1 : 0.8198438975859779, 0.88070817417898
poly 1 6 0.3 : 0.8172949373305602, 0.8777778061865384

poly 1 6 0.5 : 0.8145973595979165, 0.8746488945420385
poly 1 6 0.7 : 0.8117375984065269, 0.871366557164906
poly 1 6 0.9 : 0.8087475371885745, 0.8680086287219697
poly 2 1 0.1 : 0.8228328264834459, 0.8836653631429727
poly 2 1 0.3 : 0.8203096429227964, 0.8807473208763792
poly 2 1 0.5 : 0.8176193803045916, 0.8776112815561459
poly 2 1 0.7 : 0.8147722131650449, 0.8743338721675835
poly 2 1 0.9 : 0.8117803295372916, 0.8709897537925801
poly 2 2 0.1 : 0.8210610209396295, 0.8819117390562358
poly 2 2 0.3 : 0.8185210606794548, 0.8789893792276591
poly 2 2 0.5 : 0.8158261116199469, 0.8758557289813013
poly 2 2 0.7 : 0.8129718392224039, 0.872578377336495
poly 2 2 0.9 : 0.8099811778498307, 0.8692234654989871
poly 2 3 0.1 : 0.8204553914672976, 0.881312393315874
poly 2 3 0.3 : 0.8179115679644997, 0.8783883279859489
poly 2 3 0.5 : 0.8152146298100537, 0.8752564826209961
poly 2 3 0.7 : 0.8123584019940286, 0.8719764402094178
poly 2 3 0.9 : 0.8093680922014833, 0.8686190917092562
poly 2 4 0.1 : 0.8201502708686051, 0.8810115361894626
poly 2 4 0.3 : 0.8176037531812812, 0.8780840990480043
poly 2 4 0.5 : 0.8149068094937355, 0.8749533099400574
poly 2 4 0.7 : 0.8120486405408986, 0.8716721872356389
poly 2 4 0.9 : 0.809058900509567, 0.868315285347399
poly 2 5 0.1 : 0.819966291965424, 0.8808298111891314
poly 2 5 0.3 : 0.8174185760801365, 0.8779002244652531
poly 2 5 0.5 : 0.8147215921933169, 0.8747715045919776
poly 2 5 0.7 : 0.8118626132943735, 0.8714905350433244
poly 2 5 0.9 : 0.8088723222837089, 0.8681309680471796
poly 2 6 0.1 : 0.819843897592906, 0.8807081741747549
poly 2 6 0.3 : 0.8172949373057061, 0.8777778062057323
poly 2 6 0.5 : 0.8145973596034486, 0.8746488945176363
poly 2 6 0.7 : 0.8117375984143147, 0.8713665571509828
poly 2 6 0.9 : 0.8087475371885745, 0.8680086287219697
poly 3 1 0.1 : 0.8228328264833806, 0.8836653631429586
poly 3 1 0.3 : 0.8203096429227713, 0.8807473208763457
poly 3 1 0.5 : 0.8176193803046152, 0.8776112815560297
poly 3 1 0.7 : 0.8147722131650935, 0.8743338721675846
poly 3 1 0.9 : 0.8117803295372916, 0.8709897537925801
poly 3 2 0.1 : 0.8210610209397713, 0.8819117390558107
poly 3 2 0.3 : 0.8185210606800007, 0.8789893792285
poly 3 2 0.5 : 0.8158261116190786, 0.8758557289810015
poly 3 2 0.7 : 0.8129718392225831, 0.8725783773373532
poly 3 2 0.9 : 0.8099811778498307, 0.8692234654989871
poly 3 3 0.1 : 0.8204553914675163, 0.8813123933152338
poly 3 3 0.3 : 0.8179115679635934, 0.8783883279897978
poly 3 3 0.5 : 0.815214629806692, 0.875256482618143
poly 3 3 0.7 : 0.8123584019924748, 0.8719764402108049
poly 3 3 0.9 : 0.8093680922014833, 0.8686190917092562
poly 3 4 0.1 : 0.8201502708625336, 0.8810115361838284
poly 3 4 0.3 : 0.8176037531872474, 0.8780840990406028
poly 3 4 0.5 : 0.8149068094938341, 0.8749533099317566
poly 3 4 0.7 : 0.8120486405446252, 0.8716721872360643
poly 3 4 0.9 : 0.809058900509567, 0.868315285347399
poly 3 5 0.1 : 0.8199662919543375, 0.8808298112067516
poly 3 5 0.3 : 0.8174185760881445, 0.877900224494629
poly 3 5 0.5 : 0.8147215921986104, 0.8747715045865787
poly 3 5 0.7 : 0.8118626132874326, 0.8714905350524172
poly 3 5 0.9 : 0.8088723222837089, 0.8681309680471796
poly 3 6 0.1 : 0.8198438975768065, 0.8807081741629245
poly 3 6 0.3 : 0.8172949373091709, 0.877777806234146
poly 3 6 0.5 : 0.8145973595830476, 0.8746488945582316
poly 3 6 0.7 : 0.8117375984104294, 0.8713665571513336
poly 3 6 0.9 : 0.8087475371885745, 0.8680086287219697
poly 4 1 0.1 : 0.8228328264834299, 0.8836653631429936
poly 4 1 0.3 : 0.8203096429228216, 0.880747320876343
poly 4 1 0.5 : 0.8176193803046751, 0.8776112815559894
poly 4 1 0.7 : 0.8147722131651746, 0.8743338721675737
poly 4 1 0.9 : 0.8117803295372916, 0.8709897537925801
poly 4 2 0.1 : 0.821061020940103, 0.8819117390570863
poly 4 2 0.3 : 0.818521060679686, 0.8789893792298937
poly 4 2 0.5 : 0.8158261116188722, 0.8758557289807145
poly 4 2 0.7 : 0.8129718392232324, 0.8725783773358378
poly 4 2 0.9 : 0.8099811778498307, 0.8692234654989871
poly 4 3 0.1 : 0.8204553914626148, 0.8813123933119942
poly 4 3 0.3 : 0.8179115679611824, 0.8783883279902903
poly 4 3 0.5 : 0.8152146298061677, 0.8752564826171296
poly 4 3 0.7 : 0.8123584019944318, 0.8719764402149125
poly 4 3 0.9 : 0.8093680922014833, 0.8686190917092562
poly 4 4 0.1 : 0.8201502708688067, 0.8810115361758402
poly 4 4 0.3 : 0.8176037531819252, 0.8780840990494773
poly 4 4 0.5 : 0.8149068094961779, 0.8749533099269977
poly 4 4 0.7 : 0.8120486405523606, 0.871672187234154
poly 4 4 0.9 : 0.809058900509567, 0.868315285347399
poly 4 5 0.1 : 0.81996629194855, 0.8808298112164873
poly 4 5 0.3 : 0.8174185760760755, 0.8779002244815872
poly 4 5 0.5 : 0.8147215921987506, 0.8747715045889682
poly 4 5 0.7 : 0.8118626132870075, 0.8714905350445667
poly 4 5 0.9 : 0.8088723222837089, 0.8681309680471796
poly 4 6 0.1 : 0.8198438975682102, 0.8807081741744814
poly 4 6 0.3 : 0.8172949373233207, 0.8777778062175304
poly 4 6 0.5 : 0.8145973595494654, 0.8746488945785277
poly 4 6 0.7 : 0.811737598414695, 0.8713665571191428
poly 4 6 0.9 : 0.8087475371885745, 0.8680086287219697
rbf 1 1 0.1 : -0.027360631223619335, 0.0015662440254313736
rbf 1 1 0.3 : -0.02710653274315935, 0.0015348740018256146
rbf 1 1 0.5 : -0.026501827448424952, 0.0014647403897590783
rbf 1 1 0.7 : -0.025707558352374437, 0.0014176825404806914
rbf 1 1 0.9 : -0.02444971498901536, 0.0013867640665964354
rbf 1 2 0.1 : -0.02737345560029252, 0.0015652602858303721
rbf 1 2 0.3 : -0.027119349701869133, 0.0015338898653123323
rbf 1 2 0.5 : -0.026514594389397673, 0.0014637547353891778
rbf 1 2 0.7 : -0.025720260371089633, 0.00141669617407858
rbf 1 2 0.9 : -0.0244623385265899, 0.0013857771650894746
rbf 1 3 0.1 : -0.027373670056562105, 0.0015652593898375367
rbf 1 3 0.3 : -0.027119564033778288, 0.0015338889687104285
rbf 1 3 0.5 : -0.02651480779109261, 0.0014637538375501524
rbf 1 3 0.7 : -0.025720472580383368, 0.00141669527548438
rbf 1 3 0.9 : -0.024462549300359227, 0.001385776266025096
rbf 1 4 0.1 : -0.027373673960909218, 0.0015652593890200794
rbf 1 4 0.3 : -0.02711956793588577, 0.0015338889678926382
rbf 1 4 0.5 : -0.0265148116761559, 0.0014637538367308078
rbf 1 4 0.7 : -0.025720476443612396, 0.0014166952746643702
rbf 1 4 0.9 : -0.024462553137245057, 0.0013857762652048633
rbf 1 5 0.1 : -0.027373674032374184, 0.0015652593890191913
rbf 1 5 0.3 : -0.027119568007309924, 0.00153388896789175
rbf 1 5 0.5 : -0.026514811747267684, 0.0014637538367301417
rbf 1 5 0.7 : -0.025720476514324363, 0.001416695274663704
rbf 1 5 0.9 : -0.02446255320747439, 0.001385776265204086
rbf 1 6 0.1 : -0.027373674033683005, 0.0015652593890191913
rbf 1 6 0.3 : -0.027119568008617943, 0.00153388896789175
rbf 1 6 0.5 : -0.026514811748570066, 0.0014637538367301417
rbf 1 6 0.7 : -0.025720476515619418, 0.001416695274663704
rbf 1 6 0.9 : -0.024462553208760562, 0.0013857762652040861
rbf 2 1 0.1 : -0.02470191761168663, 0.003058875643896175
rbf 2 1 0.3 : -0.024014419883098537, 0.002983523514223596
rbf 2 1 0.5 : -0.023334745528094558, 0.002883780492882737
rbf 2 1 0.7 : -0.022527577524228494, 0.0027649756909003953
rbf 2 1 0.9 : -0.021781957029003384, 0.002639286078094827
rbf 2 2 0.1 : -0.024727259414636205, 0.003056902681489615
rbf 2 2 0.3 : -0.024039693382083272, 0.002981549019693608
rbf 2 2 0.5 : -0.023359942225393792, 0.002881803788273185
rbf 2 2 0.7 : -0.02255275393080778, 0.002762996479499069
rbf 2 2 0.9 : -0.021807085090491007, 0.0026373036626700097
rbf 2 3 0.1 : -0.02472768314079645, 0.003056900883510738
rbf 2 3 0.3 : -0.024040115882170587, 0.002981547219634284
rbf 2 3 0.5 : -0.02336036390453433, 0.002881801985684218
rbf 2 3 0.7 : -0.022553174637438066, 0.002762994674155972
rbf 2 3 0.9 : -0.021807466054202827, 0.0026373018546451688
rbf 2 4 0.1 : -0.024727690854553508, 0.0030569008818703836
rbf 2 4 0.3 : -0.02404012357347827, 0.00298154721799182
rbf 2 4 0.5 : -0.023360371580797158, 0.0028818019840395337
rbf 2 4 0.7 : -0.0225531822957989, 0.002762994672508623
rbf 2 4 0.9 : -0.02180747369643434, 0.002637301852995488
rbf 2 5 0.1 : -0.02472769099574439, 0.003056900881868718
rbf 2 5 0.3 : -0.024040123714257965, 0.002981547217990266
rbf 2 5 0.5 : -0.023360371721301298, 0.0028818019840379794
rbf 2 5 0.7 : -0.022553182435974862, 0.0027629946725071797
rbf 2 5 0.9 : -0.021807473836314494, 0.002637301852993823

rbf 2 6 0.1 : -0.024727690998330186, 0.0030569008818687182
rbf 2 6 0.3 : -0.02404012371683617, 0.002981547217990266
rbf 2 6 0.5 : -0.023360371723874396, 0.0028818019840379794
rbf 2 6 0.7 : -0.022553182438542096, 0.0027629946725071797
rbf 2 6 0.9 : -0.021807473838876268, 0.002637301852993823
rbf 3 1 0.1 : -0.021861623481479065, 0.004174432005777162
rbf 3 1 0.3 : -0.02110600434649217, 0.004067354048377103
rbf 3 1 0.5 : -0.02020763754191406, 0.003918998273017782
rbf 3 1 0.7 : -0.01915743725008747, 0.003667729746627657
rbf 3 1 0.9 : -0.018071376691967032, 0.003349991109892314
rbf 3 2 0.1 : -0.021899470850652403, 0.004171455940156665
rbf 3 2 0.3 : -0.02114375625178515, 0.004064374314709296
rbf 3 2 0.5 : -0.020245300878799232, 0.003916014436663229
rbf 3 2 0.7 : -0.019195023990497485, 0.0036647391392713446
rbf 3 2 0.9 : -0.018108927742550306, 0.003346992515235736
rbf 3 3 0.1 : -0.021900090766342428, 0.004171453227239441
rbf 3 3 0.3 : -0.021144358554814426, 0.004064371598713978
rbf 3 3 0.5 : -0.02024589825692278, 0.003916011716660339
rbf 3 3 0.7 : -0.019195687930650563, 0.003664736413015568
rbf 3 3 0.9 : -0.018109555029501935, 0.003346989781815357
rbf 3 4 0.1 : -0.021900102286829214, 0.004171453224764088
rbf 3 4 0.3 : -0.021144370049059315, 0.004064371596236072
rbf 3 4 0.5 : -0.02024590971998399, 0.00391601171417888
rbf 3 4 0.7 : -0.019195699366842244, 0.0036647364105283353
rbf 3 4 0.9 : -0.018109566446450474, 0.003346989779321463
rbf 3 5 0.1 : -0.021900102497695383, 0.004171453224761756
rbf 3 5 0.3 : -0.02114437025944409, 0.00406437159623374
rbf 3 5 0.5 : -0.02024590992979678, 0.003916011714176548
rbf 3 5 0.7 : -0.019195699576161917, 0.003664736410526115
rbf 3 5 0.9 : -0.018109566655416475, 0.0033469897793192427
rbf 3 6 0.1 : -0.021900102501557227, 0.004171453224761756
rbf 3 6 0.3 : -0.02114437026329701, 0.00406437159623374
rbf 3 6 0.5 : -0.020245909933639304, 0.003916011714176548
rbf 3 6 0.7 : -0.019195699579995472, 0.003664736410526115
rbf 3 6 0.9 : -0.01810956665924346, 0.0033469897793192427
rbf 4 1 0.1 : -0.01796372922414795, 0.004593111704033315
rbf 4 1 0.3 : -0.016917670319564593, 0.004161555730900823
rbf 4 1 0.5 : -0.015884806562994847, 0.003703039121627616
rbf 4 1 0.7 : -0.014936145719956962, 0.0032226359253160197
rbf 4 1 0.9 : -0.014002978236026342, 0.0026179367877500193
rbf 4 2 0.1 : -0.018013997306204965, 0.004589103811644235
rbf 4 2 0.3 : -0.01696790798811798, 0.0041575348559158565
rbf 4 2 0.5 : -0.015935086534401854, 0.0036990071656313184
rbf 4 2 0.7 : -0.014986370388657466, 0.0032186660090515185
rbf 4 2 0.9 : -0.014053144938224382, 0.0026139612397154677
rbf 4 3 0.1 : -0.018014837276205897, 0.004589100161245807
rbf 4 3 0.3 : -0.016968763801893737, 0.004157531194582176
rbf 4 3 0.5 : -0.015935911362900245, 0.0036990034936942306
rbf 4 3 0.7 : -0.014987222838264235, 0.0032186623518379864
rbf 4 3 0.9 : -0.014054021809108197, 0.0026139575737710308
rbf 4 4 0.1 : -0.01801485256402611, 0.004589100157915471
rbf 4 4 0.3 : -0.016968779073742456, 0.004157531191241848
rbf 4 4 0.5 : -0.01593592662082961, 0.0036990034903440216
rbf 4 4 0.7 : -0.014987202457065108, 0.0032186623484868893
rbf 4 4 0.9 : -0.014054037058116675, 0.0026139575704106077
rbf 4 5 0.1 : -0.018014852843841033, 0.0045891001579123625
rbf 4 5 0.3 : -0.01696877935326313, 0.00415753119123885
rbf 4 5 0.5 : -0.015935926900093246, 0.003699003490341024
rbf 4 5 0.7 : -0.014987202736149952, 0.0032186623484837806
rbf 4 5 0.9 : -0.01405403733721291, 0.00261395757040761
rbf 4 6 0.1 : -0.018014852848965646, 0.0045891001579123625
rbf 4 6 0.3 : -0.016968779358382237, 0.00415753119123885
rbf 4 6 0.5 : -0.015935926905207686, 0.003699003490341024
rbf 4 6 0.7 : -0.014987202741261151, 0.0032186623484837806
rbf 4 6 0.9 : -0.014054037342324332, 0.00261395757040761
sigmoid 1 1 0.1 : -0.027851622231682516, -0.0004817540112838081
sigmoid 1 1 0.3 : -0.028038560906177113, -
0.00015274162024248028
sigmoid 1 1 0.5 : -0.02932756328703263, -8.395628023483148e-05
sigmoid 1 1 0.7 : -0.027370762665524496, -
0.00015275252801516537
sigmoid 1 1 0.9 : -0.027425853345986927, -
0.00035162489155515786
sigmoid 1 2 0.1 : -0.02785157494444097, -0.0004817589184518134
sigmoid 1 2 0.3 : -0.02803863742477839, -0.00015273486547884296
sigmoid 1 2 0.5 : -0.029327682929540178, -8.393840810549946e-05
sigmoid 1 2 0.7 : -0.02737078105549471, -0.00015273486546996118
sigmoid 1 2 0.9 : -0.027425939282063806, -0.0003516451206677651
sigmoid 1 3 0.1 : -0.02785157494475108, -0.0004817589182650739
sigmoid 1 3 0.3 : -0.028038637425086766, -0.0001527348652718974
sigmoid 1 3 0.5 : -0.029327682929847977, -8.39384078961114e-05
sigmoid 1 3 0.7 : -0.027370781055785543, -0.0001527348652718974
sigmoid 1 3 0.9 : -0.02742593928234638, -0.0003516451204561566
sigmoid 1 4 0.1 : -0.02785157494475108, -0.0004817589182650739
sigmoid 1 4 0.3 : -0.028038637425086766, -0.0001527348652718974
sigmoid 1 4 0.5 : -0.029327682929847977, -8.39384078961114e-05
sigmoid 1 4 0.7 : -0.027370781055785543, -0.0001527348652718974
sigmoid 1 4 0.9 : -0.02742593928234638, -0.0003516451204561566
sigmoid 1 5 0.1 : -0.02785157494475108, -0.0004817589182650739
sigmoid 1 5 0.3 : -0.028038637425086766, -0.0001527348652718974
sigmoid 1 5 0.5 : -0.029327682929847977, -8.39384078961114e-05
sigmoid 1 5 0.7 : -0.027370781055785543, -0.0001527348652718974
sigmoid 1 5 0.9 : -0.02742593928234638, -0.0003516451204561566
sigmoid 1 6 0.1 : -0.02785157494475108, -0.0004817589182650739
sigmoid 1 6 0.3 : -0.028038637425086766, -0.0001527348652718974
sigmoid 1 6 0.5 : -0.029327682929847977, -8.39384078961114e-05
sigmoid 1 6 0.7 : -0.027370781055785543, -0.0001527348652718974
sigmoid 1 6 0.9 : -0.02742593928234638, -0.0003516451204561566
sigmoid 2 1 0.1 : -0.027851669519565726, -0.0004817491052644396
sigmoid 2 1 0.3 : -0.02803848438784313, -0.00015274837616163772
sigmoid 2 1 0.5 : -0.0293274436447148, -8.397415308203371e-05
sigmoid 2 1 0.7 : -0.027370744276163705, -
0.00015277019126735958
sigmoid 2 1 0.9 : -0.027425767410060732, -0.0003516046653644356
sigmoid 2 2 0.1 : -0.02785157494413082, -0.000481758918638997
sigmoid 2 2 0.3 : -0.028038637424470013, -
0.00015273486568623262
sigmoid 2 2 0.5 : -0.029327682929232157, -8.393840831510957e-05
sigmoid 2 2 0.7 : -0.027370781055203696, -
0.00015273486566780292
sigmoid 2 2 0.9 : -0.0274259392817811, -0.00035164512087915156
sigmoid 2 3 0.1 : -0.02785157494475108, -0.0004817589182650739
sigmoid 2 3 0.3 : -0.028038637425086766, -0.0001527348652718974
sigmoid 2 3 0.5 : -0.029327682929847977, -8.39384078961114e-05
sigmoid 2 3 0.7 : -0.027370781055785543, -0.0001527348652718974
sigmoid 2 3 0.9 : -0.02742593928234638, -0.0003516451204561566
sigmoid 2 4 0.1 : -0.02785157494475108, -0.0004817589182650739
sigmoid 2 4 0.3 : -0.028038637425086766, -0.0001527348652718974
sigmoid 2 4 0.5 : -0.029327682929847977, -8.39384078961114e-05
sigmoid 2 4 0.7 : -0.027370781055785543, -0.0001527348652718974
sigmoid 2 4 0.9 : -0.02742593928234638, -0.0003516451204561566
sigmoid 2 5 0.1 : -0.02785157494475108, -0.0004817589182650739
sigmoid 2 5 0.3 : -0.028038637425086766, -0.0001527348652718974
sigmoid 2 5 0.5 : -0.029327682929847977, -8.39384078961114e-05
sigmoid 2 5 0.7 : -0.027370781055785543, -0.0001527348652718974
sigmoid 2 5 0.9 : -0.02742593928234638, -0.0003516451204561566
sigmoid 2 6 0.1 : -0.02785157494475108, -0.0004817589182650739
sigmoid 2 6 0.3 : -0.028038637425086766, -0.0001527348652718974
sigmoid 2 6 0.5 : -0.029327682929847977, -8.39384078961114e-05
sigmoid 2 6 0.7 : -0.027370781055785543, -0.0001527348652718974
sigmoid 2 6 0.9 : -0.02742593928234638, -0.0003516451204561566
sigmoid 3 1 0.1 : -0.02785171680840075, -0.0004817442002060801
sigmoid 3 1 0.3 : -0.028038407870084825, -0.0001527551330298138
sigmoid 3 1 0.5 : -0.02932732400289364, -8.399202643771808e-05
sigmoid 3 1 0.7 : -0.02737072588770264, -0.00015278785502759185
sigmoid 3 1 0.9 : -0.027425681474567566, -0.0003515844418842118
sigmoid 3 2 0.1 : -0.027851574943820935, -
0.00048175891882595856
sigmoid 3 2 0.3 : -0.028038637424161905, -
0.00015273486589362228
sigmoid 3 2 0.5 : -0.02932768292892458, -8.393840852449763e-05
sigmoid 3 2 0.7 : -0.027370781054913217, -
0.00015273486586608875
sigmoid 3 2 0.9 : -0.027425939281498703, -0.0003516451210909821
sigmoid 3 3 0.1 : -0.02785157494475108, -0.0004817589182650739
sigmoid 3 3 0.3 : -0.028038637425086766, -0.0001527348652718974
sigmoid 3 3 0.5 : -0.029327682929847977, -8.39384078961114e-05
sigmoid 3 3 0.7 : -0.027370781055785543, -0.0001527348652718974

sigmoid 3 3 0.9 : -0.02742593928234638, -0.0003516451204561566
sigmoid 3 4 0.1 : -0.02785157494475108, -0.0004817589182650739
sigmoid 3 4 0.3 : -0.028038637425086766, -0.0001527348652718974
sigmoid 3 4 0.5 : -0.029327682929847977, -8.39384078961114e-05
sigmoid 3 4 0.7 : -0.027370781055785543, -0.0001527348652718974
sigmoid 3 4 0.9 : -0.02742593928234638, -0.0003516451204561566
sigmoid 3 5 0.1 : -0.02785157494475108, -0.0004817589182650739
sigmoid 3 5 0.3 : -0.028038637425086766, -0.0001527348652718974
sigmoid 3 5 0.5 : -0.029327682929847977, -8.39384078961114e-05
sigmoid 3 5 0.7 : -0.027370781055785543, -0.0001527348652718974
sigmoid 3 5 0.9 : -0.02742593928234638, -0.0003516451204561566
sigmoid 3 6 0.1 : -0.02785157494475108, -0.0004817589182650739
sigmoid 3 6 0.3 : -0.028038637425086766, -0.0001527348652718974
sigmoid 3 6 0.5 : -0.029327682929847977, -8.39384078961114e-05
sigmoid 3 6 0.7 : -0.027370781055785543, -0.0001527348652718974
sigmoid 3 6 0.9 : -0.02742593928234638, -0.0003516451204561566
sigmoid 4 1 0.1 : -0.02785176409818768, -0.0004817392961098399
sigmoid 4 1 0.3 : -0.028038331352902412, -
0.00015276189084700853
sigmoid 4 1 0.5 : -0.029327204361570346, -8.400990030232869e-05
sigmoid 4 1 0.7 : -0.02737070750014232, -0.00015280551929675035
sigmoid 4 1 0.9 : -0.027425595539508275, -
0.00035156422111426444
sigmoid 4 2 0.1 : -0.02785157494351074, -0.0004817589190129201
sigmoid 4 2 0.3 : -0.028038637423853353, -0.0001527348661007899
sigmoid 4 2 0.5 : -0.02932768292861656, -8.393840873432978e-05
sigmoid 4 2 0.7 : -0.027370781054622075, -0.0001527348660639305
sigmoid 4 2 0.9 : -0.027425939281215596, -0.0003516451213019245
sigmoid 4 3 0.1 : -0.02785157494475108, -0.0004817589182650739
sigmoid 4 3 0.3 : -0.028038637425086766, -0.0001527348652718974
sigmoid 4 3 0.5 : -0.029327682929847977, -8.39384078961114e-05
sigmoid 4 3 0.7 : -0.027370781055785543, -0.0001527348652718974
sigmoid 4 3 0.9 : -0.02742593928234638, -0.0003516451204561566
sigmoid 4 4 0.1 : -0.02785157494475108, -0.0004817589182650739
sigmoid 4 4 0.3 : -0.028038637425086766, -0.0001527348652718974
sigmoid 4 4 0.5 : -0.029327682929847977, -8.39384078961114e-05
sigmoid 4 4 0.7 : -0.027370781055785543, -0.0001527348652718974
sigmoid 4 4 0.9 : -0.02742593928234638, -0.0003516451204561566
sigmoid 4 5 0.1 : -0.02785157494475108, -0.0004817589182650739
sigmoid 4 5 0.3 : -0.028038637425086766, -0.0001527348652718974
sigmoid 4 5 0.5 : -0.029327682929847977, -8.39384078961114e-05
sigmoid 4 5 0.7 : -0.027370781055785543, -0.0001527348652718974
sigmoid 4 5 0.9 : -0.02742593928234638, -0.0003516451204561566
sigmoid 4 6 0.1 : -0.02785157494475108, -0.0004817589182650739
sigmoid 4 6 0.3 : -0.028038637425086766, -0.0001527348652718974
sigmoid 4 6 0.5 : -0.029327682929847977, -8.39384078961114e-05
sigmoid 4 6 0.7 : -0.027370781055785543, -0.0001527348652718974
sigmoid 4 6 0.9 : -0.02742593928234638, -0.0003516451204561566

Grid search of ElasticNet, Morgan(r=2,2048)
ElasticNet(alpha= i, l1_ratio= j, max_iter=100000)
i j : accuracy of prediction using cross validation, accuracy of prediction using test data
0.001 0.0 : 0.9246769908220823, 0.9723815207831754
0.001 0.2 : 0.9254790774947275, 0.9736735143687847
0.001 0.4 : 0.9258719941072602, 0.9749021988701542
0.001 0.6 : 0.9253033155127255, 0.9759002240649929
0.001 0.8 : 0.9243243844336956, 0.9764615458027965
0.001 1.0 : 0.9079385452825974, 0.9698990846762324
0.01 0.0 : 0.90429612229701, 0.9496233085756626
0.01 0.2 : 0.9057189300845618, 0.951061764132663
0.01 0.4 : 0.9063815313462129, 0.9527361642472769
0.01 0.6 : 0.9073033756256874, 0.9546979663150035
0.01 0.8 : 0.9079349255247149, 0.9561185543592923
0.01 1.0 : 0.8978977611698996, 0.9517405066048256
0.1 0.0 : 0.7965136585165595, 0.8286636230803281
0.1 0.2 : 0.7848509019502078, 0.8122558104604733
0.1 0.4 : 0.7768855285226947, 0.8022188104677056
0.1 0.6 : 0.774363900872598, 0.7985839283655496
0.1 0.8 : 0.7793774409074459, 0.8072767799846609
0.1 1.0 : 0.7963141998797142, 0.8257450079645513

Grid search of RandomForest, Morgan(r=2,2048)
RandomForestRegressor(n_estimators= i)
i: accuracy of prediction using cross validation, accuracy of prediction using test data
100 1.0 : 0.7895607221851826, 0.8731454248915441
500 1.0 : 0.7892525542349736, 0.8614140825202652
1000 1.0 : 0.792305838608602, 0.8647865196449329
2000 1.0 : 0.7914678120630099, 0.8623239929368959

Grid search of NeuralNetwork, Morgan(r=2,2048)
MLPRegressor(activation=act, alpha=a, batch_size=batch, beta_1=0.9,
beta_2=0.999, early_stopping=False, epsilon=1e-08,
hidden_layer_sizes=hid, learning_rate='constant',
learning_rate_init=0.001, max_iter=100000, momentum=0.9,
n_iter_no_change=10, nesterovs_momentum=True,
power_t=0.5,
random_state=1, shuffle=True, solver='adam', tol=0.0001,
validation_fraction=0.1, verbose=False, warm_start=False)
act (hid) a batch: accuracy of prediction using cross validation, accuracy of prediction using test data
relu (200,) 0.0001 500 : 0.8626477538141153, 0.9540072762527638
relu (200,) 0.0001 200 : 0.8858803029700046, 0.9502585182367908
relu (200,) 0.0001 100 : 0.8867572759365743, 0.9523251819606722
relu (200,) 0.0001 50 : 0.892482136029523, 0.9542891724510043
relu (200,) 0.001 500 : 0.8624375567802041, 0.9540763023418394
relu (200,) 0.001 200 : 0.8859616138169022, 0.9508202802157302
relu (200,) 0.001 100 : 0.8869152629323844, 0.9524860804239931
relu (200,) 0.001 50 : 0.8918186644289132, 0.9543935205728136
relu (200,) 0.01 500 : 0.8625400591176875, 0.9545097146522372
relu (200,) 0.01 200 : 0.8865372851640576, 0.9514719827794154
relu (200,) 0.01 100 : 0.8885664606935849, 0.9540103617935664
relu (200,) 0.01 50 : 0.8937982324088708, 0.9562949326163875
relu (200, 200) 0.0001 500 : 0.8567597903923472,
0.9440395005758236
relu (200, 200) 0.0001 200 : 0.8814816813227073,
0.9406232286463357
relu (200, 200) 0.0001 100 : 0.8838718087502695,
0.9462778100742297
relu (200, 200) 0.0001 50 : 0.8889710039756803,
0.9499367985719847
relu (200, 200) 0.001 500 : 0.8569709937611016,
0.9434937643258849
relu (200, 200) 0.001 200 : 0.8818085230998338,
0.9415956858277538
relu (200, 200) 0.001 100 : 0.8836496050740242,
0.946333876622408
relu (200, 200) 0.001 50 : 0.8888061943797698,
0.9484244257027477
relu (200, 200) 0.01 500 : 0.8563084380302779,
0.9437487687824283
relu (200, 200) 0.01 200 : 0.8811032176758475,
0.9418158486021677
relu (200, 200) 0.01 100 : 0.884000531129432, 0.9475606874380486
relu (200, 200) 0.01 50 : 0.8894150247343063, 0.9489851728378216
relu (100,) 0.0001 500 : 0.8627814378046278, 0.9558583864722148
relu (100,) 0.0001 200 : 0.885528876943128, 0.9517056129316046
relu (100,) 0.0001 100 : 0.8862180805415285, 0.9539481752564714
relu (100,) 0.0001 50 : 0.8915313831840237, 0.9560777084481825
relu (100,) 0.001 500 : 0.8626899086910653, 0.955876294796057
relu (100,) 0.001 200 : 0.8855554111074462, 0.9517618212648431
relu (100,) 0.001 100 : 0.886264298000895, 0.954061133416154
relu (100,) 0.001 50 : 0.8914571657302128, 0.9556592400390148
relu (100,) 0.01 500 : 0.8628414439183416, 0.9473537681878417
relu (100,) 0.01 200 : 0.8863735274528366, 0.9479978077280883
relu (100,) 0.01 100 : 0.8874167990276149, 0.9528541425740251
relu (100,) 0.01 50 : 0.8927377418202799, 0.9575799813680584
relu (100, 100) 0.0001 500 : 0.860755633354775,
0.9471594596550548
relu (100, 100) 0.0001 200 : 0.8815552841780938,
0.9441857314576464
relu (100, 100) 0.0001 100 : 0.8862895571695637,
0.9461981254195687
relu (100, 100) 0.0001 50 : 0.8896625880862142,
0.9504887282714637
relu (100, 100) 0.001 500 : 0.8602708115367129,
0.9487643131604875

relu (100, 100) 0.001 200 : 0.8809510222313822, 0.9432767104615248
relu (100, 100) 0.001 100 : 0.8857233390509748, 0.946384852909779
relu (100, 100) 0.001 50 : 0.8912749546093528, 0.94892068248401
relu (100, 100) 0.01 500 : 0.860475618360037, 0.9486735102427897
relu (100, 100) 0.01 200 : 0.8823448116912938, 0.9441985758005175
relu (100, 100) 0.01 100 : 0.8847647542542896, 0.9489009110428168
relu (100, 100) 0.01 50 : 0.8899893736967112, 0.9503711964130941
relu (100, 200) 0.0001 500 : 0.8562278519042875, 0.9495235863119865
relu (100, 200) 0.0001 200 : 0.8822879978267272, 0.9444320251525058
relu (100, 200) 0.0001 100 : 0.8850766724843016, 0.9495114885420174
relu (100, 200) 0.0001 50 : 0.8912758017552417, 0.9462734591592743
relu (100, 200) 0.001 500 : 0.8557743820805287, 0.9461243237116987
relu (100, 200) 0.001 200 : 0.8819184966874334, 0.944311150131086
relu (100, 200) 0.001 100 : 0.8834859637716088, 0.9485747049047538
relu (100, 200) 0.001 50 : 0.8909358367016653, 0.9474258089173587
relu (100, 200) 0.01 500 : 0.8555209445189416, 0.9473999678585835
relu (100, 200) 0.01 200 : 0.883090515441187, 0.9440639163592265
relu (100, 200) 0.01 100 : 0.8836377877690131, 0.9519246309193399
relu (100, 200) 0.01 50 : 0.8906862587829931, 0.9463733523573423
relu (50,) 0.0001 500 : 0.8644563781444241, 0.9554481999154545
relu (50,) 0.0001 200 : 0.8891680247941359, 0.952096024348903
relu (50,) 0.0001 100 : 0.8890307019538793, 0.9540465855110749
relu (50,) 0.0001 50 : 0.892574658289413, 0.9557314947955146
relu (50,) 0.001 500 : 0.8644692582208359, 0.9554743988331316
relu (50,) 0.001 200 : 0.8892281197770998, 0.9521580338990181
relu (50,) 0.001 100 : 0.8893430960376346, 0.9541657996391731
relu (50,) 0.001 50 : 0.8926865496851841, 0.9558670138837067
relu (50,) 0.01 500 : 0.8645804067061496, 0.9557071978977236
relu (50,) 0.01 200 : 0.8897717896175772, 0.9527582602342115
relu (50,) 0.01 100 : 0.8903488737227411, 0.9552673549636359
relu (50,) 0.01 50 : 0.8940566789519876, 0.9571142446058398
relu (50, 50) 0.0001 500 : 0.8601663813987456, 0.9486072765750974
relu (50, 50) 0.0001 200 : 0.8858697821980644, 0.9487404759076976
relu (50, 50) 0.0001 100 : 0.8871472570261899, 0.9464464968424163
relu (50, 50) 0.0001 50 : 0.8933465036447542, 0.9464034639537818
relu (50, 50) 0.001 500 : 0.8602404527555372, 0.950019118445338
relu (50, 50) 0.001 200 : 0.8856488845845268, 0.9491606762498984
relu (50, 50) 0.001 100 : 0.8871083189974932, 0.9457662516573987
relu (50, 50) 0.001 50 : 0.8966368687875026, 0.9448576107900882
relu (50, 50) 0.01 500 : 0.8594204204694098, 0.9510662073604423
relu (50, 50) 0.01 200 : 0.8859610201993794, 0.9500548563115205
relu (50, 50) 0.01 100 : 0.8876881320665027, 0.9466585621961235
relu (50, 50) 0.01 50 : 0.8943618285460737, 0.9486542298288583
relu (50, 100) 0.0001 500 : 0.8623155352741092, 0.9511377071875196
relu (50, 100) 0.0001 200 : 0.8839228028983823, 0.9443815883281388
relu (50, 100) 0.0001 100 : 0.8869029894932228, 0.943309920715689
relu (50, 100) 0.0001 50 : 0.8909021439083858, 0.949492721553072
relu (50, 100) 0.001 500 : 0.8623318645981936, 0.9517525484761905
relu (50, 100) 0.001 200 : 0.8832945700709885, 0.9439913565618611
relu (50, 100) 0.001 100 : 0.8862000997929969, 0.9408799403844993
relu (50, 100) 0.001 50 : 0.8917495100762112, 0.9437243655127828
relu (50, 100) 0.01 500 : 0.8627362022459419, 0.9515600375508159
relu (50, 100) 0.01 200 : 0.8841936917539386, 0.945161046769025
relu (50, 100) 0.01 100 : 0.8857559332408599, 0.9424014280281744
relu (50, 100) 0.01 50 : 0.890491236541258, 0.9511088850038478
relu (50, 200) 0.0001 500 : 0.8562859425577998, 0.9477792851288936
relu (50, 200) 0.0001 200 : 0.8875013019982159, 0.941771101846938
relu (50, 200) 0.0001 100 : 0.890107812389731, 0.9464912628617039
relu (50, 200) 0.0001 50 : 0.8924586547152996, 0.9512177592102278
relu (50, 200) 0.001 500 : 0.856038394988699, 0.9485700793901297
relu (50, 200) 0.001 200 : 0.8864430661765803, 0.943678664626109
relu (50, 200) 0.001 100 : 0.8890726271440862, 0.946871876784044
relu (50, 200) 0.001 50 : 0.894574000203518, 0.9516548525581107
relu (50, 200) 0.01 500 : 0.8562978565281195, 0.9482694846106932
relu (50, 200) 0.01 200 : 0.8874817226017162, 0.9433352632566073
relu (50, 200) 0.01 100 : 0.88939231965555, 0.9464748129112965
relu (50, 200) 0.01 50 : 0.89207856853203, 0.9477190718647336
tanh (200,) 0.0001 500 : 0.8665778371789074, 0.9253599370114767
tanh (200,) 0.0001 200 : 0.8878134178472502, 0.9212106464179459
tanh (200,) 0.0001 100 : 0.889893753537079, 0.9273172779490971
tanh (200,) 0.0001 50 : 0.8954324789284994, 0.9309603583088012
tanh (200,) 0.001 500 : 0.8670625551200359, 0.9263466038974679
tanh (200,) 0.001 200 : 0.8889155836388583, 0.922025225827548
tanh (200,) 0.001 100 : 0.8910760932129055, 0.9282653872872502
tanh (200,) 0.001 50 : 0.8960224139287136, 0.9325276073827172
tanh (200,) 0.01 500 : 0.8714526443261106, 0.9329187790981339
tanh (200,) 0.01 200 : 0.8944815702430621, 0.9274669450345618
tanh (200,) 0.01 100 : 0.8965801562331153, 0.9364923331891897
tanh (200,) 0.01 50 : 0.8998846632172268, 0.936154490188781
tanh (200, 200) 0.0001 500 : 0.870849176710365, 0.9357390907548517
tanh (200, 200) 0.0001 200 : 0.8967125360200459, 0.9288929073485518
tanh (200, 200) 0.0001 100 : 0.9014549340880229, 0.9355385372887437
tanh (200, 200) 0.0001 50 : 0.9053427267583658, 0.9359446310062992
tanh (200, 200) 0.001 500 : 0.8717688144108224, 0.9361876582657206
tanh (200, 200) 0.001 200 : 0.8979341937852874, 0.9319637811080942
tanh (200, 200) 0.001 100 : 0.901577713224589, 0.9363329471787998
tanh (200, 200) 0.001 50 : 0.905839172189092, 0.9358190860633527
tanh (200, 200) 0.01 500 : 0.8805971239366194, -0.01992879296173533
tanh (200, 200) 0.01 200 : 0.9029681092011328, 0.9344484247872475
tanh (200, 200) 0.01 100 : 0.9052888379046979, 0.9370213839652902
tanh (200, 200) 0.01 50 : 0.9094265236011838, 0.9415848149020406
tanh (100,) 0.0001 500 : 0.8513371781288356, 0.9220644280258987
tanh (100,) 0.0001 200 : 0.8749555184834945, 0.9187027022934502
tanh (100,) 0.0001 100 : 0.8790174141470247, 0.9248653564740422
tanh (100,) 0.0001 50 : 0.8863626195591753, 0.9305223525189719
tanh (100,) 0.001 500 : 0.8530217350832643, 0.9233800319363004
tanh (100,) 0.001 200 : 0.8775072402325446, 0.9209121982883427
tanh (100,) 0.001 100 : 0.8818836127605547, 0.92753985394826
tanh (100,) 0.001 50 : 0.8875950870504115, 0.9319294375830478
tanh (100,) 0.01 500 : 0.8645832469716609, 0.9314392365132151
tanh (100,) 0.01 200 : 0.8860044662876568, 0.9290462128751772
tanh (100,) 0.01 100 : 0.8889169145663531, 0.932828783368641
tanh (100,) 0.01 50 : 0.8938932405749235, 0.9397795029007362
tanh (100, 100) 0.0001 500 : 0.8663775398532028, 0.9348471791794375
tanh (100, 100) 0.0001 200 : 0.8991595740362364, 0.9273337833670272
tanh (100, 100) 0.0001 100 : 0.9030706427126688, 0.9389643945188662
tanh (100, 100) 0.0001 50 : 0.9064100837617308, 0.9393999067527594

tanh (100, 100) 0.001 500 : 0.8680593776166912, 0.9355170189372921
tanh (100, 100) 0.001 200 : 0.8991536277464315, 0.9281246938017147
tanh (100, 100) 0.001 100 : 0.9035841287055479, 0.9401845699846987
tanh (100, 100) 0.001 50 : 0.9073693458226977, 0.9423349302456628
tanh (100, 100) 0.01 500 : 0.8818554428978047, 0.9377089621739103
tanh (100, 100) 0.01 200 : 0.9041961276870374, 0.9325213650942112
tanh (100, 100) 0.01 100 : 0.9066400565034517, 0.9425812379765486
tanh (100, 100) 0.01 50 : 0.911184538129417, 0.9451287572002542
tanh (100, 200) 0.0001 500 : 0.8652968205260096, 0.928487622257228
tanh (100, 200) 0.0001 200 : 0.8937738777812602, 0.9306235716666209
tanh (100, 200) 0.0001 100 : 0.8982649762301488, 0.9308161685338496
tanh (100, 200) 0.0001 50 : 0.9058476264944437, 0.9383629870819257
tanh (100, 200) 0.001 500 : 0.8657555928957092, 0.9267957876008568
tanh (100, 200) 0.001 200 : 0.8942723329523327, 0.930968689038334
tanh (100, 200) 0.001 100 : 0.8984424425348824, 0.9311556360459178
tanh (100, 200) 0.001 50 : 0.9060723083479196, 0.9354518159081479
tanh (100, 200) 0.01 500 : 0.8703155384835952, 0.930786932079323
tanh (100, 200) 0.01 200 : 0.8961012718868316, 0.9321672725364757
tanh (100, 200) 0.01 100 : 0.9000551177852081, 0.9336282859018845
tanh (100, 200) 0.01 50 : 0.9073517248902627, 0.9402580307792393
tanh (50,) 0.0001 500 : 0.8461349093249776, 0.9229187334345776
tanh (50,) 0.0001 200 : 0.8702243809242687, 0.9212088053680942
tanh (50,) 0.0001 100 : 0.8763802441143895, 0.9210326964265089
tanh (50,) 0.0001 50 : 0.8829741248967542, 0.9267654699349431
tanh (50,) 0.001 500 : 0.8490320126062857, 0.9238662077224302
tanh (50,) 0.001 200 : 0.8740922883469132, 0.9230896434027175
tanh (50,) 0.001 100 : 0.8800077443475104, 0.9244212859590607
tanh (50,) 0.001 50 : 0.8877042688675196, 0.9283409615184232
tanh (50,) 0.01 500 : 0.8661894126363798, 0.9246445189637262
tanh (50,) 0.01 200 : 0.88365458838548, 0.9249648629709767
tanh (50,) 0.01 100 : 0.8885362904602927, 0.9297407927469472
tanh (50,) 0.01 50 : 0.895727623547913, 0.938464825366171
tanh (50, 50) 0.0001 500 : 0.8594979821616324, 0.925714480377392
tanh (50, 50) 0.0001 200 : 0.8905355474164377, 0.9303530406474486
tanh (50, 50) 0.0001 100 : 0.895562213386534, 0.9375785164992758
tanh (50, 50) 0.0001 50 : 0.8988343939532225, 0.9404874787946254
tanh (50, 50) 0.001 500 : 0.8618028838485557, 0.9289591231090857
tanh (50, 50) 0.001 200 : 0.8897576282847574, 0.9294353623101788
tanh (50, 50) 0.001 100 : 0.8967214336344431, 0.938377432427873
tanh (50, 50) 0.001 50 : 0.9002367436535561, 0.9425883511718713
tanh (50, 50) 0.01 500 : 0.8789279156702982, 0.9325358953306403
tanh (50, 50) 0.01 200 : 0.9007674213072064, 0.9342167330260671
tanh (50, 50) 0.01 100 : 0.9020188873711685, 0.9421937526141289
tanh (50, 50) 0.01 50 : 0.9035816851725265, 0.9411900340176573
tanh (50, 100) 0.0001 500 : 0.8631899410065011, 0.9246157126182777
tanh (50, 100) 0.0001 200 : 0.8934719392079933, 0.9268174972491705
tanh (50, 100) 0.0001 100 : 0.896275139821568, 0.9337569779105346
tanh (50, 100) 0.0001 50 : 0.9025093319283686, 0.9395685126179223
tanh (50, 100) 0.001 500 : 0.8640463475865877, 0.9251345428581014
tanh (50, 100) 0.001 200 : 0.8938904702634239, 0.9278115238906304
tanh (50, 100) 0.001 100 : 0.8966453130221603, 0.9342910084661662
tanh (50, 100) 0.001 50 : 0.9025181390516132, 0.9419552136913868
tanh (50, 100) 0.01 500 : 0.8725140260689479, 0.9328669250938175
tanh (50, 100) 0.01 200 : 0.8978442048295079, 0.9310335985358769
tanh (50, 100) 0.01 100 : 0.9005502466551096, 0.938489334155461
tanh (50, 100) 0.01 50 : 0.9044751112045658, 0.9431098073399392
tanh (50, 200) 0.0001 500 : 0.8736329899726479, 0.926065669896998
tanh (50, 200) 0.0001 200 : 0.9010962802854028, 0.923429060757982
tanh (50, 200) 0.0001 100 : 0.9020433554804932, 0.9329265821243518
tanh (50, 200) 0.0001 50 : 0.90669487977611, 0.9352717549725759
tanh (50, 200) 0.001 500 : 0.8738248861329133, 0.9260340074005545
tanh (50, 200) 0.001 200 : 0.9014253076525621, 0.9236831181142386
tanh (50, 200) 0.001 100 : 0.9023569007171763, 0.933323709074331
tanh (50, 200) 0.001 50 : 0.9074335129238982, 0.9352682812200688
tanh (50, 200) 0.01 500 : 0.8756755418435554, 0.9290179746471348
tanh (50, 200) 0.01 200 : 0.9034056766893552, 0.926088661587193
tanh (50, 200) 0.01 100 : 0.9040715777733489, 0.9326278714461003
tanh (50, 200) 0.01 50 : 0.9072650183687998, 0.93620440066658
logistic (200,) 0.0001 500 : 0.867856849738528, 0.9280901926243551
logistic (200,) 0.0001 200 : 0.8962227720667559, 0.9229525464626116
logistic (200,) 0.0001 100 : 0.8973490062611088, 0.929102420124674
logistic (200,) 0.0001 50 : 0.9021290645104608, 0.9348496643688339
logistic (200,) 0.001 500 : 0.869744649522158, 0.9303698591463022
logistic (200,) 0.001 200 : 0.8989329120184127, 0.9252524187301246
logistic (200,) 0.001 100 : 0.9004273706610079, 0.938260038789231
logistic (200,) 0.001 50 : 0.9044830508911315, 0.9394133906761092
logistic (200,) 0.01 500 : 0.8853263301186456, 0.9487109642020726
logistic (200,) 0.01 200 : 0.9106573024867608, 0.9539772581365037
logistic (200,) 0.01 100 : 0.9113184626437054, 0.953910958181051
logistic (200,) 0.01 50 : 0.9130859045723076, 0.9566645532999928
logistic (200, 200) 0.0001 500 : 0.8638568338181349, 0.9230170693226449
logistic (200, 200) 0.0001 200 : 0.8928905601634108, 0.9047085279767735
logistic (200, 200) 0.0001 100 : 0.8955673050499581, 0.9198551300096681
logistic (200, 200) 0.0001 50 : 0.9004825770787164, 0.9292840121306215
logistic (200, 200) 0.001 500 : 0.8739225386483588, 0.9281708627818639
logistic (200, 200) 0.001 200 : 0.8977345571353658, 0.9109584758769996
logistic (200, 200) 0.001 100 : 0.8987346163481675, 0.9221355149181643
logistic (200, 200) 0.001 50 : 0.9051301507039271, 0.9341907330678133
logistic (200, 200) 0.01 500 : 0.8890494562675952, 0.9337995247378562
logistic (200, 200) 0.01 200 : 0.8913482194765109, 0.9229766097338596
logistic (200, 200) 0.01 100 : 0.8975541786352537, 0.9364912440214705
logistic (200, 200) 0.01 50 : 0.9020260051520905, 0.9461157660338662
logistic (100,) 0.0001 500 : 0.8670292220526582, 0.9289840493780945
logistic (100,) 0.0001 200 : 0.8902929408864317, 0.9232102546796461
logistic (100,) 0.0001 100 : 0.8909193115501598, 0.9272931258897372
logistic (100,) 0.0001 50 : 0.89526438292372, 0.9314806521892762
logistic (100,) 0.001 500 : 0.8708106356091742, 0.9324712378649459

logistic (100,) 0.001 200 : 0.8957326938346843, 0.9274721228251877
logistic (100,) 0.001 100 : 0.8962869922764318, 0.9332640299228057
logistic (100,) 0.001 50 : 0.9001293700672385, 0.9377201638475985
logistic (100,) 0.01 500 : 0.8900122783203945, 0.9426624105499701
logistic (100,) 0.01 200 : 0.9055775124976039, 0.9437425717830601
logistic (100,) 0.01 100 : 0.9056290571355164, 0.948028525866672
logistic (100,) 0.01 50 : 0.9076774767226274, 0.9533413058102768
logistic (100, 100) 0.0001 500 : 0.8687830188561992, 0.919991489532571
logistic (100, 100) 0.0001 200 : 0.8896529185118423, 0.9086707287447128
logistic (100, 100) 0.0001 100 : 0.8911176944229615, 0.9226216626710035
logistic (100, 100) 0.0001 50 : 0.9027727599194089, 0.9314911186484504
logistic (100, 100) 0.001 500 : 0.878083567415995, 0.9273792794729435
logistic (100, 100) 0.001 200 : 0.8936854109649859, 0.917603769403147
logistic (100, 100) 0.001 100 : 0.8951692650972977, 0.9291895897070775
logistic (100, 100) 0.001 50 : 0.9001299524071763, 0.9322229417849415
logistic (100, 100) 0.01 500 : 0.8728213911927405, 0.9267721853756743
logistic (100, 100) 0.01 200 : 0.8833231621831337, 0.9141339301459965
logistic (100, 100) 0.01 100 : 0.8861546531467888, 0.9283935619164665
logistic (100, 100) 0.01 50 : 0.8972966760337272, 0.9378845936962269
logistic (100, 200) 0.0001 500 : 0.8681030934178912, 0.9128081604268418
logistic (100, 200) 0.0001 200 : 0.888289190272174, 0.902336457352211
logistic (100, 200) 0.0001 100 : 0.8917033515526605, 0.9185535413476846
logistic (100, 200) 0.0001 50 : 0.8992292591620605, 0.928827532315435
logistic (100, 200) 0.001 500 : 0.8724936349720224, 0.9217928121820953
logistic (100, 200) 0.001 200 : 0.8950088196092942, 0.915335076707303
logistic (100, 200) 0.001 100 : 0.8955040138969224, 0.9243685764548759
logistic (100, 200) 0.001 50 : 0.8992952307069949, 0.9271523600071618
logistic (100, 200) 0.01 500 : 0.8890601525848414, 0.9235874712261709
logistic (100, 200) 0.01 200 : 0.8903912650163198, 0.9205910978662303
logistic (100, 200) 0.01 100 : 0.894615122031879, 0.9264362926869154
logistic (100, 200) 0.01 50 : 0.9011571298429886, 0.9329926160648975
logistic (50,) 0.0001 500 : 0.8667514267113562, 0.9262245746852785
logistic (50,) 0.0001 200 : 0.8881400981349479, 0.9209632389844579
logistic (50,) 0.0001 100 : 0.8868226664316049, 0.9278455717072059
logistic (50,) 0.0001 50 : 0.8920138904590683, 0.9296704836101262
logistic (50,) 0.001 500 : 0.8728029063044023, 0.929522767772641
logistic (50,) 0.001 200 : 0.8928915063854482, 0.9296279129783485
logistic (50,) 0.001 100 : 0.8927561590030789, 0.9362456403563256
logistic (50,) 0.001 50 : 0.8964529781680554, 0.9365562768432454
logistic (50,) 0.01 500 : 0.8866666669554695, 0.933303692390638
logistic (50,) 0.01 200 : 0.8978127578870747, 0.9403298311893992
logistic (50,) 0.01 100 : 0.902223220411224, 0.9479440269236032
logistic (50,) 0.01 50 : 0.9060153771888876, 0.9498128232391977
logistic (50, 50) 0.0001 500 : 0.8673904944073898, 0.9223571055309225
logistic (50, 50) 0.0001 200 : 0.8950070048072829, 0.9220820814871501
logistic (50, 50) 0.0001 100 : 0.8981674068656244, 0.9290930733951447
logistic (50, 50) 0.0001 50 : 0.9061873355496939, 0.9393136221031612
logistic (50, 50) 0.001 500 : 0.8830433490422095, 0.9255541336199582
logistic (50, 50) 0.001 200 : 0.89404553832711, 0.9298902055116726
logistic (50, 50) 0.001 100 : 0.8967098302928654, 0.9356223642075487
logistic (50, 50) 0.001 50 : 0.9079303275630306, 0.9435308228010976
logistic (50, 50) 0.01 500 : 0.8691391927058479, 0.9325225617695692
logistic (50, 50) 0.01 200 : 0.8783446646052667, 0.925758148918816
logistic (50, 50) 0.01 100 : 0.8835308052423304, 0.9320352182556622
logistic (50, 50) 0.01 50 : 0.8941847620765268, 0.9405615536766314
logistic (50, 100) 0.0001 500 : 0.873017367095492, 0.9270205479492569
logistic (50, 100) 0.0001 200 : 0.888274675915647, 0.916866499833467
logistic (50, 100) 0.0001 100 : 0.8954468371496972, 0.9243800371857299
logistic (50, 100) 0.0001 50 : 0.9031448429393703, 0.9343802703355025
logistic (50, 100) 0.001 500 : 0.8790890119313707, 0.9309206529070135
logistic (50, 100) 0.001 200 : 0.891568703710969, 0.9226487155432472
logistic (50, 100) 0.001 100 : 0.8975655055280942, 0.9301523895274106
logistic (50, 100) 0.001 50 : 0.90534570367183, 0.9360193359476114
logistic (50, 100) 0.01 500 : 0.888283152147823, 0.9266327642798546
logistic (50, 100) 0.01 200 : 0.889484593229241, 0.9325533543518385
logistic (50, 100) 0.01 100 : 0.8950283088307431, 0.9387053504548009
logistic (50, 100) 0.01 50 : 0.9007773003218766, 0.9400825953043518
logistic (50, 200) 0.0001 500 : 0.8626508915278315, 0.922196028576909
logistic (50, 200) 0.0001 200 : 0.8937425845945315, 0.911215322286158
logistic (50, 200) 0.0001 100 : 0.8969166967044704, 0.922646310014201
logistic (50, 200) 0.0001 50 : 0.9002101575768096, 0.9260647128935562
logistic (50, 200) 0.001 500 : 0.8672250424808363, 0.9168406274065843
logistic (50, 200) 0.001 200 : 0.8970739458787303, 0.9152197074584846
logistic (50, 200) 0.001 100 : 0.8968862110849998, 0.9260904528723125
logistic (50, 200) 0.001 50 : 0.9018714312264384, 0.9302926520169472
logistic (50, 200) 0.01 500 : 0.8861909618580123, 0.9291766223597244
logistic (50, 200) 0.01 200 : 0.9024761015369138, 0.9296558765933959
logistic (50, 200) 0.01 100 : 0.9021707095423551, 0.9361253130329114
logistic (50, 200) 0.01 50 : 0.9052814172535399, 0.9410798926078926

Grid search of LightGBM, Morgan(r=2,2048)
lgb.LGBMRegressor(boosting_type = "gbdt", num_leaves = j,max_depth = 0)

j: accuracy of prediction using cross validation, accuracy of prediction using test data
10 : 0.7698300857530949, 0.849807769648899
50 : 0.781369278638787, 0.8485689227098541
100 : 0.781369278638787, 0.8485689227098541
150 : 0.781369278638787, 0.8485689227098541

Grid search of SVR, Morgan(r=2, 2048)
SVR(C= c_num, kernel = ker, epsilon = e, gamma = r,degree = 3, coef0=1)
Ker c_num r e: accuracy of prediction using cross validation, accuracy of prediction using test data
linear 1 1 0.1 6 : 0.893146236342055, 0.9683832989405823
linear 1 1 0.3 6 : 0.8935098843776677, 0.9678082788436517
linear 1 1 0.5 6 : 0.8928911050550787, 0.9673214477382609
linear 1 1 0.7 6 : 0.892082915543092, 0.9662669401057911
linear 1 1 0.9 6 : 0.8910697940122645, 0.9646250099632373
linear 1 2 0.1 6 : 0.893146236342055, 0.9683832989405823
linear 1 2 0.3 6 : 0.8935098843776677, 0.9678082788436517
linear 1 2 0.5 6 : 0.8928911050550787, 0.9673214477382609
linear 1 2 0.7 6 : 0.892082915543092, 0.9662669401057911
linear 1 2 0.9 6 : 0.8910697940122645, 0.9646250099632373
linear 1 3 0.1 6 : 0.893146236342055, 0.9683832989405823
linear 1 3 0.3 6 : 0.8935098843776677, 0.9678082788436517
linear 1 3 0.5 6 : 0.8928911050550787, 0.9673214477382609
linear 1 3 0.7 6 : 0.892082915543092, 0.9662669401057911
linear 1 3 0.9 6 : 0.8910697940122645, 0.9646250099632373
linear 1 4 0.1 6 : 0.893146236342055, 0.9683832989405823
linear 1 4 0.3 6 : 0.8935098843776677, 0.9678082788436517
linear 1 4 0.5 6 : 0.8928911050550787, 0.9673214477382609
linear 1 4 0.7 6 : 0.892082915543092, 0.9662669401057911
linear 1 4 0.9 6 : 0.8910697940122645, 0.9646250099632373
linear 1 5 0.1 6 : 0.893146236342055, 0.9683832989405823
linear 1 5 0.3 6 : 0.8935098843776677, 0.9678082788436517
linear 1 5 0.5 6 : 0.8928911050550787, 0.9673214477382609
linear 1 5 0.7 6 : 0.892082915543092, 0.9662669401057911
linear 1 5 0.9 6 : 0.8910697940122645, 0.9646250099632373
linear 1 6 0.1 6 : 0.893146236342055, 0.9683832989405823
linear 1 6 0.3 6 : 0.8935098843776677, 0.9678082788436517
linear 1 6 0.5 6 : 0.8928911050550787, 0.9673214477382609
linear 1 6 0.7 6 : 0.892082915543092, 0.9662669401057911
linear 1 6 0.9 6 : 0.8910697940122645, 0.9646250099632373
linear 2 1 0.1 6 : 0.9204469802778166, 0.9731540943765135
linear 2 1 0.3 6 : 0.9205112324012923, 0.9724318570883759
linear 2 1 0.5 6 : 0.9202812891222386, 0.9718618148735467
linear 2 1 0.7 6 : 0.9192677011784646, 0.9708113554997357
linear 2 1 0.9 6 : 0.9178228435519878, 0.9688897036777235
linear 2 2 0.1 6 : 0.9204469802778166, 0.9731540943765135
linear 2 2 0.3 6 : 0.9205112324012923, 0.9724318570883759
linear 2 2 0.5 6 : 0.9202812891222386, 0.9718618148735467
linear 2 2 0.7 6 : 0.9192677011784646, 0.9708113554997357
linear 2 2 0.9 6 : 0.9178228435519878, 0.9688897036777235
linear 2 3 0.1 6 : 0.9204469802778166, 0.9731540943765135
linear 2 3 0.3 6 : 0.9205112324012923, 0.9724318570883759
linear 2 3 0.5 6 : 0.9202812891222386, 0.9718618148735467
linear 2 3 0.7 6 : 0.9192677011784646, 0.9708113554997357
linear 2 3 0.9 6 : 0.9178228435519878, 0.9688897036777235
linear 2 4 0.1 6 : 0.9204469802778166, 0.9731540943765135
linear 2 4 0.3 6 : 0.9205112324012923, 0.9724318570883759
linear 2 4 0.5 6 : 0.9202812891222386, 0.9718618148735467
linear 2 4 0.7 6 : 0.9192677011784646, 0.9708113554997357
linear 2 4 0.9 6 : 0.9178228435519878, 0.9688897036777235
linear 2 5 0.1 6 : 0.9204469802778166, 0.9731540943765135
linear 2 5 0.3 6 : 0.9205112324012923, 0.9724318570883759
linear 2 5 0.5 6 : 0.9202812891222386, 0.9718618148735467
linear 2 5 0.7 6 : 0.9192677011784646, 0.9708113554997357
linear 2 5 0.9 6 : 0.9178228435519878, 0.9688897036777235
linear 2 6 0.1 6 : 0.9204469802778166, 0.9731540943765135
linear 2 6 0.3 6 : 0.9205112324012923, 0.9724318570883759
linear 2 6 0.5 6 : 0.9202812891222386, 0.9718618148735467
linear 2 6 0.7 6 : 0.9192677011784646, 0.9708113554997357
linear 2 6 0.9 6 : 0.9178228435519878, 0.9688897036777235
linear 3 1 0.1 6 : 0.924613140113596, 0.9730032846847895
linear 3 1 0.3 6 : 0.9246851559776339, 0.9722836455128037
linear 3 1 0.5 6 : 0.9239945038690902, 0.9718367911590408
linear 3 1 0.7 6 : 0.9231398736652429, 0.9708631654484875
linear 3 1 0.9 6 : 0.9215860469387926, 0.9692904764724256
linear 3 2 0.1 6 : 0.924613140113596, 0.9730032846847895
linear 3 2 0.3 6 : 0.9246851559776339, 0.9722836455128037
linear 3 2 0.5 6 : 0.9239945038690902, 0.9718367911590408
linear 3 2 0.7 6 : 0.9231398736652429, 0.9708631654484875
linear 3 2 0.9 6 : 0.9215860469387926, 0.9692904764724256
linear 3 3 0.1 6 : 0.924613140113596, 0.9730032846847895
linear 3 3 0.3 6 : 0.9246851559776339, 0.9722836455128037
linear 3 3 0.5 6 : 0.9239945038690902, 0.9718367911590408
linear 3 3 0.7 6 : 0.9231398736652429, 0.9708631654484875
linear 3 3 0.9 6 : 0.9215860469387926, 0.9692904764724256
linear 3 4 0.1 6 : 0.924613140113596, 0.9730032846847895
linear 3 4 0.3 6 : 0.9246851559776339, 0.9722836455128037
linear 3 4 0.5 6 : 0.9239945038690902, 0.9718367911590408
linear 3 4 0.7 6 : 0.9231398736652429, 0.9708631654484875
linear 3 4 0.9 6 : 0.9215860469387926, 0.9692904764724256
linear 3 5 0.1 6 : 0.924613140113596, 0.9730032846847895
linear 3 5 0.3 6 : 0.9246851559776339, 0.9722836455128037
linear 3 5 0.5 6 : 0.9239945038690902, 0.9718367911590408
linear 3 5 0.7 6 : 0.9231398736652429, 0.9708631654484875
linear 3 5 0.9 6 : 0.9215860469387926, 0.9692904764724256
linear 3 6 0.1 6 : 0.924613140113596, 0.9730032846847895
linear 3 6 0.3 6 : 0.9246851559776339, 0.9722836455128037
linear 3 6 0.5 6 : 0.9239945038690902, 0.9718367911590408
linear 3 6 0.7 6 : 0.9231398736652429, 0.9708631654484875
linear 3 6 0.9 6 : 0.9215860469387926, 0.9692904764724256
linear 4 1 0.1 6 : 0.9252697220055219, 0.9726839375381845
linear 4 1 0.3 6 : 0.9255853813120423, 0.9721624175859596
linear 4 1 0.5 6 : 0.9248923181076835, 0.9718713324900435
linear 4 1 0.7 6 : 0.9241327247513187, 0.9710505225422348
linear 4 1 0.9 6 : 0.9222471195547598, 0.9693481526838854
linear 4 2 0.1 6 : 0.9252697220055219, 0.9726839375381845
linear 4 2 0.3 6 : 0.9255853813120423, 0.9721624175859596
linear 4 2 0.5 6 : 0.9248923181076835, 0.9718713324900435
linear 4 2 0.7 6 : 0.9241327247513187, 0.9710505225422348
linear 4 2 0.9 6 : 0.9222471195547598, 0.9693481526838854
linear 4 3 0.1 6 : 0.9252697220055219, 0.9726839375381845
linear 4 3 0.3 6 : 0.9255853813120423, 0.9721624175859596
linear 4 3 0.5 6 : 0.9248923181076835, 0.9718713324900435
linear 4 3 0.7 6 : 0.9241327247513187, 0.9710505225422348
linear 4 3 0.9 6 : 0.9222471195547598, 0.9693481526838854
linear 4 4 0.1 6 : 0.9252697220055219, 0.9726839375381845
linear 4 4 0.3 6 : 0.9255853813120423, 0.9721624175859596
linear 4 4 0.5 6 : 0.9248923181076835, 0.9718713324900435
linear 4 4 0.7 6 : 0.9241327247513187, 0.9710505225422348
linear 4 4 0.9 6 : 0.9222471195547598, 0.9693481526838854
linear 4 5 0.1 6 : 0.9252697220055219, 0.9726839375381845
linear 4 5 0.3 6 : 0.9255853813120423, 0.9721624175859596
linear 4 5 0.5 6 : 0.9248923181076835, 0.9718713324900435
linear 4 5 0.7 6 : 0.9241327247513187, 0.9710505225422348
linear 4 5 0.9 6 : 0.9222471195547598, 0.9693481526838854
linear 4 6 0.1 6 : 0.9252697220055219, 0.9726839375381845
linear 4 6 0.3 6 : 0.9255853813120423, 0.9721624175859596
linear 4 6 0.5 6 : 0.9248923181076835, 0.9718713324900435
linear 4 6 0.7 6 : 0.9241327247513187, 0.9710505225422348
linear 4 6 0.9 6 : 0.9222471195547598, 0.9693481526838854
poly 1 1 0.1 6 : 0.8247165526577241, 0.887389315778859
poly 1 1 0.3 6 : 0.8221328513703561, 0.8845032344524612
poly 1 1 0.5 6 : 0.8193775615228226, 0.8814003771343725
poly 1 1 0.7 6 : 0.8165261170057401, 0.8782924531642338
poly 1 1 0.9 6 : 0.813546692926692, 0.8750960567783499
poly 1 2 0.1 6 : 0.8229097836850169, 0.8856189761432903
poly 1 2 0.3 6 : 0.8203122338740634, 0.8827223864663795
poly 1 2 0.5 6 : 0.8175533679241156, 0.8796105018407575
poly 1 2 0.7 6 : 0.8147015511615532, 0.8764923723494453
poly 1 2 0.9 6 : 0.8117185604889254, 0.8732976868227109
poly 1 3 0.1 6 : 0.8222937333346995, 0.8850127068271001
poly 1 3 0.3 6 : 0.8196919695384064, 0.8821137681228162
poly 1 3 0.5 6 : 0.8169320610542004, 0.8789976410367659
poly 1 3 0.7 6 : 0.8140797902680454, 0.8758769685088168
poly 1 3 0.9 6 : 0.8110948295219298, 0.8726827449406043
poly 1 4 0.1 6 : 0.8219823226608586, 0.8847066117743676

poly 1 4 0.3 6 : 0.81937840017574, 0.8818068972743957
poly 1 4 0.5 6 : 0.8166184150711194, 0.8786880746293543
poly 1 4 0.7 6 : 0.8137663088085798, 0.875566103545406
poly 1 4 0.9 6 : 0.8107806675189533, 0.8723718330388999
poly 1 5 0.1 6 : 0.8217950178708555, 0.8845219163363887
poly 1 5 0.3 6 : 0.8191900984732412, 0.8816215744173731
poly 1 5 0.5 6 : 0.8164298032248695, 0.8785012497441458
poly 1 5 0.7 6 : 0.8135771969341882, 0.8753795972392946
poly 1 5 0.9 6 : 0.8105905629066541, 0.8721849049343458
poly 1 6 0.1 6 : 0.8216698809063663, 0.8843979707850654
poly 1 6 0.3 6 : 0.8190636894162608, 0.8814970435362374
poly 1 6 0.5 6 : 0.8163040451218014, 0.8783761202390262
poly 1 6 0.7 6 : 0.8134505130120473, 0.8752545010975336
poly 1 6 0.9 6 : 0.8104651875970139, 0.8720603272619807
poly 2 1 0.1 6 : 0.8247165526577112, 0.8873893157789164
poly 2 1 0.3 6 : 0.8221328513703412, 0.8845032344523053
poly 2 1 0.5 6 : 0.8193775615227672, 0.88140037713456
poly 2 1 0.7 6 : 0.8165261170057846, 0.8782924531642863
poly 2 1 0.9 6 : 0.813546692926692, 0.8750960567783499
poly 2 2 0.1 6 : 0.8229097836854699, 0.8856189761432489
poly 2 2 0.3 6 : 0.820312233874325, 0.882722386465428
poly 2 2 0.5 6 : 0.8175533679242684, 0.8796105018407274
poly 2 2 0.7 6 : 0.8147015511617883, 0.8764923723492354
poly 2 2 0.9 6 : 0.8117185604889254, 0.8732976868227109
poly 2 3 0.1 6 : 0.8222937333338957, 0.8850127068296587
poly 2 3 0.3 6 : 0.8196919887856788, 0.8821137681239283
poly 2 3 0.5 6 : 0.8169320610544594, 0.8789976410394913
poly 2 3 0.7 6 : 0.8140797902673773, 0.8758769685084868
poly 2 3 0.9 6 : 0.8110948295219298, 0.8726827449406043
poly 2 4 0.1 6 : 0.8219823226596688, 0.8847066117793196
poly 2 4 0.3 6 : 0.8193784001783747, 0.8818068972709212
poly 2 4 0.5 6 : 0.8166184150774592, 0.8786880746302993
poly 2 4 0.7 6 : 0.8137663088075872, 0.875566103543228
poly 2 4 0.9 6 : 0.8107806675189533, 0.8723718330388999
poly 2 5 0.1 6 : 0.8217950178596425, 0.8845219163319639
poly 2 5 0.3 6 : 0.8191900984739304, 0.8816214832270021
poly 2 5 0.5 6 : 0.8164298032370461, 0.8785012497530513
poly 2 5 0.7 6 : 0.813577196931428, 0.8753795972362626
poly 2 5 0.9 6 : 0.8105905629066541, 0.8721849049343458
poly 2 6 0.1 6 : 0.8216698808916758, 0.8843979708117211
poly 2 6 0.3 6 : 0.8190636893838681, 0.8814970435265841
poly 2 6 0.5 6 : 0.8163040451225549, 0.8783761202775592
poly 2 6 0.7 6 : 0.8134505129943695, 0.8752545011059805
poly 2 6 0.9 6 : 0.8104651875970139, 0.8720603272619807
poly 3 1 0.1 6 : 0.8247165526577103, 0.8873893157790135
poly 3 1 0.3 6 : 0.8221328513702755, 0.8845032344526056
poly 3 1 0.5 6 : 0.8193775615228216, 0.8814003771345429
poly 3 1 0.7 6 : 0.8165261170058065, 0.8782924531643812
poly 3 1 0.9 6 : 0.813546692926692, 0.8750960567783499
poly 3 2 0.1 6 : 0.8229097836853179, 0.8856189761428341
poly 3 2 0.3 6 : 0.8203122338749289, 0.8827223864655565
poly 3 2 0.5 6 : 0.8175533679242915, 0.8796105018396613
poly 3 2 0.7 6 : 0.8147015511616456, 0.8764923723495796
poly 3 2 0.9 6 : 0.8117185604889254, 0.8732976868227109
poly 3 3 0.1 6 : 0.8222937333357084, 0.8850127068189134
poly 3 3 0.3 6 : 0.8196919887853241, 0.8821137681223578
poly 3 3 0.5 6 : 0.8169320610521533, 0.8789976410385614
poly 3 3 0.7 6 : 0.8140797902678267, 0.8758769685123248
poly 3 3 0.9 6 : 0.8110948295219298, 0.8726827449406043
poly 3 4 0.1 6 : 0.821982322657987, 0.884706611800941
poly 3 4 0.3 6 : 0.819378400180953, 0.881806897268766
poly 3 4 0.5 6 : 0.8166184150775996, 0.8786880746276393
poly 3 4 0.7 6 : 0.8137663088086082, 0.8755661035456097
poly 3 4 0.9 6 : 0.8107806675189533, 0.8723718330388999
poly 3 5 0.1 6 : 0.8217950178579898, 0.8845219163421006
poly 3 5 0.3 6 : 0.8191900984845388, 0.8816214832104745
poly 3 5 0.5 6 : 0.8164298032165593, 0.8785012497353336
poly 3 5 0.7 6 : 0.8135771969390607, 0.8753795972373538
poly 3 5 0.9 6 : 0.8105905629066541, 0.8721849049343458
poly 3 6 0.1 6 : 0.8216698808917146, 0.8843979708284953
poly 3 6 0.3 6 : 0.8190636893965557, 0.8814970435420633
poly 3 6 0.5 6 : 0.816304045116327, 0.878376120266825
poly 3 6 0.7 6 : 0.8134505129851434, 0.875254501085494
poly 3 6 0.9 6 : 0.8104651875970139, 0.8720603272619807
poly 4 1 0.1 6 : 0.8247165526577147, 0.8873893157787048
poly 4 1 0.3 6 : 0.8221328513703658, 0.884503234452705
poly 4 1 0.5 6 : 0.819377561522775, 0.8814003771343433
poly 4 1 0.7 6 : 0.8165261170057952, 0.8782924531644544
poly 4 1 0.9 6 : 0.813546692926692, 0.8750960567783499
poly 4 2 0.1 6 : 0.8229097836850678, 0.885618976142878
poly 4 2 0.3 6 : 0.8203122338751813, 0.8827223864660299
poly 4 2 0.5 6 : 0.8175533679241141, 0.8796105018396443
poly 4 2 0.7 6 : 0.8147015511612572, 0.8764923723499191
poly 4 2 0.9 6 : 0.8117185604889254, 0.8732976868227109
poly 4 3 0.1 6 : 0.8222937333332485, 0.8850127068136281
poly 4 3 0.3 6 : 0.8196919887845336, 0.8821137681192774
poly 4 3 0.5 6 : 0.81693206105182, 0.8789976410358497
poly 4 3 0.7 6 : 0.8140797902711941, 0.8758769685144455
poly 4 3 0.9 6 : 0.8110948295219298, 0.8726827449406043
poly 4 4 0.1 6 : 0.8219823226561702, 0.8847066117989126
poly 4 4 0.3 6 : 0.8193784001716932, 0.8818068972631784
poly 4 4 0.5 6 : 0.8166184150770857, 0.8786880746302521
poly 4 4 0.7 6 : 0.8137663088133046, 0.8755661035310441
poly 4 4 0.9 6 : 0.8107806675189533, 0.8723718330388999
poly 4 5 0.1 6 : 0.8217950178570707, 0.8845219163446598
poly 4 5 0.3 6 : 0.8191900984734101, 0.8816214831960318
poly 4 5 0.5 6 : 0.8164298032227555, 0.8785012497392761
poly 4 5 0.7 6 : 0.8135771969425403, 0.8753795972604824
poly 4 5 0.9 6 : 0.8105905629066541, 0.8721849049343458
poly 4 6 0.1 6 : 0.8216698809019478, 0.8843979708761897
poly 4 6 0.3 6 : 0.8190636894292957, 0.8814970435319643
poly 4 6 0.5 6 : 0.8163040451315118, 0.8783761202642251
poly 4 6 0.7 6 : 0.813450512985513, 0.8752545010959452
poly 4 6 0.9 6 : 0.8104651875970139, 0.8720603272619807
rbf 1 1 0.1 6 : -0.027361185480199522, 0.0015656200748469518
rbf 1 1 0.3 6 : -0.027107086480153964, 0.001534249631325979
rbf 1 1 0.5 6 : -0.026502380982870565, 0.0014641151670391483
rbf 1 1 0.7 6 : -0.025708111449294302, 0.0014170567902724862
rbf 1 1 0.9 6 : -0.024450268058890324, 0.0013861379888290282
rbf 1 2 0.1 6 : -0.027373456784752247, 0.0015652595099064914
rbf 1 2 0.3 6 : -0.02711935088421149, 0.001533889088634271
rbf 1 2 0.5 6 : -0.02651459557214304, 0.0014637539578753467
rbf 1 2 0.7 6 : -0.025720261553581647, 0.0014166953959140471
rbf 1 2 0.9 6 : -0.024462339711972935, 0.0013857763865200434
rbf 1 3 0.1 6 : -0.027373670059248224, 0.0015652593890600475
rbf 1 3 0.3 6 : -0.027119564036458144, 0.0015338889679324952
rbf 1 3 0.5 6 : -0.026514807793774065, 0.001463753836770998
rbf 1 3 0.7 6 : -0.025720472583065, 0.0014166952747045602
rbf 1 3 0.9 6 : -0.024462549303050852, 0.0013857762652449424
rbf 1 4 0.1 6 : -0.027373673960915522, 0.0015652593890194133
rbf 1 4 0.3 6 : -0.027119567935892118, 0.001533888967891972
rbf 1 4 0.5 6 : -0.026514811676162254, 0.0014637538367301417
rbf 1 4 0.7 6 : -0.0257204764436187, 0.001416695274663704
rbf 1 4 0.9 6 : -0.02446255313725145, 0.0013857762652040861
rbf 1 5 0.1 6 : -0.027373674032374184, 0.0015652593890191913
rbf 1 5 0.3 6 : -0.027119568007309924, 0.00153388896789175
rbf 1 5 0.5 6 : -0.026514811747267684, 0.0014637538367301417
rbf 1 5 0.7 6 : -0.025720476514324363, 0.001416695274663704
rbf 1 5 0.9 6 : -0.02446255320747439, 0.0013857762652040861
rbf 1 6 0.1 6 : -0.027373674033683005, 0.0015652593890191913
rbf 1 6 0.3 6 : -0.027119568008617943, 0.00153388896789175
rbf 1 6 0.5 6 : -0.026514811748570066, 0.0014637538367301417
rbf 1 6 0.7 6 : -0.025720476515619418, 0.001416695274663704
rbf 1 6 0.9 6 : -0.024462553208760562, 0.0013857762652040861
rbf 2 1 0.1 6 : -0.02470302482151303, 0.0030576236247074062
rbf 2 1 0.3 6 : -0.02401552587083473, 0.002982270066922088
rbf 2 1 0.5 6 : -0.023335818229143303, 0.002882525293136151
rbf 2 1 0.7 6 : -0.02252868275345663, 0.002763718585641506
rbf 2 1 0.9 6 : -0.021783062977180866, 0.002638027128444631
rbf 2 2 0.1 6 : -0.024727261789248178, 0.0030569011244697686
rbf 2 2 0.3 6 : -0.02403969575648741, 0.0029815474608717585
rbf 2 2 0.5 6 : -0.023359944600415926, 0.002881802227263086
rbf 2 2 0.7 6 : -0.022552756310951595, 0.002762994916109207
rbf 2 2 0.9 6 : -0.021807087477847542, 0.0026373020969681082
rbf 2 3 0.1 6 : -0.02472768314619036, 0.0030569008819504306
rbf 2 3 0.3 6 : -0.024040115887567028, 0.0029815472180722002
rbf 2 3 0.5 6 : -0.0233603639099353, 0.002881801984119914
rbf 2 3 0.7 6 : -0.022553174642856798, 0.002762994672589114

rbf 2 3 0.9 6 : -0.0218074660596451, 0.0026373018530760906
rbf 2 4 0.1 6 : -0.02472769085456634, 0.0030569008818687182
rbf 2 4 0.3 6 : -0.024040123573491147, 0.002981547217990488
rbf 2 4 0.5 6 : -0.02336037158080999, 0.0028818019840379794
rbf 2 4 0.7 6 : -0.022553182295811824, 0.0027629946725072907
rbf 2 4 0.9 6 : -0.021807473696447355, 0.002637301852993823
rbf 2 5 0.1 6 : -0.02472769099574439, 0.0030569008818687182
rbf 2 5 0.3 6 : -0.024040123714258056, 0.002981547217990266
rbf 2 5 0.5 6 : -0.023360371721301298, 0.0028818019840379794
rbf 2 5 0.7 6 : -0.02255318243597495, 0.0027629946725071797
rbf 2 5 0.9 6 : -0.021807473836314494, 0.002637301852993823
rbf 2 6 0.1 6 : -0.024727690998330186, 0.0030569008818687182
rbf 2 6 0.3 6 : -0.02404012371683617, 0.002981547217990266
rbf 2 6 0.5 6 : -0.023360371723874396, 0.0028818019840379794
rbf 2 6 0.7 6 : -0.022553182438542096, 0.0027629946725071797
rbf 2 6 0.9 6 : -0.021807473838876268, 0.002637301852993823
rbf 3 1 0.1 6 : -0.0218632869686596, 0.004172543030019904
rbf 3 1 0.3 6 : -0.021107669529722005, 0.004065462952045462
rbf 3 1 0.5 6 : -0.020209304680771735, 0.003917104410684047
rbf 3 1 0.7 6 : -0.01915910672839378, 0.003665831617341797
rbf 3 1 0.9 6 : -0.018073049484420612, 0.003348088121566839
rbf 3 2 0.1 6 : -0.021899474447146617, 0.0041714535908613515
rbf 3 2 0.3 6 : -0.021143730712387045, 0.004064371962761659
rbf 3 2 0.5 6 : -0.020245304503964424, 0.003916012081261244
rbf 3 2 0.7 6 : -0.019195042147035137, 0.0036647367784851115
rbf 3 2 0.9 6 : -0.018108931408870044, 0.0033469901482838793
rbf 3 3 0.1 6 : -0.021900090774547732, 0.004171453224885213
rbf 3 3 0.3 6 : -0.021144358563062005, 0.004064371596356975
rbf 3 3 0.5 6 : -0.020245898265220184, 0.003916011714300227
rbf 3 3 0.7 6 : -0.0191956879390065, 0.0036647364106497937
rbf 3 3 0.9 6 : -0.018109555037929725, 0.0033469897794433656
rbf 3 4 0.1 6 : -0.02190010228684871, 0.0041714532247619784
rbf 3 4 0.3 6 : -0.02114437004907903, 0.00406437159623374
rbf 3 4 0.5 6 : -0.020245909720003796, 0.003916011714176548
rbf 3 4 0.7 6 : -0.019195699366862273, 0.003664736410526115
rbf 3 4 0.9 6 : -0.01810956644647064, 0.0033469897793192427
rbf 3 5 0.1 6 : -0.021900102497695383, 0.004171453224761756
rbf 3 5 0.3 6 : -0.021144370259444135, 0.00406437159623374
rbf 3 5 0.5 6 : -0.02024590992979678, 0.003916011714176548
rbf 3 5 0.7 6 : -0.019195699576162007, 0.003664736410526115
rbf 3 5 0.9 6 : -0.01810956665541652, 0.0033469897793192427
rbf 3 6 0.1 6 : -0.021900102501557227, 0.004171453224761756
rbf 3 6 0.3 6 : -0.02114437026329701, 0.00406437159623374
rbf 3 6 0.5 6 : -0.020245909933639304, 0.003916011714176548
rbf 3 6 0.7 6 : -0.019195699579995472, 0.003664736410526115
rbf 3 6 0.9 6 : -0.01810956665924346, 0.0033469897793192427
rbf 4 1 0.1 6 : -0.01796596458769919, 0.004590570526344706
rbf 4 1 0.3 6 : -0.016919881457998054, 0.004159007059541864
rbf 4 1 0.5 6 : -0.01588706636604047, 0.0037004837033469684
rbf 4 1 0.7 6 : -0.014938398105271889, 0.003220073346200114
rbf 4 1 0.9 6 : -0.014005200574639299, 0.0026153659623878722
rbf 4 2 0.1 6 : -0.018014002214815195, 0.004589100650704614
rbf 4 2 0.3 6 : -0.016967958745444146, 0.004157531685572091
rbf 4 2 0.5 6 : -0.015935099612088964, 0.0036990039861698465
rbf 4 2 0.7 6 : -0.014986375406067554, 0.003218662820780538
rbf 4 2 0.9 6 : -0.014053212194342435, 0.0026139580412026797
rbf 4 3 0.1 6 : -0.01801483728749993, 0.004589100158078119
rbf 4 3 0.3 6 : -0.01696876381329382, 0.004157531191405162
rbf 4 3 0.5 6 : -0.015935911374417345, 0.0036990034905078906
rbf 4 3 0.7 6 : -0.014987222849900306, 0.0032186623486428756
rbf 4 3 0.9 6 : -0.0140540218208695, 0.002613957570565817
rbf 4 4 0.1 6 : -0.018014852564053153, 0.0045891001579124735
rbf 4 4 0.3 6 : -0.016968779073769903, 0.00415753119123885
rbf 4 4 0.5 6 : -0.01593592662085728, 0.003699003490341024
rbf 4 4 0.7 6 : -0.014987202457093085, 0.0032186623484838917
rbf 4 4 0.9 6 : -0.014054037058145008, 0.00261395757040761
rbf 4 5 0.1 6 : -0.018014852843841078, 0.0045891001579123625
rbf 4 5 0.3 6 : -0.016968779353263175, 0.00415753119123885
rbf 4 5 0.5 6 : -0.015935926900093288, 0.003699003490341024
rbf 4 5 0.7 6 : -0.014987202736149997, 0.0032186623484837806
rbf 4 5 0.9 6 : -0.014054037337212999, 0.00261395757040761
rbf 4 6 0.1 6 : -0.018014852848965646, 0.0045891001579123625
rbf 4 6 0.3 6 : -0.016968779358382237, 0.00415753119123885
rbf 4 6 0.5 6 : -0.015935926905207686, 0.003699003490341024
rbf 4 6 0.7 6 : -0.014987202741261151, 0.0032186623484837806
rbf 4 6 0.9 6 : -0.014054037342324332, 0.00261395757040761
sigmoid 1 1 0.1 6 : -0.027851629798317214, -
0.0004816896164898665
sigmoid 1 1 0.3 6 : -0.028038493455110735, -
0.00015268313984639903
sigmoid 1 1 0.5 6 : -0.02932752177807143, -8.39050488989912e-05
sigmoid 1 1 0.7 6 : -0.027370689812752903, -
0.00015270189372440512
sigmoid 1 1 0.9 6 : -0.027425784338072034, -
0.00035155537022624195
sigmoid 1 2 0.1 6 : -0.02785157494388204, -
0.00048175891811008675
sigmoid 1 2 0.3 6 : -0.028038637424220126, -
0.0001527348651340077
sigmoid 1 2 0.5 6 : -0.02932768292898431, -8.393840775977601e-05
sigmoid 1 2 0.7 6 : -0.02737078105492676, -
0.00015273486512534795
sigmoid 1 2 0.9 6 : -0.027425939281486667, -
0.0003516451203253723
sigmoid 1 3 0.1 6 : -0.02785157494475108, -
0.0004817589182650739
sigmoid 1 3 0.3 6 : -0.028038637425086766, -
0.0001527348652718974
sigmoid 1 3 0.5 6 : -0.029327682929847977, -8.39384078961114e-05
sigmoid 1 3 0.7 6 : -0.027370781055785543, -
0.0001527348652718974
sigmoid 1 3 0.9 6 : -0.02742593928234638, -
0.0003516451204561566
sigmoid 1 4 0.1 6 : -0.02785157494475108, -
0.0004817589182650739
sigmoid 1 4 0.3 6 : -0.028038637425086766, -
0.0001527348652718974
sigmoid 1 4 0.5 6 : -0.029327682929847977, -8.39384078961114e-05
sigmoid 1 4 0.7 6 : -0.027370781055785543, -
0.0001527348652718974
sigmoid 1 4 0.9 6 : -0.02742593928234638, -
0.0003516451204561566
sigmoid 1 5 0.1 6 : -0.02785157494475108, -
0.0004817589182650739
sigmoid 1 5 0.3 6 : -0.028038637425086766, -
0.0001527348652718974
sigmoid 1 5 0.5 6 : -0.029327682929847977, -8.39384078961114e-05
sigmoid 1 5 0.7 6 : -0.027370781055785543, -
0.0001527348652718974
sigmoid 1 5 0.9 6 : -0.02742593928234638, -
0.0003516451204561566
sigmoid 1 6 0.1 6 : -0.02785157494475108, -
0.0004817589182650739
sigmoid 1 6 0.3 6 : -0.028038637425086766, -
0.0001527348652718974
sigmoid 1 6 0.5 6 : -0.029327682929847977, -8.39384078961114e-05
sigmoid 1 6 0.7 6 : -0.027370781055785543, -
0.0001527348652718974
sigmoid 1 6 0.9 6 : -0.02742593928234638, -
0.0003516451204561566
sigmoid 2 1 0.1 6 : -0.02785168465395995, -
0.0004816203167767874
sigmoid 2 1 0.3 6 : -0.02803834948638313, -
0.00015263141643240274
sigmoid 2 1 0.5 6 : -0.02932736062739103, -8.387169079315804e-05
sigmoid 2 1 0.7 6 : -0.027370598571331416, -
0.00015266892306553537
sigmoid 2 1 0.9 6 : -0.027425629394853733, -
0.00035146562562760053
sigmoid 2 2 0.1 6 : -0.027851574943013, -0.00048175891795532166
sigmoid 2 2 0.3 6 : -0.02803863742335344, -
0.00015273486499634004
sigmoid 2 2 0.5 6 : -0.029327682928120602, -8.393840762344063e-
05
sigmoid 2 2 0.7 6 : -0.027370781054068028, -
0.0001527348649787985
sigmoid 2 2 0.9 6 : -0.027425939280626865, -
0.000351645120194366

sigmoid 2 3 0.1 6 : -0.02785157494475108, -
0.0004817589182650739
sigmoid 2 3 0.3 6 : -0.028038637425086766, -
0.0001527348652718974
sigmoid 2 3 0.5 6 : -0.029327682929847977, -8.39384078961114e-05
sigmoid 2 3 0.7 6 : -0.027370781055785543, -
0.0001527348652718974
sigmoid 2 3 0.9 6 : -0.02742593928234638, -
0.0003516451204561566
sigmoid 2 4 0.1 6 : -0.02785157494475108, -
0.0004817589182650739
sigmoid 2 4 0.3 6 : -0.028038637425086766, -
0.0001527348652718974
sigmoid 2 4 0.5 6 : -0.029327682929847977, -8.39384078961114e-05
sigmoid 2 4 0.7 6 : -0.027370781055785543, -
0.0001527348652718974
sigmoid 2 4 0.9 6 : -0.02742593928234638, -
0.0003516451204561566
sigmoid 2 5 0.1 6 : -0.02785157494475108, -
0.0004817589182650739
sigmoid 2 5 0.3 6 : -0.028038637425086766, -
0.0001527348652718974
sigmoid 2 5 0.5 6 : -0.029327682929847977, -8.39384078961114e-05
sigmoid 2 5 0.7 6 : -0.027370781055785543, -
0.0001527348652718974
sigmoid 2 5 0.9 6 : -0.02742593928234638, -
0.0003516451204561566
sigmoid 2 6 0.1 6 : -0.02785157494475108, -
0.0004817589182650739
sigmoid 2 6 0.3 6 : -0.028038637425086766, -
0.0001527348652718974
sigmoid 2 6 0.5 6 : -0.029327682929847977, -8.39384078961114e-05
sigmoid 2 6 0.7 6 : -0.027370781055785543, -
0.0001527348652718974
sigmoid 2 6 0.9 6 : -0.02742593928234638, -
0.0003516451204561566
sigmoid 3 1 0.1 6 : -0.027851739511678762, -
0.0004815510191251704
sigmoid 3 1 0.3 6 : -0.028038205518903105, -
0.00015257969503013058
sigmoid 3 1 0.5 6 : -0.029327199477806286, -8.383833357883397e-
05
sigmoid 3 1 0.7 6 : -0.02737050733152078, -
0.00015263595329484403
sigmoid 3 1 0.9 6 : -0.02742547445269068, -
0.0003513758866602323 4
sigmoid 3 2 0.1 6 : -0.02785157494214414, -
0.0004817589178005566
sigmoid 3 2 0.3 6 : -0.028038637422486846, -
0.00015273486485845034
sigmoid 3 2 0.5 6 : -0.02932768292725698, -8.393840748732728e-05
sigmoid 3 2 0.7 6 : -0.02737078105320965, -
0.00015273486483224907
sigmoid 3 2 0.9 6 : -0.027425939279767463, -
0.0003516451200633597
sigmoid 3 3 0.1 6 : -0.02785157494475108, -
0.0004817589182650739
sigmoid 3 3 0.3 6 : -0.028038637425086766, -
0.0001527348652718974
sigmoid 3 3 0.5 6 : -0.029327682929847977, -8.39384078961114e-05
sigmoid 3 3 0.7 6 : -0.027370781055785543, -
0.0001527348652718974
sigmoid 3 3 0.9 6 : -0.02742593928234638, -
0.0003516451204561566
sigmoid 3 4 0.1 6 : -0.02785157494475108, -
0.0004817589182650739
sigmoid 3 4 0.3 6 : -0.028038637425086766, -
0.0001527348652718974
sigmoid 3 4 0.5 6 : -0.029327682929847977, -8.39384078961114e-05
sigmoid 3 4 0.7 6 : -0.027370781055785543, -
0.0001527348652718974
sigmoid 3 4 0.9 6 : -0.02742593928234638, -
0.0003516451204561566
sigmoid 3 5 0.1 6 : -0.02785157494475108, -
0.0004817589182650739
sigmoid 3 5 0.3 6 : -0.028038637425086766, -
0.0001527348652718974
sigmoid 3 5 0.5 6 : -0.029327682929847977, -8.39384078961114e-05
sigmoid 3 5 0.7 6 : -0.027370781055785543, -
0.0001527348652718974
sigmoid 3 5 0.9 6 : -0.02742593928234638, -
0.0003516451204561566
sigmoid 3 6 0.1 6 : -0.02785157494475108, -
0.0004817589182650739
sigmoid 3 6 0.3 6 : -0.028038637425086766, -
0.0001527348652718974
sigmoid 3 6 0.5 6 : -0.029327682929847977, -8.39384078961114e-05
sigmoid 3 6 0.7 6 : -0.027370781055785543, -
0.0001527348652718974
sigmoid 3 6 0.9 6 : -0.02742593928234638, -
0.0003516451204561566
sigmoid 4 1 0.1 6 : -0.027851794371474982, -
0.0004814817235365698
sigmoid 4 1 0.3 6 : -0.028038061552671946, -
0.00015252797563980458
sigmoid 4 1 0.5 6 : -0.029327038329318177, -8.380497725624103e-
05
sigmoid 4 1 0.7 6 : -0.027370416093322313, -
0.0001526029844127752
sigmoid 4 1 0.9 6 : -0.02742531951158433 4, -
0.0003512861533243594
sigmoid 4 2 0.1 6 : -0.02785157494127506, -
0.00048175891764556944
sigmoid 4 2 0.3 6 : -0.028038637421620206, -
0.00015273486472078268
sigmoid 4 2 0.5 6 : -0.029327682926393227, -8.393840735121394e-
05
sigmoid 4 2 0.7 6 : -0.02737078105235069, -
0.00015273486468569963
sigmoid 4 2 0.9 6 : -0.027425939278907574, -
0.00035164511993235337
sigmoid 4 3 0.1 6 : -0.02785157494475108, -
0.0004817589182650739
sigmoid 4 3 0.3 6 : -0.028038637425086766, -
0.0001527348652718974
sigmoid 4 3 0.5 6 : -0.029327682929847977, -8.39384078961114e-05
sigmoid 4 3 0.7 6 : -0.027370781055785543, -
0.0001527348652718974
sigmoid 4 3 0.9 6 : -0.02742593928234638, -
0.0003516451204561566
sigmoid 4 4 0.1 6 : -0.02785157494475108, -
0.0004817589182650739
sigmoid 4 4 0.3 6 : -0.028038637425086766, -
0.0001527348652718974
sigmoid 4 4 0.5 6 : -0.029327682929847977, -8.39384078961114e-05
sigmoid 4 4 0.7 6 : -0.027370781055785543, -
0.0001527348652718974
sigmoid 4 4 0.9 6 : -0.02742593928234638, -
0.0003516451204561566
sigmoid 4 5 0.1 6 : -0.02785157494475108, -
0.0004817589182650739
sigmoid 4 5 0.3 6 : -0.028038637425086766, -
0.0001527348652718974
sigmoid 4 5 0.5 6 : -0.029327682929847977, -8.39384078961114e-05
sigmoid 4 5 0.7 6 : -0.027370781055785543, -
0.0001527348652718974
sigmoid 4 5 0.9 6 : -0.02742593928234638, -
0.0003516451204561566
sigmoid 4 6 0.1 6 : -0.02785157494475108, -
0.0004817589182650739
sigmoid 4 6 0.3 6 : -0.028038637425086766, -
0.0001527348652718974
sigmoid 4 6 0.5 6 : -0.029327682929847977, -8.39384078961114e-05
sigmoid 4 6 0.7 6 : -0.027370781055785543, -
0.0001527348652718974
sigmoid 4 6 0.9 6 : -0.02742593928234638, -
0.0003516451204561566

Grid search of ElasticNet, Morgan(r=3)
ElasticNet(alpha= i, l1_ratio= j, max_iter=100000)
i j : accuracy of prediction using cross validation, accuracy of prediction using test data
0.001 0.0 : 0.8932060206953019, 0.9395137886924076
0.001 0.2 : 0.8935652476341698, 0.9447667507717836
0.001 0.4 : 0.8939464249289444, 0.948929349094488
0.001 0.6 : 0.8947038597523542, 0.9531143124509337
0.001 0.8 : 0.8970983338468133, 0.9545627195930549
0.001 1.0 : 0.8998195509240563, 0.9381824584441755
0.01 0.0 : 0.8815096664270738, 0.9256591324818334
0.01 0.2 : 0.8816862588839374, 0.9298150593357114
0.01 0.4 : 0.8830885015074268, 0.9332297868480401
0.01 0.6 : 0.8862578268438084, 0.9367463592656516
0.01 0.8 : 0.8903940149445256, 0.9375976069715511
0.01 1.0 : 0.8919859799036489, 0.9234980950839158
0.1 0.0 : 0.7966248034488657, 0.8286728067183181
0.1 0.2 : 0.7870560112999947, 0.8189121408282474
0.1 0.4 : 0.7817264032906921, 0.8119333116344501
0.1 0.6 : 0.7827186092059033, 0.8105732782545237
0.1 0.8 : 0.7913240414335696, 0.8136987373449783
0.1 1.0 : 0.8137439115064492, 0.827756570432095

Grid search of RandomForest, Morgan(r=3)
RandomForestRegressor(n_estimators= i)
i: accuracy of prediction using cross validation, accuracy of prediction using test data
100 1.0 : 0.7728589788887733, 0.8396542723737799
500 1.0 : 0.7722612266271465, 0.8337212680838879
1000 1.0 : 0.7725439923772381, 0.8398505906516754
2000 1.0 : 0.7723019474560914, 0.8364588558586676

Grid search of NeuralNetwork, Morgan(r=3)
MLPRegressor(activation=act, alpha=a, batch_size=batch, beta_1=0.9,
beta_2=0.999, early_stopping=False, epsilon=1e-08,
hidden_layer_sizes=hid, learning_rate='constant',
learning_rate_init=0.001, max_iter=100000, momentum=0.9,
n_iter_no_change=10, nesterovs_momentum=True,
power_t=0.5,
random_state=1, shuffle=True, solver='adam', tol=0.0001,
validation_fraction=0.1, verbose=False, warm_start=False)
act (hid) a batch: accuracy of prediction using cross validation, accuracy of prediction using test data
relu (200,) 0.0001 500 : 0.8089442345501506, 0.9104184092834429
relu (200,) 0.0001 200 : 0.8361999903510245, 0.9052483696835272
relu (200,) 0.0001 100 : 0.8372661610514414, 0.9068072984681104
relu (200,) 0.0001 50 : 0.8429309562791175, 0.9105155545480297
relu (200,) 0.001 500 : 0.8088962234572833, 0.9106392692193611
relu (200,) 0.001 200 : 0.836298545419303, 0.9054918192127358
relu (200,) 0.001 100 : 0.8374996415359417, 0.90697779959818
relu (200,) 0.001 50 : 0.8428114498660755, 0.9108292038123276
relu (200,) 0.01 500 : 0.8091966501220158, 0.9119520448946926
relu (200,) 0.01 200 : 0.8374768526734178, 0.9063812502903191
relu (200,) 0.01 100 : 0.8392116606698098, 0.9088378133701687
relu (200,) 0.01 50 : 0.8471301745298243, 0.9144954083655985
relu (200, 200) 0.0001 500 : 0.8055726680136951,
0.9080967372514874
relu (200, 200) 0.0001 200 : 0.8331180675625763,
0.9015146591983008
relu (200, 200) 0.0001 100 : 0.8340575340945101,
0.9039276554147353
relu (200, 200) 0.0001 50 : 0.8406058657859411,
0.9096537241278997
relu (200, 200) 0.001 500 : 0.8062857830536825,
0.9079018228718965
relu (200, 200) 0.001 200 : 0.8334878315912377,
0.8992583798626759
relu (200, 200) 0.001 100 : 0.8331485541732476,
0.9033618119581382
relu (200, 200) 0.001 50 : 0.8411515592095234,
0.9091410772468915
relu (200, 200) 0.01 500 : 0.8054413536959117,
0.9081600693430746
relu (200, 200) 0.01 200 : 0.8330926626706228,
0.9009504480703405
relu (200, 200) 0.01 100 : 0.8345255387947075, 0.902673530831824
relu (200, 200) 0.01 50 : 0.841439613528269, 0.9087600986417866
relu (100,) 0.0001 500 : 0.8064820913622226, 0.913722871279751
relu (100,) 0.0001 200 : 0.8349840522543402, 0.9039402048205367
relu (100,) 0.0001 100 : 0.838043836362073, 0.9059613803147961
relu (100,) 0.0001 50 : 0.8428662151566618, 0.9100467934156079
relu (100,) 0.001 500 : 0.8065065029188789, 0.9138487059260881
relu (100,) 0.001 200 : 0.8351124362007043, 0.9040510926464269
relu (100,) 0.001 100 : 0.8381653850595093, 0.9062453337952786
relu (100,) 0.001 50 : 0.8428958297818065, 0.9103679981002573
relu (100,) 0.01 500 : 0.8068124835277615, 0.9149201655548201
relu (100,) 0.01 200 : 0.8363805195316347, 0.9050766188898226
relu (100,) 0.01 100 : 0.8382482140978329, 0.9086800707116031
relu (100,) 0.01 50 : 0.8466555534750204, 0.9124912309726154
relu (100, 100) 0.0001 500 : 0.8032451105358487,
0.9101987278473092
relu (100, 100) 0.0001 200 : 0.8335465974016467,
0.9023925224374106
relu (100, 100) 0.0001 100 : 0.8374382967511261,
0.9098070040800502
relu (100, 100) 0.0001 50 : 0.8427687105929026,
0.9114593276842253
relu (100, 100) 0.001 500 : 0.8036677722060815,
0.9118682655154394
relu (100, 100) 0.001 200 : 0.8331061642376735,
0.9023724508433958
relu (100, 100) 0.001 100 : 0.8367172603379217,
0.9118739755832589
relu (100, 100) 0.001 50 : 0.8424852269764032,
0.9114770514510414
relu (100, 100) 0.01 500 : 0.8038624514147227,
0.9133655146447702
relu (100, 100) 0.01 200 : 0.8339117783014294,
0.9015531270218685
relu (100, 100) 0.01 100 : 0.8369395680406674,
0.9121312916414381
relu (100, 100) 0.01 50 : 0.8417938328042297, 0.9128557979814265
relu (100, 200) 0.0001 500 : 0.8060543411285929,
0.9037890729437514
relu (100, 200) 0.0001 200 : 0.8333309302113836,
0.8972359446632783
relu (100, 200) 0.0001 100 : 0.836690169803453,
0.9045139223616964
relu (100, 200) 0.0001 50 : 0.8419030297501449,
0.9094220126174812
relu (100, 200) 0.001 500 : 0.8062338884293982,
0.905318693384775
relu (100, 200) 0.001 200 : 0.8348942450904385,
0.8970670056102056
relu (100, 200) 0.001 100 : 0.8367254509843587,
0.9053740262447894
relu (100, 200) 0.001 50 : 0.844344492102325, 0.9131915929435871
relu (100, 200) 0.01 500 : 0.8061441463987468,
0.9043604291936728
relu (100, 200) 0.01 200 : 0.8360202292839272,
0.8989928121403611
relu (100, 200) 0.01 100 : 0.8357206948986049,
0.9057297446670034
relu (100, 200) 0.01 50 : 0.8427913054146746, 0.9162317310986614
relu (50,) 0.0001 500 : 0.8068686251644921, 0.9141995657992223
relu (50,) 0.0001 200 : 0.8359206477419596, 0.9077219873318102
relu (50,) 0.0001 100 : 0.8360010449677737, 0.9071147237997331
relu (50,) 0.0001 50 : 0.8404546642316342, 0.9099931192124388
relu (50,) 0.001 500 : 0.8069001465504921, 0.9142883352439017
relu (50,) 0.001 200 : 0.8360220928036309, 0.9077948190706323
relu (50,) 0.001 100 : 0.8361409707934829, 0.9085012377323337
relu (50,) 0.001 50 : 0.8406877580308827, 0.9102828550704654
relu (50,) 0.01 500 : 0.8071439624390402, 0.9150972417538803
relu (50,) 0.01 200 : 0.8373069986890311, 0.9086023032444911
relu (50,) 0.01 100 : 0.8373684735047568, 0.9091523881289757
relu (50,) 0.01 50 : 0.8432756313745655, 0.9131319665423062

relu (50, 50) 0.0001 500 : 0.8079413836967392, 0.9095936107287239
relu (50, 50) 0.0001 200 : 0.840901568722602, 0.8988295332028645
relu (50, 50) 0.0001 100 : 0.8402946330591916, 0.907936048022221
relu (50, 50) 0.0001 50 : 0.8449374823393218, 0.9114804395789944
relu (50, 50) 0.001 500 : 0.8102091210688336, 0.9079126701237213
relu (50, 50) 0.001 200 : 0.840856527642261, 0.8987289741606194
relu (50, 50) 0.001 100 : 0.8408264500824651, 0.9072235408544957
relu (50, 50) 0.001 50 : 0.8427000772032113, 0.9131828240097708
relu (50, 50) 0.01 500 : 0.808627658071536, 0.9082909819264738
relu (50, 50) 0.01 200 : 0.8406413117485437, 0.8974989980693615
relu (50, 50) 0.01 100 : 0.8403104350590589, 0.9078865742261649
relu (50, 50) 0.01 50 : 0.8441679010681584, 0.9091536508234337
relu (50, 100) 0.0001 500 : 0.8051313574700508, 0.9000294816703865
relu (50, 100) 0.0001 200 : 0.8337548329359621, 0.8902483538767677
relu (50, 100) 0.0001 100 : 0.8347913647132554, 0.9032509162233514
relu (50, 100) 0.0001 50 : 0.8414743853038086, 0.9045366128094143
relu (50, 100) 0.001 500 : 0.8054318201821411, 0.901916024279334
relu (50, 100) 0.001 200 : 0.833947343769833, 0.8961056838179797
relu (50, 100) 0.001 100 : 0.8347709946748696, 0.904770216454311
relu (50, 100) 0.001 50 : 0.8408265080069445, 0.9040213798818888
relu (50, 100) 0.01 500 : 0.8058700408371016, 0.9034146534154213
relu (50, 100) 0.01 200 : 0.8329729882626957, 0.897918855550676
relu (50, 100) 0.01 100 : 0.8346485645183618, 0.9030428089909522
relu (50, 100) 0.01 50 : 0.8409415224809479, 0.9013768511779917
relu (50, 200) 0.0001 500 : 0.8050737750167765, 0.9027977650376731
relu (50, 200) 0.0001 200 : 0.8333226264299654, 0.8942657419414812
relu (50, 200) 0.0001 100 : 0.8392729040919326, 0.9011689263213247
relu (50, 200) 0.0001 50 : 0.8435371988675039, 0.9041278409395038
relu (50, 200) 0.001 500 : 0.8044809132055117, 0.8981634697272509
relu (50, 200) 0.001 200 : 0.8332086162198065, 0.8958590395335397
relu (50, 200) 0.001 100 : 0.8373568920128033, 0.9002287967401889
relu (50, 200) 0.001 50 : 0.8448191127639483, 0.9048951737201498
relu (50, 200) 0.01 500 : 0.805934404722022, 0.9023981288257654
relu (50, 200) 0.01 200 : 0.834737508886807, 0.8919029842636321
relu (50, 200) 0.01 100 : 0.8381699008626196, 0.9010616738633541
relu (50, 200) 0.01 50 : 0.8439889041742576, 0.9067955063345225
tanh (200,) 0.0001 500 : 0.8171944655994572, 0.8935081471482709
tanh (200,) 0.0001 200 : 0.8451740252517161, 0.8893289152317485
tanh (200,) 0.0001 100 : 0.8458854721949999, 0.8955244821431222
tanh (200,) 0.0001 50 : 0.8520473670814639, 0.8991001002672425
tanh (200,) 0.001 500 : 0.8177809539788102, 0.8943385238505897
tanh (200,) 0.001 200 : 0.8462922462927509, 0.8904832068676093
tanh (200,) 0.001 100 : 0.8467102661372692, 0.8966150082559698
tanh (200,) 0.001 50 : 0.852703943162596, 0.8999848488013756
tanh (200,) 0.01 500 : 0.8231263678993483, 0.9011348061786189
tanh (200,) 0.01 200 : 0.8540147413195729, 0.9037258918362392
tanh (200,) 0.01 100 : 0.8546527706847268, 0.9039844004883315
tanh (200,) 0.01 50 : 0.8570280335405259, 0.9063663283057022
tanh (200, 200) 0.0001 500 : 0.8070749173583518, -0.020816323433473283
tanh (200, 200) 0.0001 200 : 0.6574585659522173, 0.9001847040965719
tanh (200, 200) 0.0001 100 : 0.8438301061440356, 0.903455333720123
tanh (200, 200) 0.0001 50 : 0.8497934445355684, 0.9119864696659692
tanh (200, 200) 0.001 500 : 0.8082904214571262, -0.020816007275319404
tanh (200, 200) 0.001 200 : 0.657523110551461, 0.8975051791940475
tanh (200, 200) 0.001 100 : 0.8453748100535933, 0.9042701672448374
tanh (200, 200) 0.001 50 : 0.8502303078297025, 0.911553222235086
tanh (200, 200) 0.01 500 : 0.818753565205494, -0.02081246905745293
tanh (200, 200) 0.01 200 : 0.6638658690542324, 0.9010681445391486
tanh (200, 200) 0.01 100 : 0.8506080421013997, 0.908412368429814
tanh (200, 200) 0.01 50 : 0.8573331078918651, 0.9139429952398833
tanh (100,) 0.0001 500 : 0.80277774474217, 0.9040103980428824
tanh (100,) 0.0001 200 : 0.8368604136544681, 0.8927714297995141
tanh (100,) 0.0001 100 : 0.8382915826566366, 0.901138592540144
tanh (100,) 0.0001 50 : 0.8488798632517248, 0.9055161887592118
tanh (100,) 0.001 500 : 0.8041458230070029, 0.9041327935872783
tanh (100,) 0.001 200 : 0.8381123078857758, 0.895216682385043
tanh (100,) 0.001 100 : 0.839136043389319, 0.9025707470647246
tanh (100,) 0.001 50 : 0.8493279196307564, 0.9079512382101789
tanh (100,) 0.01 500 : 0.8154379935854514, 0.9063827713407642
tanh (100,) 0.01 200 : 0.8466953780977701, 0.9045924509521911
tanh (100,) 0.01 100 : 0.846399657912373, 0.9102746992351586
tanh (100,) 0.01 50 : 0.8557882925894743, 0.9117095595015738
tanh (100, 100) 0.0001 500 : 0.8016538335258778, 0.9058947880499775
tanh (100, 100) 0.0001 200 : 0.8400673568321093, 0.8940426764218629
tanh (100, 100) 0.0001 100 : 0.8442697914492318, 0.9063192403789171
tanh (100, 100) 0.0001 50 : 0.8513382919622586, 0.9115945630754194
tanh (100, 100) 0.001 500 : 0.8052099760360623, 0.9050241310383628
tanh (100, 100) 0.001 200 : 0.8421929165081601, 0.8954620946796288
tanh (100, 100) 0.001 100 : 0.8468148733277472, 0.9063949267945695
tanh (100, 100) 0.001 50 : 0.8483082037708577, 0.9126307140845394
tanh (100, 100) 0.01 500 : 0.8130200904695695, 0.9075635341017096
tanh (100, 100) 0.01 200 : 0.8484596616078525, 0.9023574126680385
tanh (100, 100) 0.01 100 : 0.8493814575375339, 0.9132477120198575
tanh (100, 100) 0.01 50 : 0.861083279496237, 0.9191486302429106
tanh (100, 200) 0.0001 500 : 0.8060435876022695, 0.8923094525285721
tanh (100, 200) 0.0001 200 : 0.8337427467249523, 0.8877850651429533
tanh (100, 200) 0.0001 100 : 0.8408146869930532, 0.9019927221009547
tanh (100, 200) 0.0001 50 : 0.8504000395782862, 0.9111184297271093
tanh (100, 200) 0.001 500 : 0.6470411217360394, 0.8924094121529891
tanh (100, 200) 0.001 200 : 0.8341284947047221, 0.8881572296546223
tanh (100, 200) 0.001 100 : 0.8407647800471476, 0.9021710318144633
tanh (100, 200) 0.001 50 : 0.8519853621069974, 0.9089824299973208
tanh (100, 200) 0.01 500 : 0.6516179150306034, 0.8940519234306537
tanh (100, 200) 0.01 200 : 0.839029520508929, 0.8887874475693143
tanh (100, 200) 0.01 100 : 0.8448342874913488, 0.9045208503283396
tanh (100, 200) 0.01 50 : 0.8528116832447667, 0.9133545659441313
tanh (50,) 0.0001 500 : 0.7850966362906455, 0.8873914751863684
tanh (50,) 0.0001 200 : 0.8220985084218718, 0.8939767445244242
tanh (50,) 0.0001 100 : 0.8274756885633089, 0.8979549193196852
tanh (50,) 0.0001 50 : 0.8367213181458981, 0.8995014157614803
tanh (50,) 0.001 500 : 0.7872472744115309, 0.8879801214942774
tanh (50,) 0.001 200 : 0.8264634595089989, 0.8964579270716672
tanh (50,) 0.001 100 : 0.8285906494195736, 0.8979533058530944
tanh (50,) 0.001 50 : 0.8361178635857021, 0.903705187805548
tanh (50,) 0.01 500 : 0.809004454140309, 0.8994485046303524
tanh (50,) 0.01 200 : 0.8410276391788474, 0.9063328587970798

tanh (50,) 0.01 100 : 0.8421379332193218, 0.9160462326798052
tanh (50,) 0.01 50 : 0.8467378064855089, 0.9110762355774774
tanh (50, 50) 0.0001 500 : 0.7938051047945728, 0.9040977880634045
tanh (50, 50) 0.0001 200 : 0.8306052828605746, 0.8927080959102175
tanh (50, 50) 0.0001 100 : 0.8412922223370316, 0.9021806331381037
tanh (50, 50) 0.0001 50 : 0.8465510786944334, 0.9027708048652927
tanh (50, 50) 0.001 500 : 0.7950929915054674, 0.8954804010987554
tanh (50, 50) 0.001 200 : 0.8309066588636046, 0.8943847216590266
tanh (50, 50) 0.001 100 : 0.8422621145870435, 0.9047076587112977
tanh (50, 50) 0.001 50 : 0.8500668096790601, 0.9060047900745226
tanh (50, 50) 0.01 500 : 0.8094892110154553, 0.9029275227992187
tanh (50, 50) 0.01 200 : 0.8399707149264733, 0.9054412077574384
tanh (50, 50) 0.01 100 : 0.8513652057075494, 0.9123027658393197
tanh (50, 50) 0.01 50 : 0.8571916973358936, 0.9191712705335825
tanh (50, 100) 0.0001 500 : 0.7979997494183931, 0.898034096882549
tanh (50, 100) 0.0001 200 : 0.8401148989171962, 0.8907170726931593
tanh (50, 100) 0.0001 100 : 0.8425839520918512, 0.8976574448619167
tanh (50, 100) 0.0001 50 : 0.8479639605225046, 0.9085050985827334
tanh (50, 100) 0.001 500 : 0.7989139900865684, 0.8968514015618796
tanh (50, 100) 0.001 200 : 0.8410352366072539, 0.8912272436406083
tanh (50, 100) 0.001 100 : 0.842236645759791, 0.9011020768007753
tanh (50, 100) 0.001 50 : 0.8533251601515902, 0.9051364884745531
tanh (50, 100) 0.01 500 : 0.8080180718862164, 0.9023863381751039
tanh (50, 100) 0.01 200 : 0.8450008981247439, 0.8938181558432313
tanh (50, 100) 0.01 100 : 0.8506974933427326, 0.9036821831045807
tanh (50, 100) 0.01 50 : 0.8562792332661596, 0.9079387625775572
tanh (50, 200) 0.0001 500 : 0.8106856797778932, 0.8848595542228023
tanh (50, 200) 0.0001 200 : 0.8459975057349641, 0.8915352910681169
tanh (50, 200) 0.0001 100 : 0.846705285091551, 0.8933374160790176
tanh (50, 200) 0.0001 50 : 0.854453528569197, 0.901936199832786
tanh (50, 200) 0.001 500 : 0.8101825024608929, 0.8850582281755792
tanh (50, 200) 0.001 200 : 0.8454547456792538, 0.8917087131588345
tanh (50, 200) 0.001 100 : 0.8496291308447972, 0.8936473137849397
tanh (50, 200) 0.001 50 : 0.8553379799540979, 0.9019293876110499
tanh (50, 200) 0.01 500 : 0.8125508189722284, 0.8869583763224199
tanh (50, 200) 0.01 200 : 0.8484397271806291, 0.8935365454285569
tanh (50, 200) 0.01 100 : 0.8491535761054309, 0.8998865510717396
tanh (50, 200) 0.01 50 : 0.8575932826741488, 0.903487100360163
logistic (200,) 0.0001 500 : 0.8101325218137466, 0.8971795744300226
logistic (200,) 0.0001 200 : 0.8461484475739169, 0.8893255112182841
logistic (200,) 0.0001 100 : 0.8470347469349655, 0.8968619674869038
logistic (200,) 0.0001 50 : 0.8536029651374626, 0.9035912249816018
logistic (200,) 0.001 500 : 0.8127074040841629, 0.9011113891216211
logistic (200,) 0.001 200 : 0.850229232584024, 0.8936806313202229
logistic (200,) 0.001 100 : 0.8519943861266295, 0.9022369924232415
logistic (200,) 0.001 50 : 0.8578515561071939, 0.9086507105442454
logistic (200,) 0.01 500 : 0.8324057797982821, 0.917585385960756
logistic (200,) 0.01 200 : 0.8694910633225528, 0.92380906400682
logistic (200,) 0.01 100 : 0.8695440491494141, 0.92242728200302
logistic (200,) 0.01 50 : 0.8736181021931817, 0.9249904333751535
logistic (200, 200) 0.0001 500 : 0.7977831473839292, 0.89026917319344
logistic (200, 200) 0.0001 200 : 0.8304276685554711, 0.8813239683574771
logistic (200, 200) 0.0001 100 : 0.8370914779319956, 0.903177649115198
logistic (200, 200) 0.0001 50 : 0.843206412809554, 0.9070106191360219
logistic (200, 200) 0.001 500 : 0.805799760562258, 0.8915400193242585
logistic (200, 200) 0.001 200 : 0.836404128126893, 0.8905956786776649
logistic (200, 200) 0.001 100 : 0.8393962904099503, 0.9058491433269494
logistic (200, 200) 0.001 50 : 0.8545260002718456, 0.9087639340665327
logistic (200, 200) 0.01 500 : 0.8323647110626904, 0.9144051264247383
logistic (200, 200) 0.01 200 : 0.8483364903331939, 0.9074889392088319
logistic (200, 200) 0.01 100 : 0.8565768154639523, 0.9159337184642511
logistic (200, 200) 0.01 50 : 0.8731608936744342, 0.9273879945105137
logistic (100,) 0.0001 500 : 0.7983667969003365, 0.8992374080285419
logistic (100,) 0.0001 200 : 0.8381768974098087, 0.8872222931407023
logistic (100,) 0.0001 100 : 0.8387106561972437, 0.897359129171948
logistic (100,) 0.0001 50 : 0.8463595185689353, 0.9012208669831736
logistic (100,) 0.001 500 : 0.803264929435079, 0.9052544898798174
logistic (100,) 0.001 200 : 0.8467920483813265, 0.89401064765654
logistic (100,) 0.001 100 : 0.8463941467551799, 0.905666826599319
logistic (100,) 0.001 50 : 0.8524558045632886, 0.9079952098864915
logistic (100,) 0.01 500 : 0.8343796090075584, 0.9145829127916819
logistic (100,) 0.01 200 : 0.8639324192710959, 0.9155457830104823
logistic (100,) 0.01 100 : 0.864226364026778, 0.920631747092267
logistic (100,) 0.01 50 : 0.8677006526799165, 0.9262445106225656
logistic (100, 100) 0.0001 500 : 0.8005947131023967, 0.8871632968147571
logistic (100, 100) 0.0001 200 : 0.8243266922010488, 0.8802486302434884
logistic (100, 100) 0.0001 100 : 0.8378978328078153, 0.8917630736818891
logistic (100, 100) 0.0001 50 : 0.8472484886223697, 0.8968989508285617
logistic (100, 100) 0.001 500 : 0.8070558779290277, 0.8869760682767502
logistic (100, 100) 0.001 200 : 0.8243557800626972, 0.886567357483985
logistic (100, 100) 0.001 100 : 0.8388488749558105, 0.9045123954909307
logistic (100, 100) 0.001 50 : 0.8524971608199874, 0.9048073127196186
logistic (100, 100) 0.01 500 : 0.8288407541447651, 0.9054928967860016
logistic (100, 100) 0.01 200 : 0.8408505267887199, 0.8953590887825575
logistic (100, 100) 0.01 100 : 0.8506722578054644, 0.9112348291589881
logistic (100, 100) 0.01 50 : 0.8640165555683037, 0.9152526968363208
logistic (100, 200) 0.0001 500 : 0.7937395192106568, 0.8793857871271117
logistic (100, 200) 0.0001 200 : 0.8154803033818337, 0.8765187623345343
logistic (100, 200) 0.0001 100 : 0.8262001917613502, 0.8878652836604963
logistic (100, 200) 0.0001 50 : 0.8322898531852474, 0.9012085722364986
logistic (100, 200) 0.001 500 : 0.7990242244412056, 0.8811513297609697
logistic (100, 200) 0.001 200 : 0.8193479179898698, 0.8822517522111724
logistic (100, 200) 0.001 100 : 0.8296613584093425,

0.8921042134451774
logistic (100, 200) 0.001 50 : 0.8389082640372036, 0.9041732737560211
logistic (100, 200) 0.01 500 : 0.8236052611061965, 0.8854401062832492
logistic (100, 200) 0.01 200 : 0.833580475261867, 0.8946714386896535
logistic (100, 200) 0.01 100 : 0.8381513484370053, 0.8998526847948527
logistic (100, 200) 0.01 50 : 0.8537706627178464, 0.9095632399542748
logistic (50,) 0.0001 500 : 0.8077984864970003, 0.8941059500679198
logistic (50,) 0.0001 200 : 0.8420159464331587, 0.8978218733961016
logistic (50,) 0.0001 100 : 0.8402065018168867, 0.8960281549801126
logistic (50,) 0.0001 50 : 0.849082757526161, 0.9081319105841056
logistic (50,) 0.001 500 : 0.8150542596433699, 0.90134860821775
logistic (50,) 0.001 200 : 0.8505064055595953, 0.9095135786443502
logistic (50,) 0.001 100 : 0.8471324594519938, 0.9114596007965449
logistic (50,) 0.001 50 : 0.8558993540431136, 0.9165256255609266
logistic (50,) 0.01 500 : 0.8454696934783981, 0.9110844935092947
logistic (50,) 0.01 200 : 0.8639000058706794, 0.9129280246379536
logistic (50,) 0.01 100 : 0.8614567600011694, 0.9236234020839837
logistic (50,) 0.01 50 : 0.8680292351691226, 0.926917801869003
logistic (50, 50) 0.0001 500 : 0.8060398895026172, 0.8774712857180098
logistic (50, 50) 0.0001 200 : 0.8340061928034126, 0.8832395389651464
logistic (50, 50) 0.0001 100 : 0.8396944006284961, 0.8936536963102985
logistic (50, 50) 0.0001 50 : 0.8548175759970833, 0.9045677921459915
logistic (50, 50) 0.001 500 : 0.812316253866889, 0.9008362614313965
logistic (50, 50) 0.001 200 : 0.8378814115350016, 0.8906015764336067
logistic (50, 50) 0.001 100 : 0.844221028494184, 0.8899376340028753
logistic (50, 50) 0.001 50 : 0.8568356713744434, 0.9183461089303696
logistic (50, 50) 0.01 500 : 0.813372184544462, 0.9126831924598119
logistic (50, 50) 0.01 200 : 0.8448462401017321, 0.8949693362072197
logistic (50, 50) 0.01 100 : 0.8546977518946257, 0.9125490044591068
logistic (50, 50) 0.01 50 : 0.8622003319729457, 0.9082852519071793
logistic (50, 100) 0.0001 500 : 0.8007503280815962, 0.8948475225630865
logistic (50, 100) 0.0001 200 : 0.8294538185832423, 0.8763803948198601
logistic (50, 100) 0.0001 100 : 0.8323866861912815, 0.8961658775758903
logistic (50, 100) 0.0001 50 : 0.8499348173191075, 0.8967799916660038
logistic (50, 100) 0.001 500 : 0.8077403785325636, 0.8872119440086955
logistic (50, 100) 0.001 200 : 0.8370136100140394, 0.8886706844404157
logistic (50, 100) 0.001 100 : 0.8379813611625909, 0.8998711794714381
logistic (50, 100) 0.001 50 : 0.8506036639190396, 0.9063960536273573
logistic (50, 100) 0.01 500 : 0.8308880617819432, -0.021444359250632372
logistic (50, 100) 0.01 200 : 0.6650723877783246, 0.8946522487448018
logistic (50, 100) 0.01 100 : 0.8498172356955059, 0.9061573336097736
logistic (50, 100) 0.01 50 : 0.8557579259664344, 0.9164248272782152
logistic (50, 200) 0.0001 500 : 0.7866348915931038, 0.8875857452382061
logistic (50, 200) 0.0001 200 : 0.8226532334749388, 0.8756219406468209
logistic (50, 200) 0.0001 100 : 0.8268782457248545, 0.8899597300997306
logistic (50, 200) 0.0001 50 : 0.843672391659769, 0.897546081739214
logistic (50, 200) 0.001 500 : 0.7917507068465597, 0.8915440876355424
logistic (50, 200) 0.001 200 : 0.8234279123759762, 0.8794134167744859
logistic (50, 200) 0.001 100 : 0.8341680361349072, 0.8942451204638747
logistic (50, 200) 0.001 50 : 0.8468250409406733, 0.9009554224402058
logistic (50, 200) 0.01 500 : 0.8183189035323745, 0.8980325287853499
logistic (50, 200) 0.01 200 : 0.8369677422164836, 0.8986211461150305
logistic (50, 200) 0.01 100 : 0.8470497105324817, 0.90543607759471
logistic (50, 200) 0.01 50 : 0.8564370458676395, 0.9081338821836392

Grid search of LightGBM, Morgan(r=3)
lgb.LGBMRegressor(boosting_type = "gbdt", num_leaves = j,max_depth = 0)
j: accuracy of prediction using cross validation, accuracy of prediction using test data
10 : 0.7791153500847718, 0.8776073620869446
50 : 0.779617125088072, 0.8784972869343217
100 : 0.779617125088072, 0.8784972869343217
150 : 0.779617125088072, 0.8784972869343217

Grid search of SVR, Morgan(r=3, 1024)
SVR(C= c_num, kernel = ker, epsilon = e, gamma = r,degree = 3, coef0=1)
Ker c_num r e: accuracy of prediction using cross validation, accuracy of prediction using test data
linear 1 1 0.1 : 0.8786327367556235, 0.9438332669952136
linear 1 1 0.3 : 0.8789182152292885, 0.9431063701614615
linear 1 1 0.5 : 0.8783404401254078, 0.9425854936234168
linear 1 1 0.7 : 0.8775397494513104, 0.9418310159531027
linear 1 1 0.9 : 0.8767463387212058, 0.9401359888259593
linear 1 2 0.1 : 0.8786327367556235, 0.9438332669952136
linear 1 2 0.3 : 0.8789182152292885, 0.9431063701614615
linear 1 2 0.5 : 0.8783404401254078, 0.9425854936234168
linear 1 2 0.7 : 0.8775397494513104, 0.9418310159531027
linear 1 2 0.9 : 0.8767463387212058, 0.9401359888259593
linear 1 3 0.1 : 0.8786327367556235, 0.9438332669952136
linear 1 3 0.3 : 0.8789182152292885, 0.9431063701614615
linear 1 3 0.5 : 0.8783404401254078, 0.9425854936234168
linear 1 3 0.7 : 0.8775397494513104, 0.9418310159531027
linear 1 3 0.9 : 0.8767463387212058, 0.9401359888259593
linear 1 4 0.1 : 0.8786327367556235, 0.9438332669952136
linear 1 4 0.3 : 0.8789182152292885, 0.9431063701614615
linear 1 4 0.5 : 0.8783404401254078, 0.9425854936234168
linear 1 4 0.7 : 0.8775397494513104, 0.9418310159531027
linear 1 4 0.9 : 0.8767463387212058, 0.9401359888259593
linear 1 5 0.1 : 0.8786327367556235, 0.9438332669952136
linear 1 5 0.3 : 0.8789182152292885, 0.9431063701614615
linear 1 5 0.5 : 0.8783404401254078, 0.9425854936234168
linear 1 5 0.7 : 0.8775397494513104, 0.9418310159531027
linear 1 5 0.9 : 0.8767463387212058, 0.9401359888259593
linear 1 6 0.1 : 0.8786327367556235, 0.9438332669952136
linear 1 6 0.3 : 0.8789182152292885, 0.9431063701614615
linear 1 6 0.5 : 0.8783404401254078, 0.9425854936234168
linear 1 6 0.7 : 0.8775397494513104, 0.9418310159531027
linear 1 6 0.9 : 0.8767463387212058, 0.9401359888259593
linear 2 1 0.1 : 0.8914596737935867, 0.9410146479806509
linear 2 1 0.3 : 0.8915581556405314, 0.9403428202767664
linear 2 1 0.5 : 0.8904702035159495, 0.9395732542990842
linear 2 1 0.7 : 0.8892863221808212, 0.9385591856149804

linear 2 1 0.9 : 0.8880258717278989, 0.9369115912632845
linear 2 2 0.1 : 0.8914596737935867, 0.9410146479806509
linear 2 2 0.3 : 0.8915581556405314, 0.9403428202767664
linear 2 2 0.5 : 0.8904702035159495, 0.9395732542990842
linear 2 2 0.7 : 0.8892863221808212, 0.9385591856149804
linear 2 2 0.9 : 0.8880258717278989, 0.9369115912632845
linear 2 3 0.1 : 0.8914596737935867, 0.9410146479806509
linear 2 3 0.3 : 0.8915581556405314, 0.9403428202767664
linear 2 3 0.5 : 0.8904702035159495, 0.9395732542990842
linear 2 3 0.7 : 0.8892863221808212, 0.9385591856149804
linear 2 3 0.9 : 0.8880258717278989, 0.9369115912632845
linear 2 4 0.1 : 0.8914596737935867, 0.9410146479806509
linear 2 4 0.3 : 0.8915581556405314, 0.9403428202767664
linear 2 4 0.5 : 0.8904702035159495, 0.9395732542990842
linear 2 4 0.7 : 0.8892863221808212, 0.9385591856149804
linear 2 4 0.9 : 0.8880258717278989, 0.9369115912632845
linear 2 5 0.1 : 0.8914596737935867, 0.9410146479806509
linear 2 5 0.3 : 0.8915581556405314, 0.9403428202767664
linear 2 5 0.5 : 0.8904702035159495, 0.9395732542990842
linear 2 5 0.7 : 0.8892863221808212, 0.9385591856149804
linear 2 5 0.9 : 0.8880258717278989, 0.9369115912632845
linear 2 6 0.1 : 0.8914596737935867, 0.9410146479806509
linear 2 6 0.3 : 0.8915581556405314, 0.9403428202767664
linear 2 6 0.5 : 0.8904702035159495, 0.9395732542990842
linear 2 6 0.7 : 0.8892863221808212, 0.9385591856149804
linear 2 6 0.9 : 0.8880258717278989, 0.9369115912632845
linear 3 1 0.1 : 0.8924496304709443, 0.9386357495198168
linear 3 1 0.3 : 0.8926896092369997, 0.9381700249678615
linear 3 1 0.5 : 0.8918397401607722, 0.937703696410767
linear 3 1 0.7 : 0.8907804071247487, 0.9369428603607767
linear 3 1 0.9 : 0.8895324439586781, 0.9357011633493504
linear 3 2 0.1 : 0.8924496304709443, 0.9386357495198168
linear 3 2 0.3 : 0.8926896092369997, 0.9381700249678615
linear 3 2 0.5 : 0.8918397401607722, 0.937703696410767
linear 3 2 0.7 : 0.8907804071247487, 0.9369428603607767
linear 3 2 0.9 : 0.8895324439586781, 0.9357011633493504
linear 3 3 0.1 : 0.8924496304709443, 0.9386357495198168
linear 3 3 0.3 : 0.8926896092369997, 0.9381700249678615
linear 3 3 0.5 : 0.8918397401607722, 0.937703696410767
linear 3 3 0.7 : 0.8907804071247487, 0.9369428603607767
linear 3 3 0.9 : 0.8895324439586781, 0.9357011633493504
linear 3 4 0.1 : 0.8924496304709443, 0.9386357495198168
linear 3 4 0.3 : 0.8926896092369997, 0.9381700249678615
linear 3 4 0.5 : 0.8918397401607722, 0.937703696410767
linear 3 4 0.7 : 0.8907804071247487, 0.9369428603607767
linear 3 4 0.9 : 0.8895324439586781, 0.9357011633493504
linear 3 5 0.1 : 0.8924496304709443, 0.9386357495198168
linear 3 5 0.3 : 0.8926896092369997, 0.9381700249678615
linear 3 5 0.5 : 0.8918397401607722, 0.937703696410767
linear 3 5 0.7 : 0.8907804071247487, 0.9369428603607767
linear 3 5 0.9 : 0.8895324439586781, 0.9357011633493504
linear 3 6 0.1 : 0.8924496304709443, 0.9386357495198168
linear 3 6 0.3 : 0.8926896092369997, 0.9381700249678615
linear 3 6 0.5 : 0.8918397401607722, 0.937703696410767
linear 3 6 0.7 : 0.8907804071247487, 0.9369428603607767
linear 3 6 0.9 : 0.8895324439586781, 0.9357011633493504
linear 4 1 0.1 : 0.8930731975063558, 0.9383317142073829
linear 4 1 0.3 : 0.8933762456634561, 0.938089531223391
linear 4 1 0.5 : 0.8924922275760292, 0.9377048696164335
linear 4 1 0.7 : 0.8913473082632304, 0.9370232486223863
linear 4 1 0.9 : 0.8899646539120628, 0.9358062609015245
linear 4 2 0.1 : 0.8930731975063558, 0.9383317142073829
linear 4 2 0.3 : 0.8933762456634561, 0.938089531223391
linear 4 2 0.5 : 0.8924922275760292, 0.9377048696164335
linear 4 2 0.7 : 0.8913473082632304, 0.9370232486223863
linear 4 2 0.9 : 0.8899646539120628, 0.9358062609015245
linear 4 3 0.1 : 0.8930731975063558, 0.9383317142073829
linear 4 3 0.3 : 0.8933762456634561, 0.938089531223391
linear 4 3 0.5 : 0.8924922275760292, 0.9377048696164335
linear 4 3 0.7 : 0.8913473082632304, 0.9370232486223863
linear 4 3 0.9 : 0.8899646539120628, 0.9358062609015245
linear 4 4 0.1 : 0.8930731975063558, 0.9383317142073829
linear 4 4 0.3 : 0.8933762456634561, 0.938089531223391
linear 4 4 0.5 : 0.8924922275760292, 0.9377048696164335
linear 4 4 0.7 : 0.8913473082632304, 0.9370232486223863
linear 4 4 0.9 : 0.8899646539120628, 0.9358062609015245
linear 4 5 0.1 : 0.8930731975063558, 0.9383317142073829
linear 4 5 0.3 : 0.8933762456634561, 0.938089531223391
linear 4 5 0.5 : 0.8924922275760292, 0.9377048696164335
linear 4 5 0.7 : 0.8913473082632304, 0.9370232486223863
linear 4 5 0.9 : 0.8899646539120628, 0.9358062609015245
linear 4 6 0.1 : 0.8930731975063558, 0.9383317142073829
linear 4 6 0.3 : 0.8933762456634561, 0.938089531223391
linear 4 6 0.5 : 0.8924922275760292, 0.9377048696164335
linear 4 6 0.7 : 0.8913473082632304, 0.9370232486223863
linear 4 6 0.9 : 0.8899646539120628, 0.9358062609015245
poly 1 1 0.1 : 0.7751662221904585, 0.8317019911710657
poly 1 1 0.3 : 0.7719850187192708, 0.8283279068859691
poly 1 1 0.5 : 0.7686283408511997, 0.8249192750349229
poly 1 1 0.7 : 0.7651306946005907, 0.8211681271660654
poly 1 1 0.9 : 0.7614729746659142, 0.8172488155391093
poly 1 2 0.1 : 0.7733327723007448, 0.8297848010621616
poly 1 2 0.3 : 0.7701408333690368, 0.8263750251944674
poly 1 2 0.5 : 0.7667693748822328, 0.8229526165785942
poly 1 2 0.7 : 0.763263782087241, 0.8191837756687002
poly 1 2 0.9 : 0.7595904219369048, 0.8152508364270917
poly 1 3 0.1 : 0.7727102360662901, 0.8291322224471679
poly 1 3 0.3 : 0.7695149111223076, 0.8257127305718414
poly 1 3 0.5 : 0.7661389888582331, 0.8222833093576726
poly 1 3 0.7 : 0.7626293214637976, 0.818509182500363
poly 1 3 0.9 : 0.7589523225997759, 0.8145707551764224
poly 1 4 0.1 : 0.772397366913347, 0.8288031449728352
poly 1 4 0.3 : 0.7691999618980266, 0.8253811358453054
poly 1 4 0.5 : 0.7658216208270682, 0.8219450978262033
poly 1 4 0.7 : 0.7623095562635486, 0.8181688540552061
poly 1 4 0.9 : 0.7586303956955132, 0.8142292617134734
poly 1 5 0.1 : 0.7722084049521664, 0.828604974018588
poly 1 5 0.3 : 0.7690100437555032, 0.825181087949346
poly 1 5 0.5 : 0.7656303601852835, 0.8217423914156065
poly 1 5 0.7 : 0.7621169931404401, 0.817963726870703
poly 1 5 0.9 : 0.7584369973419116, 0.8140228743148978
poly 1 6 0.1 : 0.7720822981547641, 0.8284729025370517
poly 1 6 0.3 : 0.7688831125729184, 0.8250477961954342
poly 1 6 0.5 : 0.7655031461822936, 0.8216058247001893
poly 1 6 0.7 : 0.7619887675041996, 0.8178269553774685
poly 1 6 0.9 : 0.7583074949643637, 0.8138855488398232
poly 2 1 0.1 : 0.7751662221905334, 0.8317019911710803
poly 2 1 0.3 : 0.7719850187193351, 0.828327906885653
poly 2 1 0.5 : 0.768628340851303, 0.8249192750349936
poly 2 1 0.7 : 0.7651306946005643, 0.8211681271661468
poly 2 1 0.9 : 0.7614729746659142, 0.8172488155391093
poly 2 2 0.1 : 0.7733327723009487, 0.8297848010643316
poly 2 2 0.3 : 0.770140833368928, 0.8263750251954224
poly 2 2 0.5 : 0.7667693748813049, 0.8229526165802523
poly 2 2 0.7 : 0.7632637820859787, 0.8191837756690521
poly 2 2 0.9 : 0.7595904219369048, 0.8152508364270917
poly 2 3 0.1 : 0.7727102360665083, 0.8291322224423358
poly 2 3 0.3 : 0.7695149111242034, 0.8257127305688758
poly 2 3 0.5 : 0.7661389888594272, 0.822283309362683
poly 2 3 0.7 : 0.7626293214643496, 0.8185091824979324
poly 2 3 0.9 : 0.7589523225997759, 0.8145707551764224
poly 2 4 0.1 : 0.7723973669142141, 0.8288031449759605
poly 2 4 0.3 : 0.7691999618922584, 0.8253811358529487
poly 2 4 0.5 : 0.7658216208303721, 0.8219450978248776
poly 2 4 0.7 : 0.762309556268597, 0.8181688540460703
poly 2 4 0.9 : 0.7586303956955132, 0.8142292617134734
poly 2 5 0.1 : 0.7722084049559745, 0.8286049739973153
poly 2 5 0.3 : 0.7690100437413614, 0.8251810879388009
poly 2 5 0.5 : 0.7656303602038604, 0.8217423914083145
poly 2 5 0.7 : 0.7621169931360469, 0.8179637268875766
poly 2 5 0.9 : 0.7584369973419116, 0.8140228743148978
poly 2 6 0.1 : 0.7720822981694869, 0.8284729025078607
poly 2 6 0.3 : 0.7688831125628095, 0.8250477962631069
poly 2 6 0.5 : 0.7655031461808234, 0.8216058247201721
poly 2 6 0.7 : 0.7619887675413779, 0.817826955319164
poly 2 6 0.9 : 0.7583074949643637, 0.8138855488398232
poly 3 1 0.1 : 0.7751662221905065, 0.8317019911711899
poly 3 1 0.3 : 0.7719850187193551, 0.8283279068856411

poly 3 1 0.5 : 0.7686283408511239, 0.8249192750351713
poly 3 1 0.7 : 0.7651306946005912, 0.8211681271663742
poly 3 1 0.9 : 0.7614729746659142, 0.8172488155391093
poly 3 2 0.1 : 0.773332772301673, 0.8297848010603668
poly 3 2 0.3 : 0.7701408333700291, 0.8263750251969563
poly 3 2 0.5 : 0.7667693748800634, 0.8229526165816379
poly 3 2 0.7 : 0.7632637820857622, 0.8191837756686244
poly 3 2 0.9 : 0.7595904219369048, 0.8152508364270917
poly 3 3 0.1 : 0.7727102360623591, 0.829132222440761
poly 3 3 0.3 : 0.7695149111311149, 0.825712730569668
poly 3 3 0.5 : 0.7661389888598278, 0.8222833093532438
poly 3 3 0.7 : 0.7626293214655788, 0.8185091825009997
poly 3 3 0.9 : 0.7589523225997759, 0.8145707551764224
poly 3 4 0.1 : 0.7723973669081363, 0.8288031449742895
poly 3 4 0.3 : 0.7691999618889555, 0.8253811358350446
poly 3 4 0.5 : 0.7658216208226485, 0.8219450978422086
poly 3 4 0.7 : 0.7623095562682464, 0.8181688540536146
poly 3 4 0.9 : 0.7586303956955132, 0.8142292617134734
poly 3 5 0.1 : 0.7722084049603146, 0.8286049740265705
poly 3 5 0.3 : 0.7690100437474543, 0.8251810879840634
poly 3 5 0.5 : 0.7656303602062599, 0.8217423914168029
poly 3 5 0.7 : 0.7621169931355221, 0.817963726872259
poly 3 5 0.9 : 0.7584369973419116, 0.8140228743148978
poly 3 6 0.1 : 0.7720822981664329, 0.8284729024564755
poly 3 6 0.3 : 0.7688831126025858, 0.8250477962620097
poly 3 6 0.5 : 0.76550314618283, 0.8216058247002078
poly 3 6 0.7 : 0.7619887675114112, 0.8178269553287405
poly 3 6 0.9 : 0.7583074949643637, 0.8138855488398232
poly 4 1 0.1 : 0.7751662221906003, 0.8317019911715072
poly 4 1 0.3 : 0.7719850187191151, 0.8283279068856009
poly 4 1 0.5 : 0.7686283408511818, 0.8249192750352103
poly 4 1 0.7 : 0.7651306946005009, 0.8211681271663425
poly 4 1 0.9 : 0.7614729746659142, 0.8172488155391093
poly 4 2 0.1 : 0.7733327723000297, 0.8297848010590992
poly 4 2 0.3 : 0.7701408333719578, 0.8263750251962325
poly 4 2 0.5 : 0.7667693748819161, 0.8229526165788397
poly 4 2 0.7 : 0.7632637820869287, 0.8191837756705775
poly 4 2 0.9 : 0.7595904219369048, 0.8152508364270917
poly 4 3 0.1 : 0.772710236065665, 0.8291322224482918
poly 4 3 0.3 : 0.7695149111302195, 0.8257127305695497
poly 4 3 0.5 : 0.76613898886097, 0.822283309360008
poly 4 3 0.7 : 0.762629321467489, 0.8185091825037409
poly 4 3 0.9 : 0.7589523225997759, 0.8145707551764224
poly 4 4 0.1 : 0.7723973669143473, 0.8288031449688158
poly 4 4 0.3 : 0.7691999618993781, 0.825381135826291
poly 4 4 0.5 : 0.7658216208274233, 0.821945097837185
poly 4 4 0.7 : 0.7623095562656511, 0.8181688540577243
poly 4 4 0.9 : 0.7586303956955132, 0.8142292617134734
poly 4 5 0.1 : 0.772208404942109, 0.828604974095108
poly 4 5 0.3 : 0.769010043775744, 0.8251810879537502
poly 4 5 0.5 : 0.7656303602258934, 0.821742391458507
poly 4 5 0.7 : 0.7621169931512637, 0.8179637268368445
poly 4 5 0.9 : 0.7584369973419116, 0.8140228743148978
poly 4 6 0.1 : 0.7720822981601659, 0.8284729024914472
poly 4 6 0.3 : 0.7688831126100577, 0.825047796209197
poly 4 6 0.5 : 0.7655031461434154, 0.8216058247067843
poly 4 6 0.7 : 0.761988767512444, 0.8178269553896964
poly 4 6 0.9 : 0.7583074949643637, 0.8138855488398232
rbf 1 1 0.1 : -0.027373659988091915, 0.001565257997329983
rbf 1 1 0.3 : -0.02711955396107877, 0.0015338875874316704
rbf 1 1 0.5 : -0.02651479769107885, 0.0014637524794048895
rbf 1 1 0.7 : -0.025720462447338433, 0.0014166939314441684
rbf 1 1 0.9 : -0.02446253911597367, 0.0013857749257387697
rbf 1 2 0.1 : -0.027373674033629846, 0.001565259389008422
rbf 1 2 0.3 : -0.027119568008564788, 0.0015338889678809808
rbf 1 2 0.5 : -0.026514811748516776, 0.0014637538367194836
rbf 1 2 0.7 : -0.02572047651556586, 0.001416695274653046
rbf 1 2 0.9 : -0.024462553208706737, 0.001385776265193428
rbf 1 3 0.1 : -0.027373674033707475, 0.0015652593890191913
rbf 1 3 0.3 : -0.027119568008642327, 0.00153388896789175
rbf 1 3 0.5 : -0.0265148117485944, 0.0014637538367301417
rbf 1 3 0.7 : -0.025720476515643576, 0.001416695274663704
rbf 1 3 0.9 : -0.024462553208784633, 0.0013857762652040861
rbf 1 4 0.1 : -0.027373674033707475, 0.0015652593890191913
rbf 1 4 0.3 : -0.027119568008642327, 0.00153388896789175
rbf 1 4 0.5 : -0.0265148117485944, 0.0014637538367301417
rbf 1 4 0.7 : -0.025720476515643576, 0.001416695274663704
rbf 1 4 0.9 : -0.024462553208784633, 0.0013857762652040861
rbf 1 5 0.1 : -0.027373674033707475, 0.0015652593890191913
rbf 1 5 0.3 : -0.027119568008642327, 0.00153388896789175
rbf 1 5 0.5 : -0.0265148117485944, 0.0014637538367301417
rbf 1 5 0.7 : -0.025720476515643576, 0.001416695274663704
rbf 1 5 0.9 : -0.024462553208784633, 0.0013857762652040861
rbf 1 6 0.1 : -0.027373674033707475, 0.0015652593890191913
rbf 1 6 0.3 : -0.027119568008642327, 0.00153388896789175
rbf 1 6 0.5 : -0.0265148117485944, 0.0014637538367301417
rbf 1 6 0.7 : -0.025720476515643576, 0.001416695274663704
rbf 1 6 0.9 : -0.024462553208784633, 0.0013857762652040861
rbf 2 1 0.1 : -0.024727662818877904, 0.0030568980761344067
rbf 2 1 0.3 : -0.024040095498605973, 0.0029815443070525527
rbf 2 1 0.5 : -0.023360343262277317, 0.002881798980601147
rbf 2 1 0.7 : -0.022553153962079263, 0.0027629915846295594
rbf 2 1 0.9 : -0.021807445371463972, 0.0026372986843252733
rbf 2 2 0.1 : -0.024727690998222807, 0.003056900881847291
rbf 2 2 0.3 : -0.024040123716728524, 0.0029815472179688385
rbf 2 2 0.5 : -0.02336037172376626, 0.002881801984016552
rbf 2 2 0.7 : -0.022553182438433426, 0.0027629946724857524
rbf 2 2 0.9 : -0.02180747383876729, 0.0026373018529726178
rbf 2 3 0.1 : -0.02472769099837846, 0.0030569008818687182
rbf 2 3 0.3 : -0.02404012371688431, 0.002981547217990266
rbf 2 3 0.5 : -0.023360371723922534, 0.0028818019840379794
rbf 2 3 0.7 : -0.022553182438589968, 0.0027629946725071797
rbf 2 3 0.9 : -0.021807473838924007, 0.002637301852993823
rbf 2 4 0.1 : -0.02472769099837846, 0.0030569008818687182
rbf 2 4 0.3 : -0.02404012371688431, 0.002981547217990266
rbf 2 4 0.5 : -0.023360371723922534, 0.0028818019840379794
rbf 2 4 0.7 : -0.022553182438589968, 0.0027629946725071797
rbf 2 4 0.9 : -0.021807473838924007, 0.002637301852993823
rbf 2 5 0.1 : -0.02472769099837846, 0.0030569008818687182
rbf 2 5 0.3 : -0.02404012371688431, 0.002981547217990266
rbf 2 5 0.5 : -0.023360371723922534, 0.0028818019840379794
rbf 2 5 0.7 : -0.022553182438589968, 0.0027629946725071797
rbf 2 5 0.9 : -0.021807473838924007, 0.002637301852993823
rbf 2 6 0.1 : -0.02472769099837846, 0.0030569008818687182
rbf 2 6 0.3 : -0.02404012371688431, 0.002981547217990266
rbf 2 6 0.5 : -0.023360371723922534, 0.0028818019840379794
rbf 2 6 0.7 : -0.022553182438589968, 0.0027629946725071797
rbf 2 6 0.9 : -0.021807473838924007, 0.002637301852993823
rbf 3 1 0.1 : -0.0219000598760394, 0.004171448709922254
rbf 3 1 0.3 : -0.021144356777607554, 0.004064367014301018
rbf 3 1 0.5 : -0.020245899651792642, 0.003916007089408313
rbf 3 1 0.7 : -0.019195635731278936, 0.0036647317922969824
rbf 3 1 0.9 : -0.01810950831612721, 0.003346985195099239
rbf 3 2 0.1 : -0.021900102501394115, 0.004171453224730337
rbf 3 2 0.3 : -0.021144370263133584, 0.004064371596202099
rbf 3 2 0.5 : -0.020245909933347499, 0.003916011714145462
rbf 3 2 0.7 : -0.019195699579830673, 0.0036647364104955837
rbf 3 2 0.9 : -0.01810956665907808, 0.0033469897792893777
rbf 3 3 0.1 : -0.021900102501629215, 0.004171453224761756
rbf 3 3 0.3 : -0.021144370263368906, 0.00406437159623374
rbf 3 3 0.5 : -0.020245909933710938, 0.003916011714176548
rbf 3 3 0.7 : -0.019195699580067015, 0.003664736410526115
rbf 3 3 0.9 : -0.018109566659314826, 0.0033469897793192427
rbf 3 4 0.1 : -0.021900102501629215, 0.004171453224761756
rbf 3 4 0.3 : -0.021144370263368906, 0.00406437159623374
rbf 3 4 0.5 : -0.020245909933710938, 0.003916011714176548
rbf 3 4 0.7 : -0.019195699580067015, 0.003664736410526115
rbf 3 4 0.9 : -0.018109566659314826, 0.0033469897793192427
rbf 3 5 0.1 : -0.021900102501629215, 0.004171453224761756
rbf 3 5 0.3 : -0.021144370263368906, 0.00406437159623374
rbf 3 5 0.5 : -0.020245909933710938, 0.003916011714176548
rbf 3 5 0.7 : -0.019195699580067015, 0.003664736410526115
rbf 3 5 0.9 : -0.018109566659314826, 0.0033469897793192427
rbf 3 6 0.1 : -0.021900102501629215, 0.004171453224761756
rbf 3 6 0.3 : -0.021144370263368906, 0.00406437159623374
rbf 3 6 0.5 : -0.020245909933710938, 0.003916011714176548
rbf 3 6 0.7 : -0.019195699580067015, 0.003664736410526115
rbf 3 6 0.9 : -0.018109566659314826, 0.0033469897793192427

rbf 4 1 0.1 : -0.018014795707431207, 0.004589094475989985
rbf 4 1 0.3 : -0.01696870540697524, 0.00415752563517402
rbf 4 1 0.5 : -0.015935883068191537, 0.0036989980646702225
rbf 4 1 0.7 : -0.014987202231491326, 0.0032186570576595575
rbf 4 1 0.9 : -0.01405397961752708, 0.002613952464571745
rbf 4 2 0.1 : -0.018014852848745377, 0.004589100157873838
rbf 4 2 0.3 : -0.01696877935816059, 0.004157531191201436
rbf 4 2 0.5 : -0.015935926904984975, 0.0036990034903048308
rbf 4 2 0.7 : -0.01498722385187783, 0.0032186623484490307
rbf 4 2 0.9 : -0.014054037342099712, 0.002613957570374145
rbf 4 3 0.1 : -0.018014852849061257, 0.0045891001579123625
rbf 4 3 0.3 : -0.016968779358477758, 0.00415753119123885
rbf 4 3 0.5 : -0.015935926905303033, 0.003699003490341024
rbf 4 3 0.7 : -0.014987202741356542, 0.0032186623484837806
rbf 4 3 0.9 : -0.014054037342419634, 0.00261395757040761
rbf 4 4 0.1 : -0.018014852849061257, 0.0045891001579123625
rbf 4 4 0.3 : -0.016968779358477758, 0.00415753119123885
rbf 4 4 0.5 : -0.015935926905303033, 0.003699003490341024
rbf 4 4 0.7 : -0.014987202741356542, 0.0032186623484837806
rbf 4 4 0.9 : -0.014054037342419634, 0.00261395757040761
rbf 4 5 0.1 : -0.018014852849061257, 0.0045891001579123625
rbf 4 5 0.3 : -0.016968779358477758, 0.00415753119123885
rbf 4 5 0.5 : -0.015935926905303033, 0.003699003490341024
rbf 4 5 0.7 : -0.014987202741356542, 0.0032186623484837806
rbf 4 5 0.9 : -0.014054037342419634, 0.00261395757040761
rbf 4 6 0.1 : -0.018014852849061257, 0.0045891001579123625
rbf 4 6 0.3 : -0.016968779358477758, 0.00415753119123885
rbf 4 6 0.5 : -0.015935926905303033, 0.003699003490341024
rbf 4 6 0.7 : -0.014987202741356542, 0.0032186623484837806
rbf 4 6 0.9 : -0.014054037342419634, 0.00261395757040761
sigmoid 1 1 0.1 : -0.02785165481189087, -0.0004817815251891844
sigmoid 1 1 0.3 : -0.02803862774396353, -0.0001527580421345487
sigmoid 1 1 0.5 : -0.029327672135454775, -8.396224731543889e-05
sigmoid 1 1 0.7 : -0.02737080281184512, -0.0001527585208407345
sigmoid 1 1 0.9 : -0.02742592792975742, -0.00035164852081615017
sigmoid 1 2 0.1 : -0.027851574944783363, -
0.00048175891844071117
sigmoid 1 2 0.3 : -0.02803863742512349, -0.00015273486544797876
sigmoid 1 2 0.5 : -0.02932768292988257, -8.393840807219277e-05
sigmoid 1 2 0.7 : -0.027370781055832127, -
0.00015273486544775672
sigmoid 1 2 0.9 : -0.027425939282392654, -0.0003516451206320159
sigmoid 1 3 0.1 : -0.02785157494475108, -0.0004817589182650739
sigmoid 1 3 0.3 : -0.028038637425086766, -0.0001527348652718974
sigmoid 1 3 0.5 : -0.029327682929847977, -8.39384078961114e-05
sigmoid 1 3 0.7 : -0.027370781055785543, -0.0001527348652718974
sigmoid 1 3 0.9 : -0.02742593928234638, -0.0003516451204561566
sigmoid 1 4 0.1 : -0.02785157494475108, -0.0004817589182650739
sigmoid 1 4 0.3 : -0.028038637425086766, -0.0001527348652718974
sigmoid 1 4 0.5 : -0.029327682929847977, -8.39384078961114e-05
sigmoid 1 4 0.7 : -0.027370781055785543, -0.0001527348652718974
sigmoid 1 4 0.9 : -0.02742593928234638, -0.0003516451204561566
sigmoid 1 5 0.1 : -0.02785157494475108, -0.0004817589182650739
sigmoid 1 5 0.3 : -0.028038637425086766, -0.0001527348652718974
sigmoid 1 5 0.5 : -0.029327682929847977, -8.39384078961114e-05
sigmoid 1 5 0.7 : -0.027370781055785543, -0.0001527348652718974
sigmoid 1 5 0.9 : -0.02742593928234638, -0.0003516451204561566
sigmoid 1 6 0.1 : -0.02785157494475108, -0.0004817589182650739
sigmoid 1 6 0.3 : -0.028038637425086766, -0.0001527348652718974
sigmoid 1 6 0.5 : -0.029327682929847977, -8.39384078961114e-05
sigmoid 1 6 0.7 : -0.027370781055785543, -0.0001527348652718974
sigmoid 1 6 0.9 : -0.02742593928234638, -0.0003516451204561566
sigmoid 2 1 0.1 : -0.027851734679337346, -0.0004818041322389721
sigmoid 2 1 0.3 : -0.02803861806295007, -0.0001527812191222111
sigmoid 2 1 0.5 : -0.029327661341170864, -8.398608685888931e-05
sigmoid 2 1 0.7 : -0.027370824568079266, -
0.00015278217653347248
sigmoid 2 1 0.9 : -0.027425916577277352, -0.0003516519218822456
sigmoid 2 2 0.1 : -0.027851574944815737, -0.0004817589186161264
sigmoid 2 2 0.3 : -0.028038637425160173, -
0.00015273486562406013
sigmoid 2 2 0.5 : -0.029327682929920586, -8.393840824871823e-05
sigmoid 2 2 0.7 : -0.02737078105587871, -0.000152734865623394
sigmoid 2 2 0.9 : -0.027425939282438794, -
0.00035164512080743116
sigmoid 2 3 0.1 : -0.02785157494475108, -0.0004817589182650739
sigmoid 2 3 0.3 : -0.028038637425086766, -0.0001527348652718974
sigmoid 2 3 0.5 : -0.029327682929847977, -8.39384078961114e-05
sigmoid 2 3 0.7 : -0.027370781055785543, -0.0001527348652718974
sigmoid 2 3 0.9 : -0.02742593928234638, -0.0003516451204561566
sigmoid 2 4 0.1 : -0.02785157494475108, -0.0004817589182650739
sigmoid 2 4 0.3 : -0.028038637425086766, -0.0001527348652718974
sigmoid 2 4 0.5 : -0.029327682929847977, -8.39384078961114e-05
sigmoid 2 4 0.7 : -0.027370781055785543, -0.0001527348652718974
sigmoid 2 4 0.9 : -0.02742593928234638, -0.0003516451204561566
sigmoid 2 5 0.1 : -0.02785157494475108, -0.0004817589182650739
sigmoid 2 5 0.3 : -0.028038637425086766, -0.0001527348652718974
sigmoid 2 5 0.5 : -0.029327682929847977, -8.39384078961114e-05
sigmoid 2 5 0.7 : -0.027370781055785543, -0.0001527348652718974
sigmoid 2 5 0.9 : -0.02742593928234638, -0.0003516451204561566
sigmoid 2 6 0.1 : -0.02785157494475108, -0.0004817589182650739
sigmoid 2 6 0.3 : -0.028038637425086766, -0.0001527348652718974
sigmoid 2 6 0.5 : -0.029327682929847977, -8.39384078961114e-05
sigmoid 2 6 0.7 : -0.027370781055785543, -0.0001527348652718974
sigmoid 2 6 0.9 : -0.02742593928234638, -0.0003516451204561566
sigmoid 3 1 0.1 : -0.027851814547090292, -
0.00048182673941310483
sigmoid 3 1 0.3 : -0.028038608382045905, -
0.00015280439623488462
sigmoid 3 1 0.5 : -0.029327650546995886, -8.400992652557449e-05
sigmoid 3 1 0.7 : -0.027370846324487406, -
0.00015280583234944523
sigmoid 3 1 0.9 : -0.027425905224906132, -
0.00035165532365399876
sigmoid 3 2 0.1 : -0.027851574944848023, -0.0004817589187917637
sigmoid 3 2 0.3 : -0.028038637425196987, -0.0001527348658001415
sigmoid 3 2 0.5 : -0.02932768292995691, -8.39384084247996e-05
sigmoid 3 2 0.7 : -0.02737078105592543, -0.0001527348657992533
sigmoid 3 2 0.9 : -0.027425939282485247, -0.0003516451209832905
sigmoid 3 3 0.1 : -0.02785157494475108, -0.0004817589182650739
sigmoid 3 3 0.3 : -0.028038637425086766, -0.0001527348652718974
sigmoid 3 3 0.5 : -0.029327682929847977, -8.39384078961114e-05
sigmoid 3 3 0.7 : -0.027370781055785543, -0.0001527348652718974
sigmoid 3 3 0.9 : -0.02742593928234638, -0.0003516451204561566
sigmoid 3 4 0.1 : -0.02785157494475108, -0.0004817589182650739
sigmoid 3 4 0.3 : -0.028038637425086766, -0.0001527348652718974
sigmoid 3 4 0.5 : -0.029327682929847977, -8.39384078961114e-05
sigmoid 3 4 0.7 : -0.027370781055785543, -0.0001527348652718974
sigmoid 3 4 0.9 : -0.02742593928234638, -0.0003516451204561566
sigmoid 3 5 0.1 : -0.02785157494475108, -0.0004817589182650739
sigmoid 3 5 0.3 : -0.028038637425086766, -0.0001527348652718974
sigmoid 3 5 0.5 : -0.029327682929847977, -8.39384078961114e-05
sigmoid 3 5 0.7 : -0.027370781055785543, -0.0001527348652718974
sigmoid 3 5 0.9 : -0.02742593928234638, -0.0003516451204561566
sigmoid 3 6 0.1 : -0.02785157494475108, -0.0004817589182650739
sigmoid 3 6 0.3 : -0.028038637425086766, -0.0001527348652718974
sigmoid 3 6 0.5 : -0.029327682929847977, -8.39384078961114e-05
sigmoid 3 6 0.7 : -0.027370781055785543, -0.0001527348652718974
sigmoid 3 6 0.9 : -0.02742593928234638, -0.0003516451204561566
sigmoid 4 1 0.1 : -0.027851894415150413, -0.0004818493467133589
sigmoid 4 1 0.3 : -0.02803859870125249, -0.0001528275734732354
sigmoid 4 1 0.5 : -0.029327639752930557, -8.403376631682669e-05
sigmoid 4 1 0.7 : -0.0273708680810703, -0.00015282948829065113
sigmoid 4 1 0.9 : -0.027425893872644425, -
0.00035165872613318605
sigmoid 4 2 0.1 : -0.027851574944880175, -
0.00048175891896717893
sigmoid 4 2 0.3 : -0.028038637425233583, -
0.00015273486597644492
sigmoid 4 2 0.5 : -0.029327682929993194, -8.393840860132507e-05
sigmoid 4 2 0.7 : -0.02737078105597197, -0.00015273486597511265
sigmoid 4 2 0.9 : -0.02742593928253152, -0.0003516451211587057
sigmoid 4 3 0.1 : -0.02785157494475108, -0.0004817589182650739
sigmoid 4 3 0.3 : -0.028038637425086766, -0.0001527348652718974
sigmoid 4 3 0.5 : -0.029327682929847977, -8.39384078961114e-05
sigmoid 4 3 0.7 : -0.027370781055785543, -0.0001527348652718974
sigmoid 4 3 0.9 : -0.02742593928234638, -0.0003516451204561566
sigmoid 4 4 0.1 : -0.02785157494475108, -0.0004817589182650739

sigmoid 4 4 0.3 : -0.028038637425086766, -0.0001527348652718974
sigmoid 4 4 0.5 : -0.029327682929847977, -8.39384078961114e-05
sigmoid 4 4 0.7 : -0.027370781055785543, -0.0001527348652718974
sigmoid 4 4 0.9 : -0.02742593928234638, -0.0003516451204561566
sigmoid 4 5 0.1 : -0.02785157494475108, -0.0004817589182650739
sigmoid 4 5 0.3 : -0.028038637425086766, -0.0001527348652718974
sigmoid 4 5 0.5 : -0.029327682929847977, -8.39384078961114e-05
sigmoid 4 5 0.7 : -0.027370781055785543, -0.0001527348652718974
sigmoid 4 5 0.9 : -0.02742593928234638, -0.0003516451204561566
sigmoid 4 6 0.1 : -0.02785157494475108, -0.0004817589182650739
sigmoid 4 6 0.3 : -0.028038637425086766, -0.0001527348652718974
sigmoid 4 6 0.5 : -0.029327682929847977, -8.39384078961114e-05
sigmoid 4 6 0.7 : -0.027370781055785543, -0.0001527348652718974
sigmoid 4 6 0.9 : -0.02742593928234638, -0.0003516451204561566

Grid search of ElasticNet, Morgan(r=3,2048)
ElasticNet(alpha= i, l1_ratio= j, max_iter=100000)
i j : accuracy of prediction using cross validation, accuracy of prediction using test data
0.001 0.0 : 0.9027948079159593, 0.9501339790552962
0.001 0.2 : 0.9047217074371072, 0.9560265951605402
0.001 0.4 : 0.9055680259430969, 0.9592879165126791
0.001 0.6 : 0.9056567124554362, 0.9613043856978162
0.001 0.8 : 0.9027588966963019, 0.9612144619291558
0.001 1.0 : 0.8900760780309447, 0.9475000552422218
0.01 0.0 : 0.8886278572807453, 0.9330262551224254
0.01 0.2 : 0.8898508022613558, 0.9375737459411725
0.01 0.4 : 0.8909986296377704, 0.9411286102739679
0.01 0.6 : 0.8922586944096791, 0.9433117387930161
0.01 0.8 : 0.8927373661089911, 0.9431181299624274
0.01 1.0 : 0.8942060346216316, 0.9385038339877707
0.1 0.0 : 0.8013904131492415, 0.831876248016772
0.1 0.2 : 0.7893668534663324, 0.8175888370591926
0.1 0.4 : 0.7817697442896451, 0.8080442301203781
0.1 0.6 : 0.780358235707731, 0.8063725191513722
0.1 0.8 : 0.7886841984224537, 0.813718563408422
0.1 1.0 : 0.8139803163142496, 0.8400068183190965

Grid search of RandomForest, Morgan(r=3,2048)
RandomForestRegressor(n_estimators= i)
i: accuracy of prediction using cross validation, accuracy of prediction using test data
100 1.0 : 0.7828043064690913, 0.8513588903095427
500 1.0 : 0.7841139154606201, 0.8492453444043279
1000 1.0 : 0.7827419594708719, 0.8464069132427052
2000 1.0 : 0.7847852317369002, 0.8479068883730849

Grid search of NeuralNetwork, Morgan(r=3,2048)
MLPRegressor(activation=act, alpha=a, batch_size=batch, beta_1=0.9,
beta_2=0.999, early_stopping=False, epsilon=1e-08,
hidden_layer_sizes=hid, learning_rate='constant',
learning_rate_init=0.001, max_iter=100000, momentum=0.9,
n_iter_no_change=10, nesterovs_momentum=True,
power_t=0.5,
random_state=1, shuffle=True, solver='adam', tol=0.0001,
validation_fraction=0.1, verbose=False, warm_start=False)
act (hid) a batch: accuracy of prediction using cross validation, accuracy of prediction using test data
relu (200,) 0.0001 500 : 0.8000822342867693, 0.9147032234533659
relu (200,) 0.0001 200 : 0.8359821480751011, 0.9088856912372286
relu (200,) 0.0001 100 : 0.8382648252765937, 0.9128212369420162
relu (200,) 0.0001 50 : 0.8453200332242565, 0.9149713025519464
relu (200,) 0.001 500 : 0.8001037278466276, 0.9149002377777199
relu (200,) 0.001 200 : 0.8361091184229388, 0.9090428170402564
relu (200,) 0.001 100 : 0.8384270111668387, 0.9131135731561275
relu (200,) 0.001 50 : 0.8457313270560531, 0.9154963807251005
relu (200,) 0.01 500 : 0.8005340432604626, 0.9170983958912686
relu (200,) 0.01 200 : 0.8386766120358796, 0.9099988373972275
relu (200,) 0.01 100 : 0.8419356888271367, 0.9159476108110108
relu (200,) 0.01 50 : 0.8509009561067217, 0.9197910860952215
relu (200, 200) 0.0001 500 : 0.7934247357572075,
0.9060631429431408
relu (200, 200) 0.0001 200 : 0.8303891069070557,
0.9009008234383843
relu (200, 200) 0.0001 100 : 0.8374912715145758,
0.9063935078215161
relu (200, 200) 0.0001 50 : 0.8438744498743842,
0.9142353972022547
relu (200, 200) 0.001 500 : 0.7937157639866396,
0.9055415757412747
relu (200, 200) 0.001 200 : 0.8304538269412675,
0.9007124872977049
relu (200, 200) 0.001 100 : 0.8373267600327946,
0.904585187454597
relu (200, 200) 0.001 50 : 0.8433733382097971, 0.914021699045994
relu (200, 200) 0.01 500 : 0.7930602367914827,
0.9065306242436201
relu (200, 200) 0.01 200 : 0.8309236367904728,
0.9004029364387768
relu (200, 200) 0.01 100 : 0.8374020151324902,
0.9057757742454735
relu (200, 200) 0.01 50 : 0.8440717020332466, 0.9137176794067445
relu (100,) 0.0001 500 : 0.7999702004410059, 0.9170383336462283
relu (100,) 0.0001 200 : 0.8373349112731177, 0.9101220691795309
relu (100,) 0.0001 100 : 0.8396685686833691, 0.9119660611619913
relu (100,) 0.0001 50 : 0.8473337021559413, 0.9157213503362903
relu (100,) 0.001 500 : 0.799997390685724, 0.9171801245830549
relu (100,) 0.001 200 : 0.8374809538261907, 0.9102259602106384
relu (100,) 0.001 100 : 0.8399075662800761, 0.912481061233048
relu (100,) 0.001 50 : 0.8452786548223991, 0.91591095404239
relu (100,) 0.01 500 : 0.8003127036694749, 0.9191245200814168
relu (100,) 0.01 200 : 0.8399504748989901, 0.9110818720091779
relu (100,) 0.01 100 : 0.842108987808342, 0.9160652125101714
relu (100,) 0.01 50 : 0.8487553839538883, 0.9194475221884238
relu (100, 100) 0.0001 500 : 0.8023352178390277,
0.9039597369837825
relu (100, 100) 0.0001 200 : 0.8360561212852762,
0.9001682955006943
relu (100, 100) 0.0001 100 : 0.8406912343694254,
0.905150150264063
relu (100, 100) 0.0001 50 : 0.8469276440061995,
0.9121438956049918
relu (100, 100) 0.001 500 : 0.8024374392890621,
0.9048200463494194
relu (100, 100) 0.001 200 : 0.8353119093191903,
0.8996164092430751
relu (100, 100) 0.001 100 : 0.8408915753412775,
0.9041923605756035
relu (100, 100) 0.001 50 : 0.8475018471024356, 0.908421114985541
relu (100, 100) 0.01 500 : 0.8018534067724452,
0.9045190310435914
relu (100, 100) 0.01 200 : 0.8365378778094218,
0.8997809399436911
relu (100, 100) 0.01 100 : 0.8419330124518147,
0.9077019674620815
relu (100, 100) 0.01 50 : 0.8471104258505585, 0.9148638349228962
relu (100, 200) 0.0001 500 : 0.7997223672497003,
0.9083232571314788
relu (100, 200) 0.0001 200 : 0.8372669485516863,
0.8963924441415679
relu (100, 200) 0.0001 100 : 0.8411761316487343,
0.9093830630627819
relu (100, 200) 0.0001 50 : 0.8491047096637135,
0.9102988636529057
relu (100, 200) 0.001 500 : 0.7985266107729865,
0.9092465397309866
relu (100, 200) 0.001 200 : 0.835545515586191,
0.8992988899430865
relu (100, 200) 0.001 100 : 0.8394709686064642,
0.9082013760424261
relu (100, 200) 0.001 50 : 0.8478713459141798,
0.9111121988299784
relu (100, 200) 0.01 500 : 0.7992490942037707,
0.9055306811430999
relu (100, 200) 0.01 200 : 0.8328341072748664,

0.9019883638149886
relu (100, 200) 0.01 100 : 0.8375321556021389, 0.9098563416914466
relu (100, 200) 0.01 50 : 0.8476842655140604, 0.9119261687244653
relu (50,) 0.0001 500 : 0.8019071525473753, 0.9172350197196106
relu (50,) 0.0001 200 : 0.8405152849275261, 0.910664860553248
relu (50,) 0.0001 100 : 0.8408292396850188, 0.9141405001007561
relu (50,) 0.0001 50 : 0.8498785321103217, 0.9172195970228669
relu (50,) 0.001 500 : 0.8019423310410474, 0.9173749124981234
relu (50,) 0.001 200 : 0.8406704780579674, 0.9107729233881823
relu (50,) 0.001 100 : 0.841096213765867, 0.9144533497554209
relu (50,) 0.001 50 : 0.8489127676225664, 0.9176807912649728
relu (50,) 0.01 500 : 0.802293432429469, 0.918629485396191
relu (50,) 0.01 200 : 0.8421434625193112, 0.9117449990572634
relu (50,) 0.01 100 : 0.8438209597330572, 0.9166896958239417
relu (50,) 0.01 50 : 0.8536780098191787, 0.9219655210249768
relu (50, 50) 0.0001 500 : 0.7994868406447095, 0.9088026701223019
relu (50, 50) 0.0001 200 : 0.8429296798430508, 0.90087198086561
relu (50, 50) 0.0001 100 : 0.844508479241955, 0.9070743939926798
relu (50, 50) 0.0001 50 : 0.8514461417469873, 0.9110274370339723
relu (50, 50) 0.001 500 : 0.8000519005848105, 0.9089504682047902
relu (50, 50) 0.001 200 : 0.8429535557685878, 0.9013102941583513
relu (50, 50) 0.001 100 : 0.8440204269479323, 0.908699181611602
relu (50, 50) 0.001 50 : 0.8522366645068619, 0.9121829435583675
relu (50, 50) 0.01 500 : 0.8018374442468534, 0.9096779369275577
relu (50, 50) 0.01 200 : 0.8438558817510327, 0.9017958410922109
relu (50, 50) 0.01 100 : 0.8445120833354528, 0.9087396202271586
relu (50, 50) 0.01 50 : 0.8501534060812169, 0.9124543131702509
relu (50, 100) 0.0001 500 : 0.7987775700386796, 0.9037380016436357
relu (50, 100) 0.0001 200 : 0.8411517186764241, 0.8988360140805507
relu (50, 100) 0.0001 100 : 0.84583701568763, 0.9020214095492657
relu (50, 100) 0.0001 50 : 0.8490645040265322, 0.9123499164544481
relu (50, 100) 0.001 500 : 0.7997932473321588, 0.9032852792888448
relu (50, 100) 0.001 200 : 0.8410656331565294, 0.8998299262498244
relu (50, 100) 0.001 100 : 0.845856479051913, 0.9039244408278345
relu (50, 100) 0.001 50 : 0.8475560236167372, 0.9117114339844999
relu (50, 100) 0.01 500 : 0.7994437567413883, 0.905277564303657
relu (50, 100) 0.01 200 : 0.8410694243674474, 0.8986826464190121
relu (50, 100) 0.01 100 : 0.846967395605923, 0.9025423931543126
relu (50, 100) 0.01 50 : 0.8501737168389394, 0.9092196975677943
relu (50, 200) 0.0001 500 : 0.7947509213579117, 0.9096725994503778
relu (50, 200) 0.0001 200 : 0.8364947989055885, 0.8994290355928184
relu (50, 200) 0.0001 100 : 0.8428534564928529, 0.9058027831079036
relu (50, 200) 0.0001 50 : 0.8489136255500085, 0.9115855677495928
relu (50, 200) 0.001 500 : 0.7946887260386231, 0.9079998302150859
relu (50, 200) 0.001 200 : 0.8371197453091448, 0.8983993808266721
relu (50, 200) 0.001 100 : 0.8422871943321167, 0.9060744038821414
relu (50, 200) 0.001 50 : 0.8481526092385732, 0.9112826012412236
relu (50, 200) 0.01 500 : 0.7948866317024713, 0.908384244622892
relu (50, 200) 0.01 200 : 0.8360914255582438, 0.8986157384118424
relu (50, 200) 0.01 100 : 0.8430889457010391, 0.9064783531679598
relu (50, 200) 0.01 50 : 0.8482783177804833, 0.9113844303165373
tanh (200,) 0.0001 500 : 0.8104070556120337, 0.8826413876334491
tanh (200,) 0.0001 200 : 0.8444415778982796, 0.8771627564795174
tanh (200,) 0.0001 100 : 0.8479118193282351, 0.8852939777908071
tanh (200,) 0.0001 50 : 0.8557442774770208, 0.8929206675319351
tanh (200,) 0.001 500 : 0.8114169936417189, 0.8839804704333406
tanh (200,) 0.001 200 : 0.8466476332229895, 0.8785559805952721
tanh (200,) 0.001 100 : 0.8494141339565531, 0.88634823423612
tanh (200,) 0.001 50 : 0.8564020255667945, 0.8908140969440462
tanh (200,) 0.01 500 : 0.8203830526386378, 0.8940246894099909
tanh (200,) 0.01 200 : 0.85579573009924, 0.8909862297182517
tanh (200,) 0.01 100 : 0.8581543229412013, 0.8964597707476696
tanh (200,) 0.01 50 : 0.864422130625327, 0.8993090669257555
tanh (200, 200) 0.0001 500 : 0.8061086366142938, 0.8970040577180819
tanh (200, 200) 0.0001 200 : 0.6682771282176613, 0.8920220017216306
tanh (200, 200) 0.0001 100 : 0.8560006233991233, 0.8966365465566183
tanh (200, 200) 0.0001 50 : 0.8628167754265512, 0.9039032937163911
tanh (200, 200) 0.001 500 : 0.808192769989996, 0.8956590417787162
tanh (200, 200) 0.001 200 : 0.6699068221822135, 0.8918701602704554
tanh (200, 200) 0.001 100 : 0.8565286580881729, 0.8977007232576244
tanh (200, 200) 0.001 50 : 0.863572316408462, 0.9023660015603189
tanh (200, 200) 0.01 500 : 0.8256966906217846, 0.9048537075227712
tanh (200, 200) 0.01 200 : 0.6824606952849095, 0.8980988480051878
tanh (200, 200) 0.01 100 : 0.8670075390858178, 0.9083064856467737
tanh (200, 200) 0.01 50 : 0.8728867937281424, 0.9137544271778936
tanh (100,) 0.0001 500 : 0.791540577927956, 0.8869779875315815
tanh (100,) 0.0001 200 : 0.8358700860908893, 0.8828855580251206
tanh (100,) 0.0001 100 : 0.8380266565520806, 0.8913513072783228
tanh (100,) 0.0001 50 : 0.8484245810008716, 0.898614955675893
tanh (100,) 0.001 500 : 0.7947038742650641, 0.8884913662169902
tanh (100,) 0.001 200 : 0.8386054442238459, 0.8847676690097257
tanh (100,) 0.001 100 : 0.8412720537373829, 0.89271983084229
tanh (100,) 0.001 50 : 0.849270294196535, 0.9019532373257043
tanh (100,) 0.01 500 : 0.8155568482362934, 0.8965864496023578
tanh (100,) 0.01 200 : 0.8492925746127777, 0.9001254503619709
tanh (100,) 0.01 100 : 0.853280773856147, 0.9082188796520493
tanh (100,) 0.01 50 : 0.8592867533589251, 0.9095090475994694
tanh (100, 100) 0.0001 500 : 0.7898959388254398, 0.8889370877571497
tanh (100, 100) 0.0001 200 : 0.8442726989751114, 0.889459772057736
tanh (100, 100) 0.0001 100 : 0.8490211408002913, 0.8975151472529966
tanh (100, 100) 0.0001 50 : 0.8590851316527853, 0.9029656138181787
tanh (100, 100) 0.001 500 : 0.7932058376326169, 0.8910351163674105
tanh (100, 100) 0.001 200 : 0.8453642272162443, 0.8915929681286946
tanh (100, 100) 0.001 100 : 0.8520261688739584, 0.8984451237067892
tanh (100, 100) 0.001 50 : 0.8595372415056476, 0.9041822041586248
tanh (100, 100) 0.01 500 : 0.816108345782849, 0.8988317674358259
tanh (100, 100) 0.01 200 : 0.8570322297217079, 0.9050961766120839
tanh (100, 100) 0.01 100 : 0.8636172915684794, 0.9030571254701506
tanh (100, 100) 0.01 50 : 0.8700339804560111, 0.9215078758197252
tanh (100, 200) 0.0001 500 : 0.7988824015355313, 0.877022176347262
tanh (100, 200) 0.0001 200 : 0.8370768092270959, 0.882704186759842
tanh (100, 200) 0.0001 100 : 0.8465554570452566, 0.8903537130260681
tanh (100, 200) 0.0001 50 : 0.8595808438651353, 0.9035462429951603
tanh (100, 200) 0.001 500 : 0.799773949814705, 0.877943122051944
tanh (100, 200) 0.001 200 : 0.8380267627268905, 0.883061455944179
tanh (100, 200) 0.001 100 : 0.8508108321830866, 0.8924453743772682
tanh (100, 200) 0.001 50 : 0.8601531546176109, 0.9063828547041187

tanh (100, 200) 0.01 500 : 0.8075907503311937,
0.8842398728343949
tanh (100, 200) 0.01 200 : 0.8459802054178868,
0.8852007241819548
tanh (100, 200) 0.01 100 : 0.8570712651568722,
0.8973394060712012
tanh (100, 200) 0.01 50 : 0.8654810428699209, 0.9105559951889867
tanh (50,) 0.0001 500 : 0.786416219518219, 0.8995604866117776
tanh (50,) 0.0001 200 : 0.8362506858805748, 0.892006642039936
tanh (50,) 0.0001 100 : 0.8357455067775728, 0.8999909789601072
tanh (50,) 0.0001 50 : 0.8421903454104814, 0.8995106456197027
tanh (50,) 0.001 500 : 0.7922789243404225, 0.9029261486069606
tanh (50,) 0.001 200 : 0.8379175046991916, 0.892969041872327
tanh (50,) 0.001 100 : 0.8412098060313952, 0.899929298164874
tanh (50,) 0.001 50 : 0.8455344755859298, 0.9065954307230786
tanh (50,) 0.01 500 : 0.8260406150406151, 0.9085245544904204
tanh (50,) 0.01 200 : 0.8556760299117852, 0.9069058511205774
tanh (50,) 0.01 100 : 0.8557007179943181, 0.9140454770151183
tanh (50,) 0.01 50 : 0.8663045461369707, 0.9174327286771012
tanh (50, 50) 0.0001 500 : 0.7836011460192747,
0.8830814934749529
tanh (50, 50) 0.0001 200 : 0.8317332318120515,
0.8747706404198486
tanh (50, 50) 0.0001 100 : 0.8421523278185633,
0.8982542796721541
tanh (50, 50) 0.0001 50 : 0.8484952934502988, 0.8999198598476861
tanh (50, 50) 0.001 500 : 0.7898580717429656, 0.8900844830793136
tanh (50, 50) 0.001 200 : 0.835959867265748, 0.8795110042422617
tanh (50, 50) 0.001 100 : 0.8463311317784772, 0.9026885196413091
tanh (50, 50) 0.001 50 : 0.8467418137510483, 0.901736818136039
tanh (50, 50) 0.01 500 : 0.8156916029301978, 0.9055149891709011
tanh (50, 50) 0.01 200 : 0.8562830649059456, 0.8920311246808369
tanh (50, 50) 0.01 100 : 0.8614452501908781, 0.906276527990398
tanh (50, 50) 0.01 50 : 0.8650992115000463, 0.9170641204551785
tanh (50, 100) 0.0001 500 : 0.78457571452088, 0.8879068096931624
tanh (50, 100) 0.0001 200 : 0.8357219661139206,
0.8833176745319308
tanh (50, 100) 0.0001 100 : 0.8443687996032239,
0.8963620623827385
tanh (50, 100) 0.0001 50 : 0.8544025362805451,
0.9031834840144268
tanh (50, 100) 0.001 500 : 0.7866676651689306,
0.8891660376074075
tanh (50, 100) 0.001 200 : 0.8375684422585495,
0.8836364732648262
tanh (50, 100) 0.001 100 : 0.8450188472470724,
0.8973861627858505
tanh (50, 100) 0.001 50 : 0.8558473111996122, 0.9028799385771127
tanh (50, 100) 0.01 500 : 0.8057897918285016, 0.8962499422533796
tanh (50, 100) 0.01 200 : 0.8491196549816789, 0.8953682359408838
tanh (50, 100) 0.01 100 : 0.855999622460297, 0.9042092996852193
tanh (50, 100) 0.01 50 : 0.8633799411866965, 0.9103307799410596
tanh (50, 200) 0.0001 500 : 0.7888821300567853,
0.8854558817274339
tanh (50, 200) 0.0001 200 : 0.8428996699346349,
0.8696289001155069
tanh (50, 200) 0.0001 100 : 0.8494398287551942,
0.8933779278869145
tanh (50, 200) 0.0001 50 : 0.8638661511152858,
0.8988495052728834
tanh (50, 200) 0.001 500 : 0.7894243083529332,
0.8858336191819096
tanh (50, 200) 0.001 200 : 0.8432844854554544,
0.8699255756502897
tanh (50, 200) 0.001 100 : 0.8492472523827811,
0.8904698816631653
tanh (50, 200) 0.001 50 : 0.8646528561513614, 0.9039848254953106
tanh (50, 200) 0.01 500 : 0.7946197661564068, 0.8893741030866918
tanh (50, 200) 0.01 200 : 0.8466471467454871, 0.875607233213348
tanh (50, 200) 0.01 100 : 0.8524606615851912, 0.8940325552076839
tanh (50, 200) 0.01 50 : 0.8642165717354697, 0.9004235292704301
logistic (200,) 0.0001 500 : 0.8084931465550429,
0.8869275743666967
logistic (200,) 0.0001 200 : 0.8501308556190382,
0.8779722877929215
logistic (200,) 0.0001 100 : 0.8519203755756933,
0.8876901926390813
logistic (200,) 0.0001 50 : 0.8604208269899182,
0.8936011128769236
logistic (200,) 0.001 500 : 0.8133805638611646,
0.8923963382558299
logistic (200,) 0.001 200 : 0.8576211633064215,
0.8841089257295328
logistic (200,) 0.001 100 : 0.8595792772719613,
0.8955238467261531
logistic (200,) 0.001 50 : 0.8650646905163027, 0.8994456586460813
logistic (200,) 0.01 500 : 0.8459602988436481, 0.9123911586306702
logistic (200,) 0.01 200 : 0.8751392001817067, 0.9234163162566343
logistic (200,) 0.01 100 : 0.8769518886250616, 0.9198827874433614
logistic (200,) 0.01 50 : 0.8796684686353331, 0.9215399475645979
logistic (200, 200) 0.0001 500 : 0.803346670289374,
0.8757810197876408
logistic (200, 200) 0.0001 200 : 0.8479410771170365,
0.8535852571888988
logistic (200, 200) 0.0001 100 : 0.8468506953127241,
0.8810736819118982
logistic (200, 200) 0.0001 50 : 0.8654476902462293,
0.8927781966613839
logistic (200, 200) 0.001 500 : 0.8191431632348805,
0.8827660924553071
logistic (200, 200) 0.001 200 : 0.8572672521643835,
0.8596239068097777
logistic (200, 200) 0.001 100 : 0.8578748523301112,
0.8863089000985781
logistic (200, 200) 0.001 50 : 0.87096202403193,
0.9045419864096476
logistic (200, 200) 0.01 500 : 0.8471531987938169,
0.8933718811091081
logistic (200, 200) 0.01 200 : 0.8579259571169924,
0.8774849971061619
logistic (200, 200) 0.01 100 : 0.8636786705197299,
0.9063995943715651
logistic (200, 200) 0.01 50 : 0.8749116556187164,
0.9219925176651885
logistic (100,) 0.0001 500 : 0.8073257458615896,
0.8943867362432301
logistic (100,) 0.0001 200 : 0.8503840407718005,
0.8834836529705304
logistic (100,) 0.0001 100 : 0.8518299644294164,
0.8922518840389516
logistic (100,) 0.0001 50 : 0.8587801671334647,
0.9000837628079538
logistic (100,) 0.001 500 : 0.8152420681564662,
0.8992058854298943
logistic (100,) 0.001 200 : 0.8598777873558063,
0.8924997690169765
logistic (100,) 0.001 100 : 0.8600799112345132,
0.8993505573111611
logistic (100,) 0.001 50 : 0.8650064968859855, 0.9093191563082635
logistic (100,) 0.01 500 : 0.8526886722144476, 0.9103535924636962
logistic (100,) 0.01 200 : 0.8746113699600067, 0.9179838289499396
logistic (100,) 0.01 100 : 0.8736184146832832, 0.9207983650353385
logistic (100,) 0.01 50 : 0.881179409782615, 0.9213191623162208
logistic (100, 100) 0.0001 500 : 0.8045500793912554,
0.8831746758151398
logistic (100, 100) 0.0001 200 : 0.8470779305028315,
0.8593165110113239
logistic (100, 100) 0.0001 100 : 0.8560490137855366,
0.878233699849338
logistic (100, 100) 0.0001 50 : 0.8680870332034978,
0.8939391387394217
logistic (100, 100) 0.001 500 : 0.8189921241205029,
0.8900343182752151
logistic (100, 100) 0.001 200 : 0.8568469324514257,
0.871366514547718
logistic (100, 100) 0.001 100 : 0.8570404424154819,
0.8877322058525713
logistic (100, 100) 0.001 50 : 0.8750259784593133,

0.9034331445513123
logistic (100, 100) 0.01 500 : 0.8266612398397676, 0.8920027807624598
logistic (100, 100) 0.01 200 : 0.8573625953937889, 0.8881950392572997
logistic (100, 100) 0.01 100 : 0.8630140151707151, 0.9022918988776203
logistic (100, 100) 0.01 50 : 0.8743989752034264, 0.9256981843052212
logistic (100, 200) 0.0001 500 : 0.8014111417073225, 0.8711897461551354
logistic (100, 200) 0.0001 200 : 0.8372638545085019, 0.8539387129119611
logistic (100, 200) 0.0001 100 : 0.8435000990113826, 0.8844794478056338
logistic (100, 200) 0.0001 50 : 0.861701982270478, 0.8970104606649358
logistic (100, 200) 0.001 500 : 0.8109881637001235, 0.8766199935764023
logistic (100, 200) 0.001 200 : 0.8460682356008592, 0.8548654350054257
logistic (100, 200) 0.001 100 : 0.8504129282167717, 0.8880116286175505
logistic (100, 200) 0.001 50 : 0.8656105822035347, 0.9008586496105896
logistic (100, 200) 0.01 500 : 0.8470250625721629, 0.8925664886795835
logistic (100, 200) 0.01 200 : 0.8540378572238151, 0.8745073009185109
logistic (100, 200) 0.01 100 : 0.8522954901962752, 0.8884017543915084
logistic (100, 200) 0.01 50 : 0.8735115152364319, 0.8952044324509293
logistic (50,) 0.0001 500 : 0.8170148656675981, 0.8992891731135474
logistic (50,) 0.0001 200 : 0.8541702514973343, 0.8913870745126075
logistic (50,) 0.0001 100 : 0.854839361045537, 0.9004592745583107
logistic (50,) 0.0001 50 : 0.8598337403410337, 0.9063299253505169
logistic (50,) 0.001 500 : 0.8305292919500289, 0.9053695760614342
logistic (50,) 0.001 200 : 0.8608173301079726, 0.9034545549724229
logistic (50,) 0.001 100 : 0.86470909411794, 0.9101864505382257
logistic (50,) 0.001 50 : 0.8653253242564869, 0.9175539976614016
logistic (50,) 0.01 500 : 0.8590962281287492, 0.9156970788888644
logistic (50,) 0.01 200 : 0.8717238428251008, 0.9171708773101598
logistic (50,) 0.01 100 : 0.8702697521066611, 0.9287671215821377
logistic (50,) 0.01 50 : 0.8790618862973808, 0.9326545611463833
logistic (50, 50) 0.0001 500 : 0.8063276061969946, 0.8770659933559277
logistic (50, 50) 0.0001 200 : 0.8521514100991304, 0.8656355105438288
logistic (50, 50) 0.0001 100 : 0.8510752431141494, 0.8942360714618095
logistic (50, 50) 0.0001 50 : 0.8637697858004538, 0.8972712577836315
logistic (50, 50) 0.001 500 : 0.8215772879904779, 0.8878477139230311
logistic (50, 50) 0.001 200 : 0.8605909391967413, 0.874535362479201
logistic (50, 50) 0.001 100 : 0.8601758005872693, 0.9111515680339266
logistic (50, 50) 0.001 50 : 0.8672913127340454, 0.9111420238485239
logistic (50, 50) 0.01 500 : 0.824116422075733, 0.8926097542259451
logistic (50, 50) 0.01 200 : 0.8675046964332502, 0.8820471065847327
logistic (50, 50) 0.01 100 : 0.8609470003961626, 0.9030574984890289
logistic (50, 50) 0.01 50 : 0.8716893754562147, 0.9146689123955569
logistic (50, 100) 0.0001 500 : 0.814405364862577, 0.8873564857859437
logistic (50, 100) 0.0001 200 : 0.844616394582338, 0.8524600141994847
logistic (50, 100) 0.0001 100 : 0.8558868657651553, 0.8725801223707053
logistic (50, 100) 0.0001 50 : 0.8658481448998945, 0.897783381740209
logistic (50, 100) 0.001 500 : 0.8262067071351252, 0.8914380496272203
logistic (50, 100) 0.001 200 : 0.8485190781184727, 0.8630934438148404
logistic (50, 100) 0.001 100 : 0.8657525583964775, 0.8921254575837437
logistic (50, 100) 0.001 50 : 0.8663327679518478, 0.9068710988142612
logistic (50, 100) 0.01 500 : 0.8510833539424374, 0.8921516475893558
logistic (50, 100) 0.01 200 : 0.8595607030039816, 0.8769494051548934
logistic (50, 100) 0.01 100 : 0.8594861627752461, 0.9023696783693608
logistic (50, 100) 0.01 50 : 0.8661882458499134, 0.908552758699157
logistic (50, 200) 0.0001 500 : 0.8009814184894364, 0.8635867768985818
logistic (50, 200) 0.0001 200 : 0.8485399337633923, 0.8594760539762832
logistic (50, 200) 0.0001 100 : 0.855777287511511, 0.8827643183608094
logistic (50, 200) 0.0001 50 : 0.862748702989316, 0.8964105876367555
logistic (50, 200) 0.001 500 : 0.8095140092735965, 0.8822208149143441
logistic (50, 200) 0.001 200 : 0.8525658291089867, 0.8635555524601701
logistic (50, 200) 0.001 100 : 0.8558937154226811, 0.8882007851455087
logistic (50, 200) 0.001 50 : 0.8675220531539102, 0.8988488389007817
logistic (50, 200) 0.01 500 : 0.8434711905759122, 0.8856854713489429
logistic (50, 200) 0.01 200 : 0.8588079307091568, 0.8724956616386377
logistic (50, 200) 0.01 100 : 0.8711479348906723, 0.900417650756469
logistic (50, 200) 0.01 50 : 0.8759072204527355, 0.9148468173067679

Grid search of LightGBM, Morgan(r=3,2048)
lgb.LGBMRegressor(boosting_type = "gbdt", num_leaves = j,max_depth = 0)
j: accuracy of prediction using cross validation, accuracy of prediction using test data
10 : 0.7801434152975454, 0.8519860146215081
50 : 0.7759366516532177, 0.8561534201573155
100 : 0.7759366516532177, 0.8561534201573155
150 : 0.7759366516532177, 0.8561534201573155

Grid search of SVR, Morgan(r=3, 2048)
SVR(C= c_num, kernel = ker, epsilon = e, gamma = r,degree = 3, coef0=1)
Ker c_num r e: accuracy of prediction using cross validation, accuracy of prediction using test data
linear 1 1 0.1 : 0.8913216616929296, 0.952261246305386
linear 1 1 0.3 : 0.8904150623635492, 0.9506748431554396
linear 1 1 0.5 : 0.8889869316884065, 0.9491691010186193
linear 1 1 0.7 : 0.8873786775431188, 0.9473036004530496
linear 1 1 0.9 : 0.8854624150939527, 0.9448273186711353
linear 1 2 0.1 : 0.8913216616929296, 0.952261246305386
linear 1 2 0.3 : 0.8904150623635492, 0.9506748431554396
linear 1 2 0.5 : 0.8889869316884065, 0.9491691010186193
linear 1 2 0.7 : 0.8873786775431188, 0.9473036004530496
linear 1 2 0.9 : 0.8854624150939527, 0.9448273186711353
linear 1 3 0.1 : 0.8913216616929296, 0.952261246305386

linear 1 3 0.3 : 0.8904150623635492, 0.9506748431554396
linear 1 3 0.5 : 0.8889869316884065, 0.9491691010186193
linear 1 3 0.7 : 0.8873786775431188, 0.9473036004530496
linear 1 3 0.9 : 0.8854624150939527, 0.9448273186711353
linear 1 4 0.1 : 0.8913216616929296, 0.952261246305386
linear 1 4 0.3 : 0.8904150623635492, 0.9506748431554396
linear 1 4 0.5 : 0.8889869316884065, 0.9491691010186193
linear 1 4 0.7 : 0.8873786775431188, 0.9473036004530496
linear 1 4 0.9 : 0.8854624150939527, 0.9448273186711353
linear 1 5 0.1 : 0.8913216616929296, 0.952261246305386
linear 1 5 0.3 : 0.8904150623635492, 0.9506748431554396
linear 1 5 0.5 : 0.8889869316884065, 0.9491691010186193
linear 1 5 0.7 : 0.8873786775431188, 0.9473036004530496
linear 1 5 0.9 : 0.8854624150939527, 0.9448273186711353
linear 1 6 0.1 : 0.8913216616929296, 0.952261246305386
linear 1 6 0.3 : 0.8904150623635492, 0.9506748431554396
linear 1 6 0.5 : 0.8889869316884065, 0.9491691010186193
linear 1 6 0.7 : 0.8873786775431188, 0.9473036004530496
linear 1 6 0.9 : 0.8854624150939527, 0.9448273186711353
linear 2 1 0.1 : 0.9013990083282544, 0.9514969401573599
linear 2 1 0.3 : 0.900060860121609, 0.9498709748817253
linear 2 1 0.5 : 0.8983361409944839, 0.9483148009233999
linear 2 1 0.7 : 0.8963765053813659, 0.9459583758437218
linear 2 1 0.9 : 0.8940125789575795, 0.9433348326207361
linear 2 2 0.1 : 0.9013990083282544, 0.9514969401573599
linear 2 2 0.3 : 0.900060860121609, 0.9498709748817253
linear 2 2 0.5 : 0.8983361409944839, 0.9483148009233999
linear 2 2 0.7 : 0.8963765053813659, 0.9459583758437218
linear 2 2 0.9 : 0.8940125789575795, 0.9433348326207361
linear 2 3 0.1 : 0.9013990083282544, 0.9514969401573599
linear 2 3 0.3 : 0.900060860121609, 0.9498709748817253
linear 2 3 0.5 : 0.8983361409944839, 0.9483148009233999
linear 2 3 0.7 : 0.8963765053813659, 0.9459583758437218
linear 2 3 0.9 : 0.8940125789575795, 0.9433348326207361
linear 2 4 0.1 : 0.9013990083282544, 0.9514969401573599
linear 2 4 0.3 : 0.900060860121609, 0.9498709748817253
linear 2 4 0.5 : 0.8983361409944839, 0.9483148009233999
linear 2 4 0.7 : 0.8963765053813659, 0.9459583758437218
linear 2 4 0.9 : 0.8940125789575795, 0.9433348326207361
linear 2 5 0.1 : 0.9013990083282544, 0.9514969401573599
linear 2 5 0.3 : 0.900060860121609, 0.9498709748817253
linear 2 5 0.5 : 0.8983361409944839, 0.9483148009233999
linear 2 5 0.7 : 0.8963765053813659, 0.9459583758437218
linear 2 5 0.9 : 0.8940125789575795, 0.9433348326207361
linear 2 6 0.1 : 0.9013990083282544, 0.9514969401573599
linear 2 6 0.3 : 0.900060860121609, 0.9498709748817253
linear 2 6 0.5 : 0.8983361409944839, 0.9483148009233999
linear 2 6 0.7 : 0.8963765053813659, 0.9459583758437218
linear 2 6 0.9 : 0.8940125789575795, 0.9433348326207361
linear 3 1 0.1 : 0.9026459870563682, 0.9504368231429694
linear 3 1 0.3 : 0.9014793742132505, 0.9490768277028723
linear 3 1 0.5 : 0.8999235666487438, 0.9476116618082541
linear 3 1 0.7 : 0.8978340821450617, 0.9455029995057427
linear 3 1 0.9 : 0.8952479784795424, 0.9431974467013425
linear 3 2 0.1 : 0.9026459870563682, 0.9504368231429694
linear 3 2 0.3 : 0.9014793742132505, 0.9490768277028723
linear 3 2 0.5 : 0.8999235666487438, 0.9476116618082541
linear 3 2 0.7 : 0.8978340821450617, 0.9455029995057427
linear 3 2 0.9 : 0.8952479784795424, 0.9431974467013425
linear 3 3 0.1 : 0.9026459870563682, 0.9504368231429694
linear 3 3 0.3 : 0.9014793742132505, 0.9490768277028723
linear 3 3 0.5 : 0.8999235666487438, 0.9476116618082541
linear 3 3 0.7 : 0.8978340821450617, 0.9455029995057427
linear 3 3 0.9 : 0.8952479784795424, 0.9431974467013425
linear 3 4 0.1 : 0.9026459870563682, 0.9504368231429694
linear 3 4 0.3 : 0.9014793742132505, 0.9490768277028723
linear 3 4 0.5 : 0.8999235666487438, 0.9476116618082541
linear 3 4 0.7 : 0.8978340821450617, 0.9455029995057427
linear 3 4 0.9 : 0.8952479784795424, 0.9431974467013425
linear 3 5 0.1 : 0.9026459870563682, 0.9504368231429694
linear 3 5 0.3 : 0.9014793742132505, 0.9490768277028723
linear 3 5 0.5 : 0.8999235666487438, 0.9476116618082541
linear 3 5 0.7 : 0.8978340821450617, 0.9455029995057427
linear 3 5 0.9 : 0.8952479784795424, 0.9431974467013425
linear 3 6 0.1 : 0.9026459870563682, 0.9504368231429694
linear 3 6 0.3 : 0.9014793742132505, 0.9490768277028723
linear 3 6 0.5 : 0.8999235666487438, 0.9476116618082541
linear 3 6 0.7 : 0.8978340821450617, 0.9455029995057427
linear 3 6 0.9 : 0.8952479784795424, 0.9431974467013425
linear 4 1 0.1 : 0.9032106845807506, 0.9505538468060981
linear 4 1 0.3 : 0.902013430774222, 0.9492022656328438
linear 4 1 0.5 : 0.9002122858399982, 0.9476072119159641
linear 4 1 0.7 : 0.8980079946920425, 0.9454940788317598
linear 4 1 0.9 : 0.8954112438177037, 0.9431895817857392
linear 4 2 0.1 : 0.9032106845807506, 0.9505538468060981
linear 4 2 0.3 : 0.902013430774222, 0.9492022656328438
linear 4 2 0.5 : 0.9002122858399982, 0.9476072119159641
linear 4 2 0.7 : 0.8980079946920425, 0.9454940788317598
linear 4 2 0.9 : 0.8954112438177037, 0.9431895817857392
linear 4 3 0.1 : 0.9032106845807506, 0.9505538468060981
linear 4 3 0.3 : 0.902013430774222, 0.9492022656328438
linear 4 3 0.5 : 0.9002122858399982, 0.9476072119159641
linear 4 3 0.7 : 0.8980079946920425, 0.9454940788317598
linear 4 3 0.9 : 0.8954112438177037, 0.9431895817857392
linear 4 4 0.1 : 0.9032106845807506, 0.9505538468060981
linear 4 4 0.3 : 0.902013430774222, 0.9492022656328438
linear 4 4 0.5 : 0.9002122858399982, 0.9476072119159641
linear 4 4 0.7 : 0.8980079946920425, 0.9454940788317598
linear 4 4 0.9 : 0.8954112438177037, 0.9431895817857392
linear 4 5 0.1 : 0.9032106845807506, 0.9505538468060981
linear 4 5 0.3 : 0.902013430774222, 0.9492022656328438
linear 4 5 0.5 : 0.9002122858399982, 0.9476072119159641
linear 4 5 0.7 : 0.8980079946920425, 0.9454940788317598
linear 4 5 0.9 : 0.8954112438177037, 0.9431895817857392
linear 4 6 0.1 : 0.9032106845807506, 0.9505538468060981
linear 4 6 0.3 : 0.902013430774222, 0.9492022656328438
linear 4 6 0.5 : 0.9002122858399982, 0.9476072119159641
linear 4 6 0.7 : 0.8980079946920425, 0.9454940788317598
linear 4 6 0.9 : 0.8954112438177037, 0.9431895817857392
poly 1 1 0.1 : 0.7743397146122625, 0.8300972711822626
poly 1 1 0.3 : 0.7710921063920976, 0.8265257352135771
poly 1 1 0.5 : 0.767674506843137, 0.8227920128010505
poly 1 1 0.7 : 0.7640747497942963, 0.818948932695244
poly 1 1 0.9 : 0.7602970334165619, 0.8148871706553016
poly 1 2 0.1 : 0.7724614609682015, 0.8280727550158646
poly 1 2 0.3 : 0.7691989558749053, 0.8244791731622418
poly 1 2 0.5 : 0.7657696183940156, 0.8207237242009038
poly 1 2 0.7 : 0.7621588963641261, 0.8168559617031365
poly 1 2 0.9 : 0.7583729131356933, 0.8127845672607916
poly 1 3 0.1 : 0.7718245271799894, 0.8273842114007938
poly 1 3 0.3 : 0.7685567184055971, 0.8237810353072712
poly 1 3 0.5 : 0.7651205914673305, 0.8200202782074979
poly 1 3 0.7 : 0.7615092394031328, 0.8161438626033113
poly 1 3 0.9 : 0.7577194275330718, 0.8120692701095219
poly 1 4 0.1 : 0.77150344449537, 0.8270375302047901
poly 1 4 0.3 : 0.7682331465971107, 0.8234309635311845
poly 1 4 0.5 : 0.7647944306900332, 0.8196649282066216
poly 1 4 0.7 : 0.7611820853343441, 0.8157848243646425
poly 1 4 0.9 : 0.7573907370583597, 0.8117093137576166
poly 1 5 0.1 : 0.7713097974423444, 0.8268279542499241
poly 1 5 0.3 : 0.7680379374080359, 0.8232188672511753
poly 1 5 0.5 : 0.7645975800611374, 0.819451793204751
poly 1 5 0.7 : 0.760985120087832, 0.8155685026394823
poly 1 5 0.9 : 0.7571923954737745, 0.8114914451153488
poly 1 6 0.1 : 0.7711808586661095, 0.8266883811329009
poly 1 6 0.3 : 0.7679075740247037, 0.8230774399821799
poly 1 6 0.5 : 0.7644661986330228, 0.8193091281435182
poly 1 6 0.7 : 0.7608531922093476, 0.8154235490336894
poly 1 6 0.9 : 0.7570599355488135, 0.8113458508876852
poly 2 1 0.1 : 0.7743397146121517, 0.830097271182333
poly 2 1 0.3 : 0.7710921063920462, 0.8265257352135708
poly 2 1 0.5 : 0.7676745068431967, 0.8227920128011624
poly 2 1 0.7 : 0.7640747497942327, 0.8189489326952958
poly 2 1 0.9 : 0.7602970334165619, 0.8148871706553016
poly 2 2 0.1 : 0.7724614609691667, 0.8280727550172267
poly 2 2 0.3 : 0.7691989558748863, 0.8244791731622225
poly 2 2 0.5 : 0.7657696183941978, 0.8207237242010053
poly 2 2 0.7 : 0.7621588963655626, 0.8168559617027664

poly 2 2 0.9 : 0.7583729131356933, 0.8127845672607916
poly 2 3 0.1 : 0.7718245271735247, 0.8273842114033952
poly 2 3 0.3 : 0.7685567184052592, 0.8237810353055077
poly 2 3 0.5 : 0.76512059146932, 0.8200202782066331
poly 2 3 0.7 : 0.7615092394012164, 0.8161438626036294
poly 2 3 0.9 : 0.7577194275330718, 0.8120692701095219
poly 2 4 0.1 : 0.7715034445032586, 0.8270375302059911
poly 2 4 0.3 : 0.7682331465890684, 0.8234309635355818
poly 2 4 0.5 : 0.7647944306883221, 0.8196649282165176
poly 2 4 0.7 : 0.7611820853336924, 0.8157848243534376
poly 2 4 0.9 : 0.7573907370583597, 0.8117093137576166
poly 2 5 0.1 : 0.7713097974359445, 0.8268279542624537
poly 2 5 0.3 : 0.7680379374158676, 0.8232188672545968
poly 2 5 0.5 : 0.7645975800638165, 0.8194517931931633
poly 2 5 0.7 : 0.760985120081334, 0.8155685026361027
poly 2 5 0.9 : 0.7571923954737745, 0.8114914451153488
poly 2 6 0.1 : 0.7711808586683567, 0.826688381142915
poly 2 6 0.3 : 0.7679075739737442, 0.8230774400017514
poly 2 6 0.5 : 0.7644661986401771, 0.8193091281764826
poly 2 6 0.7 : 0.7608531922403537, 0.8154235490056866
poly 2 6 0.9 : 0.7570599355488135, 0.8113458508876852
poly 3 1 0.1 : 0.7743397146120914, 0.8300972711824322
poly 3 1 0.3 : 0.7710921063922612, 0.8265257352139584
poly 3 1 0.5 : 0.7676745068433073, 0.822792012800926
poly 3 1 0.7 : 0.7640747497941358, 0.8189489326952697
poly 3 1 0.9 : 0.7602970334165619, 0.8148871706553016
poly 3 2 0.1 : 0.7724614609679161, 0.828072755019335
poly 3 2 0.3 : 0.7691989558748857, 0.8244791731620437
poly 3 2 0.5 : 0.7657696183947726, 0.8207237242015276
poly 3 2 0.7 : 0.7621588963652546, 0.8168559617006556
poly 3 2 0.9 : 0.7583729131356933, 0.8127845672607916
poly 3 3 0.1 : 0.7718245271762738, 0.8273842113965653
poly 3 3 0.3 : 0.7685567184031358, 0.8237810353179041
poly 3 3 0.5 : 0.7651205914725019, 0.8200202782027654
poly 3 3 0.7 : 0.7615092393970986, 0.8161438626068388
poly 3 3 0.9 : 0.7577194275330718, 0.8120692701095219
poly 3 4 0.1 : 0.7715034445094789, 0.8270375302106971
poly 3 4 0.3 : 0.7682331465917032, 0.8234309635494528
poly 3 4 0.5 : 0.7647944306874142, 0.8196649282271548
poly 3 4 0.7 : 0.7611820853295727, 0.8157848243677218
poly 3 4 0.9 : 0.7573907370583597, 0.8117093137576166
poly 3 5 0.1 : 0.7713097974336258, 0.8268279542631709
poly 3 5 0.3 : 0.7680379374054809, 0.823218867256166
poly 3 5 0.5 : 0.764597580047688, 0.8194517931973182
poly 3 5 0.7 : 0.7609851200649891, 0.8155685026248543
poly 3 5 0.9 : 0.7571923954737745, 0.8114914451153488
poly 3 6 0.1 : 0.771180858671982, 0.8266883811399023
poly 3 6 0.3 : 0.7679075739896488, 0.8230774399757934
poly 3 6 0.5 : 0.7644661986500612, 0.8193091281477844
poly 3 6 0.7 : 0.7608531922216669, 0.8154235490430719
poly 3 6 0.9 : 0.7570599355488135, 0.8113458508876852
poly 4 1 0.1 : 0.7743397146123036, 0.8300972711823468
poly 4 1 0.3 : 0.7710921063923907, 0.826525735213946
poly 4 1 0.5 : 0.7676745068430273, 0.8227920128010215
poly 4 1 0.7 : 0.764074749794154, 0.8189489326952417
poly 4 1 0.9 : 0.7602970334165619, 0.8148871706553016
poly 4 2 0.1 : 0.7724614609663126, 0.8280727550192293
poly 4 2 0.3 : 0.7691989558747783, 0.8244791731615849
poly 4 2 0.5 : 0.7657696183921576, 0.8207237242023432
poly 4 2 0.7 : 0.7621588963632371, 0.816855961699146
poly 4 2 0.9 : 0.7583729131356933, 0.8127845672607916
poly 4 3 0.1 : 0.771824527183569, 0.8273842113977994
poly 4 3 0.3 : 0.768556718404404, 0.8237810353175772
poly 4 3 0.5 : 0.7651205914738535, 0.8200202782121171
poly 4 3 0.7 : 0.7615092394013516, 0.8161438626065619
poly 4 3 0.9 : 0.7577194275330718, 0.8120692701095219
poly 4 4 0.1 : 0.771503444518809, 0.8270375301943935
poly 4 4 0.3 : 0.7682331465954768, 0.8234309635349956
poly 4 4 0.5 : 0.7647944306830707, 0.8196649282099533
poly 4 4 0.7 : 0.7611820853312501, 0.8157848243858147
poly 4 4 0.9 : 0.7573907370583597, 0.8117093137576166
poly 4 5 0.1 : 0.7713097974465526, 0.8268279542979898
poly 4 5 0.3 : 0.7680379373912922, 0.8232188672950194
poly 4 5 0.5 : 0.764597580052419, 0.8194517932344385
poly 4 5 0.7 : 0.7609851201015244, 0.8155685026154034
poly 4 5 0.9 : 0.7571923954737745, 0.8114914451153488
poly 4 6 0.1 : 0.7711808587059352, 0.8266883811203334
poly 4 6 0.3 : 0.7679075739924999, 0.8230774399960963
poly 4 6 0.5 : 0.764466198608205, 0.8193091281479914
poly 4 6 0.7 : 0.7608531921860846, 0.8154235490530822
poly 4 6 0.9 : 0.7570599355488135, 0.8113458508876852
rbf 1 1 0.1 : -0.027373660125215426, 0.0015652579959628543
rbf 1 1 0.3 : -0.027119554098106756, 0.0015338875860686496
rbf 1 1 0.5 : -0.026514797827770664, 0.0014637524780443112
rbf 1 1 0.7 : -0.02572046258355374, 0.001416693930086365 7
rbf 1 1 0.9 : -0.024462539251464133, 0.0013857749243819661
rbf 1 2 0.1 : -0.027373674033629936, 0.001565259389008422
rbf 1 2 0.3 : -0.027119568008564875, 0.0015338889678809808
rbf 1 2 0.5 : -0.02651481174851682, 0.0014637538367194836
rbf 1 2 0.7 : -0.02572047651556595, 0.001416695274653046
rbf 1 2 0.9 : -0.024462553208706782, 0.001385776265193428
rbf 1 3 0.1 : -0.027373674033707475, 0.0015652593890191913
rbf 1 3 0.3 : -0.027119568008642327, 0.00153388896789175
rbf 1 3 0.5 : -0.0265148117485944, 0.0014637538367301417
rbf 1 3 0.7 : -0.025720476515643576, 0.001416695274663704
rbf 1 3 0.9 : -0.024462553208784633, 0.0013857762652040861
rbf 1 4 0.1 : -0.027373674033707475, 0.0015652593890191913
rbf 1 4 0.3 : -0.027119568008642327, 0.00153388896789175
rbf 1 4 0.5 : -0.0265148117485944, 0.0014637538367301417
rbf 1 4 0.7 : -0.025720476515643576, 0.001416695274663704
rbf 1 4 0.9 : -0.024462553208784633, 0.0013857762652040861
rbf 1 5 0.1 : -0.027373674033707475, 0.0015652593890191913
rbf 1 5 0.3 : -0.027119568008642327, 0.00153388896789175
rbf 1 5 0.5 : -0.0265148117485944, 0.0014637538367301417
rbf 1 5 0.7 : -0.025720476515643576, 0.001416695274663704
rbf 1 5 0.9 : -0.024462553208784633, 0.0013857762652040861
rbf 1 6 0.1 : -0.027373674033707475, 0.0015652593890191913
rbf 1 6 0.3 : -0.027119568008642327, 0.00153388896789175
rbf 1 6 0.5 : -0.0265148117485944, 0.0014637538367301417
rbf 1 6 0.7 : -0.025720476515643576, 0.001416695274663704
rbf 1 6 0.9 : -0.024462553208784633, 0.0013857762652040861
rbf 2 1 0.1 : -0.024727663090390005, 0.0030568980734195783
rbf 2 1 0.3 : -0.024040066622352363, 0.0029815443048419876
rbf 2 1 0.5 : -0.023360343532272853, 0.0028817989778963105
rbf 2 1 0.7 : -0.022553154231272375, 0.002762991581923502
rbf 2 1 0.9 : -0.0218074456398313, 0.0026372986816324273
rbf 2 2 0.1 : -0.024727690998222897, 0.003056900881847291
rbf 2 2 0.3 : -0.02404012371672857, 0.0029815472179688385
rbf 2 2 0.5 : -0.02336037172376635, 0.002881801984016552
rbf 2 2 0.7 : -0.022553182438433562, 0.0027629946724857524
rbf 2 2 0.9 : -0.02180747383876742, 0.0026373018529726178
rbf 2 3 0.1 : -0.02472769099837846, 0.003056900881868718 2
rbf 2 3 0.3 : -0.02404012371688431, 0.002981547217990266
rbf 2 3 0.5 : -0.023360371723922534, 0.0028818019840379794
rbf 2 3 0.7 : -0.022553182438589968, 0.0027629946725071797
rbf 2 3 0.9 : -0.021807473838924007, 0.002637301852993823
rbf 2 4 0.1 : -0.02472769099837846, 0.0030569008818687182
rbf 2 4 0.3 : -0.02404012371688431, 0.002981547217990266
rbf 2 4 0.5 : -0.023360371723922534, 0.0028818019840379794
rbf 2 4 0.7 : -0.022553182438589968, 0.0027629946725071797
rbf 2 4 0.9 : -0.021807473838924007, 0.002637301852993823
rbf 2 5 0.1 : -0.02472769099837846, 0.0030569008818687182
rbf 2 5 0.3 : -0.02404012371688431, 0.002981547217990266
rbf 2 5 0.5 : -0.023360371723922534, 0.0028818019840379794
rbf 2 5 0.7 : -0.022553182438589968, 0.0027629946725071797
rbf 2 5 0.9 : -0.021807473838924007, 0.002637301852993823
rbf 2 6 0.1 : -0.02472769099837846, 0.0030569008818687182
rbf 2 6 0.3 : -0.02404012371688431, 0.002981547217990266
rbf 2 6 0.5 : -0.023360371723922534, 0.0028818019840379794
rbf 2 6 0.7 : -0.022553182438589968, 0.0027629946725071797
rbf 2 6 0.9 : -0.021807473838924007, 0.002637301852993823
rbf 3 1 0.1 : -0.02190006027926721, 0.004171448705884595
rbf 3 1 0.3 : -0.021144357179523298, 0.00406436701027002
rbf 3 1 0.5 : -0.02024590005217104, 0.003916007085378759
rbf 3 1 0.7 : -0.019195657241674713, 0.0036647317882932 96
rbf 3 1 0.9 : -0.01810950871300272, 0.00334698519111154
rbf 3 2 0.1 : -0.021900102501394337, 0.004171453224730337
rbf 3 2 0.3 : -0.02114437026313376, 0.004064371596202099

rbf 3 2 0.5 : -0.02024590993347508, 0.003916011714145462
rbf 3 2 0.7 : -0.019195699579830895, 0.0036647364104955837
rbf 3 2 0.9 : -0.018109566659078258, 0.0033469897792893777
rbf 3 3 0.1 : -0.021900102501629215, 0.004171453224761756
rbf 3 3 0.3 : -0.021144370263368906, 0.00406437159623374
rbf 3 3 0.5 : -0.020245909933710938, 0.003916011714176548
rbf 3 3 0.7 : -0.019195699580067015, 0.003664736410526115
rbf 3 3 0.9 : -0.018109566659314826, 0.0033469897793192427
rbf 3 4 0.1 : -0.021900102501629215, 0.004171453224761756
rbf 3 4 0.3 : -0.021144370263368906, 0.00406437159623374
rbf 3 4 0.5 : -0.020245909933710938, 0.003916011714176548
rbf 3 4 0.7 : -0.019195699580067015, 0.003664736410526115
rbf 3 4 0.9 : -0.018109566659314826, 0.0033469897793192427
rbf 3 5 0.1 : -0.021900102501629215, 0.004171453224761756
rbf 3 5 0.3 : -0.021144370263368906, 0.00406437159623374
rbf 3 5 0.5 : -0.020245909933710938, 0.003916011714176548
rbf 3 5 0.7 : -0.019195699580067015, 0.003664736410526115
rbf 3 5 0.9 : -0.018109566659314826, 0.0033469897793192427
rbf 3 6 0.1 : -0.021900102501629215, 0.004171453224761756
rbf 3 6 0.3 : -0.021144370263368906, 0.00406437159623374
rbf 3 6 0.5 : -0.020245909933710938, 0.003916011714176548
rbf 3 6 0.7 : -0.019195699580067015, 0.003664736410526115
rbf 3 6 0.9 : -0.018109566659314826, 0.0033469897793192427
rbf 4 1 0.1 : -0.018014796237226925, 0.004589094470685118
rbf 4 1 0.3 : -0.016968722599413556, 0.004157525629893688
rbf 4 1 0.5 : -0.015935883593390265, 0.0036989980594426264
rbf 4 1 0.7 : -0.014987192201449905, 0.00321865705281465 5
rbf 4 1 0.9 : -0.014053980138115273, 0.002613952459381008
rbf 4 2 0.1 : -0.018014852848745645, 0.004589100157873838
rbf 4 2 0.3 : -0.016968779358160768, 0.004157531191201436
rbf 4 2 0.5 : -0.015935926904985197, 0.0036990034903048308
rbf 4 2 0.7 : -0.014987223851878095, 0.0032186623484490307
rbf 4 2 0.9 : -0.014054037342099979, 0.0026139575703744145
rbf 4 3 0.1 : -0.018014852849061257, 0.0045891001579123625
rbf 4 3 0.3 : -0.016968779358477758, 0.00415753119123885
rbf 4 3 0.5 : -0.015935926905303033, 0.003699003490341024
rbf 4 3 0.7 : -0.014987202741356542, 0.0032186623484837806
rbf 4 3 0.9 : -0.014054037342419634, 0.00261395757040761
rbf 4 4 0.1 : -0.018014852849061257, 0.0045891001579123625
rbf 4 4 0.3 : -0.016968779358477758, 0.00415753119123885
rbf 4 4 0.5 : -0.015935926905303033, 0.003699003490341024
rbf 4 4 0.7 : -0.014987202741356542, 0.0032186623484837806
rbf 4 4 0.9 : -0.014054037342419634, 0.00261395757040761
rbf 4 5 0.1 : -0.018014852849061257, 0.0045891001579123625
rbf 4 5 0.3 : -0.016968779358477758, 0.00415753119123885
rbf 4 5 0.5 : -0.015935926905303033, 0.003699003490341024
rbf 4 5 0.7 : -0.014987202741356542, 0.0032186623484837806
rbf 4 5 0.9 : -0.014054037342419634, 0.00261395757040761
rbf 4 6 0.1 : -0.018014852849061257, 0.0045891001579123625
rbf 4 6 0.3 : -0.016968779358477758, 0.00415753119123885
rbf 4 6 0.5 : -0.015935926905303033, 0.003699003490341024
rbf 4 6 0.7 : -0.014987202741356542, 0.0032186623484837806
rbf 4 6 0.9 : -0.014054037342419634, 0.00261395757040761
sigmoid 1 1 0.1 : -0.027851666283777155, -
0.00048176599173288537
sigmoid 1 1 0.3 : -0.028038579705349464, -
0.00015274351596450408
sigmoid 1 1 0.5 : -0.02932762945662799, -8.394825546287343e-05
sigmoid 1 1 0.7 : -0.02737075119194312, -0.00015274501835849108
sigmoid 1 1 0.9 : -0.027425878739029484, -0.0003516196183293019
sigmoid 1 2 0.1 : -0.027851574944466682, -0.0004817589183876425
sigmoid 1 2 0.3 : -0.02803863742481001, -0.0001527348653924676
sigmoid 1 2 0.5 : -0.029327682929571487, -8.393840801601549e-05
sigmoid 1 2 0.7 : -0.027370781055516956, -
0.00015273486539224557
sigmoid 1 2 0.9 : -0.027425939282075217, -0.0003516451205780591
sigmoid 1 3 0.1 : -0.02785157494475108, -0.0004817589182650739
sigmoid 1 3 0.3 : -0.028038637425086766, -0.0001527348652718974
sigmoid 1 3 0.5 : -0.029327682929847977, -8.39384078961114e-05
sigmoid 1 3 0.7 : -0.027370781055785543, -0.0001527348652718974
sigmoid 1 3 0.9 : -0.02742593928234638, -0.0003516451204561566
sigmoid 1 4 0.1 : -0.02785157494475108, -0.0004817589182650739
sigmoid 1 4 0.3 : -0.028038637425086766, -0.0001527348652718974
sigmoid 1 4 0.5 : -0.029327682929847977, -8.39384078961114e-05
sigmoid 1 4 0.7 : -0.027370781055785543, -0.0001527348652718974
sigmoid 1 4 0.9 : -0.02742593928234638, -0.0003516451204561566
sigmoid 1 5 0.1 : -0.02785157494475108, -0.0004817589182650739
sigmoid 1 5 0.3 : -0.028038637425086766, -0.0001527348652718974
sigmoid 1 5 0.5 : -0.029327682929847977, -8.39384078961114e-05
sigmoid 1 5 0.7 : -0.027370781055785543, -0.0001527348652718974
sigmoid 1 5 0.9 : -0.02742593928234638, -0.0003516451204561566
sigmoid 1 6 0.1 : -0.02785157494475108, -0.0004817589182650739
sigmoid 1 6 0.3 : -0.028038637425086766, -0.0001527348652718974
sigmoid 1 6 0.5 : -0.029327682929847977, -8.39384078961114e-05
sigmoid 1 6 0.7 : -0.027370781055785543, -0.0001527348652718974
sigmoid 1 6 0.9 : -0.02742593928234638, -0.0003516451204561566
sigmoid 2 1 0.1 : -0.027851757623764774, -0.0004817730655040098
sigmoid 2 1 0.3 : -0.028038521986069664, -
0.00015275216695753713
sigmoid 2 1 0.5 : -0.029327575983875143, -8.395810332584297e-05
sigmoid 2 1 0.7 : -0.0273707213286317, -0.00015275517174151432
sigmoid 2 1 0.9 : -0.027425818196180795, -
0.00035159411828966647
sigmoid 2 2 0.1 : -0.027851574944182157, -0.0004817589185099891
sigmoid 2 2 0.3 : -0.028038637424533074, -0.0001527348655128158
sigmoid 2 2 0.5 : -0.02932768292929495, -8.393840813569753e-05
sigmoid 2 2 0.7 : -0.027370781055248282, -0.0001527348655123717
sigmoid 2 2 0.9 : -0.0274259392818041, -0.0003516451207001836
sigmoid 2 3 0.1 : -0.02785157494475108, -0.0004817589182650739
sigmoid 2 3 0.3 : -0.028038637425086766, -0.0001527348652718974
sigmoid 2 3 0.5 : -0.029327682929847977, -8.39384078961114e-05
sigmoid 2 3 0.7 : -0.027370781055785543, -0.0001527348652718974
sigmoid 2 3 0.9 : -0.02742593928234638, -0.0003516451204561566
sigmoid 2 4 0.1 : -0.02785157494475108, -0.0004817589182650739
sigmoid 2 4 0.3 : -0.028038637425086766, -0.0001527348652718974
sigmoid 2 4 0.5 : -0.029327682929847977, -8.39384078961114e-05
sigmoid 2 4 0.7 : -0.027370781055785543, -0.0001527348652718974
sigmoid 2 4 0.9 : -0.02742593928234638, -0.0003516451204561566
sigmoid 2 5 0.1 : -0.02785157494475108, -0.0004817589182650739
sigmoid 2 5 0.3 : -0.028038637425086766, -0.0001527348652718974
sigmoid 2 5 0.5 : -0.029327682929847977, -8.39384078961114e-05
sigmoid 2 5 0.7 : -0.027370781055785543, -0.0001527348652718974
sigmoid 2 5 0.9 : -0.02742593928234638, -0.0003516451204561566
sigmoid 2 6 0.1 : -0.02785157494475108, -0.0004817589182650739
sigmoid 2 6 0.3 : -0.028038637425086766, -0.0001527348652718974
sigmoid 2 6 0.5 : -0.029327682929847977, -8.39384078961114e-05
sigmoid 2 6 0.7 : -0.027370781055785543, -0.0001527348652718974
sigmoid 2 6 0.9 : -0.02742593928234638, -0.0003516451204561566
sigmoid 3 1 0.1 : -0.027851848964713977, -0.000481780139577781
sigmoid 3 1 0.3 : -0.028038464267246876, -
0.00015276081825010834
sigmoid 3 1 0.5 : -0.02932752251158872, -8.396795148502001e-05
sigmoid 3 1 0.7 : -0.027370691465850604, -0.0001527653254209671
sigmoid 3 1 0.9 : -0.027425757653800266, -0.0003515686203361401
sigmoid 3 2 0.1 : -0.02785157494389785, -0.0004817589186325577
sigmoid 3 2 0.3 : -0.02803863742425645, -0.00015273486563316396
sigmoid 3 2 0.5 : -0.029327682929018505, -8.393840825560162e-05
sigmoid 3 2 0.7 : -0.027370781054979965, -
0.00015273486563294192
sigmoid 3 2 0.9 : -0.02742593928153316, -0.0003516451208220861
sigmoid 3 3 0.1 : -0.02785157494475108, -0.0004817589182650739
sigmoid 3 3 0.3 : -0.028038637425086766, -0.0001527348652718974
sigmoid 3 3 0.5 : -0.029327682929847977, -8.39384078961114e-05
sigmoid 3 3 0.7 : -0.027370781055785543, -0.0001527348652718974
sigmoid 3 3 0.9 : -0.02742593928234638, -0.0003516451204561566
sigmoid 3 4 0.1 : -0.02785157494475108, -0.0004817589182650739
sigmoid 3 4 0.3 : -0.028038637425086766, -0.0001527348652718974
sigmoid 3 4 0.5 : -0.029327682929847977, -8.39384078961114e-05
sigmoid 3 4 0.7 : -0.027370781055785543, -0.0001527348652718974
sigmoid 3 4 0.9 : -0.02742593928234638, -0.0003516451204561566
sigmoid 3 5 0.1 : -0.02785157494475108, -0.0004817589182650739
sigmoid 3 5 0.3 : -0.028038637425086766, -0.0001527348652718974
sigmoid 3 5 0.5 : -0.029327682929847977, -8.39384078961114e-05
sigmoid 3 5 0.7 : -0.027370781055785543, -0.0001527348652718974
sigmoid 3 5 0.9 : -0.02742593928234638, -0.0003516451204561566
sigmoid 3 6 0.1 : -0.02785157494475108, -0.0004817589182650739
sigmoid 3 6 0.3 : -0.028038637425086766, -0.0001527348652718974
sigmoid 3 6 0.5 : -0.029327682929847977, -8.39384078961114e-05

sigmoid 3 6 0.7 : -0.027370781055785543, -0.0001527348652718974
sigmoid 3 6 0.9 : -0.02742593928234638, -0.0003516451204561566
sigmoid 4 1 0.1 : -0.027851940306625834, -0.0004817872139561974
sigmoid 4 1 0.3 : -0.02803840654888239, -0.0001527694698443818
sigmoid 4 1 0.5 : -0.029327469039770414, -8.397779994195886e-05
sigmoid 4 1 0.7 : -0.027370661603600865, -0.0001527754793977376
sigmoid 4 1 0.9 : -0.02742569711188856, -0.0003515431244711653
sigmoid 4 2 0.1 : -0.027851574943613365, -0.00048175891875468224
sigmoid 4 2 0.3 : -0.028038637423979475, -0.00015273486575395623
sigmoid 4 2 0.5 : -0.029327682928741883, -8.393840837572775e-05
sigmoid 4 2 0.7 : -0.027370781054711336, -0.0001527348657532901
sigmoid 4 2 0.9 : -0.027425939281261868, -0.0003516451209439886
sigmoid 4 3 0.1 : -0.02785157494475108, -0.0004817589182650739
sigmoid 4 3 0.3 : -0.028038637425086766, -0.0001527348652718974
sigmoid 4 3 0.5 : -0.029327682929847977, -8.39384078961114e-05
sigmoid 4 3 0.7 : -0.027370781055785543, -0.0001527348652718974
sigmoid 4 3 0.9 : -0.02742593928234638, -0.0003516451204561566
sigmoid 4 4 0.1 : -0.02785157494475108, -0.0004817589182650739
sigmoid 4 4 0.3 : -0.028038637425086766, -0.0001527348652718974
sigmoid 4 4 0.5 : -0.029327682929847977, -8.39384078961114e-05
sigmoid 4 4 0.7 : -0.027370781055785543, -0.0001527348652718974
sigmoid 4 4 0.9 : -0.02742593928234638, -0.0003516451204561566
sigmoid 4 5 0.1 : -0.02785157494475108, -0.0004817589182650739
sigmoid 4 5 0.3 : -0.028038637425086766, -0.0001527348652718974
sigmoid 4 5 0.5 : -0.029327682929847977, -8.39384078961114e-05
sigmoid 4 5 0.7 : -0.027370781055785543, -0.0001527348652718974
sigmoid 4 5 0.9 : -0.02742593928234638, -0.0003516451204561566
sigmoid 4 6 0.1 : -0.02785157494475108, -0.0004817589182650739
sigmoid 4 6 0.3 : -0.028038637425086766, -0.0001527348652718974
sigmoid 4 6 0.5 : -0.029327682929847977, -8.39384078961114e-05
sigmoid 4 6 0.7 : -0.027370781055785543, -0.0001527348652718974
sigmoid 4 6 0.9 : -0.02742593928234638, -0.0003516451204561566

Grid search of ElasticNet, Morgan(r=4)
ElasticNet(alpha= i, l1_ratio= j, max_iter=100000)
i j : accuracy of prediction using cross validation, accuracy of prediction using test data
0.001 0.0 : 0.8841099352366861, 0.9357607531581581
0.001 0.2 : 0.8868086388646897, 0.9392934025052001
0.001 0.4 : 0.8899422549207952, 0.9419308312279067
0.001 0.6 : 0.892190986882011, 0.9439377394843131
0.001 0.8 : 0.8927015450618985, 0.9471948738974874
0.001 1.0 : 0.8898632528369053, 0.9476868107050438
0.01 0.0 : 0.8742856887221706, 0.9260059168401777
0.01 0.2 : 0.8774982272444396, 0.9293197477176289
0.01 0.4 : 0.882007413831332, 0.931184800014732
0.01 0.6 : 0.8862028098421391, 0.932714977505907
0.01 0.8 : 0.8921191357274694, 0.9348010906935087
0.01 1.0 : 0.893774713063214, 0.9320607593701308
0.1 0.0 : 0.7943056167678663, 0.8330846754869311
0.1 0.2 : 0.7872426905117592, 0.8226808111924235
0.1 0.4 : 0.7838430283872306, 0.8137896003062834
0.1 0.6 : 0.7846405309019943, 0.8088858197954056
0.1 0.8 : 0.7931133690345956, 0.8086849335420097
0.1 1.0 : 0.8149906957535883, 0.8115668141246511

Grid search of RandomForest, Morgan(r=4)
RandomForestRegressor(n_estimators= i)
i: accuracy of prediction using cross validation, accuracy of prediction using test data
100 1.0 : 0.767695713591609, 0.8113096547579648
500 1.0 : 0.7701857623328542, 0.8066236040250777
1000 1.0 : 0.7678365595168545, 0.8115457946830973
2000 1.0 : 0.7678220056255293, 0.8167403138310062

Grid search of NeuralNetwork, Morgan(r=4)
MLPRegressor(activation=act, alpha=a, batch_size=batch, beta_1=0.9, beta_2=0.999, early_stopping=False, epsilon=1e-08, hidden_layer_sizes=hid, learning_rate='constant', learning_rate_init=0.001, max_iter=100000, momentum=0.9, n_iter_no_change=10, nesterovs_momentum=True, power_t=0.5, random_state=1, shuffle=True, solver='adam', tol=0.0001, validation_fraction=0.1, verbose=False, warm_start=False)
act (hid) a batch: accuracy of prediction using cross validation, accuracy of prediction using test data
relu (200,) 0.0001 500 : 0.7776960720334831, 0.8950310161883396
relu (200,) 0.0001 200 : 0.813988831480439, 0.8874032255355188
relu (200,) 0.0001 100 : 0.8149496113082673, 0.8908978342733129
relu (200,) 0.0001 50 : 0.8214739692438021, 0.895768360741513
relu (200,) 0.001 500 : 0.7779457006994288, 0.8951710640557358
relu (200,) 0.001 200 : 0.8141297288954666, 0.8875661425948319
relu (200,) 0.001 100 : 0.8151642894772623, 0.8913229363884849
relu (200,) 0.001 50 : 0.821851294857229, 0.8962953502331416
relu (200,) 0.01 500 : 0.778476750563977, 0.8970523714578462
relu (200,) 0.01 200 : 0.8162060791543626, 0.8882354053551051
relu (200,) 0.01 100 : 0.8180124840838309, 0.894039841262106
relu (200,) 0.01 50 : 0.8271222628704613, 0.9004050505511164
relu (200, 200) 0.0001 500 : 0.7841485694172564, 0.8952814851975572
relu (200, 200) 0.0001 200 : 0.8160400979062257, 0.8885193199453758
relu (200, 200) 0.0001 100 : 0.8186698450805467, 0.8957757403016807
relu (200, 200) 0.0001 50 : 0.8247181530624568, 0.9029693869238058
relu (200, 200) 0.001 500 : 0.7841371528172612, 0.8942603017089271
relu (200, 200) 0.001 200 : 0.8159893456749897, 0.8890790267751489
relu (200, 200) 0.001 100 : 0.8186919985238678, 0.8957221363710219
relu (200, 200) 0.001 50 : 0.8250094557562611, 0.9021715744277917
relu (200, 200) 0.01 500 : 0.7852670494702002, 0.8945845659696067
relu (200, 200) 0.01 200 : 0.8157691574997973, 0.890239429104516
relu (200, 200) 0.01 100 : 0.8194477769720306, 0.8955644469735355
relu (200, 200) 0.01 50 : 0.825186458717553, 0.9045744837583756
relu (100,) 0.0001 500 : 0.7703371160620521, 0.8972381480961394
relu (100,) 0.0001 200 : 0.8157857599330738, 0.8868968231369934
relu (100,) 0.0001 100 : 0.8164234075229573, 0.8917901977626154
relu (100,) 0.0001 50 : 0.8221268876760959, 0.8948554361840324
relu (100,) 0.001 500 : 0.7703850987418827, 0.897437832876633
relu (100,) 0.001 200 : 0.814272958568526, 0.8876773587189368
relu (100,) 0.001 100 : 0.8166801329701083, 0.8921088904615236
relu (100,) 0.001 50 : 0.8226006629159637, 0.8953101702801403
relu (100,) 0.01 500 : 0.7709307987548273, 0.8983969138513588
relu (100,) 0.01 200 : 0.8160542607885646, 0.8887186978529125
relu (100,) 0.01 100 : 0.8187108972427953, 0.8940360298955199
relu (100,) 0.01 50 : 0.8271040457811629, 0.899264324646666
relu (100, 100) 0.0001 500 : 0.7816299618268088, 0.8981701881889853
relu (100, 100) 0.0001 200 : 0.8139765241542678, 0.8913269661605006
relu (100, 100) 0.0001 100 : 0.8194278343798495, 0.8974056573166915
relu (100, 100) 0.0001 50 : 0.8261697042775484, 0.9029894560977394
relu (100, 100) 0.001 500 : 0.7810156139622676, 0.8977064425467554
relu (100, 100) 0.001 200 : 0.8139796660521359, 0.8908224476446425
relu (100, 100) 0.001 100 : 0.8189521632402792, 0.896483817878845
relu (100, 100) 0.001 50 : 0.8260665888424729, 0.9029807857644404
relu (100, 100) 0.01 500 : 0.7822195178301242, 0.8995478115180762
relu (100, 100) 0.01 200 : 0.8136736603846533, 0.8914630786118539
relu (100, 100) 0.01 100 : 0.8180669919249398, 0.896030460191008
relu (100, 100) 0.01 50 : 0.8281216179241919, 0.9025045935920574
relu (100, 200) 0.0001 500 : 0.7854500569932127, 0.8939395414643587

relu (100, 200) 0.0001 200 : 0.8213343209777413, 0.886765959043825
relu (100, 200) 0.0001 100 : 0.8185388097230122, 0.8927928894206828
relu (100, 200) 0.0001 50 : 0.8286618718635623, 0.9004819657845609
relu (100, 200) 0.001 500 : 0.7843612301185802, 0.889622766173033
relu (100, 200) 0.001 200 : 0.8218072300899069, 0.8869462818276471
relu (100, 200) 0.001 100 : 0.8199695998033372, 0.8941728420295382
relu (100, 200) 0.001 50 : 0.829112530989782, 0.9031085962632122
relu (100, 200) 0.01 500 : 0.7836852621594312, 0.8916664811427111
relu (100, 200) 0.01 200 : 0.8221943506887965, 0.8853490623356773
relu (100, 200) 0.01 100 : 0.8199651305230139, 0.8932045703878009
relu (100, 200) 0.01 50 : 0.8310646690962351, 0.9019326048820672
relu (50,) 0.0001 500 : 0.7749445915757178, 0.8965566539654255
relu (50,) 0.0001 200 : 0.8121175046632144, 0.8891267962596328
relu (50,) 0.0001 100 : 0.8128124313388512, 0.8916887438565192
relu (50,) 0.0001 50 : 0.8184492611963237, 0.8950404915011271
relu (50,) 0.001 500 : 0.7749912077648364, 0.8967123193602605
relu (50,) 0.001 200 : 0.8123668534588158, 0.8898570121588144
relu (50,) 0.001 100 : 0.8132188609441469, 0.8919997085896124
relu (50,) 0.001 50 : 0.8189506360066343, 0.8953996489047673
relu (50,) 0.01 500 : 0.7754905408681816, 0.8981550114839185
relu (50,) 0.01 200 : 0.8141735172772371, 0.8908453889425607
relu (50,) 0.01 100 : 0.8157090694579837, 0.8945298235131118
relu (50,) 0.01 50 : 0.8236861617922683, 0.8994505802201904
relu (50, 50) 0.0001 500 : 0.7821554674472783, 0.8879714285530174
relu (50, 50) 0.0001 200 : 0.8167369400586987, 0.8821532436021277
relu (50, 50) 0.0001 100 : 0.8139400878761928, 0.8908214507802571
relu (50, 50) 0.0001 50 : 0.8265370850585271, 0.899612677337299
relu (50, 50) 0.001 500 : 0.7817714222974607, 0.890160635002887
relu (50, 50) 0.001 200 : 0.8185167838051519, 0.8795405661321457
relu (50, 50) 0.001 100 : 0.8193502413931665, 0.8905461907015105
relu (50, 50) 0.001 50 : 0.8254324656843774, 0.8947095587391807
relu (50, 50) 0.01 500 : 0.7802411935978281, 0.8896490616521341
relu (50, 50) 0.01 200 : 0.8161483060990721, 0.8812402134418291
relu (50, 50) 0.01 100 : 0.820703992027774, 0.8921376147748126
relu (50, 50) 0.01 50 : 0.8289751791818236, 0.9006602102772212
relu (50, 100) 0.0001 500 : 0.7736047870597118, 0.8869549808327329
relu (50, 100) 0.0001 200 : 0.8161333529200283, 0.8856433053114466
relu (50, 100) 0.0001 100 : 0.8178847966692757, 0.895206205169611
relu (50, 100) 0.0001 50 : 0.8219100869570723, 0.8993614744668066
relu (50, 100) 0.001 500 : 0.7745358457105276, 0.8872884371084426
relu (50, 100) 0.001 200 : 0.8171179524298843, 0.8860700192785842
relu (50, 100) 0.001 100 : 0.818492611204177, 0.8929406339514275
relu (50, 100) 0.001 50 : 0.8212477724739851, 0.8980811027102394
relu (50, 100) 0.01 500 : 0.775224972240037, 0.8876862880713766
relu (50, 100) 0.01 200 : 0.8189644830394329, 0.88701236193766
relu (50, 100) 0.01 100 : 0.8171658908045168, 0.8912587489240581
relu (50, 100) 0.01 50 : 0.8218897954696102, 0.8991697334923864
relu (50, 200) 0.0001 500 : 0.7791617187270032, 0.8896468163738205
relu (50, 200) 0.0001 200 : 0.8164157144101308, 0.8810968950472464
relu (50, 200) 0.0001 100 : 0.8185680562181487, 0.8910382572959118
relu (50, 200) 0.0001 50 : 0.8224931214606913, 0.8948506732094021
relu (50, 200) 0.001 500 : 0.7793212602679332, 0.8877963499062179
relu (50, 200) 0.001 200 : 0.8161562888072738, 0.8816496681059389
relu (50, 200) 0.001 100 : 0.8189549685953981, 0.8901456761553324
relu (50, 200) 0.001 50 : 0.8255441769872961, 0.8934607030342281
relu (50, 200) 0.01 500 : 0.7790712204728545, 0.8911300305453123
relu (50, 200) 0.01 200 : 0.815324808239885, 0.8823507147075448
relu (50, 200) 0.01 100 : 0.8195297992508237, 0.8917002014099633
relu (50, 200) 0.01 50 : 0.8271158137393858, 0.8951987933822629
tanh (200,) 0.0001 500 : 0.8111678535065074, 0.8972017165369526
tanh (200,) 0.0001 200 : 0.837620472719071, 0.8903254570416339
tanh (200,) 0.0001 100 : 0.8380052149775183, 0.898406542393062
tanh (200,) 0.0001 50 : 0.8468304807099788, 0.9039530320086112
tanh (200,) 0.001 500 : 0.8116999981174067, 0.898403166656622
tanh (200,) 0.001 200 : 0.8387316786104199, 0.8915177382918376
tanh (200,) 0.001 100 : 0.8393655461530571, 0.8994705586491728
tanh (200,) 0.001 50 : 0.846289421248092, 0.9051359677859827
tanh (200,) 0.01 500 : 0.8168821789235204, 0.9070926529881388
tanh (200,) 0.01 200 : 0.8469518395724812, 0.903223487972396
tanh (200,) 0.01 100 : 0.8471373315772384, 0.9064437279619652
tanh (200,) 0.01 50 : 0.8507405314689187, 0.9106972986332382
tanh (200, 200) 0.0001 500 : 0.7929884717181823, -0.02084621004539189
tanh (200, 200) 0.0001 200 : 0.4919499242954826, 0.8909420181514315
tanh (200, 200) 0.0001 100 : 0.8323719167530268, 0.8980756132899199
tanh (200, 200) 0.0001 50 : 0.8410375127760895, 0.9048291991773919
tanh (200, 200) 0.001 500 : 0.7941332178351377, -0.020845889037345744
tanh (200, 200) 0.001 200 : 0.49262346836021714, 0.8914468575464161
tanh (200, 200) 0.001 100 : 0.8345363281676585, 0.8984930353604207
tanh (200, 200) 0.001 50 : 0.8411066169609407, 0.9051696182762816
tanh (200, 200) 0.01 500 : 0.8042032856715936, -0.020842761998645987
tanh (200, 200) 0.01 200 : 0.49643044228179667, 0.8979256464815408
tanh (200, 200) 0.01 100 : 0.840433998479142, 0.9022662152651937
tanh (200, 200) 0.01 50 : 0.8507541205876684, 0.9097409790405424
tanh (100,) 0.0001 500 : 0.80128786866648, 0.9009606280089351
tanh (100,) 0.0001 200 : 0.8355126227559202, 0.8942506173344843
tanh (100,) 0.0001 100 : 0.8368489006001516, 0.9029035010156887
tanh (100,) 0.0001 50 : 0.8487248094094729, 0.9117421225158853
tanh (100,) 0.001 500 : 0.8029384800406689, 0.901442196871229
tanh (100,) 0.001 200 : 0.8341418687350816, 0.8971604629168403
tanh (100,) 0.001 100 : 0.8378868682601663, 0.9069419242254074
tanh (100,) 0.001 50 : 0.8498995619135155, 0.9111472125269947
tanh (100,) 0.01 500 : 0.8127749185998546, 0.9120492948799344
tanh (100,) 0.01 200 : 0.8426142967399782, 0.9111664919256603
tanh (100,) 0.01 100 : 0.8451645379468079, 0.9134861140562331
tanh (100,) 0.01 50 : 0.8549910100764441, 0.9168114097137764
tanh (100, 100) 0.0001 500 : 0.799148444228748, 0.897969902034861
tanh (100, 100) 0.0001 200 : 0.8326029391296071, 0.886035232248912
tanh (100, 100) 0.0001 100 : 0.8385666819621885, 0.9025318682946206
tanh (100, 100) 0.0001 50 : 0.847414057877897, 0.9039837677999636
tanh (100, 100) 0.001 500 : 0.7981473508042807, 0.9010669830900098
tanh (100, 100) 0.001 200 : 0.8336541492600507, 0.8874448444559836
tanh (100, 100) 0.001 100 : 0.8423639903238822, 0.9037421454644549
tanh (100, 100) 0.001 50 : 0.849145086190467, 0.909713108992564
tanh (100, 100) 0.01 500 : 0.8123488269838166, 0.9097173468504296
tanh (100, 100) 0.01 200 : 0.8422605236831429,

0.8922309179973728
tanh (100, 100) 0.01 100 : 0.8455699656617846, 0.9098681099673745
tanh (100, 100) 0.01 50 : 0.8532450426894901, 0.9170933818761604
tanh (100, 200) 0.0001 500 : 0.7917984858263621, 0.8958588910204576
tanh (100, 200) 0.0001 200 : 0.8313480383618721, 0.8846240246423124
tanh (100, 200) 0.0001 100 : 0.8349252303705527, 0.8939362970789821
tanh (100, 200) 0.0001 50 : 0.843014506128729, 0.9015930326141077
tanh (100, 200) 0.001 500 : 0.7923292716492234, 0.895914557977537
tanh (100, 200) 0.001 200 : 0.8318883478827148, 0.884898152981654
tanh (100, 200) 0.001 100 : 0.8352608997359667, 0.8957430524854287
tanh (100, 200) 0.001 50 : 0.8409771330031648, 0.9035730643599833
tanh (100, 200) 0.01 500 : 0.7971455661415714, 0.8972249821744528
tanh (100, 200) 0.01 200 : 0.8361043344866937, 0.8882013665310539
tanh (100, 200) 0.01 100 : 0.8398178325940018, 0.8996864640826939
tanh (100, 200) 0.01 50 : 0.8456628994124422, 0.9092925201757431
tanh (50,) 0.0001 500 : 0.7942494443520098, 0.8930521734836574
tanh (50,) 0.0001 200 : 0.8251996467618241, 0.8910568785080356
tanh (50,) 0.0001 100 : 0.8262467283126302, 0.9030642452250538
tanh (50,) 0.0001 50 : 0.8356376132410162, 0.9001829197745614
tanh (50,) 0.001 500 : 0.797484053245752, 0.8988378117063371
tanh (50,) 0.001 200 : 0.8239929570146536, 0.8957287783011794
tanh (50,) 0.001 100 : 0.8274020965345812, 0.9057139029201113
tanh (50,) 0.001 50 : 0.8418231532173138, 0.9100144039387972
tanh (50,) 0.01 500 : 0.8141251537418608, 0.9088308756539984
tanh (50,) 0.01 200 : 0.8403986780862331, 0.9125888898585304
tanh (50,) 0.01 100 : 0.8386402761947063, 0.9181302084539249
tanh (50,) 0.01 50 : 0.8505027422669647, 0.9149139913892517
tanh (50, 50) 0.0001 500 : 0.7817155486820331, 0.8911676709322592
tanh (50, 50) 0.0001 200 : 0.82217937426909, 0.8913366876300453
tanh (50, 50) 0.0001 100 : 0.8312755012168911, 0.893131840984809
tanh (50, 50) 0.0001 50 : 0.8357339369870331, 0.8984980371095364
tanh (50, 50) 0.001 500 : 0.7838415954015027, 0.8973802454861557
tanh (50, 50) 0.001 200 : 0.825163810577078, 0.8939662670041024
tanh (50, 50) 0.001 100 : 0.8316347495849532, 0.9021789607423779
tanh (50, 50) 0.001 50 : 0.8382090194155006, 0.9079298633443942
tanh (50, 50) 0.01 500 : 0.7987153275130516, 0.9016132335127235
tanh (50, 50) 0.01 200 : 0.8441264868932284, 0.9086386081791501
tanh (50, 50) 0.01 100 : 0.8478858733299621, 0.9067005419211259
tanh (50, 50) 0.01 50 : 0.848440625313968, 0.9184690510391106
tanh (50, 100) 0.0001 500 : 0.7823500633341575, 0.8911233332637154
tanh (50, 100) 0.0001 200 : 0.8316757634753863, 0.8895806505266081
tanh (50, 100) 0.0001 100 : 0.8326893076146634, 0.8969840747098987
tanh (50, 100) 0.0001 50 : 0.8395385524253346, 0.9072934883161765
tanh (50, 100) 0.001 500 : 0.7829767124059099, 0.891185815302102
tanh (50, 100) 0.001 200 : 0.8307755901440693, 0.8930931762088463
tanh (50, 100) 0.001 100 : 0.8344232558939731, 0.899875077966523
tanh (50, 100) 0.001 50 : 0.8424704860725015, 0.9069401966884356
tanh (50, 100) 0.01 500 : 0.7915038943601647, 0.8928633655457745
tanh (50, 100) 0.01 200 : 0.838019440583999, 0.8913129811728333
tanh (50, 100) 0.01 100 : 0.8415570136141961, 0.9048477926806788
tanh (50, 100) 0.01 50 : 0.8485182832166134, 0.911896109378308
tanh (50, 200) 0.0001 500 : 0.6387092428170349, 0.8824580674470548
tanh (50, 200) 0.0001 200 : 0.8316704096159342, 0.8848340983185413
tanh (50, 200) 0.0001 100 : 0.8343927101005819, 0.8908828871550255
tanh (50, 200) 0.0001 50 : 0.8456220246798523, 0.897514480256046
tanh (50, 200) 0.001 500 : 0.6389340681248431, 0.882755844635247
tanh (50, 200) 0.001 200 : 0.831930198530064, 0.8851214402101846
tanh (50, 200) 0.001 100 : 0.8350298547303717, 0.8910965948454066
tanh (50, 200) 0.001 50 : 0.8463366525190015, 0.8975587589898901
tanh (50, 200) 0.01 500 : 0.6410770816723529, 0.8852351680475166
tanh (50, 200) 0.01 200 : 0.8323204149946944, 0.8870871455439111
tanh (50, 200) 0.01 100 : 0.8410102968442608, 0.8930290017066018
tanh (50, 200) 0.01 50 : 0.8447084699802527, 0.9008963297663601
logistic (200,) 0.0001 500 : 0.8026912510168437, 0.900743799175453
logistic (200,) 0.0001 200 : 0.8389166522109137, 0.8909356495867724
logistic (200,) 0.0001 100 : 0.8398463421743066, 0.8998163270579785
logistic (200,) 0.0001 50 : 0.8474707913710195, 0.9058232664607316
logistic (200,) 0.001 500 : 0.8051702359904196, 0.9050871207572269
logistic (200,) 0.001 200 : 0.8437072903849374, 0.8957013179819306
logistic (200,) 0.001 100 : 0.845250577819273, 0.9066437444706316
logistic (200,) 0.001 50 : 0.8520465688706913, 0.9106329864118564
logistic (200,) 0.01 500 : 0.824826122962983, 0.9217216433567081
logistic (200,) 0.01 200 : 0.8649038028351251, 0.9228135007048068
logistic (200,) 0.01 100 : 0.8655040443060479, 0.9254301739848821
logistic (200,) 0.01 50 : 0.8650199314058368, 0.923414578646877
logistic (200, 200) 0.0001 500 : 0.7862565335874052, 0.8900358415177652
logistic (200, 200) 0.0001 200 : 0.8251984179593534, 0.8795846645533661
logistic (200, 200) 0.0001 100 : 0.8224501050905868, 0.8976520295679666
logistic (200, 200) 0.0001 50 : 0.8519997614636416, 0.9038311657363103
logistic (200, 200) 0.001 500 : 0.7947636181491349, 0.8951162830143631
logistic (200, 200) 0.001 200 : 0.828471951171838, 0.8892659819496445
logistic (200, 200) 0.001 100 : 0.8295993448133652, 0.9032432858707924
logistic (200, 200) 0.001 50 : 0.8588128670029823, 0.9100498243979372
logistic (200, 200) 0.01 500 : 0.8290937929220521, 0.9151708718279707
logistic (200, 200) 0.01 200 : 0.8574327633594244, 0.9067962416871349
logistic (200, 200) 0.01 100 : 0.8613832388466551, 0.9144355473097733
logistic (200, 200) 0.01 50 : 0.875888809294042, 0.9250703431645375
logistic (100,) 0.0001 500 : 0.7904372964572609, 0.89798517820943
logistic (100,) 0.0001 200 : 0.8291541112633736, 0.885332507843566
logistic (100,) 0.0001 100 : 0.8312197266092658, 0.8988826652111856
logistic (100,) 0.0001 50 : 0.8389826204537398, 0.906094808288643
logistic (100,) 0.001 500 : 0.7948660013223974, 0.9046054528207952
logistic (100,) 0.001 200 : 0.836499741650532, 0.8959958129715022
logistic (100,) 0.001 100 : 0.8390639875564865, 0.904039755729629
logistic (100,) 0.001 50 : 0.8458121142098933, 0.9083841981291165
logistic (100,) 0.01 500 : 0.8270526913245521, 0.9176830620027
logistic (100,) 0.01 200 : 0.8578762238210172, 0.9173816675567492
logistic (100,) 0.01 100 : 0.8603488946150424, 0.9225461567156376
logistic (100,) 0.01 50 : 0.8653682202648234, 0.9273563878090301
logistic (100, 100) 0.0001 500 : 0.7826717650525176, 0.8791000204522861
logistic (100, 100) 0.0001 200 : 0.8106254502934505, 0.8655356629319305
logistic (100, 100) 0.0001 100 : 0.8231887725279472, 0.8856694075743753

logistic (100, 100) 0.0001 50 : 0.8346254632676733,
0.8960103880795024
logistic (100, 100) 0.001 500 : 0.7911537901284105,
0.8883990477374508
logistic (100, 100) 0.001 200 : 0.8178767450479638,
0.8819874615548662
logistic (100, 100) 0.001 100 : 0.8287552019117133,
0.8936340725138494
logistic (100, 100) 0.001 50 : 0.8436101605775951,
0.9064924249273736
logistic (100, 100) 0.01 500 : 0.8167423788431927,
0.9060145291006699
logistic (100, 100) 0.01 200 : 0.8372869225765832,
0.8949525505325792
logistic (100, 100) 0.01 100 : 0.8429306716737088,
0.9158814178900714
logistic (100, 100) 0.01 50 : 0.8538664203301938,
0.9182664781864299
logistic (100, 200) 0.0001 500 : 0.7768523045103972,
0.8665885391216257
logistic (100, 200) 0.0001 200 : 0.8015058469461879,
0.86694149255265
logistic (100, 200) 0.0001 100 : 0.8149783213839223,
0.8784519986320238
logistic (100, 200) 0.0001 50 : 0.8282227301607842,
0.8910812735594107
logistic (100, 200) 0.001 500 : 0.7818989724305118,
0.8682910565456373
logistic (100, 200) 0.001 200 : 0.8039499697034541,
0.8717241681332464
logistic (100, 200) 0.001 100 : 0.808097687900583,
0.8699019961037858
logistic (100, 200) 0.001 50 : 0.8317649262707671,
0.8962226191775586
logistic (100, 200) 0.01 500 : 0.8056800942025832, -
0.026474455680523645
logistic (100, 200) 0.01 200 : 0.8109124457920457,
0.8894739314745639
logistic (100, 200) 0.01 100 : 0.8250462145876067,
0.8933897716504222
logistic (100, 200) 0.01 50 : 0.8440550629166379,
0.9107670574106901
logistic (50,) 0.0001 500 : 0.8060933787874429,
0.8940881431137457
logistic (50,) 0.0001 200 : 0.8378134177142913,
0.8882454206439507
logistic (50,) 0.0001 100 : 0.837944660365449, 0.9025551696250284
logistic (50,) 0.0001 50 : 0.8445241088107733, 0.9080913646032622
logistic (50,) 0.001 500 : 0.8138857212206773, 0.9034102989726329
logistic (50,) 0.001 200 : 0.8441918851602768, 0.9023422296761269
logistic (50,) 0.001 100 : 0.840383013281004, 0.910183002550275
logistic (50,) 0.001 50 : 0.8514854292376157, 0.9145156771482026
logistic (50,) 0.01 500 : 0.8427707555710997, 0.9184177064748331
logistic (50,) 0.01 200 : 0.8563694851799969, 0.9159264445949858
logistic (50,) 0.01 100 : 0.8628961753534405, 0.9231784898702737
logistic (50,) 0.01 50 : 0.8650922683539193, 0.9315442137882968
logistic (50, 50) 0.0001 500 : 0.7963998480092526,
0.8821108234576129
logistic (50, 50) 0.0001 200 : 0.8239677234731273,
0.8678101499447612
logistic (50, 50) 0.0001 100 : 0.8322192224993336,
0.8978900630266473
logistic (50, 50) 0.0001 50 : 0.8343405211005228,
0.9036011972790525
logistic (50, 50) 0.001 500 : 0.8074327016687857,
0.8885377104630326
logistic (50, 50) 0.001 200 : 0.8210273990291788,
0.8814755471136617
logistic (50, 50) 0.001 100 : 0.8421093868285157,
0.8927665561354488
logistic (50, 50) 0.001 50 : 0.8438872470799964,
0.9128967031696368
logistic (50, 50) 0.01 500 : 0.8169370856947541,
0.9158502437151013
logistic (50, 50) 0.01 200 : 0.8449716339205902,
0.9003496728053307
logistic (50, 50) 0.01 100 : 0.8476205443218298,
0.9172523093105175
logistic (50, 50) 0.01 50 : 0.8676820496848517, 0.916852985389052
logistic (50, 100) 0.0001 500 : 0.786777380487177,
0.8861399220436753
logistic (50, 100) 0.0001 200 : 0.6324645062047238,
0.8737722359296232
logistic (50, 100) 0.0001 100 : 0.8274871795093546,
0.8875247951846099
logistic (50, 100) 0.0001 50 : 0.8431174557233032,
0.8962087560770357
logistic (50, 100) 0.001 500 : 0.7937556916398367, -
0.021481651425574233
logistic (50, 100) 0.001 200 : 0.64862864092297,
0.879164523925296
logistic (50, 100) 0.001 100 : 0.6488715880001833,
0.8887795570759557
logistic (50, 100) 0.001 50 : 0.8389591662148128,
0.9004644628240397
logistic (50, 100) 0.01 500 : 0.8193225105725883, -
0.0214658199905593
logistic (50, 100) 0.01 200 : 0.3028389090011057,
0.9005600815471264
logistic (50, 100) 0.01 100 : 0.8386510092301862,
0.9064992799435214
logistic (50, 100) 0.01 50 : 0.8512003277690875,
0.9074728906762585
logistic (50, 200) 0.0001 500 : 0.7643095324268676,
0.8801698502770141
logistic (50, 200) 0.0001 200 : 0.6212197936314459,
0.8655945270813308
logistic (50, 200) 0.0001 100 : 0.8120549430383439,
0.8814682689146354
logistic (50, 200) 0.0001 50 : 0.8225463586145733,
0.8893511133134453
logistic (50, 200) 0.001 500 : 0.7692911139890753,
0.8814630440631361
logistic (50, 200) 0.001 200 : 0.6243404221748814,
0.870811202248239
logistic (50, 200) 0.001 100 : 0.8155978259683216,
0.8819713124308641
logistic (50, 200) 0.001 50 : 0.830167121716574,
0.8915859968579278
logistic (50, 200) 0.01 500 : 0.7987941032266603,
0.8940760916360196
logistic (50, 200) 0.01 200 : 0.47228917320465735,
0.8905045792515947
logistic (50, 200) 0.01 100 : 0.8359019122884733,
0.8952406100740798
logistic (50, 200) 0.01 50 : 0.8359222879616752,
0.9009047151321558

Grid search of LightGBM, Morgan(r=4)
lgb.LGBMRegressor(boosting_type = "gbdt", num_leaves = j,max_depth = 0)
j: accuracy of prediction using cross validation, accuracy of prediction using test data
10 : 0.7853780667661746, 0.8496815332806869
50 : 0.78326399772228, 0.855639883420542
100 : 0.78326399772228, 0.855639883420542
150 : 0.78326399772228, 0.855639883420542

Grid search of SVR, Morgan(r=4, 1024)
SVR(C= c_num, kernel = ker, epsilon = e, gamma = r,degree = 3, coef0=1)
Ker c_num r e: accuracy of prediction using cross validation, accuracy of prediction using test data
linear 1 1 0.1 : 0.873683894062605, 0.9351571322788231
linear 1 1 0.3 : 0.8741124013016656, 0.9356077086596957
linear 1 1 0.5 : 0.8739887313003913, 0.9356436472523848
linear 1 1 0.7 : 0.8736091073278139, 0.9352710672659027
linear 1 1 0.9 : 0.87294135340561, 0.9347711028508674

linear 1 2 0.1 : 0.873683894062605, 0.9351571322788231
linear 1 2 0.3 : 0.8741124013016656, 0.9356077086596957
linear 1 2 0.5 : 0.8739887313003913, 0.9356436472523848
linear 1 2 0.7 : 0.8736091073278139, 0.9352710672659027
linear 1 2 0.9 : 0.87294135340561, 0.9347711028508674
linear 1 3 0.1 : 0.873683894062605, 0.9351571322788231
linear 1 3 0.3 : 0.8741124013016656, 0.9356077086596957
linear 1 3 0.5 : 0.8739887313003913, 0.9356436472523848
linear 1 3 0.7 : 0.8736091073278139, 0.9352710672659027
linear 1 3 0.9 : 0.87294135340561, 0.9347711028508674
linear 1 4 0.1 : 0.873683894062605, 0.9351571322788231
linear 1 4 0.3 : 0.8741124013016656, 0.9356077086596957
linear 1 4 0.5 : 0.8739887313003913, 0.9356436472523848
linear 1 4 0.7 : 0.8736091073278139, 0.9352710672659027
linear 1 4 0.9 : 0.87294135340561, 0.9347711028508674
linear 1 5 0.1 : 0.873683894062605, 0.9351571322788231
linear 1 5 0.3 : 0.8741124013016656, 0.9356077086596957
linear 1 5 0.5 : 0.8739887313003913, 0.9356436472523848
linear 1 5 0.7 : 0.8736091073278139, 0.9352710672659027
linear 1 5 0.9 : 0.87294135340561, 0.9347711028508674
linear 1 6 0.1 : 0.873683894062605, 0.9351571322788231
linear 1 6 0.3 : 0.8741124013016656, 0.9356077086596957
linear 1 6 0.5 : 0.8739887313003913, 0.9356436472523848
linear 1 6 0.7 : 0.8736091073278139, 0.9352710672659027
linear 1 6 0.9 : 0.87294135340561, 0.9347711028508674
linear 2 1 0.1 : 0.8831911249218788, 0.937071533572022
linear 2 1 0.3 : 0.8832284272653969, 0.9372943959478759
linear 2 1 0.5 : 0.882754409303859, 0.9368906869615834
linear 2 1 0.7 : 0.8821498341723715, 0.9358723384594122
linear 2 1 0.9 : 0.8811761365005653, 0.934457790769197
linear 2 2 0.1 : 0.8831911249218788, 0.937071533572022
linear 2 2 0.3 : 0.8832284272653969, 0.9372943959478759
linear 2 2 0.5 : 0.882754409303859, 0.9368906869615834
linear 2 2 0.7 : 0.8821498341723715, 0.9358723384594122
linear 2 2 0.9 : 0.8811761365005653, 0.934457790769197
linear 2 3 0.1 : 0.8831911249218788, 0.937071533572022
linear 2 3 0.3 : 0.8832284272653969, 0.9372943959478759
linear 2 3 0.5 : 0.882754409303859, 0.9368906869615834
linear 2 3 0.7 : 0.8821498341723715, 0.9358723384594122
linear 2 3 0.9 : 0.8811761365005653, 0.934457790769197
linear 2 4 0.1 : 0.8831911249218788, 0.937071533572022
linear 2 4 0.3 : 0.8832284272653969, 0.9372943959478759
linear 2 4 0.5 : 0.882754409303859, 0.9368906869615834
linear 2 4 0.7 : 0.8821498341723715, 0.9358723384594122
linear 2 4 0.9 : 0.8811761365005653, 0.934457790769197
linear 2 5 0.1 : 0.8831911249218788, 0.937071533572022
linear 2 5 0.3 : 0.8832284272653969, 0.9372943959478759
linear 2 5 0.5 : 0.882754409303859, 0.9368906869615834
linear 2 5 0.7 : 0.8821498341723715, 0.9358723384594122
linear 2 5 0.9 : 0.8811761365005653, 0.934457790769197
linear 2 6 0.1 : 0.8831911249218788, 0.937071533572022
linear 2 6 0.3 : 0.8832284272653969, 0.9372943959478759
linear 2 6 0.5 : 0.882754409303859, 0.9368906869615834
linear 2 6 0.7 : 0.8821498341723715, 0.9358723384594122
linear 2 6 0.9 : 0.8811761365005653, 0.934457790769197
linear 3 1 0.1 : 0.8846725147964959, 0.9352764646489975
linear 3 1 0.3 : 0.8847023627631891, 0.9359326737712877
linear 3 1 0.5 : 0.8841849901312411, 0.9356952402833539
linear 3 1 0.7 : 0.8834878787107083, 0.9350268875780259
linear 3 1 0.9 : 0.8823953658660137, 0.933934615953797
linear 3 2 0.1 : 0.8846725147964959, 0.9352764646489975
linear 3 2 0.3 : 0.8847023627631891, 0.9359326737712877
linear 3 2 0.5 : 0.8841849901312411, 0.9356952402833539
linear 3 2 0.7 : 0.8834878787107083, 0.9350268875780259
linear 3 2 0.9 : 0.8823953658660137, 0.933934615953797
linear 3 3 0.1 : 0.8846725147964959, 0.9352764646489975
linear 3 3 0.3 : 0.8847023627631891, 0.9359326737712877
linear 3 3 0.5 : 0.8841849901312411, 0.9356952402833539
linear 3 3 0.7 : 0.8834878787107083, 0.9350268875780259
linear 3 3 0.9 : 0.8823953658660137, 0.933934615953797
linear 3 4 0.1 : 0.8846725147964959, 0.9352764646489975
linear 3 4 0.3 : 0.8847023627631891, 0.9359326737712877
linear 3 4 0.5 : 0.8841849901312411, 0.9356952402833539
linear 3 4 0.7 : 0.8834878787107083, 0.9350268875780259
linear 3 4 0.9 : 0.8823953658660137, 0.933934615953797
linear 3 5 0.1 : 0.8846725147964959, 0.9352764646489975
linear 3 5 0.3 : 0.8847023627631891, 0.9359326737712877
linear 3 5 0.5 : 0.8841849901312411, 0.9356952402833539
linear 3 5 0.7 : 0.8834878787107083, 0.9350268875780259
linear 3 5 0.9 : 0.8823953658660137, 0.933934615953797
linear 3 6 0.1 : 0.8846725147964959, 0.9352764646489975
linear 3 6 0.3 : 0.8847023627631891, 0.9359326737712877
linear 3 6 0.5 : 0.8841849901312411, 0.9356952402833539
linear 3 6 0.7 : 0.8834878787107083, 0.9350268875780259
linear 3 6 0.9 : 0.8823953658660137, 0.933934615953797
linear 4 1 0.1 : 0.8847477454915926, 0.9347323513461686
linear 4 1 0.3 : 0.8847378515214659, 0.9355341761405427
linear 4 1 0.5 : 0.8842145947698574, 0.9352657927058106
linear 4 1 0.7 : 0.8836339692546554, 0.934497457486268
linear 4 1 0.9 : 0.8825320443369236, 0.9335216305253994
linear 4 2 0.1 : 0.8847477454915926, 0.9347323513461686
linear 4 2 0.3 : 0.8847378515214659, 0.9355341761405427
linear 4 2 0.5 : 0.8842145947698574, 0.9352657927058106
linear 4 2 0.7 : 0.8836339692546554, 0.934497457486268
linear 4 2 0.9 : 0.8825320443369236, 0.9335216305253994
linear 4 3 0.1 : 0.8847477454915926, 0.9347323513461686
linear 4 3 0.3 : 0.8847378515214659, 0.9355341761405427
linear 4 3 0.5 : 0.8842145947698574, 0.9352657927058106
linear 4 3 0.7 : 0.8836339692546554, 0.934497457486268
linear 4 3 0.9 : 0.8825320443369236, 0.9335216305253994
linear 4 4 0.1 : 0.8847477454915926, 0.9347323513461686
linear 4 4 0.3 : 0.8847378515214659, 0.9355341761405427
linear 4 4 0.5 : 0.8842145947698574, 0.9352657927058106
linear 4 4 0.7 : 0.8836339692546554, 0.934497457486268
linear 4 4 0.9 : 0.8825320443369236, 0.9335216305253994
linear 4 5 0.1 : 0.8847477454915926, 0.9347323513461686
linear 4 5 0.3 : 0.8847378515214659, 0.9355341761405427
linear 4 5 0.5 : 0.8842145947698574, 0.9352657927058106
linear 4 5 0.7 : 0.8836339692546554, 0.934497457486268
linear 4 5 0.9 : 0.8825320443369236, 0.9335216305253994
linear 4 6 0.1 : 0.8847477454915926, 0.9347323513461686
linear 4 6 0.3 : 0.8847378515214659, 0.9355341761405427
linear 4 6 0.5 : 0.8842145947698574, 0.9352657927058106
linear 4 6 0.7 : 0.8836339692546554, 0.934497457486268
linear 4 6 0.9 : 0.8825320443369236, 0.9335216305253994
poly 1 1 0.1 : 0.7546186460558326, 0.8146881464499465
poly 1 1 0.3 : 0.7513337706144352, 0.8108000413914541
poly 1 1 0.5 : 0.7478226849398923, 0.8069556042264957
poly 1 1 0.7 : 0.7440978573540027, 0.8029844585752399
poly 1 1 0.9 : 0.7401995522026612, 0.798905163042552
poly 1 2 0.1 : 0.7526680697129893, 0.812570003561801
poly 1 2 0.3 : 0.7493673616332482, 0.8086627471227259
poly 1 2 0.5 : 0.7458337219449255, 0.8048018847704134
poly 1 2 0.7 : 0.7421006626680577, 0.8008218487102582
poly 1 2 0.9 : 0.738181538136482, 0.7967172695573449
poly 1 3 0.1 : 0.7520051411402079, 0.8118491240683194
poly 1 3 0.3 : 0.7486997023356871, 0.8079364365920109
poly 1 3 0.5 : 0.745157613230399, 0.8040685191055102
poly 1 3 0.7 : 0.7414231151183308, 0.8000865168149749
poly 1 3 0.9 : 0.7374971660901949, 0.7959728847384169
poly 1 4 0.1 : 0.7516718243061024, 0.8114859797133469
poly 1 4 0.3 : 0.7483638836298592, 0.8075696445746787
poly 1 4 0.5 : 0.7448172589790113, 0.8036990361126694
poly 1 4 0.7 : 0.741081741653272, 0.7997158765345238
poly 1 4 0.9 : 0.7371522241442012, 0.7955986424608936
poly 1 5 0.1 : 0.7514708582488225, 0.8112675401808087
poly 1 5 0.3 : 0.7481611195411684, 0.8073484804597792
poly 1 5 0.5 : 0.7446127105789774, 0.8034768207445836
poly 1 5 0.7 : 0.7408760845326717, 0.7994935720407832
poly 1 5 0.9 : 0.7369444222698458, 0.7953721451422863
poly 1 6 0.1 : 0.7513366584033638, 0.8111218235853552
poly 1 6 0.3 : 0.7480256917407544, 0.8072013837896115
poly 1 6 0.5 : 0.7444752535950625, 0.8033269333269322
poly 1 6 0.7 : 0.7407388714556771, 0.7993430655064305
poly 1 6 0.9 : 0.7368059043106767, 0.7952218984251949
poly 2 1 0.1 : 0.7546186460557358, 0.8146881464498461
poly 2 1 0.3 : 0.7513337706145229, 0.8108000413917733
poly 2 1 0.5 : 0.7478226849397357, 0.8069556042266415

poly 2 1 0.7 : 0.7440978573540079, 0.802984458575027
poly 2 1 0.9 : 0.7401995522026612, 0.798905163042552
poly 2 2 0.1 : 0.7526680697131708, 0.8125700035608288
poly 2 2 0.3 : 0.7493673616335667, 0.8086627471234247
poly 2 2 0.5 : 0.7458337219441601, 0.8048018847702976
poly 2 2 0.7 : 0.7421006626678537, 0.8008218487095646
poly 2 2 0.9 : 0.738181538136482, 0.7967172695573449
poly 2 3 0.1 : 0.7520051411398091, 0.8118491240691421
poly 2 3 0.3 : 0.7486997023394897, 0.8079364365870727
poly 2 3 0.5 : 0.7451576132353243, 0.8040685191057596
poly 2 3 0.7 : 0.7414231151213319, 0.8000865168128104
poly 2 3 0.9 : 0.7374971660901949, 0.7959728847384169
poly 2 4 0.1 : 0.7516718243081755, 0.8114859797227332
poly 2 4 0.3 : 0.7483638836355919, 0.8075696445880444
poly 2 4 0.5 : 0.7448172589674391, 0.8036990361113796
poly 2 4 0.7 : 0.7410817416501205, 0.7997158765505756
poly 2 4 0.9 : 0.7371522241442012, 0.7955986424608936
poly 2 5 0.1 : 0.7514708582429308, 0.8112675401696683
poly 2 5 0.3 : 0.748161119555534, 0.8073484804823297
poly 2 5 0.5 : 0.744612710560313, 0.8034768207415427
poly 2 5 0.7 : 0.7408760845410878, 0.79949357203529
poly 2 5 0.9 : 0.7369444222698458, 0.7953721451422863
poly 2 6 0.1 : 0.7513366584030285, 0.8111218235879714
poly 2 6 0.3 : 0.7480256917549923, 0.8072013838011829
poly 2 6 0.5 : 0.7444752535825653, 0.8033269334191763
poly 2 6 0.7 : 0.7407388714601563, 0.7993430655062886
poly 2 6 0.9 : 0.7368059043106767, 0.7952218984251949
poly 3 1 0.1 : 0.754618646055569, 0.8146881464500455
poly 3 1 0.3 : 0.7513337706147012, 0.8108000413915976
poly 3 1 0.5 : 0.7478226849399388, 0.8069556042267915
poly 3 1 0.7 : 0.7440978573540448, 0.8029844585749649
poly 3 1 0.9 : 0.7401995522026612, 0.798905163042552
poly 3 2 0.1 : 0.7526680697116872, 0.8125700035604818
poly 3 2 0.3 : 0.7493673616337178, 0.8086627471239253
poly 3 2 0.5 : 0.7458337219442344, 0.80480188477153
poly 3 2 0.7 : 0.7421006626672652, 0.8008218487091434
poly 3 2 0.9 : 0.738181538136482, 0.7967172695573449
poly 3 3 0.1 : 0.7520051411387999, 0.8118491240603529
poly 3 3 0.3 : 0.7486997023384996, 0.807936436590473
poly 3 3 0.5 : 0.7451576132297941, 0.8040685191057385
poly 3 3 0.7 : 0.7414231151214501, 0.8000865168151368
poly 3 3 0.9 : 0.7374971660901949, 0.7959728847384169
poly 3 4 0.1 : 0.7516718243107984, 0.8114859797271622
poly 3 4 0.3 : 0.7483638836420455, 0.8075696445712264
poly 3 4 0.5 : 0.7448172589582142, 0.8036990361025625
poly 3 4 0.7 : 0.7410817416512754, 0.7997158765356179
poly 3 4 0.9 : 0.7371522241442012, 0.7955986424608936
poly 3 5 0.1 : 0.7514708582543184, 0.811267540184619
poly 3 5 0.3 : 0.7481611195348503, 0.8073484804393344
poly 3 5 0.5 : 0.7446127105489931, 0.8034768207337494
poly 3 5 0.7 : 0.7408760845460031, 0.7994935720509745
poly 3 5 0.9 : 0.7369444222698458, 0.7953721451422863
poly 3 6 0.1 : 0.7513366584356651, 0.8111218235883829
poly 3 6 0.3 : 0.7480256917797745, 0.8072013837715516
poly 3 6 0.5 : 0.7444752536223804, 0.8033269333537412
poly 3 6 0.7 : 0.7407388714635441, 0.79934306549555
poly 3 6 0.9 : 0.7368059043106767, 0.7952218984251949
poly 4 1 0.1 : 0.7546186460554585, 0.814688146450308
poly 4 1 0.3 : 0.7513337706146256, 0.8108000413912884
poly 4 1 0.5 : 0.747822684940127, 0.8069556042266876
poly 4 1 0.7 : 0.7440978573540689, 0.8029844585752985
poly 4 1 0.9 : 0.7401995522026612, 0.798905163042552
poly 4 2 0.1 : 0.7526680697121607, 0.812570003557661
poly 4 2 0.3 : 0.7493673616335592, 0.8086627471245827
poly 4 2 0.5 : 0.745833721946149, 0.8048018847719046
poly 4 2 0.7 : 0.7421006626669497, 0.8008218487087085
poly 4 2 0.9 : 0.738181538136482, 0.7967172695573449
poly 4 3 0.1 : 0.7520051411416293, 0.81184912406941
poly 4 3 0.3 : 0.7486997023370314, 0.8079364365957642
poly 4 3 0.5 : 0.745157613230764, 0.8040685191024627
poly 4 3 0.7 : 0.7414231151167087, 0.800086516826599
poly 4 3 0.9 : 0.7374971660901949, 0.7959728847384169
poly 4 4 0.1 : 0.7516718243123929, 0.8114859797171765
poly 4 4 0.3 : 0.7483638836292211, 0.807569644551838
poly 4 4 0.5 : 0.7448172589668156, 0.8036990360978503
poly 4 4 0.7 : 0.7410817416508342, 0.7997158765237671
poly 4 4 0.9 : 0.7371522241442012, 0.7955986424608936
poly 4 5 0.1 : 0.751470858251607, 0.8112675401876595
poly 4 5 0.3 : 0.7481611195263176, 0.8073484804299358
poly 4 5 0.5 : 0.7446127105814838, 0.8034768207500352
poly 4 5 0.7 : 0.7408760845445685, 0.7994935720494231
poly 4 5 0.9 : 0.7369444222698458, 0.7953721451422863
poly 4 6 0.1 : 0.7513366584354373, 0.8111218235497063
poly 4 6 0.3 : 0.7480256917963543, 0.8072013836919746
poly 4 6 0.5 : 0.7444752535909702, 0.8033269332104973
poly 4 6 0.7 : 0.7407388715058758, 0.7993430655107967
poly 4 6 0.9 : 0.7368059043106767, 0.7952218984251949
rbf 1 1 0.1 : -0.027373660206448002, 0.001565258005155834
rbf 1 1 0.3 : -0.027119554179198736, 0.0015338875954057363
rbf 1 1 0.5 : -0.026514797908722, 0.001463752487367853
rbf 1 1 0.7 : -0.02572046266453354, 0.0014166939394202327
rbf 1 1 0.9 : -0.024462500471238702, 0.001385774933252426
rbf 1 2 0.1 : -0.027373674033629936, 0.001565259389008422
rbf 1 2 0.3 : -0.027119568008564875, 0.0015338889678809808
rbf 1 2 0.5 : -0.026514811748516866, 0.0014637538367194836
rbf 1 2 0.7 : -0.025720476515565992, 0.001416695274653046
rbf 1 2 0.9 : -0.024462553208706782, 0.001385776265193428
rbf 1 3 0.1 : -0.027373674033707475, 0.0015652593890191913
rbf 1 3 0.3 : -0.027119568008642327, 0.00153388896789175
rbf 1 3 0.5 : -0.0265148117485944, 0.0014637538367301417
rbf 1 3 0.7 : -0.025720476515643576, 0.001416695274663704
rbf 1 3 0.9 : -0.024462553208784633, 0.0013857762652040861
rbf 1 4 0.1 : -0.027373674033707475, 0.0015652593890191913
rbf 1 4 0.3 : -0.027119568008642327, 0.00153388896789175
rbf 1 4 0.5 : -0.0265148117485944, 0.0014637538367301417
rbf 1 4 0.7 : -0.025720476515643576, 0.001416695274663704
rbf 1 4 0.9 : -0.024462553208784633, 0.0013857762652040861
rbf 1 5 0.1 : -0.027373674033707475, 0.0015652593890191913
rbf 1 5 0.3 : -0.027119568008642327, 0.00153388896789175
rbf 1 5 0.5 : -0.0265148117485944, 0.0014637538367301417
rbf 1 5 0.7 : -0.025720476515643576, 0.001416695274663704
rbf 1 5 0.9 : -0.024462553208784633, 0.0013857762652040861
rbf 1 6 0.1 : -0.027373674033707475, 0.0015652593890191913
rbf 1 6 0.3 : -0.027119568008642327, 0.00153388896789175
rbf 1 6 0.5 : -0.0265148117485944, 0.0014637538367301417
rbf 1 6 0.7 : -0.025720476515643576, 0.001416695274663704
rbf 1 6 0.9 : -0.024462553208784633, 0.0013857762652040861
rbf 2 1 0.1 : -0.024727663251135068, 0.003056898092060223
rbf 2 1 0.3 : -0.02404009592956342, 0.002981544322684493
rbf 2 1 0.5 : -0.02336034369222064, 0.0028817989942167
rbf 2 1 0.7 : -0.02255315439074357, 0.002762991596187092
rbf 2 1 0.9 : -0.021807445798656833, 0.0026372986938206777
rbf 2 2 0.1 : -0.02472769099822294, 0.003056900881847291
rbf 2 2 0.3 : -0.02404012371672861, 0.0029815472179688385
rbf 2 2 0.5 : -0.023360371723766392, 0.0028818019840016552
rbf 2 2 0.7 : -0.022553182438433562, 0.0027629946724857524
rbf 2 2 0.9 : -0.02180747383876742, 0.0026373018529726178
rbf 2 3 0.1 : -0.02472769099837846, 0.0030569008818687182
rbf 2 3 0.3 : -0.02404012371688431, 0.002981547217990266
rbf 2 3 0.5 : -0.023360371723922534, 0.0028818019840379794
rbf 2 3 0.7 : -0.022553182438589968, 0.0027629946725071797
rbf 2 3 0.9 : -0.021807473838924007, 0.002637301852993823
rbf 2 4 0.1 : -0.02472769099837846, 0.0030569008818687182
rbf 2 4 0.3 : -0.02404012371688431, 0.002981547217990266
rbf 2 4 0.5 : -0.023360371723922534, 0.0028818019840379794
rbf 2 4 0.7 : -0.022553182438589968, 0.0027629946725071797
rbf 2 4 0.9 : -0.021807473838924007, 0.002637301852993823
rbf 2 5 0.1 : -0.02472769099837846, 0.0030569008818687182
rbf 2 5 0.3 : -0.02404012371688431, 0.002981547217990266
rbf 2 5 0.5 : -0.023360371723922534, 0.0028818019840379794
rbf 2 5 0.7 : -0.022553182438589968, 0.0027629946725071797
rbf 2 5 0.9 : -0.021807473838924007, 0.002637301852993823
rbf 2 6 0.1 : -0.02472769099837846, 0.0030569008818687182
rbf 2 6 0.3 : -0.02404012371688431, 0.002981547217990266
rbf 2 6 0.5 : -0.023360371723922534, 0.0028818019840379794
rbf 2 6 0.7 : -0.022553182438589968, 0.0027629946725071797
rbf 2 6 0.9 : -0.021807473838924007, 0.002637301852993823
rbf 3 1 0.1 : -0.0219000605178278, 0.004171448725588944

rbf 3 1 0.3 : -0.021144357417401994, 0.0040643670284410405
rbf 3 1 0.5 : -0.02024590028931601, 0.003916007101118613
rbf 3 1 0.7 : -0.019195636366006096, 0.003664731801778287
rbf 3 1 0.9 : -0.01810950894559187, 0.0033469852021307256
rbf 3 2 0.1 : -0.021900102501394337, 0.004171453224730337
rbf 3 2 0.3 : -0.02114437026313376, 0.004064371596202099
rbf 3 2 0.5 : -0.02024590993347517, 0.003916011714145462
rbf 3 2 0.7 : -0.019195699579830937, 0.0036647364104955837
rbf 3 2 0.9 : -0.018109566659078258, 0.0033469897792893777
rbf 3 3 0.1 : -0.021900102501629215, 0.004171453224761756
rbf 3 3 0.3 : -0.021144370263368906, 0.00406437159623374
rbf 3 3 0.5 : -0.020245909933710938, 0.003916011714176548
rbf 3 3 0.7 : -0.019195699580067015, 0.003664736410526115
rbf 3 3 0.9 : -0.018109566659314826, 0.0033469897793192427
rbf 3 4 0.1 : -0.021900102501629215, 0.004171453224761756
rbf 3 4 0.3 : -0.021144370263368906, 0.00406437159623374
rbf 3 4 0.5 : -0.020245909933710938, 0.003916011714176548
rbf 3 4 0.7 : -0.019195699580067015, 0.003664736410526115
rbf 3 4 0.9 : -0.018109566659314826, 0.0033469897793192427
rbf 3 5 0.1 : -0.021900102501629215, 0.004171453224761756
rbf 3 5 0.3 : -0.021144370263368906, 0.00406437159623374
rbf 3 5 0.5 : -0.020245909933710938, 0.003916011714176548
rbf 3 5 0.7 : -0.019195699580067015, 0.003664736410526115
rbf 3 5 0.9 : -0.018109566659314826, 0.0033469897793192427
rbf 3 6 0.1 : -0.021900102501629215, 0.004171453224761756
rbf 3 6 0.3 : -0.021144370263368906, 0.00406437159623374
rbf 3 6 0.5 : -0.020245909933710938, 0.003916011714176548
rbf 3 6 0.7 : -0.019195699580067015, 0.003664736410526115
rbf 3 6 0.9 : -0.018109566659314826, 0.0033469897793192427
rbf 4 1 0.1 : -0.01801479655058049, 0.004589094489156453
rbf 4 1 0.3 : -0.016968722911716316, 0.004157525645991922
rbf 4 1 0.5 : -0.015935840727171822, 0.00369899807358276
rbf 4 1 0.7 : -0.014987167110211086, 0.0032186570643942813
rbf 4 1 0.9 : -0.014053941582535501, 0.002613952468489278
rbf 4 2 0.1 : -0.01801485284874569, 0.004589100157873838
rbf 4 2 0.3 : -0.016968779358160813, 0.004157531191201436
rbf 4 2 0.5 : -0.015935926904985243, 0.0036990034903048308
rbf 4 2 0.7 : -0.014987223851878095, 0.0032186623484490307
rbf 4 2 0.9 : -0.014054037342099979, 0.0026139575703744145
rbf 4 3 0.1 : -0.018014852849061257, 0.0045891001579123625
rbf 4 3 0.3 : -0.016968779358477758, 0.00415753119123885
rbf 4 3 0.5 : -0.015935926905303033, 0.003699003490341024
rbf 4 3 0.7 : -0.014987202741356542, 0.0032186623484837806
rbf 4 3 0.9 : -0.014054037342419634, 0.00261395757040761
rbf 4 4 0.1 : -0.018014852849061257, 0.0045891001579123625
rbf 4 4 0.3 : -0.016968779358477758, 0.00415753119123885
rbf 4 4 0.5 : -0.015935926905303033, 0.003699003490341024
rbf 4 4 0.7 : -0.014987202741356542, 0.0032186623484837806
rbf 4 4 0.9 : -0.014054037342419634, 0.00261395757040761
rbf 4 5 0.1 : -0.018014852849061257, 0.0045891001579123625
rbf 4 5 0.3 : -0.016968779358477758, 0.00415753119123885
rbf 4 5 0.5 : -0.015935926905303033, 0.003699003490341024
rbf 4 5 0.7 : -0.014987202741356542, 0.0032186623484837806
rbf 4 5 0.9 : -0.014054037342419634, 0.00261395757040761
rbf 4 6 0.1 : -0.018014852849061257, 0.0045891001579123625
rbf 4 6 0.3 : -0.016968779358477758, 0.00415753119123885
rbf 4 6 0.5 : -0.015935926905303033, 0.003699003490341024
rbf 4 6 0.7 : -0.014987202741356542, 0.0032186623484837806
rbf 4 6 0.9 : -0.014054037342419634, 0.00261395757040761
sigmoid 1 1 0.1 : -0.02785166028996544, -0.00048177410340799476
sigmoid 1 1 0.3 : -0.02803864106557148, -0.00015275043162876756
sigmoid 1 1 0.5 : -0.029327686018558598, -8.395440265673848e-05
sigmoid 1 1 0.7 : -0.02737081310365812, -0.00015275063107411668
sigmoid 1 1 0.9 : -0.027425941148526433, -0.00035164248973940104
sigmoid 1 2 0.1 : -0.027851574944839273, -0.00048175891836943485
sigmoid 1 2 0.3 : -0.028038637425174963, -0.0001527348653764804
sigmoid 1 2 0.5 : -0.02932768292993613, -8.393840800091645e-05
sigmoid 1 2 0.7 : -0.02737078105587769, -0.0001527348653764804
sigmoid 1 2 0.9 : -0.027425939282243786, -0.00035164512056096164
sigmoid 1 3 0.1 : -0.02785157494475108, -0.0004817589182650739
sigmoid 1 3 0.3 : -0.028038637425086766, -0.0001527348652718974
sigmoid 1 3 0.5 : -0.029327682929847977, -8.39384078961114e-05
sigmoid 1 3 0.7 : -0.027370781055785543, -0.0001527348652718974
sigmoid 1 3 0.9 : -0.02742593928234638, -0.0003516451204561566
sigmoid 1 4 0.1 : -0.02785157494475108, -0.0004817589182650739
sigmoid 1 4 0.3 : -0.028038637425086766, -0.0001527348652718974
sigmoid 1 4 0.5 : -0.029327682929847977, -8.39384078961114e-05
sigmoid 1 4 0.7 : -0.027370781055785543, -0.0001527348652718974
sigmoid 1 4 0.9 : -0.02742593928234638, -0.0003516451204561566
sigmoid 1 5 0.1 : -0.02785157494475108, -0.0004817589182650739
sigmoid 1 5 0.3 : -0.028038637425086766, -0.0001527348652718974
sigmoid 1 5 0.5 : -0.029327682929847977, -8.39384078961114e-05
sigmoid 1 5 0.7 : -0.027370781055785543, -0.0001527348652718974
sigmoid 1 5 0.9 : -0.02742593928234638, -0.0003516451204561566
sigmoid 1 6 0.1 : -0.02785157494475108, -0.0004817589182650739
sigmoid 1 6 0.3 : -0.028038637425086766, -0.0001527348652718974
sigmoid 1 6 0.5 : -0.029327682929847977, -8.39384078961114e-05
sigmoid 1 6 0.7 : -0.027370781055785543, -0.0001527348652718974
sigmoid 1 6 0.9 : -0.02742593928234638, -0.0003516451204561566
sigmoid 2 1 0.1 : -0.02785174563541415, -0.0004817892886335162
sigmoid 2 1 0.3 : -0.028038644706131332, -0.00015276599806823832
sigmoid 2 1 0.5 : -0.029327689107343957, -8.397039749885593e-05
sigmoid 2 1 0.7 : -0.027370845151641187, -0.00015276639695827043
sigmoid 2 1 0.9 : -0.02742594301478145, -0.00035163985958797106
sigmoid 2 2 0.1 : -0.027851574944927203, -0.00048175891847401786
sigmoid 2 2 0.3 : -0.028038637425263156, -0.0001527348654815075
sigmoid 2 2 0.5 : -0.0293276829300241, -8.393840810572151e-05
sigmoid 2 2 0.7 : -0.02737078105596975, -0.00015273486548084136
sigmoid 2 2 0.9 : -0.0274259392825293, -0.0003516451206653226
sigmoid 2 3 0.1 : -0.02785157494475108, -0.0004817589182650739
sigmoid 2 3 0.3 : -0.028038637425086766, -0.0001527348652718974
sigmoid 2 3 0.5 : -0.029327682929847977, -8.39384078961114e-05
sigmoid 2 3 0.7 : -0.027370781055785543, -0.0001527348652718974
sigmoid 2 3 0.9 : -0.02742593928234638, -0.0003516451204561566
sigmoid 2 4 0.1 : -0.02785157494475108, -0.0004817589182650739
sigmoid 2 4 0.3 : -0.028038637425086766, -0.0001527348652718974
sigmoid 2 4 0.5 : -0.029327682929847977, -8.39384078961114e-05
sigmoid 2 4 0.7 : -0.027370781055785543, -0.0001527348652718974
sigmoid 2 4 0.9 : -0.02742593928234638, -0.0003516451204561566
sigmoid 2 5 0.1 : -0.02785157494475108, -0.0004817589182650739
sigmoid 2 5 0.3 : -0.028038637425086766, -0.0001527348652718974
sigmoid 2 5 0.5 : -0.029327682929847977, -8.39384078961114e-05
sigmoid 2 5 0.7 : -0.027370781055785543, -0.0001527348652718974
sigmoid 2 5 0.9 : -0.02742593928234638, -0.0003516451204561566
sigmoid 2 6 0.1 : -0.02785157494475108, -0.0004817589182650739
sigmoid 2 6 0.3 : -0.028038637425086766, -0.0001527348652718974
sigmoid 2 6 0.5 : -0.029327682929847977, -8.39384078961114e-05
sigmoid 2 6 0.7 : -0.027370781055785543, -0.0001527348652718974
sigmoid 2 6 0.9 : -0.02742593928234638, -0.0003516451204561566
sigmoid 3 1 0.1 : -0.027851830981096892, -0.0004818044739398619
sigmoid 3 1 0.3 : -0.028038648346765838, -0.0001527815645889774
sigmoid 3 1 0.5 : -0.029327692196203438, -8.398639242224171e-05
sigmoid 3 1 0.7 : -0.027370877199734876, -0.00015278216292347047
sigmoid 3 1 0.9 : -0.027425944881110987, -0.0003516372300007564
sigmoid 3 2 0.1 : -0.027851574945015445, -0.0004817589185783788
sigmoid 3 2 0.3 : -0.028038637425351443, -0.00015273486558631255
sigmoid 3 2 0.5 : -0.029327682930112342, -8.39384082107486e-05
sigmoid 3 2 0.7 : -0.027370781056062297, -0.00015273486558520233
sigmoid 3 2 0.9 : -0.027425939282621003, -0.0003516451207699056
sigmoid 3 3 0.1 : -0.02785157494475108, -0.0004817589182650739
sigmoid 3 3 0.3 : -0.028038637425086766, -0.0001527348652718974
sigmoid 3 3 0.5 : -0.029327682929847977, -8.39384078961114e-05
sigmoid 3 3 0.7 : -0.027370781055785543, -0.0001527348652718974
sigmoid 3 3 0.9 : -0.02742593928234638, -0.0003516451204561566
sigmoid 3 4 0.1 : -0.02785157494475108, -0.0004817589182650739
sigmoid 3 4 0.3 : -0.028038637425086766, -0.0001527348652718974
sigmoid 3 4 0.5 : -0.029327682929847977, -8.39384078961114e-05
sigmoid 3 4 0.7 : -0.027370781055785543, -0.0001527348652718974
sigmoid 3 4 0.9 : -0.02742593928234638, -0.0003516451204561566
sigmoid 3 5 0.1 : -0.02785157494475108, -0.0004817589182650739

sigmoid 3 5 0.3 : -0.028038637425086766, -0.0001527348652718974
sigmoid 3 5 0.5 : -0.029327682929847977, -8.39384078961114e-05
sigmoid 3 5 0.7 : -0.027370781055785543, -0.0001527348652718974
sigmoid 3 5 0.9 : -0.02742593928234638, -0.0003516451204561566
sigmoid 3 6 0.1 : -0.02785157494475108, -0.0004817589182650739
sigmoid 3 6 0.3 : -0.028038637425086766, -0.0001527348652718974
sigmoid 3 6 0.5 : -0.029327682929847977, -8.39384078961114e-05
sigmoid 3 6 0.7 : -0.027370781055785543, -0.0001527348652718974
sigmoid 3 6 0.9 : -0.02742593928234638, -0.0003516451204561566
sigmoid 4 1 0.1 : -0.02785191632701465, -0.00048181965932947435
sigmoid 4 1 0.3 : -0.028038651987475925, -0.00015279713119231708
sigmoid 4 1 0.5 : -0.02932769528513841, -8.40023874273399e-05
sigmoid 4 1 0.7 : -0.027370909247939102, -0.00015279792897149314
sigmoid 4 1 0.9 : -0.027425946747515396, -0.00035163460097931143
sigmoid 4 2 0.1 : -0.027851574945103465, -0.0004817589186827398
sigmoid 4 2 0.3 : -0.028038637425439772, -0.00015273486569089556
sigmoid 4 2 0.5 : -0.02932768293020027, -8.393840831555366e-05
sigmoid 4 2 0.7 : -0.027370781056154404, -0.00015273486569000738
sigmoid 4 2 0.9 : -0.0274259392827124, -0.00035164512087404454
sigmoid 4 3 0.1 : -0.02785157494475108, -0.0004817589182650739
sigmoid 4 3 0.3 : -0.028038637425086766, -0.0001527348652718974
sigmoid 4 3 0.5 : -0.029327682929847977, -8.39384078961114e-05
sigmoid 4 3 0.7 : -0.027370781055785543, -0.0001527348652718974
sigmoid 4 3 0.9 : -0.02742593928234638, -0.0003516451204561566
sigmoid 4 4 0.1 : -0.02785157494475108, -0.0004817589182650739
sigmoid 4 4 0.3 : -0.028038637425086766, -0.0001527348652718974
sigmoid 4 4 0.5 : -0.029327682929847977, -8.39384078961114e-05
sigmoid 4 4 0.7 : -0.027370781055785543, -0.0001527348652718974
sigmoid 4 4 0.9 : -0.02742593928234638, -0.0003516451204561566
sigmoid 4 5 0.1 : -0.02785157494475108, -0.0004817589182650739
sigmoid 4 5 0.3 : -0.028038637425086766, -0.0001527348652718974
sigmoid 4 5 0.5 : -0.029327682929847977, -8.39384078961114e-05
sigmoid 4 5 0.7 : -0.027370781055785543, -0.0001527348652718974
sigmoid 4 5 0.9 : -0.02742593928234638, -0.0003516451204561566
sigmoid 4 6 0.1 : -0.02785157494475108, -0.0004817589182650739
sigmoid 4 6 0.3 : -0.028038637425086766, -0.0001527348652718974
sigmoid 4 6 0.5 : -0.029327682929847977, -8.39384078961114e-05
sigmoid 4 6 0.7 : -0.027370781055785543, -0.0001527348652718974
sigmoid 4 6 0.9 : -0.02742593928234638, -0.0003516451204561566

Grid search of ElasticNet, Morgan(r=4,2048)
ElasticNet(alpha= i, l1_ratio= j, max_iter=100000)
i j : accuracy of prediction using cross validation, accuracy of prediction using test data
0.001 0.0 : 0.8961389510009272, 0.9396052004331126
0.001 0.2 : 0.898974108745213, 0.947280686027679
0.001 0.4 : 0.9017108468957125, 0.9513628130667905
0.001 0.6 : 0.9040740478688395, 0.9532123868080933
0.001 0.8 : 0.9040677139973378, 0.9521172695476053
0.001 1.0 : 0.8915027355630499, 0.9385802805134725
0.01 0.0 : 0.881590823162304, 0.926739991154929
0.01 0.2 : 0.8850478692282941, 0.9328316581534756
0.01 0.4 : 0.8875915193912087, 0.9364960184391592
0.01 0.6 : 0.8908550371160834, 0.9391884754565657
0.01 0.8 : 0.8938652039476545, 0.9396902006388186
0.01 1.0 : 0.8920065789898152, 0.9356220123438451
0.1 0.0 : 0.7976735640686483, 0.8324847329372873
0.1 0.2 : 0.7878873563592208, 0.8199456173744302
0.1 0.4 : 0.7819389755571456, 0.8110376250412029
0.1 0.6 : 0.7806391111525668, 0.8090582294882422
0.1 0.8 : 0.789208262748814, 0.8153324719076087
0.1 1.0 : 0.8166675977011224, 0.8415066383677469

Grid search of RandomForest, Morgan(r=4,2048)
RandomForestRegressor(n_estimators= i)
i: accuracy of prediction using cross validation, accuracy of prediction using test data
100 1.0 : 0.7761956953283156, 0.8380862795380379
500 1.0 : 0.7758484033772934, 0.8507622280426231
1000 1.0 : 0.7794382978679872, 0.8467736236143539
2000 1.0 : 0.7773959838363362, 0.8480944148259353

Grid search of NeuralNetwork, Morgan(r=4,2048)
MLPRegressor(activation=act, alpha=a, batch_size=batch, beta_1=0.9,
beta_2=0.999, early_stopping=False, epsilon=1e-08,
hidden_layer_sizes=hid, learning_rate='constant',
learning_rate_init=0.001, max_iter=100000, momentum=0.9,
n_iter_no_change=10, nesterovs_momentum=True, power_t=0.5,
random_state=1, shuffle=True, solver='adam', tol=0.0001,
validation_fraction=0.1, verbose=False, warm_start=False)
act (hid) a batch: accuracy of prediction using cross validation, accuracy of prediction using test data
relu (200,) 0.0001 500 : 0.7695378740416403, 0.891111704612585
relu (200,) 0.0001 200 : 0.8120078096629904, 0.8800980261242278
relu (200,) 0.0001 100 : 0.8147138879273614, 0.8875712930215505
relu (200,) 0.0001 50 : 0.8248358293261709, 0.8945718957199839
relu (200,) 0.001 500 : 0.7695988263850418, 0.8912984599040702
relu (200,) 0.001 200 : 0.8123654436048493, 0.8802472360497237
relu (200,) 0.001 100 : 0.8151990529334208, 0.8879621478312729
relu (200,) 0.001 50 : 0.8254946586512005, 0.8949958387527649
relu (200,) 0.01 500 : 0.7702796595896182, 0.8931544035489761
relu (200,) 0.01 200 : 0.8161969607589885, 0.8815455061821503
relu (200,) 0.01 100 : 0.8201850295702293, 0.8915394292150505
relu (200,) 0.01 50 : 0.8324216693922205, 0.898351341981521
relu (200, 200) 0.0001 500 : 0.764110839469458, 0.8867201567982532
relu (200, 200) 0.0001 200 : 0.8055608597429016, 0.8796757710650115
relu (200, 200) 0.0001 100 : 0.8136224191625228, 0.8905512357518995
relu (200, 200) 0.0001 50 : 0.8221555969436206, 0.8970382897902072
relu (200, 200) 0.001 500 : 0.7640716278214577, 0.8865029018663041
relu (200, 200) 0.001 200 : 0.8043406079417428, 0.8791371635950734
relu (200, 200) 0.001 100 : 0.8149916016097013, 0.8918786958160524
relu (200, 200) 0.001 50 : 0.8224160240465528, 0.8986111951549238
relu (200, 200) 0.01 500 : 0.7652172966216148, 0.8848642672701861
relu (200, 200) 0.01 200 : 0.805444905125781, 0.881146914547812
relu (200, 200) 0.01 100 : 0.8140290678347526, 0.8903193729619114
relu (200, 200) 0.01 50 : 0.825001442210511, 0.8976679070160413
relu (100,) 0.0001 500 : 0.769565335572173, 0.8909962793787567
relu (100,) 0.0001 200 : 0.8136202742352088, 0.8790610916973122
relu (100,) 0.0001 100 : 0.8164300574100659, 0.8868196894223388
relu (100,) 0.0001 50 : 0.8264683156246979, 0.8948653184476
relu (100,) 0.001 500 : 0.7696136389525277, 0.8911479812870564
relu (100,) 0.001 200 : 0.8139051320341715, 0.8791890826689001
relu (100,) 0.001 100 : 0.8169755639226282, 0.8871417089544269
relu (100,) 0.001 50 : 0.8267109819971694, 0.8949702290620395
relu (100,) 0.01 500 : 0.7701099408232279, 0.8936566895239176
relu (100,) 0.01 200 : 0.8172722839408488, 0.880280440120844
relu (100,) 0.01 100 : 0.8208850482330309, 0.8907945816176159
relu (100,) 0.01 50 : 0.8311547938104097, 0.8985218075510634
relu (100, 100) 0.0001 500 : 0.7639580058344704, 0.8860919010618515
relu (100, 100) 0.0001 200 : 0.8109260932250535, 0.8813735920011481
relu (100, 100) 0.0001 100 : 0.8159304655830919, 0.8893125390215838
relu (100, 100) 0.0001 50 : 0.8231808637551069, 0.9016202034021553
relu (100, 100) 0.001 500 : 0.7646666301105931, 0.886125932851026
relu (100, 100) 0.001 200 : 0.8127268740626216, 0.8797862325320011
relu (100, 100) 0.001 100 : 0.8150915873767133, 0.8894372270446804

relu (100, 100) 0.001 50 : 0.8227591448621363, 0.9032584688202324
relu (100, 100) 0.01 500 : 0.7633493389083641, 0.8865568708519196
relu (100, 100) 0.01 200 : 0.8125454161253256, 0.8815029018079829
relu (100, 100) 0.01 100 : 0.8164208643191808, 0.8915416879322079
relu (100, 100) 0.01 50 : 0.8253706678311626, 0.9022861060386262
relu (100, 200) 0.0001 500 : 0.7703592221286516, 0.8934136948203406
relu (100, 200) 0.0001 200 : 0.8093023542733346, 0.8792088653348858
relu (100, 200) 0.0001 100 : 0.8141611431687498, 0.8864371381597982
relu (100, 200) 0.0001 50 : 0.8251465913006975, 0.8956660442436635
relu (100, 200) 0.001 500 : 0.7704987444615496, 0.8933139969763444
relu (100, 200) 0.001 200 : 0.8088129482196651, 0.87742081650067
relu (100, 200) 0.001 100 : 0.8154292475700702, 0.8892345525718628
relu (100, 200) 0.001 50 : 0.8265942287953486, 0.896361457757078
relu (100, 200) 0.01 500 : 0.7724540614706967, 0.8942625769771229
relu (100, 200) 0.01 200 : 0.8093103075628798, 0.8775318152572881
relu (100, 200) 0.01 100 : 0.8164080950604177, 0.8892073667496385
relu (100, 200) 0.01 50 : 0.8260041568143025, 0.8975591395639803
relu (50,) 0.0001 500 : 0.7720408130203624, 0.8933615082262325
relu (50,) 0.0001 200 : 0.8202822969879507, 0.879865423941355
relu (50,) 0.0001 100 : 0.8201394913377305, 0.8877425916909424
relu (50,) 0.0001 50 : 0.8293284805532828, 0.8941815112598599
relu (50,) 0.001 500 : 0.7720976978099386, 0.8935113636366393
relu (50,) 0.001 200 : 0.8205732265736184, 0.8799939918272062
relu (50,) 0.001 100 : 0.820485198076765, 0.8880624460600405
relu (50,) 0.001 50 : 0.8300388409166018, 0.8946914430842422
relu (50,) 0.01 500 : 0.772654778222452, 0.8949432253328412
relu (50,) 0.01 200 : 0.8232011066436294, 0.8812029416850639
relu (50,) 0.01 100 : 0.8242665518617276, 0.8908027875837672
relu (50,) 0.01 50 : 0.837480429115336, 0.8995725484788099
relu (50, 50) 0.0001 500 : 0.7725758385359918, 0.8880283930705557
relu (50, 50) 0.0001 200 : 0.8169051652163567, 0.8784868673215349
relu (50, 50) 0.0001 100 : 0.822469759334829, 0.8875028888501136
relu (50, 50) 0.0001 50 : 0.8278750007553295, 0.8948762767538998
relu (50, 50) 0.001 500 : 0.772839544727794, 0.8885759099579768
relu (50, 50) 0.001 200 : 0.8169380681607283, 0.878001922477983
relu (50, 50) 0.001 100 : 0.8193496462672739, 0.8866789510928421
relu (50, 50) 0.001 50 : 0.8284407207008622, 0.8960262554888537
relu (50, 50) 0.01 500 : 0.7710720523783462, 0.8902303510950792
relu (50, 50) 0.01 200 : 0.8182717428540783, 0.8790904078602427
relu (50, 50) 0.01 100 : 0.8204645962711481, 0.8900044330700225
relu (50, 50) 0.01 50 : 0.8301715912201848, 0.8977954307410108
relu (50, 100) 0.0001 500 : 0.7702636624199084, 0.8921481942300022
relu (50, 100) 0.0001 200 : 0.8136356916192826, 0.8797107561790174
relu (50, 100) 0.0001 100 : 0.8190888897431782, 0.8881586961084657
relu (50, 100) 0.0001 50 : 0.8285376360332475, 0.8950912652775389
relu (50, 100) 0.001 500 : 0.7706104072780577, 0.8913110115065136
relu (50, 100) 0.001 200 : 0.8144736186302426, 0.8790575054976955
relu (50, 100) 0.001 100 : 0.8198469393056161, 0.8878338022222337
relu (50, 100) 0.001 50 : 0.8282125473860227, 0.8978923737566467
relu (50, 100) 0.01 500 : 0.7707160393478099, 0.89262699938396
relu (50, 100) 0.01 200 : 0.8145857982354725, 0.8791425587693893
relu (50, 100) 0.01 100 : 0.8213639879538125, 0.8903167272203979
relu (50, 100) 0.01 50 : 0.829591346815031, 0.8977274943085917
relu (50, 200) 0.0001 500 : 0.765827310507157, 0.8866565478150187
relu (50, 200) 0.0001 200 : 0.811701817849606, 0.8783325760595421
relu (50, 200) 0.0001 100 : 0.8163762803112604, 0.8926622429548761
relu (50, 200) 0.0001 50 : 0.8242649376136904, 0.8964485063784728
relu (50, 200) 0.001 500 : 0.7654930307855884, 0.890341180502923
relu (50, 200) 0.001 200 : 0.8123027296614932, 0.8772848282226737
relu (50, 200) 0.001 100 : 0.8180123313639868, 0.8930227374933287
relu (50, 200) 0.001 50 : 0.8247246899427042, 0.8973923788379325
relu (50, 200) 0.01 500 : 0.7651593633103824, 0.8878435155715393
relu (50, 200) 0.01 200 : 0.8125817545395699, 0.8794387503051821
relu (50, 200) 0.01 100 : 0.8168657556867777, 0.8940037752325127
relu (50, 200) 0.01 50 : 0.8273940392569656, 0.8989317873590159
tanh (200,) 0.0001 500 : 0.776182930710174, 0.87370762497005
tanh (200,) 0.0001 200 : 0.8175353460308298, 0.867033540587635
tanh (200,) 0.0001 100 : 0.8239235386851357, 0.8763702916791591
tanh (200,) 0.0001 50 : 0.8360209476543847, 0.8837043987054651
tanh (200,) 0.001 500 : 0.7774020950856754, 0.8755752416091259
tanh (200,) 0.001 200 : 0.8198243546843622, 0.868837916457206
tanh (200,) 0.001 100 : 0.826151652582338, 0.8779128922297081
tanh (200,) 0.001 50 : 0.8363851165180657, 0.8847078426346531
tanh (200,) 0.01 500 : 0.788611566761092, 0.8869196593014891
tanh (200,) 0.01 200 : 0.8356781170090581, 0.8845142177609318
tanh (200,) 0.01 100 : 0.8389632917558174, 0.8914798481327013
tanh (200,) 0.01 50 : 0.8478183431757828, 0.8939665904205095
tanh (200, 200) 0.0001 500 : 0.4521959253905671, -0.02137609620282155
tanh (200, 200) 0.0001 200 : 0.4931302540504087, 0.879427266122853
tanh (200, 200) 0.0001 100 : 0.8214891950268859, 0.8850236267580044
tanh (200, 200) 0.0001 50 : 0.8309888150869135, 0.8784094230014612
tanh (200, 200) 0.001 500 : 0.4538983660886299, -0.02137602796844229
tanh (200, 200) 0.001 200 : 0.49508944443216707, 0.8780399112593305
tanh (200, 200) 0.001 100 : 0.8231491626605363, 0.8900409485952292
tanh (200, 200) 0.001 50 : 0.834702922251279, 0.8797052619562926
tanh (200, 200) 0.01 500 : 0.4686453496895556, -0.02137510669313758
tanh (200, 200) 0.01 200 : 0.5040501358085532, 0.8916395935126921
tanh (200, 200) 0.01 100 : 0.8382841125480727, 0.8974972955452171
tanh (200, 200) 0.01 50 : 0.8459487942598767, 0.8989212083466253
tanh (100,) 0.0001 500 : 0.7589023822601654, 0.8838313404316115
tanh (100,) 0.0001 200 : 0.8124890024153109, 0.8769522652484711
tanh (100,) 0.0001 100 : 0.8160805716658619, 0.8876445645776273
tanh (100,) 0.0001 50 : 0.828812729615134, 0.8948262841679139
tanh (100,) 0.001 500 : 0.7624807905740236, 0.8854361040510469
tanh (100,) 0.001 200 : 0.815692495539073, 0.8823671498981367
tanh (100,) 0.001 100 : 0.8206569366128804, 0.8895952052361332
tanh (100,) 0.001 50 : 0.832743969699826, 0.8975603071048175
tanh (100,) 0.01 500 : 0.7904452299455097, 0.8944896559815487
tanh (100,) 0.01 200 : 0.8364495500513222, 0.8994972818663041
tanh (100,) 0.01 100 : 0.8379461326765221, 0.8994946999899591
tanh (100,) 0.01 50 : 0.8459245963703168, 0.9112015956400775
tanh (100, 100) 0.0001 500 : 0.7407646116825249, 0.8796941333176101
tanh (100, 100) 0.0001 200 : 0.8109988362425755, 0.8732838026938174
tanh (100, 100) 0.0001 100 : 0.8215669573166752, 0.8800643914924774
tanh (100, 100) 0.0001 50 : 0.8352368391121324, 0.8893597090821833
tanh (100, 100) 0.001 500 : 0.7442216564021252,

0.881032542064203
tanh (100, 100) 0.001 200 : 0.812193188160488, 0.8743814006922814
tanh (100, 100) 0.001 100 : 0.8240292750738221, 0.8795780476592497
tanh (100, 100) 0.001 50 : 0.8393843070265994, 0.8890990234670764
tanh (100, 100) 0.01 500 : 0.7682225476213121, 0.8910009947320144
tanh (100, 100) 0.01 200 : 0.8356673821646217, 0.8819141672059454
tanh (100, 100) 0.01 100 : 0.8447826756373702, 0.8985937098530128
tanh (100, 100) 0.01 50 : 0.8506270338093355, 0.9099504287002509
tanh (100, 200) 0.0001 500 : 0.7493185022656952, 0.8511306696887405
tanh (100, 200) 0.0001 200 : 0.8057531938329567, 0.8644001420980495
tanh (100, 200) 0.0001 100 : 0.8165627735378154, 0.880452177163126
tanh (100, 200) 0.0001 50 : 0.8313828419144121, 0.8912841518479192
tanh (100, 200) 0.001 500 : 0.7503417547187347, 0.8524920868090953
tanh (100, 200) 0.001 200 : 0.8065160744654207, 0.8646809005658235
tanh (100, 200) 0.001 100 : 0.8137963381143447, 0.8815563663991769
tanh (100, 200) 0.001 50 : 0.8307367129994141, 0.8922457136889699
tanh (100, 200) 0.01 500 : 0.75982680633873, 0.862193789661058
tanh (100, 200) 0.01 200 : 0.8140532994572635, 0.8687120076728772
tanh (100, 200) 0.01 100 : 0.8260795912599187, 0.8800998416425808
tanh (100, 200) 0.01 50 : 0.8333864252513239, 0.8984600025033647
tanh (50,) 0.0001 500 : 0.7548839562355857, 0.8952073279448935
tanh (50,) 0.0001 200 : 0.8097104173287804, 0.8851328812473739
tanh (50,) 0.0001 100 : 0.8154156936349043, 0.8897524211801138
tanh (50,) 0.0001 50 : 0.8180930103919307, 0.8944219154733146
tanh (50,) 0.001 500 : 0.7585797247537585, 0.8974721640188433
tanh (50,) 0.001 200 : 0.816210659032226, 0.8931128863180456
tanh (50,) 0.001 100 : 0.820846754501177, 0.89906032155478
tanh (50,) 0.001 50 : 0.8251268111265485, 0.9081753054627149
tanh (50,) 0.01 500 : 0.8016150535808546, 0.9118877760819584
tanh (50,) 0.01 200 : 0.8390552112907436, 0.9082637336591626
tanh (50,) 0.01 100 : 0.8433340674322505, 0.9121969941182861
tanh (50,) 0.01 50 : 0.849231621368633, 0.9225218013094009
tanh (50, 50) 0.0001 500 : 0.7343394412186808, 0.8643115062655045
tanh (50, 50) 0.0001 200 : 0.7976214723833831, 0.8698808321435918
tanh (50, 50) 0.0001 100 : 0.8115870561472083, 0.8832096171552025
tanh (50, 50) 0.0001 50 : 0.8291281771715345, 0.8937434066317683
tanh (50, 50) 0.001 500 : 0.7364674822583603, 0.8848853688586011
tanh (50, 50) 0.001 200 : 0.8089351724782168, 0.8772982033554712
tanh (50, 50) 0.001 100 : 0.8150195635570807, 0.8894404471217007
tanh (50, 50) 0.001 50 : 0.8302349250076935, 0.8961278711626617
tanh (50, 50) 0.01 500 : 0.7728907177309867, 0.8894920795965182
tanh (50, 50) 0.01 200 : 0.8328259164475341, 0.8822078740180805
tanh (50, 50) 0.01 100 : 0.8414845246360487, 0.9037293981782392
tanh (50, 50) 0.01 50 : 0.8474904824753565, 0.9114975013062478
tanh (50, 100) 0.0001 500 : 0.7387185421412374, 0.88381309147516
tanh (50, 100) 0.0001 200 : 0.8070779163628814, 0.8734851092459653
tanh (50, 100) 0.0001 100 : 0.8147746129152823, 0.879507501263813
tanh (50, 100) 0.0001 50 : 0.8306292266675308, 0.8918068348714802
tanh (50, 100) 0.001 500 : 0.7408182804974364, 0.8840996492399231
tanh (50, 100) 0.001 200 : 0.8101153164076141, 0.8755988845491555
tanh (50, 100) 0.001 100 : 0.8160220312794191, 0.8821214565559081
tanh (50, 100) 0.001 50 : 0.8299246983657664, 0.8953071318587268
tanh (50, 100) 0.01 500 : 0.7628476840689611, 0.8931928537541709
tanh (50, 100) 0.01 200 : 0.8214830398336884, 0.8829855279133889
tanh (50, 100) 0.01 100 : 0.8284739798196272, 0.888112522779948
tanh (50, 100) 0.01 50 : 0.8419352582706487, 0.8975883288685275
tanh (50, 200) 0.0001 500 : 0.7409694253381547, 0.8683792947213489
tanh (50, 200) 0.0001 200 : 0.8114070742567355, 0.8554309233176031
tanh (50, 200) 0.0001 100 : 0.8200730812025979, 0.8772536947010138
tanh (50, 200) 0.0001 50 : 0.8364861026754997, 0.8792885621595461
tanh (50, 200) 0.001 500 : 0.7416308772316746, 0.8687312072739748
tanh (50, 200) 0.001 200 : 0.8124668335090272, 0.8558660049225373
tanh (50, 200) 0.001 100 : 0.8204479943479583, 0.8775741179787603
tanh (50, 200) 0.001 50 : 0.834359264583387, 0.8832531637942588
tanh (50, 200) 0.01 500 : 0.747881984269647, 0.8759674152927592
tanh (50, 200) 0.01 200 : 0.8189204723765794, 0.8619609496687272
tanh (50, 200) 0.01 100 : 0.8247625460249788, 0.8791835877849263
tanh (50, 200) 0.01 50 : 0.8393445901301938, 0.8836771945898791
logistic (200,) 0.0001 500 : 0.7719352547757683, 0.883581246819691
logistic (200,) 0.0001 200 : 0.8238964640818036, 0.8745132211572975
logistic (200,) 0.0001 100 : 0.8262483280402322, 0.8831093450323502
logistic (200,) 0.0001 50 : 0.837242621607366, 0.8886143732725269
logistic (200,) 0.001 500 : 0.7777097420865808, 0.8896677093564985
logistic (200,) 0.001 200 : 0.8352144761835799, 0.8812026832599786
logistic (200,) 0.001 100 : 0.837479456940333, 0.889051163644971
logistic (200,) 0.001 50 : 0.8457159269266192, 0.8952342455755864
logistic (200,) 0.01 500 : 0.8205157077374035, 0.9028635550254122
logistic (200,) 0.01 200 : 0.8628437804802793, 0.9173752147157965
logistic (200,) 0.01 100 : 0.8630148783185161, 0.9166058347433207
logistic (200,) 0.01 50 : 0.8651410404385727, 0.913182464589705
logistic (200, 200) 0.0001 500 : 0.7657113187170669, 0.8578165679615444
logistic (200, 200) 0.0001 200 : 0.8162254649730908, 0.8436178383624486
logistic (200, 200) 0.0001 100 : 0.8236734209541975, 0.8751587510771989
logistic (200, 200) 0.0001 50 : 0.8399424258779209, 0.8875664981785342
logistic (200, 200) 0.001 500 : 0.7828699044655221, 0.8676716255775051
logistic (200, 200) 0.001 200 : 0.8278569580059111, 0.847511139028945
logistic (200, 200) 0.001 100 : 0.8332759531569363, 0.881809123522428
logistic (200, 200) 0.001 50 : 0.853753064832147, 0.8971419303746455
logistic (200, 200) 0.01 500 : 0.8269034715418864, 0.8960266790410788
logistic (200, 200) 0.01 200 : 0.8465782463181555, 0.8629310489471421
logistic (200, 200) 0.01 100 : 0.8535681254014283, 0.904739488803443
logistic (200, 200) 0.01 50 : 0.8670477133506095, 0.9096151597398103
logistic (100,) 0.0001 500 : 0.7752638739447064, 0.8881472514860884
logistic (100,) 0.0001 200 : 0.827114013847431, 0.8781598836392923
logistic (100,) 0.0001 100 : 0.8299156806031016, 0.8856495614939988
logistic (100,) 0.0001 50 : 0.840275628547011, 0.8939396401620074

logistic (100,) 0.001 500 : 0.7844335219996965,
0.8945059338072454
logistic (100,) 0.001 200 : 0.8404513811408243,
0.8900361582249914
logistic (100,) 0.001 100 : 0.8422364125269711,
0.8939071874336547
logistic (100,) 0.001 50 : 0.8497415420832224, 0.900457372695798
logistic (100,) 0.01 500 : 0.8344533204843761, 0.9023303757675554
logistic (100,) 0.01 200 : 0.8613420873484434, 0.9100636522142601
logistic (100,) 0.01 100 : 0.8630202922837211, 0.9125133533770922
logistic (100,) 0.01 50 : 0.8720684756282949, 0.9121083199552806
logistic (100, 100) 0.0001 500 : 0.7634652762839782,
0.8674241688364792
logistic (100, 100) 0.0001 200 : 0.825054552567248,
0.8426399727389602
logistic (100, 100) 0.0001 100 : 0.8329768410144585,
0.8711812954740229
logistic (100, 100) 0.0001 50 : 0.8498725570441034,
0.8892199414266072
logistic (100, 100) 0.001 500 : 0.7811439883758088,
0.8784116087430699
logistic (100, 100) 0.001 200 : 0.830584125856703,
0.8525035952101541
logistic (100, 100) 0.001 100 : 0.8377894548695402,
0.8760023843276163
logistic (100, 100) 0.001 50 : 0.8575910517554813,
0.9030069790348849
logistic (100, 100) 0.01 500 : 0.8080697866106613,
0.9014596987644432
logistic (100, 100) 0.01 200 : 0.8544560355900434,
0.8599244594818862
logistic (100, 100) 0.01 100 : 0.8587317296064263,
0.9039795383411585
logistic (100, 100) 0.01 50 : 0.8641025012472945,
0.9159140936228981
logistic (100, 200) 0.0001 500 : 0.750494195291075,
0.8553801091888703
logistic (100, 200) 0.0001 200 : 0.7994543674702418,
0.8239909346646449
logistic (100, 200) 0.0001 100 : 0.815534506581821,
0.863845907549292
logistic (100, 200) 0.0001 50 : 0.8382093661063111,
0.8848149486625747
logistic (100, 200) 0.001 500 : 0.7612619957349402,
0.853919324010778
logistic (100, 200) 0.001 200 : 0.8055610687616515,
0.8272090602586953
logistic (100, 200) 0.001 100 : 0.8250576091986082,
0.8787017116236192
logistic (100, 200) 0.001 50 : 0.84272979369144,
0.8931503510601556
logistic (100, 200) 0.01 500 : 0.8085948860573543,
0.8722049131290817
logistic (100, 200) 0.01 200 : 0.827422829550129,
0.8490499211315358
logistic (100, 200) 0.01 100 : 0.8356925681807279,
0.8740123135883655
logistic (100, 200) 0.01 50 : 0.8530117318828273,
0.8844091382670959
logistic (50,) 0.0001 500 : 0.7880216390128421,
0.8856492354703116
logistic (50,) 0.0001 200 : 0.8363560672064798,
0.8854421332100654
logistic (50,) 0.0001 100 : 0.8369554377073494,
0.8941613671572353
logistic (50,) 0.0001 50 : 0.8425826799590241, 0.8966594619508831
logistic (50,) 0.001 500 : 0.8065103224194715, 0.8915231831875469
logistic (50,) 0.001 200 : 0.8486871120656311, 0.9020316531863718
logistic (50,) 0.001 100 : 0.8483225326950755, 0.9064665766090048
logistic (50,) 0.001 50 : 0.8550813656023089, 0.9094628690681944
logistic (50,) 0.01 500 : 0.845953176841071, 0.9113335134369501
logistic (50,) 0.01 200 : 0.8618221876506507, 0.9109452216999677
logistic (50,) 0.01 100 : 0.8614161198410077, 0.9211150188496215
logistic (50,) 0.01 50 : 0.870222476640602, 0.92283260722114
logistic (50, 50) 0.0001 500 : 0.7695294516585252,
0.8546143685890759
logistic (50, 50) 0.0001 200 : 0.8238298456922477,
0.8512398351296696
logistic (50, 50) 0.0001 100 : 0.83561399199564,
0.8732248795566324
logistic (50, 50) 0.0001 50 : 0.8429928839941331,
0.8969212127958418
logistic (50, 50) 0.001 500 : 0.7951769668625394,
0.8885137470697665
logistic (50, 50) 0.001 200 : 0.8332032820457436,
0.8674748590055704
logistic (50, 50) 0.001 100 : 0.8409006075806793,
0.8988110585765853
logistic (50, 50) 0.001 50 : 0.8509268044562728,
0.8984051220167611
logistic (50, 50) 0.01 500 : 0.8156389018936228,
0.896132573445552
logistic (50, 50) 0.01 200 : 0.8514531368958445,
0.8685734583763692
logistic (50, 50) 0.01 100 : 0.8538409848357869,
0.8947424684186189
logistic (50, 50) 0.01 50 : 0.864836139060133, 0.9008514563970458
logistic (50, 100) 0.0001 500 : 0.7709141999530249,
0.8584846139798464
logistic (50, 100) 0.0001 200 : 0.812765992607311,
0.8366578524251093
logistic (50, 100) 0.0001 100 : 0.8349765746798813,
0.8855052309744204
logistic (50, 100) 0.0001 50 : 0.8468307817347762,
0.8944446334886818
logistic (50, 100) 0.001 500 : 0.7851079677615422,
0.8687174549368583
logistic (50, 100) 0.001 200 : 0.8197120724885021,
0.8433701631779632
logistic (50, 100) 0.001 100 : 0.8300129880299909,
0.8946380301644872
logistic (50, 100) 0.001 50 : 0.8504723072835578,
0.8983787945956665
logistic (50, 100) 0.01 500 : 0.8236406530970495,
0.8781277850134974
logistic (50, 100) 0.01 200 : 0.8441054637316394,
0.8647075862434168
logistic (50, 100) 0.01 100 : 0.8499403288862621,
0.8850745578585482
logistic (50, 100) 0.01 50 : 0.8667121917234223,
0.9054575049806679
logistic (50, 200) 0.0001 500 : 0.754239878045417,
0.8403698325795662
logistic (50, 200) 0.0001 200 : 0.810925009319859,
0.844144164400245
logistic (50, 200) 0.0001 100 : 0.8184962953613544,
0.8737854133627182
logistic (50, 200) 0.0001 50 : 0.8411740355534689,
0.8851190625815542
logistic (50, 200) 0.001 500 : 0.7637663624647816,
0.8479952547760996
logistic (50, 200) 0.001 200 : 0.8167945062058655,
0.8473899965629503
logistic (50, 200) 0.001 100 : 0.8200495397409219,
0.8828424601664653
logistic (50, 200) 0.001 50 : 0.8462574328118935,
0.8912724852463099
logistic (50, 200) 0.01 500 : 0.8081844936178714,
0.8695122039300766
logistic (50, 200) 0.01 200 : 0.8371777006040018,
0.876458925090211
logistic (50, 200) 0.01 100 : 0.8520313778590796,
0.8951145593981376
logistic (50, 200) 0.01 50 : 0.8575987824113984,
0.9003560908271623

Grid search of LightGBM, Morgan(r=4,2048)
lgb.LGBMRegressor(boosting_type = "gbdt", num_leaves =

j,max_depth = 0)
j: accuracy of prediction using cross validation, accuracy of prediction using test data
10 : 0.7785605687905894, 0.8531616746008784
50 : 0.7803697097007957, 0.8561794833842031
100 : 0.7803697097007957, 0.8561794833842031
150 : 0.7803697097007957, 0.8561794833842031

Grid search of SVR, Morgan(r=4, 2048)
SVR(C= c_num, kernel = ker, epsilon = e, gamma = r,degree = 3, coef0=1)
Ker c_num r e: accuracy of prediction using cross validation, accuracy of prediction using test data
linear 1 1 0.1 : 0.8884129454045565, 0.9419209130882819
linear 1 1 0.3 : 0.8871712530950469, 0.9407173844468545
linear 1 1 0.5 : 0.8853708459084212, 0.9391557013478362
linear 1 1 0.7 : 0.8833548596359572, 0.9374484691533025
linear 1 1 0.9 : 0.881067970775135, 0.93570846668608
linear 1 2 0.1 : 0.8884129454045565, 0.9419209130882819
linear 1 2 0.3 : 0.8871712530950469, 0.9407173844468545
linear 1 2 0.5 : 0.8853708459084212, 0.9391557013478362
linear 1 2 0.7 : 0.8833548596359572, 0.9374484691533025
linear 1 2 0.9 : 0.881067970775135, 0.93570846668608
linear 1 3 0.1 : 0.8884129454045565, 0.9419209130882819
linear 1 3 0.3 : 0.8871712530950469, 0.9407173844468545
linear 1 3 0.5 : 0.8853708459084212, 0.9391557013478362
linear 1 3 0.7 : 0.8833548596359572, 0.9374484691533025
linear 1 3 0.9 : 0.881067970775135, 0.93570846668608
linear 1 4 0.1 : 0.8884129454045565, 0.9419209130882819
linear 1 4 0.3 : 0.8871712530950469, 0.9407173844468545
linear 1 4 0.5 : 0.8853708459084212, 0.9391557013478362
linear 1 4 0.7 : 0.8833548596359572, 0.9374484691533025
linear 1 4 0.9 : 0.881067970775135, 0.93570846668608
linear 1 5 0.1 : 0.8884129454045565, 0.9419209130882819
linear 1 5 0.3 : 0.8871712530950469, 0.9407173844468545
linear 1 5 0.5 : 0.8853708459084212, 0.9391557013478362
linear 1 5 0.7 : 0.8833548596359572, 0.9374484691533025
linear 1 5 0.9 : 0.881067970775135, 0.93570846668608
linear 1 6 0.1 : 0.8884129454045565, 0.9419209130882819
linear 1 6 0.3 : 0.8871712530950469, 0.9407173844468545
linear 1 6 0.5 : 0.8853708459084212, 0.9391557013478362
linear 1 6 0.7 : 0.8833548596359572, 0.9374484691533025
linear 1 6 0.9 : 0.881067970775135, 0.93570846668608
linear 2 1 0.1 : 0.8958655391760756, 0.9407278490856402
linear 2 1 0.3 : 0.8942592414327823, 0.9394200008224591
linear 2 1 0.5 : 0.8923091046856954, 0.937732416950623
linear 2 1 0.7 : 0.8901136387725466, 0.9358066437626267
linear 2 1 0.9 : 0.8876033693371023, 0.9337724261557635
linear 2 2 0.1 : 0.8958655391760756, 0.9407278490856402
linear 2 2 0.3 : 0.8942592414327823, 0.9394200008224591
linear 2 2 0.5 : 0.8923091046856954, 0.937732416950623
linear 2 2 0.7 : 0.8901136387725466, 0.9358066437626267
linear 2 2 0.9 : 0.8876033693371023, 0.9337724261557635
linear 2 3 0.1 : 0.8958655391760756, 0.9407278490856402
linear 2 3 0.3 : 0.8942592414327823, 0.9394200008224591
linear 2 3 0.5 : 0.8923091046856954, 0.937732416950623
linear 2 3 0.7 : 0.8901136387725466, 0.9358066437626267
linear 2 3 0.9 : 0.8876033693371023, 0.9337724261557635
linear 2 4 0.1 : 0.8958655391760756, 0.9407278490856402
linear 2 4 0.3 : 0.8942592414327823, 0.9394200008224591
linear 2 4 0.5 : 0.8923091046856954, 0.937732416950623
linear 2 4 0.7 : 0.8901136387725466, 0.9358066437626267
linear 2 4 0.9 : 0.8876033693371023, 0.9337724261557635
linear 2 5 0.1 : 0.8958655391760756, 0.9407278490856402
linear 2 5 0.3 : 0.8942592414327823, 0.9394200008224591
linear 2 5 0.5 : 0.8923091046856954, 0.937732416950623
linear 2 5 0.7 : 0.8901136387725466, 0.9358066437626267
linear 2 5 0.9 : 0.8876033693371023, 0.9337724261557635
linear 2 6 0.1 : 0.8958655391760756, 0.9407278490856402
linear 2 6 0.3 : 0.8942592414327823, 0.9394200008224591
linear 2 6 0.5 : 0.8923091046856954, 0.937732416950623
linear 2 6 0.7 : 0.8901136387725466, 0.9358066437626267
linear 2 6 0.9 : 0.8876033693371023, 0.9337724261557635
linear 3 1 0.1 : 0.8969945281656363, 0.9395387831122916
linear 3 1 0.3 : 0.895287471525342, 0.9384235090702554
linear 3 1 0.5 : 0.8932061592760558, 0.936868185320464
linear 3 1 0.7 : 0.8908301311238901, 0.9351932213931721
linear 3 1 0.9 : 0.8881931342883906, 0.9334336930677293
linear 3 2 0.1 : 0.8969945281656363, 0.9395387831122916
linear 3 2 0.3 : 0.895287471525342, 0.9384235090702554
linear 3 2 0.5 : 0.8932061592760558, 0.936868185320464
linear 3 2 0.7 : 0.8908301311238901, 0.9351932213931721
linear 3 2 0.9 : 0.8881931342883906, 0.9334336930677293
linear 3 3 0.1 : 0.8969945281656363, 0.9395387831122916
linear 3 3 0.3 : 0.895287471525342, 0.9384235090702554
linear 3 3 0.5 : 0.8932061592760558, 0.936868185320464
linear 3 3 0.7 : 0.8908301311238901, 0.9351932213931721
linear 3 3 0.9 : 0.8881931342883906, 0.9334336930677293
linear 3 4 0.1 : 0.8969945281656363, 0.9395387831122916
linear 3 4 0.3 : 0.895287471525342, 0.9384235090702554
linear 3 4 0.5 : 0.8932061592760558, 0.936868185320464
linear 3 4 0.7 : 0.8908301311238901, 0.9351932213931721
linear 3 4 0.9 : 0.8881931342883906, 0.9334336930677293
linear 3 5 0.1 : 0.8969945281656363, 0.9395387831122916
linear 3 5 0.3 : 0.895287471525342, 0.9384235090702554
linear 3 5 0.5 : 0.8932061592760558, 0.936868185320464
linear 3 5 0.7 : 0.8908301311238901, 0.9351932213931721
linear 3 5 0.9 : 0.8881931342883906, 0.9334336930677293
linear 3 6 0.1 : 0.8969945281656363, 0.9395387831122916
linear 3 6 0.3 : 0.895287471525342, 0.9384235090702554
linear 3 6 0.5 : 0.8932061592760558, 0.936868185320464
linear 3 6 0.7 : 0.8908301311238901, 0.9351932213931721
linear 3 6 0.9 : 0.8881931342883906, 0.9334336930677293
linear 4 1 0.1 : 0.8971776037231566, 0.939281853063647
linear 4 1 0.3 : 0.8954092813586847, 0.938146982525819
linear 4 1 0.5 : 0.8932996915791087, 0.9365907946318475
linear 4 1 0.7 : 0.8909386077987957, 0.9349466096714066
linear 4 1 0.9 : 0.8883585356911979, 0.9332254619174953
linear 4 2 0.1 : 0.8971776037231566, 0.939281853063647
linear 4 2 0.3 : 0.8954092813586847, 0.938146982525819
linear 4 2 0.5 : 0.8932996915791087, 0.9365907946318475
linear 4 2 0.7 : 0.8909386077987957, 0.9349466096714066
linear 4 2 0.9 : 0.8883585356911979, 0.9332254619174953
linear 4 3 0.1 : 0.8971776037231566, 0.939281853063647
linear 4 3 0.3 : 0.8954092813586847, 0.938146982525819
linear 4 3 0.5 : 0.8932996915791087, 0.9365907946318475
linear 4 3 0.7 : 0.8909386077987957, 0.9349466096714066
linear 4 3 0.9 : 0.8883585356911979, 0.9332254619174953
linear 4 4 0.1 : 0.8971776037231566, 0.939281853063647
linear 4 4 0.3 : 0.8954092813586847, 0.938146982525819
linear 4 4 0.5 : 0.8932996915791087, 0.9365907946318475
linear 4 4 0.7 : 0.8909386077987957, 0.9349466096714066
linear 4 4 0.9 : 0.8883585356911979, 0.9332254619174953
linear 4 5 0.1 : 0.8971776037231566, 0.939281853063647
linear 4 5 0.3 : 0.8954092813586847, 0.938146982525819
linear 4 5 0.5 : 0.8932996915791087, 0.9365907946318475
linear 4 5 0.7 : 0.8909386077987957, 0.9349466096714066
linear 4 5 0.9 : 0.8883585356911979, 0.9332254619174953
linear 4 6 0.1 : 0.8971776037231566, 0.939281853063647
linear 4 6 0.3 : 0.8954092813586847, 0.938146982525819
linear 4 6 0.5 : 0.8932996915791087, 0.9365907946318475
linear 4 6 0.7 : 0.8909386077987957, 0.9349466096714066
linear 4 6 0.9 : 0.8883585356911979, 0.9332254619174953
poly 1 1 0.1 : 0.7510490926189907, 0.811254476571938
poly 1 1 0.3 : 0.7476163913179631, 0.8073512145300794
poly 1 1 0.5 : 0.743969142627298, 0.803346256343104
poly 1 1 0.7 : 0.7401087825658711, 0.7992593179537812
poly 1 1 0.9 : 0.736067185417771, 0.7950518130627893
poly 1 2 0.1 : 0.7490293854577688, 0.8090391234940422
poly 1 2 0.3 : 0.7455802650930196, 0.8051161059388152
poly 1 2 0.5 : 0.7419207453267905, 0.8010984766238067
poly 1 2 0.7 : 0.7380378384927913, 0.7969910563548148
poly 1 2 0.9 : 0.7339895094625298, 0.7927676167833584
poly 1 3 0.1 : 0.7483440161181045, 0.808285268380824
poly 1 3 0.3 : 0.7448890473715041, 0.80435508924169
poly 1 3 0.5 : 0.7412249160021374, 0.8003345244523171
poly 1 3 0.7 : 0.7373353450759336, 0.7962223143654327
poly 1 3 0.9 : 0.7332832868027427, 0.791989124783418

poly 1 4 0.1 : 0.7479984791001496, 0.8079044872503685
poly 1 4 0.3 : 0.7445406287421967, 0.8039719038413613
poly 1 4 0.5 : 0.7408738819846772, 0.7999492389103224
poly 1 4 0.7 : 0.7369816072960391, 0.7958333230516814
poly 1 4 0.9 : 0.7329280690533018, 0.7915970661416252
poly 1 5 0.1 : 0.7477910980565258, 0.8076761091992833
poly 1 5 0.3 : 0.744331450227275, 0.8037414412180239
poly 1 5 0.5 : 0.740662938391174, 0.7997176091966633
poly 1 5 0.7 : 0.7367687437153172, 0.7956001493928975
poly 1 5 0.9 : 0.7327143295315224, 0.7913620684383075
poly 1 6 0.1 : 0.7476521568019324, 0.8075227694342983
poly 1 6 0.3 : 0.7441910379097617, 0.8035867196811601
poly 1 6 0.5 : 0.7405217478918915, 0.7995622339946927
poly 1 6 0.7 : 0.7366267494707648, 0.7954440675224766
poly 1 6 0.9 : 0.7325722855782536, 0.7912033457767111
poly 2 1 0.1 : 0.7510490926190452, 0.8112544765719745
poly 2 1 0.3 : 0.747616391317876, 0.8073512145305921
poly 2 1 0.5 : 0.7439691426273353, 0.8033462563430052
poly 2 1 0.7 : 0.7401087825658194, 0.7992593179537943
poly 2 1 0.9 : 0.736067185417771, 0.7950518130627893
poly 2 2 0.1 : 0.7490293854570818, 0.8090391234939499
poly 2 2 0.3 : 0.7455802650914302, 0.8051161059380366
poly 2 2 0.5 : 0.7419207453280521, 0.8010984766230617
poly 2 2 0.7 : 0.7380378384928459, 0.7969910563546815
poly 2 2 0.9 : 0.7339895094625298, 0.7927676167833584
poly 2 3 0.1 : 0.7483440161206893, 0.8082852683721276
poly 2 3 0.3 : 0.7448890473732362, 0.8043550892430956
poly 2 3 0.5 : 0.7412249160024101, 0.8003345244512552
poly 2 3 0.7 : 0.7373353450779432, 0.7962223143647327
poly 2 3 0.9 : 0.7332832868027427, 0.791989124783418
poly 2 4 0.1 : 0.7479984790939477, 0.8079044872519404
poly 2 4 0.3 : 0.7445406287506143, 0.8039719038363279
poly 2 4 0.5 : 0.7408738819684692, 0.7999492389077821
poly 2 4 0.7 : 0.7369816073013028, 0.7958333230563392
poly 2 4 0.9 : 0.7329280690533018, 0.7915970661416252
poly 2 5 0.1 : 0.7477910980564841, 0.8076761092165705
poly 2 5 0.3 : 0.7443314502305965, 0.8037414412077705
poly 2 5 0.5 : 0.7406629383730243, 0.7997176092362843
poly 2 5 0.7 : 0.7367687437133303, 0.7956001494046545
poly 2 5 0.9 : 0.7327143295315224, 0.7913620684383075
poly 2 6 0.1 : 0.7476521567787543, 0.8075227693493614
poly 2 6 0.3 : 0.7441910378921917, 0.8035867197067431
poly 2 6 0.5 : 0.7405217479260733, 0.799562233990582
poly 2 6 0.7 : 0.7366267494642986, 0.7954440675346006
poly 2 6 0.9 : 0.7325722855782536, 0.7912033457767111
poly 3 1 0.1 : 0.75104909261923, 0.8112544765724019
poly 3 1 0.3 : 0.7476163913177789, 0.8073512145306986
poly 3 1 0.5 : 0.7439691426272013, 0.8033462563429538
poly 3 1 0.7 : 0.7401087825657897, 0.7992593179539264
poly 3 1 0.9 : 0.736067185417771, 0.7950518130627893
poly 3 2 0.1 : 0.7490293854578975, 0.8090391234917631
poly 3 2 0.3 : 0.745580265093521, 0.8051161059375462
poly 3 2 0.5 : 0.7419207453284062, 0.8010984766251894
poly 3 2 0.7 : 0.7380378384923872, 0.7969910563569351
poly 3 2 0.9 : 0.7339895094625298, 0.7927676167833584
poly 3 3 0.1 : 0.7483440161246724, 0.808285268371324
poly 3 3 0.3 : 0.7448890473682799, 0.8043550892460105
poly 3 3 0.5 : 0.7412249160051163, 0.8003345244454365
poly 3 3 0.7 : 0.7373353450791861, 0.7962223143695117
poly 3 3 0.9 : 0.7332832868027427, 0.791989124783418
poly 3 4 0.1 : 0.7479984790779814, 0.8079044872466212
poly 3 4 0.3 : 0.7445406287483237, 0.803971903836756
poly 3 4 0.5 : 0.7408738819663399, 0.7999492389024101
poly 3 4 0.7 : 0.7369816072954317, 0.7958333230407646
poly 3 4 0.9 : 0.7329280690533018, 0.7915970661416252
poly 3 5 0.1 : 0.7477910980689036, 0.8076761091918673
poly 3 5 0.3 : 0.7443314502292699, 0.8037414411735424
poly 3 5 0.5 : 0.7406629383984796, 0.7997176091849899
poly 3 5 0.7 : 0.7367687437136448, 0.7956001493909588
poly 3 5 0.9 : 0.7327143295315224, 0.7913620684383075
poly 3 6 0.1 : 0.7476521567892644, 0.8075227694356826
poly 3 6 0.3 : 0.7441910379027389, 0.8035867197438946
poly 3 6 0.5 : 0.7405217479027438, 0.7995622339848503
poly 3 6 0.7 : 0.7366267494600518, 0.7954440675584287
poly 3 6 0.9 : 0.7325722855782536, 0.7912033457767111
poly 4 1 0.1 : 0.7510490926191616, 0.811254476571728
poly 4 1 0.3 : 0.7476163913177749, 0.8073512145303317
poly 4 1 0.5 : 0.7439691426272897, 0.8033462563428742
poly 4 1 0.7 : 0.740108782565777, 0.7992593179537125
poly 4 1 0.9 : 0.736067185417771, 0.7950518130627893
poly 4 2 0.1 : 0.749029385456295, 0.8090391234905062
poly 4 2 0.3 : 0.7455802650944134, 0.8051161059386653
poly 4 2 0.5 : 0.7419207453266348, 0.8010984766260683
poly 4 2 0.7 : 0.7380378384929507, 0.7969910563580771
poly 4 2 0.9 : 0.7339895094625298, 0.7927676167833584
poly 4 3 0.1 : 0.7483440161250821, 0.8082852683748197
poly 4 3 0.3 : 0.7448890473627066, 0.8043550892545728
poly 4 3 0.5 : 0.7412249160081752, 0.8003345244571075
poly 4 3 0.7 : 0.7373353450689683, 0.7962223143786058
poly 4 3 0.9 : 0.7332832868027427, 0.791989124783418
poly 4 4 0.1 : 0.7479984790736447, 0.807904487263623
poly 4 4 0.3 : 0.7445406287493148, 0.8039719038683086
poly 4 4 0.5 : 0.7408738819741908, 0.7999492389174511
poly 4 4 0.7 : 0.7369816072947704, 0.7958333230550931
poly 4 4 0.9 : 0.7329280690533018, 0.7915970661416252
poly 4 5 0.1 : 0.7477910980331176, 0.8076761091825618
poly 4 5 0.3 : 0.7443314502217037, 0.803741441188544
poly 4 5 0.5 : 0.7406629383955864, 0.7997176092020464
poly 4 5 0.7 : 0.7367687436920106, 0.7956001494107422
poly 4 5 0.9 : 0.7327143295315224, 0.7913620684383075
poly 4 6 0.1 : 0.7476521568408104, 0.8075227694874466
poly 4 6 0.3 : 0.7441910378924386, 0.8035867196903664
poly 4 6 0.5 : 0.7405217478409142, 0.7995622340414781
poly 4 6 0.7 : 0.7366267495313623, 0.7954440675126409
poly 4 6 0.9 : 0.7325722855782536, 0.7912033457767111
rbf 1 1 0.1 : -0.02737366020648242, 0.0015652580037851527
rbf 1 1 0.3 : -0.02711955417923386, 0.001533887594037608
rbf 1 1 0.5 : -0.02651479790875788, 0.0014637524860022788
rbf 1 1 0.7 : -0.02572046266457111, 0.0014166939380562127
rbf 1 1 0.9 : -0.024462500471276138, 0.0013857749318897383
rbf 1 2 0.1 : -0.027373674033629936, 0.001565259389008422
rbf 1 2 0.3 : -0.027119568008564875, 0.0015338889678809808
rbf 1 2 0.5 : -0.026514811748516866, 0.0014637538367194836
rbf 1 2 0.7 : -0.025720476515565992, 0.00141669527465304
rbf 1 2 0.9 : -0.024462553208706782, 0.001385776265193428
rbf 1 3 0.1 : -0.027373674033707475, 0.0015652593890191913
rbf 1 3 0.3 : -0.027119568008642327, 0.00153388896789175
rbf 1 3 0.5 : -0.0265148117485944, 0.0014637538367301417
rbf 1 3 0.7 : -0.025720476515643576, 0.001416695274663704
rbf 1 3 0.9 : -0.024462553208784633, 0.0013857762652040861
rbf 1 4 0.1 : -0.027373674033707475, 0.0015652593890191913
rbf 1 4 0.3 : -0.027119568008642327, 0.00153388896789175
rbf 1 4 0.5 : -0.0265148117485944, 0.0014637538367301417
rbf 1 4 0.7 : -0.025720476515643576, 0.001416695274663704
rbf 1 4 0.9 : -0.024462553208784633, 0.0013857762652040861
rbf 1 5 0.1 : -0.027373674033707475, 0.0015652593890191913
rbf 1 5 0.3 : -0.027119568008642327, 0.00153388896789175
rbf 1 5 0.5 : -0.0265148117485944, 0.0014637538367301417
rbf 1 5 0.7 : -0.025720476515643576, 0.001416695274663704
rbf 1 5 0.9 : -0.024462553208784633, 0.0013857762652040861
rbf 1 6 0.1 : -0.027373674033707475, 0.0015652593890191913
rbf 1 6 0.3 : -0.027119568008642327, 0.00153388896789175
rbf 1 6 0.5 : -0.0265148117485944, 0.0014637538367301417
rbf 1 6 0.7 : -0.025720476515643576, 0.001416695274663704
rbf 1 6 0.9 : -0.024462553208784633, 0.0013857762652040861
rbf 2 1 0.1 : -0.024727663251207323, 0.0030568980893341813
rbf 2 1 0.3 : -0.02404009592963572, 0.0029815443199633362
rbf 2 1 0.5 : -0.02336034369229272, 0.0028817989915013165
rbf 2 1 0.7 : -0.02255315439081751, 0.00276299159347759
rbf 2 1 0.9 : -0.021807445798730462, 0.0026372986911172847
rbf 2 2 0.1 : -0.02472769099822294, 0.003056900881847291
rbf 2 2 0.3 : -0.02404012371672861, 0.0029815472179688385
rbf 2 2 0.5 : -0.023360371723766392, 0.00288180198401655
rbf 2 2 0.7 : -0.022553182438433562, 0.0027629946724857524
rbf 2 2 0.9 : -0.021807473838767742, 0.0026373018529726178
rbf 2 3 0.1 : -0.02472769099837846, 0.003056900881868718
rbf 2 3 0.3 : -0.02404012371688431, 0.002981547217990266
rbf 2 3 0.5 : -0.023360371723922534, 0.0028818019840379794

rbf 2 3 0.7 : -0.022553182438589968, 0.0027629946725071797
rbf 2 3 0.9 : -0.021807473838924007, 0.002637301852993823
rbf 2 4 0.1 : -0.02472769099837846, 0.0030569008818687182
rbf 2 4 0.3 : -0.02404012371688431, 0.002981547217990266
rbf 2 4 0.5 : -0.023360371723922534, 0.0028818019840379794
rbf 2 4 0.7 : -0.022553182438589968, 0.0027629946725071797
rbf 2 4 0.9 : -0.021807473838924007, 0.002637301852993823
rbf 2 5 0.1 : -0.02472769099837846, 0.0030569008818687182
rbf 2 5 0.3 : -0.02404012371688431, 0.002981547217990266
rbf 2 5 0.5 : -0.023360371723922534, 0.0028818019840379794
rbf 2 5 0.7 : -0.022553182438589968, 0.0027629946725071797
rbf 2 5 0.9 : -0.021807473838924007, 0.002637301852993823
rbf 2 6 0.1 : -0.02472769099837846, 0.0030569008818687182
rbf 2 6 0.3 : -0.02404012371688431, 0.002981547217990266
rbf 2 6 0.5 : -0.023360371723922534, 0.0028818019840379794
rbf 2 6 0.7 : -0.022553182438589968, 0.0027629946725071797
rbf 2 6 0.9 : -0.021807473838924007, 0.002637301852993823
rbf 3 1 0.1 : -0.02190006051793434, 0.004171448721535631
rbf 3 1 0.3 : -0.02114435741751066, 0.004064367024394389
rbf 3 1 0.5 : -0.02024590028942628, 0.003916007097081287
rbf 3 1 0.7 : -0.01919563636611521, 0.0036647317977546168
rbf 3 1 0.9 : -0.018109508945703734, 0.0033469851981235976
rbf 3 2 0.1 : -0.021900102501394337, 0.004171453224730337
rbf 3 2 0.3 : -0.02114437026313376, 0.004064371596202099
rbf 3 2 0.5 : -0.02024590993347517, 0.003916011714145462
rbf 3 2 0.7 : -0.019195699579830937, 0.0036647364104955837
rbf 3 2 0.9 : -0.018109566659078258, 0.0033469897792893777
rbf 3 3 0.1 : -0.021900102501629215, 0.004171453224761756
rbf 3 3 0.3 : -0.021144370263368906, 0.00406437159623374
rbf 3 3 0.5 : -0.020245909933710938, 0.003916011714176548
rbf 3 3 0.7 : -0.019195699580067015, 0.003664736410526115
rbf 3 3 0.9 : -0.018109566659314826, 0.0033469897793192427
rbf 3 4 0.1 : -0.021900102501629215, 0.004171453224761756
rbf 3 4 0.3 : -0.021144370263368906, 0.00406437159623374
rbf 3 4 0.5 : -0.020245909933710938, 0.003916011714176548
rbf 3 4 0.7 : -0.019195699580067015, 0.003664736410526115
rbf 3 4 0.9 : -0.018109566659314826, 0.0033469897793192427
rbf 3 5 0.1 : -0.021900102501629215, 0.004171453224761756
rbf 3 5 0.3 : -0.021144370263368906, 0.00406437159623374
rbf 3 5 0.5 : -0.020245909933710938, 0.003916011714176548
rbf 3 5 0.7 : -0.019195699580067015, 0.003664736410526115
rbf 3 5 0.9 : -0.018109566659314826, 0.0033469897793192427
rbf 3 6 0.1 : -0.021900102501629215, 0.004171453224761756
rbf 3 6 0.3 : -0.021144370263368906, 0.00406437159623374
rbf 3 6 0.5 : -0.020245909933710938, 0.003916011714176548
rbf 3 6 0.7 : -0.019195699580067015, 0.003664736410526115
rbf 3 6 0.9 : -0.018109566659314826, 0.0033469897793192427
rbf 4 1 0.1 : -0.0180147965507206, 0.004589094483824496
rbf 4 1 0.3 : -0.016968752063141235, 0.004157525640684279
rbf 4 1 0.5 : -0.015935840727316863, 0.0036989980683035384
rbf 4 1 0.7 : -0.014987167111819933, 0.0032186570587708907
rbf 4 1 0.9 : -0.014054003781555191, 0.002613952463258906
rbf 4 2 0.1 : -0.01801485284874569, 0.004589100157873838
rbf 4 2 0.3 : -0.016968779358160813, 0.004157531191201436
rbf 4 2 0.5 : -0.015935926904985243, 0.0036990034903048308
rbf 4 2 0.7 : -0.014987223851878095, 0.0032186623484490307
rbf 4 2 0.9 : -0.014054037342099979, 0.0026139575703744145
rbf 4 3 0.1 : -0.018014852849061257, 0.0045891001579123625
rbf 4 3 0.3 : -0.016968779358477758, 0.00415753119123885
rbf 4 3 0.5 : -0.015935926905303033, 0.003699003490341024
rbf 4 3 0.7 : -0.014987202741356542, 0.0032186623484837806
rbf 4 3 0.9 : -0.014054037342419634, 0.00261395757040761
rbf 4 4 0.1 : -0.018014852849061257, 0.0045891001579123625
rbf 4 4 0.3 : -0.016968779358477758, 0.00415753119123885
rbf 4 4 0.5 : -0.015935926905303033, 0.003699003490341024
rbf 4 4 0.7 : -0.014987202741356542, 0.0032186623484837806
rbf 4 4 0.9 : -0.014054037342419634, 0.00261395757040761
rbf 4 5 0.1 : -0.018014852849061257, 0.0045891001579123625
rbf 4 5 0.3 : -0.016968779358477758, 0.00415753119123885
rbf 4 5 0.5 : -0.015935926905303033, 0.003699003490341024
rbf 4 5 0.7 : -0.014987202741356542, 0.0032186623484837806
rbf 4 5 0.9 : -0.014054037342419634, 0.00261395757040761
rbf 4 6 0.1 : -0.018014852849061257, 0.0045891001579123625
rbf 4 6 0.3 : -0.016968779358477758, 0.00415753119123885
rbf 4 6 0.5 : -0.015935926905303033, 0.003699003490341024
rbf 4 6 0.7 : -0.014987202741356542, 0.0032186623484837806
rbf 4 6 0.9 : -0.014054037342419634, 0.00261395757040761
sigmoid 1 1 0.1 : -0.027851678082275465, -0.000481767492397589
sigmoid 1 1 0.3 : -0.028038598613550337, -0.000152744900453472
sigmoid 1 1 0.5 : -0.029327649088433237, -8.3949395648375e-05
sigmoid 1 1 0.7 : -0.02737076999122836, -0.0001527460770722744
sigmoid 1 1 0.9 : -0.02742589813888454, -0.00035162237767938365
sigmoid 1 2 0.1 : -0.0278515749445718, -0.0004817589183705451
sigmoid 1 2 0.3 : -0.028038637424910594, -
0.00015273486537625836
sigmoid 1 2 0.5 : -0.029327682929672427, -8.393840800002828e-05
sigmoid 1 2 0.7 : -0.027370781055612258, -
0.00015273486537581427
sigmoid 1 2 0.9 : -0.02742593928217043, -0.0003516451205616278
sigmoid 1 3 0.1 : -0.02785157494475108, -0.0004817589182650739
sigmoid 1 3 0.3 : -0.028038637425086766, -0.0001527348652718974
sigmoid 1 3 0.5 : -0.029327682929847977, -8.39384078961114e-05
sigmoid 1 3 0.7 : -0.027370781055785543, -0.0001527348652718974
sigmoid 1 3 0.9 : -0.02742593928234638, -0.0003516451204561566
sigmoid 1 4 0.1 : -0.02785157494475108, -0.0004817589182650739
sigmoid 1 4 0.3 : -0.028038637425086766, -0.0001527348652718974
sigmoid 1 4 0.5 : -0.029327682929847977, -8.39384078961114e-05
sigmoid 1 4 0.7 : -0.027370781055785543, -0.0001527348652718974
sigmoid 1 4 0.9 : -0.02742593928234638, -0.0003516451204561566
sigmoid 1 5 0.1 : -0.02785157494475108, -0.0004817589182650739
sigmoid 1 5 0.3 : -0.028038637425086766, -0.0001527348652718974
sigmoid 1 5 0.5 : -0.029327682929847977, -8.39384078961114e-05
sigmoid 1 5 0.7 : -0.027370781055785543, -0.0001527348652718974
sigmoid 1 5 0.9 : -0.02742593928234638, -0.0003516451204561566
sigmoid 1 6 0.1 : -0.02785157494475108, -0.0004817589182650739
sigmoid 1 6 0.3 : -0.028038637425086766, -0.0001527348652718974
sigmoid 1 6 0.5 : -0.029327682929847977, -8.39384078961114e-05
sigmoid 1 6 0.7 : -0.027370781055785543, -0.0001527348652718974
sigmoid 1 6 0.9 : -0.02742593928234638, -0.0003516451204561566
sigmoid 2 1 0.1 : -0.027851781220634607, -
0.00048177606678478924
sigmoid 2 1 0.3 : -0.028038559802391116, -0.0001527549358864011
sigmoid 2 1 0.5 : -0.02932761524740446, -8.396038364955061e-05
sigmoid 2 1 0.7 : -0.02737075892710559, -0.00015275728912178543
sigmoid 2 1 0.9 : -0.027425856995805686, -0.0003515996367922103
sigmoid 2 2 0.1 : -0.027851574944392433, -
0.00048175891847601626
sigmoid 2 2 0.3 : -0.028038637424734246, -
0.00015273486548039727
sigmoid 2 2 0.5 : -0.029327682929496703, -8.39384081037231e-05
sigmoid 2 2 0.7 : -0.02737078105543893, -0.00015273486548017523
sigmoid 2 2 0.9 : -0.02742593928199453, -0.0003516451206668769
sigmoid 2 3 0.1 : -0.02785157494475108, -0.0004817589182650739
sigmoid 2 3 0.3 : -0.028038637425086766, -0.0001527348652718974
sigmoid 2 3 0.5 : -0.029327682929847977, -8.39384078961114e-05
sigmoid 2 3 0.7 : -0.027370781055785543, -0.0001527348652718974
sigmoid 2 3 0.9 : -0.02742593928234638, -0.0003516451204561566
sigmoid 2 4 0.1 : -0.02785157494475108, -0.0004817589182650739
sigmoid 2 4 0.3 : -0.028038637425086766, -0.0001527348652718974
sigmoid 2 4 0.5 : -0.029327682929847977, -8.39384078961114e-05
sigmoid 2 4 0.7 : -0.027370781055785543, -0.0001527348652718974
sigmoid 2 4 0.9 : -0.02742593928234638, -0.0003516451204561566
sigmoid 2 5 0.1 : -0.02785157494475108, -0.0004817589182650739
sigmoid 2 5 0.3 : -0.028038637425086766, -0.0001527348652718974
sigmoid 2 5 0.5 : -0.029327682929847977, -8.39384078961114e-05
sigmoid 2 5 0.7 : -0.027370781055785543, -0.0001527348652718974
sigmoid 2 5 0.9 : -0.02742593928234638, -0.0003516451204561566
sigmoid 2 6 0.1 : -0.02785157494475108, -0.0004817589182650739
sigmoid 2 6 0.3 : -0.028038637425086766, -0.0001527348652718974
sigmoid 2 6 0.5 : -0.029327682929847977, -8.39384078961114e-05
sigmoid 2 6 0.7 : -0.027370781055785543, -0.0001527348652718974
sigmoid 2 6 0.9 : -0.02742593928234638, -0.0003516451204561566
sigmoid 3 1 0.1 : -0.02785188435982775, -0.0004817846414260085
sigmoid 3 1 0.3 : -0.028038520991608883, -
0.00015276497157112878
sigmoid 3 1 0.5 : -0.02932758140676075, -8.397137189986026e-05
sigmoid 3 1 0.7 : -0.027370747863417266, -0.0001527685014193203
sigmoid 3 1 0.9 : -0.027425815853109235, -0.0003515768977946365

sigmoid 3 2 0.1 : -0.027851574944213288, -0.0004817589185817095
sigmoid 3 2 0.3 : -0.02803863742455821, -0.00015273486558475824
sigmoid 3 2 0.5 : -0.029327682929321107, -8.393840820786203e-05
sigmoid 3 2 0.7 : -0.027370781055265782, -0.00015273486558431415
sigmoid 3 2 0.9 : -0.0274259392818188, -0.00035164512077212606
sigmoid 3 3 0.1 : -0.02785157494475108, -0.0004817589182650739
sigmoid 3 3 0.3 : -0.028038637425086766, -0.0001527348652718974
sigmoid 3 3 0.5 : -0.029327682929847977, -8.39384078961114e-05
sigmoid 3 3 0.7 : -0.027370781055785543, -0.0001527348652718974
sigmoid 3 3 0.9 : -0.02742593928234638, -0.0003516451204561566
sigmoid 3 4 0.1 : -0.02785157494475108, -0.0004817589182650739
sigmoid 3 4 0.3 : -0.028038637425086766, -0.0001527348652718974
sigmoid 3 4 0.5 : -0.029327682929847977, -8.39384078961114e-05
sigmoid 3 4 0.7 : -0.027370781055785543, -0.0001527348652718974
sigmoid 3 4 0.9 : -0.02742593928234638, -0.0003516451204561566
sigmoid 3 5 0.1 : -0.02785157494475108, -0.0004817589182650739
sigmoid 3 5 0.3 : -0.028038637425086766, -0.0001527348652718974
sigmoid 3 5 0.5 : -0.029327682929847977, -8.39384078961114e-05
sigmoid 3 5 0.7 : -0.027370781055785543, -0.0001527348652718974
sigmoid 3 5 0.9 : -0.02742593928234638, -0.0003516451204561566
sigmoid 3 6 0.1 : -0.02785157494475108, -0.0004817589182650739
sigmoid 3 6 0.3 : -0.028038637425086766, -0.0001527348652718974
sigmoid 3 6 0.5 : -0.029327682929847977, -8.39384078961114e-05
sigmoid 3 6 0.7 : -0.027370781055785543, -0.0001527348652718974
sigmoid 3 6 0.9 : -0.02742593928234638, -0.0003516451204561566
sigmoid 4 1 0.1 : -0.02785198749985649, -0.000481793216322135
sigmoid 4 1 0.3 : -0.028038482181204395, -0.00015277500750854323
sigmoid 4 1 0.5 : -0.0293275475665034, -8.398236039974805e-05
sigmoid 4 1 0.7 : -0.027370736800164018, -0.00015277971396709944
sigmoid 4 1 0.9 : -0.02742577471079626, -0.0003515541606879946
sigmoid 4 2 0.1 : -0.027851574944033876, -0.0004817589186871807
sigmoid 4 2 0.3 : -0.028038637424381906, -0.0001527348656891192
sigmoid 4 2 0.5 : -0.029327682929145428, -8.39384083117789e-05
sigmoid 4 2 0.7 : -0.027370781055092497, -0.00015273486568867511
sigmoid 4 2 0.9 : -0.027425939281642896, -0.0003516451208773752
sigmoid 4 3 0.1 : -0.02785157494475108, -0.0004817589182650739
sigmoid 4 3 0.3 : -0.028038637425086766, -0.0001527348652718974
sigmoid 4 3 0.5 : -0.029327682929847977, -8.39384078961114e-05
sigmoid 4 3 0.7 : -0.027370781055785543, -0.0001527348652718974
sigmoid 4 3 0.9 : -0.02742593928234638, -0.0003516451204561566
sigmoid 4 4 0.1 : -0.02785157494475108, -0.0004817589182650739
sigmoid 4 4 0.3 : -0.028038637425086766, -0.0001527348652718974
sigmoid 4 4 0.5 : -0.029327682929847977, -8.39384078961114e-05
sigmoid 4 4 0.7 : -0.027370781055785543, -0.0001527348652718974
sigmoid 4 4 0.9 : -0.02742593928234638, -0.0003516451204561566
sigmoid 4 5 0.1 : -0.02785157494475108, -0.0004817589182650739
sigmoid 4 5 0.3 : -0.028038637425086766, -0.0001527348652718974
sigmoid 4 5 0.5 : -0.029327682929847977, -8.39384078961114e-05
sigmoid 4 5 0.7 : -0.027370781055785543, -0.0001527348652718974
sigmoid 4 5 0.9 : -0.02742593928234638, -0.0003516451204561566
sigmoid 4 6 0.1 : -0.02785157494475108, -0.0004817589182650739
sigmoid 4 6 0.3 : -0.028038637425086766, -0.0001527348652718974
sigmoid 4 6 0.5 : -0.029327682929847977, -8.39384078961114e-05
sigmoid 4 6 0.7 : -0.027370781055785543, -0.0001527348652718974
sigmoid 4 6 0.9 : -0.02742593928234638, -0.0003516451204561566

Grid search of ElasticNet, Topological
ElasticNet(alpha= i, l1_ratio= j, max_iter=100000)
i j : accuracy of prediction using cross validation, accuracy of prediction using test data
0.001 0.0 : 0.8864180736734684, 0.909905047215466
0.001 0.2 : 0.8858361362759816, 0.9069422475015044
0.001 0.4 : 0.8841598739579117, 0.8952828790344256
0.001 0.6 : 0.8781799596468444, 0.887464545125728
0.001 0.8 : 0.8646519885040638, 0.8817074134995585
0.001 1.0 : 0.8482351792772752, 0.8735246450907566
0.01 0.0 : 0.8856674839436275, 0.912500683812765
0.01 0.2 : 0.8874821727124867, 0.9076722858134403
0.01 0.4 : 0.8818591650265029, 0.9000665805625658
0.01 0.6 : 0.875026287182483, 0.8929334019383773
0.01 0.8 : 0.8662199343689917, 0.8868454087534487
0.01 1.0 : 0.8390104159072662, 0.8956702644815486
0.1 0.0 : 0.8678790453461019, 0.8893993972236347
0.1 0.2 : 0.8566579394405835, 0.8753358988812201
0.1 0.4 : 0.8406940763291543, 0.8605466203605296
0.1 0.6 : 0.8277178984949354, 0.8488348598932843
0.1 0.8 : 0.8151982755763161, 0.8415757012253897
0.1 1.0 : 0.7998712771858066, 0.8331741953840978

Grid search of RandomForest, Topological
RandomForestRegressor(n_estimators= i)
i: accuracy of prediction using cross validation, accuracy of prediction using test data
100 1.0 : 0.7423673322945199, 0.8002304575848476
500 1.0 : 0.7493296457464784, 0.8033162936854047
1000 1.0 : 0.7501060627551379, 0.8016934332262956
2000 1.0 : 0.7509369707487541, 0.8012938736101327

Grid search of NeuralNetwork, Topological
MLPRegressor(activation=act, alpha=a, batch_size=batch, beta_1=0.9,
beta_2=0.999, early_stopping=False, epsilon=1e-08,
hidden_layer_sizes=hid, learning_rate='constant',
learning_rate_init=0.001, max_iter=100000, momentum=0.9,
n_iter_no_change=10, nesterovs_momentum=True, power_t=0.5,
random_state=1, shuffle=True, solver='adam', tol=0.0001,
validation_fraction=0.1, verbose=False, warm_start=False)
act (hid) a batch: accuracy of prediction using cross validation, accuracy of prediction using test data
relu (200,) 0.0001 500 : 0.8726034876039659, 0.9145888279010317
relu (200,) 0.0001 200 : 0.8703269473787791, 0.9154579290296508
relu (200,) 0.0001 100 : 0.8746594515146215, 0.9152900905835845
relu (200,) 0.0001 50 : 0.872532326397273, 0.9183143626274616
relu (200,) 0.001 500 : 0.8726394320460557, 0.9145798905434617
relu (200,) 0.001 200 : 0.8709354576747842, 0.9153546839214142
relu (200,) 0.001 100 : 0.8743472135511696, 0.9154974294596488
relu (200,) 0.001 50 : 0.870245196675554, 0.9225357993952389
relu (200,) 0.01 500 : 0.872468062578584, 0.914605031967196
relu (200,) 0.01 200 : 0.8703758492389193, 0.915617885060796
relu (200,) 0.01 100 : 0.8748509786967358, 0.9148941467483017
relu (200,) 0.01 50 : 0.8725111302248967, 0.9216052358208284
relu (200, 200) 0.0001 500 : 0.8625119618078918, 0.9156092800227197
relu (200, 200) 0.0001 200 : 0.8636899893245402, 0.9180678787849503
relu (200, 200) 0.0001 100 : 0.8599558654071068, 0.9112190717070936
relu (200, 200) 0.0001 50 : 0.8619008453506105, 0.9191963923292499
relu (200, 200) 0.001 500 : 0.861977421623329, 0.9183336744600199
relu (200, 200) 0.001 200 : 0.8616204220963773, 0.9151298611702873
relu (200, 200) 0.001 100 : 0.8572026801943651, 0.9168498332525032
relu (200, 200) 0.001 50 : 0.8617004843697794, 0.9084016677998352
relu (200, 200) 0.01 500 : 0.8605787627427839, 0.9151889597468041
relu (200, 200) 0.01 200 : 0.8590131239709061, 0.9117748059702003
relu (200, 200) 0.01 100 : 0.8585687924437575, 0.9160708065934653
relu (200, 200) 0.01 50 : 0.8562054181176023, 0.9152285200746686
relu (100,) 0.0001 500 : 0.8673916672646769, 0.9163341089479283
relu (100,) 0.0001 200 : 0.8680904291661662, 0.9188412371124469
relu (100,) 0.0001 100 : 0.8714264656077912, 0.9196380253301215
relu (100,) 0.0001 50 : 0.8678125343263929, 0.918965947333476
relu (100,) 0.001 500 : 0.8677582338436934, 0.9160352628007908
relu (100,) 0.001 200 : 0.8713275405035056, 0.9194588306687176
relu (100,) 0.001 100 : 0.8708719136080644, 0.9197072446586527
relu (100,) 0.001 50 : 0.8678197375356966, 0.9166963505473033
relu (100,) 0.01 500 : 0.8687339108792728, 0.9163697374084036
relu (100,) 0.01 200 : 0.8706621611157352, 0.9194626150745031

relu (100,) 0.01 100 : 0.8725447862498878, 0.9193901166627877
relu (100,) 0.01 50 : 0.8697949865565555, 0.921296080753514
relu (100, 100) 0.0001 500 : 0.8567993352457426, 0.910797712183666
relu (100, 100) 0.0001 200 : 0.864266788422524, 0.9112892653748179
relu (100, 100) 0.0001 100 : 0.867184504529685, 0.9143524902911051
relu (100, 100) 0.0001 50 : 0.861521938004113, 0.9137741487663074
relu (100, 100) 0.001 500 : 0.8588072696958523, 0.9126324788319882
relu (100, 100) 0.001 200 : 0.863473193778642, 0.9115246528916228
relu (100, 100) 0.001 100 : 0.8664484513188112, 0.9118772676719497
relu (100, 100) 0.001 50 : 0.8640603841950554, 0.9124595594795901
relu (100, 100) 0.01 500 : 0.8549995250921242, 0.9110513599717993
relu (100, 100) 0.01 200 : 0.8629313635753896, 0.9077537575165929
relu (100, 100) 0.01 100 : 0.8666385190228624, 0.9145361092406297
relu (100, 100) 0.01 50 : 0.8658688019526162, 0.9144116090437586
relu (100, 200) 0.0001 500 : 0.8654747180424074, 0.9172315890833037
relu (100, 200) 0.0001 200 : 0.8668211824398258, 0.9149252453585713
relu (100, 200) 0.0001 100 : 0.8733623512429134, 0.9145552960677099
relu (100, 200) 0.0001 50 : 0.8645535661816975, 0.9170714663163745
relu (100, 200) 0.001 500 : 0.8701126546624316, 0.9187897301671348
relu (100, 200) 0.001 200 : 0.8681163392184313, 0.9137718075870329
relu (100, 200) 0.001 100 : 0.8674091200728873, 0.8962658828423798
relu (100, 200) 0.001 50 : 0.8655402476376197, 0.9196430474084506
relu (100, 200) 0.01 500 : 0.8675390401751815, 0.9162122278450533
relu (100, 200) 0.01 200 : 0.8710523551117794, 0.9114286043882497
relu (100, 200) 0.01 100 : 0.8687092729661214, 0.9157528058839
relu (100, 200) 0.01 50 : 0.8641001632318531, 0.9115882808756828
relu (50,) 0.0001 500 : 0.8713116147147234, 0.9143104660625523
relu (50,) 0.0001 200 : 0.8720353495048977, 0.9098140684196897
relu (50,) 0.0001 100 : 0.8756423312900605, 0.9128821231907378
relu (50,) 0.0001 50 : 0.872843643101092, 0.9120350697481266
relu (50,) 0.001 500 : 0.8711786605609705, 0.9144570882141657
relu (50,) 0.001 200 : 0.871998246111111, 0.9098360052164254
relu (50,) 0.001 100 : 0.875141223909085, 0.9128726699515769
relu (50,) 0.001 50 : 0.8731433233158039, 0.9123077528991463
relu (50,) 0.01 500 : 0.8715769829355786, 0.9141123295194744
relu (50,) 0.01 200 : 0.8720200985905379, 0.9098846350210071
relu (50,) 0.01 100 : 0.8753456018022145, 0.9129284142416473
relu (50,) 0.01 50 : 0.873431809149378, 0.9116858585684646
relu (50, 50) 0.0001 500 : 0.8705171991323146, 0.9148162314938648
relu (50, 50) 0.0001 200 : 0.8627970133500641, 0.9079121863804062
relu (50, 50) 0.0001 100 : 0.871591937599737, 0.9065504962866349
relu (50, 50) 0.0001 50 : 0.8740925685263361, 0.9123152454809138
relu (50, 50) 0.001 500 : 0.8708484095650085, 0.9147460681045273
relu (50, 50) 0.001 200 : 0.8643807502953317, 0.9069815970884978
relu (50, 50) 0.001 100 : 0.8696586163619221, 0.9055078861978068
relu (50, 50) 0.001 50 : 0.8726592524041136, 0.9193546964195852
relu (50, 50) 0.01 500 : 0.8699146537923161, 0.9148287386694933
relu (50, 50) 0.01 200 : 0.8678191676358674, 0.9087269843455905
relu (50, 50) 0.01 100 : 0.8725759433992876, 0.9105134691575094
relu (50, 50) 0.01 50 : 0.8702000655272111, 0.920336233677304
relu (50, 100) 0.0001 500 : 0.8730278103673687, 0.913536688966746
relu (50, 100) 0.0001 200 : 0.8658858058200668, 0.9124546288308868
relu (50, 100) 0.0001 100 : 0.8631087704062814, 0.9084088463062584
relu (50, 100) 0.0001 50 : 0.8662874680701214, 0.9191293139376582
relu (50, 100) 0.001 500 : 0.8718738353537884, 0.9160497811830806
relu (50, 100) 0.001 200 : 0.8702552018410407, 0.9130618872576454
relu (50, 100) 0.001 100 : 0.8597050172474845, 0.9040122840427826
relu (50, 100) 0.001 50 : 0.8664114964673759, 0.9145080122985262
relu (50, 100) 0.01 500 : 0.8715514952712251, 0.9146526615261562
relu (50, 100) 0.01 200 : 0.8689896882920186, 0.9155590087025895
relu (50, 100) 0.01 100 : 0.8643140898277124, 0.9097565882633126
relu (50, 100) 0.01 50 : 0.8623155802446847, 0.921282384953836
relu (50, 200) 0.0001 500 : 0.8639326305650548, 0.9112695269379887
relu (50, 200) 0.0001 200 : 0.8716833484358995, 0.9102699103432347
relu (50, 200) 0.0001 100 : 0.8683267639180017, 0.9093026619447601
relu (50, 200) 0.0001 50 : 0.8635613668306645, 0.911927899371355
relu (50, 200) 0.001 500 : 0.8647100423119968, 0.9111450711325697
relu (50, 200) 0.001 200 : 0.8667261173570553, 0.9101722154637968
relu (50, 200) 0.001 100 : 0.8563929071819203, 0.919643902274951
relu (50, 200) 0.001 50 : 0.8589221140815319, 0.9139304560083574
relu (50, 200) 0.01 500 : 0.8657394527344568, 0.9186982874817994
relu (50, 200) 0.01 200 : 0.8710357116748998, 0.9109599302866742
relu (50, 200) 0.01 100 : 0.8665948861832267, 0.9172875867150161
relu (50, 200) 0.01 50 : 0.8621150667999148, 0.9130552068003336
tanh (200,) 0.0001 500 : 0.8290808670896229, 0.8791614536277325
tanh (200,) 0.0001 200 : 0.8186192547728947, 0.880239150658239
tanh (200,) 0.0001 100 : 0.819132672619763, 0.880306018763133
tanh (200,) 0.0001 50 : 0.809159610985696, 0.8606861771154845
tanh (200,) 0.001 500 : 0.8220767872746297, 0.8762138043090126
tanh (200,) 0.001 200 : 0.8102347971048578, 0.8754537939670473
tanh (200,) 0.001 100 : 0.8078453925652962, 0.877307962845493
tanh (200,) 0.001 50 : 0.8078676442375933, 0.8671913965996421
tanh (200,) 0.01 500 : 0.8158602859175422, 0.8758972743443906
tanh (200,) 0.01 200 : 0.805783307295689, 0.8878526754258541
tanh (200,) 0.01 100 : 0.8120160272749185, 0.8770110809572612
tanh (200,) 0.01 50 : 0.8109573698033149, 0.863492693371961
tanh (200, 200) 0.0001 500 : -0.00879881367411035, -0.022780517724600102
tanh (200, 200) 0.0001 200 : -0.008845218953796285, 0.8702277113795998
tanh (200, 200) 0.0001 100 : 0.3139790110426469, 0.8745860212675596
tanh (200, 200) 0.0001 50 : 0.8232957174666625, 0.8549287780984408
tanh (200, 200) 0.001 500 : -0.008798825557613776, -0.022780591760320545
tanh (200, 200) 0.001 200 : -0.008845240644439345, 0.8847608482309105
tanh (200, 200) 0.001 100 : 0.16000782462532945, 0.8877525125938835
tanh (200, 200) 0.001 50 : 0.8273880964902454, 0.8656494147595186
tanh (200, 200) 0.01 500 : -0.008798936531888835, -0.02278133162294038
tanh (200, 200) 0.01 200 : -0.008845811769027812, 0.8617773625637988
tanh (200, 200) 0.01 100 : 0.1610351135524873, 0.8847833747679076
tanh (200, 200) 0.01 50 : 0.8300527211860201, 0.8798523982191235
tanh (100,) 0.0001 500 : 0.8104123434556059, 0.867846128222691
tanh (100,) 0.0001 200 : 0.6354142090087417, 0.865613861838583
tanh (100,) 0.0001 100 : 0.8146714598280976, 0.8665107254745534
tanh (100,) 0.0001 50 : 0.7949195269086373, 0.8800085431155306

tanh (100,) 0.001 500 : 0.815446563934443, 0.8665800208013091
tanh (100,) 0.001 200 : 0.787241517374478, 0.8591878480226195
tanh (100,) 0.001 100 : 0.8053771784932016, 0.8485389133634581
tanh (100,) 0.001 50 : 0.7959600446131291, 0.8780039126861184
tanh (100,) 0.01 500 : 0.809481984300756, 0.8623807698998338
tanh (100,) 0.01 200 : 0.8083614943804763, 0.8606588106473869
tanh (100,) 0.01 100 : 0.7993511379870712, 0.8554068160267255
tanh (100,) 0.01 50 : 0.8054867084026587, 0.8776694472588931
tanh (100, 100) 0.0001 500 : 0.1551876175602058, -0.018038414198181973
tanh (100, 100) 0.0001 200 : -0.009635936332223417, -0.017282719780136624
tanh (100, 100) 0.0001 100 : 0.653676389692758, -0.017864480485773493
tanh (100, 100) 0.0001 50 : 0.7800148627455377, 0.8430087508949113
tanh (100, 100) 0.001 500 : 0.16420649910886834, -0.01803854524800319
tanh (100, 100) 0.001 200 : -0.009636212809087752, -0.017282850089730184
tanh (100, 100) 0.001 100 : 0.6418553072435659, -0.017864581998757734
tanh (100, 100) 0.001 50 : 0.7905094976890557, 0.839793811854634
tanh (100, 100) 0.01 500 : 0.1571548344520382, -0.018039791675798167
tanh (100, 100) 0.01 200 : -0.00963832269431939, -0.01728383125704913
tanh (100, 100) 0.01 100 : 0.6477973952425551, -0.017865650353104723
tanh (100, 100) 0.01 50 : 0.8161563778499954, 0.8702303447873688
tanh (100, 200) 0.0001 500 : 0.44739295189902595, -0.017998205754724994
tanh (100, 200) 0.0001 200 : 0.6156930963746986, 0.8291314762497457
tanh (100, 200) 0.0001 100 : 0.3273618875973202, 0.8361471999942821
tanh (100, 200) 0.0001 50 : 0.6310776119073432, 0.8559201919035686
tanh (100, 200) 0.001 500 : 0.4555190402376679, -0.01799829336565706
tanh (100, 200) 0.001 200 : 0.6234277927334384, 0.8515418232253672
tanh (100, 200) 0.001 100 : 0.3215812720033794, 0.8592438715740447
tanh (100, 200) 0.001 50 : 0.6447672660863579, 0.8452165494258226
tanh (100, 200) 0.01 500 : 0.45393380758457935, -0.017999174863491563
tanh (100, 200) 0.01 200 : 0.6333878084227738, 0.8804585934151236
tanh (100, 200) 0.01 100 : 0.32711986937326293, 0.8536144880011164
tanh (100, 200) 0.01 50 : 0.6433079890941782, 0.8595469704671594
tanh (50,) 0.0001 500 : 0.7943675509871764, 0.8359306042280636
tanh (50,) 0.0001 200 : 0.7629623960054378, 0.866344210557864
tanh (50,) 0.0001 100 : 0.787641986367403, 0.8474146063103734
tanh (50,) 0.0001 50 : 0.7973300273202799, 0.8584809791332131
tanh (50,) 0.001 500 : 0.7880397533183456, 0.853988996637942
tanh (50,) 0.001 200 : 0.7875729914857729, 0.853686587623924
tanh (50,) 0.001 100 : 0.7973501284995751, 0.8531777901026298
tanh (50,) 0.001 50 : 0.790332478469455, 0.8510521858470022
tanh (50,) 0.01 500 : 0.7611011136325672, 0.8456493020369055
tanh (50,) 0.01 200 : 0.7959917240045352, 0.8636399487328386
tanh (50,) 0.01 100 : 0.823852667369465, 0.8451722205963025
tanh (50,) 0.01 50 : 0.7914723746834362, 0.8472259955026922
tanh (50, 50) 0.0001 500 : -0.008817726619046118, -0.01896086357992699
tanh (50, 50) 0.0001 200 : -0.00895881550886637, -0.018069894727086533
tanh (50, 50) 0.0001 100 : 0.30802065996315964, -0.016682673857985497
tanh (50, 50) 0.0001 50 : 0.5963546566836491, -0.01780178147386202
tanh (50, 50) 0.001 500 : -0.008817759575513983, -0.018961067652341512
tanh (50, 50) 0.001 200 : -0.008958892179045597, -0.018069973749101242
tanh (50, 50) 0.001 100 : 0.30971350941315773, -0.016682844131253427
tanh (50, 50) 0.001 50 : 0.6032059937885117, -0.017802059188406272
tanh (50, 50) 0.01 500 : -0.008818358350334909, -0.018963106400444518
tanh (50, 50) 0.01 200 : -0.008959591492108699, -0.018070763423732483
tanh (50, 50) 0.01 100 : -0.008299330665948767, -0.01668453663159064
tanh (50, 50) 0.01 50 : 0.3214733893100889, -0.017804862214578332
tanh (50, 100) 0.0001 500 : 0.31218219574667117, -0.02047562523825852
tanh (50, 100) 0.0001 200 : 0.45724934683025875, 0.853946914869076
tanh (50, 100) 0.0001 100 : 0.609603925831214, 0.8411867114859585
tanh (50, 100) 0.0001 50 : 0.619212506827917, 0.8363476213866462
tanh (50, 100) 0.001 500 : 0.31945583385442744, -0.020475759984181607
tanh (50, 100) 0.001 200 : 0.46424555876512075, 0.8589066558733689
tanh (50, 100) 0.001 100 : 0.6103545182318071, 0.8346301343031673
tanh (50, 100) 0.001 50 : 0.6067993147321908, 0.8380035057166875
tanh (50, 100) 0.01 500 : 0.31338329626910405, -0.02047711974797184
tanh (50, 100) 0.01 200 : 0.45443073393788136, 0.7820725163403407
tanh (50, 100) 0.01 100 : 0.60207525749934, 0.8507304099995017
tanh (50, 100) 0.01 50 : 0.6150905124838182, 0.8567958587066486
tanh (50, 200) 0.0001 500 : -0.008888320926123017, -0.016139055703394467
tanh (50, 200) 0.0001 200 : -0.009334436171438521, -0.016383405300696596
tanh (50, 200) 0.0001 100 : 0.16001732298204105, 0.8497624460618576
tanh (50, 200) 0.0001 50 : 0.15204723081015598, -0.016172023396866075
tanh (50, 200) 0.001 500 : -0.00888833639652189, -0.016139150386045076
tanh (50, 200) 0.001 200 : -0.009334584958297754, -0.016383438273094297
tanh (50, 200) 0.001 100 : 0.1521523547936814, 0.8438690278518474
tanh (50, 200) 0.001 50 : 0.15997474167889236, -0.016177961073838176
tanh (50, 200) 0.01 500 : -0.008888489866334748, -0.016140097670606535
tanh (50, 200) 0.01 200 : -0.00933559137791371, -0.01638377275266234
tanh (50, 200) 0.01 100 : 0.15554253108223443, -0.017678293308157844
tanh (50, 200) 0.01 50 : 0.13951088923090005, -0.016196861662051143
logistic (200,) 0.0001 500 : 0.8284524812340862, 0.8808725532519792
logistic (200,) 0.0001 200 : 0.8312124420695707, 0.8658864953722559
logistic (200,) 0.0001 100 : 0.8281837323866922, 0.8845719637332835
logistic (200,) 0.0001 50 : 0.8322925537009123, 0.8842915607899067
logistic (200,) 0.001 500 : 0.8290976517983552, 0.8776755149835048
logistic (200,) 0.001 200 : 0.8316760799209441, 0.8698517483443234
logistic (200,) 0.001 100 : 0.831482191616318, 0.8829197737658209
logistic (200,) 0.001 50 : 0.8242429217433738, 0.8831235654842428
logistic (200,) 0.01 500 : 0.8204256843601023, 0.8812570168957415

logistic (200,) 0.01 200 : 0.835378747457616, 0.8792129534734976
logistic (200,) 0.01 100 : 0.8372369286808985, 0.8862386156172739
logistic (200,) 0.01 50 : 0.8409906303368461, 0.8909303664386573
logistic (200, 200) 0.0001 500 : 0.8554363372015941, 0.871431092274723
logistic (200, 200) 0.0001 200 : 0.8472840267729852, 0.8682107087666648
logistic (200, 200) 0.0001 100 : 0.8510436039026773, 0.8820175707132997
logistic (200, 200) 0.0001 50 : 0.8458048458154256, 0.8972947752028444
logistic (200, 200) 0.001 500 : 0.8472402551481174, 0.8868107493370602
logistic (200, 200) 0.001 200 : 0.8457293466532085, 0.8666827682801006
logistic (200, 200) 0.001 100 : 0.8496286239186495, 0.8710439424393204
logistic (200, 200) 0.001 50 : 0.8294735048379355, 0.896940180682958
logistic (200, 200) 0.01 500 : 0.8425959164344876, 0.8831568525377003
logistic (200, 200) 0.01 200 : 0.8399538665950546, 0.8688263509162383
logistic (200, 200) 0.01 100 : 0.8359082647830368, 0.8950109271994338
logistic (200, 200) 0.01 50 : 0.8402720220960365, 0.8978121496514035
logistic (100,) 0.0001 500 : 0.8292190993137716, 0.8761829250067182
logistic (100,) 0.0001 200 : 0.8279719692364251, 0.8635977294123443
logistic (100,) 0.0001 100 : 0.8321450322635666, 0.8740397657761201
logistic (100,) 0.0001 50 : 0.8289033349650425, 0.8886073903546642
logistic (100,) 0.001 500 : 0.8161941382585118, 0.8726052031227776
logistic (100,) 0.001 200 : 0.834068965823251, 0.8636951288321777
logistic (100,) 0.001 100 : 0.8328130269950795, 0.8801896780294479
logistic (100,) 0.001 50 : 0.8268762669197327, 0.8865852424369918
logistic (100,) 0.01 500 : 0.818101334193711, 0.8767348685006311
logistic (100,) 0.01 200 : 0.8314968477818651, 0.8734613641129416
logistic (100,) 0.01 100 : 0.8360899073315338, 0.8819227708314326
logistic (100,) 0.01 50 : 0.8381687227568942, 0.8952307965927072
logistic (100, 100) 0.0001 500 : 0.6775200895643071, -0.017877472980130538
logistic (100, 100) 0.0001 200 : 0.6771017106719848, 0.863407300206927
logistic (100, 100) 0.0001 100 : 0.8333977952648592, 0.8705038851864453
logistic (100, 100) 0.0001 50 : 0.832789678208876, 0.8765778522690021
logistic (100, 100) 0.001 500 : 0.6763522772290683, -0.01787656066536636
logistic (100, 100) 0.001 200 : 0.6724064730904372, 0.8583913033327226
logistic (100, 100) 0.001 100 : 0.8237361961293402, 0.8922370790332809
logistic (100, 100) 0.001 50 : 0.8471839552032051, 0.876657992846569
logistic (100, 100) 0.01 500 : 0.6613312053351942, -0.017489569713940112
logistic (100, 100) 0.01 200 : 0.6716872744153948, 0.8485929943243358
logistic (100, 100) 0.01 100 : 0.8280410283416509, 0.8699359131979336
logistic (100, 100) 0.01 50 : 0.8320426926689629, 0.8996031107236987
logistic (100, 200) 0.0001 500 : 0.8348058993049197, 0.8555381364468968
logistic (100, 200) 0.0001 200 : 0.8277008249902668, 0.8482268587299093
logistic (100, 200) 0.0001 100 : 0.8187118498955165, 0.870134494067702
logistic (100, 200) 0.0001 50 : 0.8403030653054813, 0.8537008703289845
logistic (100, 200) 0.001 500 : 0.818000449544621, 0.8538438958876526
logistic (100, 200) 0.001 200 : 0.8230959319978224, 0.8577363812609001
logistic (100, 200) 0.001 100 : 0.8235443615161728, 0.8709806194653785
logistic (100, 200) 0.001 50 : 0.8164630593844409, 0.8791507177070924
logistic (100, 200) 0.01 500 : 0.8204299032590894, 0.8748052206095838
logistic (100, 200) 0.01 200 : 0.8249795748777867, 0.854098906899831
logistic (100, 200) 0.01 100 : 0.8230262190854521, 0.8645600430057441
logistic (100, 200) 0.01 50 : 0.8296513780928713, 0.8818719965101681
logistic (50,) 0.0001 500 : 0.8199634021196026, 0.8770206450842646
logistic (50,) 0.0001 200 : 0.8182727484074261, 0.8755181641197557
logistic (50,) 0.0001 100 : 0.8158517176826251, 0.8791451342698309
logistic (50,) 0.0001 50 : 0.8202442376424471, 0.8845948010304815
logistic (50,) 0.001 500 : 0.7971702677119085, 0.8793583239389338
logistic (50,) 0.001 200 : 0.8211244944448269, 0.8743683294475784
logistic (50,) 0.001 100 : 0.8171803765854684, 0.8800275266916919
logistic (50,) 0.001 50 : 0.8293691251717729, 0.8778553807317074
logistic (50,) 0.01 500 : 0.8061499080573153, 0.8816509248408168
logistic (50,) 0.01 200 : 0.8134245744756573, 0.8850017873132193
logistic (50,) 0.01 100 : 0.8172348385157843, 0.8776273245800338
logistic (50,) 0.01 50 : 0.8193681079601864, 0.8899162131648167
logistic (50, 50) 0.0001 500 : 0.7867073544098689, 0.837080638481388
logistic (50, 50) 0.0001 200 : 0.8086935301077711, 0.8485753623464298
logistic (50, 50) 0.0001 100 : 0.8060488423179718, 0.8342031231722109
logistic (50, 50) 0.0001 50 : 0.8212956841523044, 0.8768008459017455
logistic (50, 50) 0.001 500 : 0.7855832008642943, 0.8538997453955715
logistic (50, 50) 0.001 200 : 0.8109510290967548, 0.8390528409418019
logistic (50, 50) 0.001 100 : 0.8135608211192455, 0.8557897290259971
logistic (50, 50) 0.001 50 : 0.8073884979425475, 0.8642806867282855
logistic (50, 50) 0.01 500 : 0.7848735472920154, 0.8515036819168746
logistic (50, 50) 0.01 200 : 0.7902557215358589, 0.8224679465055627
logistic (50, 50) 0.01 100 : 0.8068455175944486, 0.8467354958916606
logistic (50, 50) 0.01 50 : 0.7999908546210721, 0.8669639890174548
logistic (50, 100) 0.0001 500 : 0.82308579713191, 0.8704379943079388
logistic (50, 100) 0.0001 200 : 0.8269209946015608, 0.8235773990356696
logistic (50, 100) 0.0001 100 : 0.8414102798112463, 0.8619898910851037
logistic (50, 100) 0.0001 50 : 0.8128229398102806, 0.8607307566725739
logistic (50, 100) 0.001 500 : 0.7970580940175143, 0.8757187200320586
logistic (50, 100) 0.001 200 : 0.8189513270500501, 0.835268727197883
logistic (50, 100) 0.001 100 : 0.8199856454029473, 0.8620371403466964
logistic (50, 100) 0.001 50 : 0.8345279029486823, 0.881070623861514

logistic (50, 100) 0.01 500 : 0.7982187019211434, 0.8728909811653064
logistic (50, 100) 0.01 200 : 0.8016196943471631, 0.8467266289148393
logistic (50, 100) 0.01 100 : 0.8217941110593611, 0.8639959242704924
logistic (50, 100) 0.01 50 : 0.8214213489163017, 0.8643268561438404
logistic (50, 200) 0.0001 500 : 0.7758064449168612, 0.8646413948459948
logistic (50, 200) 0.0001 200 : 0.8027275934671116, 0.8550131286006858
logistic (50, 200) 0.0001 100 : 0.803646898382391, 0.8612514782128178
logistic (50, 200) 0.0001 50 : 0.8102075490383427, 0.8775425444010914
logistic (50, 200) 0.001 500 : 0.7797684477826351, 0.8453937004841537
logistic (50, 200) 0.001 200 : 0.7979989261695695, 0.8546869150118601
logistic (50, 200) 0.001 100 : 0.7934043105956364, 0.8525947841046648
logistic (50, 200) 0.001 50 : 0.8034838642717042, 0.8767852785619462
logistic (50, 200) 0.01 500 : 0.7684194066477021, 0.868274741203244
logistic (50, 200) 0.01 200 : 0.7879074277138645, 0.8371626720044675
logistic (50, 200) 0.01 100 : 0.8051207745752487, 0.8666019508259182
logistic (50, 200) 0.01 50 : 0.799556926461763, 0.87227085767999

Grid search of LightGBM, Topological
lgb.LGBMRegressor(boosting_type = "gbdt", num_leaves = j,max_depth = 0)
j: accuracy of prediction using cross validation, accuracy of prediction using test data
10 : 0.8059561724825205, 0.8450069564248711
50 : 0.797853921250244, 0.8633168549178787
100 : 0.797853921250244, 0.8633168549178787
150 : 0.797853921250244, 0.8633168549178787

Grid search of SVR, Topological
SVR(C= c_num, kernel = ker, epsilon = e, gamma = r,degree = 3, coef0=1)
Ker c_num r e: accuracy of prediction using cross validation, accuracy of prediction using test data
linear 1 1 0.1 : 0.8865072594279644, 0.9144413967636131
linear 1 1 0.3 : 0.886190207251713, 0.9174815124873049
linear 1 1 0.5 : 0.8853720307412317, 0.9171055048596317
linear 1 1 0.7 : 0.8842245858400464, 0.9158417006798951
linear 1 1 0.9 : 0.8826681819777049, 0.913511629251007
linear 1 2 0.1 : 0.8865072594279644, 0.9144413967636131
linear 1 2 0.3 : 0.886190207251713, 0.9174815124873049
linear 1 2 0.5 : 0.8853720307412317, 0.9171055048596317
linear 1 2 0.7 : 0.8842245858400464, 0.9158417006798951
linear 1 2 0.9 : 0.8826681819777049, 0.913511629251007
linear 1 3 0.1 : 0.8865072594279644, 0.9144413967636131
linear 1 3 0.3 : 0.886190207251713, 0.9174815124873049
linear 1 3 0.5 : 0.8853720307412317, 0.9171055048596317
linear 1 3 0.7 : 0.8842245858400464, 0.9158417006798951
linear 1 3 0.9 : 0.8826681819777049, 0.913511629251007
linear 1 4 0.1 : 0.8865072594279644, 0.9144413967636131
linear 1 4 0.3 : 0.886190207251713, 0.9174815124873049
linear 1 4 0.5 : 0.8853720307412317, 0.9171055048596317
linear 1 4 0.7 : 0.8842245858400464, 0.9158417006798951
linear 1 4 0.9 : 0.8826681819777049, 0.913511629251007
linear 1 5 0.1 : 0.8865072594279644, 0.9144413967636131
linear 1 5 0.3 : 0.886190207251713, 0.9174815124873049
linear 1 5 0.5 : 0.8853720307412317, 0.9171055048596317
linear 1 5 0.7 : 0.8842245858400464, 0.9158417006798951
linear 1 5 0.9 : 0.8826681819777049, 0.913511629251007
linear 1 6 0.1 : 0.8865072594279644, 0.9144413967636131
linear 1 6 0.3 : 0.886190207251713, 0.9174815124873049
linear 1 6 0.5 : 0.8853720307412317, 0.9171055048596317
linear 1 6 0.7 : 0.8842245858400464, 0.9158417006798951
linear 1 6 0.9 : 0.8826681819777049, 0.913511629251007
linear 2 1 0.1 : 0.8857385552414794, 0.9066768559090652
linear 2 1 0.3 : 0.8854588413581755, 0.9125736553005868
linear 2 1 0.5 : 0.884748350529837, 0.9128107726319626
linear 2 1 0.7 : 0.8837248167893875, 0.9119018808806217
linear 2 1 0.9 : 0.8823409181260937, 0.9101286480569222
linear 2 2 0.1 : 0.8857385552414794, 0.9066768559090652
linear 2 2 0.3 : 0.8854588413581755, 0.9125736553005868
linear 2 2 0.5 : 0.884748350529837, 0.9128107726319626
linear 2 2 0.7 : 0.8837248167893875, 0.9119018808806217
linear 2 2 0.9 : 0.8823409181260937, 0.9101286480569222
linear 2 3 0.1 : 0.8857385552414794, 0.9066768559090652
linear 2 3 0.3 : 0.8854588413581755, 0.9125736553005868
linear 2 3 0.5 : 0.884748350529837, 0.9128107726319626
linear 2 3 0.7 : 0.8837248167893875, 0.9119018808806217
linear 2 3 0.9 : 0.8823409181260937, 0.9101286480569222
linear 2 4 0.1 : 0.8857385552414794, 0.9066768559090652
linear 2 4 0.3 : 0.8854588413581755, 0.9125736553005868
linear 2 4 0.5 : 0.884748350529837, 0.9128107726319626
linear 2 4 0.7 : 0.8837248167893875, 0.9119018808806217
linear 2 4 0.9 : 0.8823409181260937, 0.9101286480569222
linear 2 5 0.1 : 0.8857385552414794, 0.9066768559090652
linear 2 5 0.3 : 0.8854588413581755, 0.9125736553005868
linear 2 5 0.5 : 0.884748350529837, 0.9128107726319626
linear 2 5 0.7 : 0.8837248167893875, 0.9119018808806217
linear 2 5 0.9 : 0.8823409181260937, 0.9101286480569222
linear 2 6 0.1 : 0.8857385552414794, 0.9066768559090652
linear 2 6 0.3 : 0.8854588413581755, 0.9125736553005868
linear 2 6 0.5 : 0.884748350529837, 0.9128107726319626
linear 2 6 0.7 : 0.8837248167893875, 0.9119018808806217
linear 2 6 0.9 : 0.8823409181260937, 0.9101286480569222
linear 3 1 0.1 : 0.8857385665549675, 0.906677547253373
linear 3 1 0.3 : 0.8854589227545459, 0.9125747643994964
linear 3 1 0.5 : 0.8847483505298323, 0.9128118426831252
linear 3 1 0.7 : 0.8837251322681091, 0.9119018808806238
linear 3 1 0.9 : 0.8823409181260937, 0.9101286480569222
linear 3 2 0.1 : 0.8857385665549675, 0.906677547253373
linear 3 2 0.3 : 0.8854589227545459, 0.9125747643994964
linear 3 2 0.5 : 0.8847483505298323, 0.9128118426831252
linear 3 2 0.7 : 0.8837251322681091, 0.9119018808806238
linear 3 2 0.9 : 0.8823409181260937, 0.9101286480569222
linear 3 3 0.1 : 0.8857385665549675, 0.906677547253373
linear 3 3 0.3 : 0.8854589227545459, 0.9125747643994964
linear 3 3 0.5 : 0.8847483505298323, 0.9128118426831252
linear 3 3 0.7 : 0.8837251322681091, 0.9119018808806238
linear 3 3 0.9 : 0.8823409181260937, 0.9101286480569222
linear 3 4 0.1 : 0.8857385665549675, 0.906677547253373
linear 3 4 0.3 : 0.8854589227545459, 0.9125747643994964
linear 3 4 0.5 : 0.8847483505298323, 0.9128118426831252
linear 3 4 0.7 : 0.8837251322681091, 0.9119018808806238
linear 3 4 0.9 : 0.8823409181260937, 0.9101286480569222
linear 3 5 0.1 : 0.8857385665549675, 0.906677547253373
linear 3 5 0.3 : 0.8854589227545459, 0.9125747643994964
linear 3 5 0.5 : 0.8847483505298323, 0.9128118426831252
linear 3 5 0.7 : 0.8837251322681091, 0.9119018808806238
linear 3 5 0.9 : 0.8823409181260937, 0.9101286480569222
linear 3 6 0.1 : 0.8857385665549675, 0.906677547253373
linear 3 6 0.3 : 0.8854589227545459, 0.9125747643994964
linear 3 6 0.5 : 0.8847483505298323, 0.9128118426831252
linear 3 6 0.7 : 0.8837251322681091, 0.9119018808806238
linear 3 6 0.9 : 0.8823409181260937, 0.9101286480569222
linear 4 1 0.1 : 0.885738503034972, 0.9066755205649947
linear 4 1 0.3 : 0.8854589227545466, 0.9125742831735577
linear 4 1 0.5 : 0.8847483505298385, 0.9128118426831285
linear 4 1 0.7 : 0.8837251322681082, 0.9119018808806154
linear 4 1 0.9 : 0.8823409181260937, 0.9101286480569222
linear 4 2 0.1 : 0.885738503034972, 0.9066755205649947
linear 4 2 0.3 : 0.8854589227545466, 0.9125742831735577
linear 4 2 0.5 : 0.8847483505298385, 0.9128118426831285
linear 4 2 0.7 : 0.8837251322681082, 0.9119018808806154
linear 4 2 0.9 : 0.8823409181260937, 0.9101286480569222

linear 4 3 0.1 : 0.885738503034972, 0.9066755205649947
linear 4 3 0.3 : 0.8854589227545466, 0.9125742831735577
linear 4 3 0.5 : 0.8847483505298385, 0.9128118426831285
linear 4 3 0.7 : 0.8837251322681082, 0.9119018808806154
linear 4 3 0.9 : 0.8823409181260937, 0.9101286480569222
linear 4 4 0.1 : 0.885738503034972, 0.9066755205649947
linear 4 4 0.3 : 0.8854589227545466, 0.9125742831735577
linear 4 4 0.5 : 0.8847483505298385, 0.9128118426831285
linear 4 4 0.7 : 0.8837251322681082, 0.9119018808806154
linear 4 4 0.9 : 0.8823409181260937, 0.9101286480569222
linear 4 5 0.1 : 0.885738503034972, 0.9066755205649947
linear 4 5 0.3 : 0.8854589227545466, 0.9125742831735577
linear 4 5 0.5 : 0.8847483505298385, 0.9128118426831285
linear 4 5 0.7 : 0.8837251322681082, 0.9119018808806154
linear 4 5 0.9 : 0.8823409181260937, 0.9101286480569222
linear 4 6 0.1 : 0.885738503034972, 0.9066755205649947
linear 4 6 0.3 : 0.8854589227545466, 0.9125742831735577
linear 4 6 0.5 : 0.8847483505298385, 0.9128118426831285
linear 4 6 0.7 : 0.8837251322681082, 0.9119018808806154
linear 4 6 0.9 : 0.8823409181260937, 0.9101286480569222
poly 1 1 0.1 : 0.8018964668450941, 0.8474884431339463
poly 1 1 0.3 : 0.8005456697789721, 0.8458785390850958
poly 1 1 0.5 : 0.7987993230938064, 0.8440708117311828
poly 1 1 0.7 : 0.7968954260963702, 0.8420453193557504
poly 1 1 0.9 : 0.7948874697443825, 0.8397927219548363
poly 1 2 0.1 : 0.8017196347061253, 0.8473013017783454
poly 1 2 0.3 : 0.8003683029396104, 0.8456885970668316
poly 1 2 0.5 : 0.7986246458327327, 0.8438800659779631
poly 1 2 0.7 : 0.7967214727577193, 0.8418534360706731
poly 1 2 0.9 : 0.7947156643287591, 0.8396034886766953
poly 1 3 0.1 : 0.801660518445124, 0.847236383612928
poly 1 3 0.3 : 0.8003101254868052, 0.8456253734439265
poly 1 3 0.5 : 0.7985653980913814, 0.8438165353727752
poly 1 3 0.7 : 0.7966633908398242, 0.8417888153729598
poly 1 3 0.9 : 0.7946574615311511, 0.8395394394575987
poly 1 4 0.1 : 0.801630897440122, 0.8472058780541196
poly 1 4 0.3 : 0.800280451343195, 0.8455932876671136
poly 1 4 0.5 : 0.7985358728985087, 0.8437845510567589
poly 1 4 0.7 : 0.7966349200428466, 0.8417572620794949
poly 1 4 0.9 : 0.7946291098534637, 0.839507753511285
poly 1 5 0.1 : 0.8016133747028352, 0.8471875023785559
poly 1 5 0.3 : 0.8002621673254835, 0.8455738055056312
poly 1 5 0.5 : 0.7985180847159677, 0.8437654517715562
poly 1 5 0.7 : 0.7966176087891553, 0.841739313798529
poly 1 5 0.9 : 0.7946115987954749, 0.8394878595777467
poly 1 6 0.1 : 0.8016016883671604, 0.8471741245987032
poly 1 6 0.3 : 0.8002503524329301, 0.8455625070937355
poly 1 6 0.5 : 0.7985070503389405, 0.8437530766204294
poly 1 6 0.7 : 0.7966054950267507, 0.8417254230307308
poly 1 6 0.9 : 0.7946002705013511, 0.8394759689242318
poly 2 1 0.1 : 0.8018963353319807, 0.8474886550510599
poly 2 1 0.3 : 0.8005453430239644, 0.8458788587128503
poly 2 1 0.5 : 0.7987993507295645, 0.844070813732988
poly 2 1 0.7 : 0.7968951862771865, 0.8420454314467178
poly 2 1 0.9 : 0.7948874697443825, 0.8397927219548363
poly 2 2 0.1 : 0.8017197898252071, 0.8473013771949939
poly 2 2 0.3 : 0.8003684757467407, 0.845688579056658
poly 2 2 0.5 : 0.798624452816773, 0.843880347762642
poly 2 2 0.7 : 0.7967213763015317, 0.8418534328319004
poly 2 2 0.9 : 0.7947156643287591, 0.8396034886766953
poly 2 3 0.1 : 0.8016606959724528, 0.847237621134176
poly 2 3 0.3 : 0.8003098174894916, 0.8456257980157882
poly 2 3 0.5 : 0.7985656428238815, 0.8438161865817877
poly 2 3 0.7 : 0.7966637307275455, 0.8417893511410347
poly 2 3 0.9 : 0.7946574615311511, 0.8395394394575987
poly 2 4 0.1 : 0.8016308755375178, 0.8472063181122466
poly 2 4 0.3 : 0.8002804639031467, 0.8455941543547854
poly 2 4 0.5 : 0.7985362426935924, 0.8437846949117229
poly 2 4 0.7 : 0.7966349936337884, 0.8417580467227452
poly 2 4 0.9 : 0.7946291098534637, 0.839507753511285
poly 2 5 0.1 : 0.801613214481678, 0.8471876179551094
poly 2 5 0.3 : 0.8002623023715216, 0.8455748282534008
poly 2 5 0.5 : 0.7985182024487496, 0.8437658240997838
poly 2 5 0.7 : 0.7966173112471465, 0.8417381227313221
poly 2 5 0.9 : 0.7946115987954749, 0.8394878595777467
poly 2 6 0.1 : 0.8016016723236596, 0.8471746133047685
poly 2 6 0.3 : 0.8002502034670842, 0.845562171427896
poly 2 6 0.5 : 0.7985067610873547, 0.8437542148882455
poly 2 6 0.7 : 0.7966057362742147, 0.841725876905205
poly 2 6 0.9 : 0.7946002705013511, 0.8394759689242318
poly 3 1 0.1 : 0.8018963356296874, 0.84748832945286
poly 3 1 0.3 : 0.8005453835074464, 0.84587804392587
poly 3 1 0.5 : 0.7987997017714463, 0.8440713536248798
poly 3 1 0.7 : 0.796895185267517, 0.8420458278055829
poly 3 1 0.9 : 0.7948874697443825, 0.8397927219548363
poly 3 2 0.1 : 0.801719779268516, 0.8473009139005081
poly 3 2 0.3 : 0.8003684925521259, 0.8456880483743668
poly 3 2 0.5 : 0.7986244657206155, 0.8438807299347708
poly 3 2 0.7 : 0.7967214487052086, 0.8418540916744314
poly 3 2 0.9 : 0.7947156643287591, 0.8396034886766953
poly 3 3 0.1 : 0.8016607890752965, 0.8472371874037443
poly 3 3 0.3 : 0.8003095257548974, 0.8456257368447533
poly 3 3 0.5 : 0.7985651826014257, 0.8438169192802372
poly 3 3 0.7 : 0.7966637364230764, 0.8417891778996072
poly 3 3 0.9 : 0.7946574615311511, 0.8395394394575987
poly 3 4 0.1 : 0.8016308890265353, 0.8472060453372374
poly 3 4 0.3 : 0.8002802277374246, 0.8455931283328016
poly 3 4 0.5 : 0.7985361831577922, 0.8437840824089864
poly 3 4 0.7 : 0.796634623103034, 0.8417577281520879
poly 3 4 0.9 : 0.7946291098534637, 0.839507753511285
poly 3 5 0.1 : 0.8016133646268544, 0.8471866704218344
poly 3 5 0.3 : 0.8002622447737524, 0.8455743855271567
poly 3 5 0.5 : 0.7985183990095104, 0.8437657710263124
poly 3 5 0.7 : 0.7966169427383927, 0.8417382016103638
poly 3 5 0.9 : 0.7946115987954749, 0.8394878595777467
poly 3 6 0.1 : 0.8016019331382441, 0.8471746863373154
poly 3 6 0.3 : 0.8002501832603841, 0.8455613171661036
poly 3 6 0.5 : 0.7985062810718301, 0.8437531728015786
poly 3 6 0.7 : 0.7966053562804428, 0.8417262713202471
poly 3 6 0.9 : 0.7946002705013511, 0.8394759689242318
poly 4 1 0.1 : 0.8018966813302033, 0.8474892120137434
poly 4 1 0.3 : 0.800545682277648, 0.8458780481004755
poly 4 1 0.5 : 0.798799361669441, 0.8440704131492153
poly 4 1 0.7 : 0.79689515750881, 0.8420458279898122
poly 4 1 0.9 : 0.7948874697443825, 0.8397927219548363
poly 4 2 0.1 : 0.8017197940474017, 0.8473009000938138
poly 4 2 0.3 : 0.8003684955081185, 0.845688010484706
poly 4 2 0.5 : 0.7986244561531991, 0.8438807298447429
poly 4 2 0.7 : 0.7967214383604674, 0.8418540760351642
poly 4 2 0.9 : 0.7947156643287591, 0.8396034886766953
poly 4 3 0.1 : 0.8016608233065636, 0.8472371821922052
poly 4 3 0.3 : 0.8003097520095809, 0.8456256300203279
poly 4 3 0.5 : 0.7985651425262648, 0.8438157328260665
poly 4 3 0.7 : 0.7966637466645218, 0.8417891810893154
poly 4 3 0.9 : 0.7946574615311511, 0.8395394394575987
poly 4 4 0.1 : 0.8016308038901538, 0.8472062804275645
poly 4 4 0.3 : 0.8002801621412188, 0.8455931173997484
poly 4 4 0.5 : 0.7985361836151311, 0.8437840847777589
poly 4 4 0.7 : 0.7966346832070662, 0.8417576920494056
poly 4 4 0.9 : 0.7946291098534637, 0.839507753511285
poly 4 5 0.1 : 0.8016129708654868, 0.8471860137426527
poly 4 5 0.3 : 0.8002624212832897, 0.8455749195268383
poly 4 5 0.5 : 0.798518248513983, 0.843765777152969
poly 4 5 0.7 : 0.7966168866517875, 0.841738216766426
poly 4 5 0.9 : 0.7946115987954749, 0.8394878595777467
poly 4 6 0.1 : 0.8016019997502781, 0.8471741099035539
poly 4 6 0.3 : 0.8002504406509559, 0.8455609912661594
poly 4 6 0.5 : 0.7985061836701967, 0.8437534730529773
poly 4 6 0.7 : 0.7966050489041732, 0.8417265297893141
poly 4 6 0.9 : 0.7946002705013511, 0.8394759689242318
rbf 1 1 0.1 : -0.02737345704667802, 0.0015652593995373332
rbf 1 1 0.3 : -0.0271193511458069, 0.0015338889784443088
rbf 1 1 0.5 : -0.02651459583382536, 0.001463753847275262
rbf 1 1 0.7 : -0.02572026181532583, 0.0014166952852039394
rbf 1 1 0.9 : -0.024462339974218185, 0.0013857762757509828
rbf 1 2 0.1 : -0.027373673960915702, 0.0015652593890191913
rbf 1 2 0.3 : -0.027119567935892208, 0.00153388896789175
rbf 1 2 0.5 : -0.02651481167616234, 0.0014637538367301417

rbf 1 2 0.7 : -0.025720476443618835, 0.001416695274663704
rbf 1 2 0.9 : -0.02446255313725163, 0.0013857762652040861
rbf 1 3 0.1 : -0.027373674033683005, 0.0015652593890191913
rbf 1 3 0.3 : -0.027119568008617943, 0.00153388896789175
rbf 1 3 0.5 : -0.026514811748570066, 0.0014637538367301417
rbf 1 3 0.7 : -0.025720476515619418, 0.001416695274663704
rbf 1 3 0.9 : -0.024462553208760562, 0.0013857762652040861
rbf 1 4 0.1 : -0.027373674033707475, 0.0015652593890191913
rbf 1 4 0.3 : -0.027119568008642327, 0.00153388896789175
rbf 1 4 0.5 : -0.0265148117485944, 0.0014637538367301417
rbf 1 4 0.7 : -0.02572047651564353, 0.001416695274663704
rbf 1 4 0.9 : -0.024462553208784633, 0.0013857762652040861
rbf 1 5 0.1 : -0.027373674033707475, 0.0015652593890191913
rbf 1 5 0.3 : -0.027119568008642327, 0.00153388896789175
rbf 1 5 0.5 : -0.0265148117485944, 0.0014637538367301417
rbf 1 5 0.7 : -0.025720476515643576, 0.001416695274663704
rbf 1 5 0.9 : -0.024462553208784633, 0.0013857762652040861
rbf 1 6 0.1 : -0.027373674033707475, 0.0015652593890191913
rbf 1 6 0.3 : -0.027119568008642327, 0.00153388896789175
rbf 1 6 0.5 : -0.0265148117485944, 0.0014637538367301417
rbf 1 6 0.7 : -0.025720476515643576, 0.001416695274663704
rbf 1 6 0.9 : -0.024462553208784633, 0.0013857762652040861
rbf 2 1 0.1 : -0.024727262314230947, 0.0030569009029373095
rbf 2 1 0.3 : -0.024039696280964362, 0.0029815472390450903
rbf 2 1 0.5 : -0.023359945125108972, 0.0028818020050814797
rbf 2 1 0.7 : -0.022552756837179232, 0.0027629946935383565
rbf 2 1 0.9 : -0.021807049139466052, 0.0026373018758366618
rbf 2 2 0.1 : -0.02472769085456661, 0.0030569008818687182
rbf 2 2 0.3 : -0.02404012357349137, 0.002981547217990266
rbf 2 2 0.5 : -0.02336037158081026, 0.0028818019840379794
rbf 2 2 0.7 : -0.02255318229581209, 0.0027629946725071797
rbf 2 2 0.9 : -0.021807473696447532, 0.002637301852993823
rbf 2 3 0.1 : -0.024727690998330186, 0.0030569008818687182
rbf 2 3 0.3 : -0.02404012371683617, 0.002981547217990266
rbf 2 3 0.5 : -0.023360371723874396, 0.0028818019840379794
rbf 2 3 0.7 : -0.022553182438542096, 0.0027629946725071797
rbf 2 3 0.9 : -0.021807473838876268, 0.002637301852993823
rbf 2 4 0.1 : -0.02472769099837846, 0.0030569008818687182
rbf 2 4 0.3 : -0.024040123716884264, 0.002981547217990266
rbf 2 4 0.5 : -0.023360371723922448, 0.0028818019840379794
rbf 2 4 0.7 : -0.022553182438589926, 0.0027629946725071797
rbf 2 4 0.9 : -0.021807473838923962, 0.002637301852993823
rbf 2 5 0.1 : -0.02472769099837846, 0.0030569008818687182
rbf 2 5 0.3 : -0.02404012371688431, 0.002981547217990266
rbf 2 5 0.5 : -0.023360371723922534, 0.0028818019840379794
rbf 2 5 0.7 : -0.022553182438589968, 0.0027629946725071797
rbf 2 5 0.9 : -0.021807473838924007, 0.002637301852993823
rbf 2 6 0.1 : -0.02472769099837846, 0.0030569008818687182
rbf 2 6 0.3 : -0.02404012371688431, 0.002981547217990266
rbf 2 6 0.5 : -0.023360371723922534, 0.0028818019840379794
rbf 2 6 0.7 : -0.022553182438589968, 0.0027629946725071797
rbf 2 6 0.9 : -0.021807473838924007, 0.002637301852993823
rbf 3 1 0.1 : -0.02189946229026498, 0.004171453260425562
rbf 3 1 0.3 : -0.02114373151365756, 0.0040643716316693945
rbf 3 1 0.5 : -0.020245272920768186, 0.003916011749356074
rbf 3 1 0.7 : -0.019195064064422018, 0.003664736445173178
rbf 3 1 0.9 : -0.0181088933526135, 0.003346989813568624
rbf 3 2 0.1 : -0.0219001022868492, 0.004171453224761756
rbf 3 2 0.3 : -0.02114437004907943, 0.00406437159623374
rbf 3 2 0.5 : -0.020245909720004285, 0.003916011714176548
rbf 3 2 0.7 : -0.019195699366862672, 0.003664736410526115
rbf 3 2 0.9 : -0.018109566446471038, 0.0033469897793192427
rbf 3 3 0.1 : -0.021900102501557227, 0.004171453224761756
rbf 3 3 0.3 : -0.02114437026329701, 0.00406437159623374
rbf 3 3 0.5 : -0.020245909933639304, 0.003916011714176548
rbf 3 3 0.7 : -0.019195699579995472, 0.003664736410526115
rbf 3 3 0.9 : -0.01810956665924346, 0.0033469897793192427
rbf 3 4 0.1 : -0.021900102501629215, 0.004171453224761756
rbf 3 4 0.3 : -0.021144370263368906, 0.00406437159623374
rbf 3 4 0.5 : -0.020245909933710938, 0.003916011714176548
rbf 3 4 0.7 : -0.019195699580067015, 0.003664736410526115
rbf 3 4 0.9 : -0.018109566659314826, 0.0033469897793192427
rbf 3 5 0.1 : -0.021900102501629215, 0.004171453224761756
rbf 3 5 0.3 : -0.021144370263368906, 0.00406437159623374
rbf 3 5 0.5 : -0.020245909933710938, 0.003916011714176548
rbf 3 5 0.7 : -0.019195699580067015, 0.003664736410526115
rbf 3 5 0.9 : -0.018109566659314826, 0.0033469897793192427
rbf 3 6 0.1 : -0.021900102501629215, 0.004171453224761756
rbf 3 6 0.3 : -0.021144370263368906, 0.00406437159623374
rbf 3 6 0.5 : -0.020245909933710938, 0.003916011714176548
rbf 3 6 0.7 : -0.019195699580067015, 0.003664736410526115
rbf 3 6 0.9 : -0.018109566659314826, 0.0033469897793192427
rbf 4 1 0.1 : -0.018014017766124324, 0.00458910020336567
rbf 4 1 0.3 : -0.016967930723202997, 0.0041575312366448625
rbf 4 1 0.5 : -0.01593503587209737, 0.003699003535510781
rbf 4 1 0.7 : -0.014986387423296143, 0.00321866239334756
rbf 4 1 0.9 : -0.014053151134004049, 0.002613957615100082
rbf 4 2 0.1 : -0.01801485256405373, 0.0045891001579123625
rbf 4 2 0.3 : -0.01696877907377057, 0.00415753119123885
rbf 4 2 0.5 : -0.015935926620857722, 0.003699003490341024
rbf 4 2 0.7 : -0.014987202457093663, 0.0032186623484837806
rbf 4 2 0.9 : -0.014054037058145475, 0.00261395757040761
rbf 4 3 0.1 : -0.018014852848965646, 0.0045891001579123625
rbf 4 3 0.3 : -0.016968779358382237, 0.00415753119123885
rbf 4 3 0.5 : -0.015935926905207686, 0.003699003490341024
rbf 4 3 0.7 : -0.014987202741261151, 0.0032186623484837806
rbf 4 3 0.9 : -0.014054037342324332, 0.00261395757040761
rbf 4 4 0.1 : -0.018014852849061257, 0.0045891001579123625
rbf 4 4 0.3 : -0.016968779358477758, 0.00415753119123885
rbf 4 4 0.5 : -0.015935926905303033, 0.003699003490341024
rbf 4 4 0.7 : -0.014987202741356542, 0.0032186623484837806
rbf 4 4 0.9 : -0.014054037342419634, 0.00261395757040761
rbf 4 5 0.1 : -0.018014852849061257, 0.0045891001579123625
rbf 4 5 0.3 : -0.016968779358477758, 0.00415753119123885
rbf 4 5 0.5 : -0.015935926905303033, 0.003699003490341024
rbf 4 5 0.7 : -0.014987202741356542, 0.0032186623484837806
rbf 4 5 0.9 : -0.014054037342419634, 0.00261395757040761
rbf 4 6 0.1 : -0.018014852849061257, 0.0045891001579123625
rbf 4 6 0.3 : -0.016968779358477758, 0.00415753119123885
rbf 4 6 0.5 : -0.015935926905303033, 0.003699003490341024
rbf 4 6 0.7 : -0.014987202741356542, 0.0032186623484837806
rbf 4 6 0.9 : -0.014054037342419634, 0.00261395757040761
sigmoid 1 1 0.1 : -0.02785157494475108, -0.0004817589182650739
sigmoid 1 1 0.3 : -0.028038637425086766, -0.0001527348652718974
sigmoid 1 1 0.5 : -0.029327682929847977, -8.39384078961114e-05
sigmoid 1 1 0.7 : -0.027370781055785543, -0.0001527348652718974
sigmoid 1 1 0.9 : -0.02742593928234638, -0.0003516451204561566
sigmoid 1 2 0.1 : -0.02785157494475108, -0.0004817589182650739
sigmoid 1 2 0.3 : -0.028038637425086766, -0.0001527348652718974
sigmoid 1 2 0.5 : -0.029327682929847977, -8.39384078961114e-05
sigmoid 1 2 0.7 : -0.027370781055785543, -0.0001527348652718974
sigmoid 1 2 0.9 : -0.02742593928234638, -0.0003516451204561566
sigmoid 1 3 0.1 : -0.02785157494475108, -0.0004817589182650739
sigmoid 1 3 0.3 : -0.028038637425086766, -0.0001527348652718974
sigmoid 1 3 0.5 : -0.029327682929847977, -8.39384078961114e-05
sigmoid 1 3 0.7 : -0.027370781055785543, -0.0001527348652718974
sigmoid 1 3 0.9 : -0.02742593928234638, -0.0003516451204561566
sigmoid 1 4 0.1 : -0.02785157494475108, -0.0004817589182650739
sigmoid 1 4 0.3 : -0.028038637425086766, -0.0001527348652718974
sigmoid 1 4 0.5 : -0.029327682929847977, -8.39384078961114e-05
sigmoid 1 4 0.7 : -0.027370781055785543, -0.0001527348652718974
sigmoid 1 4 0.9 : -0.02742593928234638, -0.0003516451204561566
sigmoid 1 5 0.1 : -0.02785157494475108, -0.0004817589182650739
sigmoid 1 5 0.3 : -0.028038637425086766, -0.0001527348652718974
sigmoid 1 5 0.5 : -0.029327682929847977, -8.39384078961114e-05
sigmoid 1 5 0.7 : -0.027370781055785543, -0.0001527348652718974
sigmoid 1 5 0.9 : -0.02742593928234638, -0.0003516451204561566
sigmoid 1 6 0.1 : -0.02785157494475108, -0.0004817589182650739
sigmoid 1 6 0.3 : -0.028038637425086766, -0.0001527348652718974
sigmoid 1 6 0.5 : -0.029327682929847977, -8.39384078961114e-05
sigmoid 1 6 0.7 : -0.027370781055785543, -0.0001527348652718974
sigmoid 1 6 0.9 : -0.02742593928234638, -0.0003516451204561566
sigmoid 2 1 0.1 : -0.02785157494475108, -0.0004817589182650739
sigmoid 2 1 0.3 : -0.028038637425086766, -0.0001527348652718974
sigmoid 2 1 0.5 : -0.029327682929847977, -8.39384078961114e-05
sigmoid 2 1 0.7 : -0.027370781055785543, -0.0001527348652718974
sigmoid 2 1 0.9 : -0.02742593928234638, -0.0003516451204561566
sigmoid 2 2 0.1 : -0.02785157494475108, -0.0004817589182650739

sigmoid 2 2 0.3 : -0.028038637425086766, -0.0001527348652718974
sigmoid 2 2 0.5 : -0.029327682929847977, -8.39384078961114e-05
sigmoid 2 2 0.7 : -0.027370781055785543, -0.0001527348652718974
sigmoid 2 2 0.9 : -0.02742593928234638, -0.0003516451204561566
sigmoid 2 3 0.1 : -0.02785157494475108, -0.0004817589182650739
sigmoid 2 3 0.3 : -0.028038637425086766, -0.0001527348652718974
sigmoid 2 3 0.5 : -0.029327682929847977, -8.39384078961114e-05
sigmoid 2 3 0.7 : -0.027370781055785543, -0.0001527348652718974
sigmoid 2 3 0.9 : -0.02742593928234638, -0.0003516451204561566
sigmoid 2 4 0.1 : -0.02785157494475108, -0.0004817589182650739
sigmoid 2 4 0.3 : -0.028038637425086766, -0.0001527348652718974
sigmoid 2 4 0.5 : -0.029327682929847977, -8.39384078961114e-05
sigmoid 2 4 0.7 : -0.027370781055785543, -0.0001527348652718974
sigmoid 2 4 0.9 : -0.02742593928234638, -0.0003516451204561566
sigmoid 2 5 0.1 : -0.02785157494475108, -0.0004817589182650739
sigmoid 2 5 0.3 : -0.028038637425086766, -0.0001527348652718974
sigmoid 2 5 0.5 : -0.029327682929847977, -8.39384078961114e-05
sigmoid 2 5 0.7 : -0.027370781055785543, -0.0001527348652718974
sigmoid 2 5 0.9 : -0.02742593928234638, -0.0003516451204561566
sigmoid 2 6 0.1 : -0.02785157494475108, -0.0004817589182650739
sigmoid 2 6 0.3 : -0.028038637425086766, -0.0001527348652718974
sigmoid 2 6 0.5 : -0.029327682929847977, -8.39384078961114e-05
sigmoid 2 6 0.7 : -0.027370781055785543, -0.0001527348652718974
sigmoid 2 6 0.9 : -0.02742593928234638, -0.0003516451204561566
sigmoid 3 1 0.1 : -0.02785157494475108, -0.0004817589182650739
sigmoid 3 1 0.3 : -0.028038637425086766, -0.0001527348652718974
sigmoid 3 1 0.5 : -0.029327682929847977, -8.39384078961114e-05
sigmoid 3 1 0.7 : -0.027370781055785543, -0.0001527348652718974
sigmoid 3 1 0.9 : -0.02742593928234638, -0.0003516451204561566
sigmoid 3 2 0.1 : -0.02785157494475108, -0.0004817589182650739
sigmoid 3 2 0.3 : -0.028038637425086766, -0.0001527348652718974
sigmoid 3 2 0.5 : -0.029327682929847977, -8.39384078961114e-05
sigmoid 3 2 0.7 : -0.027370781055785543, -0.0001527348652718974
sigmoid 3 2 0.9 : -0.02742593928234638, -0.0003516451204561566
sigmoid 3 3 0.1 : -0.02785157494475108, -0.0004817589182650739
sigmoid 3 3 0.3 : -0.028038637425086766, -0.0001527348652718974
sigmoid 3 3 0.5 : -0.029327682929847977, -8.39384078961114e-05
sigmoid 3 3 0.7 : -0.027370781055785543, -0.0001527348652718974
sigmoid 3 3 0.9 : -0.02742593928234638, -0.0003516451204561566
sigmoid 3 4 0.1 : -0.02785157494475108, -0.0004817589182650739
sigmoid 3 4 0.3 : -0.028038637425086766, -0.0001527348652718974
sigmoid 3 4 0.5 : -0.029327682929847977, -8.39384078961114e-05
sigmoid 3 4 0.7 : -0.027370781055785543, -0.0001527348652718974
sigmoid 3 4 0.9 : -0.02742593928234638, -0.0003516451204561566
sigmoid 3 5 0.1 : -0.02785157494475108, -0.0004817589182650739
sigmoid 3 5 0.3 : -0.028038637425086766, -0.0001527348652718974
sigmoid 3 5 0.5 : -0.029327682929847977, -8.39384078961114e-05
sigmoid 3 5 0.7 : -0.027370781055785543, -0.0001527348652718974
sigmoid 3 5 0.9 : -0.02742593928234638, -0.0003516451204561566
sigmoid 3 6 0.1 : -0.02785157494475108, -0.0004817589182650739
sigmoid 3 6 0.3 : -0.028038637425086766, -0.0001527348652718974
sigmoid 3 6 0.5 : -0.029327682929847977, -8.39384078961114e-05
sigmoid 3 6 0.7 : -0.027370781055785543, -0.0001527348652718974
sigmoid 3 6 0.9 : -0.02742593928234638, -0.0003516451204561566
sigmoid 4 1 0.1 : -0.02785157494475108, -0.0004817589182650739
sigmoid 4 1 0.3 : -0.028038637425086766, -0.0001527348652718974
sigmoid 4 1 0.5 : -0.029327682929847977, -8.39384078961114e-05
sigmoid 4 1 0.7 : -0.027370781055785543, -0.0001527348652718974
sigmoid 4 1 0.9 : -0.02742593928234638, -0.0003516451204561566
sigmoid 4 2 0.1 : -0.02785157494475108, -0.0004817589182650739
sigmoid 4 2 0.3 : -0.028038637425086766, -0.0001527348652718974
sigmoid 4 2 0.5 : -0.029327682929847977, -8.39384078961114e-05
sigmoid 4 2 0.7 : -0.027370781055785543, -0.0001527348652718974
sigmoid 4 2 0.9 : -0.02742593928234638, -0.0003516451204561566
sigmoid 4 3 0.1 : -0.02785157494475108, -0.0004817589182650739
sigmoid 4 3 0.3 : -0.028038637425086766, -0.0001527348652718974
sigmoid 4 3 0.5 : -0.029327682929847977, -8.39384078961114e-05
sigmoid 4 3 0.7 : -0.027370781055785543, -0.0001527348652718974
sigmoid 4 3 0.9 : -0.02742593928234638, -0.0003516451204561566
sigmoid 4 4 0.1 : -0.02785157494475108, -0.0004817589182650739
sigmoid 4 4 0.3 : -0.028038637425086766, -0.0001527348652718974
sigmoid 4 4 0.5 : -0.029327682929847977, -8.39384078961114e-05
sigmoid 4 4 0.7 : -0.027370781055785543, -0.0001527348652718974
sigmoid 4 4 0.9 : -0.02742593928234638, -0.0003516451204561566
sigmoid 4 5 0.1 : -0.02785157494475108, -0.0004817589182650739
sigmoid 4 5 0.3 : -0.028038637425086766, -0.0001527348652718974
sigmoid 4 5 0.5 : -0.029327682929847977, -8.39384078961114e-05
sigmoid 4 5 0.7 : -0.027370781055785543, -0.0001527348652718974
sigmoid 4 5 0.9 : -0.02742593928234638, -0.0003516451204561566
sigmoid 4 6 0.1 : -0.02785157494475108, -0.0004817589182650739
sigmoid 4 6 0.3 : -0.028038637425086766, -0.0001527348652718974
sigmoid 4 6 0.5 : -0.029327682929847977, -8.39384078961114e-05
sigmoid 4 6 0.7 : -0.027370781055785543, -0.0001527348652718974
sigmoid 4 6 0.9 : -0.02742593928234638, -0.0003516451204561566

Grid search of ElasticNet, MACCS
ElasticNet(alpha= i, l1_ratio= j, max_iter=100000)
i j : accuracy of prediction using cross validation, accuracy of prediction using test data
0.001 0.0 : 0.8449939594898164, 0.8738308116296882
0.001 0.2 : 0.8433128342022428, 0.8739982237871107
0.001 0.4 : 0.8407023614228029, 0.8739704348545376
0.001 0.6 : 0.836604864916848, 0.873414746072541
0.001 0.8 : 0.8298847617084926, 0.8718423093726742
0.001 1.0 : 0.8173252099967401, 0.868240755054
0.01 0.0 : 0.8336097138965503, 0.8425338675790939
0.01 0.2 : 0.8370471758518996, 0.8468555260024428
0.01 0.4 : 0.8403115937283584, 0.8520168226642562
0.01 0.6 : 0.8437437673967076, 0.8578454736445872
0.01 0.8 : 0.846297973709682, 0.8631507377775826
0.01 1.0 : 0.845132612216964, 0.8659116444741578
0.1 0.0 : 0.6895464870005161, 0.7012801296263389
0.1 0.2 : 0.6939677845498522, 0.7071451850349039
0.1 0.4 : 0.6997448665701619, 0.715695207341881
0.1 0.6 : 0.7102959291843476, 0.7270982377938501
0.1 0.8 : 0.7301451711980617, 0.7434629657813336
0.1 1.0 : 0.7690105431484315, 0.7788875028394961

Grid search of RandomForest, MACCS
RandomForestRegressor(n_estimators= i)
i: accuracy of prediction using cross validation, accuracy of prediction using test data
100 1.0 : 0.7903681867472993, 0.8099179141901498
500 1.0 : 0.7932964556336424, 0.8108957673411256
1000 1.0 : 0.7921153886687657, 0.8099787065966266
2000 1.0 : 0.7914630223822856, 0.8092010438622361

Grid search of NeuralNetwork, MACCS
MLPRegressor(activation=act, alpha=a, batch_size=batch, beta_1=0.9,
beta_2=0.999, early_stopping=False, epsilon=1e-08,
hidden_layer_sizes=hid, learning_rate='constant',
learning_rate_init=0.001, max_iter=100000, momentum=0.9,
n_iter_no_change=10, nesterovs_momentum=True,
power_t=0.5,
random_state=1, shuffle=True, solver='adam', tol=0.0001,
validation_fraction=0.1, verbose=False, warm_start=False)
act (hid) a batch: accuracy of prediction using cross validation, accuracy of prediction using test data
relu (200,) 0.0001 500 : 0.7599083613374579, 0.871885708701881
relu (200,) 0.0001 200 : 0.8447920064431951, 0.9119320258164503
relu (200,) 0.0001 100 : 0.838464642608001, 0.9068131073859176
relu (200,) 0.0001 50 : 0.8404362080606017, 0.9104630172233327
relu (200,) 0.001 500 : 0.7618319599623599, 0.8717431598533476
relu (200,) 0.001 200 : 0.8448042084273292, 0.9101289090740293
relu (200,) 0.001 100 : 0.8462910416718294, 0.9051191496111594
relu (200,) 0.001 50 : 0.8472043404621399, 0.9143356619069367
relu (200,) 0.01 500 : 0.7700738850717895, 0.8721358526250658
relu (200,) 0.01 200 : 0.847190898374682, 0.9081241336388357
relu (200,) 0.01 100 : 0.8414975011783608, 0.9071442263056707
relu (200,) 0.01 50 : 0.8465836650188487, 0.9119651951032324
relu (200, 200) 0.0001 500 : 0.8100163019611211, 0.9030986141094172
relu (200, 200) 0.0001 200 : 0.841621577069809, 0.9104707294677993
relu (200, 200) 0.0001 100 : 0.8368646410621924, 0.9102216183785168
relu (200, 200) 0.0001 50 : 0.8406067625910785,

0.9064787270914938
relu (200, 200) 0.001 500 : 0.8092129778739263, 0.9049512589821088
relu (200, 200) 0.001 200 : 0.8406515853066086, 0.9041692583619598
relu (200, 200) 0.001 100 : 0.8305945898849965, 0.9086824070644792
relu (200, 200) 0.001 50 : 0.839477852569156, 0.903142445015867
relu (200, 200) 0.01 500 : 0.811573385054779, 0.9046502806560819
relu (200, 200) 0.01 200 : 0.8419906776467154, 0.9050088590312022
relu (200, 200) 0.01 100 : 0.8340627479064008, 0.9077353303514237
relu (200, 200) 0.01 50 : 0.8374802572044157, 0.903035852951961
relu (100,) 0.0001 500 : 0.7620270168681479, 0.8956287003879224
relu (100,) 0.0001 200 : 0.8498107854330138, 0.922772289259241
relu (100,) 0.0001 100 : 0.8475859685546905, 0.912499261662453
relu (100,) 0.0001 50 : 0.8514395344770114, 0.9074326959207031
relu (100,) 0.001 500 : 0.7174839548343528, 0.8964834441647456
relu (100,) 0.001 200 : 0.8488704832330622, 0.9067949889378776
relu (100,) 0.001 100 : 0.8569947133836877, 0.9115220290135863
relu (100,) 0.001 50 : 0.8463412359097523, 0.9182897047958799
relu (100,) 0.01 500 : 0.7661967609306869, 0.9018965284551781
relu (100,) 0.01 200 : 0.8511463386198324, 0.9086518933645404
relu (100,) 0.01 100 : 0.8429898353768304, 0.9089821065653917
relu (100,) 0.01 50 : 0.8411173017497692, 0.9188853373996411
relu (100, 100) 0.0001 500 : 0.7897915230799429, 0.8876743061856183
relu (100, 100) 0.0001 200 : 0.8495171823548203, 0.9105718476232993
relu (100, 100) 0.0001 100 : 0.8442482917625999, 0.9102261845647744
relu (100, 100) 0.0001 50 : 0.8377300437796915, 0.9080481566194724
relu (100, 100) 0.001 500 : 0.792872844984584, 0.892784807708561
relu (100, 100) 0.001 200 : 0.8476414182417912, 0.8916733528441154
relu (100, 100) 0.001 100 : 0.8470031414185837, 0.9005773171293094
relu (100, 100) 0.001 50 : 0.8372476422389002, 0.9233466926953131
relu (100, 100) 0.01 500 : 0.7949925162767413, 0.8897320560759555
relu (100, 100) 0.01 200 : 0.8418124227233704, 0.8919790043008367
relu (100, 100) 0.01 100 : 0.8477597614181102, 0.9010343498871055
relu (100, 100) 0.01 50 : 0.8401932944014249, 0.9173056069263016
relu (100, 200) 0.0001 500 : 0.7981308211898948, 0.8876261843457208
relu (100, 200) 0.0001 200 : 0.83698300818634, 0.8845388992209714
relu (100, 200) 0.0001 100 : 0.8414316969057151, 0.8890720120189255
relu (100, 200) 0.0001 50 : 0.8389656132830178, 0.8936280275716334
relu (100, 200) 0.001 500 : 0.8127454087413499, 0.8970285147106593
relu (100, 200) 0.001 200 : 0.8400032169577176, 0.8809664958777654
relu (100, 200) 0.001 100 : 0.8367988127724205, 0.8986228598580596
relu (100, 200) 0.001 50 : 0.8464397725163592, 0.8974292726209228
relu (100, 200) 0.01 500 : 0.8198348962728998, 0.8951546001447918
relu (100, 200) 0.01 200 : 0.8382943639181025, 0.8788368131840208
relu (100, 200) 0.01 100 : 0.8398949218381828, 0.8943377230117923
relu (100, 200) 0.01 50 : 0.8475009009549611, 0.8884880880546463
relu (50,) 0.0001 500 : 0.7491658058347466, 0.8765010909158006
relu (50,) 0.0001 200 : 0.8481737837859878, 0.9191607760582364
relu (50,) 0.0001 100 : 0.8502707829828655, 0.9088699069472227
relu (50,) 0.0001 50 : 0.843446401100068, 0.9139384038757065
relu (50,) 0.001 500 : 0.7497650012373333, 0.8763415133776058
relu (50,) 0.001 200 : 0.8475963316772734, 0.9189569585410079
relu (50,) 0.001 100 : 0.8508919956067149, 0.908735929432786
relu (50,) 0.001 50 : 0.8459242790677738, 0.9120966064009265
relu (50,) 0.01 500 : 0.7612522428832186, 0.8765522236423691
relu (50,) 0.01 200 : 0.8510915499205973, 0.9211847166494621
relu (50,) 0.01 100 : 0.8507845723109353, 0.9089463339853702
relu (50,) 0.01 50 : 0.8432089235055408, 0.9122408629054484
relu (50, 50) 0.0001 500 : 0.7755761398076592, 0.8921166393913187
relu (50, 50) 0.0001 200 : 0.8413833743323711, 0.9010703276272627
relu (50, 50) 0.0001 100 : 0.8332598652388201, 0.8994747536654432
relu (50, 50) 0.0001 50 : 0.833046333858021, 0.8894480066462631
relu (50, 50) 0.001 500 : 0.7586249917628228, 0.8925974995880296
relu (50, 50) 0.001 200 : 0.8390142071055695, 0.8963921810383744
relu (50, 50) 0.001 100 : 0.8296183755527705, 0.8922383358528606
relu (50, 50) 0.001 50 : 0.8351547568654928, 0.9003004297663472
relu (50, 50) 0.01 500 : 0.7631477223539512, 0.8917216793499687
relu (50, 50) 0.01 200 : 0.834908558682265, 0.8955964147029324
relu (50, 50) 0.01 100 : 0.8293327298374622, 0.8958254216687122
relu (50, 50) 0.01 50 : 0.8328810253254723, 0.8890091147732853
relu (50, 100) 0.0001 500 : 0.7824510629685724, 0.8975627697362804
relu (50, 100) 0.0001 200 : 0.8410824539585681, 0.8992701580831508
relu (50, 100) 0.0001 100 : 0.8413310663860016, 0.9081263116085876
relu (50, 100) 0.0001 50 : 0.8405249513000822, 0.9026793804444972
relu (50, 100) 0.001 500 : 0.7879601579528343, 0.9018548300314676
relu (50, 100) 0.001 200 : 0.8392938347717399, 0.9028084984982083
relu (50, 100) 0.001 100 : 0.8408830403605559, 0.8978534593793057
relu (50, 100) 0.001 50 : 0.8326954999989946, 0.9055549329268657
relu (50, 100) 0.01 500 : 0.8030693497805552, 0.8978075419413468
relu (50, 100) 0.01 200 : 0.8471719580897593, 0.9060630497854278
relu (50, 100) 0.01 100 : 0.8462786507991972, 0.9000435894496466
relu (50, 100) 0.01 50 : 0.8461347504775848, 0.9088707627071726
relu (50, 200) 0.0001 500 : 0.7909962258187168, 0.8747615790287556
relu (50, 200) 0.0001 200 : 0.8414729192876955, 0.8701527361315766
relu (50, 200) 0.0001 100 : 0.8244185344129168, 0.8679818260110648
relu (50, 200) 0.0001 50 : 0.8333926072279307, 0.8736535890136187
relu (50, 200) 0.001 500 : 0.7750226832322127, 0.8723413103784131
relu (50, 200) 0.001 200 : 0.8340603505998642, 0.8776860332829348
relu (50, 200) 0.001 100 : 0.8373943493799871, 0.8781322144516884
relu (50, 200) 0.001 50 : 0.8198335272150435, 0.8870786829216195
relu (50, 200) 0.01 500 : 0.7838816601157118, 0.8801356095147389
relu (50, 200) 0.01 200 : 0.8409657329065308, 0.889257934528876
relu (50, 200) 0.01 100 : 0.8280560041754237, 0.884679807435828
relu (50, 200) 0.01 50 : 0.826763976959333, 0.8848650244986459
tanh (200,) 0.0001 500 : 0.8330387026772884, 0.8912723298931037
tanh (200,) 0.0001 200 : 0.8506622381103176, 0.8763471136012003
tanh (200,) 0.0001 100 : 0.8446041467272046, 0.8849906325569293
tanh (200,) 0.0001 50 : 0.8441006421691768, 0.8885916077197287
tanh (200,) 0.001 500 : 0.8330750554115915, 0.8913180254719524
tanh (200,) 0.001 200 : 0.8505928786739944, 0.8766292722718229
tanh (200,) 0.001 100 : 0.8445544666345921, 0.8852406677257872
tanh (200,) 0.001 50 : 0.8422663271340017, 0.8888917309317254
tanh (200,) 0.01 500 : 0.8333949358810175, 0.8905310575192816
tanh (200,) 0.01 200 : 0.850557208300275, 0.8786579424421405
tanh (200,) 0.01 100 : 0.8448775317043662, 0.8883396843927093
tanh (200,) 0.01 50 : 0.842988143069336, 0.8916180070770793

tanh (200, 200) 0.0001 500 : 0.8073585739694773, -0.026186209296115592
tanh (200, 200) 0.0001 200 : -0.009257399162132973, 0.8736880814215067
tanh (200, 200) 0.0001 100 : 0.49052614250001636, 0.888361619112906
tanh (200, 200) 0.0001 50 : 0.81729370658002, 0.8843278644573463
tanh (200, 200) 0.001 500 : 0.8082582565253794, -0.026186326461304255
tanh (200, 200) 0.001 200 : -0.009257623402565373, 0.8737016063747602
tanh (200, 200) 0.001 100 : 0.49036198989493196, 0.8886916357072617
tanh (200, 200) 0.001 50 : 0.8185186127937755, 0.8843174768308046
tanh (200, 200) 0.01 500 : 0.8089127844496922, -0.02618749579757118
tanh (200, 200) 0.01 200 : -0.009259257475278204, 0.8740056516126182
tanh (200, 200) 0.01 100 : 0.4894015167970334, 0.890717112832393
tanh (200, 200) 0.01 50 : 0.8173372495138903, 0.8881715746463807
tanh (100,) 0.0001 500 : 0.8316293553290809, 0.8817933813425648
tanh (100,) 0.0001 200 : 0.8407515706587443, 0.869509848156403
tanh (100,) 0.0001 100 : 0.8410090281605861, 0.870987161628462
tanh (100,) 0.0001 50 : 0.8399454345148991, 0.8795001522384001
tanh (100,) 0.001 500 : 0.8315859088479247, 0.8817527793181058
tanh (100,) 0.001 200 : 0.8419528617326755, 0.8690495620735601
tanh (100,) 0.001 100 : 0.8406834221899173, 0.8738101771443778
tanh (100,) 0.001 50 : 0.8369815379139279, 0.8805839229647854
tanh (100,) 0.01 500 : 0.8301339995254707, 0.882992745256721
tanh (100,) 0.01 200 : 0.8435665172258867, 0.8741950854183518
tanh (100,) 0.01 100 : 0.8373029421519824, 0.8806380329488257
tanh (100,) 0.01 50 : 0.8396817893767837, 0.8803848143758877
tanh (100, 100) 0.0001 500 : 0.14282547334107737, -0.02305675096279791
tanh (100, 100) 0.0001 200 : -0.008216761115244654, 0.8346994450951548
tanh (100, 100) 0.0001 100 : 0.4789965391009102, -0.01632034497091528
tanh (100, 100) 0.0001 50 : 0.8148912203716044, 0.8617772124172769
tanh (100, 100) 0.001 500 : 0.14310045235738092, -0.023056889758358512
tanh (100, 100) 0.001 200 : -0.008216876260246631, 0.8548177072830457
tanh (100, 100) 0.001 100 : 0.4791799692545009, -0.016320474023870046
tanh (100, 100) 0.001 50 : 0.8133645411683746, 0.8627005043942689
tanh (100, 100) 0.01 500 : 0.1431652388335805, -0.023058281676632708
tanh (100, 100) 0.01 200 : -0.008218003963965792, 0.8621240978236884
tanh (100, 100) 0.01 100 : 0.477302590514328, -0.01632150789647091
tanh (100, 100) 0.01 50 : 0.8116853899067109, 0.8661995385838677
tanh (100, 200) 0.0001 500 : 0.791027992941973, -0.011837189056501218
tanh (100, 200) 0.0001 200 : 0.8306415799826302, 0.8710112101121595
tanh (100, 200) 0.0001 100 : 0.8224964270721846, 0.8766682001834185
tanh (100, 200) 0.0001 50 : 0.8221369885629691, 0.87855775685625
tanh (100, 200) 0.001 500 : 0.7930516780766037, -0.011837151131385282
tanh (100, 200) 0.001 200 : 0.8306514149888091, 0.8711064176048784
tanh (100, 200) 0.001 100 : 0.8224955189044509, 0.876746225235202
tanh (100, 200) 0.001 50 : 0.8221331856211321, 0.8775443431922199
tanh (100, 200) 0.01 500 : 0.7914735047375964, -0.011836773850982807
tanh (100, 200) 0.01 200 : 0.829685379718461, 0.8711941072395277
tanh (100, 200) 0.01 100 : 0.8221045033842325, 0.8775350959016849
tanh (100, 200) 0.01 50 : 0.8214153632489867, 0.8817939258903452
tanh (50,) 0.0001 500 : 0.8204589443185745, -0.019475203420279863
tanh (50,) 0.0001 200 : 0.840884377005656, 0.8551302608849354
tanh (50,) 0.0001 100 : 0.8322368135724794, 0.8549421634369666
tanh (50,) 0.0001 50 : 0.8383413182332845, 0.8712812202139641
tanh (50,) 0.001 500 : 0.8235889851206595, -0.019475145196488075
tanh (50,) 0.001 200 : 0.8399196211481378, 0.8560398774104846
tanh (50,) 0.001 100 : 0.8306064959981742, 0.8596103373424195
tanh (50,) 0.001 50 : 0.8346810955880553, 0.8709048008444132
tanh (50,) 0.01 500 : 0.8217426658579591, -0.019474401795457563
tanh (50,) 0.01 200 : 0.8386298776505179, 0.8590069375244359
tanh (50,) 0.01 100 : 0.8395020768423886, 0.8710096807654868
tanh (50,) 0.01 50 : 0.833728709072669, 0.8729381630938329
tanh (50, 50) 0.0001 500 : 0.7764181263226644, -0.027371036995926135
tanh (50, 50) 0.0001 200 : 0.8056100792445451, 0.8475807342150604
tanh (50, 50) 0.0001 100 : 0.8036141563612873, 0.8402896721511268
tanh (50, 50) 0.0001 50 : 0.8185380490790044, 0.8403703107798317
tanh (50, 50) 0.001 500 : 0.7744649627736628, -0.02737126905845444
tanh (50, 50) 0.001 200 : 0.8087174620172604, 0.8475469034830965
tanh (50, 50) 0.001 100 : 0.8069363795892566, 0.8430479886898297
tanh (50, 50) 0.001 50 : 0.8081639188370925, 0.8434224100947996
tanh (50, 50) 0.01 500 : 0.7584320329855465, -0.02737354972657635
tanh (50, 50) 0.01 200 : 0.8108813723443656, 0.8471843434943421
tanh (50, 50) 0.01 100 : 0.8077316750948184, 0.8543571846523448
tanh (50, 50) 0.01 50 : 0.8159124452427168, 0.8440021095814875
tanh (50, 100) 0.0001 500 : 0.14113122830990515, -0.0199884872619438
tanh (50, 100) 0.0001 200 : 0.14972769592945004, 0.8610603574765325
tanh (50, 100) 0.0001 100 : 0.3066269066191995, 0.8794405338604616
tanh (50, 100) 0.0001 50 : 0.8285296045757466, 0.8783858348420235
tanh (50, 100) 0.001 500 : 0.14148759600422572, -0.019988610100253767
tanh (50, 100) 0.001 200 : 0.1483699037802102, 0.8613294340298977
tanh (50, 100) 0.001 100 : 0.3087748483616231, 0.8797179909841493
tanh (50, 100) 0.001 50 : 0.827781040196661, 0.8796874358754978
tanh (50, 100) 0.01 500 : 0.14217205803869257, -0.019989946280805748
tanh (50, 100) 0.01 200 : 0.1484866155431282, 0.8641429457173574
tanh (50, 100) 0.01 100 : 0.3114595129590463, 0.884181268119392
tanh (50, 100) 0.01 50 : 0.8208223896985507, 0.8783928049266986
tanh (50, 200) 0.0001 500 : 0.7871975373948372, 0.8775633267574142
tanh (50, 200) 0.0001 200 : 0.8370893018829124, 0.864758787150258
tanh (50, 200) 0.0001 100 : 0.8347032325249643, 0.874837271904366
tanh (50, 200) 0.0001 50 : 0.8365636536140251, 0.882062166925911
tanh (50, 200) 0.001 500 : 0.7891209440271194, 0.8775926302166743
tanh (50, 200) 0.001 200 : 0.837039884893707, 0.8647936649366406
tanh (50, 200) 0.001 100 : 0.8367839612327032, 0.8748691117222045
tanh (50, 200) 0.001 50 : 0.8364822728058556, 0.8819860679913325
tanh (50, 200) 0.01 500 : 0.7879872391530652, 0.877850091395751
tanh (50, 200) 0.01 200 : 0.8369271339194659, 0.8648455625586253
tanh (50, 200) 0.01 100 : 0.8366359912030713, 0.8750987535758307
tanh (50, 200) 0.01 50 : 0.8342814298346859, 0.8823634185426397
logistic (200,) 0.0001 500 : 0.8205043768135146, 0.8934510844571504
logistic (200,) 0.0001 200 : 0.8441480876962164, 0.8903882667544805

logistic (200,) 0.0001 100 : 0.8386166755447796, 0.8967140943103815
logistic (200,) 0.0001 50 : 0.8360048154509704, 0.899453203524052
logistic (200,) 0.001 500 : 0.8206664723918158, 0.894065749377699
logistic (200,) 0.001 200 : 0.8439434956324359, 0.8916357413960765
logistic (200,) 0.001 100 : 0.8387700384751311, 0.899093650607614
logistic (200,) 0.001 50 : 0.8354848467365569, 0.9024759968665732
logistic (200,) 0.01 500 : 0.8220270999812913, 0.8961070550136038
logistic (200,) 0.01 200 : 0.8441257798423475, 0.8963734257054004
logistic (200,) 0.01 100 : 0.8398215745138768, 0.9049830274206037
logistic (200,) 0.01 50 : 0.8399923669873438, 0.9066380455036143
logistic (200, 200) 0.0001 500 : -0.008849198519006275, -0.021273300239627035
logistic (200, 200) 0.0001 200 : -0.00937233531406818, 0.8487198829765983
logistic (200, 200) 0.0001 100 : -0.008872331416834855, -0.019384355151327748
logistic (200, 200) 0.0001 50 : 0.8145519640963986, 0.8794422703051225
logistic (200, 200) 0.001 500 : -0.00884920685368864, -0.021273342885140156
logistic (200, 200) 0.001 200 : -0.009372447285769159, 0.8496217192849161
logistic (200, 200) 0.001 100 : -0.008895862332499949, -0.0193832466533177
logistic (200, 200) 0.001 50 : 0.8160802360830312, 0.8812607575508254
logistic (200, 200) 0.01 500 : -0.008849610831226817, -0.021273934035060504
logistic (200, 200) 0.01 200 : -0.009373933783483501, 0.8602554468651035
logistic (200, 200) 0.01 100 : 0.15064117183607997, -0.019370461169686326
logistic (200, 200) 0.01 50 : 0.8205308691138514, 0.8846508240934712
logistic (100,) 0.0001 500 : 0.8283191995527531, 0.8788040812353997
logistic (100,) 0.0001 200 : 0.8453494383310435, 0.8764137681479742
logistic (100,) 0.0001 100 : 0.8388169502747228, 0.8834414508150877
logistic (100,) 0.0001 50 : 0.833902155188327, 0.8844894789523533
logistic (100,) 0.001 500 : 0.8290587510669557, 0.8807349577361492
logistic (100,) 0.001 200 : 0.8456530557287634, 0.8791446579665493
logistic (100,) 0.001 100 : 0.837935094670974, 0.8861681899438865
logistic (100,) 0.001 50 : 0.8348584198485943, 0.8877724271047887
logistic (100,) 0.01 500 : 0.8291197416464581, 0.8867782469815687
logistic (100,) 0.01 200 : 0.8443979356880629, 0.8892777782344955
logistic (100,) 0.01 100 : 0.8361220302656989, 0.8949183447493373
logistic (100,) 0.01 50 : 0.8363382616600348, 0.8954206809821538
logistic (100, 100) 0.0001 500 : -0.008797329336860792, -0.02709840762944915
logistic (100, 100) 0.0001 200 : -0.009472246300007292, -0.017311025347125808
logistic (100, 100) 0.0001 100 : -0.008270719512040569, -0.016542470463576375
logistic (100, 100) 0.0001 50 : -0.009037242671194523, -0.016987627245216297
logistic (100, 100) 0.001 500 : -0.00879727007009432, -0.02709388064563778
logistic (100, 100) 0.001 200 : -0.009472118391544493, -0.01731094104546793
logistic (100, 100) 0.001 100 : -0.008270730235428925, -0.01654252328774275
logistic (100, 100) 0.001 50 : -0.009036754926484836, -0.016986733081111316
logistic (100, 100) 0.01 500 : -0.008799809391726487, -0.027052626294554738
logistic (100, 100) 0.01 200 : -0.009470724473842474, 0.863519480719393
logistic (100, 100) 0.01 100 : -0.008270748816475404, -0.016543315848341678
logistic (100, 100) 0.01 50 : 0.15005829918771668, 0.86738561806395
logistic (100, 200) 0.0001 500 : 0.6073356640018216, -0.016618914117302097
logistic (100, 200) 0.0001 200 : -0.009687790172517908, 0.8502440100288918
logistic (100, 200) 0.0001 100 : 0.3206883164491854, 0.8518272258790041
logistic (100, 200) 0.0001 50 : 0.8200380219265584, 0.8652738141740705
logistic (100, 200) 0.001 500 : 0.6073040514911398, -0.016618921835180656
logistic (100, 200) 0.001 200 : -0.00968783522275527, 0.8515069887458353
logistic (100, 200) 0.001 100 : 0.3205674152700594, 0.8545685429985119
logistic (100, 200) 0.001 50 : 0.8193841609364844, 0.8697230590264345
logistic (100, 200) 0.01 500 : 0.6086190423363912, -0.016619110080636323
logistic (100, 200) 0.01 200 : -0.009688233724599992, 0.8597964156652408
logistic (100, 200) 0.01 100 : 0.49750356056099854, 0.8619607480003738
logistic (100, 200) 0.01 50 : 0.8217601486738596, 0.8740953170040712
logistic (50,) 0.0001 500 : 0.8191447837105235, 0.8792554096294731
logistic (50,) 0.0001 200 : 0.8398254286688671, 0.8643522484229149
logistic (50,) 0.0001 100 : 0.834448944236476, 0.8779688854449631
logistic (50,) 0.0001 50 : 0.8323668711246256, 0.8740615910638004
logistic (50,) 0.001 500 : 0.8215661461467381, 0.879644867517144
logistic (50,) 0.001 200 : 0.8401714550276583, 0.8681029746599189
logistic (50,) 0.001 100 : 0.8311998335912048, 0.8801281404735941
logistic (50,) 0.001 50 : 0.8318164171584715, 0.8746605352796599
logistic (50,) 0.01 500 : 0.8238769352646547, 0.885381428706224
logistic (50,) 0.01 200 : 0.839395458754792, 0.8694279651908201
logistic (50,) 0.01 100 : 0.8325023161133634, 0.8788595138363821
logistic (50,) 0.01 50 : 0.8345383916124296, 0.8884963832651205
logistic (50, 50) 0.0001 500 : -0.008857300863543794, -0.022258386273691366
logistic (50, 50) 0.0001 200 : -0.007780273557692263, -0.01768106387348989
logistic (50, 50) 0.0001 100 : -0.009250282578177327, -0.018489511748701393
logistic (50, 50) 0.0001 50 : -0.008530760605313326, -0.01798963348728355
logistic (50, 50) 0.001 500 : -0.008857330427911236, -0.022258867488457623
logistic (50, 50) 0.001 200 : -0.007780437863778689, -0.01768122257430771
logistic (50, 50) 0.001 100 : -0.009250435676936331, -0.018489869494732414
logistic (50, 50) 0.001 50 : -0.00853098439244837, -0.017990109998594184
logistic (50, 50) 0.01 500 : -0.008854143208930455, -0.022263886807059707
logistic (50, 50) 0.01 200 : -0.007782106874289596, -0.0176829152677207
logistic (50, 50) 0.01 100 : -0.009251963210711579, -0.018071371664022617
logistic (50, 50) 0.01 50 : -0.00875662577265599, -0.017793645303691097
logistic (50, 100) 0.0001 500 : -0.008873610247566877, -0.017686231693154975
logistic (50, 100) 0.0001 200 : -0.008967222896700554, -0.01786160964728789
logistic (50, 100) 0.0001 100 : -0.008539454128988799, -0.018790192775981485
logistic (50, 100) 0.0001 50 : 0.15311021885875475, -0.01718965782302151
logistic (50, 100) 0.001 500 : -0.00887360281123759, -

0.017685224078027195
logistic (50, 100) 0.001 200 : -0.008967316966162153, -0.017861464340313438
logistic (50, 100) 0.001 100 : -0.0085395447553402, -0.018789955065489083
logistic (50, 100) 0.001 50 : 0.1528933323620298, -0.01718914255188797
logistic (50, 100) 0.01 500 : -0.008873092168453578, -0.01767572382442606
logistic (50, 100) 0.01 200 : -0.008968221128389197, -0.01786009129421373
logistic (50, 100) 0.01 100 : -0.008540089126523976, -0.0187876604485977
logistic (50, 100) 0.01 50 : 0.1547042205415754, -0.017182346071401344
logistic (50, 200) 0.0001 500 : -0.009141737427802176, -0.0201105878804555
logistic (50, 200) 0.0001 200 : -0.010442396116802356, 0.8545188331648751
logistic (50, 200) 0.0001 100 : -0.00837935023856744, 0.8688394087378368
logistic (50, 200) 0.0001 50 : 0.8168936091903193, 0.8720448895021795
logistic (50, 200) 0.001 500 : -0.009141732677413205, -0.020110919358281798
logistic (50, 200) 0.001 200 : -0.010442353762042344, 0.8539814253291534
logistic (50, 200) 0.001 100 : -0.008379246353329428, 0.8683140007987302
logistic (50, 200) 0.001 50 : 0.8196788106128705, 0.8720913928739806
logistic (50, 200) 0.01 500 : -0.009141700903413551, -0.02011425352025764
logistic (50, 200) 0.01 200 : -0.01044200559524886, 0.857986544942131
logistic (50, 200) 0.01 100 : -0.008378453647109475, 0.8717349177927679
logistic (50, 200) 0.01 50 : 0.8187114784602085, 0.8743412632885639

Grid search of LightGBM, MACCS
lgb.LGBMRegressor(boosting_type = "gbdt", num_leaves = j,max_depth = 0)
j: accuracy of prediction using cross validation, accuracy of prediction using test data
10 : 0.7989005734031982, 0.8483276920145358
50 : 0.8009956429751608, 0.8611063302977302
100 : 0.8009956429751608, 0.8611063302977302
150 : 0.8009956429751608, 0.8611063302977302

Grid search of SVR, MACCS
SVR(C= c_num, kernel = ker, epsilon = e, gamma = r,degree = 3, coef0=1)
Ker c_num r e: accuracy of prediction using cross validation, accuracy of prediction using test data
linear 1 1 0.1 : 0.7894148199665266, 0.8129787663406082
linear 1 1 0.3 : 0.7899455580154353, 0.8169645325126426
linear 1 1 0.5 : 0.7909718247368811, 0.8177000759160311
linear 1 1 0.7 : 0.7924284236404514, 0.8192901793416294
linear 1 1 0.9 : 0.7929829041384888, 0.8206041902515624
linear 1 2 0.1 : 0.7894148199665266, 0.8129787663406082
linear 1 2 0.3 : 0.7899455580154353, 0.8169645325126426
linear 1 2 0.5 : 0.7909718247368811, 0.8177000759160311
linear 1 2 0.7 : 0.7924284236404514, 0.8192901793416294
linear 1 2 0.9 : 0.7929829041384888, 0.8206041902515624
linear 1 3 0.1 : 0.7894148199665266, 0.8129787663406082
linear 1 3 0.3 : 0.7899455580154353, 0.8169645325126426
linear 1 3 0.5 : 0.7909718247368811, 0.8177000759160311
linear 1 3 0.7 : 0.7924284236404514, 0.8192901793416294
linear 1 3 0.9 : 0.7929829041384888, 0.8206041902515624
linear 1 4 0.1 : 0.7894148199665266, 0.8129787663406082
linear 1 4 0.3 : 0.7899455580154353, 0.8169645325126426
linear 1 4 0.5 : 0.7909718247368811, 0.8177000759160311
linear 1 4 0.7 : 0.7924284236404514, 0.8192901793416294
linear 1 4 0.9 : 0.7929829041384888, 0.8206041902515624
linear 1 5 0.1 : 0.7894148199665266, 0.8129787663406082
linear 1 5 0.3 : 0.7899455580154353, 0.8169645325126426
linear 1 5 0.5 : 0.7909718247368811, 0.8177000759160311
linear 1 5 0.7 : 0.7924284236404514, 0.8192901793416294
linear 1 5 0.9 : 0.7929829041384888, 0.8206041902515624
linear 1 6 0.1 : 0.7894148199665266, 0.8129787663406082
linear 1 6 0.3 : 0.7899455580154353, 0.8169645325126426
linear 1 6 0.5 : 0.7909718247368811, 0.8177000759160311
linear 1 6 0.7 : 0.7924284236404514, 0.8192901793416294
linear 1 6 0.9 : 0.7929829041384888, 0.8206041902515624
linear 2 1 0.1 : 0.8191724718860417, 0.8445854687828537
linear 2 1 0.3 : 0.8204247281324321, 0.8443499714997997
linear 2 1 0.5 : 0.819937144616045, 0.8446765733919072
linear 2 1 0.7 : 0.8196892537539646, 0.8462923138824274
linear 2 1 0.9 : 0.8216496654055196, 0.8461913653379184
linear 2 2 0.1 : 0.8191724718860417, 0.8445854687828537
linear 2 2 0.3 : 0.8204247281324321, 0.8443499714997997
linear 2 2 0.5 : 0.819937144616045, 0.8446765733919072
linear 2 2 0.7 : 0.8196892537539646, 0.8462923138824274
linear 2 2 0.9 : 0.8216496654055196, 0.8461913653379184
linear 2 3 0.1 : 0.8191724718860417, 0.8445854687828537
linear 2 3 0.3 : 0.8204247281324321, 0.8443499714997997
linear 2 3 0.5 : 0.819937144616045, 0.8446765733919072
linear 2 3 0.7 : 0.8196892537539646, 0.8462923138824274
linear 2 3 0.9 : 0.8216496654055196, 0.8461913653379184
linear 2 4 0.1 : 0.8191724718860417, 0.8445854687828537
linear 2 4 0.3 : 0.8204247281324321, 0.8443499714997997
linear 2 4 0.5 : 0.819937144616045, 0.8446765733919072
linear 2 4 0.7 : 0.8196892537539646, 0.8462923138824274
linear 2 4 0.9 : 0.8216496654055196, 0.8461913653379184
linear 2 5 0.1 : 0.8191724718860417, 0.8445854687828537
linear 2 5 0.3 : 0.8204247281324321, 0.8443499714997997
linear 2 5 0.5 : 0.819937144616045, 0.8446765733919072
linear 2 5 0.7 : 0.8196892537539646, 0.8462923138824274
linear 2 5 0.9 : 0.8216496654055196, 0.8461913653379184
linear 2 6 0.1 : 0.8191724718860417, 0.8445854687828537
linear 2 6 0.3 : 0.8204247281324321, 0.8443499714997997
linear 2 6 0.5 : 0.819937144616045, 0.8446765733919072
linear 2 6 0.7 : 0.8196892537539646, 0.8462923138824274
linear 2 6 0.9 : 0.8216496654055196, 0.8461913653379184
linear 3 1 0.1 : 0.830018719956491, 0.852203075046162
linear 3 1 0.3 : 0.8296939576240486, 0.8545681134608959
linear 3 1 0.5 : 0.8284595305826399, 0.8554858231809714
linear 3 1 0.7 : 0.8299954890059729, 0.8559489115504606
linear 3 1 0.9 : 0.8314852162057509, 0.856916236066776
linear 3 2 0.1 : 0.830018719956491, 0.852203075046162
linear 3 2 0.3 : 0.8296939576240486, 0.8545681134608959
linear 3 2 0.5 : 0.8284595305826399, 0.8554858231809714
linear 3 2 0.7 : 0.8299954890059729, 0.8559489115504606
linear 3 2 0.9 : 0.8314852162057509, 0.856916236066776
linear 3 3 0.1 : 0.830018719956491, 0.852203075046162
linear 3 3 0.3 : 0.8296939576240486, 0.8545681134608959
linear 3 3 0.5 : 0.8284595305826399, 0.8554858231809714
linear 3 3 0.7 : 0.8299954890059729, 0.8559489115504606
linear 3 3 0.9 : 0.8314852162057509, 0.856916236066776
linear 3 4 0.1 : 0.830018719956491, 0.852203075046162
linear 3 4 0.3 : 0.8296939576240486, 0.8545681134608959
linear 3 4 0.5 : 0.8284595305826399, 0.8554858231809714
linear 3 4 0.7 : 0.8299954890059729, 0.8559489115504606
linear 3 4 0.9 : 0.8314852162057509, 0.856916236066776
linear 3 5 0.1 : 0.830018719956491, 0.852203075046162
linear 3 5 0.3 : 0.8296939576240486, 0.8545681134608959
linear 3 5 0.5 : 0.8284595305826399, 0.8554858231809714
linear 3 5 0.7 : 0.8299954890059729, 0.8559489115504606
linear 3 5 0.9 : 0.8314852162057509, 0.856916236066776
linear 3 6 0.1 : 0.830018719956491, 0.852203075046162
linear 3 6 0.3 : 0.8296939576240486, 0.8545681134608959
linear 3 6 0.5 : 0.8284595305826399, 0.8554858231809714
linear 3 6 0.7 : 0.8299954890059729, 0.8559489115504606
linear 3 6 0.9 : 0.8314852162057509, 0.856916236066776
linear 4 1 0.1 : 0.8318910120591422, 0.8541987911784463
linear 4 1 0.3 : 0.8332876006230512, 0.8555971060031782
linear 4 1 0.5 : 0.8325783753831526, 0.8578149980287543

linear 4 1 0.7 : 0.8343640076428684, 0.8595838673942121
linear 4 1 0.9 : 0.8364202963729991, 0.8602525224702615
linear 4 2 0.1 : 0.8318910120591422, 0.8541987911784463
linear 4 2 0.3 : 0.8332876006230512, 0.8555971060031782
linear 4 2 0.5 : 0.8325783753831526, 0.8578149980287543
linear 4 2 0.7 : 0.8343640076428684, 0.8595838673942121
linear 4 2 0.9 : 0.8364202963729991, 0.8602525224702615
linear 4 3 0.1 : 0.8318910120591422, 0.8541987911784463
linear 4 3 0.3 : 0.8332876006230512, 0.8555971060031782
linear 4 3 0.5 : 0.8325783753831526, 0.8578149980287543
linear 4 3 0.7 : 0.8343640076428684, 0.8595838673942121
linear 4 3 0.9 : 0.8364202963729991, 0.8602525224702615
linear 4 4 0.1 : 0.8318910120591422, 0.8541987911784463
linear 4 4 0.3 : 0.8332876006230512, 0.8555971060031782
linear 4 4 0.5 : 0.8325783753831526, 0.8578149980287543
linear 4 4 0.7 : 0.8343640076428684, 0.8595838673942121
linear 4 4 0.9 : 0.8364202963729991, 0.8602525224702615
linear 4 5 0.1 : 0.8318910120591422, 0.8541987911784463
linear 4 5 0.3 : 0.8332876006230512, 0.8555971060031782
linear 4 5 0.5 : 0.8325783753831526, 0.8578149980287543
linear 4 5 0.7 : 0.8343640076428684, 0.8595838673942121
linear 4 5 0.9 : 0.8364202963729991, 0.8602525224702615
linear 4 6 0.1 : 0.8318910120591422, 0.8541987911784463
linear 4 6 0.3 : 0.8332876006230512, 0.8555971060031782
linear 4 6 0.5 : 0.8325783753831526, 0.8578149980287543
linear 4 6 0.7 : 0.8343640076428684, 0.8595838673942121
linear 4 6 0.9 : 0.8364202963729991, 0.8602525224702615
poly 1 1 0.1 : 0.8051994347340543, 0.888080773755111
poly 1 1 0.3 : 0.8065988961762539, 0.8877238700492058
poly 1 1 0.5 : 0.8072740013294576, 0.8867373218710776
poly 1 1 0.7 : 0.8077970734765045, 0.8852853726826697
poly 1 1 0.9 : 0.8079921430088252, 0.8838650551499754
poly 1 2 0.1 : 0.8049930850065306, 0.8876911261527101
poly 1 2 0.3 : 0.8063577450410886, 0.8873623935070835
poly 1 2 0.5 : 0.8070326300546193, 0.8863854713757074
poly 1 2 0.7 : 0.8075206028273684, 0.8849016128877369
poly 1 2 0.9 : 0.8076954228862201, 0.883480312071612
poly 1 3 0.1 : 0.8049228397505683, 0.8875603059026138
poly 1 3 0.3 : 0.8062751377928684, 0.8872393189682771
poly 1 3 0.5 : 0.806949559641627, 0.8862661004674799
poly 1 3 0.7 : 0.8074259261961684, 0.8847677936222991
poly 1 3 0.9 : 0.8075932309101805, 0.8833472647678988
poly 1 4 0.1 : 0.8048873376219836, 0.8874942640019665
poly 1 4 0.3 : 0.8062323980619084, 0.8871776548517889
poly 1 4 0.5 : 0.8069072644720237, 0.8862039708951164
poly 1 4 0.7 : 0.8073773662933693, 0.8846995713340968
poly 1 4 0.9 : 0.8075424493589409, 0.8832794651538445
poly 1 5 0.1 : 0.8048662373365069, 0.8874541325472106
poly 1 5 0.3 : 0.8062066788264722, 0.8871398679494599
poly 1 5 0.5 : 0.8068810136556135, 0.8861644889393964
poly 1 5 0.7 : 0.8073500887672755, 0.8846593596286664
poly 1 5 0.9 : 0.8075126751294619, 0.8832396987513546
poly 1 6 0.1 : 0.8048507223509102, 0.8874277467582924
poly 1 6 0.3 : 0.806189811336739, 0.8871148789011818
poly 1 6 0.5 : 0.8068644437794749, 0.8861374679001912
poly 1 6 0.7 : 0.8073304607938512, 0.8846326019296618
poly 1 6 0.9 : 0.8074918840985295, 0.8832135296220393
poly 2 1 0.1 : 0.8051991860028551, 0.8880802434608084
poly 2 1 0.3 : 0.8065993285752132, 0.8877241898037261
poly 2 1 0.5 : 0.807273576363319, 0.8867378011811666
poly 2 1 0.7 : 0.8077972633760006, 0.8852849772054486
poly 2 1 0.9 : 0.807992270052873, 0.8838653299686254
poly 2 2 0.1 : 0.8049929534371323, 0.8876908835154107
poly 2 2 0.3 : 0.8063577374875832, 0.8873624980646475
poly 2 2 0.5 : 0.8070321602666718, 0.8863862052678863
poly 2 2 0.7 : 0.807520782681217, 0.8849027978924862
poly 2 2 0.9 : 0.807695265716589, 0.8834788823965156
poly 2 3 0.1 : 0.8049224367956516, 0.8875602743329581
poly 2 3 0.3 : 0.8062747234764618, 0.8872401045155371
poly 2 3 0.5 : 0.80694941266218, 0.8862666322794269
poly 2 3 0.7 : 0.8074261648199801, 0.8847671735030133
poly 2 3 0.9 : 0.8075939242249346, 0.8833472232118872
poly 2 4 0.1 : 0.8048870910703269, 0.8874940808950005
poly 2 4 0.3 : 0.8062322779538315, 0.8871784370572252
poly 2 4 0.5 : 0.8069071353654733, 0.8862042431681785
poly 2 4 0.7 : 0.8073785014419445, 0.8847004639568704
poly 2 4 0.9 : 0.8075422929707325, 0.8832800116492698
poly 2 5 0.1 : 0.8048659245548269, 0.8874542504331181
poly 2 5 0.3 : 0.8062067827739245, 0.8871391172739358
poly 2 5 0.5 : 0.8068818067359155, 0.8861649884711619
poly 2 5 0.7 : 0.8073493594390019, 0.8846589107981447
poly 2 5 0.9 : 0.8075126826235778, 0.883239451083655
poly 2 6 0.1 : 0.8048514998107456, 0.8874277478125618
poly 2 6 0.3 : 0.8061897239271412, 0.8871147355406308
poly 2 6 0.5 : 0.8068648510561065, 0.8861379560715636
poly 2 6 0.7 : 0.8073304674720501, 0.8846326545052996
poly 2 6 0.9 : 0.8074919131002167, 0.8832131522831353
poly 3 1 0.1 : 0.8051989650307855, 0.8880803990360617
poly 3 1 0.3 : 0.8065984556388777, 0.8877246095147199
poly 3 1 0.5 : 0.8072743048907188, 0.8867378848207215
poly 3 1 0.7 : 0.807797394053846, 0.8852853730582361
poly 3 1 0.9 : 0.8079919317221919, 0.8838651983043838
poly 3 2 0.1 : 0.8049934952433363, 0.8876913127400746
poly 3 2 0.3 : 0.8063578775587501, 0.8873626078122177
poly 3 2 0.5 : 0.8070322170358242, 0.8863865921922859
poly 3 2 0.7 : 0.8075207928671138, 0.8849002991794623
poly 3 2 0.9 : 0.8076943329986129, 0.8834797705676042
poly 3 3 0.1 : 0.8049231139508617, 0.8875599610096269
poly 3 3 0.3 : 0.8062745990872573, 0.8872398198168645
poly 3 3 0.5 : 0.8069493652526661, 0.8862662124064818
poly 3 3 0.7 : 0.8074255613460142, 0.8847676707043803
poly 3 3 0.9 : 0.8075939337295713, 0.883347513198939
poly 3 4 0.1 : 0.8048863750734364, 0.8874944225929605
poly 3 4 0.3 : 0.8062321737235123, 0.8871773087069952
poly 3 4 0.5 : 0.8069076137541835, 0.8862044720496284
poly 3 4 0.7 : 0.8073778475240194, 0.8846994568659164
poly 3 4 0.9 : 0.8075423990967515, 0.8832809261259136
poly 3 5 0.1 : 0.8048652243654242, 0.8874547351387905
poly 3 5 0.3 : 0.8062068921015764, 0.8871390098818845
poly 3 5 0.5 : 0.8068811288922954, 0.886164261145117
poly 3 5 0.7 : 0.8073496026072993, 0.8846592165015762
poly 3 5 0.9 : 0.8075121904096859, 0.8832409049650685
poly 3 6 0.1 : 0.8048508507624655, 0.8874275557599719
poly 3 6 0.3 : 0.8061891285945257, 0.8871151300714101
poly 3 6 0.5 : 0.8068641012147422, 0.8861386157045623
poly 3 6 0.7 : 0.8073311842998159, 0.8846328760577498
poly 3 6 0.9 : 0.8074917814207586, 0.8832144000974604
poly 4 1 0.1 : 0.8051993774934465, 0.8880811241206417
poly 4 1 0.3 : 0.8065989823297912, 0.887723943196628
poly 4 1 0.5 : 0.8072738909946338, 0.8867378848172106
poly 4 1 0.7 : 0.8077966443009454, 0.8852859389234675
poly 4 1 0.9 : 0.807992000762991, 0.8838658028892676
poly 4 2 0.1 : 0.8049933184099345, 0.8876913128105486
poly 4 2 0.3 : 0.8063579708511801, 0.8873629633588824
poly 4 2 0.5 : 0.8070317304375051, 0.8863856277754121
poly 4 2 0.7 : 0.8075208596471445, 0.8849015639555863
poly 4 2 0.9 : 0.8076941486994444, 0.8834797706055202
poly 4 3 0.1 : 0.8049232389548804, 0.8875602301524218
poly 4 3 0.3 : 0.8062740224526859, 0.8872397197170439
poly 4 3 0.5 : 0.8069496678287307, 0.8862663566562786
poly 4 3 0.7 : 0.80742597055338, 0.8847673157572284
poly 4 3 0.9 : 0.8075942196090462, 0.8833474349577848
poly 4 4 0.1 : 0.8048873904319581, 0.8874936206577925
poly 4 4 0.3 : 0.8062321720073452, 0.8871773746632452
poly 4 4 0.5 : 0.8069068383468905, 0.8862049878874487
poly 4 4 0.7 : 0.8073782761342285, 0.8847007730405395
poly 4 4 0.9 : 0.8075426914827858, 0.8832800053294511
poly 4 5 0.1 : 0.8048654192494841, 0.8874539292777022
poly 4 5 0.3 : 0.8062073740287194, 0.8871402403170217
poly 4 5 0.5 : 0.8068822335732854, 0.8861640305255079
poly 4 5 0.7 : 0.8073493452607238, 0.8846592144479685
poly 4 5 0.9 : 0.807512232952781, 0.8832410484757717
poly 4 6 0.1 : 0.8048512647906152, 0.8874280889258722
poly 4 6 0.3 : 0.806189839028652, 0.8871147269112125
poly 4 6 0.5 : 0.8068644451092307, 0.8861383812867881
poly 4 6 0.7 : 0.8073308226540595, 0.8846321922558898
poly 4 6 0.9 : 0.8074918306425634, 0.8832142724978402
rbf 1 1 0.1 : 0.01159242916249601, 0.052159513229659615

rbf 1 1 0.3 : 0.011833330655033846, 0.052155823008875446
rbf 1 1 0.5 : 0.012823118979450454, 0.05210550851728124
rbf 1 1 0.7 : 0.014113806410476548, 0.05208916812553621
rbf 1 1 0.9 : 0.015540379010733884, 0.052079842903100304
rbf 1 2 0.1 : 0.006701780394683299, 0.04416580779702117
rbf 1 2 0.3 : 0.006826927787711701, 0.04415822546374348
rbf 1 2 0.5 : 0.00788296749393096, 0.04409267365406644
rbf 1 2 0.7 : 0.00913036472191504, 0.044068533771681295
rbf 1 2 0.9 : 0.010627043308740137, 0.04405716203431642
rbf 1 3 0.1 : 0.00615942825865543, 0.043325328907168004
rbf 1 3 0.3 : 0.006269443747379699, 0.0433176907836057
rbf 1 3 0.5 : 0.007342553976457578, 0.04324810884869412
rbf 1 3 0.7 : 0.008584206825445717, 0.043223359350452406
rbf 1 3 0.9 : 0.010091029279285225, 0.04320999740981657
rbf 1 4 0.1 : 0.0060520024534820305, 0.04320286362270076
rbf 1 4 0.3 : 0.006159806184027006, 0.04319522106681195
rbf 1 4 0.5 : 0.007235532503262809, 0.043125029830289985
rbf 1 4 0.7 : 0.008476552669913717, 0.04310023543059027
rbf 1 4 0.9 : 0.009984744525529133, 0.04308657835833718
rbf 1 5 0.1 : 0.006023316349065899, 0.04318100514818657
rbf 1 5 0.3 : 0.006130951875919011, 0.04317336195454324
rbf 1 5 0.5 : 0.007206794977983622, 0.04310308713170019
rbf 1 5 0.7 : 0.00844773573564459, 0.043078286793091
rbf 1 5 0.9 : 0.009956177160672897, 0.043064590431194594
rbf 1 6 0.1 : 0.006014175424674084, 0.04317603813019788
rbf 1 6 0.3 : 0.006121789539329825, 0.04316839484892743
rbf 1 6 0.5 : 0.007197667114362804, 0.04309810885538068
rbf 1 6 0.7 : 0.008438561900789, 0.04307330752959315
rbf 1 6 0.9 : 0.009947073568035814, 0.04305960588345248
rbf 2 1 0.1 : 0.05317794102911644, 0.09511127237717709
rbf 2 1 0.3 : 0.05424215620378407, 0.09491650718273226
rbf 2 1 0.5 : 0.05536488307191321, 0.09466292629850015
rbf 2 1 0.7 : 0.05674353932535507, 0.09431958036860477
rbf 2 1 0.9 : 0.05807531400300485, 0.09385614709318135
rbf 2 2 0.1 : 0.04379706371255483, 0.08049336670353013
rbf 2 2 0.3 : 0.0448553869074968, 0.08032532431838446
rbf 2 2 0.5 : 0.046025420109295466, 0.08010418896352056
rbf 2 2 0.7 : 0.04741800462288244, 0.07978159257440665
rbf 2 2 0.9 : 0.04876244400129368, 0.07935667169104821
rbf 2 3 0.1 : 0.042761368464605896, 0.07891688794769158
rbf 2 3 0.3 : 0.043818055900522325, 0.07874515578162411
rbf 2 3 0.5 : 0.044972234863088545, 0.07852257189083012
rbf 2 3 0.7 : 0.04636957115832248, 0.07819453831843282
rbf 2 3 0.9 : 0.04771468201344964, 0.07777338797307287
rbf 2 4 0.1 : 0.04255308129915987, 0.078684991165889
rbf 2 4 0.3 : 0.04361044208277378, 0.07851275343323383
rbf 2 4 0.5 : 0.04476139675900115, 0.07828997407045168
rbf 2 4 0.7 : 0.04615436856542594, 0.07796122320672538
rbf 2 4 0.9 : 0.04749983917391385, 0.07753937778212971
rbf 2 5 0.1 : 0.04249655078028369, 0.07864300978300776
rbf 2 5 0.3 : 0.04355430202889763, 0.0784707032682711
rbf 2 5 0.5 : 0.044705012217221785, 0.07824789706491975
rbf 2 5 0.7 : 0.0460953791657613, 0.0779190486511745
rbf 2 5 0.9 : 0.047440868218620105, 0.07749711154597216
rbf 2 6 0.1 : 0.04247837507094678, 0.07863330967525195
rbf 2 6 0.3 : 0.043535859898939155, 0.07846099383263339
rbf 2 6 0.5 : 0.04468682867388398, 0.07823818397607507
rbf 2 6 0.7 : 0.04607636099476331, 0.07790932242005022
rbf 2 6 0.9 : 0.04742162989854024, 0.0774874656136949
rbf 3 1 0.1 : 0.08938826342605125, 0.1273320577219571
rbf 3 1 0.3 : 0.09035204084183839, 0.1267113543879328
rbf 3 1 0.5 : 0.09135294979354687, 0.1260369939120729
rbf 3 1 0.7 : 0.09243227707218463, 0.1252285647611896
rbf 3 1 0.9 : 0.0935274049227615, 0.1243409884417137
rbf 3 2 0.1 : 0.0764889671279085, 0.10724196761016458
rbf 3 2 0.3 : 0.07742678293989189, 0.10670058420166861
rbf 3 2 0.5 : 0.07842563932186139, 0.10608753266066184
rbf 3 2 0.7 : 0.07944943749150093, 0.10550616967834514
rbf 3 2 0.9 : 0.08051289420959058, 0.1048175585957114
rbf 3 3 0.1 : 0.07501407028379559, 0.10503256585943743
rbf 3 3 0.3 : 0.07593835335136075, 0.1044836565075753
rbf 3 3 0.5 : 0.07691365960583323, 0.1039515647816236
rbf 3 3 0.7 : 0.07793415572241484, 0.10340585139204672
rbf 3 3 0.9 : 0.07898905435531212, 0.10272702598650751
rbf 3 4 0.1 : 0.07470480974051938, 0.10470604699851649
rbf 3 4 0.3 : 0.0756265231152761, 0.10415319804605816
rbf 3 4 0.5 : 0.0766022642083386, 0.10363547551938712
rbf 3 4 0.7 : 0.07762130031887193, 0.10309372297520125
rbf 3 4 0.9 : 0.07867634366068177, 0.10242209909206368
rbf 3 5 0.1 : 0.07461808895479236, 0.10464608028696099
rbf 3 5 0.3 : 0.07553960082688707, 0.10409302651500163
rbf 3 5 0.5 : 0.07651579552420355, 0.10357557420235164
rbf 3 5 0.7 : 0.07753482486705791, 0.10303755439128093
rbf 3 5 0.9 : 0.07859010635033659, 0.1023676585077673
rbf 3 6 0.1 : 0.07459004182238334, 0.1046320349749158
rbf 3 6 0.3 : 0.0755112292594827, 0.10407875192900051
rbf 3 6 0.5 : 0.0764874730091609, 0.10356135980185044
rbf 3 6 0.7 : 0.07750687718576091, 0.10302476916759451
rbf 3 6 0.9 : 0.07856210030246333, 0.10235518049762737
rbf 4 1 0.1 : 0.11853525476190722, 0.15269361585411412
rbf 4 1 0.3 : 0.11924814398119192, 0.15225130827598377
rbf 4 1 0.5 : 0.11989315953449467, 0.1517133238483147
rbf 4 1 0.7 : 0.12057858699934343, 0.15087226995752634
rbf 4 1 0.9 : 0.12123754411194139, 0.14992854396892652
rbf 4 2 0.1 : 0.10236121972448256, 0.1285759064464579
rbf 4 2 0.3 : 0.10305780750613462, 0.12840894025790706
rbf 4 2 0.5 : 0.1037379943070859, 0.12813429317207303
rbf 4 2 0.7 : 0.10442384620434993, 0.12759675578786722
rbf 4 2 0.9 : 0.10523930723245886, 0.1269480323933252
rbf 4 3 0.1 : 0.10044358885417841, 0.12593071577321358
rbf 4 3 0.3 : 0.10114111628119929, 0.12580039800450304
rbf 4 3 0.5 : 0.10181741353686047, 0.1255506954807779
rbf 4 3 0.7 : 0.10250469678969412, 0.1250231998925324
rbf 4 3 0.9 : 0.10333797621677347, 0.1243574782999125
rbf 4 4 0.1 : 0.10003964405921413, 0.12554113405340206
rbf 4 4 0.3 : 0.10074332970250119, 0.1254114711176071
rbf 4 4 0.5 : 0.10141984790941379, 0.1251688014047283
rbf 4 4 0.7 : 0.10210393267436338, 0.12464400002617004
rbf 4 4 0.9 : 0.10293892207395561, 0.12397779718649349
rbf 4 5 0.1 : 0.09992644744943983, 0.12547040630565665
rbf 4 5 0.3 : 0.10063109154496544, 0.1253405037974049
rbf 4 5 0.5 : 0.10130986363938163, 0.12509878263221297
rbf 4 5 0.7 : 0.10199219839368354, 0.12457459090802048
rbf 4 5 0.9 : 0.10282761295139711, 0.12390861780934159
rbf 4 6 0.1 : 0.09988982373725608, 0.12545362662033965
rbf 4 6 0.3 : 0.10059441636540689, 0.1253241731338094
rbf 4 6 0.5 : 0.10127393449921099, 0.12508175224055273
rbf 4 6 0.7 : 0.10195551190481049, 0.12455816906158235
rbf 4 6 0.9 : 0.10279156382268853, 0.12389199880275426
sigmoid 1 1 0.1 : -0.027851574945407975, -0.0004817589189465288
sigmoid 1 1 0.3 : -0.028038637425741884, -
0.00015273486594669095
sigmoid 1 1 0.5 : -0.0293276829305003, -8.393840856868451e-05
sigmoid 1 1 0.7 : -0.02737078105644315, -0.000152734865946913
sigmoid 1 1 0.9 : -0.02742593928300203, -0.0003516451211358351
sigmoid 1 2 0.1 : -0.02785157494475108, -0.0004817589182650739
sigmoid 1 2 0.3 : -0.028038637425086766, -0.0001527348652718974
sigmoid 1 2 0.5 : -0.029327682929847977, -8.39384078961114e-05
sigmoid 1 2 0.7 : -0.027370781055785543, -0.0001527348652718974
sigmoid 1 2 0.9 : -0.02742593928234638, -0.0003516451204561566
sigmoid 1 3 0.1 : -0.02785157494475108, -0.0004817589182650739
sigmoid 1 3 0.3 : -0.028038637425086766, -0.0001527348652718974
sigmoid 1 3 0.5 : -0.029327682929847977, -8.39384078961114e-05
sigmoid 1 3 0.7 : -0.027370781055785543, -0.0001527348652718974
sigmoid 1 3 0.9 : -0.02742593928234638, -0.0003516451204561566
sigmoid 1 4 0.1 : -0.02785157494475108, -0.0004817589182650739
sigmoid 1 4 0.3 : -0.028038637425086766, -0.0001527348652718974
sigmoid 1 4 0.5 : -0.029327682929847977, -8.39384078961114e-05
sigmoid 1 4 0.7 : -0.027370781055785543, -0.0001527348652718974
sigmoid 1 4 0.9 : -0.02742593928234638, -0.0003516451204561566
sigmoid 1 5 0.1 : -0.02785157494475108, -0.0004817589182650739
sigmoid 1 5 0.3 : -0.028038637425086766, -0.0001527348652718974
sigmoid 1 5 0.5 : -0.029327682929847977, -8.39384078961114e-05
sigmoid 1 5 0.7 : -0.027370781055785543, -0.0001527348652718974
sigmoid 1 5 0.9 : -0.02742593928234638, -0.0003516451204561566
sigmoid 1 6 0.1 : -0.02785157494475108, -0.0004817589182650739
sigmoid 1 6 0.3 : -0.028038637425086766, -0.0001527348652718974
sigmoid 1 6 0.5 : -0.029327682929847977, -8.39384078961114e-05

sigmoid 1 6 0.7 : -0.027370781055785543, -0.0001527348652718974
sigmoid 1 6 0.9 : -0.02742593928234638, -0.0003516451204561566
sigmoid 2 1 0.1 : -0.027851574946064873, -
0.00048175891962798367
sigmoid 2 1 0.3 : -0.02803863742639705, -0.0001527348666214845
sigmoid 2 1 0.5 : -0.02932768293115257 6, -8.393840924125762e-05
sigmoid 2 1 0.7 : -0.027370781057100935, -
0.00015273486662170654
sigmoid 2 1 0.9 : -0.02742593928365795, -0.0003516451218152916
sigmoid 2 2 0.1 : -0.02785157494475108, -0.0004817589182650739
sigmoid 2 2 0.3 : -0.028038637425086766, -0.0001527348652718974
sigmoid 2 2 0.5 : -0.029327682929847977, -8.39384078961114e-05
sigmoid 2 2 0.7 : -0.027370781055785543, -0.0001527348652718974
sigmoid 2 2 0.9 : -0.02742593928234638, -0.0003516451204561566
sigmoid 2 3 0.1 : -0.02785157494475108, -0.0004817589182650739
sigmoid 2 3 0.3 : -0.028038637425086766, -0.0001527348652718974
sigmoid 2 3 0.5 : -0.029327682929847977, -8.39384078961114e-05
sigmoid 2 3 0.7 : -0.027370781055785543, -0.0001527348652718974
sigmoid 2 3 0.9 : -0.02742593928234638, -0.0003516451204561566
sigmoid 2 4 0.1 : -0.02785157494475108, -0.0004817589182650739
sigmoid 2 4 0.3 : -0.028038637425086766, -0.0001527348652718974
sigmoid 2 4 0.5 : -0.029327682929847977, -8.39384078961114e-05
sigmoid 2 4 0.7 : -0.027370781055785543, -0.0001527348652718974
sigmoid 2 4 0.9 : -0.02742593928234638, -0.0003516451204561566
sigmoid 2 5 0.1 : -0.02785157494475108, -0.0004817589182650739
sigmoid 2 5 0.3 : -0.028038637425086766, -0.0001527348652718974
sigmoid 2 5 0.5 : -0.029327682929847977, -8.39384078961114e-05
sigmoid 2 5 0.7 : -0.027370781055785543, -0.0001527348652718974
sigmoid 2 5 0.9 : -0.02742593928234638, -0.0003516451204561566
sigmoid 2 6 0.1 : -0.02785157494475108, -0.0004817589182650739
sigmoid 2 6 0.3 : -0.028038637425086766, -0.0001527348652718974
sigmoid 2 6 0.5 : -0.029327682929847977, -8.39384078961114e-05
sigmoid 2 6 0.7 : -0.027370781055785543, -0.0001527348652718974
sigmoid 2 6 0.9 : -0.02742593928234638, -0.0003516451204561566
sigmoid 3 1 0.1 : -0.027851574946721726, -0.0004817589203092165
sigmoid 3 1 0.3 : -0.02803863742705217, -0.00015273486729627805
sigmoid 3 1 0.5 : -0.029327682931804943, -8.393840991360868e-05
sigmoid 3 1 0.7 : -0.027370781057758718, -
0.00015273486729627805
sigmoid 3 1 0.9 : -0.027425939284313826, -0.000351645122494304
sigmoid 3 2 0.1 : -0.02785157494475108, -0.0004817589182650739
sigmoid 3 2 0.3 : -0.028038637425086766, -0.0001527348652718974
sigmoid 3 2 0.5 : -0.029327682929847977, -8.39384078961114e-05
sigmoid 3 2 0.7 : -0.027370781055785543, -0.0001527348652718974
sigmoid 3 2 0.9 : -0.02742593928234638, -0.0003516451204561566
sigmoid 3 3 0.1 : -0.02785157494475108, -0.0004817589182650739
sigmoid 3 3 0.3 : -0.028038637425086766, -0.0001527348652718974
sigmoid 3 3 0.5 : -0.029327682929847977, -8.39384078961114e-05
sigmoid 3 3 0.7 : -0.027370781055785543, -0.0001527348652718974
sigmoid 3 3 0.9 : -0.02742593928234638, -0.0003516451204561566
sigmoid 3 4 0.1 : -0.02785157494475108, -0.0004817589182650739
sigmoid 3 4 0.3 : -0.028038637425086766, -0.0001527348652718974
sigmoid 3 4 0.5 : -0.029327682929847977, -8.39384078961114e-05
sigmoid 3 4 0.7 : -0.027370781055785543, -0.0001527348652718974
sigmoid 3 4 0.9 : -0.02742593928234638, -0.0003516451204561566
sigmoid 3 5 0.1 : -0.02785157494475108, -0.0004817589182650739
sigmoid 3 5 0.3 : -0.028038637425086766, -0.0001527348652718974
sigmoid 3 5 0.5 : -0.029327682929847977, -8.39384078961114e-05
sigmoid 3 5 0.7 : -0.027370781055785543, -0.0001527348652718974
sigmoid 3 5 0.9 : -0.02742593928234638, -0.0003516451204561566
sigmoid 3 6 0.1 : -0.02785157494475108, -0.0004817589182650739
sigmoid 3 6 0.3 : -0.028038637425086766, -0.0001527348652718974
sigmoid 3 6 0.5 : -0.029327682929847977, -8.39384078961114e-05
sigmoid 3 6 0.7 : -0.027370781055785543, -0.0001527348652718974
sigmoid 3 6 0.9 : -0.02742593928234638, -0.0003516451204561566
sigmoid 4 1 0.1 : -0.027851574947378888, -
0.00048175892099089346
sigmoid 4 1 0.3 : -0.028038637427707423, -0.0001527348679710716
sigmoid 4 1 0.5 : -0.02932768293245731, -8.393841058640383e-05
sigmoid 4 1 0.7 : -0.027370781058416505, -
0.00015273486797173774
sigmoid 4 1 0.9 : -0.027425939284969614, -
0.00035164512317398255
sigmoid 4 2 0.1 : -0.02785157494475108, -0.0004817589182650739
sigmoid 4 2 0.3 : -0.028038637425086766, -0.0001527348652718974
sigmoid 4 2 0.5 : -0.029327682929847977, -8.39384078961114e-05
sigmoid 4 2 0.7 : -0.027370781055785543, -0.0001527348652718974
sigmoid 4 2 0.9 : -0.02742593928234638, -0.0003516451204561566
sigmoid 4 3 0.1 : -0.02785157494475108, -0.0004817589182650739
sigmoid 4 3 0.3 : -0.028038637425086766, -0.0001527348652718974
sigmoid 4 3 0.5 : -0.029327682929847977, -8.39384078961114e-05
sigmoid 4 3 0.7 : -0.027370781055785543, -0.0001527348652718974
sigmoid 4 3 0.9 : -0.02742593928234638, -0.0003516451204561566
sigmoid 4 4 0.1 : -0.02785157494475108, -0.0004817589182650739
sigmoid 4 4 0.3 : -0.028038637425086766, -0.0001527348652718974
sigmoid 4 4 0.5 : -0.029327682929847977, -8.39384078961114e-05
sigmoid 4 4 0.7 : -0.027370781055785543, -0.0001527348652718974
sigmoid 4 4 0.9 : -0.02742593928234638, -0.0003516451204561566
sigmoid 4 5 0.1 : -0.02785157494475108, -0.0004817589182650739
sigmoid 4 5 0.3 : -0.028038637425086766, -0.0001527348652718974
sigmoid 4 5 0.5 : -0.029327682929847977, -8.39384078961114e-05
sigmoid 4 5 0.7 : -0.027370781055785543, -0.0001527348652718974
sigmoid 4 5 0.9 : -0.02742593928234638, -0.0003516451204561566
sigmoid 4 6 0.1 : -0.02785157494475108, -0.0004817589182650739
sigmoid 4 6 0.3 : -0.028038637425086766, -0.0001527348652718974
sigmoid 4 6 0.5 : -0.029327682929847977, -8.39384078961114e-05
sigmoid 4 6 0.7 : -0.027370781055785543, -0.0001527348652718974
sigmoid 4 6 0.9 : -0.02742593928234638, -0.0003516451204561566

3. Results of analysis with RMSE

RMSE	EN	SVR	NN (relu)	NN (logistic)	RF	LGBM
MACCS	5.24	5.30	4.51	4.63	6.02	5.60
Topological	3.92	3.83	3.85	3.92	6.51	5.54
Morgan (r =4)	3.60	3.83	4.92	5.77	5.93	5.73
Morgan (r =3)	3.31	3.49	4.41	3.83	5.92	5.79
Morgan (r =2)	2.88	2.93	3.68	3.14	5.77	5.80
Avalon	2.71	2.74	2.98	3.50	5.97	4.73

Color scale: 7.0, 5, 2.5

Heat map of accuracy of the prediction by various machine learning algorithm and fingerprints.

RMSE

A	EN	SVR	NN_r	NN_l
MACCS	7.74	8.14	7.48	7.23
Topological	7.76	8.12	7.62	9.68
Morgan (r =2)	3.89	4.15	5.18	4.77
Avalon	3.41	3.70	3.69	4.92

B	EN	SVR	NN_r	NN_l
MACCS	9.41	9.36	7.86	7.44
Topological	9.37	9.43	11.49	9.27
Morgan (r =2)	5.39	5.52	9.49	5.60
Avalon	5.78	5.94	6.75	6.72

C	EN	SVR	NN_r	NN_l
MACCS	13.14	12.70	12.50	11.37
Topological	12.42	12.83	13.09	12.16
Morgan (r =2)	10.03	10.37	10.98	10.47
Avalon	6.09	6.67	7.29	8.11

D	EN	SVR	NN_r	NN_l
MACCS	13.42	12.03	12.31	11.88
Topological	13.18	13.59	14.75	14.89
Morgan (r =2)	10.69	11.02	13.07	11.55
Avalon	7.47	8.18	8.40	9.34

Color scale: 15, 13, 10, 7.5, 5.0, 3.0

Heat maps of the result (RMSE) of AD

4. List of SMILES and BDE$_{DFT}$

(1) Fig2_716HVIs

O=C(O[I]1OOC(C)(C)C)C2=C1C=CC=C2, [O]OC(C)(C)C, O=C(O[I]1)C2=C1C=CC=C2, 19.6
O=C(O[I]3N=[N+]=[N-])C4=C3C=CC=C4, [N]=[N+]=[N-], O=C(O[I]1)C2=C1C=CC=C2, 23.7
O=C(O[I]5C(F)(F)F)C6=C5C=CC=C6, F[C](F)F, O=C(O[I]1)C2=C1C=CC=C2, 31.4
Br[I]7OC(C8=C7C=CC=C8)=O, [Br], O=C(O[I]1)C2=C1C=CC=C2, 37.2
O=C(O[I]9OC(C)=O)C%10=C9C=CC=C%10, [O]C(C)=O, O=C(O[I]1)C2=C1C=CC=C2, 37.9
O=C(O[I]%11OC(C(F)(F)F)=O)C%12=C%11C=CC=C%12, [O]C(C(F)(F)F)=O,
O=C(O[I]1)C2=C1C=CC=C2, 46.8
O[I]%13OC(C%14=C%13C=CC=C%14)=O, [OH], O=C(O[I]1)C2=C1C=CC=C2, 47.6
O=C(O[I]%15OC(C(C%16=CC=CC=C%16)=O)=O)C%17=C%15C=CC=C%17,
[O]C(C(C1=CC=CC=C1)=O)=O, O=C(O[I]1)C2=C1C=CC=C2, 47.7
Cl[I]1OC(C2=C1C=CC=C2)=O, [Cl], O=C(O[I]1)C2=C1C=CC=C2, 47.7
O=C(O[I]3N4C(C(C=CC=C5)=C5C4=O)=O)C6=C3C=CC=C6, O=C1C2=C(C=CC=C2)C([N]1)=O,
O=C(O[I]1)C2=C1C=CC=C2, 54.1
O=C(O[I]7C#N)C8=C7C=CC=C8, [C]#N, O=C(O[I]1)C2=C1C=CC=C2, 65.4
O=C(O[I]9C#C[Si](C(C)C)(C(C)C)C(C)C)C%10=C9C=CC=C%10, [C]#C[Si](C(C)C)(C(C)C)C(C)C,
O=C(O[I]1)C2=C1C=CC=C2, 67.6
F[I]%11OC(C%12=C%11C=CC=C%12)=O, [F], O=C(O[I]1)C2=C1C=CC=C2, 69.8
CC(O[I]1OOC(C)(C)C)(C)C2=C1C=CC=C2, [O]OC(C)(C)C, CC(O[I]1)(C)C2=C1C=CC=C2, 15.7
CC(O[I]3N=[N+]=[N-])(C)C4=C3C=CC=C4, [N]=[N+]=[N-], CC(O[I]1)(C)C2=C1C=CC=C2, 20.8
CC(O[I]5C(F)(F)F)(C)C6=C5C=CC=C6, F[C](F)F, CC(O[I]1)(C)C2=C1C=CC=C2, 24.8
Br[I]7OC(C)(C)C8=C7C=CC=C8, [Br], CC(O[I]1)(C)C2=C1C=CC=C2, 35.7
CC(O[I]9OC(C)=O)(C)C%10=C9C=CC=C%10, [O]C(C)=O, CC(O[I]1)(C)C2=C1C=CC=C2, 36.7
CC(O[I]%11OC(C(F)(F)F)=O)(C)C%12=C%11C=CC=C%12, [O]C(C(F)(F)F)=O,
CC(O[I]1)(C)C2=C1C=CC=C2, 48.7
O[I]%13OC(C)(C)C%14=C%13C=CC=C%14, [OH], CC(O[I]1)(C)C2=C1C=CC=C2, 43.0
CC(O[I]%15OC(C(C%16=CC=CC=C%16)=O)=O)(C)C%17=C%15C=CC=C%17,
[O]C(C(C1=CC=CC=C1)=O)=O, CC(O[I]1)(C)C2=C1C=CC=C2, 44.1
Cl[I]1OC(C)(C)C2=C1C=CC=C2, [Cl], CC(O[I]1)(C)C2=C1C=CC=C2, 46.4
CC(O[I]3N4C(C(C=CC=C5)=C5C4=O)=O)(C)C6=C3C=CC=C6, O=C1C2=C(C=CC=C2)C([N]1)=O,
CC(O[I]1)(C)C2=C1C=CC=C2, 50.7
CC(O[I]7C#N)(C)C8=C7C=CC=C8, [C]#N, CC(O[I]1)(C)C2=C1C=CC=C2, 61.6
CC(O[I]9C#C[Si](C(C)C)(C(C)C)C(C)C)(C)C%10=C9C=CC=C%10, [C]#C[Si](C(C)C)(C(C)C)C(C)C,
CC(O[I]1)(C)C2=C1C=CC=C2, 59.2
F[I]%11OC(C)(C)C%12=C%11C=CC=C%12, [F], CC(O[I]1)(C)C2=C1C=CC=C2, 68.2
O=C1C2=C([I](OOC(C)(C)C)N1S(C3=CC=C(C=C3)C)(=O)=O)C=CC=C2, [O]OC(C)(C)C,
O=C1C2=C([I]N1S(C3=CC=C(C=C3)C)(=O)=O)C=CC=C2, 22.7
O=C4C5=C([I](N=[N+]=[N-])N4S(C6=CC=C(C=C6)C)(=O)=O)C=CC=C5, [N]=[N+]=[N-],
O=C1C2=C([I]N1S(C3=CC=C(C=C3)C)(=O)=O)C=CC=C2, 27.7
O=C7C8=C([I](C(F)(F)F)N7S(C9=CC=C(C=C9)C)(=O)=O)C=CC=C8, F[C](F)F,
O=C1C2=C([I]N1S(C3=CC=C(C=C3)C)(=O)=O)C=CC=C2, 33.7
Br[I](C%10=C(C%11=O)C=CC=C%10)N%11S(C%12=CC=C(C=C%12)C)(=O)=O, [Br],
O=C1C2=C([I]N1S(C3=CC=C(C=C3)C)(=O)=O)C=CC=C2, 41.6
O=C%13C%14=C([I](OC(C)=O)N%13S(C%15=CC=C(C=C%15)C)(=O)=O)C=CC=C%14,
[O]C(C)=O, O=C1C2=C([I]N1S(C3=CC=C(C=C3)C)(=O)=O)C=CC=C2, 42.7
O=C%16C%17=C([I](OC(C(F)(F)F)=O)N%16S(C%18=CC=C(C=C%18)C)(=O)=O)C=CC=C%17,
[O]C(C(F)(F)F)=O, O=C1C2=C([I]N1S(C3=CC=C(C=C3)C)(=O)=O)C=CC=C2, 52.4
O[I](C%19=C(C%20=O)C=CC=C%19)N%20S(C%21=CC=C(C=C%21)C)(=O)=O, [OH],

O=C1C2=C([I]N1S(C3=CC=C(C=C3)C)(=O)=O)C=CC=C2, 51.7
O=C%22C%23=C([I](OC(C(C%24=CC=CC=C%24)=O)=O)N%22S(C%25=CC=C(C=C%25)C)(=O)=O)C=CC=C%23, [O]C(C(C1=CC=CC=C1)=O)=O,
O=C1C2=C([I]N1S(C3=CC=C(C=C3)C)(=O)=O)C=CC=C2, 54.8
Cl[I](C1=C(C2=O)C=CC=C1)N2S(C3=CC=C(C=C3)C)(=O)=O, [Cl],
O=C1C2=C([I]N1S(C3=CC=C(C=C3)C)(=O)=O)C=CC=C2, 52.3
O=C4C5=C([I](N6C(C(C=CC=C7)=C7C6=O)=O)N4S(C8=CC=C(C=C8)C)(=O)=O)C=CC=C5,
O=C1C2=C(C=CC=C2)C([N]1)=O, O=C1C2=C([I]N1S(C3=CC=C(C=C3)C)(=O)=O)C=CC=C2, 57.4
O=C9C%10=C([I](C#N)N9S(C%11=CC=C(C=C%11)C)(=O)=O)C=CC=C%10, [C]#N,
O=C1C2=C([I]N1S(C3=CC=C(C=C3)C)(=O)=O)C=CC=C2, 68.3
O=C%12C%13=C([I](C#C[Si](C(C)C)(C(C)C)C(C)C)N%12S(C%14=CC=C(C=C%14)C)(=O)=O)C=CC=C%13, [C]#C[Si](C(C)C)(C(C)C)C(C)C,
O=C1C2=C([I]N1S(C3=CC=C(C=C3)C)(=O)=O)C=CC=C2, 69.9
F[I](C%15=C(C%16=O)C=CC=C%15)N%16S(C%17=CC=C(C=C%17)C)(=O)=O, [F],
O=C1C2=C([I]N1S(C3=CC=C(C=C3)C)(=O)=O)C=CC=C2, 75.1
O=S(C1=C2C=CC=C1)(O[I]2OOC(C)(C)C)=O, [O]OC(C)(C)C, O=S(C1=C2C=CC=C1)(O[I]2)=O, 21.0
O=S(C3=C4C=CC=C3)(O[I]4N=[N+]=[N-])=O, [N]=[N+]=[N-], O=S(C1=C2C=CC=C1)(O[I]2)=O, 26.1
O=S(C5=C6C=CC=C5)(O[I]6C(F)(F)F)=O, F[C](F)F, O=S(C1=C2C=CC=C1)(O[I]2)=O, 36.7
Br[I]7OS(C8=C7C=CC=C8)(=O)=O, [Br], O=S(C1=C2C=CC=C1)(O[I]2)=O, 38.2
O=S(C9=C%10C=CC=C9)(O[I]%10OC(C)=O)=O, [O]C(C)=O, O=S(C1=C2C=CC=C1)(O[I]2)=O, 40.0
O=C(C(F)(F)F)O[I]%11OS(C%12=C%11C=CC=C%12)(=O)=O, [O]C(C(F)(F)F)=O,
O=S(C1=C2C=CC=C1)(O[I]2)=O, 46.2
O[I]%13OS(C%14=C%13C=CC=C%14)(=O)=O, [OH], O=S(C1=C2C=CC=C1)(O[I]2)=O, 50.8
O=C(C(C%15=CC=CC=C%15)=O)O[I]%16OS(C%17=C%16C=CC=C%17)(=O)=O,
[O]C(C(C1=CC=CC=C1)=O)=O, O=S(C1=C2C=CC=C1)(O[I]2)=O, 45.0
Cl[I]1OS(C2=C1C=CC=C2)(=O)=O, [Cl], O=S(C1=C2C=CC=C1)(O[I]2)=O, 48.2
O=C3C4=C(C=CC=C4)C(N3[I]5OS(C6=C5C=CC=C6)(=O)=O)=O,
O=C1C2=C(C=CC=C2)C([N]1)=O, O=S(C1=C2C=CC=C1)(O[I]2)=O, 56.3
O=S(C7=C8C=CC=C7)(O[I]8C#N)=O, [C]#N, O=S(C1=C2C=CC=C1)(O[I]2)=O, 68.1
O=S(C9=C%10C=CC=C9)(O[I]%10C#C[Si](C(C)C)(C(C)C)C(C)C)=O,
[C]#C[Si](C(C)C)(C(C)C)C(C)C, O=S(C1=C2C=CC=C1)(O[I]2)=O, 73.8
F[I]%11OS(C%12=C%11C=CC=C%12)(=O)=O, [F], O=S(C1=C2C=CC=C1)(O[I]2)=O, 70.2
O=C1C2=C([I](N=[N+]=[N-])O1)C=CC(OC)=C2, [N]=[N+]=[N-], O=C1C2=C([I]O1)C=CC(OC)=C2, 24.0
O=C3C4=C([I](N=[N+]=[N-])O3)C=CC(C(C)(C)C)=C4, [N]=[N+]=[N-],
O=C1C2=C([I]O1)C=CC(C(C)(C)C)=C2, 23.7
O=C5C6=C([I](N=[N+]=[N-])O5)C=CC(C)=C6, [N]=[N+]=[N-], O=C1C2=C([I]O1)C=CC(C)=C2, 23.8
O=C7C8=C([I](N=[N+]=[N-])O7)C=CC(C(C)C)=C8, [N]=[N+]=[N-],
O=C1C2=C([I]O1)C=CC(C(C)C)=C2, 23.8
O=C9C%10=C([I](N=[N+]=[N-])O9)C=C(C(C)(C)C)C=C%10, [N]=[N+]=[N-],
O=C1C2=C([I]O1)C=C(C(F)(F)F)C=C2, 24.0
O=C%11C%12=C([I](N=[N+]=[N-])O%11)C=C(C)C=C%12, [N]=[N+]=[N-],
O=C1C2=C([I]O1)C=C(C)C=C2, 23.6
O=C%13C%14=C([I](N=[N+]=[N-])O%13)C=C(C(C)C)C=C%14, [N]=[N+]=[N-],
O=C1C2=C([I]O1)C=C(C(C)C)C=C2, 23.7
O=C1C2=C([I](N=[N+]=[N-])O1)C=CC(F)=C2, [N]=[N+]=[N-], O=C1C2=C([I]O1)C=CC(F)=C2, 23.7
O=C3C4=C([I](N=[N+]=[N-])O3)C=C(OC)C=C4, [N]=[N+]=[N-], O=C1C2=C([I]O1)C=C(OC)C=C2, 23.7
O=C5C6=C([I](N=[N+]=[N-])O5)C=CC(Br)=C6, [N]=[N+]=[N-], O=C1C2=C([I]O1)C=CC(Br)=C2,

23.5
O=C7C8=C([I](N=[N+]=[N-])O7)C=CC(Cl)=C8, [N]=[N+]=[N-], O=C1C2=C([I]O1)C=CC(Cl)=C2, 23.5
O=C9C%10=C([I](N=[N+]=[N-])O9)C=C(F)C=C%10, [N]=[N+]=[N-], O=C1C2=C([I]O1)C=C(F)C=C2, 23.1
O=C%11C%12=C([I](N=[N+]=[N-])O%11)C=C(Cl)C=C%12, [N]=[N+]=[N-], O=C1C2=C([I]O1)C=C(Cl)C=C2, 23.1
O=C%13C%14=C([I](N=[N+]=[N-])O%13)C=C(C(OC)=O)C=C%14, [N]=[N+]=[N-], O=C1C2=C([I]O1)C=C(C(OC)=O)C=C2, 23.4
O=C1C2=C([I](N=[N+]=[N-])O1)C=C(Br)C=C2, [N]=[N+]=[N-], O=C1C2=C([I]O1)C=C(Br)C=C2, 23.1
O=C3C4=C([I](N=[N+]=[N-])O3)C=C(C(F)(F)F)C=C4, [N]=[N+]=[N-], O=C1C2=C([I]O1)C=C(C(F)(F)F)C=C2, 23.2
O=C5C6=C([I](N=[N+]=[N-])O5)C=CC(C(OC)=O)=C6, [N]=[N+]=[N-], O=C1C2=C([I]O1)C=CC(C(OC)=O)=C2, 23.2
O=C7C8=C([I](N=[N+]=[N-])O7)C=CC(C(F)(F)F)=C8, [N]=[N+]=[N-], O=C1C2=C([I]O1)C=CC(C(F)(F)F)=C2, 23.3
O=C9C%10=C([I](N=[N+]=[N-])O9)C=C(C#N)C=C%10, [N]=[N+]=[N-], O=C1C2=C([I]O1)C=C(C#N)C=C2, 23.1
O=C%11C%12=C([I](N=[N+]=[N-])O%11)C=CC(C#N)=C%12, [N]=[N+]=[N-], O=C1C2=C([I]O1)C=CC(C#N)=C2, 23.0
O=C%13C%14=C([I](N=[N+]=[N-])O%13)C=C([N+]([O-])=O)C=C%14, [N]=[N+]=[N-], O=C1C2=C([I]O1)C=C([N+]([O-])=O)C=C2, 23.0
O=C%15C%16=C([I](N=[N+]=[N-])O%15)C=CC([N+]([O-])=O)=C%16, [N]=[N+]=[N-], O=C1C2=C([I]O1)C=CC([N+]([O-])=O)=C2, 22.8
O=C1C2=C([I](C(F)(F)F)O1)C=CC(OC)=C2, F[C](F)F, O=C1C2=C([I]O1)C=CC(OC)=C2, 32.1
O=C3C4=C([I](C(F)(F)F)O3)C=CC(C(C)(C)C)=C4, F[C](F)F, O=C1C2=C([I]O1)C=CC(C(C)(C)C)=C2, 31.6
O=C5C6=C([I](C(F)(F)F)O5)C=CC(C)=C6, F[C](F)F, O=C1C2=C([I]O1)C=CC(C)=C2, 31.6
O=C7C8=C([I](C(F)(F)F)O7)C=CC(C(C)C)=C8, F[C](F)F, O=C1C2=C([I]O1)C=CC(C(C)C)=C2, 31.6
O=C9C%10=C([I](C(F)(F)F)O9)C=C(C(F)(F)F)C=C%10, F[C](F)F, O=C1C2=C([I]O1)C=C(C(F)(F)F)C=C2, 31.6
O=C%11C%12=C([I](C(F)(F)F)O%11)C=C(C)C=C%12, F[C](F)F, O=C1C2=C([I]O1)C=C(C)C=C2, 31.3
O=C%13C%14=C([I](C(F)(F)F)O%13)C=C(C(C)C)C=C%14, F[C](F)F, O=C1C2=C([I]O1)C=C(C(C)C)C=C2, 31.4
O=C1C2=C([I](C(F)(F)F)O1)C=CC(F)=C2, F[C](F)F, O=C1C2=C([I]O1)C=CC(F)=C2, 31.6
O=C3C4=C([I](C(F)(F)F)O3)C=C(OC)C=C4, F[C](F)F, O=C1C2=C([I]O1)C=C(OC)C=C2, 30.9
O=C5C6=C([I](C(F)(F)F)O5)C=CC(Br)=C6, F[C](F)F, O=C1C2=C([I]O1)C=CC(Br)=C2, 31.4
O=C7C8=C([I](C(F)(F)F)O7)C=CC(Cl)=C8, F[C](F)F, O=C1C2=C([I]O1)C=CC(Cl)=C2, 31.5
O=C9C%10=C([I](C(F)(F)F)O9)C=C(F)C=C%10, F[C](F)F, O=C1C2=C([I]O1)C=C(F)C=C2, 30.8
O=C%11C%12=C([I](C(F)(F)F)O%11)C=C(Cl)C=C%12, F[C](F)F, O=C1C2=C([I]O1)C=C(Cl)C=C2, 30.9
O=C%13C%14=C([I](C(F)(F)F)O%13)C=C(C(OC)=O)C=C%14, F[C](F)F, O=C1C2=C([I]O1)C=C(C(OC)=O)C=C2, 31.7
O=C1C2=C([I](C(F)(F)F)O1)C=C(Br)C=C2, F[C](F)F, O=C1C2=C([I]O1)C=C(Br)C=C2, 30.9
O=C3C4=C([I](C(F)(F)F)O3)C=C(C(F)(F)F)C=C4, F[C](F)F, O=C1C2=C([I]O1)C=C(C(F)(F)F)C=C2, 31.4
O=C5C6=C([I](C(F)(F)F)O5)C=CC(C(OC)=O)=C6, F[C](F)F, O=C1C2=C([I]O1)C=CC(C(OC)=O)=C2, 31.0
O=C7C8=C([I](C(F)(F)F)O7)C=CC(C(F)(F)F)=C8, F[C](F)F, O=C1C2=C([I]O1)C=CC(C(F)(F)F)=C2,

31.2
O=C9C%10=C([I](C(F)(F)F)O9)C=C(C#N)C=C%10, F[C](F)F, O=C1C2=C([I]O1)C=C(C#N)C=C2, 31.2
O=C%11C%12=C([I](C(F)(F)F)O%11)C=CC(C#N)=C%12, F[C](F)F, O=C1C2=C([I]O1)C=CC(C#N)=C2, 31.0
O=C%13C%14=C([I](C(F)(F)F)O%13)C=C([N+]([O-])=O)C=C%14, F[C](F)F, O=C1C2=C([I]O1)C=C([N+]([O-])=O)C=C2, 31.3
O=C%15C%16=C([I](C(F)(F)F)O%15)C=CC([N+]([O-])=O)=C%16, F[C](F)F, O=C1C2=C([I]O1)C=CC([N+]([O-])=O)=C2, 30.7
Cl[I]1C2=C(C(O1)=O)C=C(OC)C=C2, [Cl], O=C1C2=C([I]O1)C=CC(OC)=C2, 48.1
Cl[I]3C4=C(C(O3)=O)C=C(C(C)(C)C)C=C4, [Cl], O=C1C2=C([I]O1)C=CC(C(C)(C)C)=C2, 47.9
Cl[I]5C6=C(C(O5)=O)C=C(C)C=C6, [Cl], O=C1C2=C([I]O1)C=CC(C)=C2, 47.9
Cl[I]7C8=C(C(O7)=O)C=C(C(C)C)C=C8, [Cl], O=C1C2=C([I]O1)C=CC(C(C)C)=C2, 47.9
Cl[I]9C%10=C(C(O9)=O)C=CC(C(F)(F)F)=C%10, [Cl], O=C1C2=C([I]O1)C=C(C(F)(F)F)C=C2, 48.1
Cl[I]%11C%12=C(C(O%11)=O)C=CC(C)=C%12, [Cl], O=C1C2=C([I]O1)C=C(C)C=C2, 47.8
Cl[I]%13C%14=C(C(O%13)=O)C=CC(C(C)C)=C%14, [Cl], O=C1C2=C([I]O1)C=C(C(C)C)C=C2, 47.8
Cl[I]1C2=C(C(O1)=O)C=C(F)C=C2, [Cl], O=C1C2=C([I]O1)C=CC(F)=C2, 47.6
Cl[I]3C4=C(C(O3)=O)C=CC(OC)=C4, [Cl], O=C1C2=C([I]O1)C=C(OC)C=C2, 48.2
Cl[I]5C6=C(C(O5)=O)C=C(Br)C=C6, [Cl], O=C1C2=C([I]O1)C=CC(Br)=C2, 47.4
Cl[I]7C8=C(C(O7)=O)C=C(Cl)C=C8, [Cl], O=C1C2=C([I]O1)C=CC(Cl)=C2, 47.4
Cl[I]9C%10=C(C(O9)=O)C=CC(F)=C%10, [Cl], O=C1C2=C([I]O1)C=C(F)C=C2, 47.0
Cl[I]%11C%12=C(C(O%11)=O)C=CC(Cl)=C%12, [Cl], O=C1C2=C([I]O1)C=C(Cl)C=C2, 47.0
Cl[I]%13C%14=C(C(O%13)=O)C=CC(C(OC)=O)=C%14, [Cl], O=C1C2=C([I]O1)C=C(C(OC)=O)C=C2, 46.9
Cl[I]1C2=C(C(O1)=O)C=CC(Br)=C2, [Cl], O=C1C2=C([I]O1)C=C(Br)C=C2, 47.0
Cl[I]3C4=C(C(O3)=O)C=CC(C(F)(F)F)=C4, [Cl], O=C1C2=C([I]O1)C=C(C(F)(F)F)C=C2, 46.9
Cl[I]5C6=C(C(O5)=O)C=C(C(OC)=O)C=C6, [Cl], O=C1C2=C([I]O1)C=CC(C(OC)=O)=C2, 47.1
Cl[I]7C8=C(C(O7)=O)C=C(C(F)(F)F)C=C8, [Cl], O=C1C2=C([I]O1)C=CC(C(F)(F)F)=C2, 47.1
Cl[I]9C%10=C(C(O9)=O)C=CC(C#N)=C%10, [Cl], O=C1C2=C([I]O1)C=C(C#N)C=C2, 46.7
Cl[I]%11C%12=C(C(O%11)=O)C=C(C#N)C=C%12, [Cl], O=C1C2=C([I]O1)C=CC(C#N)=C2, 46.8
Cl[I]%13C%14=C(C(O%13)=O)C=CC([N+]([O-])=O)=C%14, [Cl], O=C1C2=C([I]O1)C=C([N+]([O-])=O)C=C2, 46.4
Cl[I]%15C%16=C(C(O%15)=O)C=C([N+]([O-])=O)C=C%16, [Cl], O=C1C2=C([I]O1)C=CC([N+]([O-])=O)=C2, 46.5
CC1(C)C2=C([I](N=[N+]=[N-])O1)C=CC(OC)=C2, [N]=[N+]=[N-], CC1(C)C2=C([I]O1)C=CC(OC)=C2, 21.2
CC3(C)C4=C([I](N=[N+]=[N-])O3)C=CC(C(C)(C)C)=C4, [N]=[N+]=[N-], CC1(C)C2=C([I]O1)C=CC(C(C)(C)C)=C2, 21.0
CC5(C)C6=C([I](N=[N+]=[N-])O5)C=CC(C)=C6, [N]=[N+]=[N-], CC1(C)C2=C([I]O1)C=CC(C)=C2, 21.0
CC7(C)C8=C([I](N=[N+]=[N-])O7)C=CC(C(C)C)=C8, [N]=[N+]=[N-], CC1(C)C2=C([I]O1)C=CC(C(C)C)=C2, 21.0
CC9(C)C%10=C([I](N=[N+]=[N-])O9)C=C(C(F)(F)F)C=C%10, [N]=[N+]=[N-], CC1(C)C2=C([I]O1)C=C(C(F)(F)F)C=C2, 21.5
CC%11(C)C%12=C([I](N=[N+]=[N-])O%11)C=C(C)C=C%12, [N]=[N+]=[N-], CC1(C)C2=C([I]O1)C=C(C)C=C2, 20.9
CC%13(C)C%14=C([I](N=[N+]=[N-])O%13)C=C(C(C)C)C=C%14, [N]=[N+]=[N-], CC1(C)C2=C([I]O1)C=C(C(C)C)C=C2, 21.0
CC1(C)C2=C([I](N=[N+]=[N-])O1)C=C(C(OC)=O)C=C2, [N]=[N+]=[N-], FC1=CC2=C([I]OC2(C)C)C=C1, 20.5
ClC3=CC([I](N=[N+]=[N-])OC4(C)C)=C4C=C3, [N]=[N+]=[N-], CC1(C)C2=C([I]O1)C=C(OC)C=C2,

21.4
FC5=CC([I](N=[N+]=[N-])OC6(C)C)=C6C=C5, [N]=[N+]=[N-], BrC1=CC2=C([I]OC2(C)C)C=C1, 20.2
ClC7=CC8=C([I](N=[N+]=[N-])OC8(C)C)C=C7, [N]=[N+]=[N-], ClC1=CC2=C([I]OC2(C)C)C=C1, 20.3
BrC9=CC%10=C([I](N=[N+]=[N-])OC%10(C)C)C=C9, [N]=[N+]=[N-], FC1=CC([I]OC2(C)C)=C2C=C1, 20.1
CC%11(C)C%12=C([I](N=[N+]=[N-])O%11)C=C(OC)C=C%12, [N]=[N+]=[N-], ClC1=CC([I]OC2(C)C)=C2C=C1, 20.0
FC%13=CC%14=C([I](N=[N+]=[N-])OC%14(C)C)C=C%13, [N]=[N+]=[N-], CC1(C)C2=C([I]O1)C=C(C(OC)=O)C=C2, 20.5
BrC1=CC([I](N=[N+]=[N-])OC2(C)C)=C2C=C1, [N]=[N+]=[N-], BrC1=CC([I]OC2(C)C)=C2C=C1, 20.0
CC3(C)C4=C([I](N=[N+]=[N-])O3)C=C(C(F)(F)F)C=C4, [N]=[N+]=[N-], CC1(C)C2=C([I]O1)C=C(C(F)(F)F)C=C2, 20.0
CC5(C)C6=C([I](N=[N+]=[N-])O5)C=CC(C(OC)=O)=C6, [N]=[N+]=[N-], CC1(C)C2=C([I]O1)C=CC(C(OC)=O)=C2, 20.0
CC7(C)C8=C([I](N=[N+]=[N-])O7)C=CC(C(F)(F)F)=C8, [N]=[N+]=[N-], CC1(C)C2=C([I]O1)C=CC(C(F)(F)F)=C2, 19.8
CC9(C)C%10=C([I](N=[N+]=[N-])O9)C=C(C#N)C=C%10, [N]=[N+]=[N-], CC1(C)C2=C([I]O1)C=C(C#N)C=C2, 19.6
CC%11(C)C%12=C([I](N=[N+]=[N-])O%11)C=CC(C#N)=C%12, [N]=[N+]=[N-], CC1(C)C2=C([I]O1)C=CC(C#N)=C2, 19.5
CC%13(C)C%14=C([I](N=[N+]=[N-])O%13)C=C([N+]([O-])=O)C=C%14, [N]=[N+]=[N-], CC1(C)C2=C([I]O1)C=C([N+]([O-])=O)C=C2, 19.4
CC%15(C)C%16=C([I](N=[N+]=[N-])O%15)C=CC([N+]([O-])=O)=C%16, [N]=[N+]=[N-], CC1(C)C2=C([I]O1)C=CC([N+]([O-])=O)=C2, 19.2
CC1(C)C2=C([I](C(F)(F)F)O1)C=CC(OC)=C2, F[C](F)F, CC1(C)C2=C([I]O1)C=CC(OC)=C2, 25.4
CC3(C)C4=C([I](C(F)(F)F)O3)C=CC(C(C)(C)C)=C4, F[C](F)F, CC1(C)C2=C([I]O1)C=CC(C(C)(C)C)=C2, 25.0
CC5(C)C6=C([I](C(F)(F)F)O5)C=CC(C)=C6, F[C](F)F, CC1(C)C2=C([I]O1)C=CC(C)=C2, 25.0
CC7(C)C8=C([I](C(F)(F)F)O7)C=CC(C(C)C)=C8, F[C](F)F, CC1(C)C2=C([I]O1)C=CC(C(C)C)=C2, 25.0
CC9(C)C%10=C([I](C(F)(F)F)O9)C=C(C(F)(F)F)C=C%10, F[C](F)F, CC1(C)C2=C([I]O1)C=C(C(F)(F)F)C=C2, 25.2
CC%11(C)C%12=C([I](C(F)(F)F)O%11)C=C(C)C=C%12, F[C](F)F, CC1(C)C2=C([I]O1)C=C(C)C=C2, 24.8
CC%13(C)C%14=C([I](C(F)(F)F)O%13)C=C(C(C)C)C=C%14, F[C](F)F, CC1(C)C2=C([I]O1)C=C(C(C)C)C=C2, 24.9
CC1(C)C2=C([I](C(F)(F)F)O1)C=C(C(OC)=O)C=C2, F[C](F)F, FC1=CC2=C([I]OC2(C)C)C=C1, 24.7
ClC3=CC([I](C(F)(F)F)OC4(C)C)=C4C=C3, F[C](F)F, CC1(C)C2=C([I]O1)C=C(OC)C=C2, 24.8
FC5=CC([I](C(F)(F)F)OC6(C)C)=C6C=C5, F[C](F)F, BrC1=CC2=C([I]OC2(C)C)C=C1, 24.4
ClC7=CC8=C([I](C(F)(F)F)OC8(C)C)C=C7, F[C](F)F, ClC1=CC2=C([I]OC2(C)C)C=C1, 24.5
BrC9=CC%10=C([I](C(F)(F)F)OC%10(C)C)C=C9, F[C](F)F, FC1=CC([I]OC2(C)C)=C2C=C1, 24.1
CC%11(C)C%12=C([I](C(F)(F)F)O%11)C=C(OC)C=C%12, F[C](F)F, ClC1=CC([I]OC2(C)C)=C2C=C1, 24.1
FC%13=CC%14=C([I](C(F)(F)F)OC%14(C)C)C=C%13, F[C](F)F, CC1(C)C2=C([I]O1)C=C(C(OC)=O)C=C2, 24.8
BrC1=CC([I](C(F)(F)F)OC2(C)C)=C2C=C1, F[C](F)F, BrC1=CC([I]OC2(C)C)=C2C=C1, 24.1
CC3(C)C4=C([I](C(F)(F)F)O3)C=C(C(F)(F)F)C=C4, F[C](F)F, CC1(C)C2=C([I]O1)C=C(C(F)(F)F)C=C2, 24.3

CC5(C)C6=C([I](C(F)(F)F)O5)C=CC(C(OC)=O)=C6, F[C](F)F,
CC1(C)C2=C([I]O1)C=CC(C(OC)=O)=C2, 24.1
CC7(C)C8=C([I](C(F)(F)F)O7)C=CC(C(F)(F)F)=C8, F[C](F)F,
CC1(C)C2=C([I]O1)C=CC(C(F)(F)F)=C2, 24.0
CC9(C)C%10=C([I](C(F)(F)F)O9)C=C(C#N)C=C%10, F[C](F)F, CC1(C)C2=C([I]O1)C=C(C#N)C=C2,
24.0
CC%11(C)C%12=C([I](C(F)(F)F)O%11)C=CC(C#N)=C%12, F[C](F)F,
CC1(C)C2=C([I]O1)C=CC(C#N)=C2, 23.8
CC%13(C)C%14=C([I](C(F)(F)F)O%13)C=C([N+]([O-])=O)C=C%14, F[C](F)F,
CC1(C)C2=C([I]O1)C=C([N+]([O-])=O)C=C2, 24.0
CC%15(C)C%16=C([I](C(F)(F)F)O%15)C=CC([N+]([O-])=O)=C%16, F[C](F)F,
CC1(C)C2=C([I]O1)C=CC([N+]([O-])=O)=C2, 23.4
Cl[I]1C2=C(C(C)(C)O1)C=C(OC)C=C2, [Cl], CC1(C)C2=C([I]O1)C=CC(OC)=C2, 46.8
Cl[I]3C4=C(C(C)(C)O3)C=C(C(C)(C)C)C=C4, [Cl], CC1(C)C2=C([I]O1)C=CC(C(C)(C)C)=C2, 46.6
Cl[I]5C6=C(C(C)(C)O5)C=C(C)C=C6, [Cl], CC1(C)C2=C([I]O1)C=CC(C)=C2, 46.6
Cl[I]7C8=C(C(C)(C)O7)C=C(C(C)C)C=C8, [Cl], CC1(C)C2=C([I]O1)C=CC(C(C)C)=C2, 46.6
Cl[I]9C%10=C(C(C)(C)O9)C=CC(C(F)(F)F)=C%10, [Cl], CC1(C)C2=C([I]O1)C=C(C(F)(F)F)C=C2,
46.9
Cl[I]%11C%12=C(C(C)(C)O%11)C=CC(C)=C%12, [Cl], CC1(C)C2=C([I]O1)C=C(C)C=C2, 46.5
Cl[I]%13C%14=C(C(C)(C)O%13)C=CC(C(C)C)=C%14, [Cl], CC1(C)C2=C([I]O1)C=C(C(C)C)C=C2,
46.6
Cl[I]1C2=C(C(C)(C)O1)C=CC(C(OC)=O)=C2, [Cl], FC1=CC2=C([I]OC2(C)C)C=C1, 46.1
Cl[I]3C4=C(C(C)(C)O3)C=CC(Cl)=C4, [Cl], CC1(C)C2=C([I]O1)C=C(OC)C=C2, 47.1
Cl[I]5C6=C(C(C)(C)O5)C=CC(F)=C6, [Cl], BrC1=CC2=C([I]OC2(C)C)C=C1, 45.8
Cl[I]7C8=C(C(C)(C)O7)C=C(Cl)C=C8, [Cl], ClC1=CC2=C([I]OC2(C)C)C=C1, 45.8
Cl[I]9C%10=C(C(C)(C)O9)C=C(Br)C=C%10, [Cl], FC1=CC([I]OC2(C)C)=C2C=C1, 45.6
Cl[I]%11C%12=C(C(C)(C)O%11)C=CC(OC)=C%12, [Cl], ClC1=CC([I]OC2(C)C)=C2C=C1, 45.4
Cl[I]%13C%14=C(C(C)(C)O%13)C=C(F)C=C%14, [Cl], CC1(C)C2=C([I]O1)C=C(C(OC)=O)C=C2,
46.0
Cl[I]1C2=C(C(C)(C)O1)C=CC(Br)=C2, [Cl], BrC1=CC([I]OC2(C)C)=C2C=C1, 45.4
Cl[I]3C4=C(C(C)(C)O3)C=CC(C(F)(F)F)=C4, [Cl], CC1(C)C2=C([I]O1)C=C(C(F)(F)F)C=C2, 45.4
Cl[I]5C6=C(C(C)(C)O5)C=C(C(OC)=O)C=C6, [Cl], CC1(C)C2=C([I]O1)C=CC(C(OC)=O)=C2, 45.5
Cl[I]7C8=C(C(C)(C)O7)C=C(C(F)(F)F)C=C8, [Cl], CC1(C)C2=C([I]O1)C=CC(C(F)(F)F)=C2, 45.3
Cl[I]9C%10=C(C(C)(C)O9)C=CC(C#N)=C%10, [Cl], CC1(C)C2=C([I]O1)C=C(C#N)C=C2, 44.9
Cl[I]%11C%12=C(C(C)(C)O%11)C=C(C#N)C=C%12, [Cl], CC1(C)C2=C([I]O1)C=CC(C#N)=C2, 45.0
Cl[I]%13C%14=C(C(C)(C)O%13)C=CC([N+]([O-])=O)=C%14, [Cl],
CC1(C)C2=C([I]O1)C=C([N+]([O-])=O)C=C2, 44.6
Cl[I]%15C%16=C(C(C)(C)O%15)C=C([N+]([O-])=O)C=C%16, [Cl],
CC1(C)C2=C([I]O1)C=CC([N+]([O-])=O)=C2, 44.6
O=C(O[I]1Cl)C2=C1C(Br)=CC=C2, [Cl], O=C(O[I]1)C2=C1C(Br)=CC=C2, 36.3
O=C(O[I]3Cl)C4=C3C(C(F)(F)F)=CC=C4, [Cl], O=C(O[I]1)C2=C1C(C(F)(F)F)=CC=C2, 36.5
O=C(O[I]5Cl)C6=C5C(Cl)=CC=C6, [Cl], O=C(O[I]1)C2=C1C(Cl)=CC=C2, 36.8
O=C(O[I]7Cl)C8=C7C(C#N)=CC=C8, [Cl], O=C(O[I]1)C2=C1C(C#N)=CC=C2, 37.5
O=C(O[I]9Cl)C%10=C9C(C(C)(C)C)=CC=C%10, [Cl], O=C(O[I]1)C2=C1C(C(C)(C)C)=CC=C2, 38.8
O=C(O[I]%11Cl)C%12=C%11C([N+]([O-])=O)=CC=C%12, [Cl],
O=C(O[I]1)C2=C1C([N+]([O-])=O)=CC=C2, 38.9
O=C(O[I]%13Cl)C%14=C%13C(F)=CC=C%14, [Cl], O=C(O[I]1)C2=C1C(F)=CC=C2, 39.3
O=C(O[I]%15Cl)C%16=C%15C(OC)=CC=C%16, [Cl], O=C(O[I]1)C2=C1C(OC)=CC=C2, 39.6
O=C(O[I]1Cl)C2=C1C(C)=CC=C2, [Cl], O=C(O[I]1)C2=C1C(C)=CC=C2, 40.3
O=C(O[I]3Cl)C4=C3C(C(C)C)=CC=C4, [Cl], O=C(O[I]1)C2=C1C(C(C)C)=CC=C2, 40.4
O=C(O[I]5Cl)C6=C5C(C(OC)=O)=CC=C6, [Cl], O=C(O[I]1)C2=C1C(C(OC)=O)=CC=C2, 40.7

Cl[I]1OC(C)(C)C2=C1C(Br)=CC=C2, [Cl], BrC1=CC=CC2=C1[I]OC2(C)C, 37.5
Cl[I]3OC(C)(C)C4=C3C(C(F)(F)F)=CC=C4, [Cl], CC(O[I]1)(C)C2=C1C(C(F)(F)F)=CC=C2, 37.4
Cl[I]5OC(C)(C)C6=C5C(Cl)=CC=C6, [Cl], ClC1=CC=CC2=C1[I]OC2(C)C, 37.5
Cl[I]7OC(C)(C)C8=C7C(C#N)=CC=C8, [Cl], CC(O[I]1)(C)C2=C1C(C#N)=CC=C2, 37.3
Cl[I]9OC(C)(C)C%10=C9C(C(C)(C)C)=CC=C%10, [Cl], CC(O[I]1)(C)C2=C1C(C(C)(C)C)=CC=C2, 40.8
Cl[I]%11OC(C)(C)C%12=C%11C([N+]([O-])=O)=CC=C%12, [Cl], CC(O[I]1)(C)C2=C1C([N+]([O-])=O)=CC=C2, 41.0
Cl[I]%13OC(C)(C)C%14=C%13C(F)=CC=C%14, [Cl], FC1=CC=CC2=C1[I]OC2(C)C, 38.2
Cl[I]%15OC(C)(C)C%16=C%15C(OC)=CC=C%16, [Cl], CC(O[I]1)(C)C2=C1C(OC)=CC=C2, 39.9
Cl[I]1OC(C)(C)C2=C1C(C)=CC=C2, [Cl], CC(O[I]1)(C)C2=C1C(C)=CC=C2, 41.2
Cl[I]1OC(C)(C)C2=C1C(C(C)C)=CC=C2, [Cl], CC(O[I]1)(C)C2=C1C(C(C)C)=CC=C2, 40.9
Cl[I]1OC(C)(C)C2=C1C(C(OC)=O)=CC=C2, [Cl], CC(O[I]1)(C)C2=C1C(C(OC)=O)=CC=C2, 44.5
O=C(O[I]1C2=CNC3=CC=CC=C32)C4=C1C=CC=C4, C12=CC=CC=C1[C]=CN2, O=C(O[I]1)C2=C1C=CC=C2, 57.6
O=C(O[I]1C2=CC3=CC=CC=C3N2)C4=C1C=CC=C4, C12=CC=CC=C1C=[C]N2, O=C(O[I]1)C2=C1C=CC=C2, 53.8
O=C(O[I]5N6C7=CC=CC=C7C=C6)C8=C5C=CC=C8, C12=CC=CC=C1C=C[N]2, O=C(O[I]1)C2=C1C=CC=C2, 26.8
O=C(O[I]9C%10=CNC=C%10)C%11=C9C=CC=C%11, C1=C[C]=CN1, O=C(O[I]1)C2=C1C=CC=C2, 56.7
O=C(O[I]%12C%13=CC=CN%13)C%14=C%12C=CC=C%14, C1=CC=[C]N1, O=C(O[I]1)C2=C1C=CC=C2, 55.8
O=C(O[I]%15N%16C=CC=C%16)C%17=C%15C=CC=C%17, C1=CC=C[N]1, O=C(O[I]1)C2=C1C=CC=C2, 28.7
O=C(O[I]%18C%19=NC=CC=C%19)C%20=C%18C=CC=C%20, [C]1=NC=CC=C1, O=C(O[I]1)C2=C1C=CC=C2, 41.8
O=C(O[I]%21C%22=CN=CC=C%22)C%23=C%21C=CC=C%23, [C]1=CN=CC=C1, O=C(O[I]1)C2=C1C=CC=C2, 47.3
O=C(O[I]1C2=CC=NC=C2)C3=C1C=CC=C3, [C]1=CC=NC=C1, O=C(O[I]1)C2=C1C=CC=C2, 45.3
O=C(O[I]4I)C5=C4C=CC=C5, [I], O=C(O[I]1)C2=C1C=CC=C2, 31.6
O=C(O[I]6OC)C7=C6C=CC=C7, C[O], O=C(O[I]1)C2=C1C=CC=C2, 35.7
O=C(O[I]8OC(C)(C)C)C9=C8C=CC=C9, [O]C(C)(C)C, O=C(O[I]1)C2=C1C=CC=C2, 38.1
O=C(O[I]%10OC%11=CC=CC=C%11)C%12=C%10C=CC=C%12, [O]C1=CC=CC=C1, O=C(O[I]1)C2=C1C=CC=C2, 21.0
O=C(O[I]%13OC(F)(F)F)C%14=C%13C=CC=C%14, [O]C(F)(F)F, O=C(O[I]1)C2=C1C=CC=C2, 53.5
O=C(O[I]%15N(S(=O)(C(F)(F)F)=O)S(=O)(C(F)(F)F)=O)C%16=C%15C=CC=C%16, O=S([N]S(=O)(C(F)(F)F)=O)(C(F)(F)F)=O, O=C(O[I]1)C2=C1C=CC=C2, 51.6
O=C(O[I]%17NS(C%18=CC=C(C=C%18)C)(=O)=O)C%19=C%17C=CC=C%19, [NH]S(C1=CC=C(C)C=C1)(=O)=O, O=C(O[I]1)C2=C1C=CC=C2, 43.1
O=C(O[I]1N(C)C)C2=C1C=CC=C2, C[N]C, O=C(O[I]1)C2=C1C=CC=C2, 25.6
O=C(O[I]1SC)C2=C1C=CC=C2, C[S], O=C(O[I]1)C2=C1C=CC=C2, 32.3
O=C(O[I]3SC4=CC=CC=C4)C5=C3C=CC=C5, [S]C1=CC=CC=C1, O=C(O[I]1)C2=C1C=CC=C2, 26.3
O=C(O[I]6S(C7=CC=CC=C7)=O)C8=C6C=CC=C8, O=[S]C1=CC=CC=C1, O=C(O[I]1)C2=C1C=CC=C2, 3.3
O=C(O[I]9S(=O)(C%10=CC=CC=C%10)=O)C%11=C9C=CC=C%11, O=[S](C2=CC=CC=C2)=O, O=C(O[I]1)C2=C1C=CC=C2, 16.4
O=C(O[I]1OS(=O)(C)=O)C2=C1C=CC=C2, [O]S(=O)(C)=O, O=C(O[I]1)C2=C1C=CC=C2, 48.2
O=C(O[I]%14OS(=O)(C(F)(F)F)=O)C%15=C%14C=CC=C%15, [O]S(=O)(C(F)(F)F)=O, O=C(O[I]1)C2=C1C=CC=C2, 52.7
O=C(O[I]%16[Se]C)C%17=C%16C=CC=C%17, [Se]C, O=C(O[I]1)C2=C1C=CC=C2, 28.8

O=C(O[I]%18[Se]C%19=CC=CC=C%19)C%20=C%18C=CC=C%20, [Se]C1=CC=CC=C1,
O=C(O[I]1)C2=C1C=CC=C2, 32.3
O=C(O[I]1[Si](C)(C)C)C2=C1C=CC=C2, C[Si](C)C, O=C(O[I]1)C2=C1C=CC=C2, 50.2
O=C(O[I]1P(C2=CC=CC=C2)C3=CC=CC=C3)C4=C1C=CC=C4,
C1([P]C2=CC=CC=C2)=CC=CC=C1, O=C(O[I]1)C2=C1C=CC=C2, 26.0
O=C(O[I]5C6=CC=CC=C6)C7=C5C=CC=C7, [C]1=CC=CC=C1, O=C(O[I]1)C2=C1C=CC=C2, 47.9
O=C(O[I]8C)C9=C8C=CC=C9, [H][C]([H])[H], O=C(O[I]1)C2=C1C=CC=C2, 36.9
O=C(O[I]%10CC)C%11=C%10C=CC=C%11, [C]C, O=C(O[I]1)C2=C1C=CC=C2, 35.2
O=C(O[I]%12C(C)(C)C)C%13=C%12C=CC=C%13, C[C](C)C, O=C(O[I]1)C2=C1C=CC=C2, 30.9
O=C(O[I]%14C=C)C%15=C%14C=CC=C%15, [C]=C, O=C(O[I]1)C2=C1C=CC=C2, 44.6
O=C(O[I]%16/C=C/C%17=CC=CC=C%17)C%18=C%16C=CC=C%18, [H]/[C]=C\C1=CC=CC=C1,
O=C(O[I]1)C2=C1C=CC=C2, 47.2
O=C(O[I]%19C(F)F)C%20=C%19C=CC=C%20, F[C]F, O=C(O[I]1)C2=C1C=CC=C2, 29.2
O=C(O[I]%21C(F)(C(F)(F)F)F)C%22=C%21C=CC=C%22, FC(F)(F)[C](F)F,
O=C(O[I]1)C2=C1C=CC=C2, 28.9
Cl[I]1N(C2=CC=CC=C2)S(C3=C1C=CC=C3)(=O)=O, [Cl],
O=S(C1=C2C=CC=C1)(N(C3=CC=CC=C3)[I]2)=O, 28.3
Cl[I]1N(C)S(C2=C1C=CC=C2)(=O)=O, [Cl], O=S(C1=C2C=CC=C1)(N(C)[I]2)=O, 38.4
Cl[I]1C2=C(N(C)C(O1)=O)C=CC=C2, [Cl], O=C1N(C)C2=C([I]O1)C=CC=C2, 47.6
Cl[I]1C2=C(N(C)C(N1C3=CC=CC=C3)=O)C=CC=C2, [Cl],
O=C1N(C)C2=C([I]N1C3=CC=CC=C3)C=CC=C2, 27.2
Cl[I]1C2=C(N(C)C(N1C)=O)C=CC=C2, [Cl], O=C1N(C)C2=C([I]N1C)C=CC=C2, 37.8
O=C1C(C)N(C)C2=C([I](Cl)O1)C=CC=C2, [Cl], O=C1C(C)N(C)C2=C([I]O1)C=CC=C2, 43.9
Cl[I]1SC(C)(C)C2=C1C=CC=C2, [Cl], CC(S[I]1)(C)C2=C1C=CC=C2, 40.1
Cl[I]1C2=C(CC(N1C3=CC=CC=C3)=O)C=CC=C2, [Cl],
O=C1CC2=C([I]N1C3=CC=CC=C3)C=CC=C2, 31.0
Cl[I]1C2=C(CC(N1C)=O)C=CC=C2, [Cl], O=C1CC2=C([I]N1C)C=CC=C2, 39.5
Cl[I]1C2=C(CC(O1)=O)C=CC=C2, [Cl], O=C1CC2=C([I]O1)C=CC=C2, 44.7
Cl[I]1C2=C(CC(C)(C)S1)C=CC=C2, [Cl], CC1(C)CC2=C([I]S1)C=CC=C2, 36.8
Cl[I]1C2=C(CC(C)(C)O1)C=CC=C2, [Cl], CC1(C)CC2=C([I]O1)C=CC=C2, 41.5
Cl[I](OC1=O)C2=C(CC1)C=CC=C2, [Cl], O=C1O[I]C2=C(CC1)C=CC=C2, 40.2
Cl[I](OC(CCC1)=O)C2=C1C=CC=C2, [Cl], O=C(CCC1)O[I]C2=C1C=CC=C2, 39.8
O=C(N(C1=CC=CC=C1)[I]2Cl)C3=C2C=CC=C3, [Cl],
O=C(N(C1=CC=CC=C1)[I]2)C3=C2C=CC=C3, 37.9
O=C(N(OC)[I]1Cl)C2=C1C=CC=C2, [Cl], O=C(N(OC)[I]1)C2=C1C=CC=C2, 34.3
Cl[I]1OC(C2=C1C=CC=C2)=NC, [Cl], CN=C(O[I]1)C2=C1C=CC=C2, 36.9
O=C(N(C)[I]1Cl)C2=C1C=CC=C2, [Cl], O=C(N(C)[I]1)C2=C1C=CC=C2, 46.4
O=C(N(C(C)=O)[I]1Cl)C2=C1C=CC=C2, [Cl], O=C(N(C(C)=O)[I]1)C2=C1C=CC=C2, 48.4
C=C[I]1OC(C)(C)C2=C1C=CC=C2, [C]=C, CC(O[I]1)(C)C2=C1C=CC=C2, 34.0
C[Si](C)(C)[I]1OC(C)(C)C2=C1C=CC=C2, C[Si](C)C, CC(O[I]1)(C)C2=C1C=CC=C2, 34.1
CC(O[I]1C(C)(C)C)(C)C2=C1C=CC=C2, C[C](C)C, CC(O[I]1)(C)C2=C1C=CC=C2, 18.5
CC(O[I]1/C=C/C2=CC=CC=C2)(C)C3=C1C=CC=C3, [H]/[C]=C\C1=CC=CC=C1,
CC(O[I]1)(C)C2=C1C=CC=C2, 36.1
CC(O[I]1SC2=CC=CC=C2)(C)C3=C1C=CC=C3, [S]C1=CC=CC=C1, CC(O[I]1)(C)C2=C1C=CC=C2,
20.5
O=S(C1=CC=CC=C1)[I]2OC(C)(C)C3=C2C=CC=C3, O=[S]C1=CC=CC=C1,
CC(O[I]1)(C)C2=C1C=CC=C2, -1.7
O=S([I]1OC(C)(C)C2=C1C=CC=C2)(C3=CC=CC=C3)=O, O=[S](C1=CC=CC=C1)=O,
CC(O[I]1)(C)C2=C1C=CC=C2, 12.2
CC(O[I]1SC)(C)C2=C1C=CC=C2, C[S], CC(O[I]1)(C)C2=C1C=CC=C2, 25.7
CC(O[I]1[Se]C2=CC=CC=C2)(C)C3=C1C=CC=C3, [Se]C1=CC=CC=C1,

CC(O[I]1)(C)C2=C1C=CC=C2, 27.4
CC(O[I]1[Se]C)(C)C2=C1C=CC=C2, [Se]C, CC(O[I]1)(C)C2=C1C=CC=C2, 23.5
CC(O[I]1N2C=CC=C2)(C)C3=C1C=CC=C3, C1=CC=C[N]1, CC(O[I]1)(C)C2=C1C=CC=C2, 23.8
CC(O[I]1C2=CNC=C2)(C)C3=C1C=CC=C3, C1=C[C]=CN1, CC(O[I]1)(C)C2=C1C=CC=C2, 44.5
CC(O[I]1C2=CC=CN2)(C)C3=C1C=CC=C3, C1=CC=[C]N1, CC(O[I]1)(C)C2=C1C=CC=C2, 45.0
CC(O[I]1C2=CC=NC=C2)(C)C3=C1C=CC=C3, [C]1=CC=NC=C1, CC(O[I]1)(C)C2=C1C=CC=C2, 35.2
CC(O[I]1C2=CN=CC=C2)(C)C3=C1C=CC=C3, [C]1=CN=CC=C1, CC(O[I]1)(C)C2=C1C=CC=C2, 36.9
CC(O[I]1C2=NC=CC=C2)(C)C3=C1C=CC=C3, [C]1=NC=CC=C1, CC(O[I]1)(C)C2=C1C=CC=C2, 30.7
CC(O[I]1P(C2=CC=CC=C2)C3=CC=CC=C3)(C)C4=C1C=CC=C4, C1([P]C2=CC=CC=C2)=CC=CC=C1, CC(O[I]1)(C)C2=C1C=CC=C2, 15.7
CC(O[I]1C2=CC=CC=C2)(C)C3=C1C=CC=C3, [C]1=CC=CC=C1, CC(O[I]1)(C)C2=C1C=CC=C2, 36.5
CC(O[I]1OS(=O)(C(F)(F)F)=O)(C)C2=C1C=CC=C2, [O]S(=O)(C(F)(F)F)=O, CC(O[I]1)(C)C2=C1C=CC=C2, 55.6
CC(C)(C)O[I]1OC(C)(C)C2=C1C=CC=C2, [O]C(C)(C)C, CC(O[I]1)(C)C2=C1C=CC=C2, 33.5
CC(O[I]1OS(=O)(C)=O)(C)C2=C1C=CC=C2, [O]S(=O)(C)=O, CC(O[I]1)(C)C2=C1C=CC=C2, 48.9
CC(O[I]1OC2=CC=CC=C2)(C)C3=C1C=CC=C3, [O]C1=CC=CC=C1, CC(O[I]1)(C)C2=C1C=CC=C2, 18.2
CC(O[I]1OC)(C)C2=C1C=CC=C2, C[O], CC(O[I]1)(C)C2=C1C=CC=C2, 31.4
CC(O[I]1OC(F)(F)F)(C)C2=C1C=CC=C2, [O]C(F)(F)F, CC(O[I]1)(C)C2=C1C=CC=C2, 53.2
CC(O[I]1N(S(=O)(C(F)(F)F)=O)S(=O)(C(F)(F)F)=O)(C)C2=C1C=CC=C2, O=S([N]S(=O)(C(F)(F)F)=O)(C(F)(F)F)=O, CC(O[I]1)(C)C2=C1C=CC=C2, 55.2
CC(O[I]1N(C)C)(C)C2=C1C=CC=C2, C[N]C, CC(O[I]1)(C)C2=C1C=CC=C2, 17.6
CC(O[I]1NS(C2=CC=C(C=C2)C)(=O)=O)(C)C3=C1C=CC=C3, [NH]S(C1=CC=C(C)C=C1)(=O)=O, CC(O[I]1)(C)C2=C1C=CC=C2, 39.3
CC(O[I]1C)(C)C2=C1C=CC=C2, [H][C]([H])[H], CC(O[I]1)(C)C2=C1C=CC=C2, 25.6
CC(O[I]1N2C3=CC=CC=C3C=C2)(C)C4=C1C=CC=C4, C12=CC=CC=C1C=C[N]2, CC(O[I]1)(C)C2=C1C=CC=C2, 22.2
CC(O[I]1C2=CNC3=CC=CC=C32)(C)C4=C1C=CC=C4, C12=CC=CC=C1[C]=CN2, CC(O[I]1)(C)C2=C1C=CC=C2, 45.9
CC(O[I]1C2=CC3=CC=CC=C3N2)(C)C4=C1C=CC=C4, C12=CC=CC=C1C=[C]N2, CC(O[I]1)(C)C2=C1C=CC=C2, 43.6
I[I]1OC(C)(C)C2=C1C=CC=C2, [I], CC(O[I]1)(C)C2=C1C=CC=C2, 31.2
CC(O[I]1CC)(C)C2=C1C=CC=C2, [C]C, CC(O[I]1)(C)C2=C1C=CC=C2, 23.5
CC(O[I]1C(F)F)(C)C2=C1C=CC=C2, F[C]F, CC(O[I]1)(C)C2=C1C=CC=C2, 21.0
CC(O[I]1C(F)(C(F)(F)F)F)(C)C2=C1C=CC=C2, FC(F)(F)[C](F)F, CC(O[I]1)(C)C2=C1C=CC=C2, 22.7
O=S(C1=C2C=CC=C1)(N(C3=CC=CC=C3)[I]2C(F)(F)F)=O, F[C](F)F, O=S(C1=C2C=CC=C1)(N(C3=CC=CC=C3)[I]2)=O, 9.8
O=S(C1=C2C=CC=C1)(N(C)[I]2C(F)(F)F)=O, F[C](F)F, O=S(C1=C2C=CC=C1)(N(C)[I]2)=O, 17.6
O=C(O1)N(C)C2=C([I]1C(F)(F)F)C=CC=C2, F[C](F)F, O=C1N(C)C2=C([I]O1)C=CC=C2, 32.5
O=C(N1C2=CC=CC=C2)N(C)C3=C([I]1C(F)(F)F)C=CC=C3, F[C](F)F, O=C1N(C)C2=C([I]N1C3=CC=CC=C3)C=CC=C2, 6.9
O=C(N1C)N(C)C2=C([I]1C(F)(F)F)C=CC=C2, F[C](F)F, O=C1N(C)C2=C([I]N1C)C=CC=C2, 15.3
O=C(O1)C(C)N(C)C2=C([I]1C(F)(F)F)C=CC=C2, F[C](F)F, O=C1C(C)N(C)C2=C([I]O1)C=CC=C2, 29.5
CC(S[I]1C(F)(F)F)(C)C2=C1C=CC=C2, F[C](F)F, CC(S[I]1)(C)C2=C1C=CC=C2, 14.1
O=C(N1C2=CC=CC=C2)CC3=C([I]1C(F)(F)F)C=CC=C3, F[C](F)F, O=C1CC2=C([I]N1C3=CC=CC=C3)C=CC=C2, 10.2

O=C(N1C)CC2=C([I]1C(F)(F)F)C=CC=C2, F[C](F)F, O=C1CC2=C([I]N1C)C=CC=C2, 16.5
O=C(O1)CC2=C([I]1C(F)(F)F)C=CC=C2, F[C](F)F, O=C1CC2=C([I]O1)C=CC=C2, 28.8
CC(S1)(C)CC2=C([I]1C(F)(F)F)C=CC=C2, F[C](F)F, CC1(C)CC2=C([I]S1)C=CC=C2, 11.6
CC(O1)(C)CC2=C([I]1C(F)(F)F)C=CC=C2, F[C](F)F, CC1(C)CC2=C([I]O1)C=CC=C2, 19.8
O=C1O[I](C(F)(F)F)C2=C(CC1)C=CC=C2, F[C](F)F, O=C1O[I]C2=C(CC1)C=CC=C2, 25.3
O=C(CCC1)O[I](C(F)(F)F)C2=C1C=CC=C2, F[C](F)F, O=C(CCC1)O[I]C2=C1C=CC=C2, 24.7
O=C(N(C1=CC=CC=C1)[I]2C(F)(F)F)C3=C2C=CC=C3, F[C](F)F,
O=C(N(C1=CC=CC=C1)[I]2)C3=C2C=CC=C3, 16.5
O=C(N(OC)[I]1C(F)(F)F)C2=C1C=CC=C2, F[C](F)F, O=C(N(OC)[I]1)C2=C1C=CC=C2, 12.5
CN=C(O[I]1C(F)(F)F)C2=C1C=CC=C2, F[C](F)F, CN=C(O[I]1)C2=C1C=CC=C2, 19.7
O=C(N(C)[I]1C(F)(F)F)C2=C1C=CC=C2, F[C](F)F, O=C(N(C)[I]1)C2=C1C=CC=C2, 23.1
O=C(N(C(C)=O)[I]1C(F)(F)F)C2=C1C=CC=C2, F[C](F)F, O=C(N(C(C)=O)[I]1)C2=C1C=CC=C2, 27.8
C=C[I]1OC(C(F)(F)F)(C(F)(F)F)C2=C1C=CC=C2, [C]=C,
FC(C(O[I]1)(C(F)(F)F)C2=C1C=CC=C2)(F)F, 45.3
C[Si](C)(C)[I]1OC(C(F)(F)F)(C(F)(F)F)C2=C1C=CC=C2, C[Si](C)C,
FC(C(O[I]1)(C(F)(F)F)C2=C1C=CC=C2)(F)F, 48.7
CC([I]1OC(C(F)(F)F)(C(F)(F)F)C2=C1C=CC=C2)(C)C, C[C](C)C,
FC(C(O[I]1)(C(F)(F)F)C2=C1C=CC=C2)(F)F, 30.6
FC(C(O[I]1/C=C/C2=CC=CC=C2)(C(F)(F)F)C3=C1C=CC=C3)(F)F, [H]/[C]=C\C1=CC=CC=C1,
FC(C(O[I]1)(C(F)(F)F)C2=C1C=CC=C2)(F)F, 47.8
FC(C(O[I]1SC2=CC=CC=C2)(C(F)(F)F)C3=C1C=CC=C3)(F)F, [S]C1=CC=CC=C1,
FC(C(O[I]1)(C(F)(F)F)C2=C1C=CC=C2)(F)F, 28.0
O=S(C1=CC=CC=C1)[I]2OC(C(F)(F)F)(C(F)(F)F)C3=C2C=CC=C3, O=[S]C1=CC=CC=C1,
FC(C(O[I]1)(C(F)(F)F)C2=C1C=CC=C2)(F)F, 5.0
O=S([I]1OC(C(F)(F)F)(C(F)(F)F)C2=C1C=CC=C2)(C3=CC=CC=C3)=O, O=[S](C1=CC=CC=C1)=O,
FC(C(O[I]1)(C(F)(F)F)C2=C1C=CC=C2)(F)F, 18.7
CS[I]1OC(C(F)(F)F)(C(F)(F)F)C2=C1C=CC=C2, C[S], FC(C(O[I]1)(C(F)(F)F)C2=C1C=CC=C2)(F)F,
34.0
FC(C(O[I]1[Se]C2=CC=CC=C2)(C(F)(F)F)C3=C1C=CC=C3)(F)F, [Se]C1=CC=CC=C1,
FC(C(O[I]1)(C(F)(F)F)C2=C1C=CC=C2)(F)F, 34.1
C[Se][I]1OC(C(F)(F)F)(C(F)(F)F)C2=C1C=CC=C2, [Se]C,
FC(C(O[I]1)(C(F)(F)F)C2=C1C=CC=C2)(F)F, 30.7
FC(C(O[I]1N2C=CC=C2)(C(F)(F)F)C3=C1C=CC=C3)(F)F, C1=CC=C[N]1,
FC(C(O[I]1)(C(F)(F)F)C2=C1C=CC=C2)(F)F, 30.9
FC(C(O[I]1C2=CNC=C2)(C(F)(F)F)C3=C1C=CC=C3)(F)F, C1=C[C]=CN1,
FC(C(O[I]1)(C(F)(F)F)C2=C1C=CC=C2)(F)F, 57.0
FC(C(O[I]1C2=CC=CN2)(C(F)(F)F)C3=C1C=CC=C3)(F)F, C1=CC=[C]N1,
FC(C(O[I]1)(C(F)(F)F)C2=C1C=CC=C2)(F)F, 56.7
FC(C(O[I]1C2=CC=NC=C2)(C(F)(F)F)C3=C1C=CC=C3)(F)F, [C]1=CC=NC=C1,
FC(C(O[I]1)(C(F)(F)F)C2=C1C=CC=C2)(F)F, 46.0
FC(C(O[I]1C2=CN=CC=C2)(C(F)(F)F)C3=C1C=CC=C3)(F)F, [C]1=CN=CC=C1,
FC(C(O[I]1)(C(F)(F)F)C2=C1C=CC=C2)(F)F, 48.0
FC(C(O[I]1C2=NC=CC=C2)(C(F)(F)F)C3=C1C=CC=C3)(F)F, [C]1=NC=CC=C1,
FC(C(O[I]1)(C(F)(F)F)C2=C1C=CC=C2)(F)F, 42.3
FC(C(O[I]1P(C2=CC=CC=C2)C3=CC=CC=C3)(C(F)(F)F)C4=C1C=CC=C4)(F)F,
C1([P]C2=CC=CC=C2)=CC=CC=C1, FC(C(O[I]1)(C(F)(F)F)C2=C1C=CC=C2)(F)F, 26.9
FC(C(O[I]1C2=CC=CC=C2)(C(F)(F)F)C3=C1C=CC=C3)(F)F, [C]1=CC=CC=C1,
FC(C(O[I]1)(C(F)(F)F)C2=C1C=CC=C2)(F)F, 48.4
O=S(O[I]1OC(C(F)(F)F)(C(F)(F)F)C2=C1C=CC=C2)(C(F)(F)F)=O, [O]S(=O)(C(F)(F)F)=O,
FC(C(O[I]1)(C(F)(F)F)C2=C1C=CC=C2)(F)F, 55.3
CC(C)(C)O[I]1OC(C(F)(F)F)(C(F)(F)F)C2=C1C=CC=C2, [O]C(C)(C)C,

FC(C(O[I]1)(C(F)(F)F)C2=C1C=CC=C2)(F)F, 40.8
O=S(O[I]1OC(C(F)(F)F)(C(F)(F)F)C2=C1C=CC=C2)(C)=O, [O]S(=O)(C)=O,
FC(C(O[I]1)(C(F)(F)F)C2=C1C=CC=C2)(F)F, 50.9
FC(C(O[I]1OC2=CC=CC=C2)(C(F)(F)F)C3=C1C=CC=C3)(F)F, [O]C1=CC=CC=C1,
FC(C(O[I]1)(C(F)(F)F)C2=C1C=CC=C2)(F)F, 23.7
CO[I]1OC(C(F)(F)F)(C(F)(F)F)C2=C1C=CC=C2, C[O], FC(C(O[I]1)(C(F)(F)F)C2=C1C=CC=C2)(F)F, 38.3
FC(O[I]1OC(C(F)(F)F)(C(F)(F)F)C2=C1C=CC=C2)(F)F, [O]C(F)(F)F,
FC(C(O[I]1)(C(F)(F)F)C2=C1C=CC=C2)(F)F, 56.1
O=S(N([I]1OC(C(F)(F)F)(C(F)(F)F)C2=C1C=CC=C2)S(=O)(C(F)(F)F)=O)(C(F)(F)F)=O,
O=S([N]S(=O)(C(F)(F)F)=O)(C(F)(F)F)=O, FC(C(O[I]1)(C(F)(F)F)C2=C1C=CC=C2)(F)F, 54.1
CN([I]1OC(C(F)(F)F)(C(F)(F)F)C2=C1C=CC=C2)C, C[N]C,
FC(C(O[I]1)(C(F)(F)F)C2=C1C=CC=C2)(F)F, 27.3
CC1=CC=C(S(N[I]2OC(C(F)(F)F)(C(F)(F)F)C3=C2C=CC=C3)(=O)=O)C=C1,
[NH]S(C1=CC=C(C)C=C1)(=O)=O, FC(C(O[I]1)(C(F)(F)F)C2=C1C=CC=C2)(F)F, 45.2
C[I]1OC(C(F)(F)F)(C(F)(F)F)C2=C1C=CC=C2, [H][C]([H])[H],
FC(C(O[I]1)(C(F)(F)F)C2=C1C=CC=C2)(F)F, 37.1
FC(C(O[I]1N2C3=CC=CC=C3C=C2)(C(F)(F)F)C4=C1C=CC=C4)(F)F, C12=CC=CC=C1C=C[N]2,
FC(C(O[I]1)(C(F)(F)F)C2=C1C=CC=C2)(F)F, 29.1
FC(C(O[I]1C2=CNC3=CC=CC=C32)(C(F)(F)F)C4=C1C=CC=C4)(F)F, C12=CC=CC=C1[C]=CN2,
FC(C(O[I]1)(C(F)(F)F)C2=C1C=CC=C2)(F)F, 58.2
FC(C(O[I]1C2=CC3=CC=CC=C3N2)(C(F)(F)F)C4=C1C=CC=C4)(F)F, C12=CC=CC=C1C=[C]N2,
FC(C(O[I]1)(C(F)(F)F)C2=C1C=CC=C2)(F)F, 54.9
I[I]1OC(C(F)(F)F)(C(F)(F)F)C2=C1C=CC=C2, [I], FC(C(O[I]1)(C(F)(F)F)C2=C1C=CC=C2)(F)F, 33.9
CC[I]1OC(C(F)(F)F)(C(F)(F)F)C2=C1C=CC=C2, [C]C, FC(C(O[I]1)(C(F)(F)F)C2=C1C=CC=C2)(F)F, 35.3
FC([I]1OC(C(F)(F)F)(C(F)(F)F)C2=C1C=CC=C2)F, F[C]F,
FC(C(O[I]1)(C(F)(F)F)C2=C1C=CC=C2)(F)F, 30.4
FC([I]1OC(C(F)(F)F)(C(F)(F)F)C2=C1C=CC=C2)(C(F)(F)F)F, FC(F)(F)[C](F)F,
FC(C(O[I]1)(C(F)(F)F)C2=C1C=CC=C2)(F)F, 30.2
O=S(C1=C2C=CC=C1)(N(C3=CC=CC=C3)[I]2C#C[Si](C)(C)C)=O, [C]#C[Si](C)(C)C,
O=S(C1=C2C=CC=C1)(N(C3=CC=CC=C3)[I]2)=O, 44.7
O=S(C1=C2C=CC=C1)(N(C)[I]2C#C[Si](C)(C)C)=O, [C]#C[Si](C)(C)C,
O=S(C1=C2C=CC=C1)(N(C)[I]2)=O, 52.2
O=C(O1)N(C)C2=C([I]1C#C[Si](C)(C)C)C=CC=C2, [C]#C[Si](C)(C)C,
O=C1N(C)C2=C([I]O1)C=CC=C2, 66.7
O=C(N1C2=CC=CC=C2)N(C)C3=C([I]1C#C[Si](C)(C)C)C=CC=C3, [C]#C[Si](C)(C)C,
O=C1N(C)C2=C([I]N1C3=CC=CC=C3)C=CC=C2, 40.0
O=C(N1C)N(C)C2=C([I]1C#C[Si](C)(C)C)C=CC=C2, [C]#C[Si](C)(C)C,
O=C1N(C)C2=C([I]N1C)C=CC=C2, 48.2
O=C(O1)C(C)N(C)C2=C([I]1C#C[Si](C)(C)C)C=CC=C2, [C]#C[Si](C)(C)C,
O=C1C(C)N(C)C2=C([I]O1)C=CC=C2, 62.8
CC(S[I]1C#C[Si](C)(C)C)(C)C2=C1C=CC=C2, [C]#C[Si](C)(C)C, CC(S[I]1)(C)C2=C1C=CC=C2, 46.7
O=C(N1C2=CC=CC=C2)CC3=C([I]1C#C[Si](C)(C)C)C=CC=C3, [C]#C[Si](C)(C)C,
O=C1CC2=C([I]N1C3=CC=CC=C3)C=CC=C2, 43.3
O=C(N1C)CC2=C([I]1C#C[Si](C)(C)C)C=CC=C2, [C]#C[Si](C)(C)C,
O=C1CC2=C([I]N1C)C=CC=C2, 49.6
O=C(O1)CC2=C([I]1C#C[Si](C)(C)C)C=CC=C2, [C]#C[Si](C)(C)C, O=C1CC2=C([I]O1)C=CC=C2, 62.9
CC(S1)(C)CC2=C([I]1C#C[Si](C)(C)C)C=CC=C2, [C]#C[Si](C)(C)C, CC1(C)CC2=C([I]S1)C=CC=C2, 43.0

CC(O1)(C)CC2=C([I]1C#C[Si](C)(C)C)C=CC=C2, [C]#C[Si](C)(C)C,
CC1(C)CC2=C([I]O1)C=CC=C2, 52.6
O=C1O[I](C#C[Si](C)(C)C)C2=C(CC1)C=CC=C2, [C]#C[Si](C)(C)C,
O=C1O[I]C2=C(CC1)C=CC=C2, 58.9
O=C(CCC1)O[I](C#C[Si](C)(C)C)C2=C1C=CC=C2, [C]#C[Si](C)(C)C,
O=C(CCC1)O[I]C2=C1C=CC=C2, 57.9
O=C(N(C1=CC=CC=C1)[I]2C#C[Si](C)(C)C)C3=C2C=CC=C3, [C]#C[Si](C)(C)C,
O=C(N(C1=CC=CC=C1)[I]2)C3=C2C=CC=C3, 51.2
O=C(N(OC)[I]1C#C[Si](C)(C)C)C2=C1C=CC=C2, [C]#C[Si](C)(C)C,
O=C(N(OC)[I]1)C2=C1C=CC=C2, 47.3
CN=C(O[I]1C#C[Si](C)(C)C)C2=C1C=CC=C2, [C]#C[Si](C)(C)C, CN=C(O[I]1)C2=C1C=CC=C2,
55.0
O=C(N(C)[I]1C#C[Si](C)(C)C)C2=C1C=CC=C2, [C]#C[Si](C)(C)C, O=C(N(C)[I]1)C2=C1C=CC=C2,
57.5
O=C(N(C(C)=O)[I]1C#C[Si](C)(C)C)C2=C1C=CC=C2, [C]#C[Si](C)(C)C,
O=C(N(C(C)=O)[I]1)C2=C1C=CC=C2, 62.9
O=C(O[I]1Cl)C2=C1SC=C2, [Cl], O=C(O[I]1)C2=C1SC=C2, 41.7
O=C1C2=CSC=C2[I](Cl)O1, [Cl], O=C1C2=CSC=C2[I]O1, 44.0
O=C1O[I](Cl)C2=C1SC=C2, [Cl], O=C1O[I]C2=C1SC=C2, 44.1
O=C3O[I](Cl)C4=NC5=CC=CC=C5C=C43, [Cl], O=C3O[I]C4=NC5=CC=CC=C5C=C43, 46.3
O=C(O[I]1Cl)C2=C1C3=CC=CC=C3N=C2, [Cl], O=C(O[I]1)C2=C1C3=CC=CC=C3N=C2, 39.1
Cl[I](OC1=O)C2=CC=NC3=CC=CC1=C32, [Cl], [I](OC1=O)C2=CC=NC3=CC=CC1=C32, 48.0
O=C(O[I]1Cl)C2=CC=NC3=CC=CC1=C32, [Cl], O=C(O[I]1)C2=CC=NC3=CC=CC1=C32, 48.8
Cl[I](OC1=O)C2=C1C3=CC=CC=C3N=C2, [Cl], [I](OC1=O)C2=C1C3=CC=CC=C3N=C2, 47.5
Cl[I]1OC(C2=NC3=CC=CC=C3C=C21)=O, [Cl], [I]1OC(C2=NC3=CC=CC=C3C=C21)=O, 46.4
Cl[I](O1)C2=C3C(N=CC=C3)=CC=C2C1=O, [Cl], [I](O1)C2=C3C(N=CC=C3)=CC=C2C1=O, 40.2
O=C1C2=C3C(N=CC=C3)=CC=C2[I](Cl)O1, [Cl], O=C1C2=C3C(N=CC=C3)=CC=C2[I]O1, 49.0
O=C(O[I]1Cl)C2=C1C=C(N=CC=C3)C3=C2, [Cl], O=C(O[I]1)C2=C1C=C(N=CC=C3)C3=C2, 47.2
Cl[I](OC1=O)C2=C1C=C(N=CC=C3)C3=C2, [Cl], [I](OC1=O)C2=C1C=C(N=CC=C3)C3=C2, 47.8
O=C1O[I](Cl)C2=CC=C3C(N=CC=C3)=C21, [Cl], O=C1O[I]C2=CC=C3C(N=CC=C3)=C21, 45.1
Cl[I]1OC(C2=CC=C3C(N=CC=C3)=C21)=O, [Cl], [I]1OC(C2=CC=C3C(N=CC=C3)=C21)=O, 37.7
O=C1O[I](Cl)C2=C1C=CN2S(C)(=O)=O, [Cl], O=C1O[I]C2=C1C=CN2S(C)(=O)=O, 28.5
Cl[I]1OC(C2=C1C=CN2S(C)(=O)=O)=O, [Cl], [I]1OC(C2=C1C=CN2S(C)(=O)=O)=O, 43.6
O=C1O[I](Cl)C2=C1C=CN2C, [Cl], O=C1O[I]C2=C1C=CN2C, 35.4
Cl[I]1OC(C2=C1C=CN2C)=O, [Cl], [I]1OC(C2=C1C=CN2C)=O, 43.9
O=C1O[I](Cl)C2=C1C=CN2C(C)=O, [Cl], O=C1O[I]C2=C1C=CN2C(C)=O, 33.3
Cl[I]1OC(C2=C1C=CN2C(C)=O)=O, [Cl], [I]1OC(C2=C1C=CN2C(C)=O)=O, 42.8
O=C1O[I](Cl)C2=CN(S(C)(=O)=O)C=C21, [Cl], O=C1O[I]C2=CN(S(C)(=O)=O)C=C21, 41.8
O=C1O[I](Cl)C2=CN(C)C=C21, [Cl], O=C1O[I]C2=CN(C)C=C21, 42.9
O=C1O[I](Cl)C2=CN(C(C)=O)C=C21, [Cl], O=C1O[I]C2=CN(C(C)=O)C=C21, 41.8
[H]N1C=CC2=C1[I](Cl)OC2=O, [Cl], [H]N1C=CC2=C1[I]OC2=O, 40.0
[H]N1C=C2C([I](Cl)OC2=O)=C1, [Cl], [H]N1C=C2C([I]OC2=O)=C1, 42.3
[H]N1C=CC2=C1C(O[I]2Cl)=O, [Cl], [H]N1C=CC2=C1C(O[I]2)=O, 42.7
Cl[I](O1)C2=NC=NC=C2C1=O, [Cl], [I](O1)C2=NC=NC=C2C1=O, 44.6
Cl[I](O1)C2=NN=CC=C2C1=O, [Cl], [I](O1)C2=NN=CC=C2C1=O, 43.9
Cl[I](O1)C2=CN=NC=C2C1=O, [Cl], [I](O1)C2=CN=NC=C2C1=O, 45.0
Cl[I](O1)C2=CC=NN=C2C1=O, [Cl], [I](O1)C2=CC=NN=C2C1=O, 45.0
Cl[I](O1)C2=NC=CN=C2C1=O, [Cl], [I](O1)C2=NC=CN=C2C1=O, 43.9
Cl[I](O1)C2=CC=CN=C2C1=O, [Cl], [I](O1)C2=CC=CN=C2C1=O, 46.0
Cl[I](O1)C2=CC=NC=C2C1=O, [Cl], [I](O1)C2=CC=NC=C2C1=O, 46.6
Cl[I](O1)C2=CN=CC=C2C1=O, [Cl], [I](O1)C2=CN=CC=C2C1=O, 46.0

Cl[I](O1)C2=NC=CC=C2C1=O, [Cl], [I](O1)C2=NC=CC=C2C1=O, 45.9
Cl[I](OC1=O)C2=C(C1=CC=C3)C3=CC=C2, [Cl], [I](OC1=O)C2=C(C1=CC=C3)C3=CC=C2, 49.3
O=C1O[I](Cl)C2=CC3=CC=CC=C3C=C21, [Cl], O=C1O[I]C2=CC3=CC=CC=C3C=C21, 47.9
Cl[I](OC1=O)C2=C1C3=CC=CC=C3C=C2, [Cl], [I](OC1=O)C2=C1C3=CC=CC=C3C=C2, 49.5
Cl[I](OC1=O)C2=C1C=CC3=CC=CC=C32, [Cl], [I](OC1=O)C2=C1C=CC3=CC=CC=C32, 39.9
Cl[I](OC1=O)C2=C1C3=CC=CC=C3N2S(C)(=O)=O, [Cl],
[I](OC1=O)C2=C1C3=CC=CC=C3N2S(C)(=O)=O, 33.7
O=C(O[I]1Cl)C2=C1C3=CC=CC=C3N2S(C)(=O)=O, [Cl],
O=C(O[I]1)C2=C1C3=CC=CC=C3N2S(C)(=O)=O, 31.0
Cl[I](OC1=O)C2=C1C3=CC=CC=C3N2C, [Cl], [I](OC1=O)C2=C1C3=CC=CC=C3N2C, 35.1
O=C(O[I]1Cl)C2=C1C3=CC=CC=C3N2C, [Cl], O=C(O[I]1)C2=C1C3=CC=CC=C3N2C, 43.5
[H]N1C2=CC=CC=C2C3=C1[I](Cl)OC3=O, [Cl], [H]N1C2=CC=CC=C2C3=C1[I]OC3=O, 40.3
[H]N1C2=CC=CC=C2C3=C1C(O[I]3Cl)=O, [Cl], [H]N1C2=CC=CC=C2C3=C1C(O[I]3)=O, 41.9
Cl[I](OC1=O)C2=C1C3=CC=CC=C3N2C(C)=O, [Cl], [I](OC1=O)C2=C1C3=CC=CC=C3N2C(C)=O,
35.9
O=C(O[I]1Cl)C2=C1C3=CC=CC=C3N2C(C)=O, [Cl], O=C(O[I]1)C2=C1C3=CC=CC=C3N2C(C)=O,
41.1
O=C1O[I](Cl)C2=C1C=CO2, [Cl], O=C1O[I]C2=C1C=CO2, 34.9
O=C1O[I](Cl)C2=COC=C21, [Cl], O=C1O[I]C2=COC=C21, 40.6
Cl[I]1OC(C2=C1C=CO2)=O, [Cl], [I]1OC(C2=C1C=CO2)=O, 40.8
O=C(O[I]1Cl)C2=C(C1=CC=C3)C3=CC4=CC=CC=C42, [Cl],
O=C(O[I]1)C2=C(C1=CC=C3)C3=CC4=CC=CC=C42, 50.0
Cl[I](OC1=O)C2=C(C1=CC=C3)C3=CC4=CC=CC=C42, [Cl],
[I](OC1=O)C2=C(C1=CC=C3)C3=CC4=CC=CC=C42, 47.6
Cl[I](OC1=O)C2=C1C=CC3=CC4=CC=CC=C4C=C32, [Cl],
[I](OC1=O)C2=C1C=CC3=CC4=CC=CC=C4C=C32, 40.2
Cl[I]1OC(C2=CC3=CC4=CC=CC=C4C=C3C=C21)=O, [Cl],
[I]1OC(C2=CC3=CC4=CC=CC=C4C=C3C=C21)=O, 47.9
O=C(O[I]1Cl)C2=C1C=CC3=CC4=CC=CC=C4C=C32, [Cl],
O=C(O[I]1)C2=C1C=CC3=CC4=CC=CC=C4C=C32, 49.4
O=C(O[I]1C(F)(F)F)C2=C1SC=C2, F[C](F)F, O=C(O[I]1)C2=C1SC=C2, 26.7
O=C1C2=CSC=C2[I](C(F)(F)F)O1, F[C](F)F, O=C1C2=CSC=C2[I]O1, 28.9
O=C1O[I](C(F)(F)F)C2=C1SC=C2, F[C](F)F, O=C1O[I]C2=C1SC=C2, 29.9
O=C1O[I](C(F)(F)F)C2=NC3=CC=CC=C3C=C21, F[C](F)F, O=C3O[I]C4=NC5=CC=CC=C5C=C43,
34.2
O=C(O[I]1C(F)(F)F)C2=C1C3=CC=CC=C3N=C2, F[C](F)F, O=C(O[I]1)C2=C1C3=CC=CC=C3N=C2,
23.9
O=C1O[I](C(F)(F)F)C2=CC=NC3=CC=CC1=C32, F[C](F)F, [I](OC1=O)C2=CC=NC3=CC=CC1=C32,
31.6
O=C(O[I]1C(F)(F)F)C2=CC=NC3=CC=CC1=C32, F[C](F)F, O=C(O[I]1)C2=CC=NC3=CC=CC1=C32,
33.2
O=C1O[I](C(F)(F)F)C2=C1C3=CC=CC=C3N=C2, F[C](F)F, [I](OC1=O)C2=C1C3=CC=CC=C3N=C2,
32.0
O=C1C2=NC3=CC=CC=C3C=C2[I](C(F)(F)F)O1, F[C](F)F, [I]1OC(C2=NC3=CC=CC=C3C=C21)=O,
29.0
O=C1O[I](C(F)(F)F)C2=C3C(N=CC=C3)=CC=C21, F[C](F)F,
[I](O1)C2=C3C(N=CC=C3)=CC=C2C1=O, 24.4
O=C1C2=C3C(N=CC=C3)=CC=C2[I](C(F)(F)F)O1, F[C](F)F,
O=C1C2=C3C(N=CC=C3)=CC=C2[I]O1, 32.5
O=C(O[I]1C(F)(F)F)C2=C1C=C(N=CC=C3)C3=C2, F[C](F)F,
O=C(O[I]1)C2=C1C=C(N=CC=C3)C3=C2, 31.4

O=C1O[I](C(F)(F)F)C2=C1C=C(N=CC=C3)C3=C2, F[C](F)F,
[I](OC1=O)C2=C1C=C(N=CC=C3)C3=C2, 31.1
O=C1O[I](C(F)(F)F)C2=CC=C3C(N=CC=C3)=C21, F[C](F)F,
O=C1O[I]C2=CC=C3C(N=CC=C3)=C21, 26.8
O=C1C2=CC=C3C(N=CC=C3)=C2[I](C(F)(F)F)O1, F[C](F)F,
[I]1OC(C2=CC=C3C(N=CC=C3)=C21)=O, 28.6
O=C1O[I](C(F)(F)F)C2=C1C=CN2S(C)(=O)=O, F[C](F)F, O=C1O[I]C2=C1C=CN2S(C)(=O)=O, 18.3
O=C(O[I]1C(F)(F)F)C2=C1C=CN2S(C)(=O)=O, F[C](F)F, [I]1OC(C2=C1C=CN2S(C)(=O)=O)=O, 29.6
O=C1O[I](C(F)(F)F)C2=C1C=CN2C, F[C](F)F, O=C1O[I]C2=C1C=CN2C, 17.3
O=C(O[I]1C(F)(F)F)C2=C1C=CN2C, F[C](F)F, [I]1OC(C2=C1C=CN2C)=O, 30.5
O=C1O[I](C(F)(F)F)C2=C1C=CN2C(C)=O, F[C](F)F, O=C1O[I]C2=C1C=CN2C(C)=O, 21.1
O=C(O[I]1C(F)(F)F)C2=C1C=CN2C(C)=O, F[C](F)F, [I]1OC(C2=C1C=CN2C(C)=O)=O, 28.2
O=C1O[I](C(F)(F)F)C2=CN(S(C)(=O)=O)C=C21, F[C](F)F, O=C1O[I]C2=CN(S(C)(=O)=O)C=C21,
27.4
O=C1O[I](C(F)(F)F)C2=CN(C)C=C21, F[C](F)F, O=C1O[I]C2=CN(C)C=C21, 27.0
O=C1O[I](C(F)(F)F)C2=CN(C(C)=O)C=C21, F[C](F)F, O=C1O[I]C2=CN(C(C)=O)C=C21, 27.7
[H]N1C=CC2=C1[I](C(F)(F)F)OC2=O, F[C](F)F, [H]N1C=CC2=C1[I]OC2=O, 22.3
[H]N1C=C2C([I](C(F)(F)F)OC2=O)=C1, F[C](F)F, [H]N1C=C2C([I]OC2=O)=C1, 27.1
[H]N1C=CC2=C1C(O[I]2C(F)(F)F)=O, F[C](F)F, [H]N1C=CC2=C1C(O[I]2)=O, 29.6
O=C1O[I](C(F)(F)F)C2=NC=NC=C21, F[C](F)F, [I](O1)C2=NC=NC=C2C1=O, 33.2
O=C1O[I](C(F)(F)F)C2=NN=CC=C21, F[C](F)F, [I](O1)C2=NN=CC=C2C1=O, 33.9
O=C1O[I](C(F)(F)F)C2=CN=NC=C21, F[C](F)F, [I](O1)C2=CN=NC=C2C1=O, 30.9
O=C1O[I](C(F)(F)F)C2=CC=NN=C21, F[C](F)F, [I](O1)C2=CC=NN=C2C1=O, 28.2
O=C1O[I](C(F)(F)F)C2=NC=CN=C21, F[C](F)F, [I](O1)C2=NC=CN=C2C1=O, 31.3
O=C1O[I](C(F)(F)F)C2=CC=CN=C21, F[C](F)F, [I](O1)C2=CC=CN=C2C1=O, 28.8
O=C1O[I](C(F)(F)F)C2=CC=NC=C21, F[C](F)F, [I](O1)C2=CC=NC=C2C1=O, 30.8
O=C1O[I](C(F)(F)F)C2=CN=CC=C21, F[C](F)F, [I](O1)C2=CN=CC=C2C1=O, 31.3
O=C1O[I](C(F)(F)F)C2=NC=CC=C21, F[C](F)F, [I](O1)C2=NC=CC=C2C1=O, 34.1
O=C1O[I](C(F)(F)F)C2=C(C1=CC=C3)C3=CC=C2, F[C](F)F,
[I](OC1=O)C2=C(C1=CC=C3)C3=CC=C2, 32.8
O=C1O[I](C(F)(F)F)C2=CC3=CC=CC=C3C=C21, F[C](F)F, O=C1O[I]C2=CC3=CC=CC=C3C=C21,
31.3
O=C1O[I](C(F)(F)F)C2=C1C3=CC=CC=C3C=C2, F[C](F)F, [I](OC1=O)C2=C1C3=CC=CC=C3C=C2,
32.4
O=C1O[I](C(F)(F)F)C2=C1C=CC3=CC=CC=C32, F[C](F)F, [I](OC1=O)C2=C1C=CC3=CC=CC=C32,
24.6
O=C1O[I](C(F)(F)F)C2=C1C3=CC=CC=C3N2S(C)(=O)=O, F[C](F)F,
[I](OC1=O)C2=C1C3=CC=CC=C3N2S(C)(=O)=O, 23.4
O=C(O[I]1C(F)(F)F)C2=C1C3=CC=CC=C3N2S(C)(=O)=O, F[C](F)F,
O=C(O[I]1)C2=C1C3=CC=CC=C3N2S(C)(=O)=O, 15.3
O=C1O[I](C(F)(F)F)C2=C1C3=CC=CC=C3N2C, F[C](F)F, [I](OC1=O)C2=C1C3=CC=CC=C3N2C,
17.5
O=C(O[I]1C(F)(F)F)C2=C1C3=CC=CC=C3N2C, F[C](F)F, O=C(O[I]1)C2=C1C3=CC=CC=C3N2C,
29.4
[H]N1C2=CC=CC=C2C3=C1[I](C(F)(F)F)OC3=O, F[C](F)F, [H]N1C2=CC=CC=C2C3=C1[I]OC3=O,
22.6
[H]N1C2=CC=CC=C2C3=C1C(O[I]3C(F)(F)F)=O, F[C](F)F,
[H]N1C2=CC=CC=C2C3=C1C(O[I]3)=O, 28.4
O=C1O[I](C(F)(F)F)C2=C1C3=CC=CC=C3N2C(C)=O, F[C](F)F,
[I](OC1=O)C2=C1C3=CC=CC=C3N2C(C)=O, 23.5
O=C(O[I]1C(F)(F)F)C2=C1C3=CC=CC=C3N2C(C)=O, F[C](F)F,

O=C(O[I]1)C2=C1C3=CC=CC=C3N2C(C)=O, 32.5
O=C1O[I](C(F)(F)F)C2=C1C=CO2, F[C](F)F, O=C1O[I]C2=C1C=CO2, 22.1
O=C1O[I](C(F)(F)F)C2=COC=C21, F[C](F)F, O=C1O[I]C2=COC=C21, 26.5
O=C(O[I]1C(F)(F)F)C2=C1C=CO2, F[C](F)F, [I]1OC(C2=C1C=CO2)=O, 27.3
O=C(O[I]1C(F)(F)F)C2=C(C1=CC=C3)C3=CC4=CC=CC=C42, F[C](F)F,
O=C(O[I]1)C2=C(C1=CC=C3)C3=CC4=CC=CC=C42, 33.3
O=C1O[I](C(F)(F)F)C2=C(C1=CC=C3)C3=CC4=CC=CC=C42, F[C](F)F,
[I](OC1=O)C2=C(C1=CC=C3)C3=CC4=CC=CC=C42, 31.5
O=C1O[I](C(F)(F)F)C2=C1C=CC3=CC4=CC=CC=C4C=C32, F[C](F)F,
[I](OC1=O)C2=C1C=CC3=CC4=CC=CC=C4C=C32, 24.7
O=C1C2=CC3=CC4=CC=CC=C4C=C3C=C2[I](C(F)(F)F)O1, F[C](F)F,
[I]1OC(C2=CC3=CC4=CC=CC=C4C=C3C=C21)=O, 31.2
O=C(O[I]1C(F)(F)F)C2=C1C=CC3=CC4=CC=CC=C4C=C32, F[C](F)F,
O=C(O[I]1)C2=C1C=CC3=CC4=CC=CC=C4C=C32, 32.3
O=C(O[I]1C#C[Si](C)(C)C)C2=C1SC=C2, [C]#C[Si](C)(C)C, O=C(O[I]1)C2=C1SC=C2, 62.2
O=C1C2=CSC=C2[I](C#C[Si](C)(C)C)O1, [C]#C[Si](C)(C)C, O=C1C2=CSC=C2[I]O1, 63.9
O=C1O[I](C#C[Si](C)(C)C)C2=C1SC=C2, [C]#C[Si](C)(C)C, O=C1O[I]C2=C1SC=C2, 64.7
O=C1O[I](C#C[Si](C)(C)C)C2=NC3=CC=CC=C3C=C21, [C]#C[Si](C)(C)C,
O=C3O[I]C4=NC5=CC=CC=C5C=C43, 67.9
O=C(O[I]1C#C[Si](C)(C)C)C2=C1C3=CC=CC=C3N=C2, [C]#C[Si](C)(C)C,
O=C(O[I]1)C2=C1C3=CC=CC=C3N=C2, 60.3
O=C1O[I](C#C[Si](C)(C)C)C2=CC=NC3=CC=CC1=C32, [C]#C[Si](C)(C)C,
[I](OC1=O)C2=CC=NC3=CC=CC1=C32, 66.8
O=C(O[I]1C#C[Si](C)(C)C)C2=CC=NC3=CC=CC1=C32, [C]#C[Si](C)(C)C,
O=C(O[I]1)C2=CC=NC3=CC=CC1=C32, 68.2
O=C1O[I](C#C[Si](C)(C)C)C2=C1C3=CC=CC=C3N=C2, [C]#C[Si](C)(C)C,
[I](OC1=O)C2=C1C3=CC=CC=C3N=C2, 68.1
O=C1C2=NC3=CC=CC=C3C=C2[I](C#C[Si](C)(C)C)O1, [C]#C[Si](C)(C)C,
[I]1OC(C2=NC3=CC=CC=C3C=C21)=O, 65.3
O=C1O[I](C#C[Si](C)(C)C)C2=C3C(N=CC=C3)=CC=C21, [C]#C[Si](C)(C)C,
[I](O1)C2=C3C(N=CC=C3)=CC=C2C1=O, 60.8
O=C1C2=C3C(N=CC=C3)=CC=C2[I](C#C[Si](C)(C)C)O1, [C]#C[Si](C)(C)C,
O=C1C2=C3C(N=CC=C3)=CC=C2[I]O1, 68.6
O=C(O[I]1C#C[Si](C)(C)C)C2=C1C=C(N=CC=C3)C3=C2, [C]#C[Si](C)(C)C,
O=C(O[I]1)C2=C1C=C(N=CC=C3)C3=C2, 67.8
O=C1O[I](C#C[Si](C)(C)C)C2=C1C=C(N=CC=C3)C3=C2, [C]#C[Si](C)(C)C,
[I](OC1=O)C2=C1C=C(N=CC=C3)C3=C2, 67.3
O=C1O[I](C#C[Si](C)(C)C)C2=CC=C3C(N=CC=C3)=C21, [C]#C[Si](C)(C)C,
O=C1O[I]C2=CC=C3C(N=CC=C3)=C21, 62.7
O=C1C2=CC=C3C(N=CC=C3)=C2[I](C#C[Si](C)(C)C)O1, [C]#C[Si](C)(C)C,
[I]1OC(C2=CC=C3C(N=CC=C3)=C21)=O, 60.2
O=C1O[I](C#C[Si](C)(C)C)C2=C1C=CN2S(C)(=O)=O, [C]#C[Si](C)(C)C,
O=C1O[I]C2=C1C=CN2S(C)(=O)=O, 53.2
O=C(O[I]1C#C[Si](C)(C)C)C2=C1C=CN2S(C)(=O)=O, [C]#C[Si](C)(C)C,
[I]1OC(C2=C1C=CN2S(C)(=O)=O)=O, 63.7
O=C1O[I](C#C[Si](C)(C)C)C2=C1C=CN2C, [C]#C[Si](C)(C)C, O=C1O[I]C2=C1C=CN2C, 53.7
O=C(O[I]1C#C[Si](C)(C)C)C2=C1C=CN2C, [C]#C[Si](C)(C)C, [I]1OC(C2=C1C=CN2C)=O, 64.3
O=C1O[I](C#C[Si](C)(C)C)C2=C1C=CN2C(C)=O, [C]#C[Si](C)(C)C,
O=C1O[I]C2=C1C=CN2C(C)=O, 55.1
O=C(O[I]1C#C[Si](C)(C)C)C2=C1C=CN2C(C)=O, [C]#C[Si](C)(C)C,
[I]1OC(C2=C1C=CN2C(C)=O)=O, 62.2

O=C1O[I](C#C[Si](C)(C)C)C2=CN(S(C)(=O)=O)C=C21, [C]#C[Si](C)(C)C,
O=C1O[I]C2=CN(S(C)(=O)=O)C=C21, 62.2
O=C1O[I](C#C[Si](C)(C)C)C2=CN(C)C=C21, [C]#C[Si](C)(C)C, O=C1O[I]C2=CN(C)C=C21, 60.7
O=C1O[I](C#C[Si](C)(C)C)C2=CN(C(C)=O)C=C21, [C]#C[Si](C)(C)C,
O=C1O[I]C2=CN(C(C)=O)C=C21, 62.0
[H]N1C=CC2=C1[I](C#C[Si](C)(C)C)OC2=O, [C]#C[Si](C)(C)C, [H]N1C=CC2=C1[I]OC2=O, 57.4
[H]N1C=C2C([I](C#C[Si](C)(C)C)OC2=O)=C1, [C]#C[Si](C)(C)C, [H]N1C=C2C([I]OC2=O)=C1, 60.8
[H]N1C=CC2=C1C(O[I]2C#C[Si](C)(C)C)=O, [C]#C[Si](C)(C)C, [H]N1C=CC2=C1C(O[I]2)=O, 63.3
O=C1O[I](C#C[Si](C)(C)C)C2=NC=NC=C21, [C]#C[Si](C)(C)C, [I](O1)C2=NC=NC=C2C1=O, 67.6
O=C1O[I](C#C[Si](C)(C)C)C2=NN=CC=C21, [C]#C[Si](C)(C)C, [I](O1)C2=NN=CC=C2C1=O, 68.2
O=C1O[I](C#C[Si](C)(C)C)C2=CN=NC=C21, [C]#C[Si](C)(C)C, [I](O1)C2=CN=NC=C2C1=O, 67.6
O=C1O[I](C#C[Si](C)(C)C)C2=CC=NN=C21, [C]#C[Si](C)(C)C, [I](O1)C2=CC=NN=C2C1=O, 65.0
O=C1O[I](C#C[Si](C)(C)C)C2=NC=CN=C21, [C]#C[Si](C)(C)C, [I](O1)C2=NC=CN=C2C1=O, 65.5
O=C1O[I](C#C[Si](C)(C)C)C2=CC=CN=C21, [C]#C[Si](C)(C)C, [I](O1)C2=CC=CN=C2C1=O, 64.7
O=C1O[I](C#C[Si](C)(C)C)C2=CC=NC=C21, [C]#C[Si](C)(C)C, [I](O1)C2=CC=NC=C2C1=O, 67.0
O=C1O[I](C#C[Si](C)(C)C)C2=CN=CC=C21, [C]#C[Si](C)(C)C, [I](O1)C2=CN=CC=C2C1=O, 67.4
O=C1O[I](C#C[Si](C)(C)C)C2=NC=CC=C21, [C]#C[Si](C)(C)C, [I](O1)C2=NC=CC=C2C1=O, 67.9
O=C1O[I](C#C[Si](C)(C)C)C2=C(C1=CC=C3)C3=CC=C2, [C]#C[Si](C)(C)C,
[I](OC1=O)C2=C(C1=CC=C3)C3=CC=C2, 67.4
O=C1O[I](C#C[Si](C)(C)C)C2=CC3=CC=CC=C3C=C21, [C]#C[Si](C)(C)C,
O=C1O[I]C2=CC3=CC=CC=C3C=C21, 66.9
O=C1O[I](C#C[Si](C)(C)C)C2=C1C3=CC=CC=C3C=C2, [C]#C[Si](C)(C)C,
[I](OC1=O)C2=C1C3=CC=CC=C3C=C2, 68.2
O=C1O[I](C#C[Si](C)(C)C)C2=C1C=CC3=CC=CC=C32, [C]#C[Si](C)(C)C,
[I](OC1=O)C2=C1C=CC3=CC=CC=C32, 60.2
O=C1O[I](C#C[Si](C)(C)C)C2=C1C3=CC=CC=C3N2S(C)(=O)=O, [C]#C[Si](C)(C)C,
[I](OC1=O)C2=C1C3=CC=CC=C3N2S(C)(=O)=O, 59.0
O=C(O[I]1C#C[Si](C)(C)C)C2=C1C3=CC=CC=C3N2S(C)(=O)=O, [C]#C[Si](C)(C)C,
O=C(O[I]1)C2=C1C3=CC=CC=C3N2S(C)(=O)=O, 51.8
O=C1O[I](C#C[Si](C)(C)C)C2=C1C3=CC=CC=C3N2C, [C]#C[Si](C)(C)C,
[I](OC1=O)C2=C1C3=CC=CC=C3N2C, 53.8
O=C(O[I]1C#C[Si](C)(C)C)C2=C1C3=CC=CC=C3N2C, [C]#C[Si](C)(C)C,
O=C(O[I]1)C2=C1C3=CC=CC=C3N2C, 64.6
[H]N1C2=CC=CC=C2C3=C1[I](C#C[Si](C)(C)C)OC3=O, [C]#C[Si](C)(C)C,
[H]N1C2=CC=CC=C2C3=C1[I]OC3=O, 58.0
[H]N1C2=CC=CC=C2C3=C1C(O[I]3C#C[Si](C)(C)C)=O, [C]#C[Si](C)(C)C,
[H]N1C2=CC=CC=C2C3=C1C(O[I]3)=O, 63.4
O=C1O[I](C#C[Si](C)(C)C)C2=C1C3=CC=CC=C3N2C(C)=O, [C]#C[Si](C)(C)C,
[I](OC1=O)C2=C1C3=CC=CC=C3N2C(C)=O, 57.8
O=C(O[I]1C#C[Si](C)(C)C)C2=C1C3=CC=CC=C3N2C(C)=O, [C]#C[Si](C)(C)C,
O=C(O[I]1)C2=C1C3=CC=CC=C3N2C(C)=O, 68.9
O=C1O[I](C#C[Si](C)(C)C)C2=C1C=CO2, [C]#C[Si](C)(C)C, O=C1O[I]C2=C1C=CO2, 56.1
O=C1O[I](C#C[Si](C)(C)C)C2=COC=C21, [C]#C[Si](C)(C)C, O=C1O[I]C2=COC=C21, 60.7
O=C(O[I]1C#C[Si](C)(C)C)C2=C1C=CO2, [C]#C[Si](C)(C)C, [I]1OC(C2=C1C=CO2)=O, 61.4
O=C(O[I]1C#C[Si](C)(C)C)C2=C(C1=CC=C3)C3=CC4=CC=CC=C42, [C]#C[Si](C)(C)C,
O=C(O[I]1)C2=C(C1=CC=C3)C3=CC4=CC=CC=C42, 67.5
O=C1O[I](C#C[Si](C)(C)C)C2=C(C1=CC=C3)C3=CC4=CC=CC=C42, [C]#C[Si](C)(C)C,
[I](OC1=O)C2=C(C1=CC=C3)C3=CC4=CC=CC=C42, 66.7
O=C1O[I](C#C[Si](C)(C)C)C2=C1C=CC3=CC4=CC=CC=C4C=C32, [C]#C[Si](C)(C)C,
[I](OC1=O)C2=C1C=CC3=CC4=CC=CC=C4C=C32, 61.2
O=C1C2=CC3=CC4=CC=CC=C4C=C3C=C2[I](C#C[Si](C)(C)C)O1, [C]#C[Si](C)(C)C,

[I]1OC(C2=CC3=CC4=CC=CC=C4C=C3C=C21)=O, 66.8
O=C(O[I]1C#C[Si](C)(C)C)C2=C1C=CC3=CC4=CC=CC=C4C=C32, [C]#C[Si](C)(C)C,
O=C(O[I]1)C2=C1C=CC3=CC4=CC=CC=C4C=C32, 68.2
O=C(CC1=CC=C(C)C=C1)O[I](OC(CC2=CC=C(C)C=C2)=O)C3=CC=CC=C3,
[O]C(CC1=CC=C(C)C=C1)=O, O=C(CC1=CC=C(C)C=C1)O[I]C2=CC=CC=C2, 44.8
O=C(CC1=CC=CC=C1)O[I](OC(CC2=CC=CC=C2)=O)C3=CC=CC=C3, [O]C(CC1=CC=CC=C1)=O,
O=C(CC3=CC=CC=C3)O[I]C4=CC=CC=C4, 45.2
O=C(C[C@]4(C5)C[C@@H](CC5C6)C[C@@H]6C4)O[I](OC(C[C@]7(C8)C[C@@H](CC8C9)C[C@@H]9C7)=O)C%10=CC=CC=C%10, [O]C(CC1(C2)C[C@@H](CC2C3)C[C@@H]3C1)=O,
O=C(CC5(C6)C[C@@H](CC6C7)C[C@@H]7C5)O[I]C8=CC=CC=C8, 45.4
O=C(C(C)C)O[I](OC(C(C)C)=O)C%11=CC=CC=C%11, [O]C(C(C)C)=O,
O=C(C(C)C)O[I]C9=CC=CC=C9, 45.5
O=C(C(C)(C)C)O[I](OC(C(C)(C)C)=O)C%12=CC=CC=C%12, [O]C(C(C)(C)C)=O,
O=C(C(C)(C)C)O[I]C%10=CC=CC=C%10, 46.3
CC(O[I](OC(C)=O)C%13=CC=CC=C%13)=O, [O]C(C)=O, CC(O[I]C%11=CC=CC=C%11)=O, 46.6
O=C(C%14=CC=CC=C%14)O[I](OC(C%15=CC=CC=C%15)=O)C%16=CC=CC=C%16,
[O]C(C1=CC=CC=C1)=O, O=C(C%12=CC=CC=C%12)O[I]C%13=CC=CC=C%13, 48.4
O=C(C(Cl)(Cl)Cl)O[I](OC(C(Cl)(Cl)Cl)=O)C1=CC=CC=C1, [O]C(C(Cl)(Cl)Cl)=O,
O=C(C(Cl)(Cl)Cl)O[I]C1=CC=CC=C1, 50.8
O=C(CC(F)(F)F)O[I](OC(CC(F)(F)F)=O)C2=CC=CC=C2, [O]C(CC(F)(F)F)=O,
O=C(CC(F)(F)F)O[I]C2=CC=CC=C2, 51.2
O=C(C(F)F)O[I](OC(C(F)F)=O)C3=CC=CC=C3, [O]C(C(F)F)=O, O=C(C(F)F)O[I]C3=CC=CC=C3,
51.4
O=C(C(F)(F)F)O[I](OC(C(F)(F)F)=O)C4=CC=CC=C4, [O]C(C(F)(F)F)=O,
O=C(C(F)(F)F)O[I]C4=CC=CC=C4, 53.0
Cl[I](Cl)C5=CC=CC=C5, [Cl], Cl[I]C5=CC=CC=C5, 42.7
F[I](F)C6=CC=CC=C6, [F], F[I]C6=CC=CC=C6, 79.3
O[I](OS(C7=CC=C(C=C7)C)(=O)=O)C8=CC=CC=C8, [OH],
CC7=CC=C(S(O[I]C8=CC=CC=C8)(=O)=O)C=C7, 49.6
O[I](OS(C9=CC=C(C=C9)C)(=O)=O)C%10=CC=CC=C%10, [O]S(C1=CC=C(C)C=C1)(=O)=O,
O[I]C9=CC=CC=C9, 51.9
F[B-](F)(F)F.C1([I+]C2=CC=CC=C2)=CC=CC=C1, C1=CC=CC=C1,
[I+]C1=CC=CC=C1.F[B-](F)(F)F, 68.2
CC(O[I](OC(C)=O)C1=CC=C(OC)C=C1)=O, [O]C(C)=O, CC(O[I]C1=CC=C(OC)C=C1)=O, 46.7
CC(O[I](OC(C)=O)C2=CC(OC)=CC=C2)=O, [O]C(C)=O, CC(O[I]C2=CC(OC)=CC=C2)=O, 46.6
CC(O[I](OC(C)=O)C3=C(OC)C=CC=C3)=O, [O]C(C)=O, CC(O[I]C3=C(OC)C=CC=C3)=O, 46.0
CC(O[I](OC(C)=O)C4=CC=C(C)C=C4)=O, [O]C(C)=O, CC(O[I]C4=CC=C(C)C=C4)=O, 46.7
CC(O[I](OC(C)=O)C5=CC(C)=CC=C5)=O, [O]C(C)=O, CC(O[I]C5=CC(C)=CC=C5)=O, 46.7
CC(O[I](OC(C)=O)C6=C(C)C=CC=C6)=O, [O]C(C)=O, CC(O[I]C6=C(C)C=CC=C6)=O, 46.8
CC(O[I](OC(C)=O)C1=CC=C(C(C)C)C=C1)=O, [O]C(C)=O, CC(O[I]C7=CC=C(C(C)C)C=C7)=O,
46.8
CC(O[I](OC(C)=O)C2=CC(C(C)C)=CC=C2)=O, [O]C(C)=O, CC(O[I]C8=CC(C(C)C)=CC=C8)=O,
46.7
CC(O[I](OC(C)=O)C3=C(C(C)C)C=CC=C3)=O, [O]C(C)=O, CC(O[I]C9=C(C(C)C)C=CC=C9)=O,
47.2
CC(O[I](OC(C)=O)C4=CC=C(C(C)(C)C)C=C4)=O, [O]C(C)=O,
CC(O[I]C%10=CC=C(C(C)(C)C)C=C%10)=O, 46.8
CC(O[I](OC(C)=O)C5=CC(C(C)(C)C)=CC=C5)=O, [O]C(C)=O,
CC(O[I]C%11=CC(C(C)(C)C)=CC=C%11)=O, 46.9
CC(O[I](OC(C)=O)C6=C(C(C)(C)C)C=CC=C6)=O, [O]C(C)=O,
CC(O[I]C%12=C(C(C)(C)C)C=CC=C%12)=O, 46.8

CC(O[I](OC(C)=O)C1=CC=C(C(F)(F)F)C=C1)=O, [O]C(C)=O,
CC(O[I]C%13=CC=C(C(F)(F)F)C=C%13)=O, 46.4
CC(O[I](OC(C)=O)C2=CC(C(F)(F)F)=CC=C2)=O, [O]C(C)=O,
CC(O[I]C%14=CC(C(F)(F)F)=CC=C%14)=O, 46.5
CC(O[I](OC(C)=O)C3=C(C(F)(F)F)C=CC=C3)=O, [O]C(C)=O,
CC(O[I]C%15=C(C(F)(F)F)C=CC=C%15)=O, 45.3
CC(O[I](OC(C)=O)C4=CC=C(C#N)C=C4)=O, [O]C(C)=O, CC(O[I]C%16=CC=C(C#N)C=C%16)=O, 46.2
CC(O[I](OC(C)=O)C5=CC(C#N)=CC=C5)=O, [O]C(C)=O, CC(O[I]C%17=CC(C#N)=CC=C%17)=O, 46.4
CC(O[I](OC(C)=O)C6=C(C#N)C=CC=C6)=O, [O]C(C)=O, CC(O[I]C%18=C(C#N)C=CC=C%18)=O, 45.4
CC(O[I](OC(C)=O)C7=CC=C(C(OC)=O)C=C7)=O, [O]C(C)=O,
CC(O[I]C%19=CC=C(C(OC)=O)C=C%19)=O, 46.4
CC(O[I](OC(C)=O)C8=CC(C(OC)=O)=CC=C8)=O, [O]C(C)=O,
CC(O[I]C%20=CC(C(OC)=O)=CC=C%20)=O, 46.6
CC(O[I](OC(C)=O)C9=C(C(OC)=O)C=CC=C9)=O, [O]C(C)=O,
CC(O[I]C%21=C(C(OC)=O)C=CC=C%21)=O, 45.7
CC(O[I](OC(C)=O)C%10=CC=C([N+]([O-])=O)C=C%10)=O, [O]C(C)=O,
CC(O[I]C%22=CC=C([N+]([O-])=O)C=C%22)=O, 46.1
CC(O[I](OC(C)=O)C%11=CC([N+]([O-])=O)=CC=C%11)=O, [O]C(C)=O,
CC(O[I]C%23=CC([N+]([O-])=O)=CC=C%23)=O, 46.4
CC(O[I](OC(C)=O)C%12=C([N+]([O-])=O)C=CC=C%12)=O, [O]C(C)=O,
CC(O[I]C%24=C([N+]([O-])=O)C=CC=C%24)=O, 45.5
CC(O[I](OC(C)=O)C%13=CC=C(F)C=C%13)=O, [O]C(C)=O, CC(O[I]C%25=CC=C(F)C=C%25)=O, 46.6
CC(O[I](OC(C)=O)C%14=CC(F)=CC=C%14)=O, [O]C(C)=O, CC(O[I]C%26=CC(F)=CC=C%26)=O, 46.3
CC(O[I](OC(C)=O)C%15=C(F)C=CC=C%15)=O, [O]C(C)=O, CC(O[I]C%27=C(F)C=CC=C%27)=O, 45.6
CC(O[I](OC(C)=O)C%16=CC=C(Cl)C=C%16)=O, [O]C(C)=O,
CC(O[I]C%28=CC=C(Cl)C=C%28)=O, 46.5
CC(O[I](OC(C)=O)C%17=CC(Cl)=CC=C%17)=O, [O]C(C)=O,
CC(O[I]C%29=CC(Cl)=CC=C%29)=O, 46.4
CC(O[I](OC(C)=O)C%18=C(Cl)C=CC=C%18)=O, [O]C(C)=O,
CC(O[I]C%30=C(Cl)C=CC=C%30)=O, 45.2
CC(O[I](OC(C)=O)C%19=CC=C(Br)C=C%19)=O, [O]C(C)=O,
CC(O[I]C%31=CC=C(Br)C=C%31)=O, 46.5
CC(O[I](OC(C)=O)C%20=CC(Br)=CC=C%20)=O, [O]C(C)=O,
CC(O[I]C%32=CC(Br)=CC=C%32)=O, 46.4
CC(O[I](OC(C)=O)C%21=C(Br)C=CC=C%21)=O, [O]C(C)=O,
CC(O[I]C%33=C(Br)C=CC=C%33)=O, 45.1
O=C(C(F)(F)F)O[I](OC(C(F)(F)F)=O)C1=CC=C(OC)C=C1, [O]C(C(F)(F)F)=O,
O=C(C(F)(F)F)O[I]C1=CC=C(OC)C=C1, 53.1
O=C(C(F)(F)F)O[I](OC(C(F)(F)F)=O)C2=CC(OC)=CC=C2, [O]C(C(F)(F)F)=O,
O=C(C(F)(F)F)O[I]C2=CC(OC)=CC=C2, 51.9
O=C(C(F)(F)F)O[I](OC(C(F)(F)F)=O)C3=C(OC)C=CC=C3, [O]C(C(F)(F)F)=O,
O=C(C(F)(F)F)O[I]C3=C(OC)C=CC=C3, 53.0
O=C(C(F)(F)F)O[I](OC(C(F)(F)F)=O)C4=CC=C(C)C=C4, [O]C(C(F)(F)F)=O,
O=C(C(F)(F)F)O[I]C4=CC=C(C)C=C4, 52.8
O=C(C(F)(F)F)O[I](OC(C(F)(F)F)=O)C5=CC(C)=CC=C5, [O]C(C(F)(F)F)=O,

O=C(C(F)(F)F)O[I]C5=CC(C)=CC=C5, 52.7
O=C(C(F)(F)F)O[I](OC(C(F)(F)F)=O)C6=C(C)C=CC=C6, [O]C(C(F)(F)F)=O,
O=C(C(F)(F)F)O[I]C6=C(C)C=CC=C6, 53.7
O=C(C(F)(F)F)O[I](OC(C(F)(F)F)=O)C7=CC=C(C(C)C)C=C7, [O]C(C(F)(F)F)=O,
O=C(C(F)(F)F)O[I]C7=CC=C(C(C)C)C=C7, 53.4
O=C(C(F)(F)F)O[I](OC(C(F)(F)F)=O)C8=CC(C(C)C)=CC=C8, [O]C(C(F)(F)F)=O,
O=C(C(F)(F)F)O[I]C8=CC(C(C)C)=CC=C8, 54.0
O=C(C(F)(F)F)O[I](OC(C(F)(F)F)=O)C9=C(C(C)C)C=CC=C9, [O]C(C(F)(F)F)=O,
O=C(C(F)(F)F)O[I]C9=C(C(C)C)C=CC=C9, 53.2
O=C(C(F)(F)F)O[I](OC(C(F)(F)F)=O)C%10=CC=C(C(C)(C)C)C=C%10, [O]C(C(F)(F)F)=O,
O=C(C(F)(F)F)O[I]C%10=CC=C(C(C)(C)C)C=C%10, 53.4
O=C(C(F)(F)F)O[I](OC(C(F)(F)F)=O)C%11=CC(C(C)(C)C)=CC=C%11, [O]C(C(F)(F)F)=O,
O=C(C(F)(F)F)O[I]C%11=CC(C(C)(C)C)=CC=C%11, 54.5
O=C(C(F)(F)F)O[I](OC(C(F)(F)F)=O)C%12=C(C(C)(C)C)C=CC=C%12, [O]C(C(F)(F)F)=O,
O=C(C(F)(F)F)O[I]C%12=C(C(C)(C)C)C=CC=C%12, 54.8
O=C(C(F)(F)F)O[I](OC(C(F)(F)F)=O)C%13=CC=C(C(F)(F)F)C=C%13, [O]C(C(F)(F)F)=O,
O=C(C(F)(F)F)O[I]C%13=CC=C(C(F)(F)F)C=C%13, 52.5
O=C(C(F)(F)F)O[I](OC(C(F)(F)F)=O)C%14=CC(C(F)(F)F)=CC=C%14, [O]C(C(F)(F)F)=O,
O=C(C(F)(F)F)O[I]C%14=CC(C(F)(F)F)=CC=C%14, 52.1
O=C(C(F)(F)F)O[I](OC(C(F)(F)F)=O)C%15=C(C(F)(F)F)C=CC=C%15, [O]C(C(F)(F)F)=O,
O=C(C(F)(F)F)O[I]C%15=C(C(F)(F)F)C=CC=C%15, 51.7
O=C(C(F)(F)F)O[I](OC(C(F)(F)F)=O)C%16=CC=C(C#N)C=C%16, [O]C(C(F)(F)F)=O,
O=C(C(F)(F)F)O[I]C%16=CC=C(C#N)C=C%16, 52.1
O=C(C(F)(F)F)O[I](OC(C(F)(F)F)=O)C%17=CC(C#N)=CC=C%17, [O]C(C(F)(F)F)=O,
O=C(C(F)(F)F)O[I]C%17=CC(C#N)=CC=C%17, 51.0
O=C(C(F)(F)F)O[I](OC(C(F)(F)F)=O)C%18=C(C#N)C=CC=C%18, [O]C(C(F)(F)F)=O,
O=C(C(F)(F)F)O[I]C%18=C(C#N)C=CC=C%18, 50.3
O=C(C(F)(F)F)O[I](OC(C(F)(F)F)=O)C%19=CC=C(C(OC)=O)C=C%19, [O]C(C(F)(F)F)=O,
O=C(C(F)(F)F)O[I]C%19=CC=C(C(OC)=O)C=C%19, 52.7
O=C(C(F)(F)F)O[I](OC(C(F)(F)F)=O)C%20=CC(C(OC)=O)=CC=C%20, [O]C(C(F)(F)F)=O,
O=C(C(F)(F)F)O[I]C%20=CC(C(OC)=O)=CC=C%20, 52.4
O=C(C(F)(F)F)O[I](OC(C(F)(F)F)=O)C%21=C(C(OC)=O)C=CC=C%21, [O]C(C(F)(F)F)=O,
O=C(C(F)(F)F)O[I]C%21=C(C(OC)=O)C=CC=C%21, 51.9
O=C(C(F)(F)F)O[I](OC(C(F)(F)F)=O)C%22=CC=C([N+]([O-])=O)C=C%22, [O]C(C(F)(F)F)=O,
O=C(C(F)(F)F)O[I]C%22=CC=C([N+]([O-])=O)C=C%22, 51.5
O=C(C(F)(F)F)O[I](OC(C(F)(F)F)=O)C%23=CC([N+]([O-])=O)=CC=C%23, [O]C(C(F)(F)F)=O,
O=C(C(F)(F)F)O[I]C%23=CC([N+]([O-])=O)=CC=C%23, 50.9
O=C(C(F)(F)F)O[I](OC(C(F)(F)F)=O)C%24=C([N+]([O-])=O)C=CC=C%24, [O]C(C(F)(F)F)=O,
O=C(C(F)(F)F)O[I]C%24=C([N+]([O-])=O)C=CC=C%24, 49.4
O=C(C(F)(F)F)O[I](OC(C(F)(F)F)=O)C%25=CC=C(F)C=C%25, [O]C(C(F)(F)F)=O,
O=C(C(F)(F)F)O[I]C%25=CC=C(F)C=C%25, 52.4
O=C(C(F)(F)F)O[I](OC(C(F)(F)F)=O)C%26=CC(F)=CC=C%26, [O]C(C(F)(F)F)=O,
O=C(C(F)(F)F)O[I]C%26=CC(F)=CC=C%26, 51.8
O=C(C(F)(F)F)O[I](OC(C(F)(F)F)=O)C%27=C(F)C=CC=C%27, [O]C(C(F)(F)F)=O,
O=C(C(F)(F)F)O[I]C%27=C(F)C=CC=C%27, 51.4
O=C(C(F)(F)F)O[I](OC(C(F)(F)F)=O)C%28=CC=C(Cl)C=C%28, [O]C(C(F)(F)F)=O,
O=C(C(F)(F)F)O[I]C%28=CC=C(Cl)C=C%28, 52.1
O=C(C(F)(F)F)O[I](OC(C(F)(F)F)=O)C%29=CC(Cl)=CC=C%29, [O]C(C(F)(F)F)=O,
O=C(C(F)(F)F)O[I]C%29=CC(Cl)=CC=C%29, 51.8
O=C(C(F)(F)F)O[I](OC(C(F)(F)F)=O)C%30=C(Cl)C=CC=C%30, [O]C(C(F)(F)F)=O,
O=C(C(F)(F)F)O[I]C%30=C(Cl)C=CC=C%30, 51.2

O=C(C(F)(F)F)O[I](OC(C(F)(F)F)=O)C%31=CC=C(Br)C=C%31, [O]C(C(F)(F)F)=O, O=C(C(F)(F)F)O[I]C%31=CC=C(Br)C=C%31, 52.0
O=C(C(F)(F)F)O[I](OC(C(F)(F)F)=O)C%32=CC(Br)=CC=C%32, [O]C(C(F)(F)F)=O, O=C(C(F)(F)F)O[I]C%32=CC(Br)=CC=C%32, 51.9
O=C(C(F)(F)F)O[I](OC(C(F)(F)F)=O)C%33=C(Br)C=CC=C%33, [O]C(C(F)(F)F)=O, O=C(C(F)(F)F)O[I]C%33=C(Br)C=CC=C%33, 51.1
F[I](OS(C1=CC=C(C=C1)C)(=O)=O)C2=CC=CC=C2, [O]S(C1=CC=C(C)C=C1)(=O)=O, F[I]C1=CC=CC=C1, 59.0
F[I](OC3=CC=CC=C3)C4=CC=CC=C4, [O]C1=CC=CC=C1, F[I]C1=CC=CC=C1, 30.5
F[I](OOC(C)(C)C)C5=CC=CC=C5, [O]OC(C)(C)C, F[I]C1=CC=CC=C1, 28.5
F[I](OC)C6=CC=CC=C6, [O]C, F[I]C1=CC=CC=C1, 45.2
F[I](O)C7=CC=CC=C7, [OH], F[I]C1=CC=CC=C1, 56.5
F[I](OC(C(C)(C)C)=O)C8=CC=CC=C8, [O]C(C(C)(C)C)=O, F[I]C1=CC=CC=C1, 56.3
F[I](OC(C9=CC=CC=C9)=O)C%10=CC=CC=C%10, [O]C(C1=CC=CC=C1)=O, F[I]C1=CC=CC=C1, 56.7
F[I](OC(C(C)C)=O)C%11=CC=CC=C%11, [O]C(C(C)C)=O, F[I]C1=CC=CC=C1, 55.5
F[I](OC(C(C%12=CC=CC=C%12)=O)=O)C%13=CC=CC=C%13, [O]C(C(C1=CC=CC=C1)=O)=O, F[I]C1=CC=CC=C1, 59.8
F[I](OC(CC%14=CC=C(C)C=C%14)=O)C%15=CC=CC=C%15, [O]C(CC1=CC=C(C)C=C1)=O, F[I]C1=CC=CC=C1, 55.6
F[I](OC(CC(F)(F)F)=O)C%16=CC=CC=C%16, [O]C(CC(F)(F)F)=O, F[I]C1=CC=CC=C1, 60.1
F[I](OC(C(F)(F)F)=O)C%17=CC=CC=C%17, [O]C(C(F)(F)F)=O, F[I]C1=CC=CC=C1, 63.5
F[I](OC(C(F)F)=O)C%18=CC=CC=C%18, [O]C(C(F)F)=O, F[I]C1=CC=CC=C1, 61.7
F[I](OC(C(Cl)(Cl)Cl)=O)C%19=CC=CC=C%19, [O]C(C(Cl)(Cl)Cl)=O, F[I]C1=CC=CC=C1, 60.6
F[I](OC(CC%20=CC=CC=C%20)=O)C%21=CC=CC=C%21, [O]C(CC1=CC=CC=C1)=O, F[I]C1=CC=CC=C1, 55.8
F[I](OC(F)(F)F)C%22=CC=CC=C%22, [O]C(F)(F)F, F[I]C1=CC=CC=C1, 63.4
F[I](OC(C)=O)C%23=CC=CC=C%23, [O]C(C)=O, F[I]C1=CC=CC=C1, 56.0
F[I](N%24C(C(C=CC=C%25)=C%25C%24=O)=O)C%26=CC=CC=C%26, O=C1C2=C(C=CC=C2)C([N]1)=O, F[I]C1=CC=CC=C1, 64.0
F[I](N=[N+]=[N-])C%27=CC=CC=C%27, [N]=[N+]=[N-], F[I]C1=CC=CC=C1, 33.0
F[I](I)C%28=CC=CC=C%28, [I], F[I]C1=CC=CC=C1, 38.5
F[I](C#N)C%29=CC=CC=C%29, [C]#N, F[I]C1=CC=CC=C1, 75.1
F[I](Cl)C%30=CC=CC=C%30, [Cl], F[I]C1=CC=CC=C1, 56.2
F[I](C(F)(F)F)C%31=CC=CC=C%31, F[C](F)F, F[I]C1=CC=CC=C1, 39.8
F[I](C#C[Si](C)(C)C)C%32=CC=CC=C%32, [C]#C[Si](C)(C)C, F[I]C1=CC=CC=C1, 74.8
F[I](Br)C%33=CC=CC=C%33, [Br], F[I]C1=CC=CC=C1, 45.5
Cl[I](OS(C1=CC=C(C=C1)C)(=O)=O)C2=CC=CC=C2, [O]S(C1=CC=C(C)C=C1)(=O)=O, Cl[I]C1=CC=CC=C1, 41.7
Cl[I](OC3=CC=CC=C3)C4=CC=CC=C4, [O]C1=CC=CC=C1, Cl[I]C1=CC=CC=C1, 14.9
Cl[I](OOC(C)(C)C)C5=CC=CC=C5, [O]OC(C)(C)C, Cl[I]C1=CC=CC=C1, 13.3
Cl[I](OC)C6=CC=CC=C6, [O]C, Cl[I]C1=CC=CC=C1, 30.0
Cl[I](O)C7=CC=CC=C7, [OH], Cl[I]C1=CC=CC=C1, 41.2
Cl[I](OC(C(C)(C)C)=O)C8=CC=CC=C8, [O]C(C(C)(C)C)=O, Cl[I]C1=CC=CC=C1, 42.0
Cl[I](OC(C9=CC=CC=C9)=O)C%10=CC=CC=C%10, [O]C(C1=CC=CC=C1)=O, Cl[I]C1=CC=CC=C1, 42.4
Cl[I](OC(C(C)C)=O)C%11=CC=CC=C%11, [O]C(C(C)C)=O, Cl[I]C1=CC=CC=C1, 41.2
Cl[I](OC(C(C%12=CC=CC=C%12)=O)=O)C%13=CC=CC=C%13, [O]C(C(C1=CC=CC=C1)=O)=O, Cl[I]C1=CC=CC=C1, 45.6
Cl[I](OC(CC%14=CC=C(C)C=C%14)=O)C%15=CC=CC=C%15, [O]C(CC1=CC=C(C)C=C1)=O, Cl[I]C1=CC=CC=C1, 41.4

Cl[I](OC(CC(F)(F)F)=O)C%16=CC=CC=C%16, [O]C(CC(F)(F)F)=O, Cl[I]C1=CC=CC=C1, 45.6
Cl[I](OC(C(F)(F)F)=O)C%17=CC=CC=C%17, [O]C(C(F)(F)F)=O, Cl[I]C1=CC=CC=C1, 49.4
Cl[I](OC(C(F)F)=O)C%18=CC=CC=C%18, [O]C(C(F)F)=O, Cl[I]C1=CC=CC=C1, 46.8
Cl[I](OC(C(Cl)(Cl)Cl)=O)C%19=CC=CC=C%19, [O]C(C(Cl)(Cl)Cl)=O, Cl[I]C1=CC=CC=C1, 46.3
Cl[I](OC(CC%20=CC=CC=C%20)=O)C%21=CC=CC=C%21, [O]C(CC1=CC=CC=C1)=O,
Cl[I]C1=CC=CC=C1, 41.6
Cl[I](OC(F)(F)F)C%22=CC=CC=C%22, [O]C(F)(F)F, Cl[I]C1=CC=CC=C1, 48.1
Cl[I](OC(C)=O)C%23=CC=CC=C%23, [O]C(C)=O, Cl[I]C1=CC=CC=C1, 41.7
Cl[I](N%24C(C(C=CC=C%25)=C%25C%24=O)=O)C%26=CC=CC=C%26,
O=C1C2=C(C=CC=C2)C([N]1)=O, Cl[I]C1=CC=CC=C1, 49.3
Cl[I](N=[N+]=[N-])C%27=CC=CC=C%27, [N]=[N+]=[N-], Cl[I]C1=CC=CC=C1, 18.8
Cl[I](I)C%28=CC=CC=C%28, [I], Cl[I]C1=CC=CC=C1, 28.9
Cl[I](F)C%30=CC=CC=C%30, [F], Cl[I]C1=CC=CC=C1, 63.6
Cl[I](C#N)C%29=CC=CC=C%29, [C]#N, Cl[I]C1=CC=CC=C1, 59.6
Cl[I](C(F)(F)F)C%31=CC=CC=C%31, F[C](F)F, Cl[I]C1=CC=CC=C1, 26.8
Cl[I](C#C[Si](C)(C)C)C%32=CC=CC=C%32, [C]#C[Si](C)(C)C, Cl[I]C1=CC=CC=C1, 60.2
Cl[I](Br)C%33=CC=CC=C%33, [Br], Cl[I]C1=CC=CC=C1, 32.9
F[I](OS(C1=CC=C(C=C1)C)(=O)=O)C2=CC=CC=C2, [F],
[I](OS(C1=CC=C(C=C1)C)(=O)=O)C2=CC=CC=C2, 71.4
F[I](OC3=CC=CC=C3)C4=CC=CC=C4, [F], [I](OC3=CC=CC=C3)C4=CC=CC=C4, 44.5
F[I](OOC(C)(C)C)C5=CC=CC=C5, [F], [I](OOC(C)(C)C)C5=CC=CC=C5, 42.9
F[I](OC)C6=CC=CC=C6, [F], [I](OC)C6=CC=CC=C6, 61.5
F[I](O)C7=CC=CC=C7, [F], [I](O)C7=CC=CC=C7, 71.3
F[I](OC(C(C)(C)C)=O)C8=CC=CC=C8, [F], [I](OC(C(C)(C)C)=O)C8=CC=CC=C8, 69.2
F[I](OC(C9=CC=CC=C9)=O)C%10=CC=CC=C%10, [F],
[I](OC(C9=CC=CC=C9)=O)C%10=CC=CC=C%10, 70.7
F[I](OC(C(C)C)=O)C%11=CC=CC=C%11, [F], [I](OC(C(C)C)=O)C%11=CC=CC=C%11, 69.2
F[I](OC(C(C%12=CC=CC=C%12)=O)=O)C%13=CC=CC=C%13, [F],
[I](OC(C(C%12=CC=CC=C%12)=O)=O)C%13=CC=CC=C%13, 37.8
F[I](OC(CC%14=CC=C(C)C=C%14)=O)C%15=CC=CC=C%15, [F],
[I](OC(CC%14=CC=C(C)C=C%14)=O)C%15=CC=CC=C%15, 68.2
F[I](OC(CC(F)(F)F)=O)C%16=CC=CC=C%16, [F], [I](OC(CC(F)(F)F)=O)C%16=CC=CC=C%16, 70.3
F[I](OC(C(F)(F)F)=O)C%17=CC=CC=C%17, [F], [I](OC(C(F)(F)F)=O)C%17=CC=CC=C%17, 68.9
F[I](OC(C(F)F)=O)C%18=CC=CC=C%18, [F], [I](OC(C(F)F)=O)C%18=CC=CC=C%18, 68.6
F[I](OC(C(Cl)(Cl)Cl)=O)C%19=CC=CC=C%19, [F], [I](OC(C(Cl)(Cl)Cl)=O)C%19=CC=CC=C%19,
69.7
F[I](OC(CC%20=CC=CC=C%20)=O)C%21=CC=CC=C%21, [F],
[I](OC(CC%20=CC=CC=C%20)=O)C%21=CC=CC=C%21, 68.4
F[I](OC(F)(F)F)C%22=CC=CC=C%22, [F], [I](OC(F)(F)F)C%22=CC=CC=C%22, 72.0
F[I](OC(C)=O)C%23=CC=CC=C%23, [F], [I](OC(C)=O)C%23=CC=CC=C%23, 69.7
F[I](N%24C(C(C=CC=C%25)=C%25C%24=O)=O)C%26=CC=CC=C%26, [F],
[I](N%24C(C(C=CC=C%25)=C%25C%24=O)=O)C%26=CC=CC=C%26, 75.9
F[I](N=[N+]=[N-])C%27=CC=CC=C%27, [F], [I](N=[N+]=[N-])C%27=CC=CC=C%27, 48.8
F[I](I)C%28=CC=CC=C%28, [F], [I](I)C%28=CC=CC=C%28, 48.7
F[I](C#N)C%29=CC=CC=C%29, [F], [I](C#N)C%29=CC=CC=C%29, 80.1
F[I](C(F)(F)F)C%31=CC=CC=C%31, [F], [I](C(F)(F)F)C%31=CC=CC=C%31, 56.2
F[I](C#C[Si](C)(C)C)C%32=CC=CC=C%32, [F], [I](C#C[Si](C)(C)C)C%32=CC=CC=C%32, 86.8
F[I](Br)C%33=CC=CC=C%33, [F], [I](Br)C%33=CC=CC=C%33, 55.8
[I](OS(C1=CC=C(C=C1)C)(=O)=O)C2=CC=CC=C2, [Cl],
[I](OS(C1=CC=C(C=C1)C)(=O)=O)C2=CC=CC=C2, 46.9
[I](OC3=CC=CC=C3)C4=CC=CC=C4, [Cl], [I](OC3=CC=CC=C3)C4=CC=CC=C4, 21.5

[I](OOC(C)(C)C)C5=CC=CC=C5, [Cl], [I](OOC(C)(C)C)C5=CC=CC=C5, 20.4
[I](OC)C6=CC=CC=C6, [Cl], [I](OC)C6=CC=CC=C6, 39.0
[I](O)C7=CC=CC=C7, [Cl], [I](O)C7=CC=CC=C7, 48.6
[I](OC(C(C)(C)C)=O)C8=CC=CC=C8, [Cl], [I](OC(C(C)(C)C)=O)C8=CC=CC=C8, 47.6
[I](OC(C9=CC=CC=C9)=O)C%10=CC=CC=C%10, [Cl],
[I](OC(C9=CC=CC=C9)=O)C%10=CC=CC=C%10, 49.1
[I](OC(C(C)C)=O)C%11=CC=CC=C%11, [Cl], [I](OC(C(C)C)=O)C%11=CC=CC=C%11, 47.5
[I](OC(C(C%12=CC=CC=C%12)=O)=O)C%13=CC=CC=C%13, [Cl],
[I](OC(C(C%12=CC=CC=C%12)=O)=O)C%13=CC=CC=C%13, 16.2
[I](OC(CC%14=CC=C(C)C=C%14)=O)C%15=CC=CC=C%15, [Cl],
[I](OC(CC%14=CC=C(C)C=C%14)=O)C%15=CC=CC=C%15, 46.6
[I](OC(CC(F)(F)F)=O)C%16=CC=CC=C%16, [Cl], [I](OC(CC(F)(F)F)=O)C%16=CC=CC=C%16, 48.5
[I](OC(C(F)(F)F)=O)C%17=CC=CC=C%17, [Cl], [I](OC(C(F)(F)F)=O)C%17=CC=CC=C%17, 47.4
[I](OC(C(F)F)=O)C%18=CC=CC=C%18, [Cl], [I](OC(C(F)F)=O)C%18=CC=CC=C%18, 46.3
[I](OC(C(Cl)(Cl)Cl)=O)C%19=CC=CC=C%19, [Cl], [I](OC(C(Cl)(Cl)Cl)=O)C%19=CC=CC=C%19, 48.1
[I](OC(CC%20=CC=CC=C%20)=O)C%21=CC=CC=C%21, [Cl],
[I](OC(CC%20=CC=CC=C%20)=O)C%21=CC=CC=C%21, 46.8
[I](OC(F)(F)F)C%22=CC=CC=C%22, [Cl], [I](OC(F)(F)F)C%22=CC=CC=C%22, 49.4
[I](OC(C)=O)C%23=CC=CC=C%23, [Cl], [I](OC(C)=O)C%23=CC=CC=C%23, 48.1
[I](N%24C(C(C=CC=C%25)=C%25C%24=O)=O)C%26=CC=CC=C%26, [Cl],
[I](N%24C(C(C=CC=C%25)=C%25C%24=O)=O)C%26=CC=CC=C%26, 53.8
[I](N=[N+]=[N-])C%27=CC=CC=C%27, [Cl], [I](N=[N+]=[N-])C%27=CC=CC=C%27, 27.3
[I](I)C%28=CC=CC=C%28, [Cl], [I](I)C%28=CC=CC=C%28, 31.8
[I](C#N)C%29=CC=CC=C%29, [Cl], [I](C#N)C%29=CC=CC=C%29, 57.2
[I](C(F)(F)F)C%31=CC=CC=C%31, [Cl], [I](C(F)(F)F)C%31=CC=CC=C%31, 35.9
[I](C#C[Si](C)(C)C)C%32=CC=CC=C%32, [Cl], [I](C#C[Si](C)(C)C)C%32=CC=CC=C%32, 64.8
[I](Br)C%33=CC=CC=C%33, [Cl], [I](Br)C%33=CC=CC=C%33, 35.9

2. Fig4_AD_A_110HVIs

O=C1O[I](C=C)C2=NC3=CC=CC=C3C=C21, C=[C], O=C1O[I]C2=NC3=CC=CC=C3C=C21, 47.9
O=C(O[I]4C=C)C5=C4C=CN5C, C=[C], O=C(O[I]4)C5=C4C=CN5C, 42.8
O=C6O[I](C=C)C7=CC=CN=C76, C=[C], O=C6O[I]C7=CC=CN=C76, 41.8
O=C8O[I](C=C)C9=CC%10=CC=CC=C%10C=C98, C=[C],
O=C8O[I]C9=CC%10=CC=CC=C%10C=C98, 44.5
O=C(O[I]%11C=C)C%12=C%11C%13=CC=CC=C%13N%12C, C=[C],
O=C(O[I]1)C2=C1C3=CC=CC=C3N2C, 43.2
O=C(O%14)N(C)C%15=C([I]%14C=C)C=CC=C%15, C=[C],
O=C%14N(C)C%15=C([I]O%14)C=CC=C%15, 45.5
CC(S[I]%16C=C)(C)C%17=C%16C=CC=C%17, C=[C], CC(S[I]%16)(C)C%17=C%16C=CC=C%17, 22.5
C=C[I]%18OC(C(F)(F)F)(C(F)(F)F)C%19=C%18C=CC=C%19, C=[C],
FC(C(O[I]%18)(C(F)(F)F)C%19=C%18C=CC=C%19)(F)F, 45.3
O=C(N(C(C)=O)[I]%20C=C)C%21=C%20C=CC=C%21, C=[C],
O=C(N(C(C)=O)[I]%20)C%21=C%20C=CC=C%21, 39.8
O=C(O[I]%22C=C)C%23=C%22C=CO%23, C=[C], O=C(O[I]%22)C%23=C%22C=CO%23, 40.1
O=C1O[I](C2=CC=CC=C2)C3=NC4=CC=CC=C4C=C31, C1=CC=CC=[C]1,
O=C1O[I]C2=NC3=CC=CC=C3C=C21, 50.2
O=C(O[I]5C6=CC=CC=C6)C7=C5C=CN7C, C1=CC=CC=[C]1, O=C(O[I]4)C5=C4C=CN5C, 45.9
O=C8O[I](C9=CC=CC=C9)C%10=CC=CN=C%108, C1=CC=CC=[C]1, O=C6O[I]C7=CC=CN=C76,

45.3
O=C%11O[I](C%12=CC=CC=C%12)C%13=CC%14=CC=CC=C%14C=C%13%11, C1=CC=CC=[C]1, O=C8O[I]C9=CC%10=CC=CC=C%10C=C98, 47.7
O=C(O[I]%15C%16=CC=CC=C%16)C%17=C%15C%18=CC=CC=C%18N%17C, C1=CC=CC=[C]1, O=C(O[I]1)C2=C1C3=CC=CC=C3N2C, 47.0
O=C(O%19)N(C)C%20=C([I]%19C%21=CC=CC=C%21)C=CC=C%20, C1=CC=CC=[C]1, O=C%14N(C)C%15=C([I]O%14)C=CC=C%15, 47.8
CC(S[I]%22C%23=CC=CC=C%23)(C)C%24=C%22C=CC=C%24, C1=CC=CC=[C]1, CC(S[I]%16)(C)C%17=C%16C=CC=C%17, 24.6
FC(C(O[I]%25C%26=CC=CC=C%26)(C(F)(F)F)C%27=C%25C=CC=C%27)(F)F, C1=CC=CC=[C]1, FC(C(O[I]%18)(C(F)(F)F)C%19=C%18C=CC=C%19)(F)F, 48.3
O=C(N(C(C)=O)[I]%28C%29=CC=CC=C%29)C%30=C%28C=CC=C%30, C1=CC=CC=[C]1, O=C(N(C(C)=O)[I]%20)C%21=C%20C=CC=C%21, 42.9
O=C(O[I]%31C%32=CC=CC=C%32)C%33=C%31C=CO%33, C1=CC=CC=[C]1, O=C(O[I]%22)C%23=C%22C=CO%23, 43.4
O=C1O[I](OS(=O)(C(F)(F)F)=O)C2=NC3=CC=CC=C3C=C21, [O]S(=O)(C(F)(F)F)=O, O=C1O[I]C2=NC3=CC=CC=C3C=C21, 50.9
O=C(O[I]4OS(=O)(C(F)(F)F)=O)C5=C4C=CN5C, [O]S(=O)(C(F)(F)F)=O, O=C(O[I]4)C5=C4C=CN5C, 49.4
O=C6O[I](OS(=O)(C(F)(F)F)=O)C7=CC=CN=C76, [O]S(=O)(C(F)(F)F)=O, O=C6O[I]C7=CC=CN=C76, 51.0
O=C8O[I](OS(=O)(C(F)(F)F)=O)C9=CC%10=CC=CC=C%10C=C98, [O]S(=O)(C(F)(F)F)=O, O=C8O[I]C9=CC%10=CC=CC=C%10C=C98, 53.0
O=C(O[I]%11OS(=O)(C(F)(F)F)=O)C%12=C%11C%13=CC=CC=C%13N%12C, [O]S(=O)(C(F)(F)F)=O, O=C(O[I]1)C2=C1C3=CC=CC=C3N2C, 49.6
O=C(O%14)N(C)C%15=C([I]%14OS(=O)(C(F)(F)F)=O)C=CC=C%15, [O]S(=O)(C(F)(F)F)=O, O=C%14N(C)C%15=C([I]O%14)C=CC=C%15, 53.5
CC(S[I]%16OS(=O)(C(F)(F)F)=O)(C)C%17=C%16C=CC=C%17, [O]S(=O)(C(F)(F)F)=O, CC(S[I]%16)(C)C%17=C%16C=CC=C%17, 52.0
FC(C(O[I]%18OS(=O)(C(F)(F)F)=O)(C(F)(F)F)C%19=C%18C=CC=C%19)(F)F, [O]S(=O)(C(F)(F)F)=O, FC(C(O[I]%18)(C(F)(F)F)C%19=C%18C=CC=C%19)(F)F, 55.8
O=C(N(C(C)=O)[I]%20OS(=O)(C(F)(F)F)=O)C%21=C%20C=CC=C%21, [O]S(=O)(C(F)(F)F)=O, O=C(N(C(C)=O)[I]%20)C%21=C%20C=CC=C%21, 48.2
O=C(O[I]%22OS(=O)(C(F)(F)F)=O)C%23=C%22C=CO%23, [O]S(=O)(C(F)(F)F)=O, O=C(O[I]%22)C%23=C%22C=CO%23, 45.9
O=C1O[I](OC)C2=NC3=CC=CC=C3C=C21, [O]C, O=C1O[I]C2=NC3=CC=CC=C3C=C21, 34.5
O=C(O[I]4OC)C5=C4C=CN5C, [O]C, O=C(O[I]4)C5=C4C=CN5C, 31.4
O=C6O[I](OC)C7=CC=CN=C76, [O]C, O=C6O[I]C7=CC=CN=C76, 33.9
O=C8O[I](OC)C9=CC%10=CC=CC=C%10C=C98, [O]C, O=C8O[I]C9=CC%10=CC=CC=C%10C=C98, 35.7
O=C(O[I]%11OC)C%12=C%11C%13=CC=CC=C%13N%12C, [O]C, O=C(O[I]1)C2=C1C3=CC=CC=C3N2C, 33.1
O=C(O%14)N(C)C%15=C([I]%14OC)C=CC=C%15, [O]C, O=C%14N(C)C%15=C([I]O%14)C=CC=C%15, 33.6
CC(S[I]%16OC)(C)C%17=C%16C=CC=C%17, [O]C, CC(S[I]%16)(C)C%17=C%16C=CC=C%17, 20.2
FC(C(O[I]%18OC)(C(F)(F)F)C%19=C%18C=CC=C%19)(F)F, [O]C, FC(C(O[I]%18)(C(F)(F)F)C%19=C%18C=CC=C%19)(F)F, 38.3
O=C(N(C(C)=O)[I]%20OC)C%21=C%20C=CC=C%21, [O]C, O=C(N(C(C)=O)[I]%20)C%21=C%20C=CC=C%21, 34.3
O=C(O[I]%22OC)C%23=C%22C=CO%23, [O]C, O=C(O[I]%22)C%23=C%22C=CO%23, 28.9
O=C1O[I](OC(F)(F)F)C2=NC3=CC=CC=C3C=C21, [O]C(F)(F)F,

O=C1O[I]C2=NC3=CC=CC=C3C=C21, 51.4
O=C(O[I]4OC(F)(F)F)C5=C4C=CN5C, [O]C(F)(F)F, O=C(O[I]4)C5=C4C=CN5C, 49.3
O=C6O[I](OC(F)(F)F)C7=CC=CN=C76, [O]C(F)(F)F, O=C6O[I]C7=CC=CN=C76, 51.9
O=C8O[I](OC(F)(F)F)C9=CC%10=CC=CC=C%10C=C98, [O]C(F)(F)F,
O=C8O[I]C9=CC%10=CC=CC=C%10C=C98, 53.7
O=C(O[I]%11OC(F)(F)F)C%12=C%11C%13=CC=CC=C%13N%12C, [O]C(F)(F)F,
O=C(O[I]1)C2=C1C3=CC=CC=C3N2C, 50.2
O=C(O%14)N(C)C%15=C([I]%14OC(F)(F)F)C=CC=C%15, [O]C(F)(F)F,
O=C%14N(C)C%15=C([I]O%14)C=CC=C%15, 53.3
CC(S[I]%16OC(F)(F)F)(C)C%17=C%16C=CC=C%17, [O]C(F)(F)F,
CC(S[I]%16)(C)C%17=C%16C=CC=C%17, 46.7
FC(C(O[I]%18OC(F)(F)F)(C(F)(F)F)C%19=C%18C=CC=C%19)(F)F, [O]C(F)(F)F,
FC(C(O[I]%18)(C(F)(F)F)C%19=C%18C=CC=C%19)(F)F, 56.0
O=C(N(C(C)=O)[I]%20OC(F)(F)F)C%21=C%20C=CC=C%21, [O]C(F)(F)F,
O=C(N(C(C)=O)[I]%20)C%21=C%20C=CC=C%21, 54.7
O=C(O[I]%22OC(F)(F)F)C%23=C%22C=CO%23, [O]C(F)(F)F,
O=C(O[I]%22)C%23=C%22C=CO%23, 46.3
O=C1O[I](N(S(=O)(C(F)(F)F)=O)S(=O)(C(F)(F)F)=O)C2=NC3=CC=CC=C3C=C21,
O=S([N]S(=O)(C(F)(F)F)=O)(C(F)(F)F)=O, O=C1O[I]C2=NC3=CC=CC=C3C=C21, 53.7
O=C(O[I]4N(S(=O)(C(F)(F)F)=O)S(=O)(C(F)(F)F)=O)C5=C4C=CN5C,
O=S([N]S(=O)(C(F)(F)F)=O)(C(F)(F)F)=O, O=C(O[I]4)C5=C4C=CN5C, 48.3
O=C6O[I](N(S(=O)(C(F)(F)F)=O)S(=O)(C(F)(F)F)=O)C7=CC=CN=C76,
O=S([N]S(=O)(C(F)(F)F)=O)(C(F)(F)F)=O, O=C6O[I]C7=CC=CN=C76, 50.0
O=C8O[I](N(S(=O)(C(F)(F)F)=O)S(=O)(C(F)(F)F)=O)C9=CC%10=CC=CC=C%10C=C98,
O=S([N]S(=O)(C(F)(F)F)=O)(C(F)(F)F)=O, O=C8O[I]C9=CC%10=CC=CC=C%10C=C98, 52.2
O=C(O[I]%11N(S(=O)(C(F)(F)F)=O)S(=O)(C(F)(F)F)=O)C%12=C%11C%13=CC=CC=C%13N%12C,
O=S([N]S(=O)(C(F)(F)F)=O)(C(F)(F)F)=O, O=C(O[I]1)C2=C1C3=CC=CC=C3N2C, 47.4
O=C(O%14)N(C)C%15=C([I]%14N(S(=O)(C(F)(F)F)=O)S(=O)(C(F)(F)F)=O)C=CC=C%15,
O=S([N]S(=O)(C(F)(F)F)=O)(C(F)(F)F)=O, O=C%14N(C)C%15=C([I]O%14)C=CC=C%15, 52.5
CC(S[I]%16N(S(=O)(C(F)(F)F)=O)S(=O)(C(F)(F)F)=O)(C)C%17=C%16C=CC=C%17,
O=S([N]S(=O)(C(F)(F)F)=O)(C(F)(F)F)=O, CC(S[I]%16)(C)C%17=C%16C=CC=C%17, 53.1
FC(C(O[I]%18N(S(=O)(C(F)(F)F)=O)S(=O)(C(F)(F)F)=O)(C(F)(F)F)C%19=C%18C=CC=C%19)(F)F,
O=S([N]S(=O)(C(F)(F)F)=O)(C(F)(F)F)=O, FC(C(O[I]%18)(C(F)(F)F)C%19=C%18C=CC=C%19)(F)F,
54.1
O=C(N(C(C)=O)[I]%20N(S(=O)(C(F)(F)F)=O)S(=O)(C(F)(F)F)=O)C%21=C%20C=CC=C%21,
O=S([N]S(=O)(C(F)(F)F)=O)(C(F)(F)F)=O, O=C(N(C(C)=O)[I]%20)C%21=C%20C=CC=C%21, 54.5
O=C(O[I]%22N(S(=O)(C(F)(F)F)=O)S(=O)(C(F)(F)F)=O)C%23=C%22C=CO%23,
O=S([N]S(=O)(C(F)(F)F)=O)(C(F)(F)F)=O, O=C(O[I]%22)C%23=C%22C=CO%23, 44.7
O=C1O[I](C)C2=NC3=CC=CC=C3C=C21, [H][C]([H])[H], O=C1O[I]C2=NC3=CC=CC=C3C=C21,
41.5
O=C(O[I]4C)C5=C4C=CN5C, [H][C]([H])[H], O=C(O[I]4)C5=C4C=CN5C, 36.0
O=C6O[I](C)C7=CC=CN=C76, [H][C]([H])[H], O=C6O[I]C7=CC=CN=C76, 34.0
O=C8O[I](C)C9=CC%10=CC=CC=C%10C=C98, [H][C]([H])[H],
O=C8O[I]C9=CC%10=CC=CC=C%10C=C98, 36.8
O=C(O[I]1)C2=C1C3=CC=CC=C3N2CC, [H][C]([H])[H], O=C(O[I]1)C2=C1C3=CC=CC=C3N2C,
35.8
O=C(O%14)N(C)C%15=C([I]%14C)C=CC=C%15, [H][C]([H])[H],
O=C%14N(C)C%15=C([I]O%14)C=CC=C%15, 37.4
CC(S[I]%16C)(C)C%17=C%16C=CC=C%17, [H][C]([H])[H],
CC(S[I]%16)(C)C%17=C%16C=CC=C%17, 14.1
FC(C(O[I]%18C)(C(F)(F)F)C%19=C%18C=CC=C%19)(F)F, [H][C]([H])[H],

FC(C(O[I]%18)(C(F)(F)F)C%19=C%18C=CC=C%19)(F)F, 37.1
O=C(N(C(C)=O)[I]%20C)C%21=C%20C=CC=C%21, [H][C]([H])[H],
O=C(N(C(C)=O)[I]%20)C%21=C%20C=CC=C%21, 32.1
O=C(O[I]%22C)C%23=C%22C=CO%23, [H][C]([H])[H], O=C(O[I]%22)C%23=C%22C=CO%23, 33.0
O=C1O[I](F)C2=NC3=CC=CC=C3C=C21, [F], O=C1O[I]C2=NC3=CC=CC=C3C=C21, 66.2
CN4C=CC5=C4C(O[I]5F)=O, [F], O=C(O[I]4)C5=C4C=CN5C, 64.7
F[I](O6)C7=CC=CN=C7C6=O, [F], O=C6O[I]C7=CC=CN=C76, 68.2
O=C8O[I](F)C9=CC%10=CC=CC=C%10C=C98, [F], O=C8O[I]C9=CC%10=CC=CC=C%10C=C98, 69.9
CN%11C%12=CC=CC=C%12C%13=C%11C(O[I]%13F)=O, [F],
O=C(O[I]1)C2=C1C3=CC=CC=C3N2C, 66.3
F[I]%14C%15=C(N(C)C(O%14)=O)C=CC=C%15, [F], O=C%14N(C)C%15=C([I]O%14)C=CC=C%15, 69.5
F[I]%16SC(C)(C)C%17=C%16C=CC=C%17, [F], CC(S[I]%16)(C)C%17=C%16C=CC=C%17, 60.1
F[I]%18OC(C(F)(F)F)(C(F)(F)F)C%19=C%18C=CC=C%19, [F],
FC(C(O[I]%18)(C(F)(F)F)C%19=C%18C=CC=C%19)(F)F, 72.5
O=C(N(C(C)=O)[I]%20F)C%21=C%20C=CC=C%21, [F],
O=C(N(C(C)=O)[I]%20)C%21=C%20C=CC=C%21, 70.7
F[I]%22OC(C%23=C%22C=CO%23)=O, [F], O=C(O[I]%22)C%23=C%22C=CO%23, 62.0
O=C1O[I](C(F)(C(F)(F)F)F)C2=NC3=CC=CC=C3C=C21, F[C](F)C(F)(F)F,
O=C1O[I]C2=NC3=CC=CC=C3C=C21, 31.7
CN4C=CC5=C4C(O[I]5C(F)(C(F)(F)F)F)=O, F[C](F)C(F)(F)F, O=C(O[I]4)C5=C4C=CN5C, 28.3
O=C6O[I](C(F)(C(F)(F)F)F)C7=CC=CN=C76, F[C](F)C(F)(F)F, O=C6O[I]C7=CC=CN=C76, 26.3
O=C8O[I](C(F)(C(F)(F)F)F)C9=CC%10=CC=CC=C%10C=C98, F[C](F)C(F)(F)F,
O=C8O[I]C9=CC%10=CC=CC=C%10C=C98, 28.8
FN%11C%12=CC=CC=C%12C%13=C%11C(O[I]%13C(F)(C(F)(F)F)F)=O, F[C](F)C(F)(F)F,
O=C(O[I]1)C2=C1C3=CC=CC=C3N2C, 26.8
O=C(O%14)N(C)C%15=C([I]%14C(F)(C(F)(F)F)F)C=CC=C%15, F[C](F)C(F)(F)F,
O=C%14N(C)C%15=C([I]O%14)C=CC=C%15, 30.7
CC(S[I]%16C(F)(C(F)(F)F)F)(C)C%17=C%16C=CC=C%17, F[C](F)C(F)(F)F,
CC(S[I]%16)(C)C%17=C%16C=CC=C%17, 12.6
FC(C(O[I]%18C(F)(C(F)(F)F)F)(C(F)(F)F)C%19=C%18C=CC=C%19)(F)F, F[C](F)C(F)(F)F,
FC(C(O[I]%18)(C(F)(F)F)C%19=C%18C=CC=C%19)(F)F, 30.2
O=C(N(C(C)=O)[I]%20C(F)(C(F)(F)F)F)C%21=C%20C=CC=C%21, F[C](F)C(F)(F)F,
O=C(N(C(C)=O)[I]%20)C%21=C%20C=CC=C%21, 25.3
O=C(O[I]%22C(F)(C(F)(F)F)F)C%23=C%22C=CO%23, F[C](F)C(F)(F)F,
O=C(O[I]%22)C%23=C%22C=CO%23, 25.2
Cl[I](C=C)C1=CC=CC=C1, [C]=C, Cl[I]C1=CC=CC=C1, 38.8
Cl[I](SC2=CC=CC=C2)C3=CC=CC=C3, [S]C1=CC=CC=C1, Cl[I]C1=CC=CC=C1, 18.9
Cl[I](SC)C4=CC=CC=C4, [S]C, Cl[I]C1=CC=CC=C1, 26.8
Cl[I]([Se]C)C5=CC=CC=C5, [Se]C, Cl[I]C1=CC=CC=C1, 24.3
Cl[I](N6C=CC=C6)C7=CC=CC=C7, [N]2C=CC=C2, Cl[I]C1=CC=CC=C1, 21.7
Cl[I](OS(=O)(C(F)(F)F)=O)C8=CC=CC=C8, [O]S(=O)(C(F)(F)F)=O, Cl[I]C1=CC=CC=C1, 49.7
Cl[I](OS(C)(=O)=O)C9=CC=CC=C9, [O]S(C)(=O)=O, Cl[I]C1=CC=CC=C1, 42.2
Cl[I](N(S(=O)(C(F)(F)F)=O)S(=O)(C(F)(F)F)=O)C%10=CC=CC=C%10,
O=S([N]S(=O)(C(F)(F)F)=O)(C(F)(F)F)=O, Cl[I]C1=CC=CC=C1, 49.5
Cl[I](N(C)C)C%11=CC=CC=C%11, C[N]C, Cl[I]C1=CC=CC=C1, 20.5
Cl[I](C(F)F)C%12=CC=CC=C%12, F[CH]F, Cl[I]C1=CC=CC=C1, 24.9
F[I](C=C)C1=CC=CC=C1, [C]=C, F[I]C1=CC=CC=C1, 50.9
F[I](SC2=CC=CC=C2)C3=CC=CC=C3, [S]C1=CC=CC=C1, F[I]C1=CC=CC=C1, 33.5
F[I](SC)C4=CC=CC=C4, [S]C, F[I]C1=CC=CC=C1, 39.5

F[I]([Se]C)C5=CC=CC=C5, [Se]C, F[I]C1=CC=CC=C1, 34.6
F[I](N6C=CC=C6)C7=CC=CC=C7, [N]2C=CC=C2, F[I]C1=CC=CC=C1, 37.8
F[I](OS(=O)(C(F)(F)F)=O)C8=CC=CC=C8, [O]S(=O)(C(F)(F)F)=O, F[I]C1=CC=CC=C1, 62.3
F[I](OS(C)(=O)=O)C9=CC=CC=C9, [O]S(C)(=O)=O, F[I]C1=CC=CC=C1, 58.4
F[I](N(S(=O)(C(F)(F)F)=O)S(=O)(C(F)(F)F)=O)C%10=CC=CC=C%10,
O=S([N]S(=O)(C(F)(F)F)=O)(C(F)(F)F)=O, F[I]C1=CC=CC=C1, 62.2
F[I](N(C)C)C%11=CC=CC=C%11, C[N]C, F[I]C1=CC=CC=C1, 34.0
F[I](C(F)F)C%12=CC=CC=C%12, F[CH]F, F[I]C1=CC=CC=C1, 37.0

(3) Fig4_AD_B_180HVIs

CC(O[I]1C=C)(C)C2=C1C=CN2C, [C]=C, CC(O[I]1)(C)C2=C1C=CN2C, 32.2
CC3(C)O[I](C=C)C4=CC=CN=C43, C=[C], CC3(C)O[I]C4=CC=CN=C43, 31.3
FC5=C(F)C(F)=C(F)C(N(C)C(O6)=O)=C5[I]6C=C, C=[C], FC5=C(F)C(F)=C(F)C(N6C)=C5[I]OC6=O, 38.5
FC7=C(F)C(F)=C(F)C8=C7[I](C=C)OC8(C)C, C=[C], FC7=C(F)C(F)=C(F)C8=C7[I]OC8(C)C, 31.1
CN([I]9C=C)S(C%10=C(C9=CC=C%11)C%11=CC=C%10)(=O)=O, C=[C], CN([I]9)S(C%10=C(C9=CC=C%11)C%11=CC=C%10)(=O)=O, 29.1
CC%12(C)O[I](C=C)C%13=CC%14=CC=CC=C%14C=C%13%12, C=[C], CC%12(C)O[I]C%13=CC%14=CC=CC=C%14C=C%13%12, 33.8
O=C(N%15C)N(C)C%16=C([I]%15C=C)C=C(C=CC=C%17)C%17=C%16, C=[C], O=C%15N(C)C%16=C([I]N%15C)C=C(C=CC=C%17)C%17=C%16, 24.7
O=C(N(C(C)=O)[I]%18C=C)C%19=C%18C%20=CC=CC=C%20N%19C, C=[C], O=C(N(C(C)=O)[I]%18)C%19=C%18C%20=CC=CC=C%20N%19C, 36.7
CC(S[I]%21C=C)(C)C%22=C%21C=CO%22, C=[C], CC(S[I]%21)(C)C%22=C%21C=CO%22, 18.6
O=C%23CCCC%24=CC%25=CC%26=CC=CC=C%26C=C%25C=C%24[I](C=C)O%23, C=[C], O=C%23CCCC%24=CC%25=CC%26=CC=CC=C%26C=C%25C=C%24[I]O%23, 27.3
CC(O[I]1C2=CC=CC=C2)(C)C3=C1C=CN3C, C1=CC=C[C]=C1, CC(O[I]1)(C)C2=C1C=CN2C, 35.0
CC4(C)O[I](C5=CC=CC=C5)C6=CC=CN=C64, C1=CC=C[C]=C1, CC3(C)O[I]C4=CC=CN=C43, 34.0
FC7=C(F)C(F)=C(F)C(N(C)C(O8)=O)=C7[I]8C9=CC=CC=C9, C1=CC=C[C]=C1, FC5=C(F)C(F)=C(F)C(N6C)=C5[I]OC6=O, 41.2
FC%10=C(F)C(F)=C(F)C%11=C%10[I](C%12=CC=CC=C%12)OC%11(C)C, C1=CC=C[C]=C1, FC7=C(F)C(F)=C(F)C8=C7[I]OC8(C)C, 33.3
CN([I]%13C%14=CC=CC=C%14)S(C%15=C(C%13=CC=C%16)C%16=CC=C%15)(=O)=O, C1=CC=C[C]=C1, CN([I]9)S(C%10=C(C9=CC=C%11)C%11=CC=C%10)(=O)=O, 39.4
CC%17(C)O[I](C%18=CC=CC=C%18)C%19=CC%20=CC=CC=C%20C=C%19%17, C1=CC=C[C]=C1, CC%12(C)O[I]C%13=CC%14=CC=CC=C%14C=C%13%12, 36.4
O=C(N%21C)N(C)C%22=C([I]%21C%23=CC=CC=C%23)C=C(C=CC=C%24)C%24=C%22, C1=CC=C[C]=C1, O=C%15N(C)C%16=C([I]N%15C)C=C(C=CC=C%17)C%17=C%16, 26.7
O=C(N(C(C)=O)[I]%25C%26=CC=CC=C%26)C%27=C%25C%28=CC=CC=C%28N%27C, C1=CC=C[C]=C1, O=C(N(C(C)=O)[I]%18)C%19=C%18C%20=CC=CC=C%20N%19C, 40.3
CC(S[I]%29C%30=CC=CC=C%30)(C)C%31=C%29C=CO%31, C1=CC=C[C]=C1, CC(S[I]%21)(C)C%22=C%21C=CO%22, 21.4
O=C%32CCCC%33=CC%34=CC%35=CC=CC=C%35C=C%34C=C%33[I](C%36=CC=CC=C%36)O%32, C1=CC=C[C]=C1, O=C%23CCCC%24=CC%25=CC%26=CC=CC=C%26C=C%25C=C%24[I]O%23, 29.6
CC(O[I]1OS(=O)(C(F)(F)F)=O)(C)C2=C1C=CN2C, [O]S(=O)(C(F)(F)F)=O, CC(O[I]1)(C)C2=C1C=CN2C, 52.0
CC3(C)O[I](OS(=O)(C(F)(F)F)=O)C4=CC=CN=C43, [O]S(=O)(C(F)(F)F)=O, CC3(C)O[I]C4=CC=CN=C43, 53.3
FC5=C(F)C(F)=C(F)C(N(C)C(O6)=O)=C5[I]6OS(=O)(C(F)(F)F)=O, [O]S(=O)(C(F)(F)F)=O,

FC5=C(F)C(F)=C(F)C(N6C)=C5[I]OC6=O, 43.4
FC7=C(F)C(F)=C(F)C8=C7[I](OS(=O)(C(F)(F)F)=O)OC8(C)C, [O]S(=O)(C(F)(F)F)=O, FC7=C(F)C(F)=C(F)C8=C7[I]OC8(C)C, 47.7
CN([I]9OS(=O)(C(F)(F)F)=O)S(C%10=C(C9=CC=C%11)C%11=CC=C%10)(=O)=O, [O]S(=O)(C(F)(F)F)=O, CN([I]9)S(C%10=C(C9=CC=C%11)C%11=CC=C%10)(=O)=O, 50.3
CC%12(C)O[I](OS(=O)(C(F)(F)F)=O)C%13=CC%14=CC=CC=C%14C=C%13%12, [O]S(=O)(C(F)(F)F)=O, CC%12(C)O[I]C%13=CC%14=CC=CC=C%14C=C%13%12, 55.8
O=C(N%15C)N(C)C%16=C([I]%15OS(=O)(C(F)(F)F)=O)C=C(C=CC=C%17)C%17=C%16, [O]S(=O)(C(F)(F)F)=O, O=C%15N(C)C%16=C([I]N%15C)C=C(C=CC=C%17)C%17=C%16, 49.1
O=C(N(C(C)=O)[I]%18OS(=O)(C(F)(F)F)=O)C%19=C%18C%20=CC=CC=C%20N%19C, [O]S(=O)(C(F)(F)F)=O, O=C(N(C(C)=O)[I]%18)C%19=C%18C%20=CC=CC=C%20N%19C, 52.3
CC(S[I]%21OS(=O)(C(F)(F)F)=O)(C)C%22=C%21C=CO%22, [O]S(=O)(C(F)(F)F)=O, CC(S[I]%21)(C)C%22=C%21C=CO%22, 44.6
O=C%23CCCC%24=CC%25=CC%26=CC=CC=C%26C=C%25C=C%24[I](OS(=O)(C(F)(F)F)=O)O%23, [O]S(=O)(C(F)(F)F)=O, O=C%23CCCC%24=CC%25=CC%26=CC=CC=C%26C=C%25C=C%24[I]O%23, 41.3
CC(O[I]1OC)(C)C2=C1C=CN2C, [O]C, CC(O[I]1)(C)C2=C1C=CN2C, 27.2
CC3(C)O[I](OC)C4=CC=CN=C43, [O]C, CC3(C)O[I]C4=CC=CN=C43, 29.1
FC5=C(F)C(F)=C(F)C(N(C)C(O6)=O)=C5[I]6OC, [O]C, FC5=C(F)C(F)=C(F)C(N6C)=C5[I]OC6=O, 26.5
FC7=C(F)C(F)=C(F)C8=C7[I](OC)OC8(C)C, [O]C, FC7=C(F)C(F)=C(F)C8=C7[I]OC8(C)C, 26.1
CN([I]9OC)S(C%10=C(C9=CC=C%11)C%11=CC=C%10)(=O)=O, [O]C, CN([I]9)S(C%10=C(C9=CC=C%11)C%11=CC=C%10)(=O)=O, 25.5
CC%12(C)O[I](OC)C%13=CC%14=CC=CC=C%14C=C%13%12, [O]C, CC%12(C)O[I]C%13=CC%14=CC=CC=C%14C=C%13%12, 31.5
O=C(N%15C)N(C)C%16=C([I]%15OC)C=C(C=CC=C%17)C%17=C%16, [O]C, O=C%15N(C)C%16=C([I]N%15C)C=C(C=CC=C%17)C%17=C%16, 19.6
O=C(N(C(C)=O)[I]%18OC)C%19=C%18C%20=CC=CC=C%20N%19C, [O]C, O=C(N(C(C)=O)[I]%18)C%19=C%18C%20=CC=CC=C%20N%19C, 30.3
CC(S[I]%21OC)(C)C%22=C%21C=CO%22, [O]C, CC(S[I]%21)(C)C%22=C%21C=CO%22, 15.3
O=C%23CCCC%24=CC%25=CC%26=CC=CC=C%26C=C%25C=C%24[I](OC)O%23, [O]C, O=C%23CCCC%24=CC%25=CC%26=CC=CC=C%26C=C%25C=C%24[I]O%23, 20.8
CC(O[I]1OC(F)(F)F)(C)C2=C1C=CN2C, [O]C(F)(F)F, CC(O[I]1)(C)C2=C1C=CN2C, 49.4
CC3(C)O[I](OC(F)(F)F)C4=CC=CN=C43, [O]C(F)(F)F, CC3(C)O[I]C4=CC=CN=C43, 51.4
FC5=C(F)C(F)=C(F)C(N(C)C(O6)=O)=C5[I]6OC(F)(F)F, [O]C(F)(F)F, FC5=C(F)C(F)=C(F)C(N6C)=C5[I]OC6=O, 43.1
FC7=C(F)C(F)=C(F)C8=C7[I](OC(F)(F)F)OC8(C)C, [O]C(F)(F)F, FC7=C(F)C(F)=C(F)C8=C7[I]OC8(C)C, 46.5
CN([I]9OC(F)(F)F)S(C%10=C(C9=CC=C%11)C%11=CC=C%10)(=O)=O, [O]C(F)(F)F, CN([I]9)S(C%10=C(C9=CC=C%11)C%11=CC=C%10)(=O)=O, 46.2
CC%12(C)O[I](OC(F)(F)F)C%13=CC%14=CC=CC=C%14C=C%13%12, [O]C(F)(F)F, CC%12(C)O[I]C%13=CC%14=CC=CC=C%14C=C%13%12, 53.7
O=C(N%15C)N(C)C%16=C([I]%15OC(F)(F)F)C=C(C=CC=C%17)C%17=C%16, [O]C(F)(F)F, O=C%15N(C)C%16=C([I]N%15C)C=C(C=CC=C%17)C%17=C%16, 45.0
O=C(N(C(C)=O)[I]%18OC(F)(F)F)C%19=C%18C%20=CC=CC=C%20N%19C, [O]C(F)(F)F, O=C(N(C(C)=O)[I]%18)C%19=C%18C%20=CC=CC=C%20N%19C, 50.0
CC(S[I]%21OC(F)(F)F)(C)C%22=C%21C=CO%22, [O]C(F)(F)F, CC(S[I]%21)(C)C%22=C%21C=CO%22, 39.6
O=C%23CCCC%24=CC%25=CC%26=CC=CC=C%26C=C%25C=C%24[I](OC(F)(F)F)O%23, [O]C(F)(F)F, O=C%23CCCC%24=CC%25=CC%26=CC=CC=C%26C=C%25C=C%24[I]O%23, 39.4
CC(O[I]1N(S(=O)(C(F)(F)F)=O)S(=O)(C(F)(F)F)=O)(C)C2=C1C=CN2C,

O=S([N]S(=O)(C(F)(F)F)=O)(C(F)(F)F)=O, CC(O[I]1)(C)C2=C1C=CN2C, 51.6
CC3(C)O[I](N(S(=O)(C(F)(F)F)=O)S(=O)(C(F)(F)F)=O)C4=CC=CN=C43,
O=S([N]S(=O)(C(F)(F)F)=O)(C(F)(F)F)=O, CC3(C)O[I]C4=CC=CN=C43, 52.9
FC5=C(F)C(F)=C(F)C(N(C)C(O6)=O)=C5[I]6N(S(=O)(C(F)(F)F)=O)S(=O)(C(F)(F)F)=O,
O=S([N]S(=O)(C(F)(F)F)=O)(C(F)(F)F)=O, FC5=C(F)C(F)=C(F)C(N6C)=C5[I]OC6=O, 41.1
FC7=C(F)C(F)=C(F)C8=C7[I](N(S(=O)(C(F)(F)F)=O)S(=O)(C(F)(F)F)=O)OC8(C)C,
O=S([N]S(=O)(C(F)(F)F)=O)(C(F)(F)F)=O, FC7=C(F)C(F)=C(F)C8=C7[I]OC8(C)C, 46.8
CN([I]9N(S(=O)(C(F)(F)F)=O)S(=O)(C(F)(F)F)=O)S(C%10=C(C9=CC=C%11)C%11=CC=C%10)(=O)=O, O=S([N]S(=O)(C(F)(F)F)=O)(C(F)(F)F)=O, CN([I]9)S(C%10=C(C9=CC=C%11)C%11=CC=C%10)(=O)=O, 49.3
CC%12(C)O[I](N(S(=O)(C(F)(F)F)=O)S(=O)(C(F)(F)F)=O)C%13=CC%14=CC=CC=C%14C=C%13%12, O=S([N]S(=O)(C(F)(F)F)=O)(C(F)(F)F)=O, CC%12(C)O[I]C%13=CC%14=CC=CC=C%14C=C%13%12, 55.2
O=C(N%15C)N(C)C%16=C([I]%15N(S(=O)(C(F)(F)F)=O)S(=O)(C(F)(F)F)=O)C=C(C=CC=C%17)C%17=C%16, O=S([N]S(=O)(C(F)(F)F)=O)(C(F)(F)F)=O, O=C%15N(C)C%16=C([I]N%15C)C=C(C=CC=C%17)C%17=C%16, 48.5
O=C(N(C(C)=O)[I]%18N(S(=O)(C(F)(F)F)=O)S(=O)(C(F)(F)F)=O)C%19=C%18C%20=CC=CC=C%20N%19C, O=S([N]S(=O)(C(F)(F)F)=O)(C(F)(F)F)=O, O=C(N(C(C)=O)[I]%18)C%19=C%18C%20=CC=CC=C%20N%19C, 48.7
CC(S[I]%21N(S(=O)(C(F)(F)F)=O)S(=O)(C(F)(F)F)=O)(C)C%22=C%21C=CO%22,
O=S([N]S(=O)(C(F)(F)F)=O)(C(F)(F)F)=O, CC(S[I]%21)(C)C%22=C%21C=CO%22, 44.8
O=C%23CCCC%24=CC%25=CC%26=CC=CC=C%26C=C%25C=C%24[I](N(S(=O)(C(F)(F)F)=O)S(=O)(C(F)(F)F)=O)O%23, O=S([N]S(=O)(C(F)(F)F)=O)(C(F)(F)F)=O, O=C%23CCCC%24=CC%25=CC%26=CC=CC=C%26C=C%25C=C%24[I]O%23, 42.9
CC(O[I]1C)(C)C2=C1C=CN2C, [H][C]([H])[H], CC(O[I]1)(C)C2=C1C=CN2C, 24.8
CC3(C)O[I](C)C4=CC=CN=C43, [H][C]([H])[H], CC3(C)O[I]C4=CC=CN=C43, 22.9
FC5=C(F)C(F)=C(F)C(N(C)C(O6)=O)=C5[I]6C, [H][C]([H])[H], FC5=C(F)C(F)=C(F)C(N6C)=C5[I]OC6=O, 31.4
FC7=C(F)C(F)=C(F)C8=C7[I](C)OC8(C)C, [H][C]([H])[H], FC7=C(F)C(F)=C(F)C8=C7[I]OC8(C)C, 23.7
CN([I]9C)S(C%10=C(C9=CC=C%11)C%11=CC=C%10)(=O)=O, [H][C]([H])[H], CN([I]9)S(C%10=C(C9=CC=C%11)C%11=CC=C%10)(=O)=O, 20.4
CC%12(C)O[I](C)C%13=CC%14=CC=CC=C%14C=C%13%12, [H][C]([H])[H], CC%12(C)O[I]C%13=CC%14=CC=CC=C%14C=C%13%12, 25.5
O=C(N%15C)N(C)C%16=C([I]%15C)C=C(C=CC=C%17)C%17=C%16, [H][C]([H])[H], O=C%15N(C)C%16=C([I]N%15C)C=C(C=CC=C%17)C%17=C%16, 16.4
O=C(N(C(C)=O)[I]%18C)C%19=C%18C%20=CC=CC=C%20N%19C, [H][C]([H])[H], O=C(N(C(C)=O)[I]%18)C%19=C%18C%20=CC=CC=C%20N%19C, 29.2
CC(S[I]%21C)(C)C%22=C%21C=CO%22, [H][C]([H])[H], CC(S[I]%21)(C)C%22=C%21C=CO%22, 11.4
O=C%23CCCC%24=CC%25=CC%26=CC=CC=C%26C=C%25C=C%24[I](C)O%23, [H][C]([H])[H], O=C%23CCCC%24=CC%25=CC%26=CC=CC=C%26C=C%25C=C%24[I]O%23, 19.0
F[I]1OC(C)(C)C2=C1C=CN2C, [F], CC(O[I]1)(C)C2=C1C=CN2C, 63.3
F[I]3C4=CC=CN=C4C(C)(C)O3, [F], CC3(C)O[I]C4=CC=CN=C43, 66.0
FC5=C(F)C(F)=C(F)C(N(C)C(O6)=O)=C5[I]6F, [F], FC5=C(F)C(F)=C(F)C(N6C)=C5[I]OC6=O, 59.1
FC7=C(F)C(F)=C(F)C8=C7[I](F)OC8(C)C, [F], FC7=C(F)C(F)=C(F)C8=C7[I]OC8(C)C, 61.1
F[I]9N(C)S(C%10=C(C9=CC=C%11)C%11=CC=C%10)(=O)=O, [F], CN([I]9)S(C%10=C(C9=CC=C%11)C%11=CC=C%10)(=O)=O, 62.1
F[I]%12C%13=CC%14=CC=CC=C%14C=C%13C(C)(C)O%12, [F], CC%12(C)O[I]C%13=CC%14=CC=CC=C%14C=C%13%12, 68.3
F[I]%15C%16=C(N(C)C(N%15C)=O)C=C%17C(C=CC=C%17)=C%16, [F],

O=C%15N(C)C%16=C([I]N%15C)C=C(C=CC=C%17)C%17=C%16, 59.3
F[I]%18N(C(C)=O)C(C%19=C%18C%20=CC=CC=C%20N%19C)=O, [F], O=C(N(C(C)=O)[I]%18)C%19=C%18C%20=CC=CC=C%20N%19C, 66.0
F[I]%21SC(C)(C)C%22=C%21C=CO%22, [F], CC(S[I]%21)(C)C%22=C%21C=CO%22, 52.5
F[I](C%23=CC%24=CC%25=CC=CC=C%25C=C%24C=C%23CCC%26)OC%26=O, [F], O=C%23CCCC%24=CC%25=CC%26=CC=CC=C%26C=C%25C=C%24[I]O%23, 55.3
CC(O[I]1C(F)(C(F)(F)F)F)(C)C2=C1C=CN2C, F[C](F)C(F)(F)F, CC(O[I]1)(C)C2=C1C=CN2C, 22.1
CC3(C)O[I](C(F)(C(F)(F)F)F)C4=CC=CN=C43, F[C](F)C(F)(F)F, CC3(C)O[I]C4=CC=CN=C43, 20.2
FC5=C(F)C(F)=C(F)C(N(C)C(O6)=O)=C5[I]6C(F)(C(F)(F)F)F, F[C](F)C(F)(F)F, FC5=C(F)C(F)=C(F)C(N6C)=C5[I]OC6=O, 23.0
FC7=C(F)C(F)=C(F)C8=C7[I](C(F)(C(F)(F)F)F)OC8(C)C, F[C](F)C(F)(F)F, FC7=C(F)C(F)=C(F)C8=C7[I]OC8(C)C, 18.6
CN([I]9C(F)(C(F)(F)F)F)S(C%10=C(C9=CC=C%11)C%11=CC=C%10)(=O)=O, F[C](F)C(F)(F)F, CN([I]9)S(C%10=C(C9=CC=C%11)C%11=CC=C%10)(=O)=O, 16.2
CC%12(C)O[I](C(F)(C(F)(F)F)F)C%13=CC%14=CC=CC=C%14C=C%13%12, F[C](F)C(F)(F)F, CC%12(C)O[I]C%13=CC%14=CC=CC=C%14C=C%13%12, 22.6
O=C(N%15C)N(C)C%16=C([I]%15C(F)(C(F)(F)F)F)C=C(C=CC=C%17)C%17=C%16, F[C](F)C(F)(F)F, O=C%15N(C)C%16=C([I]N%15C)C=C(C=CC=C%17)C%17=C%16, 13.9
O=C(N(C(C)=O)[I]%18C(F)(C(F)(F)F)F)C%19=C%18C%20=CC=CC=C%20N%19C, F[C](F)C(F)(F)F, O=C(N(C(C)=O)[I]%18)C%19=C%18C%20=CC=CC=C%20N%19C, 21.3
CC(S[I]%21C(F)(C(F)(F)F)F)(C)C%22=C%21C=CO%22, F[C](F)C(F)(F)F, CC(S[I]%21)(C)C%22=C%21C=CO%22, 8.7
O=C%23CCCC%24=CC%25=CC%26=CC=CC=C%26C=C%25C=C%24[I](C(F)(C(F)(F)F)F)O%23, F[C](F)C(F)(F)F, O=C%23CCCC%24=CC%25=CC%26=CC=CC=C%26C=C%25C=C%24[I]O%23, 14.8
O=C1O[I](C(F)(F)F)C2=C1N=NN2C, F[C](F)F, O=C1O[I]C2=C1N=NN2C, 14.9
O=C3O[I](C(F)(F)F)C4=C3N=CS4, F[C](F)F, O=C3O[I]C4=C3N=CS4, 23.8
CC(O[I]5C(F)(F)F)(C)C6=C5C=CN6C, F[C](F)F, CC(O[I]5)(C)C6=C5C=CN6C, 23.8
O=C7O[I](C(F)(F)F)C8=C7C(C=CC=C9C=C%10)=C9C%11=C%10C=CC=C%118, F[C](F)F, O=C7O[I]C8=C7C(C=CC=C9C=C%10)=C9C%11=C%10C=CC=C%118, 26.5
CC%12(C)O[I](C(F)(F)F)C%13=CC=CN=C%13%12, F[C](F)F, CC%12(C)O[I]C%13=CC=CN=C%13%12, 22.2
O=C(O[I]%14C(F)(F)F)C%15=C%14C%16=C(C%17=C%15C=CC=N%17)N=CC=C%16, F[C](F)F, O=C(O[I]%14)C%15=C%14C%16=C(C%17=C%15C=CC=N%17)N=CC=C%16, 26.4
FC%18=C(F)C(F)=C(F)C(N(C)C(O%19)=O)=C%18[I]%19C(F)(F)F, F[C](F)F, FC%18=C(F)C(F)=C(F)C(N%19C)=C%18[I]OC%19=O, 24.5
FC%20=C(F)C(F)=C(F)C%21=C%20[I](C(F)(F)F)OC%21(C)C, F[C](F)F, FC%20=C(F)C(F)=C(F)C%21=C%20[I]OC%21(C)C, 20.7
CN([I]%22C(F)(F)F)S(C%23=C(C%22=CC=C%24)C%24=CC=C%23)(=O)=O, F[C](F)F, CN([I]%22)S(C%23=C(C%22=CC=C%24)C%24=CC=C%23)(=O)=O, 17.9
CC%25(C)O[I](C(F)(F)F)C%26=CC%27=CC=CC=C%27C=C%26%25, F[C](F)F, CC%25(C)O[I]C%26=CC%27=CC=CC=C%27C=C%26%25, 24.6
O=C(N%28C)N(C)C%29=C([I]%28C(F)(F)F)C=C(C=CC=C%30)C%30=C%29, F[C](F)F, O=C%28N(C)C%29=C([I]N%28C)C=C(C=CC=C%30)C%30=C%29, 15.3
O=C(O[I]%31C(F)(F)F)C%32=C%31C=C(N(C(C)=O)C=C%33)C%33=C%32, F[C](F)F, O=C(O[I]%31)C%32=C%31C=C(N(C(C)=O)C=C%33)C%33=C%32, 31.4
O=C%34O[I](C(F)(F)F)C%35=C%36C(N(C(C)=O)C=C%36)=CC=C%35%34, F[C](F)F, O=C%34O[I]C%35=C%36C(N(C(C)=O)C=C%36)=CC=C%35%34, 25.6
O=C(N(C(C)=O)[I]%37C(F)(F)F)C%38=C%37C%39=CC=CC=C%39N%38C, F[C](F)F, O=C(N(C(C)=O)[I]%37)C%38=C%37C%39=CC=CC=C%39N%38C, 23.9
O=C(O[I]%40C(F)(F)F)C%41=C%40C%42=CC=CC=C%42C%41, F[C](F)F,

O=C(O[I]%40)C%41=C%40C%42=CC=CC=C%42C%41, 25.0
CC(S[I]%43C(F)(F)F)(C)C%44=C%43C=CO%44, F[C](F)F, CC(S[I]%43)(C)C%44=C%43C=CO%44, 10.3
O=C(O[I]%45C(F)(F)F)C(C%45=C%46)=CC%47=C%46C%48=CC=CC=C%48O%47, F[C](F)F, O=C(O[I]%45)C(C%45=C%46)=CC%47=C%46C%48=CC=CC=C%48O%47, 31.7
O=C(O[I]%49C(F)(F)F)C%50=C%49CCCC%50, F[C](F)F, O=C(O[I]%49)C%50=C%49CCCC%50, 32.8
O=C(O[I]%51C(F)(F)F)C%52=C%51C%53=CC=CC=C%53S%52, F[C](F)F, O=C(O[I]%51)C%52=C%51C%53=CC=CC=C%53S%52, 25.4
O=C(O[I]%54C(F)(F)F)C%55=C%54C%56=CC=CC=C%56O%55, F[C](F)F, O=C(O[I]%54)C%55=C%54C%56=CC=CC=C%56O%55, 26.2
O=C%57O[I](C(F)(F)F)C%58=C%57C=C%59C%58=CC=CC=C%59, F[C](F)F, O=C%57O[I]C%58=C%57C=C%59C%58=CC=CC=C%59, 24.2
O=C%60CCCC%61=CC%62=CC%63=CC=CC=C%63C=C%62C=C%61[I](C(F)(F)F)O%60, F[C](F)F, O=C%60CCCC%61=CC%62=CC%63=CC=CC=C%63C=C%62C=C%61[I]O%60, 16.1
O=C%64O[I](C(F)(F)F)C%65=C%64C=CC%66=CC%67=CC=CC=C%67N=C%66%65, F[C](F)F, O=C%64O[I]C%65=C%64C=CC%66=CC%67=CC=CC=C%67N=C%66%65, 29.5
O=C1O[I](C(F)(F)F)C2=C3C(OC=C3)=CC=C21, F[C](F)F, O=C1O[I]C2=C3C(OC=C3)=CC=C21, 26.4
FC1=C(F)C(F)=C(F)C2=C1[I](C(F)(F)F)OC2=O, F[C](F)F, FC1=C(F)C(F)=C(F)C2=C1[I]OC2=O, 26.7
O=C1O[I](Cl)C2=C1N=NN2C, [Cl], O=C1O[I]C2=C1N=NN2C, 31.7
O=C3O[I](Cl)C4=C3N=CS4, [Cl], O=C3O[I]C4=C3N=CS4, 39.1
Cl[I]5OC(C)(C)C6=C5C=CN6C, [Cl], CC(O[I]5)(C)C6=C5C=CN6C, 42.6
O=C7O[I](Cl)C8=C7C(C=CC=C9C=C%10)=C9C%11=C%10C=CC=C%118, [Cl], O=C7O[I]C8=C7C(C=CC=C9C=C%10)=C9C%11=C%10C=CC=C%118, 41.7
Cl[I]%12C%13=CC=CN=C%13C(C)(C)O%12, [Cl], CC%12(C)O[I]C%13=CC=CN=C%13%12, 44.3
O=C(O[I]%14Cl)C%15=C%14C%16=C(C%17=C%15C=CC=N%17)N=CC=C%16, [Cl], O=C(O[I]%14)C%15=C%14C%16=C(C%17=C%15C=CC=N%17)N=CC=C%16, 41.9
FC%18=C(F)C(F)=C(F)C(N(C)C(O%19)=O)=C%18[I]%19Cl, [Cl], FC%18=C(F)C(F)=C(F)C(N%19C)=C%18[I]OC%19=O, 36.9
FC%20=C(F)C(F)=C(F)C%21=C%20[I](Cl)OC%21(C)C, [Cl], FC%20=C(F)C(F)=C(F)C%21=C%20[I]OC%21(C)C, 37.7
Cl[I]%22N(C)S(C%23=C(C%22=CC=C%24)C%24=CC=C%23)(=O)=O, [Cl], CN([I]%22)S(C%23=C(C%22=CC=C%24)C%24=CC=C%23)(=O)=O, 39.2
Cl[I]%25C%26=CC%27=CC=CC=C%27C=C%26C(C)(C)O%25, [Cl], CC%25(C)O[I]C%26=CC%27=CC=CC=C%27C=C%26%25, 46.5
Cl[I]%28C%29=C(N(C)C(N%28C)=O)C=C%30C(C=CC=C%30)=C%29, [Cl], O=C%28N(C)C%29=C([I]N%28C)C=C(C=CC=C%30)C%30=C%29, 37.9
Cl[I]%31OC(C%32=C%31C=C(N(C(C)=O)C=C%33)C%33=C%32)=O, [Cl], O=C(O[I]%31)C%32=C%31C=C(N(C(C)=O)C=C%33)C%33=C%32, 47.4
Cl[I](O%34)C%35=C%36C(N(C(C)=O)C=C%36)=CC=C%35C%34=O, [Cl], O=C%34O[I]C%35=C%36C(N(C(C)=O)C=C%36)=CC=C%35%34, 43.0
Cl[I]%37N(C(C)=O)C(C%38=C%37C%39=CC=CC=C%39N%38C)=O, [Cl], O=C(N(C(C)=O)[I]%37)C%38=C%37C%39=CC=CC=C%39N%38C, 42.6
Cl[I]%40OC(C%41=C%40C%42=CC=CC=C%42C%41)=O, [Cl], O=C(O[I]%40)C%41=C%40C%42=CC=CC=C%42C%41, 40.7
Cl[I]%43SC(C)(C)C%44=C%43C=CO%44, [Cl], CC(S[I]%43)(C)C%44=C%43C=CO%44, 33.3
Cl[I]%45OC(C(C%45=C%46)=CC%47=C%46C%48=CC=CC=C%48O%47)=O, [Cl], O=C(O[I]%45)C(C%45=C%46)=CC%47=C%46C%48=CC=CC=C%48O%47, 48.3
Cl[I]%49OC(C%50=C%49CCCC%50)=O, [Cl], O=C(O[I]%49)C%50=C%49CCCC%50, 49.1
O=C(O[I]%51Cl)C%52=C%51C%53=CC=CC=C%53S%52, [Cl], O=C(O[I]%51)C%52=C%51C%53=CC=CC=C%53S%52, 40.6

O=C(O[I]%54Cl)C%55=C%54C%56=CC=CC=C%56O%55, [Cl], O=C(O[I]%54)C%55=C%54C%56=CC=CC=C%56O%55, 40.4
Cl[I](OC%57=O)C%58=C%57C=C%59C%58=CC=CC=C%59, [Cl], O=C%57O[I]C%58=C%57C=C%59C%58=CC=CC=C%59, 41.2
Cl[I](C%60=CC%61=CC%62=CC=CC=C%62C=C%61C=C%60CCC%63)OC%63=O, [Cl], O=C%60CCCC%61=CC%62=CC%63=CC=CC=C%63C=C%62C=C%61[I]O%60, 33.1
Cl[I](OC%64=O)C%65=C%64C=CC%66=CC%67=CC=CC=C%67N=C%66%65, [Cl], O=C%64O[I]C%65=C%64C=CC%66=CC%67=CC=CC=C%67N=C%66%65, 38.5
Cl[I](O1)C2=C3C(OC=C3)=CC=C2C1=O, [Cl], O=C1O[I]C2=C3C(OC=C3)=CC=C21, 43.6
FC1=C(F)C(F)=C(F)C2=C1[I](Cl)OC2=O, [Cl], FC1=C(F)C(F)=C(F)C2=C1[I]OC2=O, 38.6
Cl[I](Cl)C1=CSC=C1, [Cl], Cl[I]C1=CSC=C1, 42.0
Cl[I](Cl)C(C=CC=C2)=C2C3=CC=CC=N3, [Cl], Cl[I]C(C=CC=C2)=C2C3=CC=CC=N3, 42.1
Cl[I](Cl)C4=CC5=CC=CC=C5C=C4, [Cl], Cl[I]C4=CC5=CC=CC=C5C=C4, 42.8
Cl[I](Cl)C6=CC=CC7=CC=CC=C76, [Cl], Cl[I]C6=CC=CC7=CC=CC=C76, 41.8
CC8=CC(C)=CC(C)=C8[I](Cl)Cl, [Cl], CC8=CC(C)=CC(C)=C8[I]Cl, 43.8
Cl[I](Cl)C(C=C9)=CN9C, [Cl], Cl[I]C(C=C9)=CN9C, 43.1
Cl[I](Cl)C%10=CN(C(C)=O)C%11=CC=CC=C%11%10, [Cl], Cl[I]C%10=CN(C(C)=O)C%11=CC=CC=C%11%10, 41.5
Cl[I](Cl)C%12=COC=C%12, [Cl], Cl[I]C%12=COC=C%12, 41.9
Cl[I](Cl)C%13=C(C=CC=C%14)C%14=CC%15=CC=CC=C%15%13, [Cl], Cl[I]C%13=C(C=CC=C%14)C%14=CC%15=CC=CC=C%15%13, 42.3
FC%16=C([I](Cl)Cl)C(F)=C(F)C(F)=C%16F, [Cl], FC%16=C([I]Cl)C(F)=C(F)C(F)=C%16F, 38.5
F[I](F)C1=CSC=C1, [F], F[I]C1=CSC=C1, 78.9
F[I](F)C(C=CC=C2)=C2C3=CC=CC=N3, [F], F[I]C(C=CC=C2)=C2C3=CC=CC=N3, 76.0
F[I](F)C4=CC5=CC=CC=C5C=C4, [F], F[I]C4=CC5=CC=CC=C5C=C4, 79.6
F[I](F)C6=CC=CC7=CC=CC=C76, [F], F[I]C6=CC=CC7=CC=CC=C76, 76.8
CC8=CC(C)=CC(C)=C8[I](F)F, [F], CC8=CC(C)=CC(C)=C8[I]F, 77.9
F[I](F)C(C=C9)=CN9C, [F], F[I]C(C=C9)=CN9C, 80.3
F[I](F)C%10=CN(C(C)=O)C%11=CC=CC=C%11%10, [F], F[I]C%10=CN(C(C)=O)C%11=CC=CC=C%11%10, 77.0
F[I](F)C%12=COC=C%12, [F], F[I]C%12=COC=C%12, 78.5
F[I](F)C%13=C(C=CC=C%14)C%14=CC%15=CC=CC=C%15%13, [F], F[I]C%13=C(C=CC=C%14)C%14=CC%15=CC=CC=C%15%13, 76.9
FC%16=C([I](F)F)C(F)=C(F)C(F)=C%16F, [F], FC%16=C([I]F)C(F)=C(F)C(F)=C%16F, 74.1
O=C(O[I](OC(C)=O)C1=CSC=C1)C, [O]C(C)=O, O=C(O[I]C1=CSC=C1)C, 46.6
O=C(O[I](OC(C)=O)C(C=CC=C2)=C2C3=CC=CC=N3)C, [O]C(C)=O, O=C(O[I]C(C=CC=C2)=C2C3=CC=CC=N3)C, 46.8
O=C(O[I](OC(C)=O)C4=CC5=CC=CC=C5C=C4)C, [O]C(C)=O, O=C(O[I]C4=CC5=CC=CC=C5C=C4)C, 46.1
O=C(O[I](OC(C)=O)C6=CC=CC7=CC=CC=C76)C, [O]C(C)=O, O=C(O[I]C6=CC=CC7=CC=CC=C76)C, 45.9
CC8=CC(C)=CC(C)=C8[I](OC(C)=O)OC(C)=O, [O]C(C)=O, CC8=CC(C)=CC(C)=C8[I]OC(C)=O, 47.1
CN9C=CC([I](OC(C)=O)OC(C)=O)=C9, [O]C(C)=O, CN9C=CC([I]OC(C)=O)=C9, 46.3
O=C(N%10C%11=CC=CC=C%11C([I](OC(C)=O)OC(C)=O)=C%10)C, [O]C(C)=O, O=C(N%10C%11=CC=CC=C%11C([I]OC(C)=O)=C%10)C, 45.5
O=C(O[I](OC(C)=O)C%12=COC=C%12)C, [O]C(C)=O, O=C(O[I]C%12=COC=C%12)C, 46.4
O=C(O[I](OC(C)=O)C%13=C(C=CC=C%14)C%14=CC%15=CC=CC=C%15%13)C, [O]C(C)=O, O=C(O[I]C%13=C(C=CC=C%14)C%14=CC%15=CC=CC=C%15%13)C, 46.1
FC%16=C([I](OC(C)=O)OC(C)=O)C(F)=C(F)C(F)=C%16F, [O]C(C)=O, FC%16=C([I]OC(C)=O)C(F)=C(F)C(F)=C%16F, 45.0
O=C(O[I](OC(C(F)(F)F)=O)C1=CSC=C1)C(F)(F)F, [O]C(C(F)(F)F)=O,

O=C(O[I]C1=CSC=C1)C(F)(F)F, 52.2
O=C(O[I](OC(C(F)(F)F)=O)C(C=CC=C2)=C2C3=CC=CC=N3)C(F)(F)F, [O]C(C(F)(F)F)=O, O=C(O[I]C(C=CC=C2)=C2C3=CC=CC=N3)C(F)(F)F, 50.5
O=C(O[I](OC(C(F)(F)F)=O)C4=CC5=CC=CC=C5C=C4)C(F)(F)F, [O]C(C(F)(F)F)=O, O=C(O[I]C4=CC5=CC=CC=C5C=C4)C(F)(F)F, 52.9
O=C(O[I](OC(C(F)(F)F)=O)C6=CC=CC7=CC=CC=C76)C(F)(F)F, [O]C(C(F)(F)F)=O, O=C(O[I]C6=CC=CC7=CC=CC=C76)C(F)(F)F, 52.9
CC8=CC(C)=CC(C)=C8[I](OC(C(F)(F)F)=O)OC(C(F)(F)F)=O, [O]C(C(F)(F)F)=O, CC8=CC(C)=CC(C)=C8[I]OC(C(F)(F)F)=O, 53.3
CN9C=CC([I](OC(C(F)(F)F)=O)OC(C(F)(F)F)=O)=C9, [O]C(C(F)(F)F)=O, CN9C=CC([I]OC(C(F)(F)F)=O)=C9, 52.1
O=C(N%10C%11=CC=CC=C%11C([I](OC(C(F)(F)F)=O)OC(C(F)(F)F)=O)=C%10)C, [O]C(C(F)(F)F)=O, O=C(N%10C%11=CC=CC=C%11C([I]OC(C(F)(F)F)=O)=C%10)C, 51.1
O=C(O[I](OC(C(F)(F)F)=O)C%12=COC=C%12)C(F)(F)F, [O]C(C(F)(F)F)=O, O=C(O[I]C%12=COC=C%12)C(F)(F)F, 51.9
O=C(O[I](OC(C(F)(F)F)=O)C%13=C(C=CC=C%14)C%14=CC%15=CC=CC=C%15%13)C(F)(F)F, [O]C(C(F)(F)F)=O, O=C(O[I]C%13=C(C=CC=C%14)C%14=CC%15=CC=CC=C%15%13)C(F)(F)F, 52.1
FC%16=C([I](OC(C(F)(F)F)=O)OC(C(F)(F)F)=O)C(F)=C(F)C(F)=C%16F, [O]C(C(F)(F)F)=O, FC%16=C([I]OC(C(F)(F)F)=O)C(F)=C(F)C(F)=C%16F, 50.2

(4) Fig4_AD_C_102HVIs
F[I](C1=CSC=C1)C2=CC=CC=C2, [C]1=CSC=C1, F[I]C1=CC=CC=C1, 57.5
F[I](C3=NC=CS3)C4=CC=CC=C4, C1=CN=[C]S1, F[I]C1=CC=CC=C1, 52.8
F[I](SC(C)(C)C)C5=CC=CC=C5, [S]C(C)(C)C, F[I]C1=CC=CC=C1, 42.1
F[I](OS(=O)(C6=CC=CC=C6)=O)C7=CC=CC=C7, [O]S(=O)(C1=CC=CC=C1)=O, F[I]C1=CC=CC=C1, 61.6
F[I](SC8=C(C)C=C(C)C=C8C)C9=CC=CC=C9, [S]C1=C(C)C=C(C)C=C1C, F[I]C1=CC=CC=C1, 30.5
F[I]([Se]C(C)(C)C)C%10=CC=CC=C%10, [Se]C(C)(C)C, F[I]C1=CC=CC=C1, 38.1
F[I]([Se]C%11=C(C)C=C(C)C=C%11C)C%12=CC=CC=C%12, [Se]C1=C(C)C=C(C)C=C1C, F[I]C1=CC=CC=C1, 34.8
F[I](C%13=C(C=CC=C%14C=CC%15=CC=C%16)C%14=C%15C%16=C%13)C%17=CC=CC=C%17, C12=[C]C=C3C(C4=CC=C3)=C1C(C=C4)=CC=C2, F[I]C1=CC=CC=C1, 55.2
F[I](OCC)C%18=CC=CC=C%18, [O]CC, F[I]C1=CC=CC=C1, 47.3
F[I](OCC(F)(F)F)C%19=CC=CC=C%19, [O]CC(F)(F)F, F[I]C1=CC=CC=C1, 53.3
F[I](C%20=CC=C(C=CC=C%21)C%21=C%20)C%22=CC=CC=C%22, C12=CC=[C]C=C1C=CC=C2, F[I]C1=CC=CC=C1, 54.2
F[I](C%23=C(C=CC=C%24)C%24=CC=C%23)C%25=CC=CC=C%25, C12=[C]C=CC=C1C=CC=C2, F[I]C1=CC=CC=C1, 55.0
F[I](C%26=C(C)C=C(C)C=C%26C)C%27=CC=CC=C%27, CC1=[C]C(C)=CC(C)=C1, F[I]C1=CC=CC=C1, 56.6
F[I](C%28=COC=C%28)C%29=CC=CC=C%29, [C]1=COC=C1, F[I]C1=CC=CC=C1, 61.0
F[I](C%30=CC=C(C(C=CC=C%31)=C%31O%32)C%32=C%30)C%33=CC=CC=C%33, C12=CC=[C]C=C1OC3=C2C=CC=C3, F[I]C1=CC=CC=C1, 53.8
F[I](C%34CCCCC%34)C%35=CC=CC=C%35, [C]1CCCCC1, F[I]C1=CC=CC=C1, 40.3
F[I](C#CC%36=CC=CC=C%36)C%37=CC=CC=C%37, [C]#CC1=CC=CC=C1, F[I]C1=CC=CC=C1, 71.3
F[I](N%38C(C=CC=C%39)=C%39C%40=C%38C=CC=C%40)C%41=CC=CC=C%41, C1(C=CC=C2)=C2C(C=CC=C3)=C3[N]1, F[I]C1=CC=CC=C1, 34.9
F[I](CC%42=CC=CC=C%42)C%43=CC=CC=C%43, [C]C1=CC=CC=C1, F[I]C1=CC=CC=C1, 31.0
F[I](C%44=CC=C(C=C(C=CC=C%45)C%45=C%46)C%46=C%44)C%47=CC=CC=C%47,

C12=CC=[C]C=C1C=C3C(C=CC=C3)=C2, F[I]C1=CC=CC=C1, 54.3
F[I](C%49(CC%50C%51)C[C@H](C%50)C[C@H]%51C%49)C%52=CC=CC=C%52,
[C]1(CC2C3)C[C@H](C2)C[C@H]3C1, F[I]C1=CC=CC=C1, 42.1
F[I](C%53=C(N=C(C=CC=C%54)C%54=C%55)C%55=CC=C%53)C%56=CC=CC=C%56,
C12=[C]C=CC=C1C=C3C(C=CC=C3)=N2, F[I]C1=CC=CC=C1, 57.3
F[I](C%57=C(F)C(F)=C(F)C(F)=C%57F)C%58=CC=CC=C%58, FC1=[C]C(F)=C(F)C(F)=C1F,
F[I]C1=CC=CC=C1, 58.9
F[I](OC(C%59=C(F)C(F)=C(F)C(F)=C%59F)=O)C%60=CC=CC=C%60,
FC1=C(C([O])=O)C(F)=C(F)C(F)=C1F, F[I]C1=CC=CC=C1, 58.5
O=C1O[I](C2=CSC=C2)C3=CC=CC=C31, [C]1=CSC=C1, O=C1O[I]C2=CC=CC=C21, 51.4
O=C4O[I](C5=NC=CS5)C6=CC=CC=C64, C1=CN=[C]S1, O=C1O[I]C2=CC=CC=C21, 45.4
O=C7O[I](SC(C)(C)C)C8=CC=CC=C87, [S]C(C)(C)C, O=C1O[I]C2=CC=CC=C21, 35.0
O=S(O[I](O9)C%10=CC=CC=C%10C9=O)(C%11=CC=CC=C%11)=O, [O]S(=O)(C1=CC=CC=C1)=O,
O=C1O[I]C2=CC=CC=C21, 52.0
CC(C=C(C)C=C%12C)=C%12S[I](O%13)C%14=CC=CC=C%14C%13=O, [S]C1=C(C)C=C(C)C=C1C,
O=C1O[I]C2=CC=CC=C21, 22.7
O=C%15O[I]([Se]C(C)(C)C)C%16=CC=CC=C%16%15, [Se]C(C)(C)C, O=C1O[I]C2=CC=CC=C21,
32.4
CC%17=CC(C)=CC(C)=C%17[Se][I](O%18)C%19=CC=CC=C%19C%18=O,
[Se]C1=C(C)C=C(C)C=C1C, O=C1O[I]C2=CC=CC=C21, 28.4
O=C%20O[I](C%21=C(C=CC=C%22C=CC%23=CC=C%24)C%22=C%23C%24=C%21)C%25=CC=C
C=C%25%20, C12=[C]C=C3C(C4=CC=C3)=C1C(C=C4)=CC=C2, O=C1O[I]C2=CC=CC=C21, 49.0
O=C%26O[I](OCC)C%27=CC=CC=C%27%26, [O]CC, O=C1O[I]C2=CC=CC=C21, 38.0
O=C%28O[I](OCC(F)(F)F)C%29=CC=CC=C%29%28, [O]CC(F)(F)F, O=C1O[I]C2=CC=CC=C21, 43.1
O=C%30O[I](C%31=CC=C(C=CC=C%32)C%32=C%31)C%33=CC=CC=C%33%30,
C12=CC=[C]C=C1C=CC=C2, O=C1O[I]C2=CC=CC=C21, 48.3
O=C%34O[I](C%35=C(C=CC=C%36)C%36=CC=C%35)C%37=CC=CC=C%37%34,
C12=[C]C=CC=C1C=CC=C2, O=C1O[I]C2=CC=CC=C21, 48.9
CC(C=C(C)C=C%38C)=C%38[I](O%39)C%40=CC=CC=C%40C%39=O, CC1=[C]C(C)=CC(C)=C1,
O=C1O[I]C2=CC=CC=C21, 51.1
O=C%41O[I](C%42=COC=C%42)C%43=CC=CC=C%43%41, [C]1=COC=C1,
O=C1O[I]C2=CC=CC=C21, 54.7
O=C%44O[I](C%45=CC=C(C(C=CC=C%46)=C%46O%47)C%47=C%45)C%48=CC=CC=C%48%44,
C12=CC=[C]C=C1OC3=C2C=CC=C3, O=C1O[I]C2=CC=CC=C21, 48.0
O=C%49O[I](C%50CCCCC%50)C%51=CC=CC=C%51%49, [C]1CCCCC1,
O=C1O[I]C2=CC=CC=C21, 36.0
O=C%52O[I](C#CC%53=CC=CC=C%53)C%54=CC=CC=C%54%52, [C]#CC1=CC=CC=C1,
O=C1O[I]C2=CC=CC=C21, 63.7
O=C%55O[I](N%56C(C=CC=C%57)=C%57C%58=C%56C=CC=C%58)C%59=CC=CC=C%59%55,
C1(C=CC=C2)=C2C(C=CC=C3)=C3[N]1, O=C1O[I]C2=CC=CC=C21, 25.6
O=C%60O[I](CC%61=CC=CC=C%61)C%62=CC=CC=C%62%60, [C]C1=CC=CC=C1,
O=C1O[I]C2=CC=CC=C21, 25.5
O=C%63O[I](C%64=CC=C(C=C(C=CC=C%65)C%65=C%66)C%66=C%64)C%67=CC=CC=C%67%6
3, C12=CC=[C]C=C1C=C3C(C=CC=C3)=C2, O=C1O[I]C2=CC=CC=C21, 48.4
O=C%70O[I](C%71(CC%72C%73)C[C@H](C%72)C[C@H]%73C%71)C%74=CC=CC=C%74%70,
[C]1(CC2C3)C[C@H](C2)C[C@H]3C1, O=C1O[I]C2=CC=CC=C21, 36.6
O=C%75O[I](C%76=C(N=C(C=CC=C%77)C%77=C%78)C%78=CC=C%76)C%79=CC=CC=C%79%7
5, C12=[C]C=CC=C1C=C3C(C=CC=C3)=N2, O=C1O[I]C2=CC=CC=C21, 51.7
FC(C(F)=C(F)C(F)=C%80F)=C%80[I](O%81)C%82=CC=CC=C%82C%81=O,
FC1=[C]C(F)=C(F)C(F)=C1F, O=C1O[I]C2=CC=CC=C21, 50.4
FC%83=C(C(O[I](O%84)C%85=CC=CC=C%85C%84=O)=O)C(F)=C(F)C(F)=C%83F,

FC1=C(C([O])=O)C(F)=C(F)C(F)=C1F, O=C1O[I]C2=CC=CC=C21, 49.4
O=C(O[I]1OCC)C2=C1C=CN2C(C)=O, [O]CC, O=C(O[I]1)C2=C1C=CN2C(C)=O, 30.9
O=C3O[I](OCC)C4=CC5=CC=CC=C5C=C43, [O]CC, O=C3O[I]C4=CC5=CC=CC=C5C=C43, 36.3
O=S(C6=C7C=CC=C6)(O[I]7OCC)=O, [O]CC, O=S(C6=C7C=CC=C6)(O[I]7)=O, 38.6
O=C(O8)N(C)C9=C([I]8OCC)C=CC=C9, [O]CC, O=C8N(C)C9=C([I]O8)C=CC=C9, 34.1
CC(S[I]%10OCC)(C)C%11=C%10C=CC=C%11, [O]CC, CC(S[I]%10)(C)C%11=C%10C=CC=C%11, 20.4
FC(C(O[I]%12OCC)(C(F)(F)F)C%13=C%12C=CC=C%13)(F)F, [O]CC, FC(C(O[I]%12)(C(F)(F)F)C%13=C%12C=CC=C%13)(F)F, 38.8
O=C(N(C(C)=O)[I]%14OCC)C%15=C%14C=CC=C%15, [O]CC, O=C(N(C(C)=O)[I]%14)C%15=C%14C=CC=C%15, 34.7
O=C(O[I]%16OCC)C%17=C%16C(C)=CC=C%17, [O]CC, O=C(O[I]%16)C%17=C%16C(C)=CC=C%17, 31.1
O=C(O[I]%18OCC)C%19=C%18C=CO%19, [O]CC, O=C(O[I]%18)C%19=C%18C=CO%19, 29.4
O=C(O[I]1OCC(F)(F)F)C2=C1C=CN2C(C)=O, [O]CC(F)(F)F, O=C(O[I]1)C2=C1C=CN2C(C)=O, 37.8
O=C3O[I](OCC(F)(F)F)C4=CC5=CC=CC=C5C=C43, [O]CC(F)(F)F, O=C3O[I]C4=CC5=CC=CC=C5C=C43, 43.4
O=S(C6=C7C=CC=C6)(O[I]7OCC(F)(F)F)=O, [O]CC(F)(F)F, O=S(C6=C7C=CC=C6)(O[I]7)=O, 43.9
O=C(O8)N(C)C9=C([I]8OCC(F)(F)F)C=CC=C9, [O]CC(F)(F)F, O=C8N(C)C9=C([I]O8)C=CC=C9, 41.0
CC(S[I]%10OCC(F)(F)F)(C)C%11=C%10C=CC=C%11, [O]CC(F)(F)F, CC(S[I]%10)(C)C%11=C%10C=CC=C%11, 30.1
FC(C(O[I]%12OCC(F)(F)F)(C(F)(F)F)C%13=C%12C=CC=C%13)(F)F, [O]CC(F)(F)F, FC(C(O[I]%12)(C(F)(F)F)C%13=C%12C=CC=C%13)(F)F, 45.9
O=C(N(C(C)=O)[I]%14OCC(F)(F)F)C%15=C%14C=CC=C%15, [O]CC(F)(F)F, O=C(N(C(C)=O)[I]%14)C%15=C%14C=CC=C%15, 42.2
O=C(O[I]%16OCC(F)(F)F)C%17=C%16C(C)=CC=C%17, [O]CC(F)(F)F, O=C(O[I]%16)C%17=C%16C(C)=CC=C%17, 37.6
O=C(O[I]%18OCC(F)(F)F)C%19=C%18C=CO%19, [O]CC(F)(F)F, O=C(O[I]%18)C%19=C%18C=CO%19, 35.9
O=C(O[I]1C2=CC=C(C=CC=C3)C3=C2)C4=C1C=CN4C(C)=O, C12=CC=[C]C=C1C=CC=C2, O=C(O[I]1)C2=C1C=CN2C(C)=O, 44.2
O=C5O[I](C6=CC=C(C=CC=C7)C7=C6)C8=CC9=CC=CC=C9C=C85, C12=CC=[C]C=C1C=CC=C2, O=C3O[I]C4=CC5=CC=CC=C5C=C43, 48.2
O=S(C%10=C%11C=CC=C%10)(O[I]%11C%12=CC(C=CC=C%13)=C%13C=C%12)=O, C12=CC=[C]C=C1C=CC=C2, O=S(C6=C7C=CC=C6)(O[I]7)=O, 57.1
O=C(O%14)N(C)C%15=C([I]%14C%16=CC(C=CC=C%17)=C%17C=C%16)C=CC=C%15, C12=CC=[C]C=C1C=CC=C2, O=C8N(C)C9=C([I]O8)C=CC=C9, 48.5
CC(S[I]%18C%19=CC(C=CC=C%20)=C%20C=C%19)(C)C%21=C%18C=CC=C%21, C12=CC=[C]C=C1C=CC=C2, CC(S[I]%10)(C)C%11=C%10C=CC=C%11, 25.2
FC(C(O[I]%22C%23=CC(C=CC=C%24)=C%24C=C%23)(C(F)(F)F)C%25=C%22C=CC=C%25)(F)F, C12=CC=[C]C=C1C=CC=C2, FC(C(O[I]%12)(C(F)(F)F)C%13=C%12C=CC=C%13)(F)F, 48.8
O=C(N(C(C)=O)[I]%26C%27=CC(C=CC=C%28)=C%28C=C%27)C%29=C%26C=CC=C%29, C12=CC=[C]C=C1C=CC=C2, O=C(N(C(C)=O)[I]%14)C%15=C%14C=CC=C%15, 43.3
O=C(O[I]%30C%31=CC(C=CC=C%32)=C%32C=C%31)C%33=C%30C(C)=CC=C%33, C12=CC=[C]C=C1C=CC=C2, O=C(O[I]%16)C%17=C%16C(C)=CC=C%17, 41.7
O=C(O[I]%34C%35=CC(C=CC=C%36)=C%36C=C%35)C%37=C%34C=CO%37, C12=CC=[C]C=C1C=CC=C2, O=C(O[I]%18)C%19=C%18C=CO%19, 43.9
O=C(O[I]1C#CC2=CC=CC=C2)C3=C1C=CN3C(C)=O, [C]#CC1=CC=CC=C1, O=C(O[I]1)C2=C1C=CN2C(C)=O, 64.3
O=C4O[I](C#CC5=CC=CC=C5)C6=CC7=CC=CC=C7C=C64, [C]#CC1=CC=CC=C1,

O=C3O[I]C4=CC5=CC=CC=C5C=C43, 69.2
O=S(C8=C9C=CC=C8)(O[I]9C#CC%10=CC=CC=C%10)=O, [C]#CC1=CC=CC=C1, O=S(C6=C7C=CC=C6)(O[I]7)=O, 75.2
O=C(O%11)N(C)C%12=C([I]%11C#CC%13=CC=CC=C%13)C=CC=C%12, [C]#CC1=CC=CC=C1, O=C8N(C)C9=C([I]O8)C=CC=C9, 68.7
CC(S[I]%14C#CC%15=CC=CC=C%15)(C)C%16=C%14C=CC=C%16, [C]#CC1=CC=CC=C1, CC(S[I]%10)(C)C%11=C%10C=CC=C%11, 48.8
FC(C(O[I]%17C#CC%18=CC=CC=C%18)(C(F)(F)F)C%19=C%17C=CC=C%19)(F)F, [C]#CC1=CC=CC=C1, FC(C(O[I]%12)(C(F)(F)F)C%13=C%12C=CC=C%13)(F)F, 70.5
O=C(N(C(C)=O)[I]%20C#CC%21=CC=CC=C%21)C%22=C%20C=CC=C%22, [C]#CC1=CC=CC=C1, O=C(N(C(C)=O)[I]%14)C%15=C%14C=CC=C%15, 65.2
O=C(O[I]%23C#CC%24=CC=CC=C%24)C%25=C%23C(C)=CC=C%25, [C]#CC1=CC=CC=C1, O=C(O[I]%16)C%17=C%16C(C)=CC=C%17, 62.0
O=C(O[I]%26C#CC%27=CC=CC=C%27)C%28=C%26C=CO%28, [C]#CC1=CC=CC=C1, O=C(O[I]%18)C%19=C%18C=CO%19, 63.6
O=C(O[I]1CC2=CC=CC=C2)C3=C1C=CN3C(C)=O, [C]C1=CC=CC=C1, O=C(O[I]1)C2=C1C=CN2C(C)=O, 22.1
O=C4O[I](CC5=CC=CC=C5)C6=CC7=CC=CC=C7C=C64, [C]C1=CC=CC=C1, O=C3O[I]C4=CC5=CC=CC=C5C=C43, 25.3
O=S(C8=C9C=CC=C8)(O[I]9CC%10=CC=CC=C%10)=O, [C]C1=CC=CC=C1, O=S(C6=C7C=CC=C6)(O[I]7)=O, 33.9
O=C(O%11)N(C)C%12=C([I]%11CC%13=CC=CC=C%13)C=CC=C%12, [C]C1=CC=CC=C1, O=C8N(C)C9=C([I]O8)C=CC=C9, 26.0
CC(S[I]%14CC%15=CC=CC=C%15)(C)C%16=C%14C=CC=C%16, [C]C1=CC=CC=C1, CC(S[I]%10)(C)C%11=C%10C=CC=C%11, 4.0
FC(C(O[I]%17CC%18=CC=CC=C%18)(C(F)(F)F)C%19=C%17C=CC=C%19)(F)F, [C]C1=CC=CC=C1, FC(C(O[I]%12)(C(F)(F)F)C%13=C%12C=CC=C%13)(F)F, 26.0
O=C(N(C(C)=O)[I]%20CC%21=CC=CC=C%21)C%22=C%20C=CC=C%22, [C]C1=CC=CC=C1, O=C(N(C(C)=O)[I]%14)C%15=C%14C=CC=C%15, 21.1
O=C(O[I]%23CC%24=CC=CC=C%24)C%25=C%23C(C)=CC=C%25, [C]C1=CC=CC=C1, O=C(O[I]%16)C%17=C%16C(C)=CC=C%17, 19.1
O=C(O[I]%26CC%27=CC=CC=C%27)C%28=C%26C=CO%28, [C]C1=CC=CC=C1, O=C(O[I]%18)C%19=C%18C=CO%19, 21.8
O=C(O[I]1N2C(C=CC=C3)=C3C4=C2C=CC=C4)C5=C1C=CN5C(C)=O, C12=C(C=CC=C2)C3=C(C=CC=C3)[N]1, O=C(O[I]1)C2=C1C=CN2C(C)=O, 20.5
O=C6O[I](N7C(C=CC=C8)=C8C9=C7C=CC=C9)C%10=CC%11=CC=CC=C%11C=C%106, C12=C(C=CC=C2)C3=C(C=CC=C3)[N]1, O=C3O[I]C4=CC5=CC=CC=C5C=C43, 25.7
O=S(C%12=C%13C=CC=C%12)(O[I]%13N%14C(C=CC=C%15)=C%15C%16=C%14C=CC=C%16)=O, C12=C(C=CC=C2)C3=C(C=CC=C3)[N]1, O=S(C6=C7C=CC=C6)(O[I]7)=O, 29.6
O=C(O%17)N(C)C%18=C([I]%17N%19C(C=CC=C%20)=C%20C%21=C%19C=CC=C%21)C=CC=C%18, C12=C(C=CC=C2)C3=C(C=CC=C3)[N]1, O=C8N(C)C9=C([I]O8)C=CC=C9, 25.2
CC(S[I]%22N%23C(C=CC=C%24)=C%24C%25=C%23C=CC=C%25)(C)C%26=C%22C=CC=C%26, C12=C(C=CC=C2)C3=C(C=CC=C3)[N]1, CC(S[I]%10)(C)C%11=C%10C=CC=C%11, 10.5
FC(C(O[I]%27N%28C(C=CC=C%29)=C%29C%30=C%28C=CC=C%30)(C(F)(F)F)C%31=C%27C=CC=C%31)(F)F, C12=C(C=CC=C2)C3=C(C=CC=C3)[N]1, FC(C(O[I]%12)(C(F)(F)F)C%13=C%12C=CC=C%13)(F)F, 27.9
O=C(N(C(C)=O)[I]%32N%33C(C=CC=C%34)=C%34C%35=C%33C=CC=C%35)C%36=C%32C=CC=C%36, C12=C(C=CC=C2)C3=C(C=CC=C3)[N]1, O=C(N(C(C)=O)[I]%14)C%15=C%14C=CC=C%15, 23.0
O=C(O[I]%37N%38C(C=CC=C%39)=C%39C%40=C%38C=CC=C%40)C%41=C%37C(C)=CC=C%41, C12=C(C=CC=C2)C3=C(C=CC=C3)[N]1, O=C(O[I]%16)C%17=C%16C(C)=CC=C%17, 20.1

O=C(O[I]%42N%43C(C=CC=C%44)=C%44C%45=C%43C=CC=C%45)C%46=C%42C=CO%46, C12=C(C=CC=C2)C3=C(C=CC=C3)[N]1, O=C(O[I]%18)C%19=C%18C=CO%19, 19.1

(5) Fig4_AD_D_169HVIs.txt

CC(O[I]1OCC)(C)C2=C1C=CN2C, [O]CC, CC(O[I]1)(C)C2=C1C=CN2C, 27.2
CC3(C)O[I](OCC)C4=CC=CN=C43, [O]CC, CC3(C)O[I]C4=CC=CN=C43, 28.6
FC5=C(F)C(F)=C(F)C(N(C)C(O6)=O)=C5[I]6OCC, [O]CC, FC5=C(F)C(F)=C(F)C(N6C)=C5[I]OC6=O, 27.0
FC7=C(F)C(F)=C(F)C8=C7[I](OCC)OC8(C)C, [O]CC, FC7=C(F)C(F)=C(F)C8=C7[I]OC8(C)C, 26.6
CN([I]9OCC)S(C%10=C(C9=CC=C%11)C%11=CC=C%10)(=O)=O, [O]CC, CN([I]9)S(C%10=C(C9=CC=C%11)C%11=CC=C%10)(=O)=O, 25.6
CC%12(C)O[I](OCC)C%13=CC%14=CC=CC=C%14C=C%13%12, [O]CC, CC%12(C)O[I]C%13=CC%14=CC=CC=C%14C=C%13%12, 30.8
O=C(N%15C)N(C)C%16=C([I]%15OCC)C=C(C=CC=C%17)C%17=C%16, [O]CC, O=C%15N(C)C%16=C([I]N%15C)C=C(C=CC=C%17)C%17=C%16, 19.8
O=C(N(C(C)=O)[I]%18OCC)C%19=C%18C%20=CC=CC=C%20N%19C, [O]CC, O=C(N(C(C)=O)[I]%18)C%19=C%18C%20=CC=CC=C%20N%19C, 30.8
CC(S[I]%21OCC)(C)C%22=C%21C=CO%22, [O]CC, CC(S[I]%21)(C)C%22=C%21C=CO%22, 15.2
O=C%23CCCC%24=CC%25=CC%26=CC=CC=C%26C=C%25C=C%24[I](OCC)O%23, [O]CC, O=C%23CCCC%24=CC%25=CC%26=CC=CC=C%26C=C%25C=C%24[I]O%23, 21.1
CC(O[I]1OCC(F)(F)F)(C)C2=C1C=CN2C, [O]CC(F)(F)F, CC(O[I]1)(C)C2=C1C=CN2C, 36.2
CC3(C)O[I](OCC(F)(F)F)C4=CC=CN=C43, [O]CC(F)(F)F, CC3(C)O[I]C4=CC=CN=C43, 37.3
FC5=C(F)C(F)=C(F)C(N(C)C(O6)=O)=C5[I]6OCC(F)(F)F, [O]CC(F)(F)F, FC5=C(F)C(F)=C(F)C(N6C)=C5[I]OC6=O, 35.0
FC7=C(F)C(F)=C(F)C8=C7[I](OCC(F)(F)F)OC8(C)C, [O]CC(F)(F)F, FC7=C(F)C(F)=C(F)C8=C7[I]OC8(C)C, 33.4
CN([I]9OCC(F)(F)F)S(C%10=C(C9=CC=C%11)C%11=CC=C%10)(=O)=O, [O]CC(F)(F)F, CN([I]9)S(C%10=C(C9=CC=C%11)C%11=CC=C%10)(=O)=O, 31.2
CC%12(C)O[I](OCC(F)(F)F)C%13=CC%14=CC=CC=C%14C=C%13%12, [O]CC(F)(F)F, CC%12(C)O[I]C%13=CC%14=CC=CC=C%14C=C%13%12, 39.8
O=C(N%15C)N(C)C%16=C([I]%15OCC(F)(F)F)C=C(C=CC=C%17)C%17=C%16, [O]CC(F)(F)F, O=C%15N(C)C%16=C([I]N%15C)C=C(C=CC=C%17)C%17=C%16, 29.1
O=C(N(C(C)=O)[I]%18OCC(F)(F)F)C%19=C%18C%20=CC=CC=C%20N%19C, [O]CC(F)(F)F, O=C(N(C(C)=O)[I]%18)C%19=C%18C%20=CC=CC=C%20N%19C, 38.0
CC(S[I]%21OCC(F)(F)F)(C)C%22=C%21C=CO%22, [O]CC(F)(F)F, CC(S[I]%21)(C)C%22=C%21C=CO%22, 24.3
O=C%23CCCC%24=CC%25=CC%26=CC=CC=C%26C=C%25C=C%24[I](OCC(F)(F)F)O%23, [O]CC(F)(F)F, O=C%23CCCC%24=CC%25=CC%26=CC=CC=C%26C=C%25C=C%24[I]O%23, 29.2
CC(O[I]1C2=CC(C=CC=C3)=C3C=C2)(C)C4=C1C=CN4C, C12=CC=[C]C=C1C=CC=C2, CC(O[I]1)(C)C2=C1C=CN2C, 35.3
CC5(C)O[I](C6=CC=C(C=CC=C7)C7=C6)C8=CC=CN=C85, C12=CC=[C]C=C1C=CC=C2, CC3(C)O[I]C4=CC=CN=C43, 34.3
FC9=C(F)C(F)=C(F)C(N(C)C(O%10)=O)=C9[I]%10C%11=CC(C=CC=C%12)=C%12C=C%11, C12=CC=[C]C=C1C=CC=C2, FC5=C(F)C(F)=C(F)C(N6C)=C5[I]OC6=O, 41.5
FC%13=C(F)C(F)=C(F)C%14=C%13[I](C%15=CC(C=CC=C%16)=C%16C=C%15)OC%14(C)C, C12=CC=[C]C=C1C=CC=C2, FC7=C(F)C(F)=C(F)C8=C7[I]OC8(C)C, 33.5
CN([I]%17C%18=CC(C=CC=C%19)=C%19C=C%18)S(C%20=C(C%17=CC=C%21)C%21=CC=C%20)(=O)=O, C12=CC=[C]C=C1C=CC=C2, CN([I]9)S(C%10=C(C9=CC=C%11)C%11=CC=C%10)(=O)=O, 31.3
CC%22(C)O[I](C%23=CC=C(C=CC=C%24)C%24=C%23)C%25=CC%26=CC=CC=C%26C=C%25%22, C12=CC=[C]C=C1C=CC=C2, CC%12(C)O[I]C%13=CC%14=CC=CC=C%14C=C%13%12, 36.9

O=C(N%27C)N(C)C%28=C([I]%27C%29=CC=C(C=CC=C%30)C%30=C%29)C=C(C=CC=C%31)C%31=C%28, C12=CC=[C]C=C1C=CC=C2, O=C%15N(C)C%16=C([I]N%15C)C=C(C=CC=C%17)C%17=C%16, 27.3
O=C(N(C(C)=O)[I]%32C%33=CC=C(C=CC=C%34)C%34=C%33)C%35=C%32C%36=CC=CC=C%36N%35C, C12=CC=[C]C=C1C=CC=C2, O=C(N(C(C)=O)[I]%18)C%19=C%18C%20=CC=CC=C%20N%19C, 40.5
CC(S[I]%37C%38=CC(C=CC=C%39)=C%39C=C%38)(C)C%40=C%37C=CO%40, C12=CC=[C]C=C1C=CC=C2, CC(S[I]%21)(C)C%22=C%21C=CO%22, 21.8
O=C%41CCCC%42=CC%43=CC%44=CC=CC=C%44C=C%43C=C%42[I](C%45=CC=C(C=CC=C%46)C%46=C%45)O%41, C12=CC=[C]C=C1C=CC=C2, O=C%23CCCC%24=CC%25=CC%26=CC=CC=C%26C=C%25C=C%24[I]O%23, 30.3
CC(O[I]1C#CC2=CC=CC=C2)(C)C3=C1C=CN3C, [C]#CC1=CC=CC=C1, CC(O[I]1)(C)C2=C1C=CN2C, 58.2
CC4(C)O[I](C#CC5=CC=CC=C5)C6=CC=CN=C64, [C]#CC1=CC=CC=C1, CC3(C)O[I]C4=CC=CN=C43, 58.5
FC7=C(F)C(F)=C(F)C(N(C)C(O8)=O)=C7[I]8C#CC9=CC=CC=C9, [C]#CC1=CC=CC=C1, FC5=C(F)C(F)=C(F)C(N6C)=C5[I]OC6=O, 60.5
FC%10=C(F)C(F)=C(F)C%11=C%10[I](C#CC%12=CC=CC=C%12)OC%11(C)C, [C]#CC1=CC=CC=C1, FC7=C(F)C(F)=C(F)C8=C7[I]OC8(C)C, 55.4
CN([I]%13C#CC%14=CC=CC=C%14)S(C%15=C(C%13=CC=C%16)C%16=CC=C%15)(=O)=O, [C]#CC1=CC=CC=C1, CN([I]9)S(C%10=C(C9=CC=C%11)C%11=CC=C%10)(=O)=O, 53.5
CC%17(C)O[I](C#CC%18=CC=CC=C%18)C%19=CC%20=CC=CC=C%20C=C%19%17, [C]#CC1=CC=CC=C1, CC%12(C)O[I]C%13=CC%14=CC=CC=C%14C=C%13%12, 60.8
O=C(N%21C)N(C)C%22=C([I]%21C#CC%23=CC=CC=C%23)C=C(C=CC=C%24)C%24=C%22, [C]#CC1=CC=CC=C1, O=C%15N(C)C%16=C([I]N%15C)C=C(C=CC=C%17)C%17=C%16, 50.1
O=C(N(C(C)=O)[I]%25C#CC%26=CC=CC=C%26)C%27=C%25C%28=CC=CC=C%28N%27C, [C]#CC1=CC=CC=C1, O=C(N(C(C)=O)[I]%18)C%19=C%18C%20=CC=CC=C%20N%19C, 60.7
CC(S[I]%29C#CC%30=CC=CC=C%30)(C)C%31=C%29C=CO%31, [C]#CC1=CC=CC=C1, CC(S[I]%21)(C)C%22=C%21C=CO%22, 44.0
O=C%32CCCC%33=CC%34=CC%35=CC=CC=C%35C=C%34C=C%33[I](C#CC%36=CC=CC=C%36)O%32, [C]#CC1=CC=CC=C1, O=C%23CCCC%24=CC%25=CC%26=CC=CC=C%26C=C%25C=C%24[I]O%23, 51.3
CC(O[I]1N2C(C=CC=C3)=C3C4=C2C=CC=C4)(C)C5=C1C=CN5C, C1(C=CC=C2)=C2C(C=CC=C3)=C3[N]1, CC(O[I]1)(C)C2=C1C=CN2C, 17.4
CC6(C)O[I](N7C8=C(C=CC=C8)C9=C7C=CC=C9)C%10=CC=CN=C%106, C1(C=CC=C2)=C2C(C=CC=C3)=C3[N]1, CC3(C)O[I]C4=CC=CN=C43, 18.5
FC%11=C(F)C(F)=C(F)C(N(C)C(O%12)=O)=C%11[I]%12N%13C(C=CC=C%14)=C%14C%15=C%13C=CC=C%15, C1(C=CC=C2)=C2C(C=CC=C3)=C3[N]1, FC5=C(F)C(F)=C(F)C(N6C)=C5[I]OC6=O, 16.3
FC%16=C(F)C(F)=C(F)C%17=C%16[I](N%18C(C=CC=C%19)=C%19C%20=C%18C=CC=C%20)OC%17(C)C, C1(C=CC=C2)=C2C(C=CC=C3)=C3[N]1, FC7=C(F)C(F)=C(F)C8=C7[I]OC8(C)C, 15.0
CN([I]%21N%22C(C=CC=C%23)=C%23C%24=C%22C=CC=C%24)S(C%25=C(C%21=CC=C%26)C%26=CC=C%25)(=O)=O, C1(C=CC=C2)=C2C(C=CC=C3)=C3[N]1, CN([I]9)S(C%10=C(C9=CC=C%11)C%11=CC=C%10)(=O)=O, 13.6
CC%27(C)O[I](N%28C%29=C(C=CC=C%29)C%30=C%28C=CC=C%30)C%31=CC%32=CC=CC=C%32C=C%31%27, C1(C=CC=C2)=C2C(C=CC=C3)=C3[N]1, CC%12(C)O[I]C%13=CC%14=CC=CC=C%14C=C%13%12, 20.8
O=C(N%33C)N(C)C%34=C([I]%33N%35C(C=CC=C%36)=C%36C%37=C%35C=CC=C%37)C=C(C=CC=C%38)C%38=C%34, C1(C=CC=C2)=C2C(C=CC=C3)=C3[N]1, O=C%15N(C)C%16=C([I]N%15C)C=C(C=CC=C%17)C%17=C%16, 10.7
O=C(N(C(C)=O)[I]%39N%40C%41=C(C=CC=C%41)C%42=C%40C=CC=C%42)C%43=C%39C%44=

CC=CC=C%44N%43C, C1(C=CC=C2)=C2C(C=CC=C3)=C3[N]1, O=C(N(C(C)=O)[I]%18)C%19=C%18C%20=CC=CC=C%20N%19C, 18.8
CC(S[I]%45N%46C(C=CC=C%47)=C%47C%48=C%46C=CC=C%48)(C)C%49=C%45C=CO%49, C1(C=CC=C2)=C2C(C=CC=C3)=C3[N]1, CC(S[I]%21)(C)C%22=C%21C=CO%22, 4.7
O=C%50CCCC%51=CC%52=CC%53=CC=CC=C%53C=C%52C=C%51[I](N%54C(C=CC=C%55)=C%55C%56=C%54C=CC=C%56)O%50, C1(C=CC=C2)=C2C(C=CC=C3)=C3[N]1, O=C%23CCCC%24=CC%25=CC%26=CC=CC=C%26C=C%25C=C%24[I]O%23, 9.5
CC(O[I]1CC2=CC=CC=C2)(C)C3=C1C=CN3C, [C]C1=CC=CC=C1, CC(O[I]1)(C)C2=C1C=CN2C, 13.7
CC4(C)O[I](CC5=CC=CC=C5)C6=CC=CN=C64, [C]C1=CC=CC=C1, CC3(C)O[I]C4=CC=CN=C43, 12.2
FC7=C(F)C(F)=C(F)C(N(C)C(O8)=O)=C7[I]8CC9=CC=CC=C9, [C]C1=CC=CC=C1, FC5=C(F)C(F)=C(F)C(N6C)=C5[I]OC6=O, 20.7
FC%10=C(F)C(F)=C(F)C%11=C%10[I](CC%12=CC=CC=C%12)OC%11(C)C, [C]C1=CC=CC=C1, FC7=C(F)C(F)=C(F)C8=C7[I]OC8(C)C, 14.0
CN([I]%13CC%14=CC=CC=C%14)S(C%15=C(C%13=CC=C%16)C%16=CC=C%15)(=O)=O, [C]C1=CC=CC=C1, CN([I]9)S(C%10=C(C9=CC=C%11)C%11=CC=C%10)(=O)=O, 9.8
CC%17(C)O[I](CC%18=CC=CC=C%18)C%19=CC%20=CC=CC=C%20C=C%19%17, [C]C1=CC=CC=C1, CC%12(C)O[I]C%13=CC%14=CC=CC=C%14C=C%13%12, 14.6
O=C(N%21C)N(C)C%22=C([I]%21CC%23=CC=CC=C%23)C=C(C=CC=C%24)C%24=C%22, [C]C1=CC=CC=C1, O=C%15N(C)C%16=C([I]N%15C)C=C(C=CC=C%17)C%17=C%16, 5.7
O=C(N(C(C)=O)[I]%25CC%26=CC=CC=C%26)C%27=C%25C%28=CC=CC=C%28N%27C, [C]C1=CC=CC=C1, O=C(N(C(C)=O)[I]%18)C%19=C%18C%20=CC=CC=C%20N%19C, 18.6
CC(S[I]%29CC%30=CC=CC=C%30)(C)C%31=C%29C=CO%31, [C]C1=CC=CC=C1, CC(S[I]%21)(C)C%22=C%21C=CO%22, 1.2
O=C%32CCCC%33=CC%34=CC%35=CC=CC=C%35C=C%34C=C%33[I](CC%36=CC=CC=C%36)O%32, [C]C1=CC=CC=C1, O=C%23CCCC%24=CC%25=CC%26=CC=CC=C%26C=C%25C=C%24[I]O%23, 8.0
F[I](OCC)C(C=CC=C1)=C1C2=CC=CC=N2, [O]CC, F[I]C(C=CC=C1)=C1C2=CC=CC=N2, 39.5
F[I](OCC(F)(F)F)C(C=CC=C3)=C3C4=CC=CC=N4, [O]CC(F)(F)F, F[I]C(C=CC=C1)=C1C2=CC=CC=N2, 51.7
F[I](C5=CC=C(C=CC=C6)C6=C5)C(C=CC=C7)=C7C8=CC=CC=N8, C12=CC=[C]C=C1C=CC=C2, F[I]C(C=CC=C1)=C1C2=CC=CC=N2, 53.0
F[I](C#CC9=CC=CC=C9)C(C=CC=C%10)=C%10C%11=CC=CC=N%11, [C]#CC1=CC=CC=C1, F[I]C(C=CC=C1)=C1C2=CC=CC=N2, 73.8
F[I](N%12C%13=CC=CC=C%13C%14=C%12C=CC=C%14)C(C=CC=C%15)=C%15C%16=CC=CC=N%16, C1(C=CC=C2)=C2C(C=CC=C3)=C3[N]1, F[I]C(C=CC=C1)=C1C2=CC=CC=N2, 33.8
F[I](CC%17=CC=CC=C%17)C(C=CC=C%18)=C%18C%19=CC=CC=N%19, [C]C1=CC=CC=C1, F[I]C(C=CC=C1)=C1C2=CC=CC=N2, 29.9
F[I](OCC)C(C=C1)=CN1C, [O]CC, F[I]C(C=C1)=CN1C, 46.0
F[I](OCC(F)(F)F)C(C=C2)=CN2C, [O]CC(F)(F)F, F[I]C(C=C1)=CN1C, 54.6
F[I](C3=CC=C(C=CC=C4)C4=C3)C(C=C5)=CN5C, C12=CC=[C]C=C1C=CC=C2, F[I]C(C=C1)=CN1C, 54.6
F[I](C#CC6=CC=CC=C6)C(C=C7)=CN7C, [C]#CC1=CC=CC=C1, F[I]C(C=C1)=CN1C, 77.6
F[I](N8C9=CC=CC=C9C%10=C8C=CC=C%10)C(C=C%11)=CN%11C, C1(C=CC=C2)=C2C(C=CC=C3)=C3[N]1, F[I]C(C=C1)=CN1C, 36.6
F[I](CC%12=CC=CC=C%12)C(C=C%13)=CN%13C, [C]C1=CC=CC=C1, F[I]C(C=C1)=CN1C, 31.8
F[I](OCC)C1=C2C=CC=CC2=CC3=C1C=CC=C3, [O]CC, F[I]C1=C2C=CC=CC2=CC3=C1C=CC=C3, 43.6
F[I](OCC(F)(F)F)C4=C5C=CC=CC5=CC6=C4C=CC=C6, [O]CC(F)(F)F, F[I]C1=C2C=CC=CC2=CC3=C1C=CC=C3, 51.7

F[I](C7=CC=C(C=CC=C8)C8=C7)C9=C%10C(C=CC=C%10)=CC%11=C9C=CC=C%11, C12=CC=[C]C=C1C=CC=C2, F[I]C1=C2C=CC=CC2=CC3=C1C=CC=C3, 52.7
F[I](C#CC%12=CC=CC=C%12)C%13=C%14C=CC=CC%14=CC%15=C%13C=CC=C%15, [C]#CC1=CC=CC=C1, F[I]C1=C2C=CC=CC2=CC3=C1C=CC=C3, 74.8
F[I](N%16C%17=CC=CC=C%17C%18=C%16C=CC=C%18)C%19=C%20C=CC=CC%20=CC%21=C%19C=CC=C%21, C1(C=CC=C2)=C2C(C=CC=C3)=C3[N]1, F[I]C1=C2C=CC=CC2=CC3=C1C=CC=C3, 34.0
F[I](CC%22=CC=CC=C%22)C%23=C%24C=CC=CC%24=CC%25=C%23C=CC=C%25, [C]C1=CC=CC=C1, F[I]C1=C2C=CC=CC2=CC3=C1C=CC=C3, 30.8
CC(O[I]1C2=COC=C2)(C)C3=C1C=CN3C, C1=[C]C=CO1, CC(O[I]1)(C)C2=C1C=CN2C, 42.2
CC1(C)O[I](C2=COC=C2)C3=CC=CN=C31, C1=[C]C=CO1, CC3(C)O[I]C4=CC=CN=C43, 41.4
F[I](C1=COC=C1)C(C=CC=C2)=C2C3=CC=CC=N3, C1=[C]C=CO1, F[I]C(C=CC=C1)=C1C2=CC=CC=N2, 58.5
FC1=C(F)C(F)=C(F)C(N2C)=C1[I](C3=COC=C3)OC2=O, C1=[C]C=CO1, FC5=C(F)C(F)=C(F)C(N6C)=C5[I]OC6=O, 47.4
FC4=C(F)C(F)=C(F)C5=C4[I](C6=COC=C6)OC5(C)C, C1=[C]C=CO1, FC7=C(F)C(F)=C(F)C8=C7[I]OC8(C)C, 40.1
CN([I]1C2=COC=C2)S(C3=C(C1=CC=C4)C4=CC=C3)(=O)=O, C1=[C]C=CO1, CN([I]9)S(C%10=C(C9=CC=C%11)C%11=CC=C%10)(=O)=O, 37.6
CC1(C)O[I](C2=COC=C2)C3=CC4=CC=CC=C4C=C31, C1=[C]C=CO1, CC%12(C)O[I]C%13=CC%14=CC=CC=C%14C=C%13%12, 43.9
O=C5N(C)C6=C([I](C7=COC=C7)N5C)C=C(C=CC=C8)C8=C6, C1=[C]C=CO1, O=C%15N(C)C%16=C([I]N%15C)C=C(C=CC=C%17)C%17=C%16, 33.8
F[I](C9=COC=C9)C%10=CN(C)C=C%10, C1=[C]C=CO1, F[I]C1=CN(C)C=C1, 61.4
O=C(N(C(C)=O)[I]1C2=COC=C2)C3=C1C4=CC=CC=C4N3C, C1=[C]C=CO1, O=C(N(C(C)=O)[I]%18)C%19=C%18C%20=CC=CC=C%20N%19C, 46.6
CC(S[I]5C6=COC=C6)(C)C7=C5C=CO7, C1=[C]C=CO1, CC(S[I]%21)(C)C%22=C%21C=CO%22, 28.3
O=C8CCCC9=CC%10=CC%11=CC=CC=C%11C=C%10C=C9[I](C%12=COC=C%12)O8, C1=[C]C=CO1, O=C%23CCCC%24=CC%25=CC%26=CC=CC=C%26C=C%25C=C%24[I]O%23, 36.3
F[I](C1=COC=C1)C2=C(C=CC=C3)C3=CC4=CC=CC=C42, C1=[C]C=CO1, F[I]C1=C(C=CC=C2)C2=CC3=CC=CC=C31, 58.3
CC(O[I]1SC(C)(C)C)(C)C2=C1C=CN2C, [S]C(C)(C)C, CC(O[I]1)(C)C2=C1C=CN2C, 24.8
CC1(C)O[I](SC(C)(C)C)C2=CC=CN=C21, [S]C(C)(C)C, CC3(C)O[I]C4=CC=CN=C43, 25.0
F[I](SC(C)(C)C)C(C=CC=C1)=C1C2=CC=CC=N2, [S]C(C)(C)C, F[I]C(C=CC=C1)=C1C2=CC=CC=N2, 37.5
FC1=C(F)C(F)=C(F)C(N(C)C(O2)=O)=C1[I]2SC(C)(C)C, [S]C(C)(C)C, FC5=C(F)C(F)=C(F)C(N6C)=C5[I]OC6=O, 27.6
FC1=C(F)C(F)=C(F)C2=C1[I](SC(C)(C)C)OC2(C)C, [S]C(C)(C)C, FC7=C(F)C(F)=C(F)C8=C7[I]OC8(C)C, 21.8
CN([I]1SC(C)(C)C)S(C2=C(C1=CC=C3)C3=CC=C2)(=O)=O, [S]C(C)(C)C, CN([I]9)S(C%10=C(C9=CC=C%11)C%11=CC=C%10)(=O)=O, 19.3
CC1(C)O[I](SC(C)(C)C)C2=CC3=CC=CC=C3C=C21, [S]C(C)(C)C, CC%12(C)O[I]C%13=CC%14=CC=CC=C%14C=C%13%12, 27.2
O=C(N1C)N(C)C2=C([I]1SC(C)(C)C)C=C(C=CC=C3)C3=C2, [S]C(C)(C)C, O=C%15N(C)C%16=C([I]N%15C)C=C(C=CC=C%17)C%17=C%16, 16.6
F[I](C1=CN(C)C=C1)SC(C)(C)C, [S]C(C)(C)C, F[I]C1=CN(C)C=C1, 42.6
O=C(N(C(C)=O)[I]1SC(C)(C)C)C2=C1C3=CC=CC=C3N2C, [S]C(C)(C)C, O=C(N(C(C)=O)[I]%18)C%19=C%18C%20=CC=CC=C%20N%19C, 27.5
CC(S[I]1SC(C)(C)C)(C)C2=C1C=CO2, [S]C(C)(C)C, CC(S[I]%21)(C)C%22=C%21C=CO%22, 13.6
O=C1CCCC2=CC3=CC4=CC=CC=C4C=C3C=C2[I](SC(C)(C)C)O1, [S]C(C)(C)C,

O=C%23CCCC%24=CC%25=CC%26=CC=CC=C%26C=C%25C=C%24[I]O%23, 18.8
F[I](SC(C)(C)C)C1=C(C=CC=C2)C2=CC3=CC=CC=C31, [S]C(C)(C)C, F[I]C1=C(C=CC=C2)C2=CC3=CC=CC=C31, 40.6
CC(O[I]1OS(=O)(C2=CC=CC=C2)=O)(C)C3=C1C=CN3C, [O]S(=O)(C1=CC=CC=C1)=O, CC(O[I]1)(C)C2=C1C=CN2C, 48.0
CC4(C)O[I](OS(=O)(C5=CC=CC=C5)=O)C6=CC=CN=C64, [O]S(=O)(C1=CC=CC=C1)=O, CC3(C)O[I]C4=CC=CN=C43, 50.6
F[I](OS(=O)(C7=CC=CC=C7)=O)C(C=CC=C8)=C8C9=CC=CC=N9, [O]S(=O)(C1=CC=CC=C1)=O, F[I]C(C=CC=C1)=C1C2=CC=CC=N2, 60.4
FC1=C(F)C(F)=C(F)C(N2C)=C1[I](OS(=O)(C3=CC=CC=C3)=O)OC2=O, [O]S(=O)(C1=CC=CC=C1)=O, FC5=C(F)C(F)=C(F)C(N6C)=C5[I]OC6=O, 41.4
FC4=C(F)C(F)=C(F)C5=C4[I](OS(=O)(C6=CC=CC=C6)=O)OC5(C)C, [O]S(=O)(C1=CC=CC=C1)=O, FC7=C(F)C(F)=C(F)C8=C7[I]OC8(C)C, 43.5
CN([I]7OS(=O)(C8=CC=CC=C8)=O)S(C9=C(C7=CC=C%10)C%10=CC=C9)(=O)=O, [O]S(=O)(C1=CC=CC=C1)=O, CN([I]9)S(C%10=C(C9=CC=C%11)C%11=CC=C%10)(=O)=O, 47.1
CC1(C)O[I](OS(=O)(C2=CC=CC=C2)=O)C3=CC4=CC=CC=C4C=C31, [O]S(=O)(C1=CC=CC=C1)=O, CC%12(C)O[I]C%13=CC%14=CC=CC=C%14C=C%13%12, 51.8
O=C5N(C)C6=C([I](OS(=O)(C7=CC=CC=C7)=O)N5C)C=C(C=CC=C8)C8=C6, [O]S(=O)(C1=CC=CC=C1)=O, O=C%15N(C)C%16=C([I]N%15C)C=C(C=CC=C%17)C%17=C%16, 45.7
F[I](OS(=O)(C9=CC=CC=C9)=O)C%10=CN(C)C=C%10, [O]S(=O)(C1=CC=CC=C1)=O, F[I]C1=CN(C)C=C1, 63.7
O=C(N(C(C)=O)[I]1OS(=O)(C2=CC=CC=C2)=O)C3=C1C4=CC=CC=C4N3C, [O]S(=O)(C1=CC=CC=C1)=O, O=C(N(C(C)=O)[I]%18)C%19=C%18C%20=CC=CC=C%20N%19C, 49.2
CC(S[I]5OS(=O)(C6=CC=CC=C6)=O)(C)C7=C5C=CO7, [O]S(=O)(C1=CC=CC=C1)=O, CC(S[I]%21)(C)C%22=C%21C=CO%22, 39.8
O=C8CCCC9=CC%10=CC%11=CC=CC=C%11C=C%10C=C9[I](OS(=O)(C%12=CC=CC=C%12)=O)O8, [O]S(=O)(C1=CC=CC=C1)=O, O=C%23CCCC%24=CC%25=CC%26=CC=CC=C%26C=C%25C=C%24[I]O%23, 44.7
F[I](OS(=O)(C1=CC=CC=C1)=O)C2=C(C=CC=C3)C3=CC4=CC=CC=C42, [O]S(=O)(C1=CC=CC=C1)=O, F[I]C1=C(C=CC=C2)C2=CC3=CC=CC=C31, 62.5
CC(O[I]1C2CCCCC2)(C)C3=C1C=CN3C, C1[C]CCCC1, CC(O[I]1)(C)C2=C1C=CN2C, 23.3
CC4(C)O[I](C5CCCCC5)C6=CC=CN=C64, C1[C]CCCC1, CC3(C)O[I]C4=CC=CN=C43, 20.7
F[I](C7CCCCC7)C(C=CC=C8)=C8C9=CC=CC=N9, C1[C]CCCC1, F[I]C(C=CC=C1)=C1C2=CC=CC=N2, 40.8
FC1=C(F)C(F)=C(F)C(N2C)=C1[I](C3CCCCC3)OC2=O, C1[C]CCCC1, FC5=C(F)C(F)=C(F)C(N6C)=C5[I]OC6=O, 31.4
FC4=C(F)C(F)=C(F)C5=C4[I](C6CCCCC6)OC5(C)C, C1[C]CCCC1, FC7=C(F)C(F)=C(F)C8=C7[I]OC8(C)C, 22.2
CN([I]7C8CCCCC8)S(C9=C(C7=CC=C%10)C%10=CC=C9)(=O)=O, C1[C]CCCC1, CN([I]9)S(C%10=C(C9=CC=C%11)C%11=CC=C%10)(=O)=O, 18.9
CC1(C)O[I](C2CCCCC2)C3=CC4=CC=CC=C4C=C31, C1[C]CCCC1, CC%12(C)O[I]C%13=CC%14=CC=CC=C%14C=C%13%12, 23.0
O=C5N(C)C6=C([I](C7CCCCC7)N5C)C=C(C=CC=C8)C8=C6, C1[C]CCCC1, O=C%15N(C)C%16=C([I]N%15C)C=C(C=CC=C%17)C%17=C%16, 14.3
F[I](C9CCCCC9)C%10=CN(C)C=C%10, C1[C]CCCC1, F[I]C1=CN(C)C=C1, 41.3
O=C(N(C(C)=O)[I]1C2CCCCC2)C3=C1C4=CC=CC=C4N3C, C1[C]CCCC1, O=C(N(C(C)=O)[I]%18)C%19=C%18C%20=CC=CC=C%20N%19C, 29.2
CC(S[I]5C6CCCCC6)(C)C7=C5C=CO7, C1[C]CCCC1, CC(S[I]%21)(C)C%22=C%21C=CO%22, 10.5
O=C8CCCC9=CC%10=CC%11=CC=CC=C%11C=C%10C=C9[I](C%12CCCCC%12)O8,

C1[C]CCCC1, O=C%23CCCC%24=CC%25=CC%26=CC=CC=C%26C=C%25C=C%24[I]O%23, 17.6
F[I](C1CCCCC1)C2=C(C=CC=C3)C3=CC4=CC=CC=C42, C1[C]CCCC1, F[I]C1=C(C=CC=C2)C2=CC3=CC=CC=C31, 41.4
CC(O[I]1C2=C(F)C(F)=C(F)C(F)=C2F)(C)C3=C1C=CN3C, FC1=C(F)C(F)=C(F)C(F)=[C]1, CC(O[I]1)(C)C2=C1C=CN2C, 41.7
CC4(C)O[I](C5=C(F)C(F)=C(F)C(F)=C5F)C6=CC=CN=C64, FC1=C(F)C(F)=C(F)C(F)=[C]1, CC3(C)O[I]C4=CC=CN=C43, 40.8
F[I](C7=C(F)C(F)=C(F)C(F)=C7F)C(C=CC=C8)=C8C9=CC=CC=N9, FC1=C(F)C(F)=C(F)C(F)=[C]1, F[I]C(C=CC=C1)=C1C2=CC=CC=N2, 57.3
FC1=C(F)C(F)=C(F)C(N2C)=C1[I](C3=C(F)C(F)=C(F)C(F)=C3F)OC2=O, FC1=C(F)C(F)=C(F)C(F)=[C]1, FC5=C(F)C(F)=C(F)C(N6C)=C5[I]OC6=O, 43.6
FC4=C(F)C(F)=C(F)C5=C4[I](C6=C(F)C(F)=C(F)C(F)=C6F)OC5(C)C, FC1=C(F)C(F)=C(F)C(F)=[C]1, FC7=C(F)C(F)=C(F)C8=C7[I]OC8(C)C, 39.6
CN([I]7C8=C(F)C(F)=C(F)C(F)=C8F)S(C9=C(C7=CC=C%10)C%10=CC=C9)(=O)=O, FC1=C(F)C(F)=C(F)C(F)=[C]1, CN([I]9)S(C%10=C(C9=CC=C%11)C%11=CC=C%10)(=O)=O, 36.2
CC1(C)O[I](C2=C(F)C(F)=C(F)C(F)=C2F)C3=CC4=CC=CC=C4C=C31, FC1=C(F)C(F)=C(F)C(F)=[C]1, CC%12(C)O[I]C%13=CC%14=CC=CC=C%14C=C%13%12, 43.4
O=C5N(C)C6=C([I](C7=C(F)C(F)=C(F)C(F)=C7F)N5C)C=C(C=CC=C8)C8=C6, FC1=C(F)C(F)=C(F)C(F)=[C]1, O=C%15N(C)C%16=C([I]N%15C)C=C(C=CC=C%17)C%17=C%16, 33.6
F[I](C9=C(F)C(F)=C(F)C(F)=C9F)C%10=CN(C)C=C%10, FC1=C(F)C(F)=C(F)C(F)=[C]1, F[I]C1=CN(C)C=C1, 59.8
O=C(N(C(C)=O)[I]1C2=C(F)C(F)=C(F)C(F)=C2F)C3=C1C4=CC=CC=C4N3C, FC1=C(F)C(F)=C(F)C(F)=[C]1, O=C(N(C(C)=O)[I]%18)C%19=C%18C%20=CC=CC=C%20N%19C, 43.2
CC(S[I]5C6=C(F)C(F)=C(F)C(F)=C6F)(C)C7=C5C=CO7, FC1=C(F)C(F)=C(F)C(F)=[C]1, CC(S[I]%21)(C)C%22=C%21C=CO%22, 27.9
O=C8CCCC9=CC%10=CC%11=CC=CC=C%11C=C%10C=C9[I](C%12=C(F)C(F)=C(F)C(F)=C%12F)O8, FC1=C(F)C(F)=C(F)C(F)=[C]1, O=C%23CCCC%24=CC%25=CC%26=CC=CC=C%26C=C%25C=C%24[I]O%23, 35.0
F[I](C1=C(F)C(F)=C(F)C(F)=C1F)C2=C(C=CC=C3)C3=CC4=CC=CC=C42, FC1=C(F)C(F)=C(F)C(F)=[C]1, F[I]C1=C(C=CC=C2)C2=CC3=CC=CC=C31, 59.5
CC(O[I]1C2=NC=CS2)(C)C3=C1C=CN3C, C1=CN=[C]S1, CC(O[I]1)(C)C2=C1C=CN2C, 34.4
CC4(C)O[I](C5=NC=CS5)C6=CC=CN=C64, C1=CN=[C]S1, CC3(C)O[I]C4=CC=CN=C43, 33.7
F[I](C7=NC=CS7)C(C=CC=C8)=C8C9=CC=CC=N9, C1=CN=[C]S1, F[I]C(C=CC=C1)=C1C2=CC=CC=N2, 49.1
FC1=C(F)C(F)=C(F)C(N2C)=C1[I](C3=NC=CS3)OC2=O, C1=CN=[C]S1, FC5=C(F)C(F)=C(F)C(N6C)=C5[I]OC6=O, 38.0
FC4=C(F)C(F)=C(F)C5=C4[I](C6=NC=CS6)OC5(C)C, C1=CN=[C]S1, FC7=C(F)C(F)=C(F)C8=C7[I]OC8(C)C, 32.9
CN([I]7C8=NC=CS8)S(C9=C(C7=CC=C%10)C%10=CC=C9)(=O)=O, C1=CN=[C]S1, CN([I]9)S(C%10=C(C9=CC=C%11)C%11=CC=C%10)(=O)=O, 28.9
CC1(C)O[I](C2=NC=CS2)C3=CC4=CC=CC=C4C=C31, C1=CN=[C]S1, CC%12(C)O[I]C%13=CC%14=CC=CC=C%14C=C%13%12, 36.0
O=C5N(C)C6=C([I](C7=NC=CS7)N5C)C=C(C=CC=C8)C8=C6, C1=CN=[C]S1, O=C%15N(C)C%16=C([I]N%15C)C=C(C=CC=C%17)C%17=C%16, 25.8
F[I](C9=NC=CS9)C%10=CN(C)C=C%10, C1=CN=[C]S1, F[I]C1=CN(C)C=C1, 53.3
O=C(N(C(C)=O)[I]1C2=NC=CS2)C3=C1C4=CC=CC=C4N3C, C1=CN=[C]S1, O=C(N(C(C)=O)[I]%18)C%19=C%18C%20=CC=CC=C%20N%19C, 38.4
CC(S[I]5C6=NC=CS6)(C)C7=C5C=CO7, C1=CN=[C]S1, CC(S[I]%21)(C)C%22=C%21C=CO%22, 20.7

O=C8CCCC9=CC%10=CC%11=CC=CC=C%11C=C%10C=C9[I](C%12=NC=CS%12)O8, C1=CN=[C]S1, O=C%23CCCC%24=CC%25=CC%26=CC=CC=C%26C=C%25C=C%24[I]O%23, 28.0
F[I](C1=NC=CS1)C2=C(C=CC=C3)C3=CC4=CC=CC=C42, C1=CN=[C]S1, F[I]C1=C(C=CC=C2)C2=CC3=CC=CC=C31, 50.7
CC(O[I]1[Se]C(C)(C)C)(C)C2=C1C=CN2C, [Se]C(C)(C)C, CC(O[I]1)(C)C2=C1C=CN2C, 23.4
CC1(C)O[I]([Se]C(C)(C)C)C2=CC=CN=C21, [Se]C(C)(C)C, CC3(C)O[I]C4=CC=CN=C43, 23.7
F[I]([Se]C(C)(C)C)C(C=CC=C1)=C1C2=CC=CC=N2, [Se]C(C)(C)C, F[I]C(C=CC=C1)=C1C2=CC=CC=N2, 34.3
FC1=C(F)C(F)=C(F)C(N(C)C(O2)=O)=C1[I]2[Se]C(C)(C)C, [Se]C(C)(C)C, FC5=C(F)C(F)=C(F)C(N6C)=C5[I]OC6=O, 24.7
FC1=C(F)C(F)=C(F)C2=C1[I]([Se]C(C)(C)C)OC2(C)C, [Se]C(C)(C)C, FC7=C(F)C(F)=C(F)C8=C7[I]OC8(C)C, 19.5
CN([I]1[Se]C(C)(C)C)S(C2=C(C1=CC=C3)C3=CC=C2)(=O)=O, [Se]C(C)(C)C, CN([I]9)S(C%10=C(C9=CC=C%11)C%11=CC=C%10)(=O)=O, 18.8
CC1(C)O[I]([Se]C(C)(C)C)C2=CC3=CC=CC=C3C=C21, [Se]C(C)(C)C, CC%12(C)O[I]C%13=CC%14=CC=CC=C%14C=C%13%12, 26.0
O=C(N1C)N(C)C2=C([I]1[Se]C(C)(C)C)C=C(C=CC=C3)C3=C2, [Se]C(C)(C)C, O=C%15N(C)C%16=C([I]N%15C)C=C(C=CC=C%17)C%17=C%16, 18.0
F[I](C1=CN(C)C=C1)[Se]C(C)(C)C, [Se]C(C)(C)C, F[I]C1=CN(C)C=C1, 38.9
O=C(N(C(C)=O)[I]1[Se]C(C)(C)C)C2=C1C3=CC=CC=C3N2C, [Se]C(C)(C)C, O=C(N(C(C)=O)[I]%18)C%19=C%18C%20=CC=CC=C%20N%19C, 25.9
CC(S[I]1[Se]C(C)(C)C)(C)C2=C1C=CO2, [Se]C(C)(C)C, CC(S[I]%21)(C)C%22=C%21C=CO%22, 14.2
O=C1CCCC2=CC3=CC4=CC=CC=C4C=C3C=C2[I]([Se]C(C)(C)C)O1, [Se]C(C)(C)C, O=C%23CCCC%24=CC%25=CC%26=CC=CC=C%26C=C%25C=C%24[I]O%23, 16.6
F[I]([Se]C(C)(C)C)C1=C(C=CC=C2)C2=CC3=CC=CC=C31, [Se]C(C)(C)C, F[I]C1=C(C=CC=C2)C2=CC3=CC=CC=C31, 36.7

4. Required time for DFT calculations of HVIs in Fig4.

Displayed time is converted to per core. Please see next section for the geometry of the log file.

IndoleAc_54_CO2I_02.log: 35.8 hours
acridineCO2I_CF3.log: 66.4 hours
AzuleneCO2I_CF3.log: 40.2 hours
Indole_NMe_IBCONAc_A_02_2.log: 270.1 hours
PyrroleNMeIBCMe2O_A_02.log: 29.2 hours
NpthIBCMe2O_C_02.log: 38.8 hours
thiazoleCO2I_02.log: 11.8 hours
perF_NMeCO2_02.log: 42.9 hours
PhenanthrolineCO2I_CF3.log: 70.0 hours
FuranIBCMe2S_A_CF3.log: 13.9 hours
Indole_NMe_IBCONAc_A_CF3.log: 68.0 hours
3Obenzofurane_45_CO2I_CF3.log: 23.6 hours
perF_NMeCO2_CF3.log: 22.6 hours
NpthISO2NMe_D_02.log: 56.5 hours
3Obenzofurane_45_CO2I_02.log: 26.5 hours
triazoleCO2I_02.log: 12.8 hours
pyreneCO2I_02.log: 78.3 hours
DibenzofuranCO2I_02.log: 35.2 hours
AnthI8BA_B_CF3.log: 96.7 hours
IndoleAc_54_CO2I_CF3.log: 53.4 hours
IndeneCO2I_CF3.log: 23.9 hours
perF_CMe2O_02.log: 28.7 hours
CyclohexeneCO2I_02.log: 20.9 hours
BenzothiopheneCO2I_CF3.log: 15.4 hours
IndeneCO2I_02.log: 29.0 hours
IndoleAc_56_CO2I_CF3.log: 27.3 hours
CyclohexeneCO2I_CF3.log: 19.3 hours
NaphIBMeUreaMe_02.log: 100.9 hours
PyrroleNMeIBCMe2O_A_CF3.log: 22.9 hours
perF_CMe2O_CF3.log: 27.8 hours
PyIBCMe2O_D_02.log: 23.9 hours
pyreneCO2I_CF3.log: 91.1 hours
acridineCO2I_02.log: 43.0 hours
AnthI8BA_B_02_2.log: 167.1 hours
PyIBCMe2O_D_CF3.log: 17.1 hours
NaphIBMeUreaMe_CF3.log: 54.0 hours
00_CF3_02.log: 0.1 hours
NpthISO2NMe_D_CF3.log: 45.9 hours
BenzothiopheneCO2I_02.log: 20.2 hours
AzuleneCO2I_02.log: 25.7 hours
DibenzofuranCO2I_CF3.log: 34.7 hours
thiazoleCO2I_CF3.log: 7.2 hours
IndoleAc_56_CO2I_02.log: 21.0 hours
3Oindole_45_CO2I_02.log: 29.8 hours
FuranIBCMe2S_A_02.log: 23.4 hours
PhenanthrolineCO2I_02.log: 53.9 hours
triazoleCO2I_CF3.log: 7.8 hours
NpthIBCMe2O_C_CF3.log: 41.0 hours
BenzoFuraneCO2I_02.log: 20.9 hours
BenzoFuraneCO2I_CF3.log: 22.2 hours
3Oindole_45_CO2I_CF3.log: 35.5 hours
NpthISO2NMe_D_Cl.log: 35.4 hours
3Obenzofurane_45_CO2I_Cl.log: 14.3 hours
triazoleCO2I_Cl.log: 4.5 hours
IndoleAc_54_CO2I_02.log: 35.8 hours
AnthI8BA_B_Cl.log: 72.1 hours
pyreneCO2I_Cl.log: 54.1 hours
Indole_NMe_IBCONAc_A_02_2.log: 270.1 hours
PyrroleNMeIBCMe2O_A_02.log: 29.2 hours
DibenzofuranCO2I_Cl.log: 18.0 hours
perF_CMe2O_Cl.log: 19.2 hours
NpthIBCMe2O_C_02.log: 38.8 hours
thiazoleCO2I_02.log: 11.8 hours
IndoleAc_56_CO2I_Cl_2.log: 17.1 hours
CyclohexeneCO2I_Cl.log: 11.4 hours
perF_NMeCO2_02.log: 42.9 hours
IndeneCO2I_Cl.log: 14.5 hours
NpthISO2NMe_D_02.log: 56.5 hours
3Obenzofurane_45_CO2I_02.log: 26.5 hours
triazoleCO2I_02.log: 12.8 hours
Indole_NMe_IBCONAc_A_Cl.log: 30.5 hours
IndoleAc_54_CO2I_Cl.log: 18.0 hours
pyreneCO2I_02.log: 78.3 hours
PyrroleNMeIBCMe2O_A_Cl.log: 14.9 hours
DibenzofuranCO2I_02.log: 35.2 hours
perF_CMe2O_02.log: 28.7 hours
NpthIBCMe2O_C_Cl.log: 28.9 hours
thiazoleCO2I_Cl.log: 3.8 hours
CyclohexeneCO2I_02.log: 20.9 hours
perF_NMeCO2_Cl.log: 15.0 hours
IndeneCO2I_02.log: 29.0 hours
AzuleneCO2I_Cl.log: 13.6 hours
NaphIBMeUreaMe_02.log: 100.9 hours
PyIBCMe2O_D_02.log: 23.9 hours
acridineCO2I_02.log: 43.0 hours
AnthI8BA_B_02_2.log: 167.1 hours
3Oindole_45_CO2I_Cl.log: 15.5 hours
FuranIBCMe2S_A_Cl.log: 10.8 hours
00_Cl_02.log: 0.0 hours
PhenanthrolineCO2I_Cl.log: 30.8 hours
BenzoFuraneCO2I_Cl.log: 11.1 hours
BenzothiopheneCO2I_02.log: 20.2 hours
AzuleneCO2I_02.log: 25.7 hours
NaphIBMeUreaMe_Cl.log: 43.2 hours
PyIBCMe2O_D_Cl.log: 11.4 hours
IndoleAc_56_CO2I_02.log: 21.0 hours
acridineCO2I_Cl.log: 37.7 hours
3Oindole_45_CO2I_02.log: 29.8 hours
FuranIBCMe2S_A_02.log: 23.4 hours
PhenanthrolineCO2I_02.log: 53.9 hours
BenzoFuraneCO2I_02.log: 20.9 hours
BenzothiopheneCO2I_Cl.log: 9.9 hours
IBA_vinyl_02.log: 0.2 hours
IBA_NTf2_02.log: 23.8 hours
IBA_Ph_02.log: 3.0 hours
IBA_CF2CF3_02.log: 0.7 hours
F_02.log: 0.0 hours
IBA_OCF3_02.log: 0.3 hours
IBA_Me_02.log: 0.1 hours
IBA_OMe_02.log: 0.1 hours
IBA_OTf_02.log: 3.3 hours
perF_NMeCO2_CF2CF3.log: 36.4 hours
PyIBCMe2O_D_CF2CF3.log: 25.1 hours
NaphIBMeUreaMe_CF2CF3.log: 69.3 hours

Indole_NMe_IBCONAc_A_02_2.log: 270.1 hours
PyrroleNMeIBCMe2O_A_02.log: 29.2 hours
NpthIBCMe2O_C_02.log: 38.8 hours
perF_NMeCO2_02.log: 42.9 hours
NpthISO2NMe_D_02.log: 56.5 hours
NpthIBCMe2O_C_CF2CF3.log: 54.1 hours
perF_CMe2O_02.log: 28.7 hours
FuranIBCMe2S_A_CF2CF3.log: 21.9 hours
perF_CMe2O_CF2CF3.log: 39.1 hours
PyrroleNMeIBCMe2O_A_CF2CF3.log: 32.9 hours
NaphIBMeUreaMe_02.log: 100.9 hours
PyIBCMe2O_D_02.log: 23.9 hours
NpthISO2NMe_D_CF2CF3.log: 64.3 hours
AnthI8BA_B_CF2CF3.log: 131.0 hours
AnthI8BA_B_02_2.log: 167.1 hours
Indole_NMe_IBCONAc_A_CF2CF3.log: 189.4 hours
FuranIBCMe2S_A_02.log: 23.4 hours
PyIBCMe2O_D_NTf2.log: 74.4 hours
Indole_NMe_IBCONAc_A_02_2.log: 270.1 hours
PyrroleNMeIBCMe2O_A_02.log: 29.2 hours
NpthIBCMe2O_C_02.log: 38.8 hours
perF_NMeCO2_02.log: 42.9 hours
NpthISO2NMe_D_02.log: 56.5 hours
AnthI8BA_B_NTf2.log: 268.0 hours
perF_CMe2O_02.log: 28.7 hours
NaphIBMeUreaMe_NTf2.log: 159.2 hours
perF_CMe2O_NTf2.log: 96.6 hours
NaphIBMeUreaMe_02.log: 100.9 hours
PyIBCMe2O_D_02.log: 23.9 hours
NpthIBCMe2O_C_NTf2.log: 126.3 hours
perF_NMeCO2_NTf2.log: 100.1 hours
AnthI8BA_B_02_2.log: 167.1 hours
FuranIBCMe2S_A_NTf2.log: 69.7 hours
PyrroleNMeIBCMe2O_A_NTf2.log: 92.3 hours
NpthISO2NMe_D_NTf2.log: 153.7 hours
FuranIBCMe2S_A_02.log: 23.4 hours
Indole_NMe_IBCONAc_A_NTf2.log: 172.9 hours
perF_NMeCO2_F.log: 35.0 hours
Indole_NMe_IBCONAc_A_02_2.log: 270.1 hours
PyrroleNMeIBCMe2O_A_02.log: 29.2 hours
NpthIBCMe2O_C_02.log: 38.8 hours
perF_NMeCO2_02.log: 42.9 hours
PyrroleNMeIBCMe2O_A_F.log: 33.6 hours
perF_CMe2O_F.log: 30.6 hours
NpthISO2NMe_D_02.log: 56.5 hours
PyIBCMe2O_D_F.log: 25.2 hours
perF_CMe2O_02.log: 28.7 hours
Indole_NMe_IBCONAc_A_F.log: 50.9 hours
NpthIBCMe2O_C_F.log: 53.6 hours
AnthI8BA_B_F.log: 80.0 hours
PyIBCMe2O_D_02.log: 23.9 hours
AnthI8BA_B_02_2.log: 167.1 hours
NpthISO2NMe_D_F.log: 51.3 hours
FuranIBCMe2S_A_F.log: 14.8 hours
NaphIBMeUreaMe_F.log: 52.6 hours
FuranIBCMe2S_A_02.log: 23.4 hours
NaphIBMeUreaMe_OCF3.log: 140.1 hours
Indole_NMe_IBCONAc_A_02_2.log: 270.1 hours
PyrroleNMeIBCMe2O_A_02.log: 29.2 hours
NpthIBCMe2O_C_02.log: 38.8 hours
perF_NMeCO2_02.log: 42.9 hours
AnthI8BA_B_OCF3.log: 177.3 hours
NpthISO2NMe_D_02.log: 56.5 hours
PyIBCMe2O_D_OCF3.log: 95.0 hours
perF_CMe2O_02.log: 28.7 hours
Indole_NMe_IBCONAc_A_OCF3.log: 149.4 hours
NaphIBMeUreaMe_02.log: 100.9 hours
PyIBCMe2O_D_02.log: 23.9 hours
AnthI8BA_B_02_2.log: 167.1 hours
FuranIBCMe2S_A_OCF3.log: 87.5 hours
PyrroleNMeIBCMe2O_A_OCF3.log: 107.1 hours
NpthISO2NMe_D_OCF3.log: 120.6 hours
perF_CMe2O_OCF3.log: 72.7 hours
FuranIBCMe2S_A_02.log: 23.4 hours
perF_NMeCO2_OCF3.log: 74.9 hours
NpthIBCMe2O_C_OCF3.log: 165.3 hours
PyrroleNMeIBCMe2O_A_OMe.log: 85.8 hours
perF_CMe2O_OMe.log: 75.7 hours
Indole_NMe_IBCONAc_A_02_2.log: 270.1 hours
PyrroleNMeIBCMe2O_A_02.log: 29.2 hours
NpthIBCMe2O_C_02.log: 38.8 hours
PyIBCMe2O_D_OMe.log: 132.3 hours
perF_NMeCO2_02.log: 42.9 hours
NaphIBMeUreaMe_OMe.log: 131.1 hours
NpthISO2NMe_D_OMe.log: 127.2 hours
NpthISO2NMe_D_02.log: 56.5 hours
perF_CMe2O_02.log: 28.7 hours
NpthIBCMe2O_C_OMe.log: 31.6 hours
NaphIBMeUreaMe_02.log: 100.9 hours
PyIBCMe2O_D_02.log: 23.9 hours
AnthI8BA_B_02_2.log: 167.1 hours
FuranIBCMe2S_A_OMe.log: 75.4 hours
Indole_NMe_IBCONAc_A_OMe.log: 124.8 hours
perF_NMeCO2_OMe.log: 71.8 hours
AnthI8BA_B_OMe.log: 164.9 hours
FuranIBCMe2S_A_02.log: 23.4 hours
NpthIBCMe2O_C_Ph.log: 67.2 hours
perF_NMeCO2_Ph.log: 44.0 hours
Indole_NMe_IBCONAc_A_02_2.log: 270.1 hours
PyrroleNMeIBCMe2O_A_02.log: 29.2 hours
NpthIBCMe2O_C_02.log: 38.8 hours
Indole_NMe_IBCONAc_A_Ph.log: 69.2 hours
perF_NMeCO2_02.log: 42.9 hours
PyrroleNMeIBCMe2O_A_Ph.log: 45.5 hours
perF_CMe2O_Ph.log: 45.1 hours
NpthISO2NMe_D_02.log: 56.5 hours
perF_CMe2O_02.log: 28.7 hours
NpthISO2NMe_D_Ph.log: 75.0 hours
AnthI8BA_B_Ph.log: 168.1 hours
NaphIBMeUreaMe_02.log: 100.9 hours
PyIBCMe2O_D_02.log: 23.9 hours
AnthI8BA_B_02_2.log: 167.1 hours
NaphIBMeUreaMe_Ph.log: 88.4 hours
PyIBCMe2O_D_Ph.log: 32.5 hours
FuranIBCMe2S_A_Ph.log: 34.8 hours
FuranIBCMe2S_A_02.log: 23.4 hours
AnthI8BA_B_Me.log: 95.9 hours
NpthISO2NMe_D_Me.log: 54.1 hours
Indole_NMe_IBCONAc_A_02_2.log: 270.1 hours
PyrroleNMeIBCMe2O_A_02.log: 29.2 hours
NpthIBCMe2O_C_02.log: 38.8 hours
perF_NMeCO2_02.log: 42.9 hours
perF_CMe2O_Me.log: 40.0 hours
PyrroleNMeIBCMe2O_A_Me.log: 37.3 hours

NpthISO2NMe_D_02.log: 56.5 hours
Indole_NMe_IBCONAc_A_Me.log: 58.5 hours
perF_CMe2O_02.log: 28.7 hours
perF_NMeCO2_Me.log: 37.7 hours
NpthIBCMe2O_C_Me.log: 51.0 hours
NaphIBMeUreaMe_02.log: 100.9 hours
PyIBCMe2O_D_02.log: 23.9 hours
AnthI8BA_B_02_2.log: 167.1 hours
FuranIBCMe2S_A_Me.log: 26.0 hours
PyIBCMe2O_D_Me.log: 26.2 hours
NaphIBMeUreaMe_Me.log: 68.8 hours
FuranIBCMe2S_A_02.log: 23.4 hours
perF_CMe2O_OTf.log: 59.2 hours
PyrroleNMeIBCMe2O_A_OTf.log: 49.2 hours
Indole_NMe_IBCONAc_A_02_2.log: 270.1 hours
PyrroleNMeIBCMe2O_A_02.log: 29.2 hours
PyIBCMe2O_D_OTf.log: 41.7 hours
NpthIBCMe2O_C_02.log: 38.8 hours
perF_NMeCO2_02.log: 42.9 hours
NpthISO2NMe_D_OTf.log: 88.5 hours
NaphIBMeUreaMe_OTf.log: 99.9 hours
NpthISO2NMe_D_02.log: 56.5 hours
perF_CMe2O_02.log: 28.7 hours
NpthIBCMe2O_C_OTf.log: 93.1 hours
NaphIBMeUreaMe_02.log: 100.9 hours
PyIBCMe2O_D_02.log: 23.9 hours
AnthI8BA_B_02_2.log: 167.1 hours
FuranIBCMe2S_A_OTf.log: 35.8 hours
perF_NMeCO2_OTf.log: 92.5 hours
Indole_NMe_IBCONAc_A_OTf.log: 79.6 hours
FuranIBCMe2S_A_02.log: 23.4 hours
AnthI8BA_B_OTf.log: 151.4 hours
Indole_NMe_IBCONAc_A_02_2.log: 270.1 hours
PyrroleNMeIBCMe2O_A_02.log: 29.2 hours
NpthIBCMe2O_C_02.log: 38.8 hours
perF_NMeCO2_02.log: 42.9 hours
NpthISO2NMe_D_02.log: 56.5 hours
FuranIBCMe2S_A_vinyl.log: 17.7 hours
perF_CMe2O_02.log: 28.7 hours
perF_CMe2O_vinyl.log: 38.4 hours
perF_NMeCO2_vinyl.log: 37.6 hours
NaphIBMeUreaMe_vinyl.log: 58.5 hours
NaphIBMeUreaMe_02.log: 100.9 hours
PyIBCMe2O_D_02.log: 23.9 hours
AnthI8BA_B_02_2.log: 167.1 hours
NpthIBCMe2O_C_vinyl.log: 50.8 hours
NpthISO2NMe_D_vinyl.log: 52.6 hours
PyIBCMe2O_D_vinyl.log: 22.6 hours
PyrroleNMeIBCMe2O_A_vinyl.log: 39.0 hours
AnthI8BA_B_vinyl.log: 83.0 hours
Indole_NMe_IBCONAc_A_vinyl.log: 73.8 hours
FuranIBCMe2S_A_02.log: 23.4 hours
Furan3I_OAc2.log: 23.6 hours
Naph1_OAc2.log: 60.7 hours
Anth_I_OAc_02.log: 170.5 hours
Py_PhI_OAc_02_A.log: 80.1 hours
Naph2_OAc_02.log: 38.4 hours
IndoleNAc3I_OAc_02.log: 75.8 hours
00PhIF_OAc_02.log: 0.8 hours
Thiophene3I_OAc2.log: 23.9 hours
Naph2_OAc2.log: 91.2 hours
Naph1_OAc_02.log: 50.3 hours
5FPhI_OAc_02.log: 49.0 hours
5FPhI_OAc2.log: 40.0 hours
Thiophene3I_OAc_02.log: 12.3 hours
Py_PhI_OAc2.log: 131.2 hours
MesityleneI_OAc_02.log: 57.7 hours
IndoleNAc3I_OAc2.log: 91.0 hours
Furan3I_OAc_02_2.log: 8.4 hours
Anth_I_OAc2.log: 137.5 hours
Me_Pyrrole_3I_OAc_02.log: 36.5 hours
MesityleneI_OAc2.log: 68.1 hours
Me_Pyrrole_3I_OAc2.log: 48.5 hours
MesityleneI_F2.log: 26.7 hours
Py_PhI_F2_02_A.log: 41.1 hours
Me_Pyrrole_3I_F2.log: 7.4 hours
Furan3I_F_02.log: 2.5 hours
Anth_I_F2.log: 47.6 hours
Naph1_F_02.log: 30.6 hours
Thiophene3I_F2.log: 3.1 hours
Py_PhI_F2.log: 49.8 hours
Naph2_F2.log: 12.6 hours
Naph2_F_02.log: 47.0 hours
Thiophene3I_F_02.log: 3.5 hours
IndoleNAc3I_F2.log: 22.5 hours
5FPhI_F_02.log: 18.0 hours
IndoleNAc3I_F_02.log: 40.9 hours
Anth_I_F_02.log: 62.7 hours
5FPhI_F2.log: 8.2 hours
Naph1_F2.log: 15.1 hours
MesityleneI_F_02.log: 30.6 hours
00F_02.log: 0.0 hours
Furan3I_F2.log: 2.8 hours
Me_Pyrrole_3I_F_02.log: 12.7 hours
Naph2_Cl2.log: 30.8 hours
Anth_I_Cl_02.log: 65.3 hours
Thiophene3I_Cl2.log: 4.4 hours
Furan3I_Cl2.log: 4.5 hours
5FPhI_Cl_02.log: 13.7 hours
00Cl_02.log: 0.0 hours
MesityleneI_Cl2.log: 32.4 hours
Thiophene3I_Cl_02.log: 7.1 hours
Furan3I_Cl_02.log: 2.6 hours
Naph2_Cl_02.log: 29.1 hours
Naph1_Cl2.log: 32.0 hours
MesityleneI_Cl_02.log: 31.0 hours
5FPhI_Cl2.log: 10.1 hours
Me_Pyrrole_3I_Cl2.log: 30.4 hours
IndoleNAc3I_Cl2.log: 40.5 hours
Anth_I_Cl2.log: 58.0 hours
Naph1_Cl_02.log: 29.6 hours
Py_PhI_Cl2.log: 84.9 hours
IndoleNAc3I_Cl_02.log: 49.9 hours
Me_Pyrrole_3I_Cl_02.log: 13.9 hours
Py_PhI_Cl_02_A.log: 50.7 hours
Furan3I_OTFA_02.log: 19.6 hours
Py_PhI_OTFA_02.log: 62.6 hours
Py_PhI_OTFA2.log: 144.8 hours
Naph1_OTFA2.log: 73.3 hours
IndoleNAc3I_OTFA2.log: 84.6 hours
Me_Pyrrole_3I_OTFA2.log: 50.1 hours
Me_Pyrrole_3I_OTFA_02.log: 32.5 hours
Naph2_OTFA_02.log: 42.1 hours
5FPhI_OTFA_02.log: 40.9 hours

Thiophene3I_OTFA_02.log: 21.9 hours
Anth_I_OTFA2.log: 109.3 hours
5FPhI_OTFA2.log: 54.4 hours
MesityleneI_OTFA_02.log: 50.8 hours
MesityleneI_OTFA2.log: 73.2 hours
IndoleNAc3I_OTFA_02.log: 56.5 hours
Thiophene3I_OTFA2.log: 33.1 hours
Anth_I_OTFA_02.log: 103.2 hours
Furan3I_OTFA2.log: 37.2 hours
Naph2_OTFA2.log: 79.0 hours
Naph1_OTFA_02.log: 45.0 hours
4FIBA_02.log: 9.9 hours
4FIBA_Cl.log: 8.2 hours
Benzofuran54_CO2I_02.log: 9.1 hours
Benzofuran54_CO2I_Cl.log: 12.5 hours
4FIBA_CF3.log: 45.1 hours
Benzofuran54_CO2I_CF3.log: 12.7 hours
IBamideAc_CF2CF3.log: 33.5 hours
PyIBA_D_02.log: 7.8 hours
IBNMeCO2_02.log: 21.9 hours
IBCCF32O_02.log: 47.9 hours
IBCH2S_02.log: 21.7 hours
IBamideAc_02_2.log: 50.3 hours
Indole_NMe_IBA_A_CF2CF3.log: 45.5 hours
QuinoIBA_L_CF2CF3.log: 34.8 hours
PyrroleNMeIBA_A_CF2CF3.log: 18.9 hours
IBCCF32O_CF2CF3.log: 43.8 hours
IBCH2S_CF2CF3.log: 33.0 hours
NpthIBA_C_CF2CF3.log: 37.1 hours
PyIBA_D_CF2CF3.log: 19.1 hours
IBNMeCO2_CF2CF3.log: 30.2 hours
Indole_NMe_IBA_A_02.log: 18.1 hours
PyrroleNMeIBA_A_02.log: 8.9 hours
FuranIBA_A_02.log: 5.2 hours
QuinoIBA_L_02.log: 20.3 hours
FuranIBA_A_CF2CF3.log: 12.1 hours
NpthIBA_C_02.log: 17.5 hours
PhICF2H_02.log: 0.1 hours
PhISMe_F.log: 8.3 hours
PhICF2H_F.log: 6.8 hours
PhIOSO2Me_F.log: 15.3 hours
PhIvinyl_02.log: 0.2 hours
radical_PhIF_02.log: 5.1 hours
PhISPh_F.log: 30.1 hours
PhISMe_02.log: 0.2 hours
PhISeMe_02.log: 0.2 hours
PhISeMe_F.log: 9.2 hours
PhINMe2_F.log: 9.5 hours
PhIPyrrole_N1_02.log: 1.1 hours
PhIOTf_02.log: 3.3 hours
PhIPyrroleN_F.log: 15.6 hours
PhIvinyl_F.log: 6.2 hours
PhISPh_02.log: 6.3 hours
PhINTf2_02.log: 23.8 hours
PhINMe2_02.log: 0.6 hours
PhINTf2_F.log: 41.0 hours
PhIOSO2Me_02.log: 2.4 hours
PhIOTf_F.log: 14.3 hours
PyIBA_D_02.log: 7.8 hours
PyrroleNMeIBA_A_F.log: 5.2 hours
IBamideAc_F.log: 10.6 hours
IBCCF32O_F.log: 22.6 hours
IBNMeCO2_02.log: 21.9 hours
IBCCF32O_02.log: 47.9 hours
Indole_NMe_IBA_A_F.log: 19.1 hours
IBCH2S_02.log: 21.7 hours
IBamideAc_02_2.log: 50.3 hours
IBCH2S_F.log: 13.4 hours
NpthIBA_C_F.log: 19.8 hours
PyIBA_D_F.log: 4.8 hours
QuinoIBA_L_F.log: 15.3 hours
Indole_NMe_IBA_A_02.log: 18.1 hours
PyrroleNMeIBA_A_02.log: 8.9 hours
FuranIBA_A_02.log: 5.2 hours
QuinoIBA_L_02.log: 20.3 hours
FuranIBA_A_F.log: 2.3 hours
IBNMeCO2_F.log: 11.7 hours
NpthIBA_C_02.log: 17.5 hours
IBA_vinyl_02.log: 0.2 hours
IBA_NTf2_02.log: 23.8 hours
IBA_Ph_02.log: 3.0 hours
IBA_CF2CF3_02.log: 0.7 hours
F_02.log: 0.0 hours
IBA_OCF3_02.log: 0.3 hours
IBA_Me_02.log: 0.1 hours
IBA_OMe_02.log: 0.1 hours
IBA_OTf_02.log: 3.3 hours
IBCCF32O_NTf2.log: 113.8 hours
PyIBA_D_02.log: 7.8 hours
IBNMeCO2_02.log: 21.9 hours
IBCCF32O_02.log: 47.9 hours
IBCH2S_02.log: 21.7 hours
PyIBA_D_NTf2.log: 54.2 hours
IBamideAc_02_2.log: 50.3 hours
NpthIBA_C_NTf2.log: 114.7 hours
IBNMeCO2_NTf2.log: 77.2 hours
QuinoIBA_L_NTf2.log: 98.0 hours
FuranIBA_A_NTf2.log: 43.0 hours
IBamideAc_NTf2.log: 91.9 hours
Indole_NMe_IBA_A_02.log: 18.1 hours
IBCH2S_NTf2.log: 88.7 hours
PyrroleNMeIBA_A_02.log: 8.9 hours
FuranIBA_A_02.log: 5.2 hours
Indole_NMe_IBA_A_NTf2.log: 121.8 hours
QuinoIBA_L_02.log: 20.3 hours
PyrroleNMeIBA_A_NTf2.log: 60.9 hours
NpthIBA_C_02.log: 17.5 hours
PyIBA_D_02.log: 7.8 hours
IBNMeCO2_02.log: 21.9 hours
IBCCF32O_02.log: 47.9 hours
IBCH2S_02.log: 21.7 hours
IBamideAc_02_2.log: 50.3 hours
Indole_NMe_IBA_A_02.log: 18.1 hours
PyrroleNMeIBA_A_02.log: 8.9 hours
FuranIBA_A_02.log: 5.2 hours
QuinoIBA_L_02.log: 20.3 hours
NpthIBA_C_02.log: 17.5 hours
NpthIBA_C_OCF3.log: 63.9 hours
PyIBA_D_02.log: 7.8 hours
IBNMeCO2_02.log: 21.9 hours
IBCCF32O_02.log: 47.9 hours
PyIBA_D_OCF3.log: 64.1 hours
IBCH2S_02.log: 21.7 hours
IBamideAc_02_2.log: 50.3 hours

IBCCF32O_OCF3.log: 68.0 hours
Indole_NMe_IBA_A_OCF3.log: 71.7 hours
PyrroleNMeIBA_A_OCF3.log: 37.3 hours
IBCH2S_OCF3.log: 58.0 hours
IBamideAc_OCF3.log: 62.1 hours
Indole_NMe_IBA_A_02.log: 18.1 hours
PyrroleNMeIBA_A_02.log: 8.9 hours
FuranIBA_A_02.log: 5.2 hours
QuinoIBA_L_02.log: 20.3 hours
FuranIBA_A_OCF3.log: 19.9 hours
QuinoIBA_L_OCF3.log: 61.5 hours
IBNMeCO2_OCF3.log: 45.9 hours
NpthIBA_C_02.log: 17.5 hours
QuinoIBA_L_OTf.log: 66.8 hours
PyIBA_D_02.log: 7.8 hours
IBNMeCO2_02.log: 21.9 hours
IBCCF32O_02.log: 47.9 hours
IBNMeCO2_OTf.log: 48.1 hours
NpthIBA_C_OTf.log: 82.2 hours
IBCH2S_02.log: 21.7 hours
IBamideAc_OTf.log: 36.3 hours
FuranIBA_A_OTf.log: 30.1 hours
IBamideAc_02_2.log: 50.3 hours
IBCCF32O_OTf.log: 111.7 hours
Indole_NMe_IBA_A_OTf.log: 158.6 hours
PyIBA_D_OTf.log: 36.8 hours
PyrroleNMeIBA_A_OTf.log: 42.5 hours
Indole_NMe_IBA_A_02.log: 18.1 hours
IBCH2S_OTf.log: 59.1 hours
PyrroleNMeIBA_A_02.log: 8.9 hours
FuranIBA_A_02.log: 5.2 hours
QuinoIBA_L_02.log: 20.3 hours
NpthIBA_C_02.log: 17.5 hours
PhICN_2.log: 10.7 hours
PhIOCOCOPh_2.log: 97.8 hours
PhIOSO2Me_2.log: 61.8 hours
PhICF2H_2.log: 23.4 hours
PhISMe_2.log: 29.9 hours
PhIOMe_2.log: 27.5 hours
PhIOPh_2.log: 164.5 hours
PhISPh_2.log: 162.5 hours
PhICF3_2.log: 22.7 hours
PhIN3_2.log: 25.4 hours
PhINPhth_2.log: 234.5 hours
PhINMe2_2.log: 43.8 hours
PhISeMe_2.log: 59.1 hours
PhIOCF3_2.log: 35.3 hours
PhIvinyl_2.log: 23.6 hours
PhIOTf_2.log: 91.5 hours
PhIBr_2.log: 8.7 hours
PhIOOtBu_2.log: 72.4 hours
PhINTf2_2.log: 71.2 hours
PyIBA_D_02.log: 7.8 hours
IBCH2S_Me.log: 27.4 hours
IBNMeCO2_02.log: 21.9 hours
IBCCF32O_02.log: 47.9 hours
IBCH2S_02.log: 21.7 hours
PyIBA_D_Me.log: 12.4 hours
IBamideAc_02_2.log: 50.3 hours
IBCCF32O_Me.log: 32.7 hours
IBNMeCO2_Me.log: 24.8 hours
Indole_NMe_IBA_A_Me.log: 31.4 hours
NpthIBA_C_Me.log: 29.5 hours
QuinoIBA_L_Me.log: 27.1 hours
FuranIBA_A_Me.log: 8.9 hours
IBamideAc_Me.log: 24.3 hours
PyrroleNMeIBA_A_Me.log: 15.3 hours
Indole_NMe_IBA_A_02.log: 18.1 hours
PyrroleNMeIBA_A_02.log: 8.9 hours
FuranIBA_A_02.log: 5.2 hours
QuinoIBA_L_02.log: 20.3 hours
NpthIBA_C_02.log: 17.5 hours
QuinoIBA_L_OMe.log: 30.8 hours
PyIBA_D_02.log: 7.8 hours
IBNMeCO2_02.log: 21.9 hours
IBCCF32O_02.log: 47.9 hours
IBNMeCO2_OMe.log: 20.9 hours
IBCH2S_02.log: 21.7 hours
NpthIBA_C_OMe.log: 32.0 hours
FuranIBA_A_OMe.log: 9.2 hours
IBamideAc_OMe.log: 28.5 hours
IBamideAc_02_2.log: 50.3 hours
Indole_NMe_IBA_A_OMe.log: 33.4 hours
IBCCF32O_OMe.log: 43.6 hours
PyIBA_D_OMe.log: 13.1 hours
PyrroleNMeIBA_A_OMe.log: 16.1 hours
IBCH2S_OMe.log: 25.3 hours
Indole_NMe_IBA_A_02.log: 18.1 hours
PyrroleNMeIBA_A_02.log: 8.9 hours
FuranIBA_A_02.log: 5.2 hours
QuinoIBA_L_02.log: 20.3 hours
NpthIBA_C_02.log: 17.5 hours
IBCCF32O_vinyl.log: 47.9 hours
PyIBA_D_02.log: 7.8 hours
FuranIBA_A_vinyl.log: 13.5 hours
IBNMeCO2_02.log: 21.9 hours
PyIBA_D_vinyl.log: 22.9 hours
IBCCF32O_02.log: 47.9 hours
QuinoIBA_L_vinyl.log: 48.5 hours
IBCH2S_02.log: 21.7 hours
IBamideAc_02_2.log: 50.3 hours
Indole_NMe_IBA_A_vinyl.log: 51.5 hours
IBCH2S_vinyl.log: 35.1 hours
IBamideAc_vinyl.log: 51.0 hours
PyrroleNMeIBA_A_vinyl.log: 39.2 hours
Indole_NMe_IBA_A_02.log: 18.1 hours
IBNMeCO2_vinyl.log: 32.4 hours
PyrroleNMeIBA_A_02.log: 8.9 hours
FuranIBA_A_02.log: 5.2 hours
QuinoIBA_L_02.log: 20.3 hours
NpthIBA_C_02.log: 17.5 hours
NpthIBA_C_vinyl.log: 46.7 hours
PhICF2H_02.log: 0.1 hours
PhIvinyl_Cl.log: 7.6 hours
PhISMe_Cl.log: 6.8 hours
PhISeMe_Cl.log: 11.6 hours
PhICF2H_Cl.log: 7.6 hours
PhIvinyl_02.log: 0.2 hours
PhISMe_02.log: 0.2 hours
PhISeMe_02.log: 0.2 hours
PhISPh_Cl.log: 15.9 hours
PhINTf2_Cl.log: 76.9 hours
PhINMe2_Cl.log: 11.4 hours
radical_PhICl_02.log: 4.2 hours

PhIOSO2Me_Cl.log: 13.3 hours
PhIPyrrole_N1_02.log: 1.1 hours
PhIOTf_02.log: 3.3 hours
PhISPh_02.log: 6.3 hours
PhINTf2_02.log: 23.8 hours
PhINMe2_02.log: 0.6 hours
PhIOSO2Me_02.log: 2.4 hours
PhIPyrroleN_Cl.log: 16.6 hours
PhIOTf_Cl.log: 15.3 hours
IBNMeCO2_Ph.log: 62.4 hours
IBCCF32O_Ph.log: 79.8 hours
PyIBA_D_02.log: 7.8 hours
IBNMeCO2_02.log: 21.9 hours
PyIBA_D_Ph.log: 40.4 hours
IBCCF32O_02.log: 47.9 hours
IBCH2S_02.log: 21.7 hours
IBamideAc_02_2.log: 50.3 hours
IBCH2S_Ph.log: 69.3 hours
IBamideAc_Ph.log: 65.8 hours
FuranIBA_A_Ph.log: 26.6 hours
PyrroleNMeIBA_A_Ph.log: 39.3 hours
QuinoIBA_L_Ph.log: 68.7 hours
Indole_NMe_IBA_A_02.log: 18.1 hours
NpthIBA_C_Ph.log: 73.6 hours
PyrroleNMeIBA_A_02.log: 8.9 hours
FuranIBA_A_02.log: 5.2 hours
QuinoIBA_L_02.log: 20.3 hours
Indole_NMe_IBA_A_Ph.log: 82.8 hours
NpthIBA_C_02.log: 17.5 hours
PhIF_Furane3_02.log: 1.1 hours
PhIF_StBu_02.log: 2.0 hours
PhIF_SeMes_02.log: 12.7 hours
PhIF_Mesitylene_02.log: 6.1 hours
PhIF_Thiophene3_02.log: 1.1 hours
PhIF_CCPh_02.log: 4.5 hours
PhIF_Cy_02.log: 6.9 hours
PhIF_5FBz_02.log: 18.7 hours
PhIF_Naph1_02.log: 7.1 hours
PhIF_OCH2CF3_02.log: 1.4 hours
PhIF_Acr_02.log: 13.7 hours
PhIF_Dibenzofuran_02.log: 11.1 hours
PhIF_5FPh_02.log: 3.7 hours
PhIF_Bn_02.log: 4.2 hours
PhIF_SetBu_02.log: 2.7 hours
PhIF_Carbazole_02.log: 11.8 hours
PhIF_SMes_02.log: 12.1 hours
PhIF_OEt_02.log: 0.8 hours
PhIF_Adm_02.log: 15.3 hours
PhIF_Anth2_02.log: 14.1 hours
PhIF_Allene_02.log: 0.4 hours
PhIF_Thiazole_02.log: 1.0 hours
PhIF_Naph2_02.log: 7.2 hours
PhIF_SO3Ph_02.log: 8.8 hours
PhIF_Pyrene_02.log: 18.3 hours
PyrroleNAcIBA_A_carbazole.log: 94.6 hours
IBNMeCO2_02.log: 21.9 hours
carbazole_02.log: 14.3 hours
IBCCF32O_02.log: 47.9 hours
NpthIBA_C_carbazole.log: 116.8 hours
IBA_oMe_carbazole.log: 183.8 hours
IBCH2S_02.log: 21.7 hours
ISOMe_carbazole.log: 86.1 hours
IBamideAc_02_2.log: 50.3 hours
IBCH2S_carbazole.log: 133.4 hours
ISOMe_02.log: 11.1 hours
IBNMeCO2_carbazole.log: 109.4 hours
IBamideAc_carbazole.log: 109.3 hours
IBA_oMe_02.log: 13.6 hours
FuranIBA_A_carbazole.log: 61.1 hours
IBCCF32O_carbazole.log: 135.1 hours
FuranIBA_A_02.log: 5.2 hours
NpthIBA_C_02.log: 17.5 hours
PyrroleNAcIBA_A_02.log: 22.6 hours
IBNMeCO2_02.log: 21.9 hours
allene_02.log: 0.6 hours
IBA_oMe_allene.log: 45.9 hours
IBCCF32O_02.log: 47.9 hours
IBamideAc_allene.log: 50.1 hours
IBCH2S_02.log: 21.7 hours
IBamideAc_02_2.log: 50.3 hours
ISOMe_02.log: 11.1 hours
PyrroleNAcIBA_A_allene.log: 45.0 hours
NpthIBA_C_allene.log: 56.5 hours
IBNMeCO2_allene.log: 39.9 hours
IBA_oMe_02.log: 13.6 hours
FuranIBA_A_allene.log: 22.4 hours
ISOMe_allene.log: 30.0 hours
FuranIBA_A_02.log: 5.2 hours
IBCCF32O_allene.log: 53.1 hours
IBCH2S_allene.log: 45.5 hours
NpthIBA_C_02.log: 17.5 hours
PyrroleNAcIBA_A_02.log: 22.6 hours
ISOMe_Bn.log: 39.9 hours
IBNMeCO2_02.log: 21.9 hours
IBCH2S_Bn.log: 57.5 hours
IBCCF32O_02.log: 47.9 hours
IBNMeCO2_Bn.log: 55.4 hours
IBCH2S_02.log: 21.7 hours
IBCCF32O_Bn.log: 69.6 hours
IBamideAc_02_2.log: 50.3 hours
ISOMe_02.log: 11.1 hours
Bn_02.log: 5.8 hours
FuranIBA_A_Bn.log: 22.7 hours
IBamideAc_Bn.log: 52.1 hours
IBA_oMe_02.log: 13.6 hours
NpthIBA_C_Bn.log: 56.1 hours
PyrroleNAcIBA_A_Bn.log: 52.8 hours
FuranIBA_A_02.log: 5.2 hours
IBA_oMe_Bn.log: 74.7 hours
NpthIBA_C_02.log: 17.5 hours
PyrroleNAcIBA_A_02.log: 22.6 hours
NpthIBA_C_CCPh.log: 78.3 hours
PyrroleNAcIBA_A_CCPh.log: 64.2 hours
IBNMeCO2_02.log: 21.9 hours
IBCCF32O_02.log: 47.9 hours
IBCH2S_02.log: 21.7 hours
ISOMe_CCPh.log: 55.5 hours
IBamideAc_02_2.log: 50.3 hours
00CCPh_02.log: 6.9 hours
ISOMe_02.log: 11.1 hours
IBCCF32O_CCPh.log: 94.9 hours
IBA_oMe_CCPh.log: 64.1 hours
IBA_oMe_02.log: 13.6 hours
IBCH2S_CCPh.log: 82.6 hours

IBamideAc_CCPh.log: 73.5 hours
FuranIBA_A_02.log: 5.2 hours
FuranIBA_A_CCPh.log: 22.8 hours
NpthIBA_C_02.log: 17.5 hours
PyrroleNAcIBA_A_02.log: 22.6 hours
IBNMeCO2_CCPh.log: 73.2 hours
IBA_oMe_OCH2CF3.log: 45.4 hours
IBNMeCO2_02.log: 21.9 hours
FuranIBA_A_OCH2CF3.log: 22.0 hours
IBCCF32O_02.log: 47.9 hours
IBCCF32O_OCH2CF3.log: 64.6 hours
IBNMeCO2_OCH2CF3.log: 36.7 hours
00OCH2CF3_02.log: 1.6 hours
IBCH2S_02.log: 21.7 hours
IBamideAc_02_2.log: 50.3 hours
ISOMe_02.log: 11.1 hours
ISOMe_OCH2CF3.log: 31.3 hours
PyrroleNAcIBA_A_OCH2CF3.log: 36.2 hours
IBA_oMe_02.log: 13.6 hours
FuranIBA_A_02.log: 5.2 hours
IBCH2S_OCH2CF3.log: 45.2 hours
NpthIBA_C_OCH2CF3.log: 43.6 hours
IBamideAc_OCH2CF3.log: 55.9 hours
NpthIBA_C_02.log: 17.5 hours
PyrroleNAcIBA_A_02.log: 22.6 hours
ISOMe_OEt.log: 35.8 hours
IBNMeCO2_02.log: 21.9 hours
IBCCF32O_02.log: 47.9 hours
IBCH2S_02.log: 21.7 hours
IBamideAc_02_2.log: 50.3 hours
ISOMe_02.log: 11.1 hours
IBCH2S_OEt.log: 56.6 hours
PyrroleNAcIBA_A_OEt.log: 51.8 hours
IBNMeCO2_OEt.log: 48.4 hours
IBA_oMe_02.log: 13.6 hours
IBA_oMe_OEt.log: 53.4 hours
00OEt_02.log: 0.6 hours
IBCCF32O_OEt.log: 89.8 hours
NpthIBA_C_OEt.log: 57.7 hours
FuranIBA_A_02.log: 5.2 hours
IBamideAc_OEt.log: 61.9 hours
FuranIBA_A_OEt.log: 9.3 hours
NpthIBA_C_02.log: 17.5 hours
PyrroleNAcIBA_A_02.log: 22.6 hours
IBNMeCO2_02.log: 21.9 hours
IBCCF32O_02.log: 47.9 hours
IBCH2S_naph.log: 69.5 hours
IBamideAc_naph.log: 66.8 hours
IBCH2S_02.log: 21.7 hours
IBamideAc_02_2.log: 50.3 hours
ISOMe_02.log: 11.1 hours
FuranIBA_A_naph.log: 31.1 hours
IBNMeCO2_naph.log: 61.5 hours
NpthIBA_C_naph.log: 71.8 hours
PyrroleNAcIBA_A_naph.log: 63.7 hours
IBA_oMe_02.log: 13.6 hours
00naph_02.log: 9.1 hours
ISOMe_naph.log: 50.4 hours
FuranIBA_A_02.log: 5.2 hours
IBCCF32O_naph.log: 90.1 hours
IBA_oMe_naph.log: 115.9 hours
NpthIBA_C_02.log: 17.5 hours
PyrroleNAcIBA_A_02.log: 22.6 hours
PhIF_SO3Ph.log: 65.8 hours
PhIF_Furane3_02.log: 1.1 hours
PhIF_StBu.log: 41.6 hours
PhIF_Dibenzofuran.log: 61.8 hours
PhIF_StBu_02.log: 3.4 hours
PhIF_CCPh.log: 38.2 hours
PhIF_Pyrene.log: 89.8 hours
PhIF_SeMes_02.log: 12.7 hours
PhIF_Mesitylene_02.log: 6.1 hours
PhIF_5FPh.log: 42.8 hours
PhIF_Thiophene3_02.log: 1.1 hours
PhIF_CCPh_02.log: 4.5 hours
PhIF_Cy_02.log: 6.9 hours
PhIF_Thiazole.log: 21.5 hours
PhIF_SMes.log: 28.2 hours
PhIF_5FBz_02.log: 18.7 hours
PhIF_Naph1_02.log: 7.1 hours
PhIF_5FBz.log: 57.1 hours
PhIF_Acr.log: 78.1 hours
PhIF_Allene.log: 19.9 hours
PhIF_Bn.log: 40.6 hours
PhIF_OCH2CF3_02.log: 1.4 hours
PhIF_Acr_02.log: 13.7 hours
PhIF_OCH2CF3.log: 24.9 hours
PhIF_Dibenzofuran_02.log: 11.1 hours
00PhIF_02.log: 5.1 hours
PhIF_5FPh_02.log: 3.7 hours
PhIF_Anth2.log: 70.0 hours
PhIF_SetBu.log: 49.6 hours
PhIF_Naph1.log: 60.0 hours
PhIF_Bn_02.log: 4.2 hours
PhIF_SetBu_02.log: 2.7 hours
PhIF_Carbazole_02.log: 11.8 hours
PhIF_SMes_02.log: 12.1 hours
PhIF_Naph2.log: 49.5 hours
PhIF_OEt_02.log: 0.8 hours
PhIF_Cy.log: 44.0 hours
PhIF_Thiophene3.log: 26.6 hours
PhIF_Furane3.log: 24.6 hours
PhIF_Adm.log: 87.6 hours
PhIF_Mesitylene.log: 68.0 hours
PhIF_Adm_02.log: 15.3 hours
PhIF_Anth2_02.log: 14.1 hours
PhIF_Allene_02.log: 0.4 hours
PhIF_Carbazole.log: 70.6 hours
PhIF_SeMes.log: 100.3 hours
PhIF_Thiazole_02.log: 1.0 hours
PhIF_Naph2_02.log: 7.2 hours
PhIF_SO3Ph_02.log: 8.8 hours
PhIF_OEt.log: 21.8 hours
PhIF_Pyrene_02.log: 18.3 hours
IBA_SeMes_02.log: 12.7 hours
IBA_OEt.log: 29.7 hours
IBA_SMes.log: 82.5 hours
IBA_Mesitylene_02.log: 6.1 hours
IBA_SMes_02.log: 12.1 hours
IBA_Thiophene3_02.log: 1.1 hours
IBA_5FPh_02.log: 3.7 hours
IBA_Allene_02.log: 0.4 hours
IBA_Adm_02.log: 15.3 hours
IBA_Carbazole.log: 75.1 hours

IBA_OCH2CF3_02.log: 1.4 hours
IBA_OCH2CF3.log: 32.3 hours
IBA_Pyrene_02.log: 18.3 hours
IBA_Adm.log: 87.7 hours
IBA_5FBz.log: 59.3 hours
IBA_SO3Ph.log: 62.0 hours
IBA_OEt_02.log: 0.8 hours
IBA_StBu.log: 46.0 hours
IBA_Cy.log: 50.5 hours
IBA_Bn.log: 40.4 hours
IBA_5FPh.log: 50.1 hours
IBA_Dibenzofuran.log: 62.6 hours
IBA_Dibenzofuran_02.log: 11.1 hours
IBA_Naph1_02.log: 7.1 hours
IBA_Allene.log: 23.7 hours
IBA_Thiazole.log: 27.5 hours
IBA_Thiophene3.log: 28.8 hours
IBA_Furane3.log: 30.3 hours
IBA_Cy_02.log: 6.9 hours
IBA_Acr_02.log: 13.7 hours
IBA_Mesitylene.log: 65.8 hours
IBA_CCPh.log: 48.0 hours
IBA_CCPh_02.log: 4.5 hours
IBA_StBu_02.log: 3.4 hours
IBA_Acr.log: 74.7 hours
IBA_SetBu_02.log: 2.7 hours
IBA_Furane3_02.log: 1.1 hours
IBA_SeMes.log: 100.9 hours
IBA_SO3Ph_02.log: 8.8 hours
IBA_Naph2_02.log: 7.2 hours
00IBA_02.log: 5.6 hours
IBA_Anth2_02.log: 14.1 hours
IBA_Anth2.log: 72.6 hours
IBA_Pyrene.log: 104.0 hours
IBA_Naph1.log: 57.3 hours
IBA_SetBu.log: 52.7 hours
IBA_Thiazole_02.log: 1.0 hours
IBA_5FBz_02.log: 18.7 hours
IBA_Bn_02.log: 4.2 hours
IBA_Naph2.log: 50.7 hours
IBA_Carbazole_02.log: 11.8 hours
AnthI8BA_B_carbazole.log: 1201.7 hours
NpthIBCMe2O_C_carbazole.log: 308.1 hours
perF_CMe2O_carbazole.log: 287.6 hours
Indole_NMe_IBCONAc_A_02_2.log: 270.1 hours
PyrroleNMeIBCMe2O_A_02.log: 29.2 hours
NpthIBCMe2O_C_02.log: 38.8 hours
perF_NMeCO2_02.log: 42.9 hours
PyIBCMe2O_D_carbazole.log: 250.6 hours
NpthISO2NMe_D_02.log: 56.5 hours
Indole_NMe_IBCONAc_A_carbazole.log: 329.1 hours
perF_CMe2O_02.log: 28.7 hours
NaphIBMeUreaMe_carbazole.log: 431.4 hours
PyrroleNMeIBCMe2O_A_carbazole.log: 274.8 hours
NaphIBMeUreaMe_02.log: 100.9 hours
PyIBCMe2O_D_02.log: 23.9 hours
AnthI8BA_B_02_2.log: 167.1 hours
FuranIBCMe2S_A_carbazole.log: 256.7 hours
00carbazole_02.log: 14.3 hours
perF_NMeCO2_carbazole.log: 296.9 hours
FuranIBCMe2S_A_02.log: 23.4 hours
NpthISO2NMe_D_carbazole.log: 325.2 hours
FuranIBCMe2S_A_OEt.log: 27.0 hours
Indole_NMe_IBCONAc_A_02_2.log: 270.1 hours
PyrroleNMeIBCMe2O_A_02.log: 29.2 hours
NpthIBCMe2O_C_02.log: 38.8 hours
perF_NMeCO2_02.log: 42.9 hours
NpthISO2NMe_D_02.log: 56.5 hours
AnthI8BA_B_OEt.log: 165.2 hours
perF_CMe2O_02.log: 28.7 hours
perF_NMeCO2_OEt.log: 49.0 hours
Indole_NMe_IBCONAc_A_OEt.log: 94.1 hours
PyIBCMe2O_D_OEt.log: 37.4 hours
NaphIBMeUreaMe_02.log: 100.9 hours
PyIBCMe2O_D_02.log: 23.9 hours
NpthISO2NMe_D_OEt.log: 81.6 hours
NaphIBMeUreaMe_OEt.log: 94.6 hours
perF_CMe2O_OEt.log: 49.4 hours
AnthI8BA_B_02_2.log: 167.1 hours
PyrroleNMeIBCMe2O_A_OEt.log: 36.6 hours
00OEt_02.log: 0.6 hours
NpthIBCMe2O_C_OEt.log: 72.5 hours
FuranIBCMe2S_A_02.log: 23.4 hours
Indole_NMe_IBCONAc_A_02_2.log: 270.1 hours
PyrroleNMeIBCMe2O_A_02.log: 29.2 hours
Me_Pyrrole_3I_02.log: 12.7 hours
NpthIBCMe2O_C_02.log: 38.8 hours
perF_NMeCO2_02.log: 42.9 hours
Anth_I_02.log: 62.7 hours
Py_PhI_SO3Ph.log: 82.6 hours
NpthISO2NMe_D_02.log: 56.5 hours
FuranIBCMe2S_A_SO3Ph.log: 75.6 hours
Me_Pyrrole_3I_SO3Ph.log: 50.6 hours
perF_CMe2O_02.log: 28.7 hours
Py_PhI_02.log: 41.1 hours
PyIBCMe2O_D_SO3Ph.log: 71.7 hours
NaphIBMeUreaMe_02.log: 100.9 hours
NpthIBCMe2O_C_SO3Ph.log: 158.4 hours
PyIBCMe2O_D_02.log: 23.9 hours
NpthISO2NMe_D_SO3Ph.log: 145.1 hours
AnthI8BA_B_02_2.log: 167.1 hours
perF_NMeCO2_SO3Ph.log: 138.3 hours
Anth_I_SO3Ph.log: 144.7 hours
NaphIBMeUreaMe_SO3Ph.log: 283.2 hours
perF_CMe2O_SO3Ph_2.log: 90.2 hours
AnthI8BA_B_SO3Ph_2.log: 233.5 hours
FuranIBCMe2S_A_02.log: 23.4 hours
PhIF_SO3Ph_02.log: 8.8 hours
Indole_NMe_IBCONAc_A_SO3Ph.log: 146.1 hours
PyrroleNMeIBCMe2O_A_SO3Ph.log: 87.6 hours
Indole_NMe_IBCONAc_A_naph.log: 291.9 hours
Indole_NMe_IBCONAc_A_02_2.log: 270.1 hours
PyrroleNMeIBCMe2O_A_02.log: 29.2 hours
NpthIBCMe2O_C_02.log: 38.8 hours
FuranIBCMe2S_A_naph.log: 165.1 hours
perF_NMeCO2_02.log: 42.9 hours
NpthISO2NMe_D_naph.log: 284.5 hours
PyrroleNMeIBCMe2O_A_naph.log: 207.6 hours
NpthISO2NMe_D_02.log: 56.5 hours
perF_CMe2O_02.log: 28.7 hours
perF_CMe2O_naph.log: 217.2 hours
perF_NMeCO2_naph.log: 223.2 hours
NpthIBCMe2O_C_naph.log: 262.5 hours
NaphIBMeUreaMe_naph.log: 286.3 hours

NaphIBMeUreaMe_02.log: 100.9 hours
PyIBCMe2O_D_02.log: 23.9 hours
AnthI8BA_B_02_2.log: 167.1 hours
AnthI8BA_B_naph.log: 356.5 hours
00naph_02.log: 9.1 hours
PyIBCMe2O_D_naph.log: 171.9 hours
FuranIBCMe2S_A_02.log: 23.4 hours
PhIF_Furane3_02.log: 1.1 hours
Indole_NMe_IBCONAc_A_02_2.log: 270.1 hours
PyrroleNMeIBCMe2O_A_02.log: 29.2 hours
Me_Pyrrole_3I_02.log: 12.7 hours
NpthIBCMe2O_C_02.log: 38.8 hours
NaphIBMeUreaMe_3Furan.log: 87.6 hours
perF_NMeCO2_02.log: 42.9 hours
Anth_I_02.log: 62.7 hours
perF_NMeCO2_3Furan.log: 45.1 hours
Py_PhI_3Furan.log: 45.4 hours
PyIBCMe2O_D_3Furan.log: 30.9 hours
perF_CMe2O_3Furan.log: 45.5 hours
PyrroleNMeIBCMe2O_A_3Furan.log: 44.8 hours
NpthISO2NMe_D_02.log: 56.5 hours
FuranIBCMe2S_A_3Furan.log: 130.8 hours
perF_CMe2O_02.log: 28.7 hours
NpthIBCMe2O_C_3Furan.log: 62.3 hours
Anth_I_3Furan.log: 94.5 hours
Py_PhI_02.log: 41.1 hours
NaphIBMeUreaMe_02.log: 100.9 hours
PyIBCMe2O_D_02.log: 23.9 hours
Indole_NMe_IBCONAc_A_3Furan.log: 90.3 hours
AnthI8BA_B_02_2.log: 167.1 hours
NpthISO2NMe_D_3Furan.log: 74.4 hours
AnthI8BA_B_3Furan.log: 136.4 hours
FuranIBCMe2S_A_02.log: 23.4 hours
Me_Pyrrole_3I_3Furan.log: 15.6 hours
Py_PhI_F2_02_A.log: 41.1 hours
perF_NMeCO2_SetBu.log: 70.1 hours
Indole_NMe_IBCONAc_A_02_2.log: 270.1 hours
Anth_I_SetBu.log: 97.2 hours
NaphIBMeUreaMe_SetBu.log: 130.2 hours
PyrroleNMeIBCMe2O_A_02.log: 29.2 hours
PyIBCMe2O_D_SetBu.log: 49.6 hours
NpthIBCMe2O_C_02.log: 38.8 hours
perF_NMeCO2_02.log: 42.9 hours
NpthIBCMe2O_C_SetBu.log: 91.0 hours
NpthISO2NMe_D_SetBu.log: 125.0 hours
NpthISO2NMe_D_02.log: 56.5 hours
Indole_NMe_IBCONAc_A_SetBu.log: 126.6 hours
AnthI8BA_B_SetBu.log: 231.7 hours
PyrroleNMeIBCMe2O_A_SetBu.log: 72.9 hours
perF_CMe2O_02.log: 28.7 hours
NaphIBMeUreaMe_02.log: 100.9 hours
PyIBCMe2O_D_02.log: 23.9 hours
Py_PhI_SetBu.log: 61.3 hours
IBA_SetBu_02.log: 2.7 hours
AnthI8BA_B_02_2.log: 167.1 hours
Anth_I_F_02.log: 62.7 hours
FuranIBCMe2S_A_02.log: 23.4 hours
perF_CMe2O_SetBu.log: 72.5 hours
FuranIBCMe2S_A_SetBu.log: 44.0 hours
Me_Pyrrole_3I_SetBu.log: 28.9 hours
Me_Pyrrole_3I_F_02.log: 12.7 hours
NaphIBMeUreaMe_CCPh.log: 177.7 hours
Indole_NMe_IBCONAc_A_02_2.log: 270.1 hours
PyrroleNMeIBCMe2O_A_02.log: 29.2 hours
NpthIBCMe2O_C_02.log: 38.8 hours
perF_NMeCO2_02.log: 42.9 hours
AnthI8BA_B_CCPh.log: 237.8 hours
NpthISO2NMe_D_02.log: 56.5 hours
00CCPh_02.log: 6.9 hours
perF_CMe2O_02.log: 28.7 hours
PyIBCMe2O_D_CCPh.log: 95.4 hours
Indole_NMe_IBCONAc_A_CCPh.log: 162.1 hours
NaphIBMeUreaMe_02.log: 100.9 hours
PyIBCMe2O_D_02.log: 23.9 hours
AnthI8BA_B_02_2.log: 167.1 hours
FuranIBCMe2S_A_CCPh.log: 94.7 hours
NpthISO2NMe_D_CCPh.log: 162.9 hours
PyrroleNMeIBCMe2O_A_CCPh.log: 118.2 hours
perF_CMe2O_CCPh.log: 151.8 hours
FuranIBCMe2S_A_02.log: 23.4 hours
perF_NMeCO2_CCPh.log: 135.9 hours
NpthIBCMe2O_C_CCPh.log: 164.3 hours
Py_PhI_F2_02_A.log: 41.1 hours
AnthI8BA_B_StBu.log: 271.8 hours
Indole_NMe_IBCONAc_A_02_2.log: 270.1 hours
PyrroleNMeIBCMe2O_A_02.log: 29.2 hours
NpthIBCMe2O_C_02.log: 38.8 hours
NaphIBMeUreaMe_StBu.log: 141.9 hours
perF_NMeCO2_02.log: 42.9 hours
Py_PhI_StBu.log: 60.5 hours
PhIF_StBu_02_2.log: 3.4 hours
NpthISO2NMe_D_02.log: 56.5 hours
PyIBCMe2O_D_StBu.log: 59.4 hours
Anth_I_StBu.log: 97.0 hours
perF_CMe2O_02.log: 28.7 hours
Me_Pyrrole_3I_StBu.log: 35.1 hours
FuranIBCMe2S_A_StBu.log: 58.6 hours
NaphIBMeUreaMe_02.log: 100.9 hours
PyIBCMe2O_D_02.log: 23.9 hours
NpthISO2NMe_D_StBu.log: 101.3 hours
PyrroleNMeIBCMe2O_A_StBu.log: 64.3 hours
AnthI8BA_B_02_2.log: 167.1 hours
Indole_NMe_IBCONAc_A_StBu.log: 127.6 hours
Anth_I_F_02.log: 62.7 hours
perF_CMe2O_StBu.log: 73.2 hours
NpthIBCMe2O_C_StBu.log: 91.5 hours
perF_NMeCO2_StBu.log: 69.9 hours
FuranIBCMe2S_A_02.log: 23.4 hours
Me_Pyrrole_3I_F_02.log: 12.7 hours
00Bn_02.log: 5.8 hours
Py_PhI_OCH2CF3_F.log: 117.6 hours
00OCH2CF3_02.log: 1.6 hours
00CCPh_02.log: 6.9 hours
00allene_02.log: 0.6 hours
00OEt_02.log: 0.6 hours
00naph_02.log: 9.1 hours
Py_PhI_Bn_F.log: 125.2 hours
00carbazole_02.log: 14.3 hours
Py_PhI_OEt_F.log: 93.8 hours
Py_PhI_CCPh_F.log: 119.2 hours
Py_PhI_Naph_F.log: 138.4 hours
Py_PhI_carbazole_F.log: 167.0 hours
Py_PhI_Allene_F.log: 86.6 hours
01Py_PhI_F2_02.log: 41.1 hours

00Bn_02.log: 5.8 hours
00OCH2CF3_02.log: 1.6 hours
Anth_I_OCH2CF3_F.log: 137.4 hours
Anth_I_OEt_F.log: 110.7 hours
00CCPh_02.log: 6.9 hours
Anth_I_carbazole_F.log: 242.2 hours
Anth_I_Naph_F.log: 169.8 hours
Anth_I_Bn_F.log: 119.8 hours
Anth_I_CCPh_F.log: 170.0 hours
00allene_02.log: 0.6 hours
00OEt_02.log: 0.6 hours
Anth_I_F_02.log: 62.7 hours
00naph_02.log: 9.1 hours
00carbazole_02.log: 14.3 hours
Anth_I_Allene_F.log: 89.0 hours
Me_Pyrrole_3I_Allene_F.log: 24.2 hours
Me_Pyrrole_3I_CCPh_F.log: 34.8 hours
Me_Pyrrole_3I_carbazole_F.log: 72.7 hours
00Bn_02.log: 5.8 hours
Me_Pyrrole_3I_Naph_F.log: 84.3 hours
00OCH2CF3_02.log: 1.6 hours
00CCPh_02.log: 6.9 hours
Me_Pyrrole_3I_OCH2CF3_F.log: 52.3 hours
Me_Pyrrole_3I_Bn_F.log: 38.9 hours
Me_Pyrrole_3I_OEt_F.log: 34.1 hours
00allene_02.log: 0.6 hours
00OEt_02.log: 0.6 hours
00naph_02.log: 9.1 hours
00carbazole_02.log: 14.3 hours
Me_Pyrrole_3I_F_02.log: 12.7 hours
Indole_NMe_IBCONAc_A_2thiazole.log: 104.4 hours
Indole_NMe_IBCONAc_A_02_2.log: 270.1 hours
PyrroleNMeIBCMe2O_A_02.log: 29.2 hours
Me_Pyrrole_3I_02.log: 12.7 hours
NpthIBCMe2O_C_02.log: 38.8 hours
NaphIBMeUreaMe_2thiazole.log: 91.9 hours
perF_NMeCO2_02.log: 42.9 hours
Anth_I_02.log: 62.7 hours
perF_CMe2O_2thiazole.log: 40.2 hours
NpthISO2NMe_D_02.log: 56.5 hours
NpthIBCMe2O_C_2thiazole.log: 71.5 hours
AnthI8BA_B_2thiazole.log: 146.5 hours
PyIBCMe2O_D_2thiazole.log: 23.4 hours
perF_CMe2O_02.log: 28.7 hours
Py_PhI_02.log: 41.1 hours
NaphIBMeUreaMe_02.log: 100.9 hours
PyIBCMe2O_D_02.log: 23.9 hours
AnthI8BA_B_02_2.log: 167.1 hours
Py_PhI_2thiazole.log: 44.6 hours
NpthISO2NMe_D_2thiazole.log: 52.0 hours
perF_NMeCO2_2thiazole.log: 49.2 hours
Anth_I_2thiazole.log: 52.1 hours
PyrroleNMeIBCMe2O_A_2thiazole.log: 40.0 hours
Me_Pyrrole_3I_2thiazole.log: 15.4 hours
FuranIBCMe2S_A_02.log: 23.4 hours
FuranIBCMe2S_A_2thiazole.log: 32.7 hours
PhIF_Thiazole_02.log: 1.0 hours
perF_CMe2O_Bn.log: 112.4 hours
Indole_NMe_IBCONAc_A_02_2.log: 270.1 hours
PyrroleNMeIBCMe2O_A_02.log: 29.2 hours
NpthIBCMe2O_C_02.log: 38.8 hours
NpthISO2NMe_D_Bn.log: 147.4 hours
perF_NMeCO2_02.log: 42.9 hours
00Bn_02.log: 5.8 hours
AnthI8BA_B_Bn.log: 191.5 hours
NpthIBCMe2O_C_Bn.log: 136.6 hours
NpthISO2NMe_D_02.log: 56.5 hours
perF_NMeCO2_Bn.log: 112.6 hours
perF_CMe2O_02.log: 28.7 hours
Indole_NMe_IBCONAc_A_Bn.log: 146.7 hours
PyrroleNMeIBCMe2O_A_Bn.log: 108.1 hours
FuranIBCMe2S_A_Bn.log: 81.7 hours
NaphIBMeUreaMe_02.log: 100.9 hours
PyIBCMe2O_D_02.log: 23.9 hours
AnthI8BA_B_02_2.log: 167.1 hours
FuranIBCMe2S_A_02.log: 23.4 hours
NaphIBMeUreaMe_Bn.log: 152.4 hours
PyIBCMe2O_D_Bn.log: 89.2 hours
AnthI8BA_B_Cy.log: 206.3 hours
NpthISO2NMe_D_Cy.log: 98.9 hours
Indole_NMe_IBCONAc_A_02_2.log: 270.1 hours
PyrroleNMeIBCMe2O_A_02.log: 29.2 hours
Me_Pyrrole_3I_02.log: 12.7 hours
NpthIBCMe2O_C_02.log: 38.8 hours
Py_PhI_Cy.log: 106.5 hours
perF_NMeCO2_02.log: 42.9 hours
Anth_I_02.log: 62.7 hours
perF_CMe2O_Cy.log: 74.2 hours
PhIF_Cy_02.log: 6.9 hours
PyrroleNMeIBCMe2O_A_Cy.log: 76.6 hours
NpthISO2NMe_D_02.log: 56.5 hours
Indole_NMe_IBCONAc_A_Cy.log: 171.0 hours
perF_CMe2O_02.log: 28.7 hours
Anth_I_Cy.log: 118.8 hours
perF_NMeCO2_Cy.log: 61.9 hours
Py_PhI_02.log: 41.1 hours
NpthIBCMe2O_C_Cy.log: 105.1 hours
Me_Pyrrole_3I_Cy.log: 42.1 hours
NaphIBMeUreaMe_02.log: 100.9 hours
PyIBCMe2O_D_02.log: 23.9 hours
AnthI8BA_B_02_2.log: 167.1 hours
FuranIBCMe2S_A_Cy.log: 86.4 hours
PyIBCMe2O_D_Cy.log: 62.1 hours
NaphIBMeUreaMe_Cy.log: 132.8 hours
FuranIBCMe2S_A_02.log: 23.4 hours
NpthIBCMe2O_C_Allene.log: 58.6 hours
Indole_NMe_IBCONAc_A_02_2.log: 270.1 hours
PyrroleNMeIBCMe2O_A_02.log: 29.2 hours
NpthIBCMe2O_C_02.log: 38.8 hours
perF_NMeCO2_02.log: 42.9 hours
FuranIBCMe2S_A_Allene.log: 22.5 hours
PyrroleNMeIBCMe2O_A_Allene.log: 30.1 hours
perF_CMe2O_Allene.log: 34.3 hours
PyIBCMe2O_D_Allene.log: 25.8 hours
NpthISO2NMe_D_02.log: 56.5 hours
perF_NMeCO2_Allene.log: 40.0 hours
NaphIBMeUreaMe_Allene.log: 71.1 hours
perF_CMe2O_02.log: 28.7 hours
NaphIBMeUreaMe_02.log: 100.9 hours
PyIBCMe2O_D_02.log: 23.9 hours
AnthI8BA_B_02_2.log: 167.1 hours
00allene_02.log: 0.6 hours
AnthI8BA_B_Allene.log: 130.2 hours
NpthISO2NMe_D_Allene.log: 62.2 hours

Indole_NMe_IBCONAc_A_Allene.log: 83.6 hours
FuranIBCMe2S_A_02.log: 23.4 hours
PyrroleNMeIBCMe2O_A_OCH2CF3.log: 40.8 hours
Indole_NMe_IBCONAc_A_02_2.log: 270.1 hours
PyrroleNMeIBCMe2O_A_02.log: 29.2 hours
NpthIBCMe2O_C_02.log: 38.8 hours
perF_NMeCO2_02.log: 42.9 hours
00OCH2CF3_02.log: 1.6 hours
NpthISO2NMe_D_02.log: 56.5 hours
NpthIBCMe2O_C_OCH2CF3.log: 83.6 hours
perF_NMeCO2_OCH2CF3.log: 194.0 hours
perF_CMe2O_02.log: 28.7 hours
NaphIBMeUreaMe_OCH2CF3.log: 92.8 hours
Indole_NMe_IBCONAc_A_OCH2CF3.log: 114.4 hours
perF_CMe2O_OCH2CF3.log: 45.4 hours
AnthI8BA_B_OCH2CF3.log: 204.2 hours
NaphIBMeUreaMe_02.log: 100.9 hours
PyIBCMe2O_D_02.log: 23.9 hours
FuranIBCMe2S_A_OCH2CF3.log: 30.4 hours
AnthI8BA_B_02_2.log: 167.1 hours
PyIBCMe2O_D_OCH2CF3.log: 37.4 hours
NpthISO2NMe_D_OCH2CF3.log: 86.6 hours
FuranIBCMe2S_A_02.log: 23.4 hours
Indole_NMe_IBCONAc_A_02_2.log: 270.1 hours
PyrroleNMeIBCMe2O_A_02.log: 29.2 hours
Me_Pyrrole_3I_02.log: 12.7 hours
NpthIBCMe2O_C_02.log: 38.8 hours
perF_CMe2O_ArF5.log: 61.2 hours
perF_NMeCO2_02.log: 42.9 hours
Anth_I_02.log: 62.7 hours
NpthIBCMe2O_C_ArF5.log: 79.8 hours
perF_NMeCO2_ArF5.log: 62.9 hours
NpthISO2NMe_D_02.log: 56.5 hours
Indole_NMe_IBCONAc_A_ArF5.log: 108.0 hours
perF_CMe2O_02.log: 28.7 hours
FuranIBCMe2S_A_ArF5.log: 45.5 hours
Py_PhI_02.log: 41.1 hours
PyrroleNMeIBCMe2O_A_ArF5.log: 57.9 hours
NpthISO2NMe_D_ArF5.log: 101.2 hours
Me_Pyrrole_3I_ArF5.log: 28.8 hours
NaphIBMeUreaMe_02.log: 100.9 hours
PyIBCMe2O_D_02.log: 23.9 hours
PhIF_5FPh_02.log: 3.7 hours
AnthI8BA_B_02_2.log: 167.1 hours
PyIBCMe2O_D_ArF5.log: 65.8 hours
Anth_I_ArF5.log: 96.7 hours
NaphIBMeUreaMe_ArF5.log: 95.0 hours
Py_PhI_ArF5.log: 67.1 hours
FuranIBCMe2S_A_02.log: 23.4 hours
AnthI8BA_B_ArF5.log: 220.8 hours

5. Cartesian Coordinates and Energies

IBA_vinyl.log
Energy (E) = -508.475695333 Hartree
Enthalpy (H) = -508.327783 Hartree
Gibbs free energy (G) = -508.378927 Hartree
Charge = 0, Spin = 1
C -1.644277 2.720998 0.046791
C -2.865590 2.058047 -0.016140
C -2.907265 0.673246 -0.057876
C -1.729927 -0.067448 -0.029524
C -0.544643 0.625628 0.039698
C -0.454410 2.002268 0.075444
H -1.610192 3.801512 0.070802
H -3.784396 2.627732 -0.038389
H -3.834250 0.118570 -0.116273
H 0.496603 2.514066 0.108450
C -1.764916 -1.587034 -0.103661
O -0.595874 -2.113687 -0.110489
O -2.845597 -2.144279 -0.155297
I 1.183169 -0.616684 0.099913
C 2.508623 1.044893 0.313369
H 2.514983 1.529019 1.280974
C 3.341830 1.379994 -0.657243
H 3.330961 0.886241 -1.621425
H 4.073542 2.167292 -0.518364

IBA_vinyl_02.log
Energy (E) = -77.8216603981 Hartree
Enthalpy (H) = -77.780890 Hartree
Gibbs free energy (G) = -77.807410 Hartree
Charge = 0, Spin = 2
C -0.704355 -0.140419 0.000022
H -1.600698 0.452487 -0.000011
C 0.585634 0.029359 -0.000062
H 1.276559 -0.807873 0.000122
H 1.036461 1.021749 0.000130

IBA_TMS.log
Energy (E) = -839.637241186 Hartree
Enthalpy (H) = -839.414709 Hartree
Gibbs free energy (G) = -839.479693 Hartree
Charge = 0, Spin = 1
C -1.635657 2.879616 0.000025
C -2.974442 2.503501 0.000003
C -3.318096 1.161857 -0.000003
C -2.342420 0.165721 -0.000002
C -1.033515 0.581613 0.000007
C -0.643101 1.908342 0.000030
H -1.356943 3.924553 0.000039
H -3.746354 3.260948 0.000001
H -4.345127 0.821091 0.000000
H 0.396184 2.201181 0.000070
C -2.776723 -1.312136 0.000008
O -1.804431 -2.118952 -0.000079
O -3.981666 -1.525352 0.000089
I 0.447925 -0.977331 0.000001
C 3.806716 -0.961855 0.000097
H 3.697871 -1.590743 -0.884358
H 4.823429 -0.562848 0.000185
H 3.697702 -1.590517 0.884699
C 2.691973 1.422170 -1.569224
H 1.930258 2.195881 -1.649620
H 3.670187 1.904789 -1.630783
H 2.589731 0.759240 -2.428916
C 2.692145 1.422287 1.569107
H 3.670331 1.904997 1.630393
H 1.930356 2.195899 1.649711
H 2.590202 0.759329 2.428813
Si 2.585688 0.438750 -0.000049

IBA_TMS_02.log
Energy (E) = -408.976783717 Hartree
Enthalpy (H) = -408.859007 Hartree
Gibbs free energy (G) = -408.898588 Hartree
Charge = 0, Spin = 2
C 1.773882 0.005390 0.175084
H 2.308745 0.888703 -0.174846
H 1.807530 0.005334 1.268947
H 2.314315 -0.874408 -0.175140
C -0.891611 1.533517 0.175043
H -1.924192 1.554769 -0.174377
H -0.907858 1.563032 1.268919
H -0.400194 2.441432 -0.175621
C -0.882314 -1.538840 0.175091
H -0.898539 -1.568297 1.268958
H -1.914667 -1.566390 -0.174541
H -0.385327 -2.443777 -0.175437
Si 0.000032 -0.000057 -0.422012

IBA_tBu.log
Energy (E) = -588.258880546 Hartree
Enthalpy (H) = -588.028617 Hartree
Gibbs free energy (G) = -588.086812 Hartree
Charge = 0, Spin = 1
C -1.792265 2.800174 0.000166
C -3.072866 2.260017 0.000024
C -3.242301 0.885469 -0.000108
C -2.143106 0.031742 -0.000077
C -0.888510 0.600437 0.000055
C -0.679483 1.967702 0.000178
H -1.649127 3.872080 0.000261
H -3.934101 2.913845 0.000037
H -4.217256 0.416263 -0.000233
H 0.307379 2.398817 0.000256
C -2.360095 -1.478147 -0.000133
O -1.267489 -2.137722 0.000157
O -3.505716 -1.894970 -0.000391
I 0.717099 -0.814255 0.000165
C 2.492255 0.530491 -0.000161
C 3.599555 -0.513625 -0.000392
H 3.563200 -1.146959 -0.888751
H 4.562841 0.003534 -0.000576
H 3.563584 -1.146973 0.887979
C 2.569041 1.352142 -1.271583
H 1.770667 2.084491 -1.366733
H 3.520164 1.894037 -1.277993
H 2.544452 0.709348 -2.151911
C 2.569533 1.352071 1.271243
H 3.520637 1.893982 1.277312
H 1.771183 2.084428 1.366595
H 2.545208 0.709316 2.151607

IBA_tBu_02.log
Energy (E) = -157.627340209 Hartree
Enthalpy (H) = -157.503651 Hartree
Gibbs free energy (G) = -157.540732 Hartree
Charge = 0, Spin = 2
C -0.000098 0.000000 -0.144858
C -1.477716 0.001891 0.014437
H -1.931263 -0.883516 -0.435502
H -1.776078 0.002272 1.074148
H -1.928998 0.888454 -0.435502
C 0.737216 -1.280515 0.014426
H 1.730458 -1.230702 -0.436257
H 0.886939 -1.538580 1.074140
H 0.194849 -2.114875 -0.434747
C 0.740491 1.278625 0.014426
H 0.890881 1.536301 1.074140
H 1.733599 1.226273 -0.436264
H 0.200257 2.114370 -0.434740

IBA_styryl.log
Energy (E) = -739.322352428 Hartree
Enthalpy (H) = -739.088118 Hartree
Gibbs free energy (G) = -739.152039 Hartree
Charge = 0, Spin = 1
C -1.584068 3.080673 0.091655
C -2.960186 3.118744 -0.108677
C -3.682365 1.941149 -0.223151
C -3.040437 0.710085 -0.132827
C -1.680623 0.716466 0.069876
C -0.920507 1.862297 0.184371
H -1.018757 3.998800 0.174331
H -3.465549 4.072134 -0.179650
H -4.751690 1.925656 -0.386731
H 0.150246 1.825330 0.326933
C -3.820904 -0.587989 -0.281202
O -3.076608 -1.629006 -0.209143
O -5.024674 -0.528437 -0.453474
I -0.808607 -1.221968 0.213245
C 1.136528 -0.455781 0.595338
H 1.321330 -0.109921 1.603574
C 2.076601 -0.498571 -0.346787
H 1.805020 -0.856018 -1.337488
C 3.482631 -0.100983 -0.207721
C 4.036604 0.320581 1.003986
C 4.298314 -0.145510 -1.338631
C 5.367645 0.691396 1.075812
H 3.425562 0.352061 1.896632
C 5.633208 0.226479 -1.268738
H 3.874992 -0.474650 -2.279885
C 6.170522 0.646589 -0.060571
H 5.785336 1.013839 2.019766
H 6.251461 0.186943 -2.154954
H 7.210699 0.935938 0.000027

IBA_styryl_02.log
Energy (E) = -308.664668433 Hartree
Enthalpy (H) = -308.537127 Hartree
Gibbs free energy (G) = -308.576796 Hartree
Charge = 0, Spin = 2
C 3.007987 0.322238 -0.000231
H 3.194060 1.380198 -0.000762
C 2.017067 -0.532269 0.000153
H 2.252744 -1.596534 0.000518
C 0.570687 -0.226419 0.000112
C 0.086390 1.083252 0.000149
C -0.344020 -1.277699 0.000007
C -1.275396 1.330569 0.000043
H 0.787054 1.908917 0.000325
C -1.710625 -1.032082 -0.000112
H 0.025154 -2.296377 0.000005
C -2.180712 0.273168 -0.000093
H -1.636288 2.350471 0.000105

H -2.406613 -1.860226 -0.000208
H -3.244376 0.469005 -0.000154

IBA_SPh.log
Energy (E) = -1060.11106915 Hartree
Enthalpy (H) = -1059.908841 Hartree
Gibbs free energy (G) = -1059.970310 Hartree
Charge = 0, Spin = 1
C 1.787653 2.799638 -1.066380
C 3.003527 2.792504 -0.389759
C 3.416125 1.659358 0.293176
C 2.613930 0.523840 0.305531
C 1.416208 0.573348 -0.369040
C 0.968854 1.674961 -1.064752
H 1.468812 3.682287 -1.603312
H 3.628423 3.674706 -0.400706
H 4.354417 1.609695 0.829529
H 0.024899 1.665762 -1.592542
C 3.042081 -0.727474 1.032063
O 2.181318 -1.692993 0.929641
O 4.096022 -0.760360 1.629249
I 0.324697 -1.253367 -0.245029
S -1.604365 -0.433419 -1.571580
C -2.683989 0.151720 -0.302823
C -3.996952 -0.311863 -0.297715
C -2.278678 1.094593 0.639724
C -4.899736 0.171618 0.639414
H -4.301225 -1.052152 -1.025567
C -3.179826 1.558693 1.585555
H -1.259801 1.462477 0.634891
C -4.493117 1.103004 1.585089
H -5.918637 -0.190861 0.636310
H -2.856777 2.283189 2.320586
H -5.194398 1.471214 2.321023

IBA_SPh_02.log
Energy (E) = -629.488575786 Hartree
Enthalpy (H) = -629.391096 Hartree
Gibbs free energy (G) = -629.428061 Hartree
Charge = 0, Spin = 2
S 2.279720 -0.000009 -0.000244
C 0.576804 -0.000128 0.000310
C -0.143868 -1.212935 0.000284
C -0.143572 1.212783 0.000358
C -1.524703 -1.209268 0.000060
H 0.413805 -2.139412 0.000621
C -1.524515 1.209370 -0.000041
H 0.414156 2.139216 0.000615
C -2.218048 0.000153 -0.000339
H -2.068420 -2.144025 -0.000141
H -2.068066 2.144220 -0.000043
H -3.299583 0.000285 -0.000950

IBA_SOPh.log
Energy (E) = -1135.24705114 Hartree
Enthalpy (H) = -1135.040191 Hartree
Gibbs free energy (G) = -1135.105493 Hartree
Charge = 0, Spin = 1
C 2.185150 2.551718 -1.396033
C 3.377705 2.529429 -0.679899
C 3.662831 1.468763 0.164865
C 2.755877 0.424003 0.307516
C 1.580050 0.486949 -0.402857
C 1.262929 1.519399 -1.265720
H 1.967051 3.371260 -2.066773
H 4.085860 3.338638 -0.791328
H 4.582484 1.404383 0.731087
H 0.347572 1.531016 -1.839268
C 3.072896 -0.756878 1.198912
O 2.161751 -1.672391 1.186427
O 4.111127 -0.773329 1.827998
I 0.304854 -1.179134 -0.073289
C -2.607717 0.401655 -0.175644
C -3.760563 -0.251893 0.228091
C -2.183996 1.583160 0.425096
C -4.502046 0.287987 1.271762
H -4.055814 -1.160704 -0.278526
C -2.930421 2.109215 1.465737
H -1.277668 2.076208 0.091177
C -4.088750 1.461286 1.888162
H -5.404040 -0.208616 1.601572
H -2.612408 3.022404 1.949005
H -4.669160 1.876585 2.700360
S -1.700070 -0.253666 -1.549254
O -2.380839 -1.502675 -1.965036

IBA_SOPh_02.log
Energy (E) = -704.661527340 Hartree
Enthalpy (H) = -704.559167 Hartree
Gibbs free energy (G) = -704.599141 Hartree
Charge = 0, Spin = 2
C -0.174604 -0.186627 -0.000064
C 0.302449 1.120936 -0.000038
C 0.699785 -1.272769 0.000155
C 1.671166 1.336698 -0.000031
H -0.407289 1.936947 -0.000241
C 2.065852 -1.044102 0.000093
H 0.307892 -2.282988 0.000145
C 2.552312 0.259516 -0.000042
H 2.054802 2.347853 -0.000210
H 2.752319 -1.879533 0.000154
H 3.619125 0.435982 0.000078
S -1.889712 -0.514617 -0.000195
O -2.599153 0.799212 0.000345

IBA_SO2Ph.log
Energy (E) = -1210.42214012 Hartree
Enthalpy (H) = -1210.209193 Hartree
Gibbs free energy (G) = -1210.275019 Hartree
Charge = 0, Spin = 1
C -1.341857 2.792058 1.309628
C -2.570381 3.058153 0.713482
C -3.209963 2.082606 -0.034781
C -2.617701 0.836274 -0.203408
C -1.392377 0.620222 0.382581
C -0.728734 1.553555 1.152227
H -0.855290 3.546559 1.912097
H -3.032592 4.026300 0.846963
H -4.173859 2.241480 -0.499483
H 0.204717 1.332872 1.648025
C -3.315419 -0.260315 -0.970645
O -2.662259 -1.382068 -0.971794
O -4.385764 -0.052425 -1.499050
I -0.631788 -1.324615 -0.006821
C 2.519492 0.161473 -0.066485
C 2.826474 -0.287857 -1.344593
C 2.833706 1.441470 0.364421
C 3.462800 0.579753 -2.217348
H 2.584147 -1.302258 -1.632464
C 3.474970 2.299134 -0.519088
H 2.599893 1.739334 1.376913
C 3.782023 1.869796 -1.804028
H 3.715215 0.251136 -3.215535
H 3.738112 3.298687 -0.202897
H 4.279186 2.542779 -2.489012
S 1.699799 -0.952336 1.031990
O 2.213981 -2.288559 0.806397
O 1.695326 -0.366053 2.356106

IBA_SO2Ph_02.log
Energy (E) = -779.815283223 Hartree
Enthalpy (H) = -779.707245 Hartree
Gibbs free energy (G) = -779.749356 Hartree
Charge = 0, Spin = 2
C 0.087540 -0.000012 -0.085634
C 0.753268 -1.216883 -0.054852
C 0.753151 1.216813 -0.054557
C 2.137648 -1.208026 0.031943
H 0.188902 -2.138384 -0.077414
C 2.137610 1.208054 0.032327
H 0.188821 2.138340 -0.076808
C 2.824946 0.000063 0.073346
H 2.679425 -2.142672 0.071053
H 2.679228 2.142781 0.071668
H 3.904337 0.000045 0.139777
S -1.681702 -0.000090 -0.244682
O -2.181178 -1.272574 0.258609
O -2.181129 1.272734 0.257792

IBA_SMe.log
Energy (E) = -868.545594177 Hartree
Enthalpy (H) = -868.399767 Hartree
Gibbs free energy (G) = -868.452653 Hartree
Charge = 0, Spin = 1
C 1.455731 2.831527 -0.246112
C 2.745450 2.351076 -0.041505
C 2.969205 0.991686 0.110711
C 1.902463 0.101190 0.064540
C 0.643112 0.616892 -0.133216
C 0.374149 1.957912 -0.294553
H 1.283813 3.891464 -0.372916
H 3.575717 3.042698 -0.006270
H 3.956131 0.575837 0.264171
H -0.631522 2.314728 -0.471741
C 2.116893 -1.386744 0.209543
O 1.018535 -2.067645 0.112746
O 3.231104 -1.828435 0.391568
I -0.872611 -0.881894 -0.161067
S -2.725110 0.726628 -0.378541
C -2.829230 1.256825 1.343700
H -1.909335 1.749501 1.655786
H -3.649000 1.969433 1.409849
H -3.039422 0.417153 2.001143

IBA_SMe_02.log
Energy (E) = -437.912357324 Hartree
Enthalpy (H) = -437.872520 Hartree
Gibbs free energy (G) = -437.900905 Hartree
Charge = 0, Spin = 2
S -0.685376 -0.000060 -0.001943
C 1.093842 -0.000329 -0.008839
H 1.495505 0.888467 -0.490047
H 1.410338 0.016678 1.037554
H 1.497124 -0.902212 -0.463380

IBA_SePh.log
Energy (E) = -671.328814996 Hartree
Enthalpy (H) = -671.127133 Hartree
Gibbs free energy (G) = -671.190730 Hartree
Charge = 0, Spin = 1
C 1.963102 2.613255 -1.446020

C 3.193709 2.712353 -0.804343
C 3.619690 1.704689 0.045980
C 2.816514 0.590566 0.260967
C 1.603876 0.532740 -0.385448
C 1.143262 1.507899 -1.242622
H 1.632654 3.397503 -2.112948
H 3.818889 3.578050 -0.973259
H 4.569338 1.738635 0.563340
H 0.187280 1.419770 -1.741882
C 3.261479 -0.521339 1.175427
O 2.400601 -1.490430 1.261563
O 4.328280 -0.462325 1.746313
I 0.526758 -1.250895 0.068984
C -2.642141 0.254266 -0.034445
C -3.950048 -0.154799 0.201057
C -2.102149 1.309927 0.694017
C -4.716523 0.494330 1.160473
H -4.362040 -0.984807 -0.357349
C -2.865944 1.941798 1.664538
H -1.087637 1.640816 0.506904
C -4.175653 1.539234 1.896708
H -5.733655 0.172864 1.338635
H -2.438289 2.754804 2.235297
H -4.769836 2.036927 2.650581
Se -1.660616 -0.617193 -1.439595

IBA_SePh_02.log
Energy (E) = -240.697160045 Hartree
Enthalpy (H) = -240.599967 Hartree
Gibbs free energy (G) = -240.638476 Hartree
Charge = 0, Spin = 2
C -0.069435 0.000348 -0.000242
C -0.766984 -1.204603 -0.000453
C -0.767223 1.204765 -0.000411
C -2.154714 -1.198940 0.000048
H -0.231419 -2.144989 -0.001343
C -2.155277 1.198603 0.000006
H -0.232121 2.145415 -0.000915
C -2.855525 -0.000137 0.000478
H -2.688067 -2.140098 0.000196
H -2.688747 2.139699 -0.000233
H -3.936555 -0.000415 0.001101
Se 1.835055 0.000005 0.000137

IBA_SeMe.log
Energy (E) = -479.764776879 Hartree
Enthalpy (H) = -479.619584 Hartree
Gibbs free energy (G) = -479.674524 Hartree
Charge = 0, Spin = 1
C 1.434580 2.932594 -0.239114
C 2.780345 2.623650 -0.067787
C 3.181387 1.304503 0.071772
C 2.237277 0.284493 0.045177
C 0.916971 0.631610 -0.118491
C 0.473707 1.926966 -0.266831
H 1.123594 3.961563 -0.355796
H 3.514538 3.417001 -0.048296
H 4.216999 1.018789 0.200043
H -0.573860 2.154279 -0.417371
C 2.648481 -1.159841 0.179689
O 1.648920 -1.985500 0.111278
O 3.813262 -1.457118 0.330992
I -0.376456 -1.063213 -0.120178
C -2.609945 0.916588 1.610599
H -1.710537 1.492846 1.812962
H -3.485266 1.534507 1.790028
H -2.648077 0.036420 2.243775
Se -2.663514 0.397425 -0.284828

IBA_SeMe_02.log
Energy (E) = -49.1372177554 Hartree
Enthalpy (H) = -49.097904 Hartree
Gibbs free energy (G) = -49.127848 Hartree
Charge = 0, Spin = 2
C 1.511292 -0.000654 -0.007617
H 1.892405 0.881037 -0.512775
H 1.823691 0.036172 1.036720
H 1.895732 -0.911553 -0.454437
Se -0.431752 -0.000051 -0.000700

IBA_Pyrrole_N1.log
Energy (E) = -639.950948609 Hartree
Enthalpy (H) = -639.771057 Hartree
Gibbs free energy (G) = -639.825443 Hartree
Charge = 0, Spin = 1
C -0.696006 3.027486 -0.000127
C -2.088004 3.001832 -0.000025
C -2.768836 1.794550 0.000059
C -2.053520 0.602705 0.000025
C -0.680505 0.672530 -0.000081
C 0.041449 1.847338 -0.000142
H -0.171703 3.972971 -0.000195
H -2.639460 3.931757 0.000004
H -3.848780 1.733323 0.000157
H 1.123269 1.864560 -0.000206
C -2.741262 -0.736559 0.000092
O -1.902041 -1.738038 -0.000038
O -3.947131 -0.821447 0.000242
I 0.219507 -1.241177 -0.000072
N 2.108114 -0.344198 -0.000019
C 3.777298 0.944336 -0.710877
H 4.472420 1.454080 -1.356448
C 2.734425 0.155196 -1.117378
H 2.388206 -0.097603 -2.105844
C 2.734204 0.155118 1.117502
H 2.387773 -0.097745 2.105878
C 3.777215 0.944211 0.711273
H 4.472241 1.453867 1.357018

IBA_Pyrrole_N1_02.log
Energy (E) = -209.322946007 Hartree
Enthalpy (H) = -209.249606 Hartree
Gibbs free energy (G) = -209.281399 Hartree
Charge = 0, Spin = 2
N 1.240230 -0.000046 0.000031
C -0.979334 0.677311 -0.000069
H -1.822883 1.347306 0.000254
C 0.424044 1.059721 0.000053
H 0.814364 2.066951 -0.000264
C 0.423958 -1.059754 0.000053
H 0.814203 -2.067014 -0.000263
C -0.979386 -0.677237 -0.000070
H -1.822987 -1.347166 0.000254

IBA_Pyrrole_C3.log
Energy (E) = -639.960416192 Hartree
Enthalpy (H) = -639.779633 Hartree
Gibbs free energy (G) = -639.835311 Hartree
Charge = 0, Spin = 1
C -1.051234 3.013832 0.010482
C -2.435010 2.870266 0.023352
C -3.005521 1.607239 0.023817
C -2.201797 0.471389 0.010810
C -0.839420 0.657655 0.001003
C -0.227121 1.893972 -0.001163
H -0.605025 3.998978 0.008189
H -3.065634 3.748789 0.032389
H -4.075841 1.449758 0.033630
H 0.849112 1.995807 -0.013197
C -2.821197 -0.919086 0.011443
O -1.946393 -1.854531 -0.001540
O -4.035403 -1.015940 0.023888
I 0.294908 -1.149688 -0.020876
C 2.143646 -0.141375 -0.021486
H 4.763590 1.075090 1.297644
N 4.026194 0.759098 0.688827
C 2.883857 0.140569 1.099923
H 2.679976 -0.048299 2.139489
C 2.870106 0.323943 -1.150632
H 2.570806 0.258049 -2.182677
C 4.029059 0.876520 -0.676574
H 4.851701 1.335808 -1.195479

IBA_Pyrrole_C3_02.log
Energy (E) = -209.288701248 Hartree
Enthalpy (H) = -209.213475 Hartree
Gibbs free energy (G) = -209.245267 Hartree
Charge = 0, Spin = 2
C 0.052603 -1.238406 0.000132
H -1.308518 1.603071 0.002401
N -0.691501 0.808055 -0.000045
C -1.094693 -0.509589 -0.000236
H -2.132278 -0.789293 -0.000536
C 1.181295 -0.401062 0.000268
H 2.221492 -0.674980 0.000200
C 0.673772 0.878597 -0.000355
H 1.181946 1.827582 -0.000605

IBA_Pyrrole_C2.log
Energy (E) = -639.958232797 Hartree
Enthalpy (H) = -639.777595 Hartree
Gibbs free energy (G) = -639.833144 Hartree
Charge = 0, Spin = 1
C -0.964350 3.015401 -0.099733
C -2.349675 2.900752 -0.035495
C -2.946381 1.651021 0.019757
C -2.165000 0.499669 0.014696
C -0.801323 0.658601 -0.044781
C -0.161935 1.879554 -0.105370
H -0.499675 3.990578 -0.149204
H -2.961476 3.792412 -0.032471
H -4.018969 1.517371 0.065766
H 0.915114 1.960292 -0.166878
C -2.804619 -0.879202 0.059850
O -1.943552 -1.830651 0.019942
O -4.016730 -0.961613 0.124306
I 0.289458 -1.177591 -0.037516
H 2.350782 0.179962 2.023516
N 2.730207 0.284646 1.095979
C 2.141929 -0.178975 -0.053522
C 2.953951 0.158681 -1.108240
H 2.759313 -0.061010 -2.144357
C 3.902399 0.910761 0.785405
H 4.524734 1.341450 1.550196
C 4.070895 0.848363 -0.575565
H 4.904381 1.249083 -1.126101

IBA_Pyrrole_C2_02.log
Energy (E) = -209.287822372 Hartree
Enthalpy (H) = -209.212894 Hartree
Gibbs free energy (G) = -209.244825 Hartree

Charge = 0, Spin = 2
H 2.010479 -0.787658 0.000437
N 1.065740 -0.442871 -0.000458
C -0.057101 -1.194569 -0.000050
C -1.174606 -0.422045 0.000399
H -2.199337 -0.746902 0.000444
C 0.683200 0.884040 0.000543
H 1.412869 1.674378 0.000726
C -0.684362 0.923255 -0.000489
H -1.286974 1.816195 -0.000815

IBA_Pyridine_C4.log
Energy (E) = -678.024775885 Hartree
Enthalpy (H) = -677.838088 Hartree
Gibbs free energy (G) = -677.895508 Hartree
Charge = 0, Spin = 1
C -1.049112 3.039596 0.000267
C -2.438276 2.969546 0.000196
C -3.075009 1.738681 0.000049
C -2.331831 0.562418 -0.000028
C -0.961650 0.676287 0.000048
C -0.283882 1.878365 0.000193
H -0.550768 3.999175 0.000379
H -3.021307 3.880135 0.000254
H -4.152169 1.638170 -0.000011
H 0.794941 1.933679 0.000247
C -3.023435 -0.791362 -0.000199
O -2.197947 -1.775578 -0.000236
O -4.238676 -0.829943 -0.000283
I 0.048163 -1.205242 -0.000083
C 2.623559 0.012870 -1.195109
C 3.891064 0.583103 -1.139646
C 3.891080 0.582790 1.139933
C 1.982977 -0.272025 0.000039
H 2.159883 -0.193832 -2.150474
H 4.421051 0.820811 -2.054422
H 4.421083 0.820234 2.054768
N 4.516190 0.865893 0.000179
C 2.623578 0.012533 1.195257
H 2.159921 -0.194442 2.150571

IBA_Pyridine_C4_02.log
Energy (E) = -247.371185563 Hartree
Enthalpy (H) = -247.290119 Hartree
Gibbs free energy (G) = -247.323298 Hartree
Charge = 0, Spin = 2
C -1.209036 0.741553 -0.000051
C -1.142215 -0.654504 -0.000064
C 1.142215 -0.654505 -0.000061
C 0.000000 1.377906 0.000063
H -2.160508 1.254796 0.000149
H -2.056151 -1.238533 0.000196
H 2.056151 -1.238533 0.000195
N -0.000000 -1.334935 0.000046
C 1.209036 0.741553 -0.000053
H 2.160508 1.254795 0.000144

IBA_Pyridine_C3.log
Energy (E) = -678.025498530 Hartree
Enthalpy (H) = -677.838724 Hartree
Gibbs free energy (G) = -677.896120 Hartree
Charge = 0, Spin = 1
C 1.066212 3.038576 -0.009582
C 2.454923 2.961265 -0.000549
C 3.085240 1.727023 0.006535
C 2.336136 0.554551 0.005025
C 0.966586 0.675374 -0.002251
C 0.295423 1.881019 -0.010276
H 0.572994 4.000781 -0.017365
H 3.042652 3.868862 -0.000354
H 4.161847 1.620968 0.012498
H -0.783189 1.940779 -0.019477
C 3.020563 -0.803327 0.008570
O 2.189727 -1.782078 -0.002420
O 4.235924 -0.847415 0.020463
I -0.058510 -1.197592 -0.004297
C -2.627914 0.017206 1.213690
C -3.892712 0.582905 1.163862
C -4.455228 0.838512 -0.081135
C -1.985101 -0.264713 0.016744
H -2.153078 -0.195194 2.163262
H -4.434700 0.821622 2.067783
H -5.441738 1.280496 -0.154424
C -2.631651 0.023067 -1.179293
H -2.152389 -0.189367 -2.129263
N -3.844492 0.567231 -1.232472

IBA_Pyridine_C3_02.log
Energy (E) = -247.368843361 Hartree
Enthalpy (H) = -247.287589 Hartree
Gibbs free energy (G) = -247.320777 Hartree
Charge = 0, Spin = 2
C 1.149638 -0.840673 -0.000061
C 1.221942 0.551566 0.000020
C 0.034988 1.275260 -0.000000
C -0.112506 -1.365990 0.000025
H 2.040784 -1.454610 0.000035
H 2.175042 1.063123 0.000060
H 0.061912 2.358645 0.000045
C -1.260282 -0.614640 0.000044
H -2.251330 -1.050052 -0.000053
N -1.175585 0.721395 -0.000036

IBA_Pyridine_C2.log
Energy (E) = -678.026473576 Hartree
Enthalpy (H) = -677.839882 Hartree
Gibbs free energy (G) = -677.897546 Hartree
Charge = 0, Spin = 1
C 1.027803 3.035889 0.001847
C 2.417100 2.973916 0.030350
C 3.060021 1.746483 0.036567
C 2.324266 0.565548 0.016184
C 0.953460 0.670561 -0.005650
C 0.270402 1.870223 -0.016810
H 0.524191 3.992761 -0.008871
H 2.995034 3.887757 0.044553
H 4.137523 1.651366 0.055572
H -0.808008 1.920261 -0.049127
C 3.027815 -0.783821 0.015096
O 2.214065 -1.773752 -0.020422
O 4.244527 -0.807097 0.045672
I -0.055229 -1.208172 -0.029013
C -2.635805 -0.057072 1.213991
C -3.898122 0.520340 1.189915
C -4.455454 0.855690 -0.034926
C -3.729458 0.599103 -1.190997
C -1.997312 -0.266019 -0.000420
H -2.159573 -0.331865 2.145018
H -4.434329 0.704782 2.110777
H -5.435177 1.306764 -0.099257
H -4.132766 0.845870 -2.165277
N -2.516685 0.045308 -1.175511

IBA_Pyridine_C2_02.log
Energy (E) = -247.379005274 Hartree
Enthalpy (H) = -247.297582 Hartree
Gibbs free energy (G) = -247.330779 Hartree
Charge = 0, Spin = 2
C -1.329456 -0.493701 -0.000242
C -1.019609 0.859188 0.000007
C 0.315941 1.260704 0.000093
C 1.307836 0.296493 -0.000338
C -0.237560 -1.344918 0.000170
H -2.347819 -0.852029 0.000085
H -1.810026 1.599031 0.000278
H 0.579758 2.308638 0.000426
H 2.357684 0.559621 0.000156
N 0.999642 -1.011694 0.000131

IBA_PPh2.log
Energy (E) = -1234.70294177 Hartree
Enthalpy (H) = -1234.404071 Hartree
Gibbs free energy (G) = -1234.478864 Hartree
Charge = 0, Spin = 1
C 3.235414 -1.786995 1.585755
C 4.445682 -1.345209 1.062010
C 4.481623 -0.236080 0.232129
C 3.311512 0.445392 -0.087543
C 2.133681 -0.023476 0.447351
C 2.053075 -1.123893 1.279081
H 3.203683 -2.650234 2.236225
H 5.359750 -1.869087 1.305832
H 5.399981 0.144254 -0.195057
H 1.115408 -1.465791 1.693967
C 3.357320 1.667935 -0.992771
O 2.208780 2.199967 -1.185653
O 4.435503 2.022605 -1.436437
I 0.405146 1.086814 -0.114776
P -1.264757 -0.386621 1.172818
C -2.659304 0.630219 0.603075
C -3.687639 0.159273 -0.212925
C -2.733325 1.937332 1.099246
C -4.758543 0.983458 -0.534458
H -3.661185 -0.854526 -0.588320
C -3.795251 2.761524 0.764365
H -1.954945 2.304892 1.759227
C -4.811700 2.285114 -0.055823
H -5.552563 0.604861 -1.163743
H -3.833369 3.771779 1.147915
H -5.643848 2.925101 -0.314829
C -1.300577 -1.848071 0.097888
C -1.263637 -3.083317 0.744599
C -1.337503 -1.814425 -1.298503
C -1.273274 -4.265708 0.014403
H -1.227722 -3.113762 1.827543
C -1.345748 -2.991832 -2.027287
H -1.359441 -0.862201 -1.814649
C -1.313582 -4.219258 -1.370985
H -1.245127 -5.217246 0.527224
H -1.370758 -2.955646 -3.107785
H -1.314834 -5.136691 -1.943596

IBA_PPh2_02.log
Energy (E) = -804.081012749 Hartree
Enthalpy (H) = -803.886835 Hartree
Gibbs free energy (G) = -803.937290 Hartree
Charge = 0, Spin = 2
P -0.000006 -1.593802 -0.000146
C -1.415933 -0.494343 -0.037372
C -1.438111 0.764342 -0.655383
C -2.611958 -0.987719 0.503560
C -2.606943 1.506071 -0.707863

H -0.535188 1.151684 -1.109342
C -3.778872 -0.241777 0.457132
H -2.613571 -1.967245 0.967523
C -3.779292 1.010093 -0.146627
H -2.607198 2.472706 -1.193921
H -4.688498 -0.636465 0.889548
H -4.689228 1.593320 -0.187497
C 1.416080 -0.494807 0.037396
C 2.612168 -0.987712 -0.503462
C 1.438067 0.764087 0.655491
C 3.779022 -0.241519 -0.456983
H 2.614093 -1.967178 -0.967588
C 2.606593 1.506053 0.707837
H 0.535261 1.151206 1.109840
C 3.779194 1.010340 0.146577
H 4.688723 -0.636186 -0.889255
H 2.606599 2.472788 1.193714
H 4.689006 1.593755 0.187364

IBA_Ph.log
Energy (E) = -661.996636130 Hartree
Enthalpy (H) = -661.797944 Hartree
Gibbs free energy (G) = -661.855427 Hartree
Charge = 0, Spin = 1
C -1.035919 3.038021 -0.000171
C -2.425565 2.977341 -0.000007
C -3.070306 1.750588 0.000092
C -2.335733 0.568820 0.000043
C -0.964594 0.673247 -0.000102
C -0.279296 1.871277 -0.000215
H -0.531242 3.994402 -0.000275
H -3.002671 3.891836 0.000043
H -4.148125 1.657045 0.000203
H 0.800356 1.916232 -0.000344
C -3.038797 -0.780743 0.000104
O -2.223056 -1.769615 0.000009
O -4.255894 -0.804504 0.000221
I 0.048299 -1.206366 -0.000071
C 1.979210 -0.288370 -0.000019
C 2.606034 -0.007902 -1.207162
C 2.605819 -0.007856 1.207233
C 3.871798 0.565103 -1.204356
H 2.111381 -0.228502 -2.144477
C 3.871579 0.565158 1.204643
H 2.111004 -0.228434 2.144468
C 4.501863 0.851539 0.000194
H 4.363856 0.786655 -2.141436
H 4.363457 0.786753 2.141806
H 5.487174 1.297030 0.000266

IBA_Ph_02.log
Energy (E) = -231.338861137 Hartree
Enthalpy (H) = -231.245833 Hartree
Gibbs free energy (G) = -231.279207 Hartree
Charge = 0, Spin = 2
C -0.000001 -1.388175 0.000195
C -1.220075 -0.767812 -0.000158
C 1.220074 -0.767813 -0.000159
C -1.208092 0.628282 -0.000092
H -2.150782 -1.319592 0.000354
C 1.208092 0.628281 -0.000091
H 2.150780 -1.319594 0.000353
C 0.000001 1.316385 0.000059
H -2.143420 1.173235 0.000209
H 2.143421 1.173233 0.000210
H 0.000001 2.397823 0.000350

IBA_OTf.log
Energy (E) = -1391.66685888 Hartree
Enthalpy (H) = -1391.525612 Hartree
Gibbs free energy (G) = -1391.591308 Hartree
Charge = 0, Spin = 1
C 2.342251 2.961082 -0.327357
C 3.653485 2.610420 -0.017333
C 3.987261 1.287418 0.222775
C 2.999283 0.313221 0.150189
C 1.713005 0.699143 -0.158033
C 1.337097 2.001505 -0.404446
H 2.090366 3.995621 -0.513867
H 4.413944 3.376432 0.035719
H 4.993686 0.974955 0.466728
H 0.319283 2.263860 -0.644021
C 3.295348 -1.126256 0.397798
O 2.218291 -1.911851 0.281986
O 4.381970 -1.551703 0.673569
I 0.449406 -0.978813 -0.199664
S -2.561344 -0.055978 -0.739363
O -2.496009 -1.494041 -0.648930
O -3.385568 0.586645 -1.708374
O -1.145508 0.506390 -0.725293
C -3.141540 0.514418 0.907872
F -3.140587 1.836110 0.960014
F -4.360019 0.063675 1.137653
F -2.316821 0.046060 1.841775

IBA_OTf_02.log
Energy (E) = -961.000639530 Hartree
Enthalpy (H) = -960.965898 Hartree
Gibbs free energy (G) = -961.008712 Hartree
Charge = 0, Spin = 2
S 0.855344 0.068918 0.001126
O 1.283948 -0.742591 -1.150659
O 1.218268 1.446990 0.002979
O 1.284382 -0.749284 1.146274
C -0.983342 -0.010674 0.000037
F -1.432932 0.595758 1.081235
F -1.431721 0.599481 -1.079634
F -1.366261 -1.270745 -0.002378

IBA_OtBu.out
Energy (E) = -663.438768773 Hartree
Enthalpy (H) = -663.203263 Hartree
Gibbs free energy (G) = -663.262844 Hartree
Charge = 0, Spin = 1
C -1.551321 2.919534 -0.312084
C -2.862215 2.597069 0.029533
C -3.226290 1.277486 0.248187
C -2.272706 0.272997 0.133420
C -0.983372 0.633052 -0.183323
C -0.581912 1.928897 -0.427304
H -1.278282 3.949347 -0.496964
H -3.601131 3.381724 0.114723
H -4.236554 0.986143 0.502472
H 0.437186 2.150466 -0.708850
C -2.619232 -1.179124 0.321070
O -1.601716 -1.972522 0.116828
O -3.739260 -1.524697 0.619538
I 0.312587 -1.018469 -0.323492
O 1.866857 0.243883 -0.724513
C 2.848797 0.481004 0.288274
C 3.789794 1.494541 -0.345280
H 3.242485 2.405645 -0.590001
H 4.600436 1.743973 0.340542
H 4.211682 1.085942 -1.262713
C 3.597523 -0.807608 0.602332
H 4.427850 -0.615542 1.282513
H 2.940450 -1.535410 1.084849
H 3.991157 -1.240623 -0.317533
C 2.233340 1.060697 1.555250
H 3.016650 1.280777 2.281190
H 1.697898 1.984854 1.337149
H 1.541598 0.356976 2.022396

IBA_OtBu_02.log
Energy (E) = -232.796419425 Hartree
Enthalpy (H) = -232.666759 Hartree
Gibbs free energy (G) = -232.703971 Hartree
Charge = 0, Spin = 2
O -0.000083 -0.282821 1.421899
C 0.000014 0.025533 0.082477
C -0.000593 -1.362805 -0.592047
H -0.889097 -1.922105 -0.305605
H -0.001297 -1.211092 -1.671818
H 0.888102 -1.922376 -0.306744
C 1.264944 0.788444 -0.304285
H 1.288087 0.969719 -1.379685
H 1.293600 1.750104 0.207884
H 2.146282 0.215177 -0.019517
C -1.264287 0.789511 -0.304237
H -1.286601 0.972002 -1.379451
H -2.146156 0.216440 -0.020712
H -1.292729 1.750598 0.209007

IBA_OSO2Me.log
Energy (E) = -1094.05977131 Hartree
Enthalpy (H) = -1093.897876 Hartree
Gibbs free energy (G) = -1093.957210 Hartree
Charge = 0, Spin = 1
C -1.441835 2.994134 -0.206551
C -2.802204 2.776783 -0.004973
C -3.294844 1.489020 0.132884
C -2.417824 0.413822 0.061098
C -1.082189 0.672991 -0.150974
C -0.544996 1.932506 -0.284058
H -1.064060 4.002696 -0.299039
H -3.475801 3.620169 0.051839
H -4.342641 1.279761 0.300975
H 0.513837 2.093467 -0.416335
C -2.880105 -0.995855 0.227824
O -1.888868 -1.880106 0.161683
O -4.028846 -1.299551 0.409035
I 0.012453 -1.122949 -0.219319
S 2.808134 0.377707 0.311203
O 2.914467 -0.735412 1.220757
O 2.526890 1.683207 0.851519
O 1.759924 0.034499 -0.785639
C 4.272006 0.476460 -0.664034
H 4.447964 -0.491344 -1.122772
H 4.134636 1.251460 -1.411736
H 5.079359 0.736511 0.015582

IBA_OSO2Me_02.log
Energy (E) = -663.400909954 Hartree
Enthalpy (H) = -663.345272 Hartree
Gibbs free energy (G) = -663.381294 Hartree
Charge = 0, Spin = 2
S 0.109257 0.073676 0.000462
O 0.687319 -0.689855 -1.132705
O 0.357601 1.482211 0.000652
O 0.687781 -0.691670 1.131222
C -1.624285 -0.204951 -0.000123
H -1.796934 -1.276640 -0.003127

H -2.033069 0.258231 0.893835
H -2.034008 0.263817 -0.890708

IBA_OPh.out
Energy (E) = -737.176832132 Hartree
Enthalpy (H) = -736.973014 Hartree
Gibbs free energy (G) = -737.031710 Hartree
Charge = 0, Spin = 1
C -1.287682 2.940097 -0.795994
C -2.588483 2.964886 -0.298757
C -3.178952 1.808254 0.186398
C -2.461544 0.618211 0.176072
C -1.177923 0.635918 -0.317664
C -0.552819 1.759180 -0.813529
H -0.836739 3.846245 -1.175861
H -3.140769 3.894199 -0.294399
H -4.187190 1.788671 0.577715
H 0.453627 1.716260 -1.203425
C -3.042604 -0.671102 0.678201
O -2.209429 -1.682508 0.573535
O -4.162144 -0.737940 1.123587
I -0.274972 -1.257980 -0.248345
O 1.429126 -0.462246 -1.115890
C 2.454836 -0.033075 -0.331671
C 3.750929 -0.253790 -0.795787
C 2.263288 0.645092 0.870264
C 4.836885 0.205579 -0.068336
H 3.880184 -0.782671 -1.729969
C 3.359421 1.094132 1.596195
H 1.261128 0.832201 1.236348
C 4.649336 0.879712 1.133340
H 5.837718 0.030564 -0.440229
H 3.198082 1.618807 2.528383
H 5.498918 1.231671 1.701463

IBA_OPh_02.log
Energy (E) = -306.561912636 Hartree
Enthalpy (H) = -306.463858 Hartree
Gibbs free energy (G) = -306.499671 Hartree
Charge = 0, Spin = 2
O 2.288792 0.000022 0.000403
C 1.042025 0.000056 -0.000066
C 0.288607 -1.235001 -0.000298
C 0.288496 1.235047 -0.000304
C -1.080401 -1.220681 -0.000040
H 0.857921 -2.154153 -0.000561
C -1.080518 1.220624 -0.000017
H 0.857769 2.154237 -0.000712
C -1.774468 -0.000051 0.000286
H -1.636417 -2.148535 -0.000127
H -1.636571 2.148450 -0.000033
H -2.855487 -0.000133 0.000849

IBA_OMe.out
Energy (E) = -545.601007760 Hartree
Enthalpy (H) = -545.452897 Hartree
Gibbs free energy (G) = -545.503416 Hartree
Charge = 0, Spin = 1
C -1.718960 2.641868 -0.204046
C -2.911050 1.949091 -0.008300
C -2.908577 0.569132 0.125285
C -1.704766 -0.123167 0.072010
C -0.547583 0.597323 -0.108288
C -0.504211 1.966528 -0.259108
H -1.730928 3.716768 -0.320172
H -3.844658 2.492797 0.031245
H -3.815029 -0.004106 0.266118
H 0.430523 2.479214 -0.432722
C -1.638390 -1.621749 0.177847
O -0.420795 -2.085562 0.057906
O -2.633164 -2.287217 0.347147
I 1.166076 -0.619669 -0.158834
O 2.333911 1.046482 -0.315612
C 2.689390 1.600094 0.931915
H 3.294630 2.486341 0.741394
H 1.809525 1.900501 1.513872
H 3.279157 0.906597 1.539032

IBA_OMe_02.log
Energy (E) = -114.960702346 Hartree
Enthalpy (H) = -114.920190 Hartree
Gibbs free energy (G) = -114.947136 Hartree
Charge = 0, Spin = 2
O 0.789129 -0.000055 -0.006941
C -0.572861 -0.000154 -0.013298
H -0.873711 0.005993 1.048695
H -1.000848 0.903240 -0.461337
H -1.001308 -0.907873 -0.452044

IBA_OCF3.out
Energy (E) = -843.261274820 Hartree
Enthalpy (H) = -843.134321 Hartree
Gibbs free energy (G) = -843.190472 Hartree
Charge = 0, Spin = 1
C -1.434959 2.943392 -0.285987
C -2.760103 2.642083 0.017352
C -3.153826 1.328495 0.216418
C -2.212659 0.311462 0.112671
C -0.911922 0.652060 -0.180723
C -0.476421 1.940567 -0.392265
H -1.136900 3.970528 -0.443237
H -3.485234 3.439778 0.095530
H -4.173660 1.054376 0.450456
H 0.551723 2.160542 -0.631800
C -2.575602 -1.126191 0.306120
O -1.543920 -1.945198 0.154047
O -3.690938 -1.491841 0.568440
I 0.302265 -1.061309 -0.287886
O 1.949504 0.207367 -0.777478
C 2.768558 0.494988 0.214670
F 2.143434 1.126699 1.235193
F 3.751597 1.290017 -0.196167
F 3.332577 -0.601116 0.758462

IBA_OCF3_02.log
Energy (E) = -412.594885123 Hartree
Enthalpy (H) = -412.573281 Hartree
Gibbs free energy (G) = -412.606358 Hartree
Charge = 0, Spin = 2
O 0.777434 0.004949 1.150202
C 0.004844 0.000055 0.034075
F -0.776265 -1.067197 -0.022463
F 0.850697 -0.009195 -0.987103
F -0.768713 1.071956 -0.035552

IBA_NTf2.log
Energy (E) = -2257.08121820 Hartree
Enthalpy (H) = -2256.905541 Hartree
Gibbs free energy (G) = -2256.985936 Hartree
Charge = 0, Spin = 1
C 2.766955 -1.825415 2.271648
C 4.123408 -1.663438 2.006760
C 4.540136 -0.864980 0.954628
C 3.591248 -0.229247 0.163129
C 2.259035 -0.418489 0.456251
C 1.799858 -1.196976 1.492886
H 2.445681 -2.446930 3.095316
H 4.853355 -2.162756 2.627726
H 5.583900 -0.709820 0.716922
H 0.753119 -1.330645 1.717127
C 3.986889 0.648658 -0.974869
O 2.942690 1.183337 -1.614259
O 5.123876 0.863345 -1.290985
I 1.071202 0.660502 -0.914291
N -0.851315 -0.109335 0.124560
S -1.280551 -1.675336 -0.164154
S -1.401957 0.769710 1.403771
O -0.368917 -2.128181 -1.173032
O -1.490879 -2.412124 1.040643
O -0.269200 1.417072 1.995091
O -2.374616 0.064020 2.172304
C -2.919282 -1.534734 -1.007607
C -2.310514 2.107741 0.512076
F -2.744712 2.977636 1.399899
F -1.488003 2.714236 -0.337699
F -3.327643 1.603353 -0.159585
F -2.825763 -0.656656 -1.993645
F -3.846465 -1.154652 -0.155239
F -3.218577 -2.721644 -1.497394

IBA_NTf2_02.log
Energy (E) = -1826.41757613 Hartree
Enthalpy (H) = -1826.347550 Hartree
Gibbs free energy (G) = -1826.408787 Hartree
Charge = 0, Spin = 2
N 0.000073 0.180779 -0.000157
S 1.403442 -0.683643 0.240819
S -1.403637 -0.683827 -0.240779
O 1.470983 -0.965042 1.639309
O 1.574394 -1.673117 -0.771487
O -1.574316 -1.673206 0.771576
O -1.471243 -0.965114 -1.639262
C 2.576369 0.700578 -0.122609
C -2.576297 0.700692 0.122633
F -3.795311 0.218692 -0.009885
F -2.383957 1.122184 1.354824
F -2.379759 1.682578 -0.730438
F 2.380516 1.682231 0.730876
F 2.383524 1.122556 -1.354558
F 3.795391 0.218235 0.009096

IBA_NMe2.log
Energy (E) = -564.997732205 Hartree
Enthalpy (H) = -564.808046 Hartree
Gibbs free energy (G) = -564.862699 Hartree
Charge = 0, Spin = 1
C 1.375946 2.858231 0.000041
C 2.691554 2.403678 0.000037
C 2.962141 1.043983 0.000015
C 1.915956 0.128747 0.000015
C 0.628530 0.611325 0.000029
C 0.317945 1.955981 0.000026
H 1.168182 3.919421 0.000048
H 3.504265 3.116831 0.000039
H 3.969771 0.649944 -0.000004
H -0.704705 2.300995 0.000008
C 2.164299 -1.361868 -0.000020
O 1.066732 -2.048734 0.000020
O 3.298663 -1.791960 -0.000022
I -0.861954 -0.895486 -0.000023
N -2.484053 0.356038 -0.000042

C -2.856958 1.044053 -1.217452
H -2.562781 0.462213 -2.089796
H -3.943893 1.163356 -1.245293
H -2.414546 2.044832 -1.308972
C -2.857053 1.043747 1.217507
H -2.563989 0.461136 2.089716
H -2.413807 2.044076 1.309892
H -3.943897 1.163976 1.244688

IBA_NMe2_02.log
Energy (E) = -134.374482477 Hartree
Enthalpy (H) = -134.291470 Hartree
Gibbs free energy (G) = -134.323967 Hartree
Charge = 0, Spin = 2
N 0.000000 -0.640482 -0.000113
C -1.179174 0.170161 -0.000036
H -2.078061 -0.442288 -0.001035
H -1.199612 0.830328 0.878967
H -1.198787 0.832679 -0.877307
C 1.179174 0.170161 -0.000028
H 2.078061 -0.442288 -0.000672
H 1.198953 0.832376 -0.877525
H 1.199446 0.830633 0.878750

IBA_NHTs.log
Energy (E) = -1305.01505673 Hartree
Enthalpy (H) = -1304.755370 Hartree
Gibbs free energy (G) = -1304.828243 Hartree
Charge = 0, Spin = 1
C 3.123030 2.736510 0.093639
C 4.394471 2.282380 0.431516
C 4.656852 0.923598 0.501738
C 3.642615 0.012710 0.231615
C 2.400319 0.502091 -0.100218
C 2.093130 1.841737 -0.178986
H 2.920012 3.797178 0.045916
H 5.179042 2.995509 0.642723
H 5.629708 0.529307 0.762857
H 1.106067 2.200777 -0.431609
C 3.876504 -1.469839 0.296516
O 2.803050 -2.165821 0.014856
O 4.956617 -1.930289 0.580258
I 1.016077 -1.053820 -0.469916
N -0.533201 0.289696 -0.975641
S -1.341352 1.181654 0.132112
O -0.718274 0.889623 1.398677
O -1.419613 2.534400 -0.365641
H -0.571539 0.679032 -1.911026
C -2.973537 0.524321 0.162737
C -3.206474 -0.653692 0.862861
C -3.993210 1.179323 -0.505649
C -4.483881 -1.182078 0.878345
H -2.394933 -1.128136 1.399295
C -5.270960 0.635064 -0.478017
H -3.783664 2.105264 -1.022684
C -5.532550 -0.546872 0.207587
H -4.680827 -2.096688 1.423962
H -6.079168 1.139493 -0.992361
C -6.915520 -1.130124 0.245694
H -7.602512 -0.559735 -0.376616
H -6.913384 -2.162540 -0.104844
H -7.305814 -1.135398 1.264156

IBA_NHTs_02.log
Energy (E) = -874.364299389 Hartree
Enthalpy (H) = -874.210967 Hartree
Gibbs free energy (G) = -874.263378 Hartree
Charge = 0, Spin = 2
N -2.397883 0.074032 1.484512
S -1.914056 -0.026174 -0.093460
O -2.353704 -1.295531 -0.595618
O -2.384080 1.212105 -0.667328
H -1.682368 0.594713 2.005652
C -0.155677 -0.012297 -0.066906
C 0.531459 -1.219017 -0.012274
C 0.514706 1.202628 -0.089081
C 1.914817 -1.197941 0.030569
H -0.021843 -2.147956 -0.019694
C 1.900712 1.203143 -0.046772
H -0.048752 2.123256 -0.161544
C 2.616440 0.008862 0.015286
H 2.464730 -2.129998 0.070294
H 2.438251 2.142546 -0.070263
C 4.117311 0.010217 0.043187
H 4.510162 1.016288 0.176985
H 4.493776 -0.616809 0.851305
H 4.519783 -0.387627 -0.889447

IBA_Me.log
Energy (E) = -470.434143308 Hartree
Enthalpy (H) = -470.292314 Hartree
Gibbs free energy (G) = -470.340822 Hartree
Charge = 0, Spin = 1
C 2.045371 -2.404981 -0.000040
C 3.093269 -1.491088 0.000021
C 2.830137 -0.130439 0.000047
C 1.519229 0.334881 0.000019
C 0.509456 -0.599511 -0.000022
C 0.726745 -1.963733 -0.000059
H 2.246457 -3.467405 -0.000078
H 4.114836 -1.845386 0.000048
H 3.613332 0.615906 0.000080
H -0.079862 -2.680713 -0.000117
C 1.231097 1.830093 0.000002
O -0.021356 2.096722 -0.000060
O 2.171329 2.603431 0.000031
I -1.445568 0.236481 -0.000005
C -2.408657 -1.704020 0.000078
H -2.143250 -2.250556 -0.898411
H -2.143150 -2.250512 0.898564
H -3.472936 -1.483261 0.000137

IBA_Me_02.log
Energy (E) = -39.7916839996 Hartree
Enthalpy (H) = -39.757680 Hartree
Gibbs free energy (G) = -39.781427 Hartree
Charge = 0, Spin = 2
C 0.000000 -0.000099 0.000261
H -0.931375 -0.537335 -0.000521
H 0.931396 -0.537298 -0.000521
H -0.000021 1.075227 -0.000521

IBA_Indole_N1.log
Energy (E) = -793.458784858 Hartree
Enthalpy (H) = -793.229221 Hartree
Gibbs free energy (G) = -793.290009 Hartree
Charge = 0, Spin = 1
C -0.841709 2.993730 -0.574541
C -2.178596 3.232881 -0.269181
C -3.019282 2.185613 0.073997
C -2.520688 0.888828 0.114622
C -1.194481 0.695618 -0.192527
C -0.320914 1.703813 -0.540486
H -0.191108 3.814789 -0.841495
H -2.561465 4.243415 -0.301378
H -4.063578 2.330749 0.315701
H 0.716943 1.516947 -0.778550
C -3.391244 -0.285578 0.475320
O -2.746134 -1.421222 0.450983
O -4.559606 -0.137961 0.748536
I -0.629181 -1.340181 -0.069347
C 3.320153 0.210251 -0.617139
C 2.281067 -0.286970 0.197191
C 2.309068 -0.171733 1.586237
C 3.410415 0.437136 2.154809
C 4.463024 0.923617 1.362291
C 4.428479 0.816651 -0.012800
H 1.500334 -0.547498 2.200425
H 3.464198 0.541056 3.229854
H 5.311530 1.391320 1.842855
H 5.242398 1.196154 -0.616654
N 1.321811 -0.847908 -0.629070
C 2.940571 -0.046459 -1.976822
H 3.504747 0.190184 -2.863240
C 1.731199 -0.667566 -1.939825
H 1.113499 -1.017684 -2.751661

IBA_Indole_N1_02.log
Energy (E) = -362.834322432 Hartree
Enthalpy (H) = -362.710810 Hartree
Gibbs free energy (G) = -362.748751 Hartree
Charge = 0, Spin = 2
C 0.260637 0.737570 -0.000251
C 0.293568 -0.680645 0.000002
C -0.863262 -1.418691 0.000189
C -2.085384 -0.724126 0.000069
C -2.131180 0.664756 -0.000006
C -0.955800 1.415626 -0.000101
H -0.829744 -2.499295 0.000217
H -3.010367 -1.284812 0.000172
H -3.088304 1.167572 0.000008
H -0.994551 2.497066 -0.000081
N 1.621227 -1.156486 -0.000357
C 1.622319 1.134215 0.000173
H 2.017756 2.137082 0.000450
C 2.376108 -0.088730 0.000116
H 3.454586 -0.162065 0.000585

IBA_Indole_C3.log
Energy (E) = -793.468212727 Hartree
Enthalpy (H) = -793.237815 Hartree
Gibbs free energy (G) = -793.299684 Hartree
Charge = 0, Spin = 1
C -1.189350 2.916543 0.908829
C -2.544591 3.081221 0.641017
C -3.301923 2.012524 0.187883
C -2.714814 0.765998 -0.005884
C -1.373720 0.645647 0.273433
C -0.580304 1.680134 0.724738
H -0.597331 3.750129 1.261054
H -3.006814 4.047815 0.787888
H -4.358193 2.096272 -0.030650
H 0.471854 1.543786 0.930988
C -3.538932 -0.414251 -0.499099
O -2.849885 -1.488367 -0.619101
O -4.723523 -0.245409 -0.725208
I -0.577024 -1.303044 -0.073805
C 2.346456 -0.054806 -0.259312
C 3.518571 -0.004242 0.520731
C 4.679050 0.622906 0.071535
C 4.638725 1.207115 -1.178857
C 3.475697 1.174857 -1.966807

C 2.329243 0.552264 -1.519227
C 1.368173 -0.758541 0.517700
H 5.575317 0.654036 0.676570
H 5.520467 1.703419 -1.560229
H 3.484392 1.648589 -2.938547
H 1.431976 0.530264 -2.125691
H 3.906136 -0.782825 2.462904
N 3.252439 -0.653932 1.708350
C 1.958749 -1.101854 1.700324
H 1.548490 -1.635511 2.541400

IBA_Indole_C3_02.log
Energy (E) = -362.795060260 Hartree
Enthalpy (H) = -362.670191 Hartree
Gibbs free energy (G) = -362.708375 Hartree
Charge = 0, Spin = 2
C -0.228788 -0.807056 0.000096
C -0.318321 0.609114 -0.000142
C 0.820385 1.414304 -0.000105
C 2.050147 0.788371 0.000009
C 2.156214 -0.613081 0.000002
C 1.033498 -1.413023 0.000027
C -1.577421 -1.247582 0.000333
H 0.741762 2.493967 -0.000121
H 2.950761 1.387316 0.000032
H 3.137637 -1.067416 0.000035
H 1.119455 -2.490994 -0.000016
H -2.002621 1.898405 0.003512
N -1.648167 0.956960 -0.000342
C -2.421337 -0.193557 -0.000315
H -3.496087 -0.144939 -0.000472

IBA_Indole_C2.log
Energy (E) = -793.463287595 Hartree
Enthalpy (H) = -793.233205 Hartree
Gibbs free energy (G) = -793.295083 Hartree
Charge = 0, Spin = 1
C -1.227953 3.052441 -0.235629
C -2.606480 3.216496 -0.140904
C -3.435816 2.112591 -0.020681
C -2.896500 0.830273 0.010341
C -1.530249 0.714294 -0.080902
C -0.664812 1.781200 -0.206247
H -0.581697 3.913544 -0.335975
H -3.030602 4.210873 -0.165609
H -4.512178 2.195517 0.049856
H 0.404800 1.646732 -0.291121
C -3.793346 -0.391793 0.122984
O -3.137810 -1.496829 0.103668
O -4.995488 -0.232685 0.213886
I -0.830994 -1.302139 -0.012049
C 3.282863 -0.151676 -0.708347
C 3.154343 0.056604 0.681304
C 4.211289 0.520205 1.465719
C 5.406588 0.775028 0.827311
C 5.556680 0.575770 -0.557348
C 4.511574 0.116465 -1.328219
H 4.099081 0.673436 2.530606
H 6.248381 1.135686 1.402515
H 6.511395 0.787750 -1.018605
H 4.633539 -0.036157 -2.392206
H 1.479783 -0.239616 1.961176
N 1.867089 -0.278073 1.032278
C 1.189322 -0.686958 -0.096451
C 2.015450 -0.623483 -1.179041
H 1.749200 -0.876491 -2.191727

IBA_Indole_C2_02.log
Energy (E) = -362.796142413 Hartree
Enthalpy (H) = -362.671647 Hartree
Gibbs free energy (G) = -362.710044 Hartree
Charge = 0, Spin = 2
C 0.311205 0.739437 0.000135
C 0.298343 -0.672825 -0.000295
C -0.881914 -1.409411 -0.000296
C -2.071345 -0.704948 -0.000025
C -2.083729 0.697870 0.000148
C -0.908836 1.424279 0.000236
H -0.870784 -2.491439 -0.000403
H -3.008727 -1.244421 0.000049
H -3.032914 1.216586 0.000223
H -0.929433 2.506118 0.000246
H 1.940987 -2.049093 0.003932
N 1.618842 -1.096664 -0.000324
C 2.395608 0.017671 0.000145
C 1.691015 1.168436 -0.000193
H 2.066896 2.175848 -0.000899

IBA_I.out
Energy (E) = -441.965163060 Hartree
Enthalpy (H) = -441.859920 Hartree
Gibbs free energy (G) = -441.910524 Hartree
Charge = 0, Spin = 1
C -1.575276 2.979141 -0.000023
C -2.937985 2.698208 0.000066
C -3.380272 1.385240 0.000097
C -2.455940 0.347814 0.000039
C -1.117493 0.667947 -0.000053
C -0.632853 1.955072 -0.000084
H -1.233083 4.004585 -0.000047
H -3.652503 3.509208 0.000113
H -4.429652 1.121805 0.000170
H 0.426400 2.170304 -0.000155
C -2.894971 -1.082783 0.000087
O -1.894099 -1.939655 -0.000051
O -4.056805 -1.403272 0.000055
I 0.080065 -1.088904 -0.000142
I 2.683422 0.376332 0.000125

IBA_I_02.log
Energy (E) = -11.3362262903 Hartree
Enthalpy (H) = -11.333866 Hartree
Gibbs free energy (G) = -11.353729 Hartree
Charge = 0, Spin = 2
I 0.000000 0.000000 0.000000

IBA_Et.log
Energy (E) = -509.708499669 Hartree
Enthalpy (H) = -509.536516 Hartree
Gibbs free energy (G) = -509.588857 Hartree
Charge = 0, Spin = 1
C -1.902666 2.652280 -0.000194
C -3.082265 1.916018 0.000109
C -3.038005 0.530924 0.000281
C -1.817491 -0.136919 0.000151
C -0.672121 0.624994 -0.000096
C -0.671115 2.007244 -0.000280
H -1.932859 3.733190 -0.000356
H -4.034830 2.427709 0.000192
H -3.929585 -0.081842 0.000483
H 0.239507 2.586511 -0.000499
C -1.772552 -1.660052 0.000118
O -0.580830 -2.125158 -0.000354
O -2.826834 -2.270217 0.000470
I 1.130702 -0.502034 -0.000167
C 2.424266 1.251214 0.000164
H 2.191697 1.828085 -0.890637
H 2.191053 1.828081 0.890800
C 3.853369 0.744988 0.000673
H 4.065299 0.141013 -0.882098
H 4.550595 1.582755 0.000844
H 4.064692 0.141134 0.883671

IBA_Et_02.log
Energy (E) = -79.0691492890 Hartree
Enthalpy (H) = -79.004730 Hartree
Gibbs free energy (G) = -79.033991 Hartree
Charge = 0, Spin = 2
C 0.790591 0.000000 -0.015294
H 1.345511 -0.922392 0.034804
H 1.345514 0.922390 0.034804
C -0.690503 0.000000 -0.001912
H -1.099784 -0.883383 -0.493318
H -1.091989 -0.000031 1.020213
H -1.099783 0.883413 -0.493265

IBA_CF2H.log
Energy (E) = -668.830131158 Hartree
Enthalpy (H) = -668.701353 Hartree
Gibbs free energy (G) = -668.754226 Hartree
Charge = 0, Spin = 1
C -1.686310 2.768974 -0.104907
C -2.946540 2.180958 -0.096703
C -3.071368 0.802020 -0.046433
C -1.938554 -0.004619 -0.013946
C -0.710027 0.612508 -0.034628
C -0.539563 1.983666 -0.072674
H -1.586102 3.845175 -0.129919
H -3.829627 2.804147 -0.121168
H -4.030821 0.302789 -0.027277
H 0.430365 2.455220 -0.048204
C -2.069755 -1.515644 0.070002
O -0.934287 -2.116984 0.135510
O -3.178251 -2.012388 0.076647
I 0.914714 -0.760679 -0.016763
C 2.405139 0.856436 -0.250823
H 2.208766 1.485884 -1.118045
F 2.494664 1.613645 0.853132
F 3.568641 0.203240 -0.415744

IBA_CF2H_02.log
Energy (E) = -238.202512843 Hartree
Enthalpy (H) = -238.179047 Hartree
Gibbs free energy (G) = -238.208101 Hartree
Charge = 0, Spin = 2
C 0.000262 0.481492 -0.143904
H 0.000091 1.433808 0.372222
F -1.091129 -0.240088 0.027277
F 1.090944 -0.240219 0.027301

IBA_CF2CF3.log
Energy (E) = -1005.73007651 Hartree
Enthalpy (H) = -1005.593318 Hartree
Gibbs free energy (G) = -1005.654762 Hartree
Charge = 0, Spin = 1
C -2.301677 2.799071 -0.000255
C -3.581877 2.256509 0.000087
C -3.753941 0.882467 0.000303
C -2.648306 0.038577 0.000275
C -1.399193 0.614878 0.000100

C -1.179399 1.977968 -0.000251
H -2.163556 3.871292 -0.000562
H -4.442718 2.910377 0.000158
H -4.729302 0.414697 0.000369
H -0.196382 2.416694 -0.000460
C -2.833566 -1.463887 -0.000021
O -1.713915 -2.111214 0.000483
O -3.950441 -1.934162 -0.000950
I 0.150037 -0.858210 0.000131
C 1.759615 0.709660 0.000135
F 1.698603 1.490494 -1.089334
F 1.698767 1.490355 1.089899
C 3.128216 0.011649 -0.000001
F 3.233430 -0.756770 1.079875
F 3.232317 -0.756877 -1.080356
F 4.109725 0.896545 -0.000636

IBA_CF2CF3_02.log
Energy (E) = -575.103651694 Hartree
Enthalpy (H) = -575.071461 Hartree
Gibbs free energy (G) = -575.110313 Hartree
Charge = 0, Spin = 2
C 0.865730 -0.000000 -0.353441
F 1.499322 -1.091147 0.002017
F 1.499329 1.091144 0.002013
C -0.607737 0.000004 -0.019743
F -1.179629 1.083433 -0.527559
F -1.179629 -1.083424 -0.527578
F -0.811388 -0.000009 1.299897

IBA_02.log
Energy (E) = -430.577331671 Hartree
Enthalpy (H) = -430.475763 Hartree
Gibbs free energy (G) = -430.521373 Hartree
Charge = 0, Spin = 2
C -2.295932 -2.095843 0.000060
C -3.160185 -1.006494 0.000036
C -2.645190 0.278274 -0.000021
C -1.268854 0.486172 0.000000
C -0.423663 -0.611519 0.000008
C -0.920565 -1.904702 0.000037
H -2.689865 -3.102908 0.000096
H -4.229627 -1.162302 0.000016
H -3.281240 1.153631 -0.000024
H -0.249021 -2.751496 -0.000026
C -0.782872 1.896162 -0.000005
O 0.502719 2.110769 0.000431
O -1.533962 2.853449 -0.000268
I 1.654401 -0.303830 -0.000039

IBCCF32O_vinyl.log
Energy (E) = -1108.27558952 Hartree
Enthalpy (H) = -1108.093283 Hartree
Gibbs free energy (G) = -1108.159188 Hartree
Charge = 0, Spin = 1
C -0.968981 3.378782 -0.208045
C 0.417320 3.429974 -0.233690
C 1.164945 2.264943 -0.146578
C 0.537286 1.026015 -0.030274
C -0.840609 1.012322 -0.027876
C -1.613432 2.152621 -0.109806
H -1.553475 4.286539 -0.262800
H 0.922041 4.382338 -0.317036
H 2.242796 2.313913 -0.168811
H -2.692363 2.102271 -0.079798
O 0.442455 -1.307199 0.464249
I -1.751229 -0.907406 0.069832
C -3.694278 -0.097422 -0.327162
H -3.866498 0.283639 -1.325626
C -4.660706 -0.191986 0.571401
H -4.481940 -0.577371 1.568386
H -5.675254 0.110873 0.339461
C 1.275291 -0.315206 0.104085
C 2.367543 -0.208091 1.199451
C 1.908292 -0.651526 -1.270554
F 3.390282 0.594839 0.860795
F 1.828560 0.285291 2.316473
F 2.879078 -1.396735 1.481025
F 2.612319 -1.774883 -1.228933
F 0.919415 -0.816573 -2.159665
F 2.706363 0.313100 -1.746874

IBCCF32O_TMS.log
Energy (E) = -1439.43363765 Hartree
Enthalpy (H) = -1439.176807 Hartree
Gibbs free energy (G) = -1439.256266 Hartree
Charge = 0, Spin = 1
C 0.315590 3.389522 0.139189
C -1.059869 3.394056 0.322755
C -1.776072 2.208759 0.266172
C -1.139678 0.990396 0.022780
C 0.228783 1.021129 -0.123573
C 0.970450 2.186590 -0.081449
H 0.879377 4.311749 0.162837
H -1.579774 4.324636 0.503268
H -2.845679 2.222290 0.409739
H 2.039632 2.175743 -0.236074
O -1.114136 -1.301353 -0.652654
I 1.243683 -0.843069 -0.412774
C 4.411782 -1.740430 0.143167
H 4.317495 -2.233067 -0.825958
H 5.477535 -1.651288 0.364368
H 3.966547 -2.386788 0.900365
C 4.367247 0.994756 -1.207636
H 3.971449 2.008550 -1.243157
H 5.446314 1.065356 -1.050272
H 4.201100 0.533396 -2.181456
C 3.639138 0.715008 1.823920
H 4.668512 0.894483 2.142328
H 3.103582 1.662812 1.860585
H 3.175172 0.039404 2.543218
Si 3.625679 -0.054292 0.135916
C -1.879990 -0.361216 -0.109148
C -3.134572 -0.175948 -1.009823
C -2.310829 -0.786913 1.322073
F -4.118471 0.549163 -0.443235
F -2.788567 0.449474 -2.139238
F -3.659306 -1.347187 -1.336049
F -3.037232 -1.897765 1.309499
F -1.205749 -1.025601 2.044081
F -3.010091 0.146588 1.986621

IBCCF32O_tBu.log
Energy (E) = -1188.05720262 Hartree
Enthalpy (H) = -1187.792630 Hartree
Gibbs free energy (G) = -1187.865544 Hartree
Charge = 0, Spin = 1
C -0.384826 3.459838 0.179480
C 0.995569 3.420503 0.305170
C 1.666082 2.209479 0.234699
C 0.972622 1.016889 0.035046
C -0.403383 1.085012 -0.060716
C -1.094699 2.280548 0.000658
H -0.915306 4.401054 0.218657
H 1.554278 4.334193 0.452143
H 2.740582 2.183429 0.332843
H -2.166114 2.314019 -0.099337
O 0.803240 -1.275209 -0.584753
I -1.425737 -0.774229 -0.290002
C -3.534265 -0.125725 0.069426
C -4.189431 -1.500137 0.086877
H -3.813382 -2.120157 0.902460
H -5.265445 -1.371892 0.232419
H -4.047357 -2.033490 -0.856032
C -3.705802 0.532115 1.423631
H -3.229750 1.507299 1.498178
H -4.775725 0.670128 1.611752
H -3.305742 -0.100732 2.216456
C -4.102089 0.679276 -1.083612
H -5.177592 0.808279 -0.923017
H -3.666650 1.670694 -1.185959
H -3.967165 0.153958 -2.029654
C 1.649629 -0.355850 -0.100054
C 2.850500 -0.261324 -1.079622
C 2.137767 -0.785932 1.308973
F 2.463627 0.345082 -2.204686
F 3.893274 0.433662 -0.591263
F 3.300438 -1.465377 -1.397730
F 2.805601 -1.932081 1.271586
F 2.918958 0.121079 1.911907
F 1.065597 -0.958934 2.094352

IBCCF32O_styryl.log
Energy (E) = -1339.12210637 Hartree
Enthalpy (H) = -1338.853502 Hartree
Gibbs free energy (G) = -1338.932348 Hartree
Charge = 0, Spin = 1
C 0.112020 3.282690 -0.117872
C 1.490189 3.435769 -0.067380
C 2.318098 2.325033 0.001387
C 1.780516 1.039397 0.025224
C 0.408810 0.925754 -0.042274
C -0.442287 2.009432 -0.112189
H -0.535674 4.147203 -0.161006
H 1.925489 4.425309 -0.078119
H 3.389184 2.452850 0.036249
H -1.514389 1.876576 -0.145384
O 1.838767 -1.316890 0.397985
I -0.365924 -1.056629 -0.078856
C -2.339749 -0.376033 -0.509992
H -2.518404 -0.020606 -1.516652
C -3.307136 -0.505167 0.396103
H -3.045161 -0.862001 1.390026
C -4.733526 -0.207528 0.214054
C -5.275488 0.192791 -1.010199
C -5.582202 -0.326912 1.314870
C -6.626227 0.471068 -1.123203
H -4.638465 0.279869 -1.880728
C -6.936935 -0.048097 1.203843
H -5.168680 -0.640488 2.265802
C -7.461951 0.352703 -0.016265
H -7.033439 0.778005 -2.076929
H -7.580241 -0.145003 2.067605
H -8.517320 0.569562 -0.108960
C 2.609920 -0.250113 0.127112
C 3.646207 -0.122326 1.273694
C 3.323882 -0.465992 -1.232385
F 4.623865 0.763447 1.017037
F 3.029770 0.278923 2.387694
F 4.228035 -1.285531 1.523818
F 4.061589 0.580237 -1.627253
F 4.113577 -1.532005 -1.211635
F 2.389396 -0.662807 -2.172279

IBCCF32O_SPh.log
Energy (E) = -1659.91258701 Hartree
Enthalpy (H) = -1659.675938 Hartree
Gibbs free energy (G) = -1659.752607 Hartree
Charge = 0, Spin = 1
C 0.149206 2.268726 2.617269
C 1.409994 1.782367 2.933145
C 2.080465 0.943770 2.055126
C 1.502289 0.591584 0.837832
C 0.245468 1.089052 0.561988
C -0.452183 1.913697 1.416308
H -0.373640 2.922667 3.300871
H 1.874797 2.053284 3.870724
H 3.053837 0.555657 2.313886
H -1.438596 2.273305 1.155910
O 1.494847 -0.271967 -1.385465
I -0.567847 0.458080 -1.304609
S -2.812785 1.439491 -0.878437
C -3.648703 0.109545 -0.071057
C -4.952262 -0.170394 -0.474448
C -3.078974 -0.615713 0.973108
C -5.681425 -1.160869 0.168258
H -5.385591 0.386836 -1.294523
C -3.807048 -1.616617 1.598954
H -2.067462 -0.404587 1.297652
C -5.110664 -1.889405 1.203219
H -6.693363 -1.370397 -0.150875
H -3.353536 -2.181592 2.401894
H -5.675852 -2.667987 1.696514
C 2.144227 -0.331406 -0.195262
C 2.075477 -1.782479 0.343709
C 3.609306 0.088566 -0.469923
F 4.105415 -0.560542 -1.511355
F 3.653355 1.394255 -0.734160
F 4.422896 -0.141364 0.571584
F 2.635632 -1.927073 1.548888
F 2.652115 -2.643514 -0.482414
F 0.785033 -2.124437 0.463188

IBCCF32O_SOPh.log
Energy (E) = -1735.04869871 Hartree
Enthalpy (H) = -1734.807361 Hartree
Gibbs free energy (G) = -1734.887978 Hartree
Charge = 0, Spin = 1
C -0.315241 0.799177 3.413658
C -1.549738 1.374296 3.142896
C -2.176773 1.155761 1.925114
C -1.572008 0.357688 0.956943
C -0.332770 -0.170687 1.243280
C 0.309950 0.017042 2.451917
H 0.161308 0.950922 4.372050
H -2.034131 1.988552 3.888812
H -3.139730 1.600655 1.725202
H 1.262321 -0.450707 2.659437
O -1.533852 -1.018595 -1.000680
I 0.551896 -1.310734 -0.301881
C 3.469183 0.206322 0.068931
C 4.035795 0.272570 -1.195667
C 3.304774 1.340436 0.856333
C 4.427820 1.508909 -1.688219
H 4.165055 -0.640840 -1.760861
C 3.692140 2.571329 0.349190
H 2.883730 1.263373 1.851067
C 4.251371 2.654954 -0.921428
H 4.871786 1.578816 -2.671636
H 3.566074 3.463484 0.946645
H 4.556552 3.616081 -1.311539
S 2.993066 -1.378005 0.701934
O 3.505340 -2.400493 -0.251474
C -2.187430 0.007903 -0.400551
C -3.654763 -0.457116 -0.217528
C -2.116598 1.261380 -1.307846
F -4.134912 -0.977261 -1.335441
F -3.710132 -1.390018 0.732792
F -4.475240 0.540335 0.146366
F -2.703042 1.057162 -2.479115
F -2.671941 2.344255 -0.751968
F -0.829031 1.547702 -1.532117

IBCCF32O_SO2Ph.log
Energy (E) = -1810.22454978 Hartree
Enthalpy (H) = -1809.977131 Hartree
Gibbs free energy (G) = -1810.057968 Hartree
Charge = 0, Spin = 1
C -0.131330 2.049062 -2.597529
C 1.120704 2.565777 -2.295023
C 1.944182 1.928326 -1.378570
C 1.519926 0.761981 -0.747341
C 0.257384 0.297734 -1.048098
C -0.581777 0.897055 -1.965177
H -0.760849 2.533016 -3.330934
H 1.465873 3.465804 -2.784093
H 2.919662 2.332106 -1.153940
H -1.542313 0.470983 -2.216594
O 1.821145 -1.279956 0.458663
I -0.324451 -1.429292 0.034599
C -3.402930 0.204995 0.248955
C -3.376684 0.252338 1.636892
C -3.845106 1.270793 -0.519937
C -3.805710 1.407856 2.269619
H -3.041430 -0.608962 2.199335
C -4.276390 2.421061 0.126777
H -3.867994 1.180512 -1.597005
C -4.251345 2.488244 1.514128
H -3.797547 1.466284 3.348805
H -4.634433 3.261387 -0.451288
H -4.585427 3.387672 2.012922
S -2.836504 -1.268923 -0.544651
O -3.254608 -2.411996 0.246066
O -3.169515 -1.179862 -1.953274
C 2.356215 -0.049450 0.242529
C 2.419499 0.720975 1.584387
C 3.778950 -0.287993 -0.323650
F 1.180134 0.791679 2.087589
F 3.187526 0.105304 2.471899
F 2.864511 1.974108 1.450438
F 4.441544 -1.170471 0.406551
F 4.514321 0.832862 -0.366259
F 3.689160 -0.759212 -1.566437

IBCCF32O_SMe.log
Energy (E) = -1468.34702720 Hartree
Enthalpy (H) = -1468.166774 Hartree
Gibbs free energy (G) = -1468.234767 Hartree
Charge = 0, Spin = 1
C -0.846370 3.368715 -0.065750
C 0.524589 3.442490 0.136623
C 1.295832 2.289790 0.156896
C 0.704495 1.043177 -0.033877
C -0.661624 1.009329 -0.219845
C -1.457311 2.133117 -0.239959
H -1.446311 4.267612 -0.089330
H 0.999113 4.403279 0.278794
H 2.360675 2.353779 0.321446
H -2.523994 2.051279 -0.402496
O 0.686352 -1.291878 -0.519751
I -1.482806 -0.945483 -0.440852
S -3.825369 -0.168391 -0.282201
C -3.897583 0.100926 1.501103
H -3.208562 0.889242 1.802307
H -4.912459 0.411052 1.741776
H -3.669264 -0.812389 2.044680
C 1.458707 -0.285796 -0.041412
C 2.699711 -0.199902 -0.964744
C 1.876515 -0.618244 1.412794
F 3.236043 -1.394545 -1.157952
F 2.337517 0.289313 -2.150755
F 3.661227 0.597644 -0.474936
F 2.589648 -1.734007 1.478303
F 2.580285 0.355935 1.999037
F 0.764482 -0.794957 2.138549

IBCCF32O_SePh.log
Energy (E) = -1271.13055329 Hartree
Enthalpy (H) = -1270.894435 Hartree
Gibbs free energy (G) = -1270.972935 Hartree
Charge = 0, Spin = 1
C -0.366213 -0.699258 3.377991
C -1.648055 -0.169891 3.425967
C -2.327134 0.133012 2.255304
C -1.735853 -0.105317 1.017181
C -0.457491 -0.624038 1.009141
C 0.247840 -0.925983 2.152626
H 0.163944 -0.936114 4.289661
H -2.122758 0.012667 4.379767
H -3.317822 0.559280 2.298329
H 1.251559 -1.327559 2.097125
O -1.719852 -0.383417 -1.353162
I 0.356498 -0.912291 -0.940550
C 3.544560 0.257717 0.016368
C 4.823627 0.485480 -0.480052
C 2.831849 1.300472 0.599464
C 5.388313 1.750743 -0.388720
H 5.371041 -0.322976 -0.946709
C 3.394435 2.567140 0.671291
H 1.838770 1.134082 0.999059
C 4.674516 2.795336 0.182570
H 6.383621 1.920011 -0.776754
H 2.830235 3.375720 1.115771
H 5.110799 3.782741 0.243754
Se 2.867832 -1.542066 -0.062544
C -2.388369 0.204392 -0.326492
C -3.834602 -0.348334 -0.375796
C -2.375358 1.740036 -0.531247
F -3.833868 -1.625253 0.005127
F -4.329593 -0.282654 -1.601464
F -4.673519 0.315620 0.432913
F -2.968198 2.090787 -1.663481
F -2.959788 2.409943 0.466824
F -1.099888 2.145126 -0.587991

IBCCF32O_SeMe.log
Energy (E) = -1079.56657853 Hartree
Enthalpy (H) = -1079.386956 Hartree
Gibbs free energy (G) = -1079.457131 Hartree
Charge = 0, Spin = 1
C -0.471712 3.376821 -0.093552
C 0.901984 3.445631 0.090446
C 1.666280 2.288638 0.118225
C 1.064549 1.043520 -0.046783
C -0.304240 1.014545 -0.212922
C -1.092621 2.142879 -0.241830
H -1.066738 4.278799 -0.122855
H 1.383978 4.405435 0.212430

H 2.733505 2.348060 0.268071
H -2.162942 2.068289 -0.389391
O 1.031570 -1.302541 -0.488527
I -1.124031 -0.943925 -0.401192
C -3.590542 0.224310 1.721572
H -2.853731 0.999990 1.915932
H -4.569725 0.564005 2.046897
H -3.326044 -0.688119 2.245730
Se -3.714817 -0.111845 -0.211834
C 1.812775 -0.287285 -0.038715
C 2.250660 -0.596540 1.414445
C 3.039110 -0.227047 -0.983729
F 1.149622 -0.754733 2.159694
F 2.959390 -1.714493 1.487160
F 2.968485 0.384386 1.970772
F 3.566706 -1.427888 -1.160610
F 2.659442 0.239518 -2.173009
F 4.010696 0.575632 -0.524672

IBCCF32O_Pyrrole_N1.log
Energy (E) = -1239.75334593 Hartree
Enthalpy (H) = -1239.538945 Hartree
Gibbs free energy (G) = -1239.608899 Hartree
Charge = 0, Spin = 1
C 0.928938 3.183743 0.065825
C -0.438568 3.413103 0.129538
C -1.334940 2.355206 0.095262
C -0.867184 1.047999 -0.014730
C 0.497450 0.861500 -0.071263
C 1.419356 1.886888 -0.029429
H 1.624030 4.010968 0.092822
H -0.812576 4.424118 0.210010
H -2.396155 2.540675 0.158234
H 2.484150 1.701745 -0.072386
O -1.040380 -1.309173 -0.376203
I 1.089379 -1.167904 -0.186613
N 3.094340 -0.576697 -0.027558
C 4.990117 0.448999 -0.584234
H 5.798992 0.858539 -1.165558
C 3.865748 -0.153418 -1.083837
H 3.551366 -0.321396 -2.100653
C 3.714355 -0.214248 1.143723
H 3.265341 -0.434309 2.098151
C 4.893504 0.410455 0.834062
H 5.614642 0.785328 1.540700
C -1.753572 -0.194970 -0.053030
C -2.848937 -0.046049 -1.136028
C -2.373382 -0.404329 1.350103
F -3.749113 0.900391 -0.837697
F -2.278573 0.288726 -2.293415
F -3.506484 -1.181419 -1.313767
F -3.175170 -1.459292 1.381670
F -3.065067 0.653042 1.784252
F -1.376876 -0.615423 2.218506

IBCCF32O_Pyrrole_C3.log
Energy (E) = -1239.75968185 Hartree
Enthalpy (H) = -1239.544539 Hartree
Gibbs free energy (G) = -1239.615631 Hartree
Charge = 0, Spin = 1
C 0.654898 3.279997 0.145850
C -0.723590 3.413827 0.231536
C -1.541125 2.295489 0.172022
C -0.994399 1.022618 0.014046
C 0.378967 0.928280 -0.057451
C 1.220666 2.019671 0.005909
H 1.294754 4.150524 0.188031
H -1.166853 4.393185 0.346966
H -2.611645 2.406256 0.251625
H 2.293326 1.901015 -0.054653
O -1.069656 -1.317659 -0.445987
I 1.181051 -1.039916 -0.257367
C 3.173906 -0.362664 -0.062196
H 5.872354 0.244585 1.502212
N 5.135125 0.118262 0.827921
C 3.872492 -0.305236 1.118268
H 3.563903 -0.527471 2.125041
C 4.054005 0.039231 -1.103946
H 3.823412 0.103536 -2.153809
C 5.257586 0.330534 -0.520473
H 6.185575 0.663222 -0.950904
C -1.819659 -0.270909 -0.066467
C -2.957841 -0.107934 -1.106765
C -2.401745 -0.551093 1.343754
F -3.909718 0.764593 -0.733206
F -2.446213 0.333699 -2.258263
F -3.559402 -1.264475 -1.343146
F -3.167098 -1.635113 1.354819
F -3.123494 0.460570 1.845478
F -1.381201 -0.758910 2.186969

IBCCF32O_Pyrrole_C2.log
Energy (E) = -1239.75848445 Hartree
Enthalpy (H) = -1239.543455 Hartree
Gibbs free energy (G) = -1239.614141 Hartree
Charge = 0, Spin = 1
C 0.732095 3.258060 0.009682
C -0.641057 3.417138 0.134013
C -1.480121 2.313124 0.122792
C -0.958477 1.028477 -0.022138
C 0.409677 0.910357 -0.130504
C 1.274000 1.985671 -0.118204
H 1.386659 4.118465 0.010488
H -1.063556 4.406656 0.239634
H -2.546132 2.444604 0.228431
H 2.342469 1.849005 -0.218685
O -1.077329 -1.313801 -0.462929
I 1.163694 -1.082033 -0.294307
H 3.242473 -0.228653 2.010266
N 3.713621 -0.129939 1.124984
C 3.155063 -0.407371 -0.096898
C 4.101551 -0.147326 -1.058167
H 3.962412 -0.259705 -2.120261
C 4.996824 0.302029 0.953360
H 5.614773 0.565823 1.793816
C 5.269525 0.300251 -0.392127
H 6.203393 0.584109 -0.845853
C -1.801368 -0.254652 -0.059581
C -2.979034 -0.088755 -1.053902
C -2.328746 -0.517902 1.374802
F -3.907801 0.789973 -0.640691
F -2.512889 0.347045 -2.225942
F -3.595599 -1.242475 -1.260877
F -3.003415 0.512060 1.900968
F -3.115079 -1.584775 1.424706
F -1.275727 -0.744848 2.174079

IBCCF32O_Pyridine_C4.log
Energy (E) = -1277.82470846 Hartree
Enthalpy (H) = -1277.603597 Hartree
Gibbs free energy (G) = -1277.676238 Hartree
Charge = 0, Spin = 1
C -0.540215 3.272892 0.058020
C 0.835209 3.421628 0.162102
C 1.664673 2.310862 0.133848
C 1.132369 1.030424 -0.008162
C -0.238675 0.921345 -0.096419
C -1.092809 2.004849 -0.066311
H -1.189477 4.137025 0.074236
H 1.266905 4.407224 0.265994
H 2.733046 2.433321 0.224801
H -2.163339 1.884401 -0.142445
O 1.237792 -1.310936 -0.458599
I -0.989739 -1.069888 -0.260769
C -3.582852 -0.218284 1.205857
C -4.919567 0.155127 1.287803
C -5.149698 0.164908 -0.979679
C -3.027280 -0.397071 -0.051595
H -2.999902 -0.359239 2.106618
H -5.384763 0.305387 2.254956
H -5.798589 0.323521 -1.833088
N -5.692565 0.345822 0.221021
C -3.822261 -0.206155 -1.170438
H -3.431902 -0.335305 -2.171420
C 1.971383 -0.254096 -0.062793
C 3.130631 -0.089686 -1.079078
C 2.522883 -0.524784 1.361009
F 3.209186 0.502105 1.877434
F 1.484205 -0.752783 2.176637
F 3.308117 -1.593053 1.390318
F 4.071065 0.782029 -0.679542
F 3.736019 -1.245602 -1.303278
F 2.643302 0.354271 -2.239594

IBCCF32O_Pyridine_C3.log
Energy (E) = -1277.82537505 Hartree
Enthalpy (H) = -1277.604186 Hartree
Gibbs free energy (G) = -1277.676762 Hartree
Charge = 0, Spin = 1
C -0.529992 3.277608 0.082657
C 0.845848 3.421458 0.187898
C 1.671926 2.308332 0.151670
C 1.135837 1.030618 -0.000028
C -0.235579 0.925918 -0.088807
C -1.086025 2.011960 -0.050535
H -1.176534 4.143691 0.103869
H 1.280556 4.405034 0.298371
H 2.740677 2.427031 0.243365
H -2.156758 1.894208 -0.128672
O 1.233773 -1.306208 -0.472027
I -0.997763 -1.059674 -0.269827
C -3.593961 -0.250077 1.219590
C -4.927755 0.117473 1.305368
C -5.635408 0.322125 0.126375
C -3.027139 -0.392957 -0.039402
H -3.007045 -0.417975 2.114113
H -5.413838 0.242074 2.262567
H -6.679507 0.610148 0.159006
C -3.819118 -0.170248 -1.159377
H -3.403933 -0.277195 -2.156297
N -5.100550 0.182706 -1.084396
C 1.970335 -0.256507 -0.064940
C 3.133722 -0.086145 -1.075654
C 2.517500 -0.541466 1.357822
F 3.735153 -1.242178 -1.310112
F 2.652661 0.372268 -2.233022
F 4.076982 0.777151 -0.663729
F 3.300450 -1.611644 1.379968
F 3.204316 0.479049 1.886382
F 1.476030 -0.774881 2.168917

IBCCF32O_Pyridine_C2.log
Energy (E) = -1277.82610144 Hartree
Enthalpy (H) = -1277.605130 Hartree
Gibbs free energy (G) = -1277.677842

Hartree
Charge = 0, Spin = 1
C -0.555287 3.266243 0.064117
C 0.815956 3.415603 0.214637
C 1.648495 2.307160 0.188623
C 1.123740 1.028738 0.006387
C -0.244227 0.916667 -0.117126
C -1.100474 1.999594 -0.097953
H -1.205831 4.129651 0.069569
H 1.242064 4.400142 0.348197
H 2.713835 2.430194 0.310196
H -2.166341 1.879534 -0.223415
O 1.253276 -1.293766 -0.527860
I -0.996195 -1.065479 -0.334467
C -3.539595 -0.262407 1.230346
C -4.866704 0.116139 1.373629
C -5.623372 0.339981 0.232355
C -5.022491 0.175952 -1.008465
C -3.034878 -0.390153 -0.057241
H -2.910411 -0.449079 2.090022
H -5.301705 0.231634 2.357061
H -6.661104 0.634056 0.297406
H -5.582615 0.338652 -1.921042
N -3.745564 -0.182066 -1.153607
C 1.967786 -0.253201 -0.066355
C 3.164643 -0.046852 -1.031369
C 2.472779 -0.571678 1.365250
F 1.410308 -0.841146 2.137363
F 3.128679 0.442210 1.944789
F 3.269688 -1.632117 1.383776
F 3.775700 -1.194082 -1.284641
F 4.093923 0.801345 -0.558040
F 2.724475 0.451506 -2.188151

IBCCF32O_PPh2.log
Energy (E) = -1834.50302945 Hartree
Enthalpy (H) = -1834.169826 Hartree
Gibbs free energy (G) = -1834.258955
Hartree
Charge = 0, Spin = 1
C 1.359696 2.758601 -2.130968
C 2.643109 2.731174 -1.603625
C 3.060917 1.659620 -0.828679
C 2.201147 0.594122 -0.570460
C 0.925176 0.665650 -1.086945
C 0.481620 1.716534 -1.866228
H 1.036690 3.585108 -2.748384
H 3.326472 3.545381 -1.800936
H 4.060823 1.645769 -0.422124
H -0.516834 1.727653 -2.281242
O 1.705164 -1.660518 0.038092
I -0.381780 -0.933831 -0.585555
P -2.522045 0.379885 -1.210976
C -3.526417 -0.887998 -0.386076
C -4.260160 -0.678822 0.781398
C -3.598133 -2.136070 -1.016471
C -5.040820 -1.699379 1.308353
H -4.231648 0.283988 1.273225
C -4.364981 -3.157675 -0.478776
H -3.056043 -2.299326 -1.942277
C -5.089513 -2.940161 0.687179
H -5.610880 -1.523292 2.210542
H -4.403467 -4.118886 -0.972669
H -5.693296 -3.733328 1.106135
C -2.378516 1.722528 0.003662
C -2.696403 2.999615 -0.459090
C -1.930471 1.571496 1.318921
C -2.579669 4.105599 0.373716
H -3.034094 3.122877 -1.481691
C -1.809519 2.674032 2.148728
H -1.668345 0.589770 1.693904
C -2.133925 3.942574 1.677098
H -2.830377 5.089878 0.002486
H -1.453240 2.546087 3.161645
H -2.032450 4.801661 2.326253
C 2.575621 -0.655411 0.240553
C 3.975714 -1.170467 -0.186095
C 2.584327 -0.258975 1.741061
F 2.972949 -1.262200 2.517494
F 1.335455 0.078211 2.101631
F 3.363387 0.795219 2.013903
F 4.219053 -2.367382 0.326026
F 4.981996 -0.365014 0.196773
F 4.031335 -1.270383 -1.515171

IBCCF32O_Ph.log
Energy (E) = -1261.79611401 Hartree
Enthalpy (H) = -1261.563035 Hartree
Gibbs free energy (G) = -1261.635788
Hartree
Charge = 0, Spin = 1
C 0.546334 3.263850 0.081809
C -0.829245 3.417498 0.176603
C -1.662161 2.309595 0.139233
C -1.133873 1.027485 -0.003867
C 0.237393 0.913153 -0.084900
C 1.094598 1.994132 -0.043837
H 1.198609 4.125631 0.106693
H -1.258262 4.404283 0.281267
H -2.730595 2.435769 0.224260
H 2.165296 1.867189 -0.111182
O -1.248481 -1.313683 -0.452817
I 0.997601 -1.074778 -0.253656
C 3.027307 -0.409268 -0.053614
C 3.794505 -0.190217 -1.190572
C 3.581141 -0.256805 1.211430
C 5.125116 0.189814 -1.059988
H 3.358688 -0.309830 -2.174587
C 4.910643 0.125254 1.338328
H 2.978974 -0.429757 2.094603
C 5.680446 0.348638 0.203164
H 5.725207 0.361641 -1.943135
H 5.344616 0.246336 2.321466
H 6.715781 0.644377 0.303572
C -1.978437 -0.254293 -0.065082
C -3.130271 -0.081918 -1.088901
C -2.543190 -0.519549 1.354980
F -1.513316 -0.751010 2.180558
F -3.334091 -1.584453 1.379811
F -3.230495 0.510440 1.865980
F -4.069458 0.796001 -0.697738
F -2.633010 0.358729 -2.246900
F -3.742425 -1.233835 -1.317863

IBCCF32O_OTf.log
Energy (E) = -1991.47012021 Hartree
Enthalpy (H) = -1991.294198 Hartree
Gibbs free energy (G) = -1991.374887
Hartree
Charge = 0, Spin = 1
C 0.698538 3.589753 0.089792
C 2.047426 3.442089 0.380974
C 2.640435 2.187976 0.369925
C 1.881511 1.065605 0.055576
C 0.540454 1.252482 -0.223537
C -0.083240 2.481608 -0.214768
H 0.242597 4.569440 0.098996
H 2.645225 4.309571 0.621550
H 3.687369 2.077810 0.608063
H -1.134411 2.577032 -0.433279
O 1.510337 -1.217238 -0.579320
I -0.427159 -0.565722 -0.633320
S -3.593099 -0.133447 -0.731307
O -3.275629 -1.508350 -1.029643
O -4.610527 0.561072 -1.448704
O -2.301342 0.668975 -0.647721
C -4.093332 -0.116325 1.036763
F -4.317899 1.124506 1.435870
F -5.186684 -0.837591 1.200011
F -3.112562 -0.634785 1.770824
C 2.412165 -0.356029 0.020597
C 2.660527 -0.850184 1.465751
C 3.693269 -0.454135 -0.842953
F 3.478414 0.138474 -2.014320
F 4.024120 -1.713957 -1.068159
F 4.732535 0.147752 -0.257194
F 3.166841 -2.072981 1.484909
F 3.476306 -0.045415 2.144443
F 1.485486 -0.873577 2.102052

IBCCF32O_OtBu.log
Energy (E) = -1263.24191327 Hartree
Enthalpy (H) = -1262.971948 Hartree
Gibbs free energy (G) = -1263.046203
Hartree
Charge = 0, Spin = 1
C 0.355604 3.439947 -0.057525
C -0.991151 3.447341 0.281795
C -1.718291 2.266276 0.315352
C -1.100214 1.059258 0.000798
C 0.243804 1.087005 -0.305345
C 0.994599 2.242609 -0.353316
H 0.913434 4.365168 -0.095411
H -1.482338 4.380817 0.518302
H -2.763689 2.279565 0.584334
H 2.040405 2.201428 -0.620761
O -1.021823 -1.230826 -0.662588
I 1.084139 -0.794152 -0.736318
O 2.943824 0.058517 -0.748553
C 3.788834 -0.120456 0.391807
C 5.033800 0.687549 0.058251
H 4.767993 1.735924 -0.083002
H 5.764205 0.615242 0.865204
H 5.481671 0.315581 -0.862549
C 4.146131 -1.592591 0.552561
H 4.891673 -1.724210 1.337401
H 3.268902 -2.181123 0.830958
H 4.550614 -1.979269 -0.383224
C 3.147410 0.411480 1.667594
H 3.832952 0.287129 2.506526
H 2.913251 1.471647 1.569767
H 2.229180 -0.128103 1.910613
C -1.786392 -0.304030 -0.020729
C -3.126208 -0.231931 -0.792524
C -2.016372 -0.762040 1.440728
F -4.062455 0.469171 -0.136907
F -2.930025 0.359131 -1.970298
F -3.617369 -1.441097 -1.014719
F -2.662906 -1.917939 1.501623
F -2.692378 0.129958 2.169492
F -0.820751 -0.927421 2.024047

IBCCF32O_OSO2Me.log
Energy (E) = -1693.86305647 Hartree
Enthalpy (H) = -1693.666572 Hartree
Gibbs free energy (G) = -1693.740749
Hartree
Charge = 0, Spin = 1
C 0.288021 3.371000 -0.320738
C -1.095675 3.457545 -0.253953

C -1.871416 2.314668 -0.122803
C -1.259681 1.067252 -0.055686
C 0.117980 1.024600 -0.145625
C 0.921756 2.134584 -0.272647
H 0.887318 4.266156 -0.406548
H -1.578060 4.423721 -0.298081
H -2.947141 2.388236 -0.072318
H 1.997607 2.052453 -0.308514
O -1.121519 -1.273139 0.430851
I 0.855891 -0.943715 -0.070958
S 3.920425 -0.028807 0.291836
O 3.857186 -1.130943 1.218747
O 3.923355 1.312481 0.819716
O 2.774960 -0.173736 -0.747389
C 5.326688 -0.224450 -0.751727
H 5.289062 -1.212483 -1.199451
H 5.304068 0.554852 -1.507192
H 6.201625 -0.119187 -0.115587
C -1.991840 -0.254700 0.105187
C -3.003306 -0.191766 1.275385
C -2.694066 -0.617856 -1.225123
F -2.400589 0.298680 2.354526
F -3.469225 -1.394489 1.570127
F -4.048003 0.593947 0.991726
F -3.509389 0.345404 -1.653355
F -1.752830 -0.797296 -2.158456
F -3.391757 -1.739412 -1.123447

IBCCF32O_OPh.log
Energy (E) = -1336.98012425 Hartree
Enthalpy (H) = -1336.741795 Hartree
Gibbs free energy (G) = -1336.815591 Hartree
Charge = 0, Spin = 1
C 0.309354 3.186810 -1.005013
C -0.988280 3.396210 -0.556775
C -1.787378 2.328293 -0.173984
C -1.292649 1.029598 -0.250316
C 0.001682 0.862158 -0.694679
C 0.826966 1.899392 -1.072168
H 0.925909 4.023428 -1.301653
H -1.382769 4.401000 -0.500471
H -2.789512 2.501137 0.187766
H 1.832963 1.706818 -1.414152
O -1.415841 -1.358130 -0.281513
I 0.643667 -1.138111 -0.724517
O 2.546237 -0.496300 -1.242260
C 3.457329 -0.196067 -0.278525
C 4.800198 -0.414248 -0.586990
C 3.119474 0.347107 0.959735
C 5.786796 -0.088107 0.328700
H 5.043537 -0.838023 -1.551519
C 4.117545 0.663500 1.873589
H 2.082908 0.529584 1.215722
C 5.453011 0.451087 1.566467
H 6.824416 -0.261455 0.075589
H 3.841749 1.082813 2.831968
H 6.224610 0.699822 2.281481
C -2.052515 -0.228397 0.153939
C -3.458980 -0.254431 -0.489064
C -2.139743 -0.276664 1.698838
F -3.349238 -0.037986 -1.798442
F -4.046871 -1.426964 -0.311113
F -4.268691 0.686280 0.014269
F -2.702170 0.813848 2.223832
F -2.814704 -1.334916 2.122799
F -0.893679 -0.358843 2.184469

IBCCF32O_OMe.log
Energy (E) = -1145.40386360 Hartree
Enthalpy (H) = -1145.221304 Hartree
Gibbs free energy (G) = -1145.287033 Hartree
Charge = 0, Spin = 1
C 1.105212 3.339292 -0.008816
C -0.266631 3.443347 0.180113
C -1.068768 2.311054 0.184307
C -0.499838 1.054821 -0.005685
C 0.866885 0.988976 -0.170765
C 1.693036 2.092731 -0.183128
H 1.722856 4.226300 -0.022566
H -0.718750 4.414927 0.321846
H -2.133818 2.400863 0.334618
H 2.755284 1.977696 -0.341694
O -0.486373 -1.283483 -0.498817
I 1.626573 -0.959401 -0.400435
O 3.525736 -0.213128 -0.264907
C 3.978537 -0.070275 1.062364
H 4.993783 0.325399 1.028132
H 3.355274 0.626656 1.636250
H 4.000184 -1.025201 1.596431
C -1.267321 -0.264304 -0.039721
C -2.470301 -0.167952 -1.008519
C -1.733551 -0.605345 1.396316
F -2.053407 0.298355 -2.184962
F -3.023567 -1.354143 -1.207522
F -3.428427 0.655331 -0.560879
F -2.448264 -1.721294 1.431879
F -2.457004 0.367490 1.957463
F -0.647290 -0.785688 2.157469

IBCCF32O_OCF3.log
Energy (E) = -1443.06436692 Hartree
Enthalpy (H) = -1442.902839 Hartree
Gibbs free energy (G) = -1442.974371 Hartree
Charge = 0, Spin = 1
C 0.469768 3.408458 -0.109891
C -0.884520 3.458009 0.191451
C -1.641975 2.296940 0.241284
C -1.044983 1.067440 -0.020554
C 0.305631 1.059942 -0.303919
C 1.089979 2.190461 -0.358703
H 1.053476 4.317021 -0.150336
H -1.357449 4.409048 0.391151
H -2.692199 2.341809 0.486162
H 2.141373 2.128298 -0.590253
O -0.985518 -1.269451 -0.538478
I 1.050557 -0.877661 -0.637776
O 3.013823 -0.040615 -0.784549
C 3.752781 -0.095624 0.305307
F 3.189258 0.557009 1.347407
F 4.946664 0.453518 0.097375
F 3.956612 -1.356651 0.734110
C -1.763274 -0.272826 0.004659
C -3.045218 -0.229610 -0.860947
C -2.082358 -0.652659 1.470408
F -2.748834 0.266133 -2.060507
F -3.553456 -1.439550 -1.026966
F -3.993275 0.542387 -0.318792
F -2.750649 -1.794090 1.546593
F -2.786248 0.286653 2.103136
F -0.925198 -0.802387 2.121538

IBCCF32O_NTf2.log
Energy (E) = -2856.88418804 Hartree
Enthalpy (H) = -2856.673896 Hartree
Gibbs free energy (G) = -2856.769540 Hartree
Charge = 0, Spin = 1
C 1.254296 -1.239721 3.256729
C 2.634626 -1.333739 3.154483
C 3.290039 -0.894252 2.013928
C 2.562742 -0.349073 0.960854
C 1.189183 -0.283930 1.097765
C 0.505430 -0.710698 2.212469
H 0.745840 -1.577933 4.147915
H 3.208320 -1.752701 3.968725
H 4.363491 -0.976249 1.935983
H -0.567069 -0.639014 2.299632
O 2.253075 0.907985 -1.054055
I 0.284373 0.516508 -0.630001
N -1.815997 -0.028558 0.188372
S -2.211974 -1.629472 0.224772
S -2.594636 1.123255 1.069708
O -1.130552 -2.294489 -0.439728
O -2.671568 -2.044775 1.511131
O -1.593466 1.925948 1.706413
O -3.724870 0.605057 1.769273
C -3.647879 -1.752046 -0.932958
C -3.263278 2.174663 -0.293291
F -3.842232 3.229958 0.240249
F -2.268293 2.567174 -1.083090
F -4.137644 1.494595 -1.009640
F -3.338876 -1.151773 -2.071570
F -4.716819 -1.192751 -0.407746
F -3.874131 -3.032248 -1.154018
C 3.168537 0.162596 -0.333438
C 4.343747 1.132570 -0.058989
C 3.615225 -1.036948 -1.203802
F 2.528511 -1.741176 -1.529003
F 4.201241 -0.637514 -2.322680
F 4.450779 -1.848161 -0.557802
F 3.964759 2.041831 0.833978
F 4.711717 1.761089 -1.162844
F 5.411923 0.492975 0.426471

IBCCF32O_NMe2.log
Energy (E) = -1164.79924041 Hartree
Enthalpy (H) = -1164.575186 Hartree
Gibbs free energy (G) = -1164.644911 Hartree
Charge = 0, Spin = 1
C 0.908897 3.319740 0.132193
C -0.473510 3.434237 0.187151
C -1.277278 2.304854 0.128016
C -0.704091 1.041654 0.001647
C 0.671168 0.961551 -0.044133
C 1.495439 2.065802 0.021626
H 1.533021 4.201310 0.176691
H -0.931071 4.409329 0.279017
H -2.350897 2.401854 0.183029
H 2.568559 1.958153 -0.017245
O -0.672088 -1.305970 -0.410917
I 1.489633 -0.988876 -0.182109
N 3.448958 -0.410333 0.012281
C 3.961639 0.001328 1.299699
H 3.383735 -0.452506 2.103695
H 4.999493 -0.329753 1.403131
H 3.949455 1.090483 1.444337
C 4.173283 0.121864 -1.120285
H 3.722946 -0.211820 -2.054134
H 4.214203 1.219944 -1.134913
H 5.205999 -0.239513 -1.097712
C -1.479999 -0.274361 -0.070939
C -2.584427 -0.186739 -1.151814
C -2.088936 -0.557882 1.324782
F -1.084856 -0.707176 2.198131
F -2.807661 -1.672345 1.333578
F -2.864429 0.433536 1.779447

F -3.144064 -1.367762 -1.366251
F -3.566181 0.670814 -0.832500
F -2.046711 0.230793 -2.299119

IBCCF32O_NHTs.log
Energy (E) = -1904.81729405 Hartree
Enthalpy (H) = -1904.523132 Hartree
Gibbs free energy (G) = -1904.611400 Hartree
Charge = 0, Spin = 1
C 1.494144 3.465427 0.142822
C 2.849706 3.250476 0.346360
C 3.368117 1.964087 0.336229
C 2.527031 0.873771 0.132116
C 1.185617 1.128077 -0.070183
C 0.639191 2.392036 -0.075587
H 1.089669 4.467471 0.155205
H 3.510722 4.089298 0.513133
H 4.425211 1.803108 0.482604
H -0.414660 2.562433 -0.237474
O 1.924162 -1.436738 0.116149
I 0.033455 -0.613424 -0.409561
N -1.694782 0.503754 -0.911929
S -2.655705 1.219711 0.198146
O -2.021545 0.988266 1.471943
O -2.938191 2.561102 -0.255892
H -1.792434 0.894853 -1.842294
C -4.165237 0.314570 0.167543
C -4.212854 -0.925964 0.793188
C -5.273281 0.841986 -0.471736
C -5.391897 -1.646721 0.762940
H -3.337734 -1.300445 1.308750
C -6.450860 0.104900 -0.490657
H -5.209123 1.818431 -0.931046
C -6.526014 -1.142598 0.119985
H -5.444522 -2.612570 1.250220
H -7.326967 0.507673 -0.983049
C -7.800815 -1.935756 0.107501
H -8.571419 -1.436334 -0.476733
H -7.639379 -2.927321 -0.316330
H -8.180467 -2.075287 1.120431
C 2.985240 -0.579433 0.091736
C 3.847288 -0.918958 1.331584
C 3.773535 -0.816452 -1.219617
F 4.805909 0.016826 -1.371080
F 2.941656 -0.614496 -2.249322
F 4.232209 -2.057639 -1.301867
F 4.075812 -2.220826 1.414377
F 5.035954 -0.300276 1.317878
F 3.204450 -0.539059 2.433741

IBCCF32O_Me.log
Energy (E) = -1070.23317240 Hartree
Enthalpy (H) = -1070.056986 Hartree
Gibbs free energy (G) = -1070.120518 Hartree
Charge = 0, Spin = 1
C 1.176759 3.355829 0.153665
C -0.207064 3.423733 0.214364
C -0.967640 2.266208 0.148350
C -0.358122 1.020277 0.012193
C 1.019951 0.987410 -0.032562
C 1.804048 2.122570 0.035417
H 1.774360 4.255508 0.198875
H -0.698881 4.381232 0.313627
H -2.043593 2.325668 0.205143
H 2.880660 2.073759 -0.008178
O -0.320084 -1.319742 -0.453504
I 1.900753 -0.941901 -0.178329
C 3.911315 -0.162140 0.101395
H 4.210437 0.452995 -0.740580
H 3.982297 0.376363 1.040647
H 4.530804 -1.055310 0.140992
C -1.122737 -0.309368 -0.081934
C -2.249014 -0.196995 -1.142251
C -1.715514 -0.617122 1.317912
F -3.247702 0.626883 -0.782074
F -1.738364 0.274047 -2.282336
F -2.788482 -1.380223 -1.392280
F -2.487338 0.363053 1.806583
F -2.433479 -1.732429 1.313954
F -0.700992 -0.781767 2.177225

IBCCF32O_Indole_N1.log
Energy (E) = -1393.26145714 Hartree
Enthalpy (H) = -1392.997381 Hartree
Gibbs free energy (G) = -1393.073383 Hartree
Charge = 0, Spin = 1
C 0.490931 2.764532 -1.484358
C -0.822752 3.159799 -1.273159
C -1.754166 2.270777 -0.757234
C -1.378241 0.964761 -0.453978
C -0.063167 0.612449 -0.670095
C 0.891757 1.470860 -1.173320
H 1.213598 3.459713 -1.887463
H -1.126337 4.169944 -1.509591
H -2.771120 2.588881 -0.585562
H 1.914638 1.159058 -1.330650
O -1.734515 -1.336724 0.078647
I 0.401929 -1.377534 -0.112569
C 4.438545 0.026354 -0.414504
C 3.270099 -0.262302 0.321413
C 3.084332 0.192861 1.626254
C 4.104919 0.926864 2.197508
C 5.284840 1.207208 1.488588
C 5.460371 0.767017 0.192810
H 2.174101 -0.019612 2.173315
H 3.994064 1.293582 3.208800
H 6.064489 1.781889 1.969935
H 6.370637 0.991414 -0.347840
N 2.429568 -1.010167 -0.484164
C 4.262450 -0.554432 -1.714623
H 4.963267 -0.538640 -2.532384
C 3.040201 -1.152545 -1.718455
H 2.542270 -1.692495 -2.508272
C -2.308131 -0.102284 0.119459
C -3.609027 -0.196026 -0.713543
C -2.612797 0.251160 1.595765
F -4.332355 -1.246091 -0.357390
F -3.298289 -0.324124 -2.003277
F -4.383877 0.889992 -0.588031
F -3.446401 -0.618105 2.149137
F -3.127825 1.473818 1.749081
F -1.461661 0.213203 2.279886

IBCCF32O_Indole_C3.log
Energy (E) = -1393.26793569 Hartree
Enthalpy (H) = -1393.003157 Hartree
Gibbs free energy (G) = -1393.079947 Hartree
Charge = 0, Spin = 1
C -0.222496 2.535356 -1.987192
C 1.106300 2.892682 -1.808277
C 1.971321 2.055577 -1.119688
C 1.523531 0.839778 -0.605612
C 0.195945 0.522079 -0.796850
C -0.692455 1.335800 -1.468784
H -0.898662 3.185394 -2.524787
H 1.473275 3.829156 -2.204856
H 3.000870 2.345591 -0.975470
H -1.726495 1.048250 -1.595051
O 1.798666 -1.346788 0.307250
I -0.461460 -1.316845 0.062745
C -3.361975 -0.021395 0.315825
C -4.640181 -0.174596 -0.256160
C -5.738949 0.561602 0.182634
C -5.527345 1.463811 1.206555
C -4.257250 1.636814 1.782254
C -3.172195 0.905188 1.346050
C -2.487050 -0.917554 -0.382578
H -6.718070 0.435261 -0.259880
H -6.356880 2.053095 1.572663
H -4.133543 2.357017 2.579037
H -2.191715 1.039293 1.787233
H -5.291082 -1.451902 -1.828908
N -4.534996 -1.125352 -1.250332
C -3.238412 -1.563645 -1.322132
H -2.943079 -2.309598 -2.041287
C 2.404718 -0.156557 0.162943
C 3.730919 -0.396986 -0.603556
C 2.686303 0.445915 1.564428
F 4.411441 -1.404094 -0.076817
F 4.547426 0.670740 -0.613264
F 3.461662 -0.701561 -1.875177
F 3.223205 1.672579 1.529312
F 3.491858 -0.326787 2.281796
F 1.521526 0.547988 2.220928

IBCCF32O_Indole_C2.log
Energy (E) = -1393.26376693 Hartree
Enthalpy (H) = -1392.999264 Hartree
Gibbs free energy (G) = -1393.076082 Hartree
Charge = 0, Spin = 1
C 0.160967 3.147894 -0.338544
C -1.190659 3.408466 -0.161313
C -2.097743 2.366328 -0.042053
C -1.666925 1.042101 -0.105068
C -0.316573 0.824159 -0.267286
C 0.613787 1.835754 -0.386529
H 0.867994 3.959228 -0.440806
H -1.543249 4.429489 -0.117493
H -3.146338 2.577330 0.101925
H 1.664116 1.620863 -0.528493
O -1.957510 -1.313298 -0.357369
I 0.291465 -1.222711 -0.297194
C 4.494072 -0.213087 -0.666311
C 4.267309 -0.018556 0.712960
C 5.278736 0.389160 1.583462
C 6.529451 0.602621 1.043074
C 6.777551 0.416584 -0.329044
C 5.776852 0.011934 -1.185262
H 5.090881 0.531963 2.639140
H 7.338281 0.919541 1.687262
H 7.772855 0.594607 -0.712402
H 5.974532 -0.131049 -2.239240
H 2.488156 -0.265131 1.855182
N 2.944353 -0.303895 0.957955
C 2.337194 -0.667517 -0.224820
C 3.247293 -0.623309 -1.239685
H 3.049363 -0.848768 -2.274456
C -2.590720 -0.179852 -0.002082
C -3.057728 -0.300310 1.471508
C -3.805684 -0.010450 -0.949919
F -3.907260 -1.304407 1.640526
F -3.635588 0.809602 1.947017
F -1.980702 -0.538703 2.234587
F -4.654501 0.955578 -0.561130
F -4.502452 -1.133047 -1.038043

F -3.374799 0.306279 -2.172357

IBCCF32O_I.log
Energy (E) = -1041.76778604 Hartree
Enthalpy (H) = -1041.627964 Hartree
Gibbs free energy (G) = -1041.693719 Hartree
Charge = 0, Spin = 1
C -0.206442 3.407995 0.103657
C 1.177352 3.457183 0.187570
C 1.927802 2.291529 0.141091
C 1.297835 1.058842 -0.000567
C -0.081755 1.051667 -0.070342
C -0.857086 2.186369 -0.021457
H -0.790219 4.316786 0.137610
H 1.679028 4.408713 0.291887
H 3.003564 2.334527 0.217257
H -1.935751 2.134177 -0.075554
O 1.188848 -1.284502 -0.475797
I -0.882661 -0.907807 -0.237265
I -3.739962 -0.053018 0.048951
C 2.021043 -0.277296 -0.058658
C 3.169978 -0.238474 -1.097391
C 2.551921 -0.627860 1.352566
F 3.655204 -1.448709 -1.319413
F 2.706105 0.243475 -2.248128
F 4.183603 0.541446 -0.703548
F 3.235066 -1.762728 1.350482
F 3.330816 0.327634 1.862616
F 1.503396 -0.773809 2.168770

IBCCF32O_Et.log
Energy (E) = -1109.50752905 Hartree
Enthalpy (H) = -1109.301208 Hartree
Gibbs free energy (G) = -1109.368588 Hartree
Charge = 0, Spin = 1
C -0.594995 3.504995 0.174518
C 0.787567 3.422881 0.250582
C 1.420030 2.191107 0.178599
C 0.681503 1.019596 0.021125
C -0.691110 1.134758 -0.038092
C -1.349551 2.347888 0.034949
H -1.092532 4.463504 0.223669
H 1.378262 4.320869 0.365938
H 2.495486 2.133585 0.246928
H -2.424696 2.414563 -0.021965
O 0.401746 -1.295410 -0.489152
I -1.779392 -0.682143 -0.217048
C -3.722379 0.275953 0.074460
H -3.684879 0.832406 1.007083
H -3.901309 0.950223 -0.758500
C -4.734186 -0.852963 0.118905
H -4.535094 -1.535986 0.945338
H -5.741351 -0.458208 0.252353
H -4.730198 -1.431924 -0.805884
C 1.300149 -0.383985 -0.085027
C 2.454627 -0.374610 -1.121336
C 1.831928 -0.775547 1.318831
F 0.789364 -0.861850 2.155735
F 2.682252 0.116580 1.844866
F 2.442254 -1.953527 1.302802
F 3.533242 0.319235 -0.718290
F 2.861132 -1.605040 -1.394116
F 2.028270 0.180976 -2.258028

IBCCF32O_CF2H.log
Energy (E) = -1268.63090618 Hartree
Enthalpy (H) = -1268.467682 Hartree
Gibbs free energy (G) = -1268.535433 Hartree
Charge = 0, Spin = 1
C 0.672494 3.414486 -0.339009
C -0.711683 3.417222 -0.431297
C -1.425294 2.235249 -0.303515
C -0.763318 1.029202 -0.083472
C 0.613038 1.058479 -0.023541
C 1.352551 2.220707 -0.136821
H 1.230304 4.336998 -0.417328
H -1.241120 4.345301 -0.594623
H -2.502205 2.244816 -0.373441
H 2.426587 2.229519 -0.037641
O -0.620686 -1.257783 0.587869
I 1.541765 -0.837802 0.212922
C 3.533523 -0.000631 -0.281554
H 3.540549 0.576717 -1.205637
F 4.045460 0.737239 0.719911
F 4.299373 -1.099232 -0.425363
C -1.465771 -0.322000 0.109613
C -2.615956 -0.179771 1.139724
C -2.015208 -0.780965 -1.265228
F -2.159587 0.429003 2.235134
F -3.087240 -1.364842 1.493050
F -3.651246 0.541452 0.680080
F -2.803624 0.124834 -1.855461
F -2.695934 -1.914458 -1.171011
F -0.974806 -0.989490 -2.083957

IBCCF32O_CF2CF3.log
Energy (E) = -1605.53103270 Hartree
Enthalpy (H) = -1605.359796 Hartree
Gibbs free energy (G) = -1605.436396 Hartree
Charge = 0, Spin = 1
C -0.379531 3.557445 0.185056
C -1.749298 3.398931 0.333825
C -2.319117 2.137337 0.266796
C -1.525380 1.013348 0.049215
C -0.164722 1.207265 -0.066855
C 0.434430 2.450028 -0.013964
H 0.067124 4.540918 0.221691
H -2.379928 4.261481 0.496558
H -3.385479 2.018416 0.381820
H 1.495709 2.584913 -0.132725
O -1.139397 -1.254700 -0.579920
I 0.938029 -0.599034 -0.331283
C 2.897730 0.467392 0.011129
F 3.212116 1.308097 -0.991320
F 2.912804 1.155811 1.163144
C 4.008024 -0.591396 0.086387
F 3.789429 -1.398202 1.118802
F 3.996359 -1.318505 -1.030152
F 5.196134 -0.027443 0.221289
C -2.072122 -0.410719 -0.079214
C -3.270922 -0.441001 -1.061778
C -2.500313 -0.900537 1.327016
F -1.408043 -0.965134 2.098773
F -3.364977 -0.080068 1.932171
F -3.044027 -2.108480 1.282457
F -3.606543 -1.685119 -1.362817
F -4.364892 0.163367 -0.574506
F -2.935076 0.183692 -2.190693

IBCCF32O_02.log
Energy (E) = -1030.37642427 Hartree
Enthalpy (H) = -1030.240158 Hartree
Gibbs free energy (G) = -1030.300393 Hartree
Charge = 0, Spin = 2
C 1.388940 3.295088 0.270089
C 0.003941 3.371877 0.299064
C -0.758292 2.222682 0.169162
C -0.154219 0.978083 -0.000726
C 1.232495 0.921967 -0.016657
C 2.007693 2.064416 0.116591
H 1.990805 4.187557 0.369423
H -0.486859 4.326531 0.424200
H -1.834740 2.291968 0.202817
H 3.085944 1.993568 0.100280
O -0.298117 -1.337554 -0.690197
I 2.222456 -0.919356 -0.202228
C -0.979897 -0.301419 -0.118611
C -2.195607 -0.091713 -1.078297
C -1.472777 -0.719241 1.292039
F -3.178506 0.610476 -0.505803
F -1.805133 0.557911 -2.169105
F -2.705060 -1.256160 -1.449882
F -2.298974 -1.757059 1.223100
F -0.420650 -1.070988 2.029892
F -2.102126 0.268734 1.932574

IBCMe2O_vinyl.log
Energy (E) = -513.009005790 Hartree
Enthalpy (H) = -512.785732 Hartree
Gibbs free energy (G) = -512.841034 Hartree
Charge = 0, Spin = 1
C 0.938875 3.113848 0.023221
C 2.261329 2.689802 -0.002470
C 2.565833 1.335238 -0.000685
C 1.555467 0.376691 0.029910
C 0.259391 0.839900 0.038419
C -0.086307 2.175444 0.035945
H 0.701030 4.168442 0.038081
H 3.060204 3.418906 -0.017214
H 3.599739 1.015933 -0.019380
H -1.120761 2.488372 0.065588
O 0.675529 -1.761218 0.541103
I -1.195872 -0.711936 0.015082
C -2.747731 0.716570 -0.476972
H -2.686835 1.205359 -1.442479
C -3.794142 0.879550 0.317125
H -3.858795 0.393474 1.284663
H -4.634272 1.502995 0.031506
C 1.792776 -1.138987 0.029596
C 2.990709 -1.498768 0.905671
H 3.064444 -2.585351 0.934978
H 3.929084 -1.098517 0.517907
H 2.831672 -1.133886 1.919729
C 2.056416 -1.571681 -1.419713
H 2.934070 -1.075153 -1.839050
H 2.208850 -2.651083 -1.438355
H 1.192856 -1.332814 -2.044463

IBCMe2O_TMS.log
Energy (E) = -844.161841587 Hartree
Enthalpy (H) = -843.864097 Hartree
Gibbs free energy (G) = -843.932964 Hartree
Charge = 0, Spin = 1
C -1.088315 3.122351 -0.206216
C -2.439302 2.902961 0.032905
C -2.929658 1.610242 0.146233
C -2.090159 0.501916 0.022291
C -0.757550 0.765745 -0.184863
C -0.229929 2.035720 -0.309148
H -0.703540 4.126577 -0.318604
H -3.114488 3.743435 0.121959
H -3.984453 1.452373 0.329103
H 0.822122 2.190826 -0.504803

O -1.671211 -1.772487 -0.501964
I 0.510809 -0.963655 -0.275433
C 3.787557 -1.227642 0.426065
H 3.808635 -1.847997 -0.471820
H 4.815621 -0.932785 0.647016
H 3.430243 -1.841936 1.253716
C 3.363657 1.276706 -1.250531
H 2.816329 2.203011 -1.420180
H 4.409638 1.537395 -1.070411
H 3.319042 0.688080 -2.167380
C 2.586359 1.254488 1.768018
H 3.568283 1.631188 2.063593
H 1.909822 2.104097 1.680273
H 2.215559 0.613413 2.568746
Si 2.719632 0.282455 0.187992
C -2.572725 -0.960245 0.114273
C -3.935735 -1.102532 -0.570811
H -4.195215 -2.160548 -0.553602
H -4.729926 -0.543194 -0.071916
H -3.862098 -0.781782 -1.609657
C -2.710870 -1.296965 1.609774
H -3.420369 -0.641477 2.121066
H -3.045454 -2.331018 1.698263
H -1.737760 -1.206879 2.098494

IBCMe2O_tBu.log
Energy (E) = -592.789324998 Hartree
Enthalpy (H) = -592.483798 Hartree
Gibbs free energy (G) = -592.545876 Hartree
Charge = 0, Spin = 1
C 1.239571 3.110371 -0.132654
C 2.562929 2.735123 0.052811
C 2.901815 1.391944 0.129667
C 1.931884 0.397331 0.022979
C 0.624453 0.807658 -0.135425
C 0.253206 2.135795 -0.221234
H 0.969281 4.154150 -0.213677
H 3.333813 3.490166 0.127411
H 3.936282 1.105777 0.267388
H -0.773356 2.425802 -0.372001
O 1.221198 -1.775631 -0.561066
I -0.771260 -0.809939 -0.222609
C -2.655380 0.355692 0.180863
C -3.607261 -0.814151 0.383297
H -3.323587 -1.423134 1.243929
H -4.615730 -0.429366 0.562132
H -3.654948 -1.459007 -0.498347
C -2.571820 1.162885 1.460382
H -1.883016 2.003103 1.398413
H -3.564195 1.564289 1.695473
H -2.261112 0.534305 2.296571
C -3.117962 1.163189 -1.017007
H -4.123067 1.552890 -0.818973
H -2.479141 2.013658 -1.246468
H -3.174586 0.538898 -1.910082
C 2.234719 -1.104800 0.079607
C 3.552684 -1.419855 -0.626602
H 4.418066 -0.990110 -0.118618
H 3.664902 -2.503418 -0.641402
H 3.518993 -1.060474 -1.654557
C 2.331097 -1.509565 1.558782
H 2.524205 -2.581296 1.612812
H 3.128090 -0.971992 2.077339
H 1.386679 -1.302146 2.066653

IBCMe2O_styryl.log
Energy (E) = -743.854973007 Hartree
Enthalpy (H) = -743.545393 Hartree
Gibbs free energy (G) = -743.613514 Hartree
Charge = 0, Spin = 1
C 1.293445 3.171578 0.203567
C 2.681232 3.231774 0.196454
C 3.435266 2.067586 0.137411
C 2.818614 0.819273 0.088803
C 1.442296 0.805984 0.083763
C 0.656142 1.938203 0.137539
H 0.705180 4.076721 0.264158
H 3.178707 4.191174 0.242757
H 4.515995 2.125799 0.131734
H -0.423101 1.871299 0.148371
O 2.728159 -1.520268 0.445314
I 0.609468 -1.147420 -0.063524
C -1.336769 -0.320594 -0.492740
H -1.467540 0.168464 -1.451192
C -2.351003 -0.519013 0.346364
H -2.150341 -1.005953 1.299531
C -3.758957 -0.145398 0.145569
C -4.232587 0.423743 -1.039407
C -4.662203 -0.365372 1.185773
C -5.566889 0.766658 -1.172610
H -3.552873 0.592905 -1.864469
C -6.000619 -0.022582 1.055059
H -4.303389 -0.809510 2.106587
C -6.456864 0.545851 -0.125472
H -5.918815 1.204962 -2.096802
H -6.685248 -0.200280 1.873292
H -7.499080 0.813615 -0.233673
C 3.567463 -0.517248 0.013624
C 4.801212 -0.496582 0.913677
H 5.249240 -1.489243 0.883821
H 5.547100 0.229968 0.586638
H 4.507688 -0.278483 1.939830
C 3.988762 -0.736022 -1.446406
H 4.645034 0.060107 -1.804613
H 4.507664 -1.692110 -1.519495
H 3.105459 -0.771064 -2.088023

IBCMe2O_SPh.log
Energy (E) = -1064.65230997 Hartree
Enthalpy (H) = -1064.374418 Hartree
Gibbs free energy (G) = -1064.440187 Hartree
Charge = 0, Spin = 1
C 1.510402 2.786126 -1.494173
C 2.634090 2.920245 -0.688579
C 3.032784 1.882815 0.144014
C 2.321068 0.687186 0.174117
C 1.212127 0.599825 -0.639935
C 0.773119 1.607289 -1.468906
H 1.203238 3.593292 -2.144283
H 3.203026 3.839786 -0.708101
H 3.903909 1.999319 0.775591
H -0.115593 1.482209 -2.072983
O 2.118248 -1.645553 0.482213
I 0.235237 -1.284391 -0.450238
S -1.866442 -0.472543 -1.644105
C -2.797016 0.137543 -0.275841
C -4.103862 -0.314472 -0.101799
C -2.281314 1.086244 0.607362
C -4.883071 0.177632 0.936176
H -4.499542 -1.056243 -0.782918
C -3.058210 1.562873 1.652940
H -1.272217 1.456183 0.470358
C -4.362704 1.113658 1.820008
H -5.896584 -0.180335 1.058884
H -2.644915 2.294786 2.334094
H -4.967929 1.490758 2.632942
C 2.636840 -0.497533 1.079694
C 1.988527 -0.244927 2.444450
H 2.169026 -1.107221 3.086299
H 2.398455 0.649181 2.917066
H 0.909688 -0.109873 2.335993
C 4.136487 -0.708743 1.237812
H 4.609969 0.122811 1.761649
H 4.291467 -1.615883 1.820334
H 4.601922 -0.835508 0.261634

IBCMe2O_SOPh.log
Energy (E) = -1139.78978022 Hartree
Enthalpy (H) = -1139.507254 Hartree
Gibbs free energy (G) = -1139.576729 Hartree
Charge = 0, Spin = 1
C 1.610870 2.357822 -2.079635
C 2.702456 2.663626 -1.276088
C 3.060916 1.831653 -0.222799
C 2.329534 0.678522 0.042204
C 1.243275 0.419533 -0.763576
C 0.858976 1.217173 -1.821640
H 1.348219 2.997032 -2.911021
H 3.285048 3.551960 -1.478412
H 3.918134 2.075869 0.391113
H 0.016158 0.954323 -2.447228
O 2.094800 -1.553432 0.800824
I 0.217306 -1.344748 -0.205231
C -2.591802 0.375602 -0.150698
C -3.267872 -0.139735 0.946456
C -2.337404 1.737850 -0.269645
C -3.679470 0.725787 1.950308
H -3.461534 -1.203791 0.983620
C -2.744550 2.591884 0.744378
H -1.831741 2.125991 -1.145197
C -3.413840 2.086913 1.854305
H -4.209860 0.337789 2.809421
H -2.549162 3.652577 0.664848
H -3.736036 2.756875 2.639686
S -2.067624 -0.742733 -1.422102
O -2.656444 -2.082801 -1.093379
C 2.609081 -0.308387 1.171709
C 4.103555 -0.500159 1.393827
H 4.576668 0.416012 1.750090
H 4.240427 -1.274387 2.147342
H 4.581899 -0.820903 0.469861
C 1.941839 0.209018 2.448560
H 2.100273 -0.513900 3.248642
H 2.358454 1.172931 2.745231
H 0.867586 0.334240 2.297323

IBCMe2O_SO2Ph.log
Energy (E) = -1214.96607664 Hartree
Enthalpy (H) = -1214.677475 Hartree
Gibbs free energy (G) = -1214.747731 Hartree
Charge = 0, Spin = 1
C 0.879508 2.600949 -1.865821
C 2.038138 3.062526 -1.254528
C 2.740142 2.255724 -0.368217
C 2.290051 0.971822 -0.078178
C 1.125110 0.564187 -0.690071
C 0.400853 1.326198 -1.582356
H 0.349360 3.223262 -2.573050
H 2.404321 4.055124 -1.478499
H 3.646672 2.621774 0.095898
H -0.484350 0.942016 -2.070461
O 2.602916 -1.299120 0.518848
I 0.568508 -1.392539 -0.090228
C -2.601494 0.147128 0.175323
C -2.732836 -0.138896 1.528087

C -2.960684 1.377488 -0.352707
C -3.237765 0.838352 2.371811
H -2.462486 -1.120241 1.895383
C -3.468820 2.347916 0.501024
H -2.861724 1.548997 -1.415687
C -3.601553 2.079105 1.857440
H -3.355561 0.632722 3.426704
H -3.764423 3.310514 0.107216
H -3.996634 2.838244 2.518800
S -1.915867 -1.101650 -0.882970
O -2.399150 -2.394461 -0.414017
O -2.162722 -0.686249 -2.257216
C 2.966244 0.000518 0.884267
C 2.507603 0.321532 2.308814
H 2.956319 -0.396174 2.995410
H 2.803478 1.331134 2.597863
H 1.420785 0.250262 2.388208
C 4.483842 0.076707 0.786911
H 4.859967 1.044485 1.121088
H 4.905492 -0.696818 1.427184
H 4.803359 -0.103206 -0.238154

IBCMe2O_SMe.log
Energy (E) = -873.085629524 Hartree
Enthalpy (H) = -872.864169 Hartree
Gibbs free energy (G) = -872.921400 Hartree
Charge = 0, Spin = 1
C 0.790775 3.128549 -0.345148
C 2.128539 2.861676 -0.082878
C 2.564561 1.555778 0.098067
C 1.670977 0.492026 0.010300
C 0.354001 0.804436 -0.247662
C -0.123552 2.083656 -0.422035
H 0.454061 4.145112 -0.493290
H 2.837241 3.676254 -0.020208
H 3.608072 1.357703 0.306124
H -1.172654 2.259024 -0.623570
O 1.086578 -1.758359 -0.420089
I -0.886415 -0.928354 -0.309945
S -2.999307 0.446701 -0.176894
C -2.879608 0.912198 1.563340
H -1.995373 1.524998 1.741257
H -3.762599 1.495431 1.817005
H -2.845447 0.033192 2.203648
C 2.035626 -0.973595 0.226933
C 3.394111 -1.309502 -0.375981
H 4.205796 -0.783376 0.128618
H 3.556442 -2.380925 -0.265858
H 3.404203 -1.061346 -1.436195
C 2.042832 -1.250128 1.733786
H 2.271990 -2.303214 1.897171
H 2.783286 -0.635128 2.248218
H 1.061668 -1.035863 2.162863

IBCMe2O_SePh.log
Energy (E) = -675.871755137 Hartree
Enthalpy (H) = -675.594400 Hartree
Gibbs free energy (G) = -675.662278 Hartree
Charge = 0, Spin = 1
C 1.698500 1.739091 -2.620158
C 2.846926 2.219850 -2.004468
C 3.258762 1.706471 -0.781661
C 2.535269 0.692495 -0.161722
C 1.399938 0.249828 -0.806370
C 0.948972 0.738135 -2.011372
H 1.380683 2.137207 -3.573559
H 3.425128 3.000681 -2.479377
H 4.150210 2.092064 -0.304220
H 0.039893 0.360594 -2.461841
O 2.329774 -1.197944 1.238741
I 0.421628 -1.288697 0.300366
C -2.716512 0.280803 -0.049226
C -3.995135 0.084300 0.463280
C -2.045497 1.470655 0.223562
C -4.595594 1.066683 1.240277
H -4.513961 -0.843246 0.260109
C -2.642654 2.443348 1.013470
H -1.058575 1.644312 -0.189107
C -3.920458 2.246398 1.522496
H -5.590436 0.903014 1.632662
H -2.110293 3.361634 1.223176
H -4.385651 3.007457 2.133948
Se -1.949454 -1.084765 -1.167802
C 2.863756 0.090964 1.198220
C 4.364428 -0.045719 1.415873
H 4.854321 0.927721 1.464693
H 4.525262 -0.559964 2.362311
H 4.807342 -0.636416 0.615701
C 2.247894 0.981061 2.281212
H 2.436901 0.533520 3.256903
H 2.676600 1.984087 2.254846
H 1.167979 1.067665 2.139208

IBCMe2O_SeMe.log
Energy (E) = -484.306824481 Hartree
Enthalpy (H) = -484.085991 Hartree
Gibbs free energy (G) = -484.145308 Hartree
Charge = 0, Spin = 1
C 0.888225 3.143064 -0.374357
C 2.244654 2.994111 -0.115550
C 2.791218 1.731939 0.075212
C 1.991056 0.595905 0.000019
C 0.650429 0.792448 -0.251396
C 0.065259 2.023757 -0.437919
H 0.465309 4.125667 -0.530263
H 2.881369 3.866744 -0.063201
H 3.848727 1.624870 0.279262
H -0.996577 2.110431 -0.635560
O 1.607697 -1.699602 -0.417375
I -0.423857 -1.050130 -0.297355
C -2.601310 0.747293 1.743023
H -1.734862 1.401631 1.815767
H -3.486526 1.284937 2.070409
H -2.459690 -0.132371 2.363494
Se -2.890061 0.222248 -0.132748
C 2.479515 -0.829000 0.232876
C 2.501342 -1.093338 1.740728
H 2.821203 -2.120803 1.914120
H 3.182716 -0.411366 2.251959
H 1.503384 -0.961785 2.164044
C 3.863333 -1.056860 -0.361970
H 4.623715 -0.458970 0.142473
H 4.115445 -2.109367 -0.239973
H 3.858358 -0.818241 -1.424270

IBCMe2O_Pyrrole_N1.log
Energy (E) = -644.493727221 Hartree
Enthalpy (H) = -644.238198 Hartree
Gibbs free energy (G) = -644.297066 Hartree
Charge = 0, Spin = 1
C -0.168143 3.121312 -0.186405
C -1.548899 3.236933 -0.082047
C -2.348856 2.105378 0.001657
C -1.777050 0.836887 -0.031241
C -0.405781 0.772104 -0.140203
C 0.430969 1.866404 -0.207354
H 0.450581 4.005224 -0.251774
H -2.005499 4.217168 -0.062791
H -3.422530 2.205112 0.094864
H 1.505708 1.763563 -0.281935
O -1.776388 -1.499769 -0.432414
I 0.283256 -1.231289 -0.139143
N 2.276396 -0.471066 0.075487
C 4.113431 0.666623 -0.479415
H 4.917108 1.091845 -1.057264
C 3.071317 -0.079660 -0.971471
H 2.833680 -0.365245 -1.983415
C 2.795422 0.050542 1.231229
H 2.311600 -0.116980 2.179969
C 3.936775 0.750127 0.927471
H 4.579509 1.251946 1.631516
C -2.550220 -0.470783 0.111145
C -3.863206 -0.437167 -0.658449
H -4.326693 -1.420117 -0.584311
H -4.553118 0.301686 -0.249130
H -3.676553 -0.215776 -1.708072
C -2.809324 -0.719308 1.599131
H -3.408743 0.081174 2.035514
H -3.335080 -1.667235 1.713636
H -1.864784 -0.774765 2.144397

IBCMe2O_Pyrrole_C3.log
Energy (E) = -644.491133570 Hartree
Enthalpy (H) = -644.235028 Hartree
Gibbs free energy (G) = -644.295352 Hartree
Charge = 0, Spin = 1
C -0.564595 3.156346 -0.145387
C -1.946864 3.136050 -0.007006
C -2.625372 1.928363 0.072029
C -1.940504 0.716172 0.002478
C -0.571629 0.782927 -0.125345
C 0.142615 1.961329 -0.196022
H -0.034077 4.096064 -0.214794
H -2.498030 4.065762 0.039148
H -3.701362 1.921707 0.189613
H 1.219576 1.958307 -0.293082
O -1.768383 -1.610006 -0.409935
I 0.383690 -1.127052 -0.170654
C 2.315539 -0.223682 0.018732
H 4.890282 0.807315 1.581803
N 4.193690 0.541595 0.905371
C 2.967193 0.018809 1.201675
H 2.650170 -0.134958 2.218929
C 3.190753 0.166797 -1.032483
H 2.992360 0.108327 -2.089742
C 4.340193 0.635454 -0.453390
H 5.244615 1.019576 -0.891734
C -2.612957 -0.658070 0.109815
C -3.922684 -0.682153 -0.677222
H -4.317646 -1.696529 -0.629577
H -4.671424 0.000594 -0.271449
H -3.732056 -0.432544 -1.720414
C -2.892683 -0.921337 1.596902
H -3.549862 -0.164268 2.030725
H -3.357883 -1.902675 1.694437
H -1.953023 -0.926083 2.154051

IBCMe2O_Pyrrole_C2.log
Energy (E) = -644.491308705 Hartree
Enthalpy (H) = -644.235274 Hartree
Gibbs free energy (G) = -644.295458 Hartree
Charge = 0, Spin = 1
C -0.446807 3.140324 -0.260629
C -1.825734 3.163915 -0.090717

C -2.538449 1.980017 0.035500
C -1.889394 0.747488 -0.013692
C -0.523164 0.772212 -0.172472
C 0.225957 1.924685 -0.293982
H 0.108050 4.061806 -0.370312
H -2.348197 4.110557 -0.059191
H -3.611704 2.008724 0.172762
H 1.299478 1.887885 -0.426780
O -1.782881 -1.592624 -0.375242
I 0.368293 -1.170211 -0.170995
H 2.317510 0.167792 2.054624
N 2.806318 0.196755 1.174376
C 2.309538 -0.280793 -0.011700
C 3.259205 -0.047856 -0.976973
H 3.164806 -0.299795 -2.020237
C 4.049453 0.729317 0.977442
H 4.612892 1.152745 1.790686
C 4.361769 0.589373 -0.351059
H 5.278397 0.905367 -0.819170
C -2.594329 -0.605499 0.139290
C -3.917178 -0.616070 -0.624010
H -4.337649 -1.618024 -0.544674
H -4.639642 0.095717 -0.220951
H -3.739710 -0.395974 -1.675980
C -2.851376 -0.830508 1.635957
H -3.481559 -0.048135 2.064349
H -3.338148 -1.797531 1.764469
H -1.902183 -0.845868 2.176733

IBCMe2O_Pyridine_C4.log
Energy (E) = -682.559117719 Hartree
Enthalpy (H) = -682.296943 Hartree
Gibbs free energy (G) = -682.358866 Hartree
Charge = 0, Spin = 1
C 0.625231 3.150535 -0.290835
C 2.002917 3.180367 -0.116809
C 2.718379 1.999680 0.024173
C 2.073765 0.764943 -0.013262
C 0.707009 0.782408 -0.175205
C -0.043319 1.932266 -0.312078
H 0.066929 4.068407 -0.411587
H 2.522507 4.128692 -0.093969
H 3.791026 2.032832 0.163851
H -1.115460 1.898606 -0.442602
O 1.986832 -1.571775 -0.388908
I -0.157364 -1.170422 -0.163062
C -2.634143 0.110681 1.260232
C -3.936438 0.588830 1.340252
C -4.285910 0.262041 -0.885662
C -2.151960 -0.304488 0.027403
H -2.015929 0.071895 2.148572
H -4.338099 0.921668 2.290588
H -4.966884 0.332911 -1.726298
N -4.753934 0.667120 0.291692
C -2.996858 -0.229249 -1.069021
H -2.671901 -0.539036 -2.054446
C 2.783160 -0.583789 0.152222
C 4.121530 -0.588911 -0.582155
H 4.834529 0.118928 -0.156346
H 4.540159 -1.591365 -0.500969
H 3.968433 -0.360225 -1.636087
C 3.006762 -0.815078 1.652715
H 3.493558 -1.781131 1.787836
H 3.626052 -0.032824 2.096444
H 2.047496 -0.834044 2.174885

IBCMe2O_Pyridine_C3.log
Energy (E) = -682.559277361 Hartree
Enthalpy (H) = -682.297042 Hartree
Gibbs free energy (G) = -682.358986 Hartree
Charge = 0, Spin = 1
C 0.639178 3.154459 -0.261265
C 2.017558 3.177017 -0.091858
C 2.728118 1.992062 0.037857
C 2.078163 0.760345 -0.007157
C 0.710905 0.784644 -0.164449
C -0.034423 1.939090 -0.289217
H 0.084576 4.075624 -0.373927
H 2.541513 4.122829 -0.064013
H 3.801369 2.019458 0.174357
H -1.107077 1.910160 -0.416629
O 1.979147 -1.573710 -0.394440
I -0.166936 -1.162330 -0.164888
C -2.657690 0.068866 1.279675
C -3.957756 0.544144 1.358670
C -4.703301 0.629030 0.188566
C -2.153449 -0.297808 0.039159
H -2.045477 -0.010426 2.170408
H -4.390447 0.841752 2.303637
H -5.722946 0.995578 0.215502
C -2.982627 -0.176342 -1.069381
H -2.623009 -0.454471 -2.055323
N -4.234217 0.277700 -1.006080
C 2.782628 -0.592483 0.146128
C 4.117102 -0.598187 -0.595759
H 4.835361 0.104862 -0.170689
H 4.532404 -1.602651 -0.522308
H 3.958591 -0.363645 -1.647609
C 3.014515 -0.832142 1.644334
H 3.498379 -1.800780 1.771452
H 3.639238 -0.054594 2.088891
H 2.057930 -0.850568 2.171540

IBCMe2O_Pyridine_C2.log
Energy (E) = -682.559286709 Hartree
Enthalpy (H) = -682.297237 Hartree
Gibbs free energy (G) = -682.359421 Hartree
Charge = 0, Spin = 1
C 0.714320 3.134212 -0.470660
C 2.082094 3.142021 -0.228797
C 2.761741 1.953081 -0.002494
C 2.089213 0.733011 -0.013406
C 0.729977 0.772261 -0.228227
C 0.018547 1.931197 -0.464114
H 0.186902 4.056621 -0.670605
H 2.623574 4.078397 -0.227667
H 3.828658 1.968806 0.178058
H -1.044058 1.911738 -0.659860
O 1.966887 -1.604717 -0.359448
I -0.187946 -1.151330 -0.174257
C -2.508607 0.466098 1.188160
C -3.809601 0.924763 1.316055
C -4.717272 0.648646 0.301183
C -4.282044 -0.075651 -0.797930
C -2.166866 -0.239450 0.038762
H -1.767716 0.654678 1.954773
H -4.113615 1.481959 2.192161
H -5.743170 0.983199 0.360132
H -4.962128 -0.316065 -1.606283
N -3.026582 -0.511591 -0.930779
C 2.757217 -0.629919 0.209218
C 4.131199 -0.673832 -0.455482
H 4.839520 0.020717 -0.000402
H 4.519594 -1.685580 -0.344361
H 4.036951 -0.452682 -1.517948
C 2.902176 -0.843354 1.722778
H 3.358498 -1.818470 1.894757
H 3.517870 -0.069162 2.185638
H 1.918653 -0.833294 2.197952

IBCMe2O_PPh2.log
Energy (E) = -1239.23698693 Hartree
Enthalpy (H) = -1238.862640 Hartree
Gibbs free energy (G) = -1238.940775 Hartree
Charge = 0, Spin = 1
C 2.978107 1.683952 -1.905492
C 4.124632 1.375325 -1.185079
C 4.114212 0.347302 -0.251602
C 2.957612 -0.393218 -0.022996
C 1.837865 -0.044998 -0.745724
C 1.807651 0.968574 -1.681488
H 2.990076 2.473536 -2.643907
H 5.034514 1.934574 -1.356505
H 5.014304 0.112616 0.301530
H 0.910611 1.192171 -2.243264
O 1.813366 -2.376053 0.575892
I 0.126568 -1.210957 -0.258050
P -1.503271 0.592384 -1.257725
C -2.916900 -0.264868 -0.506808
C -3.758299 0.294455 0.456444
C -3.215948 -1.543287 -0.994264
C -4.858713 -0.410743 0.924894
H -3.560173 1.289057 0.833026
C -4.308612 -2.250506 -0.516529
H -2.592937 -1.978701 -1.768797
C -5.134063 -1.685601 0.447801
H -5.503944 0.039966 1.667234
H -4.518687 -3.239156 -0.901662
H -5.989227 -2.232935 0.819966
C -1.113979 1.931123 -0.089505
C -0.849554 3.185880 -0.637795
C -0.998921 1.766531 1.294273
C -0.481060 4.254355 0.170998
H -0.932700 3.320011 -1.710213
C -0.635662 2.830665 2.103410
H -1.196828 0.797560 1.737251
C -0.373564 4.077317 1.542290
H -0.277936 5.220509 -0.270754
H -0.550513 2.688872 3.172475
H -0.084491 4.905208 2.175501
C 2.832925 -1.537389 0.986202
C 4.117665 -2.357975 1.051411
H 4.958208 -1.780195 1.439781
H 3.941554 -3.201662 1.717592
H 4.365361 -2.740571 0.062189
C 2.535215 -0.921846 2.360865
H 2.398285 -1.727377 3.082687
H 3.347041 -0.271583 2.692824
H 1.617842 -0.328808 2.320599

IBCMe2O_Ph.log
Energy (E) = -666.528855178 Hartree
Enthalpy (H) = -666.254785 Hartree
Gibbs free energy (G) = -666.316807 Hartree
Charge = 0, Spin = 1
C -0.610118 3.149317 -0.256322
C -1.989536 3.186449 -0.097730
C -2.712934 2.008838 0.026809
C -2.075781 0.770070 -0.013960
C -0.707323 0.779736 -0.162569
C 0.050729 1.926955 -0.280125
H -0.044820 4.064883 -0.362169
H -2.503840 4.137717 -0.072603
H -3.786680 2.047369 0.156982
H 1.124622 1.885675 -0.395323

O -2.002384 -1.567783 -0.387666
I 0.159503 -1.172941 -0.156820
C 2.148767 -0.323194 0.024962
C 2.954220 -0.200317 -1.100147
C 2.640970 0.039516 1.273158
C 4.250396 0.289031 -0.978672
H 2.573249 -0.480343 -2.075132
C 3.934550 0.531229 1.395042
H 2.014567 -0.055390 2.152286
C 4.738763 0.655925 0.268390
H 4.875624 0.384556 -1.856352
H 4.315037 0.814771 2.367250
H 5.746525 1.037009 0.363420
C -2.797536 -0.574411 0.138039
C -4.126005 -0.565400 -0.615927
H -4.554873 -1.564112 -0.541115
H -4.839246 0.149287 -0.201641
H -3.954490 -0.338716 -1.667538
C -3.047823 -0.801674 1.635694
H -3.666362 -0.013455 2.070398
H -3.545680 -1.763248 1.763129
H -2.096233 -0.830292 2.171350

IBCMe2O_OTf.log
Energy (E) = -1396.22247140 Hartree
Enthalpy (H) = -1396.005187 Hartree
Gibbs free energy (G) = -1396.075347
Hartree
Charge = 0, Spin = 1
C 2.041440 3.159171 -0.373891
C 3.312986 2.854498 0.094433
C 3.672524 1.540592 0.366574
C 2.761078 0.511590 0.163791
C 1.510294 0.862278 -0.306292
C 1.105827 2.150395 -0.579497
H 1.769540 4.183555 -0.585066
H 4.031459 3.647224 0.250046
H 4.663160 1.312687 0.737822
H 0.109155 2.362664 -0.932871
O 2.153932 -1.722987 -0.353328
I 0.337134 -0.870344 -0.529220
S -2.777576 0.012230 -0.681057
O -2.655077 -1.422739 -0.796279
O -3.755433 0.716942 -1.446877
O -1.410897 0.649337 -0.741727
C -3.199722 0.307794 1.082002
F -3.265508 1.606236 1.332865
F -4.360464 -0.252875 1.374595
F -2.254628 -0.227771 1.856114
C 3.012621 -0.952741 0.479280
C 2.707519 -1.216083 1.950689
H 2.843139 -2.276146 2.162698
H 3.374403 -0.634141 2.586997
H 1.681094 -0.935570 2.192780
C 4.421139 -1.396613 0.127788
H 5.153279 -0.901626 0.765615
H 4.498493 -2.471026 0.286708
H 4.640363 -1.175060 -0.914874

IBCMe2O_OtBu.log
Energy (E) = -667.981831099 Hartree
Enthalpy (H) = -667.670820 Hartree
Gibbs free energy (G) = -667.734528
Hartree
Charge = 0, Spin = 1
C -1.054013 3.140031 -0.414717
C -2.361794 2.947225 0.014393
C -2.841535 1.669144 0.271122
C -2.018001 0.561953 0.093667
C -0.725124 0.800115 -0.318924
C -0.207624 2.049840 -0.582980
H -0.690640 4.136462 -0.624428
H -3.014616 3.799473 0.145977
H -3.860999 1.530184 0.607085
H 0.815756 2.157690 -0.912826
O -1.589181 -1.709933 -0.405000
I 0.381302 -0.979408 -0.535215
O 2.101723 0.183146 -0.708179
C 2.968989 0.315789 0.408651
C 4.066854 1.258997 -0.064048
H 3.630853 2.218329 -0.346236
H 4.803441 1.423874 0.724237
H 4.565049 0.835643 -0.935771
C 3.570953 -1.036620 0.776716
H 4.327234 -0.927886 1.555460
H 2.802514 -1.715596 1.153924
H 4.034468 -1.483845 -0.103250
C 2.259235 0.918956 1.617260
H 2.960637 1.041784 2.443774
H 1.841836 1.895227 1.369274
H 1.448563 0.273394 1.964751
C -2.411157 -0.885793 0.368588
C -3.849940 -1.170293 -0.037108
H -4.040177 -2.233512 0.103935
H -4.559876 -0.608353 0.571188
H -3.998323 -0.924106 -1.087367
C -2.213007 -1.165602 1.861058
H -2.852562 -0.525902 2.471121
H -2.450748 -2.210446 2.061035
H -1.174775 -0.981972 2.147871

IBCMe2O_OSO2Me.log
Energy (E) = -1098.61173925 Hartree
Enthalpy (H) = -1098.373905 Hartree
Gibbs free energy (G) = -1098.437387
Hartree
Charge = 0, Spin = 1
C -0.981851 3.180825 -0.046875
C -2.366510 3.076070 -0.013899
C -2.983517 1.831768 0.017644
C -2.216134 0.673023 0.017385
C -0.845814 0.831041 -0.026903
C -0.187169 2.038544 -0.058771
H -0.508633 4.152454 -0.054306
H -2.972209 3.971802 -0.005230
H -4.062974 1.759383 0.046256
H 0.891951 2.097320 -0.066133
O -1.780186 -1.591433 0.575041
I 0.088467 -1.057614 -0.016608
S 3.084837 0.217027 0.171419
O 3.217179 -0.927282 1.043515
O 2.978438 1.521192 0.784182
O 1.915170 -0.007052 -0.798765
C 4.456818 0.245505 -0.937842
H 4.511338 -0.713978 -1.442709
H 4.308435 1.056063 -1.644801
H 5.344659 0.417557 -0.335185
C -2.768657 -0.743163 0.016645
C -3.990768 -0.889434 0.906812
H -4.831184 -0.315939 0.515563
H -4.275224 -1.940266 0.936201
H -3.763576 -0.555736 1.917515
C -3.088378 -1.162384 -1.416360
H -3.444931 -2.192166 -1.418754
H -3.855876 -0.515176 -1.841790
H -2.200871 -1.094390 -2.047890

IBCMe2O_OPh.log
Energy (E) = -741.723048914 Hartree
Enthalpy (H) = -741.443589 Hartree
Gibbs free energy (G) = -741.506977
Hartree
Charge = 0, Spin = 1
C -0.872206 2.991824 -1.093557
C -2.115000 3.144234 -0.490160
C -2.756263 2.061377 0.098073
C -2.164985 0.802548 0.076560
C -0.935765 0.698085 -0.536846
C -0.254766 1.746265 -1.115770
H -0.379259 3.839520 -1.548182
H -2.587381 4.116945 -0.474774
H -3.717793 2.194234 0.576906
H 0.713081 1.591242 -1.570201
O -2.173019 -1.567268 0.097821
I -0.194508 -1.267974 -0.454152
O 1.664098 -0.533601 -1.162868
C 2.609366 -0.102896 -0.302695
C 3.948120 -0.312422 -0.647252
C 2.319510 0.567675 0.888166
C 4.965042 0.140349 0.176216
H 4.161311 -0.833178 -1.570891
C 3.347563 1.012295 1.710357
H 1.289798 0.755791 1.167875
C 4.674270 0.804533 1.363537
H 5.995027 -0.030563 -0.109196
H 3.103200 1.530720 2.628459
H 5.470217 1.153336 2.006653
C -2.725492 -0.448241 0.741586
C -4.234841 -0.551718 0.590054
H -4.559451 -1.500356 1.015421
H -4.744928 0.255500 1.116750
H -4.508903 -0.526634 -0.463272
C -2.331420 -0.428861 2.219566
H -2.758612 0.436679 2.727706
H -2.689997 -1.340912 2.696458
H -1.244834 -0.383049 2.323701

IBCMe2O_OMe.log
Energy (E) = -550.144628869 Hartree
Enthalpy (H) = -549.920981 Hartree
Gibbs free energy (G) = -549.975943
Hartree
Charge = 0, Spin = 1
C -0.955816 3.078006 -0.254039
C -2.259566 2.648004 -0.037034
C -2.546236 1.295623 0.099754
C -1.528610 0.350915 0.013813
C -0.251410 0.821734 -0.195346
C 0.079308 2.152837 -0.330092
H -0.741227 4.131377 -0.368535
H -3.060224 3.372595 0.023459
H -3.564870 0.972317 0.271309
H 1.105038 2.445831 -0.503149
O -0.637032 -1.794097 -0.451561
I 1.166108 -0.733812 -0.278990
O 2.659439 0.704253 -0.137866
C 2.918332 1.107632 1.180254
H 3.718035 1.850404 1.165395
H 2.039193 1.567079 1.654027
H 3.242944 0.275470 1.816416
C -1.705710 -1.154712 0.184274
C -2.985077 -1.650661 -0.474129
H -3.013435 -2.736363 -0.390602
H -3.872743 -1.243228 0.011492
H -2.992639 -1.379879 -1.528762
C -1.715932 -1.476642 1.680666
H -2.544131 -0.977599 2.186287
H -1.810839 -2.554756 1.810534
H -0.783796 -1.148861 2.145307

IBCMe2O_OCF3.log
Energy (E) = -847.811577179 Hartree
Enthalpy (H) = -847.608842 Hartree
Gibbs free energy (G) = -847.669791 Hartree
Charge = 0, Spin = 1
C -0.931300 3.176071 -0.128482
C -2.302699 3.006015 0.013053
C -2.852391 1.734158 0.106446
C -2.031398 0.612657 0.070778
C -0.675947 0.832108 -0.062000
C -0.087785 2.071536 -0.176452
H -0.509277 4.168407 -0.200734
H -2.949987 3.871414 0.048446
H -3.922906 1.610108 0.205703
H 0.978515 2.176487 -0.296357
O -1.463485 -1.636857 0.566152
I 0.355712 -1.003325 -0.108867
O 2.105615 0.123564 -0.823731
C 3.066442 0.276397 0.043347
F 2.686692 0.994317 1.135391
F 4.117662 0.912965 -0.480654
F 3.527619 -0.897569 0.541347
C -2.522193 -0.826702 0.099107
C -2.930553 -1.241837 -1.313148
H -3.247471 -2.284421 -1.302130
H -3.749532 -0.620470 -1.676827
H -2.091939 -1.135596 -2.003861
C -3.670717 -1.027465 1.073722
H -4.559770 -0.484351 0.752747
H -3.910953 -2.089008 1.111206
H -3.382968 -0.695023 2.069409

IBCMe2O_NTf2.log
Energy (E) = -2261.63790434 Hartree
Enthalpy (H) = -2261.386219 Hartree
Gibbs free energy (G) = -2261.471121 Hartree
Charge = 0, Spin = 1
C 2.419455 -1.557647 2.692685
C 3.764158 -1.643906 2.358880
C 4.253009 -1.020515 1.217891
C 3.396730 -0.299411 0.395117
C 2.068392 -0.246778 0.767689
C 1.537684 -0.846989 1.884808
H 2.044426 -2.037858 3.584832
H 4.439585 -2.199674 2.994152
H 5.302176 -1.094079 0.963365
H 0.494659 -0.766938 2.148311
O 2.893628 1.465445 -1.104150
I 1.012007 0.905516 -0.653243
N -1.055743 -0.074123 0.174602
S -1.232491 -1.675758 -0.080208
S -1.873499 0.767836 1.306044
O -0.154226 -2.047024 -0.953451
O -1.498918 -2.406928 1.118734
O -0.922874 1.557579 2.035599
O -2.889174 0.008894 1.963888
C -2.749889 -1.798952 -1.125919
C -2.747344 1.974415 0.217727
F -3.397611 2.836677 0.973939
F -1.858862 2.628363 -0.527963
F -3.595460 1.349961 -0.578654
F -2.637033 -0.979305 -2.160801
F -3.824211 -1.488469 -0.429920
F -2.844953 -3.042938 -1.559771
C 3.794334 0.378642 -0.903834
C 5.173520 1.008493 -0.845766
H 5.240413 1.700286 -0.008384
H 5.943220 0.243404 -0.747064
H 5.348661 1.553074 -1.772240
C 3.692935 -0.626792 -2.047232
H 4.417057 -1.429078 -1.905581
H 2.700010 -1.079001 -2.088377
H 3.897289 -0.122586 -2.991415

IBCMe2O_NMe2.log
Energy (E) = -569.535162186 Hartree
Enthalpy (H) = -569.270197 Hartree
Gibbs free energy (G) = -569.329109 Hartree
Charge = 0, Spin = 1
C -0.659704 3.147640 -0.111293
C -2.029297 2.930173 -0.025323
C -2.536337 1.638080 0.020988
C -1.681528 0.540617 -0.029658
C -0.330842 0.797210 -0.112568
C 0.211199 2.064795 -0.146658
H -0.267481 4.154310 -0.152594
H -2.706409 3.773034 0.005877
H -3.604570 1.479276 0.095899
H 1.278885 2.211367 -0.210700
O -1.114348 -1.709409 -0.479707
I 0.865151 -0.954161 -0.127404
N 2.661516 0.069096 0.156303
C 2.947207 0.684525 1.429123
H 2.426489 0.160967 2.231261
H 4.021318 0.630564 1.638278
H 2.660822 1.746033 1.477195
C 3.340887 0.683262 -0.959134
H 3.040339 0.214806 -1.896311
H 3.154526 1.764496 -1.046870
H 4.424234 0.555742 -0.855370
C -2.122154 -0.919292 0.054631
C -3.392046 -1.164664 -0.752063
H -3.613889 -2.230673 -0.716532
H -4.246978 -0.619527 -0.348789
H -3.235079 -0.875449 -1.790297
C -2.359111 -1.260295 1.530293
H -3.142379 -0.637190 1.966273
H -2.647499 -2.308857 1.606713
H -1.440813 -1.109046 2.101886

IBCMe2O_NHTs.log
Energy (E) = -1309.55963719 Hartree
Enthalpy (H) = -1309.224302 Hartree
Gibbs free energy (G) = -1309.302055 Hartree
Charge = 0, Spin = 1
C 2.718427 2.982298 0.035917
C 3.949144 2.601186 0.554382
C 4.273089 1.257645 0.688237
C 3.373560 0.274913 0.288523
C 2.169743 0.700612 -0.230686
C 1.797046 2.018848 -0.359320
H 2.463595 4.028325 -0.058058
H 4.658677 3.356130 0.864443
H 5.227039 0.968113 1.109508
H 0.828208 2.307215 -0.740844
O 2.787092 -1.878425 -0.494267
I 0.948146 -0.946019 -0.760199
N -0.772211 0.360009 -1.080519
S -1.516208 1.138430 0.129845
O -0.831026 0.745524 1.339460
O -1.650929 2.537079 -0.213855
H -0.909768 0.812596 -1.977764
C -3.145080 0.461917 0.198301
C -3.316730 -0.814070 0.722361
C -4.221672 1.196092 -0.265659
C -4.586970 -1.357016 0.770046
H -2.458397 -1.356717 1.097501
C -5.493017 0.636656 -0.209529
H -4.057617 2.192900 -0.650473
C -5.692500 -0.640030 0.303713
H -4.733729 -2.349060 1.179818
H -6.344205 1.203475 -0.565805
C -7.066506 -1.242727 0.371385
H -7.809976 -0.580876 -0.069282
H -7.101985 -2.195512 -0.157969
H -7.355901 -1.436493 1.405068
C 3.586559 -1.223677 0.454750
C 3.171044 -1.614907 1.874133
H 3.269421 -2.694556 1.988441
H 3.795845 -1.115180 2.615630
H 2.133250 -1.330966 2.060383
C 5.024960 -1.635558 0.183302
H 5.708105 -1.215132 0.921945
H 5.086303 -2.721753 0.236917
H 5.326555 -1.314994 -0.812607

IBCMe2O_Me.log
Energy (E) = -474.966306570 Hartree
Enthalpy (H) = -474.749262 Hartree
Gibbs free energy (G) = -474.801957 Hartree
Charge = 0, Spin = 1
C -1.376249 2.940523 -0.050835
C -2.585151 2.260875 0.016591
C -2.608121 0.873420 0.037645
C -1.427005 0.136712 -0.016724
C -0.248519 0.847722 -0.072013
C -0.184038 2.227096 -0.086194
H -1.354290 4.021129 -0.078127
H -3.513797 2.814388 0.050204
H -3.554489 0.351735 0.095623
H 0.757138 2.753057 -0.136932
O -0.164261 -1.800561 -0.517964
I 1.486383 -0.384655 -0.085857
C 2.780444 1.347863 0.319803
H 2.828101 2.012383 -0.537343
H 2.446776 1.872869 1.209938
H 3.760770 0.909712 0.496015
C -1.363552 -1.395674 0.019446
C -2.496069 -2.005044 -0.804846
H -2.353374 -3.085086 -0.812340
H -3.482129 -1.789523 -0.389549
H -2.446237 -1.641190 -1.830453
C -1.493432 -1.831915 1.486183
H -2.436921 -1.503557 1.927604
H -1.432929 -2.919595 1.531284
H -0.672692 -1.414611 2.073870

IBCMe2O_Indole_N1.log
Energy (E) = -798.002041001 Hartree
Enthalpy (H) = -797.696855 Hartree
Gibbs free energy (G) = -797.762103 Hartree
Charge = 0, Spin = 1
C -0.384902 2.869087 -1.164885
C -1.662344 3.267635 -0.791364
C -2.560496 2.353610 -0.256920
C -2.194817 1.020453 -0.097756
C -0.919450 0.671360 -0.482907
C 0.010634 1.545764 -1.003628
H 0.310840 3.583602 -1.581504
H -1.960311 4.299763 -0.915975
H -3.550381 2.675330 0.039716
H 1.003649 1.225184 -1.287531
O -2.620997 -1.303636 0.077649
I -0.536776 -1.383544 -0.132058

C 3.540435 0.056897 -0.438927
C 2.390933 -0.299052 0.302351
C 2.233473 0.071753 1.638802
C 3.254891 0.784953 2.233593
C 4.413707 1.129812 1.517082
C 4.564579 0.774910 0.192398
H 1.340869 -0.193993 2.192588
H 3.163016 1.084666 3.268891
H 5.195924 1.686072 2.016090
H 5.458801 1.048892 -0.352754
N 1.539544 -1.004080 -0.522349
C 3.339748 -0.443122 -1.766539
H 4.017916 -0.366222 -2.600017
C 2.122298 -1.058627 -1.771868
H 1.612274 -1.552831 -2.584366
C -3.075655 -0.058994 0.524253
C -4.527643 0.074697 0.088424
H -5.085277 -0.766977 0.497186
H -4.976613 0.997872 0.456502
H -4.596501 0.046595 -0.997867
C -2.962030 0.043779 2.047245
H -3.313169 1.012735 2.405137
H -3.558559 -0.747048 2.501957
H -1.922347 -0.077637 2.360120

IBCMe2O_Indole_C3.log
Energy (E) = -797.999800964 Hartree
Enthalpy (H) = -797.694099 Hartree
Gibbs free energy (G) = -797.760472 Hartree
Charge = 0, Spin = 1
C 0.784674 2.807305 -1.403827
C 2.087085 3.119320 -1.034717
C 2.886426 2.171089 -0.411867
C 2.404997 0.889143 -0.152936
C 1.107538 0.625206 -0.529515
C 0.276386 1.541140 -1.140102
H 0.161894 3.542807 -1.893914
H 2.481715 4.106663 -1.233805
H 3.897650 2.425515 -0.121687
H -0.736958 1.282100 -1.413565
O 2.648804 -1.430623 0.257640
I 0.456510 -1.343980 -0.026351
C -2.468878 -0.031728 0.295838
C -3.731760 -0.091852 -0.328020
C -4.836860 0.595046 0.171039
C -4.650330 1.353911 1.310120
C -3.397301 1.434353 1.940421
C -2.306255 0.751371 1.443259
C -1.574773 -0.839999 -0.482656
H -5.803068 0.540033 -0.313067
H -5.486024 1.901068 1.724872
H -3.291601 2.043774 2.827411
H -1.338194 0.816134 1.926038
H -4.343110 -1.142830 -2.072619
N -3.603972 -0.905585 -1.432518
C -2.304679 -1.346081 -1.518243
H -1.995458 -1.995582 -2.320891
C 3.210170 -0.208949 0.552336
C 4.660928 -0.210016 0.073994
H 5.192240 0.705303 0.340631
H 5.164534 -1.052451 0.547083
H 4.694605 -0.347141 -1.006228
C 3.157215 0.073696 2.060848
H 3.708888 -0.709477 2.581501
H 3.587157 1.046410 2.309695
H 2.120577 0.054964 2.405845

IBCMe2O_Indole_C2.log
Energy (E) = -797.997473215 Hartree
Enthalpy (H) = -797.691912 Hartree
Gibbs free energy (G) = -797.758255 Hartree
Charge = 0, Spin = 1
C -0.859783 3.072624 -0.553223
C -2.206854 3.313447 -0.312248
C -3.073453 2.261671 -0.050242
C -2.612840 0.946496 -0.029522
C -1.270127 0.756457 -0.260486
C -0.372426 1.771506 -0.519805
H -0.186440 3.890161 -0.770193
H -2.584413 4.326852 -0.332966
H -4.120575 2.459865 0.138624
H 0.672831 1.566271 -0.709102
O -2.867140 -1.402233 -0.219144
I -0.678659 -1.292166 -0.141351
C 3.545272 -0.265788 -0.621863
C 3.311508 0.102662 0.720436
C 4.314137 0.633521 1.531734
C 5.565552 0.792672 0.971963
C 5.820605 0.433174 -0.362881
C 4.826894 -0.092672 -1.161234
H 4.120237 0.909982 2.559686
H 6.367285 1.201871 1.571714
H 6.814843 0.572821 -0.764997
H 5.031005 -0.368163 -2.187551
H 1.529748 -0.008310 1.876184
N 1.991175 -0.168683 0.995663
C 1.386851 -0.688228 -0.129645
C 2.303255 -0.761676 -1.137720
H 2.113300 -1.120121 -2.136175
C -3.493270 -0.273161 0.266316
C -3.679945 -0.359257 1.787178
H -4.162462 0.534097 2.189138
H -4.290560 -1.233190 2.014883
H -2.709810 -0.477799 2.275318
C -4.850917 -0.140782 -0.419408
H -5.404109 -1.062329 -0.241556
H -5.435730 0.694558 -0.030827
H -4.714098 -0.019131 -1.493171

IBCMe2O_I.log
Energy (E) = -446.515555986 Hartree
Enthalpy (H) = -446.334350 Hartree
Gibbs free energy (G) = -446.389164 Hartree
Charge = 0, Spin = 1
C 1.094930 3.178005 -0.161420
C 2.465685 3.027318 -0.000134
C 3.031169 1.762240 0.092875
C 2.232036 0.627185 0.016368
C 0.875527 0.828197 -0.138546
C 0.270681 2.059336 -0.227698
H 0.658016 4.163503 -0.238253
H 3.099807 3.901463 0.053353
H 4.099989 1.652589 0.222784
H -0.800805 2.155287 -0.341383
O 1.828261 -1.642292 -0.518419
I -0.140810 -1.038331 -0.221452
I -2.897508 0.230196 0.135505
C 2.733665 -0.800587 0.154380
C 4.077844 -1.009593 -0.526478
H 4.863737 -0.442326 -0.026952
H 4.330610 -2.067396 -0.473155
H 4.022080 -0.711332 -1.571691
C 2.820151 -1.160026 1.636427
H 3.143432 -2.196093 1.734198
H 3.530198 -0.511049 2.150059
H 1.847600 -1.047611 2.118903

IBCMe2O_Et.log
Energy (E) = -514.240120548 Hartree
Enthalpy (H) = -513.992932 Hartree
Gibbs free energy (G) = -514.049479 Hartree
Charge = 0, Spin = 1
C -1.379366 3.027416 0.051305
C -2.637193 2.451980 -0.071533
C -2.775191 1.071527 -0.106667
C -1.662765 0.237921 -0.012529
C -0.432043 0.846136 0.096453
C -0.253810 2.216057 0.127907
H -1.268568 4.102233 0.090331
H -3.514236 3.081652 -0.136873
H -3.758889 0.631698 -0.206972
H 0.725409 2.660204 0.223302
O -0.587559 -1.804708 0.515933
I 1.197371 -0.521064 0.165562
C 2.674979 1.080358 -0.185981
H 2.364577 1.640795 -1.064733
H 2.695185 1.734824 0.681952
C 4.000710 0.373981 -0.392507
H 3.965892 -0.292596 -1.255619
H 4.806481 1.089272 -0.560597
H 4.274266 -0.224786 0.478650
C -1.727034 -1.294712 -0.059437
C -2.937426 -1.809096 0.718262
H -3.885575 -1.507772 0.269330
H -2.887850 -2.897427 0.722214
H -2.894632 -1.455878 1.747928
C -1.839701 -1.709818 -1.533849
H -1.870110 -2.798428 -1.586368
H -2.734593 -1.299496 -2.006916
H -0.964510 -1.360733 -2.086442

IBCMe2O_CF2H.log
Energy (E) = -673.367484911 Hartree
Enthalpy (H) = -673.163194 Hartree
Gibbs free energy (G) = -673.220112 Hartree
Charge = 0, Spin = 1
C -1.049234 3.119724 -0.035698
C -2.379180 2.734977 -0.143891
C -2.724185 1.390565 -0.136836
C -1.746323 0.405645 -0.023028
C -0.438525 0.827335 0.061020
C -0.054055 2.154444 0.062874
H -0.780658 4.166703 -0.018048
H -3.152269 3.486996 -0.223999
H -3.763632 1.100420 -0.216412
H 0.977214 2.453039 0.175283
O -0.969428 -1.742775 0.601837
I 0.938095 -0.791616 0.139292
C 2.608337 0.595832 -0.395072
H 2.404146 1.232871 -1.256581
F 3.000856 1.366683 0.643318
F 3.628218 -0.242270 -0.690794
C -2.029767 -1.100588 -0.009340
C -3.292263 -1.409936 0.789527
H -4.189200 -0.996837 0.325504
H -3.396969 -2.493037 0.839195
H -3.195814 -1.022723 1.802927
C -2.199348 -1.565644 -1.461137
H -2.379723 -2.640679 -1.464830
H -3.031635 -1.057906 -1.952449
H -1.289489 -1.364851 -2.030967

IBCMe2O_CF2CF3.log
Energy (E) = -1010.27081566 Hartree
Enthalpy (H) = -1010.058412 Hartree

Gibbs free energy (G) = -1010.123931 Hartree
Charge = 0, Spin = 1
C 1.846661 3.085085 0.099633
C 3.145737 2.661826 -0.147130
C 3.438330 1.308255 -0.234248
C 2.437132 0.353817 -0.077087
C 1.159113 0.818671 0.144043
C 0.825239 2.153807 0.244726
H 1.619998 4.138388 0.186172
H 3.936642 3.390169 -0.262898
H 4.454159 0.985203 -0.419607
H -0.180761 2.483646 0.445013
O 1.655501 -1.779908 0.573054
I -0.228026 -0.791784 0.326383
C -1.960719 0.669122 -0.014872
F -2.187073 1.481224 1.041506
F -1.780008 1.442712 -1.103518
C -3.241680 -0.140521 -0.238530
F -3.119312 -0.886622 -1.333432
F -3.436337 -0.952320 0.802842
F -4.302960 0.641102 -0.371758
C 2.664001 -1.156553 -0.151481
C 3.993881 -1.550071 0.479816
H 4.051130 -2.637735 0.487133
H 4.845456 -1.163852 -0.081755
H 4.042159 -1.190829 1.506713
C 2.632827 -1.580492 -1.623596
H 3.423978 -1.091948 -2.194800
H 2.762512 -2.661258 -1.680236
H 1.674164 -1.320498 -2.076861

IBCMe2O_02.log
Energy (E) = -435.128381607 Hartree
Enthalpy (H) = -434.950725 Hartree
Gibbs free energy (G) = -435.000670 Hartree
Charge = 0, Spin = 2
C 1.581213 -2.781094 0.031990
C 2.693282 -1.953596 0.023903
C 2.528574 -0.576442 -0.006534
C 1.265000 0.011075 -0.032118
C 0.164962 -0.843132 -0.018446
C 0.311375 -2.222113 0.012901
H 1.693696 -3.856275 0.052516
H 3.688586 -2.375963 0.038133
H 3.405608 0.055948 -0.017323
H -0.563404 -2.857032 0.020810
O 0.070905 1.919499 -0.856531
I -1.808313 -0.089046 -0.020504
C 1.087192 1.525974 -0.015400
C 2.301344 2.263335 -0.616610
H 2.049918 3.317625 -0.711931
H 3.155132 2.171198 0.053715
H 2.552120 1.859845 -1.595029
C 0.872741 2.015515 1.419729
H 1.737121 1.765726 2.036266
H 0.731810 3.096194 1.412742
H -0.011324 1.549047 1.852576

IBamideAc_02_2.log
Energy (E) = -563.232388911 Hartree
Enthalpy (H) = -563.078008 Hartree
Gibbs free energy (G) = -563.134117 Hartree
Charge = 0, Spin = 2
C 3.645894 -0.122611 0.243935
C 3.538030 1.256860 0.114302
C 2.293427 1.829288 -0.070519
C 1.139778 1.045970 -0.130361
C 1.269194 -0.330437 0.007105
C 2.511550 -0.919369 0.192604
H 4.612739 -0.585136 0.387600
H 4.420484 1.879402 0.156442
H 2.167653 2.898351 -0.175698
H 2.593297 -1.991782 0.298362
C -0.140657 1.788438 -0.332606
O -0.178633 2.998252 -0.376916
I -0.396473 -1.621592 -0.026448
N -1.286557 1.053471 -0.590389
C -3.541257 0.219594 -0.137262
H -4.498853 0.732451 -0.093916
H -3.390968 -0.239644 -1.110372
H -3.544221 -0.558972 0.626750
C -2.449389 1.202994 0.179395
O -2.563942 2.078126 1.007632

IBamideMe_02.log
Energy (E) = -449.975919215 Hartree
Enthalpy (H) = -449.832902 Hartree
Gibbs free energy (G) = -449.883969 Hartree
Charge = 0, Spin = 2
C 2.961883 -1.463744 -0.110120
C 3.457786 -0.167507 -0.047029
C 2.576766 0.897657 0.010701
C 1.197366 0.692353 -0.012263
C 0.718576 -0.612529 -0.056053
C 1.592748 -1.689037 -0.102747
H 3.638202 -2.306504 -0.154230
H 4.524149 0.009289 -0.041399
H 2.927173 1.918930 0.071637
H 1.206230 -2.698057 -0.126212
C 0.336580 1.911214 0.028674
O 0.739708 2.966374 0.498545
I -1.340347 -1.054991 0.066902
N -0.868663 1.840731 -0.621735
C -1.917994 2.701720 -0.163131
H -2.828586 2.104793 -0.069372
H -1.685708 3.196911 0.779941
H -2.122376 3.452298 -0.930560

IBamideMe_O_02.log
Energy (E) = -449.972743280 Hartree
Enthalpy (H) = -449.829882 Hartree
Gibbs free energy (G) = -449.881647 Hartree
Charge = 0, Spin = 2
C 0.121441 -3.213581 -0.094282
C 1.484938 -2.982976 0.018091
C 1.947171 -1.683214 0.127935
C 1.065962 -0.597361 0.150237
C -0.303753 -0.845004 0.031700
C -0.767039 -2.147425 -0.097095
H -0.258680 -4.221934 -0.186319
H 2.183243 -3.808026 0.017954
H 3.004201 -1.477444 0.210767
H -1.827594 -2.327448 -0.201116
C 1.641658 0.767912 0.314747
I -1.771101 0.666889 -0.063301
O 1.070038 1.684301 0.881698
N 2.940196 0.901008 -0.145802
C 3.340728 2.214886 -0.540194
H 3.638959 2.168545 -1.592975
H 2.559248 2.962212 -0.402987
H 4.240670 2.498083 0.009832

IBamideOMe_02.log
Energy (E) = -525.111183131 Hartree
Enthalpy (H) = -524.961911 Hartree
Gibbs free energy (G) = -525.014137 Hartree
Charge = 0, Spin = 2
C -3.461134 0.454249 -0.067324
C -3.105584 1.796038 -0.012791
C -1.767015 2.145694 0.018800
C -0.771249 1.170958 -0.025231
C -1.142864 -0.168852 -0.054992
C -2.482482 -0.529689 -0.074089
H -4.503208 0.166023 -0.090706
H -3.867483 2.562463 0.007381
H -1.457287 3.180602 0.074774
H -2.759583 -1.574236 -0.084204
C 0.640891 1.652185 -0.004976
O 0.995923 2.600679 0.673648
I 0.280299 -1.722454 0.073661
N 1.437562 1.033137 -0.964949
O 2.743740 1.104418 -0.815685
C 3.315909 1.214027 0.497789
H 4.307827 0.783491 0.400064
H 3.364685 2.251735 0.809869
H 2.720125 0.639586 1.206608

IBamidePh_02.log
Energy (E) = -641.557198767 Hartree
Enthalpy (H) = -641.357932 Hartree
Gibbs free energy (G) = -641.416790 Hartree
Charge = 0, Spin = 2
C -4.204018 0.666080 0.098084
C -3.769302 1.984843 0.115298
C -2.412763 2.253109 0.096225
C -1.467331 1.225582 0.079829
C -1.921057 -0.090417 0.044387
C -3.282045 -0.368752 0.049770
H -5.260792 0.436342 0.111033
H -4.482679 2.796608 0.141910
H -2.040016 3.268042 0.096880
H -3.619226 -1.394770 0.009744
C -0.035108 1.670390 0.094455
O 0.277035 2.759725 -0.356423
I -0.637267 -1.755804 -0.146529
N 0.846094 0.871836 0.792603
C 2.110190 0.678366 0.395407
C 2.993085 0.067206 1.324504
C 2.599724 0.990758 -0.903117
C 4.298529 -0.199162 0.982129
H 2.594835 -0.176161 2.299602
C 3.907100 0.713610 -1.229793
H 1.933431 1.449991 -1.617780
C 4.763293 0.124127 -0.295040
H 4.964460 -0.660846 1.698002
H 4.275732 0.951256 -2.218246
H 5.788687 -0.087929 -0.564778

IBCH2CH2CH2CO2_02.log
Energy (E) = -548.399426644 Hartree
Enthalpy (H) = -548.209552 Hartree
Gibbs free energy (G) = -548.265223 Hartree
Charge = 0, Spin = 2
C 3.545344 -0.184885 0.777835
C 3.445236 -1.483087 0.293484
C 2.283629 -1.885603 -0.341066
C 1.192678 -1.028303 -0.515456
C 1.320838 0.266397 -0.018002
C 2.482323 0.689297 0.620443
H 4.446083 0.150486 1.273106
H 4.269666 -2.173861 0.404753
H 2.203408 -2.894951 -0.729423

H 2.551423 1.701998 0.991801
I -0.240036 1.685829 -0.191636
C -0.824824 -2.520849 -0.285514
H -1.602664 -3.015429 -0.868166
H -0.159367 -3.297844 0.095285
C -0.030502 -1.581143 -1.198843
H 0.292699 -2.145121 -2.075434
H -0.679735 -0.783046 -1.556127
C -1.467495 -1.803993 0.896045
H -0.767188 -1.131869 1.398946
H -1.815187 -2.511019 1.652379
C -2.660527 -1.005856 0.479554
O -3.281508 -0.245143 1.299951
O -3.185668 -1.007370 -0.635111

IBCH2CH2CO2_02.log
Energy (E) = -509.120542144 Hartree
Enthalpy (H) = -508.960627 Hartree
Gibbs free energy (G) = -509.014212 Hartree
Charge = 0, Spin = 2
C -2.799240 -1.552661 0.750529
C -3.417867 -0.470300 0.138710
C -2.651696 0.441560 -0.565624
C -1.265972 0.311315 -0.681670
C -0.669779 -0.782351 -0.058607
C -1.425865 -1.708106 0.650416
H -3.380363 -2.276899 1.304429
H -4.488998 -0.340586 0.207623
H -3.128872 1.285874 -1.051195
H -0.937786 -2.547362 1.125016
I 1.416751 -1.106498 -0.133550
C -0.335481 2.664060 -0.627448
H 0.146507 3.434177 -1.228656
C 0.513197 2.443430 0.580935
O 0.055468 1.890590 1.635011
O 1.703362 2.747980 0.698904
H -1.310778 3.025345 -0.295851
C -0.518185 1.372643 -1.444448
H -1.086596 1.629155 -2.339012
H 0.453757 1.008597 -1.772306

IBCH2CH2O_02.log
Energy (E) = -474.408149595 Hartree
Enthalpy (H) = -474.200911 Hartree
Gibbs free energy (G) = -474.254371 Hartree
Charge = 0, Spin = 2
C -1.158429 3.143481 0.370653
C 0.110100 3.446501 -0.102639
C 0.933620 2.427047 -0.554382
C 0.527272 1.092127 -0.545001
C -0.753614 0.818395 -0.067829
C -1.592795 1.825480 0.385154
H -1.814642 3.926560 0.724908
H 0.455377 4.470993 -0.123841
H 1.922891 2.658506 -0.932109
H -2.580993 1.581456 0.748560
I -1.492936 -1.158981 -0.030894
C 1.461583 0.022922 -1.015069
H 0.913967 -0.734046 -1.579810
H 2.211197 0.450462 -1.684314
O 1.403052 -0.911797 1.225256
C 2.208454 -0.696226 0.139903
C 2.827198 -1.991947 -0.371645
H 3.465095 -1.800050 -1.236162
H 3.420340 -2.459827 0.413111
H 2.034113 -2.679022 -0.669448
C 3.300702 0.236484 0.706391
H 3.798087 -0.242680 1.547298
H 4.029537 0.426434 -0.082783
H 2.861671 1.176017 1.036679

IBCH2CH2S_02.log
Energy (E) = -797.351772283 Hartree
Enthalpy (H) = -797.145340 Hartree
Gibbs free energy (G) = -797.199764 Hartree
Charge = 0, Spin = 2
C -1.472552 3.078535 0.460419
C -0.231778 3.474537 -0.018142
C 0.641862 2.525291 -0.523451
C 0.320182 1.166936 -0.559735
C -0.937570 0.803170 -0.082907
C -1.828841 1.738433 0.422544
H -2.168066 3.806107 0.855469
H 0.052024 4.517820 -0.004179
H 1.606453 2.831467 -0.911204
H -2.796398 1.421141 0.784664
I -1.576617 -1.212613 -0.119034
C 1.316652 0.190138 -1.109058
H 0.800783 -0.641336 -1.591318
H 1.910249 0.688244 -1.882423
C 2.331535 -0.392195 -0.100619
C 3.146236 -1.462025 -0.817632
H 3.644419 -1.034730 -1.692741
H 3.902787 -1.874957 -0.150999
H 2.501126 -2.276367 -1.148674
C 3.257677 0.705573 0.425593
H 3.989240 0.287189 1.117939
H 3.798584 1.153306 -0.413645
H 2.697645 1.483026 0.943565
S 1.522592 -1.066425 1.363767

IBCH2CO2_02.log
Energy (E) = -469.848742629 Hartree
Enthalpy (H) = -469.717633 Hartree
Gibbs free energy (G) = -469.766680 Hartree
Charge = 0, Spin = 2
C 2.636361 -1.861571 -0.479276
C 3.343485 -0.738995 -0.071387
C 2.662895 0.376877 0.389197
C 1.270489 0.402151 0.459349
C 0.588020 -0.735272 0.037816
C 1.249939 -1.860875 -0.427654
H 3.157034 -2.737505 -0.840270
H 4.423677 -0.731116 -0.113095
H 3.211014 1.255638 0.703995
H 0.689536 -2.727180 -0.749297
I -1.516844 -0.756014 0.084995
C 0.571618 1.637162 0.952230
H -0.210873 1.379364 1.668418
H 1.282707 2.307066 1.430978
C -0.055433 2.435016 -0.175768
O 0.295534 3.554961 -0.484475
O -1.016109 1.869476 -0.854590

IBCH2CONMe_02.log
Energy (E) = -489.253161126 Hartree
Enthalpy (H) = -489.080891 Hartree
Gibbs free energy (G) = -489.135043 Hartree
Charge = 0, Spin = 2
C -3.269916 -0.441594 0.648491
C -3.430010 0.804527 0.058747
C -2.357582 1.405244 -0.580201
C -1.109024 0.788683 -0.653940
C -0.975736 -0.461189 -0.052305
C -2.037935 -1.077227 0.593727
H -4.098000 -0.922917 1.150240
H -4.386044 1.307895 0.098052
H -2.476562 2.380706 -1.034769
H -1.903225 -2.048571 1.048195
I 0.874369 -1.484340 -0.102123
C 0.030414 1.480908 -1.345504
H 0.494057 0.852052 -2.105721
H -0.334620 2.381304 -1.843167
C 1.134719 1.913446 -0.400552
O 2.272920 2.134231 -0.765266
N 0.721144 2.171049 0.893544
C 1.620324 1.813612 1.944644
H 2.632152 1.642963 1.569429
H 1.609986 2.569080 2.728591
H 1.257828 0.877841 2.390341

IBCH2CONPh_02.log
Energy (E) = -680.833873340 Hartree
Enthalpy (H) = -680.604955 Hartree
Gibbs free energy (G) = -680.667896 Hartree
Charge = 0, Spin = 2
C -3.813985 1.008560 1.443730
C -3.436802 2.252210 0.955827
C -2.392055 2.342986 0.052071
C -1.698259 1.213616 -0.386798
C -2.089501 -0.021388 0.123171
C -3.139290 -0.128632 1.026173
H -4.630317 0.917768 2.147038
H -3.954179 3.145946 1.276103
H -2.084761 3.308930 -0.330635
H -3.426826 -1.100223 1.402272
I -1.127423 -1.818972 -0.437495
C -0.558743 1.421107 -1.347930
H -0.358551 0.512353 -1.916122
H -0.788955 2.230775 -2.037708
C 0.700916 1.824036 -0.598802
O 1.187004 2.933046 -0.690485
N 1.150800 0.843767 0.265584
C 2.426654 0.464059 0.329074
C 3.451462 0.925174 -0.549003
C 2.769469 -0.494083 1.325326
C 4.735643 0.452136 -0.414858
H 3.204504 1.660104 -1.300711
C 4.058834 -0.955010 1.442121
H 1.973062 -0.840055 1.969781
C 5.049752 -0.484381 0.575220
H 5.510828 0.807263 -1.080098
H 4.308670 -1.682617 2.201936
H 6.063768 -0.847795 0.668219

IBCH2S_02.log
Energy (E) = -758.072926822 Hartree
Enthalpy (H) = -757.896017 Hartree
Gibbs free energy (G) = -757.946567 Hartree
Charge = 0, Spin = 2
C 1.408034 -2.996796 -0.157777
C 2.565836 -2.238294 -0.131682
C 2.486587 -0.856576 -0.035065
C 1.267016 -0.179434 0.034928
C 0.119641 -0.974913 0.000403
C 0.179000 -2.357076 -0.090763
H 1.450727 -4.074284 -0.234729
H 3.534671 -2.715328 -0.187605
H 3.405236 -0.289304 -0.015544
H -0.735279 -2.932820 -0.118143
I -1.832808 -0.153173 0.055066
C 1.234903 1.333143 0.204809
S 0.134524 2.112351 -1.003804

C 2.585928 1.989261 -0.103165
H 2.472693 3.072250 -0.056016
H 3.325822 1.701406 0.646644
H 2.950698 1.717698 -1.093065
C 0.861185 1.693886 1.644456
H 1.605297 1.277660 2.327853
H 0.844016 2.778306 1.753620
H -0.116198 1.305777 1.922468

IBMeAlaO_02.log
Energy (E) = -603.688013414 Hartree
Enthalpy (H) = -603.479941 Hartree
Gibbs free energy (G) = -603.538746 Hartree
Charge = 0, Spin = 2
C -0.317409 -3.255474 -0.043738
C 1.030470 -2.988913 0.136082
C 1.457700 -1.692366 0.371827
C 0.553094 -0.628926 0.428362
C -0.796865 -0.904953 0.170688
C -1.225780 -2.206051 -0.037579
H -0.663111 -4.264512 -0.218087
H 1.756644 -3.788860 0.101778
H 2.508736 -1.502303 0.522938
H -2.271830 -2.393020 -0.235035
I -2.204934 0.629454 -0.144041
N 0.994697 0.682097 0.682679
C 0.422982 1.421436 1.796383
H -0.432273 0.895764 2.209088
H 1.179278 1.526426 2.581289
H 0.107687 2.415488 1.477729
C 2.353069 1.098619 0.338024
H 2.317784 2.190023 0.387062
C 3.510056 0.594078 1.197227
H 4.158432 1.412042 1.502529
H 3.146205 0.096559 2.096632
H 4.116415 -0.106547 0.622248
C 2.435917 0.812989 -1.147609
O 3.426257 0.500493 -1.768098
O 1.253148 0.984579 -1.663245

IBMeUreaMe_02.log
Energy (E) = -544.573930953 Hartree
Enthalpy (H) = -544.383527 Hartree
Gibbs free energy (G) = -544.441462 Hartree
Charge = 0, Spin = 2
C -3.360018 0.270847 0.679698
C -3.116304 1.597580 0.349704
C -1.866132 1.970430 -0.115595
C -0.847581 1.033399 -0.256897
C -1.111088 -0.296176 0.063368
C -2.357797 -0.678229 0.536617
H -4.330880 -0.031403 1.047380
H -3.894834 2.339216 0.461395
H -1.647431 3.000133 -0.366034
H -2.544530 -1.712443 0.788545
I 0.385366 -1.759092 -0.177021
C 1.514594 1.573251 0.014099
O 2.623521 1.850618 -0.410682
N 1.214334 1.429354 1.376876
C 2.304406 0.943851 2.160918
H 3.262715 1.291939 1.768116
H 2.173016 1.220763 3.203906
H 2.315102 -0.153618 2.099088
N 0.405972 1.450645 -0.780628
C 0.537788 1.528806 -2.223386
H 0.380572 0.547486 -2.677753
H -0.196087 2.225852 -2.629384
H 1.540458 1.876485 -2.452596

IBMeUreaPh_02.log
Energy (E) = -736.157893449 Hartree
Enthalpy (H) = -735.910984 Hartree
Gibbs free energy (G) = -735.975669 Hartree
Charge = 0, Spin = 2
C -3.056407 0.981118 2.210228
C -3.068542 2.114143 1.406822
C -2.374893 2.112624 0.208160
C -1.662326 0.990758 -0.204848
C -1.670392 -0.142543 0.605040
C -2.357693 -0.148982 1.810369
H -3.591878 0.971316 3.149624
H -3.611273 2.996344 1.716571
H -2.357286 2.986984 -0.428901
H -2.348365 -1.033837 2.430798
I -0.637247 -1.877210 0.008106
C 0.359321 1.188385 -1.550777
O 0.967445 1.144100 -2.606809
N 0.931817 1.521743 -0.320101
N -0.998300 1.015750 -1.460238
C -1.767569 0.665625 -2.638462
H -2.133041 -0.362081 -2.571024
H -2.621230 1.336536 -2.743145
H -1.118199 0.761015 -3.503549
C 2.095361 0.989268 0.053457
C 2.668123 1.491087 1.258403
C 2.783222 -0.057329 -0.632947
C 3.849192 0.985036 1.744981
H 2.130722 2.280302 1.765301
C 3.963524 -0.550275 -0.128699
H 2.365501 -0.438983 -1.551870
C 4.505449 -0.038380 1.054797
H 4.271926 1.376044 2.660268
H 4.479726 -1.343051 -0.652980
H 5.435094 -0.436904 1.437019

IBNMeCO2_02.log
Energy (E) = -525.164092466 Hartree
Enthalpy (H) = -525.014950 Hartree
Gibbs free energy (G) = -525.066556 Hartree
Charge = 0, Spin = 2
C 1.706552 -2.839866 -0.360095
C 2.783690 -1.977509 -0.217720
C 2.566801 -0.627043 -0.001535
C 1.276228 -0.102208 0.091470
C 0.208323 -0.989684 -0.032145
C 0.416240 -2.339624 -0.268494
H 1.862641 -3.894815 -0.535319
H 3.795761 -2.351028 -0.287306
H 3.407689 0.047284 0.083939
H -0.432733 -3.001101 -0.367178
I -1.764742 -0.312686 0.196497
C 0.413378 2.108791 -0.512726
O 0.634086 3.310287 -0.578805
N 1.115733 1.279469 0.338734
C 2.099500 1.958205 1.173387
H 2.388608 1.292581 1.983239
H 2.983964 2.259214 0.609291
H 1.645509 2.856898 1.581508
O -0.478403 1.597300 -1.307008

ISamideMe_02.log
Energy (E) = -885.130367942 Hartree
Enthalpy (H) = -884.985212 Hartree
Gibbs free energy (G) = -885.038488 Hartree
Charge = 0, Spin = 2
C 0.634751 -3.257123 0.146351
C 1.929669 -2.773315 0.033107
C 2.139248 -1.411586 -0.122938
C 1.056956 -0.540890 -0.177218
C -0.242996 -1.024390 -0.054890
C -0.447626 -2.387047 0.109533
H 0.457264 -4.316669 0.269318
H 2.772557 -3.448650 0.067036
H 3.134188 -0.998981 -0.213873
H -1.454200 -2.767790 0.208501
I -1.939730 0.220631 -0.048849
N 0.852573 2.015808 0.843519
C 1.049016 1.379019 2.116681
H 0.305994 0.586310 2.267168
H 2.038002 0.911376 2.173572
H 0.937568 2.116661 2.906295
S 1.438507 1.173234 -0.460789
O 0.742536 1.617464 -1.629178
O 2.871976 1.311773 -0.361418

ISamidePh_02.log
Energy (E) = -1076.71206872 Hartree
Enthalpy (H) = -1076.510471 Hartree
Gibbs free energy (G) = -1076.571301 Hartree
Charge = 0, Spin = 2
C -2.018498 2.188708 -2.080508
C -1.308227 3.091453 -1.302241
C -0.642822 2.641428 -0.173559
C -0.692205 1.298227 0.185039
C -1.411827 0.393892 -0.589840
C -2.067732 0.847464 -1.727746
H -2.540155 2.523052 -2.966759
H -1.270496 4.137720 -1.570393
H -0.077424 3.316845 0.453968
H -2.622437 0.147143 -2.335858
I -1.576195 -1.657559 -0.154297
N 1.137661 -0.388780 1.352389
S 0.132866 0.875492 1.711252
O -0.855586 0.367005 2.614625
O 0.933569 2.016230 2.091872
C 2.073943 -0.305643 0.403079
C 2.782607 -1.518942 0.149914
C 2.422951 0.848562 -0.361926
C 3.749610 -1.584474 -0.822408
H 2.507421 -2.376685 0.747186
C 3.401347 0.763597 -1.323222
H 1.946210 1.792590 -0.149113
C 4.063382 -0.444369 -1.567969
H 4.269126 -2.513658 -1.010117
H 3.669461 1.643870 -1.890973
H 4.828127 -0.491958 -2.330611

Cl_02.log
Energy (E) = -460.049844190 Hartree
Enthalpy (H) = -460.047484 Hartree
Gibbs free energy (G) = -460.065521 Hartree
Charge = 0, Spin = 2
Cl 0.000000 0.000000 0.000000

IBamideAc_Cl.log
Energy (E) = -1023.36124028 Hartree
Enthalpy (H) = -1023.202574 Hartree
Gibbs free energy (G) = -1023.259978 Hartree
Charge = 0, Spin = 1
C -3.330911 1.546118 0.000128
C -2.712659 2.792793 0.000013
C -1.331007 2.887001 -0.000074

C -0.558995 1.730627 -0.000022
C -1.210364 0.516654 0.000084
C -2.579447 0.375401 0.000155
H -4.409601 1.475173 0.000189
H -3.314714 3.690471 -0.000014
H -0.809906 3.835080 -0.000186
H -3.045050 -0.598980 0.000217
C 0.932684 1.822625 -0.000115
O 1.500240 2.886690 -0.000529
I 0.127010 -1.117936 0.000105
Cl -1.785365 -2.788022 -0.000278
N 1.516114 0.564100 0.000264
C 3.321220 -1.097895 -0.000251
H 4.405362 -1.139361 -0.000564
H 2.946531 -1.613424 0.886208
H 2.945995 -1.612824 -0.886846
C 2.904433 0.355751 0.000181
O 3.709576 1.249271 0.000242

IBamideMe_Cl.log
Energy (E) = -910.101963335 Hartree
Enthalpy (H) = -909.954242 Hartree
Gibbs free energy (G) = -910.006886 Hartree
Charge = 0, Spin = 1
C -1.431872 3.030726 -0.000136
C -2.698933 2.455115 -0.000041
C -2.842055 1.076731 0.000051
C -1.712389 0.268238 0.000025
C -0.475557 0.878109 -0.000065
C -0.286199 2.240508 -0.000138
H -1.325389 4.106543 -0.000214
H -3.575021 3.088421 -0.000033
H -3.807510 0.588310 0.000153
H 0.705333 2.669418 -0.000171
C -1.822668 -1.219148 0.000086
O -2.892248 -1.804803 0.000391
I 1.100212 -0.532150 -0.000052
Cl 2.861700 1.335430 0.000223
N -0.602958 -1.802360 -0.000247
C -0.450106 -3.239525 -0.000109
H 0.083350 -3.582684 0.888036
H -1.447632 -3.675269 -0.000450
H 0.084098 -3.582693 -0.887779

IBamideMe_O_Cl.log
Energy (E) = -910.083420685 Hartree
Enthalpy (H) = -909.936184 Hartree
Gibbs free energy (G) = -909.988421 Hartree
Charge = 0, Spin = 1
C 0.057931 3.311995 -0.000367
C 1.448871 3.244543 0.000082
C 2.094731 2.020232 0.000316
C 1.350473 0.843620 0.000111
C -0.023448 0.956992 -0.000213
C -0.707363 2.151802 -0.000507
H -0.440404 4.271215 -0.000583
H 2.028676 4.157030 0.000224
H 3.172309 1.928590 0.000613
H -1.786606 2.182860 -0.000790
C 1.969779 -0.500651 0.000115
I -0.950638 -0.946295 -0.000243
Cl -3.236879 0.064375 0.000811
O 1.092928 -1.506774 -0.000586
N 3.227725 -0.641854 0.000648
C 3.729055 -2.001284 0.000337
H 4.815970 -1.989156 0.002582
H 3.379725 -2.554770 0.876269
H 3.383425 -2.552812 -0.878311

IBamideOMe_Cl.log
Energy (E) = -985.216964776 Hartree
Enthalpy (H) = -985.064065 Hartree
Gibbs free energy (G) = -985.118869 Hartree
Charge = 0, Spin = 1
C -1.537345 3.139622 0.154398
C -0.280620 3.735116 0.117988
C 0.859772 2.955565 0.008823
C 0.746417 1.573245 -0.069346
C -0.519386 1.018817 -0.020108
C -1.675781 1.756615 0.085183
H -2.424857 3.750632 0.241491
H -0.194914 4.811091 0.176116
H 1.855405 3.378379 -0.018287
H -2.643606 1.278043 0.122124
C 1.959739 0.714399 -0.179098
O 3.099673 1.124497 -0.075638
I -0.493194 -1.092829 -0.139514
Cl -3.019744 -1.233556 0.219452
N 1.583701 -0.569147 -0.412093
O 2.510795 -1.568525 -0.396547
C 3.022328 -1.772821 0.917735
H 3.702813 -2.616369 0.847922
H 3.553680 -0.885672 1.257803
H 2.206015 -2.012798 1.605060

IBamidePh_Cl.log
Energy (E) = -1101.66946878 Hartree
Enthalpy (H) = -1101.465783 Hartree
Gibbs free energy (G) = -1101.525658 Hartree
Charge = 0, Spin = 1
C -3.950670 1.446573 0.048303
C -3.367526 2.708720 0.107290
C -1.988693 2.841670 0.127981
C -1.185874 1.707832 0.091147
C -1.802298 0.474888 0.029671
C -3.166834 0.297664 0.008444
H -5.026985 1.346797 0.031176
H -3.994912 3.588527 0.136755
H -1.494673 3.803105 0.173366
H -3.603699 -0.688892 -0.041640
C 0.300765 1.822631 0.113916
O 0.870777 2.898670 0.116418
I -0.407064 -1.108861 -0.025296
Cl -2.270622 -2.836366 -0.255913
N 0.892744 0.598777 0.151763
C 2.284402 0.378593 0.083896
C 2.830668 -0.685281 0.800312
C 3.113641 1.172259 -0.708826
C 4.186780 -0.966294 0.716269
H 2.195233 -1.272733 1.452624
C 4.469604 0.892942 -0.774196
H 2.692030 2.004684 -1.250212
C 5.012165 -0.176567 -0.071101
H 4.597128 -1.794899 1.277046
H 5.108140 1.515308 -1.386513
H 6.070477 -0.388606 -0.133734

IBCH2CH2CH2CO2_Cl.log
Energy (E) = -1008.51549731 Hartree
Enthalpy (H) = -1008.320433 Hartree
Gibbs free energy (G) = -1008.376083 Hartree
Charge = 0, Spin = 1
C -0.570298 3.033194 -0.891675
C -1.765095 3.045356 -0.182599
C -2.097636 1.978778 0.632988
C -1.268315 0.860833 0.776066
C -0.087886 0.899434 0.047060
C 0.279939 1.946158 -0.779368
H -0.300631 3.858047 -1.535412
H -2.436280 3.889043 -0.264664
H -3.023944 1.992894 1.195568
H 1.215094 1.918157 -1.319274
I 1.243895 -0.767548 0.046619
Cl 3.147626 0.819126 0.404583
C -2.832575 -1.147403 1.059556
H -2.767000 -2.123785 1.537700
H -3.807800 -0.733624 1.322854
C -1.724557 -0.256969 1.673749
H -2.111270 0.192889 2.589497
H -0.878144 -0.874909 1.961304
C -2.797838 -1.313347 -0.461439
H -3.234192 -0.453434 -0.970283
H -3.388024 -2.182230 -0.756273
C -1.400798 -1.458003 -1.028862
O -1.094801 -1.098234 -2.140768
O -0.544388 -1.933808 -0.132057

IBCH2CH2CO2_Cl.log
Energy (E) = -969.237197423 Hartree
Enthalpy (H) = -969.072125 Hartree
Gibbs free energy (G) = -969.125738 Hartree
Charge = 0, Spin = 1
C -1.049825 2.940105 -0.800214
C -2.244475 2.784204 -0.110541
C -2.475519 1.633437 0.621926
C -1.534872 0.603278 0.707942
C -0.349608 0.812888 0.012986
C -0.090456 1.942779 -0.742329
H -0.858975 3.829689 -1.383023
H -2.998693 3.557862 -0.147728
H -3.411168 1.506177 1.152468
H 0.850404 2.056792 -1.260069
I 1.210063 -0.647376 0.017987
Cl 2.880024 1.194627 0.309275
C -2.488677 -1.684625 0.449812
H -2.656993 -2.632256 0.960772
C -1.452853 -1.858806 -0.638130
O -1.644272 -1.637665 -1.806604
O -0.272619 -2.184854 -0.108918
H -3.420461 -1.333180 0.010788
C -1.908750 -0.655871 1.438748
H -2.647705 -0.412856 2.201209
H -1.044819 -1.094134 1.937599

IBCH2CH2O_Cl.log
Energy (E) = -934.525723386 Hartree
Enthalpy (H) = -934.314533 Hartree
Gibbs free energy (G) = -934.369364 Hartree
Charge = 0, Spin = 1
C -0.910207 3.279419 0.449416
C 0.330729 3.648489 -0.054382
C 1.211050 2.687601 -0.530522
C 0.877950 1.334607 -0.510127
C -0.362374 1.014260 0.024397
C -1.270064 1.938824 0.494862
H -1.600695 4.029361 0.808480
H 0.610507 4.692544 -0.084949
H 2.168981 2.979737 -0.942386
H -2.234680 1.623404 0.866159
I -0.726567 -1.048817 0.207372
Cl -3.151895 -0.614921 -0.523084
C 1.771629 0.274673 -1.072692
H 1.219615 -0.269883 -1.846697

H 2.635901 0.728585 -1.558265
O 1.226449 -1.071621 0.887316
C 2.257120 -0.747695 -0.024216
C 2.736737 -2.003030 -0.734978
H 3.521622 -1.772950 -1.457713
H 3.123565 -2.714937 -0.006598
H 1.904546 -2.468625 -1.268916
C 3.363309 -0.135399 0.822512
H 3.638522 -0.828649 1.616192
H 4.244309 0.074413 0.213227
H 3.011225 0.790430 1.279039

IBCH2CH2S_Cl.log
Energy (E) = -1257.46138761 Hartree
Enthalpy (H) = -1257.251457 Hartree
Gibbs free energy (G) = -1257.308196 Hartree
Charge = 0, Spin = 1
C -1.142761 3.214443 0.595295
C 0.054949 3.659439 0.050868
C 0.949107 2.758043 -0.507488
C 0.681613 1.389688 -0.523787
C -0.517493 0.994410 0.051679
C -1.442541 1.858607 0.598060
H -1.848679 3.916758 1.015419
H 0.287609 4.715337 0.049406
H 1.870483 3.108889 -0.956232
H -2.379061 1.486135 0.987705
I -0.858643 -1.088876 0.169118
Cl -3.308651 -0.580274 -0.689817
C 1.616206 0.417946 -1.178446
H 1.043772 -0.243704 -1.834482
H 2.309577 0.968673 -1.819467
C 2.465800 -0.450652 -0.237916
C 3.124970 -1.550829 -1.056063
H 3.733883 -1.113853 -1.852328
H 3.766064 -2.166359 -0.425592
H 2.370712 -2.195630 -1.509311
C 3.514762 0.408079 0.460507
H 4.084425 -0.183892 1.177687
H 4.210720 0.810958 -0.280271
H 3.045510 1.234096 0.995348
S 1.464527 -1.184836 1.106218

IBCH2CO2_Cl.log
Energy (E) = -929.971591401 Hartree
Enthalpy (H) = -929.836355 Hartree
Gibbs free energy (G) = -929.887042 Hartree
Charge = 0, Spin = 1
C 0.961680 3.100413 0.584810
C 2.230701 2.833254 0.086647
C 2.539076 1.580231 -0.419974
C 1.588891 0.560508 -0.445437
C 0.341741 0.872914 0.070961
C -0.006304 2.105840 0.582167
H 0.719952 4.077720 0.977281
H 2.985356 3.607336 0.091822
H 3.526906 1.375717 -0.811638
H -1.004494 2.292939 0.950651
I -1.047777 -0.712579 0.111530
Cl -2.874958 0.885620 -0.489909
C 1.907635 -0.794206 -1.001513
H 1.221115 -1.023765 -1.823965
H 2.920628 -0.824689 -1.390002
C 1.764836 -1.903518 0.033938
O 2.638469 -2.704989 0.237404
O 0.619964 -1.910850 0.696804

IBCH2CONMe_Cl.log
Energy (E) = -949.368682196 Hartree
Enthalpy (H) = -949.191351 Hartree
Gibbs free energy (G) = -949.246868 Hartree
Charge = 0, Spin = 1
C -1.294689 3.044633 0.868154
C -0.198285 3.697941 0.320256
C 0.786282 2.984363 -0.346450
C 0.702763 1.599554 -0.482166
C -0.403209 0.993896 0.092495
C -1.408382 1.665279 0.754825
H -2.064836 3.601296 1.382731
H -0.111281 4.771934 0.409225
H 1.635558 3.496264 -0.779798
H -2.262705 1.133465 1.148047
I -0.464004 -1.115765 -0.063597
Cl -3.021139 -0.943287 -0.398033
C 1.756296 0.816917 -1.209017
H 1.299388 0.266864 -2.038322
H 2.514985 1.478033 -1.616550
C 2.464326 -0.179159 -0.300935
O 3.672022 -0.161945 -0.150263
N 1.627616 -1.041241 0.331323
C 2.144880 -2.000686 1.288246
H 3.207748 -1.806463 1.413379
H 1.642512 -1.894888 2.250725
H 2.020795 -3.027280 0.938141

IBCH2CONPh_Cl.log
Energy (E) = -1140.93503311 Hartree
Enthalpy (H) = -1140.701898 Hartree
Gibbs free energy (G) = -1140.764746 Hartree
Charge = 0, Spin = 1
C -3.609810 0.874459 1.708522
C -3.562212 2.178709 1.232729
C -2.634198 2.544127 0.269285
C -1.728000 1.616956 -0.242836
C -1.806984 0.333708 0.272844
C -2.718423 -0.073379 1.224009
H -4.337201 0.588663 2.454858
H -4.256282 2.915867 1.611894
H -2.605413 3.558596 -0.106403
H -2.747778 -1.100239 1.558794
I -0.361379 -1.021771 -0.455834
Cl -2.172783 -2.841029 -0.471842
C -0.732628 1.992726 -1.299059
H -0.875630 1.357182 -2.179915
H -0.867918 3.025630 -1.605641
C 0.715076 1.854236 -0.844588
O 1.499339 2.775096 -0.956293
N 1.012363 0.642698 -0.288145
C 2.318039 0.316438 0.163074
C 3.446073 0.616893 -0.597659
C 2.463982 -0.347986 1.378330
C 4.703147 0.257949 -0.135316
H 3.330057 1.145100 -1.531891
C 3.723344 -0.713752 1.829663
H 1.584889 -0.562230 1.973225
C 4.848061 -0.410637 1.074122
H 5.575576 0.500293 -0.727021
H 3.824785 -1.228452 2.775400
H 5.831261 -0.691414 1.425864

IBCH2S_Cl.log
Energy (E) = -1218.18767788 Hartree
Enthalpy (H) = -1218.007476 Hartree
Gibbs free energy (G) = -1218.061104 Hartree
Charge = 0, Spin = 1
C -0.456065 3.262314 -0.291173
C -1.818499 3.050164 -0.152702
C -2.317261 1.764186 0.009250
C -1.471394 0.657437 0.024442
C -0.118929 0.924797 -0.123440
C 0.419523 2.181839 -0.271454
H -0.063845 4.260961 -0.420668
H -2.501697 3.888083 -0.169429
H -3.381825 1.616676 0.127006
H 1.488522 2.314298 -0.362028
I 1.150351 -0.795419 -0.140117
Cl 3.231758 0.726133 0.454314
C -1.989775 -0.745014 0.283020
S -1.008510 -1.902521 -0.721167
C -3.432310 -0.936968 -0.173601
H -3.700558 -1.990307 -0.090219
H -4.114739 -0.373598 0.464754
H -3.564265 -0.621419 -1.207632
C -1.876892 -1.062640 1.773477
H -2.484094 -0.358143 2.345718
H -2.232348 -2.075170 1.963017
H -0.847876 -0.984813 2.124080

IBMeAlaO_Cl.log
Energy (E) = -1063.80957290 Hartree
Enthalpy (H) = -1063.597454 Hartree
Gibbs free energy (G) = -1063.658458 Hartree
Charge = 0, Spin = 1
C 0.574378 3.119277 -1.245087
C -0.613505 3.438809 -0.596086
C -1.283863 2.491354 0.161152
C -0.786258 1.192614 0.289422
C 0.411235 0.925645 -0.358828
C 1.103865 1.843946 -1.123035
H 1.095718 3.862107 -1.831508
H -1.021384 4.436382 -0.683662
H -2.207177 2.748408 0.663383
H 2.046788 1.580791 -1.580491
I 1.174526 -0.993509 -0.052187
Cl 3.306197 0.068428 0.712080
N -1.402084 0.191846 1.076410
C -1.870199 0.669184 2.371258
H -1.104491 1.294271 2.826705
H -2.801062 1.246871 2.314847
H -2.045625 -0.188426 3.019617
C -2.411532 -0.641955 0.408771
H -2.870701 -1.227397 1.207840
C -3.512248 0.159053 -0.287549
H -4.279870 -0.520937 -0.645826
H -3.965412 0.875579 0.399287
H -3.110997 0.703915 -1.142725
C -1.894413 -1.696863 -0.572197
O -2.707981 -2.454390 -1.048097
O -0.627165 -1.754129 -0.915492

IBMeUreaMe_Cl.log
Energy (E) = -1004.68689816 Hartree
Enthalpy (H) = -1004.491265 Hartree
Gibbs free energy (G) = -1004.548417 Hartree
Charge = 0, Spin = 1
C -0.551220 3.301891 0.886724
C 0.771924 3.549709 0.548317
C 1.585671 2.537335 0.067120
C 1.101359 1.235608 -0.098869
C -0.234183 1.036105 0.241117
C -1.063234 2.022331 0.729092
H -1.185575 4.092678 1.259483
H 1.181917 4.543532 0.664202

H 2.616327 2.747625 -0.178883
H -2.098714 1.799910 0.943831
I -0.953395 -0.915336 -0.034040
Cl -3.217867 0.064418 -0.792567
C 2.041344 -1.036573 -0.025570
O 3.050291 -1.702244 -0.167930
N 0.971429 -1.418587 0.732100
C 1.070568 -2.728649 1.352954
H 2.015177 -2.798827 1.887787
H 0.253041 -2.846375 2.063293
H 1.043044 -3.544452 0.626024
N 1.922172 0.226965 -0.624784
C 3.061714 0.622877 -1.439944
H 2.755665 1.424243 -2.108611
H 3.908980 0.951898 -0.834707
H 3.382642 -0.237033 -2.018068

IBMeUreaPh_Cl.log
Energy (E) = -1196.25325276 Hartree
Enthalpy (H) = -1196.001788 Hartree
Gibbs free energy (G) = -1196.066971 Hartree
Charge = 0, Spin = 1
C -3.645941 0.183895 1.834383
C -3.669147 1.549475 1.583736
C -2.722848 2.139612 0.762297
C -1.718667 1.378748 0.154847
C -1.741858 0.013946 0.422989
C -2.664514 -0.598107 1.243892
H -4.386723 -0.274275 2.473212
H -4.430822 2.168344 2.037643
H -2.750143 3.205664 0.589736
H -2.637417 -1.669180 1.384968
I -0.231820 -1.097736 -0.508974
Cl -1.911265 -2.996822 -0.878953
C 0.605263 1.813769 -0.542517
O 1.360778 2.711436 -0.858666
N 0.969413 0.616413 0.009840
N -0.784371 1.973128 -0.708929
C -1.164652 3.219878 -1.360140
H -2.196761 3.139735 -1.693595
H -1.054297 4.082360 -0.700012
H -0.513807 3.373851 -2.214271
C 2.323484 0.315512 0.314317
C 2.571375 -0.422520 1.470498
C 3.385193 0.676908 -0.512616
C 3.864358 -0.794091 1.802880
H 1.736845 -0.689853 2.106919
C 4.678973 0.311629 -0.166522
H 3.193587 1.254556 -1.403352
C 4.924939 -0.424152 0.985176
H 4.043959 -1.365105 2.703641
H 5.500414 0.602173 -0.807554
H 5.935876 -0.707554 1.244092

IBNMeCO2_Cl.log
Energy (E) = -985.291888163 Hartree
Enthalpy (H) = -985.138317 Hartree
Gibbs free energy (G) = -985.191370 Hartree
Charge = 0, Spin = 1
C 0.225096 3.280679 0.542627
C 1.574696 3.175188 0.234210
C 2.140907 1.951145 -0.080068
C 1.373404 0.781405 -0.105978
C 0.022083 0.937080 0.194064
C -0.563796 2.140647 0.523019
H -0.214860 4.235812 0.788868
H 2.201137 4.056391 0.245473
H 3.196248 1.888551 -0.301433
H -1.623332 2.191055 0.728991
I -1.117051 -0.817996 0.143281
Cl -3.050121 0.433140 -0.810188
C 1.735690 -1.619737 0.290514
O 2.535102 -2.522003 0.254525
N 1.944419 -0.446891 -0.461154
C 3.168430 -0.433434 -1.250373
H 3.083346 0.324633 -2.025901
H 4.050823 -0.236648 -0.638148
H 3.290069 -1.411476 -1.702764
O 0.640190 -1.628936 1.023503

ISamideMe_Cl.log
Energy (E) = -1345.24363758 Hartree
Enthalpy (H) = -1345.093915 Hartree
Gibbs free energy (G) = -1345.148016 Hartree
Charge = 0, Spin = 1
C 0.280557 3.354391 -0.328116
C 1.650931 3.186509 -0.174238
C 2.183673 1.922828 0.046402
C 1.323204 0.839785 0.109299
C -0.030275 1.027802 -0.065922
C -0.588996 2.266309 -0.275675
H -0.126671 4.340423 -0.502062
H 2.307163 4.043652 -0.226868
H 3.245841 1.759572 0.168875
H -1.656263 2.384853 -0.394305
I -1.150501 -0.792080 -0.016234
Cl -3.297601 0.554960 0.287782
N 0.798966 -1.649907 -0.421656
C 1.096480 -1.660155 -1.858012
H 1.068593 -0.658094 -2.302078
H 2.085060 -2.085093 -2.014486
H 0.362263 -2.288887 -2.356371
S 1.883837 -0.806664 0.468726
O 1.606137 -1.050285 1.854737
O 3.209136 -0.964635 -0.064116

ISamidePh_Cl.log
Energy (E) = -1536.80852599 Hartree
Enthalpy (H) = -1536.602985 Hartree
Gibbs free energy (G) = -1536.665912 Hartree
Charge = 0, Spin = 1
C -2.616775 1.979399 -1.878295
C -1.945753 3.090796 -1.384313
C -0.983032 2.946610 -0.393453
C -0.706712 1.677593 0.087150
C -1.372745 0.587763 -0.428867
C -2.337985 0.699714 -1.401912
H -3.362506 2.097787 -2.651695
H -2.173379 4.073516 -1.772365
H -0.442759 3.791834 0.011350
H -2.854778 -0.170363 -1.779734
I -0.737133 -1.274701 0.399246
Cl -2.813916 -2.398799 -0.533630
N 1.011838 -0.076477 0.975078
S 0.452766 1.400588 1.402160
O -0.332368 1.228702 2.591160
O 1.483816 2.396625 1.333356
C 2.105558 -0.206037 0.068047
C 2.966413 -1.278091 0.285109
C 2.316554 0.640466 -1.018365
C 4.023711 -1.512646 -0.580331
H 2.796189 -1.912200 1.145243
C 3.385613 0.409714 -1.871631
H 1.661921 1.484039 -1.188363
C 4.237946 -0.666539 -1.660657
H 4.686278 -2.348795 -0.403816
H 3.551348 1.075412 -2.707643
H 5.066837 -0.841634 -2.332439

CF3_02.log
Energy (E) = -337.417524124 Hartree
Enthalpy (H) = -337.400615 Hartree
Gibbs free energy (G) = -337.431653 Hartree
Charge = 0, Spin = 2
C -0.000037 -0.000168 0.320601
F 1.146978 -0.500228 -0.071249
F -1.006817 -0.742911 -0.071263
F -0.140136 1.243251 -0.071222

IBamideAc_CF3.log
Energy (E) = -900.697141195 Hartree
Enthalpy (H) = -900.522869 Hartree
Gibbs free energy (G) = -900.586965 Hartree
Charge = 0, Spin = 1
C 1.002626 3.500477 0.000028
C -0.306737 3.967271 0.000016
C -1.361777 3.071448 0.000040
C -1.124039 1.700152 0.000063
C 0.187408 1.275802 0.000060
C 1.268086 2.136284 0.000051
H 1.831078 4.194915 0.000024
H -0.499518 5.030911 -0.000010
H -2.396341 3.387489 0.000042
H 2.288777 1.792330 0.000072
C -2.302751 0.758135 0.000097
O -3.427190 1.208974 0.000163
I 0.407532 -0.835854 0.000069
N -1.891457 -0.543785 0.000203
C -2.103678 -2.971456 0.000350
H -2.867604 -3.742289 0.000427
H -1.475571 -3.090748 -0.884936
H -1.475695 -3.090398 0.885769
C -2.781758 -1.615947 0.000020
O -3.984336 -1.516835 -0.000378
C 2.660296 -0.720339 -0.000203
F 3.186328 -0.139091 1.077453
F 3.185929 -0.138769 -1.077881
F 3.039078 -2.002534 -0.000446

IBamideMe_CF3.log
Energy (E) = -787.433448086 Hartree
Enthalpy (H) = -787.270343 Hartree
Gibbs free energy (G) = -787.330666 Hartree
Charge = 0, Spin = 1
C -0.897067 3.250871 0.000394
C -2.271941 3.044527 0.000321
C -2.781659 1.756494 0.000156
C -1.921545 0.664428 0.000039
C -0.563282 0.906180 0.000080
C -0.017178 2.173950 0.000274
H -0.495959 4.254794 0.000551
H -2.942656 3.892478 0.000400
H -3.842881 1.546114 0.000111
H 1.045703 2.350612 0.000355
C -2.476335 -0.731621 -0.000103
O -3.686043 -0.930551 0.000013
I 0.600922 -0.869562 -0.000163
N -1.494898 -1.635286 0.000144
C -1.792979 -3.047253 0.000225
H -1.385620 -3.540580 0.885905
H -2.875433 -3.173028 -0.000316
H -1.384698 -3.540840 -0.884887
C 2.550609 0.301712 -0.000086

F 2.755921 1.064964 1.077706
F 2.755800 1.065102 -1.077799
F 3.489787 -0.655298 -0.000172

IBamideMe_O_CF3.log
Energy (E) = -787.424789391 Hartree
Enthalpy (H) = -787.261956 Hartree
Gibbs free energy (G) = -787.320733 Hartree
Charge = 0, Spin = 1
C 0.482563 3.290419 0.000668
C 1.871632 3.199197 0.000638
C 2.491936 1.963069 0.000438
C 1.732115 0.793964 0.000219
C 0.361367 0.927682 0.000248
C -0.298014 2.142156 0.000505
H -0.002899 4.256277 0.000815
H 2.468233 4.100886 0.000804
H 3.567087 1.848424 0.000427
H -1.372344 2.223693 0.000538
C 2.361518 -0.557921 -0.000058
I -0.640310 -0.955391 0.000047
O 1.509173 -1.553050 -0.000384
N 3.632525 -0.641287 0.000366
C 4.164595 -1.988846 0.000287
H 5.251848 -1.954469 0.001374
H 3.828547 -2.552069 0.876164
H 3.830342 -2.551159 -0.876865
C -2.664302 -0.014120 -0.000609
F -2.919694 0.725855 -1.077678
F -2.920044 0.725942 1.076327
F -3.510919 -1.046917 -0.000786

IBamideOMe_CF3.log
Energy (E) = -862.550823915 Hartree
Enthalpy (H) = -862.382405 Hartree
Gibbs free energy (G) = -862.443944 Hartree
Charge = 0, Spin = 1
C -0.309057 3.472634 0.282341
C 1.048568 3.711675 0.106072
C 1.923463 2.652100 -0.070300
C 1.451083 1.345160 -0.074328
C 0.090822 1.144014 0.084950
C -0.808030 2.174386 0.273342
H -0.994432 4.296219 0.425834
H 1.421104 4.726517 0.110570
H 2.987410 2.790653 -0.210424
H -1.863144 2.004895 0.412932
C 2.412429 0.206745 -0.260077
O 3.575994 0.376357 -0.589056
I -0.484908 -0.898233 0.004616
N 1.778221 -0.940379 0.026805
O 2.403803 -2.120940 -0.322051
C 3.324624 -2.493620 0.691052
H 3.747629 -3.447007 0.383624
H 4.113771 -1.746413 0.774500
H 2.813316 -2.605732 1.650029
C -2.686927 -0.379101 -0.068033
F -3.030513 0.416358 -1.080838
F -3.177649 0.156678 1.053409
F -3.291091 -1.559414 -0.248195

IBamidePh_CF3.log
Energy (E) = -979.003966207 Hartree
Enthalpy (H) = -978.784800 Hartree
Gibbs free energy (G) = -978.851071 Hartree
Charge = 0, Spin = 1
C 3.310171 2.446764 -0.139203
C 2.474560 3.557158 -0.103824
C 1.100155 3.391508 -0.067709
C 0.546209 2.115614 -0.065138
C 1.406553 1.036321 -0.088077
C 2.780958 1.161063 -0.132539
H 4.383500 2.571539 -0.171845
H 2.900054 4.550979 -0.107929
H 0.411665 4.225525 -0.043728
H 3.441564 0.310345 -0.163936
C -0.948399 1.956463 -0.046115
O -1.671604 2.935495 0.080918
I 0.405673 -0.834798 -0.047280
N -1.298320 0.670979 -0.213647
C -2.608852 0.184622 -0.114966
C -2.941415 -0.965521 -0.835832
C -3.576417 0.771334 0.706666
C -4.204200 -1.530271 -0.729320
H -2.210968 -1.393659 -1.512322
C -4.838220 0.205755 0.798132
H -3.332005 1.671123 1.249215
C -5.160177 -0.946826 0.089679
H -4.442864 -2.419075 -1.298078
H -5.580121 0.671510 1.433387
H -6.147857 -1.379522 0.170577
C 2.342398 -1.973520 0.200121
F 3.048589 -1.631181 1.277449
F 3.160153 -1.926202 -0.854595
F 1.950979 -3.243517 0.351304

IBCH2CH2CH2CO2_CF3.log
Energy (E) = -885.860248737 Hartree
Enthalpy (H) = -885.649530 Hartree
Gibbs free energy (G) = -885.711530 Hartree
Charge = 0, Spin = 1
C 0.669959 2.945905 1.040983
C 1.738472 3.132159 0.173533
C 2.082159 2.139170 -0.726576
C 1.380653 0.931793 -0.812930
C 0.307748 0.806008 0.057632
C -0.051832 1.765067 0.989267
H 0.405458 3.703315 1.764782
H 2.311438 4.048452 0.207515
H 2.925159 2.278584 -1.393229
H -0.865473 1.592990 1.677531
I -0.875617 -0.969551 0.084020
C 3.164472 -0.824891 -1.262693
H 3.191820 -1.811025 -1.724180
H 4.035798 -0.278105 -1.629964
C 1.879194 -0.119466 -1.763619
H 2.091708 0.362231 -2.719663
H 1.108374 -0.863556 -1.941906
C 3.297029 -0.961920 0.255517
H 3.623310 -0.028491 0.715671
H 4.055961 -1.708916 0.493608
C 1.995976 -1.345575 0.943820
O 1.749184 -0.987311 2.082657
O 1.168294 -1.984752 0.164710
C -2.662107 0.317908 -0.269437
F -2.443039 1.237869 -1.201189
F -3.102718 0.937110 0.825490
F -3.634914 -0.491613 -0.694875

IBCH2CH2CO2_CF3.log
Energy (E) = -846.582217814 Hartree
Enthalpy (H) = -846.401486 Hartree
Gibbs free energy (G) = -846.461850 Hartree
Charge = 0, Spin = 1
C 0.809759 2.970041 0.947715
C 1.899576 3.101515 0.097696
C 2.262340 2.056419 -0.733824
C 1.553054 0.852740 -0.770466
C 0.458790 0.778365 0.080154
C 0.082427 1.790983 0.946777
H 0.527950 3.771417 1.615466
H 2.476410 4.015862 0.093300
H 3.127802 2.150441 -1.378193
H -0.757815 1.670033 1.613906
I -0.762430 -0.964676 0.119601
C 3.056604 -1.112678 -0.736437
H 3.374353 -1.995389 -1.291274
C 2.301593 -1.532648 0.517943
O 2.692070 -1.242686 1.630427
O 1.180407 -2.142954 0.232583
H 3.929471 -0.525302 -0.454908
C 2.088311 -0.282295 -1.595544
H 2.600855 0.124407 -2.467139
H 1.285913 -0.929578 -1.948098
C -2.531415 0.349556 -0.220291
F -2.299775 1.284443 -1.132776
F -2.975045 0.950508 0.884047
F -3.505426 -0.446168 -0.665530

IBCH2CH2O_CF3.log
Energy (E) = -811.859530548 Hartree
Enthalpy (H) = -811.633081 Hartree
Gibbs free energy (G) = -811.694332 Hartree
Charge = 0, Spin = 1
C 0.031199 3.365934 -0.546989
C -1.188740 3.546557 0.090566
C -1.864178 2.462249 0.632236
C -1.341050 1.172605 0.558361
C -0.123648 1.037360 -0.096329
C 0.574216 2.091763 -0.650777
H 0.560906 4.208462 -0.968721
H -1.616854 4.536720 0.165347
H -2.815141 2.605192 1.130157
H 1.516364 1.934720 -1.155457
I 0.571907 -0.941375 -0.323733
C -2.043289 -0.011932 1.137186
H -1.363259 -0.529662 1.822357
H -2.905988 0.308778 1.722336
O -1.488004 -1.208873 -0.901421
C -2.490764 -1.024875 0.053544
C -2.824923 -2.345298 0.737421
H -3.593512 -2.221327 1.503664
H -3.177484 -3.060288 -0.005719
H -1.927322 -2.753162 1.209301
C -3.709279 -0.476802 -0.680107
H -3.980247 -1.163643 -1.481255
H -4.561521 -0.361897 -0.006238
H -3.468628 0.489708 -1.125526
C 2.621851 -0.199475 0.310352
F 2.585294 0.659660 1.331035
F 3.329236 0.379601 -0.672128
F 3.316281 -1.276846 0.706223

IBCH2CH2S_CF3.log
Energy (E) = -1134.78970160 Hartree
Enthalpy (H) = -1134.564454 Hartree
Gibbs free energy (G) = -1134.627637 Hartree
Charge = 0, Spin = 1
C 0.359856 3.314844 -0.724272
C -0.807740 3.608111 -0.033598
C -1.519449 2.597907 0.596349
C -1.094437 1.270203 0.556185
C 0.076669 1.025405 -0.147533

C 0.812463 2.003678 -0.787670
H 0.920017 4.097636 -1.215905
H -1.165379 4.627290 0.015542
H -2.428711 2.827974 1.138296
H 1.717051 1.758962 -1.324774
I 0.702645 -0.990190 -0.313110
C -1.879533 0.189585 1.233098
H -1.204039 -0.478534 1.774043
H -2.535495 0.646493 1.979683
C -2.759245 -0.660573 0.299581
C -3.353717 -1.797273 1.120275
H -3.949292 -1.400667 1.948636
H -3.995384 -2.418401 0.495669
H -2.563393 -2.429764 1.526885
C -3.873201 0.199972 -0.286402
H -4.496410 -0.395823 -0.953639
H -4.504530 0.597245 0.515140
H -3.462437 1.029694 -0.861790
S -1.802990 -1.337122 -1.102501
C 2.770757 -0.248887 0.418328
F 2.695602 0.623482 1.425946
F 3.530872 0.306937 -0.537486
F 3.426984 -1.331074 0.859431

IBCH2CO2_CF3.log
Energy (E) = -807.314950913 Hartree
Enthalpy (H) = -807.164084 Hartree
Gibbs free energy (G) = -807.221148
Hartree
Charge = 0, Spin = 1
C -0.763320 3.156993 -0.679199
C -2.004090 3.135466 -0.055763
C -2.484340 1.967116 0.514718
C -1.741024 0.786297 0.492704
C -0.508245 0.859389 -0.137668
C 0.000002 1.999214 -0.730371
H -0.388821 4.063944 -1.131808
H -2.605110 4.033446 -0.020487
H -3.456075 1.951133 0.990677
H 0.960084 1.996960 -1.224870
I 0.597053 -0.939529 -0.238232
C -2.276616 -0.479783 1.081286
H -1.574786 -0.870287 1.826171
H -3.227460 -0.302420 1.574364
C -2.472179 -1.581399 0.024407
O -3.521440 -2.180134 -0.038825
O -1.440399 -1.779852 -0.741982
C 2.448798 0.181781 0.316267
F 2.238905 1.072096 1.278254
F 3.012143 0.813835 -0.715408
F 3.321080 -0.724634 0.759422

IBCH2CONMe_CF3.log
Energy (E) = -826.700525811 Hartree
Enthalpy (H) = -826.507871 Hartree
Gibbs free energy (G) = -826.570037
Hartree
Charge = 0, Spin = 1
C -0.115132 3.270244 0.954708
C 1.051412 3.636857 0.298182
C 1.773686 2.700075 -0.425708
C 1.353634 1.373659 -0.522252
C 0.182622 1.054469 0.149490
C -0.560581 1.956953 0.884158
H -0.678037 3.995018 1.525410
H 1.404320 4.657326 0.353817
H 2.685776 2.987494 -0.932368
H -1.461678 1.655905 1.397348
I -0.441933 -0.966373 0.055838
C 2.153600 0.360792 -1.282775
H 1.530916 -0.097537 -2.058433
H 3.003902 0.831237 -1.767901
C 2.692527 -0.755429 -0.384215
O 3.880862 -1.045529 -0.383509
N 1.752184 -1.340290 0.373546
C 2.181237 -2.411904 1.248576
H 2.923176 -2.054453 1.964495
H 1.323822 -2.799409 1.802465
H 2.638082 -3.230750 0.689074
C -2.571918 -0.217722 -0.264851
F -2.663066 0.804459 -1.116182
F -3.213595 0.137092 0.858629
F -3.246290 -1.251414 -0.788221

IBCH2CONPh_CF3.log
Energy (E) = -1018.27039028 Hartree
Enthalpy (H) = -1018.021757 Hartree
Gibbs free energy (G) = -1018.090854
Hartree
Charge = 0, Spin = 1
C -3.043354 1.859350 1.795265
C -2.783808 3.086626 1.200655
C -1.854826 3.189108 0.176157
C -1.161508 2.072564 -0.290878
C -1.454721 0.869721 0.333293
C -2.365843 0.727732 1.361350
H -3.762498 1.776543 2.597640
H -3.304813 3.971138 1.539932
H -1.651888 4.148293 -0.282110
H -2.547872 -0.231724 1.822609
I -0.350718 -0.812042 -0.314962
C -0.150701 2.191069 -1.388043
H -0.413225 1.515477 -2.209613
H -0.135378 3.202410 -1.784159
C 1.283285 1.860686 -0.955208
O 2.190598 2.616284 -1.264007
N 1.377299 0.719466 -0.235714
C 2.614529 0.178283 0.164117
C 3.772664 0.263895 -0.614206
C 2.666960 -0.524053 1.370647
C 4.944349 -0.339786 -0.183515
H 3.747346 0.820150 -1.538237
C 3.840809 -1.130725 1.791870
H 1.778103 -0.566708 1.988633
C 4.988066 -1.041797 1.015278
H 5.834428 -0.260514 -0.793908
H 3.859951 -1.665060 2.732555
H 5.906859 -1.509582 1.341683
C -2.276634 -2.003155 -0.330943
F -3.302788 -1.329834 -0.845667
F -2.647504 -2.450983 0.874487
F -2.050201 -3.073163 -1.099377

IBCH2S_CF3.log
Energy (E) = -1095.51482850 Hartree
Enthalpy (H) = -1095.319113 Hartree
Gibbs free energy (G) = -1095.378816
Hartree
Charge = 0, Spin = 1
C -0.515951 3.234252 -0.438915
C -1.868543 3.150691 -0.152848
C -2.458011 1.918760 0.097291
C -1.721301 0.734994 0.067745
C -0.369646 0.872576 -0.207581
C 0.251916 2.076098 -0.464755
H -0.052664 4.186497 -0.654627
H -2.474836 4.045843 -0.132669
H -3.515388 1.874279 0.315019
H 1.301446 2.130322 -0.707019
I 0.764465 -0.939042 -0.285961
C -2.359638 -0.614374 0.367550
S -1.641135 -1.846272 -0.758813
C -3.862962 -0.611990 0.107741
H -4.238442 -1.629255 0.214277
H -4.389632 0.013362 0.831833
H -4.086252 -0.265004 -0.900572
C -2.113396 -0.973395 1.834799
H -2.575897 -0.227169 2.485594
H -2.549440 -1.949451 2.046045
H -1.050255 -1.015905 2.070512
C 2.660235 0.254793 0.318951
F 2.500247 1.073436 1.362059
F 3.261504 0.957025 -0.654094
F 3.517878 -0.709168 0.686330

IBMeAlaO_CF3.log
Energy (E) = -941.155207138 Hartree
Enthalpy (H) = -940.927595 Hartree
Gibbs free energy (G) = -940.994939
Hartree
Charge = 0, Spin = 1
C -0.046446 3.055132 -1.379978
C -1.168479 3.308465 -0.601728
C -1.687239 2.328585 0.232540
C -1.094949 1.068099 0.313833
C 0.044656 0.871761 -0.456816
C 0.577056 1.815996 -1.311687
H 0.348157 3.816353 -2.037702
H -1.650795 4.274790 -0.653733
H -2.567532 2.528700 0.829096
H 1.454073 1.602904 -1.904634
I 0.940721 -1.007606 -0.211049
N -1.563272 0.026749 1.146314
C -1.778608 0.428596 2.526740
H -0.872837 0.892749 2.916453
H -2.609900 1.130153 2.664591
H -1.992630 -0.461659 3.118090
C -2.653065 -0.794238 0.594892
H -2.791382 -1.600113 1.323192
C -3.974180 -0.051370 0.435430
H -4.745744 -0.752668 0.132325
H -4.277027 0.428726 1.367548
H -3.898872 0.703363 -0.348097
C -2.297063 -1.519454 -0.720099
O -3.223306 -1.902707 -1.408852
O -1.052500 -1.739859 -0.999922
C 2.798804 0.041108 0.405149
F 2.565907 0.968296 1.328702
F 3.435990 0.627748 -0.609093
F 3.617293 -0.876376 0.924258

IBMeUreaMe_CF3.log
Energy (E) = -882.019456725 Hartree
Enthalpy (H) = -881.808563 Hartree
Gibbs free energy (G) = -881.872271
Hartree
Charge = 0, Spin = 1
C 0.222083 3.251252 -1.074779
C -1.030949 3.549163 -0.557379
C -1.811073 2.567282 0.029703
C -1.365475 1.243058 0.133319
C -0.087305 0.994559 -0.367938
C 0.698304 1.952417 -0.976489
H 0.826160 4.013897 -1.544041
H -1.416357 4.557402 -0.623322
H -2.793248 2.817072 0.403472
H 1.675412 1.697672 -1.360037
I 0.648077 -0.956010 -0.128149
C -2.403028 -0.992678 0.112801
O -3.420984 -1.606252 0.393777

N -1.458603 -1.380750 -0.767862
C -1.687338 -2.676375 -1.377209
H -2.655406 -2.693249 -1.877246
H -0.911602 -2.863369 -2.121123
H -1.689087 -3.492505 -0.648064
N -2.154519 0.262619 0.735380
C -3.191377 0.692699 1.660448
H -2.790961 1.471974 2.306078
H -4.080973 1.064266 1.146113
H -3.490996 -0.162553 2.256228
C 2.655042 -0.071895 0.496472
F 2.559040 0.963300 1.329326
F 3.437187 0.304605 -0.525842
F 3.297710 -1.059240 1.136216

IBMeUreaPh_CF3.log
Energy (E) = -1073.58944216 Hartree
Enthalpy (H) = -1073.322556 Hartree
Gibbs free energy (G) = -1073.393999 Hartree
Charge = 0, Spin = 1
C -3.122933 1.321958 2.070272
C -2.914068 2.624417 1.637049
C -1.946541 2.914164 0.689645
C -1.152164 1.907703 0.125956
C -1.419443 0.611914 0.563653
C -2.359995 0.300305 1.524940
H -3.869213 1.098017 2.818408
H -3.501305 3.431524 2.053045
H -1.783405 3.937978 0.386004
H -2.508787 -0.722674 1.838339
I -0.290447 -0.907889 -0.335500
C 1.178885 1.805029 -0.692218
O 2.038212 2.511585 -1.191245
N 1.336887 0.669236 0.023510
N -0.185947 2.201236 -0.839653
C -0.366181 3.417940 -1.618859
H -1.415811 3.509187 -1.891176
H -0.045989 4.311131 -1.077794
H 0.240625 3.344874 -2.514919
C 2.606568 0.121872 0.291270
C 2.721080 -0.668218 1.439615
C 3.722866 0.264308 -0.538027
C 3.913145 -1.297870 1.758659
H 1.859039 -0.759832 2.089813
C 4.915913 -0.363644 -0.206608
H 3.651888 0.877809 -1.421684
C 5.021775 -1.147025 0.935177
H 3.978469 -1.899087 2.655933
H 5.773478 -0.238750 -0.854887
H 5.956630 -1.631702 1.181582
C -2.177202 -2.135519 -0.550599
F -3.222309 -1.423947 -0.962714
F -2.536096 -2.776481 0.567791
F -1.912087 -3.065169 -1.473717

IBNMeCO2_CF3.log
Energy (E) = -862.636497032 Hartree
Enthalpy (H) = -862.467376 Hartree
Gibbs free energy (G) = -862.526786 Hartree
Charge = 0, Spin = 1
C -0.374830 3.234447 -0.708129
C -1.670619 3.236432 -0.208637
C -2.279902 2.063605 0.202394
C -1.614529 0.830416 0.145836
C -0.301519 0.884004 -0.325016
C 0.317908 2.035394 -0.769284
H 0.094745 4.147653 -1.042819
H -2.225187 4.163154 -0.153671
H -3.299236 2.086932 0.558652
H 1.329207 2.006785 -1.147665
I 0.753598 -0.927215 -0.321740
C -2.226821 -1.523230 -0.258296
O -3.113855 -2.332528 -0.099864
N -2.228386 -0.345739 0.564517
C -3.357885 -0.238065 1.475317
H -3.130412 0.497741 2.244768
H -4.280502 0.041931 0.961305
H -3.517837 -1.209506 1.929526
O -1.255561 -1.588779 -1.108176
C 2.547001 0.095866 0.517738
F 2.241968 0.950512 1.484246
F 3.261766 0.746615 -0.400815
F 3.319570 -0.865446 1.025824

ISamideMe_CF3.log
Energy (E) = -1222.57899778 Hartree
Enthalpy (H) = -1222.413823 Hartree
Gibbs free energy (G) = -1222.474338 Hartree
Charge = 0, Spin = 1
C -0.405353 3.322229 0.397842
C -1.763492 3.247380 0.119755
C -2.354486 2.021477 -0.156195
C -1.570528 0.878971 -0.167282
C -0.226577 0.976416 0.114603
C 0.386915 2.176774 0.404427
H 0.050089 4.274620 0.630025
H -2.365528 4.145042 0.130173
H -3.413202 1.925186 -0.355086
H 1.434696 2.247674 0.648885
I 0.805595 -0.905384 0.119505
N -1.372065 -1.634220 0.474414
C -1.759471 -1.515076 1.878983
H -1.641909 -0.494684 2.269893
H -2.799683 -1.808808 2.011724
H -1.133168 -2.183589 2.467408
S -2.254761 -0.736034 -0.518378
O -1.872822 -1.034556 -1.873557
O -3.645842 -0.683797 -0.144934
C 2.766630 0.164115 -0.199807
F 3.234954 0.788410 0.886658
F 2.751710 1.041408 -1.198631
F 3.631147 -0.806585 -0.506878

ISamidePh_CF3.log
Energy (E) = -1414.14779114 Hartree
Enthalpy (H) = -1413.926643 Hartree
Gibbs free energy (G) = -1413.995276 Hartree
Charge = 0, Spin = 1
C 1.084187 2.711398 2.130816
C 0.070610 3.501574 1.606463
C -0.658616 3.065897 0.507869
C -0.354190 1.842452 -0.066997
C 0.657831 1.078269 0.468230
C 1.390477 1.475540 1.566264
H 1.638705 3.042202 2.997609
H -0.160655 4.453798 2.062599
H -1.468641 3.646970 0.087779
H 2.162454 0.861166 2.001224
I 0.950589 -0.811546 -0.505355
N -1.313979 -0.343483 -1.128782
S -1.254051 1.219484 -1.480707
O -0.352666 1.375447 -2.592962
O -2.532824 1.880586 -1.492985
C -2.212286 -0.854604 -0.165601
C -2.361074 -2.245052 -0.150725
C -2.902895 -0.097363 0.784266
C -3.165946 -2.865654 0.789236
H -1.844721 -2.822376 -0.907744
C -3.716417 -0.727026 1.717191
H -2.832465 0.980331 0.780748
C -3.850405 -2.107889 1.732342
H -3.268131 -3.942612 0.778103
H -4.252062 -0.124939 2.439176
H -4.486675 -2.587768 2.463021
C 3.047022 -0.927288 0.277734
F 3.119379 -1.225448 1.575649
F 3.749160 0.178464 0.067749
F 3.610101 -1.925462 -0.401269

CCTMS_02.log
Energy (E) = -485.013591030 Hartree
Enthalpy (H) = -484.884456 Hartree
Gibbs free energy (G) = -484.929443 Hartree
Charge = 0, Spin = 2
C 2.814807 0.000145 0.000296
C 1.602841 0.000218 0.000390
Si -0.242888 -0.000004 0.000011
C -0.814964 1.092466 -1.394415
H -1.905494 1.122701 -1.432832
H -0.452137 2.112557 -1.266031
H -0.452302 0.723622 -2.354175
C -0.815701 0.661165 1.643128
H -1.906255 0.678893 1.688125
H -0.452907 0.040134 2.462521
H -0.453490 1.676920 1.803623
C -0.814873 -1.753860 -0.249151
H -1.905405 -1.802235 -0.256565
H -0.451685 -2.152737 -1.196621
H -0.452554 -2.400610 0.550316

IBamideAc_CCTMS.log
Energy (E) = -1048.34998675 Hartree
Enthalpy (H) = -1048.062744 Hartree
Gibbs free energy (G) = -1048.143250 Hartree
Charge = 0, Spin = 1
C 0.309922 3.558006 -0.000196
C 1.658766 3.896952 -0.000103
C 2.625935 2.905849 0.000025
C 2.260649 1.562711 0.000070
C 0.913890 1.271542 -0.000021
C -0.082216 2.224363 -0.000153
H -0.447067 4.329976 -0.000302
H 1.951885 4.937604 -0.000132
H 3.685445 3.124398 0.000101
H -1.126750 1.947841 -0.000222
C 3.338320 0.506093 0.000225
O 4.502700 0.843757 0.000087
I 0.461215 -0.802115 0.000071
N 2.793893 -0.746152 0.000038
C 2.764249 -3.179042 -0.000418
H 3.445466 -4.024008 -0.000573
H 2.126963 -3.233424 0.884858
H 2.127048 -3.233006 -0.885779
C 3.575622 -1.897293 -0.000070
O 4.783070 -1.924860 0.000111
C -1.606565 -0.406003 0.000041
C -2.818773 -0.331717 0.000030
Si -4.662883 -0.221022 0.000014
C -5.185093 0.697959 -1.532467
C -5.185481 0.693718 1.534891
C -5.329902 -1.958766 -0.002460
H -4.852064 0.180398 -2.432480
H -6.272582 0.782593 -1.573519
H -4.768576 1.705647 -1.549208

H -6.272984 0.778198 1.575920
H -4.852646 0.173697 2.433557
H -4.768998 1.701367 1.554504
H -6.421377 -1.946300 -0.002595
H -4.998435 -2.505256 -0.885784
H -4.998686 -2.507657 0.879468

IBamideMe_CCTMS.log
Energy (E) = -935.085064286 Hartree
Enthalpy (H) = -934.808991 Hartree
Gibbs free energy (G) = -934.885388 Hartree
Charge = 0, Spin = 1
C -0.997152 3.261001 -0.000195
C -2.377193 3.434849 -0.000203
C -3.219927 2.334578 -0.000177
C -2.690726 1.048972 -0.000147
C -1.317694 0.919006 -0.000139
C -0.444569 1.984413 -0.000161
H -0.340027 4.119686 -0.000214
H -2.792245 4.433342 -0.000228
H -4.298251 2.422895 -0.000179
H 0.625756 1.834575 -0.000152
C -3.597008 -0.149382 -0.000123
O -4.816918 -0.016510 -0.000078
I -0.654507 -1.095058 -0.000085
N -2.888977 -1.280179 -0.000138
C -3.565032 -2.555880 -0.000082
H -3.308108 -3.143226 0.885038
H -4.641018 -2.382564 -0.000552
H -3.307378 -3.143650 -0.884706
C 1.385276 -0.452524 -0.000071
C 2.583220 -0.245046 -0.000059
Si 4.399085 0.066367 0.000293
C 4.827401 1.036050 1.532179
C 4.828001 1.036002 -1.531454
C 5.263074 -1.583858 0.000486
H 4.554953 0.485124 2.432841
H 5.899495 1.238175 1.569569
H 4.303473 1.992346 1.551128
H 5.900111 1.238125 -1.568431
H 4.555905 0.485049 -2.432206
H 4.304082 1.992299 -1.550637
H 6.346122 -1.447454 0.000699
H 4.995316 -2.165766 0.882903
H 4.995666 -2.165789 -0.882022

IBamideMe_O_CCTMS.log
Energy (E) = -935.077687582 Hartree
Enthalpy (H) = -934.801931 Hartree
Gibbs free energy (G) = -934.876481 Hartree
Charge = 0, Spin = 1
C 0.924398 3.204922 0.000033
C 2.308838 3.357248 -0.000188
C 3.138864 2.249798 -0.000409
C 2.597316 0.964783 -0.000420
C 1.224534 0.862928 -0.000201
C 0.359277 1.936271 0.000029
H 0.279282 4.072563 0.000205
H 2.738039 4.349840 -0.000183
H 4.217274 2.328749 -0.000582
H -0.712417 1.796961 0.000203
C 3.443712 -0.263917 -0.000660
I 0.537202 -1.153411 -0.000231
O 2.766205 -1.382947 -0.000606
N 4.711866 -0.131324 -0.000766
C 5.458974 -1.372296 -0.000903
H 6.526059 -1.159498 -0.000820
H 5.221772 -1.983848 0.875258
H 5.221870 -1.983571 -0.877284
C -1.458197 -0.539921 0.000137
C -2.648943 -0.304360 0.000351
Si -4.461204 0.049112 0.000844
C -4.858874 1.027132 -1.532651
C -4.858058 1.027061 1.534595
C -5.352908 -1.584959 0.001046
H -4.598807 0.467954 -2.431834
H -5.925257 1.256680 -1.573052
H -4.310808 1.969729 -1.551724
H -5.924421 1.256600 1.575579
H -4.597505 0.467843 2.433613
H -4.309989 1.969661 1.553417
H -6.433178 -1.428435 0.001338
H -5.096768 -2.171625 -0.881577
H -5.096297 -2.171661 0.883508

IBamideOMe_TMSCC.log
Energy (E) = -1010.20318147 Hartree
Enthalpy (H) = -1009.921805 Hartree
Gibbs free energy (G) = -1010.000619 Hartree
Charge = 0, Spin = 1
C 0.479195 3.379698 0.186749
C 1.831614 3.693047 0.114624
C 2.775750 2.685418 -0.004849
C 2.381053 1.353459 -0.051326
C 1.025496 1.082245 0.019838
C 0.057131 2.055077 0.137322
H -0.258744 4.164481 0.279273
H 2.146598 4.726765 0.150994
H 3.837487 2.884106 -0.069520
H -0.992304 1.800699 0.186898
C 3.416818 0.273386 -0.192189
O 4.606039 0.522597 -0.336961
I 0.533857 -0.980757 -0.081455
N 2.803576 -0.910816 -0.110468
O 3.543250 -2.052090 -0.346301
C 4.348162 -2.353698 0.782266
H 4.864791 -3.282094 0.550150
H 5.071884 -1.557256 0.954060
H 3.724765 -2.489738 1.670060
C -1.537007 -0.538317 -0.036062
C -2.743849 -0.400982 -0.012849
Si -4.568914 -0.138182 0.020622
C -5.286747 -0.798109 -1.565051
C -4.855150 1.697380 0.168145
C -5.265091 -1.040342 1.492864
H -5.087095 -1.864772 -1.671590
H -6.368505 -0.653388 -1.584372
H -4.861184 -0.287578 -2.429416
H -5.923523 1.919489 0.191265
H -4.409808 2.090083 1.083104
H -4.418825 2.229722 -0.677952
H -6.346419 -0.902332 1.549365
H -5.064642 -2.110185 1.427590
H -4.827684 -0.671254 2.420964

IBamidePh_CCTMS.log
Energy (E) = -1126.65612839 Hartree
Enthalpy (H) = -1126.324037 Hartree
Gibbs free energy (G) = -1126.406395 Hartree
Charge = 0, Spin = 1
C 1.057999 3.749583 -0.115458
C -0.167595 4.404722 -0.061741
C -1.346448 3.677723 -0.025287
C -1.315229 2.287000 -0.045670
C -0.079169 1.675361 -0.094921
C 1.116113 2.360194 -0.132008
H 1.978612 4.315998 -0.142320
H -0.198957 5.485430 -0.047676
H -2.320317 4.146634 0.019345
H 2.062721 1.839650 -0.167710
C -2.600085 1.507346 -0.006912
O -3.668994 2.089699 0.129567
I -0.167373 -0.444357 -0.101322
N -2.368219 0.194019 -0.159920
C -3.355560 -0.794209 -0.056028
C -3.182517 -1.980971 -0.774559
C -4.478086 -0.665070 0.768787
C -4.092941 -3.021865 -0.661157
H -2.344371 -2.067593 -1.456295
C -5.386804 -1.706543 0.868372
H -4.630352 0.255837 1.309505
C -5.201336 -2.890608 0.162898
H -3.940738 -3.930085 -1.229233
H -6.252383 -1.591532 1.507675
H -5.917169 -3.696543 0.249409
C 1.941525 -0.565844 -0.004243
C 3.139493 -0.759473 0.048974
Si 4.966183 -1.000792 0.132772
C 5.364512 -1.923738 1.699430
C 5.742748 0.693138 0.140596
C 5.498241 -1.970115 -1.364910
H 4.878406 -2.899694 1.713424
H 6.441079 -2.081402 1.786948
H 5.031043 -1.368433 2.576519
H 6.830367 0.617318 0.191705
H 5.486094 1.244937 -0.764604
H 5.404848 1.273649 0.999911
H 6.578275 -2.127941 -1.354030
H 5.014910 -2.947208 -1.391157
H 5.242423 -1.442513 -2.284209

IBCH2CH2CH2CO2_CCTMS.log
Energy (E) = -1033.50994848 Hartree
Enthalpy (H) = -1033.186224 Hartree
Gibbs free energy (G) = -1033.264803 Hartree
Charge = 0, Spin = 1
C -0.719859 2.860447 -1.122053
C -1.705484 3.374479 -0.288154
C -2.320639 2.556870 0.642095
C -1.991194 1.203496 0.785342
C -0.997376 0.744148 -0.064881
C -0.360883 1.527893 -1.011190
H -0.239410 3.484757 -1.861846
H -1.995596 4.413175 -0.366697
H -3.088838 2.957041 1.293666
H 0.399856 1.107728 -1.653364
I -0.364780 -1.304304 -0.034695
C -4.194297 0.055188 1.338676
H -4.496066 -0.852749 1.859260
H -4.865602 0.850143 1.671736
C -2.752601 0.388646 1.793231
H -2.799602 0.963248 2.720335
H -2.221968 -0.533508 2.010918
C -4.382034 -0.126006 -0.168293
H -4.431906 0.833759 -0.684090
H -5.326895 -0.634855 -0.366301
C -3.255535 -0.908564 -0.826241
O -2.950876 -0.721236 -1.992523
O -2.619843 -1.692853 -0.003074
C 1.606064 -0.632820 0.067613
C 2.779961 -0.327326 0.094319
Si 4.554603 0.170598 0.133627
C 5.026352 0.521991 1.900175
C 4.734719 1.698368 -0.916777
C 5.561700 -1.235537 -0.555914

H 4.891609 -0.361861 2.524384
H 6.073177 0.824835 1.963648
H 4.416003 1.324382 2.315645
H 5.770295 2.043370 -0.917574
H 4.443965 1.499696 -1.948855
H 4.109236 2.507717 -0.538495
H 6.623155 -0.980903 -0.554989
H 5.431140 -2.139756 0.039357
H 5.268751 -1.461403 -1.581602

IBCH2CH2CO2_CCTMS.log
Energy (E) = -994.232688323 Hartree
Enthalpy (H) = -993.938981 Hartree
Gibbs free energy (G) = -994.016507 Hartree
Charge = 0, Spin = 1
C -0.746774 2.948017 -0.999350
C -1.763481 3.441835 -0.191642
C -2.450103 2.591978 0.656591
C -2.157376 1.226661 0.743591
C -1.132235 0.784602 -0.078866
C -0.425853 1.601891 -0.944663
H -0.207587 3.599984 -1.671754
H -2.023429 4.490731 -0.226993
H -3.247265 2.975586 1.281701
H 0.368669 1.201334 -1.558291
I -0.531748 -1.266911 -0.073479
C -4.214414 -0.151577 0.818356
H -4.805798 -0.832294 1.431995
C -3.695923 -0.902766 -0.403274
O -4.120917 -0.677024 -1.518124
O -2.754485 -1.753732 -0.096499
H -4.847464 0.672513 0.491445
C -3.012823 0.359364 1.622462
H -3.345345 0.949195 2.477042
H -2.452042 -0.494416 2.001295
C 1.448198 -0.640698 0.030224
C 2.628385 -0.361520 0.060547
Si 4.412710 0.105672 0.107134
C 4.931153 0.258048 1.888360
C 4.583077 1.735867 -0.777698
C 5.379424 -1.229650 -0.757438
H 4.798341 -0.686332 2.416991
H 5.983657 0.539093 1.957404
H 4.343339 1.018441 2.403330
H 5.622000 2.070364 -0.769072
H 4.264452 1.651675 -1.817264
H 3.976639 2.505182 -0.298324
H 6.444299 -0.989841 -0.757395
H 5.250501 -2.191558 -0.260400
H 5.057409 -1.339045 -1.793382

IBCH2CH2O_CCTMS.log
Energy (E) = -959.508545398 Hartree
Enthalpy (H) = -959.169114 Hartree
Gibbs free energy (G) = -959.246812 Hartree
Charge = 0, Spin = 1
C -0.580070 3.258122 -0.661356
C -1.722652 3.595518 0.052260
C -2.480948 2.610093 0.667765
C -2.125583 1.264272 0.586661
C -0.986274 0.973599 -0.151633
C -0.202092 1.925976 -0.768955
H 0.017393 4.024053 -1.135727
H -2.021905 4.631539 0.133692
H -3.365246 2.875942 1.233674
H 0.688394 1.639900 -1.311732
I -0.534417 -1.075888 -0.386263
C -2.912349 0.187974 1.262475
H -2.241174 -0.389948 1.907709
H -3.676586 0.628043 1.904606
O -2.657580 -1.139609 -0.743394
C -3.563516 -0.792554 0.259251
C -4.016509 -2.033296 1.020507
H -4.711576 -1.784598 1.825864
H -4.504694 -2.722905 0.331921
H -3.147826 -2.535987 1.453214
C -4.752109 -0.117103 -0.415855
H -5.164495 -0.792347 -1.165020
H -5.533173 0.133293 0.306058
H -4.423075 0.792679 -0.920425
C 1.531262 -0.569233 -0.105110
C 2.725667 -0.384328 0.027019
Si 4.524541 -0.070550 0.229723
C 4.958704 -0.215707 2.036177
C 4.884261 1.650997 -0.390018
C 5.461657 -1.333526 -0.770385
H 4.730366 -1.213047 2.413539
H 6.023071 -0.029785 2.192271
H 4.397593 0.505561 2.631236
H 5.944519 1.886538 -0.280175
H 4.624118 1.748030 -1.444758
H 4.313228 2.393401 0.168840
H 6.537823 -1.176034 -0.676360
H 5.237239 -2.345368 -0.431217
H 5.200486 -1.267125 -1.827052

IBCH2CH2S_CCTMS.log
Energy (E) = -1282.43662003 Hartree
Enthalpy (H) = -1282.098373 Hartree
Gibbs free energy (G) = -1282.177564 Hartree
Charge = 0, Spin = 1
C -0.445983 3.224842 -0.878080
C -1.528075 3.606945 -0.096212
C -2.242597 2.658143 0.619361
C -1.912272 1.303160 0.570506
C -0.831594 0.970527 -0.233311
C -0.086468 1.885893 -0.949117
H 0.119860 3.960963 -1.431669
H -1.812463 4.648595 -0.038483
H -3.077789 2.958527 1.240482
H 0.762552 1.565874 -1.536980
I -0.345572 -1.082889 -0.419100
C -2.690133 0.294945 1.360230
H -2.004738 -0.403632 1.848481
H -3.229966 0.816339 2.156370
C -3.714321 -0.518703 0.553978
C -4.307026 -1.577312 1.474187
H -4.798039 -1.107669 2.332536
H -5.042455 -2.175556 0.936511
H -3.527264 -2.247686 1.838157
C -4.815990 0.398899 0.036051
H -5.541676 -0.174887 -0.540806
H -5.336835 0.874749 0.873560
H -4.405491 1.171778 -0.613826
S -2.934078 -1.314893 -0.892669
C 1.733075 -0.495072 -0.099775
C 2.928315 -0.330707 0.059375
Si 4.727997 -0.060834 0.308234
C 4.980598 0.797781 1.943261
C 5.367922 0.999832 -1.085515
C 5.578067 -1.719794 0.304539
H 4.595373 0.193123 2.764944
H 6.042115 0.978372 2.123327
H 4.465232 1.758662 1.962942
H 6.438790 1.179142 -0.971864
H 5.206094 0.517067 -2.049907
H 4.863933 1.966910 -1.103474
H 6.653316 -1.600401 0.450927
H 5.196233 -2.355831 1.103826
H 5.420706 -2.237634 -0.642184

IBCH2CO2_CCTMS.log
Energy (E) = -954.966185562 Hartree
Enthalpy (H) = -954.702326 Hartree
Gibbs free energy (G) = -954.775681 Hartree
Charge = 0, Spin = 1
C -0.914422 3.119074 -0.784182
C -2.082075 3.456081 -0.111496
C -2.828879 2.481432 0.531163
C -2.439551 1.140546 0.526331
C -1.272921 0.855638 -0.165784
C -0.498549 1.795480 -0.815560
H -0.328900 3.876127 -1.286258
H -2.413251 4.485085 -0.087746
H -3.736514 2.747628 1.057156
H 0.410696 1.509784 -1.326137
I -0.684269 -1.178962 -0.258831
C -3.255083 0.096343 1.220784
H -2.632023 -0.431787 1.951236
H -4.086439 0.550243 1.751824
C -3.805418 -0.966200 0.254664
O -4.977751 -1.266856 0.287385
O -2.911085 -1.469088 -0.539969
C 1.311574 -0.607688 -0.047580
C 2.496064 -0.364254 0.051997
Si 4.289884 0.043998 0.205788
C 4.765691 -0.084621 2.000449
C 4.518497 1.782678 -0.421969
C 5.246343 -1.172486 -0.828598
H 4.598087 -1.093358 2.378969
H 5.822259 0.155019 2.133527
H 4.182702 0.605884 2.610530
H 5.564784 2.083478 -0.343730
H 4.222177 1.863227 -1.468428
H 3.920566 2.488485 0.155658
H 6.316234 -0.964427 -0.769595
H 5.082285 -2.194123 -0.484699
H 4.948285 -1.116822 -1.875938

IBCH2CONMe_CCTMS.log
Energy (E) = -974.350111713 Hartree
Enthalpy (H) = -974.044432 Hartree
Gibbs free energy (G) = -974.122579 Hartree
Charge = 0, Spin = 1
C 0.552369 3.171478 0.991583
C 1.663852 3.663601 0.320837
C 2.460236 2.815544 -0.433495
C 2.177274 1.453288 -0.540530
C 1.061781 1.008346 0.153190
C 0.240095 1.821155 0.906680
H -0.071373 3.828478 1.581077
H 1.912033 4.714123 0.384488
H 3.323266 3.202171 -0.959843
H -0.628836 1.417984 1.407807
I 0.628863 -1.066583 0.053468
C 3.051265 0.543846 -1.349535
H 2.450352 0.037578 -2.112428
H 3.830128 1.110809 -1.851289
C 3.728281 -0.524247 -0.488705
O 4.944566 -0.661679 -0.490985
N 2.864393 -1.242476 0.242851
C 3.429782 -2.253813 1.112443
H 4.121457 -1.810752 1.831519
H 2.629995 -2.754166 1.662732
H 3.987622 -3.003314 0.547342

C -1.453816 -0.549435 -0.026080
C -2.656909 -0.380844 -0.073913
Si -4.471916 -0.088601 -0.145586
C -4.991308 -0.011755 -1.932852
C -4.825624 1.529744 0.709614
C -5.328713 -1.493530 0.728236
H -4.762711 -0.945904 -2.446882
H -6.065568 0.164853 -2.013224
H -4.475296 0.794587 -2.454986
H -5.893935 1.752551 0.684248
H -4.512578 1.496601 1.753861
H -4.297756 2.351106 0.223571
H -6.411152 -1.353496 0.708632
H -5.102969 -2.447376 0.250364
H -5.014262 -1.556009 1.770513

IBCH2CONPh_CCTMS.log
Energy (E) = -1165.91998885 Hartree
Enthalpy (H) = -1165.558363 Hartree
Gibbs free energy (G) = -1165.644062 Hartree
Charge = 0, Spin = 1
C -0.987982 3.236944 1.751379
C -0.166225 4.201471 1.183272
C 0.720231 3.862664 0.172348
C 0.818989 2.553781 -0.301702
C -0.015221 1.626966 0.303457
C -0.915040 1.923764 1.306573
H -1.680867 3.497669 2.538834
H -0.216900 5.224874 1.528565
H 1.354252 4.618435 -0.272870
H -1.551480 1.156609 1.725508
I 0.163801 -0.382811 -0.339409
C 1.773295 2.199014 -1.399086
H 1.225962 1.734183 -2.226764
H 2.259821 3.091316 -1.782869
C 2.881214 1.228508 -0.974333
O 4.041694 1.479032 -1.260454
N 2.413571 0.162037 -0.289516
C 3.256718 -0.888048 0.123144
C 4.333319 -1.348188 -0.641367
C 2.967821 -1.533362 1.328309
C 5.089122 -2.424084 -0.201314
H 4.577326 -0.844385 -1.563713
C 3.724462 -2.613006 1.759566
H 2.155294 -1.158717 1.939079
C 4.790855 -3.065879 0.995229
H 5.922101 -2.765321 -0.802177
H 3.485209 -3.093251 2.699306
H 5.386446 -3.904553 1.329280
C -1.947224 -0.556422 -0.236536
C -3.143020 -0.766837 -0.200635
Si -4.961046 -1.059768 -0.135249
C -5.703218 -0.520618 -1.755667
C -5.648635 -0.051405 1.273003
C -5.244471 -2.877812 0.152078
H -5.282526 -1.087708 -2.586583
H -6.783721 -0.675858 -1.752625
H -5.513707 0.537319 -1.939711
H -6.729259 -0.185491 1.348804
H -5.204052 -0.351421 2.222588
H -5.450182 1.010911 1.126110
H -6.313162 -3.094874 0.200571
H -4.815357 -3.473362 -0.654263
H -4.790478 -3.201837 1.089027

IBCH2S_CCTMS.log
Energy (E) = -1243.16350880 Hartree
Enthalpy (H) = -1242.854894 Hartree
Gibbs free energy (G) = -1242.931146 Hartree
Charge = 0, Spin = 1
C -0.813835 3.152162 -0.572695
C -2.137860 3.372578 -0.229396
C -2.962319 2.307333 0.106884
C -2.499747 0.991003 0.099917
C -1.168657 0.825647 -0.250122
C -0.314697 1.854922 -0.577443
H -0.167788 3.974727 -0.845319
H -2.536880 4.377749 -0.223796
H -3.989420 2.501055 0.381042
H 0.715566 1.655865 -0.837490
I -0.442789 -1.188657 -0.316932
C -3.387145 -0.170466 0.524672
S -3.014939 -1.595732 -0.536778
C -4.870253 0.139451 0.346844
H -5.442921 -0.766132 0.544752
H -5.204578 0.904142 1.051378
H -5.082710 0.467061 -0.670282
C -3.120977 -0.484112 1.999051
H -3.362699 0.383448 2.618469
H -3.740945 -1.325974 2.306655
H -2.077507 -0.747038 2.173681
C 1.582341 -0.408746 -0.096325
C 2.776268 -0.204910 0.025671
Si 4.571927 0.136089 0.216864
C 5.078370 -0.289835 1.958974
C 4.857490 1.948000 -0.119251
C 5.508059 -0.909560 -1.009363
H 4.892408 -1.342774 2.173311
H 6.142405 -0.096601 2.108311
H 4.520737 0.304366 2.683627
H 5.914844 2.196845 -0.010676
H 4.551377 2.210580 -1.132614
H 4.291467 2.568131 0.576850
H 6.581683 -0.730161 -0.925989
H 5.328292 -1.971003 -0.835487
H 5.204324 -0.679801 -2.031174

IBMeAlaO_CCTMS.log
Energy (E) = -1088.80516187 Hartree
Enthalpy (H) = -1088.464486 Hartree
Gibbs free energy (G) = -1088.547953 Hartree
Charge = 0, Spin = 1
C -0.485421 2.849499 -1.605669
C -1.501634 3.349668 -0.800723
C -2.139246 2.533209 0.121303
C -1.785947 1.189155 0.261322
C -0.754021 0.741297 -0.551812
C -0.096687 1.522986 -1.479166
H 0.009516 3.486959 -2.324512
H -1.801291 4.384465 -0.894110
H -2.926485 2.929767 0.748883
H 0.710243 1.117017 -2.073191
I -0.183470 -1.260102 -0.256259
N -2.369770 0.320393 1.213761
C -2.404816 0.876811 2.557622
H -1.416058 1.247579 2.825741
H -3.123521 1.695954 2.683275
H -2.677153 0.084717 3.254989
C -3.639756 -0.313414 0.824768
H -3.909595 -0.943107 1.677789
C -4.780806 0.667343 0.572241
H -5.694320 0.106622 0.397483
H -4.930935 1.337462 1.420899
H -4.586708 1.263766 -0.320063
C -3.530905 -1.293756 -0.359125
O -4.578469 -1.704112 -0.825089
O -2.360458 -1.650947 -0.768711
C 1.789250 -0.640329 -0.042679
C 2.961575 -0.354720 0.083527
Si 4.740142 0.094036 0.275228
C 4.959439 0.913039 1.932857
C 5.186209 1.263156 -1.104455
C 5.738137 -1.474043 0.169708
H 4.671113 0.241519 2.742106
H 6.002404 1.197335 2.084268
H 4.349183 1.813567 2.008233
H 6.233525 1.561390 -1.029456
H 5.037146 0.795035 -2.077953
H 4.575237 2.165669 -1.065164
H 6.802247 -1.256535 0.278572
H 5.454314 -2.172951 0.957041
H 5.590637 -1.968990 -0.790556

IBMeUreaMe_CCTMS.log
Energy (E) = -1029.66869881 Hartree
Enthalpy (H) = -1029.344780 Hartree
Gibbs free energy (G) = -1029.424833 Hartree
Charge = 0, Spin = 1
C -0.370308 3.085640 -1.257984
C -1.509417 3.578759 -0.636271
C -2.357041 2.739282 0.066017
C -2.101576 1.365350 0.181248
C -0.933248 0.918632 -0.437372
C -0.079523 1.733980 -1.150316
H 0.288726 3.738522 -1.811581
H -1.750159 4.630908 -0.705115
H -3.246236 3.143093 0.527405
H 0.817944 1.322896 -1.591753
I -0.448303 -1.114242 -0.207930
C -3.451644 -0.688127 0.381290
O -4.524661 -1.116824 0.780015
N -2.661823 -1.255361 -0.547050
C -3.155870 -2.505995 -1.088260
H -4.143182 -2.368613 -1.529664
H -2.475727 -2.851507 -1.868824
H -3.251629 -3.286573 -0.327373
N -2.954245 0.537085 0.911421
C -3.814327 1.153186 1.908849
H -3.238001 1.883136 2.474379
H -4.686852 1.639730 1.465718
H -4.176212 0.374544 2.571444
C 1.609862 -0.549353 -0.003784
C 2.803729 -0.357723 0.121980
Si 4.603386 -0.028712 0.313419
C 5.001295 0.062093 2.130977
C 4.981198 1.594287 -0.522263
C 5.545193 -1.417896 -0.496101
H 4.757311 -0.875390 2.631700
H 6.064174 0.260109 2.281956
H 4.435815 0.858990 2.614952
H 6.040626 1.838959 -0.425525
H 4.740201 1.551895 -1.585112
H 4.404733 2.405736 -0.076549
H 6.620805 -1.255206 -0.405027
H 5.307905 -2.375072 -0.030633
H 5.302240 -1.489488 -1.556752

IBMeUreaPh_CCTMS.log
Energy (E) = -1221.23902954 Hartree
Enthalpy (H) = -1220.859148 Hartree
Gibbs free energy (G) = -1220.946930 Hartree
Charge = 0, Spin = 1
C -1.043929 3.007335 2.046056
C -0.187438 3.983632 1.554499
C 0.753750 3.682831 0.584729

C 0.878249 2.389020 0.059980
C -0.016156 1.448253 0.566850
C -0.955274 1.719210 1.540112
H -1.774986 3.240791 2.806418
H -0.244084 4.993599 1.937032
H 1.420297 4.454585 0.228312
H -1.627109 0.941861 1.877670
I 0.092161 -0.485583 -0.246647
C 2.762793 1.047248 -0.820114
O 3.839075 1.179667 -1.379676
N 2.328333 0.019756 -0.060433
N 1.809585 2.104326 -0.941853
C 2.258271 3.211708 -1.773004
H 1.401637 3.834491 -2.023579
H 3.024221 3.818118 -1.284053
H 2.694724 2.804994 -2.678881
C 3.134734 -1.104453 0.200714
C 2.879426 -1.803011 1.385569
C 4.115931 -1.596450 -0.665941
C 3.578864 -2.956403 1.702500
H 2.134869 -1.407372 2.066191
C 4.816969 -2.748721 -0.337382
H 4.331419 -1.063418 -1.578013
C 4.556032 -3.437202 0.840274
H 3.366561 -3.474244 2.628683
H 5.577051 -3.113114 -1.016361
H 5.108873 -4.334147 1.084348
C -2.020373 -0.587885 -0.223326
C -3.222904 -0.756139 -0.247854
Si -5.051295 -0.981993 -0.275177
C -5.695212 -0.374929 -1.913455
C -5.766922 0.016296 1.126201
C -5.414850 -2.794669 -0.050634
H -5.255249 -0.935647 -2.738697
H -6.779431 -0.490529 -1.965640
H -5.459548 0.679652 -2.059449
H -6.854309 -0.076601 1.147056
H -5.380584 -0.325427 2.087111
H -5.520480 1.073180 1.018112
H -6.491903 -2.972115 -0.057866
H -4.970133 -3.385367 -0.852085
H -5.018023 -3.159473 0.897218

IBNMeCO2_CCTMS.log
Energy (E) = -1010.28782619 Hartree
Enthalpy (H) = -1010.005692 Hartree
Gibbs free energy (G) = -1010.082003 Hartree
Charge = 0, Spin = 1
C -0.686714 3.076727 -0.981112
C -1.878224 3.412832 -0.352050
C -2.680409 2.442403 0.222395
C -2.327987 1.084048 0.200778
C -1.110885 0.799749 -0.421000
C -0.299561 1.745821 -1.011748
H -0.063203 3.832818 -1.435403
H -2.196640 4.445761 -0.315891
H -3.611626 2.727636 0.689280
H 0.635908 1.451839 -1.467849
I -0.481001 -1.207916 -0.408334
C -3.495469 -1.089568 0.129421
O -4.500439 -1.664539 0.488395
N -3.130814 0.122935 0.806781
C -4.066909 0.561842 1.829250
H -3.573150 1.273241 2.489355
H -4.963547 1.019847 1.404108
H -4.380406 -0.309895 2.392697
O -2.691763 -1.435691 -0.817709
C 1.485279 -0.618096 -0.078581
C 2.657491 -0.358597 0.094656
Si 4.431873 0.076902 0.361208
C 4.771929 0.042680 2.190524
C 4.692250 1.784632 -0.334995
C 5.471538 -1.179130 -0.536611
H 4.583348 -0.947751 2.605696
H 5.813707 0.300190 2.390212
H 4.139513 0.756140 2.719543
H 5.726785 2.102364 -0.193241
H 4.475445 1.809108 -1.403500
H 4.045625 2.510469 0.159347
H 6.532465 -0.956440 -0.409045
H 5.289889 -2.184043 -0.154417
H 5.252043 -1.179128 -1.604628

ISamideMe_CCTMS.log
Energy (E) = -1370.23097257 Hartree
Enthalpy (H) = -1369.952868 Hartree
Gibbs free energy (G) = -1370.030529 Hartree
Charge = 0, Spin = 1
C 0.576357 3.201781 -0.479581
C 1.915680 3.447094 -0.205017
C 2.770771 2.398759 0.109108
C 2.272081 1.105989 0.149024
C 0.945536 0.888967 -0.144487
C 0.069033 1.904751 -0.452515
H -0.085022 4.019542 -0.729853
H 2.295353 4.458711 -0.239241
H 3.819450 2.555942 0.322283
H -0.971100 1.705919 -0.669952
I 0.343643 -1.172211 -0.117203
N 2.652209 -1.419093 -0.347991
C 3.057300 -1.302514 -1.748055
H 2.739760 -0.355277 -2.206930
H 4.139906 -1.374867 -1.841888
H 2.606325 -2.121008 -2.307187
S 3.285999 -0.297347 0.598433
O 2.938443 -0.593618 1.964540
O 4.643134 0.045230 0.250966
C -1.671198 -0.554859 -0.031224
C -2.861005 -0.318432 0.025208
Si -4.657677 0.091800 0.120311
C -4.827573 1.899831 -0.297960
C -5.245914 -0.250763 1.852031
C -5.562785 -0.966402 -1.115226
H -4.458931 2.104940 -1.303827
H -5.873582 2.208226 -0.253369
H -4.264809 2.517084 0.403465
H -6.306431 -0.011578 1.949850
H -5.111485 -1.301458 2.110610
H -4.695053 0.347639 2.578142
H -6.632014 -0.747897 -1.093539
H -5.200732 -0.784788 -2.127567
H -5.431005 -2.026053 -0.894453

ISamidePh_CCTMS.log
Energy (E) = -1561.80017843 Hartree
Enthalpy (H) = -1561.466135 Hartree
Gibbs free energy (G) = -1561.551666 Hartree
Charge = 0, Spin = 1
C 0.231756 2.768684 2.034738
C -0.994115 3.385061 1.821031
C -1.864242 2.899538 0.853316
C -1.496383 1.794065 0.102490
C -0.281085 1.196344 0.343668
C 0.607874 1.653360 1.289492
H 0.903899 3.144451 2.793471
H -1.274431 4.245167 2.412835
H -2.827441 3.354629 0.664833
H 1.558589 1.165920 1.454396
I 0.129986 -0.549374 -0.839991
N -2.269712 -0.425229 -1.039325
S -2.536415 1.142856 -1.197488
O -1.897259 1.566510 -2.417954
O -3.884399 1.564129 -0.913325
C -2.875206 -1.179353 -0.010809
C -2.773210 -2.569110 -0.133759
C -3.504224 -0.654900 1.122023
C -3.273409 -3.411637 0.844440
H -2.311316 -2.969796 -1.027824
C -4.011163 -1.507114 2.094232
H -3.624489 0.412549 1.234537
C -3.897102 -2.884321 1.968989
H -3.185846 -4.483186 0.722742
H -4.502903 -1.083135 2.959909
H -4.296374 -3.538519 2.731724
C 2.147948 -0.411604 -0.356429
C 3.341242 -0.419641 -0.137220
Si 5.153521 -0.387466 0.224796
C 5.395106 0.782882 1.653710
C 6.038163 0.215575 -1.296159
C 5.681118 -2.114695 0.672382
H 4.850632 0.442170 2.535239
H 6.451851 0.853332 1.917291
H 5.044114 1.784401 1.401911
H 7.114612 0.257185 -1.120328
H 5.860043 -0.448490 -2.142396
H 5.701654 1.214422 -1.575017
H 6.748151 -2.139475 0.900833
H 5.139399 -2.476667 1.546617
H 5.496768 -2.806642 -0.149865

AnthIBA_A_02.log
Energy (E) = -737.570484444 Hartree
Enthalpy (H) = -737.369999 Hartree
Gibbs free energy (G) = -737.428262 Hartree
Charge = 0, Spin = 2
C 5.930941 -0.266438 -0.000239
C 4.951314 -1.209872 -0.000164
C 3.578699 -0.828059 -0.000126
C 3.243556 0.559218 -0.000169
C 4.299193 1.516858 -0.000244
C 5.599729 1.117942 -0.000279
C 2.544140 -1.757935 -0.000042
C 1.906111 0.959988 -0.000136
C 0.866275 0.031714 -0.000061
C 1.210233 -1.362335 -0.000004
C 0.179118 -2.342072 0.000103
H 0.461481 -3.387526 0.000135
C -1.129820 -1.986340 0.000167
C -1.464066 -0.609951 0.000109
C -0.536532 0.390582 -0.000016
H 2.777311 -2.817014 0.000001
H 6.971192 -0.562700 -0.000268
H 5.194686 -2.265246 -0.000133
H 4.038195 2.567569 -0.000276
H 6.393567 1.852462 -0.000338
H 1.680231 2.014310 -0.000175
H -1.912676 -2.731105 0.000252
C -0.983539 1.816530 -0.000141
O -0.245788 2.786870 -0.000421
O -2.264894 2.064547 -0.000044
I -3.522465 -0.182284 0.000226

AnthIBA_B_02.log
Energy (E) = -737.574635748 Hartree
Enthalpy (H) = -737.374292 Hartree
Gibbs free energy (G) = -737.432462

Hartree
Charge = 0, Spin = 2
C -6.094526 0.316151 0.000005
C -5.011970 1.138455 0.000010
C -3.690188 0.603946 0.000006
C -3.520149 -0.817823 -0.000003
C -4.680897 -1.645200 -0.000009
C -5.925577 -1.097604 -0.000004
C -2.564610 1.423502 0.000011
C -2.233677 -1.353954 -0.000007
C -1.109615 -0.533830 -0.000003
C -1.279051 0.887006 0.000007
C -0.122091 1.709405 0.000012
H -0.215396 2.789121 0.000018
C 1.132274 1.176130 0.000007
C 1.282750 -0.237134 -0.000001
H -2.688708 2.500410 0.000018
H -7.093208 0.730957 0.000008
H -5.131404 2.214741 0.000017
H -4.547325 -2.719866 -0.000016
H -6.799117 -1.735297 -0.000008
H -2.108092 -2.430877 -0.000015
I 3.227866 -1.032685 -0.000008
C 0.212914 -1.069194 -0.000007
H 0.337759 -2.144255 -0.000014
C 2.282045 2.123800 0.000013
O 3.492222 1.639354 0.000010
O 2.145633 3.333827 0.000015

AnthIBA_C_02.log
Energy (E) = -737.574957570 Hartree
Enthalpy (H) = -737.374475 Hartree
Gibbs free energy (G) = -737.432294
Hartree
Charge = 0, Spin = 2
C 4.964652 -1.434278 0.000150
C 3.618047 -1.623945 0.000130
C 2.727055 -0.511697 0.000025
C 3.273131 0.808476 -0.000044
C 4.689015 0.967001 -0.000019
C 5.508198 -0.118690 0.000078
C 1.344088 -0.680014 0.000011
C 2.403251 1.895985 -0.000131
C 1.022168 1.732421 -0.000126
C 0.472574 0.404957 -0.000050
C -0.952097 0.293970 -0.000024
C -1.763878 1.384392 0.000008
H 0.950021 -1.688741 0.000046
H 5.633433 -2.284096 0.000229
H 3.197488 -2.621862 0.000181
H 5.095879 1.970521 -0.000080
H 6.581903 0.010785 0.000093
H 2.810314 2.900928 -0.000191
C 0.145796 2.857701 -0.000121
H 0.583299 3.847783 -0.000197
C -3.254967 1.309259 0.000123
O -3.955377 2.304302 0.000348
O -3.825334 0.136971 -0.000036
C -1.197773 2.692063 -0.000040
H -1.888801 3.523692 0.000007
I -1.862517 -1.604807 -0.000045

AnthIBA_D_02.log
Energy (E) = -737.555808294 Hartree
Enthalpy (H) = -737.355354 Hartree
Gibbs free energy (G) = -737.413103
Hartree
Charge = 0, Spin = 2
C 2.022262 3.396322 -0.442960
C 0.672163 3.474859 -0.349742
C -0.115125 2.310398 -0.119140
C 0.510355 1.017289 -0.006499
C 1.954436 1.000577 0.046246
C 2.650371 2.153798 -0.197110
C -1.496026 2.436701 -0.052092
C -0.354510 -0.083467 0.018422
C -1.750876 0.027642 0.113761
C -2.327525 1.337481 0.106431
C -3.740114 1.490965 0.205567
H -4.142651 2.496141 0.192236
C -4.555234 0.410097 0.313009
C -3.990873 -0.892590 0.323859
C -2.646402 -1.077362 0.221402
H -1.934647 3.424331 -0.137266
H 2.622372 4.273635 -0.637756
H 0.153375 4.419279 -0.457527
H 3.727611 2.097301 -0.128047
H -5.626490 0.532757 0.390882
H -4.640060 -1.752106 0.420365
H -2.247561 -2.080486 0.248151
C 2.832507 -0.101245 0.585691
O 2.306977 -1.134010 1.181571
O 4.046366 0.022685 0.617706
I 0.439035 -1.998661 -0.356346

AnthIBA_E_02.log
Energy (E) = -737.549193070 Hartree
Enthalpy (H) = -737.348862 Hartree
Gibbs free energy (G) = -737.406435
Hartree
Charge = 0, Spin = 2
C -4.910622 -0.310320 -0.640475
C -4.220181 0.844800 -0.461343
C -2.826535 0.818925 -0.166872
C -2.141443 -0.428657 -0.023404
C -2.906955 -1.616789 -0.229538
C -4.232378 -1.553098 -0.537114
C -2.125580 1.999803 0.035016
C -0.758176 -0.437034 0.278334
C -0.025250 0.769846 0.235075
C -0.748349 2.011578 0.215442
C -0.070879 3.253050 0.386443
H -0.669560 4.154508 0.416017
C 1.279633 3.303709 0.499078
C 2.025733 2.109211 0.335303
C 1.399708 0.909030 0.193221
H -2.660260 2.943231 0.029192
H -5.966561 -0.291094 -0.872644
H -4.705446 1.808972 -0.547915
H -2.419269 -2.574570 -0.165936
H -4.778839 -2.469687 -0.713781
H 1.797810 4.241456 0.639146
H 3.103554 2.163947 0.271488
I 2.609190 -0.667757 -0.499336
C -0.212698 -1.667045 0.942074
O -0.947928 -2.506421 1.444928
O 1.054841 -1.822041 1.185296

FuranIBA_A_02.log
Energy (E) = -428.384876973 Hartree
Enthalpy (H) = -428.314444 Hartree
Gibbs free energy (G) = -428.359256
Hartree
Charge = 0, Spin = 2
C 2.002218 -2.152371 0.000194
C 0.643541 -2.149502 0.000111
C 0.277039 -0.777171 0.000090
C 1.432972 -0.062887 0.000030
O 2.492266 -0.900875 0.000228
H 2.726554 -2.947014 0.000263
H -0.008678 -3.004301 0.000094
C 1.664132 1.377169 -0.000080
I -1.616025 0.057440 -0.000029
O 2.745111 1.938399 -0.000125
O 0.614131 2.149424 -0.000214

FuranIBA_B_02.log
Energy (E) = -428.385607437 Hartree
Enthalpy (H) = -428.315056 Hartree
Gibbs free energy (G) = -428.360064
Hartree
Charge = 0, Spin = 2
C 2.468058 -1.026744 0.000167
C 1.500511 -0.073189 0.000066
C 0.256982 -0.777894 0.000052
O 1.914640 -2.246666 0.000197
H 3.541249 -0.961590 0.000217
I -1.624452 0.090840 -0.000075
C 0.564422 -2.092166 0.000119
H -0.027935 -2.988071 0.000131
C 1.730133 1.370262 0.000005
O 0.699636 2.165385 -0.000085
O 2.818475 1.922972 0.000037

FuranIBA_C_02.log
Energy (E) = -428.385212358 Hartree
Enthalpy (H) = -428.315258 Hartree
Gibbs free energy (G) = -428.360229
Hartree
Charge = 0, Spin = 2
C 0.274487 -0.694433 -0.000022
C 1.506918 -0.117691 0.000041
O 0.380312 -2.025756 0.000166
C 1.713580 -2.326056 0.000030
H 1.956540 -3.372302 0.000083
C 1.808729 1.305224 0.000085
O 2.934715 1.798494 -0.000134
O 0.869876 2.187831 -0.000045
C 2.449196 -1.196847 -0.000154
H 3.521145 -1.106561 -0.000270
I -1.612724 0.131569 0.000008

Indole_NAc_IBA_A_02.log
Energy (E) = -714.565503857 Hartree
Enthalpy (H) = -714.392166 Hartree
Gibbs free energy (G) = -714.452066
Hartree
Charge = 0, Spin = 2
C -0.381411 1.340038 -0.021425
C -1.702489 0.850780 -0.075060
C -2.781829 1.735443 -0.086022
C -2.504488 3.090087 -0.080128
C -1.192119 3.581055 -0.049544
C -0.123768 2.711277 -0.014945
C 0.475623 0.197429 0.025692
H -3.805763 1.399737 -0.105235
H -3.328919 3.789630 -0.099328
H -1.023020 4.648509 -0.046805
H 0.897060 3.068884 0.021053
N -1.647174 -0.544218 -0.060232
C -0.301977 -0.916684 -0.001590
C 0.162090 -2.286771 -0.257312
O 1.396289 -2.572869 0.015904
O -0.472886 -3.187438 -0.781951
I 2.544865 0.243748 0.049944
C -2.693744 -1.437113 0.309832
C -4.061231 -1.140495 -0.235465
H -4.627279 -0.557563 0.491513
H -4.023026 -0.599945 -1.177656
H -4.567570 -2.092921 -0.363986

O -2.453032 -2.379159 1.012400

Indole_NAc_IBA_B_02.log
Energy (E) = -714.571259435 Hartree
Enthalpy (H) = -714.396932 Hartree
Gibbs free energy (G) = -714.455838 Hartree
Charge = 0, Spin = 2
C -1.765230 -0.884989 -0.001781
C -1.923577 0.511290 0.013465
C -3.198827 1.073119 0.046345
C -4.284915 0.212130 0.041486
C -4.128835 -1.175305 0.011853
C -2.867539 -1.738032 -0.005686
H -3.379536 2.132710 0.081535
H -5.279781 0.635360 0.064867
H -5.003658 -1.810438 0.009166
H -2.712772 -2.806766 -0.018314
N -0.628001 1.091580 0.021532
C -0.352612 -1.152278 0.000469
C 0.285485 0.038320 0.021985
C 0.307988 -2.465984 -0.021339
O -0.295749 -3.523276 -0.030496
O 1.617452 -2.472796 -0.032319
I 2.351764 0.129042 0.001390
C -0.251008 2.447837 0.011949
C -1.321600 3.480695 -0.170229
H -1.945522 3.260217 -1.034142
H -1.956797 3.531575 0.713226
H -0.819426 4.433165 -0.302622
O 0.910049 2.753956 0.134161

Indole_NH_IBA_A_02.log
Energy (E) = -562.046713296 Hartree
Enthalpy (H) = -561.913520 Hartree
Gibbs free energy (G) = -561.964316 Hartree
Charge = 0, Spin = 2
C -1.385825 -0.363956 0.000106
C -2.119050 0.847967 0.000073
C -3.515598 0.854505 0.000120
C -4.156241 -0.365340 0.000191
C -3.440090 -1.578211 0.000221
C -2.064271 -1.589065 0.000182
C -0.014327 0.012220 0.000035
H -4.071681 1.781963 0.000099
H -5.237273 -0.394117 0.000224
H -3.985629 -2.511470 0.000274
H -1.508980 -2.518293 0.000201
H -1.419081 2.877170 -0.000022
N -1.225505 1.886792 0.000003
C 0.045789 1.373699 0.000007
C 1.215376 2.249920 -0.000025
O 2.387536 1.662572 -0.000419
O 1.152098 3.465729 -0.000470
I 1.680935 -1.172069 0.000016

Indole_NH_IBA_B_02.log
Energy (E) = -562.044982596 Hartree
Enthalpy (H) = -561.912535 Hartree
Gibbs free energy (G) = -561.963685 Hartree
Charge = 0, Spin = 2
C -1.847152 0.295047 0.000061
C -1.802389 -1.109570 0.000157
C -2.949881 -1.895830 0.000255
C -4.162641 -1.233992 0.000251
C -4.228772 0.167856 0.000158
C -3.086091 0.943360 0.000061
H -2.897012 -2.976039 0.000320
H -5.079530 -1.807175 0.000323
H -5.197695 0.647832 0.000171
H -3.136362 2.022641 0.000007
H -0.114215 -2.426714 0.000133
N -0.467410 -1.482907 0.000102
C -0.481171 0.751679 0.000038
C 0.310651 -0.368524 -0.000025
C -0.030350 2.124417 0.000015
O -0.764004 3.124408 0.000043
O 1.206373 2.459383 -0.000048
I 2.374047 -0.524475 -0.000141

Indole_NMe_IBA_A_02.log
Energy (E) = -601.314763861 Hartree
Enthalpy (H) = -601.151835 Hartree
Gibbs free energy (G) = -601.206362 Hartree
Charge = 0, Spin = 2
C 1.170686 -0.771202 -0.000032
C 2.112165 0.283258 -0.000069
C 3.487697 0.035675 -0.000103
C 3.894190 -1.281324 -0.000109
C 2.966710 -2.341652 -0.000080
C 1.611799 -2.100037 -0.000042
C -0.097330 -0.138904 0.000015
H 4.203726 0.846465 -0.000123
H 4.951417 -1.508997 -0.000137
H 3.331406 -3.359370 -0.000085
H 0.896026 -2.912331 -0.000015
N 1.448203 1.480746 -0.000071
C 0.094984 1.215894 -0.000003
C -0.972239 2.223490 0.000011
O -2.197376 1.760602 0.000035
O -0.822992 3.433908 -0.000072
I -1.971850 -1.012026 0.000076
C 2.114603 2.769472 -0.000047
H 2.741311 2.863244 -0.887008
H 1.368953 3.555732 -0.000167
H 2.741118 2.863330 0.887043

Indole_NMe_IBA_B_02.log
Energy (E) = -601.315120384 Hartree
Enthalpy (H) = -601.153029 Hartree
Gibbs free energy (G) = -601.208344 Hartree
Charge = 0, Spin = 2
C 1.846534 0.463889 -0.000251
C 1.809575 -0.941562 -0.000140
C 2.969541 -1.712242 -0.000179
C 4.175892 -1.038326 -0.000306
C 4.229763 0.363588 -0.000409
C 3.078409 1.125321 -0.000397
H 2.940586 -2.793056 -0.000135
H 5.097407 -1.604108 -0.000352
H 5.193772 0.853402 -0.000528
H 3.114889 2.205184 -0.000504
N 0.477661 -1.339670 -0.000060
C 0.480765 0.908750 -0.000244
C -0.298755 -0.222329 0.000067
C 0.020488 2.277407 -0.000421
O 0.745429 3.282155 -0.000101
O -1.222353 2.600959 0.000197
I -2.366909 -0.344683 0.000287
C 0.004393 -2.706688 -0.000047
H -0.595690 -2.908116 0.886257
H -0.595403 -2.908291 -0.886510
H 0.862723 -3.370881 0.000166

Indole_NMs_IBA_A_02.log
Energy (E) = -1149.73548275 Hartree
Enthalpy (H) = -1149.559783 Hartree
Gibbs free energy (G) = -1149.621383 Hartree
Charge = 0, Spin = 2
C -0.360624 1.505560 -0.020644
C 1.045760 1.408977 -0.119276
C 1.848034 2.540686 -0.133668
C 1.206203 3.768235 -0.052735
C -0.189279 3.883176 0.038570
C -0.982562 2.758709 0.054164
C -0.874069 0.185485 -0.026261
H 2.919590 2.471709 -0.218829
H 1.806694 4.667313 -0.069362
H -0.638696 4.864329 0.094169
H -2.060259 2.822225 0.123079
N 1.367308 0.041272 -0.187240
C 0.205760 -0.694821 -0.096250
C 0.126353 -2.199429 -0.146813
O 0.401878 -2.972775 0.739430
O -0.309280 -2.313881 -1.331583
I -2.855599 -0.351704 0.082399
C 2.951958 -1.167854 1.617598
H 2.864325 -0.278956 2.236702
H 2.138817 -1.875289 1.776282
H 3.917345 -1.647331 1.764477
S 2.908703 -0.674914 -0.068964
O 2.870195 -1.829434 -0.907106
O 3.840119 0.390305 -0.294204

Indole_NMs_IBA_B_02.log
Energy (E) = -1149.72837761 Hartree
Enthalpy (H) = -1149.552397 Hartree
Gibbs free energy (G) = -1149.614731 Hartree
Charge = 0, Spin = 2
C 1.691728 1.255672 -0.045711
C 1.899089 -0.121273 -0.212122
C 3.177743 -0.667220 -0.281770
C 4.240714 0.214516 -0.178758
C 4.047672 1.591712 -0.021987
C 2.776788 2.128745 0.042357
H 3.338993 -1.721992 -0.430084
H 5.247454 -0.176484 -0.232607
H 4.908226 2.242807 0.045480
H 2.605889 3.188551 0.159762
N 0.625239 -0.741896 -0.291289
C 0.268779 1.464727 0.002140
C -0.332153 0.256597 -0.126042
C -0.428202 2.746484 0.152860
O 0.130196 3.822936 0.260121
O -1.738282 2.729082 0.183580
I -2.393249 0.047265 -0.109548
C 0.490325 -2.455988 1.752450
H 1.462622 -2.070114 2.051042
H -0.322166 -1.859834 2.162725
H 0.384085 -3.500508 2.034866
S 0.387741 -2.387618 -0.004931
O 1.510523 -3.067607 -0.572807
O -0.947231 -2.696428 -0.416858

NpthIBA_A_02.log
Energy (E) = -584.079187931 Hartree
Enthalpy (H) = -583.928023 Hartree
Gibbs free energy (G) = -583.979604 Hartree
Charge = 0, Spin = 2
C 0.453972 2.553362 -0.000354
C -0.463592 1.477613 -0.000114
C -0.000385 0.191746 -0.000029
H 0.040499 3.552642 -0.000263

C -1.912896 1.839537 0.000284
O -2.798310 0.883022 -0.000560
O -2.293969 2.994978 0.001273
I -1.428760 -1.357167 -0.000100
C 1.793657 2.313795 -0.000576
H 2.502684 3.131673 -0.000971
C 3.266719 -1.635609 0.000582
C 4.162918 -0.547330 0.000212
C 3.681233 0.731926 -0.000250
C 2.290795 0.987660 -0.000313
C 1.385509 -0.110336 0.000009
C 1.916042 -1.421675 0.000452
H 3.650415 -2.646434 0.000992
H 5.228819 -0.728958 0.000292
H 4.356944 1.577963 -0.000541
H 1.239353 -2.265154 0.000676

NpthIBA_B_02.log
Energy (E) = -584.074374329 Hartree
Enthalpy (H) = -583.923217 Hartree
Gibbs free energy (G) = -583.975305 Hartree
Charge = 0, Spin = 2
C 0.418257 0.478134 0.000063
C -0.439590 -0.592269 -0.000052
C -0.133104 1.868776 0.000132
O -1.428863 2.019935 -0.000037
O 0.529679 2.891405 0.000311
I -2.524322 -0.311730 -0.000188
C 1.334220 -2.182654 -0.000014
C -0.005179 -1.929195 -0.000089
H -0.725951 -2.734251 -0.000176
H 1.696095 -3.203312 -0.000039
C 2.276551 -1.129679 0.000092
C 3.659288 -1.423667 0.000154
C 4.585813 -0.420089 0.000246
C 4.152901 0.919770 0.000276
C 2.820197 1.238585 0.000221
C 1.832592 0.221376 0.000131
H 3.962947 -2.463158 0.000124
H 5.642787 -0.648323 0.000293
H 4.885066 1.715983 0.000343
H 2.509924 2.269483 0.000248

NpthIBA_C_02.log
Energy (E) = -584.079207714 Hartree
Enthalpy (H) = -583.928189 Hartree
Gibbs free energy (G) = -583.980062 Hartree
Charge = 0, Spin = 2
C -1.299777 1.531724 -0.000082
C 0.005800 1.114989 -0.000039
C 0.283311 -0.267039 0.000017
C -0.714233 -1.195977 0.000030
H -1.491321 2.598345 -0.000125
H -0.492599 -2.255376 0.000074
C 1.064134 2.164686 -0.000055
O 2.313123 1.791362 -0.000033
O 0.816099 3.356410 -0.000126
I 2.290166 -0.890196 0.000085
C -3.131884 -1.710314 -0.000003
C -4.429485 -1.278290 -0.000047
C -4.727127 0.103274 -0.000103
C -3.717878 1.025088 -0.000115
C -2.365054 0.608406 -0.000071
C -2.067452 -0.778681 -0.000014
H -2.900514 -2.768020 0.000040
H -5.238048 -1.996557 -0.000038
H -5.758878 0.426653 -0.000137
H -3.933359 2.085973 -0.000158

NpthIBA_D_02.log
Energy (E) = -584.062697768 Hartree
Enthalpy (H) = -583.911680 Hartree
Gibbs free energy (G) = -583.963465 Hartree
Charge = 0, Spin = 2
C 1.111084 -3.055170 0.382513
C 2.266577 -2.357776 0.188980
C 2.251276 -0.955851 0.006208
C 1.025784 -0.220973 0.048845
C -0.149406 -1.013181 0.147939
C -0.115450 -2.370008 0.328358
H 4.378299 -0.883605 -0.275486
H 1.120749 -4.124956 0.534939
H 3.223938 -2.862669 0.168258
C 3.474310 -0.288611 -0.236783
C 1.108961 1.205965 -0.031010
H -1.039937 -2.925148 0.402904
C 2.323908 1.798921 -0.287681
C 3.510456 1.061419 -0.423654
H 2.343923 2.878389 -0.332784
H 4.442049 1.570591 -0.625975
I -2.059475 -0.219738 -0.224748
C 0.042145 2.205863 0.335377
O -1.033413 1.837112 0.967455
O 0.231575 3.404131 0.193198

PyIBA_A_02.log
Energy (E) = -446.610148485 Hartree
Enthalpy (H) = -446.520534 Hartree
Gibbs free energy (G) = -446.565848 Hartree
Charge = 0, Spin = 2
C 2.176772 -2.087071 0.000434
C 3.107638 -1.056936 0.000347
C 2.646219 0.249081 0.000134
C 1.277989 0.484231 0.000018
C 0.453017 -0.629015 0.000125
H 2.490692 -3.122966 0.000598
H 4.164542 -1.278774 0.000444
H 3.308134 1.105479 0.000055
C 0.780853 1.890876 -0.000206
O -0.508138 2.079229 -0.000364
O 1.539197 2.840636 -0.000359
I -1.639168 -0.303007 -0.000051
N 0.858449 -1.872895 0.000324

PyIBA_B_02.log
Energy (E) = -446.604939671 Hartree
Enthalpy (H) = -446.515350 Hartree
Gibbs free energy (G) = -446.560835 Hartree
Charge = 0, Spin = 2
C 3.066038 -1.126398 -0.000001
C 1.285634 0.460584 -0.000008
C 0.415181 -0.612956 -0.000002
H 4.120854 -1.370101 -0.000010
C 0.834883 1.887493 -0.000011
O -0.447194 2.119650 0.000002
O 1.615060 2.817899 -0.000001
I -1.652605 -0.276744 0.000005
C 0.916381 -1.908068 -0.000006
H 0.248142 -2.760883 0.000002
C 2.647703 0.194604 -0.000011
H 3.341642 1.023970 -0.000018
N 2.219940 -2.156783 -0.000003

PyIBA_C_02.log
Energy (E) = -446.605465528 Hartree
Enthalpy (H) = -446.515942 Hartree
Gibbs free energy (G) = -446.561519 Hartree
Charge = 0, Spin = 2
C -1.279665 0.447492 0.000206
C -0.404135 -0.622830 0.000002
C -0.854146 1.873557 0.000392
O 0.424385 2.134616 0.000033
O -1.637900 2.802435 0.000137
I 1.663071 -0.272738 -0.000116
C -0.897071 -1.914809 -0.000129
H -0.243065 -2.774198 -0.000281
C -2.646204 0.166531 0.000274
H -3.338675 1.000052 0.000406
C -2.277317 -2.075479 -0.000083
H -2.699774 -3.073232 -0.000162
N -3.143130 -1.062954 0.000125

PyIBA_D_02.log
Energy (E) = -446.600397996 Hartree
Enthalpy (H) = -446.511208 Hartree
Gibbs free energy (G) = -446.556992 Hartree
Charge = 0, Spin = 2
C 2.259229 -2.107487 -0.000003
C 3.086441 -0.988278 0.000008
C 1.301512 0.440853 0.000007
C 0.405553 -0.619153 -0.000003
H 2.680542 -3.102530 -0.000008
H 4.163985 -1.097045 0.000012
C 0.866654 1.880889 0.000013
O -0.404971 2.144882 0.000015
O 1.644868 2.811534 0.000025
I -1.662862 -0.274839 -0.000009
C 0.888232 -1.919023 -0.000008
H 0.209979 -2.760704 -0.000017
N 2.616042 0.249799 0.000012

PyraIBA_02.log
Energy (E) = -462.628692679 Hartree
Enthalpy (H) = -462.551546 Hartree
Gibbs free energy (G) = -462.597044 Hartree
Charge = 0, Spin = 2
C -3.039844 -1.035809 0.000032
C -2.141508 -2.097786 0.000255
N -0.827746 -1.886227 0.000160
C -0.431532 -0.636088 -0.000024
C -1.312719 0.441166 -0.000083
N -2.619822 0.221419 -0.000075
H -4.108505 -1.206464 -0.000026
H -2.478864 -3.125864 0.000235
C -0.864847 1.877785 -0.000194
I 1.646595 -0.275077 0.000053
O -1.642751 2.804797 -0.000269
O 0.416939 2.103883 -0.000173

PyridaIBA_A_02.log
Energy (E) = -462.594100779 Hartree
Enthalpy (H) = -462.517657 Hartree
Gibbs free energy (G) = -462.563541 Hartree
Charge = 0, Spin = 2
N 3.093950 -1.060471 -0.000203
C 2.239217 -2.079794 0.000024
C 0.858358 -1.925153 0.000152
C 0.381821 -0.634593 0.000030
C 1.316099 0.394685 -0.000215
N 2.624275 0.164325 -0.000322
H 0.202484 -2.783378 0.000341

H 2.688073 -3.064052 0.000110
C 0.941183 1.849599 -0.000391
I -1.674630 -0.242265 0.000222
O -0.318422 2.159703 -0.000231
O 1.745574 2.756803 -0.000538

PyridaIBA_B_02.log
Energy (E) = -462.599410846 Hartree
Enthalpy (H) = -462.522470 Hartree
Gibbs free energy (G) = -462.568018 Hartree
Charge = 0, Spin = 2
N -3.077529 -1.168255 -0.000074
C -2.642013 0.081519 -0.000060
C -1.288695 0.423617 -0.000027
C -0.394821 -0.615833 -0.000011
C -0.901375 -1.911620 -0.000026
N -2.202815 -2.159968 -0.000056
H -3.386855 0.866910 -0.000072
I 1.662726 -0.247446 0.000037
H -0.253131 -2.777679 -0.000012
C -0.901800 1.865871 -0.000011
O 0.372480 2.143276 0.000002
O -1.716211 2.764433 -0.000025

PyridaIBA_C_02.log
Energy (E) = -462.602208772 Hartree
Enthalpy (H) = -462.525288 Hartree
Gibbs free energy (G) = -462.570562 Hartree
Charge = 0, Spin = 2
N -0.820965 -1.884998 0.000001
C -0.443072 -0.630318 -0.000013
C -1.296180 0.465042 -0.000010
C -2.998585 -1.173704 0.000002
N -2.117145 -2.165609 0.000006
H -4.035247 -1.481101 0.000005
C -0.830083 1.887514 -0.000011
O -1.615073 2.812339 0.000011
O 0.455228 2.089998 0.000001
C -2.639813 0.171582 -0.000002
H -3.364098 0.975064 -0.000001
I 1.631911 -0.276964 0.000001

PyrmIBA_02.log
Energy (E) = -462.641408231 Hartree
Enthalpy (H) = -462.563733 Hartree
Gibbs free energy (G) = -462.609048 Hartree
Charge = 0, Spin = 2
C -2.156859 -2.072502 0.000207
N -0.834681 -1.884890 -0.000013
C -0.433336 -0.638252 -0.000002
C -1.289312 0.447293 0.000157
C -2.643765 0.133159 0.000240
N -3.082979 -1.121246 0.000210
H -2.498045 -3.099634 -0.000111
H -3.368642 0.938922 0.000335
C -0.852192 1.870469 0.000202
O -1.642426 2.791913 0.000311
O 0.430897 2.103341 0.000060
I 1.645950 -0.271686 -0.000177

PyrroleIBA_A_02.log
Energy (E) = -408.541035990 Hartree
Enthalpy (H) = -408.457437 Hartree
Gibbs free energy (G) = -408.502122 Hartree
Charge = 0, Spin = 2
C 2.075421 -2.138842 -0.000049
C 0.695559 -2.148265 0.000006
C 0.293809 -0.799373 -0.000007
C 1.420829 -0.016400 0.000083
H 2.770027 -2.960419 -0.000098
H 0.063117 -3.018182 -0.000019
C 1.577289 1.428839 0.000163
I -1.608373 0.024339 -0.000051
O 2.653057 2.003528 0.000091
O 0.479791 2.146451 0.000010
N 2.502327 -0.850582 0.000095
H 3.454126 -0.512881 0.000155

PyrroleIBA_B_02.log
Energy (E) = -408.538023915 Hartree
Enthalpy (H) = -408.454682 Hartree
Gibbs free energy (G) = -408.499891 Hartree
Charge = 0, Spin = 2
C 2.515589 -0.957713 0.000177
C 1.492262 -0.038167 0.000049
C 0.274903 -0.767952 0.000070
H 3.578825 -0.794513 0.000215
I -1.626782 0.065470 -0.000058
C 0.584976 -2.096343 0.000152
H -0.047101 -2.965681 0.000175
C 1.681762 1.405540 -0.000062
O 0.643891 2.188114 -0.000147
O 2.758518 1.989781 -0.000053
N 1.957082 -2.192419 0.000235
H 2.471943 -3.058119 0.000319

PyrroleIBA_C_02.log
Energy (E) = -408.536870368 Hartree
Enthalpy (H) = -408.454142 Hartree
Gibbs free energy (G) = -408.499233 Hartree
Charge = 0, Spin = 2
C 0.265326 -0.702922 0.000016
C 1.508077 -0.110438 -0.000016
C 1.793255 -2.335089 0.000010
H 2.139679 -3.352966 0.000010
C 1.797772 1.309095 -0.000065
O 2.935124 1.808476 -0.000031
O 0.898337 2.213289 -0.000045
C 2.475705 -1.155865 0.000050
H 3.543244 -1.019713 0.000074
I -1.626587 0.136084 0.000002
N 0.445743 -2.045901 0.000051
H -0.302505 -2.721290 0.000075

PyrroleINAcBA_B_02.log
Energy (E) = -561.072985133 Hartree
Enthalpy (H) = -560.948967 Hartree
Gibbs free energy (G) = -561.002268 Hartree
Charge = 0, Spin = 2
C 1.728695 1.044244 -0.000301
C 0.364042 1.078351 0.000002
C -0.092913 -0.273276 -0.000138
H 2.426538 1.862611 -0.000362
I -2.092684 -0.823924 -0.000016
C 0.992810 -1.086194 -0.000350
H 1.099626 -2.155284 -0.000445
C -0.439140 2.300970 0.000253
O -1.740006 2.179398 0.000392
O 0.000682 3.438127 0.000267
N 2.116344 -0.272005 -0.000058
C 3.438756 -0.780534 0.000175
C 4.531783 0.246762 -0.000037
H 4.459976 0.883378 -0.881442
H 4.460222 0.883451 0.881333
H 5.483121 -0.273651 -0.000149
O 3.617346 -1.968831 -0.000068

PyrroleINMeBA_B_02.log
Energy (E) = -447.807052144 Hartree
Enthalpy (H) = -447.694376 Hartree
Gibbs free energy (G) = -447.744663 Hartree
Charge = 0, Spin = 2
C -2.396166 0.381697 -0.000455
C -1.072133 0.765438 0.000034
C -0.301426 -0.424248 0.000056
H -3.283973 0.991116 -0.000598
I 1.772725 -0.526260 0.000035
C -1.168993 -1.478531 -0.000652
H -0.987605 -2.539154 -0.001017
C -0.614408 2.144540 0.000074
O 0.655903 2.408550 0.000425
O -1.328924 3.142716 -0.000133
N -2.447163 -0.970060 -0.001046
C -3.656290 -1.768957 0.001091
H -3.692451 -2.408673 -0.878693
H -3.704616 -2.387302 0.895530
H -4.514948 -1.103530 -0.012989

PyrroleINMsBA_B_02.log
Energy (E) = -996.226053028 Hartree
Enthalpy (H) = -996.100082 Hartree
Gibbs free energy (G) = -996.157141 Hartree
Charge = 0, Spin = 2
C -1.137428 1.302261 -0.133134
C 0.223584 1.192747 -0.052650
C 0.544234 -0.196410 -0.055969
H -1.773691 2.169573 -0.173687
I 2.473074 -0.957903 0.015361
C -0.615327 -0.899931 -0.138281
H -0.822626 -1.953449 -0.196342
C 1.147622 2.325592 0.016387
O 2.426003 2.077817 0.080709
O 0.829469 3.503109 0.025727
N -1.642847 0.030689 -0.193027
C -3.587030 -0.422405 1.599019
H -2.953042 -1.193313 2.028390
H -3.377194 0.554934 2.024799
H -4.637604 -0.677219 1.717777
S -3.286672 -0.353205 -0.135447
O -3.394947 -1.670649 -0.683731
O -3.970027 0.776432 -0.686325

PyrroleNAcIBA_A_02.log
Energy (E) = -561.060124227 Hartree
Enthalpy (H) = -560.937004 Hartree
Gibbs free energy (G) = -560.991087 Hartree
Charge = 0, Spin = 2
C -1.513386 -1.880433 0.140336
C -0.153940 -1.984603 0.102762
C 0.350363 -0.664474 0.006381
C -0.710371 0.206902 -0.010849
H -2.261559 -2.652560 0.170138
H 0.423433 -2.891645 0.134963
C -0.649184 1.656847 0.161855
I 2.363703 -0.167565 -0.036939
O -1.512753 2.389011 0.647082
O 0.427366 2.287727 -0.123144
N -1.870586 -0.554214 0.069508
C -3.193314 -0.103962 -0.221066
C -4.293027 -1.028985 0.220270

H -4.365334 -1.877087 -0.461279
H -4.118567 -1.408536 1.225078
H -5.224994 -0.475043 0.181037
O -3.371858 0.930457 -0.796049

PyrroleNAcIBA_C_02.log
Energy (E) = -561.065969098 Hartree
Enthalpy (H) = -560.941821 Hartree
Gibbs free energy (G) = -560.994346 Hartree
Charge = 0, Spin = 2
C 0.174846 2.650720 -0.000058
C 1.451824 2.201257 -0.000136
N 1.442607 0.803458 -0.000183
C -0.670657 1.500950 -0.000024
C 0.124274 0.394766 -0.000147
C -2.138481 1.498570 0.000030
O -2.825166 2.503098 0.000155
O -2.737872 0.333695 -0.000110
I -0.624107 -1.530884 0.000049
C 2.590561 -0.020642 -0.000091
C 3.909372 0.698538 0.000384
H 4.006081 1.330654 -0.881605
H 4.005502 1.330491 0.882560
H 4.692254 -0.051833 0.000570
O 2.488766 -1.218807 -0.000336
H -0.161047 3.673136 0.000004
H 2.380357 2.741347 -0.000285

PyrroleNMeIBA_A_02.log
Energy (E) = -447.809766112 Hartree
Enthalpy (H) = -447.696539 Hartree
Gibbs free energy (G) = -447.744485 Hartree
Charge = 0, Spin = 2
C 1.915831 -2.005338 -0.000410
C 0.537637 -2.092111 0.000298
C 0.075411 -0.768989 -0.000073
C 1.162931 0.075588 0.000107
H 2.657653 -2.786454 -0.000677
H -0.048823 -2.993463 0.000520
C 1.169856 1.530231 0.000204
I -1.871730 -0.055558 -0.000141
O 2.163080 2.241696 0.000159
O 0.001963 2.125667 0.000032
N 2.293042 -0.703111 0.000179
C 3.667913 -0.230166 0.000441
H 3.862481 0.376730 -0.879607
H 3.862403 0.376226 0.880856
H 4.318860 -1.100890 0.000211

PyrroleNMeIBA_C_02.log
Energy (E) = -447.807126415 Hartree
Enthalpy (H) = -447.694919 Hartree
Gibbs free energy (G) = -447.743626 Hartree
Charge = 0, Spin = 2
C 0.214909 -0.551744 0.000023
C 1.546434 -0.190628 0.000113
C 1.414685 -2.422440 0.000285
H 1.564794 -3.488221 0.000399
C 2.088682 1.151836 0.000044
O 3.297294 1.437520 0.000017
O 1.367289 2.205456 -0.000185
C 2.306174 -1.391483 0.000131
H 3.380430 -1.454846 0.000104
I -1.472209 0.650353 -0.000175
N 0.136147 -1.906035 0.000303
C -1.077394 -2.696947 0.000477
H -1.674543 -2.485664 0.886079
H -1.674578 -2.485964 -0.885171
H -0.799678 -3.747132 0.000650

PyrroleNMsIBA_A_02.log
Energy (E) = -996.209948661 Hartree
Enthalpy (H) = -996.083874 Hartree
Gibbs free energy (G) = -996.139825 Hartree
Charge = 0, Spin = 2
C -1.067463 1.918676 0.000187
C 0.295517 2.041814 -0.000008
C 0.811469 0.730604 0.000010
C -0.232498 -0.156578 0.000141
H -1.813823 2.690779 0.000277
H 0.851726 2.962474 -0.000050
C -0.148921 -1.617757 0.000225
I 2.801274 0.152316 -0.000042
O -1.080886 -2.398056 0.000022
O 1.054141 -2.129802 0.000178
N -1.402937 0.586658 -0.000118
C -3.967519 1.411605 0.000894
H -3.786285 1.980279 0.907622
H -3.786797 1.981251 -0.905326
H -4.986670 1.029604 0.000975
S -3.000278 -0.064004 -0.000174
O -3.190957 -0.720977 -1.254086
O -3.190317 -0.722400 1.253090

PyrroleNMsIBA_C_02.log
Energy (E) = -996.221563763 Hartree
Enthalpy (H) = -996.095791 Hartree
Gibbs free energy (G) = -996.151972 Hartree
Charge = 0, Spin = 2
C 0.310162 0.416693 -0.074375
C 1.229584 1.427717 -0.018072
C -0.795531 2.380392 -0.119494
H -1.670676 3.001502 -0.177585
C 2.680630 1.274393 0.064542
O 3.484324 2.192465 0.110603
O 3.196918 0.078766 0.100666
C 0.527590 2.668450 -0.051253
H 0.982717 3.643564 -0.034210
I 0.738460 -1.609472 -0.038349
N -0.938633 0.993386 -0.142469
C -2.587105 -0.252582 1.569983
H -1.784443 -0.954364 1.784318
H -2.528143 0.633734 2.195243
H -3.555755 -0.736626 1.671755
S -2.462012 0.246267 -0.113716
O -3.391566 1.305643 -0.363823
O -2.391106 -0.910641 -0.949733

QuinoIBA_A_02.log
Energy (E) = -600.114505542 Hartree
Enthalpy (H) = -599.975124 Hartree
Gibbs free energy (G) = -600.026272 Hartree
Charge = 0, Spin = 2
C 0.447188 2.573787 -0.000162
C -0.470849 1.497934 -0.000127
C -0.009855 0.214758 -0.000065
H 0.032937 3.572864 -0.000116
C -1.931653 1.827558 0.000028
O -2.771902 0.833773 -0.000911
O -2.327039 2.977718 0.000897
I -1.369644 -1.370669 0.000049
C 1.789370 2.335737 -0.000180
H 2.499051 3.153129 -0.000291
C 3.066483 -1.618172 0.000214
C 4.054751 -0.608889 0.000054
C 3.658080 0.697873 -0.000061
C 2.280045 1.007127 -0.000096
C 1.373743 -0.082193 -0.000040
H 3.362237 -2.661216 0.000153
H 5.100174 -0.881806 0.000020
H 4.378966 1.505878 -0.000157
N 1.780783 -1.372633 0.000075

QuinoIBA_B_02.log
Energy (E) = -600.104120607 Hartree
Enthalpy (H) = -599.964902 Hartree
Gibbs free energy (G) = -600.016601 Hartree
Charge = 0, Spin = 2
C 0.421522 0.454990 -0.000012
C -0.521925 -0.543713 0.000121
C 0.128476 1.926902 -0.000042
O -0.989766 2.398781 0.000383
O 1.166527 2.720309 0.000216
I -2.599714 -0.245305 -0.000109
C 1.196650 -2.255429 0.000270
C -0.121875 -1.903524 0.000321
H -0.887007 -2.666695 0.000516
H 1.484716 -3.299010 0.000397
C 2.199537 -1.260483 0.000066
C 3.584542 -1.528488 -0.000024
C 4.475398 -0.489611 -0.000166
C 3.976115 0.830558 -0.000216
C 1.792437 0.089897 -0.000019
H 3.924177 -2.556627 0.000016
H 5.542321 -0.658074 -0.000250
H 4.650686 1.678449 -0.000309
N 2.695797 1.089117 -0.000168

QuinoIBA_C_02.log
Energy (E) = -600.109836326 Hartree
Enthalpy (H) = -599.970866 Hartree
Gibbs free energy (G) = -600.022574 Hartree
Charge = 0, Spin = 2
C -1.321681 1.522035 -0.000041
C -0.014889 1.109512 -0.000005
C 0.275435 -0.270508 0.000009
C -0.715339 -1.207726 -0.000013
H -1.544501 2.580847 -0.000051
H -0.487354 -2.265849 -0.000003
C 1.040208 2.165850 0.000018
O 2.290701 1.798523 0.000068
O 0.787087 3.355292 0.000024
I 2.284483 -0.883816 0.000061
C -3.144197 -1.711265 -0.000079
C -4.420291 -1.229891 -0.000118
C -4.624557 0.171767 -0.000118
C -2.377832 0.587279 -0.000068
C -2.067686 -0.794730 -0.000052
H -2.940018 -2.774638 -0.000072
H -5.272895 -1.893952 -0.000146
H -5.636223 0.562696 -0.000166
N -3.656279 1.049811 -0.000103

QuinoIBA_D_02.log
Energy (E) = -600.111717835 Hartree
Enthalpy (H) = -599.972645 Hartree
Gibbs free energy (G) = -600.024247 Hartree
Charge = 0, Spin = 2
C -1.307386 1.539383 -0.000119
C -0.004119 1.116411 -0.000054
C 0.267464 -0.267072 0.000020

C -0.729571 -1.196472 0.000025
H -1.496059 2.606566 -0.000176
H -0.537243 -2.259907 0.000081
C 1.060275 2.162358 -0.000065
O 2.306022 1.779510 0.000021
O 0.812382 3.353574 -0.000100
I 2.273816 -0.889610 0.000119
C -4.296051 -1.319634 -0.000076
C -4.693267 0.038730 -0.000177
C -2.370566 0.615580 -0.000116
C -2.079837 -0.770991 -0.000049
H -5.054321 -2.095274 -0.000105
H -5.744443 0.288613 -0.000242
N -3.050179 -1.721319 -0.000031
C -3.729593 1.004417 -0.000184
H -3.980266 2.057625 -0.000248

QuinoIBA_E_02.log
Energy (E) = -600.108212423 Hartree
Enthalpy (H) = -599.968972 Hartree
Gibbs free energy (G) = -600.020769 Hartree
Charge = 0, Spin = 2
C 0.421334 0.481810 -0.000003
C -0.429208 -0.593511 0.000010
C -0.129283 1.872634 -0.000039
O -1.425040 2.021185 -0.000074
O 0.541750 2.889609 -0.000047
I -2.512039 -0.312621 -0.000032
C 1.355844 -2.176714 0.000076
C 0.015959 -1.927973 0.000048
H -0.700424 -2.737122 0.000055
H 1.749447 -3.183524 0.000106
C 2.289143 -1.113843 0.000065
C 4.489324 -0.496325 0.000075
C 4.146768 0.870887 0.000035
C 1.833305 0.230009 0.000023
H 5.532318 -0.794024 0.000099
H 4.927024 1.618869 0.000024
C 2.830255 1.237772 0.000008
H 2.538976 2.274810 -0.000023
N 3.602347 -1.457846 0.000090

QuinoIBA_F_02.log
Energy (E) = -600.111299921 Hartree
Enthalpy (H) = -599.972176 Hartree
Gibbs free energy (G) = -600.023615 Hartree
Charge = 0, Spin = 2
C 0.503206 2.536477 -0.000243
C -0.434644 1.477313 -0.000069
C 0.004300 0.183130 0.000003
H 0.105145 3.542223 -0.000174
C -1.877033 1.864484 0.000220
O -2.779549 0.924029 -0.000578
O -2.240353 3.025217 0.001096
I -1.440884 -1.351175 -0.000085
C 1.840093 2.280786 -0.000442
H 2.578866 3.069284 -0.000739
C 3.269432 -1.618965 0.000567
C 4.104246 -0.480298 0.000034
C 2.311192 0.945796 -0.000280
C 1.384520 -0.133619 0.000037
H 3.705475 -2.607413 0.001058
H 5.181731 -0.603840 0.000035
C 1.916554 -1.442076 0.000471
H 1.248901 -2.293354 0.000737
N 3.653535 0.747607 -0.000338

QuinoIBA_G_02.log
Energy (E) = -600.104022902 Hartree
Enthalpy (H) = -599.965377 Hartree
Gibbs free energy (G) = -600.017344 Hartree
Charge = 0, Spin = 2
C 4.670395 0.130290 0.000208
C 3.642075 1.030056 0.000206
C 2.302953 0.572851 0.000126
C 2.043187 -0.820974 0.000049
C 3.126851 -1.729563 0.000053
C 4.410876 -1.260790 0.000131
H 5.694229 0.478074 0.000269
H 3.806989 2.098231 0.000263
C 0.694766 -1.234778 -0.000030
H 2.921387 -2.792556 -0.000007
H 5.239671 -1.955398 0.000134
C -0.287826 -0.288479 -0.000027
C 0.061619 1.081658 0.000053
H 0.460499 -2.291760 -0.000090
N 1.303178 1.483905 0.000126
C -0.968137 2.182817 0.000059
O -0.678361 3.360775 0.000141
O -2.225059 1.856990 -0.000001
I -2.305626 -0.861218 -0.000142

QuinoIBA_H_02.log
Energy (E) = -600.105225121 Hartree
Enthalpy (H) = -599.966027 Hartree
Gibbs free energy (G) = -600.017817 Hartree
Charge = 0, Spin = 2
C 4.546941 -0.485812 0.000508
C 3.596146 -1.466502 0.000329
C 2.224360 -1.130026 0.000177
C 1.824979 0.235016 0.000210
C 2.835176 1.228395 0.000398
C 4.156732 0.868907 0.000541
H 5.597242 -0.743201 0.000624
H 3.846964 -2.517865 0.000297
C 0.420255 0.504086 0.000046
H 2.549843 2.266917 0.000423
H 4.914331 1.640731 0.000682
C -0.426267 -0.569147 -0.000120
C 0.063137 -1.892910 -0.000140
N 1.336453 -2.160603 0.000004
I -2.509571 -0.329639 -0.000344
H -0.631572 -2.725987 -0.000270
C -0.137318 1.896699 0.000051
O -1.434706 2.028483 0.000097
O 0.528514 2.914298 0.000457

QuinoIBA_I_02.log
Energy (E) = -600.095019519 Hartree
Enthalpy (H) = -599.955930 Hartree
Gibbs free energy (G) = -600.007540 Hartree
Charge = 0, Spin = 2
C 1.167988 -3.034609 0.343548
C 2.313583 -2.317029 0.169081
C 2.270182 -0.914106 -0.002425
C 1.027202 -0.208416 0.037685
C -0.133740 -1.017915 0.133174
C -0.074383 -2.375099 0.296328
H 1.196498 -4.105917 0.483077
H 3.290414 -2.778482 0.148480
C 1.103530 1.216132 -0.029830
H -0.987080 -2.949799 0.367547
C 3.481306 0.990069 -0.371698
N 3.465710 -0.305191 -0.210061
H 4.443126 1.453198 -0.560363
C 0.024445 2.225095 0.298466
O 0.227628 3.414698 0.126232
O -1.057061 1.858047 0.917662
C 2.327584 1.787524 -0.261019
H 2.393325 2.864587 -0.306089
I -2.056735 -0.238474 -0.201759

QuinoIBA_J_02.log
Energy (E) = -600.095313858 Hartree
Enthalpy (H) = -599.956384 Hartree
Gibbs free energy (G) = -600.008154 Hartree
Charge = 0, Spin = 2
C -3.548537 0.941112 -0.412959
C -3.461691 -0.406286 -0.229804
C -2.212222 -1.023273 0.015344
C -1.016500 -0.238924 0.054257
C -2.391357 1.726897 -0.282051
H -4.499445 1.414425 -0.612511
H -4.323508 -1.057764 -0.264520
C 0.175585 -1.002935 0.141203
H -2.455181 2.804599 -0.329945
C -1.116876 -2.999963 0.368338
N -2.244146 -2.369530 0.185485
H -1.162944 -4.071750 0.525159
C 0.134270 -2.356473 0.320343
H 1.041158 -2.938866 0.391585
I 2.081332 -0.196193 -0.211316
C -1.153252 1.182552 -0.032430
C -0.120876 2.221658 0.309049
O -0.353752 3.413548 0.174436
O 0.987138 1.907464 0.911047

QuinoIBA_K_02.log
Energy (E) = -600.110305034 Hartree
Enthalpy (H) = -599.971198 Hartree
Gibbs free energy (G) = -600.022694 Hartree
Charge = 0, Spin = 2
C 4.151447 -0.530422 0.000118
C 3.657231 0.744502 -0.000105
C 2.264344 0.978112 -0.000146
C 1.377314 -0.135170 -0.000000
C 3.272346 -1.633651 0.000283
H 5.219604 -0.698599 0.000166
H 4.301157 1.612641 -0.000252
C -0.003271 0.170305 -0.000016
H 3.672478 -2.637950 0.000477
C 0.554189 2.492325 -0.000219
N 1.837089 2.270321 -0.000304
H 0.201004 3.517482 -0.000239
C -0.426449 1.466105 -0.000050
I -1.456694 -1.349188 -0.000057
C 1.918724 -1.440251 0.000212
H 1.248722 -2.289493 0.000305
C -1.854484 1.886697 0.000148
O -2.774547 0.961437 -0.000127
O -2.196224 3.053482 0.000548

QuinoIBA_L_02.log
Energy (E) = -600.115030692 Hartree
Enthalpy (H) = -599.975979 Hartree
Gibbs free energy (G) = -600.027509 Hartree
Charge = 0, Spin = 2
C 4.335723 -1.312883 0.000215
C 3.024260 -1.705815 0.000096
C 2.000771 -0.735711 0.000096
C 2.342702 0.640082 0.000194
C 4.681605 0.056773 0.000329

H 5.120377 -2.057156 0.000226
H 2.736464 -2.747555 0.000008
H 5.724090 0.343151 0.000423
C -0.217885 -0.246324 -0.000065
N 0.699085 -1.147188 -0.000041
C -0.010006 1.148852 0.000044
C 3.705807 1.015065 0.000316
H 3.956884 2.067988 0.000399
C -1.107148 2.158396 0.000015
O -0.883224 3.353620 0.000154
O -2.335755 1.725790 0.000037
C 1.289632 1.576545 0.000168
H 1.481417 2.643471 0.000236
I -2.234599 -0.913675 -0.000207

ThiopheneIBA_A_02.log
Energy (E) = -751.316045935 Hartree
Enthalpy (H) = -751.248135 Hartree
Gibbs free energy (G) = -751.293615 Hartree
Charge = 0, Spin = 2
C -2.084506 -2.031749 0.000061
C -0.718882 -2.013108 0.000094
C -0.234876 -0.685611 0.000011
C -1.222020 0.254246 -0.000078
H -2.723280 -2.899244 0.000115
H -0.094482 -2.892383 0.000186
C -1.080800 1.712836 -0.000159
I 1.776203 -0.142949 0.000068
O -2.011183 2.497049 -0.000164
O 0.134786 2.195710 -0.000082
S -2.766457 -0.474613 -0.000096

ThiopheneIBA_B_02.log
Energy (E) = -751.315946451 Hartree
Enthalpy (H) = -751.248241 Hartree
Gibbs free energy (G) = -751.293807 Hartree
Charge = 0, Spin = 2
C -2.531733 0.173857 0.000209
C -1.221212 0.555138 0.000141
C -0.345854 -0.562693 -0.000029
H -3.384431 0.832589 0.000348
I 1.726777 -0.329830 -0.000125
C -0.995834 -1.753248 -0.000091
H -0.576762 -2.744431 -0.000206
C -0.817481 1.973397 0.000271
O 0.459326 2.246895 0.000205
O -1.593765 2.911185 0.000384
S -2.688112 -1.511907 -0.000078

ThiopheneIBA_C_02.log
Energy (E) = -751.312942832 Hartree
Enthalpy (H) = -751.245307 Hartree
Gibbs free energy (G) = -751.291056 Hartree
Charge = 0, Spin = 2
C -0.358204 -0.517172 0.000103
C -1.384729 0.382411 0.000080
C -2.557722 -1.605286 0.000096
H -3.350753 -2.333665 0.000075
C -1.227041 1.843149 0.000093
O -2.146980 2.643323 -0.000094
O -0.025426 2.350350 -0.000127
C -2.659191 -0.252351 0.000102
H -3.584376 0.303430 0.000112
I 1.664329 -0.058804 0.000018
S -0.923358 -2.119190 -0.000140

AnthIBA_A_Cl.log
Energy (E) = -1197.70070076 Hartree
Enthalpy (H) = -1197.496208 Hartree
Gibbs free energy (G) = -1197.556734 Hartree
Charge = 0, Spin = 1
C -6.293842 -0.537973 0.000123
C -5.254478 -1.414686 -0.000003
C -3.909419 -0.943710 -0.000045
C -3.667604 0.464173 0.000078
C -4.784428 1.350015 0.000218
C -6.055282 0.865597 0.000236
C -2.819426 -1.808127 -0.000176
C -2.359913 0.951313 0.000044
C -1.268163 0.085924 -0.000093
C -1.512423 -1.329318 -0.000187
C -0.417933 -2.238644 -0.000258
H -0.631691 -3.300005 -0.000395
C 0.872305 -1.807514 -0.000185
C 1.074057 -0.416693 -0.000077
C 0.102952 0.531640 -0.000058
H -2.987326 -2.879287 -0.000240
H -7.312206 -0.902234 0.000140
H -5.427835 -2.483670 -0.000099
H -4.593417 2.415573 0.000303
H -6.896134 1.545694 0.000349
H -2.191108 2.016784 0.000095
H 1.706570 -2.491642 -0.000264
C 0.515466 1.976681 -0.000087
O -0.254283 2.906063 -0.000155
O 1.824321 2.148954 -0.000257
I 3.020278 0.416682 0.000033
Cl 4.139229 -1.812796 0.000263

AnthIBA_B_Cl.log
Energy (E) = -1197.70253505 Hartree
Enthalpy (H) = -1197.498140 Hartree
Gibbs free energy (G) = -1197.558517 Hartree
Charge = 0, Spin = 1
C 6.388442 0.138285 0.000004
C 5.363478 1.031208 -0.000006
C 4.009167 0.585416 -0.000006
C 3.745987 -0.822021 0.000005
C 4.848529 -1.725383 0.000014
C 6.126553 -1.261350 0.000013
C 2.941006 1.478333 -0.000010
C 2.427136 -1.271573 0.000002
C 1.360291 -0.378600 -0.000001
C 1.621378 1.031430 -0.000006
C 0.526149 1.937316 -0.000002
H 0.689975 3.008324 0.000002
C -0.756156 1.481088 -0.000006
C -0.969545 0.086900 -0.000004
H 3.137702 2.544258 -0.000016
H 7.412493 0.485725 0.000002
H 5.554449 2.097046 -0.000016
H 4.643406 -2.788580 0.000019
H 6.956070 -1.955210 0.000019
H 2.228560 -2.337086 0.000008
I -3.039432 -0.341720 0.000018
Cl -2.615332 -2.795361 -0.000050
C 0.007202 -0.840151 0.000009
H -0.203209 -1.900750 0.000015
C -1.925396 2.411988 0.000012
O -3.088821 1.786419 0.000034
O -1.805458 3.611239 -0.000067

AnthIBA_C_Cl.log
Energy (E) = -1197.69056965 Hartree
Enthalpy (H) = -1197.486048 Hartree
Gibbs free energy (G) = -1197.546992 Hartree
Charge = 0, Spin = 1
C -5.033856 -1.360905 -0.409076
C -3.680351 -1.486383 -0.453052
C -2.840473 -0.362242 -0.205559
C -3.442394 0.900010 0.081455
C -4.863109 0.992945 0.118220
C -5.633333 -0.102988 -0.118633
C -1.452109 -0.475865 -0.238020
C -2.618031 2.003551 0.285961
C -1.232021 1.904418 0.241677
C -0.627543 0.614559 0.015324
C 0.795013 0.614324 -0.008090
C 1.552685 1.735190 0.028369
H -1.026067 -1.439043 -0.468469
H -5.665692 -2.219049 -0.592556
H -3.213830 -2.439974 -0.666062
H -5.313420 1.953147 0.336628
H -6.711611 -0.025116 -0.088399
H -3.064749 2.975951 0.459750
C -0.415369 3.068525 0.357584
H -0.906665 4.018356 0.525292
C 3.031958 1.678722 -0.196425
O 3.727390 2.662231 -0.168264
O 3.465279 0.466759 -0.476649
C 0.929403 2.999336 0.215125
H 1.577207 3.864422 0.244940
I 2.094885 -1.100908 -0.074928
Cl 0.525398 -2.924215 0.652365

AnthIBA_D_Cl.log
Energy (E) = -1197.68326055 Hartree
Enthalpy (H) = -1197.478700 Hartree
Gibbs free energy (G) = -1197.538370 Hartree
Charge = 0, Spin = 1
C -2.760407 3.062851 0.766046
C -1.452136 3.424646 0.704881
C -0.451300 2.487012 0.323337
C -0.824914 1.129826 0.042755
C -2.226286 0.816721 -0.017553
C -3.139770 1.762977 0.356002
C 0.884412 2.866504 0.253304
C 0.225759 0.235681 -0.157169
C 1.563133 0.597626 -0.318813
C 1.888232 1.978551 -0.112477
C 3.237218 2.409792 -0.266552
H 3.459686 3.454640 -0.090764
C 4.208693 1.537246 -0.638443
C 3.880160 0.176351 -0.880749
C 2.610552 -0.281933 -0.717008
H 1.147585 3.893874 0.477794
H -3.518048 3.773167 1.064701
H -1.136669 4.432455 0.944157
H -4.184678 1.500349 0.262856
H 5.229298 1.871251 -0.762071
H 4.654134 -0.506618 -1.202288
H 2.390687 -1.320671 -0.910928
C -2.795156 -0.396704 -0.709848
O -1.923229 -1.222613 -1.272459
O -3.986885 -0.543728 -0.829050
I -0.312937 -1.797959 -0.023461
Cl 1.572673 -2.265809 1.559161

AnthIBA_E_Cl.log
Energy (E) = -1197.68061248 Hartree
Enthalpy (H) = -1197.476042 Hartree
Gibbs free energy (G) = -1197.536084 Hartree

Charge = 0, Spin = 1
C -5.233426 -0.121218 -0.860093
C -4.516979 0.996544 -0.577824
C -3.143976 0.904677 -0.208470
C -2.507612 -0.373037 -0.096659
C -3.299936 -1.519032 -0.408518
C -4.602628 -1.390434 -0.786186
C -2.421963 2.054330 0.082724
C -1.143924 -0.444500 0.280468
C -0.388932 0.744145 0.366489
C -1.060055 2.013845 0.347737
C -0.342268 3.220317 0.587345
H -0.905475 4.144741 0.603554
C 1.002287 3.221357 0.770963
C 1.714190 2.001798 0.639085
C 1.031297 0.849145 0.443687
H -2.926390 3.014132 0.077812
H -6.273080 -0.051909 -1.148950
H -4.963976 1.981020 -0.636751
H -2.851489 -2.495533 -0.356345
H -5.168707 -2.276860 -1.038820
H 1.545562 4.136648 0.953845
H 2.794731 2.007391 0.634291
I 2.129343 -0.870691 -0.067353
Cl 3.797311 0.516047 -1.328022
C -0.619036 -1.736179 0.850939
O -1.360359 -2.622250 1.205641
O 0.677499 -1.829794 1.102772

FuranIBA_A_Cl.log
Energy (E) = -888.501969364 Hartree
Enthalpy (H) = -888.426996 Hartree
Gibbs free energy (G) = -888.473155 Hartree
Charge = 0, Spin = 1
C 1.784530 2.543365 -0.000205
C 0.458264 2.236687 -0.000281
C 0.456819 0.823153 -0.000088
C 1.729877 0.380481 -0.000116
O 2.560104 1.433576 -0.000264
H 2.304740 3.484091 -0.000250
H -0.379565 2.909681 -0.000357
C 2.081141 -1.054891 -0.000002
I -0.907745 -0.706555 0.000088
Cl -2.744232 0.955258 0.000105
O 3.196062 -1.502263 -0.000095
O 0.965520 -1.776125 0.000149

FuranIBA_B_Cl.log
Energy (E) = -888.502302460 Hartree
Enthalpy (H) = -888.427211 Hartree
Gibbs free energy (G) = -888.473387 Hartree
Charge = 0, Spin = 1
C 2.516174 1.544871 -0.000145
C 1.792014 0.399120 0.000075
C 0.446065 0.825917 0.000047
O 1.687278 2.611739 -0.000048
H 3.572044 1.743668 -0.000253
I -0.892591 -0.725705 0.000073
Cl -2.734314 0.937558 -0.000529
C 0.405539 2.171516 0.000104
H -0.384036 2.898219 0.000169
C 2.100390 -1.051381 0.000214
O 0.984607 -1.774497 0.000265
O 3.208314 -1.519526 0.000216

FuranIBA_C_Cl.log
Energy (E) = -888.493181552 Hartree
Enthalpy (H) = -888.418374 Hartree
Gibbs free energy (G) = -888.464741 Hartree
Charge = 0, Spin = 1
C 0.513750 0.787043 0.000023
C 1.803269 0.412251 -0.000146
O 0.322388 2.093144 0.000109
C 1.589964 2.625130 -0.000072
H 1.637277 3.698294 -0.000110
C 2.115206 -1.034389 -0.000368
O 3.222494 -1.503828 0.000103
O 0.998673 -1.760965 0.000131
C 2.527203 1.648449 0.000098
H 3.595514 1.773720 0.000150
I -0.882509 -0.726396 0.000096
Cl -2.712035 0.927605 -0.000300

Indole_NAc_IBA_A_Cl.log
Energy (E) = -1174.68315685 Hartree
Enthalpy (H) = -1174.505144 Hartree
Gibbs free energy (G) = -1174.566266 Hartree
Charge = 0, Spin = 1
C -0.521121 1.369163 -0.014755
C -1.892032 1.008297 -0.041612
C -2.892864 1.979262 -0.021361
C -2.506182 3.304287 -0.026050
C -1.154361 3.671775 -0.032153
C -0.157629 2.720358 -0.018209
C 0.166903 0.118086 0.031376
H -3.939245 1.717382 0.002076
H -3.265991 4.073477 -0.020122
H -0.889423 4.719594 -0.036592
H 0.881671 3.005953 -0.004416
N -1.996877 -0.382460 -0.020007
C -0.721956 -0.903276 0.028711
C -0.289276 -2.310473 -0.180123
O 1.025523 -2.406847 -0.060403
O -1.018130 -3.218256 -0.479322
I 2.122545 -0.574477 0.003005
Cl 3.129795 1.696965 0.060698
C -3.177532 -1.149377 0.263298
C -4.410223 -0.780056 -0.512392
H -5.112366 -0.264028 0.142334
H -4.191794 -0.159597 -1.376952
H -4.878897 -1.709845 -0.826032
O -3.131087 -2.034288 1.065753

Indole_NAc_IBA_B_Cl.log
Energy (E) = -1174.67949428 Hartree
Enthalpy (H) = -1174.501590 Hartree
Gibbs free energy (G) = -1174.562198 Hartree
Charge = 0, Spin = 1
C 2.147267 -0.752927 0.063654
C 2.092495 0.652420 0.145604
C 3.250846 1.404128 0.301108
C 4.452050 0.715110 0.378584
C 4.512788 -0.679135 0.300393
C 3.361619 -1.428702 0.143879
H 3.241605 2.478528 0.387372
H 5.365591 1.277866 0.512917
H 5.472050 -1.172763 0.368394
H 3.378048 -2.507731 0.083944
N 0.731632 1.056184 0.018899
C 0.792005 -1.218439 -0.073639
C 0.023886 -0.119684 -0.091917
C 0.166996 -2.550745 -0.213077
O 0.779551 -3.589537 -0.220085
O -1.146842 -2.452824 -0.371632
I -2.005727 -0.526076 0.035779
Cl -2.595877 1.746244 0.833224
C 0.281816 2.226138 -0.668235
C 0.970389 3.512505 -0.338108
H 1.248952 3.560607 0.711784
H 1.868616 3.613506 -0.948170
H 0.291511 4.321002 -0.591065
O -0.616124 2.125807 -1.459289

Indole_NH_IBA_A_Cl.log
Energy (E) = -1022.16519157 Hartree
Enthalpy (H) = -1022.027703 Hartree
Gibbs free energy (G) = -1022.080462 Hartree
Charge = 0, Spin = 1
C -1.558539 -0.309165 0.000091
C -2.410264 0.829871 0.000062
C -3.799727 0.709927 0.000121
C -4.330931 -0.560617 0.000202
C -3.503664 -1.697176 0.000231
C -2.131281 -1.589297 0.000180
C -0.245683 0.246323 0.000029
H -4.432240 1.587124 0.000103
H -5.404707 -0.687351 0.000247
H -3.957621 -2.678217 0.000298
H -1.506076 -2.467990 0.000212
H -1.943407 2.927959 -0.000072
N -1.635243 1.966994 -0.000025
C -0.327147 1.601337 -0.000067
C 0.850585 2.487113 -0.000225
O 1.965502 1.784715 -0.000255
O 0.771286 3.692522 -0.000324
I 1.734387 -0.366662 -0.000046
Cl 1.153755 -2.773246 0.000159

Indole_NH_IBA_B_Cl.log
Energy (E) = -1022.16139643 Hartree
Enthalpy (H) = -1022.024241 Hartree
Gibbs free energy (G) = -1022.076840 Hartree
Charge = 0, Spin = 1
C 2.176760 0.311515 -0.000054
C 2.038692 -1.094233 -0.000113
C 3.139241 -1.946001 -0.000194
C 4.388086 -1.355756 -0.000214
C 4.543433 0.040117 -0.000157
C 3.451385 0.883694 -0.000076
H 3.023377 -3.020902 -0.000235
H 5.268135 -1.984105 -0.000274
H 5.540955 0.457144 -0.000177
H 3.559509 1.959190 -0.000035
H 0.235663 -2.309249 -0.000050
N 0.679727 -1.401258 -0.000065
C 0.846919 0.853735 0.000011
C 0.027785 -0.221318 0.000000
C 0.251088 2.194753 0.000069
O 0.854517 3.235616 0.000095
O -1.094861 2.143083 0.000121
I -1.975392 0.226676 0.000077
Cl -2.408677 -2.255043 -0.000013

Indole_NMe_IBA_A_Cl.log
Energy (E) = -1061.43585793 Hartree
Enthalpy (H) = -1061.268690 Hartree
Gibbs free energy (G) = -1061.325058 Hartree
Charge = 0, Spin = 1
C 1.245795 -0.858796 0.000033
C 2.382338 -0.007168 -0.000187
C 3.684388 -0.510487 -0.000253
C 3.838229 -1.879064 -0.000104

C 2.723977 -2.736955 0.000134
C 1.436709 -2.248704 0.000211
C 0.152552 0.049015 0.000290
H 4.536765 0.155500 -0.000453
H 4.832893 -2.303155 -0.000158
H 2.883724 -3.806148 0.000293
H 0.591529 -2.918171 0.000372
N 1.981603 1.306828 -0.000203
C 0.617199 1.331579 0.000152
C -0.295877 2.496279 -0.000003
O -1.554492 2.107454 0.000033
O 0.061883 3.651850 -0.000232
I -1.917905 -0.013334 0.000016
Cl -2.026925 -2.488625 -0.000131
C 2.882970 2.443668 0.000274
H 3.515039 2.417215 -0.887025
H 2.291858 3.352955 0.000415
H 3.514862 2.416684 0.887659

Indole_NMe_IBA_B_Cl.log
Energy (E) = -1061.42347653 Hartree
Enthalpy (H) = -1061.256487 Hartree
Gibbs free energy (G) = -1061.313837 Hartree
Charge = 0, Spin = 1
C 2.216514 0.398016 -0.000068
C 2.089346 -1.004498 -0.000081
C 3.207525 -1.837890 -0.000174
C 4.447016 -1.228800 -0.000242
C 4.585575 0.169006 -0.000223
C 3.480798 0.993952 -0.000137
H 3.125611 -2.915388 -0.000207
H 5.334619 -1.846582 -0.000317
H 5.577317 0.599543 -0.000282
H 3.566387 2.071325 -0.000127
N 0.733884 -1.339119 0.000031
C 0.888019 0.922601 0.000015
C 0.063687 -0.156720 0.000058
C 0.348269 2.284751 0.000113
O 1.004803 3.294883 0.000037
O -0.989678 2.285966 0.000266
I -1.950288 0.407338 0.000112
Cl -2.892159 -1.920862 -0.000345
C 0.228903 -2.700269 0.000271
H -0.373883 -2.893868 0.883386
H -0.373420 -2.894384 -0.883049
H 1.083237 -3.368739 0.000707

Indole_NMs_IBA_A_Cl.log
Energy (E) = -1609.83667705 Hartree
Enthalpy (H) = -1609.656599 Hartree
Gibbs free energy (G) = -1609.720700 Hartree
Charge = 0, Spin = 1
C -0.070382 1.451101 0.162924
C -1.450761 1.226787 0.355595
C -2.353604 2.281449 0.470457
C -1.845441 3.564392 0.398490
C -0.477443 3.801433 0.216924
C 0.418885 2.760219 0.100820
C 0.487975 0.135477 0.083157
H -3.403256 2.096694 0.638284
H -2.521147 4.402450 0.498721
H -0.116261 4.819245 0.173772
H 1.471630 2.946983 -0.032352
N -1.699063 -0.159127 0.392325
C -0.474093 -0.806743 0.228773
C -0.101748 -2.236832 0.463690
O 1.173907 -2.425687 0.145806
O -0.810822 -3.082475 0.927671
I 2.385438 -0.697178 -0.069089
Cl 3.559992 1.485930 -0.299840
C -3.181470 -0.094495 -1.849319
H -3.165078 0.991003 -1.810844
H -2.314071 -0.494516 -2.368591
H -4.098885 -0.451046 -2.311690
S -3.189688 -0.747005 -0.207862
O -3.110053 -2.166437 -0.285499
O -4.201041 -0.081891 0.557293

Indole_NMs_IBA_B_Cl.log
Energy (E) = -1609.83346389 Hartree
Enthalpy (H) = -1609.653581 Hartree
Gibbs free energy (G) = -1609.716801 Hartree
Charge = 0, Spin = 1
C 1.946760 -1.168375 0.215979
C 2.013193 0.197674 0.531283
C 3.201109 0.821947 0.876870
C 4.333913 0.020840 0.915641
C 4.281525 -1.345657 0.617755
C 3.091289 -1.959509 0.266224
H 3.233774 1.874478 1.116896
H 5.279229 0.463636 1.197424
H 5.189038 -1.930837 0.670262
H 3.030265 -3.013882 0.037231
N 0.713626 0.780312 0.384795
C 0.563344 -1.453316 -0.083236
C -0.096283 -0.290090 0.033617
C -0.174605 -2.681564 -0.469215
O 0.356585 -3.755647 -0.616052
O -1.451885 -2.425291 -0.674545
I -2.167201 -0.507313 0.061460
Cl -2.586159 1.606511 1.224181
C 1.636334 1.656816 -2.010492
H 2.684857 1.627480 -1.732902
H 1.278267 0.680413 -2.333881
H 1.453441 2.400502 -2.782430
S 0.660768 2.167861 -0.627825
O 1.331472 3.212363 0.080913
O -0.694877 2.285391 -1.072311

NpthIBA_A_Cl.log
Energy (E) = -1044.19425823 Hartree
Enthalpy (H) = -1044.039086 Hartree
Gibbs free energy (G) = -1044.093639 Hartree
Charge = 0, Spin = 1
C -0.987289 2.717242 0.298698
C 0.066121 1.801878 0.094567
C -0.213855 0.475731 -0.026406
H -0.722159 3.761056 0.395872
C 1.463646 2.316233 -0.073655
O 2.323460 1.372592 -0.395294
O 1.737153 3.485214 0.027610
I 1.639614 -0.615383 -0.089424
Cl 0.839681 -2.904945 0.545024
C -2.268053 2.260956 0.367798
H -3.089250 2.943337 0.545446
C -3.189530 -1.759414 -0.471497
C -4.219098 -0.846757 -0.168714
C -3.910343 0.450957 0.127640
C -2.568093 0.892676 0.156803
C -1.523363 -0.052075 -0.080958
C -1.877243 -1.375904 -0.429686
H -3.437138 -2.774759 -0.748006
H -5.250489 -1.169950 -0.194710
H -4.689539 1.175766 0.326400
H -1.107820 -2.087674 -0.677007

NpthIBA_B_Cl.log
Energy (E) = -1044.20469742 Hartree
Enthalpy (H) = -1044.049517 Hartree
Gibbs free energy (G) = -1044.103793 Hartree
Charge = 0, Spin = 1
C 0.838044 0.554115 -0.000087
C -0.127218 -0.410196 -0.000285
C 0.405161 1.994532 0.000009
O -0.905769 2.147836 -0.000325
O 1.161168 2.934712 0.000277
I -2.082243 0.401289 -0.000014
Cl -3.169090 -1.842859 0.000284
C 1.398712 -2.198044 -0.000100
C 0.094497 -1.787695 -0.000392
H -0.726687 -2.487669 -0.000373
H 1.629741 -3.255737 -0.000038
C 2.467202 -1.272502 -0.000060
C 3.803801 -1.734410 -0.000007
C 4.845143 -0.850421 0.000111
C 4.585768 0.534855 0.000160
C 3.304164 1.018154 0.000067
C 2.203900 0.125785 -0.000048
H 3.979454 -2.802809 -0.000024
H 5.865657 -1.207981 0.000183
H 5.413672 1.230822 0.000280
H 3.113340 2.078237 0.000048

NpthIBA_C_Cl.log
Energy (E) = -1044.20704519 Hartree
Enthalpy (H) = -1044.051966 Hartree
Gibbs free energy (G) = -1044.106073 Hartree
Charge = 0, Spin = 1
C 1.734536 1.625798 0.000031
C 0.393311 1.355141 0.000020
C -0.011604 0.015681 -0.000036
C 0.833525 -1.042403 -0.000096
H 2.047598 2.662997 -0.000014
H 0.477708 -2.063439 -0.000153
C -0.631992 2.443919 -0.000035
O -1.871741 1.989478 -0.000076
O -0.340901 3.613252 -0.000022
I -2.120503 -0.124413 0.000013
Cl -2.038826 -2.611967 0.000050
C 3.175712 -1.817651 -0.000102
C 4.514309 -1.538126 -0.000039
C 4.967899 -0.200009 0.000069
C 4.072482 0.832605 0.000107
C 2.679902 0.577496 0.000034
C 2.226168 -0.769516 -0.000073
H 2.822282 -2.840683 -0.000154
H 5.234610 -2.344680 -0.000068
H 6.029966 0.002697 0.000123
H 4.410186 1.860992 0.000204

NpthIBA_D_Cl.log
Energy (E) = -1044.19293722 Hartree
Enthalpy (H) = -1044.037706 Hartree
Gibbs free energy (G) = -1044.091672 Hartree
Charge = 0, Spin = 1
C 0.603888 3.113322 -0.574092
C 1.894053 2.780842 -0.282224
C 2.265970 1.442646 -0.016747
C 1.298482 0.395464 -0.074765
C -0.031283 0.826347 -0.300109
C -0.388767 2.114071 -0.555518
H 4.313084 1.958571 0.373901
H 0.323316 4.134836 -0.785513

H 2.664469 3.540303 -0.242241
C 3.603934 1.141597 0.328208
C 1.744807 -0.944536 0.124618
H -1.424957 2.379965 -0.704145
C 3.051530 -1.178912 0.477789
C 3.987378 -0.139403 0.603853
H 3.346633 -2.210344 0.611633
H 5.008114 -0.363186 0.879568
I -1.584698 -0.595445 -0.077852
C 0.939046 -2.171653 -0.198373
O -0.191320 -1.967595 -0.862300
O 1.355797 -3.278958 0.034397
Cl -3.139513 1.165420 0.789431

PyIBA_A_Cl.log
Energy (E) = -906.734627372 Hartree
Enthalpy (H) = -906.641091 Hartree
Gibbs free energy (G) = -906.689048 Hartree
Charge = 0, Spin = 1
C 1.609728 2.629974 -0.000441
C 2.854971 2.013275 -0.000342
C 2.925249 0.626584 -0.000115
C 1.745378 -0.103716 0.000009
C 0.592073 0.646903 -0.000118
H 1.512655 3.707104 -0.000616
H 3.751238 2.615943 -0.000449
H 3.862600 0.085593 -0.000027
C 1.690778 -1.600531 0.000245
O 0.467160 -2.064542 0.000310
O 2.697479 -2.266761 0.000326
I -1.153157 -0.619267 0.000110
N 0.469078 1.928254 -0.000322
Cl -2.654053 1.311189 -0.000176

PyIBA_B_Cl.log
Energy (E) = -906.729728304 Hartree
Enthalpy (H) = -906.636105 Hartree
Gibbs free energy (G) = -906.683875 Hartree
Charge = 0, Spin = 1
C 2.838594 2.008919 0.000001
C 1.725205 -0.088501 0.000025
C 0.555647 0.633318 0.000013
H 3.741336 2.606665 -0.000005
C 1.681646 -1.590895 -0.000017
O 0.455094 -2.063238 -0.000008
O 2.683783 -2.258366 -0.000035
I -1.134903 -0.627753 0.000008
Cl -2.677776 1.304454 -0.000015
C 0.539540 2.014288 -0.000002
H -0.381332 2.579676 -0.000021
C 2.913360 0.623248 0.000031
H 3.850857 0.084837 0.000043
N 1.689461 2.684944 -0.000022

PyIBA_C_Cl.log
Energy (E) = -906.731199530 Hartree
Enthalpy (H) = -906.637646 Hartree
Gibbs free energy (G) = -906.685323 Hartree
Charge = 0, Spin = 1
C -1.715736 -0.086142 0.000011
C -0.550850 0.638358 0.000080
C -1.677807 -1.581450 -0.000112
O -0.447037 -2.057397 0.000277
O -2.674168 -2.256685 -0.000519
I 1.137221 -0.631237 0.000111
Cl 2.674494 1.315002 -0.000365
C -0.526272 2.010433 0.000248
H 0.388900 2.582240 0.000471
C -2.904817 0.639469 0.000015
H -3.842161 0.096128 -0.000085
C -1.778652 2.624224 0.000086
H -1.837151 3.705630 0.000154
N -2.936318 1.965689 -0.000036

PyIBA_D_Cl.log
Energy (E) = -906.725481107 Hartree
Enthalpy (H) = -906.632051 Hartree
Gibbs free energy (G) = -906.679864 Hartree
Charge = 0, Spin = 1
C 1.736034 2.651881 0.000010
C 2.900015 1.885733 0.000002
C 1.734798 -0.069685 0.000004
C 0.548774 0.635686 0.000009
H 1.792177 3.730661 0.000011
H 3.869503 2.368473 0.000005
C 1.693493 -1.578010 -0.000009
O 0.454480 -2.047442 0.000019
O 2.676117 -2.263594 -0.000046
I -1.125784 -0.634150 0.000011
Cl -2.686146 1.312386 -0.000031
C 0.506064 2.011738 0.000011
H -0.428995 2.552899 0.000015
N 2.905505 0.558794 -0.000003

PyraIBA_Cl.log
Energy (E) = -922.750216719 Hartree
Enthalpy (H) = -922.669015 Hartree
Gibbs free energy (G) = -922.716958 Hartree
Charge = 0, Spin = 1
C 2.816950 1.951721 -0.000030
C 1.594443 2.620387 0.000337
N 0.450539 1.934514 0.000177
C 0.573952 0.649614 -0.000293
C 1.762018 -0.058666 -0.000201
N 2.899295 0.625916 -0.000201
H 3.746960 2.505099 0.000192
H 1.532228 3.699767 0.000579
C 1.735352 -1.566681 -0.000072
I -1.137085 -0.630762 0.000091
Cl -2.664828 1.284362 -0.000244
O 2.736310 -2.229465 -0.000147
O 0.506598 -2.034269 0.000182

PyridaIBA_A_Cl.log
Energy (E) = -922.717641463 Hartree
Enthalpy (H) = -922.636917 Hartree
Gibbs free energy (G) = -922.684632 Hartree
Charge = 0, Spin = 1
N 2.908464 1.920791 0.000192
C 1.767143 2.604477 -0.000279
C 0.505383 2.011114 -0.000546
C 0.533152 0.646243 -0.000238
C 1.729928 -0.038755 0.000306
N 2.892049 0.608273 0.000511
H -0.407780 2.587675 -0.000921
H 1.869318 3.680984 -0.000510
C 1.718251 -1.548297 0.000132
I -1.125138 -0.643975 -0.000030
Cl -2.676677 1.299418 0.000271
O 0.483647 -2.029526 -0.000285
O 2.709795 -2.218005 -0.000056

PyridaIBA_B_Cl.log
Energy (E) = -922.722558710 Hartree
Enthalpy (H) = -922.641639 Hartree
Gibbs free energy (G) = -922.689358 Hartree
Charge = 0, Spin = 1
N 2.866873 2.015850 0.000006
C 2.880678 0.691204 -0.000031
C 1.713081 -0.069269 0.000110
C 0.547365 0.634440 0.000149
C 0.556997 2.015685 0.000112
N 1.719185 2.666137 -0.000033
H 3.843496 0.197234 0.000109
I -1.137050 -0.636453 -0.000007
Cl -2.658754 1.302193 -0.000081
H -0.340255 2.616082 -0.000162
C 1.697576 -1.571237 0.000065
O 0.475214 -2.053968 0.000291
O 2.710115 -2.220715 -0.000347

PyridaIBA_C_Cl.log
Energy (E) = -922.723452373 Hartree
Enthalpy (H) = -922.642685 Hartree
Gibbs free energy (G) = -922.690609 Hartree
Charge = 0, Spin = 1
N 0.435586 1.931046 -0.000462
C 0.580609 0.649475 -0.000123
C 1.757121 -0.073214 0.000236
C 2.717068 2.085259 0.000018
N 1.532707 2.685544 -0.000416
H 3.563772 2.757813 0.000078
C 1.736089 -1.576093 0.000458
O 2.761356 -2.211688 0.000564
O 0.522549 -2.053725 0.000329
C 2.893136 0.700217 0.000355
H 3.867342 0.230269 0.000682
I -1.136877 -0.628064 -0.000148
Cl -2.666475 1.258391 0.000025

PyrmIBA_Cl.log
Energy (E) = -922.763809134 Hartree
Enthalpy (H) = -922.682275 Hartree
Gibbs free energy (G) = -922.730142 Hartree
Charge = 0, Spin = 1
C 1.632100 2.601800 -0.000159
N 0.466155 1.939133 -0.000227
C 0.580889 0.655642 -0.000122
C 1.742198 -0.074235 -0.000003
C 2.890275 0.710105 0.000077
N 2.833299 2.039936 0.000085
H 1.577671 3.681835 -0.000110
H 3.861065 0.228647 0.000306
C 1.720334 -1.570914 -0.000050
O 2.738146 -2.217602 0.000381
O 0.502030 -2.049716 -0.000357
I -1.145690 -0.630721 -0.000025
Cl -2.654678 1.286377 0.000203

PyrroleIBA_A_Cl.log
Energy (E) = -868.660851719 Hartree
Enthalpy (H) = -868.572987 Hartree
Gibbs free energy (G) = -868.619439 Hartree
Charge = 0, Spin = 1
C -1.802833 2.567914 -0.000157
C -0.464613 2.229469 -0.000081
C -0.463951 0.826843 0.000022
C -1.738162 0.348816 0.000106
H -2.258668 3.542369 -0.000197
H 0.379186 2.895179 -0.000085

C -2.053968 -1.086472 0.000029
I 0.919100 -0.696432 -0.000086
Cl 2.750514 0.969088 0.000346
O -3.177097 -1.531429 0.000076
O -0.943647 -1.805878 -0.000168
N -2.559610 1.430182 0.000013
H -3.567157 1.366642 0.000093

PyrroleIBA_B_Cl.log
Energy (E) = -868.657550016 Hartree
Enthalpy (H) = -868.569579 Hartree
Gibbs free energy (G) = -868.615981 Hartree
Charge = 0, Spin = 1
C -2.567050 1.500522 0.000137
C -1.790010 0.369776 0.000003
C -0.460743 0.816967 0.000023
H -3.636999 1.607085 0.000185
I 0.898213 -0.720606 -0.000086
Cl 2.739548 0.963051 0.000263
C -0.409232 2.176009 0.000079
H 0.418116 2.860572 0.000084
C -2.077186 -1.078923 -0.000122
O -0.952762 -1.795377 -0.000196
O -3.177766 -1.568292 -0.000133
N -1.723869 2.574135 0.000206
H -2.022073 3.536908 0.000301

PyrroleIBA_C_Cl.log
Energy (E) = -868.652953147 Hartree
Enthalpy (H) = -868.565357 Hartree
Gibbs free energy (G) = -868.611775 Hartree
Charge = 0, Spin = 1
C 0.464321 0.790519 0.000044
C 1.777619 0.445753 0.000059
C 1.563415 2.679536 0.000014
H 1.687312 3.747931 -0.000004
C 2.117007 -0.987895 0.000081
O 3.223490 -1.455748 0.000061
O 1.011757 -1.758400 0.000073
C 2.494264 1.674000 0.000047
H 3.564137 1.787556 0.000064
I -0.854823 -0.774126 0.000038
Cl -2.695412 0.948952 -0.000262
N 0.298589 2.123856 -0.000016
H -0.595700 2.595723 -0.000042

PyrroleINAcBA_B_Cl.log
Energy (E) = -1021.19137618 Hartree
Enthalpy (H) = -1021.063022 Hartree
Gibbs free energy (G) = -1021.117770 Hartree
Charge = 0, Spin = 1
C -2.079974 1.116746 0.000019
C -0.725801 1.266899 0.000222
C -0.189221 -0.037564 0.000261
H -2.854712 1.861938 -0.000183
I 1.865871 -0.054078 0.000168
Cl 1.816545 -2.532127 -0.000394
C -1.171966 -0.966173 -0.000377
H -1.173854 -2.040129 -0.000398
C 0.180049 2.438023 0.000016
O 1.453100 2.059792 0.000123
O -0.185310 3.586016 -0.000548
N -2.351576 -0.237743 -0.000492
C -3.629001 -0.860305 -0.000087
C -4.804997 0.069896 0.000084
H -4.786706 0.710809 0.881085
H -4.787741 0.709613 -0.881860
H -5.708660 -0.529489 0.000928
O -3.702075 -2.058480 0.000531

PyrroleINMeBA_B_Cl.log
Energy (E) = -907.926678267 Hartree
Enthalpy (H) = -907.810288 Hartree
Gibbs free energy (G) = -907.859139 Hartree
Charge = 0, Spin = 1
Imaginary frequency: -32.6644
C -2.731487 0.131054 -0.000893
C -1.493000 0.727014 -0.000062
C -0.570150 -0.325476 0.000144
H -3.714092 0.570316 -0.001343
I 1.377077 0.322476 0.000167
Cl 2.127658 -2.059565 -0.000307
C -1.211603 -1.526249 -0.000653
H -0.842717 -2.535746 -0.000874
C -1.016239 2.123063 -0.000041
O 0.317035 2.180249 0.000375
O -1.721422 3.099959 -0.000194
N -2.550677 -1.220263 -0.001009
C -3.615576 -2.206144 0.001397
H -3.546773 -2.843444 -0.877728
H -3.563887 -2.822188 0.896784
H -4.569602 -1.686971 -0.014233

PyrroleINMsBA_B_Cl.log
Energy (E) = -1456.34453196 Hartree
Enthalpy (H) = -1456.214125 Hartree
Gibbs free energy (G) = -1456.272539 Hartree
Charge = 0, Spin = 1
C 1.480289 1.438843 0.124582
C 0.117101 1.419010 0.048877
C -0.259669 0.061419 0.049865
H 2.182611 2.252696 0.170568
I -2.295729 -0.206355 -0.022058
Cl -1.928991 -2.661247 0.013392
C 0.824737 -0.746632 0.128172
H 0.948047 -1.813005 0.180210
C -0.926106 2.469892 -0.011070
O -2.143088 1.935888 -0.051664
O -0.707920 3.653274 -0.025518
N 1.899537 0.126462 0.178050
C 3.830301 -0.454249 -1.594771
H 3.158457 -1.189861 -2.028417
H 3.679991 0.532648 -2.023692
H 4.866070 -0.767736 -1.703289
S 3.520576 -0.365357 0.136667
O 3.535772 -1.685317 0.687554
O 4.265914 0.720412 0.694509

PyrroleNAcIBA_A_Cl.log
Energy (E) = -1021.18081300 Hartree
Enthalpy (H) = -1021.052703 Hartree
Gibbs free energy (G) = -1021.107616 Hartree
Charge = 0, Spin = 1
C 1.680219 1.942726 0.077031
C 0.312553 1.939637 0.045498
C -0.026430 0.575165 -0.014634
C 1.081007 -0.213359 -0.019998
H 2.356504 2.778947 0.090282
H -0.349810 2.786022 0.056923
C 0.956373 -1.687105 0.140470
I -1.783183 -0.492145 -0.016412
Cl -3.065731 1.638122 -0.034495
O 1.843675 -2.454261 0.394480
O -0.320134 -2.046125 0.020199
N 2.158914 0.646982 0.033298
C 3.536161 0.304250 -0.173479
C 4.520609 1.327252 0.319529
H 4.548172 2.179039 -0.360879
H 4.258601 1.689250 1.311786
H 5.501276 0.863471 0.331042
O 3.830959 -0.714788 -0.721241

PyrroleNAcIBA_C_Cl.log
Energy (E) = -1021.17041023 Hartree
Enthalpy (H) = -1021.042402 Hartree
Gibbs free energy (G) = -1021.097366 Hartree
Charge = 0, Spin = 1
C -0.437964 2.872856 -0.399647
C 0.905845 2.667940 -0.414306
N 1.177439 1.317609 -0.160626
C -1.046964 1.608445 -0.141251
C -0.039654 0.716325 -0.004673
C -2.443408 1.152453 0.048597
O -3.411435 1.863665 -0.036117
O -2.485789 -0.136144 0.369894
I -0.674256 -1.253090 0.075731
Cl 1.490476 -2.219094 -0.648415
C 2.397908 0.861511 0.427551
C 3.630812 1.587897 -0.017000
H 3.612551 1.781486 -1.087497
H 3.705870 2.541912 0.506032
H 4.491069 0.981561 0.246369
O 2.370051 -0.023148 1.234483
H -0.953884 3.805903 -0.545783
H 1.717714 3.364673 -0.527156

PyrroleNMeIBA_A_Cl.log
Energy (E) = -907.931366686 Hartree
Enthalpy (H) = -907.813978 Hartree
Gibbs free energy (G) = -907.864503 Hartree
Charge = 0, Spin = 1
C 1.878800 2.238810 0.000210
C 0.503620 2.092022 0.000150
C 0.315785 0.707077 0.000098
C 1.516747 0.058809 -0.000004
H 2.469630 3.139439 0.000288
H -0.240595 2.867354 0.000196
C 1.621860 -1.406475 -0.000104
I -1.267375 -0.606923 0.000052
Cl -2.851855 1.296678 -0.000219
O 2.665479 -2.019037 -0.000209
O 0.420888 -1.962321 0.000203
N 2.484160 1.016688 -0.000013
C 3.916309 0.765907 -0.000152
H 4.198129 0.194522 0.880209
H 4.198000 0.194697 -0.880667
H 4.428448 1.724542 -0.000093

PyrroleNMeIBA_C_Cl.log
Energy (E) = -907.916130557 Hartree
Enthalpy (H) = -907.798836 Hartree
Gibbs free energy (G) = -907.848985 Hartree
Charge = 0, Spin = 1
C -0.499981 0.713233 0.000032
C -1.828062 0.394345 -0.000069
C -1.587441 2.608643 0.000262
H -1.688840 3.680690 0.000439
C -2.250035 -1.014518 -0.000267
O -3.387561 -1.405389 -0.000466
O -1.197199 -1.841794 -0.000409
C -2.532608 1.620530 -0.000073

H -3.601962 1.738001 -0.000184
I 0.724742 -0.969848 -0.000078
Cl 2.885912 0.312976 0.000537
N -0.322345 2.055589 0.000116
C 0.893844 2.853509 0.000178
H 1.493223 2.649622 0.882753
H 1.493299 2.649661 -0.882353
H 0.592643 3.897257 0.000184

PyrroleNMsIBA_A_Cl.log
Energy (E) = -1456.33119131 Hartree
Enthalpy (H) = -1456.200838 Hartree
Gibbs free energy (G) = -1456.258712 Hartree
Charge = 0, Spin = 1
C 1.243846 1.941291 0.000836
C -0.128107 1.947135 0.000411
C -0.482413 0.589142 0.000026
C 0.620477 -0.204610 0.000142
H 1.916938 2.778595 0.001368
H -0.777841 2.803536 0.000270
C 0.515988 -1.682023 0.000210
I -2.234305 -0.487760 -0.000064
Cl -3.517996 1.634448 -0.000074
O 1.447626 -2.441203 0.000781
O -0.759590 -2.046790 -0.000468
N 1.704667 0.640025 0.000684
C 4.200453 1.639679 -0.000985
H 3.971827 2.192601 -0.906981
H 3.974104 2.192785 0.905476
H 5.247206 1.341860 -0.002268
S 3.345178 0.097977 -0.000109
O 3.570129 -0.548074 1.252754
O 3.568693 -0.548336 -1.253081

PyrroleNMsIBA_C_Cl.log
Energy (E) = -1456.31871011 Hartree
Enthalpy (H) = -1456.188652 Hartree
Gibbs free energy (G) = -1456.246577 Hartree
Charge = 0, Spin = 1
C -0.053716 0.597930 0.228705
C -0.639129 1.809094 0.359402
C 1.471709 1.948209 1.094736
H 2.456101 2.215801 1.440993
C -2.040850 1.991953 -0.099396
O -2.631566 3.040948 -0.046154
O -2.516329 0.873601 -0.618633
C 0.346765 2.685352 0.914673
H 0.202788 3.725505 1.151627
I -1.402739 -0.935561 -0.169281
Cl -0.030709 -2.741469 0.764196
N 1.247335 0.621314 0.675814
C 2.701706 1.185637 -1.518640
H 1.712356 1.420232 -1.908102
H 3.183967 2.061327 -1.096832
H 3.316937 0.733698 -2.293020
S 2.521294 -0.047020 -0.265457
O 3.669156 -0.045101 0.585157
O 2.004254 -1.228544 -0.879666

QuinoIBA_A_Cl.log
Energy (E) = -1060.22569819 Hartree
Enthalpy (H) = -1060.082650 Hartree
Gibbs free energy (G) = -1060.138102 Hartree
Charge = 0, Spin = 1
C -1.136416 2.718138 0.141003
C -0.030084 1.845048 0.053035
C -0.234199 0.502856 -0.002227
H -0.919883 3.776784 0.186713
C 1.358972 2.412506 -0.025444
O 2.271359 1.491122 -0.186721
O 1.561643 3.601170 0.033553
I 1.619585 -0.565880 -0.047536
Cl 0.991596 -2.940856 0.253220
C -2.401850 2.213286 0.162391
H -3.260564 2.868312 0.235821
C -2.892122 -1.896031 -0.208758
C -4.054128 -1.102034 -0.089656
C -3.911708 0.249556 0.041279
C -2.618934 0.817865 0.065629
C -1.520696 -0.078794 -0.033557
H -2.978461 -2.969557 -0.333166
H -5.028928 -1.567231 -0.113267
H -4.771897 0.902295 0.121022
N -1.678920 -1.408157 -0.182756

QuinoIBA_B_Cl.log
Energy (E) = -1060.22719496 Hartree
Enthalpy (H) = -1060.084334 Hartree
Gibbs free energy (G) = -1060.139006 Hartree
Charge = 0, Spin = 1
C 0.849274 0.552631 0.000047
C -0.115075 -0.410983 0.000004
C 0.427387 2.002259 0.000050
O -0.895964 2.143081 0.000013
O 1.169290 2.942395 0.000103
I -2.066237 0.408955 -0.000009
Cl -3.156506 -1.849060 -0.000054
C 1.399885 -2.213466 0.000103
C 0.100039 -1.791186 0.000022
H -0.728027 -2.483444 0.000230
H 1.626066 -3.272288 0.000328
C 2.466911 -1.288016 0.000043
C 3.813509 -1.715842 0.000016
C 4.809887 -0.785051 -0.000049
C 4.449290 0.581152 -0.000105
C 2.211663 0.108937 -0.000022
H 4.028184 -2.777208 0.000071
H 5.852382 -1.069121 -0.000043
H 5.224802 1.339652 -0.000188
N 3.214929 1.013626 -0.000084

QuinoIBA_C_Cl.log
Energy (E) = -1060.23752908 Hartree
Enthalpy (H) = -1060.094496 Hartree
Gibbs free energy (G) = -1060.148406 Hartree
Charge = 0, Spin = 1
C 1.756540 1.610898 -0.000231
C 0.414125 1.345857 -0.000092
C -0.004205 0.010103 -0.000089
C 0.831805 -1.056285 -0.000210
H 2.101227 2.636216 -0.000070
H 0.468200 -2.074677 -0.000299
C -0.607113 2.442033 0.000036
O -1.849354 1.993099 0.000144
O -0.309937 3.608425 0.000023
I -2.112614 -0.116058 0.000042
Cl -2.042162 -2.604751 -0.000061
C 3.185380 -1.824514 0.000101
C 4.508599 -1.495048 0.000197
C 4.873404 -0.126596 0.000129
C 2.690187 0.551226 -0.000185
C 2.223024 -0.788829 -0.000042
H 2.856968 -2.855969 0.000114
H 5.278058 -2.253693 0.000292
H 5.923512 0.144496 0.000231
N 4.014453 0.858599 -0.000066

QuinoIBA_D_Cl.log
Energy (E) = -1060.23840873 Hartree
Enthalpy (H) = -1060.095340 Hartree
Gibbs free energy (G) = -1060.149228 Hartree
Charge = 0, Spin = 1
C 1.747038 1.627292 -0.000160
C 0.406202 1.355487 -0.000071
C 0.000934 0.015850 -0.000102
C 0.842579 -1.045200 -0.000258
H 2.060945 2.664313 -0.000083
H 0.511867 -2.073475 -0.000336
C -0.618169 2.447470 0.000020
O -1.855636 1.995457 0.000017
O -0.318193 3.614953 0.000072
I -2.109481 -0.124875 0.000016
Cl -2.032397 -2.601682 0.000075
C 4.371119 -1.576387 0.000086
C 4.925343 -0.274046 0.000166
C 2.686338 0.575748 -0.000034
C 2.235130 -0.769852 -0.000172
H 5.031445 -2.436692 0.000073
H 5.998601 -0.149507 0.000295
N 3.086646 -1.826343 -0.000054
C 4.083071 0.798487 0.000094
H 4.456997 1.814369 0.000181

QuinoIBA_E_Cl.log
Energy (E) = -1060.23782348 Hartree
Enthalpy (H) = -1060.094618 Hartree
Gibbs free energy (G) = -1060.148647 Hartree
Charge = 0, Spin = 1
C 0.842125 0.557857 -0.000082
C -0.119700 -0.409232 0.000019
C 0.412907 1.998734 -0.000050
O -0.895237 2.153527 0.000044
O 1.178278 2.932202 -0.000138
I -2.074694 0.400594 0.000059
Cl -3.152952 -1.839931 -0.000112
C 1.415473 -2.195377 -0.000064
C 0.111192 -1.786507 0.000127
H -0.707601 -2.489660 0.000296
H 1.677099 -3.244089 0.000057
C 2.478178 -1.261135 -0.000156
C 4.737425 -0.922820 0.000138
C 4.573709 0.478965 0.000097
C 2.204885 0.131706 -0.000151
H 5.734746 -1.349160 0.000207
H 5.444681 1.118895 0.000298
C 3.315595 1.010981 -0.000071
H 3.147031 2.075531 -0.000098
N 3.738281 -1.767006 -0.000006

QuinoIBA_F_Cl.log
Energy (E) = -1060.22688229 Hartree
Enthalpy (H) = -1060.083744 Hartree
Gibbs free energy (G) = -1060.138149 Hartree
Charge = 0, Spin = 1
C -1.035930 2.691744 0.280217
C 0.034958 1.792493 0.089840
C -0.220640 0.461285 -0.025974
H -0.785539 3.739764 0.373022
C 1.425932 2.329053 -0.064470
O 2.305947 1.397238 -0.368236
O 1.679001 3.502246 0.033278
I 1.650839 -0.599546 -0.084101

Cl 0.851593 -2.901606 0.509650
C -2.311509 2.221746 0.344838
H -3.159117 2.871705 0.507948
C -3.202662 -1.750292 -0.449742
C -4.175156 -0.780306 -0.130224
C -2.584677 0.845742 0.146568
C -1.521989 -0.081187 -0.079032
H -3.506567 -2.751887 -0.716958
H -5.225426 -1.049832 -0.120099
C -1.883074 -1.403665 -0.424231
H -1.125231 -2.126782 -0.676190
N -3.885824 0.466390 0.139786

QuinoIBA_G_Cl.log
Energy (E) = -1060.22964196 Hartree
Enthalpy (H) = -1060.086794 Hartree
Gibbs free energy (G) = -1060.140834 Hartree
Charge = 0, Spin = 1
C -4.917371 -0.169379 -0.000213
C -3.999645 0.843035 -0.000215
C -2.616125 0.546503 -0.000132
C -2.200056 -0.812491 -0.000053
C -3.172029 -1.839280 -0.000052
C -4.501352 -1.521395 -0.000130
H -5.974195 0.059370 -0.000277
H -4.287397 1.884734 -0.000277
C -0.813438 -1.083827 0.000022
H -2.844152 -2.870741 0.000012
H -5.244742 -2.306522 -0.000128
C 0.013701 -0.010260 0.000018
C -0.458479 1.313387 -0.000058
H -0.443633 -2.100626 0.000078
N -1.736839 1.578082 -0.000132
C 0.545504 2.441740 -0.000062
O 0.234608 3.599177 -0.000132
O 1.797213 2.007417 0.000029
I 2.112940 -0.091242 0.000110
Cl 2.083962 -2.586938 0.000104

QuinoIBA_H_Cl.log
Energy (E) = -1060.23236834 Hartree
Enthalpy (H) = -1060.089194 Hartree
Gibbs free energy (G) = -1060.143347 Hartree
Charge = 0, Spin = 1
C -4.796308 -0.905031 -0.000533
C -3.735099 -1.765082 -0.000345
C -2.413823 -1.264012 -0.000213
C -2.193255 0.142218 -0.000277
C -3.312402 1.009634 -0.000473
C -4.578563 0.489207 -0.000596
H -5.807315 -1.288842 -0.000634
H -3.858554 -2.838726 -0.000292
C -0.837414 0.577462 -0.000136
H -3.146551 2.074271 -0.000523
H -5.427384 1.159283 -0.000744
C 0.118573 -0.391428 0.000044
C -0.165252 -1.765782 0.000101
N -1.405694 -2.175737 -0.000026
I 2.080459 0.387232 0.000234
Cl 3.118525 -1.864195 0.000477
H 0.624667 -2.503661 0.000249
C -0.395989 2.021115 -0.000174
O 0.911966 2.157846 -0.000005
O -1.157849 2.955410 -0.000339

QuinoIBA_I_Cl.log
Energy (E) = -1060.22433834 Hartree
Enthalpy (H) = -1060.081117 Hartree
Gibbs free energy (G) = -1060.134964 Hartree
Charge = 0, Spin = 1
C 0.634337 3.109132 -0.530516
C 1.925405 2.768827 -0.254911
C 2.284626 1.424217 -0.002670
C 1.303160 0.390495 -0.061887
C -0.024199 0.826005 -0.279918
C -0.370181 2.119423 -0.518778
H 0.359102 4.134548 -0.730316
H 2.718697 3.501336 -0.211427
C 1.761212 -0.944330 0.122452
H -1.403217 2.398233 -0.664771
C 3.952022 -0.045263 0.546467
N 3.584820 1.185020 0.300937
H 4.992835 -0.205382 0.803197
C 0.955334 -2.183592 -0.173234
O 1.390221 -3.278891 0.078837
O -0.176366 -1.989463 -0.825776
C 3.074759 -1.142191 0.449670
H 3.426034 -2.155315 0.582964
I -1.588662 -0.593632 -0.080857
Cl -3.157519 1.159084 0.740864

QuinoIBA_J_Cl.log
Energy (E) = -1060.22353292 Hartree
Enthalpy (H) = -1060.080430 Hartree
Gibbs free energy (G) = -1060.134217 Hartree
Charge = 0, Spin = 1
C -4.002157 -0.089258 0.549306
C -3.594238 1.187152 0.290221
C -2.245066 1.463489 -0.031409
C -1.297608 0.395555 -0.079740
C -3.082721 -1.147393 0.442764
H -5.031735 -0.296866 0.804203
H -4.267954 2.031658 0.323776
C 0.034261 0.823079 -0.274547
H -3.398152 -2.172825 0.576294
C -0.682462 3.067234 -0.510166
N -1.924860 2.762006 -0.259944
H -0.451554 4.107547 -0.706818
C 0.365266 2.118604 -0.500329
H 1.388422 2.436694 -0.630437
I 1.594020 -0.599426 -0.074555
C -1.766129 -0.935526 0.116725
C -0.959349 -2.169097 -0.166808
O -1.380921 -3.270934 0.078557
O 0.184601 -1.977370 -0.813098
Cl 3.159040 1.178396 0.721424

QuinoIBA_K_Cl.log
Energy (E) = -1060.22398787 Hartree
Enthalpy (H) = -1060.080944 Hartree
Gibbs free energy (G) = -1060.135767 Hartree
Charge = 0, Spin = 1
C -4.216534 -0.948550 -0.110650
C -3.926805 0.369526 0.104388
C -2.590571 0.824454 0.117005
C -1.531467 -0.124368 -0.058061
C -3.175781 -1.871141 -0.335420
H -5.244273 -1.283972 -0.125793
H -4.695753 1.114123 0.253222
C -0.241843 0.443256 -0.018180
H -3.411165 -2.907002 -0.535694
C -1.174244 2.616505 0.218462
N -2.390553 2.160607 0.270302
H -0.997706 3.682989 0.302275
C -0.039254 1.780893 0.067758
I 1.681818 -0.539360 -0.067511
C -1.867201 -1.473601 -0.309780
Cl 1.033879 -2.915340 0.385157
H -1.086463 -2.192498 -0.490215
C 1.320998 2.389770 -0.048130
O 2.256279 1.494489 -0.284478
O 1.507862 3.576577 0.030748

QuinoIBA_L_Cl.log
Energy (E) = -1060.24022236 Hartree
Enthalpy (H) = -1060.097275 Hartree
Gibbs free energy (G) = -1060.151519 Hartree
Charge = 0, Spin = 1
C -4.396109 -1.576749 -0.000001
C -3.046449 -1.807665 0.000042
C -2.154167 -0.718522 -0.000040
C -2.656024 0.610757 -0.000173
C -4.905214 -0.259426 -0.000125
H -5.084337 -2.410698 0.000061
H -2.631763 -2.805386 0.000138
H -5.974774 -0.102596 -0.000153
C -0.050840 0.059834 -0.000092
N -0.806526 -0.955520 0.000011
C -0.388346 1.416907 -0.000253
C -4.055454 0.812387 -0.000208
H -4.435620 1.825632 -0.000304
C 0.669717 2.474277 -0.000526
O 0.388572 3.648597 0.000879
O 1.884684 1.984733 0.000645
C -1.731344 1.678530 -0.000265
H -2.059332 2.711512 -0.000391
I 2.102698 -0.174666 0.000065
Cl 1.911051 -2.616538 -0.000306

ThiopheneIBA_A_Cl.log
Energy (E) = -1211.43799442 Hartree
Enthalpy (H) = -1211.365872 Hartree
Gibbs free energy (G) = -1211.413215 Hartree
Charge = 0, Spin = 1
C 1.876471 2.387727 0.000150
C 0.551468 2.049658 0.000141
C 0.445010 0.647342 -0.000047
C 1.608943 -0.043683 -0.000121
H 2.288289 3.383272 0.000193
H -0.274957 2.740577 0.000214
C 1.642086 -1.525298 -0.000094
I -1.202160 -0.620258 0.000070
Cl -2.726316 1.335096 -0.000186
O 2.655595 -2.175162 -0.000147
O 0.417591 -2.027411 0.000080
S 2.919947 1.036205 -0.000035

ThiopheneIBA_B_Cl.log
Energy (E) = -1211.43777767 Hartree
Enthalpy (H) = -1211.365864 Hartree
Gibbs free energy (G) = -1211.413197 Hartree
Charge = 0, Spin = 1
C 2.806453 0.472964 0.000119
C 1.673786 -0.284071 0.000072
C 0.529870 0.534943 -0.000094
H 3.823010 0.116529 0.000253
I -1.223810 -0.576433 -0.000089
Cl -2.544811 1.527224 0.000221
C 0.745626 1.868093 -0.000230
H 0.032689 2.673706 -0.000386
C 1.525353 -1.763752 0.000182
O 0.251698 -2.132393 0.000037

O 2.453082 -2.530942 0.000246
S 2.433955 2.133470 -0.000092

ThiopheneIBA_C_Cl.log
Energy (E) = -1211.43142699 Hartree
Enthalpy (H) = -1211.359306 Hartree
Gibbs free energy (G) = -1211.406709 Hartree
Charge = 0, Spin = 1
C 0.511623 0.596481 0.000229
C 1.757689 0.069712 -0.000041
C 2.174183 2.329584 -0.000091
H 2.668210 3.287105 -0.000165
C 1.913550 -1.408344 0.000008
O 2.977861 -1.970383 -0.000537
O 0.741433 -2.034451 0.000068
C 2.742132 1.093544 -0.000281
H 3.802653 0.895681 -0.000506
I -1.000742 -0.804372 0.000382
Cl -2.650994 1.048770 -0.001161
S 0.455372 2.285792 0.000311

AnthIBA_A_CF3.log
Energy (E) = -1075.04222240 Hartree
Enthalpy (H) = -1074.822146 Hartree
Gibbs free energy (G) = -1074.889482 Hartree
Charge = 0, Spin = 1
C -6.610795 -0.817707 0.000137
C -5.514956 -1.622963 0.000050
C -4.204692 -1.062564 0.000018
C -4.057852 0.357846 0.000078
C -5.231754 1.166942 0.000169
C -6.467460 0.598827 0.000198
C -3.057271 -1.848722 -0.000072
C -2.787073 0.933972 0.000047
C -1.634567 0.148839 -0.000037
C -1.787036 -1.279600 -0.000097
C -0.634853 -2.112346 -0.000204
H -0.772559 -3.186153 -0.000288
C 0.619043 -1.589801 -0.000210
C 0.735071 -0.183230 -0.000110
C -0.297455 0.696994 -0.000061
H -3.149511 -2.929155 -0.000121
H -7.602408 -1.249803 0.000161
H -5.616481 -2.701244 0.000003
H -5.111547 2.242790 0.000215
H -7.352216 1.220799 0.000266
H -2.688775 2.007889 0.000096
H 1.477020 -2.239689 -0.000317
C 0.000179 2.191271 -0.000110
O -0.884024 3.023023 0.000126
O 1.264753 2.460665 -0.000266
I 2.656055 0.736091 -0.000046
C 3.779293 -1.193119 0.000119
F 3.576711 -1.947820 1.077378
F 3.576745 -1.947988 -1.077036
F 5.059077 -0.810366 0.000106

AnthIBA_B_CF3.log
Energy (E) = -1075.04474089 Hartree
Enthalpy (H) = -1074.824684 Hartree
Gibbs free energy (G) = -1074.891519 Hartree
Charge = 0, Spin = 1
C -6.613496 -0.174671 0.000023
C -5.668528 0.802406 -0.000002
C -4.280597 0.474786 0.000001
C -3.898418 -0.905245 0.000032
C -4.920107 -1.899646 0.000057
C -6.233086 -1.547051 0.000053
C -3.293134 1.456027 -0.000024
C -2.546227 -1.239272 0.000035
C -1.560627 -0.256288 0.000008
C -1.939784 1.124201 -0.000022
C -0.922953 2.116560 -0.000041
H -1.173372 3.170613 -0.000047
C 0.396380 1.780094 -0.000039
C 0.729177 0.409732 -0.000025
H -3.580023 2.501261 -0.000045
H -7.663489 0.084148 0.000021
H -5.949962 1.848020 -0.000024
H -4.624809 -2.941539 0.000080
H -7.000222 -2.309362 0.000073
H -2.257551 -2.284323 0.000057
I 2.839555 0.118927 -0.000066
C -0.172965 -0.596223 0.000006
H 0.093876 -1.641748 0.000030
C 1.467332 2.846115 -0.000007
O 2.667805 2.360212 -0.000003
O 1.158906 4.018674 -0.000107
C 2.650900 -2.100289 0.000086
F 2.039314 -2.587879 1.077018
F 2.039500 -2.588039 -1.076878
F 3.908101 -2.547272 0.000235

AnthIBA_C_CF3.log
Energy (E) = -1075.03472223 Hartree
Enthalpy (H) = -1074.814530 Hartree
Gibbs free energy (G) = -1074.880992 Hartree
Charge = 0, Spin = 1
C -5.006761 -1.220538 -0.628676
C -3.650487 -1.293677 -0.698899
C -2.844765 -0.178119 -0.330216
C -3.483797 1.023205 0.103146
C -4.906604 1.061246 0.163063
C -5.644070 -0.025701 -0.190258
C -1.453912 -0.232252 -0.384306
C -2.695385 2.127530 0.417828
C -1.306784 2.084632 0.349992
C -0.665213 0.848698 -0.015143
C 0.755306 0.891155 -0.045586
C 1.488876 2.018400 0.072277
H -0.997063 -1.132537 -0.764786
H -5.611915 -2.073261 -0.904488
H -3.156812 -2.201176 -1.024088
H -5.385374 1.974646 0.493211
H -6.723778 0.011344 -0.141768
H -3.174533 3.058262 0.699965
C -0.517655 3.251618 0.575534
H -1.029085 4.165429 0.850197
C 2.976438 2.045615 -0.228978
O 3.598304 3.074495 -0.065718
O 3.414276 0.925727 -0.695720
C 0.826213 3.234662 0.399738
H 1.451071 4.110836 0.503199
I 2.038880 -0.826879 -0.279760
C 0.697599 -2.419718 0.560643
F -0.065492 -2.007231 1.560396
F -0.063983 -3.068337 -0.320934
F 1.579723 -3.301240 1.036801

AnthIBA_D_CF3.log
Energy (E) = -1075.02633025 Hartree
Enthalpy (H) = -1074.806125 Hartree
Gibbs free energy (G) = -1074.871976 Hartree
Charge = 0, Spin = 1
C -3.805542 1.932544 1.056407
C -2.718000 2.742097 0.979567
C -1.484453 2.256792 0.458833
C -1.380370 0.882758 0.049636
C -2.582514 0.097362 0.012570
C -3.734290 0.620733 0.527639
C -0.379843 3.096558 0.372581
C -0.097575 0.448852 -0.290211
C 1.004385 1.283254 -0.476010
C 0.839826 2.665112 -0.132439
C 1.939425 3.557454 -0.285926
H 1.794885 4.592952 -0.004507
C 3.128429 3.125268 -0.780233
C 3.279407 1.765307 -1.161526
C 2.260394 0.877146 -1.010963
H -0.480014 4.125768 0.698090
H -4.739294 2.298367 1.459814
H -2.758407 3.772962 1.308429
H -4.621038 0.005637 0.453774
H 3.956174 3.810068 -0.899363
H 4.215324 1.434080 -1.589852
H 2.394392 -0.142971 -1.339308
C -2.733448 -1.189821 -0.783662
O -1.710735 -1.515311 -1.520389
O -3.790568 -1.785558 -0.755380
I 0.183604 -1.650170 -0.326608
C 1.933029 -1.524684 1.080285
F 3.119263 -1.613709 0.479691
F 1.926667 -0.441021 1.843007
F 1.810098 -2.595410 1.869524

AnthIBA_E_CF3.log
Energy (E) = -1075.02271932 Hartree
Enthalpy (H) = -1074.802521 Hartree
Gibbs free energy (G) = -1074.869057 Hartree
Charge = 0, Spin = 1
C 5.457354 0.048993 1.133976
C 4.720597 1.130063 0.772629
C 3.388953 0.972917 0.290223
C 2.819579 -0.333866 0.145971
C 3.633369 -1.439897 0.541514
C 4.891387 -1.248482 1.027454
C 2.648632 2.085659 -0.086021
C 1.497895 -0.475370 -0.343312
C 0.703533 0.678781 -0.501066
C 1.317369 1.978078 -0.461921
C 0.578634 3.145249 -0.811617
H 1.106103 4.090744 -0.813124
C -0.739032 3.084112 -1.127790
C -1.413333 1.840918 -1.009390
C -0.711716 0.724687 -0.693889
H 3.112031 3.065755 -0.067103
H 6.464833 0.168756 1.508392
H 5.118859 2.134004 0.851547
H 3.231073 -2.434087 0.458974
H 5.472666 -2.105358 1.340635
H -1.295015 3.967130 -1.406604
H -2.485542 1.806495 -1.133595
I -1.821830 -0.981752 -0.153542
C 1.056534 -1.804369 -0.940384
O 1.873317 -2.675462 -1.163036
O -0.186010 -1.900431 -1.313758
C -3.271947 0.286343 0.984202
F -2.687335 1.221383 1.722887
F -4.193466 0.880450 0.221280
F -3.911788 -0.556982 1.799109

FuranIBA_A_CF3.log
Energy (E) = -765.849284123 Hartree
Enthalpy (H) = -765.758589 Hartree

Gibbs free energy (G) = -765.811167 Hartree
Charge = 0, Spin = 1
C 1.776297 2.663039 -0.000385
C 0.505369 2.180797 -0.000149
C 0.691472 0.773081 -0.000102
C 2.014140 0.506224 -0.000230
O 2.688724 1.665644 -0.000398
H 2.164415 3.665568 -0.000528
H -0.404693 2.752090 -0.000069
C 2.602326 -0.866877 -0.000221
I -0.495353 -0.916241 0.000207
O 3.794062 -1.065254 -0.000170
O 1.639705 -1.742921 0.000179
C -2.290528 0.387144 0.000011
F -2.333737 1.167581 1.074794
F -2.334012 1.166842 -1.075302
F -3.363407 -0.398543 0.000420

FuranIBA_B_CF3.log
Energy (E) = -765.848665963 Hartree
Enthalpy (H) = -765.757870 Hartree
Gibbs free energy (G) = -765.810503 Hartree
Charge = 0, Spin = 1
C -2.644874 1.758984 -0.000085
C -2.077056 0.529979 -0.000205
C -0.686399 0.781205 0.000137
O -1.687988 2.717374 0.000277
H -3.667845 2.087152 -0.000266
I 0.481774 -0.925834 0.000148
C -0.477663 2.114945 0.000478
H 0.390464 2.746415 0.000763
C -2.614389 -0.866324 -0.000386
O -1.645635 -1.737663 0.000008
O -3.802796 -1.095789 -0.000786
C 2.283765 0.371579 -0.000073
F 2.329251 1.153773 -1.074583
F 2.330179 1.152956 1.075003
F 3.355500 -0.415390 -0.000813

FuranIBA_C_CF3.log
Energy (E) = -765.841656367 Hartree
Enthalpy (H) = -765.751137 Hartree
Gibbs free energy (G) = -765.803938 Hartree
Charge = 0, Spin = 1
C -0.743215 0.740780 -0.000124
C -2.072415 0.569626 -0.000384
O -0.346863 2.009742 -0.000125
C -1.518242 2.729791 -0.000323
H -1.400003 3.797711 -0.000339
C -2.639881 -0.813062 -0.000213
O -3.831370 -1.023287 -0.000519
O -1.686587 -1.705448 0.000292
C -2.591298 1.905463 -0.000498
H -3.629091 2.188913 -0.000701
I 0.460570 -0.941059 0.000261
C 2.256050 0.371930 0.000056
F 2.308595 1.141369 -1.076288
F 2.308865 1.141807 1.076057
F 3.314925 -0.437147 0.000113

Indole_NAc_IBA_A_CF3.log
Energy (E) = -1052.03855570 Hartree
Enthalpy (H) = -1051.844633 Hartree
Gibbs free energy (G) = -1051.911640 Hartree
Charge = 0, Spin = 1
C -0.562056 1.353754 0.025809
C -1.975107 1.276585 0.086324
C -2.768405 2.422694 0.115224
C -2.128777 3.645327 0.097986
C -0.732394 3.738855 0.048730
C 0.055757 2.609246 0.009255
C -0.139843 -0.013834 -0.016471
H -3.841500 2.347320 0.173787
H -2.721019 4.549276 0.131043
H -0.263729 4.712972 0.041312
H 1.126377 2.709857 -0.035233
N -2.357868 -0.067064 0.053931
C -1.216033 -0.839562 0.015807
C -1.105427 -2.317966 0.254687
O 0.114751 -2.714806 0.135565
O -2.075275 -2.979560 0.566888
I 1.680128 -1.048456 0.020134
C -3.716869 -0.483695 -0.114594
C -3.995036 -1.658121 -1.003937
H -4.287939 -2.507960 -0.394185
H -4.830000 -1.371532 -1.640665
H -3.138601 -1.949861 -1.603095
O -4.586163 0.187792 0.375412
C 2.993893 0.733009 -0.095137
F 2.804347 1.464446 -1.185200
F 2.922583 1.523481 0.967224
F 4.220844 0.212336 -0.146759

Indole_NAc_IBA_B_CF3.log
Energy (E) = -1052.02886098 Hartree
Enthalpy (H) = -1051.835006 Hartree
Gibbs free energy (G) = -1051.900762 Hartree
Charge = 0, Spin = 1
C 2.516591 -0.480840 0.105545
C 2.241112 0.897386 0.125686
C 3.256647 1.822699 0.338488
C 4.542839 1.330611 0.509211
C 4.823778 -0.037363 0.474049
C 3.812334 -0.959671 0.277359
H 3.081109 2.883497 0.404119
H 5.346409 2.032399 0.686110
H 5.841449 -0.374075 0.614048
H 3.988410 -2.025608 0.258953
N 0.842144 1.069136 -0.087927
C 1.264956 -1.171950 -0.068542
C 0.324252 -0.223495 -0.160140
C 0.940595 -2.625803 -0.198418
O 1.808797 -3.469773 -0.096654
O -0.315650 -2.806124 -0.451818
I -1.642540 -0.952438 -0.142710
C 0.212159 2.124032 -0.772180
C 0.856968 3.473012 -0.734241
H 1.078272 3.772451 0.288524
H 1.789177 3.461971 -1.298519
H 0.167649 4.175634 -1.190156
O -0.822131 1.907391 -1.358067
C -2.654751 0.909018 0.571254
F -1.842624 1.704760 1.257034
F -3.282932 1.604007 -0.353414
F -3.577319 0.432482 1.414151

Indole_NH_IBA_A_CF3.log
Energy (E) = -899.512591715 Hartree
Enthalpy (H) = -899.359393 Hartree
Gibbs free energy (G) = -899.418542 Hartree
Charge = 0, Spin = 1
C -1.551582 -0.628304 0.000051
C -2.758495 0.125741 -0.000111
C -4.012634 -0.482025 -0.000106
C -4.060287 -1.858701 0.000071
C -2.881757 -2.621235 0.000257
C -1.638190 -2.027652 0.000256
C -0.524964 0.369638 -0.000013
H -4.914598 0.114808 -0.000229
H -5.018160 -2.359968 0.000077
H -2.951354 -3.700062 0.000407
H -0.755227 -2.644880 0.000399
H -3.065667 2.258508 -0.000272
N -2.442986 1.463109 -0.000199
C -1.095619 1.602757 -0.000093
C -0.369307 2.903590 -0.000005
O 0.907169 2.722875 0.000126
O -0.998496 3.944744 -0.000242
I 1.561828 0.497883 -0.000001
C 1.880193 -1.690035 0.000013
F 1.388864 -2.288600 1.077533
F 1.388838 -2.288546 -1.077518
F 3.204018 -1.845246 -0.000005

Indole_NH_IBA_B_CF3.log
Energy (E) = -899.501970568 Hartree
Enthalpy (H) = -899.349215 Hartree
Gibbs free energy (G) = -899.408728 Hartree
Charge = 0, Spin = 1
C -2.476949 0.130885 0.000092
C -2.119880 -1.232939 0.000064
C -3.071387 -2.249398 0.000093
C -4.397644 -1.863367 0.000151
C -4.771644 -0.509015 0.000181
C -3.826344 0.495975 0.000154
H -2.788302 -3.293147 0.000071
H -5.167559 -2.622892 0.000175
H -5.822725 -0.255302 0.000229
H -4.096375 1.542463 0.000180
H -0.187440 -2.165187 -0.000043
N -0.731224 -1.317917 0.000006
C -1.255395 0.883678 0.000057
C -0.268424 -0.036708 -0.000010
C -0.937569 2.332340 0.000080
O -1.794016 3.188969 -0.000186
O 0.358225 2.544691 -0.000265
I 1.660331 0.735578 -0.000121
C 2.443413 -1.352954 0.000092
F 2.046001 -2.038541 1.074916
F 2.045323 -2.039010 -1.074180
F 3.770975 -1.303157 -0.000334

Indole_NMe_IBA_A_CF3.log
Energy (E) = -938.782133938 Hartree
Enthalpy (H) = -938.599285 Hartree
Gibbs free energy (G) = -938.662075 Hartree
Charge = 0, Spin = 1
C 1.119000 1.090437 -0.000006
C 2.489200 0.719730 0.000173
C 3.517059 1.662892 0.000217
C 3.165610 2.994902 0.000084
C 1.816243 3.383209 -0.000177
C 0.797243 2.455560 -0.000255
C 0.437833 -0.162341 -0.000357
H 4.552697 1.350669 0.000305
H 3.937161 3.752281 0.000088
H 1.570306 4.436008 -0.000401
H -0.225086 2.793858 -0.000605
N 2.599992 -0.648562 0.000079
C 1.345772 -1.184331 -0.000086
C 0.962905 -2.634298 0.000023
O -0.318966 -2.772742 -0.000464

O 1.797452 -3.520464 0.000636
I -1.538978 -0.834650 -0.000080
C 3.867049 -1.357922 -0.000182
H 4.440084 -1.089444 0.887134
H 3.662833 -2.422646 -0.000201
H 4.439859 -1.089318 -0.887608
C -2.464936 1.176813 0.000138
F -2.175616 1.895268 1.078119
F -2.175934 1.895571 -1.077734
F -3.777086 0.933909 0.000302

Indole_NMe_IBA_B_CF3.log
Energy (E) = -938.764115810 Hartree
Enthalpy (H) = -938.581497 Hartree
Gibbs free energy (G) = -938.644278 Hartree
Charge = 0, Spin = 1
C 2.542810 0.227334 0.073108
C 2.217071 -1.137615 -0.029788
C 3.190860 -2.133970 -0.022718
C 4.504220 -1.722993 0.106002
C 4.844432 -0.364841 0.218772
C 3.877767 0.620159 0.202533
H 2.936945 -3.182784 -0.095495
H 5.289414 -2.466525 0.125405
H 5.885800 -0.091619 0.319283
H 4.120069 1.670219 0.282701
N 0.837094 -1.267533 -0.165071
C 1.309032 0.949429 0.007860
C 0.345875 0.008426 -0.137637
C 0.998412 2.398548 -0.005254
O 1.854776 3.247394 0.119316
O -0.278809 2.614602 -0.194466
I -1.597516 0.815142 -0.103683
C 0.206426 -2.497573 -0.596235
H -0.068594 -3.130889 0.244801
H -0.678870 -2.277223 -1.183447
H 0.903212 -3.035891 -1.236251
C -2.689687 -1.116178 0.220121
F -2.911966 -1.852944 -0.867511
F -2.129784 -1.891043 1.144160
F -3.877179 -0.711056 0.676052

Indole_NMs_IBA_A_CF3.log
Energy (E) = -1487.18050980 Hartree
Enthalpy (H) = -1486.984736 Hartree
Gibbs free energy (G) = -1487.055247 Hartree
Charge = 0, Spin = 1
C 0.217603 1.361624 -0.144177
C 1.614131 1.296234 -0.341042
C 2.396015 2.443115 -0.452762
C 1.756518 3.665080 -0.366687
C 0.374232 3.748220 -0.170331
C -0.401445 2.612378 -0.060270
C -0.190131 -0.013465 -0.071570
H 3.458440 2.371200 -0.627711
H 2.336914 4.571888 -0.464146
H -0.097189 4.719007 -0.109364
H -1.462757 2.707658 0.089065
N 2.011896 -0.052058 -0.381164
C 0.874383 -0.838828 -0.223048
C 0.695544 -2.324386 -0.458653
O -0.463739 -2.696634 -0.007413
O 1.503966 -2.985433 -1.058545
I -2.003965 -1.047866 0.094373
C 3.517996 0.190477 1.829463
H 3.385788 1.267698 1.787115
H 2.707210 -0.296501 2.365615
H 4.475866 -0.064152 2.276926
S 3.568514 -0.473604 0.192487
O 3.651169 -1.890997 0.291184
O 4.488206 0.291210 -0.595963
C -3.319313 0.743693 0.089622
F -3.177871 1.528233 1.153413
F -3.210893 1.488785 -1.002849
F -4.547195 0.222107 0.121851

Indole_NMs_IBA_B_CF3.log
Energy (E) = -1487.18603463 Hartree
Enthalpy (H) = -1486.990367 Hartree
Gibbs free energy (G) = -1487.059142 Hartree
Charge = 0, Spin = 1
C 2.390975 0.837044 -0.302425
C 2.172087 -0.528055 -0.532078
C 3.194148 -1.397409 -0.881369
C 4.459278 -0.842063 -1.012787
C 4.692028 0.522187 -0.802566
C 3.665495 1.380199 -0.447194
H 3.007927 -2.446613 -1.055887
H 5.283840 -1.480399 -1.299000
H 5.693720 0.909807 -0.926424
H 3.818523 2.437108 -0.282995
N 0.790576 -0.821181 -0.303635
C 1.114578 1.420974 0.038163
C 0.228129 0.414285 0.031150
C 0.715783 2.822519 0.388483
O 1.542049 3.714049 0.390048
O -0.535498 2.889102 0.699201
I -1.783130 1.005921 0.144405
C 1.528297 -1.763188 2.126280
H 2.563946 -1.926822 1.844947
H 1.350718 -0.730519 2.422970
H 1.222432 -2.444680 2.916291
S 0.485297 -2.121353 0.748276
O 0.934445 -3.308848 0.090612
O -0.878938 -1.958539 1.167051
C -2.695737 -0.822348 -0.772770
F -1.841921 -1.548708 -1.467345
F -3.340226 -1.575473 0.092997
F -3.597947 -0.308346 -1.613760

NpthIBA_A_CF3.log
Energy (E) = -921.538621860 Hartree
Enthalpy (H) = -921.367792 Hartree
Gibbs free energy (G) = -921.427922 Hartree
Charge = 0, Spin = 1
C -2.120008 2.315524 0.480794
C -0.801864 1.924598 0.159849
C -0.552575 0.609472 -0.067069
H -2.282244 3.367804 0.669525
C 0.262960 2.989704 -0.035750
O 1.357218 2.518560 -0.529931
O 0.003138 4.146264 0.222064
I 1.568116 0.274274 -0.269975
C -3.112162 1.383105 0.540809
H -4.122181 1.667195 0.807640
C -2.409488 -2.580990 -0.697952
C -3.696381 -2.194140 -0.272771
C -3.915290 -0.913546 0.152012
C -2.864337 0.031154 0.199043
C -1.547949 -0.387510 -0.154511
C -1.364652 -1.700952 -0.638749
H -2.248385 -3.577033 -1.086104
H -4.511397 -2.903337 -0.311266
H -4.906141 -0.588546 0.443199
H -0.399530 -1.999850 -1.014600
C 1.660519 -1.851909 0.439760
F 0.768572 -2.137256 1.375797
F 1.602163 -2.785347 -0.508621
F 2.878624 -1.918330 0.980651

NpthIBA_B_CF3.log
Energy (E) = -921.546195779 Hartree
Enthalpy (H) = -921.375434 Hartree
Gibbs free energy (G) = -921.436602 Hartree
Charge = 0, Spin = 1
C 1.238036 0.623002 -0.000085
C 0.164909 -0.219215 0.000075
C 0.997519 2.128323 -0.000358
O -0.256176 2.444692 0.000352
O 1.911746 2.926380 0.000561
I -1.716566 0.778510 -0.000012
C 1.469532 -2.186700 0.000273
C 0.228115 -1.618573 0.000228
H -0.650939 -2.239899 0.000332
H 1.568888 -3.264734 0.000418
C 2.639637 -1.395893 0.000131
C 3.909612 -2.017955 0.000138
C 5.051021 -1.268111 -0.000034
C 4.959728 0.137917 -0.000231
C 3.746211 0.773011 -0.000239
C 2.541954 0.023967 -0.000043
H 3.952770 -3.099959 0.000270
H 6.020625 -1.747119 -0.000036
H 5.865232 0.729583 -0.000386
H 3.682177 1.847815 -0.000395
C -2.913312 -1.105897 -0.000167
F -2.739265 -1.867727 -1.077474
F -2.739056 -1.868053 1.076886
F -4.177577 -0.675170 0.000034

NpthIBA_C_CF3.log
Energy (E) = -921.549480411 Hartree
Enthalpy (H) = -921.378755 Hartree
Gibbs free energy (G) = -921.439328 Hartree
Charge = 0, Spin = 1
C 2.230500 1.532056 0.000321
C 0.861655 1.507281 0.000154
C 0.229451 0.259391 -0.000203
C 0.886182 -0.930365 -0.000440
H 2.714767 2.501130 0.000575
H 0.389066 -1.888062 -0.000776
C 0.064437 2.792192 0.000147
O -1.214868 2.595068 -0.000491
O 0.636330 3.861177 0.000314
I -1.892142 0.450790 -0.000239
C 3.055674 -2.106363 -0.000495
C 4.422110 -2.064752 -0.000304
C 5.101224 -0.825218 0.000127
C 4.398970 0.347272 0.000351
C 2.982984 0.338498 0.000146
C 2.303850 -0.907806 -0.000265
H 2.530119 -3.052957 -0.000809
H 4.991239 -2.984275 -0.000472
H 6.182423 -0.810233 0.000286
H 4.909835 1.301533 0.000688
C -2.207919 -1.753520 0.000380
F -1.721861 -2.365784 -1.076666
F -1.720566 -2.365227 1.077144
F -3.533163 -1.906874 0.001196

NpthIBA_D_CF3.log
Energy (E) = -921.535550652 Hartree
Enthalpy (H) = -921.364639 Hartree
Gibbs free energy (G) = -921.424642

Hartree
Charge = 0, Spin = 1
C 0.556802 3.033376 -0.832267
C 1.837513 2.895784 -0.384221
C 2.354701 1.634280 -0.007346
C 1.549369 0.459440 -0.103735
C 0.201062 0.694224 -0.471694
C -0.290323 1.907675 -0.850326
H 4.265757 2.443476 0.546631
H 0.170384 3.993996 -1.140392
H 2.491712 3.755409 -0.312341
C 3.680732 1.535199 0.473918
C 2.153016 -0.800772 0.178129
H -1.321056 2.025678 -1.147966
C 3.436900 -0.837307 0.662259
C 4.203421 0.327652 0.837978
H 3.854907 -1.815396 0.857578
H 5.213415 0.256605 1.216846
I -1.203346 -0.874839 -0.249279
C 1.549356 -2.139638 -0.196462
O 0.517811 -2.077561 -0.991049
O 2.080309 -3.164876 0.175003
C -2.716732 0.555498 0.568031
F -2.215988 1.410518 1.450311
F -3.368992 1.255822 -0.363295
F -3.612534 -0.215926 1.187899

PyIBA_A_CF3.log
Energy (E) = -784.084744906 Hartree
Enthalpy (H) = -783.975541 Hartree
Gibbs free energy (G) = -784.029772
Hartree
Charge = 0, Spin = 1
C -1.324109 2.788941 -0.000137
C -2.675042 2.467125 -0.000009
C -3.054765 1.131591 0.000048
C -2.075377 0.146614 -0.000027
C -0.786714 0.622184 -0.000141
H -0.986423 3.816376 -0.000161
H -3.413261 3.255741 0.000030
H -4.089094 0.813140 0.000126
C -2.390190 -1.334131 -0.000012
O -1.334702 -2.068533 0.000260
O -3.552793 -1.685009 0.000447
I 0.689141 -0.950101 -0.000218
N -0.370998 1.848177 -0.000209
C 2.341046 0.532039 0.000225
F 2.354137 1.296793 -1.077303
F 2.354402 1.295937 1.078352
F 3.452810 -0.211063 -0.000197

PyIBA_B_CF3.log
Energy (E) = -784.075192317 Hartree
Enthalpy (H) = -783.965860 Hartree
Gibbs free energy (G) = -784.020137
Hartree
Charge = 0, Spin = 1
C 2.768849 2.395667 0.000013
C 2.062501 0.120857 -0.000027
C 0.780248 0.615394 0.000087
H 3.546397 3.149255 -0.000027
C 2.340915 -1.372714 -0.000178
O 1.263060 -2.079719 -0.000144
O 3.488663 -1.760929 -0.000146
I -0.691138 -0.922712 0.000138
C 0.514075 1.973598 0.000141
H -0.486141 2.380223 0.000203
C 3.093622 1.047697 -0.000061
H 4.112719 0.686814 -0.000159
N 1.515030 2.848283 0.000099
C -2.370721 0.531273 -0.000138
F -2.391420 1.308267 1.076988
F -2.391024 1.308472 -1.077120
F -3.472957 -0.216294 -0.000394

PyIBA_C_CF3.log
Energy (E) = -784.074861898 Hartree
Enthalpy (H) = -783.965623 Hartree
Gibbs free energy (G) = -784.019780
Hartree
Charge = 0, Spin = 1
C 2.049241 0.122570 -0.000072
C 0.772462 0.620015 -0.000094
C 2.330905 -1.363448 -0.000125
O 1.250253 -2.073506 0.000099
O 3.475224 -1.760160 0.000383
I -0.693065 -0.928747 -0.000125
C 0.503504 1.971233 -0.000074
H -0.486345 2.395847 -0.000086
C 3.082408 1.059703 -0.000018
H 4.100952 0.691282 -0.000007
C 1.619196 2.805448 -0.000034
H 1.474837 3.878997 -0.000020
N 2.876605 2.370472 -0.000009
C -2.368334 0.534910 0.000104
F -2.385488 1.315101 1.076829
F -2.386286 1.314577 -1.076987
F -3.476373 -0.204025 0.000695

PyIBA_D_CF3.log
Energy (E) = -784.066754713 Hartree
Enthalpy (H) = -783.957657 Hartree
Gibbs free energy (G) = -784.012068
Hartree
Charge = 0, Spin = 1
C -1.570228 2.828075 0.000195
C -2.853224 2.289802 -0.000183
C -2.071688 0.142647 -0.000085
C -0.772942 0.617407 0.000179
H -1.421400 3.898002 0.000345
H -3.718378 2.941867 -0.000427
C -2.343407 -1.356396 -0.000269
O -1.244741 -2.052653 0.000213
O -3.471217 -1.779716 -0.000307
I 0.676357 -0.925851 0.000203
C -0.484101 1.967177 0.000357
H 0.520975 2.357023 0.000632
N -3.100130 0.986903 -0.000322
C 2.371923 0.527180 -0.000185
F 2.405372 1.310274 -1.076953
F 2.406608 1.309537 1.077091
F 3.470516 -0.227756 -0.001067

PyraIBA_CF3.log
Energy (E) = -800.098969080 Hartree
Enthalpy (H) = -800.002086 Hartree
Gibbs free energy (G) = -800.056376
Hartree
Charge = 0, Spin = 1
C -2.662611 2.393739 -0.000012
C -1.324829 2.775851 0.000006
N -0.362924 1.850912 0.000006
C -0.773171 0.621327 -0.000026
C -2.088472 0.188841 -0.000020
N -3.038061 1.119327 -0.000014
H -3.447366 3.139364 -0.000013
H -1.022871 3.813886 0.000014
C -2.418078 -1.296936 0.000123
I 0.672612 -0.952835 -0.000054
O -3.567271 -1.664637 -0.000018
O -1.346210 -2.021861 0.000038
C 2.341264 0.519372 0.000025
F 2.360231 1.284294 -1.078360
F 2.359143 1.285399 1.077647
F 3.446393 -0.232565 0.000957

PyridaIBA_A_CF3.log
Energy (E) = -800.059687525 Hartree
Enthalpy (H) = -799.963288 Hartree
Gibbs free energy (G) = -800.017614
Hartree
Charge = 0, Spin = 1
N 2.861746 2.319039 0.000070
C 1.617842 2.783174 -0.000030
C 0.488322 1.967312 -0.000077
C 0.755691 0.624970 -0.000017
C 2.059611 0.168376 0.000072
N 3.080950 1.024162 0.000118
H -0.498393 2.400708 -0.000157
H 1.517904 3.860064 -0.000072
C 2.357372 -1.326991 0.000116
I -0.678969 -0.935873 -0.000041
O 1.263663 -2.030720 -0.000019
O 3.488818 -1.735155 0.000194
C -2.365275 0.525153 -0.000028
F -2.382269 1.307312 1.076880
F -2.382364 1.307260 -1.076971
F -3.472507 -0.213115 0.000032

PyridaIBA_B_CF3.log
Energy (E) = -800.069023836 Hartree
Enthalpy (H) = -799.972361 Hartree
Gibbs free energy (G) = -800.026577
Hartree
Charge = 0, Spin = 1
N -2.797162 2.404700 -0.000012
C -3.047286 1.104226 0.000025
C -2.041561 0.136701 0.000013
C -0.767738 0.615439 -0.000058
C -0.530673 1.979820 -0.000084
N -1.550692 2.833108 -0.000055
H -4.081930 0.787674 0.000091
I 0.696725 -0.936429 -0.000099
H 0.449878 2.429101 -0.000106
C -2.350079 -1.350328 0.000133
O -1.280810 -2.068488 0.000146
O -3.504709 -1.714020 0.000081
C 2.359089 0.534192 0.000094
F 2.357023 1.310490 -1.076777
F 2.356717 1.310686 1.076822
F 3.474511 -0.189058 0.000308

PyridaIBA_C_CF3.log
Energy (E) = -800.076500557 Hartree
Enthalpy (H) = -799.979977 Hartree
Gibbs free energy (G) = -800.034172
Hartree
Charge = 0, Spin = 1
N -0.349460 1.841505 -0.000347
C -0.782743 0.620606 -0.000135
C -2.086199 0.171043 0.000094
C -2.537396 2.496975 -0.000008
N -1.249855 2.821253 -0.000297
H -3.214524 3.340203 0.000028
C -2.428097 -1.308396 0.000055
O -3.597913 -1.630441 0.000533
O -1.381260 -2.049220 -0.000324
C -3.015713 1.185639 0.000186
H -4.069573 0.941623 0.000372
I 0.675881 -0.946907 -0.000155

C 2.345234 0.515079 0.000154
F 2.365517 1.272431 -1.077779
F 2.365163 1.271756 1.078626
F 3.438262 -0.253458 0.000107

PyrmIBA_CF3.log
Energy (E) = -800.114525050 Hartree
Enthalpy (H) = -800.017273 Hartree
Gibbs free energy (G) = -800.071430 Hartree
Charge = 0, Spin = 1
C 1.371820 2.763308 -0.000155
N 0.379761 1.861465 -0.000167
C 0.778560 0.629029 -0.000135
C 2.066791 0.166803 -0.000052
C 3.010368 1.190598 -0.000066
N 2.665564 2.476884 -0.000122
H 1.081649 3.805164 -0.000235
H 4.062139 0.930418 0.000026
C 2.406357 -1.307111 0.000121
O 3.572793 -1.640293 -0.000056
O 1.356409 -2.049429 -0.000056
I -0.685904 -0.955151 -0.000163
C -2.340547 0.524452 0.000295
F -2.345752 1.287934 1.077924
F -2.347122 1.287133 -1.077884
F -3.451778 -0.215705 0.001260

PyrroleIBA_A_CF3.log
Energy (E) = -746.008879535 Hartree
Enthalpy (H) = -745.905284 Hartree
Gibbs free energy (G) = -745.958156 Hartree
Charge = 0, Spin = 1
C -1.801576 2.684666 -0.000161
C -0.520265 2.176006 0.000434
C -0.698235 0.777202 -0.000020
C -2.023999 0.471153 -0.000432
H -2.127067 3.710030 -0.000303
H 0.392222 2.743860 0.000864
C -2.583720 -0.904238 -0.000868
I 0.510970 -0.906648 0.000294
O -3.786908 -1.083031 0.000259
O -1.635838 -1.786015 0.000456
N -2.693322 1.649786 -0.000598
H -3.702173 1.700318 -0.001072
C 2.299449 0.397761 -0.000218
F 2.344067 1.176668 -1.075219
F 2.344631 1.176693 1.074717
F 3.373622 -0.388171 -0.000497

PyrroleIBA_B_CF3.log
Energy (E) = -746.002079165 Hartree
Enthalpy (H) = -745.898476 Hartree
Gibbs free energy (G) = -745.951315 Hartree
Charge = 0, Spin = 1
C -2.698401 1.725927 0.000250
C -2.077000 0.504610 0.000102
C -0.700730 0.775955 0.000192
H -3.747276 1.962743 0.000222
I 0.485218 -0.921922 -0.000047
C -0.477588 2.122793 0.000473
H 0.420482 2.711682 0.000622
C -2.596215 -0.892861 -0.000090
O -1.621585 -1.760918 -0.000225
O -3.783138 -1.139587 -0.000341
N -1.727255 2.687417 0.000463
H -1.899079 3.680302 0.000606
C 2.287437 0.377473 -0.000089
F 2.342156 1.161905 -1.074952
F 2.342484 1.161523 1.075040
F 3.361235 -0.410554 -0.000392

PyrroleIBA_C_CF3.log
Energy (E) = -745.993452283 Hartree
Enthalpy (H) = -745.890232 Hartree
Gibbs free energy (G) = -745.943400 Hartree
Charge = 0, Spin = 1
C -0.716023 0.758849 0.000031
C -2.058168 0.558293 -0.000160
C -1.620310 2.761233 0.000038
H -1.633038 3.837078 0.000102
C -2.602236 -0.826492 -0.000333
O -3.787337 -1.067541 -0.000297
O -1.639023 -1.720959 -0.000084
C -2.643650 1.852451 -0.000146
H -3.698460 2.064704 -0.000269
I 0.452033 -0.951728 0.000091
N -0.418658 2.081405 0.000152
H 0.497356 2.500405 0.000295
C 2.258680 0.356487 0.000022
F 2.300759 1.149310 -1.074799
F 2.300313 1.150022 1.074333
F 3.344274 -0.409039 0.000500

PyrroleINAcBA_B_CF3.log
Energy (E) = -898.537756965 Hartree
Enthalpy (H) = -898.393670 Hartree
Gibbs free energy (G) = -898.454799 Hartree
Charge = 0, Spin = 1
C 2.497602 0.881281 -0.000221
C 1.210529 1.322941 -0.000226
C 0.403938 0.164009 0.000058
H 3.411483 1.447433 -0.000436
I -1.631587 0.559974 0.000188
C 1.168200 -0.955909 0.000483
H 0.957094 -2.009058 0.000729
C 0.614332 2.692645 -0.000093
O -0.685973 2.639240 0.000644
O 1.306307 3.688094 -0.000504
N 2.476050 -0.502042 0.000196
C 3.584740 -1.384799 0.000296
C 4.934130 -0.731020 0.000279
H 5.054013 -0.101266 -0.880780
H 5.053791 -0.100698 0.880967
H 5.687498 -1.510970 0.000639
O 3.398834 -2.572311 0.001023
C -2.026096 -1.624170 -0.000582
F -1.521006 -2.220035 -1.075748
F -1.521935 -2.220854 1.074559
F -3.345919 -1.794647 -0.001221

PyrroleINMeBA_B_CF3.log
Energy (E) = -785.270971825 Hartree
Enthalpy (H) = -785.138011 Hartree
Gibbs free energy (G) = -785.195444 Hartree
Charge = 0, Spin = 1
C -2.916961 0.646374 -0.013245
C -1.958114 -0.335458 0.001688
C -0.731165 0.338855 0.005449
H -3.989911 0.560350 -0.021120
I 0.913301 -0.920154 0.002199
C -0.931078 1.691004 -0.010413
H -0.256694 2.528121 -0.014832
C -2.030977 -1.823378 0.000937
O -0.839238 -2.356991 0.007154
O -3.087246 -2.418908 -0.004351
N -2.291409 1.859693 -0.023827
C -2.960318 3.145414 0.024713
H -2.407545 3.869216 -0.569375
H -3.957968 3.041051 -0.393442
H -3.039675 3.508286 1.048393
C 2.237471 0.864714 -0.001706
F 2.054711 1.625418 -1.079750
F 2.050169 1.633008 1.070099
F 3.500165 0.439944 0.002282

PyrroleINMsBA_B_CF3.log
Energy (E) = -1333.69041276 Hartree
Enthalpy (H) = -1333.544297 Hartree
Gibbs free energy (G) = -1333.609139 Hartree
Charge = 0, Spin = 1
C -1.877762 1.430888 -0.146438
C -0.529990 1.621188 -0.061917
C 0.048783 0.335231 -0.049237
H -2.689654 2.134817 -0.204189
I 2.121417 0.346548 0.029704
C -0.906106 -0.628395 -0.124886
H -0.887544 -1.702107 -0.166137
C 0.312020 2.855348 0.003356
O 1.578405 2.556813 0.059929
O -0.178570 3.962541 0.005846
N -2.099411 0.068800 -0.191931
C -3.960300 -0.665136 1.596684
H -3.196604 -1.253952 2.097045
H -3.970205 0.363374 1.947190
H -4.939983 -1.121995 1.716031
S -3.622225 -0.661789 -0.132092
O -3.417925 -2.007364 -0.576746
O -4.519161 0.242738 -0.782027
C 2.099127 -1.873182 -0.010059
F 1.437919 -2.380608 1.027514
F 1.543919 -2.348196 -1.119805
F 3.360800 -2.290490 0.046921

PyrroleNAcIBA_A_CF3.log
Energy (E) = -898.526349735 Hartree
Enthalpy (H) = -898.382605 Hartree
Gibbs free energy (G) = -898.444111 Hartree
Charge = 0, Spin = 1
C -1.776202 1.980516 -0.075228
C -0.418957 1.817816 -0.045481
C -0.235585 0.417531 0.021517
C -1.429845 -0.232887 0.029327
H -2.351699 2.889359 -0.085103
H 0.319807 2.598088 -0.060385
C -1.529423 -1.720389 -0.160695
I 1.410604 -0.840624 0.018129
O -2.555749 -2.272362 -0.479946
O -0.357524 -2.264274 -0.005581
N -2.394819 0.747383 -0.023080
C -3.802265 0.570079 0.211126
C -4.674803 1.625598 -0.408206
H -4.621656 2.545263 0.175791
H -4.365684 1.848940 -1.427296
H -5.698125 1.265645 -0.389413
O -4.197888 -0.338008 0.876098
C 2.747551 0.931399 0.013608
F 2.588686 1.679638 -1.074386
F 2.557513 1.708196 1.075907
F 4.001083 0.482533 0.037010

PyrroleNAcIBA_C_CF3.log
Energy (E) = -898.519902018 Hartree

Enthalpy (H) = -898.375990 Hartree
Gibbs free energy (G) = -898.436249 Hartree
Charge = 0, Spin = 1
C 2.613492 1.774515 0.517584
C 1.535196 2.595845 0.473640
N 0.396462 1.847969 0.144019
C 2.156640 0.454943 0.216163
C 0.825827 0.544919 0.006546
C 2.889169 -0.835409 0.022502
O 4.080741 -0.919310 0.231995
O 2.091948 -1.762132 -0.413255
I -0.149153 -1.285695 -0.248504
C -0.763944 2.384613 -0.447747
C -1.017450 3.841304 -0.200980
H -0.888999 4.091890 0.850471
H -0.321365 4.444441 -0.784091
H -2.029707 4.063343 -0.521185
O -1.471772 1.677800 -1.117958
H 3.633819 2.045241 0.727659
H 1.458500 3.660652 0.603724
C -2.193660 -0.646952 0.400342
F -2.160090 0.374853 1.247495
F -3.056371 -0.402140 -0.563043
F -2.628132 -1.723054 1.065706

PyrroleNMeIBA_A_CF3.log
Energy (E) = -785.278790799 Hartree
Enthalpy (H) = -785.145682 Hartree
Gibbs free energy (G) = -785.202316 Hartree
Charge = 0, Spin = 1
C 1.898830 2.345053 0.000079
C 0.559937 2.010488 0.000127
C 0.561729 0.605889 0.000020
C 1.841282 0.129466 -0.000029
H 2.360327 3.318283 0.000098
H -0.271315 2.691483 0.000180
C 2.192171 -1.314984 -0.000037
I -0.869896 -0.892297 0.000041
O 3.351367 -1.688495 -0.000092
O 1.125367 -2.049744 0.000057
N 2.662323 1.214751 -0.000013
C 4.116521 1.172536 -0.000063
H 4.476875 0.645328 0.878558
H 4.476817 0.645355 -0.878724
H 4.482356 2.196381 -0.000059
C -2.466435 0.643234 -0.000052
F -2.409521 1.421502 1.075119
F -2.409277 1.421585 -1.075147
F -3.636189 0.006633 -0.000207

PyrroleNMeIBA_C_CF3.log
Energy (E) = -785.255972247 Hartree
Enthalpy (H) = -785.123044 Hartree
Gibbs free energy (G) = -785.180966 Hartree
Charge = 0, Spin = 1
C -0.864958 0.687250 -0.001676
C -2.189066 0.358100 0.011243
C -1.969770 2.580147 0.014699
H -2.081281 3.651524 0.015988
C -2.642993 -1.055642 0.005520
O -3.812326 -1.366902 0.018845
O -1.626134 -1.881450 -0.018574
C -2.901143 1.579571 0.021811
H -3.972439 1.678727 0.034159
I 0.410243 -0.981225 -0.012563
N -0.701169 2.038478 0.001615
C 0.503265 2.842189 -0.048700
H 1.037095 2.693807 -0.983818
H 0.204752 3.884441 0.018727
H 1.160922 2.619085 0.786527
C 2.378127 0.094323 0.016810
F 2.630836 0.869354 -1.035117
F 2.620636 0.798489 1.120812
F 3.242210 -0.925709 -0.012081

PyrroleNMsIBA_A_CF3.log
Energy (E) = -1333.67764925 Hartree
Enthalpy (H) = -1333.531583 Hartree
Gibbs free energy (G) = -1333.595787 Hartree
Charge = 0, Spin = 1
C -1.395586 1.899296 0.000105
C -0.031375 1.778203 0.000284
C 0.201547 0.387814 0.000043
C -0.971368 -0.299550 -0.000128
H -1.986233 2.796277 0.000106
H 0.676817 2.586572 0.000422
C -1.047557 -1.794600 -0.000195
I 1.881047 -0.826294 0.000247
O -2.105289 -2.380554 -0.000976
O 0.147063 -2.303110 0.000466
N -1.970133 0.644157 -0.000095
C -4.345385 1.904127 0.000567
H -4.062550 2.430642 0.906883
H -4.062884 2.431161 -0.905551
H -5.417309 1.716484 0.000708
S -3.661377 0.276757 -0.000024
O -3.964463 -0.329980 -1.255922
O -3.964138 -0.330759 1.255578
C 3.162912 0.984191 -0.000231
F 2.962280 1.739225 -1.075852
F 2.962204 1.740208 1.074676
F 4.429418 0.575408 0.000013

PyrroleNMsIBA_C_CF3.log
Energy (E) = -1333.67137847 Hartree
Enthalpy (H) = -1333.525532 Hartree
Gibbs free energy (G) = -1333.588803 Hartree
Charge = 0, Spin = 1
C -0.070314 -0.871504 0.222761
C 0.325100 -2.144513 0.433255
C -1.789305 -1.920545 1.162558
H -2.800779 -2.012837 1.521675
C 1.675921 -2.622025 -0.012556
O 2.024620 -3.767329 0.183357
O 2.315821 -1.683256 -0.632407
C -0.785009 -2.822090 1.033630
H -0.790389 -3.856883 1.330998
I 1.449499 0.452074 -0.340777
N -1.364154 -0.669360 0.671303
C -2.830799 -1.059305 -1.553597
H -1.880837 -1.450336 -1.914559
H -3.449353 -1.843119 -1.128198
H -3.354429 -0.533869 -2.348542
S -2.489967 0.144389 -0.308779
O -3.650765 0.354517 0.497688
O -1.767249 1.223529 -0.916830
C 0.527445 2.334453 0.452862
F 0.066022 3.142839 -0.477138
F -0.394508 2.109877 1.369635
F 1.566623 2.939051 1.035636

QuinoIBA_A_CF3.log
Energy (E) = -937.580165336 Hartree
Enthalpy (H) = -937.421333 Hartree
Gibbs free energy (G) = -937.482106 Hartree
Charge = 0, Spin = 1
C -3.052844 1.198040 0.000604
C -1.657563 1.432052 0.000280
C -0.818939 0.367094 0.000070
H -3.685153 2.075493 0.000758
C -1.137681 2.861142 -0.000119
O 0.142912 2.919750 -0.000409
O -1.938310 3.775462 -0.000168
I 1.256303 0.894080 -0.000214
C -3.542948 -0.074190 0.000632
H -4.609743 -0.258617 0.000898
C -0.812953 -3.211413 -0.000759
C -2.185225 -3.538123 -0.000434
C -3.101691 -2.523566 0.000064
C -2.661707 -1.182561 0.000241
C -1.258941 -0.970979 -0.000063
H -0.065906 -3.997206 -0.001160
H -2.491039 -4.574178 -0.000594
H -4.165183 -2.727007 0.000314
N -0.366988 -1.979915 -0.000562
C 2.360771 -1.052919 0.000373
F 2.224763 -1.797610 1.080299
F 2.225673 -1.797748 -1.079587
F 3.614386 -0.564029 0.000881

QuinoIBA_B_CF3.log
Energy (E) = -937.566707020 Hartree
Enthalpy (H) = -937.408264 Hartree
Gibbs free energy (G) = -937.469401 Hartree
Charge = 0, Spin = 1
C -1.241800 0.610195 0.013559
C -0.173465 -0.234196 -0.021585
C -1.001711 2.117762 0.097045
O 0.233405 2.428479 -0.176344
O -1.868199 2.894413 0.407935
I 1.697059 0.782803 -0.086299
C -1.476463 -2.206640 0.001255
C -0.236709 -1.635002 -0.024696
H 0.643846 -2.253978 -0.050532
H -1.577285 -3.284547 0.003522
C -2.641216 -1.409323 0.005721
C -3.929710 -1.989425 0.002790
C -5.025593 -1.179410 -0.026252
C -4.821433 0.218550 -0.068791
C -2.543095 0.007145 -0.004312
H -4.021090 -3.068423 0.018018
H -6.029019 -1.580470 -0.029367
H -5.677755 0.882903 -0.118652
N -3.645285 0.788713 -0.059004
C 2.897288 -1.097649 0.075466
F 2.662216 -1.802580 1.180333
F 2.785663 -1.920262 -0.967438
F 4.160562 -0.666072 0.121547

QuinoIBA_C_CF3.log
Energy (E) = -937.579737490 Hartree
Enthalpy (H) = -937.421054 Hartree
Gibbs free energy (G) = -937.481449 Hartree
Charge = 0, Spin = 1
C -2.254028 1.507804 0.000446
C -0.884734 1.491182 0.000192
C -0.236970 0.250599 -0.000302
C -0.881120 -0.946830 -0.000664
H -2.768536 2.459178 0.000807
H -0.375476 -1.900094 -0.001177
C -0.095436 2.783772 0.000141
O 1.185605 2.594120 -0.000812

O -0.673987 3.847493 0.000339
I 1.881339 0.461568 -0.000329
C -3.060067 -2.121947 -0.000773
C -4.420523 -2.031984 -0.000465
C -5.020498 -0.748805 0.000187
C -2.990576 0.304041 0.000175
C -2.296500 -0.932135 -0.000430
H -2.555456 -3.079988 -0.001248
H -5.044081 -2.914499 -0.000694
H -6.102089 -0.667306 0.000462
N -4.348902 0.372300 0.000506
C 2.214155 -1.741264 0.000636
F 1.731237 -2.357208 1.077358
F 1.733222 -2.358085 -1.076497
F 3.540187 -1.883154 0.001882

QuinoIBA_D_CF3.log
Energy (E) = -937.582074990 Hartree
Enthalpy (H) = -937.423311 Hartree
Gibbs free energy (G) = -937.483655 Hartree
Charge = 0, Spin = 1
C 2.246255 1.526269 -0.000174
C 0.877337 1.504955 -0.000108
C 0.240993 0.258761 0.000166
C 0.889766 -0.935070 0.000366
H 2.734467 2.493459 -0.000376
H 0.414034 -1.902521 0.000601
C 0.085583 2.795139 -0.000261
O -1.192434 2.603934 0.000263
O 0.667857 3.858878 -0.000053
I -1.881127 0.453646 0.000146
C 4.263049 -2.091026 0.000469
C 5.039888 -0.907695 0.000162
C 2.988460 0.328073 0.000015
C 2.307610 -0.914504 0.000306
H 4.760091 -3.054980 0.000652
H 6.118212 -0.976601 0.000113
N 2.954608 -2.108194 0.000539
C 4.402618 0.298218 -0.000067
H 4.951624 1.231161 -0.000306
C -2.200450 -1.748080 -0.000433
F -1.714253 -2.356219 -1.077183
F -1.716184 -2.356608 1.076979
F -3.526343 -1.894853 -0.001631

QuinoIBA_E_CF3.log
Energy (E) = -937.580237457 Hartree
Enthalpy (H) = -937.421407 Hartree
Gibbs free energy (G) = -937.482262 Hartree
Charge = 0, Spin = 1
C -1.242194 0.624468 0.000168
C -0.170834 -0.219302 0.000420
C -1.009104 2.130034 0.000242
O 0.241114 2.451265 0.000805
O -1.933205 2.917809 -0.000181
I 1.709576 0.779755 0.000192
C -1.481741 -2.187813 0.000727
C -0.240818 -1.619325 0.000764
H 0.637360 -2.242347 0.001164
H -1.610907 -3.260827 0.001025
C -2.648438 -1.389709 0.000310
C -4.931784 -1.333114 -0.000169
C -4.939691 0.077267 -0.000448
C -2.542441 0.026050 0.000070
H -5.869237 -1.878941 -0.000260
H -5.881985 0.606779 -0.000756
C -3.756155 0.759553 -0.000324
H -3.715542 1.836246 -0.000523
N -3.836368 -2.048034 0.000203
C 2.904160 -1.103086 -0.000648
F 2.726669 -1.862532 1.076660
F 2.724984 -1.862432 -1.077729
F 4.168162 -0.674413 -0.001589

QuinoIBA_F_CF3.log
Energy (E) = -937.570509248 Hartree
Enthalpy (H) = -937.411725 Hartree
Gibbs free energy (G) = -937.471669 Hartree
Charge = 0, Spin = 1
C -2.209488 2.224919 0.463563
C -0.872777 1.883730 0.158139
C -0.569547 0.579365 -0.063209
H -2.410419 3.271725 0.646298
C 0.152595 2.988835 -0.026562
O 1.270656 2.558469 -0.505967
O -0.153952 4.134437 0.225382
I 1.564737 0.327766 -0.260646
C -3.171140 1.261322 0.518350
H -4.198652 1.485379 0.767196
C -2.371812 -2.632665 -0.673300
C -3.639024 -2.199423 -0.227862
C -2.865217 -0.081655 0.188933
C -1.529592 -0.449119 -0.149604
H -2.245035 -3.636898 -1.050569
H -4.475434 -2.889430 -0.226238
C -1.324922 -1.758948 -0.631308
H -0.358776 -2.054385 -1.008192
N -3.883156 -0.977232 0.168119
C 1.719907 -1.804493 0.419529
F 0.830283 -2.130532 1.346064
F 1.688739 -2.724048 -0.544917
F 2.935624 -1.847847 0.964807

QuinoIBA_G_CF3.log
Energy (E) = -937.570691194 Hartree
Enthalpy (H) = -937.412163 Hartree
Gibbs free energy (G) = -937.472809 Hartree
Charge = 0, Spin = 1
C -5.061016 -0.794010 -0.000041
C -4.334344 0.363438 0.000115
C -2.920156 0.313424 -0.000059
C -2.275618 -0.949799 -0.000350
C -3.050956 -2.132209 -0.000528
C -4.415329 -2.052763 -0.000373
H -6.141617 -0.753602 0.000085
H -4.798984 1.339210 0.000356
C -0.863424 -0.969117 -0.000417
H -2.547787 -3.090857 -0.000757
H -5.009954 -2.955839 -0.000492
C -0.227232 0.231227 -0.000234
C -0.925914 1.455644 -0.000088
H -0.354012 -1.921068 -0.000613
N -2.232389 1.480566 0.000046
C -0.152840 2.769514 0.000178
O -0.730279 3.826912 -0.000101
O 1.134831 2.583125 -0.000417
I 1.873813 0.481159 -0.000101
C 2.239581 -1.721829 0.000495
F 1.762853 -2.344641 1.077031
F 1.765058 -2.344975 -1.076842
F 3.566984 -1.848681 0.001859

QuinoIBA_H_CF3.log
Energy (E) = -937.576524566 Hartree
Enthalpy (H) = -937.417675 Hartree
Gibbs free energy (G) = -937.478654 Hartree
Charge = 0, Spin = 1
C -4.984871 -1.331862 0.000078
C -3.823445 -2.050323 0.000021
C -2.576348 -1.385942 -0.000014
C -2.528594 0.036953 -0.000002
C -3.751692 0.753567 0.000039
C -4.942355 0.078263 0.000087
H -5.939524 -1.840117 0.000110
H -3.809587 -3.131008 0.000002
C -1.238768 0.647936 -0.000033
H -3.717706 1.830051 0.000037
H -5.868016 0.637578 0.000128
C -0.171364 -0.197810 -0.000006
C -0.288578 -1.598674 -0.000102
N -1.463280 -2.165275 -0.000105
I 1.720130 0.774007 0.000119
H 0.572304 -2.250681 -0.000206
C -0.997834 2.158113 -0.000267
O 0.253982 2.465408 -0.000278
O -1.921345 2.944259 -0.000010
C 2.882774 -1.125042 -0.000037
F 2.684317 -1.876679 1.077442
F 2.683616 -1.877040 -1.077139
F 4.154730 -0.724019 -0.000517

QuinoIBA_I_CF3.log
Energy (E) = -937.568447602 Hartree
Enthalpy (H) = -937.409513 Hartree
Gibbs free energy (G) = -937.469404 Hartree
Charge = 0, Spin = 1
C 0.587608 3.036299 -0.785657
C 1.872108 2.888309 -0.353665
C 2.377121 1.617547 0.010345
C 1.555285 0.455606 -0.088214
C 0.208827 0.697711 -0.448854
C -0.272952 1.919500 -0.811401
H 0.206116 4.002616 -1.082264
H 2.553641 3.723373 -0.275322
C 2.171343 -0.799845 0.177986
H -1.302570 2.052145 -1.106242
C 4.162329 0.412622 0.778787
N 3.660901 1.573885 0.446343
H 5.183837 0.406966 1.142017
C 1.568822 -2.149425 -0.173624
O 2.111495 -3.159845 0.219013
O 0.538677 -2.094924 -0.962483
C 3.459295 -0.799258 0.637346
H 3.930677 -1.751389 0.835995
I -1.205389 -0.871448 -0.250557
C -2.733779 0.548946 0.544876
F -2.246646 1.397603 1.439035
F -3.364635 1.251963 -0.396913
F -3.638698 -0.226685 1.142452

QuinoIBA_J_CF3.log
Energy (E) = -937.566099424 Hartree
Enthalpy (H) = -937.407287 Hartree
Gibbs free energy (G) = -937.467077 Hartree
Charge = 0, Spin = 1
C -4.215695 0.372494 0.792507
C -3.670966 1.572940 0.438815
C -2.337983 1.647977 -0.026631
C -1.551786 0.456601 -0.109287
C -3.465995 -0.807701 0.634068
H -5.232572 0.317371 1.155377
H -4.220164 2.502098 0.495631
C -0.200253 0.689828 -0.446875

H -3.902723 -1.777502 0.829696
C -0.642188 2.997485 -0.769566
N -1.874961 2.878844 -0.364305
H -0.301902 3.984025 -1.062232
C 0.266358 1.915495 -0.802820
H 1.291996 2.091003 -1.085970
I 1.209610 -0.880237 -0.235019
C -2.174625 -0.792685 0.172344
C -1.567737 -2.135225 -0.172890
O -2.105087 -3.155915 0.198416
O -0.518541 -2.080900 -0.947309
C 2.734858 0.566022 0.533021
F 3.351853 1.267969 -0.419808
F 2.254620 1.418675 1.428538
F 3.651956 -0.200110 1.125567

QuinoIBA_K_CF3.log
Energy (E) = -937.568628342 Hartree
Enthalpy (H) = -937.409882 Hartree
Gibbs free energy (G) = -937.469993 Hartree
Charge = 0, Spin = 1
C -3.657081 -2.294316 -0.228289
C -3.900652 -1.006597 0.160692
C -2.862791 -0.048888 0.188376
C -1.535685 -0.462388 -0.142279
C -2.361325 -2.678638 -0.628746
H -4.462142 -3.015349 -0.256102
H -4.887902 -0.664635 0.437348
C -0.576969 0.565639 -0.063681
H -2.184828 -3.683927 -0.984723
C -2.261901 2.144219 0.438077
N -3.190425 1.232996 0.498848
H -2.516098 3.179924 0.631720
C -0.902464 1.860769 0.142545
I 1.567639 0.351407 -0.248680
C -1.326857 -1.785329 -0.584319
H -0.353056 -2.081609 -0.938490
C 0.090008 2.993762 -0.023877
O 1.228695 2.587296 -0.471913
O -0.256155 4.130517 0.216176
C 1.775844 -1.786670 0.393004
F 0.922960 -2.138311 1.342207
F 1.733568 -2.691436 -0.582117
F 3.009144 -1.808232 0.899376

QuinoIBA_L_CF3.log
Energy (E) = -937.589749046 Hartree
Enthalpy (H) = -937.431149 Hartree
Gibbs free energy (G) = -937.491579 Hartree
Charge = 0, Spin = 1
C 4.241210 -2.078240 0.000104
C 2.872345 -2.062333 0.000095
C 2.189642 -0.830008 0.000040
C 2.920352 0.386757 -0.000004
C 4.977903 -0.873102 0.000059
H 4.768502 -3.022339 0.000146
H 2.286317 -2.970396 0.000129
H 6.058389 -0.910230 0.000067
C 0.262276 0.323351 -0.000021
N 0.821320 -0.820374 0.000030
C 0.833490 1.597518 -0.000071
C 4.333482 0.333348 0.000007
H 4.888683 1.262377 -0.000027
C -0.003433 2.856483 -0.000150
O 0.555898 3.934488 -0.000083
O -1.268561 2.619004 -0.000142
C 2.202550 1.603615 -0.000057
H 2.710173 2.561317 -0.000092
I -1.902718 0.398749 -0.000021
C -2.020783 -1.817356 0.000061
F -1.488046 -2.368112 -1.077731
F -1.488133 -2.368020 1.077943
F -3.331617 -2.083863 0.000019

ThiopheneIBA_A_CF3.log
Energy (E) = -1088.78417978 Hartree
Enthalpy (H) = -1088.696367 Hartree
Gibbs free energy (G) = -1088.750079 Hartree
Charge = 0, Spin = 1
C -1.795972 2.554099 0.000163
C -0.546374 2.001738 0.000178
C -0.667113 0.594077 0.000086
C -1.928573 0.106104 -0.000070
H -2.034683 3.604633 0.000220
H 0.368596 2.569303 0.000274
C -2.263133 -1.354266 -0.000225
I 0.780850 -0.922454 0.000228
O -3.417057 -1.724602 -0.000058
O -1.189369 -2.078391 0.000162
S -3.041547 1.387691 -0.000017
C 2.401461 0.592011 -0.000315
F 2.368374 1.371766 -1.076138
F 2.369307 1.371621 1.075650
F 3.550699 -0.079561 -0.000850

ThiopheneIBA_B_CF3.log
Energy (E) = -1088.78256320 Hartree
Enthalpy (H) = -1088.694930 Hartree
Gibbs free energy (G) = -1088.748628 Hartree
Charge = 0, Spin = 1
C 2.945351 0.995408 -0.000203
C 2.024498 -0.007223 -0.000137
C 0.720264 0.523112 0.000120
H 4.016080 0.876864 -0.000406
I -0.761406 -0.958216 0.000218
C 0.626002 1.874686 0.000387
H -0.237422 2.515037 0.000623
C 2.272712 -1.487708 -0.000125
O 1.162818 -2.163631 0.000163
O 3.400092 -1.930848 -0.000589
S 2.204868 2.526709 0.000140
C -2.339326 0.605884 -0.000253
F -2.284207 1.387052 1.075106
F -2.284440 1.386075 -1.076325
F -3.509384 -0.029010 0.000183

ThiopheneIBA_C_CF3.log
Energy (E) = -1088.77625760 Hartree
Enthalpy (H) = -1088.688487 Hartree
Gibbs free energy (G) = -1088.742535 Hartree
Charge = 0, Spin = 1
C -0.747240 0.589343 0.000062
C -2.057905 0.257996 -0.000195
C -2.143236 2.562567 0.000393
H -2.490277 3.582579 0.000612
C -2.487648 -1.180242 -0.000269
O -3.662267 -1.476324 -0.000854
O -1.472736 -1.992847 0.000319
C -2.877285 1.419049 -0.000171
H -3.954981 1.368628 -0.000404
I 0.569868 -1.025926 0.000318
S -0.452267 2.263338 0.000235
C 2.306139 0.362621 -0.000307
F 2.335034 1.140306 -1.076197
F 2.336169 1.140294 1.075551
F 3.395641 -0.403397 -0.000865

AnthIBA_A_CCTMS.log
Energy (E) = -1222.69615791 Hartree
Enthalpy (H) = -1222.363131 Hartree
Gibbs free energy (G) = -1222.446985 Hartree
Charge = 0, Spin = 1
C 6.896618 2.221783 0.000011
C 5.618005 2.684917 -0.000067
C 4.518603 1.777985 -0.000047
C 4.778435 0.373805 0.000056
C 6.133103 -0.071006 0.000135
C 7.158688 0.822307 0.000114
C 3.196120 2.208882 -0.000125
C 3.721305 -0.536737 0.000077
C 2.394164 -0.108190 0.000003
C 2.136880 1.305582 -0.000103
C 0.795954 1.781304 -0.000190
H 0.627482 2.850945 -0.000269
C -0.258491 0.924614 -0.000182
C 0.031236 -0.453847 -0.000088
C 1.266874 -1.012424 0.000010
H 2.980758 3.271783 -0.000206
H 7.726026 2.916218 -0.000004
H 5.411275 3.748156 -0.000146
H 6.321034 -1.137165 0.000214
H 8.182950 0.474955 0.000176
H 3.927246 -1.595589 0.000165
H -1.278063 1.280956 -0.000251
C 1.395324 -2.532253 0.000127
O 2.479093 -3.082232 0.000414
O 0.254872 -3.135987 -0.000126
I -1.570077 -1.858879 -0.000097
C -3.016895 -0.359925 -0.000024
C -3.940652 0.427083 0.000031
Si -5.306723 1.670376 0.000090
C -4.503673 3.351172 -0.001074
C -6.327709 1.412607 -1.534057
C -6.326173 1.414063 1.535501
H -5.260016 4.138019 -0.001067
H -3.877833 3.485954 0.881950
H -3.878654 3.485163 -0.884800
H -6.761533 0.412430 -1.553029
H -7.144635 2.135396 -1.574319
H -5.722677 1.535227 -2.432765
H -5.720158 1.537393 2.433452
H -7.142995 2.136947 1.576034
H -6.760043 0.413929 1.555802

AnthIBA_B_CCTMS.log
Energy (E) = -1222.69817586 Hartree
Enthalpy (H) = -1222.365186 Hartree
Gibbs free energy (G) = -1222.448624 Hartree
Charge = 0, Spin = 1
C 6.473259 2.214206 -0.000193
C 6.006988 0.937324 -0.000235
C 4.605858 0.671653 -0.000182
C 3.695226 1.776514 -0.000084
C 4.224506 3.100404 -0.000042
C 5.567357 3.312964 -0.000095
C 4.102767 -0.626487 -0.000224
C 2.324153 1.530598 -0.000031
C 1.822676 0.232118 -0.000073
C 2.731754 -0.875508 -0.000172
C 2.210532 -2.198648 -0.000216
H 2.871762 -3.056923 -0.000291
C 0.869053 -2.430873 -0.000165
C 0.012098 -1.312275 -0.000066

H 4.790630 -1.464261 -0.000299
H 7.537808 2.404741 -0.000233
H 6.689372 0.096470 -0.000309
H 3.530929 3.932259 0.000033
H 5.957791 4.321599 -0.000063
H 1.634841 2.367597 0.000044
I -2.041007 -1.870958 0.000001
C 0.417351 -0.026166 -0.000019
H -0.280128 0.801445 0.000056
C 0.312379 -3.837471 -0.000210
O -0.980057 -3.865720 -0.000188
O 1.067499 -4.787592 -0.000336
C -2.647499 0.118932 0.000128
C -3.108561 1.241474 0.000205
Si -3.819635 2.947159 0.000337
C -3.223462 3.817390 1.534166
C -3.223503 3.817586 -1.533398
C -5.673059 2.780167 0.000387
H -3.622637 4.832481 1.575612
H -3.544210 3.290700 2.433317
H -2.135009 3.883004 1.552356
H -3.544275 3.291021 -2.432613
H -3.622654 4.832691 -1.574702
H -2.135048 3.883176 -1.551595
H -6.018306 2.241089 0.882967
H -6.142983 3.765367 0.000479
H -6.018397 2.241208 -0.882229

AnthIBA_C_CCTMS.log
Energy (E) = -1222.68956024 Hartree
Enthalpy (H) = -1222.356504 Hartree
Gibbs free energy (G) = -1222.437628 Hartree
Charge = 0, Spin = 1
C -3.852679 2.596458 -1.165479
C -2.825229 1.708103 -1.244537
C -1.577174 1.981447 -0.613332
C -1.412516 3.215055 0.085899
C -2.509507 4.122325 0.143722
C -3.692002 3.821375 -0.458169
C -0.524556 1.070221 -0.662512
C -0.172363 3.503223 0.651288
C 0.888823 2.605547 0.592458
C 0.687398 1.322570 -0.029807
C 1.818654 0.458430 -0.010555
C 3.059435 0.832202 0.367167
H -0.668650 0.161502 -1.226094
H -4.798934 2.378930 -1.641817
H -2.934792 0.772711 -1.781457
H -2.379463 5.056504 0.675819
H -4.519524 4.515789 -0.407149
H -0.016368 4.464322 1.128265
C 2.184281 2.961580 1.074952
H 2.305637 3.936104 1.530903
C 4.269354 -0.061785 0.148185
O 5.358672 0.314362 0.531646
O 3.983142 -1.143026 -0.489175
C 3.244084 2.130798 0.921166
H 4.250097 2.388149 1.222091
I 1.762047 -1.637421 -0.504519
C -0.268370 -1.883009 -0.142837
C -1.421835 -2.139142 0.131830
Si -3.194430 -2.386079 0.588387
C -3.340151 -3.988365 1.523206
C -3.680700 -0.931013 1.641592
C -4.197305 -2.437399 -0.980528
H -4.376183 -4.163619 1.818689
H -3.016572 -4.831550 0.912252
H -2.729378 -3.971251 2.426180
H -3.548840 0.005094 1.095580
H -4.728289 -1.005055 1.939429
H -3.073081 -0.882421 2.545645
H -3.869724 -3.245859 -1.634618
H -5.253221 -2.593648 -0.752303
H -4.109533 -1.499665 -1.531378

AnthIBA_D_CCTMS.log
Energy (E) = -1222.67914892 Hartree
Enthalpy (H) = -1222.346031 Hartree
Gibbs free energy (G) = -1222.426016 Hartree
Charge = 0, Spin = 1
C -4.109046 1.816198 1.883515
C -3.085430 2.625869 1.509446
C -2.041673 2.140081 0.670851
C -2.053246 0.765829 0.246115
C -3.215602 -0.022958 0.551368
C -4.181174 0.501380 1.362377
C -1.000361 2.979425 0.293099
C -0.913394 0.334464 -0.434822
C 0.098190 1.170761 -0.910832
C 0.035729 2.550250 -0.526366
C 1.045065 3.450797 -0.975743
H 0.980586 4.483707 -0.657459
C 2.043777 3.030441 -1.794664
C 2.082316 1.673485 -2.216114
C 1.155751 0.776068 -1.783708
H -1.007301 4.007369 0.637579
H -4.896800 2.182546 2.526861
H -3.034331 3.657336 1.835162
H -5.049447 -0.118237 1.542149
H 2.798747 3.721775 -2.142076
H 2.853885 1.352039 -2.902840
H 1.203383 -0.243694 -2.135901
C -3.571577 -1.324719 -0.152388
O -2.782978 -1.662313 -1.127303
O -4.578821 -1.919000 0.176196
I -0.621923 -1.770124 -0.521495
C 1.324130 -1.490229 0.167311
C 2.442965 -1.224855 0.551438
Si 4.084380 -0.535232 1.027319
C 5.169996 -0.567354 -0.486012
C 3.745584 1.218957 1.554971
C 4.815076 -1.546845 2.407195
H 6.148343 -0.133711 -0.271423
H 5.321413 -1.585303 -0.846043
H 4.711384 0.011942 -1.289554
H 3.101404 1.244525 2.434501
H 4.670959 1.746293 1.793462
H 3.239981 1.760248 0.751617
H 4.969175 -2.581017 2.097820
H 5.780709 -1.138312 2.710449
H 4.160408 -1.549460 3.279087

AnthIBA_E_CCTMS.log
Energy (E) = -1222.67403566 Hartree
Enthalpy (H) = -1222.340866 Hartree
Gibbs free energy (G) = -1222.424233 Hartree
Charge = 0, Spin = 1
C 5.927092 1.357192 1.612206
C 5.065025 2.128238 0.901918
C 3.892633 1.561778 0.322712
C 3.624594 0.161141 0.451239
C 4.559889 -0.608200 1.210596
C 5.653765 -0.026334 1.776146
C 3.020550 2.356699 -0.408424
C 2.460745 -0.391366 -0.136480
C 1.488922 0.476830 -0.676878
C 1.819959 1.860631 -0.895804
C 0.935404 2.723288 -1.605341
H 1.263227 3.739563 -1.784476
C -0.279443 2.297909 -2.032264
C -0.706994 0.991770 -1.679526
C 0.141726 0.156491 -1.034691
H 3.268906 3.397244 -0.585782
H 6.812470 1.788559 2.059161
H 5.238669 3.188759 0.767509
H 4.380472 -1.661270 1.337683
H 6.329866 -0.632165 2.364510
H -0.947287 2.949081 -2.577256
H -1.726628 0.693235 -1.879731
I -0.717965 -1.609601 -0.240662
C 2.400391 -1.887165 -0.411405
O 3.409801 -2.560564 -0.332504
O 1.272948 -2.356837 -0.845177
C -2.502012 -0.593092 0.142414
C -3.579152 -0.095544 0.398043
Si -5.197308 0.707408 0.772351
C -6.555706 -0.464194 0.274713
C -5.263461 1.069293 2.596855
C -5.274688 2.280804 -0.222580
H -7.533285 -0.022657 0.476883
H -6.503029 -0.696669 -0.789285
H -6.488015 -1.401029 0.828523
H -4.450917 1.732973 2.893748
H -6.207520 1.551568 2.856995
H -5.180439 0.152472 3.181222
H -5.220288 2.070698 -1.291415
H -6.209297 2.810808 -0.030393
H -4.449890 2.946193 0.035065

FuranIBA_A_CCTMS.log
Energy (E) = -913.500501275 Hartree
Enthalpy (H) = -913.296814 Hartree
Gibbs free energy (G) = -913.366204 Hartree
Charge = 0, Spin = 1
C 1.960003 2.924695 -0.000018
C 0.898270 2.075062 -0.000061
C 1.508066 0.796481 -0.000009
C 2.848810 0.942571 0.000048
O 3.135590 2.254797 -0.000009
H 2.023903 3.997874 -0.000024
H -0.147854 2.323113 -0.000105
C 3.818095 -0.196165 0.000038
I 0.857338 -1.158205 -0.000009
O 5.016259 -0.032205 0.000065
O 3.151526 -1.311228 -0.000033
C -1.120974 -0.537380 -0.000077
C -2.294344 -0.231929 -0.000094
Si -4.087408 0.223871 0.000016
C -4.422366 1.221382 1.534805
C -4.422613 1.221147 -1.534871
C -5.061903 -1.361222 0.000200
H -5.473729 1.511463 1.577912
H -4.193001 0.647652 2.433122
H -3.821684 2.131320 1.553013
H -4.193397 0.647275 -2.433136
H -5.473987 1.511213 -1.577845
H -3.821941 2.131087 -1.553325
H -4.837136 -1.960412 0.882911
H -6.132393 -1.148127 0.000257
H -4.837256 -1.960526 -0.882465

FuranIBA_B_CCTMS.log
Energy (E) = -913.500037447 Hartree
Enthalpy (H) = -913.296237 Hartree
Gibbs free energy (G) = -913.365645 Hartree

Charge = 0, Spin = 1
C 3.044354 2.338337 0.000016
C 2.891173 0.992640 -0.000005
C 1.492230 0.800091 0.000040
O 1.834497 2.950719 0.000014
H 3.912317 2.971565 0.000016
I 0.876899 -1.167744 0.000038
C 0.873719 1.997438 0.000020
H -0.152921 2.311498 0.000031
C 3.826098 -0.178432 -0.000051
O 3.159933 -1.295384 -0.000102
O 5.029303 -0.036801 -0.000073
C -1.107308 -0.552650 0.000034
C -2.280135 -0.244438 0.000032
Si -4.072600 0.213491 -0.000014
C -4.407607 1.211160 1.534820
C -4.407212 1.212365 -1.534153
C -5.049894 -1.369996 -0.000783
H -5.459060 1.501035 1.577207
H -4.178548 0.637884 2.433453
H -3.807383 2.121415 1.552840
H -4.177905 0.639796 -2.433172
H -5.458660 1.502252 -1.576590
H -3.807005 2.122648 -1.551302
H -4.826369 -1.969949 0.881702
H -6.119940 -1.154715 -0.000834
H -4.826136 -1.969217 -0.883706

FuranIBA_C_CCTMS.log
Energy (E) = -913.492670305 Hartree
Enthalpy (H) = -913.289092 Hartree
Gibbs free energy (G) = -913.358394 Hartree
Charge = 0, Spin = 1
C -1.555627 0.792356 0.000049
C -2.877785 1.017710 0.000033
O -0.805513 1.887964 -0.000001
C -1.713934 2.919419 0.000067
H -1.288207 3.906004 0.000117
C -3.824589 -0.140177 0.000013
O -5.026478 0.009369 -0.000109
O -3.168434 -1.265991 0.000046
C -2.982217 2.446850 -0.000092
H -3.891256 3.022366 -0.000172
I -0.890040 -1.166375 0.000012
C 1.085673 -0.549683 -0.000013
C 2.262000 -0.258689 -0.000028
Si 4.052098 0.205796 0.000006
C 4.381187 1.204342 -1.535152
C 4.381174 1.204131 1.535298
C 5.038042 -1.372931 -0.000173
H 5.429457 1.505861 -1.575455
H 4.160713 0.626552 -2.433095
H 3.769120 2.106392 -1.555127
H 4.160716 0.626232 2.433175
H 5.429432 1.505682 1.575639
H 3.769075 2.106158 1.555375
H 4.818310 -1.973827 -0.883001
H 6.106845 -1.151336 -0.000187
H 4.818378 -1.974048 0.882522

Indole_NAc_IBA_A_CCTMS.log
Energy (E) = -1199.69338111 Hartree
Enthalpy (H) = -1199.386438 Hartree
Gibbs free energy (G) = -1199.467749 Hartree
Charge = 0, Spin = 1
C -0.678652 1.193180 0.042177
C -1.952382 1.806533 0.097819
C -2.085586 3.194216 0.139130
C -0.929568 3.950412 0.137984
C 0.337527 3.354796 0.092831
C 0.474594 1.984316 0.041675
C -0.965559 -0.206046 -0.017311
H -3.058737 3.652866 0.193275
H -1.008810 5.028033 0.180498
H 1.222774 3.977402 0.097530
H 1.450461 1.529439 0.000695
N -2.935883 0.811245 0.046321
C -2.303490 -0.418889 0.000028
C -2.894477 -1.786045 0.213980
O -1.994502 -2.696076 0.076045
O -4.060623 -1.929106 0.526035
I 0.178244 -1.949613 0.015614
C -4.320426 1.103990 -0.139938
C -5.135678 0.183131 -0.996982
H -5.758264 -0.441478 -0.362735
H -5.771013 0.816710 -1.612412
H -4.527373 -0.469829 -1.614894
O -4.757585 2.129342 0.315429
C 1.992540 -0.959812 -0.021100
C 3.104890 -0.478674 -0.041120
Si 4.716488 0.429086 -0.061308
C 4.276619 2.240299 -0.023976
C 5.684373 -0.064841 1.447689
C 5.621268 -0.016403 -1.623744
H 5.178965 2.854118 -0.030304
H 3.675727 2.515792 -0.892690
H 3.707699 2.486448 0.874563
H 5.894398 -1.134784 1.446863
H 6.637873 0.465363 1.478809
H 5.135932 0.171529 2.359797
H 5.035503 0.247155 -2.504693
H 6.572468 0.516066 -1.677941
H 5.831532 -1.085516 -1.664745

Indole_NAc_IBA_B_CCTMS.log
Energy (E) = -1199.68010899 Hartree
Enthalpy (H) = -1199.373445 Hartree
Gibbs free energy (G) = -1199.454261 Hartree
Charge = 0, Spin = 1
C -3.056371 0.818807 -0.200163
C -2.007473 1.746441 -0.056493
C -2.244409 3.112274 -0.162095
C -3.545791 3.515878 -0.424221
C -4.590048 2.599417 -0.572624
C -4.356683 1.240795 -0.463646
H -1.465730 3.852028 -0.074111
H -3.750294 4.573176 -0.524126
H -5.587927 2.960786 -0.778622
H -5.138487 0.503085 -0.575261
N -0.811902 1.020482 0.220194
C -2.492603 -0.499316 -0.059180
C -1.190348 -0.314805 0.190527
C -3.084158 -1.871288 -0.134800
O -4.268378 -2.029052 -0.358670
O -2.188511 -2.779601 0.088499
I -0.015014 -2.038407 0.075352
C 0.241209 1.463968 1.056841
C 0.710744 2.874989 0.869469
H 0.670534 3.178719 -0.174303
H 0.090605 3.552167 1.457446
H 1.728729 2.933335 1.245948
O 0.729996 0.707852 1.857298
C 1.744019 -0.985228 -0.168573
C 2.803302 -0.420078 -0.331074
Si 4.359438 0.569759 -0.385862
C 5.720645 -0.482782 -1.092090
C 4.036043 2.056936 -1.461406
C 4.716318 1.079681 1.368252
H 6.658123 0.075452 -1.119276
H 5.878348 -1.376518 -0.487874
H 5.486074 -0.798489 -2.108973
H 3.224516 2.664342 -1.057214
H 4.926280 2.685445 -1.522584
H 3.761516 1.758554 -2.473496
H 4.965075 0.212529 1.980763
H 5.550177 1.781990 1.415912
H 3.838165 1.551234 1.813478

Indole_NH_IBA_A_CCTMS.log
Energy (E) = -1047.16519451 Hartree
Enthalpy (H) = -1046.899069 Hartree
Gibbs free energy (G) = -1046.973676 Hartree
Charge = 0, Spin = 1
C 0.929748 1.491274 -0.000134
C 2.093536 2.309356 0.000146
C 2.015076 3.701245 0.000155
C 0.762208 4.275908 -0.000127
C -0.397855 3.484385 -0.000434
C -0.329212 2.107726 -0.000451
C 1.447488 0.159630 -0.000033
H 2.912153 4.305544 0.000363
H 0.670493 5.353247 -0.000124
H -1.366147 3.966877 -0.000662
H -1.229500 1.512889 -0.000701
H 4.177096 1.760107 0.000509
N 3.202407 1.496021 0.000329
C 2.803974 0.198590 0.000169
C 3.700826 -0.993007 0.000118
O 3.014030 -2.082304 -0.000135
O 4.908555 -0.838491 0.000393
I 0.701988 -1.785066 -0.000137
C -1.266124 -1.159808 -0.000182
C -2.433285 -0.834673 -0.000157
Si -4.136402 -0.116261 0.000161
C -4.271731 0.935411 1.530423
C -4.272152 0.935894 -1.529733
C -5.374847 -1.503092 0.000053
H -5.241840 1.433588 1.573056
H -4.157193 0.335315 2.433386
H -3.496807 1.704560 1.538936
H -4.157879 0.336087 -2.432921
H -5.242308 1.434025 -1.571892
H -3.497278 1.705090 -1.538252
H -5.259407 -2.132775 0.882560
H -6.390949 -1.104792 0.000227
H -5.259577 -2.132412 -0.882737

Indole_NH_IBA_B_CCTMS.log
Energy (E) = -1047.15523402 Hartree
Enthalpy (H) = -1046.889428 Hartree
Gibbs free energy (G) = -1046.965234 Hartree
Charge = 0, Spin = 1
C 2.954693 1.036103 -0.000001
C 1.843825 1.908596 -0.000050
C 1.989921 3.292984 -0.000046
C 3.280059 3.787729 0.000020
C 4.396002 2.934708 0.000073
C 4.249382 1.562319 0.000063
H 1.133846 3.954013 -0.000087
H 3.434322 4.858269 0.000031
H 5.387072 3.367313 0.000122
H 5.097305 0.891892 0.000099
H -0.272719 1.447042 -0.000153
N 0.687008 1.135632 -0.000096
C 2.437391 -0.303959 -0.000023

C 1.097337 -0.157974 -0.000058
C 3.032363 -1.664921 -0.000022
O 4.229946 -1.853833 -0.000033
O 2.104646 -2.592384 -0.000024
I -0.019502 -1.893658 0.000023
C -1.759158 -0.722499 0.000003
C -2.786744 -0.075817 -0.000014
Si -4.378468 0.868285 0.000014
C -4.421375 1.918742 1.535763
C -4.418964 1.922814 -1.533011
C -5.758983 -0.378563 -0.002746
H -5.351678 2.487828 1.580724
H -4.358262 1.303415 2.433601
H -3.593778 2.628782 1.554711
H -4.354393 1.309869 -2.432374
H -5.349226 2.491969 -1.577945
H -3.591388 2.632959 -1.548766
H -5.712110 -1.017926 0.879112
H -6.726430 0.126784 -0.002869
H -5.710662 -1.015583 -0.886223

Indole_NMe_IBA_A_CCTMS.log
Energy (E) = -1086.43495225 Hartree
Enthalpy (H) = -1086.139234 Hartree
Gibbs free energy (G) = -1086.217383 Hartree
Charge = 0, Spin = 1
C 0.844852 1.330963 0.000226
C 2.057998 2.067612 -0.000055
C 2.078825 3.462742 -0.000029
C 0.868995 4.123262 0.000314
C -0.342286 3.411749 0.000646
C -0.368976 2.033507 0.000611
C 1.281345 -0.024794 0.000101
H 3.016874 4.001645 -0.000262
H 0.850582 5.204403 0.000344
H -1.275750 3.958865 0.000952
H -1.309097 1.504969 0.000906
N 3.124562 1.202316 -0.000314
C 2.645312 -0.076626 -0.000212
C 3.417517 -1.363709 -0.000233
O 2.618523 -2.373233 0.000135
O 4.635934 -1.380710 -0.000569
I 0.366304 -1.894123 0.000266
C 4.513590 1.623148 -0.000607
H 4.719380 2.221961 0.886646
H 5.137545 0.736096 -0.000689
H 4.719034 2.221880 -0.887996
C -1.548583 -1.112558 0.000158
C -2.690863 -0.707957 -0.000023
Si -4.335236 0.134841 -0.000277
C -4.392238 1.194253 1.529747
C -4.391078 1.195394 -1.529554
C -5.675228 -1.154466 -0.001265
H -5.321773 1.764603 1.572118
H -4.323298 0.587867 2.433142
H -3.561105 1.902421 1.537637
H -4.321538 0.589652 -2.433336
H -5.320548 1.765831 -1.572172
H -3.559896 1.903517 -1.536366
H -5.608062 -1.791299 0.881129
H -6.658453 -0.680772 -0.001466
H -5.607388 -1.790660 -0.884067

Indole_NMe_IBA_B_CCTMS.log
Energy (E) = -1086.41885905 Hartree
Enthalpy (H) = -1086.123259 Hartree
Gibbs free energy (G) = -1086.202858 Hartree
Charge = 0, Spin = 1
C -3.185624 0.766140 0.017389
C -2.218154 1.789212 -0.014999
C -2.579579 3.136584 -0.015459
C -3.929767 3.426964 0.016425
C -4.903939 2.415390 0.048297
C -4.546966 1.083308 0.049496
H -1.847812 3.932122 -0.034394
H -4.243739 4.462059 0.018519
H -5.948998 2.692050 0.072968
H -5.277008 0.286871 0.074095
N -0.951463 1.209517 -0.046050
C -2.476412 -0.474874 0.009719
C -1.163787 -0.142382 -0.028238
C -2.932670 -1.886379 0.023564
O -4.108583 -2.184644 0.055917
O -1.922910 -2.713693 -0.007420
I 0.121240 -1.804136 -0.017650
C 0.284172 1.959476 -0.128874
H 0.871470 1.655208 -0.992581
H 0.028929 3.009139 -0.235728
H 0.890727 1.833137 0.766097
C 1.880688 -0.677256 -0.003307
C 2.978342 -0.160924 0.012890
Si 4.563735 0.784487 0.028517
C 4.073469 2.583785 0.032146
C 5.508055 0.339969 1.567886
C 5.526751 0.352357 -1.502984
H 4.954567 3.227566 0.023780
H 3.473186 2.825858 -0.846727
H 3.489092 2.826936 0.921107
H 5.751992 -0.722608 1.585200
H 6.443034 0.900911 1.617111
H 4.929186 0.567930 2.463258
H 4.961121 0.592617 -2.403614
H 6.464792 0.909511 -1.533567
H 5.766150 -0.711083 -1.528616

Indole_NMs_IBA_A_CCTMS.log
Energy (E) = -1634.83549652 Hartree
Enthalpy (H) = -1634.526721 Hartree
Gibbs free energy (G) = -1634.611640 Hartree
Charge = 0, Spin = 1
C 0.409903 1.042791 -0.177582
C 1.707092 1.565503 -0.361701
C 1.931692 2.934872 -0.484108
C 0.832749 3.772516 -0.426265
C -0.460200 3.267516 -0.248671
C -0.683701 1.911408 -0.125427
C 0.611587 -0.373478 -0.082612
H 2.926954 3.319435 -0.644991
H 0.978952 4.838516 -0.532446
H -1.300557 3.948346 -0.212391
H -1.681717 1.528580 0.008442
N 2.632309 0.503724 -0.378597
C 1.922076 -0.687091 -0.212257
C 2.363727 -2.123138 -0.423972
O 1.431519 -2.921801 -0.005953
O 3.393910 -2.407830 -0.980864
I -0.648415 -2.028037 0.081984
C 3.854411 1.346227 1.857207
H 3.274843 2.263732 1.808899
H 3.319863 0.556937 2.380043
H 4.821235 1.523325 2.322273
S 4.207867 0.777181 0.221580
O 4.886352 -0.470095 0.320710
O 4.729686 1.867116 -0.549188
C -2.389346 -0.896863 0.082624
C -3.468709 -0.344268 0.085024
Si -5.018933 0.662939 0.065060
C -4.464045 2.440288 -0.042156
C -6.017417 0.176038 -1.425946
C -5.951188 0.340451 1.641671
H -5.324752 3.110752 -0.067418
H -3.851381 2.712032 0.819629
H -3.876272 2.612003 -0.945892
H -6.297538 -0.876879 -1.384174
H -6.933766 0.766424 -1.480508
H -5.454138 0.339627 -2.344947
H -5.350098 0.600915 2.513193
H -6.866167 0.934722 1.671530
H -6.229953 -0.710362 1.725048

Indole_NMs_IBA_B_CCTMS.log
Energy (E) = -1634.83948172 Hartree
Enthalpy (H) = -1634.530868 Hartree
Gibbs free energy (G) = -1634.614328 Hartree
Charge = 0, Spin = 1
C -2.842763 0.787644 -0.543257
C -1.712980 1.613711 -0.448802
C -1.764236 2.975336 -0.699495
C -2.998106 3.491083 -1.073225
C -4.131194 2.678156 -1.188176
C -4.070692 1.318901 -0.928348
H -0.883369 3.596181 -0.621069
H -3.080006 4.546271 -1.295153
H -5.068769 3.122836 -1.491945
H -4.930262 0.669791 -1.015560
N -0.593570 0.826800 -0.024206
C -2.411886 -0.557044 -0.229727
C -1.108803 -0.467835 0.068145
C -3.119589 -1.881080 -0.213315
O -4.306968 -1.942258 -0.470868
O -2.317709 -2.832928 0.125296
I -0.052901 -2.273848 0.087573
C -1.069911 1.954860 2.402295
H -1.539306 2.853792 2.017334
H -1.779025 1.130988 2.472915
H -0.599967 2.134005 3.366259
S 0.240396 1.445872 1.330791
O 0.972132 2.590078 0.869783
O 0.902466 0.339684 1.959054
C 1.735128 -1.322324 -0.231244
C 2.747553 -0.698104 -0.456872
Si 4.183433 0.454041 -0.643594
C 5.620927 -0.498687 -1.344309
C 3.624046 1.809225 -1.788199
C 4.548611 1.122116 1.052328
H 6.486547 0.155381 -1.463526
H 5.907828 -1.319504 -0.686388
H 5.377124 -0.916577 -2.321456
H 2.752903 2.312995 -1.366321
H 4.414020 2.550384 -1.922502
H 3.354325 1.416685 -2.768934
H 4.820366 0.322806 1.742222
H 5.369818 1.840388 1.018610
H 3.666149 1.627030 1.448330

NpthIBA_A_CCTMS.log
Energy (E) = -1069.19212048 Hartree
Enthalpy (H) = -1068.908413 Hartree
Gibbs free energy (G) = -1068.983845 Hartree
Charge = 0, Spin = 1
C 3.435828 1.468153 0.791694
C 2.844411 0.258266 0.366520
C 1.560413 0.281995 -0.073535
H 4.455779 1.412838 1.146380
C 3.685290 -1.008596 0.324466

O 3.075043 -1.994520 -0.234707
O 4.820180 -0.978990 0.755449
I 0.818084 -1.723085 -0.346062
C 2.717239 2.625224 0.757364
H 3.147040 3.553885 1.111197
C -1.044320 2.772376 -1.124702
C -0.492335 3.942202 -0.564901
C 0.717715 3.883196 0.067697
C 1.412890 2.659277 0.204783
C 0.806083 1.461326 -0.276589
C -0.413368 1.565752 -0.987156
H -1.966769 2.830731 -1.687528
H -1.008776 4.886269 -0.669993
H 1.183091 4.779634 0.457846
H -0.836351 0.692354 -1.456367
C -1.177110 -1.217482 -0.091800
C -2.346072 -0.977010 0.121906
Si -4.069294 -0.373752 0.391935
C -5.104804 -1.762960 1.067818
C -3.946455 1.048473 1.586156
C -4.704395 0.213065 -1.258211
H -6.129467 -1.426983 1.236243
H -5.135564 -2.604202 0.374864
H -4.706760 -2.121549 2.017378
H -3.279775 1.817437 1.190635
H -4.925665 1.499758 1.755585
H -3.550067 0.723111 2.548218
H -4.731815 -0.598670 -1.985542
H -5.712489 0.620680 -1.165143
H -4.058622 0.999309 -1.654598

NpthIBA_B_CCTMS.log
Energy (E) = -1069.20005832 Hartree
Enthalpy (H) = -1068.916325 Hartree
Gibbs free energy (G) = -1068.993380 Hartree
Charge = 0, Spin = 1
C -2.282194 -0.401913 -0.000189
C -0.961116 -0.065091 -0.000062
C -2.668499 -1.877980 -0.000129
O -1.646937 -2.666782 -0.000021
O -3.829397 -2.236346 -0.000158
I 0.371351 -1.726260 0.000188
C -1.343973 2.260418 -0.000245
C -0.443941 1.233445 -0.000082
H 0.621009 1.412956 0.000027
H -0.997126 3.286203 -0.000267
C -2.735709 2.014299 -0.000391
C -3.642965 3.099381 -0.000564
C -4.991008 2.880214 -0.000713
C -5.481557 1.559199 -0.000694
C -4.631882 0.485034 -0.000525
C -3.226790 0.678034 -0.000366
H -3.241778 4.105353 -0.000576
H -5.680866 3.713228 -0.000845
H -6.549732 1.388352 -0.000815
H -5.009057 -0.523774 -0.000507
C 2.058260 -0.502351 0.000217
C 3.116895 0.091362 0.000249
Si 4.730902 0.991223 0.000352
C 4.802630 2.043639 -1.533381
C 4.802546 2.043459 1.534212
C 6.083374 -0.287345 0.000326
H 5.745014 2.592904 -1.574402
H 4.728460 1.431567 -2.432619
H 3.989152 2.769666 -1.551545
H 4.728325 1.431278 2.433372
H 5.744931 2.592714 1.575350
H 3.989069 2.769486 1.552421
H 6.021248 -0.924368 -0.882325
H 7.062179 0.195769 0.000387
H 6.021184 -0.924456 0.882909

NpthIBA_C_CCTMS.log
Energy (E) = -1069.20297354 Hartree
Enthalpy (H) = -1068.919292 Hartree
Gibbs free energy (G) = -1068.996712 Hartree
Charge = 0, Spin = 1
C 3.496072 0.234858 -0.000099
C 2.542291 -0.747336 -0.000188
C 1.202573 -0.348237 -0.000057
C 0.778422 0.939795 0.000152
H 4.535076 -0.072128 -0.000202
H -0.271544 1.202301 0.000245
C 2.920406 -2.212982 -0.000416
O 1.892097 -2.995545 -0.000437
O 4.090476 -2.535033 -0.000522
I -0.137178 -2.001055 -0.000205
C 1.406755 3.328998 0.000471
C 2.372602 4.296743 0.000563
C 3.740659 3.942456 0.000436
C 4.113051 2.627333 0.000219
C 3.137625 1.600667 0.000120
C 1.763425 1.959880 0.000248
H 0.356876 3.594080 0.000570
H 2.092309 5.341316 0.000735
H 4.492558 4.719634 0.000511
H 5.158376 2.346056 0.000121
C -1.792318 -0.741558 0.000017
C -2.821361 -0.098402 0.000131
Si -4.392801 0.874438 0.000161
C -4.415565 1.927107 1.535241
C -4.413292 1.930678 -1.532498
C -5.802313 -0.340572 -0.002231
H -5.331072 2.520021 1.576949
H -4.370306 1.311040 2.433663
H -3.568869 2.614189 1.554810
H -4.366747 1.316712 -2.432291
H -5.328692 2.523758 -1.574167
H -3.566524 2.617748 -1.549211
H -5.769912 -0.980814 0.879683
H -6.757995 0.186778 -0.002300
H -5.768690 -0.978850 -0.885519

NpthIBA_D_CCTMS.log
Energy (E) = -1069.18743960 Hartree
Enthalpy (H) = -1068.903566 Hartree
Gibbs free energy (G) = -1068.980153 Hartree
Charge = 0, Spin = 1
C 0.550514 2.816101 -1.117844
C 1.761973 3.145180 -0.585949
C 2.621349 2.159253 -0.048050
C 2.256616 0.777084 -0.071145
C 0.949323 0.522573 -0.559218
C 0.123805 1.474454 -1.075116
H 4.094776 3.606979 0.538039
H -0.099876 3.568198 -1.540801
H 2.093519 4.175625 -0.562019
C 3.850671 2.551958 0.530423
C 3.204054 -0.175070 0.409544
H -0.865840 1.217192 -1.424961
C 4.370478 0.263634 0.984591
C 4.698320 1.627361 1.068235
H 5.053663 -0.498275 1.334301
H 5.631427 1.932153 1.521304
I 0.098669 -1.419193 -0.338450
C 3.104450 -1.669999 0.184187
O 2.215096 -2.030121 -0.692181
O 3.878355 -2.417065 0.747246
C -1.787179 -0.577914 -0.047758
C -2.932536 -0.216973 0.127946
Si -4.660840 0.370555 0.402049
C -5.806041 -0.742159 -0.554522
C -5.020941 0.292336 2.226120
C -4.754927 2.123305 -0.222150
H -6.842468 -0.426078 -0.422737
H -5.577817 -0.717572 -1.620437
H -5.723067 -1.775074 -0.215251
H -4.330437 0.921134 2.788822
H -6.036380 0.636964 2.430094
H -4.929085 -0.727724 2.600162
H -4.525454 2.172960 -1.287193
H -5.757533 2.528572 -0.074311
H -4.050322 2.765167 0.307844

PyIBA_A_CCTMS.log
Energy (E) = -931.735445512 Hartree
Enthalpy (H) = -931.513254 Hartree
Gibbs free energy (G) = -931.584754 Hartree
Charge = 0, Spin = 1
C 1.187544 2.916965 0.000009
C 2.559461 3.129603 0.000004
C 3.415000 2.035403 -0.000001
C 2.880769 0.753612 -0.000001
C 1.506315 0.708508 0.000004
H 0.486663 3.740966 0.000012
H 2.945539 4.138608 0.000004
H 4.493146 2.130359 -0.000004
C 3.733959 -0.498224 -0.000008
O 3.032970 -1.573635 -0.000010
O 4.943670 -0.379442 -0.000012
I 0.730483 -1.317656 0.000004
N 0.662052 1.684894 0.000009
C -1.209891 -0.637046 0.000011
C -2.377018 -0.311596 0.000013
Si -4.137649 0.243162 -0.000003
C -4.955215 -0.422127 1.534247
C -4.122964 2.104771 -0.000762
C -4.955593 -0.423362 -1.533513
H -6.000747 -0.111158 1.574771
H -4.926782 -1.511997 1.553181
H -4.458052 -0.055690 2.432788
H -3.610778 2.488888 -0.883352
H -5.141707 2.496534 -0.000795
H -3.610563 2.489613 0.881386
H -4.927262 -1.513249 -1.551527
H -6.001109 -0.112338 -1.574077
H -4.458586 -0.057722 -2.432463

PyIBA_B_CCTMS.log
Energy (E) = -931.729527000 Hartree
Enthalpy (H) = -931.507199 Hartree
Gibbs free energy (G) = -931.577887 Hartree
Charge = 0, Spin = 1
C 2.539444 3.118110 -0.000022
C 2.850862 0.755258 0.000039
C 1.479787 0.675712 -0.000035
H 2.932847 4.127145 -0.000021
C 3.715503 -0.495240 0.000098
O 3.016214 -1.575899 -0.000005
O 4.922825 -0.378433 0.000186
I 0.748742 -1.318184 -0.000040
C 0.668525 1.793682 -0.000104
H -0.411288 1.720505 -0.000170
C 3.398861 2.029219 0.000050
H 4.475361 2.129795 0.000114

N 1.209882 3.009273 -0.000099
C -1.213116 -0.660495 -0.000028
C -2.381438 -0.335847 -0.000006
Si -4.140213 0.240697 0.000055
C -4.960008 -0.412704 1.536703
C -4.081984 2.101039 -0.002242
C -4.961194 -0.416455 -1.534367
H -6.000958 -0.087265 1.579590
H -4.947032 -1.502798 1.556149
H -4.456071 -0.052193 2.433783
H -3.563743 2.475969 -0.885634
H -5.091100 2.516707 -0.002377
H -3.563068 2.478141 0.879830
H -4.948076 -1.506595 -1.551175
H -6.002214 -0.091263 -1.577337
H -4.457926 -0.058063 -2.432674

PyIBA_C_CCTMS.log
Energy (E) = -931.729402411 Hartree
Enthalpy (H) = -931.507197 Hartree
Gibbs free energy (G) = -931.577713 Hartree
Charge = 0, Spin = 1
C -2.834659 0.759759 0.000029
C -1.467575 0.679360 0.000011
C -3.703886 -0.479664 0.000038
O -3.007561 -1.566829 0.000031
O -4.911128 -0.367048 -0.000061
I -0.753734 -1.325953 0.000023
C -0.650018 1.784763 -0.000031
H 0.428591 1.721083 -0.000068
C -3.374384 2.045953 0.000022
H -4.453098 2.144436 0.000011
C -1.308158 3.012512 -0.000025
H -0.726845 3.926714 -0.000070
N -2.632813 3.147233 -0.000009
C 1.212465 -0.666296 0.000042
C 2.379638 -0.336169 0.000058
Si 4.138542 0.237730 0.000027
C 4.079878 2.099323 -0.001168
C 4.959814 -0.413292 1.536491
C 4.959960 -0.415345 -1.535526
H 5.088243 2.516582 -0.001832
H 3.561339 2.473416 -0.884963
H 3.562028 2.474562 0.882543
H 4.947964 -1.503370 1.556383
H 6.000460 -0.086844 1.578806
H 4.455803 -0.053303 2.433739
H 4.456361 -0.055985 -2.433261
H 6.000832 -0.089636 -1.577994
H 4.947389 -1.505434 -1.554240

PyIBA_D_CCTMS.log
Energy (E) = -931.720901683 Hartree
Enthalpy (H) = -931.498816 Hartree
Gibbs free energy (G) = -931.569873 Hartree
Charge = 0, Spin = 1
C -1.429625 3.029336 0.000110
C -2.821592 3.011777 0.000004
C -2.909592 0.728427 -0.000019
C -1.528302 0.686734 0.000081
H -0.892055 3.966496 0.000158
H -3.376829 3.942256 -0.000033
C -3.712081 -0.567877 -0.000067
O -2.940687 -1.612603 -0.000161
O -4.917895 -0.546826 -0.000325
I -0.730208 -1.270492 0.000100
C -0.748689 1.821622 0.000149
H 0.331546 1.771254 0.000226
N -3.544082 1.898831 -0.000060
C 1.222233 -0.535386 0.000260
C 2.393842 -0.218111 0.000357
Si 4.185462 0.241491 -0.000068
C 4.521073 1.240192 -1.534347
C 4.521779 1.240239 1.534024
C 5.163078 -1.341642 -0.000277
H 5.572799 1.529125 -1.576836
H 4.291372 0.667514 -2.433232
H 3.921814 2.151150 -1.551939
H 4.292493 0.667590 2.433034
H 5.573524 1.529174 1.576018
H 3.922527 2.151197 1.551863
H 4.939153 -1.941279 -0.882879
H 6.233132 -1.126259 -0.000532
H 4.939569 -1.941259 0.882444

PyraIBA_CCTMS.log
Energy (E) = -947.750282974 Hartree
Enthalpy (H) = -947.540391 Hartree
Gibbs free energy (G) = -947.611823 Hartree
Charge = 0, Spin = 1
C -2.609615 3.045665 0.000008
C -1.226100 2.907095 0.000047
N -0.674407 1.691435 0.000056
C -1.506123 0.701986 0.000026
C -2.890246 0.783872 -0.000008
N -3.428918 1.999225 -0.000018
H -3.064047 4.028301 -0.000010
H -0.562548 3.760830 0.000068
C -3.745877 -0.476278 -0.000030
I -0.737064 -1.304643 0.000024
O -4.950588 -0.390385 -0.000071
O -3.014402 -1.540372 -0.000049
C 1.207137 -0.619863 0.000057
C 2.375613 -0.298242 0.000063
Si 4.143294 0.238647 -0.000010
C 4.148562 2.100184 -0.003704
C 4.950422 -0.433839 1.536185
C 4.952145 -0.439888 -1.532634
H 5.171888 2.479749 -0.003976
H 3.642015 2.489322 -0.887346
H 3.641171 2.492858 0.877884
H 4.909203 -1.523242 1.556885
H 5.999504 -0.135238 1.577325
H 4.456881 -0.059983 2.433652
H 4.459580 -0.069600 -2.432110
H 6.001259 -0.141360 -1.573785
H 4.911047 -1.529362 -1.549072

PyridaIBA_A_CCTMS.log
Energy (E) = -947.715021458 Hartree
Enthalpy (H) = -947.505646 Hartree
Gibbs free energy (G) = -947.576253 Hartree
Charge = 0, Spin = 1
N -2.634393 3.096492 -0.000059
C -1.310069 2.991254 -0.000093
C -0.633696 1.772927 -0.000076
C -1.448996 0.677284 -0.000039
C -2.823310 0.808589 0.000011
N -3.384689 2.017461 0.000007
H 0.446011 1.713763 -0.000091
H -0.766153 3.926191 -0.000131
C -3.712531 -0.431819 0.000104
I -0.769646 -1.326769 -0.000044
O -3.002308 -1.518544 -0.000115
O -4.912812 -0.334100 0.000329
C 1.206292 -0.676756 0.000022
C 2.372229 -0.340842 0.000045
Si 4.132826 0.232278 0.000050
C 4.072393 2.094018 0.000007
C 4.951909 -0.419839 1.536672
C 4.951815 -0.419920 -1.536592
H 5.080604 2.511615 -0.000343
H 3.554745 2.468915 -0.884028
H 3.555304 2.468974 0.884344
H 4.939337 -1.509893 1.556024
H 5.992774 -0.094280 1.579655
H 4.447884 -0.059886 2.433909
H 4.447714 -0.059949 -2.433784
H 5.992717 -0.094529 -1.579749
H 4.939051 -1.509979 -1.555900

PyridaIBA_B_CCTMS.log
Energy (E) = -947.724296957 Hartree
Enthalpy (H) = -947.514622 Hartree
Gibbs free energy (G) = -947.585283 Hartree
Charge = 0, Spin = 1
N 2.486016 3.159205 -0.000005
C 3.280619 2.099058 -0.000000
C 2.801721 0.787912 0.000007
C 1.447171 0.668836 0.000011
C 0.634811 1.786875 0.000006
N 1.175367 3.002489 -0.000001
H 4.349142 2.269817 -0.000010
I 0.764189 -1.345025 0.000013
H -0.445170 1.735425 0.000004
C 3.714378 -0.427306 0.000006
O 3.047476 -1.527160 0.000017
O 4.915118 -0.263121 -0.000054
C -1.200245 -0.712077 -0.000017
C -2.363816 -0.371538 -0.000021
Si -4.113503 0.240875 -0.000004
C -4.943335 -0.397782 1.536561
C -4.005608 2.098585 0.000099
C -4.943222 -0.397670 -1.536681
H -5.976757 -0.049594 1.581653
H -4.954628 -1.487891 1.554037
H -4.430513 -0.049720 2.433479
H -3.478765 2.461476 -0.883338
H -5.003123 2.541219 0.001101
H -3.477086 2.461254 0.882626
H -4.954048 -1.487782 -1.554432
H -5.976790 -0.049914 -1.581713
H -4.430543 -0.049169 -2.433513

PyridaIBA_C_CCTMS.log
Energy (E) = -947.727936405 Hartree
Enthalpy (H) = -947.518398 Hartree
Gibbs free energy (G) = -947.588826 Hartree
Charge = 0, Spin = 1
N 0.654716 1.674113 0.000577
C 1.505451 0.701893 0.000263
C 2.884257 0.766619 -0.000086
C 2.442019 3.095051 0.000139
N 1.126130 2.920012 0.000514
H 2.759543 4.128995 0.000104
C 3.754088 -0.479115 -0.000291
O 4.960448 -0.339089 -0.000473
O 3.056513 -1.552781 -0.000265
C 3.371143 2.053405 -0.000150
H 4.440626 2.216045 -0.000397
I 0.731787 -1.308720 0.000359
C -1.203065 -0.642201 0.000749
C -2.369313 -0.315982 0.001050
Si -4.132413 0.244215 -0.000193

C -4.445293 1.123819 1.608714
C -4.354017 1.387722 -1.450248
C -5.202023 -1.270866 -0.161417
H -5.474272 1.485389 1.652110
H -4.284120 0.458791 2.457591
H -3.779934 1.980427 1.718873
H -4.147077 0.874411 -2.389647
H -5.378183 1.763361 -1.487405
H -3.681129 2.242367 -1.376741
H -5.040913 -1.956831 0.670657
H -6.256854 -0.990328 -0.167871
H -4.989729 -1.805439 -1.087692

PyrmIBA_CCTMS.log
Energy (E) = -947.766186552 Hartree
Enthalpy (H) = -947.555910 Hartree
Gibbs free energy (G) = -947.627247 Hartree
Charge = 0, Spin = 1
C 1.223123 2.906103 0.000026
N 0.652514 1.692911 0.000032
C 1.489578 0.708420 0.000018
C 2.858020 0.773286 -0.000004
C 3.337697 2.079842 -0.000010
N 2.527620 3.137588 0.000005
H 0.556416 3.757928 0.000033
H 4.409157 2.240899 -0.000025
C 3.737444 -0.458906 -0.000022
O 4.943370 -0.316351 -0.000056
O 3.049500 -1.542453 -0.000002
I 0.736951 -1.325667 0.000014
C -1.204124 -0.659206 0.000035
C -2.368760 -0.325656 0.000041
Si -4.127594 0.243650 -0.000010
C -4.945882 -0.416318 1.535438
C -4.091199 2.104266 -0.001594
C -4.946622 -0.418912 -1.533942
H -5.989020 -0.097765 1.577608
H -4.925622 -1.506342 1.554264
H -4.444950 -0.053241 2.433207
H -3.575088 2.482516 -0.884398
H -5.105450 2.507376 -0.001740
H -3.574758 2.484008 0.880378
H -4.926558 -1.508970 -1.550852
H -5.989733 -0.100265 -1.576212
H -4.446032 -0.057490 -2.432566

PyrroleIBA_A_CCTMS.log
Energy (E) = -893.659370383 Hartree
Enthalpy (H) = -893.442804 Hartree
Gibbs free energy (G) = -893.513319 Hartree
Charge = 0, Spin = 1
C -1.959271 2.952370 0.000048
C -0.900252 2.068830 -0.000052
C -1.506261 0.799203 0.000019
C -2.861272 0.916249 0.000021
H -1.951344 4.028210 0.000082
H 0.148275 2.307977 -0.000096
C -3.810265 -0.227633 0.000040
I -0.845226 -1.161323 0.000037
O -5.012418 -0.035647 -0.000121
O -3.165902 -1.348376 -0.000091
N -3.130096 2.245944 0.000003
H -4.072261 2.609528 0.000005
C 1.130209 -0.544277 -0.000080
C 2.302614 -0.235919 -0.000112
Si 4.090569 0.231494 -0.000006
C 4.421468 1.231538 -1.534372
C 4.421199 1.231748 1.534288
C 5.080104 -1.344855 0.000141
H 5.470549 1.530276 -1.575460
H 4.198230 0.655754 -2.432961
H 3.812710 2.136095 -1.553695
H 4.197807 0.656106 2.432928
H 5.470258 1.530538 1.575527
H 3.812401 2.136287 1.553345
H 4.860699 -1.946229 -0.882491
H 6.148689 -1.122095 0.000174
H 4.860628 -1.946190 0.882777

PyrroleIBA_B_CCTMS.log
Energy (E) = -893.652534337 Hartree
Enthalpy (H) = -893.435950 Hartree
Gibbs free energy (G) = -893.505417 Hartree
Charge = 0, Spin = 1
C -3.102957 2.327280 0.000083
C -2.898009 0.971775 0.000026
C -1.506529 0.801523 -0.000020
H -4.023303 2.883573 0.000150
I -0.876734 -1.164985 -0.000008
C -0.868660 2.005603 -0.000113
H 0.173811 2.265700 -0.000206
C -3.817393 -0.204306 0.000031
O -3.146160 -1.321263 -0.000022
O -5.024149 -0.078543 0.000047
N -1.877668 2.936001 -0.000033
H -1.730617 3.932584 -0.000059
C 1.112986 -0.541404 -0.000116
C 2.287019 -0.235870 -0.000113
Si 4.078716 0.210050 0.000018
C 4.425968 1.207227 -1.533622
C 4.425512 1.208149 1.533162
C 5.052980 -1.375825 0.000636
H 5.479528 1.489722 -1.574538
H 4.193753 0.635834 -2.432739
H 3.831650 2.121437 -1.552584
H 4.193003 0.637307 2.432553
H 5.479064 1.490650 1.574238
H 3.831206 2.122383 1.551381
H 4.826894 -1.975102 -0.881718
H 6.123863 -1.164496 0.000721
H 4.826647 -1.974571 0.883286

PyrroleIBA_C_CCTMS.log
Energy (E) = -893.646305227 Hartree
Enthalpy (H) = -893.430022 Hartree
Gibbs free energy (G) = -893.499626 Hartree
Charge = 0, Spin = 1
C 1.493020 0.786058 -0.000028
C 2.820924 1.058644 0.000004
C 1.635777 2.971761 0.000004
H 1.273841 3.984987 0.000015
C 3.792770 -0.070959 0.000025
O 4.992096 0.092591 0.000074
O 3.173060 -1.228810 0.000025
C 2.914133 2.477851 -0.000002
H 3.825835 3.049596 0.000014
I 0.939284 -1.196080 -0.000015
N 0.748803 1.912892 -0.000063
H -0.259912 1.948767 -0.000113
C -1.069164 -0.585545 -0.000009
C -2.238137 -0.256998 -0.000016
Si -4.032397 0.192739 0.000015
C -4.375397 1.187186 1.535696
C -4.374016 1.192744 -1.532365
C -5.001150 -1.395454 -0.003287
H -5.429617 1.466485 1.580028
H -4.139475 0.615339 2.433520
H -3.785384 2.104243 1.555247
H -4.137165 0.624213 -2.432047
H -5.428225 1.472083 -1.576688
H -3.784112 2.109948 -1.547992
H -4.773970 -1.995313 0.878320
H -6.072460 -1.186562 -0.003328
H -4.773264 -1.992086 -0.886899

PyrroleINAcBA_B_CCTMS.log
Energy (E) = -1046.18930462 Hartree
Enthalpy (H) = -1045.932204 Hartree
Gibbs free energy (G) = -1046.009693 Hartree
Charge = 0, Spin = 1
C -3.145660 1.162716 0.000217
C -2.707944 -0.125872 0.000197
C -1.298604 -0.050063 0.000144
H -4.152068 1.540877 0.000251
I -0.327905 -1.875413 0.000157
C -0.876917 1.235564 0.000170
H 0.092996 1.697158 0.000140
C -3.410619 -1.446822 0.000252
O -2.555736 -2.424884 0.000200
O -4.621703 -1.522131 0.000306
N -2.036748 1.994375 0.000202
C -2.018498 3.407934 0.000187
C -3.361993 4.075312 0.000266
H -3.933753 3.784286 -0.880396
H -3.933655 3.784266 0.880987
H -3.206320 5.148502 0.000272
O -0.966783 3.992331 0.000209
C 1.506633 -0.906277 -0.000099
C 2.589817 -0.361396 -0.000248
Si 4.236285 0.481616 -0.000581
C 4.338225 1.529068 -1.535085
C 4.339064 1.528942 1.533963
C 5.542865 -0.843790 -0.000593
H 5.297994 2.047108 -1.578949
H 4.241614 0.919373 -2.433831
H 3.549043 2.281457 -1.550384
H 4.242877 0.919066 2.432636
H 5.298971 2.046771 1.577377
H 3.550051 2.281489 1.549900
H 5.459206 -1.478456 -0.883173
H 6.537696 -0.394605 -0.000616
H 5.459103 -1.478091 0.882248

PyrroleINMeBA_B_CCTMS.log
Energy (E) = -932.921466335 Hartree
Enthalpy (H) = -932.675560 Hartree
Gibbs free energy (G) = -932.749326 Hartree
Charge = 0, Spin = 1
C 2.947516 2.055709 -0.015316
C 2.771857 0.694021 0.000194
C 1.386220 0.500924 0.007169
H 3.855765 2.633650 -0.025184
I 0.789786 -1.476440 0.003720
C 0.730547 1.697672 -0.007750
H -0.316532 1.946066 -0.009659
C 3.710859 -0.465187 -0.002794
O 3.059206 -1.594516 0.004771
O 4.915707 -0.320444 -0.010850
N 1.717170 2.650908 -0.023517
C 1.481947 4.079999 0.028167
H 0.602349 4.328888 -0.560997
H 1.332854 4.418114 1.052832
H 2.340518 4.596063 -0.393664
C -1.202747 -0.859330 0.003107

C -2.355407 -0.481964 0.001619
Si -4.076685 0.180252 -0.002179
C -4.935507 -0.391353 1.546407
C -3.920561 2.038164 -0.034919
C -4.948689 -0.444495 -1.522628
H -5.955245 -0.003841 1.583456
H -4.986994 -1.479771 1.586600
H -4.409110 -0.045786 2.436528
H -3.385045 2.365733 -0.927345
H -4.903299 2.512656 -0.038559
H -3.376858 2.396423 0.840625
H -4.999686 -1.533679 -1.525016
H -5.969023 -0.058986 -1.563851
H -4.430476 -0.129378 -2.428705

PyrroleINMsBA_B_CCTMS.log
Energy (E) = -1481.34274388 Hartree
Enthalpy (H) = -1481.083681 Hartree
Gibbs free energy (G) = -1481.163804 Hartree
Charge = 0, Spin = 1
C 2.854529 0.721172 -0.193636
C 2.446906 -0.577489 -0.098608
C 1.039979 -0.538284 -0.034401
H 3.836415 1.151809 -0.286696
I 0.106371 -2.379164 0.061587
C 0.577253 0.734594 -0.086699
H -0.407708 1.165147 -0.083702
C 3.175261 -1.886583 -0.060496
O 2.338340 -2.877865 0.026543
O 4.385301 -1.943241 -0.101835
N 1.718263 1.511842 -0.194891
C 1.945459 3.483701 1.608411
H 1.119736 3.032189 2.151197
H 2.900107 3.053968 1.899716
H 1.952254 4.562987 1.741346
S 1.707604 3.194536 -0.113306
O 0.377930 3.587469 -0.476944
O 2.866527 3.639334 -0.824165
C -1.715879 -1.382051 0.060450
C -2.703192 -0.678789 0.032614
Si -4.098225 0.533916 -0.032451
C -3.297985 2.216839 -0.010636
C -5.049044 0.252322 -1.605556
C -5.174435 0.276138 1.462988
H -4.052920 3.004456 -0.041257
H -2.706807 2.358139 0.896073
H -2.636467 2.357333 -0.867294
H -5.471334 -0.752482 -1.635338
H -5.870778 0.966106 -1.686403
H -4.407231 0.375810 -2.478141
H -4.606746 0.412801 2.383778
H -5.999742 0.990389 1.465185
H -5.598666 -0.728252 1.473470

PyrroleNAcIBA_A_CCTMS.log
Energy (E) = -1046.17736723 Hartree
Enthalpy (H) = -1045.920623 Hartree
Gibbs free energy (G) = -1045.998829 Hartree
Charge = 0, Spin = 1
C 1.819062 2.217764 0.079109
C 0.635584 1.533257 0.048292
C 1.020014 0.177830 -0.022641
C 2.372118 0.043369 -0.032741
H 1.992760 3.279347 0.092581
H -0.359294 1.941418 0.066003
C 3.033447 -1.296021 0.151839
I -0.034734 -1.599687 -0.018579
O 4.195344 -1.415106 0.464540
O 2.151830 -2.238206 0.000300
N 2.874402 1.326087 0.023222
C 4.235649 1.717343 -0.205596
C 4.615419 3.041202 0.398565
H 4.210566 3.856692 -0.202006
H 4.233435 3.140404 1.412594
H 5.698009 3.112633 0.389518
O 4.967394 1.033247 -0.854723
C -1.837088 -0.565217 -0.010652
C -2.921081 -0.021416 -0.008414
Si -4.576717 0.799529 -0.005776
C -4.678604 1.879194 1.507188
C -4.716662 1.813917 -1.560089
C -5.865971 -0.541792 0.039031
H -5.645565 2.383638 1.551050
H -4.562866 1.290134 2.417345
H -3.900732 2.643463 1.498868
H -4.624875 1.186260 -2.446879
H -5.683976 2.317965 -1.600752
H -3.937892 2.575962 -1.604587
H -5.762707 -1.155644 0.934172
H -6.866940 -0.106524 0.041276
H -5.783662 -1.194440 -0.830464

PyrroleNAcIBA_C_CCTMS.log
Energy (E) = -1046.17085979 Hartree
Enthalpy (H) = -1045.914147 Hartree
Gibbs free energy (G) = -1045.989521 Hartree
Charge = 0, Spin = 1
C 2.986097 2.171760 0.642561
C 1.756844 2.721636 0.469630
N 0.861688 1.732703 0.036049
C 2.878672 0.781952 0.330377
C 1.595995 0.570627 -0.035306
C 3.874771 -0.336124 0.308801
O 5.029897 -0.155738 0.633172
O 3.328109 -1.434821 -0.113427
I 1.038260 -1.430934 -0.212069
C -0.312104 2.024476 -0.700939
C -1.008838 3.300347 -0.324683
H -1.016359 3.449706 0.753366
H -0.504715 4.149512 -0.787081
H -2.023281 3.253581 -0.710657
O -0.685755 1.284235 -1.571227
H 3.886231 2.673215 0.953834
H 1.427307 3.742382 0.553440
C -0.976323 -1.038881 0.017080
C -2.159501 -0.842548 0.187590
Si -3.921582 -0.308551 0.300115
C -4.977895 -1.768035 0.762000
C -3.996418 1.020337 1.605631
C -4.374538 0.384169 -1.366119
H -6.026786 -1.473569 0.827469
H -4.896895 -2.561316 0.018436
H -4.681253 -2.177930 1.727794
H -3.347006 1.857186 1.340907
H -5.012858 1.404367 1.708344
H -3.676690 0.640345 2.576194
H -4.354693 -0.392334 -2.131297
H -5.372621 0.825408 -1.352692
H -3.656399 1.152050 -1.660238

PyrroleNMeIBA_A_CCTMS.log
Energy (E) = -932.929507452 Hartree
Enthalpy (H) = -932.683469 Hartree
Gibbs free energy (G) = -932.756624 Hartree
Charge = 0, Spin = 1
C 1.783051 2.660203 0.000004
C 0.699436 1.804252 -0.000064
C 1.279928 0.528184 -0.000064
C 2.641446 0.614406 -0.000021
H 1.806610 3.737161 0.000016
H -0.343990 2.066790 -0.000113
C 3.544127 -0.568113 0.000047
I 0.555075 -1.409289 -0.000045
O 4.756643 -0.442184 0.000152
O 2.857459 -1.664138 -0.000018
N 2.944341 1.942017 0.000003
C 4.288103 2.497530 0.000048
H 4.832764 2.163521 0.878623
H 4.832947 2.163185 -0.878281
H 4.204828 3.581725 -0.000162
C -1.392670 -0.704937 0.000002
C -2.529272 -0.283747 0.000027
Si -4.226680 0.443060 0.000068
C -5.109355 -0.129914 1.534680
C -4.003890 2.292653 0.000135
C -5.109297 -0.129506 -1.534738
H -6.116008 0.290106 1.575169
H -5.196715 -1.216618 1.554259
H -4.575922 0.182167 2.432909
H -3.451913 2.617176 -0.883054
H -4.970062 2.800133 -0.000250
H -3.452485 2.617351 0.883610
H -5.196515 -1.216213 -1.554753
H -6.115990 0.290419 -1.575146
H -4.575836 0.182982 -2.432814

PyrroleNMeIBA_C_CCTMS.log
Energy (E) = -932.910934224 Hartree
Enthalpy (H) = -932.665031 Hartree
Gibbs free energy (G) = -932.737155 Hartree
Charge = 0, Spin = 1
C -1.631105 0.732809 -0.000003
C -2.992432 0.808092 -0.000339
C -2.122617 2.866574 0.000063
H -1.911973 3.923089 0.000214
C -3.834816 -0.417917 -0.000353
O -5.045561 -0.374726 -0.000665
O -3.096950 -1.497672 0.000060
C -3.309593 2.187036 -0.000331
H -4.302428 2.601786 -0.000551
I -0.878602 -1.217496 0.000208
N -1.070879 1.973050 0.000277
C 0.320517 2.382773 0.000668
H 0.839581 2.016968 0.883861
H 0.839825 2.017914 -0.882777
H 0.338659 3.469456 0.001275
C 1.127122 -0.622346 0.000053
C 2.323784 -0.422191 -0.000114
Si 4.108806 0.044968 -0.000195
C 4.142712 1.909798 0.000462
C 4.903826 -0.642372 1.534556
C 4.903547 -0.641206 -1.535602
H 5.168991 2.280771 0.000774
H 3.642506 2.309120 -0.883538
H 3.642183 2.308581 0.884527
H 4.838363 -1.730575 1.555646
H 5.959426 -0.367670 1.573581
H 4.420613 -0.258082 2.433236
H 4.420225 -0.256147 -2.433882
H 5.959160 -0.366514 -1.574541
H 4.838082 -1.729389 -1.557580

PyrroleNMsIBA_A_CCTMS.log
Energy (E) = -1481.32885971 Hartree
Enthalpy (H) = -1481.069793 Hartree

Gibbs free energy (G) = -1481.150605 Hartree
Charge = 0, Spin = 1
C -1.546558 1.945039 -0.000291
C -0.309066 1.357136 -0.000264
C -0.575082 -0.024108 -0.000084
C -1.911295 -0.268036 -0.000008
H -1.792431 2.990498 -0.000398
H 0.645800 1.852254 -0.000358
C -2.486587 -1.652042 -0.000003
I 0.611991 -1.716593 0.000003
O -3.681079 -1.846651 -0.000158
O -1.528406 -2.524851 0.000106
N -2.521488 0.964891 -0.000121
C -4.314928 2.965965 0.000249
H -3.867622 3.363274 0.906165
H -3.867981 3.363461 -0.905763
H -5.385926 3.158446 0.000478
S -4.231593 1.202134 0.000059
O -4.728634 0.737542 -1.255173
O -4.728368 0.737292 1.255304
C 2.325928 -0.547260 -0.000057
C 3.364021 0.079498 -0.000055
Si 4.953748 1.023736 0.000032
C 4.994203 2.076749 -1.534117
C 4.994200 2.076449 1.534390
C 6.338725 -0.218965 -0.000099
H 5.920819 2.652049 -1.576669
H 4.936166 1.462244 -2.432881
H 4.160756 2.779795 -1.551842
H 4.936160 1.461772 2.433036
H 5.920803 2.651759 1.577065
H 4.160739 2.779476 1.552238
H 6.293228 -0.857315 -0.882809
H 7.304653 0.289332 -0.000061
H 6.293256 -0.857510 0.882468

PyrroleNMsIBA_C_CCTMS.log
Energy (E) = -1481.32384831 Hartree
Enthalpy (H) = -1481.065034 Hartree
Gibbs free energy (G) = -1481.143035 Hartree
Charge = 0, Spin = 1
C -1.501585 0.294135 0.212089
C -2.732021 0.463075 0.736147
C -1.449580 2.215940 1.319970
H -1.011234 3.139439 1.660836
C -3.803018 -0.566421 0.505360
O -4.917894 -0.410020 0.959863
O -3.360525 -1.532529 -0.231370
C -2.702923 1.712394 1.440568
H -3.529381 2.140061 1.982286
I -1.045929 -1.634883 -0.439886
N -0.656543 1.335273 0.551676
C -0.835693 3.198140 -1.392676
H -1.744226 2.636464 -1.605300
H -1.030134 4.036853 -0.732635
H -0.364341 3.524732 -2.316510
S 0.314841 2.095690 -0.629016
O 1.303802 2.840999 0.091784
O 0.669971 1.102190 -1.597491
C 0.967525 -1.350570 -0.155031
C 2.131722 -1.136328 0.099256
Si 3.855059 -0.545637 0.422553
C 4.333346 0.524674 -1.020428
C 3.780677 0.452634 1.990453
C 4.968523 -2.027485 0.591690
H 5.338567 0.928508 -0.886595
H 4.310640 -0.036199 -1.955042
H 3.636903 1.359784 -1.109549
H 3.445690 -0.152260 2.833379
H 4.763008 0.861885 2.233214
H 3.087186 1.286340 1.867419
H 4.965199 -2.628246 -0.318243
H 5.995364 -1.709998 0.781602
H 4.653824 -2.663862 1.419233

QuinoIBA_A_CCTMS.log
Energy (E) = -1085.22708668 Hartree
Enthalpy (H) = -1084.955447 Hartree
Gibbs free energy (G) = -1085.031705 Hartree
Charge = 0, Spin = 1
C 3.888887 0.973113 0.484012
C 3.009530 -0.107252 0.239865
C 1.701871 0.154681 -0.011783
H 4.918969 0.715515 0.689163
C 3.571670 -1.523246 0.196321
O 2.701285 -2.385946 -0.175358
O 4.742854 -1.693999 0.475630
I 0.519239 -1.620669 -0.240477
C 3.432146 2.256785 0.453118
H 4.097355 3.089008 0.646323
C -0.534617 2.903344 -0.596412
C 0.263128 4.034440 -0.322945
C 1.567824 3.840330 0.033928
C 2.079859 2.528926 0.135780
C 1.177450 1.462625 -0.124742
H -1.566885 3.028825 -0.907552
H -0.157170 5.025763 -0.411890
H 2.226987 4.675939 0.233805
N -0.098900 1.672900 -0.504108
C -1.372015 -0.814597 -0.076492
C -2.557704 -0.599575 0.055241
Si -4.326854 -0.123029 0.228590
C -4.421219 1.713066 -0.084681
C -5.317714 -1.065653 -1.034553
C -4.888965 -0.530359 1.955881
H -5.445184 2.075706 0.020871
H -3.793524 2.256662 0.623255
H -4.080251 1.948170 -1.094149
H -5.234877 -2.141108 -0.874756
H -6.373462 -0.795729 -0.971223
H -4.972158 -0.847094 -2.045339
H -4.291414 0.001015 2.697186
H -5.933626 -0.246516 2.095933
H -4.800090 -1.598791 2.154771

QuinoIBA_B_CCTMS.log
Energy (E) = -1085.22058878 Hartree
Enthalpy (H) = -1084.949210 Hartree
Gibbs free energy (G) = -1085.026946 Hartree
Charge = 0, Spin = 1
C -2.272432 -0.377667 0.022099
C -0.947362 -0.063929 -0.010292
C -2.691223 -1.847715 0.114802
O -1.686504 -2.648528 -0.083377
O -3.820924 -2.182083 0.370464
I 0.337107 -1.761969 -0.065722
C -1.276866 2.273299 0.001902
C -0.402132 1.224901 -0.014572
H 0.666904 1.380071 -0.028404
H -0.909438 3.291910 0.003811
C -2.671088 2.048643 0.002051
C -3.586966 3.124561 -0.007753
C -4.924658 2.863736 -0.034090
C -5.339544 1.513152 -0.063711
C -3.189793 0.725609 0.000144
H -3.206364 4.138573 0.000693
H -5.658804 3.656731 -0.042703
H -6.398365 1.279726 -0.107346
N -4.521658 0.493885 -0.047653
C 2.051010 -0.564165 -0.019728
C 3.108784 0.031097 0.000782
Si 4.689726 0.984400 0.035133
C 5.656133 0.566049 -1.499109
C 4.230243 2.789874 0.066313
C 5.626964 0.505651 1.569700
H 6.597898 1.117503 -1.519012
H 5.888185 -0.498618 -1.538303
H 5.094736 0.822307 -2.397990
H 3.633748 3.026136 0.948370
H 5.125555 3.413664 0.088450
H 3.652810 3.061628 -0.818226
H 5.858926 -0.559752 1.571217
H 6.567778 1.055860 1.629427
H 5.048232 0.726148 2.467061

QuinoIBA_C_CCTMS.log
Energy (E) = -1085.23419245 Hartree
Enthalpy (H) = -1084.962599 Hartree
Gibbs free energy (G) = -1085.037787 Hartree
Charge = 0, Spin = 1
C 3.338842 0.598446 -0.000056
C 2.533126 -0.509201 -0.000037
C 1.148428 -0.312174 0.000008
C 0.541382 0.901234 0.000042
H 4.412723 0.469194 -0.000091
H -0.535187 1.013937 0.000083
C 3.117230 -1.908501 -0.000060
O 2.209669 -2.829250 -0.000006
O 4.319793 -2.060309 -0.000056
I 0.062199 -2.143874 0.000039
C 0.842875 3.358321 0.000066
C 1.703124 4.416553 0.000047
C 3.096487 4.162574 -0.000011
C 2.781975 1.896544 -0.000027
C 1.371438 2.047492 0.000027
H -0.231897 3.498531 0.000112
H 1.344719 5.436068 0.000077
H 3.788942 4.997619 -0.000025
N 3.622677 2.966078 -0.000045
C -1.737788 -1.102740 0.000096
C -2.770787 -0.466800 0.000104
Si -4.226007 0.669675 -0.000026
C -3.489565 2.384658 -0.000101
C -5.227312 0.363366 -1.536548
C -5.227511 0.363643 1.536419
H -4.267630 3.149602 -0.000060
H -2.868297 2.535351 0.885041
H -2.868417 2.535344 -0.885327
H -5.608156 -0.658059 -1.558862
H -6.081130 1.041986 -1.577715
H -4.627047 0.519323 -2.433216
H -4.627439 0.519819 2.433172
H -6.081291 1.042346 1.577200
H -5.608459 -0.657734 1.558899

QuinoIBA_D_CCTMS.log
Energy (E) = -1085.23680043 Hartree
Enthalpy (H) = -1084.965101 Hartree
Gibbs free energy (G) = -1085.041878 Hartree
Charge = 0, Spin = 1
C 3.300792 0.693263 -0.000053
C 2.540590 -0.445714 -0.000043
C 1.150508 -0.296195 0.000004
C 0.497284 0.891850 0.000036

H 4.378482 0.580886 -0.000106
H -0.579214 0.986306 0.000039
C 3.180627 -1.819573 -0.000089
O 2.313383 -2.774345 0.000025
O 4.390773 -1.916219 -0.000192
I 0.124944 -2.166028 0.000027
C 1.356803 4.354228 0.000089
C 2.771768 4.362483 0.000058
C 2.695389 1.967717 -0.000003
C 1.281009 2.072395 0.000047
H 0.818370 5.295816 0.000158
H 3.300431 5.304918 0.000072
N 0.630695 3.265175 0.000094
C 3.437575 3.171533 0.000004
H 4.519213 3.126123 -0.000034
C -1.701516 -1.188142 0.000086
C -2.734261 -0.552257 0.000096
Si -4.183372 0.597035 -0.000021
C -5.186415 0.281362 1.534711
C -3.448844 2.308396 -0.000046
C -5.186342 0.281695 -1.534862
H -6.041336 0.958651 1.576497
H -5.565501 -0.740835 1.553500
H -4.587728 0.436907 2.432498
H -2.823720 2.469511 -0.879863
H -4.233714 3.066928 -0.000124
H -2.823726 2.469670 0.879742
H -5.565459 -0.740476 -1.554077
H -6.041233 0.959040 -1.576417
H -4.587624 0.437602 -2.432571

QuinoIBA_E_CCTMS.log
Energy (E) = -1085.23454741 Hartree
Enthalpy (H) = -1084.962725 Hartree
Gibbs free energy (G) = -1085.039419 Hartree
Charge = 0, Spin = 1
C 2.286245 -0.395897 -0.000026
C 0.965044 -0.061602 0.000002
C 2.684472 -1.867797 -0.000060
O 1.670738 -2.662976 0.000010
O 3.851509 -2.208692 -0.000075
I -0.361058 -1.727125 0.000030
C 1.344825 2.268880 0.000031
C 0.449141 1.238441 0.000026
H -0.616416 1.415836 0.000042
H 1.023389 3.300880 0.000051
C 2.737221 2.019889 0.000014
C 4.843598 2.905446 0.000019
C 5.430683 1.623157 -0.000014
C 3.223750 0.685438 -0.000018
H 5.474243 3.788267 0.000036
H 6.507428 1.527779 -0.000024
C 4.631305 0.515197 -0.000034
H 5.033925 -0.484538 -0.000061
N 3.551140 3.107768 0.000031
C -2.047923 -0.510997 0.000054
C -3.106601 0.082227 0.000071
Si -4.722097 0.982494 -0.000020
C -4.792814 2.032237 1.535051
C -4.791455 2.034302 -1.533739
C -6.071407 -0.299209 -0.001467
H -5.735383 2.581087 1.576986
H -4.718355 1.419009 2.433462
H -3.979797 2.758719 1.553858
H -4.716189 1.422287 -2.432910
H -5.733990 2.583203 -1.575772
H -3.978431 2.760819 -1.550840
H -6.008565 -0.936624 0.880843
H -7.051078 0.182124 -0.001569
H -6.007799 -0.935446 -0.884572

QuinoIBA_F_CCTMS.log
Energy (E) = -1085.22526413 Hartree
Enthalpy (H) = -1084.953486 Hartree
Gibbs free energy (G) = -1085.028488 Hartree
Charge = 0, Spin = 1
C 3.621073 1.218952 0.732012
C 2.894382 0.071217 0.342050
C 1.604875 0.219608 -0.055537
H 4.641362 1.058240 1.052368
C 3.604570 -1.272417 0.291052
O 2.881790 -2.198363 -0.236880
O 4.748507 -1.352959 0.688118
I 0.670302 -1.705752 -0.328979
C 3.032595 2.446583 0.705688
H 3.550546 3.341970 1.018823
C -0.712928 3.007723 -0.982280
C 0.056421 4.053115 -0.431262
C 1.716432 2.605790 0.206484
C 0.970423 1.470075 -0.229928
H -1.642612 3.229230 -1.487376
H -0.306055 5.074259 -0.477123
C -0.257833 1.723947 -0.882163
H -0.819894 0.915949 -1.322602
N 1.227284 3.867997 0.121014
C -1.280355 -1.032349 -0.094156
C -2.450763 -0.775522 0.094547
Si -4.198389 -0.232602 0.342902
C -5.295656 -1.299997 -0.714427
C -4.621493 -0.403777 2.145601
C -4.259752 1.549659 -0.199452
H -6.338696 -0.997200 -0.606270
H -5.027417 -1.217567 -1.768019
H -5.218469 -2.348910 -0.426971
H -3.954155 0.197822 2.763178
H -5.644847 -0.072367 2.330580
H -4.538996 -1.441154 2.470936
H -3.982543 1.643432 -1.251088
H -5.263045 1.961009 -0.076805
H -3.569051 2.155537 0.389269

QuinoIBA_G_CCTMS.log
Energy (E) = -1085.22532235 Hartree
Enthalpy (H) = -1084.953878 Hartree
Gibbs free energy (G) = -1085.029303 Hartree
Charge = 0, Spin = 1
C -3.139907 4.185432 -0.000033
C -3.647128 2.916009 -0.000055
C -2.774958 1.801428 0.000020
C -1.374188 2.026608 0.000142
C -0.875371 3.349528 0.000136
C -1.742691 4.406077 0.000051
H -3.809696 5.034505 -0.000101
H -4.709605 2.717719 -0.000144
C -0.527314 0.896378 0.000220
H 0.197187 3.504956 0.000161
H -1.363577 5.418812 0.000046
C -1.125614 -0.319836 0.000199
C -2.524807 -0.483823 -0.000006
H 0.548924 1.017940 0.000260
N -3.315081 0.557842 -0.000081
C -3.116551 -1.890546 -0.000214
O -4.311000 -2.057202 -0.000841
O -2.194144 -2.805014 0.000439
I -0.055654 -2.146077 0.000152
C 1.751443 -1.105638 -0.000122
C 2.776892 -0.456988 -0.000110
Si 4.222699 0.692313 -0.000120
C 5.226235 0.393238 -1.536598
C 3.473459 2.401537 -0.000207
C 5.226463 0.393967 1.536355
H 6.074922 1.078254 -1.577779
H 5.614720 -0.625308 -1.558773
H 4.624899 0.544677 -2.433320
H 2.852208 2.549675 0.885368
H 4.246650 3.171462 -0.001300
H 2.850529 2.548835 -0.884727
H 5.615577 -0.624317 1.558904
H 6.074772 1.079514 1.576971
H 4.625312 0.545568 2.433169

QuinoIBA_H_CCTMS.log
Energy (E) = -1085.23083905 Hartree
Enthalpy (H) = -1084.958967 Hartree
Gibbs free energy (G) = -1085.035830 Hartree
Charge = 0, Spin = 1
C 4.841076 2.972429 0.000041
C 3.484569 3.131840 0.000097
C 2.634048 2.003117 0.000050
C 3.194237 0.693995 -0.000054
C 4.605672 0.562104 -0.000117
C 5.399744 1.677013 -0.000069
H 5.490687 3.837270 0.000079
H 3.015417 4.105607 0.000179
C 2.287107 -0.408029 -0.000077
H 5.027657 -0.429125 -0.000203
H 6.475204 1.561952 -0.000117
C 0.964108 -0.089540 0.000004
C 0.473802 1.225147 0.000085
N 1.294823 2.240189 0.000109
I -0.359527 -1.749535 0.000063
H -0.589735 1.425876 0.000136
C 2.700339 -1.881741 -0.000199
O 1.689847 -2.678292 0.000057
O 3.870406 -2.206228 -0.000089
C -2.042622 -0.536312 -0.000073
C -3.094494 0.068010 -0.000123
Si -4.693871 0.999131 -0.000050
C -4.742063 2.047731 1.536177
C -4.739836 2.051647 -1.533662
C -6.066142 -0.258037 -0.002594
H -5.672658 2.616668 1.578366
H -4.681092 1.432266 2.434087
H -3.913493 2.756365 1.555216
H -4.677623 1.438479 -2.433057
H -5.670333 2.620754 -1.575716
H -3.911198 2.760276 -1.549707
H -6.015661 -0.896966 0.879433
H -7.036693 0.241463 -0.002647
H -6.014432 -0.894779 -0.886130

QuinoIBA_I_CCTMS.log
Energy (E) = -1085.22102614 Hartree
Enthalpy (H) = -1084.949112 Hartree
Gibbs free energy (G) = -1085.025536 Hartree
Charge = 0, Spin = 1
C 0.565914 2.837472 -1.035494
C 1.793101 3.152186 -0.533418
C 2.651938 2.148517 -0.027085
C 2.265677 0.773024 -0.057961
C 0.950139 0.531091 -0.523317
C 0.123677 1.499291 -1.005001
H -0.087862 3.600742 -1.432717
H 2.159315 4.168315 -0.496681
C 3.228618 -0.175602 0.391899

H -0.874978 1.259031 -1.340782
C 4.657819 1.674880 0.965178
N 3.836542 2.570550 0.482358
H 5.590011 2.039776 1.381853
C 3.125804 -1.677047 0.189736
O 3.912089 -2.405571 0.758743
O 2.225048 -2.041667 -0.665530
C 4.397874 0.292655 0.924409
H 5.125371 -0.429725 1.266620
I 0.090078 -1.414943 -0.330191
C -1.793303 -0.585695 -0.048705
C -2.939236 -0.223914 0.119282
Si -4.670510 0.367042 0.381174
C -5.806038 -0.738293 -0.594342
C -5.044636 0.277230 2.201336
C -4.748906 2.123186 -0.234139
H -6.843250 -0.421301 -0.471455
H -5.566811 -0.707390 -1.657646
H -5.728523 -1.773369 -0.260475
H -4.357373 0.900565 2.773944
H -6.060770 0.623255 2.399228
H -4.958868 -0.745545 2.569306
H -4.508034 2.177874 -1.296400
H -5.751172 2.532207 -0.094829
H -4.047597 2.759106 0.307258

QuinoIBA_J_CCTMS.log
Energy (E) = -1085.21911398 Hartree
Enthalpy (H) = -1084.947320 Hartree
Gibbs free energy (G) = -1085.023647
Hartree
Charge = 0, Spin = 1
C -4.721976 1.663501 0.970303
C -3.842549 2.576997 0.466667
C -2.601690 2.162891 -0.070067
C -2.261355 0.771774 -0.080114
C -4.413633 0.292814 0.904626
H -5.666410 1.982764 1.388710
H -4.045438 3.638582 0.456748
C -0.940331 0.517706 -0.515438
H -5.119509 -0.456212 1.236549
C -0.615135 2.815938 -1.003021
N -1.788210 3.143180 -0.539383
H 0.010513 3.610856 -1.392775
C -0.120762 1.493494 -0.984020
H 0.890385 1.291451 -1.305338
I -0.088283 -1.426930 -0.306542
C -3.234355 -0.165113 0.375080
C -3.128412 -1.661580 0.177072
O -3.925230 -2.400100 0.717002
O -2.201214 -2.035081 -0.654681
C 1.795749 -0.578385 -0.040918
C 2.942239 -0.213889 0.119044
Si 4.674815 0.379377 0.368741
C 5.803640 -0.716564 -0.624963
C 4.743833 2.139778 -0.235387
C 5.065754 0.277892 2.184733
H 6.841239 -0.398011 -0.509639
H 5.731604 -1.754126 -0.297645
H 5.554326 -0.678764 -1.685729
H 4.047414 2.771342 0.317286
H 5.746911 2.549240 -0.103341
H 4.492598 2.201505 -1.294874
H 4.985232 -0.747524 2.546498
H 6.083019 0.624480 2.375653
H 4.382642 0.896077 2.767797

QuinoIBA_K_CCTMS.log
Energy (E) = -1085.22344246 Hartree
Enthalpy (H) = -1084.951734 Hartree
Gibbs free energy (G) = -1085.026955
Hartree
Charge = 0, Spin = 1
C 0.142244 4.135840 -0.408968
C 1.376355 3.874380 0.116700
C 1.861291 2.550787 0.208020
C 1.019097 1.471890 -0.206074
C -0.655854 3.077592 -0.886747
H -0.213270 5.154165 -0.484767
H 2.037200 4.658911 0.457327
C 1.605463 0.196594 -0.052767
H -1.610656 3.291573 -1.348107
C 3.633715 1.179270 0.665048
N 3.133228 2.381084 0.657377
H 4.661191 1.030276 0.976111
C 2.897290 0.020415 0.305002
I 0.626500 -1.717413 -0.306015
C -0.232252 1.779795 -0.787836
H -0.848923 0.989305 -1.183418
C 3.590895 -1.328539 0.264547
O 2.837006 -2.251969 -0.219531
O 4.744204 -1.413443 0.632254
C -1.314390 -1.023785 -0.090484
C -2.485781 -0.764505 0.086416
Si -4.228179 -0.193933 0.313394
C -5.325112 -1.217461 -0.786514
C -4.688509 -0.393999 2.103872
C -4.236517 1.599398 -0.193302
H -6.363770 -0.895395 -0.692986
H -5.034001 -1.119875 -1.832687
H -5.275136 -2.273173 -0.518533
H -4.021007 0.180737 2.746324
H -5.708208 -0.045124 2.276509
H -4.633874 -1.439391 2.408783
H -3.937916 1.706724 -1.237764
H -5.231015 2.033965 -0.079583
H -3.540520 2.174947 0.419194

QuinoIBA_L_CCTMS.log
Energy (E) = -1085.24022182 Hartree
Enthalpy (H) = -1084.968644 Hartree
Gibbs free energy (G) = -1085.045599
Hartree
Charge = 0, Spin = 1
C -1.770924 4.344616 0.000894
C -0.952468 3.247042 0.001373
C -1.515727 1.955775 0.000691
C -2.925883 1.797187 -0.000409
C -3.175462 4.197462 -0.000244
H -1.341363 5.337105 0.001366
H 0.125168 3.332573 0.002401
H -3.802754 5.078059 -0.000616
C -1.259189 -0.274495 0.000537
N -0.693630 0.862006 0.001122
C -2.619489 -0.596911 -0.000450
C -3.741698 2.952128 -0.000874
H -4.816997 2.828750 -0.001706
C -3.112664 -2.027671 -0.000815
O -4.310658 -2.233804 -0.001767
O -2.154783 -2.883706 0.000002
C -3.455598 0.486677 -0.000942
H -4.524596 0.307124 -0.001728
I 0.005127 -2.050126 0.000975
C 1.709599 -0.897132 0.001440
C 2.752549 -0.280116 0.001721
Si 4.319009 0.694979 -0.000281
C 4.217241 1.928644 -1.391001
C 4.461844 1.556457 1.643770
C 5.729799 -0.491782 -0.259078
H 5.119866 2.541358 -1.426820
H 4.109703 1.426363 -2.352707
H 3.361979 2.592676 -1.261022
H 4.495020 0.836158 2.461639
H 5.372430 2.157054 1.683894
H 3.611769 2.218263 1.812746
H 5.629961 -1.018553 -1.208484
H 6.680837 0.043852 -0.267845
H 5.768480 -1.235058 0.537733

ThiopheneIBA_A_CCTMS.log
Energy (E) = -1236.43646702 Hartree
Enthalpy (H) = -1236.235681 Hartree
Gibbs free energy (G) = -1236.305795
Hartree
Charge = 0, Spin = 1
C -1.539418 2.847615 0.000071
C -0.649429 1.810450 0.000096
C -1.366069 0.596334 0.000048
C -2.714353 0.690910 0.000004
H -1.306264 3.899486 0.000089
H 0.424971 1.905731 0.000128
C -3.629794 -0.497503 -0.000044
I -0.669032 -1.376265 0.000020
O -4.833825 -0.351063 -0.000152
O -2.951371 -1.598477 0.000034
S -3.167181 2.328507 -0.000016
C 1.291991 -0.707120 0.000014
C 2.446326 -0.336444 0.000013
Si 4.176070 0.315494 -0.000011
C 4.029516 2.172633 -0.000488
C 5.029316 -0.295458 1.535826
C 5.029541 -0.296154 -1.535452
H 5.016055 2.639176 -0.000527
H 3.492922 2.520536 -0.884306
H 3.492759 2.521008 0.883046
H 5.069897 -1.384875 1.555347
H 6.053059 0.080689 1.578009
H 4.508628 0.039369 2.433314
H 4.509103 0.038466 -2.433163
H 6.053370 0.079762 -1.577575
H 5.069895 -1.385588 -1.554586

ThiopheneIBA_B_CCTMS.log
Energy (E) = -1236.43518078 Hartree
Enthalpy (H) = -1236.234563 Hartree
Gibbs free energy (G) = -1236.304506
Hartree
Charge = 0, Spin = 1
C -3.108633 2.073676 -0.000040
C -2.787177 0.750598 -0.000022
C -1.389332 0.590731 0.000002
H -4.102197 2.490189 -0.000061
I -0.764765 -1.403435 0.000020
C -0.649672 1.722831 0.000010
H 0.420717 1.839903 0.000030
C -3.704993 -0.439843 -0.000035
O -3.039514 -1.553610 -0.000045
O -4.909085 -0.297446 -0.000027
S -1.717597 3.057578 0.000020
C 1.216607 -0.780697 0.000046
C 2.374283 -0.419588 0.000048
Si 4.109276 0.217602 -0.000038
C 3.976833 2.076158 -0.000335
C 4.958335 -0.398593 1.535968
C 4.958534 -0.399174 -1.535696
H 4.966651 2.535670 -0.000588
H 3.443093 2.428567 -0.884209
H 3.443479 2.428795 0.883681
H 4.991035 -1.488252 1.556267
H 5.984726 -0.029721 1.577919

H 4.440023 -0.059469 2.433232
H 4.440363 -0.060566 -2.433227
H 5.984851 -0.030050 -1.577542
H 4.991556 -1.488826 -1.555473

ThiopheneIBA_C_CCTMS.log
Energy (E) = -1236.42982865 Hartree
Enthalpy (H) = -1236.228944 Hartree
Gibbs free energy (G) = -1236.298163 Hartree
Charge = 0, Spin = 1
C -1.390018 0.644106 -0.000026
C -2.706467 0.943753 -0.000024
C -1.721534 3.029739 0.000092
H -1.564051 4.095693 0.000132
C -3.730475 -0.156109 -0.000094
O -4.915451 0.100008 -0.000126
O -3.175998 -1.330916 -0.000053
C -2.900741 2.352840 0.000019
H -3.879818 2.806468 0.000003
I -0.911476 -1.375362 0.000021
S -0.352594 1.987379 0.000054
C 1.101514 -0.867652 0.000285
C 2.264600 -0.525682 0.000241
Si 3.992986 0.130811 -0.000021
C 4.850818 -0.479012 1.533955
C 4.849019 -0.475509 -1.536392
C 3.826792 1.985360 0.002093
H 5.872640 -0.097589 1.575875
H 4.329255 -0.147971 2.432325
H 4.897189 -1.568231 1.551644
H 4.326693 -0.141980 -2.433397
H 5.870954 -0.094389 -1.578382
H 4.894945 -1.564696 -1.556873
H 3.282609 2.323271 0.885237
H 4.806786 2.465367 0.001825
H 3.281261 2.325077 -0.879530

PhIBr_02.log
Energy (E) = -2817.30045940 Hartree
Enthalpy (H) = -2817.200322 Hartree
Gibbs free energy (G) = -2817.248481 Hartree
Charge = 0, Spin = 2
C -3.634655 1.335858 0.048720
C -3.579801 0.094405 0.667554
C -2.414371 -0.660494 0.621398
C -1.318236 -0.150779 -0.055239
C -1.349619 1.085367 -0.678654
C -2.521701 1.828616 -0.619749
H -4.543026 1.920717 0.090180
H -4.442216 -0.292033 1.193044
H -2.364469 -1.624421 1.107520
H -0.476299 1.468843 -1.187178
H -2.558679 2.797308 -1.098703
I 0.447844 -1.304458 -0.145391
Br 2.273121 1.247658 0.219906

PhICCTMS_02.log
Energy (E) = -727.824843249 Hartree
Enthalpy (H) = -727.596643 Hartree
Gibbs free energy (G) = -727.668270 Hartree
Charge = 0, Spin = 2
C -3.238768 2.949299 0.021851
C -3.925271 1.933511 0.672955
C -3.436758 0.632908 0.651684
C -2.258666 0.375736 -0.030130
C -1.556260 1.372473 -0.685523
C -2.058185 2.667701 -0.652750
H -3.621743 3.960229 0.042815
H -4.842713 2.147682 1.203762
H -3.964439 -0.160805 1.161429
H -0.629400 1.148711 -1.194546
H -1.517731 3.456995 -1.156966
I -1.537103 -1.608964 -0.083152
C 0.968490 -0.518708 0.105712
C 2.143440 -0.194684 0.085868
Si 3.894043 0.347613 0.083003
C 4.908111 -0.903956 -0.857221
H 5.959356 -0.609712 -0.879182
H 4.840953 -1.888015 -0.392049
H 4.559204 -0.994384 -1.886488
C 4.002983 2.015192 -0.746710
H 3.645094 1.962522 -1.775637
H 3.399309 2.752965 -0.216825
H 5.035084 2.370897 -0.764538
C 4.491004 0.467713 1.845446
H 3.895219 1.186137 2.409498
H 4.417094 -0.497312 2.347920
H 5.533832 0.789516 1.878724

PhICF3_02.log
Energy (E) = -580.221760597 Hartree
Enthalpy (H) = -580.105719 Hartree
Gibbs free energy (G) = -580.161895 Hartree
Charge = 0, Spin = 2
C 2.267000 2.354574 0.026295
C 1.701268 1.916839 1.216092
C 0.494909 1.227639 1.208912
C -0.136148 0.984642 -0.001859
C 0.416122 1.415878 -1.198991
C 1.623268 2.103229 -1.177826
H 3.207432 2.887795 0.037171
H 2.198378 2.106561 2.157601
H 0.056183 0.878909 2.132895
H -0.086337 1.217849 -2.135134
H 2.059031 2.439769 -2.108660
I -1.952547 -0.086818 -0.025062
C 1.511965 -1.806428 -0.119055
F 1.633461 -2.962317 -0.728635
F 1.186363 -1.965064 1.142306
F 2.600176 -1.084592 -0.244448

PhICN_02.log
Energy (E) = -335.451874969 Hartree
Enthalpy (H) = -335.344698 Hartree
Gibbs free energy (G) = -335.394142 Hartree
Charge = 0, Spin = 2
C -3.468116 0.264481 0.004972
C -2.966820 -0.907886 0.554172
C -1.601393 -1.162700 0.531109
C -0.766371 -0.226802 -0.056650
C -1.238855 0.950862 -0.608904
C -2.606731 1.189930 -0.569634
H -4.531053 0.460297 0.030479
H -3.633582 -1.627363 1.008807
H -1.202424 -2.068235 0.965485
H -0.561882 1.677631 -1.033716
H -2.993570 2.108625 -0.987919
I 1.305695 -0.634927 -0.120132
C 1.701855 1.907125 0.638432
N 1.342751 3.001448 0.488983

PhII_02.log
Energy (E) = -254.148909162 Hartree
Enthalpy (H) = -254.048804 Hartree
Gibbs free energy (G) = -254.097755 Hartree
Charge = 0, Spin = 2
C -3.634235 -1.887218 -0.050046
C -3.723794 -0.770130 -0.869277
C -2.743593 0.213425 -0.822721
C -1.685981 0.055753 0.057938
C -1.575556 -1.051048 0.883467
C -2.561990 -2.026530 0.821600
H -4.397723 -2.651459 -0.093288
H -4.554582 -0.659876 -1.552466
H -2.804304 1.081327 -1.463660
H -0.733934 -1.158789 1.553161
H -2.485583 -2.897943 1.457013
I -0.200035 1.551453 0.146153
I 2.285450 -0.814071 -0.146653

PhIN3_02.log
Energy (E) = -406.811296691 Hartree
Enthalpy (H) = -406.699148 Hartree
Gibbs free energy (G) = -406.750984 Hartree
Charge = 0, Spin = 2
C 3.022030 -1.179568 -0.203974
C 2.322382 -1.412769 0.971210
C 0.957171 -1.160096 1.037797
C 0.304672 -0.677277 -0.086673
C 0.989130 -0.443245 -1.270626
C 2.354255 -0.695467 -1.321590
H 4.085038 -1.371085 -0.249026
H 2.836329 -1.786085 1.846310
H 0.411175 -1.334679 1.953963
H 0.467660 -0.058865 -2.135842
H 2.894075 -0.510769 -2.240395
I -1.756114 -0.245353 0.014215
N 0.245051 2.816807 -0.484752
N 1.080365 2.453110 0.254615
N 1.914859 2.083762 0.989380

PhINPhth_02.log
Energy (E) = -754.800278409 Hartree
Enthalpy (H) = -754.588945 Hartree
Gibbs free energy (G) = -754.653502 Hartree
Charge = 0, Spin = 2
C -4.095279 2.289344 0.975566
C -4.383225 0.984756 1.353225
C -3.596208 -0.065007 0.896239
C -2.531279 0.223312 0.058620
C -2.220705 1.514996 -0.331396
C -3.017095 2.551306 0.139666
H -4.709021 3.103160 1.336547
H -5.217516 0.777492 2.009160
H -3.806004 -1.082124 1.195045
H -1.371824 1.705408 -0.974294
H -2.788071 3.567438 -0.150762
I -1.346822 -1.363701 -0.654330
C 2.842186 0.749440 -0.331880
C 2.982515 -0.173077 0.692378
C 4.213555 -0.458094 1.249691
C 5.314365 0.220621 0.733723
C 5.172848 1.150543 -0.298149
C 3.925591 1.433144 -0.848434
C 1.391415 0.809877 -0.690596
C 1.624782 -0.725904 0.997954
H 4.308171 -1.181764 2.047521
H 6.299823 0.024862 1.134347
H 6.051478 1.656104 -0.675408
H 3.800798 2.150137 -1.648146
N 0.721374 0.048476 0.263790
O 0.892171 1.417888 -1.610259

O 1.360744 -1.644817 1.732672

PhIOAc_02.log
Energy (E) = -471.049640993 Hartree
Enthalpy (H) = -470.896363 Hartree
Gibbs free energy (G) = -470.953232 Hartree
Charge = 0, Spin = 2
C -3.878490 1.251510 0.093624
C -3.758641 -0.029395 0.615533
C -2.541794 -0.697343 0.559047
C -1.458164 -0.068014 -0.034356
C -1.557841 1.211973 -0.557491
C -2.778916 1.870012 -0.487134
H -4.826695 1.768781 0.143197
H -4.610423 -0.513461 1.073355
H -2.442906 -1.690935 0.973625
H -0.693609 1.690260 -0.995564
H -2.866862 2.870267 -0.888617
I 0.393265 -1.061927 -0.145285
O 1.598832 1.232476 0.222664
C 2.824460 0.822626 0.187661
O 3.222268 -0.319979 0.039634
C 3.800471 1.988579 0.332356
H 4.097788 2.294590 -0.669404
H 3.348709 2.832844 0.843140
H 4.675659 1.630151 0.866561

PhIOCF3_02.log
Energy (E) = -655.411656258 Hartree
Enthalpy (H) = -655.290805 Hartree
Gibbs free energy (G) = -655.346240 Hartree
Charge = 0, Spin = 2
C -3.533934 1.437745 0.018450
C -3.258762 0.614658 1.101989
C -2.155578 -0.229145 1.077102
C -1.352269 -0.231521 -0.050807
C -1.603022 0.582308 -1.142814
C -2.706554 1.423488 -1.097426
H -4.390962 2.096256 0.045791
H -3.896175 0.630587 1.975137
H -1.925437 -0.861185 1.922752
H -0.940507 0.576703 -1.995675
H -2.914716 2.070926 -1.937893
I 0.327682 -1.494534 -0.106428
O 1.576996 0.595680 -0.956458
C 2.029713 1.249439 0.097743
F 2.621015 2.378377 -0.310572
F 1.063348 1.601902 0.969989
F 2.934206 0.558579 0.813560

PhIOCOCOPh_02.log
Energy (E) = -775.904487988 Hartree
Enthalpy (H) = -775.683306 Hartree
Gibbs free energy (G) = -775.755834 Hartree
Charge = 0, Spin = 2
C 1.945580 -1.499966 2.308631
C 1.868052 -2.193257 1.106672
C 0.747644 -2.070335 0.296082
C -0.293491 -1.251775 0.709002
C -0.234101 -0.552321 1.903941
C 0.897615 -0.680025 2.702075
H 2.822666 -1.595680 2.933920
H 2.685469 -2.826856 0.789462
H 0.689451 -2.598172 -0.645475
H -1.046236 0.094077 2.205324
H 0.956231 -0.124297 3.627742
I -1.990970 -1.048089 -0.527323
O -2.210660 2.380811 1.377496
C -2.080350 2.626472 0.251856
C 1.008480 2.152542 0.565646
O -1.980246 2.885383 -0.874908
O 1.390396 2.589532 1.596709
C 1.725067 1.330411 -0.425169
C 3.010058 0.863283 -0.144394
C 1.095364 1.008158 -1.623504
C 3.661366 0.067676 -1.071437
H 3.469013 1.127324 0.799243
C 1.750358 0.207194 -2.547005
H 0.098146 1.384902 -1.810749
C 3.029315 -0.261191 -2.267809
H 4.656767 -0.302029 -0.865601
H 1.267730 -0.051856 -3.479130
H 3.540500 -0.885705 -2.988524

PhIOMe_02.log
Energy (E) = -357.765633430 Hartree
Enthalpy (H) = -357.625461 Hartree
Gibbs free energy (G) = -357.678412 Hartree
Charge = 0, Spin = 2
C -3.459309 0.473557 -0.033004
C -3.088105 -0.721168 0.569319
C -1.760176 -1.129311 0.559023
C -0.812645 -0.327896 -0.060157
C -1.165155 0.869038 -0.666317
C -2.497792 1.264388 -0.647581
H -4.494323 0.786537 -0.023856
H -3.831301 -1.343049 1.049787
H -1.468671 -2.059750 1.025401
H -0.411860 1.486732 -1.135970
H -2.779888 2.195992 -1.119358
I 1.205603 -0.945255 -0.086177
O 1.646596 2.690019 -0.399374
C 1.420991 2.747218 0.945581
H 1.290777 3.817309 1.181117
H 0.509518 2.225128 1.258815
H 2.289169 2.414511 1.525264

PhIOOtBu_02.log
Energy (E) = -550.729355173 Hartree
Enthalpy (H) = -550.494374 Hartree
Gibbs free energy (G) = -550.556891 Hartree
Charge = 0, Spin = 2
C -0.430564 3.169738 -0.240571
C 0.412071 2.970547 0.846419
C 1.198494 1.827923 0.924161
C 1.132073 0.890213 -0.096715
C 0.305804 1.079893 -1.193053
C -0.479009 2.225223 -1.257629
H -1.043609 4.058945 -0.295740
H 0.458723 3.703235 1.640732
H 1.848537 1.666741 1.772486
H 0.248712 0.331816 -1.970152
H -1.134908 2.368800 -2.105797
I 2.260761 -0.882208 0.049900
O -1.380798 -1.660595 -1.035605
O -2.296937 -0.739305 -1.012945
C -2.955414 -0.626807 0.296802
C -3.953886 0.491387 0.083219
H -4.668736 0.227422 -0.695772
H -4.497284 0.675555 1.009402
H -3.434495 1.406640 -0.204135
C -3.623989 -1.952819 0.599872
H -4.329908 -2.213864 -0.188626
H -2.875438 -2.738777 0.682772
H -4.164561 -1.878827 1.543282
C -1.910800 -0.263128 1.333394
H -1.114968 -1.007949 1.343287
H -1.481384 0.716112 1.118591
H -2.377821 -0.232621 2.317964

PhIOPh_02.log
Energy (E) = -549.370164787 Hartree
Enthalpy (H) = -549.172913 Hartree
Gibbs free energy (G) = -549.232110 Hartree
Charge = 0, Spin = 2
C -2.694793 2.373298 0.221621
C -2.566451 1.553817 1.337114
C -1.934574 0.321279 1.232590
C -1.436279 -0.078332 0.000778
C -1.562831 0.723083 -1.121971
C -2.194249 1.955509 -1.003860
H -3.182407 3.334480 0.309030
H -2.957340 1.871896 2.294299
H -1.824719 -0.315366 2.099266
H -1.146388 0.413009 -2.069224
H -2.276007 2.591935 -1.873839
I -0.392552 -1.904030 -0.148423
O 1.318656 1.970014 -1.855237
C 1.789253 1.480629 -0.808159
C 2.927288 0.587653 -0.840919
C 1.214098 1.774158 0.486964
C 3.424719 0.043952 0.312211
H 3.352123 0.373955 -1.811921
C 1.728484 1.210711 1.624299
H 0.356425 2.434494 0.505930
C 2.830781 0.345424 1.548805
H 4.273322 -0.625891 0.278768
H 1.282139 1.424352 2.586459
H 3.226189 -0.096452 2.452731

F_02.log
Energy (E) = -99.6796169388 Hartree
Enthalpy (H) = -99.677256 Hartree
Gibbs free energy (G) = -99.694430 Hartree
Charge = 0, Spin = 2
F 0.000000 0.000000 0.000000

PhIBr_F.log
Energy (E) = -2917.07029857 Hartree
Enthalpy (H) = -2916.966577 Hartree
Gibbs free energy (G) = -2917.014716 Hartree
Charge = 0, Spin = 1
C -3.658020 -1.273675 -0.038978
C -3.521047 0.036076 -0.477981
C -2.282261 0.665201 -0.446424
C -1.209018 -0.061320 0.029852
C -1.304643 -1.362579 0.474431
C -2.555509 -1.968162 0.438299
H -4.626556 -1.753383 -0.066867
H -4.377901 0.580286 -0.849680
H -2.163540 1.691273 -0.757714
H -0.440766 -1.902051 0.833679
H -2.658451 -2.985876 0.787637
I 0.668198 0.950229 0.072305
F -0.467037 2.656669 0.217825
Br 2.006829 -1.317590 -0.160424

PhICCTMS_F.log
Energy (E) = -827.644727655 Hartree
Enthalpy (H) = -827.412232 Hartree
Gibbs free energy (G) = -827.482450 Hartree

Charge = 0, Spin = 1
C -3.030506 3.104212 0.021089
C -3.821057 1.987322 0.252683
C -3.269684 0.710853 0.236127
C -1.916951 0.600790 -0.017702
C -1.098743 1.687900 -0.249217
C -1.674390 2.953409 -0.231188
H -3.471019 4.091696 0.035016
H -4.878446 2.099020 0.448978
H -3.862691 -0.178096 0.391611
H -0.041918 1.569824 -0.439633
H -1.052250 3.818164 -0.416346
I -1.087100 -1.374902 -0.028876
F -3.124729 -1.994503 -0.047806
C 0.849794 -0.567696 0.013807
C 2.019243 -0.240120 0.022168
Si 3.777195 0.311109 0.038242
C 4.666429 -0.520095 -1.370800
H 5.713203 -0.211706 -1.397581
H 4.636964 -1.605176 -1.267113
H 4.212235 -0.260555 -2.327515
C 3.776098 2.162438 -0.177842
H 3.312818 2.444943 -1.124052
H 3.225911 2.649120 0.628418
H 4.796054 2.551069 -0.172781
C 4.531947 -0.164698 1.672328
H 3.998113 0.302765 2.500228
H 4.500752 -1.244839 1.818383
H 5.575334 0.152701 1.718974

PhICF3_F.log
Energy (E) = -679.992211108 Hartree
Enthalpy (H) = -679.872599 Hartree
Gibbs free energy (G) = -679.925154 Hartree
Charge = 0, Spin = 1
C -3.677732 1.037308 0.057104
C -3.428861 -0.225321 0.579008
C -2.153179 -0.772466 0.524956
C -1.154006 -0.017495 -0.057106
C -1.367204 1.237141 -0.593660
C -2.652275 1.763453 -0.531329
H -4.675016 1.452646 0.101468
H -4.228790 -0.796265 1.029261
H -1.940822 -1.767645 0.883921
H -0.574937 1.799291 -1.063304
H -2.844033 2.740926 -0.951198
I 0.785090 -0.877470 -0.104745
F -0.315461 -2.684375 -0.241904
C 1.683628 1.148939 0.177781
F 2.941230 0.918733 0.573939
F 1.083688 1.879606 1.114235
F 1.751833 1.891325 -0.933957

PhICN_F.log
Energy (E) = -435.261034573 Hartree
Enthalpy (H) = -435.149553 Hartree
Gibbs free energy (G) = -435.197795 Hartree
Charge = 0, Spin = 1
C -3.591362 0.306397 0.020705
C -3.030896 -0.927276 0.320233
C -1.651677 -1.101369 0.303420
C -0.875883 -0.006905 -0.020039
C -1.393416 1.235946 -0.322541
C -2.775978 1.381736 -0.301856
H -4.665300 0.429896 0.036296
H -3.662522 -1.767923 0.570891
H -1.195378 -2.057585 0.507204
H -0.763359 2.077514 -0.568581
H -3.205845 2.344163 -0.540981
I 1.241310 -0.318074 -0.026962
F 0.758415 -2.329719 -0.206856
C 1.480571 1.813137 0.196347
N 1.701297 2.941336 0.301180

PhII_F.log
Energy (E) = -353.907270911 Hartree
Enthalpy (H) = -353.803737 Hartree
Gibbs free energy (G) = -353.853257 Hartree
Charge = 0, Spin = 1
C -3.533632 -2.064555 -0.050000
C -3.721041 -0.796352 -0.582478
C -2.697035 0.142120 -0.544353
C -1.501534 -0.241037 0.029498
C -1.278240 -1.487635 0.574111
C -2.319795 -2.406787 0.529383
H -4.336777 -2.787500 -0.083992
H -4.666502 -0.525808 -1.031155
H -2.836487 1.141323 -0.926632
H -0.329299 -1.753959 1.017091
H -2.172927 -3.390607 0.952161
I 0.018402 1.249804 0.090352
F -1.545587 2.577411 0.190257
I 2.218578 -0.773477 -0.116329

PhIN3_F.log
Energy (E) = -506.571505702 Hartree
Enthalpy (H) = -506.454179 Hartree
Gibbs free energy (G) = -506.505356 Hartree
Charge = 0, Spin = 1
C -3.614253 0.524426 -0.006173
C -3.154163 -0.635220 -0.614934
C -1.805508 -0.969421 -0.571752
C -0.953900 -0.109613 0.092738
C -1.374739 1.050432 0.711283
C -2.728457 1.361736 0.657303
H -4.665439 0.774294 -0.045920
H -3.842423 -1.290703 -1.130048
H -1.431110 -1.878289 -1.016917
H -0.677625 1.692353 1.229855
H -3.083141 2.261408 1.140326
I 1.115695 -0.612942 0.143759
F 0.469393 -2.526061 -0.270292
N 1.599371 1.451914 0.590495
N 1.524117 2.207827 -0.371367
N 1.466436 2.958456 -1.215513

PhINPhth_F.log
Energy (E) = -854.603194342 Hartree
Enthalpy (H) = -854.387128 Hartree
Gibbs free energy (G) = -854.451487 Hartree
Charge = 0, Spin = 1
C -2.973704 3.231756 0.162263
C -3.622101 2.199192 0.827024
C -3.145389 0.897339 0.738352
C -2.012956 0.679490 -0.020451
C -1.349277 1.677577 -0.704524
C -1.847035 2.971673 -0.605939
H -3.353543 4.241449 0.235556
H -4.503573 2.400480 1.419495
H -3.650053 0.074926 1.221765
H -0.492022 1.460963 -1.325542
H -1.353875 3.770996 -1.141177
I -1.291320 -1.302052 -0.127720
F -3.288548 -1.795727 -0.106763
C 2.840006 0.128794 -0.616194
C 2.790862 0.025655 0.764406
C 3.893518 0.291103 1.551217
C 5.064082 0.668692 0.897092
C 5.113259 0.773044 -0.492890
C 3.993174 0.503893 -1.276059
C 1.491511 -0.213135 -1.151747
C 1.413097 -0.398057 1.146229
H 3.840783 0.206668 2.627848
H 5.952868 0.885420 1.473883
H 6.039202 1.068376 -0.967222
H 4.016303 0.581188 -2.354327
N 0.700880 -0.520646 -0.049437
O 1.120749 -0.208534 -2.302527
O 0.971792 -0.603684 2.249171

PhIOAc_F.log
Energy (E) = -570.842741012 Hartree
Enthalpy (H) = -570.684757 Hartree
Gibbs free energy (G) = -570.740005 Hartree
Charge = 0, Spin = 1
C -3.740774 1.459323 0.066642
C -3.633068 0.154511 0.527413
C -2.411482 -0.506302 0.489587
C -1.319495 0.172778 -0.016912
C -1.393452 1.471678 -0.484632
C -2.625426 2.113055 -0.438554
H -4.695257 1.966414 0.098609
H -4.499519 -0.359206 0.919851
H -2.317400 -1.529386 0.820365
H -0.520951 1.977481 -0.865785
H -2.706418 3.127545 -0.803419
I 0.514920 -0.879204 -0.074464
F -0.642338 -2.555918 -0.201942
O 1.368718 1.087351 0.108818
C 2.676619 0.972668 0.137894
O 3.238687 -0.106004 0.057166
C 3.396261 2.285337 0.275971
H 3.157338 2.919716 -0.576056
H 3.051940 2.796670 1.172917
H 4.466187 2.112768 0.325292

PhIOCF3_F.log
Energy (E) = -755.208008988 Hartree
Enthalpy (H) = -755.082793 Hartree
Gibbs free energy (G) = -755.137797 Hartree
Charge = 0, Spin = 1
C -3.476298 1.809149 0.073698
C -3.522900 0.555560 0.666583
C -2.433763 -0.304765 0.585124
C -1.321088 0.138070 -0.100351
C -1.234764 1.376149 -0.703010
C -2.338327 2.215532 -0.609253
H -4.328515 2.470807 0.143012
H -4.406821 0.235469 1.200113
H -2.460449 -1.288185 1.027854
H -0.344233 1.686137 -1.226879
H -2.298642 3.190101 -1.075008
I 0.334103 -1.193607 -0.221891
F -0.967785 -2.655834 0.273360
O 1.541869 0.468080 -0.790017
C 2.180184 1.090199 0.180221
F 2.888152 2.111246 -0.296820
F 1.344203 1.586963 1.118474
F 3.032980 0.284607 0.844229

PhIOCOBn_F.log
Energy (E) = -801.677775844 Hartree
Enthalpy (H) = -801.433467 Hartree

Gibbs free energy (G) = -801.501568 Hartree
Charge = 0, Spin = 1
C -3.539158 2.987290 -0.260813
C -3.785424 2.154867 0.822199
C -3.116612 0.943172 0.944364
C -2.207770 0.599803 -0.038358
C -1.940907 1.405133 -1.130016
C -2.622285 2.611849 -1.233082
H -4.064705 3.928114 -0.349324
H -4.500367 2.442459 1.580329
H -3.316028 0.273480 1.767027
H -1.221141 1.110765 -1.877041
H -2.433033 3.254607 -2.081580
I -1.229251 -1.262066 0.174578
F -2.903493 -1.838301 1.187879
O 0.357225 -0.271351 -0.893993
C 1.359772 -1.106495 -1.021233
O 1.313601 -2.259216 -0.628989
C 2.590372 -0.488833 -1.649485
H 3.187451 -1.294273 -2.070986
H 2.281489 0.193428 -2.440018
C 3.365394 0.252854 -0.587510
C 4.382757 -0.385594 0.114017
C 3.039952 1.567283 -0.265680
C 5.074743 0.283575 1.114782
H 4.627514 -1.413014 -0.126129
C 3.729801 2.238258 0.733933
H 2.236462 2.058177 -0.801571
C 4.750694 1.597582 1.425549
H 5.866822 -0.221347 1.651288
H 3.471839 3.261279 0.973200
H 5.290855 2.120609 2.203112

PhIOCOCCl3_F.log
Energy (E) = -1949.37536494 Hartree
Enthalpy (H) = -1949.242323 Hartree
Gibbs free energy (G) = -1949.307268 Hartree
Charge = 0, Spin = 1
C -3.815748 2.736543 0.022900
C -4.339016 1.538053 0.487752
C -3.567934 0.382301 0.473915
C -2.279521 0.472569 -0.015675
C -1.724964 1.647158 -0.487648
C -2.516468 2.789256 -0.462765
H -4.422222 3.631469 0.037429
H -5.350548 1.493119 0.866045
H -3.968288 -0.561222 0.812102
H -0.710075 1.686592 -0.852275
H -2.107395 3.720544 -0.828469
I -1.151183 -1.319059 -0.045879
F -2.921502 -2.279175 -0.179599
O 0.527831 0.042367 0.150542
C 1.646602 -0.591267 0.000441
O 1.776052 -1.781759 -0.161390
C 2.860241 0.374601 0.050845
Cl 4.349414 -0.489353 -0.227979
Cl 2.887160 1.133791 1.641905
Cl 2.637222 1.606860 -1.197376

PhIOCOCF2H_F.log
Energy (E) = -769.241329884 Hartree
Enthalpy (H) = -769.096285 Hartree
Gibbs free energy (G) = -769.156346 Hartree
Charge = 0, Spin = 1
C -3.435983 2.430083 0.049568
C -3.779120 1.171786 0.524258
C -2.856049 0.133781 0.495514
C -1.601685 0.399381 -0.018722
C -1.224867 1.638557 -0.500549
C -2.165902 2.660535 -0.460635
H -4.160054 3.232580 0.075910
H -4.767326 0.988310 0.921996
H -3.115212 -0.855301 0.840986
H -0.232588 1.816191 -0.883185
H -1.896227 3.638508 -0.833811
I -0.237065 -1.219449 -0.064063
F -1.869070 -2.413886 -0.151622
O 1.249928 0.356904 0.071103
C 2.437480 -0.158372 0.034332
O 2.694153 -1.343653 -0.042814
C 3.556911 0.888714 0.094282
F 3.399487 1.771626 -0.913717
F 3.477017 1.565311 1.257034
H 4.531613 0.410247 0.013576

PhIOCOCF3_F.log
Energy (E) = -868.459114702 Hartree
Enthalpy (H) = -868.321777 Hartree
Gibbs free energy (G) = -868.383293 Hartree
Charge = 0, Spin = 1
C -3.607798 2.501144 0.054200
C -3.971865 1.261813 0.562229
C -3.077489 0.199116 0.530677
C -1.830649 0.422459 -0.020335
C -1.433699 1.640866 -0.536854
C -2.345965 2.688320 -0.493085
H -4.309681 3.322963 0.083119
H -4.953926 1.112709 0.988384
H -3.353858 -0.775769 0.902353
H -0.448056 1.783442 -0.950222
H -2.061255 3.651338 -0.892901
I -0.512359 -1.232369 -0.070187
F -2.170053 -2.380061 -0.148402
O 1.024433 0.311477 0.058108
C 2.194065 -0.240836 0.035589
O 2.433109 -1.424516 -0.031651
C 3.322513 0.815109 0.102955
F 4.512829 0.244559 0.044029
F 3.210922 1.670906 -0.914323
F 3.238167 1.508734 1.237057

PhIOCOCH2CF3_F.log
Energy (E) = -907.749905809 Hartree
Enthalpy (H) = -907.582874 Hartree
Gibbs free energy (G) = -907.646931 Hartree
Charge = 0, Spin = 1
C -2.711309 3.234343 0.054262
C -3.526352 2.163672 0.394088
C -3.028924 0.866554 0.370305
C -1.710549 0.685610 -0.001009
C -0.869990 1.727573 -0.346704
C -1.391467 3.015575 -0.315364
H -3.105567 4.240921 0.075540
H -4.555197 2.329235 0.681434
H -3.656591 0.021903 0.610127
H 0.159171 1.558424 -0.623349
H -0.754293 3.846390 -0.584117
I -1.005525 -1.312907 -0.042581
F -2.938272 -1.843172 -0.356417
O 0.894326 -0.367125 0.340795
C 1.863694 -1.231919 0.254897
O 1.702599 -2.423207 0.074640
C 3.246491 -0.630804 0.419059
H 3.536579 -0.691981 1.467292
H 3.948743 -1.220512 -0.164189
C 3.355880 0.812379 -0.010530
F 2.726405 1.651802 0.813230
F 4.642307 1.183331 -0.044640
F 2.856200 1.004092 -1.239222

PhIOCOCH2ptol_F.log
Energy (E) = -840.954579144 Hartree
Enthalpy (H) = -840.681215 Hartree
Gibbs free energy (G) = -840.754251 Hartree
Charge = 0, Spin = 1
C 3.345496 3.276170 0.545132
C 3.627453 2.635829 -0.653577
C 3.129137 1.363571 -0.905492
C 2.351525 0.764701 0.067381
C 2.054069 1.374317 1.272130
C 2.564048 2.646092 1.503908
H 3.738044 4.265803 0.734161
H 4.237768 3.121900 -1.401827
H 3.360716 0.842335 -1.821862
H 1.440059 0.882008 2.009209
H 2.348383 3.138899 2.441742
I 1.630430 -1.178126 -0.349955
F 3.294362 -1.363499 -1.517213
O 0.003608 -0.580942 0.927153
C -0.871299 -1.557010 0.974419
O -0.698749 -2.623944 0.411880
C -2.123103 -1.212651 1.751962
H -2.607309 -2.145782 2.030343
H -1.837692 -0.666340 2.650428
C -3.036681 -0.371084 0.894367
C -4.085783 -0.953138 0.194327
C -2.817942 0.997363 0.754272
C -4.906756 -0.182242 -0.619032
H -4.257650 -2.018904 0.283594
C -3.638424 1.762583 -0.058107
H -1.990308 1.456596 1.281626
C -4.699299 1.185845 -0.755746
H -5.721150 -0.651448 -1.158019
H -3.455762 2.826324 -0.156791
C -5.600103 2.027615 -1.615054
H -6.193944 1.409045 -2.286504
H -6.290427 2.611629 -1.003649
H -5.025650 2.731421 -2.217500

PhIOCOCOPh_F.log
Energy (E) = -875.646976437 Hartree
Enthalpy (H) = -875.420741 Hartree
Gibbs free energy (G) = -875.489921 Hartree
Charge = 0, Spin = 1
C -4.354014 2.663092 0.606280
C -4.726626 1.403760 1.055460
C -3.902423 0.307086 0.835426
C -2.715108 0.516479 0.160983
C -2.311774 1.754789 -0.301994
C -3.154031 2.835805 -0.069695
H -5.001117 3.511494 0.781178
H -5.661060 1.265008 1.580915
H -4.188815 -0.682556 1.157119
H -1.372104 1.886765 -0.815251
H -2.862783 3.814431 -0.424607
I -1.503779 -1.187611 -0.177862
F -3.230443 -2.253704 -0.103014
O 0.095561 0.269397 -0.141500
C 1.213816 -0.281712 -0.495401
C 2.351899 0.735711 -0.689053
O 1.362216 -1.469690 -0.722090
O 2.115749 1.724283 -1.344955
C 3.681587 0.444672 -0.105678

C 4.709005 1.355501 -0.358527
C 3.919942 -0.667393 0.700228
C 5.964534 1.153431 0.185042
H 4.493975 2.212331 -0.982357
C 5.179870 -0.864122 1.247893
H 3.130601 -1.381333 0.884195
C 6.199713 0.042011 0.990005
H 6.761057 1.857524 -0.012812
H 5.366532 -1.727150 1.871892
H 7.181450 -0.116382 1.416087

PhIOCOiPr_F.log
Energy (E) = -649.391075642 Hartree
Enthalpy (H) = -649.173628 Hartree
Gibbs free energy (G) = -649.236109
Hartree
Charge = 0, Spin = 1
C -3.503682 2.372247 0.154826
C -3.781709 1.115868 0.674794
C -2.837212 0.099406 0.601648
C -1.624778 0.378873 0.000094
C -1.316863 1.619329 -0.528331
C -2.277062 2.620603 -0.445517
H -4.244265 3.157839 0.214705
H -4.736339 0.917089 1.141516
H -3.048419 -0.889648 0.978693
H -0.358234 1.810412 -0.984416
H -2.058522 3.596341 -0.856713
I -0.224821 -1.205096 -0.106197
F -1.855800 -2.433031 -0.057215
O 1.206938 0.396119 -0.092789
C 2.413706 -0.111906 -0.164069
O 2.612168 -1.314275 -0.231239
C 3.535972 0.902956 -0.100299
H 4.320311 0.510999 -0.748875
C 3.110860 2.291398 -0.552379
H 2.349745 2.690302 0.118529
H 3.967142 2.966025 -0.540004
H 2.698398 2.278678 -1.560985
C 4.053444 0.922778 1.339289
H 4.365198 -0.070970 1.656428
H 4.903018 1.600359 1.423143
H 3.270769 1.275893 2.013244

PhIOCOPh_F.log
Energy (E) = -762.408663313 Hartree
Enthalpy (H) = -762.193985 Hartree
Gibbs free energy (G) = -762.256620
Hartree
Charge = 0, Spin = 1
C -3.554639 2.955768 0.011020
C -4.092397 1.813756 0.588260
C -3.383899 0.618669 0.574280
C -2.139386 0.607177 -0.026225
C -1.576951 1.725991 -0.612478
C -2.303561 2.910181 -0.588947
H -4.112766 3.881763 0.024893
H -5.067676 1.844741 1.053530
H -3.799723 -0.284218 0.995087
H -0.601793 1.684054 -1.071716
H -1.885499 3.795983 -1.046608
I -1.105825 -1.236329 -0.047200
F -2.960188 -2.083870 -0.052727
O 0.629099 0.032313 0.018455
C 1.702407 -0.721888 -0.004730
O 1.640716 -1.941223 -0.064837
C 2.991198 0.021114 0.044450
C 3.021131 1.407606 0.158581
C 4.177221 -0.703504 -0.023038
C 4.239530 2.068616 0.203036
H 2.088672 1.951447 0.217741
C 5.393653 -0.040366 0.019489
H 4.120006 -1.779869 -0.108629
C 5.424276 1.345163 0.132392
H 4.267835 3.145890 0.293868
H 6.317328 -0.600204 -0.034177
H 6.373997 1.862237 0.166670

PhIOCOtBu_F.log
Energy (E) = -688.668599503 Hartree
Enthalpy (H) = -688.422336 Hartree
Gibbs free energy (G) = -688.487002
Hartree
Charge = 0, Spin = 1
C -3.661021 2.454874 0.056816
C -3.997830 1.211161 0.572752
C -3.084621 0.164601 0.537821
C -1.843257 0.402229 -0.020994
C -1.477434 1.628439 -0.545150
C -2.406895 2.660524 -0.501644
H -4.377648 3.264038 0.086584
H -4.974383 1.046110 1.006041
H -3.340787 -0.815390 0.910768
H -0.497913 1.783916 -0.969630
H -2.143506 3.626146 -0.910389
I -0.492109 -1.223551 -0.074566
F -2.164270 -2.394787 -0.130882
O 0.985330 0.329967 0.044194
C 2.181848 -0.209410 0.008345
O 2.345982 -1.415132 -0.082049
C 3.315659 0.801037 0.104891
C 4.646642 0.086238 -0.075579
H 4.703164 -0.395008 -1.051532
H 5.459673 0.809848 0.007094
H 4.780838 -0.683518 0.682960
C 3.130548 1.869483 -0.972533
H 3.951741 2.585919 -0.912289
H 3.135436 1.429813 -1.971018
H 2.191888 2.404676 -0.830520
C 3.247238 1.456660 1.486074
H 3.368692 0.716916 2.278558
H 4.052826 2.187706 1.576079
H 2.294447 1.966412 1.625302

PhIOH_F.log
Energy (E) = -418.287055787 Hartree
Enthalpy (H) = -418.170455 Hartree
Gibbs free energy (G) = -418.215945
Hartree
Charge = 0, Spin = 1
C 3.550435 -0.045049 0.006608
C 2.873243 1.154421 0.179650
C 1.483434 1.189258 0.174735
C 0.811430 -0.004034 -0.002195
C 1.451029 -1.214389 -0.180240
C 2.841208 -1.224173 -0.175505
H 4.631710 -0.060221 0.009749
H 3.422716 2.075158 0.318811
H 0.935665 2.112270 0.287795
H 0.880514 -2.120272 -0.324062
H 3.364160 -2.159491 -0.319582
I -1.312094 0.017318 -0.010370
F -1.111838 2.068530 -0.171288
O -1.311275 -2.028050 0.130665
H -1.261716 -2.293853 1.054848

PhIOMe_F.log
Energy (E) = -457.547012174 Hartree
Enthalpy (H) = -457.400741 Hartree
Gibbs free energy (G) = -457.449841
Hartree
Charge = 0, Spin = 1
C -3.610456 0.253764 0.016896
C -3.046866 -0.934666 0.461111
C -1.669528 -1.120626 0.424896
C -0.893929 -0.088052 -0.063546
C -1.420112 1.106346 -0.514934
C -2.799829 1.269273 -0.471913
H -4.683109 0.387287 0.047766
H -3.676493 -1.728356 0.838777
H -1.209889 -2.043508 0.744035
H -0.771307 1.882681 -0.894778
H -3.235398 2.193232 -0.826297
I 1.212258 -0.338114 -0.125580
F 0.789108 -2.355118 0.075521
O 1.406250 1.691800 -0.298002
C 1.300313 2.374524 0.927148
H 1.472240 3.435023 0.741069
H 0.304320 2.261599 1.374088
H 2.040418 2.030387 1.657455

PhIOOtBu_F.log
Energy (E) = -650.478458708 Hartree
Enthalpy (H) = -650.239920 Hartree
Gibbs free energy (G) = -650.301759
Hartree
Charge = 0, Spin = 1
C -3.558647 2.288002 -0.142792
C -3.818242 1.023884 -0.653022
C -2.867559 0.013942 -0.556354
C -1.670925 0.315241 0.062826
C -1.374722 1.560962 0.578886
C -2.341991 2.553446 0.469964
H -4.304426 3.066979 -0.222988
H -4.764160 0.811939 -1.131904
H -3.051882 -0.977656 -0.939715
H -0.423139 1.758511 1.047199
H -2.134177 3.536753 0.868544
I -0.205402 -1.221169 0.211183
F -1.678601 -2.485516 -0.487119
O 1.127150 0.178173 0.958424
O 1.606306 0.977004 -0.117531
C 3.010480 0.742390 -0.280409
C 3.371309 1.671978 -1.425858
H 3.135420 2.702410 -1.161981
H 4.438334 1.598125 -1.634748
H 2.816663 1.402293 -2.324335
C 3.749776 1.122051 0.991388
H 3.542885 2.161676 1.245658
H 3.432139 0.488530 1.817569
H 4.824245 1.001648 0.848250
C 3.266973 -0.708286 -0.658753
H 3.023490 -1.373786 0.170009
H 2.671264 -0.982441 -1.530832
H 4.320680 -0.846470 -0.901767

PhIOPh_F.log
Energy (E) = -649.123143215 Hartree
Enthalpy (H) = -648.921070 Hartree
Gibbs free energy (G) = -648.978579
Hartree
Charge = 0, Spin = 1
C -2.801288 2.932086 -0.188848
C -3.347795 1.887235 0.543028
C -2.721364 0.646333 0.580869
C -1.546920 0.498633 -0.130003
C -0.974172 1.514474 -0.869365
C -1.620169 2.744874 -0.893487
H -3.297007 3.892788 -0.211726
H -4.268087 2.028667 1.092503

H -3.135836 -0.181333 1.135324
H -0.056857 1.354835 -1.415849
H -1.191501 3.554184 -1.467785
I -0.568187 -1.388959 -0.079368
F -2.262495 -2.072031 0.854327
O 1.047100 -0.551350 -1.075772
C 2.075101 -0.000300 -0.381469
C 3.354512 -0.128277 -0.923173
C 1.904752 0.710086 0.805450
C 4.441607 0.447914 -0.287059
H 3.469101 -0.682836 -1.844366
C 3.003116 1.278521 1.438855
H 0.916316 0.831211 1.230254
C 4.274822 1.153442 0.899841
H 5.427900 0.341038 -0.719165
H 2.856704 1.827211 2.359793
H 5.125630 1.598043 1.396899

PhIOTs_F.log
Energy (E) = -1236.84569840 Hartree
Enthalpy (H) = -1236.600022 Hartree
Gibbs free energy (G) = -1236.668119 Hartree
Charge = 0, Spin = 1
C 1.797253 -2.991492 0.762075
C 0.906163 -3.320971 -0.250397
C -0.239503 -2.560662 -0.453931
C -0.449068 -1.482519 0.382325
C 0.407730 -1.128445 1.404686
C 1.547386 -1.902453 1.586870
H 2.685507 -3.589707 0.913975
H 1.095811 -4.171440 -0.890208
H -0.951564 -2.811513 -1.225329
H 0.212245 -0.269988 2.029161
H 2.238166 -1.644763 2.377634
I -2.166514 -0.299577 0.008143
F -2.985354 -1.988968 -0.734073
O -1.114353 1.337330 0.911900
S -0.384034 2.387802 0.015186
O -1.075453 2.467456 -1.246771
O -0.182515 3.561528 0.811971
C 1.170805 1.607214 -0.253459
C 1.313460 0.726405 -1.317448
C 2.195627 1.805525 0.658085
C 2.490766 0.008177 -1.442810
H 0.506547 0.616271 -2.030319
C 3.372652 1.084832 0.513237
H 2.060455 2.516636 1.461657
C 3.528192 0.167085 -0.523400
H 2.610564 -0.689834 -2.262576
H 4.183018 1.232077 1.216514
C 4.774599 -0.660792 -0.642018
H 4.574363 -1.691083 -0.337325
H 5.133155 -0.691930 -1.670506
H 5.571897 -0.273211 -0.010244

PhIBr_Cl.log
Energy (E) = -3277.40829598 Hartree
Enthalpy (H) = -3277.305064 Hartree
Gibbs free energy (G) = -3277.355026 Hartree
Charge = 0, Spin = 1
C -3.363844 -1.917015 -0.017715
C -3.319250 -0.751207 -0.770326
C -2.183788 0.049648 -0.758206
C -1.119903 -0.363751 0.016661
C -1.124815 -1.515048 0.776415
C -2.273596 -2.296390 0.753546
H -4.252655 -2.532328 -0.031737
H -4.166990 -0.455928 -1.372292
H -2.144734 0.970664 -1.320867
H -0.263683 -1.809419 1.358499
H -2.308760 -3.201400 1.343396
I 0.627085 0.856255 0.041554
Cl -0.886174 2.887810 0.230831
Br 2.150784 -1.333808 -0.174449

PhICCTMS_Cl.log
Energy (E) = -1187.97956984 Hartree
Enthalpy (H) = -1187.747441 Hartree
Gibbs free energy (G) = -1187.818249 Hartree
Charge = 0, Spin = 1
C 2.381739 3.510233 -0.024529
C 3.180175 2.548173 -0.627554
C 2.808861 1.208205 -0.608781
C 1.624969 0.885783 0.018347
C 0.801578 1.811394 0.625991
C 1.198963 3.143219 0.602566
H 2.683411 4.548423 -0.040163
H 4.101923 2.831037 -1.116291
H 3.435991 0.441512 -1.040936
H -0.123563 1.518801 1.101396
H 0.578211 3.888196 1.080595
I 1.017599 -1.173895 0.039748
Cl 3.608842 -1.819187 0.099616
C -0.978136 -0.547835 -0.026578
C -2.170131 -0.319999 -0.045345
Si -3.973764 0.069093 -0.075573
C -4.843293 -1.142930 1.037925
H -5.916613 -0.944848 1.052709
H -4.694247 -2.167093 0.694718
H -4.472066 -1.072343 2.060703
C -4.174723 1.813144 0.547825
H -3.804810 1.909030 1.569314
H -3.627450 2.517709 -0.079470
H -5.226975 2.103081 0.541911
C -4.575574 -0.084417 -1.829975
H -4.045092 0.604872 -2.487484
H -4.424062 -1.095590 -2.208756
H -5.641568 0.142726 -1.889683

PhICF3_Cl.log
Energy (E) = -1040.32949723 Hartree
Enthalpy (H) = -1040.210355 Hartree
Gibbs free energy (G) = -1040.265361 Hartree
Charge = 0, Spin = 1
C -3.491513 1.544860 0.036429
C -3.268057 0.448740 0.860352
C -2.049922 -0.217111 0.829101
C -1.085734 0.255410 -0.037539
C -1.273236 1.334802 -0.877337
C -2.501484 1.984645 -0.830545
H -4.445228 2.053187 0.065006
H -4.042912 0.101559 1.529107
H -1.871280 -1.094755 1.432973
H -0.502451 1.665612 -1.557159
H -2.678824 2.829124 -1.481500
I 0.786990 -0.746592 -0.069931
Cl -0.640549 -2.982001 -0.218130
C 1.766700 1.263727 0.178816
F 3.010218 1.022250 0.594393
F 1.158271 2.021744 1.082220
F 1.846955 1.958030 -0.957670

PhICN_Cl.log
Energy (E) = -795.594252820 Hartree
Enthalpy (H) = -795.483331 Hartree
Gibbs free energy (G) = -795.533266 Hartree
Charge = 0, Spin = 1
C -3.663160 -0.228568 0.001946
C -2.891664 -1.191655 0.637913
C -1.504999 -1.095051 0.639904
C -0.945382 -0.012212 -0.002795
C -1.673640 0.967477 -0.645199
C -3.058173 0.844204 -0.638176
H -4.740717 -0.315009 0.003767
H -3.361567 -2.026686 1.137911
H -0.890294 -1.851075 1.105734
H -1.198577 1.806111 -1.132952
H -3.656003 1.592791 -1.138441
I 1.190442 0.154816 0.008617
Cl 1.310764 -2.401537 -0.273198
C 0.876969 2.283175 0.267827
N 0.804431 3.430074 0.377587

PhII_Cl.log
Energy (E) = -714.250039982 Hartree
Enthalpy (H) = -714.146936 Hartree
Gibbs free energy (G) = -714.198234 Hartree
Charge = 0, Spin = 1
C -2.739255 -2.971913 -0.025739
C -3.085261 -1.911024 -0.851977
C -2.341838 -0.737591 -0.835070
C -1.258428 -0.680013 0.017461
C -0.884959 -1.710880 0.855142
C -1.646991 -2.871978 0.825646
H -3.324632 -3.880577 -0.043997
H -3.936163 -1.987947 -1.513919
H -2.614214 0.103695 -1.455456
H -0.025547 -1.628373 1.505047
H -1.380274 -3.695400 1.472862
I -0.134817 1.126106 0.058870
Cl -2.324616 2.411521 0.187145
I 2.446887 -0.458311 -0.116583

PhIN3_Cl.log
Energy (E) = -866.906883713 Hartree
Enthalpy (H) = -866.790165 Hartree
Gibbs free energy (G) = -866.843019 Hartree
Charge = 0, Spin = 1
C -3.522449 -0.987060 -0.063333
C -2.634614 -1.653877 0.770121
C -1.280390 -1.342538 0.748755
C -0.864351 -0.355320 -0.120246
C -1.717262 0.331705 -0.961430
C -3.066269 -0.000125 -0.926444
H -4.573736 -1.238127 -0.040728
H -2.988920 -2.421160 1.443937
H -0.575197 -1.870387 1.373875
H -1.349514 1.104251 -1.621492
H -3.754997 0.516790 -1.579695
I 1.196435 0.186249 -0.155273
Cl 1.779478 -2.281344 0.149407
N 0.559992 2.275611 -0.453332
N -0.100796 2.704568 0.484124
N -0.731737 3.143187 1.316225

PhINPhth_Cl.log
Energy (E) = -1214.93772289 Hartree
Enthalpy (H) = -1214.722209 Hartree
Gibbs free energy (G) = -1214.788537 Hartree
Charge = 0, Spin = 1
C -2.254043 3.606566 0.041952
C -2.845211 2.757134 0.968549

C -2.578821 1.394177 0.939792
C -1.711180 0.934822 -0.029352
C -1.108284 1.744642 -0.970888
C -1.392897 3.104452 -0.924610
H -2.470440 4.665515 0.070346
H -3.516553 3.149974 1.719041
H -3.045008 0.715280 1.638827
H -0.450738 1.334061 -1.725215
H -0.944466 3.764732 -1.653616
I -1.257470 -1.131004 -0.076797
Cl -3.767054 -1.548169 -0.059554
C 2.998214 -0.081453 -0.619733
C 2.941043 -0.103039 0.764613
C 4.059165 0.127232 1.540347
C 5.254451 0.381728 0.871216
C 5.311808 0.403582 -0.522009
C 4.175593 0.172073 -1.294223
C 1.628572 -0.355520 -1.142568
C 1.534649 -0.401146 1.163106
H 3.999929 0.108262 2.619777
H 6.156227 0.566105 1.439002
H 6.256921 0.604188 -1.007890
H 4.204942 0.186769 -2.374987
N 0.821308 -0.546723 -0.027413
O 1.259909 -0.388301 -2.293843
O 1.073531 -0.501354 2.272856

PhIOAc_Cl.log
Energy (E) = -931.177869027 Hartree
Enthalpy (H) = -931.020473 Hartree
Gibbs free energy (G) = -931.077473 Hartree
Charge = 0, Spin = 1
C -3.495563 1.987316 0.051681
C -3.435170 0.821808 0.803663
C -2.292362 0.032402 0.782161
C -1.232732 0.445014 -0.001434
C -1.260192 1.600209 -0.759442
C -2.413049 2.374665 -0.726577
H -4.389708 2.594902 0.070974
H -4.276739 0.519001 1.410673
H -2.242585 -0.887194 1.346999
H -0.407225 1.898992 -1.349214
H -2.459986 3.280272 -1.315067
I 0.517435 -0.747471 -0.046710
O 1.476072 1.203791 0.118663
C 2.769701 1.000346 0.152426
O 3.260202 -0.115373 0.090927
C 3.580343 2.262413 0.271939
H 3.374504 2.906968 -0.580805
H 3.282556 2.800454 1.169874
H 4.636221 2.016293 0.310638
Cl -0.950419 -2.786241 -0.218333

PhIOCF3_Cl.log
Energy (E) = -1115.54158456 Hartree
Enthalpy (H) = -1115.417021 Hartree
Gibbs free energy (G) = -1115.474156 Hartree
Charge = 0, Spin = 1
C -2.661270 2.833879 0.072305
C -2.883555 1.759192 0.921998
C -2.097556 0.616638 0.829902
C -1.102974 0.602961 -0.124357
C -0.845837 1.651069 -0.984109
C -1.648820 2.779753 -0.876132
H -3.278602 3.717923 0.150282
H -3.667501 1.801787 1.664819
H -2.270007 -0.230017 1.477560
H -0.042330 1.604465 -1.703001
H -1.475037 3.615496 -1.539102
I 0.140937 -1.118635 -0.277244
Cl -1.806512 -2.569730 0.214774
O 1.730907 0.261481 -0.778697
C 2.433939 0.728974 0.229284
F 3.370421 1.576590 -0.193836
F 1.676903 1.391677 1.133744
F 3.059752 -0.241962 0.927696

PhIOCOBn_Cl.log
Energy (E) = -1162.01302836 Hartree
Enthalpy (H) = -1161.769314 Hartree
Gibbs free energy (G) = -1161.839196 Hartree
Charge = 0, Spin = 1
C -3.287667 3.303046 0.043509
C -3.373235 2.463704 1.146149
C -2.771429 1.212008 1.122209
C -2.092830 0.840066 -0.021466
C -1.987495 1.652107 -1.134653
C -2.598280 2.899078 -1.091945
H -3.761297 4.274682 0.068528
H -3.908905 2.777175 2.031112
H -2.848417 0.542718 1.966772
H -1.437342 1.332534 -2.006482
H -2.534268 3.550473 -1.952310
I -1.169016 -1.064512 -0.075476
Cl -3.244946 -2.033921 0.964089
O 0.462634 0.099008 -0.948149
C 1.446848 -0.726153 -1.182865
O 1.377833 -1.924990 -0.960838
C 2.704377 -0.063531 -1.707021
H 3.188097 -0.771857 -2.377876
H 2.441453 0.839397 -2.253449
C 3.608719 0.267994 -0.544286
C 4.269264 -0.752204 0.136139
C 3.769562 1.581993 -0.120145
C 5.086667 -0.459192 1.217597
H 4.126190 -1.776926 -0.184457
C 4.589308 1.877815 0.961779
H 3.249522 2.375891 -0.642278
C 5.250317 0.857763 1.631886
H 5.597283 -1.258334 1.738058
H 4.710904 2.904525 1.280252
H 5.890230 1.086416 2.473440

PhIOCOCCl3_Cl.log
Energy (E) = -2309.71050792 Hartree
Enthalpy (H) = -2309.578064 Hartree
Gibbs free energy (G) = -2309.644407 Hartree
Charge = 0, Spin = 1
C -3.258342 3.244250 0.007389
C -3.815803 2.201779 0.735211
C -3.214269 0.949555 0.738006
C -2.058877 0.788706 0.000291
C -1.473835 1.802891 -0.733186
C -2.093755 3.046203 -0.722098
H -3.733234 4.215466 0.008938
H -4.720438 2.355562 1.306282
H -3.647671 0.127238 1.288377
H -0.557179 1.644523 -1.281384
H -1.659299 3.857520 -1.288853
I -1.129519 -1.118687 -0.012364
Cl -3.352131 -2.193188 -0.213024
O 0.681193 0.135852 0.170201
C 1.725608 -0.615165 0.060229
O 1.735427 -1.820764 -0.041936
C 3.036095 0.219840 0.054344
Cl 4.431085 -0.816432 -0.101054
Cl 3.124224 1.132282 1.559267
Cl 2.959671 1.332692 -1.318442

PhIOCOCF2H_Cl.log
Energy (E) = -1129.57529782 Hartree
Enthalpy (H) = -1129.430973 Hartree
Gibbs free energy (G) = -1129.492433 Hartree
Charge = 0, Spin = 1
C -3.154817 2.865121 -0.001497
C -3.391767 1.804982 0.863186
C -2.560702 0.692105 0.850920
C -1.506352 0.683439 -0.040351
C -1.240450 1.720931 -0.912700
C -2.084596 2.823838 -0.885015
H -3.807205 3.727076 0.012986
H -4.223332 1.837434 1.552818
H -2.744910 -0.145689 1.507660
H -0.397266 1.683019 -1.585168
H -1.901199 3.648282 -1.559546
I -0.232725 -1.007940 -0.070340
Cl -2.240714 -2.476257 0.000731
O 1.321192 0.567597 -0.127318
C 2.474788 -0.023125 -0.097131
O 2.656480 -1.222061 -0.082508
C 3.634286 0.987860 -0.102725
F 3.532658 1.773409 0.991657
F 4.806924 0.343408 -0.057390
H 3.600491 1.620545 -0.991080

PhIOCOCF3_Cl.log
Energy (E) = -1228.79444184 Hartree
Enthalpy (H) = -1228.657729 Hartree
Gibbs free energy (G) = -1228.720779 Hartree
Charge = 0, Spin = 1
C -3.022344 3.098337 0.039092
C -3.442494 2.024133 0.811917
C -2.733564 0.829724 0.794937
C -1.613275 0.757523 -0.008347
C -1.164393 1.805461 -0.788202
C -1.889393 2.990129 -0.756087
H -3.580244 4.024167 0.056812
H -4.322567 2.108585 1.433563
H -3.059471 -0.017583 1.380519
H -0.272492 1.716131 -1.389087
H -1.562360 3.826035 -1.358150
I -0.523000 -1.060032 -0.047470
Cl -2.652294 -2.318482 -0.169926
O 1.189870 0.352366 0.070435
C 2.279681 -0.337653 0.057660
O 2.376726 -1.544088 0.011782
C 3.532848 0.570703 0.102862
F 4.643337 -0.145074 0.064491
F 3.531167 1.405638 -0.937685
F 3.535494 1.299679 1.217667

PhIOCOCH2CF3_Cl.log
Energy (E) = -1268.08484957 Hartree
Enthalpy (H) = -1267.918406 Hartree
Gibbs free energy (G) = -1267.984199 Hartree
Charge = 0, Spin = 1
C -1.936301 3.671358 0.064960
C -2.839863 2.787113 0.638094
C -2.584011 1.421562 0.630885
C -1.413790 0.986858 0.042030
C -0.490379 1.837702 -0.535856
C -0.768494 3.198905 -0.518558
H -2.142730 4.732610 0.073003

H -3.748540 3.152743 1.095156
H -3.288320 0.723207 1.058839
H 0.425316 1.466511 -0.971332
H -0.063794 3.885883 -0.965485
I -1.008611 -1.098680 0.023736
Cl -3.411343 -1.547021 -0.477555
O 1.004351 -0.362884 0.468514
C 1.852637 -1.344156 0.386371
O 1.543425 -2.508840 0.219812
C 3.302125 -0.915418 0.526646
H 3.564493 -0.877691 1.583076
H 3.929260 -1.656185 0.038416
C 3.596168 0.438573 -0.074534
F 3.102340 1.448456 0.642140
F 4.919202 0.625666 -0.161780
F 3.098767 0.546004 -1.315703

PhIOCOCH2ptol_Cl.log
Energy (E) = -1201.28977497 Hartree
Enthalpy (H) = -1201.016991 Hartree
Gibbs free energy (G) = -1201.092183 Hartree
Charge = 0, Spin = 1
C 3.045395 3.515706 0.453954
C 3.135544 2.910367 -0.792236
C 2.702331 1.602394 -0.968861
C 2.183890 0.936910 0.124420
C 2.078946 1.511207 1.377046
C 2.519777 2.819237 1.533978
H 3.387831 4.532936 0.584441
H 3.544092 3.451371 -1.634133
H 2.786319 1.114559 -1.929144
H 1.656772 0.964141 2.206120
H 2.453015 3.288237 2.505704
I 1.519863 -1.057907 -0.125165
Cl 3.601070 -1.516701 -1.466059
O -0.158298 -0.315130 1.056630
C -1.003255 -1.303648 1.196406
O -0.796467 -2.420887 0.752384
C -2.280206 -0.919939 1.915656
H -2.708138 -1.827265 2.335966
H -2.039180 -0.221706 2.715799
C -3.232384 -0.285600 0.931805
C -4.197334 -1.049861 0.287132
C -3.128742 1.066248 0.615635
C -5.050723 -0.471271 -0.642928
H -4.275086 -2.106671 0.511833
C -3.982066 1.639809 -0.313531
H -2.364940 1.664373 1.098146
C -4.960356 0.881488 -0.954680
H -5.798321 -1.080291 -1.137055
H -3.889924 2.693631 -0.549217
C -5.902634 1.515963 -1.938528
H -6.346418 0.769703 -2.596180
H -6.717183 2.029447 -1.424162
H -5.390739 2.254609 -2.555107

PhIOCOCOPh_Cl.log
Energy (E) = -1235.98230581 Hartree
Enthalpy (H) = -1235.756672 Hartree
Gibbs free energy (G) = -1235.827295 Hartree
Charge = 0, Spin = 1
C -3.835787 3.122461 0.639991
C -4.165828 1.977446 1.352337
C -3.479352 0.790778 1.127825
C -2.471708 0.797238 0.184262
C -2.114179 1.917478 -0.541339
C -2.815494 3.092624 -0.301092
H -4.375950 4.041781 0.818934
H -4.958358 1.999624 2.086980
H -3.739380 -0.110767 1.663113
H -1.305750 1.889978 -1.255913
H -2.557836 3.983354 -0.856529
I -1.416459 -1.008294 -0.176863
Cl -3.573727 -2.267356 -0.063122
O 0.288785 0.386668 -0.208752
C 1.350212 -0.266150 -0.551323
C 2.565730 0.640487 -0.814954
O 1.407104 -1.472672 -0.721581
O 2.429980 1.551933 -1.597817
C 3.843319 0.332227 -0.132801
C 4.938282 1.152385 -0.409709
C 3.967993 -0.712372 0.781089
C 6.149009 0.927252 0.219859
H 4.810486 1.957824 -1.120036
C 5.183514 -0.933239 1.413303
H 3.125436 -1.358146 0.982435
C 6.271076 -0.116828 1.132852
H 6.998393 1.561106 0.005081
H 5.283041 -1.744936 2.120581
H 7.217910 -0.293330 1.625575

PhIOCOiPr_Cl.log
Energy (E) = -1009.72619316 Hartree
Enthalpy (H) = -1009.509313 Hartree
Gibbs free energy (G) = -1009.573756 Hartree
Charge = 0, Spin = 1
C -2.802876 3.094304 0.122199
C -3.179976 2.014160 0.908567
C -2.464690 0.824460 0.854505
C -1.378694 0.757476 0.004294
C -0.976210 1.814829 -0.789878
C -1.706292 2.994906 -0.723625
H -3.366365 4.015984 0.167204
H -4.033087 2.089832 1.568013
H -2.761829 -0.029014 1.446495
H -0.113406 1.731153 -1.433194
H -1.413866 3.834076 -1.339207
I -0.275189 -1.048865 -0.090167
Cl -2.455566 -2.311321 -0.107649
O 1.396957 0.345199 -0.045486
C 2.500233 -0.357565 -0.095943
O 2.497774 -1.577536 -0.156543
C 3.770748 0.464222 -0.018373
H 4.552450 -0.162248 -0.447330
C 3.656934 1.778996 -0.776709
H 2.886953 2.405245 -0.325991
H 4.605283 2.315205 -0.736649
H 3.397389 1.620679 -1.823296
C 4.075202 0.710747 1.459945
H 4.170379 -0.226130 2.007261
H 5.006894 1.267349 1.561458
H 3.274735 1.299675 1.910430

PhIOCOPh_Cl.log
Energy (E) = -1122.74385519 Hartree
Enthalpy (H) = -1122.529760 Hartree
Gibbs free energy (G) = -1122.594270 Hartree
Charge = 0, Spin = 1
C -3.020182 3.378929 0.010343
C -3.530932 2.384907 0.834155
C -2.971379 1.113484 0.827432
C -1.902330 0.879192 -0.014637
C -1.369602 1.846732 -0.845425
C -1.944783 3.111146 -0.826198
H -3.463171 4.365192 0.018571
H -4.367842 2.592183 1.486069
H -3.373546 0.326795 1.449234
H -0.525800 1.629636 -1.482839
H -1.549557 3.883221 -1.471450
I -1.037339 -1.053060 -0.040362
Cl -3.357145 -2.020721 -0.094855
O 0.798015 0.120971 0.029364
C 1.801052 -0.720166 0.015472
O 1.638468 -1.932335 -0.029559
C 3.150988 -0.092122 0.054472
C 3.301620 1.288584 0.139384
C 4.268907 -0.919355 0.006296
C 4.573331 1.840922 0.174335
H 2.419826 1.912356 0.181565
C 5.538643 -0.364520 0.039843
H 4.117864 -1.988079 -0.057196
C 5.690256 1.015123 0.123676
H 4.695808 2.913295 0.242061
H 6.409852 -1.004050 0.001616
H 6.681570 1.447659 0.150774

PhIOCOtBu_Cl.log
Energy (E) = -1049.00380563 Hartree
Enthalpy (H) = -1048.758128 Hartree
Gibbs free energy (G) = -1048.824331 Hartree
Charge = 0, Spin = 1
C -3.098267 3.046239 0.044215
C -3.479665 1.966213 0.828690
C -2.741588 0.789450 0.807245
C -1.629020 0.735565 -0.008699
C -1.221802 1.792966 -0.800310
C -1.974834 2.959877 -0.767211
H -3.679546 3.957702 0.063532
H -4.353804 2.031916 1.461104
H -3.040843 -0.064473 1.397539
H -0.338884 1.718774 -1.417252
H -1.679459 3.798941 -1.381527
I -0.491244 -1.050186 -0.054997
Cl -2.648229 -2.349268 -0.154463
O 1.150199 0.373518 0.056312
C 2.272459 -0.301618 0.033793
O 2.297375 -1.520907 -0.032847
C 3.519880 0.570158 0.106727
C 4.757611 -0.306041 -0.016368
H 4.766628 -0.839339 -0.966387
H 5.649904 0.319817 0.044357
H 4.791568 -1.046781 0.781261
C 3.475840 1.596746 -1.025252
H 4.373266 2.216495 -0.982122
H 3.446815 1.108604 -2.000646
H 2.602272 2.240979 -0.929058
C 3.514467 1.298341 1.452248
H 3.534374 0.590917 2.282428
H 4.402745 1.929023 1.521142
H 2.629704 1.926584 1.548173

PhIOH_Cl.log
Energy (E) = -778.620601641 Hartree
Enthalpy (H) = -778.504547 Hartree
Gibbs free energy (G) = -778.551951 Hartree
Charge = 0, Spin = 1
C 3.645197 0.362113 -0.014319
C 2.800465 1.393318 0.371942
C 1.422178 1.209903 0.383830
C 0.935322 -0.025989 0.013146
C 1.744195 -1.076150 -0.374060
C 3.118271 -0.866463 -0.388052
H 4.715289 0.516784 -0.026440
H 3.206253 2.351527 0.664874

H 0.755342 2.013351 0.659946
H 1.318087 -2.029666 -0.648768
H 3.771687 -1.670565 -0.696755
I -1.169490 -0.399241 0.026880
Cl -1.597663 2.113376 -0.234627
O -0.750527 -2.411808 0.192066
H -0.612973 -2.634993 1.119711

PhIOMe_Cl.log
Energy (E) = -817.880842317 Hartree
Enthalpy (H) = -817.735092 Hartree
Gibbs free energy (G) = -817.786114 Hartree
Charge = 0, Spin = 1
C -3.676640 -0.378072 -0.004867
C -2.875335 -1.338704 0.596466
C -1.490234 -1.219630 0.568983
C -0.952435 -0.119893 -0.065909
C -1.717905 0.858045 -0.670665
C -3.099891 0.715220 -0.637138
H -4.752622 -0.482445 0.017347
H -3.321309 -2.190520 1.090583
H -0.855434 -1.972649 1.012413
H -1.250531 1.708557 -1.145882
H -3.720488 1.462811 -1.111254
I 1.162907 0.155599 -0.125850
Cl 1.457541 -2.396092 -0.007019
O 0.816059 2.183645 -0.227492
C 0.459347 2.762984 1.004108
H 0.358068 3.837658 0.849670
H -0.498304 2.377401 1.375187
H 1.218448 2.597124 1.775365

PhIOOtBu_Cl.log
Energy (E) = -1010.81234865 Hartree
Enthalpy (H) = -1010.574298 Hartree
Gibbs free energy (G) = -1010.637682 Hartree
Charge = 0, Spin = 1
C -3.354601 2.732377 -0.130299
C -3.823505 1.455977 0.146035
C -2.940786 0.385464 0.232433
C -1.599133 0.641489 0.042009
C -1.092979 1.897704 -0.230172
C -1.996775 2.950043 -0.319442
H -4.048297 3.558900 -0.199811
H -4.879609 1.281305 0.296765
H -3.297173 -0.614842 0.428331
H -0.035948 2.063049 -0.370177
H -1.628070 3.941957 -0.540645
I -0.193869 -0.956595 0.208906
Cl -2.004782 -2.613307 -0.519288
O 1.200245 0.477989 0.843025
O 1.937415 0.933642 -0.278161
C 3.301342 0.505708 -0.150879
C 3.950489 1.086546 -1.394604
H 3.841683 2.170629 -1.404525
H 5.012172 0.841187 -1.401606
H 3.488814 0.677037 -2.292852
C 3.908182 1.088723 1.113616
H 3.826750 2.175555 1.098901
H 3.389053 0.709842 1.992068
H 4.961691 0.815116 1.181671
C 3.380492 -1.013040 -0.155427
H 2.924598 -1.427168 0.744580
H 2.879733 -1.416153 -1.037543
H 4.423323 -1.329630 -0.177844

PhIOPh_Cl.log
Energy (E) = -1009.45621362 Hartree
Enthalpy (H) = -1009.254730 Hartree
Gibbs free energy (G) = -1009.314566 Hartree
Charge = 0, Spin = 1
C -2.008565 3.489791 -0.135843
C -2.628029 2.578518 0.707317
C -2.262350 1.236624 0.687682
C -1.268098 0.855932 -0.188431
C -0.624745 1.733863 -1.038394
C -1.012408 3.067925 -1.005847
H -2.302135 4.530170 -0.115165
H -3.402078 2.902289 1.388944
H -2.750338 0.518557 1.329711
H 0.158070 1.393752 -1.699859
H -0.527330 3.773371 -1.665800
I -0.621784 -1.184899 -0.247840
Cl -2.832610 -1.931853 0.748268
O 1.159136 -0.525553 -1.133504
C 2.216869 -0.171149 -0.360990
C 3.489578 -0.504803 -0.825883
C 2.089606 0.535713 0.833598
C 4.613739 -0.126863 -0.110006
H 3.569613 -1.056612 -1.752377
C 3.223227 0.902131 1.547893
H 1.108973 0.811012 1.200999
C 4.489147 0.576962 1.082790
H 5.594622 -0.389815 -0.483604
H 3.110807 1.450173 2.473954
H 5.367832 0.864824 1.642846

PhIOTS_Cl.log
Energy (E) = -1597.17637075 Hartree
Enthalpy (H) = -1596.931056 Hartree
Gibbs free energy (G) = -1597.005375 Hartree
Charge = 0, Spin = 1
C -3.404175 -3.405905 0.407284
C -3.442359 -2.792094 -0.837545
C -2.870330 -1.539197 -1.018090
C -2.272347 -0.940877 0.072810
C -2.212399 -1.522412 1.324587
C -2.792229 -2.775064 1.482120
H -3.853331 -4.380319 0.540228
H -3.916236 -3.283740 -1.675387
H -2.907711 -1.045814 -1.978411
H -1.716926 -1.029659 2.147227
H -2.762878 -3.252184 2.451516
I -1.393894 0.972714 -0.169831
Cl -3.326240 1.676067 -1.530679
O 0.199635 0.037149 1.054391
S 1.407171 0.959693 1.329757
O 1.097182 2.246816 0.732372
O 1.764102 0.894822 2.717456
C 2.696013 0.217714 0.389155
C 2.747678 0.441079 -0.981399
C 3.628180 -0.587406 1.020272
C 3.747435 -0.160631 -1.723974
H 2.023222 1.097787 -1.445320
C 4.626623 -1.183040 0.260708
H 3.568908 -0.728448 2.090322
C 4.699055 -0.981210 -1.114055
H 3.801712 0.010509 -2.792232
H 5.364751 -1.811275 0.743401
C 5.785138 -1.616683 -1.933893
H 5.365349 -2.201103 -2.753191
H 6.432252 -0.858123 -2.376242
H 6.403546 -2.275618 -1.327066

PhIF_Br_02.log
Energy (E) = -2574.48790818 Hartree
Enthalpy (H) = -2574.485548 Hartree
Gibbs free energy (G) = -2574.504738 Hartree
Charge = 0, Spin = 2
Br 0.000000 0.000000 0.000000

PhIF_CCTMS_02.log
Energy (E) = -485.013591030 Hartree
Enthalpy (H) = -484.884456 Hartree
Gibbs free energy (G) = -484.929443 Hartree
Charge = 0, Spin = 2
C 2.814807 0.000145 0.000296
C 1.602841 0.000218 0.000390
Si -0.242888 -0.000004 0.000011
C -0.814964 1.092466 -1.394415
H -1.905494 1.122701 -1.432832
H -0.452137 2.112557 -1.266031
H -0.452302 0.723622 -2.354175
C -0.815701 0.661165 1.643128
H -1.906255 0.678893 1.688125
H -0.452907 0.040134 2.462521
H -0.453490 1.676920 1.803623
C -0.814873 -1.753860 -0.249151
H -1.905405 -1.802235 -0.256565
H -0.451685 -2.152737 -1.196621
H -0.452554 -2.400610 0.550316

PhIF_CF3_02.log
Energy (E) = -337.417524124 Hartree
Enthalpy (H) = -337.400615 Hartree
Gibbs free energy (G) = -337.431653 Hartree
Charge = 0, Spin = 2
C -0.000037 -0.000168 0.320601
F 1.146978 -0.500228 -0.071249
F -1.006817 -0.742911 -0.071263
F -0.140136 1.243251 -0.071222

PhIF_Cl_02.log
Energy (E) = -460.049844190 Hartree
Enthalpy (H) = -460.047484 Hartree
Gibbs free energy (G) = -460.065521 Hartree
Charge = 0, Spin = 2
Cl 0.000000 0.000000 0.000000

PhIF_I_02.log
Energy (E) = -11.3362262903 Hartree
Enthalpy (H) = -11.333866 Hartree
Gibbs free energy (G) = -11.353729 Hartree
Charge = 0, Spin = 2
I 0.000000 0.000000 0.000000

PhIF_OAc_02.log
Energy (E) = -228.239449373 Hartree
Enthalpy (H) = -228.186999 Hartree
Gibbs free energy (G) = -228.221197 Hartree
Charge = 0, Spin = 2
O 0.814870 1.028580 0.001326
C 0.099265 0.001821 -0.006820
O 0.806002 -1.033427 0.001395
C -1.386307 0.004349 -0.003063
H -1.730709 -0.116234 1.023617
H -1.757279 0.948812 -0.393066
H -1.756738 -0.830824 -0.593027

PhIF_OCF3_02.log

Energy (E) = -412.594885123 Hartree
Enthalpy (H) = -412.573281 Hartree
Gibbs free energy (G) = -412.606358 Hartree
Charge = 0, Spin = 2
O 0.777434 0.004949 1.150202
C 0.004844 0.000055 0.034075
F -0.776265 -1.067197 -0.022463
F 0.850697 -0.009195 -0.987103
F -0.768713 1.071956 -0.035552

PhIF_OCOCCl3_02.log
Energy (E) = -1606.76526555 Hartree
Enthalpy (H) = -1606.737255 Hartree
Gibbs free energy (G) = -1606.780801 Hartree
Charge = 0, Spin = 2
O 2.037249 -0.874763 -0.084631
C 1.308315 0.177749 -0.081554
O 1.875958 1.248908 -0.094281
C -0.211449 -0.006327 -0.008905
Cl -0.980635 1.230019 -0.969475
Cl -0.655454 0.147480 1.686224
Cl -0.592550 -1.614069 -0.600629

PhIF_OCOCF2H_02_2.log
Energy (E) = -426.629398478 Hartree
Enthalpy (H) = -426.589359 Hartree
Gibbs free energy (G) = -426.627536 Hartree
Charge = 0, Spin = 2
O 1.348764 1.121093 -0.132289
C 0.802072 -0.045775 0.053087
O 1.502569 -1.026816 0.013889
C -0.698228 -0.049604 0.368288
H -0.866164 -0.129343 1.443824
F -1.249654 1.090242 -0.086600
F -1.257853 -1.096087 -0.249497

PhIF_OMe_02.log
Energy (E) = -114.960702346 Hartree
Enthalpy (H) = -114.920190 Hartree
Gibbs free energy (G) = -114.947136 Hartree
Charge = 0, Spin = 2
O 0.789129 -0.000055 -0.006941
C -0.572861 -0.000154 -0.013298
H -0.873711 0.005993 1.048695
H -1.000848 0.903240 -0.461337
H -1.001308 -0.907873 -0.452044

PhIF_OPh_02.log
Energy (E) = -306.561912636 Hartree
Enthalpy (H) = -306.463858 Hartree
Gibbs free energy (G) = -306.499671 Hartree
Charge = 0, Spin = 2
O 2.288792 0.000022 0.000403
C 1.042025 0.000056 -0.000066
C 0.288607 -1.235001 -0.000298
C 0.288496 1.235047 -0.000304
C -1.080401 -1.220681 -0.000040
H 0.857921 -2.154153 -0.000561
C -1.080518 1.220624 -0.000017
H 0.857769 2.154237 -0.000712
C -1.774468 -0.000051 0.000286
H -1.636417 -2.148535 -0.000127
H -1.636571 2.148450 -0.000033
H -2.855487 -0.000133 0.000849

PhIF_02.log
Energy (E) = -342.508874074 Hartree
Enthalpy (H) = -342.408548 Hartree
Gibbs free energy (G) = -342.453548 Hartree
Charge = 0, Spin = 2
C -3.445010 0.158741 0.002945
C -2.631686 1.274562 -0.142933
C -1.247578 1.145844 -0.154882
C -0.711686 -0.120328 -0.011717
C -1.497585 -1.250723 0.136848
C -2.878602 -1.101261 0.142987
H -4.520431 0.270609 0.010064
H -3.070853 2.257036 -0.248609
H -0.594879 2.000590 -0.250605
H -1.050871 -2.228882 0.250255
H -3.507204 -1.973202 0.260357
I 1.384969 -0.349592 -0.049025
F 1.534862 1.951246 0.304153

PhIF_Br.log
Energy (E) = -2917.07029857 Hartree
Enthalpy (H) = -2916.966577 Hartree
Gibbs free energy (G) = -2917.014716 Hartree
Charge = 0, Spin = 1
C -3.658020 -1.273675 -0.038978
C -3.521047 0.036076 -0.477981
C -2.282261 0.665201 -0.446424
C -1.209018 -0.061320 0.029852
C -1.304643 -1.362579 0.474431
C -2.555509 -1.968162 0.438299
H -4.626556 -1.753383 -0.066867
H -4.377901 0.580286 -0.849680
H -2.163540 1.691273 -0.757714
H -0.440766 -1.902051 0.833679
H -2.658451 -2.985876 0.787637
I 0.668198 0.950229 0.072305
F -0.467037 2.656669 0.217825
Br 2.006829 -1.317590 -0.160424

PhIF_CCTMS.log
Energy (E) = -827.644727655 Hartree
Enthalpy (H) = -827.412232 Hartree
Gibbs free energy (G) = -827.482450 Hartree
Charge = 0, Spin = 1
C -3.030506 3.104212 0.021089
C -3.821057 1.987322 0.252683
C -3.269684 0.710853 0.236127
C -1.916951 0.600790 -0.017702
C -1.098743 1.687900 -0.249217
C -1.674390 2.953409 -0.231188
H -3.471019 4.091696 0.035016
H -4.878446 2.099020 0.448978
H -3.862691 -0.178096 0.391611
H -0.041918 1.569824 -0.439633
H -1.052250 3.818164 -0.416346
I -1.087100 -1.374902 -0.028876
F -3.124729 -1.994503 -0.047806
C 0.849794 -0.567696 0.013807
C 2.019243 -0.240120 0.022168
Si 3.777195 0.311109 0.038242
C 4.666429 -0.520095 -1.370800
H 5.713203 -0.211706 -1.397581
H 4.636964 -1.605176 -1.267113
H 4.212235 -0.260555 -2.327515
C 3.776098 2.162438 -0.177842
H 3.312818 2.444943 -1.124052
H 3.225911 2.649120 0.628418
H 4.796054 2.551069 -0.172781
C 4.531947 -0.164698 1.672328
H 3.998113 0.302765 2.500228
H 4.500752 -1.244839 1.818383
H 5.575334 0.152701 1.718974

PhIF_CF3.log
Energy (E) = -679.992211108 Hartree
Enthalpy (H) = -679.872599 Hartree
Gibbs free energy (G) = -679.925154 Hartree
Charge = 0, Spin = 1
C -3.677732 1.037308 0.057104
C -3.428861 -0.225321 0.579008
C -2.153179 -0.772466 0.524956
C -1.154006 -0.017495 -0.057106
C -1.367204 1.237141 -0.593660
C -2.652275 1.763453 -0.531329
H -4.675016 1.452646 0.101468
H -4.228790 -0.796265 1.029261
H -1.940822 -1.767645 0.883921
H -0.574937 1.799291 -1.063304
H -2.844033 2.740926 -0.951198
I 0.785090 -0.877470 -0.104745
F -0.315461 -2.684375 -0.241904
C 1.683628 1.148939 0.177781
F 2.941230 0.918733 0.573939
F 1.083688 1.879606 1.114235
F 1.751833 1.891325 -0.933957

PhIF_Cl.log
Energy (E) = -802.649539364 Hartree
Enthalpy (H) = -802.545628 Hartree
Gibbs free energy (G) = -802.592456 Hartree
Charge = 0, Spin = 1
C 3.638486 0.404770 -0.020280
C 3.126327 -0.846107 -0.335510
C 1.755035 -1.072181 -0.320841
C 0.936247 -0.012098 0.014249
C 1.405930 1.244585 0.331753
C 2.781788 1.443833 0.313969
H 4.706923 0.569770 -0.034100
H 3.789910 -1.658402 -0.597005
H 1.342878 -2.044361 -0.541476
H 0.733424 2.052121 0.578633
H 3.175287 2.418914 0.564534
I -1.161309 -0.402140 0.036140
F -0.631519 -2.365538 0.232546
Cl -1.669310 2.016966 -0.228174

PhIF_CN.log
Energy (E) = -435.261034573 Hartree
Enthalpy (H) = -435.149553 Hartree
Gibbs free energy (G) = -435.197795 Hartree
Charge = 0, Spin = 1
C -3.591362 0.306397 0.020705
C -3.030896 -0.927276 0.320233
C -1.651677 -1.101369 0.303420
C -0.875883 -0.006905 -0.020039
C -1.393416 1.235946 -0.322541
C -2.775978 1.381736 -0.301856
H -4.665300 0.429896 0.036296
H -3.662522 -1.767923 0.570891
H -1.195378 -2.057585 0.507204
H -0.763359 2.077514 -0.568581
H -3.205845 2.344163 -0.540981
I 1.241310 -0.318074 -0.026962
F 0.758415 -2.329719 -0.206856

C 1.480571 1.813137 0.196347
N 1.701297 2.941336 0.301180

PhIF_I.log
Energy (E) = -353.907270911 Hartree
Enthalpy (H) = -353.803737 Hartree
Gibbs free energy (G) = -353.853257 Hartree
Charge = 0, Spin = 1
C -3.533632 -2.064555 -0.050000
C -3.721041 -0.796352 -0.582478
C -2.697035 0.142120 -0.544353
C -1.501534 -0.241037 0.029498
C -1.278240 -1.487635 0.574111
C -2.319795 -2.406787 0.529383
H -4.336777 -2.787500 -0.083992
H -4.666502 -0.525808 -1.031155
H -2.836487 1.141323 -0.926632
H -0.329299 -1.753959 1.017091
H -2.172927 -3.390607 0.952161
I 0.018402 1.249804 0.090352
F -1.545587 2.577411 0.190257
I 2.218578 -0.773477 -0.116329

PhIF_N3.log
Energy (E) = -506.571505702 Hartree
Enthalpy (H) = -506.454179 Hartree
Gibbs free energy (G) = -506.505356 Hartree
Charge = 0, Spin = 1
C -3.614253 0.524426 -0.006173
C -3.154163 -0.635220 -0.614934
C -1.805508 -0.969421 -0.571752
C -0.953900 -0.109613 0.092738
C -1.374739 1.050432 0.711283
C -2.728457 1.361736 0.657303
H -4.665439 0.774294 -0.045920
H -3.842423 -1.290703 -1.130048
H -1.431110 -1.878289 -1.016917
H -0.677625 1.692353 1.229855
H -3.083141 2.261408 1.140326
I 1.115695 -0.612942 0.143759
F 0.469393 -2.526061 -0.270292
N 1.599371 1.451914 0.590495
N 1.524117 2.207827 -0.371367
N 1.466436 2.958456 -1.215513

PhIF_NPhth.log
Energy (E) = -854.603194342 Hartree
Enthalpy (H) = -854.387128 Hartree
Gibbs free energy (G) = -854.451487 Hartree
Charge = 0, Spin = 1
C -2.973704 3.231756 0.162263
C -3.622101 2.199192 0.827024
C -3.145389 0.897339 0.738352
C -2.012956 0.679490 -0.020451
C -1.349277 1.677577 -0.704524
C -1.847035 2.971673 -0.605939
H -3.353543 4.241449 0.235556
H -4.503573 2.400480 1.419495
H -3.650053 0.074926 1.221765
H -0.492022 1.460963 -1.325542
H -1.353875 3.770996 -1.141177
I -1.291320 -1.302052 -0.127720
F -3.288548 -1.795727 -0.106763
C 2.840006 0.128794 -0.616194
C 2.790862 0.025655 0.764406
C 3.893518 0.291103 1.551217
C 5.064082 0.668692 0.897092
C 5.113259 0.773044 -0.492890
C 3.993174 0.503893 -1.276059
C 1.491511 -0.213135 -1.151747
C 1.413097 -0.398057 1.146229
H 3.840783 0.206668 2.627848
H 5.952868 0.885420 1.473883
H 6.039202 1.068376 -0.967222
H 4.016303 0.581188 -2.354327
N 0.700880 -0.520646 -0.049437
O 1.120749 -0.208534 -2.302527
O 0.971792 -0.603684 2.249171

PhIF_OAc.log
Energy (E) = -570.842741012 Hartree
Enthalpy (H) = -570.684757 Hartree
Gibbs free energy (G) = -570.740005 Hartree
Charge = 0, Spin = 1
C -3.740774 1.459323 0.066642
C -3.633068 0.154511 0.527413
C -2.411482 -0.506302 0.489587
C -1.319495 0.172778 -0.016912
C -1.393452 1.471678 -0.484632
C -2.625426 2.113055 -0.438554
H -4.695257 1.966414 0.098609
H -4.499519 -0.359206 0.919851
H -2.317400 -1.529386 0.820365
H -0.520951 1.977481 -0.865785
H -2.706418 3.127545 -0.803419
I 0.514920 -0.879204 -0.074464
F -0.642338 -2.555918 -0.201942
O 1.368718 1.087351 0.108818
C 2.676619 0.972668 0.137894
O 3.238687 -0.106004 0.057166
C 3.396261 2.285337 0.275971
H 3.157338 2.919716 -0.576056
H 3.051940 2.796670 1.172917
H 4.466187 2.112768 0.325292

PhIF_OCF3.log
Energy (E) = -755.208008988 Hartree
Enthalpy (H) = -755.082793 Hartree
Gibbs free energy (G) = -755.137797 Hartree
Charge = 0, Spin = 1
C -3.476298 1.809149 0.073698
C -3.522900 0.555560 0.666583
C -2.433763 -0.304765 0.585124
C -1.321088 0.138070 -0.100351
C -1.234764 1.376149 -0.703010
C -2.338327 2.215532 -0.609253
H -4.328515 2.470807 0.143012
H -4.406821 0.235469 1.200113
H -2.460449 -1.288185 1.027854
H -0.344233 1.686137 -1.226879
H -2.298642 3.190101 -1.075008
I 0.334103 -1.193607 -0.221891
F -0.967785 -2.655834 0.273360
O 1.541869 0.468080 -0.790017
C 2.180184 1.090199 0.180221
F 2.888152 2.111246 -0.296820
F 1.344203 1.586963 1.118474
F 3.032980 0.284607 0.844229

PhIF_OCOBn.log
Energy (E) = -801.677775844 Hartree
Enthalpy (H) = -801.433467 Hartree
Gibbs free energy (G) = -801.501568 Hartree
Charge = 0, Spin = 1
C -3.539158 2.987290 -0.260813
C -3.785424 2.154867 0.822199
C -3.116612 0.943172 0.944364
C -2.207770 0.599803 -0.038358
C -1.940907 1.405133 -1.130016
C -2.622285 2.611849 -1.233082
H -4.064705 3.928114 -0.349324
H -4.500367 2.442459 1.580329
H -3.316028 0.273480 1.767027
H -1.221141 1.110765 -1.877041
H -2.433033 3.254607 -2.081580
I -1.229251 -1.262066 0.174578
F -2.903493 -1.838301 1.187879
O 0.357225 -0.271351 -0.893993
C 1.359772 -1.106495 -1.021233
O 1.313601 -2.259216 -0.628989
C 2.590372 -0.488833 -1.649485
H 3.187451 -1.294273 -2.070986
H 2.281489 0.193428 -2.440018
C 3.365394 0.252854 -0.587510
C 4.382757 -0.385594 0.114017
C 3.039952 1.567283 -0.265680
C 5.074743 0.283575 1.114782
H 4.627514 -1.413014 -0.126129
C 3.729801 2.238258 0.733933
H 2.236462 2.058177 -0.801571
C 4.750694 1.597582 1.425549
H 5.866822 -0.221347 1.651288
H 3.471839 3.261279 0.973200
H 5.290855 2.120609 2.203112

PhIF_OCOCCl3.log
Energy (E) = -1949.37536494 Hartree
Enthalpy (H) = -1949.242323 Hartree
Gibbs free energy (G) = -1949.307268 Hartree
Charge = 0, Spin = 1
C -3.815748 2.736543 0.022900
C -4.339016 1.538053 0.487752
C -3.567934 0.382301 0.473915
C -2.279521 0.472569 -0.015675
C -1.724964 1.647158 -0.487648
C -2.516468 2.789256 -0.462765
H -4.422222 3.631469 0.037429
H -5.350548 1.493119 0.866045
H -3.968288 -0.561222 0.812102
H -0.710075 1.686592 -0.852275
H -2.107395 3.720544 -0.828469
I -1.151183 -1.319059 -0.045879
F -2.921502 -2.279175 -0.179599
O 0.527831 0.042367 0.150542
C 1.646602 -0.591267 0.000441
O 1.776052 -1.781759 -0.161390
C 2.860241 0.374601 0.050845
Cl 4.349414 -0.489353 -0.227979
Cl 2.887160 1.133791 1.641905
Cl 2.637222 1.606860 -1.197376

PhIF_OCOCF2H.log
Energy (E) = -769.241329884 Hartree
Enthalpy (H) = -769.096285 Hartree
Gibbs free energy (G) = -769.156346 Hartree
Charge = 0, Spin = 1
C -3.435983 2.430083 0.049568
C -3.779120 1.171786 0.524258
C -2.856049 0.133781 0.495514
C -1.601685 0.399381 -0.018722
C -1.224867 1.638557 -0.500549
C -2.165902 2.660535 -0.460635

H -4.160054 3.232580 0.075910
H -4.767326 0.988310 0.921996
H -3.115212 -0.855301 0.840986
H -0.232588 1.816191 -0.883185
H -1.896227 3.638508 -0.833811
I -0.237065 -1.219449 -0.064063
F -1.869070 -2.413886 -0.151622
O 1.249928 0.356904 0.071103
C 2.437480 -0.158372 0.034332
O 2.694153 -1.343653 -0.042814
C 3.556911 0.888714 0.094282
F 3.399487 1.771626 -0.913717
F 3.477017 1.565311 1.257034
H 4.531613 0.410247 0.013576

PhIF_OCOCF3.log
Energy (E) = -868.459114702 Hartree
Enthalpy (H) = -868.321777 Hartree
Gibbs free energy (G) = -868.383293 Hartree
Charge = 0, Spin = 1
C -3.607798 2.501144 0.054200
C -3.971865 1.261813 0.562229
C -3.077489 0.199116 0.530677
C -1.830649 0.422459 -0.020335
C -1.433699 1.640866 -0.536854
C -2.345965 2.688320 -0.493085
H -4.309681 3.322963 0.083119
H -4.953926 1.112709 0.988384
H -3.353858 -0.775769 0.902353
H -0.448056 1.783442 -0.950222
H -2.061255 3.651338 -0.892901
I -0.512359 -1.232369 -0.070187
F -2.170053 -2.380061 -0.148402
O 1.024433 0.311477 0.058108
C 2.194065 -0.240836 0.035589
O 2.433109 -1.424516 -0.031651
C 3.322513 0.815109 0.102955
F 4.512829 0.244559 0.044029
F 3.210922 1.670906 -0.914323
F 3.238167 1.508734 1.237057

PhIF_OCOCH2CF3.log
Energy (E) = -907.749905809 Hartree
Enthalpy (H) = -907.582874 Hartree
Gibbs free energy (G) = -907.646931 Hartree
Charge = 0, Spin = 1
C -2.711309 3.234343 0.054262
C -3.526352 2.163672 0.394088
C -3.028924 0.866554 0.370305
C -1.710549 0.685610 -0.001009
C -0.869990 1.727573 -0.346704
C -1.391467 3.015575 -0.315364
H -3.105567 4.240921 0.075540
H -4.555197 2.329235 0.681434
H -3.656591 0.021903 0.610127
H 0.159171 1.558424 -0.623349
H -0.754293 3.846390 -0.584117
I -1.005525 -1.312907 -0.042581
F -2.938272 -1.843172 -0.356417
O 0.894326 -0.367125 0.340795
C 1.863694 -1.231919 0.254897
O 1.702599 -2.423207 0.074640
C 3.246491 -0.630804 0.419059
H 3.536579 -0.691981 1.467292
H 3.948743 -1.220512 -0.164189
C 3.355880 0.812379 -0.010530
F 2.726405 1.651802 0.813230
F 4.642307 1.183331 -0.044640
F 2.856200 1.004092 -1.239222

PhIF_OCOCH2ptol.log
Energy (E) = -840.954579144 Hartree
Enthalpy (H) = -840.681215 Hartree
Gibbs free energy (G) = -840.754251 Hartree
Charge = 0, Spin = 1
C 3.345496 3.276170 0.545132
C 3.627453 2.635829 -0.653577
C 3.129137 1.363571 -0.905492
C 2.351525 0.764701 0.067381
C 2.054069 1.374317 1.272130
C 2.564048 2.646092 1.503908
H 3.738044 4.265803 0.734161
H 4.237768 3.121900 -1.401827
H 3.360716 0.842335 -1.821862
H 1.440059 0.882008 2.009209
H 2.348383 3.138899 2.441742
I 1.630430 -1.178126 -0.349955
F 3.294362 -1.363499 -1.517213
O 0.003608 -0.580942 0.927153
C -0.871299 -1.557010 0.974419
O -0.698749 -2.623944 0.411880
C -2.123103 -1.212651 1.751962
H -2.607309 -2.145782 2.030343
H -1.837692 -0.666340 2.650428
C -3.036681 -0.371084 0.894367
C -4.085783 -0.953138 0.194327
C -2.817942 0.997363 0.754272
C -4.906756 -0.182242 -0.619032
H -4.257650 -2.018904 0.283594
C -3.638424 1.762583 -0.058107
H -1.990308 1.456596 1.281626
C -4.699299 1.185845 -0.755746
H -5.721150 -0.651448 -1.158019
H -3.455762 2.826324 -0.156791
C -5.600103 2.027615 -1.615054
H -6.193944 1.409045 -2.286504
H -6.290427 2.611629 -1.003649
H -5.025650 2.731421 -2.217500

PhIF_OCOCOPh.log
Energy (E) = -875.646976437 Hartree
Enthalpy (H) = -875.420741 Hartree
Gibbs free energy (G) = -875.489921 Hartree
Charge = 0, Spin = 1
C -4.354014 2.663092 0.606280
C -4.726626 1.403760 1.055460
C -3.902423 0.307086 0.835426
C -2.715108 0.516479 0.160983
C -2.311774 1.754789 -0.301994
C -3.154031 2.835805 -0.069695
H -5.001117 3.511494 0.781178
H -5.661060 1.265008 1.580915
H -4.188815 -0.682556 1.157119
H -1.372104 1.886765 -0.815251
H -2.862783 3.814431 -0.424607
I -1.503779 -1.187611 -0.177862
F -3.230443 -2.253704 -0.103014
O 0.095561 0.269397 -0.141500
C 1.213816 -0.281712 -0.495401
C 2.351899 0.735711 -0.689053
O 1.362216 -1.469690 -0.722090
O 2.115749 1.724283 -1.344955
C 3.681587 0.444672 -0.105678
C 4.709005 1.355501 -0.358527
C 3.919942 -0.667393 0.700228
C 5.964534 1.153431 0.185042
H 4.493975 2.212331 -0.982357
C 5.179870 -0.864122 1.247893
H 3.130601 -1.381333 0.884195
C 6.199713 0.042011 0.990005
H 6.761057 1.857524 -0.012812
H 5.366532 -1.727150 1.871892
H 7.181450 -0.116382 1.416087

PhIF_OCOiPr.log
Energy (E) = -649.391075642 Hartree
Enthalpy (H) = -649.173628 Hartree
Gibbs free energy (G) = -649.236109 Hartree
Charge = 0, Spin = 1
C -3.503682 2.372247 0.154826
C -3.781709 1.115868 0.674794
C -2.837212 0.099406 0.601648
C -1.624778 0.378873 0.000094
C -1.316863 1.619329 -0.528331
C -2.277062 2.620603 -0.445517
H -4.244265 3.157839 0.214705
H -4.736339 0.917089 1.141516
H -3.048419 -0.889648 0.978693
H -0.358234 1.810412 -0.984416
H -2.058522 3.596341 -0.856713
I -0.224821 -1.205096 -0.106197
F -1.855800 -2.433031 -0.057215
O 1.206938 0.396119 -0.092789
C 2.413706 -0.111906 -0.164069
O 2.612168 -1.314275 -0.231239
C 3.535972 0.902956 -0.100299
H 4.320311 0.510999 -0.748875
C 3.110860 2.291398 -0.552379
H 2.349745 2.690302 0.118529
H 3.967142 2.966025 -0.540004
H 2.698398 2.278678 -1.560985
C 4.053444 0.922778 1.339289
H 4.365198 -0.070970 1.656428
H 4.903018 1.600359 1.423143
H 3.270769 1.275893 2.013244

PhIF_OCOPh.log
Energy (E) = -762.408663313 Hartree
Enthalpy (H) = -762.193985 Hartree
Gibbs free energy (G) = -762.256620 Hartree
Charge = 0, Spin = 1
C -3.554639 2.955768 0.011020
C -4.092397 1.813756 0.588260
C -3.383899 0.618669 0.574280
C -2.139386 0.607177 -0.026225
C -1.576951 1.725991 -0.612478
C -2.303561 2.910181 -0.588947
H -4.112766 3.881763 0.024893
H -5.067676 1.844741 1.053530
H -3.799723 -0.284218 0.995087
H -0.601793 1.684054 -1.071716
H -1.885499 3.795983 -1.046608
I -1.105825 -1.236329 -0.047200
F -2.960188 -2.083870 -0.052727
O 0.629099 0.032313 0.018455
C 1.702407 -0.721888 -0.004730
O 1.640716 -1.941223 -0.064837
C 2.991198 0.021114 0.044450
C 3.021131 1.407606 0.158581
C 4.177221 -0.703504 -0.023038
C 4.239530 2.068616 0.203036
H 2.088672 1.951447 0.217741
C 5.393653 -0.040366 0.019489
H 4.120006 -1.779869 -0.108629

C 5.424276 1.345163 0.132392
H 4.267835 3.145890 0.293868
H 6.317328 -0.600204 -0.034177
H 6.373997 1.862237 0.166670

PhIF_OCOtBu.log
Energy (E) = -688.668599503 Hartree
Enthalpy (H) = -688.422336 Hartree
Gibbs free energy (G) = -688.487002 Hartree
Charge = 0, Spin = 1
C -3.661021 2.454874 0.056816
C -3.997830 1.211161 0.572752
C -3.084621 0.164601 0.537821
C -1.843257 0.402229 -0.020994
C -1.477434 1.628439 -0.545150
C -2.406895 2.660524 -0.501644
H -4.377648 3.264038 0.086584
H -4.974383 1.046110 1.006041
H -3.340787 -0.815390 0.910768
H -0.497913 1.783916 -0.969630
H -2.143506 3.626146 -0.910389
I -0.492109 -1.223551 -0.074566
F -2.164270 -2.394787 -0.130882
O 0.985330 0.329967 0.044194
C 2.181848 -0.209410 0.008345
O 2.345982 -1.415132 -0.082049
C 3.315659 0.801037 0.104891
C 4.646642 0.086238 -0.075579
H 4.703164 -0.395008 -1.051532
H 5.459673 0.809848 0.007094
H 4.780838 -0.683518 0.682960
C 3.130548 1.869483 -0.972533
H 3.951741 2.585919 -0.912289
H 3.135436 1.429813 -1.971018
H 2.191888 2.404676 -0.830520
C 3.247238 1.456660 1.486074
H 3.368692 0.716916 2.278558
H 4.052826 2.187706 1.576079
H 2.294447 1.966412 1.625302

PhIF_OH.log
Energy (E) = -418.287055787 Hartree
Enthalpy (H) = -418.170455 Hartree
Gibbs free energy (G) = -418.215945 Hartree
Charge = 0, Spin = 1
C 3.550435 -0.045049 0.006608
C 2.873243 1.154421 0.179650
C 1.483434 1.189258 0.174735
C 0.811430 -0.004034 -0.002195
C 1.451029 -1.214389 -0.180240
C 2.841208 -1.224173 -0.175505
H 4.631710 -0.060221 0.009749
H 3.422716 2.075158 0.318811
H 0.935665 2.112270 0.287795
H 0.880514 -2.120272 -0.324062
H 3.364160 -2.159491 -0.319582
I -1.312094 0.017318 -0.010370
F -1.111838 2.068530 -0.171288
O -1.311275 -2.028050 0.130665
H -1.261716 -2.293853 1.054848

PhIF_OMe.log
Energy (E) = -457.547012174 Hartree
Enthalpy (H) = -457.400741 Hartree
Gibbs free energy (G) = -457.449841 Hartree
Charge = 0, Spin = 1
C -3.610456 0.253764 0.016896
C -3.046866 -0.934666 0.461111
C -1.669528 -1.120626 0.424896
C -0.893929 -0.088052 -0.063546
C -1.420112 1.106346 -0.514934
C -2.799829 1.269273 -0.471913
H -4.683109 0.387287 0.047766
H -3.676493 -1.728356 0.838777
H -1.209889 -2.043508 0.744035
H -0.771307 1.882681 -0.894778
H -3.235398 2.193232 -0.826297
I 1.212258 -0.338114 -0.125580
F 0.789108 -2.355118 0.075521
O 1.406250 1.691800 -0.298002
C 1.300313 2.374524 0.927148
H 1.472240 3.435023 0.741069
H 0.304320 2.261599 1.374088
H 2.040418 2.030387 1.657455

PhIF_OOtBu.log
Energy (E) = -650.478458708 Hartree
Enthalpy (H) = -650.239920 Hartree
Gibbs free energy (G) = -650.301759 Hartree
Charge = 0, Spin = 1
C -3.558647 2.288002 -0.142792
C -3.818242 1.023884 -0.653022
C -2.867559 0.013942 -0.556354
C -1.670925 0.315241 0.062826
C -1.374722 1.560962 0.578886
C -2.341991 2.553446 0.469964
H -4.304426 3.066979 -0.222988
H -4.764160 0.811939 -1.131904
H -3.051882 -0.977656 -0.939715
H -0.423139 1.758511 1.047199
H -2.134177 3.536753 0.868544
I -0.205402 -1.221169 0.211183
F -1.678601 -2.485516 -0.487119
O 1.127150 0.178173 0.958424
O 1.606306 0.977004 -0.117531
C 3.010480 0.742390 -0.280409
C 3.371309 1.671978 -1.425858
H 3.135420 2.702410 -1.161981
H 4.438334 1.598125 -1.634748
H 2.816663 1.402293 -2.324335
C 3.749776 1.122051 0.991388
H 3.542885 2.161676 1.245658
H 3.432139 0.488530 1.817569
H 4.824245 1.001648 0.848250
C 3.266973 -0.708286 -0.658753
H 3.023490 -1.373786 0.170009
H 2.671264 -0.982441 -1.530832
H 4.320680 -0.846470 -0.901767

PhIF_OPh.log
Energy (E) = -649.123143215 Hartree
Enthalpy (H) = -648.921070 Hartree
Gibbs free energy (G) = -648.978579 Hartree
Charge = 0, Spin = 1
C -2.801288 2.932086 -0.188848
C -3.347795 1.887235 0.543028
C -2.721364 0.646333 0.580869
C -1.546920 0.498633 -0.130003
C -0.974172 1.514474 -0.869365
C -1.620169 2.744874 -0.893487
H -3.297007 3.892788 -0.211726
H -4.268087 2.028667 1.092503
H -3.135836 -0.181333 1.135324
H -0.056857 1.354835 -1.415849
H -1.191501 3.554184 -1.467785
I -0.568187 -1.388959 -0.079368
F -2.262495 -2.072031 0.854327
O 1.047100 -0.551350 -1.075772
C 2.075101 -0.000300 -0.381469
C 3.354512 -0.128277 -0.923173
C 1.904752 0.710086 0.805450
C 4.441607 0.447914 -0.287059
H 3.469101 -0.682836 -1.844366
C 3.003116 1.278521 1.438855
H 0.916316 0.831211 1.230254
C 4.274822 1.153442 0.899841
H 5.427900 0.341038 -0.719165
H 2.856704 1.827211 2.359793
H 5.125630 1.598043 1.396899

PhIF_OTs.log
Energy (E) = -1236.84569840 Hartree
Enthalpy (H) = -1236.600022 Hartree
Gibbs free energy (G) = -1236.668119 Hartree
Charge = 0, Spin = 1
C 1.797253 -2.991492 0.762075
C 0.906163 -3.320971 -0.250397
C -0.239503 -2.560662 -0.453931
C -0.449068 -1.482519 0.382325
C 0.407730 -1.128445 1.404686
C 1.547386 -1.902453 1.586870
H 2.685507 -3.589707 0.913975
H 1.095811 -4.171440 -0.890208
H -0.951564 -2.811513 -1.225329
H 0.212245 -0.269988 2.029161
H 2.238166 -1.644763 2.377634
I -2.166514 -0.299577 0.008143
F -2.985354 -1.988968 -0.734073
O -1.114353 1.337330 0.911900
S -0.384034 2.387802 0.015186
O -1.075453 2.467456 -1.246771
O -0.182515 3.561528 0.811971
C 1.170805 1.607214 -0.253459
C 1.313460 0.726405 -1.317448
C 2.195627 1.805525 0.658085
C 2.490766 0.008177 -1.442810
H 0.506547 0.616271 -2.030319
C 3.372652 1.084832 0.513237
H 2.060455 2.516636 1.461657
C 3.528192 0.167085 -0.523400
H 2.610564 -0.689834 -2.262576
H 4.183018 1.232077 1.216514
C 4.774599 -0.660792 -0.642018
H 4.574363 -1.691083 -0.337325
H 5.133155 -0.691930 -1.670506
H 5.571897 -0.273211 -0.010244

PhICl_02.log
Energy (E) = -702.867236299 Hartree
Enthalpy (H) = -702.767057 Hartree
Gibbs free energy (G) = -702.813755 Hartree
Charge = 0, Spin = 2
C -3.553128 0.583511 0.036612
C -3.180272 -0.667796 0.508252
C -1.848244 -1.060845 0.472553
C -0.911389 -0.182029 -0.046070
C -1.258007 1.071217 -0.520720
C -2.594019 1.448980 -0.472847
H -4.590603 0.885923 0.069932
H -3.922597 -1.343203 0.910641
H -1.551310 -2.030763 0.845728
H -0.502798 1.744520 -0.900243
H -2.880287 2.426697 -0.835010

I 1.108246 -0.788340 -0.129549
Cl 2.045936 1.937682 0.406374

PhICl_Br.log
Energy (E) = -3277.40829598 Hartree
Enthalpy (H) = -3277.305064 Hartree
Gibbs free energy (G) = -3277.355026 Hartree
Charge = 0, Spin = 1
C -3.363844 -1.917015 -0.017715
C -3.319250 -0.751207 -0.770326
C -2.183788 0.049648 -0.758206
C -1.119903 -0.363751 0.016661
C -1.124815 -1.515048 0.776415
C -2.273596 -2.296390 0.753546
H -4.252655 -2.532328 -0.031737
H -4.166990 -0.455928 -1.372292
H -2.144734 0.970664 -1.320867
H -0.263683 -1.809419 1.358499
H -2.308760 -3.201400 1.343396
I 0.627085 0.856255 0.041554
Cl -0.886174 2.887810 0.230831
Br 2.150784 -1.333808 -0.174449

PhICl_CCTMS.log
Energy (E) = -1187.97956984 Hartree
Enthalpy (H) = -1187.747441 Hartree
Gibbs free energy (G) = -1187.818249 Hartree
Charge = 0, Spin = 1
C 2.381739 3.510233 -0.024529
C 3.180175 2.548173 -0.627554
C 2.808861 1.208205 -0.608781
C 1.624969 0.885783 0.018347
C 0.801578 1.811394 0.625991
C 1.198963 3.143219 0.602566
H 2.683411 4.548423 -0.040163
H 4.101923 2.831037 -1.116291
H 3.435991 0.441512 -1.040936
H -0.123563 1.518801 1.101396
H 0.578211 3.888196 1.080595
I 1.017599 -1.173895 0.039748
Cl 3.608842 -1.819187 0.099616
C -0.978136 -0.547835 -0.026578
C -2.170131 -0.319999 -0.045345
Si -3.973764 0.069093 -0.075573
C -4.843293 -1.142930 1.037925
H -5.916613 -0.944848 1.052709
H -4.694247 -2.167093 0.694718
H -4.472066 -1.072343 2.060703
C -4.174723 1.813144 0.547825
H -3.804810 1.909030 1.569314
H -3.627450 2.517709 -0.079470
H -5.226975 2.103081 0.541911
C -4.575574 -0.084417 -1.829975
H -4.045092 0.604872 -2.487484
H -4.424062 -1.095590 -2.208756
H -5.641568 0.142726 -1.889683

PhICl_CF3.log
Energy (E) = -1040.32949723 Hartree
Enthalpy (H) = -1040.210355 Hartree
Gibbs free energy (G) = -1040.265361 Hartree
Charge = 0, Spin = 1
C -3.491513 1.544860 0.036429
C -3.268057 0.448740 0.860352
C -2.049922 -0.217111 0.829101
C -1.085734 0.255410 -0.037539
C -1.273236 1.334802 -0.877337
C -2.501484 1.984645 -0.830545
H -4.445228 2.053187 0.065006
H -4.042912 0.101559 1.529107
H -1.871280 -1.094755 1.432973
H -0.502451 1.665612 -1.557159
H -2.678824 2.829124 -1.481500
I 0.786990 -0.746592 -0.069931
Cl -0.640549 -2.982001 -0.218130
C 1.766700 1.263727 0.178816
F 3.010218 1.022250 0.594393
F 1.158271 2.021744 1.082220
F 1.846955 1.958030 -0.957670

PhICl_CN.log
Energy (E) = -795.594252820 Hartree
Enthalpy (H) = -795.483331 Hartree
Gibbs free energy (G) = -795.533266 Hartree
Charge = 0, Spin = 1
C -3.663160 -0.228568 0.001946
C -2.891664 -1.191655 0.637913
C -1.504999 -1.095051 0.639904
C -0.945382 -0.012212 -0.002795
C -1.673640 0.967477 -0.645199
C -3.058173 0.844204 -0.638176
H -4.740717 -0.315009 0.003767
H -3.361567 -2.026686 1.137911
H -0.890294 -1.851075 1.105734
H -1.198577 1.806111 -1.132952
H -3.656003 1.592791 -1.138441
I 1.190442 0.154816 0.008617
Cl 1.310764 -2.401537 -0.273198
C 0.876969 2.283175 0.267827
N 0.804431 3.430074 0.377587

PhICl_F.log
Energy (E) = -802.649539364 Hartree
Enthalpy (H) = -802.545628 Hartree
Gibbs free energy (G) = -802.592456 Hartree
Charge = 0, Spin = 1
C 3.638486 0.404770 -0.020280
C 3.126327 -0.846107 -0.335510
C 1.755035 -1.072181 -0.320841
C 0.936247 -0.012098 0.014249
C 1.405930 1.244585 0.331753
C 2.781788 1.443833 0.313969
H 4.706923 0.569770 -0.034100
H 3.789910 -1.658402 -0.597005
H 1.342878 -2.044361 -0.541476
H 0.733424 2.052121 0.578633
H 3.175287 2.418914 0.564534
I -1.161309 -0.402140 0.036140
F -0.631519 -2.365538 0.232546
Cl -1.669310 2.016966 -0.228174

PhICl_I.log
Energy (E) = -714.250039982 Hartree
Enthalpy (H) = -714.146936 Hartree
Gibbs free energy (G) = -714.198234 Hartree
Charge = 0, Spin = 1
C -2.739255 -2.971913 -0.025739
C -3.085261 -1.911024 -0.851977
C -2.341838 -0.737591 -0.835070
C -1.258428 -0.680013 0.017461
C -0.884959 -1.710880 0.855142
C -1.646991 -2.871978 0.825646
H -3.324632 -3.880577 -0.043997
H -3.936163 -1.987947 -1.513919
H -2.614214 0.103695 -1.455456
H -0.025547 -1.628373 1.505047
H -1.380274 -3.695400 1.472862
I -0.134817 1.126106 0.058870
Cl -2.324616 2.411521 0.187145
I 2.446887 -0.458311 -0.116583

PhICl_N3.log
Energy (E) = -866.906883713 Hartree
Enthalpy (H) = -866.790165 Hartree
Gibbs free energy (G) = -866.843019 Hartree
Charge = 0, Spin = 1
C -3.522449 -0.987060 -0.063333
C -2.634614 -1.653877 0.770121
C -1.280390 -1.342538 0.748755
C -0.864351 -0.355320 -0.120246
C -1.717262 0.331705 -0.961430
C -3.066269 -0.000125 -0.926444
H -4.573736 -1.238127 -0.040728
H -2.988920 -2.421160 1.443937
H -0.575197 -1.870387 1.373875
H -1.349514 1.104251 -1.621492
H -3.754997 0.516790 -1.579695
I 1.196435 0.186249 -0.155273
Cl 1.779478 -2.281344 0.149407
N 0.559992 2.275611 -0.453332
N -0.100796 2.704568 0.484124
N -0.731737 3.143187 1.316225

PhICl_NPhth.log
Energy (E) = -1214.93772289 Hartree
Enthalpy (H) = -1214.722209 Hartree
Gibbs free energy (G) = -1214.788537 Hartree
Charge = 0, Spin = 1
C -2.254043 3.606566 0.041952
C -2.845211 2.757134 0.968549
C -2.578821 1.394177 0.939792
C -1.711180 0.934822 -0.029352
C -1.108284 1.744642 -0.970888
C -1.392897 3.104452 -0.924610
H -2.470440 4.665515 0.070346
H -3.516553 3.149974 1.719041
H -3.045008 0.715280 1.638827
H -0.450738 1.334061 -1.725215
H -0.944466 3.764732 -1.653616
I -1.257470 -1.131004 -0.076797
Cl -3.767054 -1.548169 -0.059554
C 2.998214 -0.081453 -0.619733
C 2.941043 -0.103039 0.764613
C 4.059165 0.127232 1.540347
C 5.254451 0.381728 0.871216
C 5.311808 0.403582 -0.522009
C 4.175593 0.172073 -1.294223
C 1.628572 -0.355520 -1.142568
C 1.534649 -0.401146 1.163106
H 3.999929 0.108262 2.619777
H 6.156227 0.566105 1.439002
H 6.256921 0.604188 -1.007890
H 4.204942 0.186769 -2.374987
N 0.821308 -0.546723 -0.027413
O 1.259909 -0.388301 -2.293843
O 1.073531 -0.501354 2.272856

PhICl_OAc.log
Energy (E) = -931.177869027 Hartree
Enthalpy (H) = -931.020473 Hartree
Gibbs free energy (G) = -931.077473 Hartree

Charge = 0, Spin = 1
C -3.495563 1.987316 0.051681
C -3.435170 0.821808 0.803663
C -2.292362 0.032402 0.782161
C -1.232732 0.445014 -0.001434
C -1.260192 1.600209 -0.759442
C -2.413049 2.374665 -0.726577
H -4.389708 2.594902 0.070974
H -4.276739 0.519001 1.410673
H -2.242585 -0.887194 1.346999
H -0.407225 1.898992 -1.349214
H -2.459986 3.280272 -1.315067
I 0.517435 -0.747471 -0.046710
O 1.476072 1.203791 0.118663
C 2.769701 1.000346 0.152426
O 3.260202 -0.115373 0.090927
C 3.580343 2.262413 0.271939
H 3.374504 2.906968 -0.580805
H 3.282556 2.800454 1.169874
H 4.636221 2.016293 0.310638
Cl -0.950419 -2.786241 -0.218333

PhICl_OCF3.log
Energy (E) = -1115.54158456 Hartree
Enthalpy (H) = -1115.417021 Hartree
Gibbs free energy (G) = -1115.474156 Hartree
Charge = 0, Spin = 1
C -2.661270 2.833879 0.072305
C -2.883555 1.759192 0.921998
C -2.097556 0.616638 0.829902
C -1.102974 0.602961 -0.124357
C -0.845837 1.651069 -0.984109
C -1.648820 2.779753 -0.876132
H -3.278602 3.717923 0.150282
H -3.667501 1.801787 1.664819
H -2.270007 -0.230017 1.477560
H -0.042330 1.604465 -1.703001
H -1.475037 3.615496 -1.539102
I 0.140937 -1.118635 -0.277244
Cl -1.806512 -2.569730 0.214774
O 1.730907 0.261481 -0.778697
C 2.433939 0.728974 0.229284
F 3.370421 1.576590 -0.193836
F 1.676903 1.391677 1.133744
F 3.059752 -0.241962 0.927696

PhICl_OCOBn.log
Energy (E) = -1162.01302836 Hartree
Enthalpy (H) = -1161.769314 Hartree
Gibbs free energy (G) = -1161.839196 Hartree
Charge = 0, Spin = 1
C -3.287667 3.303046 0.043509
C -3.373235 2.463704 1.146149
C -2.771429 1.212008 1.122209
C -2.092830 0.840066 -0.021466
C -1.987495 1.652107 -1.134653
C -2.598280 2.899078 -1.091945
H -3.761297 4.274682 0.068528
H -3.908905 2.777175 2.031112
H -2.848417 0.542718 1.966772
H -1.437342 1.332534 -2.006482
H -2.534268 3.550473 -1.952310
I -1.169016 -1.064512 -0.075476
Cl -3.244946 -2.033921 0.964089
O 0.462634 0.099008 -0.948149
C 1.446848 -0.726153 -1.182865
O 1.377833 -1.924990 -0.960838
C 2.704377 -0.063531 -1.707021
H 3.188097 -0.771857 -2.377876
H 2.441453 0.839397 -2.253449
C 3.608719 0.267994 -0.544286
C 4.269264 -0.752204 0.136139
C 3.769562 1.581993 -0.120145
C 5.086667 -0.459192 1.217597
H 4.126190 -1.776926 -0.184457
C 4.589308 1.877815 0.961779
H 3.249522 2.375891 -0.642278
C 5.250317 0.857763 1.631886
H 5.597283 -1.258334 1.738058
H 4.710904 2.904525 1.280252
H 5.890230 1.086416 2.473440

PhICl_OCOCCl3.log
Energy (E) = -2309.71050792 Hartree
Enthalpy (H) = -2309.578064 Hartree
Gibbs free energy (G) = -2309.644407 Hartree
Charge = 0, Spin = 1
C -3.258342 3.244250 0.007389
C -3.815803 2.201779 0.735211
C -3.214269 0.949555 0.738006
C -2.058877 0.788706 0.000291
C -1.473835 1.802891 -0.733186
C -2.093755 3.046203 -0.722098
H -3.733234 4.215466 0.008938
H -4.720438 2.355562 1.306282
H -3.647671 0.127238 1.288377
H -0.557179 1.644523 -1.281384
H -1.659299 3.857520 -1.288853
I -1.129519 -1.118687 -0.012364
Cl -3.352131 -2.193188 -0.213024
O 0.681193 0.135852 0.170201
C 1.725608 -0.615165 0.060229
O 1.735427 -1.820764 -0.041936
C 3.036095 0.219840 0.054344
Cl 4.431085 -0.816432 -0.101054
Cl 3.124224 1.132282 1.559267
Cl 2.959671 1.332692 -1.318442

PhICl_OCOCF2H.log
Energy (E) = -1129.57529782 Hartree
Enthalpy (H) = -1129.430973 Hartree
Gibbs free energy (G) = -1129.492433 Hartree
Charge = 0, Spin = 1
C -3.154817 2.865121 -0.001497
C -3.391767 1.804982 0.863186
C -2.560702 0.692105 0.850920
C -1.506352 0.683439 -0.040351
C -1.240450 1.720931 -0.912700
C -2.084596 2.823838 -0.885015
H -3.807205 3.727076 0.012986
H -4.223332 1.837434 1.552818
H -2.744910 -0.145689 1.507660
H -0.397266 1.683019 -1.585168
H -1.901199 3.648282 -1.559546
I -0.232725 -1.007940 -0.070340
Cl -2.240714 -2.476257 0.000731
O 1.321192 0.567597 -0.127318
C 2.474788 -0.023125 -0.097131
O 2.656480 -1.222061 -0.082508
C 3.634286 0.987860 -0.102725
F 3.532658 1.773409 0.991657
F 4.806924 0.343408 -0.057390
H 3.600491 1.620545 -0.991080

PhICl_OCOCF3.log
Energy (E) = -1228.79444184 Hartree
Enthalpy (H) = -1228.657729 Hartree
Gibbs free energy (G) = -1228.720779 Hartree
Charge = 0, Spin = 1
C -3.022344 3.098337 0.039092
C -3.442494 2.024133 0.811917
C -2.733564 0.829724 0.794937
C -1.613275 0.757523 -0.008347
C -1.164393 1.805461 -0.788202
C -1.889393 2.990129 -0.756087
H -3.580244 4.024167 0.056812
H -4.322567 2.108585 1.433563
H -3.059471 -0.017583 1.380519
H -0.272492 1.716131 -1.389087
H -1.562360 3.826035 -1.358150
I -0.523000 -1.060032 -0.047470
Cl -2.652294 -2.318482 -0.169926
O 1.189870 0.352366 0.070435
C 2.279681 -0.337653 0.057660
O 2.376726 -1.544088 0.011782
C 3.532848 0.570703 0.102862
F 4.643337 -0.145074 0.064491
F 3.531167 1.405638 -0.937685
F 3.535494 1.299679 1.217667

PhICl_OCOCH2CF3.log
Energy (E) = -1268.08484957 Hartree
Enthalpy (H) = -1267.918406 Hartree
Gibbs free energy (G) = -1267.984199 Hartree
Charge = 0, Spin = 1
C -1.936301 3.671358 0.064960
C -2.839863 2.787113 0.638094
C -2.584011 1.421562 0.630885
C -1.413790 0.986858 0.042030
C -0.490379 1.837702 -0.535856
C -0.768494 3.198905 -0.518558
H -2.142730 4.732610 0.073003
H -3.748540 3.152743 1.095156
H -3.288320 0.723207 1.058839
H 0.425316 1.466511 -0.971332
H -0.063794 3.885883 -0.965485
I -1.008611 -1.098680 0.023736
Cl -3.411343 -1.547021 -0.477555
O 1.004351 -0.362884 0.468514
C 1.852637 -1.344156 0.386371
O 1.543425 -2.508840 0.219812
C 3.302125 -0.915418 0.526646
H 3.564493 -0.877691 1.583076
H 3.929260 -1.656185 0.038416
C 3.596168 0.438573 -0.074534
F 3.102340 1.448456 0.642140
F 4.919202 0.625666 -0.161780
F 3.098767 0.546004 -1.315703

PhICl_OCOCH2ptol.log
Energy (E) = -1201.28977497 Hartree
Enthalpy (H) = -1201.016991 Hartree
Gibbs free energy (G) = -1201.092183 Hartree
Charge = 0, Spin = 1
C 3.045395 3.515706 0.453954
C 3.135544 2.910367 -0.792236
C 2.702331 1.602394 -0.968861
C 2.183890 0.936910 0.124420
C 2.078946 1.511207 1.377046
C 2.519777 2.819237 1.533978
H 3.387831 4.532936 0.584441
H 3.544092 3.451371 -1.634133
H 2.786319 1.114559 -1.929144

H 1.656772 0.964141 2.206120
H 2.453015 3.288237 2.505704
I 1.519863 -1.057907 -0.125165
Cl 3.601070 -1.516701 -1.466059
O -0.158298 -0.315130 1.056630
C -1.003255 -1.303648 1.196406
O -0.796467 -2.420887 0.752384
C -2.280206 -0.919939 1.915656
H -2.708138 -1.827265 2.335966
H -2.039180 -0.221706 2.715799
C -3.232384 -0.285600 0.931805
C -4.197334 -1.049861 0.287132
C -3.128742 1.066248 0.615635
C -5.050723 -0.471271 -0.642928
H -4.275086 -2.106671 0.511833
C -3.982066 1.639809 -0.313531
H -2.364940 1.664373 1.098146
C -4.960356 0.881488 -0.954680
H -5.798321 -1.080291 -1.137055
H -3.889924 2.693631 -0.549217
C -5.902634 1.515963 -1.938528
H -6.346418 0.769703 -2.596180
H -6.717183 2.029447 -1.424162
H -5.390739 2.254609 -2.555107

PhICl_OCOCOPh.log
Energy (E) = -1235.98230581 Hartree
Enthalpy (H) = -1235.756672 Hartree
Gibbs free energy (G) = -1235.827295 Hartree
Charge = 0, Spin = 1
C -3.835787 3.122461 0.639991
C -4.165828 1.977446 1.352337
C -3.479352 0.790778 1.127825
C -2.471708 0.797238 0.184262
C -2.114179 1.917478 -0.541339
C -2.815494 3.092624 -0.301092
H -4.375950 4.041781 0.818934
H -4.958358 1.999624 2.086980
H -3.739380 -0.110767 1.663113
H -1.305750 1.889978 -1.255913
H -2.557836 3.983354 -0.856529
I -1.416459 -1.008294 -0.176863
Cl -3.573727 -2.267356 -0.063122
O 0.288785 0.386668 -0.208752
C 1.350212 -0.266150 -0.551323
C 2.565730 0.640487 -0.814954
O 1.407104 -1.472672 -0.721581
O 2.429980 1.551933 -1.597817
C 3.843319 0.332227 -0.132801
C 4.938282 1.152385 -0.409709
C 3.967993 -0.712372 0.781089
C 6.149009 0.927252 0.219859
H 4.810486 1.957824 -1.120036
C 5.183514 -0.933239 1.413303
H 3.125436 -1.358146 0.982435
C 6.271076 -0.116828 1.132852
H 6.998393 1.561106 0.005081
H 5.283041 -1.744936 2.120581
H 7.217910 -0.293330 1.625575

PhICl_OCOiPr.log
Energy (E) = -1009.72619316 Hartree
Enthalpy (H) = -1009.509313 Hartree
Gibbs free energy (G) = -1009.573756 Hartree
Charge = 0, Spin = 1
C -2.802876 3.094304 0.122199
C -3.179976 2.014160 0.908567
C -2.464690 0.824460 0.854505
C -1.378694 0.757476 0.004294
C -0.976210 1.814829 -0.789878
C -1.706292 2.994906 -0.723625
H -3.366365 4.015984 0.167204
H -4.033087 2.089832 1.568013
H -2.761829 -0.029014 1.446495
H -0.113406 1.731153 -1.433194
H -1.413866 3.834076 -1.339207
I -0.275189 -1.048865 -0.090167
Cl -2.455566 -2.311321 -0.107649
O 1.396957 0.345199 -0.045486
C 2.500233 -0.357565 -0.095943
O 2.497774 -1.577536 -0.156543
C 3.770748 0.464222 -0.018373
H 4.552450 -0.162248 -0.447330
C 3.656934 1.778996 -0.776709
H 2.886953 2.405245 -0.325991
H 4.605283 2.315205 -0.736649
H 3.397389 1.620679 -1.823296
C 4.075202 0.710747 1.459945
H 4.170379 -0.226130 2.007261
H 5.006894 1.267349 1.561458
H 3.274735 1.299675 1.910430

PhICl_OCOPh.log
Energy (E) = -1122.74385519 Hartree
Enthalpy (H) = -1122.529760 Hartree
Gibbs free energy (G) = -1122.594270 Hartree
Charge = 0, Spin = 1
C -3.020182 3.378929 0.010343
C -3.530932 2.384907 0.834155
C -2.971379 1.113484 0.827432
C -1.902330 0.879192 -0.014637
C -1.369602 1.846732 -0.845425
C -1.944783 3.111146 -0.826198
H -3.463171 4.365192 0.018571
H -4.367842 2.592183 1.486069
H -3.373546 0.326795 1.449234
H -0.525800 1.629636 -1.482839
H -1.549557 3.883221 -1.471450
I -1.037339 -1.053060 -0.040362
Cl -3.357145 -2.020721 -0.094855
O 0.798015 0.120971 0.029364
C 1.801052 -0.720166 0.015472
O 1.638468 -1.932335 -0.029559
C 3.150988 -0.092122 0.054472
C 3.301620 1.288584 0.139384
C 4.268907 -0.919355 0.006296
C 4.573331 1.840922 0.174335
H 2.419826 1.912356 0.181565
C 5.538643 -0.364520 0.039843
H 4.117864 -1.988079 -0.057196
C 5.690256 1.015123 0.123676
H 4.695808 2.913295 0.242061
H 6.409852 -1.004050 0.001616
H 6.681570 1.447659 0.150774

PhICl_OCOtBu.log
Energy (E) = -1049.00380563 Hartree
Enthalpy (H) = -1048.758128 Hartree
Gibbs free energy (G) = -1048.824331 Hartree
Charge = 0, Spin = 1
C -3.098267 3.046239 0.044215
C -3.479665 1.966213 0.828690
C -2.741588 0.789450 0.807245
C -1.629020 0.735565 -0.008699
C -1.221802 1.792966 -0.800310
C -1.974834 2.959877 -0.767211
H -3.679546 3.957702 0.063532
H -4.353804 2.031916 1.461104
H -3.040843 -0.064473 1.397539
H -0.338884 1.718774 -1.417252
H -1.679459 3.798941 -1.381527
I -0.491244 -1.050186 -0.054997
Cl -2.648229 -2.349268 -0.154463
O 1.150199 0.373518 0.056312
C 2.272459 -0.301618 0.033793
O 2.297375 -1.520907 -0.032847
C 3.519880 0.570158 0.106727
C 4.757611 -0.306041 -0.016368
H 4.766628 -0.839339 -0.966387
H 5.649904 0.319817 0.044357
H 4.791568 -1.046781 0.781261
C 3.475840 1.596746 -1.025252
H 4.373266 2.216495 -0.982122
H 3.446815 1.108604 -2.000646
H 2.602272 2.240979 -0.929058
C 3.514467 1.298341 1.452248
H 3.534374 0.590917 2.282428
H 4.402745 1.929023 1.521142
H 2.629704 1.926584 1.548173

PhICl_OH.log
Energy (E) = -778.620601641 Hartree
Enthalpy (H) = -778.504547 Hartree
Gibbs free energy (G) = -778.551951 Hartree
Charge = 0, Spin = 1
C 3.645197 0.362113 -0.014319
C 2.800465 1.393318 0.371942
C 1.422178 1.209903 0.383830
C 0.935322 -0.025989 0.013146
C 1.744195 -1.076150 -0.374060
C 3.118271 -0.866463 -0.388052
H 4.715289 0.516784 -0.026440
H 3.206253 2.351527 0.664874
H 0.755342 2.013351 0.659946
H 1.318087 -2.029666 -0.648768
H 3.771687 -1.670565 -0.696755
I -1.169490 -0.399241 0.026880
Cl -1.597663 2.113376 -0.234627
O -0.750527 -2.411808 0.192066
H -0.612973 -2.634993 1.119711

PhICl_OMe.log
Energy (E) = -817.880842317 Hartree
Enthalpy (H) = -817.735092 Hartree
Gibbs free energy (G) = -817.786114 Hartree
Charge = 0, Spin = 1
C -3.676640 -0.378072 -0.004867
C -2.875335 -1.338704 0.596466
C -1.490234 -1.219630 0.568983
C -0.952435 -0.119893 -0.065909
C -1.717905 0.858045 -0.670665
C -3.099891 0.715220 -0.637138
H -4.752622 -0.482445 0.017347
H -3.321309 -2.190520 1.090583
H -0.855434 -1.972649 1.012413
H -1.250531 1.708557 -1.145882
H -3.720488 1.462811 -1.111254
I 1.162907 0.155599 -0.125850
Cl 1.457541 -2.396092 -0.007019
O 0.816059 2.183645 -0.227492
C 0.459347 2.762984 1.004108
H 0.358068 3.837658 0.849670
H -0.498304 2.377401 1.375187
H 1.218448 2.597124 1.775365

PhICl_OOtBu.log
Energy (E) = -1010.81234865 Hartree
Enthalpy (H) = -1010.574298 Hartree
Gibbs free energy (G) = -1010.637682 Hartree
Charge = 0, Spin = 1

C -3.354601 2.732377 -0.130299
C -3.823505 1.455977 0.146035
C -2.940786 0.385464 0.232433
C -1.599133 0.641489 0.042009
C -1.092979 1.897704 -0.230172
C -1.996775 2.950043 -0.319442
H -4.048297 3.558900 -0.199811
H -4.879609 1.281305 0.296765
H -3.297173 -0.614842 0.428331
H -0.035948 2.063049 -0.370177
H -1.628070 3.941957 -0.540645
I -0.193869 -0.956595 0.208906
Cl -2.004782 -2.613307 -0.519288
O 1.200245 0.477989 0.843025
O 1.937415 0.933642 -0.278161
C 3.301342 0.505708 -0.150879
C 3.950489 1.086546 -1.394604
H 3.841683 2.170629 -1.404525
H 5.012172 0.841187 -1.401606
H 3.488814 0.677037 -2.292852
C 3.908182 1.088723 1.113616
H 3.826750 2.175555 1.098901
H 3.389053 0.709842 1.992068
H 4.961691 0.815116 1.181671
C 3.380492 -1.013040 -0.155427
H 2.924598 -1.427168 0.744580
H 2.879733 -1.416153 -1.037543
H 4.423323 -1.329630 -0.177844

PhICl_OPh.log
Energy (E) = -1009.45621362 Hartree
Enthalpy (H) = -1009.254730 Hartree
Gibbs free energy (G) = -1009.314566 Hartree
Charge = 0, Spin = 1

C -2.008565 3.489791 -0.135843
C -2.628029 2.578518 0.707317
C -2.262350 1.236624 0.687682
C -1.268098 0.855932 -0.188431
C -0.624745 1.733863 -1.038394
C -1.012408 3.067925 -1.005847
H -2.302135 4.530170 -0.115165
H -3.402078 2.902289 1.388944
H -2.750338 0.518557 1.329711
H 0.158070 1.393752 -1.699859
H -0.527330 3.773371 -1.665800
I -0.621784 -1.184899 -0.247840
Cl -2.832610 -1.931853 0.748268
O 1.159136 -0.525553 -1.133504
C 2.216869 -0.171149 -0.360990
C 3.489578 -0.504803 -0.825883
C 2.089606 0.535713 0.833598
C 4.613739 -0.126863 -0.110006
H 3.569613 -1.056612 -1.752377
C 3.223227 0.902131 1.547893
H 1.108973 0.811012 1.200999
C 4.489147 0.576962 1.082790
H 5.594622 -0.389815 -0.483604
H 3.110807 1.450173 2.473954
H 5.367832 0.864824 1.642846

PhICl_OTS.log
Energy (E) = -1597.17637075 Hartree
Enthalpy (H) = -1596.931056 Hartree
Gibbs free energy (G) = -1597.005375 Hartree
Charge = 0, Spin = 1

C -3.404175 -3.405905 0.407284
C -3.442359 -2.792094 -0.837545
C -2.870330 -1.539197 -1.018090
C -2.272347 -0.940877 0.072810
C -2.212399 -1.522412 1.324587
C -2.792229 -2.775064 1.482120
H -3.853331 -4.380319 0.540228
H -3.916236 -3.283740 -1.675387
H -2.907711 -1.045814 -1.978411
H -1.716926 -1.029659 2.147227
H -2.762878 -3.252184 2.451516
I -1.393894 0.972714 -0.169831
Cl -3.326240 1.676067 -1.530679
O 0.199635 0.037149 1.054391
S 1.407171 0.959693 1.329757
O 1.097182 2.246816 0.732372
O 1.764102 0.894822 2.717456
C 2.696013 0.217714 0.389155
C 2.747678 0.441079 -0.981399
C 3.628180 -0.587406 1.020272
C 3.747435 -0.160631 -1.723974
H 2.023222 1.097787 -1.445320
C 4.626623 -1.183040 0.260708
H 3.568908 -0.728448 2.090322
C 4.699055 -0.981210 -1.114055
H 3.801712 0.010509 -2.792232
H 5.364751 -1.811275 0.743401
C 5.785138 -1.616683 -1.933893
H 5.365349 -2.201103 -2.753191
H 6.432252 -0.858123 -2.376242
H 6.403546 -2.275618 -1.327066

FuranIBA_A_vinyl.log
Energy (E) = -506.276500178 Hartree
Enthalpy (H) = -506.159193 Hartree
Gibbs free energy (G) = -506.209104 Hartree
Charge = 0, Spin = 1

C -1.767003 2.548923 0.036638
C -0.450329 2.218702 0.111140
C -0.464924 0.800359 0.065650
C -1.741825 0.374709 -0.032020
O -2.548575 1.449871 -0.050097
H -2.273355 3.497311 0.032867
H 0.386826 2.890499 0.177234
C -2.164276 -1.063272 -0.125864
I 0.975921 -0.675488 0.108226
O -3.333518 -1.373911 -0.211099
O -1.114089 -1.810172 -0.100729
C 2.541145 0.765395 0.294964
H 2.615180 1.251424 1.258403
C 3.383742 0.995067 -0.696332
H 3.296632 0.499398 -1.655362
H 4.201189 1.696600 -0.578771

IBamideAc_vinyl.log
Energy (E) = -641.123195309 Hartree
Enthalpy (H) = -640.922367 Hartree
Gibbs free energy (G) = -640.983230 Hartree
Charge = 0, Spin = 1

C -2.820166 -2.345158 -0.050900
C -1.895797 -3.383320 -0.067567
C -0.539232 -3.105760 -0.044850
C -0.080759 -1.791848 0.002095
C -1.028535 -0.791075 0.028132
C -2.389281 -1.024822 -0.002181
H -3.880525 -2.554800 -0.078478
H -2.237574 -4.408387 -0.104995
H 0.214853 -3.881292 -0.066409
H -3.105532 -0.216265 -0.004535
C 1.413654 -1.542812 -0.009507
O 2.163980 -2.498126 -0.000570
I -0.277312 1.192847 0.133463
N 1.682330 -0.212065 -0.032855
C 3.041232 1.797391 -0.042930
H 4.079688 2.109716 -0.093334
H 2.493279 2.234052 -0.880702
H 2.597785 2.167762 0.883919
C 2.977131 0.281408 -0.097156
O 3.986242 -0.378340 -0.195518
C -2.246546 2.044002 0.293495
H -2.760301 1.902489 1.235691
C -2.731193 2.804607 -0.673980
H -2.207598 2.945574 -1.612263
H -3.677692 3.320761 -0.561627

IBCCF32O_vinyl.log
Energy (E) = -1108.27558954 Hartree
Enthalpy (H) = -1108.093284 Hartree
Gibbs free energy (G) = -1108.159190 Hartree
Charge = 0, Spin = 1

C -0.969212 3.378736 0.207873
C 0.417091 3.429969 0.233656
C 1.164765 2.264974 0.146602
C 0.537218 1.026006 0.030155
C -0.840664 1.012276 0.027769
C -1.613577 2.152532 0.109637
H -1.553739 4.286470 0.262619
H 0.921785 4.382340 0.317125
H 2.242616 2.314000 0.169013
H -2.692502 2.102122 0.079533
O 0.442638 -1.307334 -0.464338
I -1.751164 -0.907461 -0.069836
C 1.275302 -0.315261 -0.104127
C 2.367638 -0.208123 -1.199414
C 1.908258 -0.651341 1.270657
F 0.919285 -0.816551 2.159643
F 2.706055 0.313492 1.747003
F 2.612482 -1.774604 1.229186
F 3.390497 0.594586 -0.860618
F 2.878953 -1.396797 -1.481225
F 1.828809 0.285530 -2.316426
C -3.694262 -0.097704 0.327212
H -3.866441 0.282933 1.325844
C -4.660671 -0.191858 -0.571401
H -4.481954 -0.576805 -1.568561
H -5.675212 0.110911 -0.339305

IBCH2S_vinyl.log
Energy (E) = -835.934783079 Hartree
Enthalpy (H) = -835.712733 Hartree
Gibbs free energy (G) = -835.768848 Hartree
Charge = 0, Spin = 1

C -0.515738 3.261119 -0.265584
C -1.873850 3.017714 -0.146752
C -2.343550 1.719365 0.004267
C -1.482563 0.621393 0.036201
C -0.131843 0.917712 -0.072492
C 0.372108 2.192241 -0.222696
H -0.141442 4.265715 -0.404092
H -2.577420 3.838469 -0.180037
H -3.407319 1.553407 0.093703
H 1.434752 2.357871 -0.332898

I 1.247054 -0.719415 -0.057011
C -2.002119 -0.798923 0.225538
S -1.048071 -1.911446 -0.844684
C -3.468388 -0.928919 -0.177102
H -3.744496 -1.981905 -0.136598
H -4.125086 -0.384603 0.506404
H -3.627092 -0.572013 -1.194378
C -1.870229 -1.182498 1.702644
H -2.466453 -0.509884 2.325641
H -2.225384 -2.203761 1.839170
H -0.835432 -1.136089 2.042124
C 2.807686 0.638728 0.666632
H 2.620207 1.150158 1.603757
C 3.976239 0.693034 0.049967
H 4.153199 0.175752 -0.887470
H 4.810711 1.253211 0.457485

IBNMeCO2_vinyl.log
Energy (E) = -603.063999921 Hartree
Enthalpy (H) = -602.868339 Hartree
Gibbs free energy (G) = -602.924641 Hartree
Charge = 0, Spin = 1
C -0.244889 3.272665 -0.518451
C -1.593144 3.165066 -0.204348
C -2.160189 1.939336 0.098270
C -1.406328 0.754338 0.109561
C -0.047919 0.916060 -0.181455
C 0.534946 2.125998 -0.502715
H 0.196630 4.226746 -0.766744
H -2.219968 4.046495 -0.208075
H -3.216552 1.881087 0.315477
H 1.592180 2.177223 -0.726501
I 1.185398 -0.776146 -0.044164
C -1.774862 -1.642261 -0.382425
O -2.602974 -2.527249 -0.300083
N -1.984459 -0.463833 0.434101
C -3.230396 -0.448348 1.182241
H -3.163067 0.284962 1.984978
H -4.093637 -0.218733 0.551830
H -3.383215 -1.438594 1.596371
O -0.723890 -1.602057 -1.111094
C 2.720529 0.369437 0.891743
H 2.422391 0.906660 1.782277
C 3.961129 0.355698 0.435815
H 4.240898 -0.181489 -0.462748
H 4.751128 0.884741 0.955127

Indole_NMe_IBA_A_vinyl.log
Energy (E) = -679.211037738 Hartree
Enthalpy (H) = -679.001608 Hartree
Gibbs free energy (G) = -679.061218 Hartree
Charge = 0, Spin = 1
C -1.127887 -0.956907 0.032803
C -2.355054 -0.245071 -0.001730
C -3.588776 -0.895259 -0.010031
C -3.589348 -2.274201 0.011806
C -2.385648 -2.996743 0.035442
C -1.163312 -2.358154 0.044489
C -0.144221 0.071893 0.026995
H -4.511257 -0.330634 -0.034842
H -4.529390 -2.808454 0.005855
H -2.419372 -4.077589 0.042672
H -0.252805 -2.938337 0.045986
N -2.101742 1.104013 -0.031073
C -0.748406 1.295685 -0.015054
C 0.010144 2.598135 -0.066867
O 1.271204 2.390436 -0.085010
O -0.583482 3.666326 -0.090218
I 1.934573 0.101757 0.089635
C -3.135859 2.121228 -0.084656
H -3.779006 2.041267 0.791893
H -2.653278 3.092467 -0.103249
H -3.740876 1.987512 -0.981602
C 2.167863 -2.005611 0.293941
H 1.806183 -2.429688 1.220443
C 2.820486 -2.703898 -0.618340
H 3.177151 -2.259893 -1.539551
H 3.020789 -3.758549 -0.471730

NpthIBA_C_vinyl.log
Energy (E) = -661.977287909 Hartree
Enthalpy (H) = -661.779920 Hartree
Gibbs free energy (G) = -661.837286 Hartree
Charge = 0, Spin = 1
C -1.763682 1.582294 0.044708
C -0.411365 1.368248 0.021084
C 0.032888 0.043747 -0.032154
C -0.785430 -1.040636 -0.053566
H -2.106223 2.609156 0.091889
H -0.409819 -2.055187 -0.074002
C 0.566039 2.530537 0.091807
O 1.791949 2.152003 0.116824
O 0.127809 3.665324 0.126919
I 2.151661 -0.143812 -0.095676
C -3.102784 -1.900954 -0.047178
C -4.449345 -1.665278 -0.024507
C -4.943308 -0.341861 0.018955
C -4.079212 0.717061 0.039518
C -2.678842 0.507295 0.016196
C -2.185997 -0.823421 -0.028681
H -2.718811 -2.913095 -0.079978
H -5.144421 -2.493771 -0.039402
H -6.011096 -0.171770 0.036710
H -4.447739 1.734324 0.074416
C 2.062032 -2.266251 -0.316820
H 1.746397 -2.631436 -1.285236
C 2.472135 -3.070193 0.649822
H 2.789564 -2.694452 1.615006
H 2.507287 -4.143860 0.506403

PyIBA_D_vinyl.log
Energy (E) = -524.494471982 Hartree
Enthalpy (H) = -524.358753 Hartree
Gibbs free energy (G) = -524.409987 Hartree
Charge = 0, Spin = 1
C -1.636220 2.707124 -0.047684
C -2.826102 1.989134 0.016845
C -1.747685 -0.030027 0.032337
C -0.533827 0.629390 -0.044115
H -1.644008 3.787280 -0.069954
H -3.775725 2.510770 0.039214
C -1.795311 -1.557727 0.108810
O -0.613915 -2.075184 0.118211
O -2.862453 -2.124007 0.158055
I 1.167017 -0.627334 -0.101110
C -0.441664 2.004237 -0.079384
H 0.509208 2.517164 -0.116461
N -2.884031 0.664261 0.059954
C 2.512019 1.025891 -0.317745
H 2.545591 1.494472 -1.292791
C 3.327293 1.371692 0.664777
H 3.292324 0.893994 1.636649
H 4.068856 2.150461 0.529308

PyrroleNMeIBA_A_vinyl.log
Energy (E) = -525.705339084 Hartree
Enthalpy (H) = -525.545647 Hartree
Gibbs free energy (G) = -525.599644 Hartree
Charge = 0, Spin = 1
C 1.828225 2.266446 -0.062408
C 0.462233 2.076405 -0.113653
C 0.309949 0.680738 -0.068399
C 1.526330 0.066369 0.006744
H 2.395731 3.181983 -0.069813
H -0.294246 2.839549 -0.162878
C 1.721622 -1.412643 0.096246
I -1.328743 -0.584086 -0.096539
O 2.849083 -1.882000 0.162812
O 0.593588 -2.023770 0.093748
N 2.459631 1.058678 0.010385
C 3.898379 0.860826 0.089629
H 4.154750 0.331123 1.002540
H 4.242020 0.262294 -0.749000
H 4.373316 1.839183 0.074699
C -2.675960 1.057039 -0.288188
H -2.630338 1.582996 -1.232219
C -3.532669 1.363766 0.669436
H -3.562621 0.828993 1.610721
H -4.242670 2.172155 0.540875

QuinoIBA_L_vinyl.log
Energy (E) = -678.018454095 Hartree
Enthalpy (H) = -677.833165 Hartree
Gibbs free energy (G) = -677.890699 Hartree
Charge = 0, Spin = 1
C 4.306688 -1.729622 -0.050354
C 2.949111 -1.901848 -0.088630
C 2.098899 -0.778570 -0.045285
C 2.653124 0.524469 0.038474
C 4.867587 -0.436195 0.032465
H 4.960164 -2.590738 -0.083115
H 2.497535 -2.882076 -0.150130
H 5.942421 -0.322754 0.061642
C 0.025634 0.096781 -0.038543
N 0.742905 -0.958823 -0.084404
C 0.417482 1.435650 0.041848
C 4.059613 0.666708 0.076379
H 4.478633 1.662631 0.140502
C -0.581796 2.579575 0.089249
O -0.144819 3.714922 0.163794
O -1.794580 2.182574 0.043584
C 1.771889 1.628372 0.081751
H 2.139181 2.646158 0.147508
I -2.117977 -0.184320 -0.109342
C -1.876057 -2.291827 -0.208220
H -1.285724 -2.653168 -1.036657
C -2.476424 -3.078919 0.666144
H -3.048709 -2.690343 1.499454
H -2.410386 -4.156401 0.576078

FuranIBA_A_Ph.log
Energy (E) = -659.797547681 Hartree
Enthalpy (H) = -659.629466 Hartree
Gibbs free energy (G) = -659.685959 Hartree
Charge = 0, Spin = 1
C 1.492300 2.888501 -0.000179
C 0.383489 2.101738 -0.000615
C 0.916681 0.786840 -0.000119
C 2.264231 0.854230 -0.000020
O 2.624664 2.149780 -0.000033
H 1.618890 3.956161 -0.000155
H -0.644914 2.417736 -0.000927
C 3.183756 -0.333096 0.000175

I 0.099734 -1.108276 -0.000085
O 4.389399 -0.198085 0.000443
O 2.476597 -1.410662 0.000069
C -1.882907 -0.306214 -0.000002
C -2.513535 -0.043047 -1.208121
C -2.513118 -0.042681 1.208272
C -3.794880 0.494488 -1.204342
H -2.012912 -0.249307 -2.145303
C -3.794450 0.494879 1.204790
H -2.012176 -0.248687 2.145341
C -4.432567 0.762327 0.000292
H -4.292434 0.702988 -2.141427
H -4.291672 0.703672 2.141986
H -5.429941 1.179994 0.000398

IBamideAc_Ph.log
Energy (E) = -794.643797809 Hartree
Enthalpy (H) = -794.392148 Hartree
Gibbs free energy (G) = -794.459458 Hartree
Charge = 0, Spin = 1
C 0.488909 3.508955 0.000220
C -0.837485 3.924370 0.000969
C -1.857314 2.987903 0.001079
C -1.575034 1.624101 0.000401
C -0.246845 1.253229 -0.000341
C 0.799941 2.154228 -0.000443
H 1.290778 4.234345 0.000152
H -1.072074 4.979854 0.001477
H -2.903277 3.264594 0.001724
H 1.830794 1.831710 -0.000997
C -2.733593 0.647096 0.000719
O -3.866080 1.087369 0.001527
I 0.129997 -0.844761 -0.001527
N -2.287098 -0.634684 -0.000279
C -2.429987 -3.055868 0.000138
H -3.165097 -3.854734 0.000688
H -1.796436 -3.149779 -0.884481
H -1.795409 -3.149513 0.884048
C -3.151180 -1.719542 0.000378
O -4.360080 -1.669136 0.001252
C 2.265009 -0.573151 -0.000630
C 2.948767 -0.516660 1.206955
C 2.950976 -0.512017 -1.206740
C 4.332579 -0.390060 1.206213
H 2.409157 -0.565326 2.144494
C 4.334793 -0.385382 -1.202866
H 2.413109 -0.557101 -2.145460
C 5.022965 -0.323650 0.002435
H 4.869361 -0.344158 2.143879
H 4.873293 -0.335844 -2.139361
H 6.099899 -0.226108 0.003578

IBCCF32O_Ph.log
Energy (E) = -1261.79611402 Hartree
Enthalpy (H) = -1261.563035 Hartree
Gibbs free energy (G) = -1261.635785 Hartree
Charge = 0, Spin = 1
C -0.546297 3.263937 0.081600
C 0.829273 3.417571 0.176449
C 1.662155 2.309630 0.139111
C 1.133831 1.027548 -0.003995
C -0.237446 0.913215 -0.085108
C -1.094594 1.994228 -0.044078
H -1.198579 4.125714 0.106441
H 1.258305 4.404347 0.281122
H 2.730592 2.435742 0.224193
H -2.165298 1.867366 -0.111491
O 1.248514 -1.313556 -0.453260
I -0.997524 -1.074734 -0.253931
C 1.978397 -0.254214 -0.065124
C 3.130555 -0.081860 -1.088557
C 2.542677 -0.519685 1.355063
F 1.512534 -0.751022 2.180331
F 3.230042 0.510141 1.866337
F 3.333353 -1.584744 1.380043
F 4.069908 0.795679 -0.696853
F 3.742435 -1.233900 -1.317578
F 2.633772 0.359197 -2.246588
C -3.027246 -0.409345 -0.053669
C -3.794655 -0.190487 -1.190506
C -3.580824 -0.256691 1.211463
C -5.125235 0.189616 -1.059723
H -3.359064 -0.310346 -2.174593
C -4.910290 0.125426 1.338557
H -2.978500 -0.429563 2.094546
C -5.680309 0.348687 0.203507
H -5.725479 0.361299 -1.942794
H -5.344072 0.246642 2.321763
H -6.715596 0.644536 0.304074

IBCH2S_Ph.log
Energy (E) = -989.453991271 Hartree
Enthalpy (H) = -989.181033 Hartree
Gibbs free energy (G) = -989.243834 Hartree
Charge = 0, Spin = 1
C -0.313717 3.137364 -0.754674
C -1.647862 3.323620 -0.433064
C -2.432693 2.245316 -0.047037
C -1.923066 0.947484 0.023089
C -0.579617 0.815188 -0.296205
C 0.232728 1.861624 -0.678777
H 0.302397 3.966580 -1.072909
H -2.087238 4.310423 -0.487649
H -3.470433 2.413805 0.201542
H 1.270457 1.697232 -0.931186
I 0.256492 -1.163213 -0.253155
C -2.785137 -0.225996 0.472517
S -2.386818 -1.663457 -0.560914
C -4.274485 0.058460 0.300324
H -4.826630 -0.854059 0.522002
H -4.620754 0.834399 0.987958
H -4.499414 0.354364 -0.724036
C -2.514523 -0.500666 1.955115
H -2.781685 0.371250 2.558855
H -3.112953 -1.353409 2.275241
H -1.465903 -0.735748 2.138939
C 2.250081 -0.297492 0.083609
C 2.563242 0.277300 1.308414
C 3.220245 -0.423039 -0.899575
C 3.851376 0.738728 1.545538
H 1.803604 0.374513 2.076001
C 4.512769 0.034648 -0.659563
H 2.975686 -0.871333 -1.855687
C 4.825988 0.616532 0.560939
H 4.096393 1.190042 2.497815
H 5.269855 -0.060554 -1.426550
H 5.829647 0.973685 0.747578

IBNMeCO2_Ph.log
Energy (E) = -756.583405149 Hartree
Enthalpy (H) = -756.337009 Hartree
Gibbs free energy (G) = -756.399850 Hartree
Charge = 0, Spin = 1
C -0.258157 3.062674 -1.131788
C -1.497091 3.347718 -0.572068
C -2.260452 2.354157 0.014802
C -1.822395 1.020693 0.077939
C -0.556663 0.788654 -0.468198
C 0.215056 1.761082 -1.072884
H 0.334445 3.835198 -1.599680
H -1.883929 4.357307 -0.603704
H -3.230546 2.598462 0.421990
H 1.187043 1.511487 -1.477196
I 0.238837 -1.149265 -0.288287
C -2.852759 -1.226845 0.025254
O -3.837665 -1.842411 0.382453
N -2.590640 0.037637 0.684931
C -3.611432 0.459035 1.629669
H -3.204476 1.228244 2.285097
H -4.507893 0.841359 1.134077
H -3.905484 -0.406926 2.212046
O -1.999587 -1.541217 -0.873718
C 2.177224 -0.378053 0.185634
C 2.361907 0.382540 1.332494
C 3.243464 -0.678760 -0.648620
C 3.630701 0.851474 1.641916
H 1.521952 0.615220 1.975148
C 4.515090 -0.214183 -0.328165
H 3.091648 -1.265396 -1.546085
C 4.706497 0.550694 0.813447
H 3.780654 1.447549 2.531648
H 5.350285 -0.445956 -0.974790
H 5.694462 0.914764 1.059653

Indole_NMe_IBA_A_Ph.log
Energy (E) = -832.732804799 Hartree
Enthalpy (H) = -832.472621 Hartree
Gibbs free energy (G) = -832.538058 Hartree
Charge = 0, Spin = 1
C -0.586341 1.345902 -0.000012
C -1.915148 1.847109 0.000092
C -2.196571 3.213078 0.000121
C -1.131891 4.088869 0.000083
C 0.189480 3.615049 -0.000032
C 0.474706 2.265497 -0.000113
C -0.767105 -0.068745 -0.000197
H -3.219170 3.566209 0.000142
H -1.315294 5.154559 0.000096
H 1.004512 4.325912 -0.000088
H 1.504348 1.939807 -0.000264
N -2.803220 0.800250 0.000039
C -2.098751 -0.371018 -0.000105
C -2.636743 -1.779644 -0.000044
O -1.685241 -2.633194 0.000052
O -3.841388 -1.988094 -0.000083
I 0.519232 -1.708716 0.000069
C -4.245311 0.963645 -0.000116
H -4.555258 1.515955 -0.887515
H -4.697057 -0.022417 0.000108
H -4.555353 1.516436 0.886945
C 2.285844 -0.515261 -0.000025
C 2.844740 -0.124855 1.208506
C 2.844837 -0.125013 -1.208555
C 3.968952 0.691230 1.205215
H 2.398404 -0.433439 2.144711
C 3.969046 0.691083 -1.205274
H 2.398593 -0.433730 -2.144761
C 4.524621 1.102938 -0.000034
H 4.405223 1.009383 2.141950
H 4.405383 1.009117 -2.142019
H 5.395739 1.743538 -0.000036

NpthIBA_C_Ph.log
Energy (E) = -815.498285486 Hartree
Enthalpy (H) = -815.250116 Hartree

Gibbs free energy (G) = -815.313771
Hartree
Charge = 0, Spin = 1
C 2.869375 -0.851823 -0.000080
C 1.595906 -1.355818 -0.000050
C 0.538323 -0.440904 0.000006
C 0.690462 0.909306 0.000047
H 3.687206 -1.562590 -0.000118
H -0.148376 1.592947 0.000092
C 1.351513 -2.856458 -0.000006
O 0.105199 -3.160779 0.000049
O 2.309754 -3.607146 -0.000033
I -1.387695 -1.360411 0.000029
C 2.247420 2.828294 0.000082
C 3.526558 3.311181 0.000059
C 4.626281 2.423420 -0.000016
C 4.422257 1.071840 -0.000068
C 3.109846 0.539394 -0.000046
C 2.007539 1.433792 0.000029
H 1.400385 3.503169 0.000141
H 3.702599 4.378346 0.000099
H 5.632102 2.820708 -0.000032
H 5.258003 0.383764 -0.000123
C -2.397943 0.523639 -0.000034
C -2.734899 1.121828 -1.207261
C -2.734852 1.121866 1.207197
C -3.412503 2.334862 -1.204615
H -2.467251 0.650701 -2.144446
C -3.412457 2.334892 1.204545
H -2.467164 0.650759 2.144380
C -3.749332 2.939536 -0.000040
H -3.676481 2.805510 -2.141716
H -3.676401 2.805573 2.141639
H -4.277383 3.883201 -0.000043

PyIBA_D_Ph.log
Energy (E) = -678.015715201 Hartree
Enthalpy (H) = -677.829190 Hartree
Gibbs free energy (G) = -677.886743
Hartree
Charge = 0, Spin = 1
C -1.040411 3.024617 -0.000028
C -2.425699 2.898089 -0.000029
C -2.336951 0.610476 -0.000005
C -0.954646 0.674483 -0.000010
H -0.575279 3.999842 -0.000032
H -3.053010 3.781845 -0.000032
C -3.052322 -0.742235 0.000008
O -2.219481 -1.726695 0.000037
O -4.261053 -0.782244 -0.000012
I 0.031295 -1.203638 0.000008
C -0.270527 1.871426 -0.000022
H 0.809622 1.919282 -0.000024
N -3.057022 1.731298 -0.000018
C 1.972139 -0.294823 -0.000002
C 2.600592 -0.016178 1.207005
C 2.600654 -0.016301 -1.207004
C 3.868638 0.552017 1.204588
H 2.106188 -0.235962 2.144855
C 3.868698 0.551898 -1.204578
H 2.106301 -0.236179 -2.144858
C 4.499856 0.836031 0.000007
H 4.361862 0.771083 2.141637
H 4.361971 0.770868 -2.141624
H 5.486978 1.277437 0.000010

PyrroleNMeIBA_A_Ph.log
Energy (E) = -679.226030720 Hartree
Enthalpy (H) = -679.015577 Hartree
Gibbs free energy (G) = -679.076488
Hartree
Charge = 0, Spin = 1
C 1.601357 2.591667 -0.000557
C 0.402742 1.907224 -0.001033
C 0.783754 0.555495 -0.000060
C 2.143140 0.436601 0.000028
H 1.787354 3.652740 -0.000623
H -0.586875 2.329890 -0.001426
C 2.876757 -0.865390 0.000079
I -0.275545 -1.224828 0.000083
O 4.099968 -0.882928 0.000232
O 2.057381 -1.852748 -0.000116
N 2.639861 1.705409 -0.000121
C 4.050850 2.056922 0.000154
H 4.540644 1.644971 0.877749
H 4.540988 1.645013 -0.877276
H 4.126923 3.141959 0.000192
C -2.137470 -0.177008 0.000072
C -2.729066 0.165491 -1.207689
C -2.728281 0.166675 1.207888
C -3.931427 0.861972 -1.204217
H -2.256903 -0.101263 -2.144279
C -3.930640 0.863172 1.204524
H -2.255506 -0.099132 2.144437
C -4.530112 1.208964 0.000179
H -4.397767 1.132940 -2.141455
H -4.396373 1.135053 2.141800
H -5.466065 1.750569 0.000213

QuinoIBA_L_Ph.log
Energy (E) = -831.537848948 Hartree
Enthalpy (H) = -831.301846 Hartree
Gibbs free energy (G) = -831.366096
Hartree
Charge = 0, Spin = 1
C 3.155890 3.433443 0.000052
C 1.928361 2.827230 0.000029
C 1.843347 1.420526 0.000018
C 3.026827 0.638824 0.000005
C 4.340163 2.663974 0.000047
H 3.224148 4.512813 0.000071
H 1.007051 3.392999 0.000023
H 5.299276 3.163180 0.000064
C 0.597809 -0.452983 -0.000040
N 0.613897 0.820939 -0.000013
C 1.665045 -1.356379 -0.000017
C 4.278311 1.297291 0.000020
H 5.179985 0.698469 0.000011
C 1.466855 -2.862990 0.000025
O 2.461360 -3.568117 0.000058
O 0.236175 -3.202867 0.000024
C 2.901516 -0.769170 -0.000003
H 3.770907 -1.416774 0.000008
I -1.357011 -1.407523 -0.000034
C -2.338966 0.478117 -0.000001
C -2.659589 1.080684 -1.207685
C -2.659893 1.080507 1.207689
C -3.314626 2.305309 -1.204461
H -2.398079 0.605234 -2.143877
C -3.314922 2.305137 1.204478
H -2.398611 0.604921 2.143875
C -3.641413 2.915159 0.000012
H -3.567958 2.781601 -2.141664
H -3.568502 2.781288 2.141686
H -4.152498 3.868228 0.000021

FuranIBA_A_OTf.log
Energy (E) = -1389.46399242 Hartree
Enthalpy (H) = -1389.353468 Hartree
Gibbs free energy (G) = -1389.417010
Hartree
Charge = 0, Spin = 1
C -2.446705 2.761997 0.099908
C -1.257076 2.148598 0.351245
C -1.568769 0.780391 0.191126
C -2.871299 0.657122 -0.133597
O -3.427051 1.874820 -0.193540
H -2.730123 3.798897 0.091836
H -0.317287 2.608745 0.601875
C -3.507281 -0.646542 -0.370794
I -0.660182 -1.047007 0.294604
O -4.649467 -0.854862 -0.658057
O -2.590835 -1.624171 -0.211726
O 1.141958 0.038715 0.939253
S 1.979759 0.836256 -0.064565
O 1.340161 0.847227 -1.351259
O 2.451457 2.049309 0.525150
C 3.423400 -0.289814 -0.221695
F 4.047830 -0.402241 0.935516
F 2.986500 -1.488792 -0.599333
F 4.254282 0.178233 -1.132897

IBamideAc_OTf.log
Energy (E) = -1524.31453766 Hartree
Enthalpy (H) = -1524.120743 Hartree
Gibbs free energy (G) = -1524.196409
Hartree
Charge = 0, Spin = 1
C 5.195825 -0.389274 -0.001058
C 5.263398 0.997387 -0.000974
C 4.097129 1.743543 -0.000638
C 2.849822 1.125726 -0.000382
C 2.817618 -0.258795 -0.000477
C 3.963106 -1.032111 -0.000810
H 6.099283 -0.982960 -0.001319
H 6.224031 1.492760 -0.001170
H 4.101223 2.825408 -0.000561
H 3.929059 -2.110090 -0.000883
C 1.623860 2.006400 -0.000022
O 1.734855 3.212460 0.000024
I 0.906870 -1.195943 -0.000102
N 0.498491 1.240201 0.000228
C -0.728447 1.781527 0.000464
O -1.176548 2.882344 0.000417
C 1.715150 -3.203049 -0.000546
H 2.295960 -3.369114 -0.900968
H 2.296325 -3.369368 0.899594
H 0.835094 -3.840940 -0.000457
O -1.637971 0.641504 0.000895
S -3.202611 0.874580 0.000465
O -3.649411 1.387573 1.251431
O -3.648723 1.387507 -1.250776
C -3.557903 -0.935103 0.000409
F -3.039041 -1.507301 -1.077109
F -3.039567 -1.507282 1.078180
F -4.866695 -1.101415 0.000085

IBCCF32O_OTf.log
Energy (E) = -1991.47076951 Hartree
Enthalpy (H) = -1991.294914 Hartree
Gibbs free energy (G) = -1991.375125
Hartree
Charge = 0, Spin = 1
C 0.516082 3.478267 0.260869
C 1.890044 3.439078 0.071066
C 2.551681 2.226824 -0.058523
C 1.833184 1.036565 -0.012998
C 0.466367 1.119102 0.172201
C -0.221708 2.301956 0.321494
H 0.004615 4.424297 0.364758

H 2.454679 4.359431 0.028129
H 3.622723 2.201491 -0.189859
H -1.286675 2.333328 0.488961
O 1.479758 -1.317647 -0.307556
I -0.420842 -0.786553 0.236122
C 2.447366 -0.347584 -0.120930
C 3.373060 -0.457171 -1.357047
C 3.206092 -0.675506 1.187743
F 2.316726 -0.704374 2.185699
F 4.125074 0.240097 1.487906
F 3.797818 -1.858379 1.127154
F 4.492154 0.257084 -1.202866
F 3.719115 -1.713021 -1.584960
F 2.732834 0.002225 -2.427473
O -2.305356 0.173100 0.869060
S -3.311659 0.692894 -0.159148
O -4.000146 1.837540 0.349387
O -2.729457 0.711996 -1.472080
C -4.503098 -0.706131 -0.154513
F -3.841538 -1.827187 -0.438165
F -5.068881 -0.829354 1.031768
F -5.430748 -0.505963 -1.070925

IBCH2S_OTf.log
Energy (E) = -1719.16057517 Hartree
Enthalpy (H) = -1718.944807 Hartree
Gibbs free energy (G) = -1719.016641 Hartree
Charge = 0, Spin = 1
C -1.201518 3.243404 -0.281699
C -2.568871 3.310593 -0.066642
C -3.309631 2.152600 0.132224
C -2.699852 0.901332 0.117162
C -1.329918 0.893361 -0.097711
C -0.553106 2.011877 -0.296599
H -0.625084 4.141533 -0.450638
H -3.068358 4.269368 -0.058615
H -4.375618 2.221048 0.300222
H 0.511744 1.947397 -0.470375
I -0.483784 -1.066297 -0.146857
C -3.472629 -0.372770 0.400484
S -2.771057 -1.696477 -0.644018
C -4.934959 -0.291506 -0.024959
H -5.398547 -1.274415 0.065143
H -5.480290 0.386303 0.632560
H -5.030093 0.053061 -1.053537
C -3.380079 -0.721229 1.884003
H -3.821554 0.085605 2.471767
H -3.921841 -1.644947 2.085103
H -2.346659 -0.845088 2.206871
O 1.562127 0.071535 0.533142
S 2.639493 0.132451 -0.505436
O 2.682564 -1.063081 -1.309835
O 2.688346 1.409625 -1.171215
C 4.136728 0.075599 0.558157
F 4.153948 1.111210 1.385808
F 4.149887 -1.043564 1.270646
F 5.220678 0.113920 -0.202792

IBNMeCO2_OTf.log
Energy (E) = -1486.25530973 Hartree
Enthalpy (H) = -1486.066067 Hartree
Gibbs free energy (G) = -1486.136729 Hartree
Charge = 0, Spin = 1
C -1.221646 3.255246 -0.619870
C -2.527697 3.338284 -0.156204
C -3.213622 2.207299 0.256226
C -2.605268 0.949998 0.228539
C -1.293206 0.916524 -0.233470
C -0.589893 2.021107 -0.665570
H -0.690790 4.138936 -0.941622
H -3.027058 4.296541 -0.121599
H -4.234885 2.290707 0.597835
H 0.432401 1.932779 -1.007977
I -0.448120 -0.990687 -0.277566
C -3.326902 -1.388933 -0.033752
O -4.223405 -2.178219 0.097021
N -3.283267 -0.192986 0.687897
C -4.386424 -0.006775 1.623755
H -4.105832 0.748743 2.353594
H -5.305791 0.291316 1.117096
H -4.571653 -0.951127 2.123853
O -2.317808 -1.581748 -0.888283
O 1.404423 0.038342 0.426026
S 2.615402 0.028165 -0.498686
O 2.724717 -1.232373 -1.179735
O 2.725611 1.250160 -1.242726
C 3.943299 0.069970 0.773471
F 3.839988 1.167952 1.502458
F 3.845809 -0.989713 1.559657
F 5.115341 0.056560 0.161920

Indole_NMe_IBA_A_OTf.log
Energy (E) = -1562.39947636 Hartree
Enthalpy (H) = -1562.196760 Hartree
Gibbs free energy (G) = -1562.269664 Hartree
Charge = 0, Spin = 1
C -1.605449 1.258289 -0.233617
C -2.988498 1.324040 0.085214
C -3.675101 2.538262 0.144177
C -2.961358 3.684545 -0.124008
C -1.593605 3.635313 -0.450392
C -0.908278 2.443978 -0.510857
C -1.324954 -0.130273 -0.181209
H -4.727606 2.570293 0.391326
H -3.460760 4.642786 -0.086051
H -1.068274 4.556561 -0.658380
H 0.137489 2.432316 -0.775333
N -3.482616 0.059696 0.301740
C -2.456736 -0.823483 0.133760
C -2.457233 -2.291557 0.228366
O -1.242560 -2.788445 -0.031904
O -3.402670 -2.988850 0.492513
I 0.263748 -1.417560 -0.426469
C -4.858879 -0.244558 0.645742
H -5.525961 0.112135 -0.138393
H -4.962028 -1.319262 0.749042
H -5.122976 0.239162 1.585524
O 1.569576 0.300622 -0.881904
S 2.300172 1.031723 0.247577
O 2.649194 2.355562 -0.161192
O 1.658488 0.787298 1.507833
C 3.862050 0.062345 0.282632
F 3.558714 -1.229421 0.435384
F 4.526668 0.211639 -0.848017
F 4.612290 0.451155 1.294863

NpthIBA_C_OTf.log
Energy (E) = -1545.16914125 Hartree
Enthalpy (H) = -1544.978515 Hartree
Gibbs free energy (G) = -1545.049451 Hartree
Charge = 0, Spin = 1
C 3.473716 -0.109536 0.147097
C 2.408674 -0.964213 0.050423
C 1.132626 -0.416898 -0.123833
C 0.864140 0.906358 -0.209391
H 4.462747 -0.529627 0.284476
H -0.134538 1.292574 -0.340552
C 2.569211 -2.441370 0.129935
O 1.410549 -3.105553 0.017862
O 3.619718 -3.001041 0.277671
I -0.284188 -1.967098 -0.248858
C 1.768700 3.196610 -0.178953
C 2.835134 4.046749 -0.079641
C 4.143060 3.541095 0.094848
C 4.359638 2.193685 0.168715
C 3.277713 1.285947 0.070235
C 1.963131 1.797960 -0.108309
H 0.763115 3.575603 -0.308148
H 2.680426 5.115575 -0.132696
H 4.973863 4.228688 0.171683
H 5.357790 1.797818 0.304551
O -1.852191 -0.435739 -0.623166
S -2.656910 0.092857 0.565146
O -3.770208 -0.747230 0.880063
O -1.779618 0.547963 1.608610
C -3.335399 1.604032 -0.236906
F -2.334700 2.391693 -0.625173
F -4.065948 1.284994 -1.288123
F -4.079232 2.254442 0.639318

PyIBA_D_OTf.log
Energy (E) = -1407.68731765 Hartree
Enthalpy (H) = -1407.558362 Hartree
Gibbs free energy (G) = -1407.623054 Hartree
Charge = 0, Spin = 1
C -1.254481 3.052925 0.116873
C -2.633533 3.078365 -0.089969
C -2.775183 0.811577 -0.071843
C -1.414097 0.724737 0.127018
H -0.696682 3.975460 0.182827
H -3.151824 4.024331 -0.184611
C -3.580793 -0.453928 -0.163389
O -2.823355 -1.555097 -0.028171
O -4.760804 -0.498493 -0.332610
I -0.807833 -1.275495 0.256594
C -0.604511 1.834110 0.232349
H 0.460259 1.763584 0.386850
N -3.383048 1.987659 -0.181317
O 1.262047 -0.555162 0.638624
S 2.192954 -0.404828 -0.567102
O 2.787938 -1.647405 -0.948270
O 1.589106 0.439846 -1.562029
C 3.500722 0.601501 0.247255
F 2.967884 1.732001 0.702605
F 4.035956 -0.062099 1.253517
F 4.432743 0.889682 -0.641882

PyrroleNMeIBA_A_OTf.log
Energy (E) = -1408.89409894 Hartree
Enthalpy (H) = -1408.741151 Hartree
Gibbs free energy (G) = -1408.809274 Hartree
Charge = 0, Spin = 1
C -2.472454 2.485244 0.252641
C -1.220262 1.931845 0.457169
C -1.428834 0.566315 0.258941
C -2.735725 0.315446 -0.048584
H -2.774959 3.517817 0.301270
H -0.310082 2.449607 0.704357
C -3.224683 -1.035712 -0.309088
I -0.349278 -1.176249 0.278543
O -4.355830 -1.344977 -0.582205
O -2.226980 -1.931923 -0.202107
N -3.380071 1.515080 -0.050929
C -4.792634 1.712211 -0.334835

H -5.029627 1.347646 -1.330792
H -5.400409 1.169001 0.383968
H -5.006734 2.775415 -0.270524
O 1.372591 0.047669 0.913219
S 2.133284 0.919633 -0.086242
O 1.514847 0.863901 -1.381593
O 2.484684 2.175108 0.500302
C 3.682666 -0.058162 -0.223713
F 4.302959 -0.109215 0.941114
F 3.376049 -1.295178 -0.606749
F 4.476389 0.491135 -1.124452

QuinoIBA_L_OTf.log
Energy (E) = -1561.20142990 Hartree
Enthalpy (H) = -1561.023000 Hartree
Gibbs free energy (G) = -1561.093755 Hartree
Charge = 0, Spin = 1
C 2.262967 4.131205 0.150961
C 1.314158 3.151093 0.272130
C 1.691961 1.800955 0.148655
C 3.051339 1.463073 -0.099584
C 3.615320 3.806825 -0.094830
H 1.975940 5.169658 0.242281
H 0.273738 3.375221 0.454981
H 4.344211 4.599790 -0.186675
C 1.139421 -0.367794 0.139992
N 0.740452 0.823815 0.269654
C 2.427783 -0.858237 -0.093120
C 4.003755 2.501410 -0.217925
H 5.037212 2.241927 -0.407121
C 2.678153 -2.322469 -0.174885
O 3.765835 -2.794377 -0.368820
O 1.580342 -3.052009 -0.000169
C 3.398527 0.099722 -0.214253
H 4.421448 -0.209067 -0.395575
I -0.221135 -2.036331 0.280720
O -1.858221 -0.637476 0.635734
S -2.601759 -0.031587 -0.561179
O -1.693280 0.253649 -1.635908
O -3.828681 -0.717741 -0.822229
C -3.084077 1.576668 0.194901
F -3.833818 1.368100 1.262370
F -2.014688 2.276687 0.545174
F -3.774960 2.263616 -0.700423

FuranIBA_A_OMe.log
Energy (E) = -543.398055530 Hartree
Enthalpy (H) = -543.280701 Hartree
Gibbs free energy (G) = -543.329809 Hartree
Charge = 0, Spin = 1
C -1.753696 2.539282 -0.147113
C -0.438048 2.207728 -0.253338
C -0.445128 0.798406 -0.116290
C -1.715372 0.382721 0.050657
O -2.533153 1.447862 0.035953
H -2.263745 3.484863 -0.185261
H 0.398400 2.862283 -0.418834
C -2.080281 -1.049389 0.191406
I 0.928279 -0.721326 -0.142846
O -3.204446 -1.455368 0.345074
O -0.985344 -1.776391 0.117256
O 2.410232 0.656787 -0.358913
C 2.821659 1.235424 0.859883
H 3.245056 0.497161 1.547688
H 3.591715 1.971329 0.630326
H 1.996681 1.746467 1.370726

IBamideAc_OMe.log
Energy (E) = -678.253897235 Hartree
Enthalpy (H) = -678.052849 Hartree
Gibbs free energy (G) = -678.112912 Hartree
Charge = 0, Spin = 1
C 3.281108 1.574785 -0.212450
C 2.656648 2.804526 -0.023023
C 1.279473 2.877254 0.107435
C 0.516517 1.715548 0.057315
C 1.171107 0.516503 -0.114525
C 2.537421 0.401105 -0.262239
H 4.354987 1.523571 -0.327062
H 3.250340 3.707234 0.013850
H 0.755324 3.813719 0.243547
H 2.995926 -0.562436 -0.428927
C -0.977794 1.785083 0.162544
O -1.536454 2.842437 0.340613
I -0.095257 -1.158667 -0.162260
N -1.543304 0.534481 0.024328
C -3.331662 -1.129142 -0.076872
H -4.415058 -1.184802 -0.050830
H -2.975563 -1.563228 -1.013350
H -2.924254 -1.712790 0.751281
C -2.924682 0.325700 0.034771
O -3.747073 1.202914 0.118615
O 1.567515 -2.379116 -0.283236
C 2.090881 -2.732622 0.974715
H 1.365536 -3.275202 1.590394
H 2.950301 -3.384272 0.813318
H 2.428203 -1.856138 1.543284

IBCCF32O_OMe.log
Energy (E) = -1145.40386361 Hartree
Enthalpy (H) = -1145.221304 Hartree
Gibbs free energy (G) = -1145.287033 Hartree
Charge = 0, Spin = 1
C -1.105292 3.339255 0.008848
C 0.266529 3.443341 -0.180106
C 1.068692 2.311046 -0.184323
C 0.499786 1.054815 0.005676
C -0.866947 0.988937 0.170804
C -1.693100 2.092671 0.183158
H -1.722976 4.226236 0.022634
H 0.718639 4.414923 -0.321852
H 2.133733 2.400881 -0.334669
H -2.755354 1.977644 0.341702
O 0.486358 -1.283521 0.498646
I -1.626549 -0.959474 0.400367
C 1.267321 -0.264226 0.039690
C 1.733648 -0.605260 -1.396317
C 2.470212 -0.167934 1.008599
F 2.053228 0.298207 2.185073
F 3.428326 0.655494 0.561130
F 3.023575 -1.354065 1.207481
F 2.457243 0.367509 -1.957393
F 2.448300 -1.721269 -1.431821
F 0.647465 -0.785501 -2.157557
O -3.525684 -0.213067 0.264946
C -3.978553 -0.070204 -1.062292
H -4.993443 0.326403 -1.028064
H -3.354761 0.625996 -1.636506
H -4.001136 -1.025279 -1.596061

IBCH2S_OMe.log
Energy (E) = -873.070510229 Hartree
Enthalpy (H) = -872.848386 Hartree
Gibbs free energy (G) = -872.905079 Hartree
Charge = 0, Spin = 1
C -0.403837 3.266599 -0.075557
C -1.773996 3.070377 -0.148713
C -2.300702 1.786339 -0.117998
C -1.476755 0.664131 -0.040706
C -0.113119 0.910332 -0.002680
C 0.445928 2.169971 0.008760
H 0.011767 4.264382 -0.085993
H -2.440761 3.918913 -0.218789
H -3.372909 1.652223 -0.147773
H 1.514969 2.284064 0.102347
I 1.164447 -0.794594 0.056615
C -2.049521 -0.734463 0.118147
S -0.972307 -1.903432 -0.759308
C -3.435970 -0.877415 -0.500622
H -3.734100 -1.925280 -0.468841
H -4.176778 -0.306797 0.062645
H -3.438566 -0.545781 -1.538262
C -2.122219 -1.066757 1.609582
H -2.775600 -0.354928 2.119777
H -2.518446 -2.073493 1.741083
H -1.137534 -1.020462 2.075783
O 2.743204 0.518095 0.635039
C 3.618227 0.793122 -0.421639
H 4.465396 1.365334 -0.035922
H 4.023480 -0.113864 -0.889551
H 3.146447 1.385926 -1.219893

IBNMeCO2_OMe.log
Energy (E) = -640.184506579 Hartree
Enthalpy (H) = -639.988684 Hartree
Gibbs free energy (G) = -640.044933 Hartree
Charge = 0, Spin = 1
C -0.167722 3.290615 -0.382385
C -1.543886 3.202525 -0.223503
C -2.158329 1.981028 -0.006896
C -1.420717 0.791654 0.060035
C -0.042476 0.924498 -0.108777
C 0.590321 2.131610 -0.320840
H 0.313333 4.244193 -0.543736
H -2.151854 4.095573 -0.270709
H -3.231189 1.936028 0.108062
H 1.666307 2.169051 -0.403693
I 1.101099 -0.817217 0.032580
C -1.780591 -1.611106 -0.377145
O -2.603727 -2.496227 -0.402435
N -2.051428 -0.422495 0.345140
C -3.349748 -0.383098 1.000847
H -3.329058 0.363681 1.792257
H -4.159391 -0.155043 0.303801
H -3.543465 -1.363102 1.422472
O -0.626261 -1.635119 -0.986849
O 2.612562 0.277303 0.874617
C 3.739095 0.411878 0.041129
H 4.485223 0.990293 0.586099
H 4.186737 -0.552961 -0.220439
H 3.508811 0.936947 -0.894308

Indole_NMe_IBA_A_OMe.log
Energy (E) = -716.334393739 Hartree
Enthalpy (H) = -716.124810 Hartree
Gibbs free energy (G) = -716.183546 Hartree
Charge = 0, Spin = 1
C 1.246960 -0.779495 -0.114168
C 2.345840 0.108625 0.022110
C 3.664202 -0.348807 0.024074
C 3.869375 -1.704276 -0.122889
C 2.791707 -2.594267 -0.277147
C 1.487019 -2.150941 -0.276224

C 0.115460 0.076445 -0.064312
H 4.491760 0.339560 0.132423
H 4.879051 -2.091465 -0.127573
H 2.995063 -3.648500 -0.404926
H 0.661962 -2.833199 -0.419513
N 1.885527 1.400080 0.127100
C 0.520704 1.369205 0.065915
C -0.458056 2.489695 0.073988
O -1.675143 2.039987 -0.077615
O -0.142727 3.655202 0.197221
I -1.928774 -0.146372 -0.175968
C 2.731012 2.571389 0.255340
H 3.399006 2.645339 -0.602759
H 2.094372 3.448683 0.300523
H 3.328173 2.503374 1.164585
O -1.742402 -2.171841 -0.165319
C -1.595107 -2.703715 1.133856
H -1.476219 -3.782809 1.035674
H -0.708511 -2.304713 1.640312
H -2.470856 -2.509055 1.760317

NpthIBA_C_OMe.log
Energy (E) = -699.102909072 Hartree
Enthalpy (H) = -698.905345 Hartree
Gibbs free energy (G) = -698.962129 Hartree
Charge = 0, Spin = 1
C -1.692106 1.642289 -0.154918
C -0.354444 1.364987 -0.079684
C 0.036996 0.033182 0.084170
C -0.821176 -1.011753 0.193518
H -1.996575 2.674615 -0.278620
H -0.467957 -2.021811 0.352107
C 0.692732 2.441286 -0.135178
O 1.908404 1.976002 0.005134
O 0.399149 3.604329 -0.285452
I 2.126596 -0.178948 0.168859
C -3.172416 -1.766310 0.221508
C -4.507177 -1.480058 0.144426
C -4.944743 -0.149006 -0.038801
C -4.037155 0.867977 -0.139412
C -2.647608 0.605908 -0.063865
C -2.209971 -0.734547 0.118060
H -2.831022 -2.783938 0.363030
H -5.236438 -2.274679 0.224195
H -6.003889 0.060772 -0.097684
H -4.362816 1.891077 -0.277561
O 1.906404 -2.205808 0.266257
C 1.832074 -2.821256 -1.000634
H 2.744062 -2.671979 -1.586586
H 1.699846 -3.891655 -0.842675
H 0.983485 -2.450556 -1.588368

PyIBA_D_OMe.log
Energy (E) = -561.621299058 Hartree
Enthalpy (H) = -561.485418 Hartree
Gibbs free energy (G) = -561.535957 Hartree
Charge = 0, Spin = 1
C -1.706382 2.630788 -0.204074
C -2.866450 1.885467 0.000323
C -1.719392 -0.079646 0.072931
C -0.535177 0.604529 -0.107455
H -1.760739 3.703690 -0.318486
H -3.827634 2.382301 0.054092
C -1.676834 -1.590164 0.182775
O -0.454347 -2.058119 0.057633
O -2.664917 -2.251908 0.356018
I 1.149961 -0.632183 -0.158807
C -0.487562 1.972915 -0.263561
H 0.447624 2.485311 -0.438994
N -2.881791 0.564503 0.130631
O 2.331199 1.030370 -0.318360
C 2.699449 1.576571 0.928441
H 3.287445 0.876736 1.529843
H 3.310292 2.459069 0.738437
H 1.826199 1.881573 1.518857

PyrroleNMeIBA_A_OMe.log
Energy (E) = -562.826569766 Hartree
Enthalpy (H) = -562.666800 Hartree
Gibbs free energy (G) = -562.720249 Hartree
Charge = 0, Spin = 1
C -1.862958 2.219031 -0.201718
C -0.493126 2.055724 -0.288582
C -0.304699 0.675507 -0.138838
C -1.503576 0.047551 0.023827
H -2.452180 3.118669 -0.263580
H 0.249207 2.816012 -0.452247
C -1.613606 -1.417701 0.166220
I 1.297171 -0.611810 -0.136257
O -2.671127 -1.996309 0.311438
O -0.428487 -1.974638 0.107860
N -2.466827 1.009439 -0.014168
C -3.895398 0.773570 0.112973
H -4.236704 0.104022 -0.671871
H -4.116838 0.310652 1.070813
H -4.406071 1.730038 0.034933
O 2.557793 0.973973 -0.338417
C 2.843781 1.618583 0.882409
H 3.374109 0.964711 1.581910
H 3.482883 2.473217 0.660906
H 1.935406 1.984745 1.375114

QuinoIBA_L_OMe.log
Energy (E) = -715.136511185 Hartree
Enthalpy (H) = -714.951104 Hartree
Gibbs free energy (G) = -715.008158 Hartree
Charge = 0, Spin = 1
C -4.384588 -1.522858 0.120021
C -3.037325 -1.761123 0.167025
C -2.132084 -0.683806 0.089029
C -2.623289 0.642800 -0.037148
C -4.881519 -0.206829 -0.005854
H -5.079945 -2.348845 0.180962
H -2.632893 -2.758486 0.266362
H -5.949439 -0.041901 -0.040600
C -0.016990 0.075321 0.065544
N -0.788390 -0.937098 0.137371
C -0.350573 1.427747 -0.063676
C -4.020854 0.853437 -0.082448
H -4.390888 1.866096 -0.178262
C 0.722124 2.476833 -0.132342
O 0.434938 3.647152 -0.249691
O 1.919858 1.976216 -0.052379
C -1.689580 1.700260 -0.113125
H -2.008051 2.731756 -0.209912
I 2.107569 -0.218903 0.146634
O 1.866619 -2.214588 0.327539
C 1.642245 -2.885576 -0.893772
H 2.470557 -2.748385 -1.596354
H 1.556596 -3.947979 -0.667373
H 0.714894 -2.558171 -1.371315

FuranIBA_A_OCF3.log
Energy (E) = -841.057727195 Hartree
Enthalpy (H) = -840.961469 Hartree
Gibbs free energy (G) = -841.015884 Hartree
Charge = 0, Spin = 1
C -1.730586 2.788105 -0.185582
C -0.530421 2.176366 -0.381747
C -0.836278 0.808335 -0.203043
C -2.148644 0.683118 0.076816
O -2.713794 1.898776 0.091699
H -2.022316 3.822455 -0.209900
H 0.416260 2.629795 -0.610123
C -2.788644 -0.624098 0.317436
I 0.111180 -1.002996 -0.258713
O -3.945768 -0.811828 0.570331
O -1.867580 -1.586109 0.208800
O 1.876537 0.050043 -0.805615
C 2.694230 0.365033 0.181111
F 3.762064 1.006938 -0.276907
F 2.107118 1.164633 1.101024
F 3.126417 -0.713979 0.862564

IBamideAc_OCF3.log
Energy (E) = -975.918324091 Hartree
Enthalpy (H) = -975.738436 Hartree
Gibbs free energy (G) = -975.804278 Hartree
Charge = 0, Spin = 1
C -0.479561 -3.608123 -0.218324
C 0.840424 -3.957594 0.052867
C 1.807950 -2.977770 0.198440
C 1.454451 -1.638746 0.074406
C 0.136589 -1.335160 -0.186595
C -0.857132 -2.275586 -0.344860
H -1.230513 -4.377010 -0.333960
H 1.109972 -4.999924 0.148276
H 2.844463 -3.206541 0.406861
H -1.874514 -1.990685 -0.559563
C 2.484019 -0.567084 0.221005
O 3.638062 -0.820349 0.455972
I -0.183096 0.735573 -0.339835
N 1.927475 0.697947 0.051044
C 1.862871 3.150036 -0.056224
H 2.532561 3.999107 0.030956
H 1.392764 3.171269 -1.041668
H 1.083489 3.233653 0.703880
C 2.673731 1.888103 0.132154
O 3.858002 1.911790 0.333561
O -2.286118 0.298659 -0.767086
C -3.103563 0.465236 0.241168
F -2.813791 -0.346622 1.290746
F -4.367680 0.216722 -0.100258
F -3.067941 1.720841 0.746775

IBCCF32O_OCF3.log
Energy (E) = -1443.06416645 Hartree
Enthalpy (H) = -1442.902643 Hartree
Gibbs free energy (G) = -1442.974227 Hartree
Charge = 0, Spin = 1
C -0.460406 3.382558 0.324113
C 0.914450 3.460938 0.150792
C 1.675739 2.310645 0.003516
C 1.058775 1.063352 0.015841
C -0.310844 1.028354 0.185887
C -1.096203 2.147407 0.351105
H -1.047961 4.281781 0.442527
H 1.401749 4.425465 0.135425
H 2.746276 2.377993 -0.114891
H -2.160461 2.065617 0.500233
O 0.905196 -1.308056 -0.289342
I -1.068927 -0.932703 0.227223
C 1.785895 -0.266037 -0.113202

C 2.699682 -0.278694 -1.361805
C 2.587113 -0.533469 1.183334
F 1.718995 -0.614809 2.197208
F 3.451144 0.441894 1.466480
F 3.255679 -1.674923 1.120213
F 3.736825 0.556518 -1.237956
F 3.179760 -1.489841 -1.592563
F 1.992549 0.099763 -2.423930
O -2.977914 -0.122966 0.760658
C -3.870473 -0.077144 -0.206366
F -4.120586 -1.288209 -0.744052
F -3.477230 0.704086 -1.239595
F -5.026817 0.410313 0.234646

IBCH2S_OCF3.log
Energy (E) = -1170.74499714 Hartree
Enthalpy (H) = -1170.543788 Hartree
Gibbs free energy (G) = -1170.606158 Hartree
Charge = 0, Spin = 1
C -0.621810 3.267619 -0.122844
C -2.007166 3.226178 -0.119802
C -2.674771 2.010419 -0.053275
C -1.975034 0.807545 0.002182
C -0.591965 0.904114 -0.009425
C 0.110584 2.086983 -0.060616
H -0.099389 4.211797 -0.180979
H -2.576307 4.144064 -0.170656
H -3.755810 1.995029 -0.043404
H 1.188710 2.090002 -0.057726
I 0.426122 -0.964639 0.043211
C -2.683983 -0.525489 0.157814
S -1.743330 -1.775204 -0.778169
C -4.078361 -0.532196 -0.459305
H -4.477896 -1.546488 -0.442863
H -4.755695 0.095101 0.121765
H -4.056104 -0.178656 -1.489150
C -2.773350 -0.888369 1.639150
H -3.348707 -0.125491 2.167468
H -3.268813 -1.851911 1.755383
H -1.787355 -0.946840 2.099934
O 2.214861 0.239599 0.864045
C 3.242202 0.234774 0.084805
F 4.334144 0.775022 0.645945
F 3.041693 0.926229 -1.080588
F 3.607412 -1.014997 -0.327992

IBNMeCO2_OCF3.log
Energy (E) = -937.848079113 Hartree
Enthalpy (H) = -937.673169 Hartree
Gibbs free energy (G) = -937.734503 Hartree
Charge = 0, Spin = 1
C -0.593513 3.283832 -0.452372
C -1.959472 3.269300 -0.204625
C -2.622989 2.084370 0.067623
C -1.937222 0.866565 0.111274
C -0.567769 0.929040 -0.133624
C 0.115219 2.092407 -0.418793
H -0.078996 4.209146 -0.665679
H -2.522172 4.192150 -0.231015
H -3.688754 2.093075 0.242472
H 1.180368 2.071138 -0.591683
I 0.425652 -0.901972 -0.052835
C -2.444698 -1.504663 -0.314255
O -3.304038 -2.347880 -0.334790
N -2.603897 -0.325262 0.432186
C -3.857533 -0.233399 1.169320
H -3.754161 0.514056 1.952641
H -4.696990 0.025118 0.521113
H -4.063822 -1.202634 1.609863
O -1.308214 -1.597594 -0.988276
O 2.038836 0.162771 0.889197
C 3.186478 0.127068 0.254992
F 3.575268 -1.133546 -0.049151
F 3.152812 0.781799 -0.933128
F 4.157316 0.679950 0.974608

Indole_NMe_IBA_A_OCF3.log
Energy (E) = -1013.99359246 Hartree
Enthalpy (H) = -1013.805144 Hartree
Gibbs free energy (G) = -1013.869486 Hartree
Charge = 0, Spin = 1
C -1.000737 1.203527 -0.221100
C -2.398007 1.151913 0.027707
C -3.185398 2.304510 0.050115
C -2.557632 3.507594 -0.185501
C -1.175873 3.573999 -0.441883
C -0.391192 2.443508 -0.462569
C -0.604844 -0.156688 -0.152756
H -4.248113 2.248413 0.243934
H -3.137266 4.420361 -0.175983
H -0.721604 4.537352 -0.626461
H 0.666076 2.506498 -0.669036
N -2.791653 -0.151645 0.219734
C -1.686807 -0.944441 0.102447
C -1.563520 -2.413745 0.191430
O -0.310439 -2.795189 -0.015380
O -2.471384 -3.177763 0.414814
I 1.113479 -1.270852 -0.355576
C -4.152153 -0.574621 0.492180
H -4.806377 -0.276018 -0.326499
H -4.164614 -1.654568 0.592628
H -4.506742 -0.117335 1.415481
O 2.194648 0.527995 -0.693597
C 2.797655 1.043489 0.360691
F 1.956657 1.233236 1.400503
F 3.329528 2.226901 0.062746
F 3.786510 0.259566 0.830312

NpthIBA_C_OCF3.log
Energy (E) = -996.763502753 Hartree
Enthalpy (H) = -996.587077 Hartree
Gibbs free energy (G) = -996.649472 Hartree
Charge = 0, Spin = 1
C 2.515117 1.370397 0.245201
C 1.159303 1.505467 0.117800
C 0.400491 0.362023 -0.153967
C 0.912747 -0.882086 -0.312699
H 3.103707 2.254631 0.457818
H 0.291885 -1.737713 -0.533307
C 0.475665 2.826126 0.257504
O -0.839008 2.750722 0.090975
O 1.060803 3.850326 0.493732
I -1.637141 0.855503 -0.307004
C 2.933640 -2.295187 -0.329274
C 4.288818 -2.423649 -0.200505
C 5.093063 -1.296251 0.080300
C 4.524902 -0.061978 0.226883
C 3.124708 0.105559 0.100000
C 2.317692 -1.030476 -0.182738
H 2.312253 -3.155226 -0.542767
H 4.752605 -3.393985 -0.312961
H 6.163062 -1.416202 0.178997
H 5.132634 0.807452 0.442214
O -2.039744 -1.198495 -0.747538
C -2.494377 -1.925907 0.251869
F -1.631499 -1.975719 1.292794
F -2.715408 -3.179786 -0.133991
F -3.648059 -1.449526 0.761377

PyIBA_D_OCF3.log
Energy (E) = -859.281880609 Hartree
Enthalpy (H) = -859.167133 Hartree
Gibbs free energy (G) = -859.223287 Hartree
Charge = 0, Spin = 1
C -1.418169 2.931318 0.285134
C -2.728558 2.571707 -0.028099
C -2.207054 0.360922 -0.114012
C -0.893474 0.655766 0.183763
H -1.159788 3.969077 0.437117
H -3.492841 3.333022 -0.123436
C -2.601609 -1.080392 -0.313749
O -1.571548 -1.910496 -0.159330
O -3.712480 -1.436742 -0.577396
I 0.284826 -1.068783 0.285868
C -0.452844 1.943354 0.399401
H 0.574648 2.162469 0.644677
N -3.121397 1.319312 -0.222055
O 1.947425 0.189100 0.783457
C 2.761551 0.480595 -0.210698
F 3.754366 1.263317 0.199235
F 3.310345 -0.612946 -0.773678
F 2.131485 1.130874 -1.218805

PyrroleNMeIBA_A_OCF3.log
Energy (E) = -860.487110837 Hartree
Enthalpy (H) = -860.348448 Hartree
Gibbs free energy (G) = -860.407550 Hartree
Charge = 0, Spin = 1
C -1.889520 2.449233 -0.289012
C -0.599189 1.977750 -0.449698
C -0.719720 0.600590 -0.247754
C -2.015818 0.265983 0.018870
H -2.260740 3.458236 -0.354550
H 0.284998 2.545991 -0.671966
C -2.426642 -1.118259 0.273183
I 0.495083 -1.053156 -0.250783
O -3.554249 -1.476660 0.515082
O -1.379212 -1.937027 0.201204
N -2.738411 1.419534 -0.007112
C -4.169198 1.521855 0.230081
H -4.714663 0.927695 -0.498219
H -4.413031 1.153030 1.222800
H -4.454041 2.566937 0.143286
O 2.104759 0.232033 -0.790534
C 2.859291 0.667447 0.198189
F 3.833364 1.448539 -0.258036
F 2.162178 1.385167 1.107245
F 3.435721 -0.334343 0.894208

QuinoIBA_L_OCF3.log
Energy (E) = -1012.79536601 Hartree
Enthalpy (H) = -1012.631152 Hartree
Gibbs free energy (G) = -1012.693650 Hartree
Charge = 0, Spin = 1
C -4.124677 -2.426447 0.229326
C -2.772981 -2.244591 0.349879
C -2.226484 -0.956910 0.187695
C -3.078200 0.144371 -0.099175
C -4.979415 -1.338488 -0.055034
H -4.547192 -3.414183 0.352119
H -2.097723 -3.060229 0.565052
H -6.043333 -1.507536 -0.145344
C -0.438914 0.394210 0.150302

N -0.874654 -0.784926 0.310862
C -1.142757 1.569976 -0.129725
C -4.469430 -0.079732 -0.216237
H -5.115905 0.760305 -0.434726
C -0.423691 2.871486 -0.266933
O -1.000421 3.901434 -0.508770
O 0.877727 2.760734 -0.092733
C -2.497473 1.422145 -0.254640
H -3.101739 2.295211 -0.471920
I 1.664415 0.825789 0.313682
O 2.011868 -1.203171 0.753846
C 2.337724 -1.978722 -0.264449
F 2.416828 -3.247695 0.120703
F 1.453739 -1.916421 -1.280755
F 3.529311 -1.644956 -0.803406

FuranIBA_A_NTf2.log
Energy (E) = -2254.87822849 Hartree
Enthalpy (H) = -2254.733208 Hartree
Gibbs free energy (G) = -2254.811775 Hartree
Charge = 0, Spin = 1
C 3.103483 -1.395116 2.346417
C 1.871837 -1.095899 1.848367
C 2.174118 -0.439477 0.634405
C 3.512644 -0.382638 0.483090
O 4.099496 -0.969991 1.534483
H 3.408526 -1.894852 3.247936
H 0.915892 -1.320621 2.288298
C 4.155798 0.249911 -0.677851
I 1.219023 0.449425 -0.941516
O 5.328825 0.346926 -0.890886
O 3.206690 0.729071 -1.506674
N -0.762493 -0.075444 0.114273
S -1.209438 -1.664242 0.094890
S -1.297765 1.004961 1.239340
O -0.302908 -2.290752 -0.821439
O -1.429076 -2.183614 1.406347
C -2.845249 -1.640159 -0.766377
O -0.153852 1.699249 1.749566
O -2.305876 0.453256 2.083533
C -2.142477 2.211194 0.125589
F -2.728516 -0.954000 -1.891924
F -3.763702 -1.092041 -0.000823
F -3.171146 -2.888042 -1.038007
F -2.556606 3.230161 0.847694
F -1.281730 2.639037 -0.794116
F -3.165771 1.637387 -0.476688

IBamideAc_NTf2.log
Energy (E) = -2389.74110718 Hartree
Enthalpy (H) = -2389.512463 Hartree
Gibbs free energy (G) = -2389.602684 Hartree
Charge = 0, Spin = 1
C -1.717251 3.533871 1.077948
C -3.106668 3.589043 1.029258
C -3.835639 2.479951 0.636385
C -3.171293 1.309639 0.286874
C -1.794553 1.298930 0.344421
C -1.029832 2.374259 0.733954
H -1.149005 4.399267 1.387791
H -3.616925 4.501849 1.301582
H -4.916354 2.477334 0.587785
H 0.047003 2.349382 0.783143
C -3.942663 0.104320 -0.125849
O -5.142622 0.091360 -0.190709
I -1.037644 -0.579423 -0.243900
N -3.098813 -0.976272 -0.413595
C -2.463347 -3.259964 -1.078130
H -2.930364 -4.197207 -1.361526
H -1.825390 -2.921503 -1.897220
H -1.842889 -3.428436 -0.195315
C -3.556995 -2.258958 -0.792380
O -4.722520 -2.527309 -0.877204
N 1.229692 0.216432 0.080884
S 1.807498 1.253280 -1.041721
S 1.830670 0.063702 1.591961
O 0.820805 1.261250 -2.084306
O 2.296468 2.470991 -0.474550
C 3.265759 0.375027 -1.758384
O 0.719761 0.083818 2.499777
O 3.011780 0.833649 1.815739
C 2.359879 -1.703662 1.540803
F 2.907711 -0.851359 -2.109376
F 4.247811 0.318408 -0.883225
F 3.656772 1.042708 -2.827138
F 2.791845 -2.052209 2.735492
F 1.321334 -2.469943 1.208928
F 3.317687 -1.875259 0.649343

IBCCF32O_NTf2.log
Energy (E) = -2856.88418796 Hartree
Enthalpy (H) = -2856.673897 Hartree
Gibbs free energy (G) = -2856.769544 Hartree
Charge = 0, Spin = 1
C 1.254118 -1.240465 3.256396
C 2.634458 -1.334374 3.154279
C 3.289945 -0.894723 2.013821
C 2.562684 -0.349532 0.960717
C 1.189122 -0.284506 1.097526
C 0.505298 -0.711381 2.212133
H 0.745604 -1.578836 4.147490
H 3.208076 -1.753380 3.968550
H 4.363408 -0.976570 1.935978
H -0.567209 -0.639747 2.299236
O 2.253148 0.907391 -1.054366
I 0.284552 0.516073 -0.630284
C 3.168680 0.162372 -0.333369
C 4.343413 1.132918 -0.058465
C 3.616061 -1.036873 -1.203800
F 2.529709 -1.741483 -1.529254
F 4.451875 -1.847808 -0.557732
F 4.202122 -0.637036 -2.322478
F 5.411775 0.493908 0.427232
F 4.711309 1.761617 -1.162234
F 3.963759 2.042019 0.834394
N -1.816252 -0.028662 0.188105
S -2.212514 -1.629413 0.224461
S -2.594372 1.123211 1.069789
O -1.131373 -2.294656 -0.440302
O -2.671987 -2.044799 1.510851
C -3.648709 -1.751564 -0.932914
O -1.592868 1.925451 1.706550
O -3.724713 0.605285 1.769395
C -3.262824 2.175075 -0.292947
F -3.339903 -1.151137 -2.071500
F -4.717416 -1.192172 -0.407294
F -3.875219 -3.031698 -1.154108
F -3.841678 3.230312 0.240858
F -2.267775 2.567663 -1.082605
F -4.137260 1.495312 -1.009525

IBCH2S_NTf2.log
Energy (E) = -2584.57837186 Hartree
Enthalpy (H) = -2584.328143 Hartree
Gibbs free energy (G) = -2584.415179 Hartree
Charge = 0, Spin = 1
C -1.917227 2.589940 2.082394
C -3.211826 2.210891 2.399778
C -3.851933 1.210220 1.679835
C -3.209533 0.563527 0.628437
C -1.915247 0.976224 0.360313
C -1.242554 1.962933 1.039922
H -1.420147 3.375542 2.632601
H -3.733591 2.699650 3.210521
H -4.862453 0.925476 1.939076
H -0.241218 2.267740 0.778612
I -1.024339 -0.076751 -1.268542
C -3.844071 -0.582896 -0.132932
S -3.334779 -0.419659 -1.884366
C -5.365541 -0.503457 -0.184965
H -5.752321 -1.272144 -0.854813
H -5.781222 -0.696492 0.804322
H -5.702583 0.473067 -0.530044
C -3.401261 -1.912631 0.472905
H -3.782683 -1.977067 1.493397
H -3.800810 -2.742742 -0.108982
H -2.316153 -1.997779 0.527178
N 1.255284 0.118067 0.059162
S 2.095647 1.468393 -0.180704
S 1.312978 -0.805242 1.379577
O 1.499744 2.085292 -1.337631
O 2.320523 2.238970 1.006444
C 3.755912 0.895299 -0.743797
O -0.038028 -1.100937 1.781580
O 2.303701 -0.423271 2.337412
C 1.895510 -2.386103 0.630594
F 3.616664 0.054958 -1.758929
F 4.395101 0.294770 0.241327
F 4.446795 1.949914 -1.141550
F 1.952456 -3.310919 1.572765
F 1.040173 -2.779726 -0.310977
F 3.090338 -2.237210 0.085947

IBNMeCO2_NTf2.log
Energy (E) = -2351.66975830 Hartree
Enthalpy (H) = -2351.446102 Hartree
Gibbs free energy (G) = -2351.531684 Hartree
Charge = 0, Spin = 1
C -1.982711 2.940660 1.719107
C -3.211751 2.460092 2.150199
C -3.762224 1.311058 1.605460
C -3.083232 0.588377 0.622576
C -1.843450 1.090301 0.243466
C -1.285185 2.247028 0.741271
H -1.562034 3.843093 2.136959
H -3.760450 2.994465 2.913065
H -4.731432 0.967699 1.936436
H -0.332253 2.609646 0.385116
I -0.878704 -0.083882 -1.184625
C -3.680103 -0.818374 -1.299937
O -4.514266 -1.529591 -1.792199
N -3.622209 -0.588338 0.077616
C -4.607923 -1.321038 0.863868
H -4.280804 -1.344783 1.900339
H -5.600200 -0.872105 0.795568
H -4.670241 -2.331737 0.475491
O -2.755085 -0.183573 -2.029840
N 1.092395 0.130787 0.072820
S 2.043534 1.436149 -0.222938
S 1.159671 -0.753302 1.456848
O 1.478874 2.041742 -1.396539
O 2.317046 2.208717 0.945868
C 3.649392 0.710777 -0.780395
O -0.184960 -0.985277 1.894431
O 2.191761 -0.312262 2.337504

C 1.712123 -2.381452 0.781866
F 3.418646 -0.186657 -1.725560
F 4.280237 0.148591 0.228333
F 4.378970 1.693393 -1.272843
F 1.724143 -3.255403 1.768416
F 0.864495 -2.787820 -0.157358
F 2.919018 -2.281641 0.257664

Indole_NMe_IBA_A_NTf2.log
Energy (E) = -2427.81208414 Hartree
Enthalpy (H) = -2427.574869 Hartree
Gibbs free energy (G) = -2427.663859 Hartree
Charge = 0, Spin = 1
C -2.174201 1.238599 0.225523
C -3.589703 1.365777 0.217807
C -4.226508 2.585721 0.452533
C -3.430998 3.681424 0.698778
C -2.028916 3.573683 0.716405
C -1.393950 2.375209 0.487679
C -1.969917 -0.138409 -0.051378
H -5.305539 2.658980 0.441145
H -3.890073 4.642482 0.884593
H -1.434091 4.453403 0.916363
H -0.317082 2.336511 0.511497
N -4.171330 0.147854 -0.038505
C -3.173071 -0.766377 -0.199680
C -3.269293 -2.211313 -0.463844
O -2.057358 -2.768224 -0.526995
O -4.285968 -2.841786 -0.603502
I -0.428034 -1.493015 -0.292779
C -5.601739 -0.085606 -0.109787
H -6.037545 0.520269 -0.903459
H -5.773114 -1.135829 -0.318487
H -6.068625 0.179224 0.838292
N 1.166033 0.143984 -0.010458
S 1.534202 1.074203 -1.325285
S 1.613301 0.481893 1.536494
O 0.672719 0.597793 -2.365640
O 1.650032 2.460383 -1.011316
C 3.228974 0.501153 -1.784436
O 0.483202 0.202847 2.370160
O 2.365691 1.690541 1.628075
C 2.792496 -0.900061 1.879549
F 3.264275 -0.824195 -1.767059
F 4.114736 0.984009 -0.938633
F 3.489998 0.933046 -3.002131
F 3.125344 -0.848971 3.152540
F 2.199595 -2.062410 1.623294
F 3.870862 -0.794465 1.128611

NpthIBA_C_NTf2.log
Energy (E) = -2410.58407864 Hartree
Enthalpy (H) = -2410.358930 Hartree
Gibbs free energy (G) = -2410.445528 Hartree
Charge = 0, Spin = 1
C 4.212612 0.630747 -0.137314
C 2.993391 1.233787 -0.292650
C 1.839956 0.463377 -0.102382
C 1.841619 -0.847710 0.233694
H 5.104215 1.228747 -0.282414
H 0.946100 -1.431086 0.389221
C 2.871598 2.674961 -0.643397
O 1.602976 3.089692 -0.742219
O 3.805159 3.407688 -0.819589
I 0.139197 1.668440 -0.426225
C 3.184552 -2.851116 0.728274
C 4.404871 -3.450613 0.874834
C 5.595603 -2.711989 0.692058
C 5.544041 -1.386300 0.363765
C 4.297276 -0.735604 0.202768
C 3.101711 -1.481028 0.389991
H 2.267937 -3.410624 0.862627
H 4.463206 -4.499244 1.131802
H 6.550882 -3.203724 0.812801
H 6.450754 -0.812887 0.220726
N -1.241996 -0.152922 0.004618
S -1.343578 -1.306392 -1.167683
S -1.643269 -0.413954 1.579372
O -0.652017 -0.753933 -2.294685
O -1.043808 -2.612938 -0.673358
C -3.130490 -1.305494 -1.640488
O -0.586433 0.118073 2.386552
O -2.196189 -1.711594 1.794495
C -3.035250 0.786346 1.758954
F -3.515166 -0.062472 -1.881439
F -3.865032 -1.824336 -0.680125
F -3.251484 -2.027608 -2.737433
F -3.422773 0.789210 3.017464
F -2.619267 2.002537 1.420402
F -4.042222 0.443272 0.978639

PyIBA_D_NTf2.log
Energy (E) = -2273.10195273 Hartree
Enthalpy (H) = -2272.938463 Hartree
Gibbs free energy (G) = -2273.018931 Hartree
Charge = 0, Spin = 1
C 2.760287 -1.775452 2.295015
C 4.102953 -1.579321 1.975582
C 3.585253 -0.256214 0.201420
C 2.240876 -0.416735 0.464778
H 2.482772 -2.388127 3.139969
H 4.878373 -2.042365 2.572735
C 4.009000 0.600785 -0.958688
O 2.965088 1.126469 -1.616842
O 5.142059 0.805187 -1.272076
I 1.083727 0.637971 -0.932650
C 1.781396 -1.175446 1.518802
H 0.735176 -1.310186 1.747935
N 4.511557 -0.837028 0.955442
N -0.855863 -0.103859 0.118796
S -1.287928 -1.672928 -0.138073
S -1.394711 0.797568 1.386691
O -0.376586 -2.146836 -1.138008
O -1.492966 -2.385738 1.082542
C -2.929050 -1.546846 -0.979244
O -0.251023 1.437935 1.966049
O -2.372062 0.114161 2.169137
C -2.290347 2.131030 0.475614
F -2.834489 -0.691849 -1.984846
F -3.850306 -1.142508 -0.131858
F -3.234938 -2.743219 -1.439985
F -2.716985 3.018373 1.348957
F -1.461377 2.715769 -0.383177
F -3.310773 1.623428 -0.188407

PyrroleNMeIBA_A_NTf2.log
Energy (E) = -2274.30849048 Hartree
Enthalpy (H) = -2274.121045 Hartree
Gibbs free energy (G) = -2274.204152 Hartree
Charge = 0, Spin = 1
C 3.124202 -1.399956 2.029676
C 1.844599 -1.100037 1.595910
C 2.057421 -0.347862 0.439451
C 3.393761 -0.203347 0.196000
H 3.432884 -1.967219 2.891601
H 0.919607 -1.394393 2.059847
C 3.900635 0.551278 -0.947261
I 0.960255 0.614160 -1.004858
O 5.058528 0.726352 -1.227575
O 2.887538 1.048835 -1.677490
N 4.051682 -0.861072 1.190271
C 5.496556 -0.961398 1.322022
H 5.920769 -1.446602 0.447055
H 5.935030 0.028768 1.413048
H 5.716134 -1.545813 2.211195
N -0.936944 -0.091780 0.113948
S -1.314674 -1.692215 -0.009493
S -1.462586 0.874333 1.339555
O -0.441575 -2.203918 -1.024033
O -1.440408 -2.325331 1.263805
C -2.996832 -1.683317 -0.775931
O -0.334478 1.601410 1.838097
O -2.391204 0.210347 2.194979
C -2.434467 2.106707 0.366968
F -2.982283 -0.903166 -1.845389
F -3.897641 -1.251555 0.080439
F -3.277221 -2.921241 -1.135100
F -2.854095 3.049248 1.185364
F -1.657627 2.646559 -0.568080
F -3.465286 1.524248 -0.214906

QuinoIBA_L_NTf2.log
Energy (E) = -2426.62218522 Hartree
Enthalpy (H) = -2426.409135 Hartree
Gibbs free energy (G) = -2426.494836 Hartree
Charge = 0, Spin = 1
C 3.785526 -3.585848 0.889482
C 2.653224 -2.846009 0.674896
C 2.774004 -1.475951 0.379358
C 4.056539 -0.865766 0.297315
C 5.065573 -2.991942 0.817799
H 3.703295 -4.639840 1.116133
H 1.659632 -3.270834 0.715225
H 5.942613 -3.599266 0.992760
C 1.805775 0.484000 -0.109255
N 1.642533 -0.738300 0.167754
C 2.983710 1.221146 -0.235331
C 5.202034 -1.662318 0.527128
H 6.179437 -1.201668 0.467179
C 2.924751 2.664903 -0.593135
O 3.902622 3.352966 -0.713059
O 1.684680 3.112152 -0.770931
C 4.134372 0.509017 -0.018142
H 5.089573 1.014834 -0.097384
I 0.116404 1.749706 -0.485368
N -1.176695 -0.069411 -0.021655
S -1.147104 -1.300002 -1.128101
S -1.530649 -0.305909 1.572061
O -0.451608 -0.764143 -2.261053
O -0.823307 -2.572363 -0.571369
C -2.918282 -1.397632 -1.644678
O -0.517511 0.330328 2.358192
O -1.994178 -1.629294 1.833186
C -3.002620 0.800383 1.712777
F -3.356888 -0.186421 -1.949749
F -3.651972 -1.907145 -0.677089
F -2.984433 -2.172675 -2.710889
F -3.399214 0.811175 2.969117
F -2.667100 2.033436 1.344617
F -3.979726 0.371613 0.936830

FuranIBA_A_Me.log
Energy (E) = -468.235984101 Hartree
Enthalpy (H) = -468.124753 Hartree
Gibbs free energy (G) = -468.171870

Hartree
Charge = 0, Spin = 1
C 1.975837 2.312267 -0.000367
C 0.621055 2.198186 -0.000224
C 0.407992 0.793791 -0.000091
C 1.604431 0.168841 -0.000112
O 2.572869 1.100908 -0.000251
H 2.627129 3.167666 -0.000506
H -0.092922 3.002536 -0.000244
C 1.799473 -1.321074 0.000011
I -1.251130 -0.427979 0.000100
O 2.909917 -1.809650 0.000094
O 0.646565 -1.893870 0.000028
C -2.563446 1.293582 0.000126
H -3.580845 0.914206 0.000182
H -2.375126 1.872911 0.897502
H -2.375223 1.872897 -0.897280

IBamideAc_Me.log
Energy (E) = -603.081607181 Hartree
Enthalpy (H) = -602.886873 Hartree
Gibbs free energy (G) = -602.945182
Hartree
Charge = 0, Spin = 1
C 3.615033 0.722086 -0.000011
C 3.234342 2.058383 0.000146
C 1.891336 2.395049 0.000207
C 0.906773 1.410587 0.000108
C 1.322174 0.094891 -0.000042
C 2.652607 -0.280505 -0.000104
H 4.661261 0.449286 -0.000062
H 3.987415 2.834094 0.000222
H 1.547343 3.420848 0.000337
H 2.959945 -1.314291 -0.000227
C -0.545633 1.845391 0.000153
O -0.796540 3.034252 0.000507
I -0.219146 -1.363547 -0.000148
N -1.371762 0.769982 -0.000041
C -3.472415 -0.441522 0.000109
H -4.543911 -0.267187 0.000220
H -3.201593 -1.022141 -0.884412
H -3.201371 -1.021975 0.884670
C -2.752794 0.895496 -0.000122
O -3.374302 1.933443 -0.000123
C 1.187804 -3.033192 0.000165
H 1.794320 -3.014076 -0.899038
H 1.794360 -3.013678 0.899335
H 0.550674 -3.914333 0.000374

IBCCF32O_Me.log
Energy (E) = -1070.23317240 Hartree
Enthalpy (H) = -1070.056986 Hartree
Gibbs free energy (G) = -1070.120515
Hartree
Charge = 0, Spin = 1
C -1.176706 3.355852 0.153625
C 0.207104 3.423739 0.214410
C 0.967664 2.266183 0.148459
C 0.358152 1.020286 0.012258
C -1.019935 0.987402 -0.032514
C -1.803997 2.122582 0.035400
H -1.774281 4.255550 0.198782
H 0.698960 4.381220 0.313666
H 2.043621 2.325619 0.205351
H -2.880609 2.073775 -0.008294
O 0.320042 -1.319680 -0.453725
I -1.900752 -0.941840 -0.178410
C 1.122780 -0.309399 -0.081977
C 2.249099 -0.196989 -1.142156
C 1.715376 -0.617224 1.317888
F 0.700761 -0.781795 2.177171
F 2.487257 0.362951 1.806492
F 2.433277 -1.732542 1.314060
F 3.247615 0.627123 -0.782016
F 2.788844 -1.380177 -1.391874
F 1.738519 0.273778 -2.282381
C -3.911350 -0.162241 0.101521
H -4.210699 0.452710 -0.740517
H -4.530673 -1.055530 0.141301
H -3.982391 0.376390 1.040694

IBCH2S_Me.log
Energy (E) = -797.891995726 Hartree
Enthalpy (H) = -797.676105 Hartree
Gibbs free energy (G) = -797.729645
Hartree
Charge = 0, Spin = 1
C -0.938537 3.174712 -0.268734
C -2.226593 2.684787 -0.134849
C -2.444028 1.322607 0.022359
C -1.394825 0.402016 0.043959
C -0.121917 0.941368 -0.079504
C 0.131807 2.288738 -0.234773
H -0.757118 4.230753 -0.411175
H -3.070496 3.360680 -0.159909
H -3.457060 0.961975 0.125712
H 1.138317 2.658641 -0.355196
I 1.532739 -0.415669 -0.085643
C -1.645550 -1.088206 0.240003
S -0.529255 -2.007729 -0.854682
C -3.072520 -1.481807 -0.131533
H -3.151079 -2.567403 -0.087752
H -3.803181 -1.065025 0.566648
H -3.314618 -1.162041 -1.144758
C -1.417642 -1.439951 1.713484
H -2.115636 -0.888131 2.349474
H -1.577305 -2.509003 1.852849
H -0.403002 -1.205001 2.034823
C 2.873088 1.206103 0.671306
H 3.739306 0.664615 1.043362
H 3.166359 1.876087 -0.130297
H 2.378707 1.735769 1.479927

IBNMeCO2_Me.log
Energy (E) = -565.021848566 Hartree
Enthalpy (H) = -564.832291 Hartree
Gibbs free energy (G) = -564.886256
Hartree
Charge = 0, Spin = 1
C -0.532236 3.246888 -0.498794
C -1.852074 2.942630 -0.192293
C -2.236011 1.645422 0.098006
C -1.318234 0.581668 0.105898
C 0.005661 0.941451 -0.167595
C 0.405410 2.225649 -0.483089
H -0.233241 4.255793 -0.742782
H -2.600313 3.723570 -0.195273
H -3.274065 1.431959 0.306357
H 1.441265 2.436074 -0.711226
I 1.470014 -0.551414 -0.016979
C -1.326874 -1.834171 -0.423686
O -2.013922 -2.834136 -0.353770
N -1.715267 -0.710505 0.409576
C -2.957862 -0.887833 1.141871
H -3.006744 -0.165919 1.956328
H -3.838804 -0.775611 0.504021
H -2.969416 -1.896579 1.539019
O -0.294845 -1.623632 -1.146694
C 2.809431 0.787978 1.041562
H 3.534731 0.157953 1.548627
H 3.308719 1.446582 0.340327
H 2.220846 1.348707 1.759866

Indole_NMe_IBA_A_Me.log
Energy (E) = -641.170010494 Hartree
Enthalpy (H) = -640.966545 Hartree
Gibbs free energy (G) = -641.023705
Hartree
Charge = 0, Spin = 1
C 1.233040 -0.808889 -0.000095
C 2.256913 0.174634 0.000067
C 3.610901 -0.159043 0.000112
C 3.945321 -1.496582 0.000044
C 2.950050 -2.486191 -0.000106
C 1.609978 -2.160156 -0.000202
C 0.031622 -0.041571 -0.000262
H 4.369099 0.612579 0.000194
H 4.986281 -1.788725 0.000059
H 3.240807 -3.527724 -0.000185
H 0.875853 -2.950635 -0.000315
N 1.692136 1.425030 0.000075
C 0.332361 1.291507 -0.000095
C -0.706187 2.386246 -0.000066
O -1.884949 1.896558 -0.000113
O -0.371366 3.562269 -0.000017
I -1.999967 -0.495580 0.000022
C 2.459418 2.657537 0.000132
H 3.090982 2.699669 -0.887405
H 1.762680 3.488744 0.000433
H 3.091401 2.699269 0.887381
C -1.732941 -2.639603 0.000210
H -1.207392 -2.938218 -0.899086
H -1.207270 -2.938049 0.899493
H -2.741465 -3.044318 0.000326

NpthIBA_C_Me.log
Energy (E) = -623.935749449 Hartree
Enthalpy (H) = -623.744487 Hartree
Gibbs free energy (G) = -623.799206
Hartree
Charge = 0, Spin = 1
C -1.524406 1.590469 0.000024
C -0.196500 1.256662 0.000380
C 0.132175 -0.103274 0.000641
C -0.784270 -1.108344 0.000648
H -1.772437 2.645214 -0.000005
H -0.514493 -2.154874 0.000561
C 0.874985 2.336561 0.000055
O 2.064431 1.859707 0.000399
O 0.529379 3.503886 -0.000206
I 2.224354 -0.476066 -0.000157
C -3.169073 -1.758853 0.000149
C -4.489396 -1.403796 -0.000104
C -4.863663 -0.040974 -0.000332
C -3.908709 0.936990 -0.000316
C -2.532633 0.602435 -0.000025
C -2.160209 -0.766800 0.000312
H -2.877123 -2.801888 0.000298
H -5.255328 -2.167368 -0.000116
H -5.912139 0.223731 -0.000563
H -4.185043 1.983612 -0.000555
C 1.960187 -2.625715 -0.000134
H 1.437085 -2.934784 -0.898747
H 2.970033 -3.027875 -0.000323
H 1.437278 -2.935185 0.898428

PyIBA_D_Me.log
Energy (E) = -486.452659803 Hartree
Enthalpy (H) = -486.323046 Hartree
Gibbs free energy (G) = -486.371635

Hartree
Charge = 0, Spin = 1
C 2.043604 -2.385403 0.000203
C 3.045334 -1.420951 0.000215
C 1.544781 0.309024 0.000081
C 0.502777 -0.603150 0.000052
H 2.289154 -3.437511 0.000260
H 4.087544 -1.717839 0.000283
C 1.261020 1.813667 0.000000
O -0.002637 2.063744 -0.000106
O 2.182923 2.596428 0.000045
I -1.431690 0.245431 -0.000095
C 0.722416 -1.965128 0.000114
H -0.081338 -2.686110 0.000099
N 2.806770 -0.115956 0.000158
C -2.408684 -1.694898 -0.000029
H -2.150837 -2.244903 -0.898942
H -2.150801 -2.244855 0.898903
H -3.471309 -1.465310 -0.000015

PyrroleNMeIBA_A_Me.log
Energy (E) = -487.665161876 Hartree
Enthalpy (H) = -487.511524 Hartree
Gibbs free energy (G) = -487.562817
Hartree
Charge = 0, Spin = 1
C 1.913170 2.117491 0.000394
C 0.533265 2.093517 0.000145
C 0.213579 0.724260 0.000053
C 1.350145 -0.031613 0.000165
H 2.585955 2.958729 0.000527
H -0.120005 2.948141 0.000048
C 1.372981 -1.526974 0.000087
I -1.565570 -0.329303 -0.000257
O 2.441173 -2.123618 0.000097
O 0.183244 -2.002902 -0.000342
N 2.395615 0.841452 0.000347
C 3.802332 0.471023 0.000540
H 4.032952 -0.125944 0.878018
H 4.033196 -0.125950 -0.876869
H 4.390340 1.386022 0.000621
C -2.697138 1.511756 0.000240
H -2.446263 2.069165 -0.895535
H -3.748211 1.240371 -0.000612
H -2.447404 2.067757 0.897214

QuinoIBA_L_Me.log
Energy (E) = -639.979065619 Hartree
Enthalpy (H) = -639.799785 Hartree
Gibbs free energy (G) = -639.854876
Hartree
Charge = 0, Spin = 1
C -4.360455 -1.435967 0.000011
C -3.026283 -1.742965 0.000001
C -2.069181 -0.708345 0.000006
C -2.492110 0.645218 -0.000003
C -4.790941 -0.091026 0.000010
H -5.095733 -2.229042 0.000020
H -2.674420 -2.765171 -0.000003
H -5.849447 0.129271 0.000016
C 0.083134 -0.042780 -0.000054
N -0.737194 -1.023068 -0.000026
C -0.176682 1.330775 -0.000019
C -3.877782 0.927482 -0.000002
H -4.196284 1.961926 -0.000009
C 0.927877 2.376003 -0.000004
O 0.597559 3.549834 0.000034
O 2.095828 1.864052 -0.000015
C -1.505670 1.657109 -0.000002
H -1.770005 2.708425 -0.000005
I 2.183674 -0.541356 0.000004
C 1.708874 -2.642848 0.000017
H 1.137493 -2.869571 0.891728
H 1.137822 -2.869560 -0.891909
H 2.664432 -3.159974 0.000187

FuranIBA_A_F.log
Energy (E) = -528.165902677 Hartree
Enthalpy (H) = -528.090435 Hartree
Gibbs free energy (G) = -528.135025
Hartree
Charge = 0, Spin = 1
C -2.200632 2.119632 0.000519
C -0.840106 2.170920 0.000194
C -0.467329 0.807780 0.000080
C -1.579776 0.047983 0.000099
O -2.659092 0.844933 0.000292
H -2.949870 2.890527 0.000752
H -0.203754 3.036401 0.000161
C -1.527878 -1.427543 -0.000100
I 1.240616 -0.304566 -0.000182
O -2.473117 -2.167893 0.000184
O -0.252576 -1.814810 -0.000028
F 2.241509 1.444956 0.000047

IBamideAc_F.log
Energy (E) = -663.027095044 Hartree
Enthalpy (H) = -662.867906 Hartree
Gibbs free energy (G) = -662.923601
Hartree
Charge = 0, Spin = 1
C 3.682972 0.210518 -0.000543
C 3.492316 1.590188 -0.000472
C 2.213386 2.122529 -0.000215
C 1.115333 1.269685 -0.000027
C 1.346496 -0.087456 -0.000112
C 2.599424 -0.661159 -0.000358
H 4.684491 -0.196666 -0.000752
H 4.349494 2.248790 -0.000617
H 2.024980 3.187664 -0.000138
H 2.715001 -1.734188 -0.000410
C -0.277073 1.812434 0.000285
O -0.494625 2.998724 0.000119
I -0.407579 -1.219777 0.000167
N -1.217105 0.788252 0.000089
C -3.441483 -0.238779 0.000211
H -4.487227 0.050085 0.000315
H -3.240001 -0.843688 -0.886296
H -3.239735 -0.843411 0.886846
C -2.600936 1.019140 -0.000146
O -3.096454 2.115166 -0.000160
F 0.806713 -2.874653 0.000018

IBCCF32O_F.log
Energy (E) = -1130.17370839 Hartree
Enthalpy (H) = -1130.032982 Hartree
Gibbs free energy (G) = -1130.094502
Hartree
Charge = 0, Spin = 1
C -1.360225 3.307582 0.116379
C 0.021304 3.445944 0.148100
C 0.848500 2.332887 0.093240
C 0.291152 1.061819 -0.006393
C -1.084324 0.966741 -0.032505
C -1.937503 2.046739 0.029712
H -1.996090 4.180419 0.161332
H 0.461798 4.430281 0.220639
H 1.920756 2.449298 0.132740
H -3.006459 1.900355 0.015418
O 0.269606 -1.305955 -0.378287
I -1.779405 -1.000981 -0.145914
C 1.076604 -0.241525 -0.058702
C 2.157841 -0.186800 -1.163111
C 1.698553 -0.512560 1.331796
F 0.702147 -0.656866 2.211552
F 2.471941 0.488047 1.756781
F 2.419294 -1.624379 1.340018
F 3.134488 0.680869 -0.874040
F 2.713717 -1.372923 -1.352472
F 1.595863 0.196818 -2.307786
F -3.648536 -0.243312 0.056894

IBCH2S_F.log
Energy (E) = -857.849627959 Hartree
Enthalpy (H) = -857.669001 Hartree
Gibbs free energy (G) = -857.720855
Hartree
Charge = 0, Spin = 1
C -1.216002 3.081242 -0.183542
C -2.456937 2.463372 -0.150718
C -2.554024 1.081047 -0.057120
C -1.414205 0.281128 -0.011773
C -0.199913 0.945719 -0.062624
C -0.057475 2.314119 -0.134347
H -1.139633 4.157123 -0.253859
H -3.358873 3.058465 -0.193124
H -3.529681 0.617080 -0.014938
H 0.930072 2.750316 -0.139214
I 1.526770 -0.280345 -0.042396
C -1.492453 -1.221238 0.197046
S -0.128156 -1.997213 -0.725208
C -2.772870 -1.830353 -0.362591
H -2.711162 -2.917166 -0.304097
H -3.637276 -1.518344 0.225708
H -2.923280 -1.543052 -1.402392
C -1.388086 -1.525072 1.691296
H -2.213414 -1.047907 2.224230
H -1.433900 -2.601867 1.852864
H -0.452743 -1.151652 2.108658
F 2.545930 1.496768 0.465853

IBNMeCO2_F.log
Energy (E) = -624.957137927 Hartree
Enthalpy (H) = -624.802981 Hartree
Gibbs free energy (G) = -624.854469
Hartree
Charge = 0, Spin = 1
C -0.885011 3.194299 -0.379497
C -2.179654 2.712886 -0.239265
C -2.421981 1.365413 -0.027829
C -1.371922 0.444500 0.054258
C -0.091124 0.969593 -0.096599
C 0.176914 2.305403 -0.310741
H -0.698976 4.246661 -0.535979
H -3.017665 3.393819 -0.296617
H -3.437984 1.013285 0.072652
H 1.201321 2.640068 -0.381479
I 1.485535 -0.378994 0.013857
C -1.000924 -1.962675 -0.349791
O -1.505906 -3.056995 -0.383705
N -1.621657 -0.905470 0.337796
C -2.879836 -1.248484 0.986103
H -3.086771 -0.522004 1.768964
H -3.712680 -1.274547 0.280432
H -2.779003 -2.238015 1.417864
O 0.131591 -1.655997 -0.954377
F 2.562862 1.131557 0.828891

Indole_NMe_IBA_A_F.log
Energy (E) = -701.102432334 Hartree

Enthalpy (H) = -700.934778 Hartree
Gibbs free energy (G) = -700.989432 Hartree
Charge = 0, Spin = 1
C 1.303597 -0.732433 0.000014
C 2.237414 0.336430 -0.000060
C 3.613808 0.103490 -0.000097
C 4.035024 -1.209003 -0.000169
C 3.118201 -2.276831 -0.000101
C 1.758847 -2.057497 0.000045
C 0.048589 -0.077423 0.000183
H 4.318863 0.923969 -0.000112
H 5.094675 -1.424966 -0.000267
H 3.493825 -3.290586 -0.000162
H 1.047829 -2.870343 0.000091
N 1.567766 1.538517 -0.000002
C 0.227448 1.271367 0.000175
C -0.931415 2.188797 -0.000029
O -2.066914 1.508530 -0.000544
O -0.862728 3.395656 0.000207
I -1.927889 -0.609922 -0.000030
C 2.200801 2.843017 0.000180
H 2.823599 2.956757 -0.886869
H 1.425316 3.601788 -0.000030
H 2.823123 2.956772 0.887566
F -1.340025 -2.541121 0.000361

NpthIBA_C_F.log
Energy (E) = -683.872478586 Hartree
Enthalpy (H) = -683.716895 Hartree
Gibbs free energy (G) = -683.769363 Hartree
Charge = 0, Spin = 1
C -1.462687 1.616406 0.000010
C -0.153577 1.218697 -0.000014
C 0.123268 -0.151685 -0.000042
C -0.814285 -1.130345 -0.000075
H -1.682331 2.677164 0.000020
H -0.540740 -2.175953 -0.000100
C 0.984128 2.189457 -0.000038
O 2.168582 1.597743 -0.000266
O 0.827055 3.383651 0.000152
I 2.181451 -0.506975 0.000009
C -3.219354 -1.680934 -0.000112
C -4.525787 -1.277540 -0.000046
C -4.851558 0.097435 0.000061
C -3.862791 1.040922 0.000094
C -2.499705 0.657402 0.000014
C -2.174394 -0.727496 -0.000061
H -2.963053 -2.732551 -0.000177
H -5.318699 -2.012890 -0.000064
H -5.889793 0.399439 0.000114
H -4.102606 2.096527 0.000173
F 1.739517 -2.483015 0.000189

PyIBA_D_F.log
Energy (E) = -546.391105314 Hartree
Enthalpy (H) = -546.297140 Hartree
Gibbs free energy (G) = -546.343311 Hartree
Charge = 0, Spin = 1
C 2.286380 -2.145739 -0.000332
C 3.163828 -1.061240 -0.000365
C 1.463670 0.445947 -0.000100
C 0.548712 -0.584637 -0.000078
H 2.670932 -3.155288 -0.000447
H 4.233686 -1.230096 -0.000474
C 0.947064 1.863876 0.000095
O -0.381295 1.913148 0.000080
O 1.656230 2.828947 0.000027
I -1.415749 0.091189 0.000147
C 0.920093 -1.911507 -0.000177
H 0.182101 -2.699701 -0.000139
N 2.771342 0.207014 -0.000245
F -1.958820 -1.863778 -0.000016

PyrroleNMeIBA_A_F.log
Energy (E) = -547.594759864 Hartree
Enthalpy (H) = -547.476866 Hartree
Gibbs free energy (G) = -547.525982 Hartree
Charge = 0, Spin = 1
C -2.037766 2.027809 -0.000206
C -0.657110 2.111198 0.000035
C -0.240225 0.777191 -0.000012
C -1.316831 -0.060637 -0.000019
H -2.771232 2.816715 -0.000325
H -0.047311 2.995651 0.000114
C -1.162326 -1.520242 -0.000001
I 1.528879 -0.248320 0.000060
O -2.075509 -2.313128 0.000329
O 0.123259 -1.850416 0.000336
N -2.431648 0.721272 -0.000064
C -3.800715 0.233308 -0.000086
H -3.982559 -0.377230 -0.880600
H -3.982631 -0.377152 0.880467
H -4.467963 1.091131 -0.000153
F 2.461163 1.539950 -0.000646

QuinoIBA_L_F.log
Energy (E) = -699.902146976 Hartree
Enthalpy (H) = -699.758699 Hartree
Gibbs free energy (G) = -699.811344 Hartree
Charge = 0, Spin = 1
C -4.424453 -1.347017 -0.000227
C -3.101321 -1.698544 -0.000333
C -2.111937 -0.695488 -0.000269
C -2.493225 0.673688 0.000127
C -4.812525 0.011165 0.000031
H -5.185200 -2.115531 -0.000364
H -2.778083 -2.729666 -0.000492
H -5.863570 0.264253 0.000080
C 0.053629 -0.114578 -0.000019
N -0.793581 -1.061293 -0.000250
C -0.165379 1.267377 0.000111
C -3.868956 1.001096 0.000250
H -4.155558 2.044812 0.000493
C 0.990189 2.215557 -0.000079
O 0.835553 3.411852 0.000485
O 2.152665 1.602556 -0.000669
C -1.477222 1.654462 0.000307
H -1.712012 2.712511 0.000562
I 2.153314 -0.533113 -0.000131
F 1.742813 -2.490436 0.001165

FuranIBA_A_CF2CF3.log
Energy (E) = -1003.53223006 Hartree
Enthalpy (H) = -1003.426040 Hartree
Gibbs free energy (G) = -1003.485785 Hartree
Charge = 0, Spin = 1
C -2.561099 2.573400 -0.000135
C -1.256520 2.192206 -0.000224
C -1.331500 0.773726 0.000021
C -2.630033 0.405181 0.000013
O -3.392982 1.508052 -0.000089
H -3.025924 3.542745 -0.000205
H -0.395154 2.833825 -0.000335
C -3.113818 -1.006924 0.000017
I -0.027382 -0.830994 0.000010
O -4.286541 -1.295629 0.000045
O -2.087861 -1.809429 -0.000023
C 1.655910 0.643165 0.000310
C 3.010018 -0.077171 -0.000183
F 1.587312 1.422688 1.086917
F 1.586977 1.423532 -1.085683
F 4.009599 0.785909 0.000004
F 3.095663 -0.848692 1.079529
F 3.095301 -0.847808 -1.080584

IBamideAc_CF2CF3.log
Energy (E) = -1138.37954301 Hartree
Enthalpy (H) = -1138.189807 Hartree
Gibbs free energy (G) = -1138.261090 Hartree
Charge = 0, Spin = 1
C -1.125906 3.704415 -0.000957
C -2.508309 3.561273 0.000035
C -3.072505 2.297795 0.000720
C -2.266618 1.163044 0.000466
C -0.900212 1.346283 -0.000387
C -0.296702 2.588885 -0.001180
H -0.677376 4.687985 -0.001590
H -3.141351 4.437455 0.000225
H -4.142003 2.134996 0.001419
H 0.771054 2.721393 -0.002011
C -2.928620 -0.192522 0.000870
O -4.137424 -0.267773 0.002272
I 0.198433 -0.477687 -0.000466
N -1.999351 -1.192874 -0.000234
C -1.151942 -3.479033 -0.000923
H -1.513327 -4.502343 -0.001105
H -0.533443 -3.318392 -0.886442
H -0.533114 -3.318836 0.884450
C -2.344345 -2.543882 -0.000462
O -3.473903 -2.967697 -0.000317
C 2.198059 0.627659 -0.000120
C 3.313237 -0.426035 0.000802
F 2.363662 1.395959 1.090433
F 2.364826 1.395154 -1.091057
F 4.509801 0.135557 0.000818
F 3.194013 -1.194891 1.081306
F 3.194572 -1.196030 -1.078993

IBCCF32O_CF2CF3.log
Energy (E) = -1605.53103272 Hartree
Enthalpy (H) = -1605.359796 Hartree
Gibbs free energy (G) = -1605.436395 Hartree
Charge = 0, Spin = 1
C 0.379750 3.557497 0.185186
C 1.749584 3.398954 0.333454
C 2.319332 2.137359 0.266252
C 1.525465 1.013386 0.048913
C 0.164799 1.207356 -0.066878
C -0.434312 2.450139 -0.013644
H -0.066853 4.540988 0.222030
H 2.380274 4.261505 0.495941
H 3.385722 2.018381 0.380958
H -1.495621 2.585080 -0.132012
O 1.139409 -1.254560 -0.580260
I -0.937957 -0.599000 -0.331229
C 2.072115 -0.410679 -0.079317
C 3.271190 -0.441272 -1.061529
C 2.499809 -0.900449 1.327092
F 1.407279 -0.964994 2.098462
F 3.364306 -0.080014 1.932521
F 3.043410 -2.108469 1.282756
F 4.365239 0.162649 -0.573876

F 3.606404 -1.685426 -1.362731
F 2.935803 0.183797 -2.190411
C -2.897679 0.467384 0.011073
C -4.008017 -0.591345 0.086269
F -3.211760 1.307960 -0.991591
F -2.913089 1.155979 1.163006
F -5.196111 -0.027344 0.221147
F -3.996381 -1.318461 -1.030281
F -3.789467 -1.398172 1.118671

IBCH2S_CF2CF3.log
Energy (E) = -1333.19873717 Hartree
Enthalpy (H) = -1332.987575 Hartree
Gibbs free energy (G) = -1333.054241 Hartree
Charge = 0, Spin = 1
C -1.484303 3.222889 -0.367917
C -2.772540 2.964745 0.070315
C -3.174353 1.663907 0.343506
C -2.306306 0.583409 0.191905
C -1.020942 0.897147 -0.222138
C -0.587325 2.171807 -0.518102
H -1.171299 4.229235 -0.607012
H -3.477686 3.776038 0.187929
H -4.187324 1.482101 0.672624
H 0.411188 2.359486 -0.879158
I 0.298705 -0.762631 -0.503326
C -2.738440 -0.845481 0.488288
S -2.019806 -1.924087 -0.788454
C -4.251087 -1.021115 0.399145
H -4.487051 -2.080840 0.491459
H -4.758683 -0.496216 1.211306
H -4.632308 -0.663806 -0.556856
C -2.279544 -1.237515 1.894494
H -2.750413 -0.586612 2.635216
H -2.567288 -2.269432 2.093801
H -1.199302 -1.155441 2.010583
C 2.094693 0.681466 0.032280
C 3.273561 -0.201853 0.439777
F 1.820532 1.499936 1.065623
F 2.509080 1.441016 -1.007319
F 4.376541 0.500463 0.662434
F 2.972494 -0.881646 1.543020
F 3.519712 -1.078228 -0.539052

IBNMeCO2_CF2CF3.log
Energy (E) = -1100.31995137 Hartree
Enthalpy (H) = -1100.135363 Hartree
Gibbs free energy (G) = -1100.201760 Hartree
Charge = 0, Spin = 1
C -1.506213 3.237553 -0.540153
C -2.702503 2.995385 0.122641
C -3.050195 1.718098 0.527032
C -2.206991 0.621118 0.299812
C -0.997412 0.920879 -0.328013
C -0.641952 2.179036 -0.772333
H -1.243238 4.231902 -0.869664
H -3.387531 3.810444 0.311749
H -4.001301 1.549873 1.010551
H 0.299039 2.339556 -1.278064
I 0.318586 -0.688243 -0.601382
C -2.488107 -1.772779 -0.207495
O -3.204837 -2.727028 -0.002747
N -2.558674 -0.663478 0.702183
C -3.553131 -0.799322 1.755592
H -3.343247 -0.077225 2.542768
H -4.571162 -0.654359 1.386338
H -3.488721 -1.806424 2.152167
O -1.648250 -1.624463 -1.179604
C 2.019990 0.611899 0.090355
C 3.153265 -0.281166 0.613859
F 1.644416 1.438979 1.069443
F 2.512009 1.343562 -0.924076
F 4.234379 0.427072 0.891304
F 2.758064 -0.919611 1.707026
F 3.454025 -1.183870 -0.322239

Indole_NMe_IBA_A_CF2CF3.log
Energy (E) = -1176.46437249 Hartree
Enthalpy (H) = -1176.266036 Hartree
Gibbs free energy (G) = -1176.336657 Hartree
Charge = 0, Spin = 1
C -1.500598 1.200788 -0.000151
C -2.909436 1.024068 0.000223
C -3.798961 2.098846 0.000293
C -3.269875 3.370256 -0.000060
C -1.880086 3.568796 -0.000483
C -0.997608 2.510649 -0.000536
C -1.001701 -0.136577 -0.000202
H -4.867318 1.929899 0.000610
H -3.930490 4.226101 -0.000028
H -1.491149 4.577568 -0.000786
H 0.060066 2.708430 -0.000857
N -3.211322 -0.314539 0.000397
C -2.045412 -1.021157 0.000108
C -1.878389 -2.511103 -0.000019
O -0.630461 -2.836049 -0.000321
O -2.832213 -3.266864 0.000118
I 0.856285 -1.104213 -0.000303
C -4.566499 -0.836628 0.000609
H -5.095137 -0.488828 -0.886700
H -4.516554 -1.919450 0.000632
H -5.094889 -0.488760 0.888038
C 2.036854 0.788721 0.000002
C 3.533098 0.434701 0.000412
F 1.789389 1.525298 1.090380
F 1.790048 1.525333 -1.090531
F 4.273720 1.528807 0.000560
F 3.817856 -0.286607 1.080472
F 3.818400 -0.286689 -1.079460

NpthIBA_C_CF2CF3.log
Energy (E) = -1159.23182466 Hartree
Enthalpy (H) = -1159.045615 Hartree
Gibbs free energy (G) = -1159.113457 Hartree
Charge = 0, Spin = 1
C -3.064231 1.161360 -0.000465
C -1.721888 1.430840 -0.000253
C -0.837080 0.347002 0.000143
C -1.223504 -0.955763 0.000423
H -3.743775 2.005142 -0.000733
H -0.535484 -1.785704 0.000865
C -1.224466 2.858424 -0.000112
O 0.067284 2.944928 0.000339
O -2.014743 3.777347 -0.000354
I 1.195764 1.004692 0.000289
C -3.090754 -2.568408 0.000498
C -4.434303 -2.820657 0.000251
C -5.363516 -1.755658 -0.000277
C -4.929220 -0.459862 -0.000533
C -3.544191 -0.165104 -0.000287
C -2.613557 -1.236369 0.000214
H -2.374429 -3.380236 0.000896
H -4.792947 -3.840857 0.000460
H -6.422735 -1.973031 -0.000472
H -5.632760 0.362694 -0.000925
C 1.967519 -1.107029 0.000067
C 3.503201 -1.069776 -0.000461
F 1.575343 -1.785945 -1.089283
F 1.576057 -1.785639 1.089910
F 4.006278 -2.291985 -0.000073
F 3.929835 -0.422523 -1.080989
F 3.930513 -0.421565 1.079264

PyIBA_D_CF2CF3.log
Energy (E) = -1021.74915000 Hartree
Enthalpy (H) = -1021.624565 Hartree
Gibbs free energy (G) = -1021.686159 Hartree
Charge = 0, Spin = 1
C -2.297608 2.783790 -0.000384
C -3.550871 2.180336 0.000394
C -2.658530 0.075951 0.000292
C -1.385931 0.617795 -0.000333
H -2.203599 3.859934 -0.000739
H -4.449035 2.786070 0.000769
C -2.857964 -1.433908 0.000422
O -1.726971 -2.076744 -0.000679
O -3.963545 -1.911442 0.001410
I 0.135590 -0.862688 -0.000583
C -1.167791 1.980943 -0.000730
H -0.186278 2.424252 -0.001330
N -3.728791 0.866503 0.000697
C 1.756662 0.705339 -0.000019
C 3.123087 0.005305 0.000622
F 1.694553 1.488050 1.089561
F 1.695607 1.487999 -1.089702
F 4.108649 0.885240 0.000556
F 3.223214 -0.763660 1.081091
F 3.223755 -0.764419 -1.079294

PyrroleNMeIBA_A_CF2CF3.log
Energy (E) = -1022.96171300 Hartree
Enthalpy (H) = -1022.813110 Hartree
Gibbs free energy (G) = -1022.876914 Hartree
Charge = 0, Spin = 1
C -2.523491 2.342076 0.000073
C -1.180368 2.025602 0.000035
C -1.163210 0.620885 0.000091
C -2.437139 0.127933 0.000207
H -2.997332 3.309349 0.000052
H -0.359591 2.718376 -0.000027
C -2.772739 -1.319232 0.000261
I 0.279196 -0.871590 0.000005
O -3.926834 -1.706940 0.000357
O -1.697705 -2.043443 0.000194
N -3.272215 1.202277 0.000208
C -4.725801 1.141440 0.000303
H -5.079566 0.609876 -0.878348
H -5.079455 0.609909 0.879018
H -5.104342 2.160643 0.000307
C 1.849674 0.717055 -0.000184
C 3.250158 0.090166 -0.000226
F 1.733691 1.490619 1.086504
F 1.733574 1.490488 -1.086954
F 4.188586 1.020422 -0.000377
F 3.391168 -0.673016 1.079843
F 3.391008 -0.673212 -1.080181

QuinoIBA_L_CF2CF3.log
Energy (E) = -1175.27198206 Hartree
Enthalpy (H) = -1175.097931 Hartree
Gibbs free energy (G) = -1175.165572 Hartree
Charge = 0, Spin = 1
C -4.105671 -2.951177 -0.000057

C -2.787246 -2.582509 -0.000179
C -2.446641 -1.215644 -0.000141
C -3.466496 -0.228841 0.000053
C -5.128635 -1.977126 0.000114
H -4.371412 -3.999379 -0.000092
H -1.986329 -3.308088 -0.000308
H -6.162817 -2.292208 0.000202
C -0.882600 0.395750 -0.000159
N -1.127574 -0.851646 -0.000225
C -1.762313 1.480546 -0.000011
C -4.817917 -0.645088 0.000172
H -5.594261 0.108966 0.000305
C -1.284126 2.914330 0.000096
O -2.105497 3.808550 0.000300
O -0.000837 3.016184 -0.000145
C -3.086407 1.131673 0.000115
H -3.823280 1.926659 0.000252
I 1.193484 1.044504 -0.000132
C 1.868763 -1.092299 0.000009
C 3.406582 -1.111492 0.000145
F 1.452894 -1.738796 -1.090417
F 1.452707 -1.738515 1.090572
F 3.860453 -2.352901 0.000503
F 3.860579 -0.481836 -1.079929
F 3.860408 -0.481257 1.079944

PhICF2H_F.log
Energy (E) = -580.773686512 Hartree
Enthalpy (H) = -580.646579 Hartree
Gibbs free energy (G) = -580.697505 Hartree
Charge = 0, Spin = 1
C 1.370177 1.302901 -0.418694
C 2.687852 1.739888 -0.330793
C 3.674219 0.890634 0.148604
C 3.352940 -0.405785 0.531222
C 2.044212 -0.863515 0.449256
C 1.081653 0.011134 -0.018085
H 0.610540 1.956532 -0.821257
H 2.935834 2.742688 -0.649718
H 4.697209 1.234889 0.213069
H 4.123273 -1.072604 0.892890
H 1.770384 -1.878047 0.695976
I -0.917677 -0.684284 -0.085160
C -1.591857 1.369401 0.406796
H -0.940072 1.863212 1.128355
F -2.824751 1.215681 0.932643
F -1.713815 2.145922 -0.691535
F 0.063514 -2.566888 -0.413960

PhINMe2_F.log
Energy (E) = -476.941871137 Hartree
Enthalpy (H) = -476.754229 Hartree
Gibbs free energy (G) = -476.807249 Hartree
Charge = 0, Spin = 1
C -3.699958 0.906752 -0.070905
C -3.411940 -0.406573 0.275301
C -2.100206 -0.866861 0.279698
C -1.104810 0.023912 -0.072600
C -1.353624 1.337476 -0.419287
C -2.673910 1.773994 -0.419151
H -4.724368 1.253461 -0.072277
H -4.208873 -1.085935 0.544693
H -1.848675 -1.888913 0.520445
H -0.537865 1.994515 -0.685222
H -2.892702 2.796282 -0.695649
I 0.905771 -0.671551 -0.041594
N 1.560190 1.333891 -0.069883
C 1.598672 1.878688 1.274725
H 2.303445 1.352822 1.936120
H 1.900283 2.928885 1.233271
H 0.605767 1.832889 1.725310
C 2.866944 1.388479 -0.699196
H 3.196483 2.429174 -0.747162
H 3.643205 0.828147 -0.153209
H 2.814367 1.003458 -1.717442
F 0.010526 -2.600561 -0.009634

PhINTf2_F.log
Energy (E) = -2169.02912044 Hartree
Enthalpy (H) = -2168.855216 Hartree
Gibbs free energy (G) = -2168.934476 Hartree
Charge = 0, Spin = 1
C -4.361582 1.449484 1.567075
C -4.552791 1.170715 0.219486
C -3.620224 0.415491 -0.477963
C -2.524489 -0.043848 0.224368
C -2.301755 0.198831 1.562895
C -3.245790 0.965996 2.235859
H -5.088424 2.047002 2.099309
H -5.422295 1.548803 -0.299101
H -3.751159 0.187190 -1.525028
H -1.441273 -0.200871 2.079318
H -3.101381 1.177408 3.285549
I -1.126468 -1.171074 -0.882999
N 0.541262 0.065176 0.141119
S 0.490499 1.700230 -0.076812
S 1.381565 -0.656333 1.353257
O -0.499012 1.910511 -1.092740
O 0.468560 2.412850 1.158548
C 2.096820 2.089175 -0.907683
O 0.524041 -1.626568 1.969644
O 2.156657 0.270275 2.111810
C 2.572825 -1.655544 0.358552
F 2.250166 1.286676 -1.949342
F 3.108956 1.944508 -0.079604
F 2.032911 3.341169 -1.320264
F 3.266137 -2.420892 1.175397
F 1.898634 -2.419896 -0.497644
F 3.385041 -0.866764 -0.317659
F -2.699023 -2.101415 -1.691513

PhIOSO2Me_F.log
Energy (E) = -1006.00702569 Hartree
Enthalpy (H) = -1005.846832 Hartree
Gibbs free energy (G) = -1005.904390 Hartree
Charge = 0, Spin = 1
C -3.512334 2.180491 -0.002676
C -2.205856 2.529235 0.311318
C -1.195626 1.573945 0.296528
C -1.551866 0.283702 -0.037411
C -2.839103 -0.106487 -0.350059
C -3.828607 0.868486 -0.328744
H -4.288660 2.932964 0.012135
H -1.961398 3.547783 0.578094
H -0.178330 1.836976 0.551217
H -3.070103 -1.135705 -0.578734
H -4.846129 0.594442 -0.569413
I -0.070088 -1.227237 -0.063313
O 1.264586 0.325525 -0.793324
S 2.454996 0.677063 0.133739
O 3.041667 -0.545750 0.625471
O 2.047247 1.668461 1.099016
C 3.549892 1.429921 -1.022273
H 3.051169 2.292063 -1.454185
H 4.431083 1.733388 -0.463162
H 3.804650 0.697017 -1.781220
F -1.537170 -2.492226 0.474626

PhIOTf_F.log
Energy (E) = -1303.61298283 Hartree
Enthalpy (H) = -1303.473687 Hartree
Gibbs free energy (G) = -1303.536782 Hartree
Charge = 0, Spin = 1
C -1.093519 3.571083 0.211784
C -1.017042 2.784495 1.354068
C -1.122974 1.403890 1.260033
C -1.314165 0.861662 0.002180
C -1.395666 1.615439 -1.155242
C -1.282223 2.993063 -1.037559
H -1.001106 4.645026 0.295082
H -0.861283 3.240232 2.321320
H -1.028382 0.773760 2.130823
H -1.534930 1.148566 -2.119137
H -1.337535 3.610417 -1.922719
I -1.460712 -1.208337 -0.162363
O 0.698538 -1.162566 -0.708938
S 1.781096 -0.998418 0.348692
O 2.765570 -2.030278 0.276917
O 1.198504 -0.636612 1.615609
C 2.604042 0.521970 -0.278228
F 3.075493 0.320022 -1.496749
F 3.600687 0.839581 0.531174
F 1.739041 1.531110 -0.312810
F -3.402796 -1.054647 0.246150

PhIPyrroleN_F.log
Energy (E) = -551.896514534 Hartree
Enthalpy (H) = -551.718332 Hartree
Gibbs free energy (G) = -551.772343 Hartree
Charge = 0, Spin = 1
C 2.519510 -0.100599 -0.001018
C 3.391212 -1.184063 -0.000988
C 2.902810 -2.482584 0.000232
C 1.533906 -2.708106 0.001451
C 0.639464 -1.642755 0.001396
C 1.164214 -0.364059 0.000204
H 2.876235 0.917935 -0.001852
H 4.456670 -1.000456 -0.001934
H 3.588142 -3.319027 0.000254
H 1.145142 -3.716828 0.002429
H -0.423429 -1.837538 0.002380
I -0.168742 1.315731 0.000011
N -1.796508 -0.007541 -0.000486
C -2.306102 -0.625166 1.115010
C -2.304590 -0.626564 -1.115873
C -3.158665 -1.618920 0.710518
H -2.020571 -0.304676 2.103373
C -3.157698 -1.619811 -0.711289
H -2.017705 -0.307330 -2.104251
H -3.730747 -2.263613 1.356385
H -3.728905 -2.265316 -1.357120
F 1.558856 2.472405 0.000590

PhISeMe_F.log
Energy (E) = -391.705063202 Hartree
Enthalpy (H) = -391.561666 Hartree
Gibbs free energy (G) = -391.615516 Hartree
Charge = 0, Spin = 1
C -3.576043 -1.683676 -0.045793
C -2.373583 -2.213396 -0.491595
C -1.224482 -1.430547 -0.510447
C -1.323844 -0.123017 -0.083861
C -2.502574 0.439810 0.362955

C -3.637294 -0.363595 0.378343
H -4.465451 -2.298518 -0.031934
H -2.319283 -3.238929 -0.829849
H -0.284946 -1.841246 -0.857722
H -2.531857 1.475733 0.664436
H -4.572084 0.055838 0.723742
I 0.410074 1.143417 -0.084406
C 1.782057 -1.572413 1.559337
H 0.741065 -1.877727 1.637911
H 2.425855 -2.427126 1.745179
H 2.000678 -0.786803 2.275675
Se 2.156857 -0.951615 -0.267670
F -0.991827 2.708180 0.137024

PhISMe_F.log
Energy (E) = -780.488179179 Hartree
Enthalpy (H) = -780.344082 Hartree
Gibbs free energy (G) = -780.395569 Hartree
Charge = 0, Spin = 1
C 3.656187 -0.767672 -0.006732
C 3.301847 0.489789 0.462445
C 1.978110 0.913856 0.425913
C 1.038919 0.042932 -0.090650
C 1.354389 -1.214265 -0.561693
C 2.685175 -1.615728 -0.520003
H 4.689298 -1.085571 0.024630
H 4.055435 1.155129 0.860597
H 1.680584 1.896614 0.759035
H 0.590768 -1.869889 -0.958639
H 2.954297 -2.593960 -0.893782
I -1.001700 0.706740 -0.118313
C -1.534295 -2.195775 1.296678
H -1.931299 -3.205995 1.371530
H -0.459840 -2.226561 1.476444
H -2.013686 -1.571990 2.047567
S -1.884283 -1.592288 -0.366887
F -0.134327 2.622531 0.157519

PhISPh_F.log
Energy (E) = -972.053429608 Hartree
Enthalpy (H) = -971.852976 Hartree
Gibbs free energy (G) = -971.913805 Hartree
Charge = 0, Spin = 1
C -2.110029 -3.325058 -0.016440
C -1.418490 -2.805620 -1.101667
C -1.060880 -1.462249 -1.126786
C -1.423517 -0.673297 -0.055582
C -2.117416 -1.153694 1.037394
C -2.457251 -2.501591 1.045934
H -2.382748 -4.371357 -0.000749
H -1.154837 -3.439544 -1.937036
H -0.514867 -1.050341 -1.963952
H -2.406758 -0.486793 1.834939
H -2.999575 -2.901968 1.891246
I -0.891474 1.396645 -0.034308
S 1.056034 1.039475 -1.562894
C 2.148313 0.192919 -0.466270
C 2.463884 -1.142070 -0.707548
C 2.730937 0.854121 0.614326
C 3.351051 -1.809787 0.126376
H 2.013182 -1.649089 -1.550466
C 3.606965 0.181026 1.450985
H 2.490280 1.894665 0.790106
C 3.920515 -1.151925 1.207748
H 3.592948 -2.845850 -0.068205
H 4.052685 0.699035 2.289317
H 4.608541 -1.673580 1.858838
F -2.527975 1.550486 1.292196

PhIvinyl_F.log
Energy (E) = -420.416760776 Hartree
Enthalpy (H) = -420.270577 Hartree
Gibbs free energy (G) = -420.319777 Hartree
Charge = 0, Spin = 1
C -3.613041 0.622847 -0.008375
C -2.687693 1.627424 -0.252050
C -1.326973 1.339981 -0.250610
C -0.931998 0.038001 -0.010036
C -1.828363 -0.986317 0.228182
C -3.182997 -0.676830 0.226895
H -4.670136 0.850951 -0.009970
H -3.016261 2.638437 -0.449828
H -0.605889 2.119274 -0.452875
H -1.459389 -1.992061 0.366434
H -3.902920 -1.462845 0.408788
I 1.140680 -0.431131 0.037629
C 1.595991 1.611875 0.509371
H 1.177783 2.003388 1.428922
C 2.441695 2.313657 -0.227912
H 2.758537 3.307637 0.066058
H 2.857006 1.924289 -1.150798
F 0.400614 -2.431438 -0.388209

PhICF2H_Cl.log
Energy (E) = -941.112391141 Hartree
Enthalpy (H) = -940.985741 Hartree
Gibbs free energy (G) = -941.038932 Hartree
Charge = 0, Spin = 1
C -1.347070 -1.396267 -0.675974
C -2.649117 -1.882380 -0.615960
C -3.612233 -1.207108 0.119559
C -3.286598 -0.037226 0.795123
C -1.993803 0.467799 0.747020
C -1.057439 -0.235352 0.015955
H -0.599094 -1.906081 -1.265644
H -2.904064 -2.782734 -1.157151
H -4.623511 -1.587417 0.158816
H -4.039999 0.493919 1.359567
H -1.730216 1.398517 1.228567
I 0.936646 0.495320 -0.028501
C 1.592207 -1.603956 0.374372
H 0.886000 -2.114032 1.029833
F 2.797463 -1.509462 0.956062
F 1.736115 -2.293314 -0.770496
Cl -0.194660 2.931634 -0.357301

PhINMe2_Cl.log
Energy (E) = -837.278426882 Hartree
Enthalpy (H) = -837.091209 Hartree
Gibbs free energy (G) = -837.146127 Hartree
Charge = 0, Spin = 1
C 3.703286 -1.026637 -0.080013
C 3.360002 0.168277 0.537808
C 2.040182 0.604900 0.545267
C 1.098176 -0.191096 -0.072793
C 1.400245 -1.386810 -0.694652
C 2.727751 -1.799812 -0.694802
H 4.733660 -1.354676 -0.085168
H 4.117607 0.772857 1.016524
H 1.761352 1.548402 0.992304
H 0.621671 -1.982217 -1.148621
H 2.992633 -2.727716 -1.182611
I -0.943095 0.428466 -0.056910
N -1.470183 -1.633519 -0.040102
C -1.320267 -2.182021 1.296314
H -1.984016 -1.710439 2.035011
H -1.553896 -3.249642 1.267982
H -0.288665 -2.073305 1.633271
C -2.843836 -1.748027 -0.499091
H -3.110684 -2.806500 -0.539918
H -3.571744 -1.253440 0.162338
H -2.945521 -1.334647 -1.502399
Cl -0.087664 2.956741 -0.081177

PhINTf2_Cl.log
Energy (E) = -2529.36685681 Hartree
Enthalpy (H) = -2529.193491 Hartree
Gibbs free energy (G) = -2529.275639 Hartree
Charge = 0, Spin = 1
C -3.728828 2.310343 1.751052
C -3.841534 2.231465 0.367647
C -3.136154 1.266206 -0.335409
C -2.347878 0.401110 0.397576
C -2.208375 0.440700 1.769441
C -2.917379 1.425945 2.447771
H -4.274371 3.073023 2.288919
H -4.465559 2.930172 -0.170952
H -3.187563 1.204919 -1.412542
H -1.569007 -0.255014 2.293666
H -2.828916 1.494334 3.522484
I -1.261487 -1.057773 -0.675837
N 0.691839 0.078308 0.124422
S 0.811522 1.661129 -0.282936
S 1.462628 -0.595466 1.396594
O -0.168064 1.857290 -1.314759
O 0.886812 2.524387 0.851110
C 2.431975 1.780145 -1.165526
O 0.514545 -1.388314 2.127954
O 2.364027 0.299361 2.047651
C 2.491865 -1.839611 0.504982
F 2.476798 0.863013 -2.119253
F 3.439517 1.609165 -0.335755
F 2.500663 2.982561 -1.707448
F 3.112920 -2.592122 1.390672
F 1.701872 -2.604604 -0.245796
F 3.374355 -1.240904 -0.271751
Cl -3.337699 -2.092813 -1.406386

PhIOSO2Me_Cl.log
Energy (E) = -1366.33903155 Hartree
Enthalpy (H) = -1366.179541 Hartree
Gibbs free energy (G) = -1366.240146 Hartree
Charge = 0, Spin = 1
C -2.110502 3.299733 0.076525
C -1.261261 2.979049 -0.974025
C -0.743331 1.695657 -1.090182
C -1.112740 0.772904 -0.134473
C -1.949565 1.050665 0.924353
C -2.452504 2.341945 1.021275
H -2.505767 4.302414 0.160928
H -0.995685 3.725602 -1.709181
H -0.065457 1.435101 -1.888677
H -2.209910 0.295026 1.650606
H -3.106547 2.594158 1.843874
I -0.296819 -1.179744 -0.283515
O 1.579575 -0.164385 -0.842208
S 2.468554 0.224963 0.369875
O 2.940744 -0.966755 1.026155
O 1.784446 1.211352 1.169383
C 3.815819 0.993325 -0.468995
H 3.437653 1.850457 -1.017346
H 4.519196 1.305576 0.298629
H 4.267804 0.263035 -1.132792

Cl -2.509433 -2.134452 0.232280

PhIOTf_Cl.log
Energy (E) = -1663.95086796 Hartree
Enthalpy (H) = -1663.812083 Hartree
Gibbs free energy (G) = -1663.876867 Hartree
Charge = 0, Spin = 1
C -0.372720 3.684292 0.200387
C -0.431928 2.890863 1.338945
C -0.747040 1.542769 1.236830
C -1.000630 1.045976 -0.027252
C -0.954284 1.803479 -1.182708
C -0.631860 3.147056 -1.054446
H -0.117843 4.730965 0.290970
H -0.219910 3.313268 2.310621
H -0.762352 0.901779 2.104779
H -1.151112 1.366592 -2.150557
H -0.580054 3.768672 -1.936858
I -1.432925 -0.994894 -0.211866
O 0.769350 -1.200121 -0.744935
S 1.831714 -1.187191 0.338894
O 2.692239 -2.325964 0.273816
O 1.266921 -0.781248 1.602025
C 2.851069 0.235567 -0.227563
F 3.334713 0.003523 -1.436340
F 3.852757 0.416240 0.617674
F 2.116118 1.344182 -0.265136
Cl -3.786302 -0.580806 0.246437

PhIPyrroleN_Cl.log
Energy (E) = -912.228901485 Hartree
Enthalpy (H) = -912.051286 Hartree
Gibbs free energy (G) = -912.106180 Hartree
Charge = 0, Spin = 1
C -1.948546 1.364077 0.349182
C -2.207073 2.730358 0.327544
C -1.229378 3.624716 -0.083173
C 0.019351 3.159942 -0.470936
C 0.306817 1.799474 -0.450180
C -0.697241 0.941145 -0.045062
H -2.709134 0.657880 0.648093
H -3.180862 3.085320 0.634527
H -1.439735 4.685149 -0.100755
H 0.785615 3.851060 -0.792883
H 1.285487 1.449178 -0.749083
I -0.249608 -1.166876 0.023833
N 1.795451 -0.657257 0.132726
C 2.398746 -0.027581 1.190804
C 2.618566 -0.569000 -0.964793
C 3.620835 0.435612 0.778153
H 1.909161 0.040302 2.148192
C 3.761896 0.089436 -0.593432
H 2.325313 -0.989242 -1.912541
H 4.341769 0.949001 1.391966
H 4.611102 0.286821 -1.225870
Cl -2.772439 -1.697608 -0.144854

PhISeMe_Cl.log
Energy (E) = -752.046617564 Hartree
Enthalpy (H) = -751.903678 Hartree
Gibbs free energy (G) = -751.959700 Hartree
Charge = 0, Spin = 1
C -2.697204 -2.806292 -0.081734
C -1.585663 -2.782853 -0.913364
C -0.747207 -1.674445 -0.932664
C -1.063312 -0.611720 -0.110670
C -2.164711 -0.595778 0.720675
C -2.984021 -1.718568 0.731792
H -3.343410 -3.673064 -0.070848
H -1.364185 -3.624355 -1.554875
H 0.130956 -1.656111 -1.562690
H -2.395879 0.271525 1.321745
H -3.850490 -1.732560 1.377954
I 0.205534 1.106302 -0.111792
C 2.013948 -1.366077 1.559426
H 1.051713 -1.868846 1.496072
H 2.792603 -2.098392 1.754584
H 2.007574 -0.620797 2.348385
Se 2.445859 -0.533509 -0.165351
Cl -1.983082 2.578957 0.035047

PhISMe_Cl.log
Energy (E) = -1140.82584504 Hartree
Enthalpy (H) = -1140.682249 Hartree
Gibbs free energy (G) = -1140.736033 Hartree
Charge = 0, Spin = 1
C 3.727088 -0.394319 -0.031129
C 3.156541 0.614075 0.733661
C 1.784294 0.834106 0.699835
C 1.022969 0.014324 -0.107619
C 1.553975 -0.999816 -0.878092
C 2.929606 -1.196154 -0.836420
H 4.796149 -0.553547 -0.002522
H 3.775170 1.240873 1.360545
H 1.330340 1.637977 1.260945
H 0.921953 -1.630403 -1.487271
H 3.371078 -1.976660 -1.440320
I -1.097867 0.313382 -0.139553
C -0.848853 -2.590207 1.382969
H -0.967551 -3.666307 1.495169
H 0.209528 -2.344253 1.464570
H -1.408999 -2.090280 2.169671
S -1.479962 -2.153375 -0.247041
Cl -0.595002 2.913840 0.044055

PhISPh_Cl.log
Energy (E) = -1332.38838716 Hartree
Enthalpy (H) = -1332.188247 Hartree
Gibbs free energy (G) = -1332.252128 Hartree
Charge = 0, Spin = 1
C -4.454124 -1.739739 0.002319
C -3.339394 -2.298769 0.611189
C -2.127614 -1.617430 0.614532
C -2.080341 -0.381063 0.001073
C -3.169867 0.206599 -0.609920
C -4.369583 -0.493240 -0.604778
H -5.393411 -2.275222 0.003559
H -3.404654 -3.265481 1.090555
H -1.251397 -2.045623 1.080330
H -3.095479 1.189983 -1.050612
H -5.238286 -0.055833 -1.076482
I -0.229176 0.662003 -0.016683
S 0.964626 -1.532379 -0.587164
C 2.564393 -0.881623 -0.248047
C 3.213330 -1.196462 0.945793
C 3.195308 -0.053589 -1.177721
C 4.481296 -0.695403 1.200700
H 2.714953 -1.835153 1.662546
C 4.458601 0.455564 -0.913028
H 2.689693 0.174543 -2.107308
C 5.104028 0.132894 0.274356
H 4.981919 -0.947306 2.125685
H 4.941113 1.098450 -1.636506
H 6.090531 0.526338 0.477858
Cl -1.598756 2.837692 0.537083

PhIvinyl_Cl.log
Energy (E) = -780.755479505 Hartree
Enthalpy (H) = -780.609746 Hartree
Gibbs free energy (G) = -780.661006 Hartree
Charge = 0, Spin = 1
C -3.698399 0.499331 -0.032763
C -2.869424 1.489655 -0.540394
C -1.488788 1.325301 -0.515548
C -0.984064 0.155644 0.018489
C -1.778393 -0.852929 0.523516
C -3.154982 -0.664810 0.495339
H -4.771306 0.631687 -0.055116
H -3.288939 2.391186 -0.964827
H -0.836951 2.090001 -0.914352
H -1.336706 -1.769657 0.888654
H -3.800264 -1.439349 0.885567
I 1.134936 -0.081828 0.090330
C 1.316633 2.017858 0.499656
H 0.762839 2.375621 1.358018
C 2.132983 2.780904 -0.205708
H 2.291056 3.820638 0.055290
H 2.677172 2.403913 -1.063790
Cl 0.664593 -2.745462 -0.378380

AnthI8BA_B_02_2.log
Energy (E) = -855.395433517 Hartree
Enthalpy (H) = -855.107029 Hartree
Gibbs free energy (G) = -855.176120 Hartree
Charge = 0, Spin = 2
C 6.737557 1.124188 0.008443
C 5.536209 1.736088 -0.168931
C 4.321218 0.992169 -0.113370
C 4.388404 -0.416455 0.133553
C 5.667421 -1.020178 0.314010
C 6.804284 -0.276665 0.253894
C 3.077477 1.594149 -0.292266
C 3.208895 -1.155414 0.189866
C 1.967740 -0.550128 0.011647
C 1.899323 0.854704 -0.234925
C 0.618043 1.446742 -0.413886
H 0.570490 2.514322 -0.602474
C -0.543603 0.732436 -0.360125
C -0.446062 -0.675800 -0.116942
H 3.026960 2.661231 -0.480018
H 7.652590 1.699171 -0.036191
H 5.478656 2.801380 -0.356363
H 5.710984 -2.086328 0.500022
H 7.769011 -0.745717 0.392172
H 3.258881 -2.222668 0.375501
I -2.188859 -1.868555 -0.054714
C 0.752076 -1.290427 0.060960
H 0.804212 -2.356797 0.238534
C -1.860597 1.441162 -0.508385
H -2.550284 0.844278 -1.109214
H -1.692415 2.380203 -1.041030
C -2.499025 1.749461 0.847628
H -2.678686 0.820663 1.391975
H -1.800894 2.339777 1.443019
C -3.806100 2.520300 0.706735
H -4.165703 2.867278 1.678153
H -3.690697 3.408372 0.081857
C -4.911879 1.702646 0.125174
O -4.927611 0.450310 0.075005
O -5.966372 2.181484 -0.354324

FuranIBCMe2S_A_02.log
Energy (E) = -755.890638021 Hartree

Enthalpy (H) = -755.744450 Hartree
Gibbs free energy (G) = -755.794342 Hartree
Charge = 0, Spin = 2
C -0.990292 2.860034 -0.194230
C 0.296305 2.446174 -0.175691
C 0.233069 1.026940 -0.045057
C -1.076920 0.678616 0.006257
O -1.829574 1.802841 -0.081956
H -1.450551 3.826916 -0.284501
H 1.178705 3.056189 -0.252190
I 1.841488 -0.283983 0.050034
S -1.101742 -1.875602 -0.910641
C -1.777113 -0.629101 0.209819
C -1.650439 -1.073685 1.667938
H -2.094322 -0.322181 2.324527
H -2.167798 -2.022683 1.807787
H -0.604678 -1.204190 1.944527
C -3.257071 -0.514566 -0.170364
H -3.736623 -1.485846 -0.052671
H -3.746060 0.200087 0.492470
H -3.378312 -0.176746 -1.197868

Indole_NMe_IBCONAc_A_02_2.log
Energy (E) = -733.972764662 Hartree
Enthalpy (H) = -733.756960 Hartree
Gibbs free energy (G) = -733.821713 Hartree
Charge = 0, Spin = 2
C -1.934580 -0.313910 -0.019493
C -2.377902 1.027281 -0.050268
C -3.741123 1.333846 -0.141947
C -4.629038 0.283291 -0.203618
C -4.197034 -1.057933 -0.176890
C -2.860406 -1.364420 -0.087329
C -0.522544 -0.257555 0.082133
H -4.094900 2.354616 -0.171005
H -5.687530 0.492611 -0.278080
H -4.930848 -1.849996 -0.229706
H -2.517337 -2.390841 -0.068919
N -1.288137 1.853019 0.024156
C -0.151572 1.067213 0.107592
C 1.184406 1.668998 0.161078
O 1.393989 2.858648 0.025272
I 0.694962 -1.937352 0.120541
C -1.354249 3.304034 0.064999
H -2.399497 3.588695 0.141769
H -0.815290 3.688813 0.925633
H -0.922370 3.743124 -0.830511
N 2.214162 0.776508 0.452269
C 3.502565 0.919410 -0.088282
C 3.980692 -0.243632 -0.904182
H 3.296500 -0.434060 -1.730920
H 4.977698 -0.025472 -1.275537
H 3.995772 -1.138630 -0.282415
O 4.154431 1.896896 0.176386

NpthIBCMe2O_C_02.log
Energy (E) = -588.630273378 Hartree
Enthalpy (H) = -588.403154 Hartree
Gibbs free energy (G) = -588.459318 Hartree
Charge = 0, Spin = 2
C 1.317959 1.446028 -0.029443
C 0.024536 0.991985 -0.036027
C -0.175456 -0.413100 -0.020574
C 0.870217 -1.290082 0.001480
H 1.515970 2.510503 -0.045760
H 0.693820 -2.358045 0.011921
O -2.159288 1.520559 -0.864226
I -2.123530 -1.227529 -0.021461
C 3.310716 -1.702743 0.024304
C 4.587425 -1.211907 0.027615
C 4.815736 0.182115 0.011787
C 3.761941 1.054122 -0.006920
C 2.431191 0.574467 -0.009562
C 2.204370 -0.821280 0.006273
H 3.127125 -2.769997 0.035201
H 5.429500 -1.890503 0.041650
H 5.830385 0.556589 0.014052
H 3.929641 2.124192 -0.020385
C -1.166569 1.943230 -0.008493
C -0.818529 3.331731 -0.582341
H -1.735971 3.911570 -0.658430
H -0.138641 3.847099 0.095183
H -0.363765 3.239603 -1.565977
C -1.674308 2.120159 1.425927
H -0.894104 2.558840 2.048915
H -2.545024 2.775612 1.417916
H -1.962915 1.160753 1.852808

NpthISO2NMe_D_02.log
Energy (E) = -1038.62044493 Hartree
Enthalpy (H) = -1038.425559 Hartree
Gibbs free energy (G) = -1038.484409 Hartree
Charge = 0, Spin = 2
C 2.822597 -2.350394 -0.466260
C 3.482396 -1.195368 -0.170445
C 2.769780 -0.002763 0.081288
C 1.341087 0.037819 0.008696
C 0.696564 -1.221769 -0.165772
C 1.419757 -2.359984 -0.423901
H 4.588052 1.060183 0.488371
H 3.358096 -3.264622 -0.679290
H 4.563196 -1.163405 -0.122293
C 3.511611 1.156087 0.420080
C 0.757498 1.343680 0.124640
H 0.897328 -3.296171 -0.558946
C 1.510406 2.440178 0.455946
C 2.899044 2.347893 0.654838
H 1.019636 3.400864 0.503940
H 3.464793 3.226589 0.928552
I -1.344601 -1.635037 0.210811
S -0.863547 1.789827 -0.442662
O -1.139434 1.046570 -1.644840
O -0.948394 3.232782 -0.487949
N -1.861585 1.381692 0.815952
C -3.251894 1.430187 0.429241
H -3.851212 0.977142 1.214778
H -3.545247 2.480973 0.324578
H -3.433386 0.938121 -0.529550

perF_CMe2O_02.log
Energy (E) = -831.916254528 Hartree
Enthalpy (H) = -831.767225 Hartree
Gibbs free energy (G) = -831.825507 Hartree
Charge = 0, Spin = 2
C -2.065368 -1.516574 -0.029586
C -2.676302 -0.279485 0.018253
C -1.898610 0.869933 0.015646
C -0.514276 0.832287 -0.058326
C 0.083657 -0.430497 -0.067587
C -0.680834 -1.583046 -0.058037
O 1.484043 1.909858 -0.795938
I 2.164594 -0.686464 0.020130
F -0.123053 -2.787178 -0.066548
F -2.791536 -2.622039 -0.029160
F -3.995405 -0.191161 0.071210
F -2.558779 2.025637 0.110879
C 0.347462 2.099905 -0.048910
C -0.290998 3.294412 -0.798217
H -1.053420 3.760153 -0.180966
H 0.499310 4.017437 -0.991793
H -0.721972 2.973245 -1.743228
C 0.649358 2.500630 1.394981
H 1.264946 3.399625 1.395268
H -0.279868 2.698388 1.931148
H 1.189594 1.702137 1.903481

perF_NMeCO2_02.log
Energy (E) = -921.954883791 Hartree
Enthalpy (H) = -921.834892 Hartree
Gibbs free energy (G) = -921.896363 Hartree
Charge = 0, Spin = 2
C 2.499000 -0.957398 0.119483
C 2.749428 0.397232 -0.020759
C 1.683523 1.269095 -0.172146
C 0.374171 0.807387 -0.192129
C 0.129079 -0.561347 -0.063184
C 1.193322 -1.430118 0.097697
I -1.814762 -1.317140 -0.128527
F 1.006615 -2.734930 0.225807
F 3.505161 -1.799402 0.266731
F 3.991068 0.847881 -0.004589
F 1.927715 2.564658 -0.304322
N -0.668459 1.747592 -0.362055
C -1.013596 2.228512 -1.689443
H -1.335005 1.398881 -2.320160
H -1.831741 2.937975 -1.589116
H -0.159607 2.729134 -2.143916
C -1.359328 2.139982 0.729956
O -2.326226 2.929507 0.718809
O -1.076362 1.726165 1.892439

PyIBCMe2O_D_02.log
Energy (E) = -451.163458306 Hartree
Enthalpy (H) = -450.997919 Hartree
Gibbs free energy (G) = -451.048115 Hartree
Charge = 0, Spin = 2
C -1.500823 -2.785469 0.019771
C -2.589123 -1.929143 0.048218
C -1.256220 -0.053245 -0.032235
C -0.109045 -0.848087 -0.037880
H -1.637479 -3.857160 0.033420
H -3.599588 -2.317591 0.091588
O -0.180958 1.935081 -0.836115
I 1.827414 -0.021544 -0.013867
C -0.232354 -2.227182 -0.015255
H 0.648322 -2.854337 -0.021437
N -2.465628 -0.605527 0.023932
C -1.184313 1.471456 -0.022553
C -2.450661 2.071405 -0.664351
H -3.310589 1.757282 -0.077505
H -2.364549 3.155859 -0.662234
H -2.564945 1.710790 -1.683750
C -1.053369 1.970532 1.417709
H -1.010357 3.059164 1.416966
H -1.909887 1.640509 2.006095
H -0.141347 1.583750 1.872671

PyrroleNMeIBCMe2O_A_02.log
Energy (E) = -452.353357008 Hartree
Enthalpy (H) = -452.164076 Hartree
Gibbs free energy (G) = -452.216098 Hartree
Charge = 0, Spin = 2

C 1.473589 2.395152 0.008022
C 0.112777 2.259995 -0.027288
C -0.139304 0.871554 -0.073239
C 1.058206 0.197179 -0.074446
H 2.091103 3.276809 0.042982
H -0.615815 3.051383 -0.020915
I -2.021957 -0.015435 -0.003500
O 0.212202 -1.917718 -0.700639
N 2.047983 1.151534 -0.013848
C 3.479882 0.954390 0.067718
H 3.738063 0.288856 0.891117
H 3.887012 0.549610 -0.857278
H 3.939979 1.921263 0.255319
C 1.236842 -1.303040 -0.016023
C 2.463173 -1.828958 -0.798170
H 3.373396 -1.664439 -0.225546
H 2.337130 -2.901627 -0.935121
H 2.535348 -1.349991 -1.772014
C 1.300340 -1.778608 1.436634
H 1.439961 -2.859388 1.464622
H 2.123802 -1.294292 1.965680
H 0.367201 -1.525106 1.939445

AnthI8BA_B_F.log
Energy (E) = -955.167199093 Hartree
Enthalpy (H) = -954.872440 Hartree
Gibbs free energy (G) = -954.939208
Hartree
Charge = 0, Spin = 1
C 6.784775 0.660784 -0.102685
C 5.674675 1.434962 0.025659
C 4.372415 0.854354 0.001957
C 4.256243 -0.564215 -0.160682
C 5.445182 -1.340623 -0.291470
C 6.668825 -0.749488 -0.263626
C 3.220000 1.625777 0.133057
C 2.991496 -1.145261 -0.185984
C 1.843381 -0.368130 -0.057003
C 1.954951 1.044281 0.106047
C 0.763582 1.806508 0.245818
H 0.861339 2.876382 0.392465
C -0.493881 1.269774 0.213724
C -0.550337 -0.146942 0.040878
H 3.310842 2.699077 0.257523
H 7.767208 1.112679 -0.083176
H 5.757348 2.507792 0.148368
H 5.348456 -2.412312 -0.413374
H 7.564477 -1.347237 -0.363742
H 2.898544 -2.218541 -0.305993
I -2.367308 -1.240518 -0.029628
C 0.542703 -0.939367 -0.084654
H 0.443800 -2.011790 -0.178814
C -1.670315 2.199575 0.383933
H -2.112950 2.402515 -0.592925
H -1.257116 3.149862 0.718663
C -2.750588 1.734130 1.380723
H -2.375219 0.880499 1.945969
H -2.901994 2.518885 2.119569
C -4.121828 1.409967 0.752377
H -4.558241 0.529392 1.229261
H -4.820252 2.229033 0.899347
C -4.066237 1.191748 -0.747031
O -3.207457 0.279659 -1.204026
O -4.727169 1.845680 -1.513177
F -1.352459 -2.670943 1.020950

FuranIBCMe2S_A_F.log
Energy (E) = -855.655340628 Hartree
Enthalpy (H) = -855.505384 Hartree
Gibbs free energy (G) = -855.556525
Hartree
Charge = 0, Spin = 1
C -1.039829 2.912810 -0.143644
C 0.230457 2.443099 -0.153914
C 0.070482 1.032550 -0.122832
C -1.242434 0.739837 -0.075404
O -1.942707 1.891860 -0.090563
H -1.451496 3.904564 -0.183228
H 1.155423 2.988398 -0.181741
I 1.409396 -0.520551 -0.004728
S -0.703171 -1.829037 -0.476609
C -1.896927 -0.583857 0.131645
C -2.183604 -0.785450 1.619046
H -2.865764 -0.012176 1.978483
H -2.638311 -1.762608 1.779486
H -1.259327 -0.733447 2.195458
C -3.173016 -0.715256 -0.692197
H -3.603554 -1.706767 -0.553117
H -3.899564 0.027973 -0.360226
H -2.969484 -0.565362 -1.750811
F 2.781748 1.035105 0.255589

Indole_NMe_IBCONAc_A_F.log
Energy (E) = -833.760061952 Hartree
Enthalpy (H) = -833.539410 Hartree
Gibbs free energy (G) = -833.603662
Hartree
Charge = 0, Spin = 1
C 2.002884 -0.420857 0.000066
C 2.582232 0.873891 -0.000091
C 3.966302 1.057455 0.000043
C 4.755602 -0.072151 0.000217
C 4.192882 -1.362219 0.000301
C 2.829455 -1.553400 0.000251
C 0.610137 -0.161707 0.000084
H 4.397874 2.049310 0.000057
H 5.831910 0.033385 0.000315
H 4.849780 -2.220921 0.000433
H 2.388218 -2.538610 0.000322
N 1.592172 1.828217 -0.000238
C 0.386128 1.182666 -0.000021
C -0.970438 1.759914 -0.000296
O -1.202162 2.947739 -0.000829
I -1.091235 -1.298261 -0.000095
C 1.836771 3.258536 0.000009
H 2.406730 3.537716 -0.885975
H 0.884083 3.775542 -0.001899
H 2.403355 3.538198 0.888017
F 0.127477 -2.941161 -0.000160
N -1.905468 0.724563 0.000038
C -3.288083 0.948840 0.000399
C -4.122716 -0.313860 -0.000145
H -3.919710 -0.918136 0.886348
H -5.169406 -0.028576 0.000119
H -3.919838 -0.917273 -0.887244
O -3.792673 2.041700 0.001140

NaphIBMeUreaMe_F.log
Energy (E) = -797.853050067 Hartree
Enthalpy (H) = -797.607441 Hartree
Gibbs free energy (G) = -797.669274
Hartree
Charge = 0, Spin = 1
C -2.625714 -0.594924 0.196345
C -2.753367 0.806706 0.038626
C -1.600926 1.594655 -0.176300
C -0.339738 1.046916 -0.234938
C -0.259707 -0.357127 -0.061214
C -1.333280 -1.163110 0.138362
H -1.726417 2.661873 -0.299121
H -1.199689 -2.233918 0.208212
I 1.640470 -1.205640 -0.165288
C 1.977322 1.772588 0.197828
O 2.710494 2.741607 0.281698
N 2.215800 0.580397 0.811580
C 3.426821 0.503218 1.609640
H 3.476765 1.363147 2.273964
H 3.397079 -0.405829 2.210388
H 4.334739 0.505344 1.000831
N 0.783274 1.835289 -0.538373
C 0.558147 3.103455 -1.215003
H -0.168528 2.953285 -2.010598
H 0.199717 3.878527 -0.533741
H 1.501167 3.440709 -1.631419
C -4.045904 1.381453 0.099256
C -5.147036 0.596814 0.304576
C -5.015878 -0.801914 0.455844
C -3.780336 -1.384931 0.401598
H -4.145702 2.453233 -0.019651
H -6.129522 1.046921 0.349944
H -5.897527 -1.407748 0.613290
H -3.664353 -2.455471 0.513376
F 0.670832 -2.862958 -0.956553

NpthIBCMe2O_C_F.log
Energy (E) = -688.420741082 Hartree
Enthalpy (H) = -688.189281 Hartree
Gibbs free energy (G) = -688.246037
Hartree
Charge = 0, Spin = 1
C 1.450219 1.538686 -0.021838
C 0.162434 1.077921 -0.035976
C -0.021522 -0.312332 -0.043948
C 0.974383 -1.231835 -0.023510
H 1.650678 2.603820 -0.007447
H 0.754686 -2.290158 -0.009159
O -2.153931 1.204324 -0.535240
I -2.054609 -0.799576 -0.085927
C 3.409547 -1.646687 -0.004237
C 4.690526 -1.168013 0.000925
C 4.929858 0.223883 -0.003470
C 3.884442 1.105713 -0.012780
C 2.547452 0.642215 -0.018292
C 2.309729 -0.756997 -0.012608
H 3.214207 -2.711529 -0.000990
H 5.526415 -1.854120 0.008717
H 5.947501 0.590163 0.000813
H 4.063122 2.173937 -0.016387
C -1.094002 1.935277 0.036402
C -0.964429 3.216338 -0.770326
H -1.916189 3.744839 -0.736407
H -0.193277 3.867108 -0.357142
H -0.725445 2.985085 -1.806820
C -1.394620 2.250919 1.501623
H -0.583736 2.826305 1.949351
H -2.319889 2.824226 1.559443
H -1.515453 1.330587 2.075931
F -1.453442 -2.733097 0.291598

NpthISO2NMe_D_F.log
Energy (E) = -1138.40184513 Hartree
Enthalpy (H) = -1138.201846 Hartree
Gibbs free energy (G) = -1138.261397
Hartree
Charge = 0, Spin = 1
C 1.608261 3.222446 -0.292224
C 2.731945 2.452718 -0.254220
C 2.650146 1.048799 -0.122662
C 1.383194 0.393305 -0.026106
C 0.252139 1.253129 -0.075577

C 0.346403 2.608028 -0.205342
H 4.784461 0.840353 -0.158705
H 1.668086 4.296558 -0.391528
H 3.715345 2.900091 -0.321618
C 3.850475 0.297791 -0.085318
C 1.424110 -1.026135 0.105434
H -0.553314 3.204907 -0.213018
C 2.600953 -1.722326 0.124533
C 3.837501 -1.059176 0.033248
H 2.556791 -2.800053 0.200178
H 4.756957 -1.626150 0.053335
I -1.751887 0.549476 0.030143
S -0.013147 -2.031840 0.300428
O -0.478452 -1.877513 1.657829
O 0.269517 -3.358648 -0.186096
N -0.995692 -1.282270 -0.766847
C -2.033507 -2.106334 -1.385907
H -2.458067 -1.546464 -2.217781
H -1.578980 -3.015626 -1.772856
H -2.834196 -2.386283 -0.694711
F -2.251680 2.467952 0.645687

perF_CMe2O_F.log
Energy (E) = -931.695507349 Hartree
Enthalpy (H) = -931.541912 Hartree
Gibbs free energy (G) = -931.600898
Hartree
Charge = 0, Spin = 1
C -2.245017 -1.421434 -0.054267
C -2.812309 -0.165419 0.060804
C -2.002190 0.961547 0.070140
C -0.629148 0.851677 -0.035728
C -0.084958 -0.421497 -0.089775
C -0.863071 -1.562355 -0.129072
O 1.535212 1.568234 -0.638086
I 2.011629 -0.334835 -0.045144
F -0.387075 -2.780521 -0.277295
F -3.019620 -2.491373 -0.106292
F -4.125451 -0.042434 0.143790
F -2.597177 2.145955 0.201692
C 0.345473 2.034780 -0.058288
C -0.127737 3.172346 -0.953247
H -0.976396 3.700648 -0.527417
H 0.705177 3.866104 -1.059344
H -0.389398 2.793572 -1.939932
C 0.568883 2.523103 1.370687
H 1.285304 3.343992 1.356520
H -0.367720 2.872990 1.805809
H 0.963455 1.722390 1.998283
F 2.016208 -2.268936 0.568758

perF_NMeCO2_F.log
Energy (E) = -1021.73187945 Hartree
Enthalpy (H) = -1021.606371 Hartree
Gibbs free energy (G) = -1021.665903
Hartree
Charge = 0, Spin = 1
C 2.487387 -1.144558 0.173247
C 2.881612 0.174856 0.024521
C 1.943694 1.188762 -0.099264
C 0.576070 0.916078 -0.137517
C 0.216544 -0.427707 -0.016215
C 1.134680 -1.442040 0.176677
I -1.805676 -0.822488 -0.024830
F 0.755729 -2.686813 0.388422
F 3.388613 -2.096344 0.337855
F 4.165688 0.478010 0.050168
F 2.395350 2.437454 -0.145617
F -1.402099 -2.378465 -1.229365
N -0.377380 1.924260 -0.284095
C -0.085166 3.115157 -1.081056
H 0.609883 2.848157 -1.872535
H -1.017228 3.462928 -1.515895
H 0.334698 3.917176 -0.478499
C -1.498577 2.019837 0.563523
O -2.088694 3.062697 0.674769
O -1.817788 0.900665 1.192099

PyIBCMe2O_D_F.log
Energy (E) = -550.950474558 Hartree
Enthalpy (H) = -550.780419 Hartree
Gibbs free energy (G) = -550.830965
Hartree
Charge = 0, Spin = 1
C 1.625100 2.773865 -0.029941
C 2.717201 1.915291 -0.007339
C 1.397422 0.058236 -0.040803
C 0.274804 0.861189 -0.066805
H 1.773446 3.843791 -0.026563
H 3.723351 2.316059 0.016075
O -0.093441 -1.728311 -0.494267
I -1.474212 -0.266124 -0.071536
C 0.344283 2.234238 -0.048118
H -0.553774 2.833855 -0.039261
N 2.616205 0.585491 -0.002335
C 1.186137 -1.445070 0.026559
C 2.202487 -2.179626 -0.827692
H 3.211266 -1.950428 -0.488440
H 2.022202 -3.250813 -0.748580
H 2.099229 -1.879759 -1.869251
C 1.284821 -1.873613 1.488809
H 1.123954 -2.949072 1.557263
H 2.266551 -1.626014 1.892473
H 0.525546 -1.366689 2.088447
F -2.424217 1.532737 0.267969

PyrroleNMeIBCMe2O_A_F.log
Energy (E) = -552.135737333 Hartree
Enthalpy (H) = -551.942204 Hartree
Gibbs free energy (G) = -551.995221
Hartree
Charge = 0, Spin = 1
C -1.671115 2.396416 0.005182
C -0.301566 2.309011 0.046890
C -0.044676 0.928289 0.098492
C -1.204184 0.217780 0.072071
H -2.312467 3.261557 -0.012994
H 0.417548 3.107234 0.040864
I 1.611639 -0.274855 0.053574
O 0.110721 -1.676860 0.402304
N -2.216483 1.131739 0.011436
C -3.637219 0.853653 0.002889
H -3.879824 0.115687 -0.760485
H -3.974968 0.484520 0.970591
H -4.166881 1.774511 -0.227350
C -1.176992 -1.288786 -0.023522
C -2.165042 -1.981225 0.905829
H -3.196115 -1.830485 0.584022
H -1.952792 -3.049602 0.890080
H -2.043042 -1.616388 1.924634
C -1.394154 -1.720766 -1.473268
H -1.332292 -2.807091 -1.538067
H -2.367148 -1.397551 -1.848970
H -0.618969 -1.284053 -2.105099
F 2.689932 1.446159 -0.206946

AnthI8BA_B_OMe.log
Energy (E) = -970.396812582 Hartree
Enthalpy (H) = -970.060434 Hartree
Gibbs free energy (G) = -970.131747
Hartree
Charge = 0, Spin = 1
C -6.837785 -0.681003 -0.021765
C -5.735744 -1.430061 0.247335
C -4.426847 -0.881737 0.105739
C -4.295257 0.477608 -0.326970
C -5.476230 1.229251 -0.599198
C -6.706478 0.670306 -0.451979
C -3.282691 -1.628811 0.375344
C -3.023826 1.026415 -0.469350
C -1.883699 0.273739 -0.199183
C -2.010740 -1.080060 0.230516
C -0.827256 -1.819136 0.498841
H -0.937652 -2.843531 0.836967
C 0.437777 -1.317128 0.356061
C 0.512995 0.044425 -0.070651
H -3.385110 -2.657790 0.701634
H -7.825237 -1.108135 0.088897
H -5.829848 -2.458340 0.573940
H -5.368085 2.255886 -0.926616
H -7.595752 1.248956 -0.661569
H -2.919403 2.055097 -0.795290
I 2.337480 1.114817 -0.317114
C -0.575101 0.810853 -0.334130
H -0.463128 1.846443 -0.627992
C 1.597664 -2.235300 0.657352
H 1.986737 -2.630937 -0.282698
H 1.173829 -3.090697 1.181999
C 2.742808 -1.639812 1.498128
H 2.417399 -0.691697 1.931291
H 2.920149 -2.292892 2.351115
C 4.078977 -1.472174 0.746200
H 4.567533 -0.541457 1.045305
H 4.763104 -2.280724 0.989064
C 3.936605 -1.497583 -0.768827
O 3.072093 -0.656568 -1.307579
O 4.554783 -2.291804 -1.438936
O 1.416331 2.716287 0.584912
C 1.232078 2.551371 1.967389
H 0.771080 3.457104 2.361971
H 0.568584 1.705479 2.191893
H 2.180188 2.391596 2.494807

FuranIBCMe2S_A_OMe.log
Energy (E) = -870.880539310 Hartree
Enthalpy (H) = -870.689008 Hartree
Gibbs free energy (G) = -870.744723
Hartree
Charge = 0, Spin = 1
C 0.773708 2.959559 -0.395296
C -0.395374 2.282240 -0.487153
C -0.023020 0.924529 -0.288603
C 1.306231 0.862958 -0.081706
O 1.810870 2.113476 -0.144940
H 1.024970 3.999333 -0.497946
H -1.379631 2.664532 -0.688165
I -1.137319 -0.808575 -0.187833
S 1.271191 -1.783708 -0.330290
C 2.152819 -0.308369 0.286375
C 3.516108 -0.239169 -0.394212
H 4.059724 0.641920 -0.048715
H 4.095877 -1.127011 -0.142712
H 3.406213 -0.188933 -1.475977
C 2.311080 -0.362661 1.806327
H 2.908251 -1.231306 2.082567
H 2.807043 0.540541 2.169411
H 1.335837 -0.447402 2.287199
O -2.815889 0.473500 -0.150586
C -3.095129 0.964931 1.130668
H -3.968102 1.618484 1.067904

H -3.326123 0.168489 1.849013
H -2.263619 1.555212 1.543018

Indole_NMe_IBCONAc_A_OMe.log
Energy (E) = -848.987945860 Hartree
Enthalpy (H) = -848.725454 Hartree
Gibbs free energy (G) = -848.793814 Hartree
Charge = 0, Spin = 1
C -1.982877 0.184653 0.135672
C -2.513384 -1.123675 -0.000987
C -3.887145 -1.368931 0.028158
C -4.723782 -0.288370 0.207086
C -4.212771 1.012458 0.363378
C -2.858529 1.262923 0.332034
C -0.578604 -0.011288 0.051005
H -4.275737 -2.372531 -0.080991
H -5.793627 -0.443238 0.236240
H -4.900788 1.832201 0.517526
H -2.466981 2.258853 0.476033
N -1.487993 -2.027975 -0.138993
C -0.306676 -1.339484 -0.097118
C 1.070702 -1.884075 -0.144838
O 1.310982 -3.061460 -0.312827
I 1.066301 1.222683 0.133122
C -1.682752 -3.460334 -0.270816
H -2.270539 -3.673185 -1.163628
H -0.712786 -3.937218 -0.350036
H -2.212282 -3.844056 0.601199
N 1.967953 -0.847870 0.031324
C 3.346712 -1.051941 0.088325
C 4.154628 0.225022 0.202803
H 3.900640 0.766647 1.116180
H 5.207239 -0.037191 0.229214
H 3.971612 0.881099 -0.650626
O 3.882948 -2.132159 0.060795
O -0.164401 2.870965 0.125244
C -0.571166 3.243463 -1.171030
H -1.222367 4.113841 -1.081953
H 0.275910 3.516299 -1.809403
H -1.136348 2.445905 -1.669295

NaphIBMeUreaMe_OMe.log
Energy (E) = -813.074118856 Hartree
Enthalpy (H) = -812.787144 Hartree
Gibbs free energy (G) = -812.853959 Hartree
Charge = 0, Spin = 1
C -2.638509 -0.551106 0.210922
C -2.845834 0.836239 0.020728
C -1.738924 1.681952 -0.206903
C -0.445950 1.208889 -0.247310
C -0.282589 -0.186482 -0.046288
C -1.314677 -1.042996 0.162843
H -1.924149 2.737566 -0.352282
H -1.133878 -2.104718 0.256194
I 1.668807 -0.936903 -0.163436
C 1.812102 2.087174 0.205194
O 2.481763 3.105251 0.262201
N 2.099046 0.930835 0.850550
C 3.313719 0.941838 1.643565
H 3.302451 1.790746 2.325072
H 3.363125 0.023560 2.229987
H 4.217223 1.027706 1.032898
N 0.625707 2.058267 -0.557620
C 0.328667 3.297541 -1.257586
H -0.379112 3.089993 -2.057669
H -0.084002 4.061626 -0.594207
H 1.253482 3.685660 -1.670421
C -4.169627 1.336953 0.067099
C -5.224545 0.495852 0.290125
C -5.013691 -0.889193 0.474266
C -3.746345 -1.400924 0.434018
H -4.330146 2.398308 -0.076240
H -6.231246 0.889966 0.324684
H -5.859154 -1.541132 0.645698
H -3.570353 -2.460528 0.571132
O 0.851773 -2.683543 -0.992819
C 0.987064 -3.794787 -0.152173
H 0.644012 -4.680528 -0.690087
H 2.026176 -3.979592 0.151806
H 0.392076 -3.705873 0.768963

NpthIBCMe2O_C_OMe.log
Energy (E) = -703.646584241 Hartree
Enthalpy (H) = -703.373466 Hartree
Gibbs free energy (G) = -703.434605 Hartree
Charge = 0, Spin = 1
C 1.636200 1.574898 0.128709
C 0.318375 1.219529 0.029618
C 0.031614 -0.140351 -0.152875
C 0.962228 -1.123721 -0.236624
H 1.913079 2.612496 0.276009
H 0.670636 -2.155025 -0.387452
O -1.951651 1.588631 -0.532486
I -2.037572 -0.495128 -0.297448
C 3.360757 -1.722380 -0.235800
C 4.672427 -1.346570 -0.144135
C 5.011414 0.011821 0.042463
C 4.032719 0.963024 0.132110
C 2.666201 0.606285 0.041240
C 2.328522 -0.759439 -0.143612
H 3.089763 -2.760751 -0.380157
H 5.456241 -2.088419 -0.214269
H 6.052310 0.297032 0.113458
H 4.287826 2.006151 0.273233
C -0.874171 2.162484 0.151814
C -0.598343 3.512869 -0.492101
H -1.507772 4.110057 -0.438915
H 0.198198 4.049236 0.025186
H -0.325713 3.379919 -1.537745
C -1.208815 2.339543 1.635201
H -0.378107 2.797853 2.173636
H -2.091784 2.972585 1.724821
H -1.423159 1.373888 2.097391
O -1.641071 -2.523886 -0.081038
C -1.487098 -2.920600 1.255558
H -1.290573 -3.994078 1.279004
H -0.644437 -2.412023 1.744951
H -2.385585 -2.729452 1.854414

NpthISO2NMe_D_OMe.log
Energy (E) = -1153.62800169 Hartree
Enthalpy (H) = -1153.386397 Hartree
Gibbs free energy (G) = -1153.450273 Hartree
Charge = 0, Spin = 1
C 0.494515 3.467714 -0.631874
C 1.810630 3.174904 -0.433830
C 2.229906 1.854798 -0.157410
C 1.284130 0.785013 -0.074887
C -0.073025 1.156526 -0.279207
C -0.458718 2.437145 -0.551202
H 4.287522 2.450044 -0.034866
H 0.170934 4.475279 -0.850088
H 2.566814 3.947698 -0.487328
C 3.611566 1.606987 0.032637
C 1.824184 -0.507656 0.188393
H -1.509449 2.654002 -0.688273
C 3.165826 -0.714454 0.351588
C 4.079488 0.352525 0.282835
H 3.508494 -1.725838 0.521576
H 5.135547 0.168471 0.418616
I -1.717497 -0.206204 -0.199838
S 0.838139 -1.963201 0.366366
O 0.218085 -1.905926 1.672581
O 1.649040 -3.116182 0.056498
N -0.222746 -1.700725 -0.832756
C -0.879933 -2.900006 -1.350295
H -1.411258 -2.630239 -2.261951
H -0.130059 -3.650055 -1.595215
H -1.590120 -3.344112 -0.643390
O -2.960400 1.388536 0.291813
C -2.855279 1.811587 1.627611
H -3.645983 2.537792 1.821449
H -2.968839 0.983936 2.336178
H -1.890807 2.296204 1.828836

perF_CMe2O_OMe.log
Energy (E) = -946.924155974 Hartree
Enthalpy (H) = -946.728938 Hartree
Gibbs free energy (G) = -946.792170 Hartree
Charge = 0, Spin = 1
C -2.041926 -1.716292 -0.187096
C -2.835502 -0.613790 0.070108
C -2.258615 0.645287 0.156823
C -0.898700 0.823059 -0.014625
C -0.117683 -0.303790 -0.212551
C -0.666717 -1.566573 -0.323848
O 1.035646 2.016042 -0.631618
I 1.946661 0.175193 -0.240184
F 0.032617 -2.656149 -0.590727
F -2.592116 -2.913547 -0.307189
F -4.141252 -0.762080 0.215609
F -3.066465 1.669913 0.426494
C -0.182776 2.181886 0.022683
C -0.937788 3.258463 -0.749360
H -1.850784 3.566398 -0.246508
H -0.271307 4.115920 -0.832955
H -1.172522 2.906941 -1.753015
C 0.008308 2.594983 1.482052
H 0.541927 3.544680 1.511782
H -0.953951 2.708312 1.983020
H 0.594169 1.847427 2.020120
O 2.449529 -1.768886 0.173348
C 2.194978 -2.160011 1.495070
H 2.561439 -3.178384 1.625883
H 2.698540 -1.515955 2.226364
H 1.121524 -2.160380 1.728014

perF_NMeCO2_OMe.log
Energy (E) = -1036.96461135 Hartree
Enthalpy (H) = -1036.797338 Hartree
Gibbs free energy (G) = -1036.861806 Hartree
Charge = 0, Spin = 1
C -2.388929 -1.351150 -0.322593
C -2.946827 -0.122937 -0.009236
C -2.146696 0.991348 0.190080
C -0.753144 0.912524 0.138788
C -0.222891 -0.349344 -0.147343
C -1.010884 -1.453935 -0.411656
I 1.843782 -0.489996 -0.237132
F -0.483252 -2.612791 -0.764391
F -3.162445 -2.398549 -0.554596
F -4.260189 -0.000260 0.051950
F -2.760627 2.152458 0.394483
N 0.060323 2.021127 0.349081

C -0.343562 3.077903 1.273295
H -0.948768 2.644625 2.065751
H 0.556827 3.512051 1.697308
H -0.902581 3.867699 0.776310
C 1.100922 2.360385 -0.557994
O 1.526438 3.489869 -0.563867
O 1.505396 1.375743 -1.316738
O 1.789385 -2.206615 0.837697
C 1.458830 -2.024770 2.194219
H 1.490609 -3.002822 2.672270
H 2.161737 -1.361617 2.708942
H 0.447416 -1.617852 2.314760

PyIBCMe2O_D_OMe.log
Energy (E) = -566.176174989 Hartree
Enthalpy (H) = -565.964483 Hartree
Gibbs free energy (G) = -566.019379 Hartree
Charge = 0, Spin = 1
C -0.996182 3.036665 0.259837
C -2.266590 2.525971 0.025432
C -1.515158 0.372757 -0.009108
C -0.227475 0.819883 0.209119
H -0.847678 4.101005 0.369690
H -3.114548 3.195303 -0.057520
O -0.647321 -1.783155 0.428709
I 1.165202 -0.742968 0.269303
C 0.074640 2.154708 0.344872
H 1.091792 2.477704 0.517597
N -2.529579 1.226472 -0.112872
C -1.721502 -1.123964 -0.183847
C -3.004060 -1.574004 0.495208
H -3.861559 -1.051562 0.074154
H -3.118277 -2.647416 0.349085
H -2.949037 -1.368481 1.563222
C -1.767757 -1.421525 -1.683258
H -1.901285 -2.493413 -1.827456
H -2.589868 -0.884842 -2.157532
H -0.833170 -1.119080 -2.161474
O 2.654144 0.706864 0.151787
C 2.923512 1.119073 -1.161459
H 3.712839 1.872481 -1.134459
H 3.266695 0.294613 -1.797549
H 2.044297 1.568664 -1.645473

PyrroleNMeIBCMe2O_A_OMe.log
Energy (E) = -567.362818571 Hartree
Enthalpy (H) = -567.127669 Hartree
Gibbs free energy (G) = -567.185055 Hartree
Charge = 0, Spin = 1
C -1.447933 2.534604 0.244996
C -0.120596 2.208223 0.374247
C -0.092900 0.805215 0.266812
C -1.344857 0.312874 0.070884
H -1.936961 3.493304 0.290329
H 0.710015 2.870279 0.541604
I 1.376353 -0.633992 0.219204
O -0.404930 -1.803844 0.289241
N -2.186648 1.388862 0.053548
C -3.627402 1.362295 -0.085861
H -3.919020 0.753853 -0.940576
H -4.105270 0.964897 0.808841
H -3.976312 2.378715 -0.250302
C -1.563534 -1.163869 -0.171066
C -2.736275 -1.738009 0.616980
H -3.697181 -1.384200 0.240479
H -2.705904 -2.822492 0.517408
H -2.640661 -1.483597 1.671839
C -1.741957 -1.419349 -1.669315
H -1.857993 -2.490261 -1.836998
H -2.615268 -0.900390 -2.071294
H -0.856740 -1.072978 -2.205467
O 2.776205 0.890824 0.208986
C 2.982541 1.440991 -1.064503
H 3.707792 2.251811 -0.974968
H 3.380908 0.709144 -1.777923
H 2.059670 1.857790 -1.490501

AnthI8BA_B_OCF3.log
Energy (E) = -1268.05853149 Hartree
Enthalpy (H) = -1267.743069 Hartree
Gibbs free energy (G) = -1267.819905 Hartree
Charge = 0, Spin = 1
C -7.070190 -0.511052 -0.013072
C -6.045268 -1.365371 0.247401
C -4.690255 -0.940625 0.118141
C -4.429559 0.406633 -0.293444
C -5.532599 1.271183 -0.556506
C -6.810520 0.828391 -0.421202
C -3.622867 -1.796265 0.381336
C -3.111802 0.835924 -0.423966
C -2.049884 -0.025268 -0.160469
C -2.304867 -1.366995 0.250309
C -1.197688 -2.219256 0.510802
H -1.404895 -3.237165 0.821527
C 0.108663 -1.833533 0.387831
C 0.306242 -0.477235 -0.014328
H -3.823457 -2.815233 0.692833
H -8.093995 -0.844465 0.088709
H -6.237591 -2.384719 0.558317
H -5.326345 2.288130 -0.866034
H -7.640496 1.491632 -0.622857
H -2.908482 1.855326 -0.730952
I 2.243541 0.369836 -0.204017
C -0.697758 0.393456 -0.281629
H -0.496163 1.413655 -0.576203
C 1.180090 -2.864855 0.649822
H 1.531943 -3.257217 -0.306982
H 0.677478 -3.699801 1.135441
C 2.374473 -2.421174 1.516603
H 2.139607 -1.474668 2.003929
H 2.494230 -3.135773 2.328571
C 3.723863 -2.334768 0.770863
H 4.291174 -1.465331 1.110415
H 4.334025 -3.210108 0.974540
C 3.587643 -2.289844 -0.738606
O 2.831406 -1.319965 -1.269589
O 4.089505 -3.116067 -1.454304
O 1.407505 2.032719 0.944607
C 1.546451 3.208165 0.397790
F 2.822083 3.458240 0.002293
F 0.799966 3.364145 -0.730532
F 1.193834 4.190492 1.224895

FuranIBCMe2S_A_OCF3.log
Energy (E) = -1168.55146462 Hartree
Enthalpy (H) = -1168.380857 Hartree
Gibbs free energy (G) = -1168.442128 Hartree
Charge = 0, Spin = 1
C -1.012216 3.026931 -0.016520
C 0.082149 2.233949 -0.099244
C -0.449456 0.918308 -0.033555
C -1.791614 0.990958 0.060483
O -2.154229 2.287131 0.075476
H -1.137447 4.093792 0.006932
H 1.112950 2.523957 -0.178192
I 0.363924 -0.970832 -0.183431
S -1.925134 -1.599983 0.619022
C -2.793828 -0.111698 0.002629
C -3.971637 0.146403 0.935961
H -4.506070 1.037719 0.604657
H -4.659763 -0.698320 0.909350
H -3.633429 0.295562 1.959535
C -3.266204 -0.308490 -1.436552
H -3.979032 -1.131057 -1.484589
H -3.749292 0.601489 -1.797640
H -2.423957 -0.538817 -2.089855
O 2.207284 0.168921 -0.860256
C 3.130730 0.266383 0.041981
F 4.219648 0.909428 -0.400274
F 2.721448 0.942771 1.157745
F 3.558710 -0.936129 0.513025

Indole_NMe_IBCONAc_A_OCF3.log
Energy (E) = -1146.65128890 Hartree
Enthalpy (H) = -1146.409915 Hartree
Gibbs free energy (G) = -1146.483913 Hartree
Charge = 0, Spin = 1
C -1.838424 0.711010 -0.236429
C -2.059277 2.091650 0.008304
C -3.341158 2.645107 0.019870
C -4.400360 1.799495 -0.222322
C -4.198209 0.430419 -0.475380
C -2.938851 -0.123948 -0.485848
C -0.426853 0.585750 -0.156057
H -3.489955 3.699201 0.210965
H -5.407201 2.193855 -0.220899
H -5.054760 -0.200887 -0.665801
H -2.797555 -1.173200 -0.690881
N -0.860038 2.731722 0.209740
C 0.135383 1.802477 0.102540
C 1.590675 2.007054 0.212436
O 2.112603 3.070644 0.453197
I 0.964628 -0.918110 -0.344203
C -0.731478 4.152601 0.478260
H -1.266715 4.405640 1.393058
H 0.318734 4.394170 0.592165
H -1.151726 4.724815 -0.348456
N 2.243084 0.792109 -0.006294
C 3.640889 0.658353 0.000907
C 4.134627 -0.756140 -0.208120
H 3.819965 -1.142795 -1.179653
H 5.218811 -0.743961 -0.171505
H 3.761436 -1.423291 0.571423
O 4.396129 1.581215 0.155447
O -0.676125 -2.304481 -0.677749
C -1.056585 -2.984427 0.375615
F -2.136635 -3.723344 0.113007
F -0.101138 -3.823072 0.835599
F -1.371201 -2.190909 1.428993

NaphIBMeUreaMe_OCF3.log
Energy (E) = -1110.74699428 Hartree
Enthalpy (H) = -1110.480685 Hartree
Gibbs free energy (G) = -1110.552621 Hartree
Charge = 0, Spin = 1
C 2.516691 -1.162207 -0.270345
C 3.257451 0.007114 0.029785
C 2.577883 1.212686 0.313050
C 1.205216 1.292186 0.315758
C 0.507378 0.095574 0.018853
C 1.105075 -1.088342 -0.267099
H 3.164941 2.095236 0.528815
H 0.516767 -1.972710 -0.467008
I -1.575735 0.261265 0.062870

C -0.483094 3.024487 -0.132024
O -0.683141 4.222948 -0.173530
N -1.200425 2.109494 -0.854866
C -2.260907 2.640923 -1.696607
H -1.862855 3.461496 -2.288797
H -2.607749 1.855888 -2.367177
H -3.103412 3.028102 -1.118881
N 0.540211 2.483934 0.658768
C 1.268491 3.463527 1.452309
H 1.798658 2.944960 2.247858
H 1.977157 4.036920 0.850995
H 0.553991 4.161100 1.876092
C 4.671119 -0.065588 0.028418
C 5.305640 -1.243153 -0.255970
C 4.562950 -2.408511 -0.550533
C 3.196398 -2.368720 -0.556674
H 5.239456 0.827676 0.255561
H 6.386285 -1.287484 -0.254787
H 5.081142 -3.331738 -0.769677
H 2.613973 -3.253871 -0.777922
O -1.523162 -1.781659 0.996448
C -2.164549 -2.688405 0.332976
F -3.432336 -2.312614 -0.003162
F -1.578724 -2.996924 -0.864837
F -2.274151 -3.842279 0.998085

NpthIBCMe2O_C_OCF3.log
Energy (E) = -1001.31432095 Hartree
Enthalpy (H) = -1001.062075 Hartree
Gibbs free energy (G) = -1001.129002 Hartree
Charge = 0, Spin = 1
C 2.424250 1.227115 0.295074
C 1.075877 1.350633 0.101835
C 0.375707 0.196728 -0.283312
C 0.927925 -1.026896 -0.464863
H 3.012225 2.084794 0.600523
H 0.332450 -1.881337 -0.753249
O -0.889340 2.535758 -0.489159
I -1.661331 0.647306 -0.553091
C 2.984105 -2.393339 -0.451510
C 4.334442 -2.495238 -0.261898
C 5.087700 -1.362117 0.116659
C 4.475729 -0.152523 0.297783
C 3.080231 -0.014411 0.109579
C 2.326038 -1.154463 -0.270342
H 2.396770 -3.255670 -0.739910
H 4.831501 -3.445604 -0.399795
H 6.154964 -1.456303 0.263993
H 5.049310 0.718874 0.588444
C 0.255348 2.608737 0.338820
C 0.993111 3.871885 -0.068789
H 0.321611 4.720774 0.051544
H 1.868901 4.034112 0.559759
H 1.301900 3.809034 -1.110578
C -0.152079 2.670434 1.809993
H 0.727764 2.753886 2.448290
H -0.795343 3.536194 1.966690
H -0.695000 1.769685 2.101803
O -2.016561 -1.519811 -0.695075
C -2.379690 -2.109234 0.409906
F -1.470674 -1.966322 1.411653
F -3.538003 -1.623960 0.918309
F -2.553706 -3.423839 0.240768

NpthISO2NMe_D_OCF3.log
Energy (E) = -1451.29301798 Hartree
Enthalpy (H) = -1451.072420 Hartree
Gibbs free energy (G) = -1451.142287 Hartree
Charge = 0, Spin = 1
C -0.014308 3.442096 -0.493158
C 1.333014 3.554249 -0.325976
C 2.138139 2.417507 -0.089315
C 1.559304 1.112644 -0.011469
C 0.145722 1.073500 -0.156179
C -0.618893 2.175289 -0.399901
H 3.923262 3.601273 0.010531
H -0.630075 4.307998 -0.687399
H 1.820501 4.519285 -0.376980
C 3.534312 2.592528 0.065416
C 2.472477 0.033542 0.175890
H -1.687815 2.085533 -0.506622
C 3.819904 0.237851 0.302984
C 4.365988 1.532010 0.266172
H 4.462705 -0.625363 0.408479
H 5.431613 1.671240 0.375863
I -0.932797 -0.746860 0.048118
S 2.016020 -1.673049 0.255304
O 1.566252 -1.964816 1.594072
O 3.078789 -2.467157 -0.304212
N 0.772065 -1.662146 -0.817381
C 0.480162 -2.944519 -1.464499
H -0.226480 -2.761888 -2.271719
H 1.402206 -3.336439 -1.887468
H 0.065483 -3.689123 -0.779675
O -2.591060 0.508945 0.882969
C -3.709845 0.409260 0.235593
F -4.730076 0.988065 0.872742
F -3.663571 0.980001 -1.006157
F -4.078753 -0.881027 -0.001521

perF_CMe2O_OCF3.log
Energy (E) = -1244.58898731 Hartree
Enthalpy (H) = -1244.414666 Hartree
Gibbs free energy (G) = -1244.483117 Hartree
Charge = 0, Spin = 1
C 1.593944 2.261647 -0.349151
C 2.717044 1.619431 0.140702
C 2.699522 0.245487 0.335848
C 1.573480 -0.501612 0.046413
C 0.447619 0.177092 -0.391171
C 0.436801 1.539033 -0.616781
O 0.414232 -2.438148 -0.628377
I -1.170990 -1.158758 -0.583780
F -0.591206 2.196448 -1.110828
F 1.623569 3.563008 -0.572004
F 3.808424 2.314514 0.404072
F 3.799850 -0.320912 0.826178
C 1.464106 -2.019803 0.212720
C 2.694458 -2.768099 -0.279875
H 3.544651 -2.629887 0.382213
H 2.437919 -3.826097 -0.310577
H 2.957754 -2.446134 -1.285958
C 1.176677 -2.340835 1.676481
H 1.052251 -3.417653 1.786479
H 2.003732 -2.010425 2.305380
H 0.267552 -1.842558 2.016985
O -2.578719 0.482009 -0.528834
C -2.915434 0.904618 0.662535
F -1.845037 1.322241 1.392591
F -3.765491 1.926135 0.602456
F -3.498422 -0.053176 1.421669

perF_NMeCO2_OCF3.log
Energy (E) = -1334.62316490 Hartree
Enthalpy (H) = -1334.476872 Hartree
Gibbs free energy (G) = -1334.546384 Hartree
Charge = 0, Spin = 1
C -1.910871 -2.119722 -0.524520
C -2.864722 -1.372240 0.146724
C -2.619611 -0.052600 0.495737
C -1.388625 0.551706 0.240394
C -0.441415 -0.242783 -0.406694
C -0.690957 -1.535886 -0.822861
I 1.360489 0.686097 -0.801857
F 0.194151 -2.223678 -1.517593
F -2.173590 -3.359563 -0.894961
F -4.041916 -1.905150 0.410806
F -3.614591 0.628865 1.051405
N -1.122106 1.875027 0.594746
C -1.714948 2.457175 1.799076
H -1.854998 1.671746 2.536571
H -1.024089 3.200261 2.185497
H -2.665506 2.942822 1.591876
C -0.583881 2.795627 -0.323390
O -0.722037 3.978254 -0.157578
O 0.065589 2.250168 -1.344492
O 2.337349 -1.087451 -0.171539
C 2.589589 -1.176576 1.121072
F 1.463537 -1.133665 1.870117
F 3.211659 -2.313327 1.402644
F 3.360625 -0.166715 1.572791

PyIBCMe2O_D_OCF3.log
Energy (E) = -863.843978981 Hartree
Enthalpy (H) = -863.653174 Hartree
Gibbs free energy (G) = -863.713757 Hartree
Charge = 0, Spin = 1
C -0.937395 3.114709 -0.432972
C -2.238904 2.883038 -0.006617
C -1.930173 0.625650 0.064437
C -0.624620 0.795835 -0.353528
H -0.595243 4.121907 -0.619701
H -2.917998 3.713075 0.145005
O -1.584062 -1.680443 -0.351604
I 0.352304 -1.050062 -0.488882
C -0.081752 2.033470 -0.604346
H 0.946005 2.147427 -0.914129
N -2.730172 1.670024 0.246235
C -2.396503 -0.783600 0.382712
C -2.243681 -1.012707 1.883613
H -2.862939 -0.307007 2.437004
H -2.550117 -2.030286 2.123456
H -1.205541 -0.874787 2.193506
C -3.827948 -1.005690 -0.065970
H -4.123444 -2.024081 0.181978
H -4.487019 -0.299449 0.435868
H -3.908006 -0.864882 -1.142477
O 2.208555 0.107175 -0.749309
C 2.898927 0.324300 0.335766
F 3.304314 -0.812669 0.948175
F 3.996065 1.047106 0.094065
F 2.192929 1.000819 1.283909

PyrroleNMeIBCMe2O_A_OCF3.log
Energy (E) = -865.030409204 Hartree
Enthalpy (H) = -864.816106 Hartree
Gibbs free energy (G) = -864.879072 Hartree
Charge = 0, Spin = 1
C -1.474473 2.695013 -0.207057
C -0.236047 2.107166 -0.273075
C -0.496654 0.738231 -0.092330
C -1.830880 0.511437 0.056093
H -1.751841 3.734215 -0.261582
H 0.715239 2.585363 -0.415879

I 0.566798 -1.018350 -0.146385
O -1.272351 -1.709035 0.496232
N -2.438640 1.730791 -0.016796
C -3.852768 2.006883 0.130566
H -4.440194 1.363758 -0.523100
H -4.179721 1.860358 1.159166
H -4.031774 3.040927 -0.152224
C -2.372118 -0.894392 0.130423
C -3.421536 -1.095689 1.214887
H -4.358492 -0.598029 0.963387
H -3.614461 -2.163781 1.306691
H -3.054335 -0.723294 2.169871
C -2.902244 -1.325875 -1.235277
H -3.246557 -2.358344 -1.178792
H -3.730592 -0.693386 -1.560154
H -2.107196 -1.257553 -1.979647
O 2.261912 0.178206 -0.830974
C 3.128398 0.521143 0.081337
F 4.135114 1.233496 -0.433183
F 2.587082 1.276950 1.072463
F 3.684867 -0.540455 0.715473

AnthI8BA_B_NTf2.log
Energy (E) = -2681.88731017 Hartree
Enthalpy (H) = -2681.522995 Hartree
Gibbs free energy (G) = -2681.623258 Hartree
Charge = 0, Spin = 1
C 7.585881 -0.889645 0.596042
C 6.784279 0.147198 0.958960
C 5.436103 0.220468 0.502808
C 4.942589 -0.820146 -0.348859
C 5.813424 -1.891409 -0.703553
C 7.093545 -1.925579 -0.247187
C 4.592971 1.269228 0.868036
C 3.628547 -0.760606 -0.805689
C 2.796968 0.293952 -0.441572
C 3.278444 1.327092 0.417379
C 2.396433 2.385307 0.776888
H 2.785460 3.176691 1.407685
C 1.096177 2.463059 0.366980
C 0.665905 1.390535 -0.468670
H 4.969644 2.050806 1.518092
H 8.607167 -0.935055 0.949057
H 7.153917 0.935822 1.602425
H 5.429064 -2.675843 -1.343145
H 7.747290 -2.742240 -0.521010
H 3.240903 -1.551484 -1.437572
I -1.363123 1.191499 -1.027580
C 1.449537 0.366252 -0.884123
H 1.077656 -0.405221 -1.545224
C 0.279603 3.676008 0.741820
H 0.219762 4.336784 -0.127549
H 0.872846 4.221648 1.473557
C -1.127666 3.422926 1.320438
H -1.224696 2.375012 1.598462
H -1.215688 3.974381 2.254140
C -2.304326 3.863628 0.417950
H -3.108244 3.124780 0.462075
H -2.720937 4.806036 0.761475
C -1.929336 4.118765 -1.025460
O -1.402096 3.100968 -1.751590
O -2.038909 5.187344 -1.556552
N -1.274374 -1.042130 -0.005672
S -0.846999 -2.274798 -0.980444
S -1.154472 -1.092414 1.617989
O -0.864052 -1.726296 -2.310537
O 0.285915 -3.009893 -0.511756
C -2.297363 -3.418562 -0.933071
O -0.491319 0.100864 2.064404
O -0.808439 -2.381512 2.124469
C -2.921848 -0.812945 2.073117
F -3.396586 -2.737929 -1.220042
F -2.418820 -3.977963 0.252667
F -2.104941 -4.350672 -1.848174
F -3.023524 -0.810292 3.387787
F -3.325774 0.368649 1.603763
F -3.691738 -1.758037 1.567036

FuranIBCMe2S_A_NTf2.log
Energy (E) = -2582.38309008 Hartree
Enthalpy (H) = -2582.163375 Hartree
Gibbs free energy (G) = -2582.248954 Hartree
Charge = 0, Spin = 1
C 2.349659 -1.661521 2.552572
C 1.282531 -1.508855 1.732990
C 1.822217 -0.825106 0.610858
C 3.132313 -0.593342 0.825335
O 3.474961 -1.107031 2.017261
H 2.465165 -2.139064 3.507917
H 0.277026 -1.850186 1.907817
I 1.093574 0.010417 -1.138615
S 3.478389 0.038959 -1.720288
C 4.058731 0.241964 0.007198
C 3.975612 1.697953 0.458086
H 4.317187 1.777124 1.491345
H 4.605130 2.324429 -0.172695
H 2.948125 2.062209 0.413676
C 5.490778 -0.277661 0.040071
H 6.126543 0.330131 -0.603392
H 5.867908 -0.206385 1.060601
H 5.542033 -1.315987 -0.282171
N -1.173343 -0.094771 0.090753
S -1.885159 -1.542842 0.133351
S -1.376738 1.083126 1.180700
O -1.178089 -2.345087 -0.827909
O -2.118524 -2.036347 1.458445
C -3.551630 -1.254991 -0.603411
O -0.077454 1.575603 1.552740
O -2.382364 0.817104 2.161163
C -2.052063 2.400672 0.081585
F -3.420293 -0.645085 -1.772551
F -4.288080 -0.513818 0.201188
F -4.134554 -2.426972 -0.784555
F -2.224025 3.502713 0.788404
F -1.189721 2.645901 -0.903132
F -3.205118 2.027541 -0.444608

Indole_NMe_IBCONAc_A_NTf2.log
Energy (E) = -2560.47234425 Hartree
Enthalpy (H) = -2560.182158 Hartree
Gibbs free energy (G) = -2560.281342 Hartree
Charge = 0, Spin = 1
C -1.740554 1.924611 0.178394
C -3.099444 2.337531 0.152678
C -3.471739 3.676948 0.282673
C -2.466924 4.603443 0.441263
C -1.116182 4.213045 0.476155
C -0.741871 2.895828 0.351626
C -1.826509 0.516978 0.019248
H -4.513076 3.967782 0.259940
H -2.718069 5.650000 0.544011
H -0.351488 4.965304 0.606242
H 0.303686 2.639922 0.388278
N -3.922365 1.249514 -0.004334
C -3.136311 0.137202 -0.082951
C -3.562527 -1.261763 -0.224258
O -4.705980 -1.631748 -0.328181
I -0.562847 -1.110994 -0.112982
C -5.371636 1.327568 -0.056136
H -5.676678 1.952203 -0.894974
H -5.774444 0.329543 -0.180841
H -5.750760 1.762490 0.868043
N -2.434240 -2.097314 -0.214783
C -2.495652 -3.505217 -0.271689
C -1.150484 -4.192612 -0.298481
H -0.576876 -3.973908 0.604839
H -1.321221 -5.262526 -0.352208
H -0.567968 -3.884485 -1.169184
O -3.532430 -4.109155 -0.293233
N 1.402972 0.271465 0.005260
S 1.866991 1.024695 -1.371205
S 1.959230 0.602745 1.502545
O 0.889358 0.642568 -2.349282
O 2.241849 2.388364 -1.175965
C 3.410167 0.134579 -1.854285
O 0.829137 0.587207 2.385277
O 2.935023 1.644824 1.523371
C 2.855995 -0.963684 1.897391
F 3.212031 -1.175031 -1.765594
F 4.405333 0.487611 -1.066540
F 3.696008 0.449972 -3.102934
F 3.236206 -0.923542 3.158128
F 2.037642 -2.001854 1.720026
F 3.908340 -1.116215 1.117338

NaphIBMeUreaMe_NTf2.log
Energy (E) = -2524.57579366 Hartree
Enthalpy (H) = -2524.260544 Hartree
Gibbs free energy (G) = -2524.357217 Hartree
Charge = 0, Spin = 1
C -2.248441 2.490387 -0.318898
C -3.399362 2.133919 0.426458
C -3.656849 0.773725 0.714285
C -2.802812 -0.224702 0.315935
C -1.658360 0.185360 -0.404658
C -1.373558 1.465322 -0.744895
H -4.554878 0.523193 1.263240
H -0.495823 1.721415 -1.321349
I -0.375945 -1.394582 -0.879026
C -2.970030 -2.599730 -0.300581
O -3.619748 -3.618658 -0.194951
N -2.132215 -2.364241 -1.379775
C -2.029731 -3.434669 -2.363205
H -3.035727 -3.726060 -2.653568
H -1.501008 -3.059801 -3.237357
H -1.525869 -4.320302 -1.971268
N -3.035509 -1.575774 0.639601
C -3.860747 -1.864182 1.805407
H -3.568160 -1.193384 2.609256
H -4.924940 -1.747964 1.592241
H -3.689616 -2.893589 2.101778
C -4.273028 3.160993 0.854459
C -4.002184 4.468907 0.559169
C -2.846241 4.819356 -0.173458
C -1.985064 3.849228 -0.605248
H -5.155799 2.893531 1.421397
H -4.674853 5.246852 0.893795
H -2.643284 5.859640 -0.386201
H -1.086159 4.099647 -1.154048
N 1.480785 0.096657 0.000819
S 2.108301 1.157348 -1.045725
S 1.481127 0.281298 1.613950
O 1.643348 0.730016 -2.340279
O 1.967136 2.525781 -0.648918
C 3.919221 0.798561 -1.052152
O 0.161247 -0.013393 2.099073

O 2.213726 1.420290 2.070115
C 2.473315 -1.198667 2.091043
F 4.112820 -0.496092 -1.256293
F 4.468971 1.154144 0.091216
F 4.467423 1.484107 -2.040130
F 2.519746 -1.273898 3.408349
F 1.898836 -2.299262 1.611738
F 3.700698 -1.113479 1.608685

NpthIBCMe2O_C_NTf2.log
Energy (E) = -2415.13978020 Hartree
Enthalpy (H) = -2414.838618 Hartree
Gibbs free energy (G) = -2414.929881 Hartree
Charge = 0, Spin = 1
C -4.124435 -0.158683 0.069932
C -2.991754 -0.867666 -0.219612
C -1.784562 -0.150099 -0.278755
C -1.655993 1.177280 -0.050386
H -5.080730 -0.664859 0.131107
H -0.712837 1.702627 -0.090175
O -1.815498 -2.632465 -1.278135
I -0.232319 -1.489799 -0.786134
C -2.779511 3.305163 0.481249
C -3.923988 3.998614 0.762749
C -5.166951 3.330499 0.820274
C -5.242654 1.983359 0.595711
C -4.077374 1.237373 0.302672
C -2.831440 1.911738 0.246699
H -1.820015 3.804170 0.433982
H -3.881354 5.063682 0.943783
H -6.063373 3.892332 1.044562
H -6.193941 1.467895 0.639140
C -2.914503 -2.371002 -0.407397
C -4.128272 -2.946168 -1.112749
H -3.962874 -4.008171 -1.287193
H -5.019404 -2.834803 -0.495450
H -4.284193 -2.447660 -2.067295
C -2.696633 -3.039913 0.946956
H -3.564556 -2.880879 1.586513
H -2.548092 -4.109725 0.801424
H -1.826897 -2.623487 1.458769
N 1.396067 0.165524 -0.025315
S 1.763266 1.371108 -1.058907
S 1.607649 0.244112 1.590196
O 1.174666 0.989252 -2.311062
O 1.536576 2.672970 -0.508488
C 3.585772 1.220901 -1.317343
O 0.409728 -0.239414 2.215632
O 2.258691 1.439373 2.023691
C 2.826654 -1.122132 1.820704
F 3.882663 -0.029420 -1.637570
F 4.242377 1.569674 -0.230095
F 3.919556 2.020767 -2.313802
F 3.057457 -1.266718 3.111679
F 2.328150 -2.255880 1.335620
F 3.956980 -0.849235 1.194741

NpthISO2NMe_D_NTf2.log
Energy (E) = -2865.12117960 Hartree
Enthalpy (H) = -2864.851662 Hartree
Gibbs free energy (G) = -2864.945363 Hartree
Charge = 0, Spin = 1
C 1.123554 2.413076 -2.469062
C 2.287239 2.933002 -1.986267
C 3.072825 2.233658 -1.039634
C 2.649657 0.964544 -0.544397
C 1.391294 0.530251 -1.026042
C 0.658250 1.180925 -1.969613
H 4.562739 3.774673 -0.969102
H 0.534091 2.939985 -3.204232
H 2.643816 3.896483 -2.326480
C 4.286161 2.800602 -0.586651
C 3.544327 0.285934 0.330691
H -0.277787 0.773308 -2.323530
C 4.723205 0.858448 0.732141
C 5.091460 2.141478 0.295859
H 5.389299 0.289001 1.366375
H 6.019501 2.577810 0.635071
I 0.495523 -1.150155 -0.116293
S 3.357162 -1.364097 0.953723
O 2.680651 -1.335626 2.223492
O 4.625179 -2.036239 0.859834
N 2.390619 -2.017279 -0.234072
C 2.383002 -3.485769 -0.265554
H 1.828890 -3.801173 -1.146591
H 3.413236 -3.821412 -0.357216
H 1.943813 -3.931969 0.629685
N -1.649138 0.174589 -0.146084
S -1.579955 1.651935 0.526597
S -2.738127 -0.276011 -1.256848
O -0.335074 1.692816 1.242508
O -1.944433 2.698809 -0.378305
C -2.876039 1.608485 1.842222
O -2.026873 -0.887252 -2.349063
O -3.782802 0.671143 -1.485957
C -3.499063 -1.698031 -0.363963
F -2.680661 0.551998 2.615998
F -4.081464 1.549485 1.311678
F -2.762323 2.709548 2.563778
F -4.367927 -2.293913 -1.157102
F -2.545680 -2.569837 -0.029870
F -4.104657 -1.287031 0.734030

perF_CMe2O_NTf2.log
Energy (E) = -2658.41271108 Hartree
Enthalpy (H) = -2658.189403 Hartree
Gibbs free energy (G) = -2658.282253 Hartree
Charge = 0, Spin = 1
C 2.804453 2.070994 -1.055129
C 4.012118 1.643319 -0.531436
C 4.115253 0.383248 0.043131
C 3.026412 -0.465621 0.101552
C 1.822997 0.009469 -0.393822
C 1.686870 1.248086 -0.981984
O 1.965388 -2.569233 0.087347
I 0.317599 -1.417424 -0.052470
F 0.559046 1.684641 -1.504761
F 2.715454 3.254358 -1.628303
F 5.068625 2.430496 -0.593480
F 5.293635 0.030367 0.548222
C 3.028729 -1.864773 0.713862
C 4.268641 -2.681250 0.387097
H 5.138730 -2.314479 0.924186
H 4.073594 -3.708665 0.690828
H 4.466021 -2.665396 -0.683071
C 2.819890 -1.746511 2.219336
H 2.737821 -2.743845 2.650141
H 3.668706 -1.232253 2.670104
H 1.918541 -1.176695 2.452787
N -1.430586 0.218501 -0.137164
S -1.346464 1.428594 0.955754
S -2.381147 0.227517 -1.466852
O -0.158099 1.151985 1.719120
O -1.593771 2.722521 0.411342
C -2.746303 1.068700 2.102873
O -1.590581 -0.224887 -2.572625
O -3.236998 1.366724 -1.539998
C -3.464896 -1.214439 -1.073960
F -2.708970 -0.208526 2.459165
F -3.895283 1.332669 1.515330
F -2.608343 1.826376 3.174924
F -4.234955 -1.466133 -2.112636
F -2.703264 -2.283510 -0.825910
F -4.209589 -0.972591 -0.012214

perF_NMeCO2_NTf2.log
Energy (E) = -2748.44306903 Hartree
Enthalpy (H) = -2748.248000 Hartree
Gibbs free energy (G) = -2748.341026 Hartree
Charge = 0, Spin = 1
C 2.472296 2.112547 -1.367478
C 3.624606 1.925315 -0.622544
C 3.810260 0.769908 0.123534
C 2.825852 -0.212266 0.200308
C 1.671864 0.018007 -0.548341
C 1.493997 1.131172 -1.345839
I 0.265892 -1.489878 -0.390210
F 0.414090 1.288405 -2.085357
F 2.321892 3.193948 -2.105054
F 4.579888 2.832065 -0.660498
F 4.974887 0.617902 0.740740
N 2.988686 -1.361740 0.978400
C 3.715894 -1.299846 2.247781
H 3.587542 -0.308705 2.673385
H 3.285459 -2.040123 2.915210
H 4.773601 -1.516724 2.121270
C 2.750352 -2.644364 0.476022
O 3.236518 -3.619186 0.979134
O 1.942011 -2.690125 -0.592686
N -1.368227 0.118621 -0.076749
S -1.037097 1.289623 1.033970
S -2.496874 0.262071 -1.269382
O 0.060026 0.768735 1.802064
O -0.987094 2.591465 0.452212
C -2.463601 1.263705 2.214675
O -1.944024 -0.280327 -2.471993
O -3.178514 1.512976 -1.214095
C -3.675120 -1.046342 -0.714736
F -2.644773 0.026874 2.645570
F -3.563171 1.712429 1.652306
F -2.129784 2.040902 3.227562
F -4.585238 -1.222252 -1.648758
F -2.993583 -2.180459 -0.543123
F -4.255139 -0.717760 0.422049

PyIBCMe2O_D_NTf2.log
Energy (E) = -2277.66951853 Hartree
Enthalpy (H) = -2277.429819 Hartree
Gibbs free energy (G) = -2277.514673 Hartree
Charge = 0, Spin = 1
C 2.436343 -1.515074 2.678951
C 3.764930 -1.569328 2.281236
C 3.373715 -0.306743 0.424059
C 2.034945 -0.230666 0.764284
H 2.114074 -2.004742 3.585625
H 4.490521 -2.111395 2.874583
O 2.911958 1.418262 -1.132780
I 1.017953 0.877999 -0.700243
C 1.521752 -0.827001 1.890338
H 0.480148 -0.767435 2.166935
N 4.229645 -0.982325 1.178032
C 3.817568 0.348085 -0.869640
C 5.189875 0.975380 -0.738638
H 5.913705 0.208870 -0.467796
H 5.470745 1.426127 -1.689077

H 5.179713 1.742817 0.032924
C 3.775708 -0.693871 -1.981527
H 4.037412 -0.222769 -2.928234
H 4.487257 -1.490158 -1.765274
H 2.783137 -1.142114 -2.066623
N -1.059512 -0.067941 0.160741
S -1.248942 -1.673502 -0.053255
S -1.851269 0.803004 1.289032
O -0.181812 -2.071459 -0.928485
O -1.500507 -2.373220 1.168074
C -2.781646 -1.812660 -1.074446
O -0.878454 1.591784 1.990856
O -2.864454 0.067631 1.976610
C -2.727277 1.998862 0.191012
F -2.677282 -1.018435 -2.129423
F -3.842506 -1.476946 -0.369849
F -2.891113 -3.065690 -1.476116
F -3.360542 2.879798 0.939391
F -1.841735 2.630619 -0.576655
F -3.589611 1.366299 -0.583125

PyrroleNMeIBCMe2O_A_NTf2.log
Energy (E) = -2278.85720384 Hartree
Enthalpy (H) = -2278.593879 Hartree
Gibbs free energy (G) = -2278.681044 Hartree
Charge = 0, Spin = 1
C 2.790079 -1.242628 2.347277
C 1.572477 -0.793451 1.900454
C 1.879725 -0.119001 0.707361
C 3.214885 -0.176038 0.440825
H 3.036955 -1.783192 3.245137
H 0.617573 -0.925033 2.377465
I 0.890543 0.793519 -0.847753
O 2.803345 1.314628 -1.307703
N 3.779386 -0.877716 1.463634
C 5.185945 -1.179340 1.637920
H 5.593276 -1.643239 0.740625
H 5.757052 -0.280657 1.866124
H 5.285660 -1.879549 2.462804
C 3.761009 0.352965 -0.859916
C 5.063040 1.127058 -0.730554
H 5.894667 0.460257 -0.503856
H 5.267527 1.616807 -1.681560
H 4.979466 1.885862 0.045409
C 3.877428 -0.776732 -1.877542
H 4.210392 -0.374740 -2.834063
H 4.592317 -1.529521 -1.541251
H 2.910333 -1.265602 -2.008954
N -1.133159 -0.070991 0.145681
S -1.343131 -1.686863 0.034809
S -1.835997 0.861076 1.286264
O -0.346879 -2.143788 -0.892087
O -1.524771 -2.320574 1.302737
C -2.942512 -1.859905 -0.871995
O -0.822472 1.689289 1.872093
O -2.795086 0.162045 2.081363
C -2.796991 1.997820 0.196183
F -2.906444 -1.123400 -1.973190
F -3.953509 -1.479086 -0.118079
F -3.088327 -3.131573 -1.200509
F -3.381007 2.912934 0.945487
F -1.975643 2.595384 -0.664116
F -3.711197 1.327778 -0.481885

AnthI8BA_B_OTf.log
Energy (E) = -1816.46851898 Hartree
Enthalpy (H) = -1816.138701 Hartree
Gibbs free energy (G) = -1816.224795 Hartree
Charge = 0, Spin = 1
C -7.277909 0.064687 0.094543
C -6.388243 -0.888448 0.480228
C -4.992175 -0.720885 0.245051
C -4.545120 0.473443 -0.408174
C -5.509908 1.449281 -0.795311
C -6.832788 1.252281 -0.552766
C -4.060845 -1.681747 0.635138
C -3.185137 0.651551 -0.646715
C -2.262674 -0.316378 -0.258897
C -2.700980 -1.504401 0.397794
C -1.726294 -2.464637 0.786474
H -2.070998 -3.366336 1.280215
C -0.384451 -2.318318 0.568881
C -0.004376 -1.106950 -0.084779
H -4.403783 -2.582302 1.132028
H -8.334700 -0.073547 0.278426
H -6.722509 -1.792691 0.973489
H -5.161644 2.349187 -1.286312
H -7.557548 1.997676 -0.850129
H -2.834195 1.555613 -1.131183
I 2.031766 -0.628707 -0.458361
C -0.871966 -0.148488 -0.491830
H -0.542725 0.761041 -0.977302
C 0.539101 -3.436962 0.985341
H 0.803285 -4.024572 0.102812
H -0.056961 -4.104659 1.605049
C 1.809536 -3.027925 1.757467
H 1.726559 -1.987234 2.070905
H 1.850850 -3.600083 2.681966
C 3.138692 -3.267896 1.006959
H 3.832493 -2.444010 1.186965
H 3.623387 -4.172829 1.361819
C 2.976634 -3.475006 -0.483290
O 2.369172 -2.505302 -1.202574
O 3.317869 -4.477274 -1.046079
O 1.454949 1.389391 0.459829
S 1.594289 2.602237 -0.435364
O 2.794413 2.528120 -1.225202
O 0.355440 2.947238 -1.078686
C 1.880817 3.890421 0.845083
F 0.844134 3.958776 1.664353
F 2.968827 3.600746 1.541935
F 2.043708 5.059513 0.247570

FuranIBCMe2S_A_OTf.log
Energy (E) = -1716.96651486 Hartree
Enthalpy (H) = -1716.781343 Hartree
Gibbs free energy (G) = -1716.852028 Hartree
Charge = 0, Spin = 1
C -2.091009 2.990735 -0.586597
C -0.969980 2.275274 -0.844191
C -1.328698 0.947400 -0.487107
C -2.596888 0.944776 -0.031338
O -3.082484 2.197236 -0.088585
H -2.336358 4.029124 -0.712980
H -0.028657 2.619728 -1.230261
I -0.338344 -0.861886 -0.394550
S -2.643303 -1.703611 -0.132785
C -3.343957 -0.178985 0.603811
C -3.140727 -0.154910 2.116594
H -3.531647 0.778036 2.526255
H -3.664811 -0.991180 2.577814
H -2.081411 -0.225753 2.365516
C -4.824746 -0.161873 0.241838
H -5.327696 -1.025645 0.676221
H -5.279294 0.741347 0.650350
H -4.966189 -0.171831 -0.837130
O 1.525024 0.646513 -0.781983
S 2.827960 -0.086873 -0.674147
O 2.593293 -1.515770 -0.618986
O 3.853655 0.427743 -1.527143
C 3.327426 0.354681 1.037019
F 3.503089 1.663112 1.151096
F 2.368189 -0.018675 1.887733
F 4.449762 -0.263826 1.365578

Indole_NMe_IBCONAc_A_OTf.log
Energy (E) = -1695.06201992 Hartree
Enthalpy (H) = -1694.806277 Hartree
Gibbs free energy (G) = -1694.889819 Hartree
Charge = 0, Spin = 1
C 1.286672 1.948642 0.169329
C 2.654390 2.259724 -0.049074
C 3.115425 3.577659 -0.078039
C 2.189040 4.577737 0.114440
C 0.829607 4.285168 0.332110
C 0.365944 2.990315 0.362502
C 1.258867 0.531207 0.121076
H 4.160861 3.798318 -0.245437
H 2.510680 5.609939 0.098531
H 0.134054 5.099325 0.479315
H -0.676700 2.771359 0.530230
N 3.379560 1.104346 -0.213725
C 2.516993 0.050573 -0.108001
C 2.812845 -1.385117 -0.213664
O 3.905952 -1.855745 -0.411626
I -0.123604 -0.984961 0.299724
C 4.810701 1.066717 -0.456258
H 5.338568 1.536088 0.373275
H 5.124811 0.033652 -0.548835
H 5.044024 1.603433 -1.375257
N 1.625994 -2.118867 -0.047510
C 1.555518 -3.528518 -0.097671
C 0.172972 -4.094765 0.122019
H -0.526560 -3.744077 -0.639621
H 0.238980 -5.175898 0.063378
H -0.217799 -3.819489 1.103798
O 2.517402 -4.217258 -0.298839
O -1.670676 0.700649 0.647276
S -3.084656 0.168549 0.697674
O -3.049772 -1.277624 0.749542
O -3.929059 0.902081 1.583404
C -3.648570 0.567125 -1.004261
F -3.615408 1.874838 -1.204670
F -2.836620 -0.021687 -1.881734
F -4.878805 0.124576 -1.191821

NaphIBMeUreaMe_OTf.log
Energy (E) = -1659.16066593 Hartree
Enthalpy (H) = -1658.879763 Hartree
Gibbs free energy (G) = -1658.960827 Hartree
Charge = 0, Spin = 1
C -1.448902 2.532482 -0.384149
C -2.763763 2.367999 0.116723
C -3.207875 1.085523 0.511745
C -2.394084 -0.019037 0.437142
C -1.087808 0.199647 -0.062625
C -0.606056 1.400518 -0.469129
H -4.220134 0.980895 0.878845
H 0.408643 1.514490 -0.830857
I 0.092236 -1.527251 -0.149147
C -2.696970 -2.441860 0.124218
O -3.471781 -3.369896 0.230735
N -1.653980 -2.440364 -0.777170
C -1.500382 -3.633794 -1.598835
H -2.466870 -3.878279 -2.031732

H -0.794322 -3.422418 -2.399768
H -1.162597 -4.498712 -1.024792
N -2.823346 -1.283089 0.891250
C -3.902983 -1.322488 1.869111
H -3.725351 -0.551882 2.615313
H -4.880432 -1.170217 1.407513
H -3.906057 -2.300253 2.339050
C -3.604515 3.503190 0.200016
C -3.150618 4.731965 -0.193742
C -1.836362 4.891969 -0.687129
C -1.000745 3.813798 -0.780107
H -4.611092 3.381358 0.579987
H -3.801139 5.593363 -0.126234
H -1.494655 5.872094 -0.989363
H 0.010683 3.917145 -1.151510
O 1.809627 -0.071365 0.582054
S 2.912235 0.176362 -0.409337
O 3.280391 -1.029134 -1.107001
O 2.699114 1.376943 -1.176524
C 4.300277 0.555854 0.733501
F 4.013469 1.621822 1.465321
F 4.522921 -0.473986 1.537348
F 5.393193 0.796840 0.025624

NpthIBCMe2O_C_OTf.log
Energy (E) = -1549.72478647 Hartree
Enthalpy (H) = -1549.458032 Hartree
Gibbs free energy (G) = -1549.534754
Hartree
Charge = 0, Spin = 1
C -3.522490 -0.061813 -0.018283
C -2.396257 -0.837351 -0.015251
C -1.159771 -0.169945 0.019699
C -0.997639 1.173624 0.069239
H -4.501903 -0.525386 -0.040965
H -0.028715 1.651329 0.114525
O -1.147546 -2.783828 -0.559807
I 0.390101 -1.595880 -0.024831
C -2.081105 3.386722 0.091158
C -3.218191 4.146375 0.078232
C -4.487657 3.529056 0.033344
C -4.596655 2.165870 0.000938
C -3.439906 1.351969 0.012820
C -2.167150 1.975497 0.060985
H -1.100996 3.845184 0.122128
H -3.149600 5.225171 0.101664
H -5.378068 4.142799 0.023672
H -5.568447 1.689485 -0.035294
C -2.371778 -2.355031 0.025691
C -3.465359 -2.983559 -0.818785
H -3.334661 -4.064599 -0.813527
H -4.449084 -2.754367 -0.409372
H -3.406636 -2.622544 -1.843555
C -2.460114 -2.829173 1.473399
H -3.408366 -2.523681 1.915353
H -2.382427 -3.915773 1.501767
H -1.656044 -2.401284 2.074614
O 1.788988 0.091720 0.652925
S 2.541669 0.950325 -0.339454
O 2.475668 0.402160 -1.667349
O 2.277827 2.347402 -0.138013
C 4.262294 0.672065 0.240800
F 4.403957 1.091456 1.487646
F 4.545960 -0.624529 0.186470
F 5.102753 1.331366 -0.539632

NpthISO2NMe_D_OTf.log
Energy (E) = -1999.70678956 Hartree
Enthalpy (H) = -1999.471628 Hartree
Gibbs free energy (G) = -1999.549329
Hartree
Charge = 0, Spin = 1
C 0.377774 3.418569 -0.304243
C 1.718900 3.613756 -0.160823
C 2.604984 2.525079 0.001997
C 2.114544 1.183698 0.032916
C 0.705293 1.054067 -0.083515
C -0.139014 2.110852 -0.262369
H 4.314501 3.815045 0.116568
H -0.298609 4.249281 -0.441592
H 2.138432 4.611330 -0.175609
C 3.990938 2.782175 0.130879
C 3.097661 0.156926 0.143546
H -1.203253 1.956081 -0.350312
C 4.433085 0.441052 0.245024
C 4.893292 1.768742 0.258394
H 5.134015 -0.381100 0.291583
H 5.950484 1.971194 0.348438
I -0.229163 -0.844843 0.042582
S 2.755331 -1.576975 0.156356
O 2.359308 -1.965948 1.484556
O 3.837246 -2.273758 -0.485426
N 1.471859 -1.607840 -0.892881
C 1.245335 -2.888663 -1.576040
H 0.489859 -2.731600 -2.342673
H 2.176755 -3.183524 -2.052663
H 0.924802 -3.685158 -0.901194
O -2.011100 0.374820 1.055266
S -3.343513 -0.289355 0.847600
O -3.161064 -1.653471 0.404630
O -4.295431 0.011026 1.868695
C -3.913832 0.610980 -0.649818
F -3.998437 1.913546 -0.402634
F -3.022601 0.438154 -1.634474
F -5.087724 0.166806 -1.056225

perF_CMe2O_OTf.log
Energy (E) = -1792.99788709 Hartree
Enthalpy (H) = -1792.809100 Hartree
Gibbs free energy (G) = -1792.885565
Hartree
Charge = 0, Spin = 1
C 1.878579 2.477451 0.089379
C 3.178237 2.007603 0.159890
C 3.429895 0.643027 0.092282
C 2.396204 -0.264212 -0.033683
C 1.101569 0.233691 -0.034434
C 0.816788 1.583764 -0.010060
O 1.388815 -2.252850 -0.809509
I -0.259724 -1.361997 -0.070108
F -0.395392 2.081906 -0.132552
F 1.646452 3.776107 0.100361
F 4.179069 2.861096 0.262557
F 4.696588 0.244058 0.164361
C 2.564358 -1.778069 -0.170105
C 3.693733 -2.175749 -1.108075
H 4.666796 -1.985086 -0.664157
H 3.596789 -3.242907 -1.302055
H 3.611091 -1.641325 -2.052651
C 2.749164 -2.402579 1.207563
H 2.821054 -3.484505 1.103114
H 3.663849 -2.026968 1.666296
H 1.914001 -2.165097 1.867719
O -1.691615 0.059550 0.990523
S -3.151153 -0.252731 0.739608
O -3.264506 -1.576010 0.169026
O -3.993104 0.146785 1.819481
C -3.501813 0.899243 -0.648428
F -3.376984 2.153453 -0.251148
F -2.629709 0.671773 -1.632723
F -4.725221 0.701675 -1.106237

perF_NMeCO2_OTf.log
Energy (E) = -1883.03051683 Hartree
Enthalpy (H) = -1882.869882 Hartree
Gibbs free energy (G) = -1882.946863
Hartree
Charge = 0, Spin = 1
C 1.637765 2.642718 -0.195662
C 2.936628 2.296408 0.140844
C 3.299339 0.967800 0.308257
C 2.361031 -0.058100 0.207078
C 1.055562 0.329097 -0.098926
C 0.693072 1.640215 -0.341999
I -0.275828 -1.233188 -0.316478
F -0.523899 1.961951 -0.734337
F 1.316872 3.905596 -0.400649
F 3.852871 3.237098 0.251552
F 4.580289 0.704548 0.533935
N 2.708196 -1.401690 0.381233
C 3.750001 -1.783880 1.336882
H 3.761411 -1.063262 2.149645
H 3.503444 -2.767406 1.725148
H 4.730996 -1.830130 0.870696
C 2.353193 -2.389203 -0.546309
O 2.952863 -3.425445 -0.618405
O 1.311175 -2.081853 -1.329254
O -1.573278 0.056304 0.944757
S -3.041299 -0.357069 0.938998
O -3.142302 -1.673712 0.353611
O -3.695134 -0.039068 2.163634
C -3.696715 0.797337 -0.331978
F -3.504561 2.048178 0.045307
F -3.051555 0.585048 -1.476993
F -4.985508 0.579865 -0.513107

PyIBCMe2O_D_OTf.log
Energy (E) = -1412.25404065 Hartree
Enthalpy (H) = -1412.048728 Hartree
Gibbs free energy (G) = -1412.118519
Hartree
Charge = 0, Spin = 1
C 1.945455 3.162501 0.224490
C 3.284611 2.812795 0.112520
C 2.801420 0.592564 -0.065168
C 1.452211 0.879113 0.045561
H 1.659084 4.198055 0.333709
H 4.051952 3.576446 0.138461
O 2.110498 -1.584867 -0.704660
I 0.344944 -0.894811 -0.015017
C 0.980628 2.162169 0.202980
H -0.072783 2.370759 0.307835
N 3.711144 1.556894 -0.023356
C 3.213416 -0.863980 -0.165650
C 4.363094 -1.051018 -1.135067
H 5.221411 -0.473173 -0.796848
H 4.625555 -2.106721 -1.179587
H 4.075165 -0.712374 -2.128522
C 3.567860 -1.372489 1.226554
H 3.842136 -2.424876 1.166643
H 4.404673 -0.802000 1.628477
H 2.722052 -1.267048 1.909016
O -1.360912 0.499472 0.741294
S -2.708257 -0.179437 0.800939
O -2.543824 -1.602412 0.609557
O -3.538392 0.316751 1.851250
C -3.448447 0.427052 -0.766685
F -3.558174 1.747621 -0.744085
F -2.660947 0.083863 -1.785759
F -4.642835 -0.107610 -0.946999

PyrroleNMeIBCMe2O_A_OTf.log
Energy (E) = -1413.44167572 Hartree
Enthalpy (H) = -1413.212837 Hartree
Gibbs free energy (G) = -1413.284976 Hartree
Charge = 0, Spin = 1
C 2.408031 2.714247 0.257523
C 1.136289 2.197363 0.276703
C 1.321704 0.822769 0.050777
C 2.645716 0.526032 -0.077705
H 2.742628 3.732839 0.358302
H 0.211001 2.722005 0.426717
I 0.190717 -0.891900 0.017551
O 1.969577 -1.637380 -0.637121
N 3.319794 1.703997 0.052491
C 4.752025 1.900264 -0.047333
H 5.280789 1.208259 0.606549
H 5.099740 1.759840 -1.069788
H 4.981906 2.914724 0.267080
C 3.105429 -0.905159 -0.181550
C 4.179658 -1.144256 -1.231398
H 5.135399 -0.723045 -0.919738
H 4.304013 -2.218935 -1.356504
H 3.882285 -0.711536 -2.184984
C 3.538331 -1.424054 1.185672
H 3.821756 -2.472952 1.103952
H 4.387112 -0.856218 1.570407
H 2.716627 -1.332189 1.897667
O -1.459609 0.565225 0.702719
S -2.831753 -0.056444 0.798529
O -2.725902 -1.491944 0.665749
O -3.634420 0.514651 1.832634
C -3.565016 0.510336 -0.787028
F -3.637109 1.832402 -0.818510
F -2.799755 0.100548 -1.797704
F -4.777796 0.004091 -0.934848

AnthI8BA_B_Ph.log
Energy (E) = -1086.78697178 Hartree
Enthalpy (H) = -1086.399988 Hartree
Gibbs free energy (G) = -1086.478421 Hartree
Charge = 0, Spin = 1
C -6.961647 -0.800973 0.041959
C -5.898219 -1.506589 0.509909
C -4.563284 -1.080953 0.242973
C -4.363880 0.108096 -0.531145
C -5.506222 0.819513 -1.003304
C -6.762871 0.381323 -0.727055
C -3.458078 -1.786717 0.711522
C -3.066625 0.535098 -0.800394
C -1.965923 -0.176202 -0.329496
C -2.159769 -1.360116 0.440236
C -1.014850 -2.063955 0.898755
H -1.178798 -2.964968 1.479592
C 0.278239 -1.689097 0.643745
C 0.419514 -0.482698 -0.109402
H -3.611421 -2.687669 1.295056
H -7.969200 -1.134365 0.250401
H -6.043299 -2.406923 1.094086
H -5.347249 1.716832 -1.588619
H -7.622171 0.927942 -1.091021
H -2.911632 1.435134 -1.384878
I 2.304845 0.414058 -0.562226
C -0.630420 0.237684 -0.579591
H -0.475443 1.148471 -1.144699
C 1.367529 -2.610764 1.144734
H 1.624106 -3.295142 0.333819
H 0.897708 -3.224080 1.913649
C 2.650084 -1.984805 1.717532
H 2.473156 -0.930692 1.942288
H 2.846542 -2.444946 2.685368
C 3.905575 -2.168563 0.841398
H 4.514332 -1.259997 0.859164
H 4.528793 -2.971121 1.227969
C 3.601096 -2.525271 -0.616609
O 2.751589 -1.755469 -1.229482
O 4.121871 -3.506855 -1.113749
C 1.554357 2.301684 0.142480
C 1.067974 2.421611 1.437843
C 1.629460 3.408313 -0.689184
C 0.645747 3.659825 1.899391
H 1.007400 1.550989 2.081285
C 1.212102 4.650688 -0.220227
H 2.003794 3.311629 -1.701307
C 0.719695 4.774715 1.070507
H 0.262461 3.756606 2.906054
H 1.267141 5.515591 -0.867244
H 0.391797 5.739145 1.433379

FuranIBCMe2S_A_Ph.log
Energy (E) = -987.266751176 Hartree
Enthalpy (H) = -987.024364 Hartree
Gibbs free energy (G) = -987.088031 Hartree
Charge = 0, Spin = 1
C -0.786749 2.966474 -0.116019
C 0.194058 2.035374 -0.117141
C -0.502816 0.791714 -0.068368
C -1.831705 1.036061 -0.035997
O -2.007480 2.380028 -0.065759
H -0.772170 4.040669 -0.152432
H 1.255541 2.204575 -0.153614
I 0.257550 -1.142703 -0.009882
S -2.483223 -1.566202 -0.106545
C -3.033767 0.157222 0.058453
C -3.710712 0.380660 1.413518
H -4.035368 1.419196 1.517879
H -4.580331 -0.272020 1.488340
H -3.027903 0.132920 2.225469
C -4.011023 0.502363 -1.067036
H -4.873163 -0.161191 -1.003015
H -4.352699 1.536323 -0.979282
H -3.540271 0.358352 -2.038391
C 2.277107 -0.299332 0.018021
C 2.877387 0.011667 1.229853
C 2.952314 -0.096039 -1.177005
C 4.162989 0.540715 1.245056
H 2.347114 -0.148557 2.160801
C 4.238321 0.432673 -1.159505
H 2.480291 -0.339877 -2.121087
C 4.841040 0.751531 0.050822
H 4.633296 0.787430 2.187483
H 4.767031 0.595242 -2.089157
H 5.841244 1.162728 0.063648

Indole_NMe_IBCONAc_A_Ph.log
Energy (E) = -965.380109771 Hartree
Enthalpy (H) = -965.066984 Hartree
Gibbs free energy (G) = -965.142892 Hartree
Charge = 0, Spin = 1
C -0.845894 1.649489 -0.000795
C -0.147122 2.884115 -0.000155
C -0.802174 4.115979 -0.000610
C -2.179963 4.111207 -0.001723
C -2.893081 2.902097 -0.002441
C -2.251008 1.681897 -0.002014
C 0.198416 0.676231 -0.000203
H -0.240455 5.040272 -0.000155
H -2.719284 5.048413 -0.002109
H -3.974235 2.927184 -0.003392
H -2.838981 0.777293 -0.002689
N 1.204512 2.652086 0.000752
C 1.418134 1.300204 0.000668
C 2.755869 0.622041 0.001216
O 3.787536 1.272061 0.002739
I 0.203231 -1.408016 -0.000076
C 2.186296 3.723537 0.001219
H 2.051084 4.343353 0.887768
H 3.177132 3.286436 0.002504
H 2.053026 4.342475 -0.886244
N 2.569264 -0.722788 -0.000175
C 3.630454 -1.613632 -0.001125
C 3.182376 -3.065012 -0.000812
H 2.578203 -3.279528 -0.884938
H 4.058012 -3.706764 -0.001037
H 2.578681 -3.279391 0.883671
O 4.807706 -1.330315 -0.001962
C -1.934338 -1.568450 0.000556
C -2.615423 -1.620741 -1.207338
C -2.614866 -1.617676 1.208900
C -4.002790 -1.693771 -1.204163
H -2.075318 -1.581490 -2.144523
C -4.002227 -1.690675 1.206556
H -2.074312 -1.576073 2.145726
C -4.693546 -1.720994 0.001391
H -4.542562 -1.722949 -2.140633
H -4.541565 -1.717446 2.143346
H -5.773704 -1.771081 0.001706

NaphIBMeUreaMe_Ph.log
Energy (E) = -929.461531489 Hartree
Enthalpy (H) = -929.124048 Hartree
Gibbs free energy (G) = -929.197336 Hartree
Charge = 0, Spin = 1
C -2.249807 1.374879 -0.581783
C -3.122550 0.465687 0.063741
C -2.620826 -0.761035 0.548030
C -1.294955 -1.123610 0.423988
C -0.448616 -0.165593 -0.197250
C -0.885561 1.022407 -0.690606
H -3.313771 -1.444499 1.019893
H -0.194385 1.722153 -1.144582
I 1.611035 -0.594068 -0.280651
C -0.016955 -3.217288 0.154190
O -0.000265 -4.404869 0.453004
N 0.633362 -2.626958 -0.856873
C 1.477363 -3.519389 -1.623963
H 0.889765 -4.320424 -2.075225
H 1.959199 -2.956714 -2.426327
H 2.249903 -4.000567 -1.014228
N -0.812837 -2.324296 0.942179
C -1.594172 -2.972589 1.980682
H -1.908299 -2.226402 2.709076
H -2.475535 -3.484524 1.583981
H -0.969837 -3.720297 2.457160
C -4.489924 0.814465 0.187896
C -4.954492 2.002263 -0.305567
C -4.078621 2.907268 -0.946485
C -2.753289 2.598116 -1.080565
H -5.160923 0.120085 0.678263
H -6.001478 2.255311 -0.206006
H -4.461455 3.843396 -1.329118
H -2.070110 3.282406 -1.568544
C 2.061703 1.470134 0.239476
C 1.633091 2.007649 1.446499
C 2.840437 2.223829 -0.626023

C 1.981035 3.308187 1.783795
H 1.021153 1.418323 2.119528
C 3.195129 3.525625 -0.283268
H 3.170165 1.807249 -1.570986
C 2.763815 4.066443 0.919240
H 1.645172 3.729699 2.721695
H 3.801340 4.114598 -0.958404
H 3.035859 5.078850 1.185005

NpthIBCMe2O_C_Ph.log
Energy (E) = -820.030552268 Hartree
Enthalpy (H) = -819.706987 Hartree
Gibbs free energy (G) = -819.775007 Hartree
Charge = 0, Spin = 1
C -2.843800 -0.324539 0.104091
C -1.648640 -0.991136 0.028218
C -0.493447 -0.212024 -0.121118
C -0.487545 1.144208 -0.194458
H -3.765499 -0.880636 0.230098
H 0.427188 1.711613 -0.308055
O -0.307480 -2.870139 -0.487457
I 1.294231 -1.374204 -0.212755
C -1.793363 3.244757 -0.218211
C -2.999670 3.885327 -0.151607
C -4.191410 3.142504 0.003726
C -4.150100 1.778144 0.088074
C -2.916790 1.086141 0.022437
C -1.722964 1.834402 -0.131038
H -0.873822 3.804675 -0.336858
H -3.046667 4.963889 -0.217147
H -5.139706 3.660273 0.055209
H -5.060949 1.203955 0.205530
C -1.483913 -2.511811 0.135160
C -2.637648 -3.237383 -0.551926
H -2.428299 -4.305813 -0.512270
H -3.596273 -3.054673 -0.063262
H -2.698427 -2.932654 -1.595914
C -1.453466 -2.877725 1.626303
H -2.380681 -2.599753 2.131393
H -1.299355 -3.953388 1.716441
H -0.624776 -2.367292 2.122395
C 2.497942 0.416509 0.030360
C 2.726352 0.939651 1.297658
C 3.087706 1.006770 -1.080188
C 3.538250 2.056113 1.453247
H 2.266123 0.481690 2.165281
C 3.903320 2.122673 -0.925030
H 2.909340 0.603187 -2.069907
C 4.126382 2.647229 0.341181
H 3.713231 2.463186 2.440179
H 4.361094 2.581387 -1.791289
H 4.759934 3.515413 0.462582

NpthISO2NMe_D_Ph.log
Energy (E) = -1270.02557544 Hartree
Enthalpy (H) = -1269.734235 Hartree
Gibbs free energy (G) = -1269.803455 Hartree
Charge = 0, Spin = 1
C 3.666497 -1.788112 1.127346
C 4.198229 -0.536807 1.049668
C 3.429306 0.558214 0.596311
C 2.064849 0.394921 0.189478
C 1.591781 -0.947858 0.225821
C 2.341847 -1.987009 0.702847
H 5.077996 1.902486 0.876498
H 4.240963 -2.623844 1.500146
H 5.220744 -0.347961 1.350427
C 4.041476 1.834814 0.571312
C 1.365553 1.588817 -0.162868
H 1.925672 -2.981379 0.762099
C 1.990011 2.806493 -0.155433
C 3.345265 2.941289 0.192708
H 1.396302 3.675017 -0.404584
H 3.810973 3.916417 0.181207
I -0.331505 -1.565542 -0.465745
S -0.344669 1.681200 -0.649420
O -0.411233 1.183219 -2.014105
O -0.783262 3.044497 -0.449415
N -0.953732 0.666094 0.427162
C 0.480252 -3.426131 -1.249506
H 0.567698 -4.174260 -0.470044
H -0.239437 -3.743001 -1.999205
H 1.438261 -3.210037 -1.710371
C -2.346055 0.589760 0.582306
C -3.285108 1.138567 -0.300545
C -2.815107 -0.150924 1.675070
C -4.642934 0.969402 -0.069181
H -2.950039 1.703883 -1.159318
C -4.172194 -0.324031 1.890844
H -2.085245 -0.564851 2.360197
C -5.098400 0.239733 1.021133
H -5.352053 1.412005 -0.756528
H -4.508153 -0.893394 2.747849
H -6.158740 0.112246 1.190965

perF_CMe2O_Ph.log
Energy (E) = -1063.31167854 Hartree
Enthalpy (H) = -1063.066091 Hartree
Gibbs free energy (G) = -1063.135932 Hartree
Charge = 0, Spin = 1
C 1.106497 2.484526 -0.337991
C 2.397383 2.171839 0.047576
C 2.758561 0.845301 0.231504
C 1.854883 -0.185517 0.038867
C 0.552825 0.160690 -0.268290
C 0.172452 1.470010 -0.491772
O 1.219292 -2.390230 -0.517814
I -0.785036 -1.507214 -0.254264
F -1.040752 1.821771 -0.900970
F 0.767309 3.744002 -0.568218
F 3.285938 3.138325 0.213558
F 4.013467 0.609900 0.615167
C 2.209650 -1.691048 0.124097
C 3.523668 -1.979826 -0.604706
H 4.393476 -1.572008 -0.095388
H 3.616784 -3.063897 -0.657859
H 3.477074 -1.592215 -1.621914
C 2.320326 -2.072584 1.604181
H 2.558404 -3.134124 1.670597
H 3.097390 -1.497208 2.110335
H 1.368225 -1.899875 2.110550
C -2.497247 -0.250123 0.132523
C -3.497345 -0.135276 -0.821263
C -2.637463 0.346924 1.377229
C -4.645365 0.591490 -0.529059
H -3.383500 -0.596209 -1.794862
C -3.779420 1.083863 1.662473
H -1.858275 0.244603 2.123975
C -4.783055 1.204760 0.709151
H -5.426497 0.685641 -1.271348
H -3.888359 1.557621 2.628731
H -5.673886 1.775676 0.932743

perF_NMeCO2_Ph.log
Energy (E) = -1153.36408242 Hartree
Enthalpy (H) = -1153.146307 Hartree
Gibbs free energy (G) = -1153.217650 Hartree
Charge = 0, Spin = 1
C 0.863152 2.602630 -0.612508
C 2.083553 2.438899 0.022637
C 2.510546 1.186012 0.430402
C 1.721228 0.039708 0.272433
C 0.477185 0.254135 -0.336173
C 0.067522 1.488590 -0.804043
I -0.790246 -1.383918 -0.542705
F -1.089399 1.633300 -1.440917
F 0.478137 3.796278 -1.038612
F 2.873100 3.485194 0.193548
F 3.734790 1.102399 0.942716
N 2.140249 -1.213725 0.673852
C 3.000154 -1.372067 1.841472
H 2.784501 -0.581509 2.557523
H 2.780841 -2.338292 2.285770
H 4.057444 -1.351335 1.584151
C 2.107443 -2.335993 -0.246960
O 2.794384 -3.293176 0.044578
O 1.337035 -2.164730 -1.253407
C -2.448537 -0.327348 0.292823
C -3.540364 -0.018362 -0.503260
C -2.426879 -0.009780 1.642606
C -4.634641 0.619092 0.067586
H -3.537168 -0.253642 -1.559573
C -3.519134 0.639750 2.203112
H -1.569210 -0.263161 2.253351
C -4.621615 0.949237 1.416568
H -5.490959 0.866006 -0.544593
H -3.510164 0.896689 3.253331
H -5.472683 1.450670 1.856239

PyIBCMe2O_D_Ph.log
Energy (E) = -682.560145341 Hartree
Enthalpy (H) = -682.297955 Hartree
Gibbs free energy (G) = -682.359783 Hartree
Charge = 0, Spin = 1
C 0.655911 3.120907 -0.257567
C 2.031848 3.071851 -0.082891
C 2.055496 0.783598 -0.016708
C 0.682220 0.766421 -0.170556
H 0.147703 4.068702 -0.360567
H 2.608815 3.988222 -0.038037
O 2.001994 -1.549121 -0.379629
I -0.155368 -1.184553 -0.145637
C -0.051187 1.925274 -0.287543
H -1.126651 1.913105 -0.401123
N 2.718626 1.938544 0.041948
C 2.796530 -0.541484 0.131385
C 4.110803 -0.489970 -0.640394
H 4.751434 0.313265 -0.279315
H 4.613976 -1.448350 -0.515609
H 3.907530 -0.339436 -1.700205
C 3.069116 -0.744281 1.627497
H 3.595719 -1.689561 1.759133
H 3.670439 0.069205 2.036023
H 2.125809 -0.795388 2.177314
C -2.146541 -0.327668 0.026460
C -2.942664 -0.192213 -1.104034
C -2.642814 0.039125 1.271866
C -4.233326 0.313418 -0.990646
H -2.559131 -0.475816 -2.077096
C -3.931061 0.547071 1.385861
H -2.024601 -0.066200 2.155725
C -4.725571 0.684225 0.253864
H -4.851466 0.418114 -1.872266
H -4.314911 0.833112 2.355984
H -5.729045 1.077821 0.342627

PyrroleNMeIBCMe2O_A_Ph.log
Energy (E) = -683.751238961 Hartree
Enthalpy (H) = -683.465611 Hartree
Gibbs free energy (G) = -683.530542 Hartree
Charge = 0, Spin = 1
C 1.186177 2.739682 -0.081793
C 0.057306 1.961137 -0.116411
C 0.541873 0.636954 -0.098061
C 1.901619 0.619596 -0.042062
H 1.293981 3.811529 -0.098738
H -0.964243 2.297823 -0.153739
I -0.391238 -1.214294 -0.056086
O 1.809024 -1.700146 -0.195647
N 2.296576 1.930561 -0.030459
C 3.654592 2.432909 -0.045653
H 4.266127 1.907465 0.684463
H 4.108328 2.320411 -1.029785
H 3.633597 3.488504 0.215222
C 2.695163 -0.672349 0.059099
C 3.825282 -0.731283 -0.972203
H 4.624447 -0.014563 -0.772174
H 4.245600 -1.736182 -0.936990
H 3.424360 -0.560344 -1.971165
C 3.265704 -0.814138 1.476724
H 3.789987 -1.768101 1.540684
H 3.959605 -0.012463 1.742641
H 2.444143 -0.820168 2.194320
C -2.313705 -0.223510 0.012137
C -2.996509 0.043804 -1.167180
C -2.858174 0.154544 1.232761
C -4.226515 0.690599 -1.126065
H -2.569607 -0.244396 -2.120131
C -4.087298 0.801876 1.273988
H -2.322815 -0.047735 2.152514
C -4.770785 1.069158 0.094348
H -4.757082 0.899370 -2.045448
H -4.509826 1.097190 2.225095
H -5.727596 1.572583 0.126432

AnthI8BA_B_Me.log
Energy (E) = -895.225114687 Hartree
Enthalpy (H) = -894.894947 Hartree
Gibbs free energy (G) = -894.964487 Hartree
Charge = 0, Spin = 1
C 6.766947 0.678183 -0.061478
C 5.656472 1.412841 0.212292
C 4.355369 0.837803 0.108783
C 4.240580 -0.533131 -0.290861
C 5.429917 -1.268950 -0.569577
C 6.652187 -0.684612 -0.458971
C 3.202957 1.570283 0.382390
C 2.976789 -1.107716 -0.395277
C 1.829162 -0.369941 -0.117650
C 1.938476 0.995430 0.275408
C 0.746202 1.723113 0.532858
H 0.846547 2.765197 0.815765
C -0.517341 1.204282 0.428293
C -0.573988 -0.178974 0.070364
H 3.291859 2.609202 0.679729
H 7.748241 1.125516 0.019986
H 5.737445 2.449922 0.513526
H 5.335101 -2.304455 -0.872546
H 7.547753 -1.251674 -0.673501
H 2.886976 -2.145449 -0.697082
I -2.384325 -1.294474 -0.033304
C 0.525284 -0.928549 -0.201849
H 0.437008 -1.965337 -0.499917
C -1.668009 2.162406 0.643008
H -1.960511 2.551347 -0.334473
H -1.243352 3.007765 1.184519
C -2.914854 1.664128 1.392288
H -2.673930 0.747491 1.933467
H -3.151345 2.390226 2.169458
C -4.169157 1.485032 0.513492
H -4.715365 0.586750 0.815294
H -4.849479 2.323406 0.640765
C -3.872335 1.395696 -0.986743
O -2.968066 0.533242 -1.340148
O -4.451907 2.141303 -1.755501
C -1.500688 -2.812189 1.260383
H -0.833183 -3.451987 0.694430
H -0.969651 -2.305290 2.059474
H -2.328941 -3.392665 1.658481

FuranIBCMe2S_A_Me.log
Energy (E) = -795.705502267 Hartree
Enthalpy (H) = -795.520221 Hartree
Gibbs free energy (G) = -795.573965 Hartree
Charge = 0, Spin = 1
C -0.951765 2.920056 -0.135267
C 0.296523 2.398274 -0.147121
C 0.091013 0.987509 -0.097501
C -1.238185 0.746110 -0.045962
O -1.881742 1.937177 -0.070145
H -1.320863 3.928444 -0.182364
H 1.219837 2.945566 -0.203534
I 1.461736 -0.569116 -0.010936
S -0.954407 -1.889271 -0.400240
C -2.023424 -0.513425 0.111920
C -2.435408 -0.653631 1.580128
H -3.060598 0.187860 1.891324
H -2.996001 -1.579604 1.706485
H -1.552974 -0.699854 2.218984
C -3.267265 -0.473778 -0.774652
H -3.805239 -1.415456 -0.669025
H -3.927452 0.344958 -0.480324
H -2.988257 -0.353583 -1.820254
C 3.076851 0.937672 0.224675
H 3.987102 0.362933 0.371132
H 3.142791 1.531483 -0.681208
H 2.864059 1.548629 1.096092

Indole_NMe_IBCONAc_A_Me.log
Energy (E) = -773.817515260 Hartree
Enthalpy (H) = -773.561157 Hartree
Gibbs free energy (G) = -773.629026 Hartree
Charge = 0, Spin = 1
C 2.005632 -0.356422 0.000174
C 2.535089 0.959262 0.000067
C 3.906637 1.214342 0.000286
C 4.762773 0.134661 0.000579
C 4.262781 -1.176865 0.000679
C 2.908045 -1.432064 0.000492
C 0.593914 -0.152227 -0.000022
H 4.279183 2.229711 0.000254
H 5.831569 0.297548 0.000752
H 4.956130 -2.006644 0.000928
H 2.569014 -2.455190 0.000616
N 1.509593 1.868837 -0.000204
C 0.320849 1.191228 -0.000227
C -1.043746 1.815360 -0.000449
O -1.177326 3.027469 -0.000912
I -1.035096 -1.447263 -0.000154
C 1.733132 3.305266 -0.000163
H 2.301256 3.586646 -0.886886
H 0.772732 3.805696 -0.001032
H 2.299745 3.586915 0.887447
N -1.976472 0.831040 -0.000020
C -3.335048 1.102756 0.000403
C -4.188401 -0.153589 0.000109
H -3.977877 -0.758849 0.884464
H -5.236509 0.129248 0.000249
H -3.977956 -0.758420 -0.884555
O -3.848505 2.199246 0.001018
C 0.143195 -3.276096 -0.000519
H 0.744796 -3.324272 -0.900526
H 0.744312 -3.324988 0.899770
H -0.600613 -4.068972 -0.001037

NaphIBMeUreaMe_Me.log
Energy (E) = -737.899919356 Hartree
Enthalpy (H) = -737.619473 Hartree
Gibbs free energy (G) = -737.683737 Hartree
Charge = 0, Spin = 1
C -2.610039 -0.576149 0.284460
C -2.749344 0.795219 -0.038350
C -1.602162 1.567251 -0.319125
C -0.326279 1.041164 -0.300524
C -0.235895 -0.349390 -0.016091
C -1.309347 -1.129484 0.278229
H -1.736659 2.617682 -0.539224
H -1.189477 -2.182070 0.500729
I 1.658493 -1.247238 -0.138772
C 1.948922 1.833764 0.233845
O 2.689318 2.808509 0.185732
N 2.083173 0.748496 1.005108
C 3.277986 0.744284 1.822858
H 3.295842 1.605629 2.492512
H 3.293390 -0.162155 2.431405
H 4.198356 0.788790 1.229737
N 0.789118 1.815070 -0.609042
C 0.576102 3.033795 -1.369276
H -0.104327 2.826470 -2.193997
H 0.167063 3.843086 -0.757583
H 1.535362 3.366170 -1.750314
C -4.049191 1.358327 -0.047442
C -5.142980 0.593534 0.250518
C -4.998247 -0.775030 0.571360
C -3.755938 -1.346398 0.587369
H -4.159034 2.407726 -0.291306
H -6.129773 1.036467 0.241888
H -5.873234 -1.366411 0.803496
H -3.630993 -2.394266 0.831896
C 0.780635 -2.960540 -1.221781
H 0.292497 -3.639962 -0.531121
H 0.080279 -2.579491 -1.958277
H 1.614662 -3.459187 -1.709553

NpthIBCMe2O_C_Me.log
Energy (E) = -628.467933971 Hartree
Enthalpy (H) = -628.201420 Hartree
Gibbs free energy (G) = -628.260157 Hartree
Charge = 0, Spin = 1
C 1.506282 1.513545 0.025476
C 0.199464 1.103737 -0.016120
C -0.037315 -0.277755 -0.058847
C 0.944253 -1.217967 -0.064532
H 1.739275 2.571025 0.069082
H 0.734076 -2.277563 -0.103360
O -2.079180 1.390578 -0.571712
I -2.103851 -0.773491 -0.095129
C 3.366457 -1.722905 -0.058638
C 4.662390 -1.287561 -0.028328

C 4.946625 0.095369 0.023469
C 3.930071 1.010012 0.042464
C 2.578474 0.590373 0.011979
C 2.296724 -0.797419 -0.036527
H 3.139191 -2.781285 -0.098609
H 5.475596 -2.000426 -0.043577
H 5.975611 0.427792 0.047239
H 4.142278 2.071429 0.079900
C -1.015919 2.038613 0.016098
C -0.739671 3.322907 -0.761227
H -1.659156 3.907136 -0.771885
H 0.047766 3.926501 -0.306392
H -0.467185 3.083836 -1.788407
C -1.311275 2.376100 1.485193
H -0.474222 2.892971 1.958828
H -2.197306 3.010517 1.522970
H -1.515723 1.463531 2.049558
C -1.652373 -2.877451 0.364820
H -1.175106 -3.370042 -0.476734
H -1.044535 -2.947746 1.262039
H -2.628118 -3.324859 0.543225

NpthISO2NMe_D_Me.log
Energy (E) = -1078.45092856 Hartree
Enthalpy (H) = -1078.215806 Hartree
Gibbs free energy (G) = -1078.277064 Hartree
Charge = 0, Spin = 1
C 1.381392 3.285550 -0.616755
C 2.548451 2.624101 -0.381470
C 2.559836 1.240849 -0.091770
C 1.344830 0.485702 -0.029297
C 0.156136 1.249599 -0.204533
C 0.169104 2.580234 -0.514069
H 4.699239 1.224555 0.074555
H 1.369726 4.336680 -0.866857
H 3.498686 3.140698 -0.426400
C 3.809401 0.609314 0.118106
C 1.483297 -0.920267 0.166609
H -0.755764 3.111649 -0.680990
C 2.711617 -1.495676 0.346549
C 3.890785 -0.730357 0.349508
H 2.751606 -2.570947 0.455119
H 4.846282 -1.211656 0.502612
I -1.806709 0.465894 0.070560
S 0.142068 -2.088949 0.168907
O -0.460669 -2.032978 1.491519
O 0.675515 -3.376150 -0.225456
N -0.755493 -1.430028 -0.974343
C -1.826571 -2.299508 -1.447382
H -2.276709 -1.844156 -2.329087
H -1.439674 -3.279986 -1.728957
H -2.616665 -2.457468 -0.698385
C -2.449850 2.330431 1.030228
H -2.718487 3.082786 0.296810
H -3.321883 2.046425 1.613839
H -1.654754 2.675591 1.683087

perF_CMe2O_Me.log
Energy (E) = -871.751516499 Hartree
Enthalpy (H) = -871.562747 Hartree
Gibbs free energy (G) = -871.623594 Hartree
Charge = 0, Spin = 1
C -2.115691 -1.597670 -0.082329
C -2.798395 -0.407327 0.089207
C -2.096685 0.789015 0.120388
C -0.719826 0.828326 -0.019626
C -0.056264 -0.380358 -0.113216
C -0.732076 -1.582051 -0.175911
O 1.329626 1.811688 -0.649122
I 2.069543 -0.184931 -0.049424
F -0.124449 -2.751127 -0.369527
F -2.777713 -2.741907 -0.163437
F -4.116649 -0.415664 0.200019
F -2.809706 1.899345 0.306646
C 0.118892 2.129047 -0.092495
C -0.552368 3.158426 -1.004264
H -1.465638 3.572532 -0.583882
H 0.170230 3.960806 -1.148028
H -0.762854 2.712697 -1.975945
C 0.260175 2.682308 1.329807
H 0.858027 3.592713 1.287864
H -0.711556 2.910323 1.771302
H 0.775143 1.959644 1.966318
C 2.355846 -2.235752 0.670391
H 1.640164 -2.456973 1.455052
H 2.285934 -2.952059 -0.137704
H 3.362766 -2.211449 1.082459

perF_NMeCO2_Me.log
Energy (E) = -961.803678943 Hartree
Enthalpy (H) = -961.642600 Hartree
Gibbs free energy (G) = -961.704654 Hartree
Charge = 0, Spin = 1
C -2.406272 -1.277100 -0.216925
C -2.886094 0.005197 -0.004562
C -2.016640 1.072056 0.149894
C -0.624423 0.911882 0.149316
C -0.179215 -0.405471 -0.031487
C -1.037715 -1.467108 -0.244685
I 1.870853 -0.751286 0.003166
F -0.568499 -2.692555 -0.469981
F -3.239125 -2.290321 -0.400365
F -4.189976 0.219011 0.000762
F -2.558634 2.281137 0.254283
N 0.242270 1.973709 0.307405
C -0.115694 3.108722 1.151144
H -0.766099 2.770032 1.955054
H 0.801870 3.512420 1.568371
H -0.609798 3.901189 0.592343
C 1.325246 2.196253 -0.633482
O 1.832645 3.298537 -0.621724
O 1.606348 1.178603 -1.354539
C 1.659936 -2.420567 1.370791
H 1.485494 -3.328449 0.808690
H 2.588175 -2.470168 1.931813
H 0.828635 -2.198586 2.031879

PyIBCMe2O_D_Me.log
Energy (E) = -490.997232339 Hartree
Enthalpy (H) = -490.792130 Hartree
Gibbs free energy (G) = -490.844696 Hartree
Charge = 0, Spin = 1
C 1.397704 2.899754 -0.055011
C 2.561961 2.149769 0.026510
C 1.420294 0.165320 -0.017180
C 0.221962 0.853481 -0.080273
H 1.438267 3.979000 -0.081098
H 3.525997 2.642375 0.076267
O 0.183049 -1.782073 -0.508668
I -1.482898 -0.399181 -0.080459
C 0.181035 2.229738 -0.094378
H -0.747450 2.779276 -0.142298
N 2.579357 0.818982 0.051974
C 1.387390 -1.359712 0.015615
C 2.531049 -1.921562 -0.822186
H 3.498140 -1.585896 -0.450695
H 2.476499 -3.008983 -0.780737
H 2.414512 -1.607044 -1.858819
C 1.548289 -1.785174 1.481217
H 1.523458 -2.873829 1.530143
H 2.487918 -1.422129 1.900262
H 0.721689 -1.394203 2.079945
C -2.784928 1.336425 0.307917
H -2.459846 1.867611 1.197901
H -2.827219 1.995838 -0.553787
H -3.766818 0.900056 0.479374

PyrroleNMeIBCMe2O_A_Me.log
Energy (E) = -492.189862374 Hartree
Enthalpy (H) = -491.961262 Hartree
Gibbs free energy (G) = -492.016687 Hartree
Charge = 0, Spin = 1
C 1.529351 2.464792 0.002024
C 0.171114 2.275572 -0.032450
C 0.006676 0.874042 -0.067243
C 1.216471 0.249875 -0.040529
H 2.105817 3.374830 0.013208
H -0.581139 3.044400 -0.040293
I -1.651372 -0.366316 -0.042175
O 0.106175 -1.777381 -0.295452
N 2.157299 1.242893 0.003728
C 3.596550 1.081924 -0.013853
H 3.906407 0.325446 0.703898
H 3.950635 0.793812 -1.002869
H 4.051537 2.030126 0.262753
C 1.347813 -1.263867 0.012548
C 2.361347 -1.789810 -1.007591
H 3.392462 -1.526999 -0.762884
H 2.274932 -2.876089 -1.016734
H 2.115461 -1.417014 -2.001854
C 1.758204 -1.685159 1.430536
H 1.812939 -2.773745 1.463202
H 2.723320 -1.273606 1.738033
H 0.994726 -1.354128 2.136248
C -2.940188 1.393246 0.143609
H -2.821507 2.016662 -0.737239
H -3.962688 1.030131 0.209386
H -2.674716 1.936027 1.045624

AnthI8BA_B_vinyl.log
Energy (E) = -933.267736001 Hartree
Enthalpy (H) = -932.931489 Hartree
Gibbs free energy (G) = -933.003326 Hartree
Charge = 0, Spin = 1
C -6.833072 -0.669464 -0.050620
C -5.737476 -1.425809 0.224289
C -4.424869 -0.879269 0.112822
C -4.282422 0.486345 -0.295991
C -5.456756 1.245470 -0.575557
C -6.690842 0.687993 -0.457241
C -3.287291 -1.634282 0.387988
C -3.007001 1.033280 -0.408542
C -1.874484 0.272797 -0.130617
C -2.011430 -1.087162 0.272700
C -0.833950 -1.836314 0.537552
H -0.955309 -2.870970 0.838763
C 0.439519 -1.344482 0.425079
C 0.523569 0.031190 0.043145
H -3.397454 -2.668759 0.693608
H -7.823345 -1.095365 0.036976
H -5.839497 -2.458963 0.532576
H -5.340830 2.276742 -0.885535
H -7.574910 1.272549 -0.672530
H -2.895357 2.066736 -0.717316

I 2.359997 1.107735 -0.098689
C -0.559705 0.803840 -0.226628
H -0.447953 1.840206 -0.519962
C 1.572634 -2.315171 0.671335
H 1.883108 -2.718856 -0.294161
H 1.127313 -3.149380 1.213348
C 2.805108 -1.815823 1.444833
H 2.563839 -0.879349 1.950636
H 3.005396 -2.521111 2.250686
C 4.088946 -1.682239 0.601445
H 4.640666 -0.784473 0.894936
H 4.751407 -2.527091 0.772360
C 3.839154 -1.628835 -0.907654
O 2.954710 -0.764837 -1.310191
O 4.431223 -2.397925 -1.641869
C 1.465421 2.699955 1.018472
H 0.978949 2.412259 1.941894
C 1.580959 3.957641 0.627166
H 2.063194 4.229380 -0.305146
H 1.197410 4.770628 1.231993

FuranIBCMe2S_A_vinyl.log
Energy (E) = -833.746468645 Hartree
Enthalpy (H) = -833.555033 Hartree
Gibbs free energy (G) = -833.611266 Hartree
Charge = 0, Spin = 1
C -0.877912 2.967814 -0.120277
C 0.320119 2.338810 -0.095745
C -0.012045 0.954003 -0.054551
C -1.356889 0.827686 -0.047353
O -1.893655 2.069708 -0.087103
H -1.156772 4.004204 -0.178127
H 1.295147 2.790850 -0.132445
I 1.212535 -0.717596 0.064608
S -1.275823 -1.808643 -0.479281
C -2.242546 -0.368569 0.066079
C -2.682023 -0.521589 1.524787
H -3.243197 0.356600 1.855943
H -3.314608 -1.404112 1.617443
H -1.813831 -0.655367 2.170988
C -3.467549 -0.206723 -0.832092
H -4.078422 -1.106227 -0.761201
H -4.065825 0.651862 -0.519720
H -3.166668 -0.076684 -1.870404
C 2.907588 0.611921 0.432105
H 2.884194 1.178078 1.355480
C 3.941308 0.629655 -0.391019
H 3.952345 0.055463 -1.310954
H 4.825407 1.220498 -0.177507

Indole_NMe_IBCONAc_A_vinyl.log
Energy (E) = -811.858721307 Hartree
Enthalpy (H) = -811.596404 Hartree
Gibbs free energy (G) = -811.665858 Hartree
Charge = 0, Spin = 1
C 2.001104 -0.194059 0.002603
C 2.529484 1.121343 0.007637
C 3.901137 1.371517 0.052724
C 4.750935 0.287114 0.099361
C 4.247621 -1.023827 0.109749
C 2.892881 -1.275179 0.064253
C 0.592164 0.008585 -0.042359
H 4.279163 2.384942 0.054093
H 5.819762 0.446387 0.135364
H 4.938862 -1.853877 0.160151
H 2.534967 -2.292672 0.094480
N 1.501993 2.029987 -0.023726
C 0.314744 1.349604 -0.048992
C -1.054937 1.959977 -0.044580
O -1.204283 3.167176 -0.127059
I -1.022550 -1.296152 -0.120325
C 1.717036 3.466992 0.005234
H 2.310051 3.765455 -0.859282
H 0.753949 3.962037 -0.020890
H 2.254005 3.739426 0.913962
N -1.976580 0.967112 0.049263
C -3.333516 1.229051 0.148392
C -4.182736 -0.029326 0.107460
H -3.927829 -0.688282 0.940326
H -5.230324 0.246421 0.178458
H -4.014601 -0.575928 -0.822941
O -3.846073 2.318741 0.273255
C 0.159727 -3.069177 -0.348109
H 0.751876 -3.131871 -1.251120
C 0.041204 -4.065749 0.511631
H -0.559682 -3.985393 1.409691
H 0.548828 -5.008782 0.346608

NaphIBMeUreaMe_vinyl.log
Energy (E) = -775.942649203 Hartree
Enthalpy (H) = -775.655962 Hartree
Gibbs free energy (G) = -775.722566 Hartree
Charge = 0, Spin = 1
C -2.621056 -0.558209 0.300617
C -2.832948 0.798952 -0.042384
C -1.728573 1.626809 -0.337252
C -0.427004 1.169143 -0.311494
C -0.263112 -0.207820 0.002446
C -1.292540 -1.041594 0.305058
H -1.919706 2.664053 -0.576930
H -1.110062 -2.084608 0.534052
I 1.677251 -1.008012 -0.093557
C 1.806619 2.094787 0.189621
O 2.485051 3.112386 0.128456
N 2.017536 1.027826 0.971445
C 3.215109 1.108676 1.781575
H 3.174273 1.967712 2.453012
H 3.298873 0.204944 2.388873
H 4.125523 1.219791 1.182662
N 0.645510 1.994781 -0.642680
C 0.363232 3.184312 -1.426763
H -0.312000 2.923374 -2.240336
H -0.082516 3.984080 -0.828558
H 1.300320 3.558032 -1.824258
C -4.160865 1.291603 -0.060579
C -5.212715 0.473597 0.247448
C -4.995743 -0.880572 0.587569
C -3.724569 -1.384423 0.612932
H -4.326499 2.329951 -0.319793
H -6.221754 0.863032 0.231666
H -5.838324 -1.514533 0.827168
H -3.543440 -2.420361 0.872241
C 0.875379 -2.793231 -1.026737
H 0.280342 -2.652704 -1.920927
C 1.185140 -3.991482 -0.559993
H 1.775624 -4.121668 0.340973
H 0.865178 -4.897095 -1.062706

NpthIBCMe2O_C_vinyl.log
Energy (E) = -666.510713736 Hartree
Enthalpy (H) = -666.237952 Hartree
Gibbs free energy (G) = -666.299338 Hartree
Charge = 0, Spin = 1
C 1.742295 1.495113 0.000757
C 0.400411 1.219495 -0.023473
C 0.027907 -0.131088 -0.029952
C 0.903499 -1.169097 -0.025982
H 2.083920 2.523557 0.013878
H 0.571471 -2.199365 -0.052284
O -1.842799 1.704992 -0.591752
I -2.077841 -0.417535 -0.031507
C 3.262534 -1.917290 -0.028884
C 4.596219 -1.615097 -0.015580
C 5.019446 -0.267548 0.008148
C 4.100923 0.745708 0.016200
C 2.713462 0.465310 0.003148
C 2.292102 -0.888049 -0.016320
H 2.928372 -2.947421 -0.047798
H 5.332862 -2.406959 -0.023229
H 6.076978 -0.040764 0.018562
H 4.420148 1.780577 0.031084
C -0.718678 2.268226 -0.024651
C -0.320939 3.489076 -0.849416
H -1.176956 4.162244 -0.880521
H 0.525625 4.025888 -0.417775
H -0.078391 3.186214 -1.867176
C -0.978669 2.686881 1.429623
H -0.094315 3.137095 1.884857
H -1.798557 3.405561 1.442514
H -1.269448 1.820486 2.027767
C -1.813926 -2.499717 0.503430
H -1.387855 -2.712778 1.477066
C -2.277622 -3.463180 -0.276903
H -2.702672 -3.253869 -1.252699
H -2.257000 -4.503488 0.028777

NpthISO2NMe_D_vinyl.log
Energy (E) = -1116.49402464 Hartree
Enthalpy (H) = -1116.252775 Hartree
Gibbs free energy (G) = -1116.316498 Hartree
Charge = 0, Spin = 1
C 0.224286 3.519861 -0.601704
C 1.554426 3.337144 -0.374205
C 2.079463 2.055194 -0.093164
C 1.230903 0.903635 -0.030596
C -0.156122 1.171539 -0.210507
C -0.639697 2.413887 -0.507028
H 4.073138 2.832810 0.063916
H -0.177873 4.493594 -0.841923
H 2.245225 4.169530 -0.417413
C 3.475095 1.931411 0.109347
C 1.881985 -0.349330 0.170165
H -1.698645 2.561321 -0.665699
C 3.236849 -0.429846 0.343591
C 4.049010 0.717385 0.337068
H 3.671398 -1.413424 0.458625
H 5.115689 0.624628 0.484121
I -1.694244 -0.290093 0.033544
S 1.062526 -1.926007 0.203991
O 0.469428 -2.059310 1.525128
O 2.028189 -2.940469 -0.162016
N -0.009633 -1.669899 -0.952846
C -0.668913 -2.885486 -1.417700
H -1.254177 -2.642846 -2.304290
H 0.063702 -3.646482 -1.689732
H -1.342222 -3.325599 -0.667792
C -2.989836 1.208792 0.871743
H -2.612595 1.741228 1.735575
C -4.225058 1.349957 0.420744
H -4.584832 0.808616 -0.447501
H -4.932138 2.010339 0.908905

perF_CMe2O_vinyl.log
Energy (E) = -909.792551962 Hartree
Enthalpy (H) = -909.597730 Hartree

Gibbs free energy (G) = -909.661469 Hartree
Charge = 0, Spin = 1
C -1.798869 -2.004379 -0.074210
C -2.761229 -1.021137 0.065201
C -2.381878 0.312775 0.090605
C -1.056091 0.693581 -0.028164
C -0.109519 -0.310796 -0.102442
C -0.461078 -1.645324 -0.147439
O 0.697566 2.165226 -0.602372
I 1.898397 0.416932 -0.017129
F 0.421792 -2.629145 -0.294059
F -2.151974 -3.279155 -0.138881
F -4.037136 -1.358133 0.157311
F -3.352851 1.211374 0.252130
C -0.572514 2.163496 -0.085959
C -1.451575 2.991452 -1.025411
H -2.453988 3.157480 -0.638531
H -0.953173 3.952154 -1.149095
H -1.508219 2.509794 -2.001041
C -0.617236 2.731768 1.336386
H -0.268307 3.763917 1.307145
H -1.627062 2.705106 1.749298
H 0.045833 2.160872 1.989995
C 2.700245 -1.489857 0.580262
H 2.241420 -1.992375 1.421158
C 3.813449 -1.917116 0.011738
H 4.259098 -1.407622 -0.836093
H 4.328096 -2.799802 0.372088

perF_NMeCO2_vinyl.log
Energy (E) = -999.844212551 Hartree
Enthalpy (H) = -999.677084 Hartree
Gibbs free energy (G) = -999.741901 Hartree
Charge = 0, Spin = 1
C -2.273892 -1.569753 -0.275240
C -2.935924 -0.401798 0.066800
C -2.238934 0.779920 0.256789
C -0.842609 0.849797 0.164262
C -0.208443 -0.359077 -0.150574
C -0.897849 -1.530119 -0.395828
I 1.872111 -0.366869 -0.258674
F -0.255042 -2.642698 -0.745337
F -2.945293 -2.690666 -0.491378
F -4.253812 -0.405706 0.164245
F -2.957869 1.873510 0.490120
N -0.145052 2.025344 0.360885
C -0.620980 3.022740 1.313178
H -1.152624 2.524709 2.121410
H 0.246550 3.541246 1.710122
H -1.272424 3.761062 0.849573
C 0.822968 2.488053 -0.613497
O 1.146326 3.655635 -0.544838
O 1.221123 1.582202 -1.425535
C 2.042781 -2.100305 0.977889
H 1.839887 -3.048842 0.504032
C 2.435445 -1.973895 2.232682
H 2.631072 -1.009758 2.685772
H 2.574178 -2.847807 2.857997

PyIBCMe2O_D_vinyl.log
Energy (E) = -529.039962150 Hartree
Enthalpy (H) = -528.828611 Hartree
Gibbs free energy (G) = -528.883826 Hartree
Charge = 0, Spin = 1
C 0.991022 3.071541 -0.025670
C 2.280543 2.559567 0.013719
C 1.544719 0.393064 -0.028878
C 0.237094 0.839540 -0.041238
H 0.823272 4.138658 -0.041495
H 3.131780 3.229647 0.044121
O 0.676761 -1.741831 -0.537502
I -1.195406 -0.716803 -0.007764
C -0.074425 2.179527 -0.040208
H -1.098873 2.525913 -0.065346
N 2.557977 1.257133 0.015562
C 1.800316 -1.111231 -0.037474
C 2.996112 -1.427916 -0.928692
H 3.895409 -0.922495 -0.579770
H 3.150184 -2.506472 -0.916978
H 2.782952 -1.118040 -1.951225
C 2.091165 -1.534733 1.407937
H 2.272322 -2.609459 1.424897
H 2.961283 -1.011545 1.807198
H 1.229537 -1.320096 2.045120
C -2.747953 0.720909 0.475108
H -2.700569 1.199598 1.446703
C -3.778704 0.903167 -0.335279
H -3.832486 0.426543 -1.308253
H -4.617522 1.532400 -0.058352

PyrroleNMeIBCMe2O_A_vinyl.log
Energy (E) = -530.231006350 Hartree
Enthalpy (H) = -529.996210 Hartree
Gibbs free energy (G) = -530.054368 Hartree
Charge = 0, Spin = 1
C 1.416819 2.576444 0.049025
C 0.089114 2.230928 0.053338
C 0.089474 0.822111 -0.008145
C 1.362329 0.341454 -0.038006
H 1.884487 3.546880 0.062047
H -0.753455 2.899551 0.075881
I -1.409121 -0.606737 0.041568
O 0.475789 -1.785997 -0.346102
N 2.182834 1.436050 0.000417
C 3.629303 1.443589 -0.070066
H 4.049663 0.710338 0.615010
H 3.978309 1.223337 -1.078212
H 3.982264 2.430753 0.218943
C 1.663705 -1.148364 -0.049426
C 2.694554 -1.518925 -1.118742
H 3.697970 -1.154022 -0.890735
H 2.726890 -2.606873 -1.172160
H 2.378122 -1.136096 -2.088930
C 2.163868 -1.575992 1.336975
H 2.339875 -2.652033 1.323646
H 3.087563 -1.070509 1.631348
H 1.393966 -1.358386 2.078903
C -2.874186 0.943883 0.364041
H -2.783190 1.507679 1.284900
C -3.867180 1.147982 -0.485375
H -3.954071 0.588261 -1.410021
H -4.637920 1.885117 -0.289566

AnthI8BA_B_CF2CF3.log
Energy (E) = -1430.52729949 Hartree
Enthalpy (H) = -1430.202073 Hartree
Gibbs free energy (G) = -1430.284051 Hartree
Charge = 0, Spin = 1
C -7.310834 0.053816 0.245170
C -6.325998 -0.746226 0.733050
C -4.961140 -0.516770 0.389351
C -4.647204 0.575590 -0.482669
C -5.709316 1.391030 -0.972819
C -6.998106 1.139708 -0.621533
C -3.934469 -1.322515 0.876066
C -3.319066 0.811468 -0.827030
C -2.298312 0.002629 -0.335507
C -2.605918 -1.086656 0.531318
C -1.541796 -1.902599 1.001860
H -1.795622 -2.740937 1.641208
C -0.224788 -1.712142 0.681155
C 0.031544 -0.584772 -0.156038
H -4.175259 -2.150702 1.533070
H -8.342453 -0.130045 0.512785
H -6.558289 -1.573636 1.391957
H -5.463618 2.214594 -1.631711
H -7.796565 1.763831 -0.998930
H -3.076975 1.638871 -1.484400
I 1.997749 0.016162 -0.698115
C -0.932884 0.227332 -0.658220
H -0.689231 1.060033 -1.304234
C 0.765373 -2.743791 1.173113
H 0.885109 -3.492292 0.385890
H 0.265859 -3.259256 1.992791
C 2.149824 -2.271395 1.648561
H 2.121200 -1.201297 1.860711
H 2.352644 -2.739925 2.610311
C 3.314868 -2.626526 0.699107
H 4.030103 -1.801247 0.652143
H 3.856961 -3.492086 1.070171
C 2.871518 -2.991272 -0.716011
O 2.148247 -2.105787 -1.359683
O 3.148724 -4.075903 -1.181692
C 1.493081 2.040950 0.184412
C 2.792777 2.725297 0.622303
F 0.889040 2.847966 -0.707383
F 0.701517 1.925732 1.257869
F 2.580042 3.963513 1.036010
F 3.358216 2.032352 1.605310
F 3.635042 2.755113 -0.413419

FuranIBCMe2S_A_CF2CF3.log
Energy (E) = -1331.01030238 Hartree
Enthalpy (H) = -1330.829778 Hartree
Gibbs free energy (G) = -1330.895997 Hartree
Charge = 0, Spin = 1
C -1.821862 2.945715 -0.188610
C -0.622587 2.334100 -0.319972
C -0.926242 0.945817 -0.200068
C -2.254799 0.810052 0.002422
O -2.812348 2.038103 0.010746
H -2.119786 3.977361 -0.230350
H 0.325637 2.806038 -0.494777
I 0.228079 -0.786695 -0.211139
S -2.221555 -1.805759 -0.492803
C -3.078928 -0.401868 0.285676
C -3.187374 -0.592926 1.799684
H -3.677296 0.268161 2.260634
H -3.770059 -1.488972 2.011340
H -2.197888 -0.714625 2.242166
C -4.467204 -0.269131 -0.333953
H -5.030674 -1.183505 -0.150611
H -5.003455 0.568625 0.115052
H -4.397247 -0.113692 -1.409110
C 2.067610 0.632231 0.008189
C 3.331696 -0.200544 0.213883
F 2.241498 1.383676 -1.097323
F 1.935556 1.460105 1.060036
F 4.419448 0.552430 0.295251
F 3.219005 -0.912711 1.333072
F 3.471645 -1.045491 -0.808659

Indole_NMe_IBCONAc_A_CF2CF3.log

Energy (E) = -1309.11368103 Hartree
Enthalpy (H) = -1308.862427 Hartree
Gibbs free energy (G) = -1308.942071 Hartree
Charge = 0, Spin = 1
C 1.484130 -1.647633 -0.116814
C 2.885988 -1.513516 0.050705
C 3.751661 -2.607626 0.023172
C 3.204206 -3.853135 -0.188502
C 1.821159 -4.006978 -0.377008
C 0.962612 -2.930287 -0.344857
C 1.008694 -0.305852 -0.022447
H 4.816073 -2.473204 0.159297
H 3.845727 -4.722895 -0.217102
H 1.419472 -4.994790 -0.555063
H -0.089393 -3.090699 -0.504354
N 3.212027 -0.190946 0.213329
C 2.066105 0.548519 0.155308
C 1.980249 2.038124 0.203425
O 2.946443 2.729642 0.460161
I -0.844917 0.649068 -0.136938
C 4.580414 0.273698 0.370462
H 5.169213 -0.022196 -0.497594
H 4.574926 1.352348 0.465390
H 5.016626 -0.172512 1.263841
N 0.698646 2.417743 -0.075350
C 0.319980 3.751595 -0.194159
C -1.175426 3.945039 -0.349134
H -1.710260 3.539303 0.512181
H -1.383228 5.007175 -0.429634
H -1.538737 3.441772 -1.247547
O 1.077664 4.692061 -0.196957
C -2.041021 -1.269425 0.005498
C -3.503218 -0.881067 0.271626
F -2.014972 -1.985680 -1.129703
F -1.627212 -2.040804 1.018759
F -4.290799 -1.942259 0.267787
F -3.601554 -0.269654 1.446301
F -3.907752 -0.036589 -0.679456

NaphIBMeUreaMe_CF2CF3.log
Energy (E) = -1273.20513424 Hartree
Enthalpy (H) = -1272.929281 Hartree
Gibbs free energy (G) = -1273.006101 Hartree
Charge = 0, Spin = 1
C 2.630589 -1.382777 -0.470762
C 3.421557 -0.459320 0.255101
C 2.849905 0.750732 0.705184
C 1.529659 1.070714 0.478108
C 0.764369 0.096429 -0.213140
C 1.270461 -1.069256 -0.692899
H 3.483552 1.452973 1.230155
H 0.647511 -1.774823 -1.225617
I -1.272137 0.534987 -0.471492
C 0.238391 3.125010 0.077924
O 0.192631 4.320805 0.318982
N -0.343544 2.505354 -0.972460
C -1.125338 3.369363 -1.836014
H -0.508030 4.186347 -2.208298
H -1.484496 2.791366 -2.688408
H -1.979621 3.821011 -1.322386
N 0.967562 2.263910 0.941985
C 1.628900 2.938317 2.047096
H 1.898512 2.200590 2.800467
H 2.523567 3.479409 1.728677
H 0.939701 3.663057 2.466631
C 4.782893 -0.771397 0.488606
C 5.318989 -1.940155 0.023192
C 4.524756 -2.859628 -0.698139
C 3.207236 -2.585412 -0.939606
H 5.391151 -0.065808 1.040620
H 6.360365 -2.167037 0.207358
H 4.963866 -3.780318 -1.056625
H 2.586285 -3.281984 -1.488886
C -1.726536 -1.652124 0.134156
C -3.163978 -1.730860 0.651409
F -1.636984 -2.489783 -0.923875
F -0.911202 -2.108455 1.098052
F -3.546923 -2.977418 0.884196
F -3.282514 -1.029211 1.773928
F -3.980088 -1.198266 -0.265013

NpthIBCMe2O_C_CF2CF3.log
Energy (E) = -1163.77254311 Hartree
Enthalpy (H) = -1163.510645 Hartree
Gibbs free energy (G) = -1163.582298 Hartree
Charge = 0, Spin = 1
C 3.007817 0.883900 0.200380
C 1.687038 1.217517 0.061940
C 0.781059 0.168689 -0.154435
C 1.130110 -1.139712 -0.256281
H 3.745751 1.658205 0.373576
H 0.416103 -1.925864 -0.448143
O -0.040021 2.675788 -0.619553
I -1.208834 0.904292 -0.344727
C 2.935159 -2.825404 -0.227150
C 4.260332 -3.134389 -0.093897
C 5.208106 -2.114582 0.146310
C 4.811110 -0.809852 0.246521
C 3.446493 -0.458464 0.114055
C 2.497961 -1.483960 -0.123120
H 2.200672 -3.599561 -0.410621
H 4.588784 -4.161775 -0.171345
H 6.253404 -2.372143 0.249972
H 5.534078 -0.024165 0.427700
C 1.126985 2.637736 0.137235
C 2.093707 3.652291 -0.459095
H 1.601598 4.623989 -0.461334
H 3.012404 3.732543 0.123447
H 2.336119 3.379878 -1.485083
C 0.847624 2.979349 1.604714
H 1.765350 2.967520 2.194570
H 0.399045 3.971659 1.653997
H 0.152402 2.261519 2.044340
C -2.017783 -1.209499 0.034187
C -3.527815 -1.114666 0.272207
F -1.835698 -2.042982 -1.014626
F -1.475449 -1.787647 1.123810
F -4.081314 -2.309267 0.419950
F -3.769080 -0.393084 1.363622
F -4.098986 -0.504722 -0.768925

NpthISO2NMe_D_CF2CF3.log
Energy (E) = -1613.75311357 Hartree
Enthalpy (H) = -1613.522808 Hartree
Gibbs free energy (G) = -1613.596661 Hartree
Charge = 0, Spin = 1
C 0.650203 3.396144 -1.071690
C 1.873117 3.445713 -0.473489
C 2.477438 2.283501 0.058967
C 1.815544 1.017757 -0.002268
C 0.505112 1.054709 -0.551006
C -0.048145 2.174938 -1.099104
H 4.222085 3.368521 0.677871
H 0.199827 4.276731 -1.506005
H 2.421154 4.376925 -0.408764
C 3.765626 2.387782 0.635338
C 2.560373 -0.111723 0.442852
H -1.030445 2.142088 -1.544817
C 3.814631 0.026094 0.973411
C 4.419678 1.287878 1.104476
H 4.348296 -0.870736 1.257330
H 5.406189 1.370020 1.537631
I -0.787219 -0.632679 -0.507188
S 2.028236 -1.802564 0.316574
O 1.212986 -2.094567 1.478967
O 3.198993 -2.624885 0.117473
N 1.165039 -1.700155 -1.045344
C 0.870945 -2.999100 -1.648844
H 0.414578 -2.827415 -2.622682
H 1.792831 -3.561361 -1.794568
H 0.191668 -3.612416 -1.043782
C -2.511609 0.781215 0.074306
C -3.566198 -0.054567 0.807649
F -2.132566 1.779147 0.884232
F -3.115327 1.318814 -1.007992
F -4.668916 0.641531 1.040068
F -3.879259 -1.108906 0.046279
F -3.082463 -0.494444 1.960286

perF_CMe2O_CF2CF3.log
Energy (E) = -1407.05220867 Hartree
Enthalpy (H) = -1406.868361 Hartree
Gibbs free energy (G) = -1406.941395 Hartree
Charge = 0, Spin = 1
C 2.156381 2.199905 -0.151838
C 3.222202 1.444628 0.304985
C 3.100804 0.065326 0.413020
C 1.924707 -0.582061 0.079729
C 0.852350 0.209821 -0.283392
C 0.954079 1.573757 -0.449097
O 0.669084 -2.353578 -0.831480
I -0.916994 -0.935842 -0.563304
F -0.031062 2.311866 -0.937608
F 2.287325 3.505321 -0.316047
F 4.364699 2.037752 0.603566
F 4.163702 -0.605153 0.852271
C 1.726215 -2.111187 0.032048
C 2.943261 -2.817351 -0.559582
H 3.800308 -2.804626 0.108996
H 2.648952 -3.849977 -0.741935
H 3.213801 -2.365121 -1.512800
C 1.447374 -2.616307 1.448770
H 1.279734 -3.692328 1.409176
H 2.290444 -2.408677 2.109310
H 0.557193 -2.140200 1.863748
C -2.226948 0.840360 0.048394
C -3.505199 0.231939 0.643421
F -1.647660 1.580874 1.007596
F -2.604214 1.634942 -0.960807
F -4.408850 1.158322 0.916004
F -4.023570 -0.626333 -0.242549
F -3.224125 -0.435794 1.756652

perF_NMeCO2_CF2CF3.log
Energy (E) = -1497.09906096 Hartree
Enthalpy (H) = -1496.943052 Hartree
Gibbs free energy (G) = -1497.016891 Hartree
Charge = 0, Spin = 1
C -2.093146 -2.283076 -0.298067
C -3.113726 -1.570783 0.312605
C -2.981716 -0.215651 0.570816
C -1.806264 0.484831 0.279033
C -0.784825 -0.280183 -0.293304
C -0.926540 -1.614910 -0.617646

I 0.971704 0.720786 -0.774707
F 0.052212 -2.273421 -1.223826
F -2.239547 -3.567611 -0.574514
F -4.246580 -2.181470 0.604857
F -4.041617 0.414156 1.064580
N -1.669499 1.843255 0.512715
C -2.330646 2.472018 1.653332
H -2.411122 1.749300 2.461887
H -1.718283 3.309796 1.972792
H -3.318705 2.849795 1.399370
C -1.250049 2.732155 -0.534908
O -1.501604 3.908263 -0.409609
O -0.630253 2.152513 -1.512348
C 2.200387 -0.904424 0.195555
C 3.347978 -0.232461 0.964892
F 1.475731 -1.624323 1.053442
F 2.735653 -1.708875 -0.723300
F 4.203761 -1.127444 1.423616
F 3.985758 0.594178 0.132245
F 2.865786 0.472072 1.978256

PyIBCMe2O_D_CF2CF3.log
Energy (E) = -1026.30203384 Hartree
Enthalpy (H) = -1026.101554 Hartree
Gibbs free energy (G) = -1026.167004 Hartree
Charge = 0, Spin = 1
C 1.874278 3.047269 -0.101564
C 3.141494 2.546743 0.160204
C 2.421522 0.378608 0.072129
C 1.129701 0.815698 -0.155505
H 1.708526 4.111639 -0.180749
H 3.976678 3.221717 0.303997
O 1.667254 -1.766275 -0.554988
I -0.224883 -0.809715 -0.309360
C 0.821231 2.154352 -0.255261
H -0.176443 2.511658 -0.453139
N 3.414729 1.246764 0.246365
C 2.682902 -1.119698 0.145782
C 4.012336 -1.457647 -0.512174
H 4.833853 -0.947400 -0.012453
H 4.157820 -2.535636 -0.454495
H 3.991318 -1.162586 -1.560323
C 2.696147 -1.525519 1.621831
H 2.866733 -2.599753 1.688258
H 3.482741 -0.998309 2.162475
H 1.737551 -1.296503 2.093255
C -1.957457 0.661960 0.007253
C -3.247130 -0.131429 0.233225
F -1.772061 1.448888 1.086525
F -2.165200 1.464190 -1.061038
F -4.301856 0.660663 0.352411
F -3.442397 -0.952887 -0.800308
F -3.135294 -0.866308 1.336753

PyrroleNMeIBCMe2O_A_CF2CF3.log
Energy (E) = -1027.49471513 Hartree
Enthalpy (H) = -1027.270737 Hartree
Gibbs free energy (G) = -1027.338894 Hartree
Charge = 0, Spin = 1
C 2.147242 2.600510 -0.040536
C 0.849686 2.169043 -0.135960
C 0.947561 0.761137 -0.142582
C 2.248772 0.370664 -0.040673
H 2.545091 3.601380 -0.027596
H -0.027954 2.784745 -0.201270
I -0.382048 -0.835789 -0.143136
O 1.546748 -1.824670 -0.355894
N 2.989585 1.516345 0.023974
C 4.432769 1.619446 0.095502
H 4.826303 0.966030 0.871753
H 4.896222 1.358173 -0.854792
H 4.690309 2.645982 0.344103
C 2.648172 -1.086699 0.068337
C 3.821515 -1.450300 -0.839674
H 4.764969 -1.023277 -0.496813
H 3.913576 -2.535823 -0.834492
H 3.621737 -1.125338 -1.860229
C 2.973895 -1.420051 1.527316
H 3.221453 -2.479351 1.598037
H 3.812090 -0.834064 1.911659
H 2.098843 -1.222698 2.148760
C -2.053032 0.688910 0.015479
C -3.416621 0.000683 0.106345
F -1.911357 1.462247 1.108504
F -2.074240 1.496561 -1.063348
F -4.408806 0.876403 0.171658
F -3.596867 -0.771688 -0.964787
F -3.454836 -0.771977 1.190763

3Obenzofurane_45_CO2I_02.log
Energy (E) = -657.083992831 Hartree
Enthalpy (H) = -656.958493 Hartree
Gibbs free energy (G) = -657.011398 Hartree
Charge = 0, Spin = 2
C -0.054539 1.028252 0.000080
C 0.343804 -0.312227 -0.000012
C -0.549228 -1.368349 0.000055
C -1.897188 -1.031933 0.000221
C -2.323229 0.293204 0.000329
C -1.410022 1.331554 0.000249
O -2.897659 -1.929121 0.000276
C -4.139472 -1.220583 0.000422
C -3.791774 0.268073 0.000533
C 0.899399 2.170041 0.000023
O 2.179473 1.922902 -0.000317
I 2.395741 -0.754339 -0.000293
O 0.539270 3.332378 -0.000137
O -4.580358 1.178323 0.000544
H -0.228953 -2.399444 -0.000029
H -1.718177 2.369518 0.000327
H -4.709666 -1.487141 0.888791
H -4.709788 -1.487004 -0.887910

3Oindole_45_CO2I_02.log
Energy (E) = -637.223144079 Hartree
Enthalpy (H) = -637.085349 Hartree
Gibbs free energy (G) = -637.139715 Hartree
Charge = 0, Spin = 2
C -0.047584 1.027289 -0.005486
C 0.352527 -0.312432 -0.004550
C -0.533656 -1.371874 -0.009617
C -1.896558 -1.061663 -0.015862
C -2.317467 0.278423 -0.008656
C -1.408849 1.315846 -0.006666
N -2.961928 -1.907714 -0.039950
C -4.215042 -1.181323 0.025133
C -3.786479 0.292306 0.004942
C 0.893585 2.171656 -0.000693
O 2.177720 1.943808 0.004037
I 2.406588 -0.754762 0.002868
O 0.532490 3.335754 -0.000662
O -4.538104 1.235210 0.003641
H -0.185798 -2.395177 -0.012133
H -1.727716 2.350862 -0.001794
H -2.885862 -2.900920 0.094804
H -4.769521 -1.379506 0.944026
H -4.866463 -1.386414 -0.824630

acridineCO2I_02.log
Energy (E) = -753.612245069 Hartree
Enthalpy (H) = -753.423526 Hartree
Gibbs free energy (G) = -753.480862 Hartree
Charge = 0, Spin = 2
C 5.378897 -0.175441 -0.000037
C 4.769117 -1.462303 0.000038
C 3.415386 -1.593752 0.000059
C 2.579954 -0.439861 0.000006
C 3.193261 0.853283 -0.000072
C 4.614137 0.948632 -0.000091
N 1.253655 -0.603153 0.000026
C 0.472666 0.472002 -0.000026
C 0.979515 1.810848 -0.000103
C 2.360511 1.966521 -0.000124
C -0.945598 0.315332 -0.000003
C -1.788855 1.377734 -0.000053
C -1.265753 2.702946 -0.000131
C 0.073959 2.912374 -0.000155
I -1.718329 -1.623877 0.000106
O -3.753112 0.008230 0.000017
C -3.277830 1.219496 -0.000027
O -4.021831 2.181928 -0.000132
H 6.457525 -0.100208 -0.000051
H 5.397509 -2.342602 0.000080
H 2.930723 -2.559986 0.000115
H 5.066609 1.932433 -0.000149
H 2.788998 2.962765 -0.000183
H -1.983496 3.511713 -0.000169
H 0.481331 3.915326 -0.000214

AnthI8BA_B_02_2.log
Energy (E) = -855.395433517 Hartree
Enthalpy (H) = -855.107029 Hartree
Gibbs free energy (G) = -855.176120 Hartree
Charge = 0, Spin = 2
C 6.737557 1.124188 0.008443
C 5.536209 1.736088 -0.168931
C 4.321218 0.992169 -0.113370
C 4.388404 -0.416455 0.133553
C 5.667421 -1.020178 0.314010
C 6.804284 -0.276665 0.253894
C 3.077477 1.594149 -0.292266
C 3.208895 -1.155414 0.189866
C 1.967740 -0.550128 0.011647
C 1.899323 0.854704 -0.234925
C 0.618043 1.446742 -0.413886
H 0.570490 2.514322 -0.602474
C -0.543603 0.732436 -0.360125
C -0.446062 -0.675800 -0.116942
H 3.026960 2.661231 -0.480018
H 7.652590 1.699171 -0.036191
H 5.478656 2.801380 -0.356363
H 5.710984 -2.086328 0.500022
H 7.769011 -0.745717 0.392172
H 3.258881 -2.222668 0.375501
I -2.188859 -1.868555 -0.054714
C 0.752076 -1.290427 0.060960
H 0.804212 -2.356797 0.238534
C -1.860597 1.441162 -0.508385
H -2.550284 0.844278 -1.109214
H -1.692415 2.380203 -1.041030
C -2.499025 1.749461 0.847628
H -2.678686 0.820663 1.391975
H -1.800894 2.339777 1.443019
C -3.806100 2.520300 0.706735

H -4.165703 2.867278 1.678153
H -3.690697 3.408372 0.081857
C -4.911879 1.702646 0.125174
O -4.927611 0.450310 0.075005
O -5.966372 2.181484 -0.354324

AzuleneCO2I_02.log
Energy (E) = -584.022206737 Hartree
Enthalpy (H) = -583.872316 Hartree
Gibbs free energy (G) = -583.925465 Hartree
Charge = 0, Spin = 2
C 1.223533 -4.143038 0.000000
C -0.052473 -3.585696 0.000000
C -0.409848 -2.243942 0.000000
C 0.409533 -1.125632 0.000000
C 1.895754 -1.088711 0.000000
C 2.765168 -2.170575 0.000000
C 2.472123 -3.528698 0.000000
C 0.000000 0.209774 0.000000
C 1.127359 1.035723 0.000000
C 2.283510 0.252877 0.000000
I -1.946384 0.937420 0.000000
O -0.027774 3.122652 0.000000
C 1.126185 2.507874 0.000000
O 2.125108 3.204833 0.000000
H 1.247789 -5.226883 0.000000
H -0.873068 -4.291601 0.000000
H -1.475432 -2.034283 0.000000
H 3.818446 -1.908435 0.000000
H 3.324522 -4.195939 0.000000
H 3.292351 0.634243 0.000000

BenzoFuraneCO2I_02.log
Energy (E) = -581.894550555 Hartree
Enthalpy (H) = -581.774356 Hartree
Gibbs free energy (G) = -581.825093 Hartree
Charge = 0, Spin = 2
C 3.218457 1.973665 0.000000
C 4.058349 0.847402 0.000000
C 3.552439 -0.439780 0.000000
C 2.171370 -0.555640 0.000000
C 1.314136 0.550902 0.000000
C 1.844925 1.842488 0.000000
O 1.465405 -1.714258 0.000000
C 0.155662 -1.358437 0.000000
C -0.000000 -0.014410 0.000000
C -0.860182 -2.412685 0.000000
O -2.107538 -2.023629 0.000000
I -1.821568 0.959218 0.000000
O -0.653111 -3.610176 0.000000
H 3.662535 2.959181 0.000000
H 5.129827 0.992126 0.000000
H 4.188421 -1.312564 0.000000
H 1.193335 2.706190 0.000000

BenzothiopheneCO2I_02.log
Energy (E) = -904.824507708 Hartree
Enthalpy (H) = -904.706575 Hartree
Gibbs free energy (G) = -904.758124 Hartree
Charge = 0, Spin = 2
C -1.820331 3.255348 0.000000
C -0.687395 4.084686 0.000000
C 0.585368 3.553623 0.000000
C 0.725036 2.165238 0.000000
C -0.405039 1.324730 0.000000
C -1.688435 1.885059 0.000000
S 2.204836 1.277182 0.000000
C 1.341754 -0.214254 0.000000
C -0.000000 -0.045264 0.000000
C 2.080760 -1.484242 0.000000
O 1.374723 -2.585950 0.000000
I -1.296080 -1.672560 0.000000
O 3.293558 -1.568184 0.000000
H -2.804974 3.701323 0.000000
H -0.815160 5.158378 0.000000
H 1.456130 4.194675 0.000000
H -2.557718 1.239918 0.000000

CyclohexeneCO2I_02.log
Energy (E) = -432.973780363 Hartree
Enthalpy (H) = -432.825111 Hartree
Gibbs free energy (G) = -432.872935 Hartree
Charge = 0, Spin = 2
C 2.443941 -1.871153 -0.379668
C 3.149976 -0.784212 0.417475
C 2.623619 0.583888 0.009890
C 1.120315 0.612588 -0.018979
C 0.403033 -0.504451 -0.017347
C 0.956894 -1.892364 -0.035204
C 0.511862 1.971764 -0.048485
O -0.787344 2.089694 -0.055436
I -1.703650 -0.383977 0.027118
O 1.193882 2.980612 -0.072678
H 2.875779 -2.852740 -0.186550
H 2.562395 -1.667482 -1.447083
H 4.228059 -0.829839 0.267634
H 2.964507 -0.943196 1.483686
H 2.997641 0.865030 -0.978695
H 2.961377 1.369423 0.686383
H 0.399793 -2.500418 -0.750037
H 0.793739 -2.348788 0.946214

DibenzofuranCO2I_02.log
Energy (E) = -735.413744494 Hartree
Enthalpy (H) = -735.243779 Hartree
Gibbs free energy (G) = -735.299690 Hartree
Charge = 0, Spin = 2
C 4.309979 -2.935298 -0.000000
C 5.282762 -1.928567 -0.000000
C 4.939120 -0.585216 -0.000000
C 3.587132 -0.294360 -0.000000
C 2.595310 -1.279113 -0.000000
C 2.961696 -2.623263 -0.000000
O 3.050482 0.961617 -0.000000
C 1.698701 0.800396 -0.000000
C 1.344092 -0.555231 -0.000000
C 0.769337 1.816065 0.000000
C -0.572796 1.450037 0.000000
C -0.935063 0.102921 0.000000
C -0.000000 -0.915007 -0.000000
C -1.567790 2.560941 0.000000
O -2.837138 2.263734 0.000000
I -2.977321 -0.394225 0.000000
O -1.244367 3.734104 0.000000
H 4.621006 -3.970549 -0.000000
H 6.328134 -2.205115 -0.000000
H 5.680907 0.199775 -0.000000
H 2.212005 -3.403344 -0.000000
H 1.031368 2.864864 0.000000
H -0.302094 -1.953211 -0.000000

FuranIBCMe2S_A_02.log
Energy (E) = -755.890638021 Hartree
Enthalpy (H) = -755.744450 Hartree
Gibbs free energy (G) = -755.794342 Hartree
Charge = 0, Spin = 2
C -0.990292 2.860034 -0.194230
C 0.296305 2.446174 -0.175691
C 0.233069 1.026940 -0.045057
C -1.076920 0.678616 0.006257
O -1.829574 1.802841 -0.081956
H -1.450551 3.826916 -0.284501
H 1.178705 3.056189 -0.252190
I 1.841488 -0.283983 0.050034
S -1.101742 -1.875602 -0.910641
C -1.777113 -0.629101 0.209819
C -1.650439 -1.073685 1.667938
H -2.094322 -0.322181 2.324527
H -2.167798 -2.022683 1.807787
H -0.604678 -1.204190 1.944527
C -3.257071 -0.514566 -0.170364
H -3.736623 -1.485846 -0.052671
H -3.746060 0.200087 0.492470
H -3.378312 -0.176746 -1.197868

IndeneCO2I_02.log
Energy (E) = -545.991107150 Hartree
Enthalpy (H) = -545.847217 Hartree
Gibbs free energy (G) = -545.898552 Hartree
Charge = 0, Spin = 2
C -3.430708 -1.506768 -0.000041
C -4.159927 -0.319024 0.000001
C -3.520646 0.917173 0.000046
C -2.137691 0.949366 0.000048
C -1.410326 -0.249701 0.000008
C -2.043200 -1.484604 -0.000035
C -1.190135 2.114892 0.000098
C 0.150518 1.442264 0.000071
C 0.000299 0.108878 0.000011
C 1.410061 2.184707 0.000085
O 2.532759 1.512076 0.000053
I 1.568712 -1.252559 -0.000063
O 1.495900 3.401571 0.000125
H -3.953289 -2.453315 -0.000077
H -5.240699 -0.360177 -0.000001
H -4.097699 1.832711 0.000081
H -1.467300 -2.401257 -0.000070
H -1.310756 2.757670 0.875278
H -1.310752 2.757738 -0.875033

Indole_NMe_IBCONAc_A_02_2.log
Energy (E) = -733.972764662 Hartree
Enthalpy (H) = -733.756960 Hartree
Gibbs free energy (G) = -733.821713 Hartree
Charge = 0, Spin = 2
C -1.934580 -0.313910 -0.019493
C -2.377902 1.027281 -0.050268
C -3.741123 1.333846 -0.141947
C -4.629038 0.283291 -0.203618
C -4.197034 -1.057933 -0.176890
C -2.860406 -1.364420 -0.087329
C -0.522544 -0.257555 0.082133
H -4.094900 2.354616 -0.171005
H -5.687530 0.492611 -0.278080
H -4.930848 -1.849996 -0.229706
H -2.517337 -2.390841 -0.068919
N -1.288137 1.853019 0.024156
C -0.151572 1.067213 0.107592
C 1.184406 1.668998 0.161078
O 1.393989 2.858648 0.025272
I 0.694962 -1.937352 0.120541
C -1.354249 3.304034 0.064999

H -2.399497 3.588695 0.141769
H -0.815290 3.688813 0.925633
H -0.922370 3.743124 -0.830511
N 2.214162 0.776508 0.452269
C 3.502565 0.919410 -0.088282
C 3.980692 -0.243632 -0.904182
H 3.296500 -0.434060 -1.730920
H 4.977698 -0.025472 -1.275537
H 3.995772 -1.138630 -0.282415
O 4.154431 1.896896 0.176386

IndoleAc_54_CO2I_02.log
Energy (E) = -714.586694015 Hartree
Enthalpy (H) = -714.412753 Hartree
Gibbs free energy (G) = -714.470867 Hartree
Charge = 0, Spin = 2
C -1.328685 0.992641 0.000000
C -2.329960 0.010608 0.000000
C -2.035473 -1.337109 0.000000
C -0.686993 -1.690629 0.000000
C 0.343532 -0.728644 0.000000
C 0.000000 0.618639 0.000000
N -0.073494 -2.944141 0.000000
C 1.309144 -2.751137 0.000000
C 1.596512 -1.432623 0.000000
I 1.506654 2.077016 0.000000
O -0.870415 3.354969 0.000000
C -1.772234 2.412580 0.000000
O -2.942926 2.748575 0.000000
O -1.934359 -4.247022 0.000000
C -0.729984 -4.182325 0.000000
C 0.157360 -5.397381 0.000000
H -3.354254 0.358576 0.000000
H -2.809115 -2.085800 0.000000
H 1.977280 -3.593462 0.000000
H 2.580184 -0.994815 0.000000
H -0.483418 -6.272320 0.000000
H 0.796700 -5.412456 0.882294
H 0.796700 -5.412456 -0.882294

IndoleAc_56_CO2I_02.log
Energy (E) = -714.585769526 Hartree
Enthalpy (H) = -714.411857 Hartree
Gibbs free energy (G) = -714.469921 Hartree
Charge = 0, Spin = 2
C 1.386707 0.791243 0.000000
C -0.000000 0.626619 0.000000
C -0.612223 -0.611252 0.000000
C 0.233608 -1.717010 0.000000
C 1.637025 -1.588820 0.000000
N -0.059898 -3.080473 0.000000
C 1.152097 -3.780484 0.000000
C 2.189244 -2.918631 0.000000
O 3.261586 2.259114 0.000000
C 2.051280 2.125302 0.000000
O 1.306178 3.195210 0.000000
O -2.325255 -2.948972 0.000000
C -1.338956 -3.645562 0.000000
C -1.396097 -5.148816 0.000000
H -1.683430 -0.724023 0.000000
H 1.156998 -4.855785 0.000000
H 3.232913 -3.181996 0.000000
H -2.441016 -5.439153 0.000000
H -0.903045 -5.556529 0.882113
H -0.903045 -5.556529 -0.882113
I -1.213846 2.342408 0.000000
C 2.209545 -0.323700 0.000000
H 3.280309 -0.166423 0.000000

NaphIBMeUreaMe_02.log
Energy (E) = -698.075540812 Hartree
Enthalpy (H) = -697.835685 Hartree
Gibbs free energy (G) = -697.899986 Hartree
Charge = 0, Spin = 2
C -2.559093 -0.608144 0.159803
C -2.608060 0.775259 -0.140991
C -1.401948 1.451323 -0.425939
C -0.195575 0.805972 -0.417041
C -0.163011 -0.583642 -0.131010
C -1.307801 -1.268764 0.155158
H -1.421855 2.512455 -0.643788
H -1.274813 -2.327238 0.378186
I 1.676418 -1.609416 -0.167107
C 1.917172 1.868806 0.181439
O 2.972105 2.419613 -0.084768
N 1.486050 1.585278 1.486753
C 2.552883 1.389965 2.414943
H 3.414135 2.016254 2.170628
H 2.205385 1.560167 3.430783
H 2.890218 0.346368 2.340941
N 0.983029 1.523233 -0.759179
C 1.286746 1.680865 -2.169400
H 1.401630 0.705181 -2.647882
H 0.483801 2.224377 -2.668311
H 2.215258 2.237063 -2.254781
C -3.858861 1.436562 -0.138678
C -5.006835 0.750929 0.147883
C -4.957802 -0.629256 0.445953
C -3.762262 -1.293204 0.451784
H -3.888057 2.494864 -0.366399
H -5.959229 1.263273 0.148257
H -5.873293 -1.159356 0.670792
H -3.717044 -2.350842 0.679914

NpthIBCMe2O_C_02.log
Energy (E) = -588.630273378 Hartree
Enthalpy (H) = -588.403154 Hartree
Gibbs free energy (G) = -588.459318 Hartree
Charge = 0, Spin = 2
C 1.317959 1.446028 -0.029443
C 0.024536 0.991985 -0.036027
C -0.175456 -0.413100 -0.020574
C 0.870217 -1.290082 0.001480
H 1.515970 2.510503 -0.045760
H 0.693820 -2.358045 0.011921
O -2.159288 1.520559 -0.864226
I -2.123530 -1.227529 -0.021461
C 3.310716 -1.702743 0.024304
C 4.587425 -1.211907 0.027615
C 4.815736 0.182115 0.011787
C 3.761941 1.054122 -0.006920
C 2.431191 0.574467 -0.009562
C 2.204370 -0.821280 0.006273
H 3.127125 -2.769997 0.035201
H 5.429500 -1.890503 0.041650
H 5.830385 0.556589 0.014052
H 3.929641 2.124192 -0.020385
C -1.166569 1.943230 -0.008493
C -0.818529 3.331731 -0.582341
H -1.735971 3.911570 -0.658430
H -0.138641 3.847099 0.095183
H -0.363765 3.239603 -1.565977
C -1.674308 2.120159 1.425927
H -0.894104 2.558840 2.048915
H -2.545024 2.775612 1.417916
H -1.962915 1.160753 1.852808

NpthISO2NMe_D_02.log
Energy (E) = -1038.62044493 Hartree
Enthalpy (H) = -1038.425559 Hartree
Gibbs free energy (G) = -1038.484409 Hartree
Charge = 0, Spin = 2
C 2.822597 -2.350394 -0.466260
C 3.482396 -1.195368 -0.170445
C 2.769780 -0.002763 0.081288
C 1.341087 0.037819 0.008696
C 0.696564 -1.221769 -0.165772
C 1.419757 -2.359984 -0.423901
H 4.588052 1.060183 0.488371
H 3.358096 -3.264622 -0.679290
H 4.563196 -1.163405 -0.122293
C 3.511611 1.156087 0.420080
C 0.757498 1.343680 0.124640
H 0.897328 -3.296171 -0.558946
C 1.510406 2.440178 0.455946
C 2.899044 2.347893 0.654838
H 1.019636 3.400864 0.503940
H 3.464793 3.226589 0.928552
I -1.344601 -1.635037 0.210811
S -0.863547 1.789827 -0.442662
O -1.139434 1.046570 -1.644840
O -0.948394 3.232782 -0.487949
N -1.861585 1.381692 0.815952
C -3.251894 1.430187 0.429241
H -3.851212 0.977142 1.214778
H -3.545247 2.480973 0.324578
H -3.433386 0.938121 -0.529550

perF_CMe2O_02.log
Energy (E) = -831.916254528 Hartree
Enthalpy (H) = -831.767225 Hartree
Gibbs free energy (G) = -831.825507 Hartree
Charge = 0, Spin = 2
C -2.065368 -1.516574 -0.029586
C -2.676302 -0.279485 0.018253
C -1.898610 0.869933 0.015646
C -0.514276 0.832287 -0.058326
C 0.083657 -0.430497 -0.067587
C -0.680834 -1.583046 -0.058037
O 1.484043 1.909858 -0.795938
I 2.164594 -0.686464 0.020130
F -0.123053 -2.787178 -0.066548
F -2.791536 -2.622039 -0.029160
F -3.995405 -0.191161 0.071210
F -2.558779 2.025637 0.110879
C 0.347462 2.099905 -0.048910
C -0.290998 3.294412 -0.798217
H -1.053420 3.760153 -0.180966
H 0.499310 4.017437 -0.991793
H -0.721972 2.973245 -1.743228
C 0.649358 2.500630 1.394981
H 1.264946 3.399625 1.395268
H -0.279868 2.698388 1.931148
H 1.189594 1.702137 1.903481

perF_NMeCO2_02.log
Energy (E) = -921.954883791 Hartree
Enthalpy (H) = -921.834892 Hartree
Gibbs free energy (G) = -921.896363 Hartree
Charge = 0, Spin = 2
C 2.499000 -0.957398 0.119483
C 2.749428 0.397232 -0.020759
C 1.683523 1.269095 -0.172146

C 0.374171 0.807387 -0.192129
C 0.129079 -0.561347 -0.063184
C 1.193322 -1.430118 0.097697
I -1.814762 -1.317140 -0.128527
F 1.006615 -2.734930 0.225807
F 3.505161 -1.799402 0.266731
F 3.991068 0.847881 -0.004589
F 1.927715 2.564658 -0.304322
N -0.668459 1.747592 -0.362055
C -1.013596 2.228512 -1.689443
H -1.335005 1.398881 -2.320160
H -1.831741 2.937975 -1.589116
H -0.159607 2.729134 -2.143916
C -1.359328 2.139982 0.729956
O -2.326226 2.929507 0.718809
O -1.076362 1.726165 1.892439

PhenanthrolineCO2I_02.log
Energy (E) = -769.638917351 Hartree
Enthalpy (H) = -769.462211 Hartree
Gibbs free energy (G) = -769.521024 Hartree
Charge = 0, Spin = 2
C -0.544337 2.587649 -0.000168
C 0.157986 3.763035 0.000091
C 1.559148 3.699622 0.000526
N 2.230236 2.570923 0.000507
C 1.554681 1.410547 0.000162
C 0.144067 1.361057 -0.000005
C 2.316105 0.173903 -0.000061
C 1.643779 -1.067125 0.000095
C 0.191212 -1.098446 0.000174
C -0.490326 0.076579 0.000012
N 3.654363 0.284852 -0.000473
C 4.377333 -0.811696 -0.000602
C 3.816702 -2.094324 -0.000264
C 2.451229 -2.224597 0.000032
C -0.551471 -2.405185 0.000342
O -1.854178 -2.376827 -0.000235
I -2.603581 0.032708 -0.000142
O -0.024482 -3.502639 0.000980
H -1.625314 2.603101 -0.000456
H -0.347153 4.717957 -0.000041
H 2.144972 4.612290 0.000900
H 5.454367 -0.683337 -0.001013
H 4.453663 -2.967526 -0.000254
H 1.989676 -3.196811 0.000215

PyIBCMe2O_D_02.log
Energy (E) = -451.163458306 Hartree
Enthalpy (H) = -450.997919 Hartree
Gibbs free energy (G) = -451.048115 Hartree
Charge = 0, Spin = 2
C -1.500823 -2.785469 0.019771
C -2.589123 -1.929143 0.048218
C -1.256220 -0.053245 -0.032235
C -0.109045 -0.848087 -0.037880
H -1.637479 -3.857160 0.033420
H -3.599588 -2.317591 0.091588
O -0.180958 1.935081 -0.836115
I 1.827414 -0.021544 -0.013867
C -0.232354 -2.227182 -0.015255
H 0.648322 -2.854337 -0.021437
N -2.465628 -0.605527 0.023932
C -1.184313 1.471456 -0.022553
C -2.450661 2.071405 -0.664351
H -3.310589 1.757282 -0.077505
H -2.364549 3.155859 -0.662234
H -2.564945 1.710790 -1.683750
C -1.053369 1.970532 1.417709
H -1.010357 3.059164 1.416966
H -1.909887 1.640509 2.006095
H -0.141347 1.583750 1.872671

pyreneCO2I_02.log
Energy (E) = -813.743309259 Hartree
Enthalpy (H) = -813.528919 Hartree
Gibbs free energy (G) = -813.589589 Hartree
Charge = 0, Spin = 2
C 1.955995 -3.335286 0.000181
C 0.597131 -3.594840 0.000084
C -0.312678 -2.550166 -0.000013
C 0.113478 -1.220688 0.000003
C 1.505883 -0.944933 0.000078
C 2.428505 -2.023162 0.000166
C 1.976014 0.396842 -0.000002
C -0.773780 -0.090643 -0.000018
C -0.360596 1.203019 0.000045
C 1.067465 1.489828 -0.000076
C 1.590756 2.787470 -0.000373
C 2.961331 3.005780 -0.000458
C 3.848584 1.946559 -0.000263
C 3.374236 0.636109 -0.000053
C 4.282491 -0.472120 0.000092
C 3.833470 -1.743716 0.000210
I -2.848404 -0.496458 -0.000120
O -2.637312 2.005574 -0.000093
C -1.372027 2.317326 0.000353
O -1.100825 3.504281 0.001029
H 2.671207 -4.148253 0.000254
H 0.238826 -4.614847 0.000063
H -1.368875 -2.777238 -0.000084
H 0.918003 3.626830 -0.000503
H 3.331328 4.021997 -0.000678
H 4.917900 2.117889 -0.000314
H 5.343818 -0.256903 0.000090
H 4.520728 -2.580325 0.000315

PyrroleNMeIBCMe2O_A_02.log
Energy (E) = -452.353357008 Hartree
Enthalpy (H) = -452.164076 Hartree
Gibbs free energy (G) = -452.216098 Hartree
Charge = 0, Spin = 2
C 1.473589 2.395152 0.008022
C 0.112777 2.259995 -0.027288
C -0.139304 0.871554 -0.073239
C 1.058206 0.197179 -0.074446
H 2.091103 3.276809 0.042982
H -0.615815 3.051383 -0.020915
I -2.021957 -0.015435 -0.003500
O 0.212202 -1.917718 -0.700639
N 2.047983 1.151534 -0.013848
C 3.479882 0.954390 0.067718
H 3.738063 0.288856 0.891117
H 3.887012 0.549610 -0.857278
H 3.939979 1.921263 0.255319
C 1.236842 -1.303040 -0.016023
C 2.463173 -1.828958 -0.798170
H 3.373396 -1.664439 -0.225546
H 2.337130 -2.901627 -0.935121
H 2.535348 -1.349991 -1.772014
C 1.300340 -1.778608 1.436634
H 1.439961 -2.859388 1.464622
H 2.123802 -1.294292 1.965680
H 0.367201 -1.525106 1.939445

thiazoleCO2I_02.log
Energy (E) = -767.345188474 Hartree
Enthalpy (H) = -767.290426 Hartree
Gibbs free energy (G) = -767.336583 Hartree
Charge = 0, Spin = 2
C -0.284229 -0.528625 -0.000179
C -1.418375 0.232892 -0.000029
N -2.595431 -0.464924 0.000137
C -2.373464 -1.737001 -0.000237
S -0.709839 -2.176919 0.000901
I 1.696158 0.078600 -0.000281
O -0.459824 2.445504 -0.000156
C -1.467581 1.700948 -0.000183
O -2.535349 2.354378 0.000577
H -3.147643 -2.488987 -0.000086

triazoleCO2I_02.log
Energy (E) = -479.843433954 Hartree
Enthalpy (H) = -479.755336 Hartree
Gibbs free energy (G) = -479.803782 Hartree
Charge = 0, Spin = 2
C -0.000000 0.543256 0.000000
C 1.366971 0.707141 0.000000
N 1.637914 2.036651 0.000000
N 0.530439 2.690028 0.000000
N -0.479576 1.798998 0.000000
I -1.205400 -1.128565 0.000000
O 2.229670 -1.528348 0.000000
C 2.412161 -0.308536 0.000000
O 3.652977 0.000670 0.000000
C -1.852355 2.259398 0.000000
H -1.817695 3.343901 0.000000
H -2.369691 1.902095 0.887842
H -2.369691 1.902095 -0.887842

3Obenzofurane_45_CO2I_Cl.log
Energy (E) = -1117.21010924 Hartree
Enthalpy (H) = -1117.080586 Hartree
Gibbs free energy (G) = -1117.135689 Hartree
Charge = 0, Spin = 1
C -0.475036 1.234107 0.000080
C 0.052986 -0.048707 0.000017
C -0.664678 -1.219746 0.000115
C -2.045410 -1.036649 0.000295
C -2.626865 0.230233 0.000379
C -1.854819 1.378590 0.000263
O -2.930950 -2.045739 0.000355
C -4.249334 -1.493044 0.000488
C -4.083873 0.025627 0.000619
C 0.435203 2.418878 -0.000038
O 1.715015 2.090678 -0.000191
I 2.166680 0.018034 -0.000261
Cl 2.329338 -2.463176 -0.000415
O 0.026452 3.551303 0.000037
O -4.973225 0.836684 0.000571
H -0.208370 -2.198118 0.000045
H -2.286109 2.371491 0.000328
H -4.782785 -1.827232 0.888794
H -4.782908 -1.827098 -0.887794

3Oindole_45_CO2I_Cl.log
Energy (E) = -1097.35039434 Hartree
Enthalpy (H) = -1097.208493 Hartree
Gibbs free energy (G) = -1097.264875 Hartree
Charge = 0, Spin = 1
C -0.468193 1.232229 -0.005070
C 0.056841 -0.051114 -0.005871

C -0.653721 -1.223625 -0.011477
C -2.045874 -1.064423 -0.016206
C -2.621777 0.219230 -0.007341
C -1.851841 1.364674 -0.005138
N -3.002886 -2.029544 -0.039309
C -4.333813 -1.458119 0.022296
C -4.084167 0.055101 0.005942
C 0.436752 2.413841 -0.000270
O 1.722012 2.088362 0.000936
I 2.171151 0.023788 0.002085
Cl 2.346392 -2.470921 0.003718
O 0.035874 3.549382 0.000759
O -4.939899 0.903704 0.006261
H -0.172882 -2.190786 -0.014947
H -2.291423 2.354204 0.000544
H -2.807626 -3.008138 0.083212
H -4.863990 -1.724106 0.938314
H -4.952706 -1.737832 -0.830529

acridineCO2I_Cl.log
Energy (E) = -1213.72474152 Hartree
Enthalpy (H) = -1213.532354 Hartree
Gibbs free energy (G) = -1213.594332 Hartree
Charge = 0, Spin = 1
C -5.474964 -0.111622 -0.000068
C -4.812150 -1.372942 -0.000155
C -3.454244 -1.448636 -0.000154
C -2.674089 -0.257109 -0.000062
C -3.338149 1.010317 0.000031
C -4.761564 1.046022 0.000023
N -1.344197 -0.365899 -0.000052
C -0.605264 0.730501 0.000047
C -1.160649 2.054398 0.000105
C -2.547682 2.154026 0.000108
C 0.815011 0.652934 0.000036
C 1.617081 1.740488 0.000021
C 1.044642 3.042939 0.000076
C -0.302895 3.192503 0.000135
I 1.947292 -1.165985 0.000011
O 3.493135 0.340021 -0.000042
C 3.110800 1.590374 -0.000104
Cl 0.290119 -3.019954 0.000118
O 3.845066 2.548533 -0.000261
H -6.555911 -0.082987 -0.000074
H -5.404590 -2.277738 -0.000228
H -2.920627 -2.389456 -0.000216
H -5.257341 2.008662 0.000092
H -3.012978 3.133585 0.000162
H 1.734983 3.875262 0.000050
H -0.753569 4.176784 0.000187

AnthI8BA_B_Cl.log
Energy (E) = -1315.50135658 Hartree
Enthalpy (H) = -1315.207205 Hartree
Gibbs free energy (G) = -1315.275694 Hartree
Charge = 0, Spin = 1
C 6.849021 0.669716 -0.032653
C 5.752364 1.429153 0.229206
C 4.440382 0.883886 0.106989
C 4.300094 -0.482856 -0.299146
C 5.475645 -1.245517 -0.563780
C 6.709090 -0.689208 -0.435220
C 3.301554 1.640894 0.371831
C 3.025776 -1.029072 -0.422936
C 1.891623 -0.265821 -0.158365
C 2.026680 1.094674 0.246845
C 0.848576 1.844060 0.509896
H 0.965393 2.876158 0.821159
C -0.420030 1.345563 0.390130
C -0.497053 -0.024250 -0.006879
H 3.410837 2.674874 0.679284
H 7.839096 1.094196 0.063568
H 5.853329 2.462881 0.535750
H 5.360708 -2.278110 -0.869043
H 7.594521 -1.275986 -0.638105
H 2.914439 -2.063984 -0.725351
I -2.357284 -1.040702 -0.224424
C 0.581093 -0.801397 -0.272783
H 0.468855 -1.840332 -0.552583
C -1.572716 2.283915 0.659103
H -1.938257 2.671142 -0.294590
H -1.142233 3.142079 1.173042
C -2.744429 1.732368 1.492739
H -2.445367 0.799183 1.970776
H -2.936033 2.421188 2.313426
C -4.065827 1.555874 0.714988
H -4.576738 0.644924 1.034981
H -4.742282 2.382960 0.911675
C -3.891937 1.532899 -0.792578
O -3.035290 0.649531 -1.305281
O -4.460921 2.315382 -1.511159
Cl -1.398822 -3.007209 1.033981

AzuleneCO2I_Cl.log
Energy (E) = -1044.13987638 Hartree
Enthalpy (H) = -1043.985434 Hartree
Gibbs free energy (G) = -1044.040771 Hartree
Charge = 0, Spin = 1
C 4.501778 -0.239289 0.000000
C 3.490483 -1.191829 0.000000
C 2.111281 -1.015043 0.000000
C 1.385604 0.167907 0.000000
C 1.935454 1.553509 0.000000
C 3.274039 1.934359 0.000000
C 4.415940 1.150555 0.000000
C 0.000000 0.361541 0.000000
C -0.313481 1.716146 0.000000
C 0.866080 2.453342 0.000000
I -1.745118 -0.794390 0.000000
O -2.575144 1.165329 0.000000
C -1.720616 2.180199 0.000000
Cl -0.632432 -3.046798 0.000000
O -2.050896 3.338786 0.000000
H 5.509452 -0.638820 0.000000
H 3.820668 -2.222824 0.000000
H 1.524640 -1.923418 0.000000
H 3.431430 3.008365 0.000000
H 5.357970 1.683715 0.000000
H 0.927420 3.529925 0.000000

BenzoFuraneCO2I_Cl.log
Energy (E) = -1042.01091636 Hartree
Enthalpy (H) = -1041.886213 Hartree
Gibbs free energy (G) = -1041.938560 Hartree
Charge = 0, Spin = 1
C 3.660567 1.342395 0.000000
C 3.395429 2.719524 0.000000
C 2.100627 3.203388 0.000000
C 1.089075 2.258881 0.000000
C 1.326463 0.871881 0.000000
C 2.645332 0.407029 0.000000
O -0.245120 2.541753 0.000000
C -0.876440 1.356188 0.000000
C 0.000000 0.333349 0.000000
C -2.344818 1.169708 0.000000
O -2.627023 -0.124736 0.000000
I -0.966670 -1.490132 0.000000
O -3.151476 2.059982 0.000000
Cl 1.164968 -2.755585 0.000000
H 4.687718 1.005758 0.000000
H 4.221294 3.417075 0.000000
H 1.873008 4.259079 0.000000
H 2.858565 -0.650027 0.000000

BenzothiopheneCO2I_Cl.log
Energy (E) = -1364.94092828 Hartree
Enthalpy (H) = -1364.818760 Hartree
Gibbs free energy (G) = -1364.872691 Hartree
Charge = 0, Spin = 1
C 3.789806 0.479476 0.000000
C 3.894137 1.877145 0.000000
C 2.763606 2.664648 0.000000
C 1.517088 2.040351 0.000000
C 1.396281 0.630761 0.000000
C 2.563583 -0.147404 0.000000
S -0.006379 2.856314 0.000000
C -0.836419 1.364572 0.000000
C -0.000000 0.307359 0.000000
C -2.313397 1.237125 0.000000
O -2.684815 -0.027947 0.000000
I -1.117948 -1.481169 0.000000
O -3.058026 2.183674 0.000000
Cl 0.809172 -3.053644 0.000000
H 4.689061 -0.120353 0.000000
H 4.870226 2.341998 0.000000
H 2.832608 3.743892 0.000000
H 2.500115 -1.222671 0.000000

CyclohexeneCO2I_Cl.log
Energy (E) = -893.103497163 Hartree
Enthalpy (H) = -892.950870 Hartree
Gibbs free energy (G) = -893.001084 Hartree
Charge = 0, Spin = 1
C 1.991172 2.459576 0.395360
C 3.026445 1.689951 -0.415081
C 3.013831 0.217842 -0.026487
C 1.611459 -0.311743 0.015312
C 0.576679 0.507844 0.029285
C 0.569855 1.988903 0.066158
C 1.409069 -1.795977 0.029156
O 0.144053 -2.164700 0.044065
I -1.262808 -0.579320 -0.000691
Cl -2.608496 1.520400 -0.069723
O 2.341765 -2.561171 0.029167
H 2.059504 3.530846 0.211320
H 2.178816 2.299530 1.460045
H 4.020468 2.109153 -0.264648
H 2.793676 1.786430 -1.479258
H 3.469840 0.064506 0.955382
H 3.584448 -0.401444 -0.718853
H -0.142878 2.353752 0.805230
H 0.231790 2.382995 -0.895377

DibenzofuranCO2I_Cl.log
Energy (E) = -1195.54225554 Hartree
Enthalpy (H) = -1195.368275 Hartree
Gibbs free energy (G) = -1195.426444 Hartree
Charge = 0, Spin = 1
C 0.439247 -5.327365 -0.000000
C -0.892997 -5.757436 -0.000000
C -1.948163 -4.857611 -0.000000
C -1.612607 -3.516448 -0.000000
C -0.292246 -3.059821 -0.000000

C 0.754034 -3.979770 -0.000000
O -2.503783 -2.479261 -0.000000
C -1.767252 -1.338037 -0.000000
C -0.390910 -1.618140 -0.000000
C -2.279009 -0.057106 -0.000000
C -1.355293 0.978753 0.000000
C 0.000000 0.687201 0.000000
C 0.535649 -0.577687 -0.000000
C -1.809312 2.404711 0.000000
O -0.812770 3.270060 0.000000
I 1.150439 2.459310 0.000000
Cl 3.286250 1.177688 0.000000
O -2.973951 2.713859 0.000000
H 1.231871 -6.062305 -0.000000
H -1.105227 -6.817706 -0.000000
H -2.979511 -5.178023 -0.000000
H 1.784240 -3.649812 -0.000000
H -3.335695 0.170101 0.000000
H 1.602002 -0.751092 -0.000000

FuranIBCMe2S_A_Cl.log
Energy (E) = -1215.99468709 Hartree
Enthalpy (H) = -1215.845077 Hartree
Gibbs free energy (G) = -1215.897833 Hartree
Charge = 0, Spin = 1
C -0.794325 2.995036 -0.202123
C 0.387367 2.335067 -0.238596
C 0.013393 0.966909 -0.159043
C -1.328701 0.882881 -0.064297
O -1.840367 2.128549 -0.091027
H -1.047591 4.037760 -0.257430
H 1.380324 2.739169 -0.314377
I 1.084621 -0.797327 -0.055360
S -1.250266 -1.737580 -0.476850
C -2.182702 -0.313224 0.185643
C -2.428184 -0.466567 1.685912
H -2.954960 0.409006 2.070743
H -3.031653 -1.354243 1.873122
H -1.483381 -0.569492 2.220586
C -3.501148 -0.229977 -0.576738
H -4.081465 -1.137697 -0.412524
H -4.078719 0.620926 -0.212593
H -3.327260 -0.110720 -1.644450
Cl 3.227812 0.669164 0.246135

IndeneCO2I_Cl.log
Energy (E) = -1006.10789134 Hartree
Enthalpy (H) = -1005.959604 Hartree
Gibbs free energy (G) = -1006.012897 Hartree
Charge = 0, Spin = 1
C 3.507348 -1.642996 0.000150
C 4.346933 -0.533384 0.000122
C 3.820849 0.753727 0.000051
C 2.448131 0.914109 0.000017
C 1.601310 -0.213012 0.000066
C 2.126461 -1.499422 0.000125
C 1.641860 2.183072 -0.000008
C 0.240024 1.674777 0.000040
C 0.251716 0.341332 0.000049
C -1.015387 2.453192 0.000013
O -2.082594 1.668136 0.000133
I -1.701816 -0.427822 0.000069
Cl -1.029808 -2.828730 -0.000537
O -1.053429 3.658858 0.000032
H 3.935050 -2.635958 0.000200
H 5.419162 -0.674042 0.000147
H 4.474427 1.616360 0.000030
H 1.483540 -2.364348 0.000123
H 1.831743 2.808262 -0.875374
H 1.831780 2.808365 0.875272

Indole_NMe_IBCONAc_A_Cl.log
Energy (E) = -1194.09248844 Hartree
Enthalpy (H) = -1193.872307 Hartree
Gibbs free energy (G) = -1193.938426 Hartree
Charge = 0, Spin = 1
C 2.020926 -0.248191 -0.000123
C 2.583879 1.054630 0.000067
C 3.963825 1.266902 -0.000090
C 4.778442 0.156904 -0.000582
C 4.238241 -1.141488 -0.000961
C 2.879084 -1.359089 -0.000744
C 0.620417 -0.003556 0.000224
H 4.371354 2.268729 0.000152
H 5.852261 0.284043 -0.000730
H 4.908290 -1.989862 -0.001443
H 2.478778 -2.359913 -0.001119
N 1.585556 1.997157 0.000335
C 0.386366 1.343038 0.000399
C -0.964232 1.938114 0.000166
O -1.177075 3.129675 0.000173
I -1.116792 -1.129728 0.000087
C 1.825754 3.429253 0.000629
H 2.394787 3.707686 -0.886044
H 0.872972 3.944977 -0.000052
H 2.393460 3.707642 0.888172
N -1.910395 0.921430 -0.000095
C -3.291935 1.156001 -0.000072
C -4.139822 -0.097055 -0.001364
H -3.945532 -0.703773 0.885252
H -5.182972 0.201096 -0.002036
H -3.944127 -0.703008 -0.888194
O -3.783079 2.254939 -0.000092
Cl 0.090596 -3.350697 0.000811

IndoleAc_54_CO2I_Cl.log
Energy (E) = -1174.70688006 Hartree
Enthalpy (H) = -1174.528769 Hartree
Gibbs free energy (G) = -1174.590015 Hartree
Charge = 0, Spin = 1
C -0.891169 1.536767 0.000000
C -2.265779 1.282923 0.000000
C -2.753016 -0.005032 0.000000
C -1.824299 -1.043293 0.000000
C -0.418878 -0.838134 0.000000
C -0.000000 0.489200 0.000000
N -2.039235 -2.419895 0.000000
C -0.797751 -3.050402 0.000000
C 0.196914 -2.140477 0.000000
I 1.996771 1.254841 0.000000
O 0.900954 3.058901 0.000000
C -0.414340 2.953572 0.000000
Cl 3.245035 -0.919718 0.000000
O -1.164251 3.896094 0.000000
O -4.314096 -2.437682 0.000000
C -3.284996 -3.066147 0.000000
C -3.242364 -4.569871 0.000000
H -2.920596 2.143679 0.000000
H -3.809677 -0.210890 0.000000
H -0.728289 -4.123209 0.000000
H 1.246531 -2.358870 0.000000
H -4.266481 -4.926626 0.000000
H -2.724051 -4.944665 0.882198
H -2.724051 -4.944665 -0.882198

IndoleAc_56_CO2I_Cl_2.log
Energy (E) = -1174.71279281 Hartree
Enthalpy (H) = -1174.534895 Hartree
Gibbs free energy (G) = -1174.595339 Hartree
Charge = 0, Spin = 1
C 0.550212 1.682959 0.000046
C 0.415387 0.301865 0.000046
C -0.771060 -0.387433 -0.000009
C -1.904118 0.425583 -0.000037
C -1.829081 1.835967 -0.000008
N -3.254390 0.081364 -0.000199
C -3.999003 1.263617 -0.000033
C -3.178594 2.334702 0.000027
O 2.080573 3.496455 0.000044
C 1.911578 2.302316 -0.000024
O 2.887387 1.415698 -0.000044
O -3.035819 -2.177209 0.000161
C -3.771396 -1.221848 -0.000306
C -5.271090 -1.335239 0.000152
H -0.829484 -1.463610 -0.000079
H -5.073739 1.226796 -0.000030
H -3.482303 3.367230 0.000088
H -5.521355 -2.390444 0.000436
H -5.697373 -0.858488 -0.882159
H -5.696914 -0.858136 0.882498
I 2.311589 -0.635285 0.000013
C -0.589459 2.466298 0.000019
H -0.472714 3.542004 0.000020
Cl 1.306060 -2.903355 -0.000035

NaphIBMeUreaMe_Cl.log
Energy (E) = -1158.18859629 Hartree
Enthalpy (H) = -1157.943499 Hartree
Gibbs free energy (G) = -1158.006846 Hartree
Charge = 0, Spin = 1
C -2.645183 -0.502688 0.309127
C -2.821864 0.863485 -0.019649
C -1.695625 1.662236 -0.316812
C -0.415861 1.157827 -0.303145
C -0.289458 -0.215101 0.024593
C -1.333188 -1.028490 0.320647
H -1.854426 2.704638 -0.558616
H -1.172466 -2.078723 0.526369
I 1.669596 -0.967660 0.013369
C 1.848328 2.023292 0.114448
O 2.557560 3.012156 0.094304
N 2.085444 0.927639 0.896530
C 3.292083 0.978214 1.705053
H 3.302950 1.907825 2.269803
H 3.285786 0.141493 2.402663
H 4.205450 0.947399 1.105450
N 0.685373 1.951348 -0.665359
C 0.440016 3.118633 -1.498950
H -0.260342 2.847194 -2.285676
H 0.040845 3.958701 -0.926254
H 1.382914 3.433109 -1.933349
C -4.134313 1.393768 -0.034429
C -5.207759 0.599815 0.261501
C -5.027484 -0.764076 0.583481
C -3.771605 -1.304062 0.605824
H -4.271741 2.438577 -0.283532
H -6.206201 1.015424 0.247626
H -5.887901 -1.378295 0.810177
H -3.618428 -2.348207 0.846878
Cl 0.781190 -3.202966 -0.930167

NpthIBCMe2O_C_Cl.log
Energy (E) = -1048.75577213 Hartree
Enthalpy (H) = -1048.524754 Hartree

Gibbs free energy (G) = -1048.583099 Hartree
Charge = 0, Spin = 1
C 1.697961 1.551023 0.029931
C 0.371058 1.221294 -0.009307
C 0.053409 -0.145418 -0.070455
C 0.956690 -1.153931 -0.081222
H 1.998372 2.591028 0.083639
H 0.647874 -2.190343 -0.113769
O -1.896274 1.611975 -0.563941
I -2.042395 -0.407176 -0.148579
C 3.338831 -1.805779 -0.070714
C 4.660186 -1.454443 -0.040505
C 5.033860 -0.093582 0.013109
C 4.079851 0.886309 0.035449
C 2.704687 0.555077 0.005332
C 2.332381 -0.812374 -0.047388
H 3.040828 -2.845678 -0.111371
H 5.425265 -2.218455 -0.057028
H 6.082282 0.170854 0.036751
H 4.361778 1.931157 0.076069
C -0.787720 2.202013 0.075135
C -0.506565 3.496841 -0.669525
H -1.403071 4.114422 -0.639619
H 0.308819 4.050495 -0.203169
H -0.255469 3.287352 -1.707650
C -1.098453 2.485654 1.544476
H -0.248198 2.961460 2.033911
H -1.964746 3.144407 1.604183
H -1.322619 1.561051 2.079402
Cl -1.780649 -2.909959 0.295234

NpthISO2NMe_D_Cl.log
Energy (E) = -1498.73498032 Hartree
Enthalpy (H) = -1498.535580 Hartree
Gibbs free energy (G) = -1498.596628 Hartree
Charge = 0, Spin = 1
C 0.283249 3.508211 -0.600469
C 1.613028 3.302239 -0.388923
C 2.117899 2.012328 -0.110138
C 1.245542 0.881280 -0.037328
C -0.131867 1.174330 -0.229824
C -0.604194 2.420404 -0.512048
H 4.127713 2.744675 0.039334
H -0.104811 4.491165 -0.824132
H 2.316663 4.123483 -0.434084
C 3.510313 1.857029 0.092525
C 1.871912 -0.377765 0.195662
H -1.664517 2.583923 -0.639340
C 3.224580 -0.493328 0.368763
C 4.061438 0.634933 0.335844
H 3.640750 -1.481584 0.508431
H 5.125780 0.521929 0.482455
I -1.639527 -0.329869 -0.055638
S 1.019898 -1.924144 0.282313
O 0.456580 -2.063712 1.603374
O 1.893743 -2.963189 -0.201096
N -0.130023 -1.648760 -0.852933
C -0.721227 -2.857423 -1.432713
H -1.349947 -2.557803 -2.269142
H 0.078321 -3.493490 -1.806302
H -1.318356 -3.432271 -0.718494
Cl -3.352441 1.420732 0.750428

perF_CMe2O_Cl.log
Energy (E) = -1292.02780846 Hartree
Enthalpy (H) = -1291.874724 Hartree
Gibbs free energy (G) = -1291.935287 Hartree
Charge = 0, Spin = 1
C -1.964743 -1.845007 -0.114444
C -2.825594 -0.785472 0.106350
C -2.329567 0.510323 0.158469
C -0.981135 0.766151 -0.000782
C -0.134725 -0.321737 -0.147108
C -0.598569 -1.618590 -0.243665
O 0.871551 2.033533 -0.707578
I 1.879233 0.312294 -0.161283
F 0.168935 -2.656082 -0.506067
F -2.441295 -3.072741 -0.218391
F -4.120637 -1.007873 0.239119
F -3.200907 1.492834 0.376406
C -0.347928 2.161394 -0.023518
C -1.159363 3.165180 -0.832465
H -2.078358 3.448778 -0.326592
H -0.537901 4.050216 -0.961456
H -1.391860 2.759842 -1.815799
C -0.154846 2.651149 1.409299
H 0.330639 3.626347 1.387248
H -1.118107 2.742843 1.912057
H 0.467008 1.961008 1.981375
Cl 2.769458 -1.900850 0.655256

perF_NMeCO2_Cl.log
Energy (E) = -1382.06615835 Hartree
Enthalpy (H) = -1381.941207 Hartree
Gibbs free energy (G) = -1382.002323 Hartree
Charge = 0, Spin = 1
C -2.253187 -1.581770 -0.280831
C -2.942009 -0.418536 0.021201
C -2.267319 0.777098 0.216165
C -0.874016 0.845636 0.177651
C -0.211616 -0.356245 -0.084448
C -0.871343 -1.539666 -0.357725
I 1.851790 -0.229391 -0.173104
F -0.218819 -2.630514 -0.710702
F -2.907710 -2.704420 -0.516706
F -4.260133 -0.435909 0.068801
F -2.999857 1.869390 0.400568
N -0.184375 2.041686 0.365553
C -0.695629 3.062332 1.279416
H -1.247874 2.575654 2.078848
H 0.154551 3.594616 1.695107
H -1.337169 3.779787 0.773127
C 0.802651 2.476790 -0.546884
O 1.103955 3.641625 -0.596712
O 1.326611 1.523018 -1.292868
Cl 2.087366 -2.228357 1.262371

PhenanthrolineCO2I_Cl.log
Energy (E) = -1229.75720037 Hartree
Enthalpy (H) = -1229.576453 Hartree
Gibbs free energy (G) = -1229.636744 Hartree
Charge = 0, Spin = 1
C 0.573295 2.385468 -0.602599
C 0.037246 3.643918 -0.666093
C -1.317986 3.811379 -0.346365
N -2.122243 2.811326 -0.063981
C -1.624606 1.566920 -0.027701
C -0.247100 1.307922 -0.222887
C -2.552389 0.460622 0.149765
C -2.092737 -0.869006 0.035941
C -0.675710 -1.116483 -0.108014
C 0.158171 -0.053690 -0.144999
N -3.841416 0.767146 0.363237
C -4.712023 -0.213105 0.457639
C -4.365758 -1.565060 0.331942
C -3.052100 -1.898026 0.120473
C -0.092711 -2.496522 -0.293890
O 1.195755 -2.478214 -0.563939
I 2.199895 -0.698039 -0.032481
O -0.736580 -3.515685 -0.260061
Cl 3.199056 1.398781 0.894894
H 1.608202 2.222253 -0.856113
H 0.639016 4.491426 -0.959705
H -1.760096 4.801468 -0.350176
H -5.742743 0.071531 0.638844
H -5.125766 -2.329782 0.406134
H -2.738337 -2.924248 0.027225

PyIBCMe2O_D_Cl.log
Energy (E) = -911.285481309 Hartree
Enthalpy (H) = -911.115938 Hartree
Gibbs free energy (G) = -911.168110 Hartree
Charge = 0, Spin = 1
C 0.985687 3.071596 -0.085657
C 2.273034 2.559869 0.003727
C 1.535418 0.400671 -0.018354
C 0.231343 0.853128 -0.101465
H 0.822134 4.138810 -0.114846
H 3.123615 3.228865 0.050567
O 0.656622 -1.740597 -0.507700
I -1.120938 -0.756064 -0.127075
C -0.087500 2.188825 -0.127078
H -1.115124 2.521155 -0.170286
N 2.548801 1.256381 0.044985
C 1.768652 -1.096086 0.071550
C 2.993855 -1.507805 -0.724163
H 3.874764 -0.993533 -0.343506
H 3.128359 -2.584661 -0.633527
H 2.858172 -1.254760 -1.774266
C 1.918605 -1.470863 1.544041
H 2.069385 -2.546860 1.624452
H 2.770189 -0.950674 1.982444
H 1.021737 -1.199336 2.105101
Cl -3.056039 0.873323 0.257643

pyreneCO2I_Cl.log
Energy (E) = -1273.86153742 Hartree
Enthalpy (H) = -1273.642918 Hartree
Gibbs free energy (G) = -1273.704718 Hartree
Charge = 0, Spin = 1
C 1.770871 3.447924 -0.405608
C 0.413028 3.487434 -0.670380
C -0.349849 2.332937 -0.600945
C 0.237873 1.114497 -0.261253
C 1.648605 1.042569 -0.088075
C 2.408778 2.238273 -0.130430
C 2.300481 -0.210409 0.078192
C -0.447250 -0.132780 -0.164886
C 0.131449 -1.353116 -0.116615
C 1.573044 -1.429622 0.012536
C 2.263672 -2.639794 0.119509
C 3.638456 -2.650479 0.309998
C 4.354394 -1.469191 0.379699
C 3.706768 -0.240454 0.254948
C 4.441530 0.989891 0.276469
C 3.824381 2.173767 0.082673
I -2.581941 -0.312007 -0.026624
O -1.998426 -2.267495 -0.561192
C -0.747777 -2.570900 -0.278090
O -0.356421 -3.709780 -0.217913
Cl -3.142059 1.941993 0.915157
H 2.362758 4.354021 -0.438178
H -0.062512 4.422627 -0.930230

H -1.404182 2.385840 -0.815850
H 1.714667 -3.564100 0.061882
H 4.150956 -3.598441 0.399688
H 5.428288 -1.482582 0.518730
H 5.512106 0.939413 0.430529
H 4.383843 3.100619 0.073224

PyrroleNMeIBCMe2O_A_Cl.log
Energy (E) = -912.472537315 Hartree
Enthalpy (H) = -912.279455 Hartree
Gibbs free energy (G) = -912.334043 Hartree
Charge = 0, Spin = 1
C 1.444243 2.568432 -0.047295
C 0.110819 2.248105 -0.107258
C 0.095993 0.843322 -0.131386
C 1.360301 0.341036 -0.069958
H 1.926211 3.531521 -0.039615
H -0.730707 2.916261 -0.133454
I -1.331103 -0.640922 -0.088536
O 0.414514 -1.761830 -0.398102
N 2.197827 1.417042 -0.014563
C 3.645331 1.389369 0.023941
H 3.996115 0.719878 0.807913
H 4.060749 1.066230 -0.929685
H 4.001941 2.392662 0.242208
C 1.599461 -1.143309 0.054724
C 2.716603 -1.664336 -0.840585
H 3.697047 -1.334403 -0.495502
H 2.691620 -2.752951 -0.813007
H 2.560600 -1.337384 -1.867483
C 1.853335 -1.509472 1.516122
H 1.981514 -2.588631 1.598961
H 2.745831 -1.015257 1.905337
H 1.000039 -1.206599 2.125179
Cl -3.119995 1.136118 0.187558

thiazoleCO2I_Cl.log
Energy (E) = -1227.46035051 Hartree
Enthalpy (H) = -1227.400150 Hartree
Gibbs free energy (G) = -1227.447357 Hartree
Charge = 0, Spin = 1
C -0.494253 0.606905 0.000004
C -1.755002 0.115569 0.000009
N -2.725758 1.075393 -0.000060
C -2.181504 2.250403 -0.000209
S -0.451111 2.296691 0.000255
I 0.987275 -0.812899 0.000009
O -0.778684 -2.001557 0.000057
C -1.949505 -1.364453 0.000033
O -3.013852 -1.915535 0.000026
Cl 2.666692 1.018828 -0.000210
H -2.739406 3.174973 -0.000263

triazoleCO2I_Cl.log
Energy (E) = -939.946390470 Hartree
Enthalpy (H) = -939.853332 Hartree
Gibbs free energy (G) = -939.902792 Hartree
Charge = 0, Spin = 1
C 0.000000 0.835445 0.000000
C 1.264752 1.342329 0.000000
N 1.164106 2.690540 0.000000
N -0.086738 3.006354 0.000000
N -0.835938 1.881222 0.000000
I -0.067599 -1.223622 0.000000
O 2.019923 -0.839547 0.000000
C 2.434539 0.435411 0.000000
O 3.587558 0.756559 0.000000
Cl -2.562698 -1.261903 0.000000
C -2.283774 2.011669 0.000000
H -2.481079 3.078834 0.000000
H -2.706639 1.546716 0.885377
H -2.706639 1.546716 -0.885377

3Obenzofurane_45_CO2I_CF3.log
Energy (E) = -994.552381178 Hartree
Enthalpy (H) = -994.407166 Hartree
Gibbs free energy (G) = -994.468724 Hartree
Charge = 0, Spin = 1
C -0.930764 1.359922 -0.000107
C -0.199988 0.183505 0.000200
C -0.727464 -1.091590 0.000479
C -2.117259 -1.139120 0.000398
C -2.896474 0.014370 0.000042
C -2.316738 1.270590 -0.000201
O -2.824503 -2.283257 0.000634
C -4.215031 -1.952314 0.000453
C -4.300313 -0.426331 0.000117
C -0.252260 2.710391 -0.000184
O 1.039965 2.629215 0.000319
I 1.897443 0.560839 0.000124
O -0.920397 3.720635 -0.000638
O -5.312360 0.225338 -0.000211
H -0.150485 -2.000985 0.000805
H -2.897821 2.183755 -0.000439
H -4.687806 -2.367887 0.888827
H -4.687674 -2.368288 -0.887804
C 2.407064 -1.609438 -0.000359
F 1.979156 -2.262104 -1.077422
F 1.979978 -2.262599 1.076739
F 3.740091 -1.633786 -0.000851

3Oindole_45_CO2I_CF3.log
Energy (E) = -974.691171320 Hartree
Enthalpy (H) = -974.533597 Hartree
Gibbs free energy (G) = -974.596016 Hartree
Charge = 0, Spin = 1
C -0.925025 1.357476 -0.008071
C -0.197996 0.180493 -0.011021
C -0.720076 -1.095414 -0.022201
C -2.116561 -1.167063 -0.028843
C -2.890726 0.003922 -0.012702
C -2.312356 1.257257 -0.008244
N -2.904461 -2.281964 -0.070822
C -4.307556 -1.928088 0.040410
C -4.307502 -0.394966 0.009683
C -0.249541 2.704236 0.000882
O 1.045849 2.625375 0.002142
I 1.899189 0.565027 0.001725
O -0.912170 3.718394 0.005741
O -5.290380 0.302435 0.008898
H -0.120095 -1.990296 -0.032740
H -2.900564 2.166074 0.002127
H -2.557008 -3.197320 0.159763
H -4.752185 -2.262092 0.979926
H -4.906732 -2.315084 -0.783492
C 2.421363 -1.605541 0.004259
F 2.011593 -2.265981 -1.077359
F 1.988724 -2.267225 1.076605
F 3.755266 -1.624292 0.018486

acridineCO2I_CF3.log
Energy (E) = -1091.07936120 Hartree
Enthalpy (H) = -1090.871168 Hartree
Gibbs free energy (G) = -1090.937338 Hartree
Charge = 0, Spin = 1
C -5.438667 0.173570 0.000227
C -4.781345 -1.088990 0.000364
C -3.423147 -1.171866 0.000338
C -2.633715 0.012993 0.000165
C -3.293284 1.281893 -0.000009
C -4.716105 1.325585 0.000040
N -1.300403 -0.096130 0.000115
C -0.562889 1.003324 -0.000090
C -1.116258 2.326018 -0.000239
C -2.501778 2.426025 -0.000203
C 0.854633 0.921333 -0.000107
C 1.670506 1.995704 -0.000139
C 1.096134 3.299517 -0.000333
C -0.250570 3.458459 -0.000394
I 1.887942 -0.954430 0.000114
O 3.566101 0.629094 0.000311
C 3.184664 1.853014 0.000192
O 3.866962 2.858791 0.000379
H -6.519305 0.208516 0.000274
H -5.376712 -1.991919 0.000488
H -2.906225 -2.121001 0.000449
H -5.204448 2.292034 -0.000067
H -2.968050 3.405125 -0.000313
H 1.792473 4.126712 -0.000403
H -0.695215 4.445594 -0.000529
C 0.286037 -2.515566 -0.000286
F -0.470602 -2.575630 -1.080408
F -0.470437 -2.576193 1.079965
F 1.075433 -3.605565 -0.000603

AnthI8BA_B_CF3.log
Energy (E) = -1192.84313155 Hartree
Enthalpy (H) = -1192.533347 Hartree
Gibbs free energy (G) = -1192.608388 Hartree
Charge = 0, Spin = 1
C -6.960855 -0.616301 0.077012
C -5.886370 -1.339563 0.489792
C -4.558771 -0.881641 0.241934
C -4.378799 0.358333 -0.452525
C -5.531859 1.086727 -0.868600
C -6.781300 0.616369 -0.613198
C -3.442009 -1.605134 0.653753
C -3.088411 0.817757 -0.701799
C -1.977305 0.089676 -0.285975
C -2.151344 -1.145923 0.403057
C -0.995261 -1.872857 0.795812
H -1.144987 -2.821960 1.298514
C 0.289830 -1.464561 0.560135
C 0.408404 -0.201140 -0.095508
H -3.580924 -2.545253 1.175690
H -7.963047 -0.973878 0.270446
H -6.017156 -2.277840 1.014450
H -5.387255 2.022999 -1.393255
H -7.649430 1.176079 -0.933469
H -2.948006 1.757257 -1.224288
I 2.286204 0.731905 -0.456205
C -0.648723 0.539115 -0.515492
H -0.510114 1.478321 -1.032851
C 1.399982 -2.417319 0.942587
H 1.649242 -3.010972 0.059818
H 0.953426 -3.116272 1.649100
C 2.686692 -1.839202 1.555928
H 2.498277 -0.825908 1.914072
H 2.918909 -2.411807 2.452550
C 3.922951 -1.890907 0.632620
H 4.515626 -0.978693 0.740805
H 4.569453 -2.719843 0.907878
C 3.586263 -2.098329 -0.842493

O 2.754065 -1.243341 -1.385761
O 4.045112 -3.045451 -1.445266
C 1.526387 2.525738 0.648759
F 0.706244 3.313736 -0.050133
F 0.910210 2.192795 1.778641
F 2.607632 3.249258 0.957362

AzuleneCO2I_CF3.log
Energy (E) = -921.481495805 Hartree
Enthalpy (H) = -921.311467 Hartree
Gibbs free energy (G) = -921.373267 Hartree
Charge = 0, Spin = 1
C -4.176346 -1.836981 -0.108054
C -2.863703 -2.266814 -0.262694
C -1.685313 -1.529751 -0.236210
C -1.505941 -0.167606 -0.051947
C -2.585662 0.854128 0.093548
C -3.956858 0.636061 0.154624
C -4.670437 -0.550857 0.081352
C -0.330190 0.589902 -0.032722
C -0.608727 1.949790 0.071243
C -1.986715 2.114440 0.156349
I 1.745366 0.227091 -0.104896
O 1.629380 2.489406 -0.186772
C 0.453861 3.002966 0.013453
O 0.175549 4.177966 0.127142
H -4.925092 -2.619321 -0.152120
H -2.737009 -3.329735 -0.424395
H -0.791417 -2.112023 -0.396981
H -4.544868 1.539837 0.279196
H -5.745965 -0.463635 0.167188
H -2.487339 3.065634 0.244047
C 1.703195 -1.988149 0.157986
F 1.317001 -2.683422 -0.912305
F 0.984834 -2.395128 1.199343
F 2.983153 -2.287935 0.390523

BenzoFuraneCO2I_CF3.log
Energy (E) = -919.357118472 Hartree
Enthalpy (H) = -919.216705 Hartree
Gibbs free energy (G) = -919.275395 Hartree
Charge = 0, Spin = 1
C 2.943073 -2.561184 0.000108
C 4.089092 -1.755868 0.000050
C 3.990708 -0.377206 -0.000031
C 2.713473 0.154666 -0.000056
C 1.543228 -0.626357 -0.000025
C 1.672431 -2.019036 0.000074
O 2.427191 1.484341 -0.000079
C 1.088711 1.589287 -0.000076
C 0.507566 0.371232 -0.000056
C 0.358915 2.896383 0.000000
O -0.920002 2.678563 -0.000039
I -1.570700 0.489732 -0.000010
O 0.944312 3.953199 0.000080
H 3.055294 -3.636329 0.000185
H 5.065070 -2.220452 0.000073
H 4.855873 0.269350 -0.000063
H 0.813370 -2.668601 0.000121
C -1.843588 -1.709398 0.000026
F -1.337216 -2.297107 1.077600
F -1.337068 -2.297067 -1.077498
F -3.163069 -1.895126 -0.000055

BenzothiopheneCO2I_CF3.log
Energy (E) = -1242.28549509 Hartree
Enthalpy (H) = -1242.147663 Hartree
Gibbs free energy (G) = -1242.208092 Hartree
Charge = 0, Spin = 1
C 3.796436 -0.081122 0.000000
C 4.219900 1.253799 0.000000
C 3.296717 2.276465 0.000000
C 1.940091 1.955761 0.000000
C 1.495534 0.612836 0.000000
C 2.457549 -0.406736 0.000000
S 0.644737 3.097408 0.000000
C -0.510426 1.842368 0.000000
C 0.059110 0.621194 0.000000
C -1.984742 2.117149 0.000000
O -2.667157 1.022060 0.000000
I -1.432850 -0.894914 0.000000
O -2.386916 3.260850 0.000000
H 4.532087 -0.873168 0.000000
H 5.276412 1.482521 0.000000
H 3.608734 3.312089 0.000000
H 2.173147 -1.443632 0.000000
C -0.117711 -2.689049 0.000000
F 0.644737 -2.817274 1.078723
F 0.644737 -2.817274 -1.078723
F -1.005834 -3.686025 0.000000

CyclohexeneCO2I_CF3.log
Energy (E) = -770.446348157 Hartree
Enthalpy (H) = -770.277971 Hartree
Gibbs free energy (G) = -770.334528 Hartree
Charge = 0, Spin = 1
C -1.776412 2.708857 -0.414597
C -2.951712 2.188064 0.400311
C -3.239892 0.738431 0.039107
C -1.984527 -0.083021 -0.001769
C -0.803279 0.501186 -0.018292
C -0.492498 1.956496 -0.050347
C -2.141738 -1.591854 -0.022208
O -1.024383 -2.236704 -0.038972
I 0.807930 -0.932959 -0.009904
O -3.261648 -2.061235 -0.024718
H -1.618950 3.774739 -0.253137
H -1.983113 2.564964 -1.477989
H -3.837284 2.800304 0.233644
H -2.707902 2.257842 1.464327
H -3.723750 0.661683 -0.938420
H -3.923503 0.259835 0.739872
H 0.288162 2.179227 -0.776484
H -0.121181 2.282685 0.923659
C 2.401140 0.626358 0.035027
F 2.299219 1.480281 1.050393
F 2.501206 1.333643 -1.089598
F 3.536127 -0.060373 0.185379

DibenzofuranCO2I_CF3.log
Energy (E) = -1072.88456610 Hartree
Enthalpy (H) = -1072.694952 Hartree
Gibbs free energy (G) = -1072.759690 Hartree
Charge = 0, Spin = 1
C 5.023472 -2.047365 -0.000017
C 5.896568 -0.953276 0.000037
C 5.426812 0.351460 0.000070
C 4.053602 0.512237 0.000047
C 3.159608 -0.561533 -0.000007
C 3.651499 -1.864854 -0.000040
O 3.399654 1.712172 0.000073
C 2.070297 1.427126 0.000035
C 1.846074 0.042510 -0.000015
C 1.051766 2.356088 0.000045
C -0.247461 1.866461 0.000001
C -0.457077 0.496667 -0.000050
C 0.544357 -0.450962 -0.000058
C -1.408397 2.838169 0.000015
O -2.562391 2.253966 -0.000024
I -2.529600 0.009894 -0.000111
O -1.194688 4.031461 0.000062
H 5.431059 -3.048544 -0.000041
H 6.963399 -1.129558 0.000054
H 6.090432 1.203550 0.000112
H 2.979422 -2.712759 -0.000081
H 1.208114 3.425459 0.000085
H 0.362730 -1.513661 -0.000097
C -2.139794 -2.182736 0.000104
F -1.487142 -2.614060 1.077069
F -1.486975 -2.614239 -1.076687
F -3.351984 -2.739436 0.000057

FuranIBCMe2S_A_CF3.log
Energy (E) = -1093.32655026 Hartree
Enthalpy (H) = -1093.161489 Hartree
Gibbs free energy (G) = -1093.220571 Hartree
Charge = 0, Spin = 1
C -0.959025 2.976111 -0.234590
C 0.177764 2.245307 -0.294561
C -0.270612 0.896130 -0.185272
C -1.614414 0.896035 -0.053675
O -2.045000 2.174879 -0.085116
H -1.148847 4.031865 -0.299860
H 1.177866 2.616787 -0.418846
I 0.726421 -0.926545 -0.099051
S -1.802475 -1.732324 -0.454612
C -2.572448 -0.219818 0.201349
C -2.815937 -0.334978 1.707542
H -3.252157 0.587857 2.097706
H -3.499270 -1.161190 1.901774
H -1.880344 -0.533375 2.231807
C -3.889553 0.022198 -0.531365
H -4.554325 -0.824498 -0.362812
H -4.371703 0.926405 -0.155689
H -3.722252 0.124200 -1.602193
C 2.667247 0.272119 0.142595
F 2.996614 0.987295 -0.938769
F 2.661138 1.104207 1.186405
F 3.632493 -0.631681 0.341298

IndeneCO2I_CF3.log
Energy (E) = -883.451524639 Hartree
Enthalpy (H) = -883.287649 Hartree
Gibbs free energy (G) = -883.347543 Hartree
Charge = 0, Spin = 1
C -2.854413 -2.611428 -0.001117
C -4.048990 -1.900512 -0.000329
C -4.039692 -0.510163 0.000452
C -2.827675 0.153559 0.000489
C -1.616824 -0.568554 -0.000163
C -1.628848 -1.957308 -0.001053
C -2.556383 1.629686 0.000977
C -1.067585 1.695691 0.000532
C -0.572719 0.458793 -0.000058
C -0.237590 2.937173 0.000303
O 1.030705 2.672379 -0.001056
I 1.547859 0.462227 -0.000517
O -0.769474 4.028921 0.000505
H -2.875335 -3.692411 -0.001821
H -4.990145 -2.432858 -0.000361
H -4.967461 0.047235 0.000986
H -0.723361 -2.538683 -0.001782
H -2.964107 2.142514 0.875013

H -2.964713 2.143168 -0.872387
C 1.838453 -1.742205 0.000670
F 1.364211 -2.355784 -1.078241
F 1.361384 -2.354679 1.078943
F 3.166888 -1.876847 0.002399

Indole_NMe_IBCONAc_A_CF3.log
Energy (E) = -1071.43143644 Hartree
Enthalpy (H) = -1071.195625 Hartree
Gibbs free energy (G) = -1071.268688 Hartree
Charge = 0, Spin = 1
C 2.005220 0.111444 -0.023271
C 2.519964 1.432561 0.001492
C 3.889067 1.702188 -0.007348
C 4.753102 0.630533 -0.043856
C 4.265098 -0.685939 -0.073233
C 2.914424 -0.956614 -0.064051
C 0.592242 0.303761 -0.004908
H 4.251901 2.720781 0.013036
H 5.820394 0.802899 -0.051701
H 4.966437 -1.508085 -0.104877
H 2.583193 -1.980199 -0.090508
N 1.486640 2.334263 0.029506
C 0.308039 1.644097 0.023587
C -1.060857 2.239526 0.034107
O -1.236850 3.441526 0.080610
I -1.082356 -0.936707 -0.006986
C 1.692700 3.773097 0.051298
H 2.249148 4.076019 -0.835459
H 0.727872 4.264280 0.067886
H 2.263265 4.046855 0.938550
N -1.983719 1.234126 -0.013069
C -3.353806 1.475364 -0.042216
C -4.190320 0.210741 -0.053736
H -3.993647 -0.392526 0.834953
H -5.239532 0.487611 -0.070689
H -3.966439 -0.393122 -0.935608
O -3.870749 2.566768 -0.062177
C 0.042298 -2.875764 0.036605
F 0.764592 -3.126884 -1.051227
F 0.816620 -3.039731 1.104214
F -0.939033 -3.783836 0.095385

IndoleAc_54_CO2I_CF3.log
Energy (E) = -1052.04787071 Hartree
Enthalpy (H) = -1051.854134 Hartree
Gibbs free energy (G) = -1051.920942 Hartree
Charge = 0, Spin = 1
C 0.026220 2.016245 0.091973
C -1.233222 2.602963 0.255820
C -2.387419 1.850127 0.253083
C -2.258944 0.479041 0.046058
C -1.007891 -0.165329 -0.123118
C 0.115897 0.655025 -0.057762
N -3.247817 -0.498568 -0.076219
C -2.623166 -1.712089 -0.341815
C -1.284059 -1.554519 -0.379130
I 2.173101 0.040458 -0.158651
O 2.320582 2.269965 -0.311942
C 1.242094 2.913841 -0.003123
O 1.130651 4.108201 0.170288
O -5.088075 0.794107 0.262442
C -4.631819 -0.298156 0.033303
C -5.488234 -1.521562 -0.151309
H -1.250929 3.677674 0.373814
H -3.357372 2.299611 0.380757
H -3.200762 -2.606207 -0.492014
H -0.586357 -2.339786 -0.590862
H -6.522777 -1.217487 -0.035854
H -5.246124 -2.282743 0.589812
H -5.342912 -1.953123 -1.141272
C 1.902765 -2.141266 0.251820
F 1.550756 -2.890445 -0.794087
F 1.082706 -2.413028 1.259621
F 3.136425 -2.513897 0.605193

IndoleAc_56_CO2I_CF3.log
Energy (E) = -1052.05613097 Hartree
Enthalpy (H) = -1051.862532 Hartree
Gibbs free energy (G) = -1051.929260 Hartree
Charge = 0, Spin = 1
C 0.193592 2.006580 0.000031
C 0.239672 0.620972 -0.000003
C -0.855396 -0.214120 -0.000058
C -2.084272 0.441236 -0.000035
C -2.192381 1.846719 0.000031
N -3.378818 -0.079281 0.000008
C -4.271593 0.997235 0.000021
C -3.596719 2.165131 0.000050
O 1.383711 4.048552 -0.000029
C 1.458110 2.837424 -0.000027
O 2.536555 2.124694 -0.000037
O -2.861639 -2.287604 -0.000004
C -3.717522 -1.435350 0.000011
C -5.188562 -1.748913 -0.000051
H -0.809542 -1.288198 -0.000094
H -5.332446 0.820853 0.000035
H -4.032135 3.149445 0.000067
H -5.295936 -2.828082 -0.000147
H -5.674542 -1.332807 -0.882159
H -5.674562 -1.332969 0.882123
I 2.240191 -0.109682 0.000011
C -1.042859 2.628698 0.000056
H -1.061677 3.710469 0.000053
C 1.601552 -2.241876 0.000043
F 0.908429 -2.593209 1.077319
F 0.908433 -2.593001 -1.077349
F 2.746662 -2.929143 -0.000009

NaphIBMeUreaMe_CF3.log
Energy (E) = -1035.52097881 Hartree
Enthalpy (H) = -1035.260605 Hartree
Gibbs free energy (G) = -1035.330501 Hartree
Charge = 0, Spin = 1
C 2.664923 -0.442172 -0.447322
C 2.963483 0.829576 0.098725
C 1.912746 1.687107 0.490649
C 0.586986 1.331060 0.373801
C 0.335285 0.033403 -0.143104
C 1.307628 -0.821550 -0.554483
H 2.167491 2.662938 0.881725
H 1.065335 -1.798043 -0.951198
I -1.675540 -0.563780 -0.217141
C -1.546066 2.485514 -0.049665
O -2.140273 3.541752 0.099970
N -1.825969 1.541342 -0.973037
C -2.977313 1.826257 -1.807179
H -2.848375 2.782055 -2.314302
H -3.073272 1.045084 -2.562571
H -3.909007 1.889808 -1.236316
N -0.442593 2.178753 0.792521
C -0.114748 3.215012 1.757916
H 0.511315 2.786228 2.538202
H 0.401958 4.062277 1.299789
H -1.039193 3.587535 2.185444
C 4.321449 1.213684 0.216174
C 5.320440 0.372406 -0.189060
C 5.017253 -0.896996 -0.730735
C 3.715350 -1.295149 -0.856773
H 4.552924 2.186890 0.630751
H 6.353891 0.677385 -0.095041
H 5.819383 -1.551039 -1.043522
H 3.468527 -2.265947 -1.268123
C -1.057604 -2.598818 0.605796
F -0.194003 -2.537028 1.617826
F -0.547207 -3.437342 -0.308621
F -2.188136 -3.158467 1.059576

NpthIBCMe2O_C_CF3.log
Energy (E) = -926.089457016 Hartree
Enthalpy (H) = -925.843045 Hartree
Gibbs free energy (G) = -925.907668 Hartree
Charge = 0, Spin = 1
C 2.185263 1.408872 0.100499
C 0.819443 1.360224 0.013728
C 0.230591 0.095278 -0.122765
C 0.923207 -1.071398 -0.183330
H 2.687056 2.362655 0.213198
H 0.445634 -2.032427 -0.304307
O -1.283902 2.257352 -0.587743
I -1.888966 0.231654 -0.224405
C 3.123098 -2.195997 -0.173213
C 4.485998 -2.122827 -0.093397
C 5.123887 -0.871124 0.056560
C 4.386234 0.278403 0.122311
C 2.973669 0.235415 0.043225
C 2.336713 -1.021956 -0.103979
H 2.624574 -3.150417 -0.287620
H 5.082034 -3.023708 -0.144249
H 6.202743 -0.826771 0.118608
H 4.870508 1.240511 0.234903
C -0.108916 2.573538 0.083718
C 0.504882 3.785596 -0.605419
H -0.233525 4.586300 -0.597871
H 1.400802 4.137765 -0.092436
H 0.748957 3.543965 -1.638725
C -0.384378 2.890597 1.557707
H 0.533847 3.152838 2.085522
H -1.083678 3.725131 1.611618
H -0.831180 2.029890 2.059612
C -2.084595 -1.982732 0.197868
F -1.710631 -2.796255 -0.799552
F -1.461048 -2.398183 1.303832
F -3.400758 -2.167067 0.381951

NpthISO2NMe_D_CF3.log
Energy (E) = -1376.06953898 Hartree
Enthalpy (H) = -1375.854667 Hartree
Gibbs free energy (G) = -1375.921632 Hartree
Charge = 0, Spin = 1
C -0.212651 3.357799 -0.951293
C 1.079718 3.504434 -0.547065
C 1.842165 2.396131 -0.112475
C 1.275996 1.083365 -0.071466
C -0.098739 1.013361 -0.427608
C -0.810688 2.086496 -0.878993
H 3.571194 3.618555 0.233196
H -0.789493 4.197906 -1.309664
H 1.560005 4.474318 -0.565065
C 3.190134 2.605724 0.264434
C 2.161563 0.022002 0.274401
H -1.841639 1.977434 -1.178252
C 3.466075 0.261826 0.611986
C 3.988515 1.566307 0.637239

H 4.099090 -0.587991 0.828135
H 5.019370 1.728955 0.917391
I -1.245060 -0.765515 -0.206037
S 1.737810 -1.702731 0.272302
O 1.100655 -1.996850 1.541005
O 2.927238 -2.456651 -0.050015
N 0.702292 -1.710077 -0.965986
C 0.419051 -3.048762 -1.480646
H -0.165066 -2.947307 -2.394294
H 1.349028 -3.562887 -1.721749
H -0.139516 -3.673913 -0.772356
C -2.990676 0.469722 0.561488
F -2.655689 1.431760 1.415177
F -3.756969 1.018746 -0.392047
F -3.751980 -0.419500 1.207956

perF_CMe2O_CF3.log
Energy (E) = -1169.36930553 Hartree
Enthalpy (H) = -1169.200893 Hartree
Gibbs free energy (G) = -1169.267263 Hartree
Charge = 0, Spin = 1
C 1.687061 2.180551 -0.173578
C 2.790195 1.399000 0.121799
C 2.659348 0.019624 0.208139
C 1.440582 -0.603594 0.006174
C 0.344828 0.208771 -0.213175
C 0.448358 1.577472 -0.337232
O 0.040412 -2.362660 -0.700748
I -1.470185 -0.899401 -0.273765
F -0.581912 2.347064 -0.656193
F 1.814895 3.489749 -0.307661
F 3.969771 1.969127 0.295074
F 3.755468 -0.676277 0.503306
C 1.217065 -2.129634 -0.010552
C 2.324778 -2.851172 -0.774512
H 3.273008 -2.844513 -0.243160
H 1.996641 -3.881562 -0.904309
H 2.452255 -2.405970 -1.760357
C 1.146514 -2.626192 1.434796
H 0.965882 -3.700788 1.426037
H 2.077845 -2.423138 1.965475
H 0.328604 -2.139986 1.969998
C -2.722112 0.862725 0.385696
F -2.173018 1.571034 1.369977
F -3.147765 1.695156 -0.555582
F -3.806044 0.241947 0.881806

perF_NMeCO2_CF3.log
Energy (E) = -1259.41505034 Hartree
Enthalpy (H) = -1259.274525 Hartree
Gibbs free energy (G) = -1259.341578 Hartree
Charge = 0, Spin = 1
C -1.649962 -2.249020 -0.374290
C -2.738505 -1.500205 0.044818
C -2.614814 -0.143333 0.296942
C -1.389411 0.524490 0.191956
C -0.309086 -0.274297 -0.197679
C -0.430488 -1.613700 -0.510734
I 1.531453 0.673290 -0.392033
F 0.618537 -2.309293 -0.929426
F -1.781063 -3.536368 -0.645406
F -3.919595 -2.078354 0.157725
F -3.724533 0.520438 0.600179
N -1.259383 1.881266 0.438076
C -2.081012 2.535195 1.453296
H -2.311816 1.821263 2.240458
H -1.506626 3.359366 1.865277
H -3.003909 2.936525 1.040220
C -0.642086 2.751559 -0.523142
O -0.872353 3.935933 -0.439931
O 0.112016 2.147038 -1.383041
C 2.561891 -0.941462 0.766587
F 1.733646 -1.587037 1.574600
F 3.179884 -1.816281 -0.010086
F 3.483632 -0.323051 1.503804

PhenanthrolineCO2I_CF3.log
Energy (E) = -1107.10128394 Hartree
Enthalpy (H) = -1106.904944 Hartree
Gibbs free energy (G) = -1106.971224 Hartree
Charge = 0, Spin = 1
C 0.549263 2.173158 -0.789333
C 0.183788 3.490155 -0.871628
C -1.124396 3.840751 -0.505046
N -2.035078 2.961137 -0.154947
C -1.702355 1.662763 -0.098104
C -0.382551 1.221200 -0.347126
C -2.746704 0.687267 0.166963
C -2.455798 -0.692940 0.091181
C -1.091746 -1.130418 -0.115467
C -0.144885 -0.177692 -0.236397
N -3.977047 1.159605 0.422296
C -4.955099 0.298231 0.594028
C -4.782871 -1.089540 0.507351
C -3.531123 -1.590331 0.255187
C -0.697178 -2.600574 -0.288282
O 0.480312 -2.736503 -0.794331
I 1.838895 -1.016444 -0.267282
O -1.459500 -3.500792 -0.000872
H 1.535266 1.865081 -1.101276
H 0.876977 4.242998 -1.217265
H -1.435562 4.879188 -0.525786
H -5.933635 0.714590 0.807635
H -5.626876 -1.750915 0.642757
H -3.342380 -2.649402 0.194172
C 2.922522 0.689708 0.707828
F 2.167722 1.377681 1.550254
F 3.521296 1.539461 -0.124966
F 3.881867 0.075383 1.400152

PyIBCMe2O_D_CF3.log
Energy (E) = -788.618918513 Hartree
Enthalpy (H) = -788.433929 Hartree
Gibbs free energy (G) = -788.492281 Hartree
Charge = 0, Spin = 1
C 1.003796 3.097759 -0.144083
C 2.329786 2.728933 0.033841
C 1.826607 0.499507 0.005387
C 0.486858 0.801719 -0.147441
H 0.727460 4.139644 -0.213058
H 3.100242 3.486008 0.118325
O 1.245330 -1.729045 -0.515462
I -0.716623 -0.940169 -0.190763
C 0.038993 2.101806 -0.225660
H -1.002015 2.354499 -0.354418
N 2.737600 1.464103 0.110753
C 2.238139 -0.964632 0.090149
C 3.545454 -1.189846 -0.655485
H 4.344574 -0.585116 -0.230329
H 3.803072 -2.245739 -0.582642
H 3.420127 -0.931909 -1.706202
C 2.396416 -1.321059 1.570825
H 2.678504 -2.370675 1.650102
H 3.161030 -0.701979 2.041112
H 1.453846 -1.173986 2.103617
C -2.559151 0.327850 0.158728
F -2.492667 1.115634 1.237055
F -2.923971 1.100050 -0.874599
F -3.547034 -0.554613 0.361414

pyreneCO2I_CF3.log
Energy (E) = -1151.20586417 Hartree
Enthalpy (H) = -1150.971711 Hartree
Gibbs free energy (G) = -1151.039400 Hartree
Charge = 0, Spin = 1
C 1.551349 3.512744 -0.573098
C 0.213308 3.349219 -0.887616
C -0.377849 2.098979 -0.799555
C 0.357881 0.989198 -0.391206
C 1.753076 1.126197 -0.158467
C 2.339638 2.415308 -0.224542
C 2.558475 -0.017365 0.094330
C -0.152924 -0.336696 -0.262012
C 0.573572 -1.465150 -0.130389
C 2.003966 -1.326311 0.063950
C 2.850429 -2.423794 0.248762
C 4.205355 -2.237057 0.484709
C 4.750639 -0.966289 0.525768
C 3.946838 0.154850 0.322050
C 4.503671 1.475560 0.312652
C 3.740222 2.555596 0.044428
I -2.268139 -0.743347 -0.266850
O -1.300104 -2.708056 -0.806526
C -0.128788 -2.819820 -0.278787
O 0.410846 -3.858136 0.044824
H 2.011152 4.491607 -0.623574
H -0.377334 4.196680 -1.206233
H -1.411127 1.985242 -1.086016
H 2.427126 -3.413874 0.221664
H 4.838572 -3.100859 0.634236
H 5.809867 -0.825606 0.702620
H 5.563855 1.580214 0.507072
H 4.168907 3.549342 0.014954
C -2.980554 1.136305 0.733106
F -3.398574 2.104552 -0.079697
F -2.106100 1.642067 1.587925
F -4.045927 0.712861 1.417040

PyrroleNMeIBCMe2O_A_CF3.log
Energy (E) = -789.811167602 Hartree
Enthalpy (H) = -789.602695 Hartree
Gibbs free energy (G) = -789.663642 Hartree
Charge = 0, Spin = 1
C 1.467652 2.620441 -0.053016
C 0.185864 2.138563 -0.119454
C 0.338211 0.735894 -0.128916
C 1.654410 0.395497 -0.055050
H 1.827356 3.635745 -0.048993
H -0.717735 2.718588 -0.164749
I -0.942158 -0.895725 -0.095621
O 1.021680 -1.827336 -0.332305
N 2.352249 1.569197 -0.007198
C 3.791670 1.727883 0.028512
H 4.229582 1.088071 0.792146
H 4.240748 1.486994 -0.934043
H 4.016002 2.762943 0.274087
C 2.108396 -1.047167 0.048188
C 3.264599 -1.371419 -0.896453
H 4.200326 -0.900401 -0.591659
H 3.403339 -2.451946 -0.884088
H 3.015485 -1.066612 -1.912389
C 2.495574 -1.358244 1.497361
H 2.781642 -2.408014 1.565232
H 3.325678 -0.741705 1.850310

H 1.635352 -1.187234 2.146658
C -2.663187 0.529078 0.082291
F -2.599979 1.301619 1.169822
F -2.787056 1.339751 -0.973268
F -3.782042 -0.202688 0.166049

thiazoleCO2I_CF3.log
Energy (E) = -1104.80489629 Hartree
Enthalpy (H) = -1104.729042 Hartree
Gibbs free energy (G) = -1104.782847 Hartree
Charge = 0, Spin = 1
C -0.732802 0.595354 -0.000090
C -2.054364 0.297910 -0.000181
N -2.867324 1.396985 -0.000292
C -2.165578 2.481666 -0.000229
S -0.449591 2.270830 -0.000219
I 0.556826 -1.028494 0.000083
O -1.487419 -1.951264 -0.000054
C -2.509835 -1.139003 -0.000146
O -3.676804 -1.440317 -0.000243
H -2.581028 3.478406 -0.000288
C 2.305667 0.349849 0.000210
F 2.336971 1.128698 1.076046
F 2.337060 1.128787 -1.075556
F 3.391438 -0.419973 0.000225

triazoleCO2I_CF3.log
Energy (E) = -817.288464586 Hartree
Enthalpy (H) = -817.179644 Hartree
Gibbs free energy (G) = -817.236660 Hartree
Charge = 0, Spin = 1
C -0.089393 1.043897 0.000000
C -1.220349 1.807481 0.000000
N -0.833550 3.103227 0.000000
N 0.454509 3.153798 0.000000
N 0.950272 1.898195 0.000000
I -0.435408 -1.008642 0.000000
O -2.477412 -0.111930 0.000000
C -2.583204 1.196978 0.000000
O -3.604773 1.833329 0.000000
C 2.391389 1.739511 0.000000
H 2.796500 2.746420 0.000000
H 2.722429 1.212892 -0.889885
H 2.722429 1.212892 0.889885
C 1.721704 -1.588586 0.000000
F 2.391389 -1.193217 1.078322
F 2.391389 -1.193217 -1.078322
F 1.680912 -2.921075 0.000000

Benzofuran54_CO2I_02.log
Energy (E) = -581.903520274 Hartree
Enthalpy (H) = -581.783171 Hartree
Gibbs free energy (G) = -581.832954 Hartree
Charge = 0, Spin = 2
C 1.295634 0.674045 0.000000
C 1.539930 2.057226 0.000000
C 0.511360 2.976044 0.000000
C -0.774603 2.457390 0.000000
C -1.065328 1.087823 -0.000000
C -0.000000 0.193754 0.000000
O -1.923609 3.170328 0.000000
C -2.943914 2.267602 -0.000000
C -2.499995 0.995648 -0.000000
I -0.370798 -1.868168 -0.000000
O 2.306598 -1.508886 0.000000
C 2.486865 -0.217244 0.000000
O 3.632507 0.196141 0.000000
H 2.575827 2.368607 0.000000
H 0.688636 4.041046 0.000000
H -3.934667 2.685795 -0.000000
H -3.101184 0.103070 -0.000000

4FIBA_02.log
Energy (E) = -827.356814462 Hartree
Enthalpy (H) = -827.283999 Hartree
Gibbs free energy (G) = -827.338091 Hartree
Charge = 0, Spin = 2
C -0.626483 -1.429291 -0.000084
C -2.013277 -1.372652 -0.000024
C -2.648755 -0.143826 0.000041
C -1.899852 1.025834 0.000022
C -0.507394 0.983105 0.000003
C 0.112388 -0.264972 -0.000035
C 0.289438 2.248552 -0.000061
O 1.589181 2.141792 0.000583
I 2.194556 -0.434289 -0.000005
F -0.045554 -2.620980 -0.000111
F -2.722641 -2.483742 -0.000022
F -3.967577 -0.087523 0.000117
F -2.567972 2.160017 0.000163
O -0.190949 3.361568 -0.000615

Benzofuran54_CO2I_Cl.log
Energy (E) = -1042.02470036 Hartree
Enthalpy (H) = -1041.900177 Hartree
Gibbs free energy (G) = -1041.952877 Hartree
Charge = 0, Spin = 1
C -1.079815 1.363959 0.000000
C -0.888896 2.751021 0.000000
C 0.379882 3.285239 0.000000
C 1.435189 2.387237 0.000000
C 1.310234 0.982818 0.000000
C -0.000000 0.510506 0.000000
O 2.737676 2.736647 0.000000
C 3.452214 1.579822 0.000000
C 2.664653 0.488314 0.000000
I -0.677460 -1.510468 0.000000
O -2.527687 -0.489883 0.000000
C -2.476107 0.828042 0.000000
Cl 1.571996 -2.598775 0.000000
O -3.447653 1.540435 0.000000
H -1.778173 3.366184 0.000000
H 0.561387 4.349728 0.000000
H 4.521815 1.690661 0.000000
H 2.993641 -0.531924 0.000000

Benzofuran54_CO2I_CF3.log
Energy (E) = -919.365978248 Hartree
Enthalpy (H) = -919.225795 Hartree
Gibbs free energy (G) = -919.284570 Hartree
Charge = 0, Spin = 1
C -1.743853 -1.098789 0.070339
C -3.101308 -0.767295 0.177135
C -3.516908 0.545148 0.172467
C -2.531604 1.508814 0.039342
C -1.152579 1.246772 -0.070264
C -0.801676 -0.103592 -0.030295
O -2.761257 2.839203 -0.027013
C -1.554234 3.434282 -0.192863
C -0.544329 2.544363 -0.229387
I 1.178803 -0.933144 -0.101154
O -0.108528 -2.759267 -0.189232
C -1.370292 -2.565636 0.013145
O -2.227742 -3.414303 0.130970
H -3.792098 -1.595278 0.254868
H -4.556801 0.825698 0.251145
H -1.564939 4.506581 -0.274100
H 0.487056 2.797967 -0.365858
C 2.340977 0.956207 0.144639
F 1.984017 1.693948 1.189819
F 3.576638 0.496443 0.363473
F 2.393264 1.742381 -0.929763

5FPhI_Cl_02.log
Energy (E) = -1198.84584934 Hartree
Enthalpy (H) = -1198.781366 Hartree
Gibbs free energy (G) = -1198.838087 Hartree
Charge = 0, Spin = 2
C 0.870807 -1.266555 0.105316
C 2.234139 -1.094546 0.282134
C 2.800497 0.152902 0.069690
C 2.008528 1.222524 -0.320057
C 0.646487 1.039852 -0.495688
C 0.070735 -0.204016 -0.283522
F -0.086720 2.071287 -0.870853
F 2.556443 2.407673 -0.524522
F 4.098459 0.321920 0.234694
F 2.994487 -2.111342 0.651269
F 0.352530 -2.465201 0.317608
I -1.981212 -0.447410 -0.495070
Cl -2.118808 1.328985 1.871629

Anth_I_Cl_02.log
Energy (E) = -1009.86217467 Hartree
Enthalpy (H) = -1009.663031 Hartree
Gibbs free energy (G) = -1009.721691 Hartree
Charge = 0, Spin = 2
C -4.179802 -1.412449 0.417294
C -3.902812 -0.083140 0.402603
C -2.575676 0.390887 0.191689
C -1.512406 -0.549602 -0.015104
C -1.850878 -1.934574 0.009168
C -3.130284 -2.347869 0.219305
C -2.302978 1.753025 0.182319
C -0.222334 -0.039976 -0.225366
C 0.069814 1.331419 -0.218847
C -1.018129 2.243772 -0.012618
C -0.768413 3.646250 -0.008671
H -1.610028 4.309885 0.145968
C 0.486073 4.134290 -0.189611
C 1.567709 3.235025 -0.379131
C 1.370756 1.888221 -0.392097
H -3.116200 2.453508 0.335823
H -5.189428 -1.763212 0.579688
H -4.683858 0.651727 0.553039
H -1.073940 -2.670040 -0.136248
H -3.351332 -3.406347 0.237135
H 0.668093 5.200012 -0.183000
H 2.567951 3.625833 -0.505582
H 2.218753 1.231608 -0.510406
I 1.354051 -1.394454 -0.575509
Cl 2.918907 -0.544624 1.770586

Furan3I_Cl_02.log
Energy (E) = -700.678110181 Hartree
Enthalpy (H) = -700.608605 Hartree
Gibbs free energy (G) = -700.653985 Hartree
Charge = 0, Spin = 2
C 1.440427 1.208846 -0.000140
C 1.083297 -0.095109 0.000008
C 2.282524 -0.869655 0.000116

C 3.283663 0.040264 0.000048
O 2.787148 1.297063 -0.000113
H 0.872451 2.120585 -0.000255
H 2.375605 -1.940890 0.000237
H 4.354398 -0.051682 0.000088
I -0.855851 -0.837953 0.000047
Cl -1.945825 1.894170 -0.000109

IndoleNAc3I_Cl_02.log
Energy (E) = -986.869810731 Hartree
Enthalpy (H) = -986.696968 Hartree
Gibbs free energy (G) = -986.756856 Hartree
Charge = 0, Spin = 2
C 0.875308 1.087289 0.029385
C 2.067435 0.333660 0.045358
C 3.293077 0.968647 0.236850
C 3.295528 2.342958 0.414086
C 2.114978 3.090219 0.403201
C 0.897695 2.467321 0.209016
C -0.190582 0.152412 -0.191357
C 0.330672 -1.085391 -0.300079
H 4.229390 0.436844 0.252322
H 4.240840 2.845850 0.564531
H 2.158757 4.160682 0.546768
H -0.026416 3.031116 0.196083
H -0.140840 -2.041720 -0.441037
I -2.204427 0.626629 -0.302754
C 2.484304 -2.186749 -0.232745
C 3.965397 -2.067539 -0.033877
H 4.408172 -1.440725 -0.807158
H 4.192310 -1.627742 0.936403
H 4.381706 -3.067515 -0.092147
N 1.713382 -1.009641 -0.158531
O 1.937640 -3.239555 -0.448829
Cl -2.876880 -1.949479 0.947735

Me_Pyrrole_3I_Cl_02.log
Energy (E) = -720.096201199 Hartree
Enthalpy (H) = -719.984626 Hartree
Gibbs free energy (G) = -720.034621 Hartree
Charge = 0, Spin = 2
C -1.435576 0.563517 0.360502
C -0.691722 -0.519897 -0.026709
C -1.555465 -1.496575 -0.572759
C -2.817225 -0.964559 -0.492257
N -2.735847 0.281470 0.065155
H -1.130050 1.506754 0.778812
H -1.284307 -2.454839 -0.979096
H -3.767485 -1.371598 -0.793311
C -3.861210 1.143155 0.360648
H -3.552202 2.182573 0.281698
H -4.653486 0.960952 -0.361589
H -4.246185 0.962108 1.363722
I 1.372696 -0.644944 0.140301
Cl 1.599939 2.239525 -0.350519

MesityleneI_Cl_02.log
Energy (E) = -820.703145723 Hartree
Enthalpy (H) = -820.515412 Hartree
Gibbs free energy (G) = -820.573167 Hartree
Charge = 0, Spin = 2
C 2.389914 -1.220365 -0.386497
C 1.020331 -1.058831 -0.575964
C 0.476015 0.178729 -0.234968
C 1.233372 1.229073 0.282642
C 2.596621 1.006929 0.446135
C 3.191860 -0.206341 0.121003
H 2.836955 -2.173607 -0.644716
H 3.207338 1.810298 0.842478
C 0.200457 -2.194298 -1.109495
H -0.301892 -1.918645 -2.037949
H 0.832903 -3.058060 -1.303269
H -0.572523 -2.479554 -0.393877
C 0.643554 2.553307 0.667971
H 0.168417 3.042494 -0.183386
H -0.121176 2.431538 1.436228
H 1.417928 3.213049 1.053732
C 4.660988 -0.423788 0.341892
H 5.222976 0.497469 0.193795
H 4.849975 -0.763088 1.361943
H 5.053830 -1.181119 -0.334861
I -1.597066 0.465379 -0.501153
Cl -2.142875 -1.368963 1.720863

Naph1_Cl_02.log
Energy (E) = -856.368839952 Hartree
Enthalpy (H) = -856.219093 Hartree
Gibbs free energy (G) = -856.271272 Hartree
Charge = 0, Spin = 2
C -2.129649 -2.218596 -0.713087
C -1.047150 -1.390860 -0.592961
C -1.205113 -0.052101 -0.166037
C -2.513081 0.412471 0.150145
C -3.609594 -0.470534 0.013044
C -3.426985 -1.756474 -0.411368
H -1.986895 -3.240843 -1.035028
H -0.056960 -1.767837 -0.803328
C -0.144169 0.877718 -0.016873
C -2.703316 1.740610 0.597570
H -4.598231 -0.101126 0.255767
H -4.272085 -2.423679 -0.510919
C -1.646051 2.593805 0.737576
C -0.342785 2.155936 0.426551
H -3.709427 2.064968 0.832017
H -1.794235 3.606151 1.086185
H 0.493047 2.831196 0.541190
I 1.831622 0.338253 -0.502347
Cl 1.850365 -1.779299 1.535949

Naph2_Cl_02.log
Energy (E) = -856.369240656 Hartree
Enthalpy (H) = -856.219592 Hartree
Gibbs free energy (G) = -856.272913 Hartree
Charge = 0, Spin = 2
C 4.169218 1.521643 -0.285133
C 2.807190 1.600277 -0.377912
C 2.005411 0.455772 -0.154874
C 2.630972 -0.774608 0.168803
C 4.041782 -0.824813 0.257846
C 4.793589 0.295850 0.035695
H 0.113131 1.456172 -0.482899
H 4.775577 2.400583 -0.456379
H 2.320111 2.536108 -0.620771
C 0.593364 0.514980 -0.248860
C 1.824704 -1.914967 0.397318
H 4.513643 -1.767555 0.505838
H 5.871747 0.248575 0.105935
C 0.462278 -1.846317 0.309150
C -0.135021 -0.613022 -0.021380
H 2.304565 -2.852305 0.649720
H -0.148849 -2.718898 0.491622
I -2.239180 -0.545812 -0.174384
Cl -2.366725 2.302152 0.503847

Py_PhI_Cl_02_A.log
Energy (E) = -949.742307755 Hartree
Enthalpy (H) = -949.568360 Hartree
Gibbs free energy (G) = -949.625821 Hartree
Charge = 0, Spin = 2
C 0.866071 1.275259 0.079378
C -0.465154 0.894608 0.240097
C -1.482201 1.826799 0.367913
C -1.176391 3.180319 0.341446
C 0.139163 3.590709 0.182664
C 1.141732 2.644455 0.051304
C 1.993725 0.321315 -0.045785
C 3.008844 -1.594355 0.696274
C 4.077662 -1.437201 -0.174634
C 4.075958 -0.340369 -1.023997
C 3.020091 0.553438 -0.961358
H -2.507726 1.497618 0.459787
H -1.971101 3.906289 0.441490
H 0.385768 4.643182 0.166809
H 2.172735 2.958904 -0.047718
H 2.967821 -2.441351 1.370964
H 4.881370 -2.159153 -0.188013
H 4.880658 -0.187533 -1.730190
H 2.971400 1.407893 -1.621796
N 1.992779 -0.739136 0.762859
I -1.055307 -1.133848 0.225217
Cl -3.705652 -0.579282 -0.861628

Thiophene3I_Cl_02.log
Energy (E) = -1023.60741050 Hartree
Enthalpy (H) = -1023.540998 Hartree
Gibbs free energy (G) = -1023.587790 Hartree
Charge = 0, Spin = 2
C -1.301857 0.994634 -0.020500
C -0.845598 -0.282860 -0.000521
C -1.864961 -1.270644 0.023484
C -3.098503 -0.695691 0.020741
S -3.005055 1.005167 -0.009931
H -0.725287 1.903812 -0.038809
H -1.686306 -2.334231 0.041681
H -4.054683 -1.190283 0.035616
I 1.189496 -0.783451 -0.005347
Cl 2.009963 2.034608 0.015564

5FPhI_Cl2.log
Energy (E) = -1658.95794646 Hartree
Enthalpy (H) = -1658.890152 Hartree
Gibbs free energy (G) = -1658.949032 Hartree
Charge = 0, Spin = 1
C 0.911133 1.027593 -0.625937
C 2.296052 1.030140 -0.626968
C 2.982680 -0.000036 -0.000030
C 2.296063 -1.030204 0.626910
C 0.911135 -1.027658 0.625878
C 0.226287 -0.000033 -0.000014
F 0.265602 -2.005117 1.230204
F 2.963219 -2.000693 1.222575
F 4.299467 -0.000024 -0.000029
F 2.963219 2.000630 -1.222616
F 0.265597 2.005069 -1.230230
I -1.842952 0.000019 0.000023
Cl -1.672735 1.962196 1.557387
Cl -1.673004 -1.962115 -1.557351

Anth_I_Cl2.log
Energy (E) = -1469.98019731 Hartree
Enthalpy (H) = -1469.777971 Hartree
Gibbs free energy (G) = -1469.838602

Hartree
Charge = 0, Spin = 1
C -2.421218 3.589581 -0.589614
C -3.071925 2.413541 -0.399026
C -2.351841 1.201051 -0.196329
C -0.917384 1.222724 -0.195676
C -0.275992 2.480623 -0.388910
C -1.001477 3.614667 -0.582508
C -3.021843 0.000061 -0.000044
C -0.270792 0.000006 -0.000030
C -0.917432 -1.222677 0.195662
C -2.351886 -1.200947 0.196279
C -3.072039 -2.413392 0.399030
H -4.153702 -2.365786 0.396751
C -2.421394 -3.589454 0.589682
C -1.001651 -3.614594 0.582625
C -0.276108 -2.480596 0.388992
H -4.106045 0.000085 -0.000057
H -2.973349 4.506140 -0.742864
H -4.153592 2.366002 -0.396793
H 0.801031 2.543784 -0.368571
H -0.486967 4.554711 -0.726159
H -2.973564 -4.505983 0.742970
H -0.487190 -4.554656 0.726345
H 0.800913 -2.543810 0.368717
I 1.842140 -0.000053 -0.000036
Cl 1.774553 -1.319708 -2.152168
Cl 1.774679 1.319636 2.152212

Furan3I_Cl2.log
Energy (E) = -1160.79564252 Hartree
Enthalpy (H) = -1160.722810 Hartree
Gibbs free energy (G) = -1160.771370
Hartree
Charge = 0, Spin = 1
C 1.950227 -1.027724 0.000158
C 1.125788 0.045556 0.000001
C 1.924977 1.224479 -0.000160
C 3.196441 0.764141 -0.000093
O 3.221505 -0.588294 0.000098
H 1.780203 -2.086848 0.000299
H 1.592254 2.245381 -0.000302
H 4.149000 1.261200 -0.000161
I -0.959415 -0.030978 -0.000003
Cl -1.042996 2.477044 0.000056
Cl -0.817538 -2.542352 -0.000050

IndoleNAc3I_Cl2.log
Energy (E) = -1446.98656374 Hartree
Enthalpy (H) = -1446.810530 Hartree
Gibbs free energy (G) = -1446.871901
Hartree
Charge = 0, Spin = 1
C -0.818078 0.967778 -0.305014
C -2.142281 0.507306 -0.158119
C -3.212616 1.346490 -0.461666
C -2.927686 2.624991 -0.910697
C -1.614609 3.080497 -1.058721
C -0.549321 2.257779 -0.753740
C 0.015593 -0.130216 0.082451
C -0.749855 -1.180302 0.437321
H -4.240295 1.040750 -0.362316
H -3.748726 3.286606 -1.149698
H -1.435293 4.087419 -1.407854
H 0.471990 2.602922 -0.839642
H -0.493495 -2.175578 0.754409
I 2.076423 -0.166730 0.039921
C -3.084144 -1.764116 0.622201
C -4.512105 -1.352648 0.438805
H -4.750754 -0.500778 1.074562
H -4.703185 -1.073037 -0.596428
H -5.132340 -2.199276 0.712709
N -2.079691 -0.821616 0.301313
O -2.758951 -2.851455 1.026027
Cl 2.045788 1.832716 1.585078
Cl 1.964904 -2.174760 -1.480131

Me_Pyrrole_3I_Cl2.log
Energy (E) = -1180.21568425 Hartree
Enthalpy (H) = -1180.100726 Hartree
Gibbs free energy (G) = -1180.152725
Hartree
Charge = 0, Spin = 1
C -1.837945 0.509092 0.179625
C -0.786585 -0.339386 -0.045213
C -1.269635 -1.630714 -0.340187
C -2.635315 -1.522005 -0.281542
N -2.968897 -0.232743 0.026802
H -1.861895 1.558204 0.410829
H -0.694086 -2.510660 -0.559899
H -3.396552 -2.266515 -0.440181
C -4.315072 0.258906 0.240320
H -4.351185 1.320707 0.010768
H -4.999660 -0.264108 -0.423161
H -4.631371 0.106689 1.271376
I 1.223540 0.228923 0.009021
Cl 0.516077 2.651304 -0.110513
Cl 1.891965 -2.186797 0.142661

MesityleneI_Cl2.log
Energy (E) = -1280.82362683 Hartree
Enthalpy (H) = -1280.632709 Hartree
Gibbs free energy (G) = -1280.695181
Hartree
Charge = 0, Spin = 1
C 2.621638 -0.716692 -0.957701
C 1.230682 -0.742156 -0.982863
C 0.588358 0.000892 -0.000555
C 1.231270 0.751168 0.976646
C 2.621627 0.735199 0.943744
C 3.329759 0.008996 -0.007197
H 3.161299 -1.281314 -1.708983
H 3.161602 1.311538 1.685933
C 0.503821 -1.535574 -2.026059
H -0.130987 -0.889842 -2.634943
H 1.213514 -2.032805 -2.682876
H -0.130710 -2.294071 -1.564837
C 0.504417 1.546909 2.018093
H -0.134426 2.300867 1.555417
H -0.126066 0.901517 2.631801
H 1.214185 2.049632 2.670640
C 4.830635 -0.009119 0.008397
H 5.232431 0.939519 0.361213
H 5.196068 -0.790298 0.677211
H 5.233088 -0.208910 -0.983295
I -1.521532 -0.002735 0.002181
Cl -1.418887 2.295836 -1.043841
Cl -1.405939 -2.301637 1.046316

Naph1_Cl2.log
Energy (E) = -1316.48607604 Hartree
Enthalpy (H) = -1316.333215 Hartree
Gibbs free energy (G) = -1316.387839
Hartree
Charge = 0, Spin = 1
C -2.713129 -1.655490 -1.312614
C -1.504862 -1.184737 -0.880198
C -1.427548 0.000748 -0.113886
C -2.631505 0.691166 0.203939
C -3.863961 0.170194 -0.255805
C -3.908145 -0.975445 -0.997867
H -2.753291 -2.563641 -1.897747
H -0.603936 -1.732698 -1.113879
C -0.237578 0.588336 0.370965
C -2.583584 1.884884 0.960332
H -4.771055 0.706204 -0.006379
H -4.854952 -1.364995 -1.345160
C -1.397165 2.405217 1.393978
C -0.190329 1.743550 1.091220
H -3.517131 2.384520 1.186409
H -1.368308 3.320882 1.966704
H 0.753832 2.159410 1.412754
I 1.632832 -0.302068 -0.045936
Cl 0.898252 -2.308173 1.297337
Cl 2.238313 1.784019 -1.328422

Naph2_Cl2.log
Energy (E) = -1316.48809483 Hartree
Enthalpy (H) = -1316.335233 Hartree
Gibbs free energy (G) = -1316.389893
Hartree
Charge = 0, Spin = 1
C 4.630448 1.000533 -0.648378
C 3.298049 1.285818 -0.761138
C 2.329374 0.383134 -0.263174
C 2.751481 -0.821153 0.354145
C 4.136809 -1.087474 0.456216
C 5.053670 -0.198642 -0.033539
H 0.616936 1.585195 -0.820189
H 5.367423 1.694389 -1.028674
H 2.963761 2.203686 -1.227542
C 0.944231 0.657717 -0.370364
C 1.778276 -1.721103 0.850186
H 4.455835 -2.008105 0.928462
H 6.110902 -0.410623 0.048824
C 0.441612 -1.455404 0.739826
C 0.065174 -0.252713 0.121475
H 2.106537 -2.636313 1.326060
H -0.300894 -2.150329 1.102002
I -2.015170 0.174418 -0.032250
Cl -2.414748 -2.247726 -0.597290
Cl -1.531795 2.585001 0.521334

Py_PhI_Cl2.log
Energy (E) = -1409.85998215 Hartree
Enthalpy (H) = -1409.682934 Hartree
Gibbs free energy (G) = -1409.742140
Hartree
Charge = 0, Spin = 1
C -0.625346 1.461277 -0.125107
C 0.669906 1.019225 0.108312
C 1.735576 1.870066 0.332734
C 1.513005 3.239245 0.326465
C 0.231989 3.724360 0.106849
C -0.815369 2.845151 -0.114701
C -1.787560 0.581714 -0.403982
C -2.623750 -1.215955 -1.535475
C -3.878265 -1.063613 -0.961977
C -4.072825 -0.020381 -0.068682
C -3.011369 0.821082 0.215461
H 2.727082 1.474217 0.499454
H 2.338616 3.915182 0.497330
H 0.048392 4.789698 0.098540
H -1.808728 3.223528 -0.317562
H -2.434696 -2.016604 -2.240638
H -4.676337 -1.748875 -1.208611
H -5.031817 0.126635 0.408767
H -3.108065 1.625433 0.931155
N -1.598536 -0.412217 -1.267868
Cl -0.663493 -1.266954 2.008877

I 1.137066 -1.044114 0.254153
Cl 2.949072 -0.658849 -1.452573

Thiophene3I_Cl2.log
Energy (E) = -1483.72506442 Hartree
Enthalpy (H) = -1483.655391 Hartree
Gibbs free energy (G) = -1483.703465 Hartree
Charge = 0, Spin = 1
C -1.745479 0.838437 -0.400536
C -0.908881 -0.139077 0.026497
C -1.537505 -1.315973 0.500519
C -2.889263 -1.189394 0.413391
S -3.354367 0.320840 -0.225427
H -1.499356 1.816004 -0.776170
H -1.019143 -2.187537 0.863323
H -3.632866 -1.913985 0.698018
I 1.186579 0.091963 0.000427
Cl 0.883973 2.587320 0.161101
Cl 1.434809 -2.404140 -0.186993

5FPhI_F2.log
Energy (E) = -938.285641737 Hartree
Enthalpy (H) = -938.217027 Hartree
Gibbs free energy (G) = -938.272511 Hartree
Charge = 0, Spin = 1
C -0.725041 1.188823 0.175514
C -2.110106 1.192525 0.176529
C -2.796888 -0.000027 0.000006
C -2.110071 -1.192552 -0.176492
C -0.725000 -1.188805 -0.175421
C -0.038568 0.000016 0.000026
F -0.080736 -2.322627 -0.369033
F -2.778145 -2.318079 -0.353668
F -4.114680 -0.000035 -0.000054
F -2.778233 2.318030 0.353629
F -0.080834 2.322693 0.369045
I 2.019879 0.000007 -0.000001
F 1.804147 1.251217 -1.563543
F 1.804088 -1.251224 1.563524

Anth_I_F2.log
Energy (E) = -749.306800908 Hartree
Enthalpy (H) = -749.103605 Hartree
Gibbs free energy (G) = -749.160773 Hartree
Charge = 0, Spin = 1
C -3.633669 2.230183 0.226892
C -2.446723 2.884624 0.154965
C -1.218200 2.167440 0.070724
C -1.235635 0.733272 0.060702
C -2.505962 0.087361 0.121592
C -3.654570 0.809890 0.208147
C -0.004938 2.840246 -0.000228
C -0.000197 0.083104 -0.000070
C 1.232979 0.737532 -0.060837
C 1.210639 2.171620 -0.071008
C 2.436722 2.892977 -0.155124
H 2.388322 3.974666 -0.162667
C 3.625906 2.242574 -0.226718
C 3.651665 0.822360 -0.207653
C 2.505510 0.095933 -0.121198
H -0.006805 3.924585 -0.000235
H -4.562395 2.779684 0.292311
H -2.402009 3.966471 0.162378
H -2.562499 -0.987926 0.064444
H -4.603685 0.293640 0.252793
H 4.552763 2.795235 -0.292002
H 4.602551 0.309346 -0.251937
H 2.565675 -0.979146 -0.063707
I 0.003513 -2.014568 -0.000019
F -1.193360 -1.896540 -1.634734
F 1.200106 -1.892256 1.634569

Furan3I_F2.log
Energy (E) = -440.126141284 Hartree
Enthalpy (H) = -440.052421 Hartree
Gibbs free energy (G) = -440.095590 Hartree
Charge = 0, Spin = 1
C 1.723731 -1.090588 0.000329
C 0.939221 0.009625 0.000049
C 1.772620 1.162501 -0.000004
C 3.029213 0.661050 -0.000198
O 3.011072 -0.693197 -0.000006
H 1.507244 -2.141046 0.000471
H 1.457774 2.189095 -0.000041
H 3.998716 1.124233 -0.000372
I -1.115923 -0.001282 -0.000004
F -0.899860 -2.011072 -0.000170
F -0.955371 2.009485 0.000076

IndoleNAc3I_F2.log
Energy (E) = -726.315516988 Hartree
Enthalpy (H) = -726.138317 Hartree
Gibbs free energy (G) = -726.195961 Hartree
Charge = 0, Spin = 1
C -0.608167 1.031886 -0.040435
C -1.928769 0.530099 -0.019315
C -3.015637 1.397780 -0.106741
C -2.759330 2.752876 -0.223998
C -1.455068 3.251629 -0.253399
C -0.371311 2.401594 -0.158498
C 0.239165 -0.123953 0.055709
C -0.515773 -1.236373 0.123474
H -4.036242 1.055529 -0.089196
H -3.593830 3.437034 -0.293370
H -1.293335 4.316537 -0.344772
H 0.641201 2.773868 -0.151374
H -0.238336 -2.273483 0.165107
I 2.299267 -0.212479 -0.005781
C -2.842804 -1.872211 0.151495
C -4.278484 -1.447601 0.089532
H -4.521608 -0.786830 0.920731
H -4.485940 -0.919794 -0.840407
H -4.884143 -2.345739 0.145832
N -1.852335 -0.869588 0.084728
O -2.507662 -3.025596 0.255475
F 2.294449 1.643223 0.802951
F 2.016196 -2.067470 -0.752949

Me_Pyrrole_3I_F2.log
Energy (E) = -459.544923806 Hartree
Enthalpy (H) = -459.429105 Hartree
Gibbs free energy (G) = -459.477314 Hartree
Charge = 0, Spin = 1
C 1.570309 0.721241 0.011692
C 0.603247 -0.246266 -0.003171
C 1.198086 -1.523319 0.000276
C 2.549306 -1.285097 0.015438
N 2.765510 0.065657 0.026476
H 1.487132 1.792222 0.015807
H 0.690890 -2.469965 0.001087
H 3.376523 -1.974195 0.022724
C 4.061838 0.708179 -0.029043
H 4.791214 0.096259 0.496593
H 4.390739 0.848136 -1.058246
H 4.005454 1.677884 0.459268
I -1.421279 0.138298 -0.001196
F -1.649703 -1.867992 -0.009771
F -0.869125 2.089310 0.006401

MesityleneI_F2.log
Energy (E) = -560.149667381 Hartree
Enthalpy (H) = -559.957780 Hartree
Gibbs free energy (G) = -560.014913 Hartree
Charge = 0, Spin = 1
C -2.447861 1.169991 -0.259955
C -1.055432 1.201029 -0.271090
C -0.406985 0.001680 -0.000514
C -1.060245 -1.197491 0.274112
C -2.449503 -1.161953 0.266461
C -3.157474 0.006099 0.002597
H -2.985858 2.087947 -0.465675
H -2.990401 -2.077368 0.477603
C -0.334884 2.482135 -0.566700
H 0.333499 2.362684 -1.421336
H -1.047742 3.268953 -0.802039
H 0.270119 2.789559 0.284703
C -0.340910 -2.478746 0.572189
H 0.265156 -2.787468 -0.277986
H 0.326021 -2.358930 1.427909
H -1.054831 -3.264777 0.806996
C -4.658823 -0.001985 -0.005393
H -5.037439 -0.581780 -0.848216
H -5.051385 -0.457021 0.904018
H -5.058827 1.007241 -0.083607
I 1.685978 -0.001623 -0.001054
F 1.560083 -1.367113 -1.503637
F 1.567424 1.364047 1.501776

Naph1_F2.log
Energy (E) = -595.813411601 Hartree
Enthalpy (H) = -595.659521 Hartree
Gibbs free energy (G) = -595.710674 Hartree
Charge = 0, Spin = 1
C -2.538210 -2.096646 -0.437292
C -1.324565 -1.490225 -0.269266
C -1.245137 -0.100617 -0.013804
C -2.452513 0.650396 0.064131
C -3.689995 -0.015057 -0.102893
C -3.736345 -1.357215 -0.350308
H -2.580112 -3.159436 -0.631901
H -0.421577 -2.079874 -0.296010
C -0.047822 0.636028 0.138653
C -2.404282 2.046040 0.286077
H -4.598703 0.570195 -0.037190
H -4.686752 -1.856288 -0.479945
C -1.213711 2.703740 0.411412
C -0.004596 1.984197 0.332078
H -3.340284 2.587432 0.344213
H -1.184100 3.772235 0.569492
H 0.942813 2.498836 0.395885
I 1.825817 -0.300098 -0.011892
F 2.311692 1.247602 -1.226682
F 1.137584 -1.713354 1.272570

Naph2_F2.log
Energy (E) = -595.818905374 Hartree
Enthalpy (H) = -595.665056 Hartree
Gibbs free energy (G) = -595.716874 Hartree
Charge = 0, Spin = 1
C -4.382500 -1.244145 0.000040
C -3.046848 -1.536581 0.000034

C -2.086057 -0.497669 0.000004
C -2.521687 0.850695 -0.000021
C -3.910384 1.121813 -0.000015
C -4.818730 0.099605 0.000016
H -0.354741 -1.806658 0.000008
H -5.112417 -2.042323 0.000062
H -2.702443 -2.563079 0.000051
C -0.698276 -0.782161 -0.000000
C -1.555144 1.883448 -0.000053
H -4.238485 2.153981 -0.000032
H -5.878540 0.315933 0.000021
C -0.214888 1.608028 -0.000054
C 0.175038 0.258748 -0.000022
H -1.890592 2.913134 -0.000075
H 0.526259 2.391742 -0.000071
I 2.250786 -0.173383 -0.000051
F 1.731044 -2.130436 0.000099
F 2.570752 1.825531 0.000251

Py_PhI_F2.log
Energy (E) = -689.187492446 Hartree
Enthalpy (H) = -689.009522 Hartree
Gibbs free energy (G) = -689.065241 Hartree
Charge = 0, Spin = 1
C -0.402640 1.373352 -0.048437
C 0.876456 0.834100 -0.011971
C 2.022474 1.606250 -0.020858
C 1.900907 2.988439 -0.051473
C 0.640511 3.567625 -0.058687
C -0.490426 2.766692 -0.061198
C -1.654388 0.581071 -0.147756
C -2.781897 -1.113285 -1.179649
C -3.930820 -0.875824 -0.438177
C -3.913481 0.154035 0.490562
C -2.756908 0.900182 0.638397
H 2.992615 1.132078 -0.019695
H 2.790139 3.602618 -0.061426
H 0.536048 4.643604 -0.078156
H -1.474257 3.214923 -0.111862
H -2.758185 -1.908144 -1.915972
H -4.810126 -1.486401 -0.585080
H -4.783987 0.366411 1.096157
H -2.689620 1.695298 1.367748
N -1.667214 -0.402128 -1.044486
F 2.805210 -0.934932 -1.020454
I 1.191301 -1.235698 0.174848
F -0.397409 -1.248323 1.430255

Thiophene3I_F2.log
Energy (E) = -763.056116166 Hartree
Enthalpy (H) = -762.985505 Hartree
Gibbs free energy (G) = -763.030024 Hartree
Charge = 0, Spin = 1
C 1.507486 -1.004631 -0.000103
C 0.715030 0.095224 0.000049
C 1.391916 1.338751 0.000189
C 2.737892 1.135288 0.000070
S 3.138457 -0.521919 -0.000067
H 1.205688 -2.036358 -0.000373
H 0.899571 2.296154 0.000434
H 3.512601 1.882913 0.000121
I -1.362296 -0.047407 -0.000023
F -1.357233 1.973169 -0.000095
F -1.058927 -2.047306 0.000193

5FPhI_OAc2.log
Energy (E) = -1195.34954669 Hartree
Enthalpy (H) = -1195.173810 Hartree
Gibbs free energy (G) = -1195.249091 Hartree
Charge = 0, Spin = 1
C 1.443790 -0.723463 0.951651
C 2.829131 -0.730173 0.956784
C 3.517923 0.000023 -0.000006
C 2.829114 0.730207 -0.956795
C 1.443773 0.723473 -0.951657
C 0.749465 0.000001 0.000000
F 0.802938 1.412693 -1.880938
F 3.495884 1.419931 -1.867002
F 4.837479 0.000034 -0.000010
F 3.495917 -1.419888 1.866987
F 0.802971 -1.412698 1.880934
I -1.338317 -0.000018 0.000007
O -0.922528 -2.087616 -0.459672
C -2.094494 -2.656916 -0.550452
C -2.059936 -4.127511 -0.851828
O -3.126857 -2.016960 -0.397573
H -1.504120 -4.641782 -0.070021
H -3.071527 -4.514182 -0.912859
H -1.534514 -4.291016 -1.790869
O -0.922575 2.087615 0.459599
C -2.094538 2.656940 0.550308
C -2.059972 4.127453 0.852077
O -3.126915 2.017026 0.397339
H -1.500403 4.641595 0.072896
H -3.071483 4.514920 0.909286
H -1.538398 4.290057 1.793441

Anth_I_OAc2.log
Energy (E) = -1006.36382352 Hartree
Enthalpy (H) = -1006.053467 Hartree
Gibbs free energy (G) = -1006.131114 Hartree
Charge = 0, Spin = 1
C 2.915536 3.106435 -1.890925
C 3.567855 2.089385 -1.272347
C 2.849762 1.038593 -0.630587
C 1.415402 1.054050 -0.637136
C 0.773435 2.147523 -1.291834
C 1.494879 3.128151 -1.897457
C 3.522402 -0.000266 -0.000031
C 0.754302 -0.000092 -0.000045
C 1.415262 -1.054344 0.637023
C 2.849623 -1.039057 0.630498
C 3.567581 -2.089937 1.272260
H 4.649568 -2.050248 1.251607
C 2.915129 -3.106927 1.890801
C 1.494474 -3.128494 1.897278
C 0.773152 -2.147766 1.291664
H 4.606775 -0.000326 -0.000008
H 3.466709 3.899597 -2.376690
H 4.649837 2.049568 -1.251666
H -0.304372 2.202744 -1.304755
H 0.979113 3.941706 -2.389059
H 3.466203 -3.900158 2.376565
H 0.978602 -3.942020 2.388816
H -0.304658 -2.202906 1.304496
I -1.359919 0.000138 0.000058
O -1.014442 1.755379 1.244284
C -2.182260 2.240904 1.575189
C -2.113248 3.461177 2.453124
O -3.229810 1.737500 1.198387
H -1.528898 4.233340 1.956161
H -3.115316 3.819999 2.663514
H -1.600701 3.208470 3.379664
O -1.014810 -1.755404 -1.244372
C -2.182805 -2.240532 -1.575056
C -2.114502 -3.460785 -2.453098
O -3.230165 -1.736800 -1.198078
H -1.529957 -4.233085 -1.956580
H -3.116762 -3.819420 -2.662904
H -1.602514 -3.208078 -3.379945

Furan3I_OAc2.log
Energy (E) = -697.181616986 Hartree
Enthalpy (H) = -697.000741 Hartree
Gibbs free energy (G) = -697.065237 Hartree
Charge = 0, Spin = 1
C 1.151330 -2.403885 0.123689
C 0.037635 -1.642919 0.002120
C -1.074840 -2.529062 -0.130117
C -0.537925 -3.768637 -0.075595
O 0.804306 -3.705450 0.077534
H 2.190727 -2.167547 0.235233
H -2.108279 -2.263541 -0.241237
H -0.967716 -4.752097 -0.129201
I -0.018341 0.440542 -0.001472
O 2.139658 0.162588 -0.005949
C 2.677732 1.357362 -0.017742
C 4.181547 1.354755 -0.019824
O 2.001458 2.372785 -0.025994
H 4.541818 0.806304 -0.888290
H 4.549746 2.374989 -0.038928
H 4.543476 0.840114 0.868575
O -2.159122 0.075745 0.015106
C -2.745313 1.247648 0.018464
C -4.247667 1.182445 0.031862
O -2.111591 2.290270 0.010735
H -4.578645 0.628571 0.908483
H -4.657621 2.186758 0.043362
H -4.594082 0.644005 -0.848564

IndoleNAc3I_OAc2.log
Energy (E) = -983.372434511 Hartree
Enthalpy (H) = -983.088358 Hartree
Gibbs free energy (G) = -983.166620 Hartree
Charge = 0, Spin = 1
C 1.403424 0.830965 0.573324
C 2.719276 0.414757 0.285733
C 3.806809 1.103763 0.819326
C 3.549162 2.191573 1.637237
C 2.245491 2.602935 1.927439
C 1.164466 1.929172 1.394844
C 0.534749 -0.078964 -0.116078
C 1.283778 -0.983752 -0.773317
H 4.828311 0.824545 0.623722
H 4.383891 2.734614 2.058630
H 2.086369 3.458098 2.569243
H 0.149333 2.245566 1.591347
H 1.007361 -1.832376 -1.372342
I -1.535333 -0.092511 -0.056497
C 3.607693 -1.533161 -1.141995
C 5.046165 -1.214969 -0.867940
H 5.293355 -0.214472 -1.220961
H 5.255127 -1.262565 0.200063
H 5.647538 -1.948753 -1.393803
N 2.627612 -0.711780 -0.551163
O 3.264156 -2.454850 -1.840544
O -1.209535 1.965998 -0.704816
C -2.390751 2.502023 -0.868891
C -2.360842 3.929416 -1.343066
O -3.421557 1.882380 -0.653145
H -1.787364 4.533814 -0.642695
H -1.856001 3.977233 -2.306295
H -3.373454 4.308985 -1.429979
O -1.180723 -2.155298 0.548899

C -2.354154 -2.695543 0.752354
C -2.305584 -4.140918 1.165334
O -3.391719 -2.064979 0.614053
H -1.793219 -4.719389 0.398756
H -1.731419 -4.234342 2.085322
H -3.313532 -4.515197 1.310062

Me_Pyrrole_3I_OAc2.log
Energy (E) = -716.598655688 Hartree
Enthalpy (H) = -716.375754 Hartree
Gibbs free energy (G) = -716.444647 Hartree
Charge = 0, Spin = 1
C -2.331215 -0.492283 -0.301916
C -1.072516 -0.881581 0.075959
C -1.118900 -2.226252 0.508899
C -2.427158 -2.613490 0.376464
N -3.152118 -1.563808 -0.116313
H -2.695628 0.453392 -0.657946
H -0.293411 -2.827046 0.841006
H -2.899394 -3.557427 0.589252
C -4.585712 -1.554977 -0.314473
H -4.914052 -2.542446 -0.630903
H -5.111038 -1.282345 0.600442
H -4.838427 -0.839896 -1.093531
I 0.617519 0.332034 0.008330
O 1.623714 -1.592150 -0.041375
C 2.911439 -1.363364 -0.099025
C 3.751366 -2.611678 -0.138306
O 3.376051 -0.235362 -0.117586
H 3.457065 -3.220647 -0.991045
H 4.800776 -2.345189 -0.207806
H 3.570751 -3.196818 0.761846
O -0.895511 1.906418 0.057656
C -0.267926 3.054946 0.054502
C -1.181071 4.251746 0.082250
O 0.949132 3.137390 0.030403
H -1.823608 4.198275 0.959195
H -0.591522 5.162365 0.100972
H -1.822150 4.237875 -0.797698

MesityleneI_OAc2.log
Energy (E) = -817.205445632 Hartree
Enthalpy (H) = -816.906499 Hartree
Gibbs free energy (G) = -816.984315 Hartree
Charge = 0, Spin = 1
C -3.157867 -0.471398 1.094458
C -1.766672 -0.490362 1.121041
C -1.106775 0.004004 0.000294
C -1.765116 0.507645 -1.117374
C -3.156243 0.503494 -1.086091
C -3.867140 0.016888 0.004101
H -3.697928 -0.847962 1.955764
H -3.695043 0.895198 -1.941422
C -1.048366 -1.028568 2.323249
H -0.394618 -0.273509 2.761948
H -1.762594 -1.341853 3.081562
H -0.430779 -1.885012 2.052442
C -1.045154 1.046642 -2.318250
H -0.421692 1.897997 -2.044951
H -0.396916 0.289201 -2.761003
H -1.758520 1.367300 -3.074289
C -5.368590 -0.003027 -0.006126
H -5.769784 0.815196 -0.602470
H -5.737283 -0.935689 -0.436835
H -5.770849 0.074521 1.002924
I 1.003501 -0.003294 -0.001048
O 0.657580 -2.148755 -0.217691
C 1.823858 -2.735841 -0.302659
C 1.747514 -4.230455 -0.466543
O 2.874339 -2.115892 -0.251729
H 1.167069 -4.468556 -1.355950
H 2.748004 -4.642523 -0.546312
H 1.229294 -4.662473 0.387909
O 0.673980 2.144584 0.217666
C 1.844772 2.722787 0.301474
C 1.779955 4.217728 0.467220
O 2.890476 2.095007 0.248014
H 1.201052 4.459327 1.356674
H 2.783590 4.621948 0.547638
H 1.265387 4.654713 -0.386928

Naph1_OAc2.log
Energy (E) = -852.869999692 Hartree
Enthalpy (H) = -852.609051 Hartree
Gibbs free energy (G) = -852.680680 Hartree
Charge = 0, Spin = 1
C -3.118217 0.699152 1.982929
C -1.949062 0.504240 1.301942
C -1.901827 -0.346286 0.172298
C -3.100792 -0.989110 -0.244740
C -4.295222 -0.758632 0.478490
C -4.308148 0.064362 1.568383
H -3.132729 1.350310 2.846143
H -1.051270 1.011905 1.623082
C -0.737711 -0.619976 -0.585016
C -3.084644 -1.851452 -1.365539
H -5.198881 -1.254946 0.146976
H -5.225605 0.232438 2.115387
C -1.931750 -2.086847 -2.058762
C -0.733828 -1.459244 -1.660034
H -4.012117 -2.325894 -1.661181
H -1.924762 -2.750085 -2.912103
H 0.183176 -1.655273 -2.197296
I 1.118096 0.235253 -0.065376
O 0.019344 2.058875 -0.537934
C 0.846375 3.061588 -0.395819
C 0.243289 4.404514 -0.707467
O 2.005143 2.906601 -0.041539
H -0.628169 4.566722 -0.075844
H 0.979531 5.184737 -0.545351
H -0.096044 4.414643 -1.741751
O 1.613127 -1.867468 0.231918
C 2.861591 -1.921702 0.616785
C 3.368337 -3.316596 0.865800
O 3.545464 -0.919330 0.757834
H 3.260533 -3.909066 -0.040937
H 4.408762 -3.280121 1.171274
H 2.763543 -3.787275 1.638801

Naph2_OAc2.log
Energy (E) = -852.871119862 Hartree
Enthalpy (H) = -852.610232 Hartree
Gibbs free energy (G) = -852.682169 Hartree
Charge = 0, Spin = 1
C -5.200194 0.657402 -0.956842
C -3.867788 0.929365 -1.099044
C -2.902344 0.185765 -0.379971
C -3.330563 -0.846342 0.492176
C -4.715794 -1.103941 0.618956
C -5.628519 -0.370761 -0.088034
H -1.189101 1.245178 -1.164732
H -5.932941 1.229704 -1.509135
H -3.530271 1.716398 -1.761667
C -1.517499 0.447362 -0.512875
C -2.361503 -1.587678 1.209655
H -5.038269 -1.893225 1.286668
H -6.685429 -0.575444 0.015455
C -1.027121 -1.325824 1.073335
C -0.628263 -0.296854 0.199767
H -2.694503 -2.373363 1.876123
H -0.287949 -1.901105 1.609872
I 1.431193 0.114496 0.008143
O 0.718298 2.162254 0.241399
C 1.765466 2.946543 0.206211
C 1.441602 4.408648 0.354332
O 2.899202 2.516551 0.064834
H 0.773533 4.715062 -0.448720
H 0.919032 4.570024 1.295377
H 2.356219 4.991483 0.325214
O 1.493778 -2.056859 -0.172526
C 2.744882 -2.399182 -0.347701
C 2.959976 -3.881160 -0.495493
O 3.647248 -1.578038 -0.385577
H 2.599836 -4.390347 0.396745
H 2.379391 -4.248500 -1.339695
H 4.014949 -4.085455 -0.644955

Py_PhI_OAc2.log
Energy (E) = -946.244313795 Hartree
Enthalpy (H) = -945.959311 Hartree
Gibbs free energy (G) = -946.035111 Hartree
Charge = 0, Spin = 1
C -0.946171 1.807643 -0.078897
C 0.303975 1.409684 0.386284
C 1.190497 2.302061 0.963751
C 0.840003 3.639325 1.083575
C -0.400408 4.065617 0.633448
C -1.277508 3.156385 0.064483
C -1.936746 0.900763 -0.713110
C -2.352751 -0.784370 -2.201359
C -3.706633 -0.800269 -1.895208
C -4.177109 0.101566 -0.950771
C -3.279992 0.965740 -0.346439
H 2.152865 1.956545 1.311677
H 1.533863 4.337165 1.530796
H -0.684472 5.105301 0.719690
H -2.237491 3.490397 -0.307651
H -1.946227 -1.469424 -2.935815
H -4.368433 -1.499687 -2.385414
H -5.224266 0.121776 -0.680155
H -3.598488 1.659433 0.419010
N -1.486949 0.049974 -1.632185
O 2.689244 0.329895 -0.567912
C 3.554554 -0.618411 -0.816209
C 4.826623 -0.135276 -1.459456
O 3.337793 -1.789502 -0.545661
H 5.299725 0.606567 -0.818534
H 5.495883 -0.973318 -1.623826
H 4.589998 0.350360 -2.404459
I 0.936909 -0.606659 0.327776
O -0.993931 -0.872841 1.292171
C -1.273406 -2.150579 1.296046
C -2.644262 -2.470532 1.829588
O -0.489064 -2.995265 0.894765
H -3.389601 -1.996474 1.190720
H -2.792571 -3.545241 1.846619
H -2.754666 -2.054652 2.829241

Thiophene3I_OAc2.log
Energy (E) = -1020.10956470 Hartree
Enthalpy (H) = -1019.931862 Hartree
Gibbs free energy (G) = -1019.996744 Hartree
Charge = 0, Spin = 1
C -2.236093 -0.818209 -0.533559

C -1.018869 -1.018087 0.033234
C -0.865812 -2.281071 0.662254
C -2.002038 -3.019749 0.546401
S -3.221130 -2.185917 -0.305362
H -2.597592 0.059161 -1.038392
H 0.036882 -2.612079 1.146694
H -2.180367 -4.013226 0.920762
I 0.494230 0.432789 0.006938
O 1.745469 -1.348792 -0.056556
C 2.989428 -0.942148 -0.101762
C 3.998171 -2.056661 -0.158405
O 3.287623 0.241353 -0.096812
H 3.798265 -2.682988 -1.025747
H 4.999228 -1.642197 -0.213514
H 3.897720 -2.680695 0.728005
O -1.225522 1.771416 0.053856
C -0.758903 2.994846 0.079656
C -1.823003 4.057802 0.109667
O 0.437676 3.235183 0.078330
H -2.468373 3.901304 0.971916
H -1.360282 5.037958 0.155937
H -2.440843 3.975989 -0.783035

5FPhI_OTFA2.log
Energy (E) = -1790.57632116 Hartree
Enthalpy (H) = -1790.441852 Hartree
Gibbs free energy (G) = -1790.529075 Hartree
Charge = 0, Spin = 1
C 0.580151 1.820523 1.051468
C 0.585777 3.205458 1.055077
C 0.001622 3.892428 0.000008
C -0.583168 3.205985 -1.055013
C -0.578876 1.821018 -1.051389
C 0.000293 1.132661 0.000039
F -1.125737 1.173889 -2.063696
F -1.131611 3.872866 -2.052848
F 0.002206 5.208867 -0.000008
F 1.134782 3.871863 2.052906
F 1.126311 1.172904 2.063853
I -0.000398 -0.942990 0.000251
O 2.137923 -0.566558 -0.158246
C 2.718024 -1.723114 -0.164744
O 2.150797 -2.791363 -0.105320
O -2.138592 -0.565312 0.158460
C -2.719288 -1.721554 0.164846
O -2.152590 -2.790123 0.105832
C 4.257538 -1.610382 -0.259833
F 4.597578 -0.962804 -1.371531
F 4.727754 -0.929641 0.782281
F 4.815861 -2.806236 -0.275317
C -4.258774 -1.608073 0.259256
F -4.598892 -0.959829 1.370595
F -4.728278 -0.927591 -0.783289
F -4.817642 -2.803667 0.275120

Anth_I_OTFA2.log
Energy (E) = -1601.59674051 Hartree
Enthalpy (H) = -1601.327707 Hartree
Gibbs free energy (G) = -1601.417980 Hartree
Charge = 0, Spin = 1
C 2.145460 -3.297383 2.937772
C 1.442664 -3.947718 1.975566
C 0.718296 -3.227276 0.982420
C 0.730302 -1.792993 0.998514
C 1.487787 -1.152754 2.022445
C 2.161984 -1.877471 2.955160
C -0.000027 -3.897181 0.000002
C -0.000008 -1.141570 -0.000006
C -0.730333 -1.792992 -0.998519
C -0.718347 -3.227276 -0.982417
C -1.442732 -3.947711 -1.975556
H -1.414192 -5.029361 -1.938023
C -2.145524 -3.297371 -2.937760
C -2.162038 -1.877459 -2.955153
C -1.487832 -1.152747 -2.022442
H -0.000035 -4.981385 0.000009
H 2.693412 -3.849391 3.688283
H 1.414108 -5.029368 1.938033
H 1.536597 -0.075190 2.062088
H 2.726508 -1.363051 3.720472
H -2.693483 -3.849379 -3.688267
H -2.726566 -1.363040 -3.720462
H -1.536634 -0.075184 -2.062085
I 0.000010 0.966710 -0.000002
O -2.118241 0.671364 0.441331
C -2.697007 1.825960 0.505563
O -2.166510 2.904511 0.367266
O 2.118274 0.671303 -0.441367
C 2.697034 1.825900 -0.505604
O 2.166530 2.904453 -0.367304
C 4.214745 1.692718 -0.776602
F 4.787766 2.881086 -0.855697
F 4.427711 1.040664 -1.917299
F 4.793704 1.007185 0.209467
C -4.214712 1.692764 0.776603
F -4.427615 1.040885 1.917414
F -4.793687 1.007056 -0.209331
F -4.787762 2.881128 0.855528

Furan3I_OTFA2.log
Energy (E) = -1292.41206842 Hartree
Enthalpy (H) = -1292.272468 Hartree
Gibbs free energy (G) = -1292.348685 Hartree
Charge = 0, Spin = 1
C -1.060809 2.677844 0.331506
C -0.002923 1.898949 0.003953
C 1.083508 2.753083 -0.349681
C 0.589248 4.002789 -0.201954
O -0.700241 3.966694 0.206717
H -2.067721 2.463547 0.632689
H 2.073958 2.467548 -0.648930
H 1.019373 4.977128 -0.343351
I 0.006269 -0.177011 -0.006708
O -2.146291 0.128424 -0.022231
C -2.724206 -1.032148 -0.019860
O -2.167050 -2.104146 -0.023496
O 2.152738 0.135026 0.036615
C 2.735351 -1.022691 0.004764
O 2.183198 -2.096578 -0.035310
C 4.276407 -0.888896 0.031441
F 4.854535 -2.069145 -0.090320
F 4.687338 -0.105703 -0.964974
F 4.663430 -0.337732 1.180104
C -4.265879 -0.904308 -0.015893
F -4.838461 -2.091785 0.044947
F -4.662143 -0.183037 1.032183
F -4.673683 -0.287013 -1.122930

IndoleNAc3I_OTFA2.log
Energy (E) = -1578.60392417 Hartree
Enthalpy (H) = -1578.361189 Hartree
Gibbs free energy (G) = -1578.451796 Hartree
Charge = 0, Spin = 1
C -0.745270 1.815499 0.732655
C -0.415368 3.136892 0.369560
C -1.005647 4.213238 1.029303
C -1.909107 3.937342 2.041614
C -2.236900 2.627251 2.402365
C -1.661680 1.556852 1.748293
C 0.025341 0.970362 -0.131192
C 0.767988 1.732658 -0.958584
H -0.787801 5.238795 0.783759
H -2.374874 4.762789 2.561722
H -2.952169 2.454632 3.193842
H -1.924567 0.538370 1.999809
H 1.481385 1.472517 -1.720055
I 0.086833 -1.089246 -0.083165
C 1.193828 4.062711 -1.422775
C 0.925149 5.493835 -1.075253
H -0.127283 5.733400 -1.223145
H 1.179938 5.692831 -0.035093
H 1.535633 6.107844 -1.728530
N 0.522917 3.064908 -0.676446
O 1.948450 3.727401 -2.299647
O -2.066205 -0.832920 -0.338923
C -2.614694 -2.004334 -0.359554
O -2.038042 -3.064966 -0.281673
O 2.226752 -0.740881 0.156732
C 2.811185 -1.891451 0.253148
O 2.266409 -2.971565 0.230832
C -4.154428 -1.915536 -0.480525
F -4.496747 -1.252768 -1.582214
F -4.696899 -3.119064 -0.523364
F -4.651137 -1.261741 0.570595
C 4.344695 -1.750809 0.403325
F 4.919827 -2.935982 0.501442
F 4.850715 -1.116927 -0.653068
F 4.636206 -1.043417 1.493017

Me_Pyrrole_3I_OTFA2.log
Energy (E) = -1311.83183235 Hartree
Enthalpy (H) = -1311.650304 Hartree
Gibbs free energy (G) = -1311.732661 Hartree
Charge = 0, Spin = 1
C 0.002032 2.591003 0.785538
C -0.000883 1.705040 -0.263479
C 0.008654 2.427818 -1.477416
C 0.016035 3.747896 -1.117939
N 0.014128 3.836040 0.250861
H -0.002255 2.426067 1.848662
H 0.015395 2.029031 -2.475853
H 0.025022 4.636694 -1.724986
C -0.025662 5.073599 1.006183
H 0.698622 5.776585 0.601335
H -1.018489 5.518518 0.969147
H 0.231312 4.865732 2.041142
I -0.000368 -0.330871 -0.047405
O 2.177539 -0.079799 -0.073242
C 2.721224 -1.243653 0.059340
O 2.141365 -2.299901 0.176867
O -2.178134 -0.080421 -0.074270
C -2.721754 -1.244400 0.057666
O -2.141883 -2.300630 0.175131
C 4.266638 -1.161889 0.056468
F 4.687956 -0.386103 1.055199
F 4.698446 -0.634673 -1.088311
F 4.806411 -2.360037 0.198379
C -4.267176 -1.162750 0.053962
F -4.698370 -0.635939 -1.091256
F -4.689155 -0.386658 1.052165
F -4.806929 -2.360890 0.195956

MesityleneI_OTFA2.log
Energy (E) = -1412.43948713 Hartree
Enthalpy (H) = -1412.181789 Hartree

Gibbs free energy (G) = -1412.271365 Hartree
Charge = 0, Spin = 1
C -0.496616 3.497983 -1.081621
C -0.511282 2.107062 -1.116244
C 0.003282 1.455103 0.000241
C 0.517355 2.103296 1.119947
C 0.506047 3.493623 1.089002
C 0.008326 4.204661 0.003024
H -0.895300 4.039458 -1.931417
H 0.896991 4.032117 1.944369
C -1.072736 1.394096 -2.310693
H -1.956801 0.816575 -2.039996
H -1.354852 2.111970 -3.077013
H -0.342404 0.708649 -2.742424
C 1.069007 1.385179 2.315865
H 0.332077 0.704983 2.744687
H 1.949574 0.801184 2.047590
H 1.354289 2.100346 3.083547
C 0.038258 5.705131 -0.005894
H -0.094776 6.107644 0.997119
H 0.998440 6.064878 -0.379584
H -0.740030 6.111717 -0.649357
I -0.002224 -0.652464 -0.000644
O 2.156352 -0.361140 -0.154901
C 2.748742 -1.511795 -0.137756
O 2.215802 -2.595782 -0.090176
O -2.159541 -0.352490 0.155254
C -2.756561 -1.500715 0.136291
O -2.227969 -2.586727 0.086283
C 4.288041 -1.357744 -0.166762
F 4.669746 -0.693911 -1.255638
F 4.692380 -0.668860 0.902508
F 4.884933 -2.536047 -0.158223
C -4.295207 -1.340480 0.166317
F -4.697155 -0.648446 -0.901846
F -4.896894 -2.516332 0.156339
F -4.673723 -0.676650 1.256265

Naph1_OTFA2.log
Energy (E) = -1448.10255782 Hartree
Enthalpy (H) = -1447.882919 Hartree
Gibbs free energy (G) = -1447.966578 Hartree
Charge = 0, Spin = 1
C 2.155859 2.675426 2.121140
C 1.437349 1.769347 1.392146
C 0.809381 2.150677 0.184282
C 0.946103 3.495548 -0.261377
C 1.702434 4.404591 0.515323
C 2.294457 4.008196 1.680519
H 2.628447 2.365275 3.042639
H 1.358796 0.749508 1.739679
C 0.037357 1.298939 -0.640065
C 0.323638 3.908101 -1.462297
H 1.797826 5.423772 0.162601
H 2.870259 4.710237 2.267206
C -0.416528 3.040517 -2.214392
C -0.563468 1.702998 -1.795789
H 0.446198 4.937673 -1.774087
H -0.892057 3.364608 -3.128744
H -1.157665 1.015199 -2.380093
I -0.286030 -0.707966 -0.092825
O 1.882742 -0.803319 -0.315636
C 2.273513 -2.012632 -0.073408
O 1.566526 -2.949769 0.218208
O -2.360868 -0.034734 -0.031792
C -3.113483 -1.039755 0.280931
O -2.742871 -2.165838 0.520776
C 3.810321 -2.152634 -0.186090
F 4.191827 -3.393598 0.059695
F 4.217773 -1.815279 -1.407490
F 4.405626 -1.343467 0.690217
C -4.609979 -0.647473 0.314476
F -4.813723 0.317521 1.208824
F -4.990250 -0.194547 -0.880132
F -5.364383 -1.684614 0.631740

Naph2_OTFA2.log
Energy (E) = -1448.10362686 Hartree
Enthalpy (H) = -1447.884039 Hartree
Gibbs free energy (G) = -1447.968025 Hartree
Charge = 0, Spin = 1
C 3.804331 -4.178978 0.993015
C 3.229915 -2.946743 1.136230
C 2.076107 -2.605112 0.392251
C 1.520182 -3.551827 -0.505106
C 2.137488 -4.818128 -0.631099
C 3.251818 -5.124060 0.100271
H 1.890361 -0.607686 1.199104
H 4.686709 -4.435376 1.562855
H 3.647550 -2.215168 1.816102
C 1.461159 -1.337449 0.526642
C 0.366650 -3.205841 -1.248947
H 1.710428 -5.538810 -1.316996
H 3.717210 -6.094723 -0.002438
C -0.219752 -1.978191 -1.118991
C 0.355130 -1.064980 -0.217293
H -0.051164 -3.933229 -1.933067
H -1.107105 -1.720457 -1.676750
I -0.537702 0.836039 -0.028057
O 1.532980 1.506254 -0.156089
C 1.562938 2.797502 -0.060129
O 0.609237 3.530895 0.055370
O -2.352415 -0.366702 0.056218
C -3.374074 0.421455 0.167913
O -3.345114 1.627730 0.236234
C -4.703416 -0.369807 0.199418
F -5.737367 0.445057 0.305163
F -4.835478 -1.082511 -0.919710
F -4.715027 -1.211084 1.231064
C 3.012042 3.338529 -0.101671
F 3.029907 4.654360 0.010329
F 3.721908 2.816391 0.899339
F 3.598679 2.999089 -1.247372

Py_PhI_OTFA2.log
Energy (E) = -1541.47434403 Hartree
Enthalpy (H) = -1541.230766 Hartree
Gibbs free energy (G) = -1541.318902 Hartree
Charge = 0, Spin = 1
C -0.346974 1.849129 -1.040233
C 0.040741 0.538520 -1.288435
C 0.447716 0.091278 -2.532037
C 0.483615 0.988844 -3.587022
C 0.121306 2.312348 -3.375651
C -0.289818 2.731219 -2.121335
C -0.872626 2.317045 0.260487
C -2.243524 1.846379 2.035380
C -1.989137 3.078400 2.623483
C -1.120907 3.950186 1.981779
C -0.549320 3.568583 0.778094
H 0.729848 -0.940962 -2.677251
H 0.793254 0.651649 -4.565706
H 0.143242 3.017411 -4.194819
H -0.611692 3.752824 -1.967679
H -2.909162 1.128022 2.499089
H -2.456387 3.341708 3.561308
H -0.886549 4.912348 2.416178
H 0.147882 4.211211 0.258644
N -1.699026 1.476707 0.880903
O 2.403273 -0.244498 -0.113901
C 3.186078 -0.921831 0.656599
O 2.858386 -1.758429 1.469207
C 4.672483 -0.551212 0.435632
F 4.865614 0.746713 0.669089
F 5.026931 -0.802005 -0.824793
F 5.461470 -1.244191 1.239322
I 0.324468 -0.867260 0.269824
O -1.728488 -1.410437 0.612399
C -2.507568 -1.474726 -0.420945
O -2.211091 -1.397819 -1.586756
C -3.977583 -1.661228 0.030713
F -4.364465 -0.619712 0.771824
F -4.107533 -2.762118 0.769652
F -4.788416 -1.755856 -1.009917

Thiophene3I_OTFA2.log
Energy (E) = -1615.34099369 Hartree
Enthalpy (H) = -1615.204600 Hartree
Gibbs free energy (G) = -1615.281630 Hartree
Charge = 0, Spin = 1
C 0.987311 2.461407 -0.562599
C 0.004260 1.744050 0.039048
C -0.984770 2.515197 0.700006
C -0.711975 3.841493 0.570239
S 0.711365 4.120432 -0.324870
H 1.849703 2.102137 -1.094871
H -1.840564 2.108591 1.211642
H -1.281434 4.667984 0.959422
I -0.027707 -0.347859 0.015914
O -2.175531 -0.007071 -0.039080
C -2.776799 -1.154979 -0.045989
O -2.244942 -2.239963 -0.037042
O 2.133407 -0.087831 0.056815
C 2.692141 -1.257086 0.038483
O 2.120942 -2.321789 0.025538
C -4.315399 -0.993127 -0.064086
F -4.694645 -0.339106 -1.159729
F -4.915013 -2.169060 -0.042016
F -4.712100 -0.291225 0.997584
C 4.235771 -1.152301 0.032838
F 4.790779 -2.348265 -0.033350
F 4.640577 -0.434799 -1.015383
F 4.657589 -0.544633 1.139651

00naph_02.log
Energy (E) = -384.840558092 Hartree
Enthalpy (H) = -384.698061 Hartree
Gibbs free energy (G) = -384.737677 Hartree
Charge = 0, Spin = 2
C -2.382451 0.793763 0.000001
C -1.228329 1.489259 0.000001
C -2.515021 -0.591815 -0.000002
C -0.017148 0.739888 -0.000001
H -1.189007 2.572130 0.000001
C -1.351630 -1.321057 -0.000000
H -3.482572 -1.075118 0.000001
C -0.087970 -0.678860 -0.000000
C 1.251656 1.365702 0.000000
H -1.382629 -2.404543 0.000002
C 1.117354 -1.421354 0.000001
C 2.398078 0.619548 -0.000001
H 1.295975 2.447907 -0.000001
C 2.330902 -0.791006 -0.000000
H 1.058046 -2.503056 0.000001

H 3.363128 1.108268 -0.000000
H 3.244406 -1.370001 0.000000

FuranIBA_A_naph.log
Energy (E) = -813.299971985 Hartree
Enthalpy (H) = -813.082413 Hartree
Gibbs free energy (G) = -813.144865 Hartree
Charge = 0, Spin = 1
C 2.111952 2.952516 -0.205878
C 1.086108 2.076695 -0.035937
C 1.731310 0.812777 -0.043312
C 3.058033 0.993238 -0.210732
O 3.298166 2.312556 -0.311717
H 2.141058 4.025418 -0.268712
H 0.040506 2.305017 0.073170
C 4.074257 -0.110337 -0.278327
I 1.093762 -1.141644 0.140315
O 5.254388 0.126972 -0.430752
O 3.472291 -1.242699 -0.151941
C -0.936278 -0.517339 0.367275
C -1.793470 -0.613170 -0.691825
C -1.364238 -0.012041 1.614527
C -3.143202 -0.206391 -0.553638
H -1.460439 -0.993427 -1.650676
C -2.661356 0.393233 1.766183
H -0.667407 0.055720 2.440004
C -3.582202 0.305772 0.693788
C -4.060018 -0.293006 -1.627546
H -3.004216 0.785097 2.715770
C -4.929494 0.714851 0.829997
C -5.356602 0.110330 -1.466559
H -3.715362 -0.684187 -2.576620
C -5.795628 0.619351 -0.224179
H -5.260640 1.104306 1.784577
H -6.052804 0.041307 -2.291039
H -6.823894 0.934364 -0.110916

IBA_oMe_naph.log
Energy (E) = -854.767601779 Hartree
Enthalpy (H) = -854.490277 Hartree
Gibbs free energy (G) = -854.556538 Hartree
Charge = 0, Spin = 1
C 2.050745 2.960498 -0.515276
C 3.364310 2.917820 -0.073344
C 3.952469 1.699704 0.223461
C 3.206834 0.532598 0.128866
C 1.877540 0.632864 -0.228829
C 1.257312 1.814197 -0.621551
H 1.614278 3.903725 -0.820465
H 3.937469 3.832418 -0.006191
H 4.990951 1.598041 0.507919
C 3.909699 -0.814935 0.279917
O 3.185226 -1.799794 -0.099067
O 5.058679 -0.828149 0.683194
I 0.888233 -1.277192 -0.105990
C -0.106218 1.948333 -1.240866
H -0.336591 1.101089 -1.885493
H -0.904344 2.026982 -0.502489
H -0.126226 2.849938 -1.850564
C -1.069463 -0.504764 0.250003
C -2.089032 -0.781986 -0.613931
C -1.296670 0.208401 1.446271
C -3.403465 -0.337280 -0.331071
H -1.912649 -1.324831 -1.536235
C -2.554758 0.661243 1.731818
H -0.474096 0.405170 2.122648
C -3.637617 0.403590 0.855602
C -4.484204 -0.599687 -1.205894
H -2.742597 1.219427 2.640697
C -4.948262 0.858451 1.131964
C -5.740658 -0.146626 -0.912851
H -4.296741 -1.165767 -2.109872
C -5.975118 0.590316 0.269184
H -5.121865 1.421664 2.040443
H -6.561964 -0.351317 -1.585865
H -6.973892 0.941760 0.489081

IBamideAc_naph.log
Energy (E) = -948.146131081 Hartree
Enthalpy (H) = -947.845027 Hartree
Gibbs free energy (G) = -947.918574 Hartree
Charge = 0, Spin = 1
C 0.478762 3.546889 -0.057406
C 1.815545 3.907227 -0.180739
C 2.796746 2.930857 -0.214478
C 2.464968 1.581330 -0.123673
C 1.128441 1.265634 0.001837
C 0.119015 2.207604 0.036503
H -0.293310 4.303538 -0.033807
H 2.088262 4.951043 -0.252716
H 3.848645 3.164179 -0.312825
H -0.919984 1.927143 0.128789
C 3.580785 0.557435 -0.179192
O 4.726569 0.949960 -0.277383
I 0.673972 -0.811089 0.149706
N 3.085392 -0.704272 -0.105818
C 3.134391 -3.127669 -0.024237
H 3.834249 -3.956463 -0.067748
H 2.594203 -3.160930 0.924345
H 2.407183 -3.227867 -0.832886
C 3.901994 -1.824019 -0.156110
O 5.104283 -1.825424 -0.292712
C -1.433766 -0.448794 0.367584
C -2.250990 -0.555504 -0.723013
C -1.963066 -0.165618 1.646743
C -3.650347 -0.380044 -0.590116
H -1.846452 -0.770029 -1.706110
C -3.310346 0.012681 1.797955
H -1.301322 -0.086107 2.499992
C -4.186984 -0.089520 0.690197
C -4.523504 -0.482966 -1.698625
H -3.728068 0.232804 2.772715
C -5.584080 0.088230 0.822929
C -5.870089 -0.305677 -1.540264
H -4.104746 -0.704655 -2.672374
C -6.405769 -0.017132 -0.265400
H -5.989267 0.308714 1.802662
H -6.532039 -0.385877 -2.391562
H -7.472668 0.120212 -0.154403

IBCCF32O_naph.log
Energy (E) = -1415.29861557 Hartree
Enthalpy (H) = -1415.016056 Hartree
Gibbs free energy (G) = -1415.095067 Hartree
Charge = 0, Spin = 1
C -0.125077 3.217440 0.099187
C -1.497174 3.422912 0.106929
C -2.367662 2.345488 0.041913
C -1.880200 1.042092 -0.039386
C -0.511904 0.876026 -0.031128
C 0.381550 1.925857 0.036494
H 0.556475 4.055491 0.142739
H -1.894166 4.426947 0.162608
H -3.433800 2.512594 0.055525
H 1.448734 1.757531 0.032859
O -2.052191 -1.300755 -0.459091
I 0.182523 -1.140516 -0.100022
C -2.766076 -0.209474 -0.134697
C -3.843282 -0.013772 -1.233089
C -3.431128 -0.429000 1.249158
F -4.775259 0.900369 -0.913893
F -3.258679 0.393113 -2.362213
F -4.478515 -1.147917 -1.486588
F -4.106819 0.636196 1.700136
F -4.265777 -1.460456 1.236809
F -2.467603 -0.688518 2.143370
C 2.212168 -0.547188 0.246634
C 3.090694 -0.511120 -0.800013
C 2.643455 -0.268276 1.563481
C 4.454346 -0.193640 -0.583018
H 2.761790 -0.720348 -1.812045
C 3.952842 0.048011 1.796362
H 1.933992 -0.302838 2.380663
C 4.890714 0.092625 0.735517
C 5.388140 -0.150316 -1.644866
H 4.294177 0.265514 2.801037
C 6.251421 0.412639 0.952058
C 6.697769 0.162602 -1.405737
H 5.045560 -0.370221 -2.648388
C 7.133724 0.446931 -0.092698
H 6.580233 0.629015 1.960934
H 7.406475 0.193284 -2.221952
H 8.172277 0.692288 0.082681

IBCH2S_naph.log
Energy (E) = -1142.95665884 Hartree
Enthalpy (H) = -1142.634239 Hartree
Gibbs free energy (G) = -1142.703038 Hartree
Charge = 0, Spin = 1
C -1.008378 3.057021 -0.976043
C -2.342045 3.322961 -0.713950
C -3.185792 2.311029 -0.275450
C -2.736163 1.002413 -0.090923
C -1.390690 0.790086 -0.352960
C -0.521673 1.768824 -0.786757
H -0.346975 3.832865 -1.335291
H -2.735250 4.320313 -0.857162
H -4.222455 2.541111 -0.076679
H 0.514070 1.539960 -0.993890
I -0.642109 -1.209372 -0.128867
C -3.662352 -0.097989 0.412678
S -3.291597 -1.625140 -0.495449
C -5.131330 0.232522 0.164661
H -5.729801 -0.637189 0.433214
H -5.466583 1.072044 0.779181
H -5.308387 0.458667 -0.886517
C -3.453909 -0.269756 1.920614
H -3.704098 0.655573 2.447204
H -4.098471 -1.071087 2.281395
H -2.422971 -0.531560 2.160073
C 1.366521 -0.395768 0.225871
C 2.377563 -0.719448 -0.631982
C 1.627251 0.350774 1.396855
C 3.710395 -0.312136 -0.364853
H 2.183912 -1.289820 -1.535313
C 2.900441 0.762640 1.674428
H 0.813586 0.601147 2.068168
C 3.974060 0.442716 0.805757
C 4.779446 -0.631171 -1.233947
H 3.110387 1.338371 2.568027
C 5.300808 0.853390 1.072017
C 6.053645 -0.220319 -0.951490
H 4.569406 -1.207795 -2.126491
C 6.317311 0.530278 0.214833
H 5.496128 1.428775 1.968699

H 6.865474 -0.469715 -1.621218
H 7.328728 0.848900 0.427570

IBNMeCO2_naph.log
Energy (E) = -910.086145910 Hartree
Enthalpy (H) = -909.790268 Hartree
Gibbs free energy (G) = -909.859156 Hartree
Charge = 0, Spin = 1
C -1.020729 2.950038 -1.375001
C -2.253269 3.315893 -0.849580
C -3.049321 2.396067 -0.190071
C -2.651755 1.059921 -0.015896
C -1.391003 0.746497 -0.532604
C -0.587348 1.643826 -1.207666
H -0.402473 3.664281 -1.898928
H -2.609426 4.330614 -0.965484
H -4.013736 2.701277 0.188506
H 0.378714 1.333011 -1.582919
I -0.650854 -1.190076 -0.187232
C -3.745918 -1.155080 0.111601
O -4.749431 -1.711067 0.512383
N -3.452183 0.152918 0.663753
C -4.464430 0.680022 1.563413
H -4.038115 1.488624 2.156136
H -5.347048 1.045974 1.031873
H -4.786402 -0.126094 2.213071
O -2.897478 -1.568140 -0.751278
C 1.300185 -0.429399 0.232617
C 2.355707 -0.852691 -0.522867
C 1.483585 0.464639 1.310580
C 3.668618 -0.404549 -0.230129
H 2.213304 -1.530158 -1.357742
C 2.738742 0.917068 1.603388
H 0.630496 0.791407 1.891670
C 3.861420 0.495456 0.847959
C 4.786126 -0.824140 -0.988231
H 2.894500 1.605146 2.425149
C 5.169641 0.947404 1.137031
C 6.040777 -0.370488 -0.685633
H 4.629597 -1.511255 -1.810556
C 6.234359 0.524759 0.388797
H 5.311684 1.634145 1.962244
H 6.891017 -0.696670 -1.268825
H 7.231368 0.875516 0.617768

ISOMe_naph.log
Energy (E) = -1250.66491317 Hartree
Enthalpy (H) = -1250.414220 Hartree
Gibbs free energy (G) = -1250.480980 Hartree
Charge = 0, Spin = 1
C 1.057240 3.173837 -0.443648
C 2.429318 3.344868 -0.311302
C 3.261321 2.247200 -0.136018
C 2.718367 0.971591 -0.086938
C 1.357259 0.836274 -0.229909
C 0.498015 1.899561 -0.406269
H 0.411613 4.028757 -0.588269
H 2.852825 4.338813 -0.351655
H 4.334492 2.346276 -0.043434
H -0.567841 1.762090 -0.523827
I 0.597739 -1.167224 -0.190521
S 3.735920 -0.475627 0.190523
O 3.528440 -0.781229 1.590216
O 5.066013 -0.125232 -0.232425
O 3.041905 -1.443667 -0.703598
C -1.378205 -0.543112 0.234739
C -2.325819 -0.603425 -0.747547
C -1.685983 -0.104111 1.540833
C -3.660274 -0.223024 -0.463498
H -2.077199 -0.934918 -1.748675
C -2.966718 0.276035 1.828347
H -0.913007 -0.068073 2.296825
C -3.983313 0.225441 0.842714
C -4.673151 -0.273468 -1.449579
H -3.223106 0.618346 2.823071
C -5.315085 0.609052 1.124029
C -5.952418 0.104109 -1.148119
H -4.416493 -0.616873 -2.443879
C -6.276165 0.549666 0.152409
H -5.558189 0.949419 2.122695
H -6.723107 0.062603 -1.905357
H -7.292040 0.844533 0.376756

NpthIBA_C_naph.log
Energy (E) = -969.000654112 Hartree
Enthalpy (H) = -968.703008 Hartree
Gibbs free energy (G) = -968.772879 Hartree
Charge = 0, Spin = 1
C 3.664750 0.542499 -0.181934
C 2.815867 -0.531291 -0.137268
C 1.449363 -0.270026 0.004564
C 0.910866 0.973876 0.099170
H 4.722861 0.337745 -0.293920
H -0.152623 1.144385 0.203721
C 3.347559 -1.951060 -0.251777
O 2.421078 -2.837552 -0.211139
O 4.547296 -2.120758 -0.369433
I 0.242520 -2.028526 0.067324
C 1.302067 3.413528 0.143739
C 2.166379 4.471915 0.096997
C 3.555916 4.255072 -0.043146
C 4.050507 2.983975 -0.133611
C 3.182153 1.866059 -0.088296
C 1.787068 2.087364 0.052635
H 0.236199 3.572867 0.250617
H 1.788707 5.483000 0.167113
H 4.226783 5.102548 -0.078714
H 5.113132 2.807600 -0.241562
C -1.556812 -0.908849 0.331034
C -2.334633 -0.616865 -0.754500
C -1.942426 -0.511534 1.630805
C -3.548910 0.094102 -0.593951
H -2.036197 -0.917290 -1.752550
C -3.108491 0.180187 1.808393
H -1.312511 -0.751421 2.477740
C -3.940156 0.499973 0.707554
C -4.377089 0.412907 -1.696039
H -3.414639 0.492298 2.799277
C -5.151787 1.212576 0.867220
C -5.542588 1.103575 -1.511357
H -4.069797 0.099328 -2.685918
C -5.933870 1.507336 -0.215384
H -5.447168 1.519093 1.862942
H -6.171163 1.343907 -2.357789
H -6.858562 2.052388 -0.083655

PyrroleNAcIBA_A_naph.log
Energy (E) = -945.976062346 Hartree
Enthalpy (H) = -945.705459 Hartree
Gibbs free energy (G) = -945.777167 Hartree
Charge = 0, Spin = 1
C -1.953239 2.259379 -0.211975
C -0.810135 1.508845 -0.239526
C -1.254957 0.182295 -0.038280
C -2.605592 0.130861 0.101862
H -2.071967 3.326475 -0.281063
H 0.196449 1.865543 -0.372541
C -3.372986 -1.169868 0.066481
I -0.258533 -1.634149 -0.021933
O -4.562820 -1.192234 -0.172845
O -2.563981 -2.157023 0.236983
N -3.041269 1.434226 0.002146
C -4.352361 1.917146 0.330091
C -4.746352 3.181714 -0.382373
H -4.233753 4.036993 0.059713
H -4.487456 3.137170 -1.438307
H -5.815292 3.317989 -0.255781
O -5.030931 1.350981 1.132440
C 1.622964 -0.661921 -0.307543
C 2.433768 -0.431832 0.767487
C 2.005672 -0.270122 -1.609242
C 3.685973 0.206771 0.592493
H 2.135564 -0.726193 1.767216
C 3.207560 0.353641 -1.799449
H 1.347117 -0.460247 -2.446931
C 4.077277 0.606344 -0.710711
C 4.551670 0.459467 1.682633
H 3.513353 0.661439 -2.791763
C 5.327009 1.246240 -0.884269
C 5.753784 1.080265 1.484633
H 4.243831 0.151710 2.674163
C 6.145393 1.477474 0.186838
H 5.622224 1.548414 -1.881365
H 6.411128 1.270021 2.322009
H 7.099098 1.967028 0.044539

Bn_02.log
Energy (E) = -270.651950925 Hartree
Enthalpy (H) = -270.530424 Hartree
Gibbs free energy (G) = -270.566749 Hartree
Charge = 0, Spin = 2
C -2.387547 0.000012 -0.000218
C -0.988104 0.000012 0.000094
H -2.941609 -0.926652 -0.000224
H -2.941713 0.926605 0.000227
C -0.251319 1.212048 0.000082
C -0.251344 -1.212036 0.000100
C 1.127002 1.206430 -0.000010
H -0.795095 2.148623 0.000236
C 1.126976 -1.206441 0.000010
H -0.795139 -2.148600 0.000263
C 1.828867 -0.000013 -0.000097
H 1.668246 2.143273 0.000004
H 1.668203 -2.143294 0.000041
H 2.909917 -0.000027 -0.000312

FuranIBA_A_Bn.log
Energy (E) = -699.077151528 Hartree
Enthalpy (H) = -698.879545 Hartree
Gibbs free energy (G) = -698.939769 Hartree
Charge = 0, Spin = 1
C -3.326870 2.303251 0.000034
C -1.973141 2.177232 0.000022
C -1.769690 0.770707 0.000011
C -2.972160 0.157609 -0.000006
O -3.934047 1.097085 0.000007
H -3.971076 3.164034 0.000051
H -1.252452 2.975749 0.000030
C -3.180124 -1.329449 -0.000028
I -0.118058 -0.465578 0.000003
O -4.294512 -1.809713 -0.000046
O -2.032932 -1.915113 -0.000032
C 1.268057 1.240974 0.000024
C 2.646610 0.685597 0.000009

H 1.032757 1.809999 0.895072
H 1.032752 1.810030 -0.895002
C 3.291161 0.396463 -1.202348
C 3.291128 0.396334 1.202357
C 4.563166 -0.156378 -1.202582
H 2.791031 0.613282 -2.139048
C 4.563131 -0.156507 1.202568
H 2.790969 0.613054 2.139064
C 5.201729 -0.433185 -0.000014
H 5.056719 -0.370417 -2.140557
H 5.056659 -0.370648 2.140532
H 6.193654 -0.863395 -0.000021

IBA_oMe_Bn.log
Energy (E) = -740.544081928 Hartree
Enthalpy (H) = -740.286554 Hartree
Gibbs free energy (G) = -740.351182 Hartree
Charge = 0, Spin = 1
C -3.430162 2.116492 0.359272
C -4.372440 1.148118 0.673046
C -4.036715 -0.191537 0.583187
C -2.748074 -0.560814 0.219696
C -1.820277 0.433885 -0.014343
C -2.121258 1.790916 -0.002126
H -3.706976 3.163580 0.369863
H -5.374781 1.443981 0.950784
H -4.742502 -0.991109 0.763336
C -2.440777 -2.033677 -0.028063
O -1.310314 -2.215881 -0.599460
O -3.274843 -2.864320 0.284331
I 0.141170 -0.375009 -0.402459
C -1.195137 2.895992 -0.421713
H -0.484509 3.169645 0.357304
H -0.633131 2.621110 -1.314694
H -1.777197 3.784572 -0.657481
C 1.449514 1.331828 0.155316
C 2.791202 0.704852 0.255764
H 1.074391 1.702954 1.103934
H 1.392402 2.086697 -0.619076
C 3.622580 0.643267 -0.862659
C 3.214698 0.116548 1.448110
C 4.862691 0.025992 -0.785190
H 3.293704 1.091026 -1.793346
C 4.454231 -0.498434 1.526747
H 2.564437 0.146879 2.314387
C 5.281292 -0.543888 0.410213
H 5.501930 -0.009802 -1.656608
H 4.775566 -0.944866 2.457687
H 6.247720 -1.024834 0.471753

IBamideAc_Bn.log
Energy (E) = -833.923210346 Hartree
Enthalpy (H) = -833.642068 Hartree
Gibbs free energy (G) = -833.713427 Hartree
Charge = 0, Spin = 1
C 2.503018 -3.388358 0.000003
C 3.757505 -2.790037 0.000119
C 3.866211 -1.409600 0.000157
C 2.730240 -0.604874 0.000074
C 1.500976 -1.230972 -0.000033
C 1.354191 -2.606587 -0.000070
H 2.409514 -4.465581 -0.000030
H 4.647704 -3.403616 0.000175
H 4.820796 -0.900289 0.000248
H 0.384907 -3.080504 -0.000166
C 2.907485 0.898766 0.000089
O 4.033831 1.354366 0.000402
I -0.196384 0.039252 -0.000125
N 1.704865 1.529208 -0.000112
C 0.149498 3.392914 -0.000151
H 0.141018 4.478458 -0.000119
H -0.378197 3.027781 -0.883671
H -0.378182 3.027726 0.883355
C 1.588852 2.911873 -0.000194
O 2.506483 3.700246 -0.000182
C -1.673669 -1.624839 -0.000026
C -2.989790 -0.937249 0.000054
H -1.496462 -2.210050 0.897759
H -1.496618 -2.210118 -0.897796
C -3.602572 -0.580459 -1.201756
C -3.602100 -0.579916 1.201957
C -4.812079 0.098360 -1.201980
H -3.127418 -0.847002 -2.138699
C -4.811598 0.098900 1.202363
H -3.126559 -0.846043 2.138823
C -5.420123 0.438327 0.000231
H -5.281134 0.361330 -2.140192
H -5.280285 0.362295 2.140639
H -6.363569 0.966429 0.000301

IBCCF32O_Bn.log
Energy (E) = -1301.07466462 Hartree
Enthalpy (H) = -1300.812069 Hartree
Gibbs free energy (G) = -1300.888497 Hartree
Charge = 0, Spin = 1
C 1.221994 3.640971 -0.304447
C 2.537958 3.206164 -0.362052
C 2.835236 1.857167 -0.239712
C 1.821025 0.920986 -0.048506
C 0.522367 1.383216 -0.005066
C 0.196762 2.720812 -0.130397
H 0.986096 4.692102 -0.394977
H 3.338364 3.918884 -0.503416
H 3.860423 1.524477 -0.295113
H -0.826098 3.061735 -0.089331
O 0.945810 -1.222594 0.530354
I -0.992612 -0.088903 0.228654
C 2.053605 -0.588967 0.106972
C 3.166061 -0.849483 1.155157
C 2.464505 -1.152971 -1.278059
F 4.387825 -0.469023 0.744160
F 2.893598 -0.170076 2.271416
F 3.239841 -2.134812 1.465070
F 3.520190 -0.533362 -1.822547
F 2.742896 -2.448783 -1.219552
F 1.436410 -0.988842 -2.121026
C -2.654000 1.334592 -0.098177
C -3.867684 0.477258 -0.070236
H -2.488678 1.809101 -1.061438
H -2.635820 2.061316 0.708789
C -4.540242 0.241226 1.128758
C -4.312694 -0.157016 -1.230708
C -5.649180 -0.591609 1.162723
H -4.191597 0.721809 2.035426
C -5.421553 -0.988990 -1.197660
H -3.784461 0.011264 -2.161967
C -6.093547 -1.206692 -0.000885
H -6.166811 -0.760395 2.097087
H -5.761597 -1.468442 -2.105377
H -6.958395 -1.855157 0.024903

IBCH2S_Bn.log
Energy (E) = -1028.73511594 Hartree
Enthalpy (H) = -1028.432893 Hartree
Gibbs free energy (G) = -1028.499510 Hartree
Charge = 0, Spin = 1
C -2.430779 3.085253 -0.008011
C -3.633907 2.485043 0.323044
C -3.718759 1.103606 0.434355
C -2.617485 0.275505 0.213731
C -1.430543 0.919720 -0.108003
C -1.310532 2.290606 -0.221125
H -2.356123 4.158427 -0.114070
H -4.515135 3.089420 0.489698
H -4.666878 0.655492 0.694411
H -0.372690 2.748160 -0.495869
I 0.294867 -0.289773 -0.474642
C -2.711974 -1.236479 0.370725
S -1.726047 -2.016276 -0.937095
C -4.143814 -1.741614 0.215796
H -4.127706 -2.830912 0.214006
H -4.777163 -1.418692 1.045962
H -4.578044 -1.403288 -0.724609
C -2.209653 -1.622187 1.765243
H -2.830389 -1.155426 2.535157
H -2.258137 -2.705301 1.876050
H -1.176800 -1.311456 1.922874
C 1.707055 1.435773 0.094693
C 3.015175 0.768554 0.228500
H 1.314329 1.849477 1.019332
H 1.674933 2.154706 -0.718196
C 3.840682 0.598349 -0.885686
C 3.419203 0.217729 1.447690
C 5.046980 -0.078785 -0.779077
H 3.532124 1.012587 -1.838805
C 4.625569 -0.455932 1.555731
H 2.778463 0.329707 2.314617
C 5.445491 -0.605497 0.442779
H 5.677412 -0.194017 -1.650492
H 4.927870 -0.866879 2.509617
H 6.386457 -1.131490 0.527554

IBNMeCO2_Bn.log
Energy (E) = -795.862708358 Hartree
Enthalpy (H) = -795.586822 Hartree
Gibbs free energy (G) = -795.653900 Hartree
Charge = 0, Spin = 1
C -2.491926 3.089441 -0.035141
C -3.587127 2.476545 0.558716
C -3.617633 1.107004 0.756128
C -2.548333 0.278515 0.378749
C -1.448855 0.941025 -0.178246
C -1.410736 2.303748 -0.405271
H -2.473297 4.155847 -0.206518
H -4.440760 3.069466 0.857972
H -4.494313 0.652188 1.193418
H -0.543771 2.756490 -0.866419
I 0.259777 -0.195141 -0.607814
C -2.240763 -2.026334 -0.463009
O -2.677648 -3.156048 -0.362599
N -2.587528 -1.091010 0.589902
C -3.515799 -1.601385 1.584510
H -3.472248 -0.977560 2.476634
H -4.544871 -1.637043 1.216673
H -3.220933 -2.616217 1.826662
O -1.510611 -1.535270 -1.390677
C 1.616514 1.305252 0.271648
C 2.946813 0.654069 0.390442
H 1.167039 1.568827 1.224900
H 1.625936 2.159223 -0.397937
C 3.881811 0.764442 -0.637926
C 3.258670 -0.119922 1.508542
C 5.111192 0.127724 -0.543894
H 3.642572 1.359709 -1.511464
C 4.487389 -0.754394 1.604358

H 2.530197 -0.218554 2.304826
C 5.416911 -0.631501 0.578072
H 5.830511 0.225355 -1.345415
H 4.720734 -1.346017 2.478937
H 6.375027 -1.127011 0.652671

ISOMe_Bn.log
Energy (E) = -1136.44042785 Hartree
Enthalpy (H) = -1136.209589 Hartree
Gibbs free energy (G) = -1136.274449 Hartree
Charge = 0, Spin = 1
C 2.507955 3.040994 0.234593
C 3.714060 2.377938 0.419177
C 3.768247 0.991674 0.363796
C 2.610669 0.263710 0.132246
C 1.430321 0.946634 -0.053826
C 1.340828 2.323753 -0.011065
H 2.467240 4.120766 0.266520
H 4.616943 2.944931 0.598231
H 4.694489 0.446426 0.486024
H 0.411099 2.847829 -0.177497
I -0.302793 -0.235647 -0.437721
S 2.620393 -1.527351 0.097882
O 2.112889 -1.903268 1.399764
O 3.964236 -1.917443 -0.238980
O 1.625180 -1.760083 -0.985173
C -1.727429 1.318604 0.145388
C -3.061483 0.663071 0.214854
H -1.677871 2.092173 -0.614085
H -1.368520 1.683121 1.103674
C -3.447828 -0.024278 1.365160
C -3.919875 0.697443 -0.882188
C -4.681928 -0.652354 1.422340
H -2.774591 -0.063964 2.213379
C -5.154929 0.067586 -0.824958
H -3.618532 1.226029 -1.778866
C -5.537399 -0.606650 0.327350
H -4.976524 -1.179030 2.319345
H -5.817978 0.103755 -1.678203
H -6.499686 -1.097495 0.372505

NpthIBA_C_Bn.log
Energy (E) = -854.776669581 Hartree
Enthalpy (H) = -854.499002 Hartree
Gibbs free energy (G) = -854.566664 Hartree
Charge = 0, Spin = 1
C 3.317980 1.039155 0.000547
C 1.976503 1.312356 0.000078
C 1.088097 0.231313 -0.000502
C 1.480526 -1.072037 -0.000601
H 3.999143 1.881830 0.000979
H 0.783167 -1.897948 -0.001030
C 1.474594 2.746679 0.000110
O 0.194522 2.830695 0.000119
O 2.286900 3.653549 0.001489
I -0.959507 0.795722 -0.001076
C 3.347897 -2.692190 -0.000227
C 4.691565 -2.944806 0.000232
C 5.619745 -1.879101 0.000809
C 5.183048 -0.583866 0.000912
C 3.798002 -0.288526 0.000445
C 2.868333 -1.360937 -0.000130
H 2.632645 -3.505599 -0.000673
H 5.050658 -3.965007 0.000154
H 6.679353 -2.095082 0.001170
H 5.885821 0.239527 0.001353
C -1.707720 -1.275857 -0.001081
C -3.189844 -1.155195 -0.000050
H -1.319659 -1.751593 0.895070
H -1.320921 -1.751266 -0.897948
C -3.892787 -1.069572 -1.201542
C -3.890936 -1.068504 1.202479
C -5.271778 -0.921000 -1.200855
H -3.351099 -1.126105 -2.138456
C -5.269909 -0.919922 1.203815
H -3.347764 -1.124212 2.138588
C -5.963259 -0.847264 0.001976
H -5.807140 -0.863021 -2.138536
H -5.803820 -0.861105 2.142271
H -7.038267 -0.732021 0.002759

PyrroleNAcIBA_A_Bn.log
Energy (E) = -831.753205030 Hartree
Enthalpy (H) = -831.502570 Hartree
Gibbs free energy (G) = -831.572298 Hartree
Charge = 0, Spin = 1
C -2.735761 2.015443 -0.078432
C -1.370923 1.921695 -0.052890
C -1.108065 0.533196 0.025190
C -2.266622 -0.177744 0.044282
H -3.360777 2.891093 -0.091066
H -0.676367 2.743078 -0.078266
C -2.300672 -1.675169 -0.146232
I 0.644888 -0.567773 0.017571
O -3.319464 -2.240515 -0.488466
O -1.123425 -2.165395 0.025317
N -3.282403 0.749721 -0.011480
C -4.676558 0.494304 0.227619
C -5.610745 1.442453 -0.470968
H -5.598922 2.415234 0.022204
H -5.320114 1.584213 -1.510002
H -6.614057 1.034678 -0.406219
O -5.019225 -0.388574 0.953488
C 1.880993 1.249797 -0.024218
C 3.302046 0.814532 -0.009922
H 1.597659 1.820248 0.855941
H 1.601511 1.775260 -0.933135
C 3.965251 0.529739 -1.203229
C 3.973703 0.633491 1.198581
C 5.280327 0.089059 -1.189267
H 3.444430 0.661250 -2.144551
C 5.288910 0.193205 1.213222
H 3.459616 0.845645 2.128710
C 5.945112 -0.078983 0.019164
H 5.786887 -0.122445 -2.120918
H 5.802243 0.062927 2.156000
H 6.970578 -0.421515 0.030309

00CCPh_02.log
Energy (E) = -307.386137042 Hartree
Enthalpy (H) = -307.280387 Hartree
Gibbs free energy (G) = -307.319052 Hartree
Charge = 0, Spin = 2
C -3.302510 0.000015 0.000078
C -2.098379 -0.000024 -0.000057
C -0.665139 -0.000015 -0.000033
C 0.039004 -1.206478 -0.000018
C 0.038981 1.206467 -0.000018
C 1.424823 -1.202887 0.000010
H -0.510412 -2.137686 -0.000029
C 1.424797 1.202905 0.000010
H -0.510459 2.137660 -0.000028
C 2.120514 0.000014 0.000023
H 1.963188 -2.140808 0.000020
H 1.963147 2.140834 0.000020
H 3.201993 0.000028 0.000043

FuranIBA_A_CCPh.log
Energy (E) = -735.876708235 Hartree
Enthalpy (H) = -735.696170 Hartree
Gibbs free energy (G) = -735.758309 Hartree
Charge = 0, Spin = 1
C 2.012833 2.969993 -0.000198
C 0.982159 2.082828 -0.000122
C 1.637606 0.827057 -0.000054
C 2.972170 1.021066 -0.000064
O 3.211916 2.342846 -0.000141
H 2.038085 4.044782 -0.000270
H -0.072112 2.294096 -0.000129
C 3.979814 -0.083527 0.000006
I 1.055052 -1.149209 0.000061
O 5.171860 0.120329 0.000005
O 3.351365 -1.220820 0.000060
C -0.942473 -0.607420 0.000054
C -2.125985 -0.366092 0.000065
C -3.524461 -0.068755 0.000025
C -4.213322 0.077085 -1.207471
C -4.213238 0.077751 1.207486
C -5.568460 0.364825 -1.204026
H -3.676103 -0.038150 -2.138682
C -5.568378 0.365493 1.203979
H -3.675956 -0.036972 2.138726
C -6.247491 0.509414 -0.000039
H -6.096104 0.476131 -2.141186
H -6.095953 0.477317 2.141116
H -7.305374 0.733743 -0.000068

IBA_oMe_CCPh.log
Energy (E) = -777.345343148 Hartree
Enthalpy (H) = -777.105002 Hartree
Gibbs free energy (G) = -777.172372 Hartree
Charge = 0, Spin = 1
C 2.069859 3.009034 -0.051615
C 3.369284 2.870021 0.411345
C 3.910666 1.606647 0.572349
C 3.131728 0.487605 0.311428
C 1.821737 0.674316 -0.081696
C 1.244233 1.914184 -0.325639
H 1.665932 3.997187 -0.234166
H 3.965478 3.750063 0.609305
H 4.934306 1.435218 0.876042
C 3.777122 -0.887349 0.343627
O 3.007393 -1.809559 -0.122973
O 4.917283 -1.004129 0.742916
I 0.823013 -1.232657 -0.268632
C -0.112203 2.178149 -0.912973
H -0.358169 1.468097 -1.701033
H -0.903167 2.118792 -0.167618
H -0.120837 3.178985 -1.340983
C -1.125243 -0.528256 -0.144005
C -2.315205 -0.354745 -0.022742
C -3.715692 -0.107485 0.116685
C -4.499631 0.165273 -1.008439
C -4.313972 -0.132828 1.380160
C -5.856516 0.408188 -0.869391
H -4.033643 0.181420 -1.984099
C -5.671107 0.111328 1.512609
H -3.704345 -0.345356 2.247487
C -6.444205 0.382026 0.389710
H -6.456945 0.617674 -1.743940
H -6.127249 0.089973 2.492740
H -7.503588 0.571632 0.495747

IBamideAc_CCPh.log

Energy (E) = -870.726351406 Hartree
Enthalpy (H) = -870.462261 Hartree
Gibbs free energy (G) = -870.535464 Hartree
Charge = 0, Spin = 1
C -0.419998 3.550316 -0.000115
C -1.766380 3.899169 0.000000
C -2.741115 2.915373 0.000083
C -2.385595 1.569652 0.000047
C -1.041066 1.268395 -0.000068
C -0.037925 2.213709 -0.000148
H 0.342858 4.316476 -0.000178
H -2.051675 4.942012 0.000027
H -3.798908 3.142089 0.000178
H 1.004586 1.928996 -0.000232
C -3.469366 0.519737 0.000135
O -4.631923 0.863698 0.000313
I -0.601221 -0.807957 -0.000097
N -2.932200 -0.736088 0.000030
C -2.919017 -3.169545 0.000180
H -3.606317 -4.009584 0.000291
H -2.282137 -3.228572 -0.885075
H -2.282153 -3.228303 0.885463
C -3.721389 -1.882034 -0.000040
O -4.929073 -1.901714 -0.000100
C 1.465021 -0.426550 -0.000063
C 2.673518 -0.369134 -0.000031
C 4.100830 -0.284757 0.000012
C 4.804859 -0.242735 1.207067
C 4.804951 -0.242772 -1.206983
C 6.187786 -0.160140 1.203819
H 4.256575 -0.276248 2.138400
C 6.187882 -0.160178 -1.203630
H 4.256747 -0.276313 -2.138362
C 6.881303 -0.118647 0.000120
H 6.725873 -0.128405 2.141165
H 6.726037 -0.128472 -2.140937
H 7.960801 -0.054375 0.000163

IBCCF32O_CCPh.log
Energy (E) = -1337.87847977 Hartree
Enthalpy (H) = -1337.632946 Hartree
Gibbs free energy (G) = -1337.712544 Hartree
Charge = 0, Spin = 1
C 0.027422 3.212265 -0.003496
C -1.340798 3.422043 0.097424
C -2.219863 2.349246 0.094538
C -1.741941 1.045202 -0.020542
C -0.377006 0.879745 -0.112176
C 0.526073 1.919866 -0.103598
H 0.712252 4.048629 -0.002404
H -1.728675 4.427565 0.181800
H -3.281469 2.520210 0.186049
H 1.588666 1.733611 -0.172489
O -1.920596 -1.307403 -0.382658
I 0.272521 -1.141870 -0.238711
C -2.623809 -0.207581 -0.034240
C -3.762966 -0.048048 -1.071485
C -3.194929 -0.412815 1.391585
F -4.665223 0.883706 -0.726685
F -3.243473 0.313180 -2.245926
F -4.416569 -1.186719 -1.244164
F -3.863960 0.647836 1.858246
F -4.003989 -1.461872 1.451564
F -2.170012 -0.632672 2.224663
C 2.272101 -0.559296 -0.117958
C 3.465873 -0.374220 -0.060876
C 4.874940 -0.142812 0.009412
C 5.507140 -0.009016 1.249139
C 5.633286 -0.048142 -1.161222
C 6.872627 0.216135 1.313515
H 4.917098 -0.084683 2.152059
C 6.998551 0.176092 -1.090290
H 5.140559 -0.153452 -2.117933
C 7.620240 0.308961 0.145604
H 7.355032 0.318373 2.275843
H 7.578974 0.247234 -1.999846
H 8.686126 0.483961 0.198572

IBCH2S_CCPh.log
Energy (E) = -1065.53950556 Hartree
Enthalpy (H) = -1065.254105 Hartree
Gibbs free energy (G) = -1065.324105 Hartree
Charge = 0, Spin = 1
C -0.942754 3.131403 -0.744788
C -2.261043 3.379035 -0.397855
C -3.088142 2.338972 0.004486
C -2.634100 1.020893 0.061761
C -1.308722 0.827584 -0.295554
C -0.452318 1.832131 -0.686567
H -0.294604 3.933766 -1.067914
H -2.653431 4.385924 -0.440740
H -4.110456 2.554314 0.280540
H 0.573646 1.611220 -0.946559
I -0.593244 -1.191087 -0.261287
C -3.522904 -0.109998 0.559600
S -3.168936 -1.594513 -0.423962
C -5.006197 0.198005 0.379833
H -5.581553 -0.691837 0.633557
H -5.329189 1.002636 1.044024
H -5.227396 0.469896 -0.651746
C -3.242521 -0.342424 2.046301
H -3.472471 0.559955 2.618710
H -3.863757 -1.161918 2.407191
H -2.198604 -0.601727 2.223900
C 1.434692 -0.417339 -0.114840
C 2.632849 -0.260898 -0.013641
C 4.041525 -0.046617 0.109883
C 4.586926 0.396645 1.318724
C 4.892607 -0.278983 -0.974909
C 5.951993 0.603090 1.436972
H 3.927883 0.574889 2.157326
C 6.257257 -0.072816 -0.850955
H 4.470252 -0.622256 -1.909384
C 6.790683 0.368906 0.353866
H 6.363108 0.946450 2.376596
H 6.906432 -0.256710 -1.696269
H 7.856018 0.529655 0.448475

IBNMeCO2_CCPh.log
Energy (E) = -832.663862391 Hartree
Enthalpy (H) = -832.404883 Hartree
Gibbs free energy (G) = -832.473783 Hartree
Charge = 0, Spin = 1
C -0.819268 3.018768 -1.190457
C -1.996803 3.408706 -0.566377
C -2.798191 2.487315 0.085005
C -2.458803 1.127082 0.148852
C -1.256252 0.788264 -0.473927
C -0.445820 1.684561 -1.138550
H -0.195480 3.736298 -1.703094
H -2.304553 4.445133 -0.593891
H -3.718230 2.813204 0.547412
H 0.479352 1.350774 -1.588154
I -0.642950 -1.220043 -0.336712
C -3.645792 -1.034851 0.248936
O -4.649500 -1.573817 0.663218
N -3.259019 0.217758 0.833724
C -4.172907 0.735182 1.839318
H -3.661294 1.485340 2.440278
H -5.072842 1.171959 1.399041
H -4.484052 -0.092592 2.466718
O -2.861503 -1.454399 -0.684959
C 1.334807 -0.639556 -0.090628
C 2.515988 -0.422144 0.043400
C 3.908406 -0.146290 0.211978
C 4.378830 0.394780 1.412026
C 4.811821 -0.415024 -0.820309
C 5.728535 0.662603 1.572828
H 3.676640 0.599944 2.208155
C 6.160725 -0.147364 -0.652430
H 4.444365 -0.834465 -1.746706
C 6.621347 0.392077 0.542652
H 6.085277 1.081791 2.503561
H 6.853947 -0.359204 -1.454812
H 7.674626 0.600866 0.671208

ISOMe_CCPh.log
Energy (E) = -1173.23908796 Hartree
Enthalpy (H) = -1173.025511 Hartree
Gibbs free energy (G) = -1173.092373 Hartree
Charge = 0, Spin = 1
C 0.919192 3.173453 -0.351024
C 2.278312 3.360870 -0.131453
C 3.117333 2.271977 0.061703
C 2.591741 0.988437 0.035048
C 1.245879 0.840337 -0.199779
C 0.378191 1.891449 -0.390060
H 0.269374 4.023611 -0.504030
H 2.685970 4.361932 -0.113720
H 4.180055 2.386494 0.227582
H -0.676517 1.734201 -0.566639
I 0.543981 -1.185053 -0.285251
S 3.601278 -0.449726 0.356249
O 3.376178 -0.746301 1.751285
O 4.936016 -0.132281 -0.069947
O 2.903894 -1.431578 -0.539629
C -1.410371 -0.621869 -0.126625
C -2.593382 -0.396453 -0.036208
C -3.989396 -0.116349 0.078469
C -4.789355 -0.043453 -1.065723
C -4.562790 0.086479 1.337307
C -6.142492 0.228278 -0.949075
H -4.339915 -0.202932 -2.036080
C -5.916412 0.359078 1.446026
H -3.938422 0.027049 2.217911
C -6.706951 0.430137 0.304974
H -6.757964 0.282692 -1.836253
H -6.356041 0.515228 2.421292
H -7.763657 0.642220 0.393126

NpthIBA_C_CCPh.log
Energy (E) = -891.579345084 Hartree
Enthalpy (H) = -891.318803 Hartree
Gibbs free energy (G) = -891.388662 Hartree
Charge = 0, Spin = 1
C 3.559137 0.616345 0.000025
C 2.737316 -0.478661 -0.000090
C 1.357880 -0.252445 -0.000062
C 0.773961 0.971383 0.000070
H 4.628740 0.443843 0.000004
H -0.301002 1.098224 0.000086
C 3.295474 -1.885406 -0.000239
O 2.372619 -2.790444 -0.000327
O 4.496521 -2.059525 -0.000261

I 0.235317 -2.059247 -0.000251
C 1.093872 3.420967 0.000331
C 1.929395 4.503253 0.000445
C 3.331306 4.325255 0.000422
C 3.867598 3.067986 0.000286
C 3.030304 1.925839 0.000166
C 1.621606 2.108204 0.000189
H 0.018884 3.550483 0.000347
H 1.518903 5.503859 0.000553
H 3.978640 5.191501 0.000513
H 4.940191 2.921687 0.000267
C -1.562194 -1.020201 -0.000111
C -2.663284 -0.521335 -0.000038
C -3.959745 0.081088 0.000077
C -4.598684 0.378883 -1.207176
C -4.598566 0.378685 1.207440
C -5.854162 0.964514 -1.203654
H -4.101258 0.145533 -2.138418
C -5.854044 0.964318 1.204137
H -4.101050 0.145183 2.138597
C -6.483370 1.258197 0.000297
H -6.343182 1.191820 -2.140851
H -6.342972 1.191470 2.141420
H -7.463590 1.715000 0.000381

PyrroleNAcIBA_A_CCPh.log
Energy (E) = -868.553540126 Hartree
Enthalpy (H) = -868.319939 Hartree
Gibbs free energy (G) = -868.391021 Hartree
Charge = 0, Spin = 1
C -1.795371 2.283520 -0.082008
C -0.661355 1.520080 -0.047755
C -1.137518 0.194129 0.023845
C -2.495554 0.151885 0.031814
H -1.896181 3.354445 -0.097378
H 0.358883 1.860221 -0.064021
C -3.243467 -1.141140 -0.150957
I -0.203164 -1.649535 0.024246
O -4.410235 -1.185705 -0.464861
O -2.425363 -2.138975 0.003931
N -2.909410 1.465960 -0.026983
C -4.241087 1.949341 0.196725
C -4.525978 3.298248 -0.404513
H -4.067984 4.082002 0.200094
H -4.134246 3.373946 -1.416866
H -5.601085 3.444206 -0.399167
O -5.022015 1.315425 0.839682
C 1.663489 -0.743471 0.015438
C 2.782152 -0.287295 0.012208
C 4.102093 0.262889 0.007210
C 4.745243 0.562927 1.211501
C 4.759821 0.505718 -1.202102
C 6.023625 1.096846 1.203178
H 4.233018 0.372303 2.144410
C 6.038216 1.039633 -1.203714
H 4.258800 0.270940 -2.131038
C 6.671706 1.336069 -0.002745
H 6.515742 1.326303 2.138230
H 6.541707 1.224513 -2.142581
H 7.669737 1.752506 -0.006603

00OEt_02.log
Energy (E) = -154.239165293 Hartree
Enthalpy (H) = -154.169145 Hartree
Gibbs free energy (G) = -154.202289 Hartree
Charge = 0, Spin = 2
O -1.246437 -0.365067 0.000000
C -0.178064 0.481334 0.000000
H -0.298212 1.157781 0.863562
H -0.298213 1.157779 -0.863563
C 1.179804 -0.195790 0.000000
H 1.286934 -0.824553 -0.882710
H 1.983618 0.540812 -0.000001
H 1.286935 -0.824551 0.882710

FuranIBA_A_OEt.log
Energy (E) = -582.677509025 Hartree
Enthalpy (H) = -582.530512 Hartree
Gibbs free energy (G) = -582.583168 Hartree
Charge = 0, Spin = 1
C 1.235055 2.839149 0.201449
C 0.070260 2.146360 0.325575
C 0.471585 0.800298 0.147198
C 1.801885 0.764514 -0.058887
O 2.285549 2.017280 -0.030388
H 1.458508 3.889270 0.256679
H -0.913302 2.532178 0.526387
C 2.550093 -0.503323 -0.253169
I -0.421597 -1.041899 0.156659
O 3.738225 -0.571381 -0.444229
O 1.706601 -1.510726 -0.178890
O -2.211335 -0.123946 0.456943
C -2.809518 0.369685 -0.729122
H -3.103849 -0.456346 -1.386012
H -2.099488 0.994293 -1.288129
C -4.017880 1.184580 -0.327854
H -3.716245 2.018183 0.306119
H -4.524527 1.579451 -1.208311
H -4.717664 0.566228 0.231712

IBA_oMe_OEt.log
Energy (E) = -624.151430167 Hartree
Enthalpy (H) = -623.944429 Hartree
Gibbs free energy (G) = -624.001348 Hartree
Charge = 0, Spin = 1
C 1.216957 2.888116 0.143704
C 2.497579 2.585863 -0.295468
C 2.873419 1.264389 -0.463572
C 1.941939 0.263298 -0.232954
C 0.654910 0.606642 0.134110
C 0.246582 1.910686 0.388572
H 0.945651 3.919283 0.333489
H 3.207245 3.382981 -0.468166
H 3.872904 0.965234 -0.747992
C 2.365796 -1.181333 -0.280789
O 1.430776 -1.996917 0.125122
O 3.480364 -1.498530 -0.625631
I -0.557351 -1.143605 0.288057
C -1.071606 2.345490 0.964385
H -1.852995 2.403898 0.207682
H -1.426558 1.661063 1.730642
H -0.952223 3.335071 1.402001
O -2.321399 -0.123259 0.254230
C -2.743372 0.224980 -1.049782
H -2.781370 -0.659617 -1.696427
H -2.031049 0.926052 -1.508153
C -4.113609 0.856338 -0.943589
H -4.074453 1.745462 -0.314505
H -4.483468 1.140909 -1.928552
H -4.813590 0.153545 -0.494496

IBamideAc_OEt.log
Energy (E) = -717.533193257 Hartree
Enthalpy (H) = -717.302468 Hartree
Gibbs free energy (G) = -717.365875 Hartree
Charge = 0, Spin = 1
C 1.837652 3.047707 -0.266213
C 0.697457 3.810320 -0.028901
C -0.533330 3.196077 0.134387
C -0.630587 1.810236 0.068923
C 0.521221 1.088420 -0.151201
C 1.763105 1.660992 -0.332165
H 2.794453 3.531471 -0.405873
H 0.773454 4.887614 0.020028
H -1.444257 3.752882 0.308753
H 2.630217 1.049251 -0.535828
C -1.963469 1.137379 0.212485
O -2.961082 1.784108 0.433255
I 0.237948 -0.992418 -0.216690
N -1.849027 -0.227724 0.055078
C -2.596075 -2.552861 -0.043015
H -3.511528 -3.133036 0.010757
H -2.106903 -2.748265 -0.999488
H -1.925354 -2.868777 0.758708
C -2.950200 -1.086234 0.093496
O -4.093855 -0.725883 0.217439
O 2.276864 -1.228754 -0.421949
C 2.973171 -1.262792 0.807736
H 2.696247 -2.151419 1.387246
H 2.721205 -0.386160 1.421921
C 4.455916 -1.274051 0.507875
H 4.736165 -0.374480 -0.040898
H 5.037414 -1.315751 1.428958
H 4.706268 -2.138023 -0.105653

IBCCF32O_OEt.log
Energy (E) = -1184.68331042 Hartree
Enthalpy (H) = -1184.471085 Hartree
Gibbs free energy (G) = -1184.540039 Hartree
Charge = 0, Spin = 1
C 1.042166 3.253784 0.181488
C -0.313264 3.431876 -0.062451
C -1.168678 2.341698 -0.133967
C -0.671107 1.053799 0.044250
C 0.681869 0.914303 0.267067
C 1.559636 1.975084 0.345263
H 1.701600 4.107850 0.246301
H -0.710483 4.428363 -0.195534
H -2.220146 2.488614 -0.328311
H 2.607694 1.803428 0.545451
O -0.797089 -1.295451 0.464519
I 1.334275 -1.075534 0.476195
C -1.503613 -0.225288 0.002275
C -1.915843 -0.500363 -1.464181
C -2.745764 -0.095695 0.916112
F -2.563316 0.523398 -2.027877
F -0.803903 -0.712301 -2.178640
F -2.681365 -1.577679 -1.568766
F -3.639281 0.788535 0.450871
F -3.366564 -1.257221 1.051185
F -2.363614 0.313779 2.125139
O 3.266220 -0.412692 0.456829
C 3.791487 -0.222090 -0.844216
H 3.125391 0.417421 -1.439763
H 3.874974 -1.179820 -1.369777
C 5.150272 0.426971 -0.706778
H 5.807258 -0.202375 -0.108641
H 5.605208 0.580684 -1.685264
H 5.058278 1.393213 -0.209956

IBCH2S_OEt.log
Energy (E) = -912.349534708 Hartree
Enthalpy (H) = -912.097710 Hartree
Gibbs free energy (G) = -912.157947

Hartree
Charge = 0, Spin = 1
C 0.011177 3.227981 -0.085383
C -1.370593 3.312606 -0.152006
C -2.145228 2.161350 -0.115167
C -1.564658 0.895901 -0.038717
C -0.178893 0.860705 -0.007800
C 0.622290 1.982451 -0.001894
H 0.619876 4.121112 -0.099955
H -1.852637 4.278185 -0.221258
H -3.222551 2.246342 -0.139184
H 1.693226 1.881011 0.087700
I 0.727189 -1.069018 0.047704
C -2.409167 -0.356547 0.127775
S -1.598578 -1.722162 -0.751729
C -3.799902 -0.215164 -0.481812
H -4.304557 -1.180490 -0.444948
H -4.405602 0.495200 0.084053
H -3.741876 0.108057 -1.520500
C -2.538253 -0.663790 1.620814
H -3.029923 0.167026 2.132653
H -3.130032 -1.568675 1.757978
H -1.561644 -0.817832 2.080832
O 2.536964 -0.097583 0.612122
C 3.431080 0.049962 -0.460306
H 2.960390 0.580504 -1.304093
H 3.755723 -0.924603 -0.850196
C 4.635414 0.831692 0.024467
H 5.108012 0.306982 0.853623
H 5.366787 0.962359 -0.774201
H 4.325696 1.815125 0.380014

IBNMeCO2_OEt.log
Energy (E) = -679.463874245 Hartree
Enthalpy (H) = -679.238393 Hartree
Gibbs free energy (G) = -679.298455
Hartree
Charge = 0, Spin = 1
C 0.107426 3.225518 -0.467209
C -1.256652 3.369389 -0.254026
C -2.054994 2.269712 0.009662
C -1.522438 0.975020 0.070926
C -0.149307 0.874392 -0.153801
C 0.665051 1.957561 -0.412167
H 0.732700 4.083781 -0.665277
H -1.709926 4.350339 -0.294985
H -3.114797 2.405062 0.166994
H 1.728495 1.815944 -0.538408
I 0.696842 -1.031472 -0.023578
C -2.295099 -1.340089 -0.302757
O -3.254644 -2.075351 -0.276345
N -2.333784 -0.112663 0.405050
C -3.578492 0.152106 1.110443
H -3.400346 0.895100 1.885511
H -4.367587 0.503447 0.441585
H -3.914924 -0.776158 1.558575
O -1.188071 -1.564439 -0.956415
O 2.397801 -0.185885 0.731239
C 3.495697 -0.200751 -0.160274
H 3.218533 0.225756 -1.134470
H 3.834444 -1.226395 -0.348534
C 4.607349 0.608743 0.469425
H 4.883683 0.177686 1.430216
H 5.486508 0.625072 -0.174799
H 4.275137 1.632818 0.638741

ISOMe_OEt.log
Energy (E) = -1020.03601166 Hartree
Enthalpy (H) = -1019.855847 Hartree
Gibbs free energy (G) = -1019.913128
Hartree
Charge = 0, Spin = 1
C 0.059299 3.213500 0.127170
C 1.439654 3.256459 -0.030497
C 2.173815 2.082328 -0.121740
C 1.512144 0.865071 -0.071180
C 0.142456 0.851643 0.049231
C -0.614384 1.996622 0.171688
H -0.505376 4.131150 0.213251
H 1.945820 4.210552 -0.072773
H 3.249866 2.085637 -0.231399
H -1.684011 1.949070 0.315326
I -0.761596 -1.065296 0.051540
S 2.394329 -0.677809 -0.027359
O 2.590417 -0.967671 1.368877
O 3.538837 -0.568397 -0.884553
O 1.319005 -1.571791 -0.628309
O -2.535345 -0.236651 0.530201
C -3.355515 0.060208 -0.594231
H -3.664146 -0.862182 -1.095752
H -2.801044 0.662074 -1.325124
C -4.559149 0.817907 -0.084548
H -4.248112 1.746173 0.394671
H -5.234312 1.058090 -0.905423
H -5.096594 0.218752 0.648247

NpthIBA_C_OEt.log
Energy (E) = -738.382417401 Hartree
Enthalpy (H) = -738.155209 Hartree
Gibbs free energy (G) = -738.215353
Hartree
Charge = 0, Spin = 1
C 1.945621 1.649191 0.211367
C 0.586491 1.523030 0.117898
C 0.053749 0.250309 -0.106140
C 0.794722 -0.876322 -0.256618
H 2.359309 2.635752 0.382420
H 0.335610 -1.834915 -0.460722
C -0.337856 2.704053 0.219393
O -1.595314 2.381572 0.055258
O 0.081311 3.819690 0.423649
I -2.045856 0.269241 -0.208945
C 3.049910 -1.881270 -0.305985
C 4.407122 -1.746117 -0.210369
C 4.985182 -0.479730 0.032352
C 4.192724 0.625015 0.172716
C 2.783667 0.519718 0.079385
C 2.204543 -0.755966 -0.162556
H 2.600962 -2.848697 -0.492861
H 5.046249 -2.611489 -0.321072
H 6.060272 -0.389479 0.105207
H 4.626535 1.599624 0.356643
O -2.034833 -1.759168 -0.409464
C -2.003182 -2.456802 0.823314
H -2.936919 -2.305108 1.375815
H -1.185840 -2.085625 1.456444
C -1.801727 -3.924051 0.518922
H -0.857808 -4.074337 -0.005872
H -1.786599 -4.510079 1.437731
H -2.606516 -4.288518 -0.117295

PyrroleNAcIBA_A_OEt.log
Energy (E) = -715.355439188 Hartree
Enthalpy (H) = -715.155315 Hartree
Gibbs free energy (G) = -715.216521
Hartree
Charge = 0, Spin = 1
C -1.542156 1.991430 -0.383912
C -0.196926 1.749906 -0.434170
C -0.078999 0.370628 -0.166646
C -1.295005 -0.199110 0.031789
H -2.072995 2.920220 -0.495904
H 0.592294 2.450721 -0.642654
C -1.428014 -1.688109 0.076156
I 1.501159 -0.937577 -0.132301
O -2.467043 -2.291618 -0.004031
O -0.231444 -2.235421 0.147009
N -2.218828 0.819632 -0.096429
C -3.608003 0.761611 0.246860
C -4.456284 1.835820 -0.376527
H -4.295438 2.784938 0.136025
H -4.220452 1.971023 -1.430252
H -5.495494 1.549755 -0.252970
O -4.017455 -0.075127 0.994245
O 2.749860 0.649386 -0.412339
C 3.050637 1.357182 0.776260
H 3.659318 0.742624 1.449259
H 2.129077 1.616210 1.315477
C 3.796949 2.613731 0.388298
H 3.177347 3.234667 -0.258886
H 4.066591 3.190408 1.273037
H 4.705587 2.357293 -0.153904

00OCH2CF3_02.log
Energy (E) = -451.863308475 Hartree
Enthalpy (H) = -451.810956 Hartree
Gibbs free energy (G) = -451.847290
Hartree
Charge = 0, Spin = 2
O -1.966742 -0.186482 0.000169
C -0.971152 0.747089 -0.000659
H -0.963799 1.365446 0.902982
H -0.963949 1.362962 -0.905972
C 0.370756 0.000861 -0.000081
F 0.501194 -0.764356 1.077654
F 0.501351 -0.766462 -1.076261
F 1.360128 0.894790 -0.000718

FuranIBA_A_OCH2CF3.log
Energy (E) = -880.308909433 Hartree
Enthalpy (H) = -880.182604 Hartree
Gibbs free energy (G) = -880.240417
Hartree
Charge = 0, Spin = 1
C 1.242843 2.931075 0.254582
C 0.264526 1.992657 0.380393
C 0.956526 0.776941 0.163744
C 2.257066 1.045148 -0.062579
O 2.447283 2.372343 -0.011456
H 1.225117 4.003209 0.331386
H -0.776039 2.144413 0.605326
C 3.263017 -0.017458 -0.293744
I 0.528632 -1.225119 0.140304
O 4.432284 0.169791 -0.503913
O 2.664284 -1.196394 -0.228168
O -1.440360 -0.740363 0.484610
C -2.156819 -0.355496 -0.647541
H -2.450077 -1.199916 -1.279310
H -1.625491 0.378435 -1.267498
C -3.433051 0.319155 -0.186540
F -4.184758 -0.487924 0.554807
F -3.172485 1.413626 0.538067
F -4.152048 0.697632 -1.248931

IBA_oMe_OCH2CF3.log
Energy (E) = -921.782835894 Hartree
Enthalpy (H) = -921.596588 Hartree
Gibbs free energy (G) = -921.659461
Hartree
Charge = 0, Spin = 1

C 1.561857 2.962945 0.184547
C 2.814948 2.817484 -0.392119
C 3.319315 1.551426 -0.636740
C 2.533551 0.447315 -0.344573
C 1.260645 0.637017 0.158436
C 0.735932 1.877148 0.498109
H 1.199578 3.952380 0.434407
H 3.408631 3.693927 -0.611150
H 4.312397 1.375714 -1.026912
C 3.103673 -0.937845 -0.470014
O 2.303893 -1.863059 0.006224
O 4.203528 -1.136247 -0.924301
I 0.287801 -1.249930 0.371443
C -0.549746 2.144506 1.228033
H -1.413229 2.158744 0.565425
H -0.749877 1.393689 1.988236
H -0.481917 3.119753 1.706623
O -1.594828 -0.420733 0.517408
C -2.180914 -0.133687 -0.711325
H -2.166673 -0.978629 -1.409275
H -1.725215 0.731549 -1.211334
C -3.637989 0.208904 -0.475296
F -4.299759 -0.801363 0.083079
F -3.773258 1.273550 0.319976
F -4.227596 0.495389 -1.642112

IBamideAc_OCH2CF3.log
Energy (E) = -1015.16623966 Hartree
Enthalpy (H) = -1014.956263 Hartree
Gibbs free energy (G) = -1015.025288 Hartree
Charge = 0, Spin = 1
C -0.638388 3.356697 0.336132
C 0.601633 3.917847 0.044656
C 1.706009 3.107386 -0.162304
C 1.572160 1.725422 -0.087391
C 0.325508 1.208327 0.185777
C -0.796301 1.976765 0.413724
H -1.495061 3.992656 0.510228
H 0.703592 4.992526 -0.011587
H 2.688845 3.504540 -0.377983
H -1.746383 1.526583 0.660569
C 2.762600 0.836224 -0.273404
O 3.847622 1.293028 -0.541239
I 0.286294 -0.892178 0.251349
N 2.426111 -0.494043 -0.091737
C 2.777610 -2.915182 0.014014
H 3.582383 -3.639153 -0.060350
H 2.294442 -3.021289 0.987428
H 2.039811 -3.122204 -0.763983
C 3.366454 -1.530352 -0.150892
O 4.547404 -1.361036 -0.312090
O -1.786912 -0.797519 0.528992
C -2.527026 -0.720655 -0.645336
H -2.538139 -1.656497 -1.215061
H -2.199133 0.087068 -1.314582
C -3.965766 -0.412818 -0.283756
F -4.498836 -1.350954 0.493618
F -4.066927 0.755595 0.362284
F -4.707411 -0.326228 -1.394417

IBCCF32O_OCH2CF3.log
Energy (E) = -1482.31577025 Hartree
Enthalpy (H) = -1482.124210 Hartree
Gibbs free energy (G) = -1482.198635 Hartree
Charge = 0, Spin = 1
C 0.616480 3.137256 0.389434
C -0.709482 3.404473 0.076524
C -1.623610 2.371625 -0.077973
C -1.213618 1.051577 0.083533
C 0.115170 0.825644 0.374452
C 1.049906 1.825215 0.539449
H 1.321271 3.946215 0.519514
H -1.038562 4.426819 -0.045091
H -2.652033 2.588275 -0.323852
H 2.073641 1.592323 0.792874
O -1.497347 -1.298057 0.425582
I 0.613849 -1.211958 0.541415
C -2.114841 -0.171483 -0.044866
C -2.458774 -0.384974 -1.538573
C -3.395535 0.000687 0.806519
F -3.017108 0.688377 -2.102081
F -1.320625 -0.638182 -2.196774
F -3.272565 -1.415369 -1.715696
F -4.209325 0.946709 0.320750
F -4.083998 -1.127803 0.870330
F -3.056628 0.352546 2.045397
O 2.607757 -0.677595 0.632078
C 3.209626 -0.477541 -0.608467
H 2.657782 0.224329 -1.247548
H 3.365643 -1.406423 -1.166508
C 4.575073 0.135222 -0.375431
F 4.479831 1.312494 0.254651
F 5.358705 -0.651778 0.355928
F 5.183117 0.350924 -1.547309

IBCH2S_OCH2CF3.log
Energy (E) = -1209.98608829 Hartree
Enthalpy (H) = -1209.754953 Hartree
Gibbs free energy (G) = -1209.820686 Hartree
Charge = 0, Spin = 1
C -0.176605 3.117235 -0.094982
C -1.530871 3.406638 -0.145898
C -2.468885 2.383913 -0.103114
C -2.081535 1.046742 -0.035435
C -0.715797 0.808305 -0.020416
C 0.245911 1.794596 -0.021265
H 0.559141 3.908618 -0.113683
H -1.864232 4.433456 -0.207369
H -3.521950 2.627922 -0.114909
H 1.291941 1.542497 0.056844
I -0.128072 -1.242958 0.021714
C -3.098017 -0.067887 0.140400
S -2.501056 -1.536169 -0.748514
C -4.456693 0.268227 -0.464996
H -5.095514 -0.614045 -0.424707
H -4.950672 1.057444 0.104224
H -4.357918 0.581368 -1.503566
C -3.260426 -0.356871 1.633152
H -3.622552 0.536436 2.147030
H -3.976971 -1.165628 1.774995
H -2.313777 -0.650182 2.087910
O 1.856836 -0.557493 0.579995
C 2.764244 -0.564719 -0.461600
H 2.402280 -0.062864 -1.372907
H 3.105403 -1.567994 -0.749085
C 4.004885 0.196683 -0.040960
F 3.720264 1.473157 0.255634
F 4.592749 -0.340094 1.025480
F 4.902272 0.210408 -1.037635

IBNMeCO2_OCH2CF3.log
Energy (E) = -977.096038357 Hartree
Enthalpy (H) = -976.891202 Hartree
Gibbs free energy (G) = -976.956372 Hartree
Charge = 0, Spin = 1
C -0.148757 3.112591 -0.561938
C -1.470987 3.435373 -0.289936
C -2.393780 2.452344 0.025470
C -2.029882 1.101657 0.082759
C -0.693974 0.822379 -0.201733
C 0.243714 1.783639 -0.515025
H 0.574051 3.879515 -0.798179
H -1.793366 4.466895 -0.324525
H -3.418805 2.725662 0.227855
H 1.273907 1.509002 -0.683956
I -0.126990 -1.185674 -0.090550
C -3.115320 -1.091097 -0.227943
O -4.157188 -1.697356 -0.164172
N -2.958414 0.127374 0.467295
C -4.122516 0.553173 1.231093
H -3.810898 1.272675 1.985430
H -4.892971 0.996764 0.596829
H -4.550296 -0.321048 1.709197
O -2.071789 -1.469406 -0.928720
O 1.728150 -0.578131 0.600306
C 2.787092 -0.766625 -0.282169
H 2.557829 -0.474671 -1.315468
H 3.163199 -1.795257 -0.293514
C 3.939714 0.111100 0.163020
F 3.607436 1.407095 0.133094
F 4.341494 -0.175352 1.397107
F 4.981236 -0.058243 -0.660922

ISOMe_OCH2CF3.log
Energy (E) = -1317.66561429 Hartree
Enthalpy (H) = -1317.506163 Hartree
Gibbs free energy (G) = -1317.568862 Hartree
Charge = 0, Spin = 1
C 0.315781 3.135017 0.132434
C 1.680007 3.350780 -0.021132
C 2.556252 2.278381 -0.115328
C 2.050015 0.988848 -0.072460
C 0.691973 0.805449 0.042799
C -0.203786 1.843726 0.169726
H -0.359895 3.973745 0.221993
H 2.063066 4.360750 -0.057060
H 3.623779 2.417278 -0.220047
H -1.259832 1.670068 0.312458
I 0.058491 -1.219013 0.030210
S 3.112470 -0.433029 -0.025757
O 3.333097 -0.710742 1.367475
O 4.233802 -0.200869 -0.886047
O 2.145058 -1.451586 -0.636066
O -1.831936 -0.625770 0.506833
C -2.682857 -0.457243 -0.590904
H -2.973693 -1.403710 -1.055331
H -2.264638 0.200149 -1.362273
C -3.951929 0.204230 -0.091668
F -4.565617 -0.527625 0.831101
F -3.694926 1.404177 0.440857
F -4.795148 0.382831 -1.112750

NpthIBA_C_OCH2CF3.log
Energy (E) = -1036.01483133 Hartree
Enthalpy (H) = -1035.808277 Hartree
Gibbs free energy (G) = -1035.873668 Hartree
Charge = 0, Spin = 1
C 3.027628 -0.477222 -0.273339
C 1.864925 -1.190660 -0.165014
C 0.687454 -0.494953 0.124456
C 0.612292 0.843575 0.327080
H 3.942560 -1.014134 -0.493096
H -0.315188 1.334800 0.586269
C 1.820294 -2.682082 -0.312764

O 0.617128 -3.180453 -0.122019
O 2.803040 -3.332947 -0.570356
I -0.969445 -1.784321 0.233256
C 1.818684 2.993265 0.400577
C 2.983673 3.700083 0.286379
C 4.195405 3.040196 -0.018765
C 4.217846 1.686052 -0.204092
C 3.029521 0.924151 -0.094294
C 1.812355 1.591334 0.213284
H 0.885088 3.490462 0.631827
H 2.982113 4.771953 0.429574
H 5.107590 3.614415 -0.104798
H 5.141901 1.172701 -0.437208
O -2.187467 -0.141098 0.517992
C -2.605525 0.480563 -0.656966
H -3.366068 -0.090501 -1.199182
H -1.779857 0.703999 -1.345190
C -3.231720 1.811011 -0.291667
F -4.278063 1.667682 0.515407
F -2.352478 2.613874 0.322962
F -3.644589 2.437269 -1.398917

PyrroleNAcIBA_A_OCH2CF3.log
Energy (E) = -1012.98770771 Hartree
Enthalpy (H) = -1012.808260 Hartree
Gibbs free energy (G) = -1012.874646 Hartree
Charge = 0, Spin = 1
C -1.484451 2.116745 -0.443355
C -0.275949 1.477838 -0.502472
C -0.579571 0.137562 -0.190717
C -1.907659 -0.030943 0.039583
H -1.709139 3.159544 -0.581078
H 0.682962 1.901665 -0.742937
C -2.480982 -1.404909 0.136712
I 0.507527 -1.601157 -0.125453
O -3.649109 -1.684026 0.101265
O -1.498015 -2.292699 0.203305
N -2.479824 1.216449 -0.111410
C -3.817777 1.591041 0.241933
C -4.303763 2.864752 -0.391492
H -3.847098 3.725339 0.098585
H -4.058055 2.903661 -1.450952
H -5.377888 2.916331 -0.248259
O -4.452671 0.925940 1.003753
O 2.200042 -0.470201 -0.461304
C 2.719922 0.151461 0.671297
H 3.249789 -0.537374 1.337551
H 1.961939 0.689755 1.255642
C 3.726971 1.188694 0.218181
F 4.723234 0.648487 -0.476719
F 3.155648 2.123807 -0.550309
F 4.251666 1.805670 1.283266

carbazole_02.log
Energy (E) = -516.348102190 Hartree
Enthalpy (H) = -516.174841 Hartree
Gibbs free energy (G) = -516.218917 Hartree
Charge = 0, Spin = 2
N 0.000000 1.692121 -0.000023
C 1.090920 0.862018 -0.000006
C -1.090921 0.862018 -0.000002
C 2.430739 1.250097 0.000006
C 0.730591 -0.512780 -0.000011
C -2.430739 1.250097 0.000013
C -0.730591 -0.512780 -0.000011
C 3.401207 0.254959 0.000013
H 2.689731 2.299691 0.000004
C 1.699782 -1.489359 -0.000004
C -3.401207 0.254959 0.000011
H -2.689731 2.299691 0.000014
C -1.699782 -1.489358 -0.000008
C 3.043736 -1.091856 0.000009
H 4.448108 0.525778 0.000022
H 1.441567 -2.540773 -0.000002
C -3.043736 -1.091856 0.000008
H -4.448108 0.525779 0.000021
H -1.441568 -2.540773 -0.000014
H 3.819478 -1.845601 0.000014
H -3.819478 -1.845601 0.000006

FuranIBA_A_carbazole.log
Energy (E) = -944.768146367 Hartree
Enthalpy (H) = -944.519649 Hartree
Gibbs free energy (G) = -944.585438 Hartree
Charge = 0, Spin = 1
C -1.510740 0.000357 2.994354
C -0.601866 0.000230 1.981667
C -1.415752 0.000091 0.822445
C -2.711557 0.000131 1.189563
O -2.783303 0.000289 2.530046
H -1.396537 0.000486 4.063282
H 0.471896 0.000239 2.056196
C -3.830289 -0.000020 0.210355
I -1.121093 -0.000147 -1.210482
O -4.996689 -0.000022 0.514677
O -3.330527 -0.000163 -1.005292
N 0.942150 -0.000073 -0.892039
C 1.641138 1.127266 -0.455992
C 1.641184 -1.127326 -0.455839
C 1.312290 2.471480 -0.593675
C 2.827143 0.723291 0.184709
C 1.312394 -2.471573 -0.593335
C 2.827171 -0.723212 0.184810
C 2.199455 3.408253 -0.088803
H 0.396754 2.782633 -1.080130
C 3.709405 1.682204 0.677152
C 2.199598 -3.408237 -0.088328
H 0.396873 -2.782835 -1.079747
C 3.709471 -1.682019 0.677392
C 3.389194 3.021436 0.537160
H 1.966636 4.460159 -0.182643
H 4.628479 1.382520 1.164626
C 3.389318 -3.021283 0.537585
H 1.966823 -4.460166 -0.182024
H 4.628531 -1.382226 1.164827
H 4.061039 3.778980 0.916071
H 4.061193 -3.778747 0.916604

IBA_oMe_carbazole.log
Energy (E) = -986.241295794 Hartree
Enthalpy (H) = -985.932701 Hartree
Gibbs free energy (G) = -986.002608 Hartree
Charge = 0, Spin = 1
C 2.026709 -1.404966 2.662848
C 3.381502 -1.573486 2.420367
C 3.960743 -0.978048 1.314056
C 3.163606 -0.257285 0.436511
C 1.806877 -0.167394 0.681263
C 1.182400 -0.686561 1.808983
H 1.586764 -1.823542 3.559370
H 3.984402 -2.140805 3.115659
H 5.019594 -1.028074 1.100292
C 3.807135 0.493045 -0.703537
O 2.964109 1.243544 -1.353536
O 4.992919 0.398015 -0.920561
I 0.851770 0.866239 -0.952777
C -0.242505 -0.478903 2.235564
H -0.549700 0.559853 2.120147
H -0.944006 -1.098170 1.676573
H -0.333424 -0.739670 3.288004
N -1.094434 0.266973 -0.499504
C -1.605780 -1.003242 -0.737099
C -2.093610 1.036104 0.082860
C -0.971190 -2.124386 -1.264037
C -2.949262 -1.049075 -0.315624
C -2.047039 2.356365 0.520510
C -3.262036 0.260853 0.210189
C -1.713249 -3.288206 -1.383878
H 0.065985 -2.095382 -1.572786
C -3.675643 -2.230428 -0.447249
C -3.195648 2.892735 1.079187
H -1.145645 2.949330 0.429076
C -4.406661 0.822783 0.771704
C -3.052943 -3.343892 -0.983871
H -1.244671 -4.172679 -1.793533
H -4.710068 -2.275624 -0.130802
C -4.367088 2.137245 1.202745
H -3.185741 3.917188 1.425831
H -5.311908 0.237131 0.870739
H -3.601898 -4.269003 -1.092246
H -5.246588 2.587921 1.641190

IBamideAc_carbazole.log
Energy (E) = -1079.62161894 Hartree
Enthalpy (H) = -1079.289443 Hartree
Gibbs free energy (G) = -1079.366220 Hartree
Charge = 0, Spin = 1
C -0.056850 3.408914 -0.002735
C 1.220957 3.959036 -0.003334
C 2.335305 3.136789 -0.002773
C 2.179569 1.754614 -0.001616
C 0.898487 1.248844 -0.001070
C -0.238357 2.030001 -0.001590
H -0.926366 4.051116 -0.003154
H 1.342597 5.033213 -0.004218
H 3.345927 3.522626 -0.003197
H -1.231640 1.606161 -0.001143
C 3.388930 0.864715 -0.001021
O 4.500513 1.340656 -0.001605
I 0.814636 -0.865336 0.000621
N 3.027121 -0.464256 0.000174
C 3.356312 -2.886391 0.002242
H 4.161015 -3.614459 0.002850
H 2.736653 -3.040802 0.888023
H 2.736809 -3.042046 -0.883425
C 3.965923 -1.499085 0.001371
O 5.160818 -1.340077 0.001876
N -1.304963 -0.792622 0.000658
C -2.090957 -0.554847 1.124363
C -2.091171 -0.556996 -1.123350
C -1.740102 -0.611345 2.470682
C -3.398573 -0.217206 0.722926
C -1.740561 -0.616110 -2.469624
C -3.398691 -0.218521 -0.722307
C -2.722468 -0.338958 3.407760
H -0.734029 -0.862621 2.781500
C -4.372997 0.047080 1.683653
C -2.723073 -0.345437 -3.407040
H -0.734549 -0.868023 -2.780120
C -4.373264 0.044008 -1.683367
C -4.028872 -0.016129 3.022174
H -2.474322 -0.377317 4.459811
H -5.382381 0.300633 1.384909
C -4.029380 -0.021767 -3.021824
H -2.475128 -0.385805 -4.459063

H -5.382591 0.298158 -1.384938
H -4.771915 0.187637 3.780696
H -4.772553 0.180587 -3.780597

IBCCF32O_carbazole.log
Energy (E) = -1546.77323743 Hartree
Enthalpy (H) = -1546.459522 Hartree
Gibbs free energy (G) = -1546.541735 Hartree
Charge = 0, Spin = 1
C -0.100218 0.074011 3.121855
C 1.253470 0.168239 3.413609
C 2.199865 0.116794 2.400753
C 1.797577 -0.041129 1.077122
C 0.444429 -0.122958 0.826346
C -0.523983 -0.066838 1.806138
H -0.834978 0.112109 3.913465
H 1.577276 0.284999 4.438286
H 3.250124 0.202561 2.634455
H -1.576388 -0.135765 1.572364
O 2.089827 -0.525262 -1.242856
I -0.048452 -0.305222 -1.226890
C 2.740555 -0.108290 -0.122341
C 3.876980 -1.127507 0.132713
C 3.310518 1.308746 -0.378979
F 4.735461 -0.718748 1.077415
F 3.351894 -2.283928 0.536546
F 4.574422 -1.354082 -0.969416
F 3.910494 1.837155 0.690746
F 4.176650 1.318219 -1.382332
F 2.291865 2.116115 -0.704785
N -2.076661 -0.075971 -0.772352
C -2.677486 1.135854 -0.442271
C -2.942319 -1.099945 -0.395523
C -2.177431 2.428130 -0.572977
C -3.955063 0.892950 0.097267
C -2.765652 -2.477787 -0.481011
C -4.125285 -0.542361 0.127333
C -2.989002 3.476473 -0.172857
H -1.187977 2.612778 -0.971809
C -4.756253 1.963920 0.488474
C -3.799258 -3.289525 -0.044489
H -1.853527 -2.905637 -0.876588
C -5.154671 -1.378885 0.554783
C -4.268233 3.251072 0.348777
H -2.624753 4.490673 -0.264804
H -5.743103 1.789257 0.898303
C -4.985368 -2.749206 0.465719
H -3.686734 -4.363762 -0.100598
H -6.070862 -0.960788 0.952410
H -4.875804 4.093685 0.648614
H -5.773428 -3.412592 0.794194

IBCH2S_carbazole.log
Energy (E) = -1274.44101408 Hartree
Enthalpy (H) = -1274.087670 Hartree
Gibbs free energy (G) = -1274.160322 Hartree
Charge = 0, Spin = 1
C 0.344075 1.351957 2.739280
C 1.638961 1.794835 2.956424
C 2.662166 1.424610 2.094315
C 2.422876 0.596684 0.998718
C 1.112097 0.183215 0.823787
C 0.066532 0.534662 1.649374
H -0.455577 1.628784 3.411247
H 1.859010 2.430765 3.802869
H 3.665559 1.783663 2.275158
H -0.937710 0.176227 1.476302
I 0.762885 -1.103263 -0.852536
C 3.519145 0.235514 0.010258
S 3.249177 -1.480974 -0.531291
C 4.908773 0.268774 0.637266
H 5.632159 -0.123615 -0.077319
H 5.204045 1.291641 0.877872
H 4.946156 -0.338823 1.540588
C 3.473159 1.210938 -1.167013
H 3.654984 2.227980 -0.812274
H 4.238102 0.944353 -1.895860
H 2.502825 1.196222 -1.664862
N -1.365932 -0.439461 -0.797324
C -1.735911 0.883246 -0.963161
C -2.370977 -1.057478 -0.070184
C -1.030793 1.919316 -1.576889
C -3.004794 1.113465 -0.387351
C -2.441470 -2.374223 0.385593
C -3.416824 -0.143678 0.190352
C -1.618886 3.170995 -1.623641
H -0.053416 1.750300 -2.013580
C -3.581306 2.380851 -0.452280
C -3.568422 -2.761640 1.089319
H -1.641223 -3.077083 0.190442
C -4.546905 -0.559077 0.893272
C -2.885159 3.403887 -1.071182
H -1.090170 3.987263 -2.097830
H -4.558547 2.561638 -0.021487
C -4.616726 -1.866568 1.340002
H -3.642960 -3.778753 1.450651
H -5.356750 0.133461 1.087661
H -3.316922 4.393730 -1.128917
H -5.485455 -2.204733 1.888144

IBNMeCO2_carbazole.log
Energy (E) = -1041.55703691 Hartree
Enthalpy (H) = -1041.230013 Hartree
Gibbs free energy (G) = -1041.302634 Hartree
Charge = 0, Spin = 1
C 0.429505 1.368660 2.828264
C 1.678067 1.976313 2.837734
C 2.637605 1.653026 1.893366
C 2.384533 0.707217 0.891163
C 1.115908 0.131400 0.913890
C 0.145229 0.431095 1.847285
H -0.318186 1.622195 3.565186
H 1.914357 2.710363 3.595830
H 3.605912 2.130005 1.929426
H -0.826209 -0.044293 1.803452
I 0.675204 -1.209492 -0.635620
C 3.670300 -0.920069 -0.443716
O 4.760667 -1.154271 -0.910678
N 3.338197 0.417695 -0.088626
C 4.394390 1.391563 -0.320694
H 3.962555 2.390543 -0.336503
H 5.177100 1.342974 0.439786
H 4.851992 1.171372 -1.278732
O 2.734461 -1.797610 -0.215489
N -1.268197 -0.433902 -0.718800
C -1.547287 0.904866 -0.971946
C -2.374058 -0.986184 -0.075861
C -0.727835 1.878627 -1.535035
C -2.857445 1.200135 -0.550802
C -2.553218 -2.275536 0.416649
C -3.389568 -0.014956 0.024140
C -1.253424 3.150019 -1.693749
H 0.287493 1.654572 -1.838830
C -3.367721 2.484466 -0.726056
C -3.770721 -2.580130 1.003601
H -1.772628 -3.021907 0.343311
C -4.609220 -0.346852 0.609697
C -2.561731 3.452515 -1.299169
H -0.638787 3.925360 -2.130681
H -4.377080 2.721081 -0.413790
C -4.792753 -1.629205 1.097200
H -3.934190 -3.575390 1.394128
H -5.399145 0.389860 0.684491
H -2.940692 4.455297 -1.440228
H -5.732645 -1.902309 1.556545

ISOMe_carbazole.log
Energy (E) = -1382.12892749 Hartree
Enthalpy (H) = -1381.847279 Hartree
Gibbs free energy (G) = -1381.917657 Hartree
Charge = 0, Spin = 1
C -0.690083 0.768037 3.050912
C -2.045487 0.983728 3.268114
C -2.971655 0.722555 2.266885
C -2.526759 0.241205 1.046114
C -1.181780 0.021574 0.866222
C -0.232544 0.277358 1.830536
H 0.024993 0.971479 3.835317
H -2.381952 1.356266 4.225424
H -4.032581 0.877002 2.409754
H 0.820973 0.096649 1.667608
I -0.636066 -0.760630 -1.041929
S -3.618207 -0.035146 -0.331724
O -3.508402 1.152390 -1.143351
O -4.903065 -0.412435 0.180884
O -2.881206 -1.197284 -0.970192
N 1.325028 -0.253514 -0.737773
C 1.788555 1.063949 -0.714945
C 2.291469 -1.068697 -0.143014
C 1.147313 2.225400 -1.129137
C 3.082480 1.083617 -0.166118
C 2.260922 -2.436141 0.102715
C 3.404368 -0.279255 0.199482
C 1.843825 3.416679 -1.007326
H 0.140083 2.208402 -1.526346
C 3.765314 2.293241 -0.058811
C 3.375786 -3.007672 0.694562
H 1.400515 -3.039014 -0.157648
C 4.517174 -0.877466 0.786699
C 3.140978 3.452846 -0.483937
H 1.371731 4.337340 -1.321323
H 4.765283 2.323933 0.354701
C 4.495476 -2.239626 1.030809
H 3.379289 -4.069656 0.898245
H 5.383015 -0.283337 1.049675
H 3.655346 4.400567 -0.406098
H 5.350202 -2.718658 1.487639

NpthIBA_C_carbazole.log
Energy (E) = -1100.47266761 Hartree
Enthalpy (H) = -1100.143981 Hartree
Gibbs free energy (G) = -1100.217392 Hartree
Charge = 0, Spin = 1
C -3.582170 0.374518 -0.000009
C -2.680339 -0.654859 -0.000002
C -1.318994 -0.337046 0.000004
C -0.821669 0.925220 0.000005
H -4.637473 0.129479 -0.000011
H 0.238442 1.141410 0.000012
C -3.114735 -2.095296 0.000009
O -2.108554 -2.929172 0.000003
O -4.285421 -2.398522 0.000006
I -0.099414 -2.067366 0.000010
C -1.315041 3.342619 -0.000006
C -2.224065 4.363920 -0.000015

C -3.610278 4.089252 -0.000022
C -4.058858 2.798163 -0.000020
C -3.144269 1.717135 -0.000012
C -1.750970 1.996907 -0.000004
H -0.251336 3.544268 -0.000001
H -1.884210 5.390584 -0.000018
H -4.315874 4.908696 -0.000029
H -5.118806 2.578338 -0.000026
N 1.619713 -0.881813 0.000007
C 2.150180 -0.256217 -1.126501
C 2.150175 -0.256198 1.126507
C 1.887116 -0.496626 -2.471526
C 3.064758 0.735230 -0.722914
C 1.887110 -0.496589 2.471536
C 3.064753 0.735243 0.722908
C 2.565927 0.264869 -3.408091
H 1.178449 -1.253765 -2.781438
C 3.741033 1.485456 -1.683091
C 2.565916 0.264922 3.408090
H 1.178444 -1.253726 2.781457
C 3.741024 1.485486 1.683076
C 3.487421 1.244934 -3.021809
H 2.379514 0.097227 -4.460141
H 4.451681 2.245536 -1.383805
C 3.487408 1.244983 3.021796
H 2.379502 0.097295 4.460143
H 4.451672 2.245562 1.383781
H 4.002084 1.818745 -3.779971
H 4.002068 1.818808 3.779950

PyrroleNAcIBA_A_carbazole.log
Energy (E) = -1077.44609020 Hartree
Enthalpy (H) = -1077.144459 Hartree
Gibbs free energy (G) = -1077.218861 Hartree
Charge = 0, Spin = 1
C 1.753903 2.050604 0.826346
C 0.625528 1.336501 0.531552
C 1.115333 0.112604 0.030278
C 2.472619 0.083242 0.020979
H 1.844131 3.055479 1.199092
H -0.397646 1.649915 0.650896
C 3.200937 -1.196255 -0.250097
I 0.214253 -1.630814 -0.592157
O 4.367696 -1.384776 -0.019126
O 2.355101 -2.093626 -0.711239
N 2.878413 1.305997 0.516723
C 4.207580 1.839910 0.487831
C 4.452595 2.970275 1.448961
H 3.999334 3.887545 1.071403
H 4.032470 2.759351 2.430398
H 5.525022 3.119273 1.517897
O 5.020991 1.409959 -0.273500
N -1.653319 -0.735126 -0.293158
C -2.242981 -0.576053 0.959822
C -2.190082 0.231299 -1.142090
C -1.995655 -1.266765 2.141959
C -3.210807 0.444569 0.895377
C -1.876601 0.506992 -2.469238
C -3.176867 0.962790 -0.453994
C -2.742826 -0.926558 3.257603
H -1.247351 -2.047126 2.193960
C -3.956230 0.764884 2.027993
C -2.579296 1.518999 -3.102232
H -1.111116 -0.048516 -2.995662
C -3.876608 1.971502 -1.112758
C -3.717299 0.076219 3.204342
H -2.569371 -1.449070 4.188524
H -4.708130 1.542881 1.986970
C -3.573378 2.243601 -2.435311
H -2.354349 1.752376 -4.134034
H -4.642931 2.534702 -0.595193
H -4.285396 0.313246 4.093186
H -4.105259 3.024395 -2.961069

IBA_5FBz_02.log
Energy (E) = -915.778050396 Hartree
Enthalpy (H) = -915.704698 Hartree
Gibbs free energy (G) = -915.756623 Hartree
Charge = 0, Spin = 2
C -1.866050 0.000120 0.000010
C -1.176267 1.203588 0.000039
C 0.207652 1.196467 0.000121
C 0.925655 0.000034 0.000146
C 0.207059 -1.196365 0.000102
C -1.176649 -1.203840 0.000062
F 0.827837 2.360447 0.000075
F 0.827610 -2.360434 -0.000290
F -1.843032 -2.344455 0.000039
F -3.182204 0.000419 -0.000136
F -1.842413 2.344521 0.000047
C 2.399649 -0.000072 -0.000102
O 3.111536 1.029700 -0.000471
O 3.111405 -1.030209 0.000485

IBA_5FPh_02.log
Energy (E) = -727.319355027 Hartree
Enthalpy (H) = -727.261652 Hartree
Gibbs free energy (G) = -727.305800 Hartree
Charge = 0, Spin = 2
C 1.210119 0.386011 0.000132
C -0.000001 1.067968 0.000099
C -1.210078 0.386124 0.000179
C -1.213294 -1.004752 0.000198
C -0.000049 -1.631612 0.000110
C 1.213297 -1.004769 0.000323
F -2.364339 -1.660568 -0.000250
F -2.346087 1.065501 -0.000029
F 0.000143 2.391002 -0.000109
F 2.346050 1.065538 -0.000115
F 2.364237 -1.660785 -0.000191

IBA_Acr_02.log
Energy (E) = -554.367628926 Hartree
Enthalpy (H) = -554.187683 Hartree
Gibbs free energy (G) = -554.233346 Hartree
Charge = 0, Spin = 2
C 3.561719 -0.738145 -0.000021
C 2.381536 -1.413362 -0.000023
C 1.141441 -0.708433 0.000064
C 1.170068 0.723188 0.000012
C 2.427008 1.393960 0.000003
C 3.587563 0.686085 0.000004
C -0.045094 1.397890 0.000013
C -1.239008 0.686205 0.000001
C -1.150136 -0.750229 -0.000052
C -2.392533 -1.418909 0.000008
C -3.605403 -0.847385 0.000047
C -3.665549 0.584353 -0.000024
C -2.519336 1.315925 -0.000017
H 4.496729 -1.282192 -0.000032
H 2.334557 -2.493499 -0.000034
H 2.433981 2.477167 -0.000000
H 4.539288 1.199691 0.000006
H -0.062148 2.482859 0.000015
H -4.517856 -1.429922 -0.000021
H -4.630123 1.074198 -0.000041
H -2.555719 2.398274 -0.000015
N 0.006806 -1.413349 0.000004

IBA_Adm_02.log
Energy (E) = -389.685207654 Hartree
Enthalpy (H) = -389.447179 Hartree
Gibbs free energy (G) = -389.486793 Hartree
Charge = 0, Spin = 2
C 1.390358 -0.374544 1.069971
H 1.660669 -1.366908 1.441845
H 2.121887 0.347363 1.443916
C 0.000161 -0.001109 1.463348
C 1.400733 -0.375820 -0.476052
H 2.394969 -0.642733 -0.842643
C 0.372581 -1.392852 -0.975421
H 0.640251 -2.394510 -0.625625
H 0.378568 -1.415325 -2.069410
C -1.018869 -1.017919 1.069534
H -2.013117 -0.756212 1.442525
H -0.759301 -2.012911 1.442044
C -1.026080 -1.024384 -0.476113
H -1.754526 -1.751564 -0.843168
C -1.393714 0.374921 -0.973832
H -1.417275 0.381946 -2.067792
H -2.394660 0.643559 -0.622741
C -0.371020 1.390087 1.071255
H 0.353282 2.119733 1.444274
H -1.362058 1.662589 1.445011
C 1.020593 1.020160 -0.974080
H 1.754080 1.752405 -0.623159
H 1.037505 1.037709 -2.068038
C -0.374701 1.401244 -0.474736
H -0.640531 2.396156 -0.840286

IBA_Allene_02.log
Energy (E) = -115.889819439 Hartree
Enthalpy (H) = -115.843393 Hartree
Gibbs free energy (G) = -115.872728 Hartree
Charge = 0, Spin = 2
C -0.116903 -0.000033 0.000132
C -1.336168 0.000007 -0.000085
H -2.397817 0.000026 0.000036
C 1.251707 0.000006 -0.000024
H 1.802940 0.927423 -0.000089
H 1.803063 -0.927333 -0.000090

IBA_Anth2_02.log
Energy (E) = -538.335846937 Hartree
Enthalpy (H) = -538.144060 Hartree
Gibbs free energy (G) = -538.189939 Hartree
Charge = 0, Spin = 2
C 3.573902 -0.758292 -0.000018
C 2.388149 -1.424086 -0.000029
C 1.151213 -0.715124 0.000007
C 1.177421 0.715179 0.000031
C 2.440659 1.376074 0.000018
C 3.600051 0.665083 -0.000004
C -0.077265 -1.372010 0.000003
C -0.025604 1.418782 0.000047
C -1.252230 0.761577 0.000049
C -1.281443 -0.672107 0.000013
C -2.537639 -1.351757 -0.000036
H -2.535555 -2.435578 -0.000056
C -3.713744 -0.659206 0.000108
C -3.622532 0.740433 -0.000042
C -2.499705 1.471744 -0.000106
H -0.096188 -2.456616 -0.000029

H 4.506232 -1.306619 -0.000029
H 2.359979 -2.506995 -0.000042
H 2.454302 2.459202 0.000022
H 4.552308 1.178174 -0.000018
H -0.005224 2.503138 0.000030
H -4.668141 -1.167764 0.000005
H -2.495116 2.555311 -0.000124

IBA_Bn_02.log
Energy (E) = -270.651950934 Hartree
Enthalpy (H) = -270.530425 Hartree
Gibbs free energy (G) = -270.566751 Hartree
Charge = 0, Spin = 2
C 1.126980 -1.206457 -0.000018
C 1.828834 0.000015 -0.000010
C 1.126966 1.206470 0.000001
C -0.251361 1.212070 -0.000012
C -0.988116 -0.000017 0.000080
C -0.251341 -1.212092 0.000050
H 1.668270 -2.143299 -0.000076
H 2.909914 0.000015 -0.000082
H 1.668227 2.143329 0.000018
H -0.795158 2.148660 0.000003
H -0.795120 -2.148693 0.000015
C -2.387486 0.000003 -0.000038
H -2.941518 -0.926625 -0.000171
H -2.941467 0.926658 -0.000021

IBA_Carbazole_02.log
Energy (E) = -516.348100928 Hartree
Enthalpy (H) = -516.174835 Hartree
Gibbs free energy (G) = -516.218913 Hartree
Charge = 0, Spin = 2
C -3.043769 1.091792 0.000015
C -3.401150 -0.255345 -0.000170
C -2.430543 -1.250161 -0.000096
C -1.090758 -0.861582 0.000139
C -0.730864 0.512817 -0.000059
C -1.700049 1.489490 0.000023
H -3.820216 1.844797 0.000207
H -4.448127 -0.525532 -0.000454
H -2.688608 -2.299983 -0.000399
H -1.441052 2.540688 0.000214
C 1.090761 -0.861579 0.000138
C 2.430528 -1.250167 -0.000087
C 3.401147 -0.255347 -0.000164
C 3.043780 1.091786 0.000005
C 1.700057 1.489485 0.000012
C 0.730875 0.512808 -0.000058
H 2.688606 -2.299985 -0.000371
H 4.448120 -0.525551 -0.000436
H 3.820230 1.844786 0.000185
H 1.441052 2.540681 0.000189
N -0.000013 -1.691985 0.000382

IBA_CCPh_02.log
Energy (E) = -307.394408491 Hartree
Enthalpy (H) = -307.289369 Hartree
Gibbs free energy (G) = -307.328010 Hartree
Charge = 0, Spin = 2
C -3.341434 -0.000300 0.000578
C -2.065368 0.000482 -0.000394
C -0.670867 0.000223 -0.000255
C 0.046428 -1.218494 -0.000136
C 0.046823 1.218692 -0.000143
C 1.425035 -1.212957 0.000071
H -0.513095 -2.143221 -0.000297
C 1.425426 1.212682 0.000077
H -0.512388 2.143613 -0.000303
C 2.114693 -0.000249 0.000184
H 1.972058 -2.145224 0.000165
H 1.972757 2.144770 0.000175
H 3.196255 -0.000418 0.000371

IBA_Cy_02.log
Energy (E) = -234.973797953 Hartree
Enthalpy (H) = -234.811246 Hartree
Gibbs free energy (G) = -234.848158 Hartree
Charge = 0, Spin = 2
C -1.273224 -0.768684 0.177641
C -1.254070 0.702108 -0.252998
C 0.000044 1.393726 0.268259
C 1.254112 0.702034 -0.253011
C 1.273175 -0.768752 0.177648
C -0.000039 -1.444988 -0.180724
H 0.000071 2.447162 -0.016513
H -1.261158 0.754039 -1.345860
H -2.152179 1.209943 0.102715
H -1.409687 -0.790845 1.271757
H -2.130848 -1.288368 -0.250348
H 1.261185 0.753951 -1.345874
H 2.152258 1.209817 0.102683
H 2.130786 -1.288496 -0.250292
H 1.409604 -0.790891 1.271774
H -0.000073 -2.467504 -0.524880
H 0.000052 1.358525 1.363954

IBA_Dibenzofuran_02.log
Energy (E) = -536.175831501 Hartree
Enthalpy (H) = -536.014340 Hartree
Gibbs free energy (G) = -536.058100 Hartree
Charge = 0, Spin = 2
C -3.112631 1.086438 -0.000006
C -3.364421 -0.267372 -0.000041
C -2.455108 -1.288851 -0.000029
C -1.137321 -0.846747 -0.000021
C -0.786796 0.509535 0.000045
C -1.779005 1.487222 0.000091
H -3.911829 1.814875 -0.000041
H -2.713414 -2.338416 -0.000075
H -1.520368 2.538599 0.000092
C 1.048253 -0.816821 0.000027
C 2.368591 -1.225056 0.000037
C 3.329080 -0.223158 0.000001
C 2.971651 1.128590 -0.000035
C 1.640949 1.515576 -0.000051
C 0.661888 0.525864 -0.000019
H 2.630235 -2.273199 0.000058
H 4.375926 -0.494455 0.000007
H 3.748242 1.880959 -0.000044
H 1.369526 2.563145 -0.000061
O -0.036137 -1.650353 0.000008

IBA_Furane3_02.log
Energy (E) = -229.142089129 Hartree
Enthalpy (H) = -229.079437 Hartree
Gibbs free energy (G) = -229.110829 Hartree
Charge = 0, Spin = 2
C 0.742300 0.787306 -0.000021
C 1.113867 -0.518439 0.000009
C -0.119050 -1.216999 0.000001
C -1.134665 -0.338494 -0.000020
O -0.603822 0.917552 0.000025
H 1.305563 1.703470 -0.000042
H 2.115555 -0.908898 0.000028
H -2.205250 -0.415232 -0.000007

IBA_Mesitylene_02.log
Energy (E) = -349.169390480 Hartree
Enthalpy (H) = -348.988661 Hartree
Gibbs free energy (G) = -349.038192 Hartree
Charge = 0, Spin = 2
C -1.221866 0.613873 -0.002483
C -0.043954 1.359702 -0.003626
C 1.178637 0.696366 -0.003529
C 1.255334 -0.701454 0.000040
C 0.047456 -1.347799 0.002653
C -1.203760 -0.782340 0.000774
H -2.178665 1.127690 -0.005583
H 2.098380 1.273213 -0.007540
C 2.573955 -1.423787 0.000771
H 2.659601 -2.082946 -0.862856
H 3.400612 -0.715352 -0.025269
H 2.680693 -2.041454 0.892489
C -2.468509 -1.595031 0.000273
H -2.518654 -2.239266 -0.877452
H -2.519655 -2.238678 0.878357
H -3.343048 -0.946130 -0.000448
C -0.103008 2.863007 0.003158
H -0.523486 3.235488 0.938542
H 0.889783 3.295462 -0.112459
H -0.731278 3.236754 -0.805979

IBA_Naph1_02.log
Energy (E) = -384.840117118 Hartree
Enthalpy (H) = -384.697433 Hartree
Gibbs free energy (G) = -384.737077 Hartree
Charge = 0, Spin = 2
C 2.427363 -0.683298 0.000025
C 1.269979 -1.411637 -0.000075
C 0.015319 -0.756201 0.000124
C -0.034822 0.668276 -0.000072
C 1.184862 1.386169 -0.000042
C 2.384300 0.728912 0.000069
H 3.384101 -1.187736 0.000067
H 1.290939 -2.493021 -0.000213
C -1.222517 -1.401470 -0.000026
C -1.295977 1.315600 -0.000038
H 1.148064 2.468943 -0.000119
H 3.309007 1.290092 0.000099
C -2.464241 0.603594 0.000084
C -2.437724 -0.817715 -0.000016
H -1.316407 2.398374 -0.000119
H -3.416953 1.116902 0.000073
H -3.358001 -1.386929 0.000013

IBA_Naph2_02.log
Energy (E) = -384.840558154 Hartree
Enthalpy (H) = -384.698064 Hartree
Gibbs free energy (G) = -384.737679 Hartree
Charge = 0, Spin = 2
C -2.331013 -0.790935 -0.000008
C -1.117308 -1.421314 -0.000023
C 0.087945 -0.678888 -0.000006
C 0.017088 0.739963 0.000052
C -1.251628 1.365683 0.000040
C -2.398189 0.619456 0.000032
H 1.382651 -2.404592 -0.000112
H -3.244371 -1.370201 -0.000043
H -1.058222 -2.503041 -0.000068
C 1.351688 -1.321086 -0.000053

C 1.228421 1.489268 -0.000100
H -1.296213 2.447891 0.000034
H -3.363127 1.108440 0.000014
C 2.382532 0.793790 -0.000028
C 2.515064 -0.591842 0.000143
H 1.189090 2.572159 -0.000152
H 3.482595 -1.075228 0.000029

IBA_OCH2CF3_02.log
Energy (E) = -451.863308129 Hartree
Enthalpy (H) = -451.810944 Hartree
Gibbs free energy (G) = -451.847285 Hartree
Charge = 0, Spin = 2
O 1.966704 -0.186565 -0.000251
C 0.971200 0.747014 0.001022
H 0.963849 1.365470 -0.902354
H 0.964015 1.361582 0.907005
C -0.370969 0.000543 0.000091
F -0.501381 -0.763652 -1.078260
F -0.501563 -0.767057 1.076014
F -1.359599 0.895168 0.001211

IBA_OEt_02.log
Energy (E) = -154.237834418 Hartree
Enthalpy (H) = -154.166474 Hartree
Gibbs free energy (G) = -154.197127 Hartree
Charge = 0, Spin = 2
O 1.181075 -0.399061 0.000002
C 0.201155 0.553625 0.000007
H 0.243676 1.180613 0.898456
H 0.243642 1.180528 -0.898511
C -1.131405 -0.217079 0.000004
H -1.201953 -0.843293 0.886107
H -1.950973 0.499324 -0.000476
H -1.201487 -0.843963 -0.885662

IBA_Pyrene_02.log
Energy (E) = -614.511736200 Hartree
Enthalpy (H) = -614.305755 Hartree
Gibbs free energy (G) = -614.352727 Hartree
Charge = 0, Spin = 2
C 3.523622 -0.109614 -0.000002
C 2.816535 -1.303518 -0.000080
C 1.421407 -1.291167 0.000052
C 0.724391 -0.050510 -0.000019
C 1.460764 1.158788 0.000028
C 2.855914 1.106506 0.000079
C 0.600002 -2.443000 -0.000029
C -0.699164 -0.025380 -0.000000
C -1.446577 -1.231375 0.000046
C -0.731432 -2.487042 -0.000003
C -2.839942 -1.173025 0.000030
H -3.404412 -2.097190 0.000043
C -3.495903 0.050140 -0.000002
C -2.774483 1.234145 -0.000020
C -1.378396 1.217862 -0.000022
C -0.607514 2.428552 -0.000041
C 0.743364 2.401566 -0.000001
H 1.315789 3.321189 0.000019
H -1.140936 3.371296 -0.000079
H 4.605053 -0.128144 0.000005
H 3.333792 -2.253166 -0.000227
H 3.413510 2.035037 0.000117
H -1.293453 -3.413801 0.000089
H -4.577263 0.079312 -0.000015
H -3.287610 2.187904 -0.000045

IBA_SeMes_02.log
Energy (E) = -358.533851269 Hartree
Enthalpy (H) = -358.349419 Hartree
Gibbs free energy (G) = -358.400241 Hartree
Charge = 0, Spin = 2
C -0.405989 1.216344 -0.003152
C -1.795866 1.192397 -0.008947
C -2.511639 0.000985 -0.008719
C -1.796037 -1.191355 -0.008981
C -0.406878 -1.215849 -0.003162
C 0.277907 0.000422 0.001107
H -2.331678 2.135964 -0.016391
H -2.332243 -2.134776 -0.016539
C 0.349856 -2.509910 -0.005787
H 0.998521 -2.580556 -0.883354
H 0.992344 -2.587462 0.875703
H -0.324771 -3.364407 -0.011365
C 0.350992 2.510272 -0.005799
H 0.993518 2.587714 0.875676
H 0.999611 2.580816 -0.883413
H -0.323413 3.364948 -0.011354
C -4.014223 -0.000358 0.015841
H -4.416076 0.914033 -0.419228
H -4.417104 -0.848028 -0.537867
H -4.389296 -0.070203 1.038791
Se 2.184172 -0.000463 0.004557

IBA_SetBu_02.log
Energy (E) = -166.964600007 Hartree
Enthalpy (H) = -166.836080 Hartree
Gibbs free energy (G) = -166.875684 Hartree
Charge = 0, Spin = 2
Se 1.183632 -0.000054 -0.022715
C -0.794722 -0.000023 -0.006923
C -1.314955 -1.253787 -0.690754
H -0.946643 -2.151087 -0.194838
H -2.409002 -1.258909 -0.658012
H -1.008708 -1.290204 -1.736695
C -1.180896 0.001724 1.468122
H -0.805003 0.889427 1.977878
H -2.272955 0.002114 1.543538
H -0.805493 -0.885022 1.979893
C -1.314760 1.252265 -0.693640
H -1.008294 1.286383 -1.739579
H -2.408823 1.257474 -0.661226
H -0.946574 2.150574 -0.199473

IBA_SMes_02.log
Energy (E) = -747.324843382 Hartree
Enthalpy (H) = -747.139963 Hartree
Gibbs free energy (G) = -747.189963 Hartree
Charge = 0, Spin = 2
C -1.261782 1.207248 -0.000789
C -1.977245 0.010827 -0.000858
C -1.268940 -1.194927 -0.000778
C 0.109746 -1.229991 -0.000210
C 0.839089 -0.001207 0.000094
C 0.121102 1.230952 -0.000234
H -1.804902 2.145616 -0.001360
H -1.820438 -2.128890 -0.001361
C 0.845097 2.541487 -0.000147
H 1.493367 2.628083 0.871874
H 1.494456 2.627719 -0.871376
H 0.136766 3.368370 -0.000720
C -3.475458 0.004490 0.001037
H -3.858844 -0.495310 0.892153
H -3.878007 1.015326 -0.023524
H -3.861872 -0.540643 -0.861482
C 0.825960 -2.544999 -0.000157
H 1.473846 -2.635474 0.871735
H 0.112430 -3.367433 -0.000487
H 1.474491 -2.635299 -0.871581
S 2.530830 -0.007835 0.000523

IBA_SO3Ph_02.log
Energy (E) = -854.954539544 Hartree
Enthalpy (H) = -854.841775 Hartree
Gibbs free energy (G) = -854.885790 Hartree
Charge = 0, Spin = 2
C -2.973093 0.000030 -0.064385
C -2.289260 1.217713 -0.050817
C -0.910266 1.227541 -0.021057
C -0.235119 -0.000091 -0.041001
C -0.910334 -1.227670 -0.021021
C -2.289328 -1.217693 -0.050849
H -4.054536 0.000088 -0.078427
H -2.839994 2.147309 -0.060689
H -0.334370 2.142269 -0.010600
H -0.334478 -2.142411 -0.010547
H -2.840094 -2.147274 -0.060835
S 1.525078 -0.000044 -0.058773
O 2.028805 -1.239872 -0.572060
O 2.028975 1.239060 -0.573684
O 1.398047 0.001029 1.477776

IBA_StBu_02.log
Energy (E) = -555.740056569 Hartree
Enthalpy (H) = -555.611151 Hartree
Gibbs free energy (G) = -555.649345 Hartree
Charge = 0, Spin = 2
C -0.297943 -0.000001 -0.007660
C -0.630186 0.002210 1.486001
H -0.230935 -0.884201 1.979600
H -0.231228 0.890264 1.976896
H -1.717192 0.002224 1.601984
C -0.858176 -1.255282 -0.665974
H -1.948712 -1.258134 -0.588548
H -0.592207 -1.292966 -1.722855
H -0.468263 -2.150192 -0.182077
C -0.858323 1.253201 -0.669722
H -0.467939 2.149567 -0.188910
H -0.592984 1.287331 -1.726870
H -1.948817 1.256619 -0.591703
S 1.504128 -0.000080 -0.088337

IBA_Thiazole_02.log
Energy (E) = -568.119787405 Hartree
Enthalpy (H) = -568.071788 Hartree
Gibbs free energy (G) = -568.104320 Hartree
Charge = 0, Spin = 2
C 0.064960 1.164772 0.000033
C 1.277729 0.560402 -0.000027
C 0.034645 -1.229629 0.000022
S -1.180929 -0.029382 -0.000014
H -0.158116 2.217999 0.000053
H 2.233267 1.059611 -0.000055
N 1.222244 -0.825823 0.000007

IBA_Thiophene3_02.log
Energy (E) = -552.075493624 Hartree
Enthalpy (H) = -552.015978 Hartree
Gibbs free energy (G) = -552.048706 Hartree
Charge = 0, Spin = 2

C 0.438225 -1.114673 0.000486
C 1.479329 -0.229957 -0.000073
C 0.974480 1.084297 -0.000521
C -0.359098 1.236667 0.000784
S -1.077495 -0.330261 -0.000302
H 0.485861 -2.191171 0.000862
H 2.520679 -0.508724 -0.000463
H -0.964234 2.126057 0.000365

IBA_5FBz.log
Energy (E) = -1346.43948824 Hartree
Enthalpy (H) = -1346.259126 Hartree
Gibbs free energy (G) = -1346.333918 Hartree
Charge = 0, Spin = 1
C -4.974969 0.725412 0.047189
C -3.907996 1.520955 -0.339330
C -2.636990 0.975902 -0.405172
C -2.401168 -0.362558 -0.104006
C -3.490956 -1.141838 0.276780
C -4.766999 -0.608198 0.360578
F -1.656377 1.777604 -0.800491
F -3.338997 -2.410625 0.608192
F -5.785272 -1.361096 0.743378
F -6.188213 1.239075 0.118412
F -4.110381 2.792518 -0.647979
C -1.036852 -0.973505 -0.199669
O -0.074614 -0.161100 0.138964
O -0.859175 -2.122747 -0.553018
I 1.834687 -1.150492 -0.126562
C 2.632522 0.772340 0.177909
O 3.892057 -1.575990 -0.241563
C 4.010004 0.738035 0.170350
C 1.896761 1.926549 0.352942
C 4.719241 -0.559721 -0.056045
C 4.707724 1.925168 0.355665
C 2.615042 3.103948 0.536018
H 0.818619 1.919805 0.343419
O 5.919014 -0.654858 -0.071633
C 4.007036 3.105761 0.540661
H 5.788567 1.883553 0.345668
H 2.073527 4.029373 0.674669
H 4.541417 4.034131 0.685210

IBA_5FPh.log
Energy (E) = -1157.98067047 Hartree
Enthalpy (H) = -1157.817748 Hartree
Gibbs free energy (G) = -1157.885558 Hartree
Charge = 0, Spin = 1
C -3.039223 0.870458 0.983778
C -3.824195 0.397694 -0.059092
C -3.293369 -0.477159 -0.995000
C -1.970234 -0.874836 -0.875850
C -1.169328 -0.419929 0.156081
C -1.722132 0.452884 1.077465
F -1.488154 -1.713344 -1.786688
F -4.049217 -0.920609 -1.985251
F -5.081176 0.784761 -0.160756
F -3.553042 1.710229 1.866691
F -0.980260 0.931931 2.070839
I 0.849538 -1.158385 0.407331
C 1.691388 0.663927 -0.298874
O 3.064330 -1.541136 0.539921
C 3.066286 0.652285 -0.282128
C 0.934063 1.736422 -0.718958
C 3.835399 -0.565907 0.182671
C 3.729887 1.794910 -0.716141
C 1.618409 2.867860 -1.149208
H -0.145608 1.712559 -0.727927
O 5.047595 -0.548598 0.186736
C 3.009180 2.897522 -1.146066
H 4.811351 1.774339 -0.699875
H 1.055948 3.727117 -1.487098
H 3.527223 3.784745 -1.482468

IBA_Acr.log
Energy (E) = -985.031566029 Hartree
Enthalpy (H) = -984.745856 Hartree
Gibbs free energy (G) = -984.814763 Hartree
Charge = 0, Spin = 1
C 3.624696 -3.158753 -1.082806
C 2.518976 -2.367939 -1.042548
C 2.596505 -1.048931 -0.509417
C 3.856349 -0.579344 -0.017072
C 4.991126 -1.438265 -0.077426
C 4.878373 -2.690037 -0.595209
C 3.907819 0.706819 0.506022
C 2.762512 1.495330 0.538603
C 1.555531 0.933597 0.011970
C 0.372175 1.736294 0.049026
C 0.395443 2.990948 0.575505
C 1.600043 3.542447 1.097290
C 2.747744 2.814332 1.075282
H 3.558815 -4.158813 -1.489356
H 1.557262 -2.701938 -1.405974
H 5.938655 -1.071374 0.297199
H 5.740954 -3.340481 -0.640335
H 4.842764 1.097296 0.893476
H -0.509703 3.584944 0.601413
H 1.589187 4.541957 1.508649
H 3.671485 3.220950 1.467950
N 1.490410 -0.299087 -0.486730
I -1.411415 1.007987 -0.877785
C -1.848705 -0.361843 0.688365
O -3.423547 0.008286 -1.530457
C -2.997614 -1.091920 0.491549
C -1.035212 -0.489196 1.795446
C -3.869692 -0.896209 -0.738841
C -3.351635 -2.014001 1.471776
C -1.410436 -1.414006 2.762585
H -0.142471 0.105932 1.919822
O -4.871659 -1.577971 -0.860711
C -2.563274 -2.175857 2.599589
H -4.257848 -2.581229 1.306257
H -0.795444 -1.536647 3.643627
H -2.844217 -2.893591 3.358180

IBA_Adm.log
Energy (E) = -820.325252302 Hartree
Enthalpy (H) = -819.981317 Hartree
Gibbs free energy (G) = -820.044216 Hartree
Charge = 0, Spin = 1
C -1.753784 0.621910 -1.265767
H -1.549042 -0.001567 -2.140999
H -1.122342 1.507801 -1.342496
C -1.466634 -0.168806 0.000002
C -3.229614 1.039926 -1.251951
H -3.436540 1.633471 -2.144101
C -4.106631 -0.210753 -1.246638
H -3.917815 -0.802847 -2.146371
H -5.162319 0.073058 -1.254177
C -2.323105 -1.434298 0.000435
H -2.103485 -2.039019 0.886887
H -2.103825 -2.039453 -0.885811
C -3.804218 -1.038286 0.000615
H -4.410181 -1.945871 0.000903
C -4.106157 -0.210234 1.247639
H -5.161836 0.073598 1.255457
H -3.917013 -0.801951 2.147550
C -1.753279 0.622435 1.265519
H -1.121809 1.508360 1.341656
H -1.548227 -0.000670 2.140948
C -3.509200 1.871652 -0.000053
H -2.882094 2.768664 -0.000353
H -4.551378 2.201376 0.000085
C -3.229130 1.040440 1.252076
H -3.435678 1.634377 2.144055
I 0.558464 -1.076421 -0.000354
C 1.820087 0.651854 -0.000248
O 2.796690 -1.937049 -0.000274
C 3.169273 0.373152 0.000268
C 1.315155 1.939935 -0.000665
C 3.715025 -1.052026 0.000386
C 4.053188 1.448343 0.000483
C 2.217176 2.997001 -0.000492
H 0.256902 2.141576 -0.001168
O 4.925329 -1.203069 0.001029
C 3.585325 2.751888 0.000108
H 5.107538 1.205209 0.000921
H 1.841778 4.011225 -0.000853
H 4.281730 3.579146 0.000252

IBA_Allene.log
Energy (E) = -546.515167947 Hartree
Enthalpy (H) = -546.362089 Hartree
Gibbs free energy (G) = -546.415943 Hartree
Charge = 0, Spin = 1
C 3.284528 0.864127 0.228155
C 2.408536 0.555004 -0.675381
H 2.448870 0.913189 -1.694493
C 4.151791 1.153720 1.155432
H 5.033130 0.544016 1.303175
H 4.013246 2.012747 1.798349
I 0.854092 -0.864755 -0.218857
C -0.643182 0.642903 -0.112177
O -1.084251 -2.035230 0.231241
C -1.907872 0.152814 0.114722
C -0.350943 1.982195 -0.267012
C -2.159244 -1.332861 0.300437
C -2.952066 1.068723 0.188686
C -1.410514 2.879461 -0.190724
H 0.658405 2.331156 -0.430594
O -3.294924 -1.721063 0.494826
C -2.705591 2.423881 0.034144
H -3.942123 0.672451 0.370879
H -1.216904 3.937042 -0.306116
H -3.520735 3.131956 0.091968

IBA_Anth2.log
Energy (E) = -968.994484106 Hartree
Enthalpy (H) = -968.696996 Hartree
Gibbs free energy (G) = -968.766824 Hartree
Charge = 0, Spin = 1
C 7.266338 0.617177 -0.214833
C 6.328467 0.594682 0.769454
C 4.971190 0.272488 0.476257
C 4.612413 -0.027998 -0.876564
C 5.625021 0.006381 -1.879437
C 6.909277 0.318434 -1.560295
C 3.990146 0.242605 1.465479
C 3.289232 -0.344604 -1.175295
C 2.310939 -0.374338 -0.185332
C 2.667914 -0.073098 1.167365
C 1.653466 -0.107287 2.169763
H 1.930643 0.120099 3.191719

C 0.367917 -0.421186 1.857505
C 0.019230 -0.714536 0.508100
C 0.952132 -0.692989 -0.480422
H 4.263411 0.470025 2.489935
H 8.293347 0.862824 0.018649
H 6.594095 0.820761 1.794692
H 5.346288 -0.222085 -2.900649
H 7.670246 0.341723 -2.328331
H 3.015997 -0.572544 -2.199640
H -0.394482 -0.446029 2.625472
H 0.685765 -0.911340 -1.508413
I -1.990328 -1.266014 0.044407
C -2.649111 0.762376 -0.030175
O -4.277268 -1.411138 -0.446422
C -3.992868 0.907087 -0.284108
C -1.780419 1.816752 0.166412
C -4.900755 -0.293037 -0.510659
C -4.500253 2.201398 -0.342132
C -2.311493 3.100382 0.103306
H -0.727466 1.665580 0.356579
O -6.082961 -0.097551 -0.726456
C -3.666224 3.291163 -0.148148
H -5.558059 2.304105 -0.544483
H -1.658665 3.949673 0.251222
H -4.067265 4.294357 -0.194762

IBA_Bn.log
Energy (E) = -701.275076601 Hartree
Enthalpy (H) = -701.046837 Hartree
Gibbs free energy (G) = -701.108323 Hartree
Charge = 0, Spin = 1
C 4.678051 -0.052291 1.202088
C 5.322443 -0.315782 -0.000354
C 4.677652 -0.052647 -1.202612
C 3.394677 0.474284 -1.202100
C 2.744625 0.752750 -0.000018
C 3.395120 0.474631 1.201932
H 5.175453 -0.257176 2.140144
H 6.322844 -0.725970 -0.000410
H 5.174655 -0.257726 -2.140834
H 2.889251 0.679234 -2.138645
H 2.890016 0.679872 2.138584
C 1.354756 1.281602 0.000086
H 1.115835 1.847016 0.896255
H 1.115603 1.846711 -0.896223
I 0.058977 -0.500135 0.000194
C -1.753817 0.608097 0.000120
O -1.620072 -2.129981 0.000329
C -2.892391 -0.164763 -0.000144
C -1.770286 1.990451 0.000181
C -2.822078 -1.684008 -0.000047
C -4.120964 0.487168 -0.000375
C -3.009267 2.621335 -0.000033
H -0.866201 2.580357 0.000468
O -3.861618 -2.317616 -0.000184
C -4.180646 1.871856 -0.000339
H -5.005927 -0.135202 -0.000608
H -3.052172 3.701826 0.000053
H -5.138840 2.372907 -0.000530

IBA_Carbazole.log
Energy (E) = -946.970661947 Hartree
Enthalpy (H) = -946.691412 Hartree
Gibbs free energy (G) = -946.758515 Hartree
Charge = 0, Spin = 1
C 3.540403 3.021931 0.516558
C 2.328496 3.408340 -0.067038
C 1.420977 2.471781 -0.533245
C 1.752759 1.126834 -0.402750
C 2.962299 0.723054 0.193970
C 3.863070 1.683160 0.650658
H 4.227106 3.779997 0.866836
H 2.094709 4.460425 -0.157228
H 0.487768 2.781929 -0.985431
H 4.798948 1.383879 1.105391
C 1.752904 -1.127010 -0.402098
C 1.421344 -2.472083 -0.531866
C 2.329016 -3.408242 -0.065150
C 3.540866 -3.021328 0.518223
C 3.863330 -1.682432 0.651571
C 2.962403 -0.722718 0.194365
H 0.488198 -2.782640 -0.983902
H 2.095398 -4.460413 -0.154774
H 4.227684 -3.779093 0.868925
H 4.799163 -1.382756 1.106135
N 1.033295 -0.000246 -0.795104
I -0.986753 -0.000534 -1.314107
C -1.539972 0.000203 0.730339
O -3.170421 -0.000607 -1.425722
C -2.904049 0.000269 0.903871
C -0.625378 0.000573 1.761579
C -3.818810 -0.000185 -0.292549
C -3.398075 0.000750 2.203045
C -1.141826 0.001033 3.053559
H 0.441627 0.000502 1.590793
O -5.020732 -0.000111 -0.159966
C -2.516357 0.001124 3.272584
H -4.472046 0.000823 2.332221
H -0.458703 0.001314 3.891409
H -2.896191 0.001493 4.284778

IBA_CCPh.log
Energy (E) = -738.077693884 Hartree
Enthalpy (H) = -737.866610 Hartree
Gibbs free energy (G) = -737.930408 Hartree
Charge = 0, Spin = 1
C -1.050217 -0.633633 0.000083
C -2.231958 -0.379373 0.000075
C -3.626893 -0.066707 0.000053
C -4.314997 0.085529 -1.207255
C -4.314199 0.089187 1.207350
C -5.666851 0.388373 -1.203861
H -3.779362 -0.037226 -2.138449
C -5.666049 0.392044 1.203937
H -3.777951 -0.030770 2.138556
C -6.344054 0.542083 0.000032
H -6.193468 0.504233 -2.141084
H -6.192041 0.510763 2.141154
H -7.399411 0.778108 0.000023
I 0.922512 -1.278111 0.000252
C 1.652945 0.720472 -0.000256
O 3.166136 -1.547745 0.000392
C 3.025958 0.788356 -0.000162
C 0.817383 1.815520 -0.000623
C 3.873478 -0.467201 0.000198
C 3.607408 2.051846 -0.000447
C 1.422028 3.068000 -0.000906
H -0.258190 1.708862 -0.000700
O 5.083600 -0.375768 0.000270
C 2.808766 3.184756 -0.000811
H 4.688133 2.099497 -0.000370
H 0.801228 3.953283 -0.001207
H 3.263519 4.165731 -0.001031

IBA_Cy.log
Energy (E) = -665.613779218 Hartree
Enthalpy (H) = -665.344299 Hartree
Gibbs free energy (G) = -665.403798 Hartree
Charge = 0, Spin = 1
C -2.218210 0.846138 -1.003425
C -3.548776 1.579911 -0.849245
C -4.655841 0.600106 -0.478736
C -4.303856 -0.156903 0.796275
C -2.966975 -0.883340 0.650521
C -1.882409 0.115944 0.282250
H -5.605100 1.122835 -0.358634
H -3.454654 2.335294 -0.061739
H -3.791943 2.108832 -1.771168
H -2.305314 0.108879 -1.808960
H -1.421054 1.533969 -1.289917
H -4.231617 0.550887 1.628258
H -5.087226 -0.870910 1.051879
H -2.714954 -1.409299 1.573110
H -3.053205 -1.635097 -0.143351
H -1.726416 0.804108 1.114580
H -4.786697 -0.115931 -1.296739
I -0.037128 -1.040070 0.072740
C 1.272347 0.641206 0.151890
O 2.144064 -1.951477 -0.221755
C 2.600530 0.338689 -0.043857
C 0.809476 1.922015 0.392867
C 3.085998 -1.090265 -0.267522
C 3.514837 1.387321 -0.007563
C 1.741852 2.952307 0.427604
H -0.234827 2.139244 0.557654
O 4.277284 -1.269899 -0.453644
C 3.091185 2.685926 0.223752
H 4.554301 1.130893 -0.163333
H 1.405556 3.962651 0.616052
H 3.809183 3.494009 0.251420

IBA_Dibenzofuran.log
Energy (E) = -966.833780138 Hartree
Enthalpy (H) = -966.566651 Hartree
Gibbs free energy (G) = -966.634423 Hartree
Charge = 0, Spin = 1
C -0.795919 -0.665907 1.573983
C -0.285068 -0.779000 0.278134
C -1.082310 -0.610615 -0.845762
C -2.414659 -0.322115 -0.614440
C -2.957067 -0.198831 0.670378
C -2.135383 -0.372794 1.780123
H -0.138602 -0.803964 2.422457
H -0.693635 -0.694740 -1.851482
H -2.529912 -0.284137 2.783536
C -4.527630 0.132676 -0.921256
C -5.741802 0.391724 -1.529301
C -6.817382 0.627220 -0.685862
C -6.676120 0.602779 0.706244
C -5.450562 0.341195 1.294527
C -4.357283 0.102184 0.464985
H -5.838580 0.407470 -2.604900
H -7.788228 0.834265 -1.114599
H -7.539964 0.791246 1.328193
H -5.345089 0.322634 2.371130
O -3.354867 -0.123544 -1.577323
I 1.775376 -1.279141 0.012819
C 2.390967 0.764628 0.044265
O 4.094776 -1.367919 -0.259870
C 3.746802 0.941712 -0.101358
C 1.487680 1.796381 0.199251
C 4.696019 -0.235123 -0.269326
C 4.229630 2.246647 -0.091049
C 1.994646 3.091284 0.205949
H 0.427503 1.619441 0.311205

O 5.886403 -0.013056 -0.392461
C 3.359950 3.314845 0.061469
H 5.297654 2.374772 -0.206561
H 1.314716 3.923710 0.324757
H 3.741658 4.326577 0.068327

IBA_Furane3.log
Energy (E) = -659.810377632 Hartree
Enthalpy (H) = -659.642348 Hartree
Gibbs free energy (G) = -659.697729 Hartree
Charge = 0, Spin = 1
C -4.012931 0.907804 0.640871
C -2.864686 0.402534 1.145690
C -2.144305 -0.118316 0.022206
C -2.919422 0.113574 -1.065396
O -4.057200 0.736374 -0.702467
H -4.861603 1.394703 1.085707
H -2.560707 0.395571 2.178034
H -2.794122 -0.098630 -2.112408
I -0.294592 -1.146212 0.016272
C 0.836248 0.662019 -0.013717
O 1.912774 -1.858581 0.008866
C 2.196845 0.464475 -0.005813
C 0.233752 1.902801 -0.032263
C 2.800478 -0.930586 0.008420
C 3.011096 1.592519 -0.020045
C 1.068360 3.015065 -0.045653
H -0.840722 2.017739 -0.039327
O 4.011174 -1.047101 0.016199
C 2.450827 2.859976 -0.038974
H 4.080110 1.426435 -0.016101
H 0.630266 4.003560 -0.061376
H 3.088584 3.733137 -0.049716

IBA_Mesitylene.log
Energy (E) = -779.831964618 Hartree
Enthalpy (H) = -779.545782 Hartree
Gibbs free energy (G) = -779.614734 Hartree
Charge = 0, Spin = 1
C -3.469527 0.280824 -1.194649
C -4.143907 0.507398 -0.000161
C -3.470081 0.279548 1.194363
C -2.158610 -0.185066 1.220509
C -1.523635 -0.426348 -0.000109
C -2.158203 -0.184121 -1.220771
H -3.973134 0.478058 -2.134439
H -3.974090 0.475768 2.134149
C -1.462729 -0.368005 2.540586
H -0.557145 0.240666 2.593530
H -2.116330 -0.073547 3.358928
H -1.163039 -1.403942 2.701654
C -1.462045 -0.366240 -2.540796
H -0.556483 0.242457 -2.593121
H -1.162330 -1.402050 -2.702492
H -2.115498 -0.071340 -3.359120
C -5.570301 0.975463 -0.000194
H -5.790325 1.571509 -0.884656
H -6.252641 0.123571 0.000084
H -5.790187 1.571962 0.883998
I 0.466819 -1.220479 -0.000051
C 1.358546 0.717182 0.000296
O 2.798041 -1.629425 -0.000488
C 2.733932 0.711931 0.000130
C 0.588139 1.863304 0.000678
C 3.535891 -0.583716 -0.000287
C 3.379776 1.944494 0.000317
C 1.257554 3.082455 0.000893
H -0.492776 1.825274 0.000890

O 4.752166 -0.510969 -0.000503
C 2.647939 3.121748 0.000698
H 4.461579 1.929630 0.000155
H 0.685127 3.999937 0.001250
H 3.157757 4.075473 0.000803

IBA_Naph1.log
Energy (E) = -815.499406585 Hartree
Enthalpy (H) = -815.251085 Hartree
Gibbs free energy (G) = -815.314042 Hartree
Charge = 0, Spin = 1
C 2.692134 1.660551 2.040424
C 1.816777 0.813646 1.417043
C 2.218690 0.047921 0.296373
C 3.552161 0.190274 -0.177201
C 4.434277 1.071041 0.491773
C 4.017809 1.790490 1.576809
H 2.364873 2.236812 2.894791
H 0.801972 0.729833 1.782863
C 1.356631 -0.839257 -0.404246
C 3.973757 -0.544224 -1.311524
H 5.447721 1.163798 0.121312
H 4.699171 2.462084 2.080577
C 3.117451 -1.383567 -1.965054
C 1.791295 -1.530252 -1.503207
H 4.994253 -0.422149 -1.652887
H 3.443964 -1.938106 -2.833655
H 1.117851 -2.196859 -2.027035
I -0.627002 -1.279652 0.280327
C -1.432021 0.571954 -0.404508
O -2.901946 -1.392603 0.830549
C -2.773495 0.731414 -0.146678
C -0.649931 1.510835 -1.046274
C -3.591446 -0.350421 0.543133
C -3.370035 1.919551 -0.557272
C -1.269148 2.689186 -1.447860
H 0.399905 1.345479 -1.241272
O -4.773627 -0.145772 0.749585
C -2.623037 2.893087 -1.201517
H -4.425886 2.034496 -0.351344
H -0.686227 3.445814 -1.954788
H -3.093133 3.814008 -1.518278

IBA_Naph2.log
Energy (E) = -815.499005041 Hartree
Enthalpy (H) = -815.250837 Hartree
Gibbs free energy (G) = -815.314502 Hartree
Charge = 0, Spin = 1
C -5.870040 0.684882 -0.199274
C -5.038430 0.580117 0.881487
C -3.691442 0.180254 0.717314
C -3.216082 -0.111771 -0.586620
C -4.098111 0.005348 -1.686706
C -5.395153 0.394646 -1.497711
H -3.176426 0.285371 2.808952
H -6.898343 0.991153 -0.064153
H -5.397219 0.800820 1.879084
C -2.805796 0.062225 1.816075
C -1.866988 -0.509589 -0.751675
H -3.726020 -0.219285 -2.678524
H -6.064362 0.482008 -2.342591
C -1.040049 -0.612985 0.331904
C -1.508090 -0.328511 1.634253
H -1.507141 -0.723998 -1.751664
H -0.838187 -0.416426 2.479899
I 0.972870 -1.272979 0.059384
C 1.738096 0.719119 0.011701
O 3.281707 -1.540982 -0.229301

C 3.103380 0.791483 -0.135770
C 0.912022 1.819077 0.123731
C 3.964152 -0.456499 -0.266802
C 3.679719 2.057494 -0.171436
C 1.511814 3.073024 0.085134
H -0.158828 1.724493 0.233343
O 5.167890 -0.324555 -0.392290
C 2.890122 3.191078 -0.060593
H 4.754189 2.103274 -0.289152
H 0.894018 3.956554 0.169224
H 3.344663 4.171877 -0.089099

IBA_OCH2CF3.log
Energy (E) = -882.512374030 Hartree
Enthalpy (H) = -882.355341 Hartree
Gibbs free energy (G) = -882.414768 Hartree
Charge = 0, Spin = 1
O -1.540908 -0.665975 0.514479
C -2.249092 -0.348739 -0.643428
H -2.535313 -1.228680 -1.228484
H -1.712512 0.348154 -1.300463
C -3.531572 0.346305 -0.234238
F -4.293713 -0.423920 0.535936
F -3.280716 1.474899 0.439956
F -4.237452 0.672362 -1.322566
I 0.384915 -1.327920 0.186303
C 1.038432 0.671507 0.162147
O 2.478506 -1.574288 -0.194720
C 2.381429 0.770913 -0.117719
C 0.216163 1.747386 0.416494
C 3.188965 -0.476217 -0.330211
C 2.950070 2.037634 -0.168938
C 0.805142 3.007033 0.360381
H -0.826214 1.617514 0.667854
O 4.366828 -0.439693 -0.588184
C 2.158231 3.151365 0.065468
H 4.006899 2.109777 -0.387921
H 0.197306 3.879653 0.554742
H 2.594280 4.139798 0.027955

IBA_OEt.log
Energy (E) = -584.880527363 Hartree
Enthalpy (H) = -584.702766 Hartree
Gibbs free energy (G) = -584.756669 Hartree
Charge = 0, Spin = 1
O -2.286248 0.030986 0.440888
C -2.856333 0.491649 -0.771672
H -2.099317 0.997450 -1.386548
H -3.243156 -0.348289 -1.359163
C -3.971418 1.451169 -0.422792
H -3.577728 2.299751 0.137192
H -4.455727 1.823679 -1.325334
H -4.715819 0.951334 0.194757
I -0.611664 -1.101964 0.184095
C 0.546828 0.652335 0.139816
O 1.390585 -1.889389 -0.129776
C 1.878839 0.403898 -0.094400
C 0.018964 1.909560 0.342295
C 2.353582 -1.013901 -0.258099
C 2.749913 1.485342 -0.149236
C 0.906229 2.979153 0.284980
H -1.031582 2.046040 0.556034
O 3.515171 -1.268270 -0.476326
C 2.260262 2.768993 0.036407
H 3.796322 1.281955 -0.332967
H 0.534422 3.982423 0.440817
H 2.933532 3.613928 -0.003883

IBA_Pyrene.log
Energy (E) = -1045.17119894 Hartree
Enthalpy (H) = -1044.859573 Hartree
Gibbs free energy (G) = -1044.929758 Hartree
Charge = 0, Spin = 1
C -1.200398 2.970728 -1.384166
C -0.498464 1.795172 -1.158407
C -1.139316 0.663064 -0.652620
C -2.527477 0.737110 -0.355532
C -3.236241 1.940990 -0.592659
C -2.555636 3.044525 -1.108679
C -0.463624 -0.582769 -0.389032
C -3.213923 -0.385939 0.182863
C -2.517141 -1.590796 0.434912
C -1.117659 -1.647143 0.130482
C -3.198047 -2.686101 0.966735
H -2.654578 -3.603252 1.156137
C -4.553613 -2.600267 1.247623
C -5.245607 -1.422995 1.003429
C -4.597460 -0.305742 0.473390
C -5.288660 0.923862 0.214917
C -4.638465 1.993710 -0.292277
H -5.163918 2.921029 -0.483862
H -6.346995 0.973263 0.438698
H -0.681329 3.833688 -1.777686
H 0.560520 1.760794 -1.377210
H -3.102343 3.961886 -1.287539
H -0.594390 -2.576253 0.327727
H -5.073483 -3.454559 1.658915
H -6.303899 -1.357271 1.223578
I 1.583741 -0.898094 -0.940862
C 2.348129 0.245979 0.686534
O 3.909961 -1.035263 -1.173725
C 3.720442 0.329356 0.719802
C 1.513389 0.840384 1.611202
C 4.590573 -0.371982 -0.312441
C 4.294859 1.066478 1.750757
C 2.111051 1.570520 2.632351
H 0.438519 0.743103 1.558989
O 5.799664 -0.253698 -0.234513
C 3.495943 1.685116 2.699336
H 5.375011 1.124263 1.769466
H 1.486952 2.048293 3.374960
H 3.948721 2.256121 3.498320

IBA_SeMes.log
Energy (E) = -789.159517421 Hartree
Enthalpy (H) = -788.870364 Hartree
Gibbs free energy (G) = -788.945919 Hartree
Charge = 0, Spin = 1
C -2.410158 -0.659884 -1.167119
C -3.299673 0.162720 -1.853374
C -3.986178 1.194270 -1.229847
C -3.789147 1.376723 0.134503
C -2.910567 0.587039 0.865072
C -2.192717 -0.419305 0.195772
H -3.466372 -0.022618 -2.908945
H -4.346591 2.149821 0.652034
C -2.810048 0.803489 2.349996
H -3.143824 -0.077797 2.899372
H -1.787381 0.994834 2.677080
H -3.432302 1.645148 2.649317
C -1.743015 -1.772262 -1.922903
H -0.761131 -1.473803 -2.293221
H -1.604708 -2.652349 -1.295365
H -2.344128 -2.049379 -2.787075
C -4.914220 2.087282 -2.001016
H -5.270541 1.598152 -2.906394
H -5.776978 2.373088 -1.400208
H -4.405835 3.005746 -2.300316
Se -1.007804 -1.506834 1.253454
I 1.389426 -1.247486 -0.076431
C 1.666599 0.825600 0.314830
O 3.381349 -0.818643 -1.021472
C 2.847945 1.310891 -0.194775
C 0.743817 1.594445 0.987194
C 3.801791 0.406416 -0.931778
C 3.131456 2.659103 -0.009138
C 1.044726 2.940971 1.160273
H -0.173235 1.168690 1.366701
O 4.843142 0.838705 -1.376414
C 2.232927 3.470166 0.665824
H 4.065967 3.029206 -0.409615
H 0.342229 3.574956 1.683488
H 2.453443 4.518917 0.808562

IBA_SetBu.log
Energy (E) = -597.596798202 Hartree
Enthalpy (H) = -597.363400 Hartree
Gibbs free energy (G) = -597.426491 Hartree
Charge = 0, Spin = 1
Se -2.111823 0.079867 0.970952
C -2.853076 0.420028 -0.861122
C -1.952046 1.362703 -1.635810
H -0.965420 0.928422 -1.809278
H -2.399611 1.566416 -2.612809
H -1.825958 2.310495 -1.112041
C -3.049471 -0.886124 -1.607411
H -3.676671 -1.573513 -1.040630
H -3.535626 -0.682434 -2.565872
H -2.097596 -1.374346 -1.820268
C -4.193642 1.074897 -0.566272
H -4.067803 2.004576 -0.010240
H -4.692963 1.307039 -1.510974
H -4.841894 0.410144 0.005697
I 0.206145 -1.137246 0.279209
C 1.383737 0.640480 0.187453
O 2.241088 -1.899966 -0.324089
C 2.685993 0.397339 -0.181067
C 0.888473 1.889704 0.487708
C 3.165033 -1.003712 -0.472857
C 3.549899 1.482923 -0.271934
C 1.769838 2.961567 0.393922
H -0.140734 2.031333 0.790144
O 4.314088 -1.200718 -0.806215
C 3.092928 2.759630 0.013319
H 4.571052 1.279450 -0.566007
H 1.415846 3.957054 0.623965
H 3.766260 3.602769 -0.055259

IBA_SMes.log
Energy (E) = -1177.94172584 Hartree
Enthalpy (H) = -1177.651962 Hartree
Gibbs free energy (G) = -1177.726614 Hartree
Charge = 0, Spin = 1
C -4.133375 -0.201743 -1.195222
C -4.759978 -0.539295 -0.000258
C -4.133549 -0.202035 1.194882
C -2.915335 0.465394 1.221363
C -2.310458 0.804279 0.000086
C -2.915156 0.465692 -1.221359
H -4.604410 -0.467764 -2.135005
H -4.604730 -0.468292 2.134523
C -2.274112 0.809496 -2.533938
H -2.144253 1.886825 -2.637175
H -1.278729 0.369097 -2.616833
H -2.881590 0.447518 -3.361241
C -6.091676 -1.232364 -0.000446
H -6.904239 -0.503321 -0.001043
H -6.211315 -1.857181 -0.884450
H -6.211931 -1.856462 0.883978
C -2.274495 0.808905 2.534117
H -2.144675 1.886212 2.637625
H -2.882092 0.446721 3.361244
H -1.279116 0.368507 2.617061
S -0.775918 1.672786 0.000306
I 0.519666 -0.491377 0.000137
C 2.401444 0.473976 0.000003
O 1.953309 -2.209262 -0.000058
C 3.457815 -0.407848 -0.000103
C 2.546338 1.846062 0.000037
C 3.214299 -1.896300 -0.000139
C 4.746278 0.113427 -0.000179
C 3.843724 2.346928 -0.000038
H 1.697960 2.514878 0.000108
O 4.144991 -2.672493 -0.000246
C 4.937085 1.486264 -0.000144
H 5.568404 -0.589696 -0.000262
H 3.994716 3.417499 -0.000016
H 5.938851 1.892854 -0.000200

IBA_SO3Ph.log
Energy (E) = -1285.61881762 Hartree
Enthalpy (H) = -1285.400350 Hartree
Gibbs free energy (G) = -1285.469055 Hartree
Charge = 0, Spin = 1
C 5.866434 0.312535 -0.991021
C 5.162352 1.478362 -0.715282
C 3.902431 1.407593 -0.137942
C 3.369760 0.160521 0.151005
C 4.063334 -1.012505 -0.110121
C 5.321526 -0.930167 -0.687072
H 6.849497 0.372314 -1.438028
H 5.596732 2.441660 -0.943715
H 3.338119 2.296591 0.105745
H 3.622938 -1.963334 0.155911
H 5.879878 -1.832721 -0.893079
S 1.771230 0.058316 0.878690
O 1.682419 -1.146427 1.666220
O 1.462666 1.321584 1.499799
O 0.880702 -0.115675 -0.385870
I -1.011028 -1.152447 -0.175192
C -1.970098 0.720400 -0.158095
O -3.003096 -1.768950 -0.134513
C -3.337958 0.562624 -0.162278
C -1.327265 1.936565 -0.134565
C -3.923874 -0.810820 -0.155999
C -4.133227 1.701913 -0.154338
C -2.143548 3.063753 -0.125075
H -0.251542 2.013463 -0.102412
O -5.106766 -1.025541 -0.164935
C -3.531150 2.949807 -0.138760
H -5.207040 1.571964 -0.156016
H -1.682438 4.041147 -0.100484
H -4.141043 3.842146 -0.130147

IBA_StBu.log
Energy (E) = -986.376708101 Hartree
Enthalpy (H) = -986.142711 Hartree
Gibbs free energy (G) = -986.203942 Hartree
Charge = 0, Spin = 1
C 2.992462 0.454419 0.538380
C 4.266196 1.173357 0.102468
H 4.863695 0.545111 -0.558792

H 4.034081 2.104640 -0.415135
H 4.862251 1.408564 0.986834
C 3.347659 -0.848329 1.238513
H 3.955078 -0.631238 2.120168
H 2.455936 -1.378151 1.575678
H 3.914982 -1.502447 0.576702
C 2.170630 1.351941 1.449431
H 1.951027 2.304688 0.967472
H 1.227055 0.882758 1.734573
H 2.732905 1.550021 2.364837
S 2.124524 0.134256 -1.049519
I 0.101416 -1.088752 -0.374228
C -1.149222 0.634500 -0.224379
O -1.896508 -1.938985 0.271416
C -2.427957 0.339558 0.187099
C -0.711164 1.902470 -0.535147
C -2.845310 -1.083906 0.477193
C -3.326807 1.392814 0.315483
C -1.627363 2.940676 -0.403787
H 0.299672 2.081078 -0.874652
O -3.973231 -1.321320 0.855561
C -2.927311 2.687255 0.023080
H -4.328997 1.150540 0.643325
H -1.319530 3.949668 -0.641299
H -3.628562 3.504415 0.120837

IBA_Thiazole.log
Energy (E) = -998.772878930 Hartree
Enthalpy (H) = -998.619848 Hartree
Gibbs free energy (G) = -998.676394
Hartree
Charge = 0, Spin = 1
C -4.110956 0.847896 0.262066
C -3.773942 0.720959 -1.050708
C -1.985524 -0.199122 -0.176499
S -2.875561 0.197503 1.236122
H -5.004379 1.275405 0.683253
H -4.379502 1.047384 -1.881768
N -2.564055 0.124906 -1.289093
I -0.083643 -1.190920 -0.090629
C 0.957588 0.668370 -0.052027
O 2.117831 -1.811227 0.018288
C 2.321558 0.522063 0.034451
C 0.311027 1.885152 -0.116304
C 2.971272 -0.848467 0.081327
C 3.093781 1.678673 0.070234
C 1.104151 3.026644 -0.080949
H -0.762812 1.965247 -0.202526
O 4.180149 -0.930080 0.167271
C 2.488292 2.923738 0.014471
H 4.165991 1.552988 0.139814
H 0.631907 3.997965 -0.131554
H 3.091922 3.820386 0.040965

IBA_Thiophene3.log
Energy (E) = -982.738508781 Hartree
Enthalpy (H) = -982.573596 Hartree
Gibbs free energy (G) = -982.630320
Hartree
Charge = 0, Spin = 1
C -3.788025 0.627622 1.044276
C -2.558645 0.095626 1.287961
C -1.909420 -0.308496 0.088504
C -2.660578 -0.077799 -1.024578
S -4.153854 0.633346 -0.621118
H -4.497336 1.011367 1.757947
H -2.128744 -0.004397 2.273344
H -2.407266 -0.282088 -2.051679
I 0.013318 -1.216202 0.021569
C 1.011188 0.669999 -0.000108
O 2.272958 -1.765431 -0.043050
C 2.382436 0.572765 -0.022930
C 0.319100 1.863726 0.011488
C 3.088085 -0.774015 -0.044035
C 3.111384 1.757891 -0.034532
C 1.069769 3.034217 -0.000256
H -0.761016 1.900926 0.025815
O 4.304352 -0.798650 -0.061191
C 2.459733 2.980924 -0.022286
H 4.189533 1.670582 -0.054000
H 0.560384 3.988034 0.007498
H 3.031606 3.898617 -0.031322

PhIF_5FBz.log
Energy (E) = -1258.38507939 Hartree
Enthalpy (H) = -1258.206422 Hartree
Gibbs free energy (G) = -1258.280286
Hartree
Charge = 0, Spin = 1
C -4.605217 0.774480 0.005780
C -3.567272 1.486576 -0.573958
C -2.302073 0.925739 -0.632745
C -2.049833 -0.347119 -0.133876
C -3.109209 -1.045740 0.435470
C -4.377169 -0.493695 0.516345
F -1.345305 1.632096 -1.215735
F -2.927584 -2.248503 0.952792
F -5.368546 -1.164688 1.081510
F -5.812569 1.305229 0.072132
F -3.792236 2.693005 -1.072019
C -0.694361 -0.981147 -0.225117
O 0.284277 -0.169955 0.059535
O -0.548531 -2.148946 -0.530257
I 2.165841 -1.226629 -0.183390
C 3.005383 0.665459 0.256970
C 4.246078 0.931829 -0.289557
C 2.326075 1.558699 1.063487
C 4.830703 2.160472 -0.008659
C 2.930635 2.781186 1.330403
H 1.354468 1.323098 1.466812
C 4.175961 3.082204 0.796426
H 5.801089 2.390986 -0.425340
H 2.420142 3.495297 1.961260
H 4.637712 4.036232 1.009929
F 4.079524 -1.869716 -0.360658
H 4.753262 0.196432 -0.895244

PhIF_5FPh.log
Energy (E) = -1069.92531358 Hartree
Enthalpy (H) = -1069.764124 Hartree
Gibbs free energy (G) = -1069.830318
Hartree
Charge = 0, Spin = 1
C -2.516535 1.105421 0.948970
C -3.421991 0.572971 0.041634
C -3.041400 -0.461949 -0.797909
C -1.747170 -0.954763 -0.718138
C -0.826245 -0.444455 0.176269
C -1.234621 0.585039 1.005297
F -1.409601 -1.944979 -1.543142
F -3.909605 -0.963787 -1.662594
F -4.650480 1.055202 -0.021273
F -2.888492 2.095395 1.745433
F -0.381634 1.117833 1.876842
I 1.168312 -1.306239 0.373712
C 1.992781 0.522602 -0.337342
C 3.254016 0.858509 0.112353
C 1.265261 1.312770 -1.204844
C 3.799357 2.058876 -0.327708
C 1.832716 2.505160 -1.638651
H 0.287642 1.017461 -1.557502
C 3.093244 2.880459 -1.195811
H 4.784317 2.345588 0.013417
H 1.284554 3.133228 -2.326981
H 3.528536 3.810591 -1.533969
F 3.206604 -1.860094 0.544419
H 3.796814 0.183825 0.756465

PhIF_Acr.log
Energy (E) = -896.971482449 Hartree
Enthalpy (H) = -896.687558 Hartree
Gibbs free energy (G) = -896.755042
Hartree
Charge = 0, Spin = 1
C 3.712131 -2.624474 -1.236998
C 2.502257 -2.006879 -1.169532
C 2.379964 -0.724078 -0.560947
C 3.553159 -0.104730 -0.024065
C 4.802061 -0.783387 -0.114793
C 4.879244 -2.006369 -0.703680
C 3.410553 1.144488 0.568157
C 2.161404 1.751586 0.626941
C 1.051399 1.049241 0.055993
C -0.242161 1.658147 0.119605
C -0.398735 2.875192 0.708540
C 0.709285 3.574725 1.268827
C 1.951575 3.025933 1.228473
H 3.797000 -3.598206 -1.700271
H 1.601803 -2.455358 -1.565585
H 5.682693 -0.303861 0.294612
H 5.828790 -2.519391 -0.771300
H 4.275597 1.647111 0.987861
H -1.382485 3.327045 0.759626
H 0.548982 4.540641 1.727478
H 2.803671 3.542832 1.652504
N 1.175170 -0.148315 -0.514058
I -1.913878 0.734335 -0.872554
C -1.942318 -0.866274 0.531735
C -2.607568 -2.021126 0.170505
C -1.291076 -0.725099 1.741363
C -2.612222 -3.078787 1.073236
C -1.312939 -1.791516 2.632224
H -0.783003 0.191095 2.006245
C -1.967540 -2.968712 2.297547
H -3.128958 -3.991845 0.810814
H -0.815627 -1.694260 3.587566
H -1.979954 -3.796761 2.993185
F -3.615686 -0.330965 -1.715866
H -3.128821 -2.059185 -0.774960

PhIF_Adm.log
Energy (E) = -732.264761541 Hartree
Enthalpy (H) = -731.922796 Hartree
Gibbs free energy (G) = -731.984140
Hartree
Charge = 0, Spin = 1
C -1.591604 0.248020 -1.421313
H -1.622223 -0.608186 -2.102621
H -0.921458 0.992227 -1.857068
C -1.096813 -0.193307 -0.052399
C -2.991402 0.855545 -1.276243
H -3.340177 1.189222 -2.255673
C -3.945254 -0.199284 -0.719619
H -4.005391 -1.048426 -1.406585
H -4.950759 0.220003 -0.624921
C -2.042095 -1.254815 0.504455
H -1.683485 -1.596751 1.480695
H -2.069977 -2.124943 -0.163415
C -3.447790 -0.665470 0.647027
H -4.117818 -1.429839 1.045317

C -3.389211 0.523586 1.604932
H -4.388178 0.952061 1.725063
H -3.051358 0.192525 2.591118
C -1.029491 0.979715 0.908420
H -0.341397 1.744171 0.539840
H -0.659329 0.643192 1.882568
C -2.925477 2.044984 -0.318011
H -2.250263 2.808114 -0.716801
H -3.915621 2.499318 -0.222574
C -2.433443 1.580101 1.052718
H -2.388901 2.430493 1.735922
I 0.811016 -1.331551 -0.148933
C 2.076249 0.358026 -0.067926
C 3.115521 0.320964 0.844569
C 1.862302 1.438031 -0.902828
C 3.949787 1.426721 0.934666
C 2.710807 2.536063 -0.804734
H 1.067740 1.428311 -1.633798
C 3.746398 2.533139 0.117998
H 4.769378 1.415758 1.639892
H 2.562205 3.384794 -1.458101
H 4.406251 3.386844 0.190853
F 2.801876 -2.280217 -0.024928
H 3.289132 -0.576850 1.417810

PhIF_Allene.log
Energy (E) = -458.456792837 Hartree
Enthalpy (H) = -458.305428 Hartree
Gibbs free energy (G) = -458.357588 Hartree
Charge = 0, Spin = 1
C 2.680123 1.554346 -0.113531
C 1.720748 1.006853 -0.789496
H 1.402667 1.370243 -1.757919
C 3.646803 2.069834 0.594192
H 4.666246 1.726052 0.479546
H 3.456027 2.858410 1.310112
I 0.797067 -0.821414 -0.083660
C -1.091998 0.156291 -0.000612
C -2.225073 -0.630559 -0.081665
C -1.140876 1.532352 0.109164
C -3.460529 0.005073 -0.052877
C -2.387429 2.148003 0.141470
H -0.238941 2.122861 0.182271
C -3.544346 1.387095 0.055738
H -4.360562 -0.591097 -0.113851
H -2.445190 3.223444 0.237896
H -4.511495 1.870279 0.080166
F -0.363513 -2.524017 0.552265
H -2.126232 -1.704852 -0.128912

PhIF_Anth2.log
Energy (E) = -880.934984084 Hartree
Enthalpy (H) = -880.639204 Hartree
Gibbs free energy (G) = -880.707216 Hartree
Charge = 0, Spin = 1
C 6.934655 0.602423 -0.105980
C 5.921223 0.914431 0.745654
C 4.587954 0.484702 0.482581
C 4.333336 -0.283649 -0.697402
C 5.422258 -0.589909 -1.564672
C 6.681243 -0.161824 -1.279770
C 3.530827 0.790455 1.338292
C 3.033635 -0.709730 -0.964644
C 1.980092 -0.404109 -0.108585
C 2.233426 0.365071 1.070841
C 1.141050 0.670469 1.937507
H 1.339094 1.251376 2.830129
C -0.116780 0.239199 1.657828
C -0.368624 -0.522245 0.479422
C 0.643059 -0.829746 -0.374219
H 3.724639 1.372493 2.232687
H 7.942654 0.933625 0.103819
H 6.107459 1.495304 1.640630
H 5.222330 -1.172065 -2.455836
H 7.500282 -0.400056 -1.944709
H 2.839830 -1.292495 -1.858586
H -0.937870 0.474765 2.323854
H 0.458856 -1.401336 -1.277908
I -2.335822 -1.301264 0.110744
C -3.066746 0.666317 -0.274451
C -4.426144 0.879356 -0.147498
C -2.179218 1.667978 -0.618480
C -4.906502 2.163730 -0.374859
C -2.682171 2.944120 -0.847537
H -1.120934 1.476327 -0.722562
C -4.041194 3.193244 -0.720747
H -5.967148 2.352846 -0.282106
H -2.003426 3.737862 -1.127716
H -4.426645 4.187992 -0.898206
F -4.415452 -1.841946 -0.257428
H -5.073759 0.046154 0.084250

PhIF_Bn.log
Energy (E) = -613.214786342 Hartree
Enthalpy (H) = -612.988345 Hartree
Gibbs free energy (G) = -613.048250 Hartree
Charge = 0, Spin = 1
C 4.161514 0.263277 1.453533
C 4.985494 0.133869 0.341434
C 4.471128 0.365710 -0.927429
C 3.141044 0.730240 -1.083174
C 2.309032 0.875019 0.026699
C 2.832517 0.624659 1.296186
H 4.555714 0.080570 2.444002
H 6.022035 -0.148674 0.463977
H 5.106150 0.263717 -1.796983
H 2.739495 0.913644 -2.073034
H 2.186394 0.718544 2.161286
C 0.873044 1.216917 -0.143648
H 0.459254 1.766791 0.697951
H 0.684171 1.737905 -1.077670
I -0.162354 -0.740813 -0.200612
C -2.074786 0.169887 -0.021234
C -3.109553 -0.592789 0.488191
C -2.239347 1.495228 -0.382696
C -4.353621 0.007730 0.638130
C -3.493175 2.076779 -0.229821
H -1.429457 2.078808 -0.792872
C -4.547685 1.336455 0.283986
H -5.174555 -0.574605 1.033442
H -3.637851 3.108640 -0.518970
H -5.521120 1.792437 0.401932
F -1.353985 -2.562171 -0.163481
H -2.933257 -1.633011 0.719762

PhIF_Carbazole.log
Energy (E) = -858.916614072 Hartree
Enthalpy (H) = -858.639031 Hartree
Gibbs free energy (G) = -858.705739 Hartree
Charge = 0, Spin = 1
C 3.190097 3.102435 0.257531
C 1.953957 3.414615 -0.320663
C 1.054863 2.424872 -0.680080
C 1.419550 1.101418 -0.449392
C 2.654275 0.773488 0.143826
C 3.545465 1.786249 0.493397
H 3.869170 3.900633 0.523749
H 1.694583 4.450860 -0.490558
H 0.101338 2.675280 -1.126708
H 4.499494 1.544774 0.945043
C 1.467717 -1.139345 -0.265698
C 1.161605 -2.497310 -0.277532
C 2.101087 -3.375774 0.235233
C 3.320342 -2.920838 0.751157
C 3.617845 -1.569754 0.768576
C 2.685256 -0.666574 0.261910
H 0.221700 -2.858082 -0.674740
H 1.887245 -4.436064 0.236600
H 4.032116 -3.635147 1.141366
H 4.558930 -1.217152 1.171675
N 0.711745 -0.064340 -0.721102
I -1.289118 -0.156020 -1.329158
C -1.921703 0.049511 0.711189
C -3.279274 0.105424 0.957666
C -0.968427 0.123508 1.707965
C -3.693344 0.242685 2.278035
C -1.408258 0.259780 3.020146
H 0.089804 0.077640 1.501292
C -2.764418 0.319885 3.305780
H -4.751803 0.288593 2.493754
H -0.677307 0.318282 3.814324
H -3.096685 0.426505 4.329224
F -3.324845 -0.229169 -1.767652
H -3.983752 0.040187 0.142667

PhIF_CCPh.log
Energy (E) = -650.020905196 Hartree
Enthalpy (H) = -649.811577 Hartree
Gibbs free energy (G) = -649.874556 Hartree
Charge = 0, Spin = 1
C -0.659647 -0.594108 0.013719
C -1.843224 -0.343228 0.013849
C -3.237343 -0.026585 0.021041
C -3.963034 -0.001342 -1.173623
C -3.890090 0.260729 1.223545
C -5.314162 0.305372 -1.163062
H -3.455848 -0.225237 -2.101999
C -5.241141 0.567727 1.228068
H -3.326213 0.238991 2.145908
C -5.955691 0.590642 0.036279
H -5.868105 0.321857 -2.091665
H -5.738193 0.788667 2.162711
H -7.010398 0.829770 0.042240
I 1.301565 -1.332499 -0.000446
C 2.072767 0.668040 -0.026084
C 3.430470 0.822549 0.172160
C 1.211516 1.727471 -0.226580
C 3.940274 2.116352 0.162893
C 1.745579 3.011206 -0.234364
H 0.152449 1.573277 -0.373276
C 3.105354 3.206461 -0.038688
H 5.000650 2.262869 0.315488
H 1.088276 3.854626 -0.394667
H 3.513926 4.207678 -0.044843
F 3.354865 -1.894623 -0.000136
H 4.056942 -0.046170 0.310060

PhIF_Cy.log
Energy (E) = -577.551611576 Hartree
Enthalpy (H) = -577.283953 Hartree
Gibbs free energy (G) = -577.341846 Hartree
Charge = 0, Spin = 1
C -2.043670 0.760869 -1.094606
C -3.250210 1.658940 -0.832078

C -4.306793 0.909227 -0.029887
C -3.721316 0.387714 1.276840
C -2.507937 -0.502520 1.015784
C -1.461982 0.254143 0.212678
H -5.165350 1.552073 0.167714
H -2.925235 2.538895 -0.266517
H -3.663708 2.019394 -1.774949
H -2.363326 -0.101782 -1.691260
H -1.292230 1.283138 -1.690207
H -3.408489 1.236083 1.894355
H -4.473059 -0.161179 1.845472
H -2.084291 -0.860908 1.955415
H -2.830802 -1.386500 0.450317
H -1.057563 1.068108 0.817892
H -4.670631 0.063253 -0.622751
I 0.186909 -1.159067 -0.134588
C 1.673869 0.355678 0.018669
C 2.945793 -0.039175 0.388591
C 1.348717 1.677332 -0.228214
C 3.922084 0.941137 0.520869
C 2.341053 2.642327 -0.095606
H 0.357361 1.972962 -0.536504
C 3.624390 2.276468 0.283498
H 4.922898 0.650494 0.809384
H 2.103310 3.677953 -0.296433
H 4.393219 3.030121 0.385465
F 1.968646 -2.443592 -0.383981
H 3.149901 -1.092084 0.522386

PhIF_Dibenzofuran.log
Energy (E) = -878.774076318 Hartree
Enthalpy (H) = -878.508676 Hartree
Gibbs free energy (G) = -878.575040 Hartree
Charge = 0, Spin = 1
C -0.333200 -0.223027 1.546287
C 0.105021 -0.680709 0.299406
C -0.768238 -0.815680 -0.771026
C -2.089343 -0.473576 -0.542145
C -2.552317 -0.005521 0.691720
C -1.659157 0.122327 1.752569
H 0.377257 -0.132578 2.358399
H -0.443737 -1.165665 -1.742178
H -1.991729 0.478748 2.718624
C -4.223595 -0.109092 -0.827453
C -5.476699 -0.017764 -1.404119
C -6.501359 0.442128 -0.589854
C -6.273116 0.793931 0.744934
C -5.009535 0.694829 1.302915
C -3.966630 0.235391 0.502176
H -5.640295 -0.292878 -2.435762
H -7.499474 0.530252 -0.996504
H -7.098583 1.147783 1.346768
H -4.836991 0.967445 2.335696
O -3.091580 -0.538180 -1.462752
I 2.153942 -1.285442 0.049740
C 2.775814 0.754527 -0.124368
C 4.121470 1.024797 0.033233
C 1.837143 1.738011 -0.371681
C 4.533503 2.349348 -0.061668
C 2.272743 3.054938 -0.468755
H 0.789565 1.506985 -0.497369
C 3.616553 3.361624 -0.309679
H 5.582553 2.582703 0.058323
H 1.552783 3.835976 -0.670993
H 3.949108 4.387979 -0.384212
F 4.285378 -1.677723 -0.181468
H 4.810500 0.207711 0.193264

PhIF_Furane3.log
Energy (E) = -571.751408020 Hartree
Enthalpy (H) = -571.585142 Hartree
Gibbs free energy (G) = -571.639156 Hartree
Charge = 0, Spin = 1
C -3.350191 1.561429 0.748541
C -2.253093 0.901326 1.182995
C -1.772113 0.152506 0.059289
C -2.624881 0.424395 -0.957977
O -3.590244 1.278225 -0.554251
H -4.040560 2.235554 1.222389
H -1.830345 0.935508 2.172400
H -2.669453 0.097269 -1.981936
I -0.164502 -1.246190 -0.011114
C 1.266177 0.343699 -0.049572
C 2.574092 0.026977 0.262342
C 0.857297 1.626165 -0.359176
C 3.509433 1.055677 0.258824
C 1.810697 2.638461 -0.361464
H -0.171067 1.849714 -0.602560
C 3.132791 2.355733 -0.049530
H 4.539402 0.829771 0.498427
H 1.510264 3.646985 -0.610217
H 3.869499 3.147414 -0.051374
F 1.610439 -2.493053 -0.055222
H 2.837592 -1.000680 0.467313

PhIF_Mesitylene.log
Energy (E) = -691.771910831 Hartree
Enthalpy (H) = -691.487469 Hartree
Gibbs free energy (G) = -691.555483 Hartree
Charge = 0, Spin = 1
C -3.122383 0.374381 -1.193891
C -3.790883 0.619887 -0.000016
C -3.122349 0.374329 1.193842
C -1.823714 -0.125706 1.216959
C -1.188425 -0.386768 -0.000052
C -1.823728 -0.125615 -1.217036
H -3.620567 0.584700 -2.134150
H -3.620569 0.584633 2.134085
C -1.130650 -0.325867 2.536732
H -0.203863 0.250968 2.582071
H -1.770165 -0.005430 3.356923
H -0.863837 -1.370212 2.701262
C -1.130653 -0.325713 -2.536812
H -0.203827 0.251057 -2.582104
H -0.863928 -1.370082 -2.701386
H -1.770120 -0.005178 -3.357000
C -5.204754 1.125728 0.000025
H -5.408328 1.729100 -0.883659
H -5.910833 0.293289 -0.001123
H -5.408928 1.727241 0.884830
I 0.766399 -1.294912 -0.000011
C 1.725812 0.620906 0.000026
C 3.107539 0.647186 -0.000185
C 0.954848 1.768759 0.000248
C 3.733890 1.888706 -0.000189
C 1.603608 2.999192 0.000238
H -0.125247 1.728631 0.000439
C 2.989595 3.060549 0.000011
H 4.814527 1.930834 -0.000343
H 1.014925 3.906331 0.000412
H 3.488427 4.020135 0.000004
F 2.835180 -2.052955 0.000134
H 3.650056 -0.288838 -0.000306

PhIF_Naph1.log
Energy (E) = -727.440192351 Hartree
Enthalpy (H) = -727.193573 Hartree
Gibbs free energy (G) = -727.254726 Hartree
Charge = 0, Spin = 1
C 2.278669 1.803830 1.905453
C 1.423284 0.897323 1.340423
C 1.842133 0.065486 0.274438
C 3.174475 0.200589 -0.203111
C 4.036357 1.144305 0.403193
C 3.601971 1.929784 1.434472
H 1.938032 2.429578 2.719001
H 0.409885 0.814288 1.710115
C 0.991799 -0.883670 -0.356861
C 3.616140 -0.607999 -1.278519
H 5.049407 1.231124 0.029639
H 4.268252 2.649171 1.890592
C 2.777901 -1.511101 -1.866387
C 1.451123 -1.644314 -1.398760
H 4.635432 -0.491624 -1.625903
H 3.118357 -2.123993 -2.689640
H 0.791890 -2.358965 -1.877062
I -0.994364 -1.335789 0.354376
C -1.729782 0.557563 -0.304183
C -2.931675 0.995835 0.217719
C -0.987335 1.298875 -1.203228
C -3.400526 2.240373 -0.188048
C -1.477390 2.537993 -1.599421
H -0.053329 0.932628 -1.604439
C -2.678543 3.010471 -1.089708
H -4.340237 2.602131 0.206232
H -0.913417 3.127926 -2.308684
H -3.054302 3.975995 -1.399800
F -3.066262 -1.617450 0.972725
H -3.483918 0.353548 0.888683

PhIF_Naph2.log
Energy (E) = -727.439388006 Hartree
Enthalpy (H) = -727.192953 Hartree
Gibbs free energy (G) = -727.254822 Hartree
Charge = 0, Spin = 1
C -5.526106 0.739750 -0.045978
C -4.579416 0.926674 0.923964
C -3.258280 0.455843 0.742245
C -2.927765 -0.215170 -0.461925
C -3.925727 -0.393964 -1.448106
C -5.195809 0.072112 -1.245616
H -2.514909 1.144601 2.646445
H -6.533916 1.103078 0.102386
H -4.826578 1.437406 1.846532
C -2.255180 0.633202 1.727440
C -1.601663 -0.682024 -0.644956
H -3.664457 -0.907488 -2.365231
H -5.953551 -0.069368 -2.004201
C -0.656723 -0.499069 0.325159
C -0.987136 0.164153 1.529568
H -1.355539 -1.183607 -1.575111
H -0.228262 0.302757 2.289984
I 1.307620 -1.330226 0.062777
C 2.134116 0.626705 -0.150970
C 3.487886 0.776247 0.081640
C 1.310657 1.682108 -0.493813
C 4.029999 2.051070 -0.033721
C 1.875299 2.947574 -0.610361
H 0.255840 1.541231 -0.680460
C 3.230126 3.133326 -0.375498
H 5.087536 2.190809 0.143557
H 1.247730 3.783247 -0.887947
H 3.663512 4.120073 -0.465418
F 3.389649 -1.930817 -0.177712
H 4.086223 -0.094613 0.307287

PhIF_OCH2CF3.log
Energy (E) = -794.459662320 Hartree
Enthalpy (H) = -794.304425 Hartree
Gibbs free energy (G) = -794.362515 Hartree
Charge = 0, Spin = 1
O -1.165235 -0.751040 0.471043
C -1.837196 -0.277001 -0.650298
H -2.109863 -1.068769 -1.356313
H -1.277691 0.497179 -1.192383
C -3.125129 0.373381 -0.190902
F -3.917572 -0.481209 0.451142
F -2.886508 1.401580 0.631145
F -3.798847 0.846349 -1.246585
I 0.760660 -1.406856 0.113739
C 1.373327 0.626002 0.071902
C 2.572696 0.932477 -0.539950
C 0.551951 1.570971 0.654652
C 2.960671 2.266939 -0.566891
C 0.961977 2.898960 0.615134
H -0.376568 1.287651 1.128622
C 2.159552 3.245991 0.005380
H 3.895452 2.534664 -1.039391
H 0.337967 3.656641 1.067918
H 2.471119 4.281054 -0.020849
F 2.732873 -1.832981 -0.259942
H 3.189855 0.153285 -0.960120

PhIF_OEt.log
Energy (E) = -496.826377341 Hartree
Enthalpy (H) = -496.650443 Hartree
Gibbs free energy (G) = -496.703036 Hartree
Charge = 0, Spin = 1
O -1.740291 0.831877 0.441666
C -1.929108 1.591288 -0.734234
H -0.974787 1.730285 -1.261492
H -2.606756 1.073653 -1.423537
C -2.501313 2.934279 -0.338972
H -1.807723 3.457965 0.319312
H -2.681726 3.552725 -1.218341
H -3.440928 2.798482 0.194441
I -0.831427 -0.972867 0.130434
C 1.036546 0.035126 0.068768
C 2.126116 -0.622933 -0.466439
C 1.100177 1.322127 0.566386
C 3.339435 0.055081 -0.502556
C 2.324020 1.979643 0.522757
H 0.221101 1.793911 0.982689
C 3.439516 1.349886 -0.012190
H 4.207255 -0.439072 -0.916964
H 2.399172 2.985176 0.913025
H 4.388501 1.867408 -0.043353
F 0.293641 -2.682615 -0.198955
H 2.028757 -1.637038 -0.822620

PhIF_Pyrene.log
Energy (E) = -957.112200774 Hartree
Enthalpy (H) = -956.802282 Hartree
Gibbs free energy (G) = -956.870750 Hartree
Charge = 0, Spin = 1
C -0.672818 2.831620 -1.568705
C -0.036581 1.641408 -1.245154
C -0.741935 0.582043 -0.671912
C -2.130250 0.746126 -0.416044
C -2.772521 1.963753 -0.751394
C -2.026886 2.993392 -1.327099
C -0.126189 -0.670395 -0.309122
C -2.884967 -0.303839 0.176271
C -2.254005 -1.521817 0.520719
C -0.850332 -1.661541 0.260768
C -3.001705 -2.545570 1.102697
H -2.509413 -3.474322 1.363095
C -4.357547 -2.376322 1.343466
C -4.984591 -1.185140 1.008999
C -4.268748 -0.137539 0.426041
C -4.890623 1.105185 0.071691
C -4.176213 2.105436 -0.488766
H -4.650117 3.042555 -0.753924
H -5.949721 1.221552 0.265732
H -0.103579 3.636887 -2.012399
H 1.022519 1.536009 -1.438866
H -2.522670 3.922107 -1.581050
H -0.378496 -2.598609 0.538558
H -4.928321 -3.176762 1.794140
H -6.042953 -1.053850 1.197666
I 1.920767 -1.146880 -0.794196
C 2.650688 0.261363 0.633710
C 3.963740 0.674465 0.514897
C 1.799363 0.738570 1.611681
C 4.434086 1.613248 1.426254
C 2.291688 1.672951 2.515671
H 0.778101 0.394200 1.690441
C 3.604252 2.113397 2.420605
H 5.459522 1.948787 1.353691
H 1.641673 2.051159 3.292551
H 3.981767 2.841734 3.125297
F 4.044754 -1.509752 -1.114013
H 4.590842 0.236213 -0.248094

PhIF_SeMes.log
Energy (E) = -701.100778425 Hartree
Enthalpy (H) = -700.813433 Hartree
Gibbs free energy (G) = -700.886880 Hartree
Charge = 0, Spin = 1
C -2.414496 -1.051616 -0.582836
C -3.483978 -0.530344 -1.305199
C -4.056958 0.695692 -0.995989
C -3.544407 1.400421 0.086950
C -2.475029 0.924553 0.837321
C -1.893245 -0.303026 0.480871
H -3.886476 -1.110338 -2.128712
H -3.995718 2.346592 0.365797
C -1.997685 1.725403 2.016532
H -2.131368 1.173685 2.947817
H -0.932240 1.950974 1.947266
H -2.553111 2.659394 2.088524
C -1.872156 -2.397803 -0.965820
H -0.924717 -2.309667 -1.500472
H -1.689797 -3.012178 -0.084218
H -2.570660 -2.914891 -1.621326
C -5.190616 1.248937 -1.810584
H -5.755035 0.451955 -2.292894
H -5.873915 1.830212 -1.192346
H -4.817491 1.909323 -2.595792
Se -0.449640 -0.963448 1.569023
I 1.647833 -1.189829 -0.225575
C 2.027147 0.898304 -0.243534
C 3.344399 1.302440 -0.319762
C 0.964080 1.777089 -0.203265
C 3.600907 2.668625 -0.323339
C 1.242300 3.138687 -0.216561
H -0.057898 1.424927 -0.182197
C 2.556889 3.582417 -0.267484
H 4.624462 3.012979 -0.374285
H 0.424525 3.845917 -0.194370
H 2.767702 4.642978 -0.274143
F 3.371647 -1.237427 -1.446705
H 4.136605 0.574489 -0.403410

PhIF_SetBu.log
Energy (E) = -509.536940429 Hartree
Enthalpy (H) = -509.305336 Hartree
Gibbs free energy (G) = -509.367317 Hartree
Charge = 0, Spin = 1
Se -1.754826 0.141358 1.012030
C -2.463105 0.784886 -0.748361
C -2.869096 -0.389857 -1.618787
H -3.595497 -1.023068 -1.110076
H -3.320512 -0.015897 -2.542679
H -2.008200 -0.999453 -1.896208
C -3.681916 1.603922 -0.354936
H -3.404564 2.443113 0.283789
H -4.156273 2.000919 -1.257016
H -4.413799 0.992269 0.173806
C -1.436143 1.652743 -1.451022
H -0.541233 1.085478 -1.714167
H -1.866177 2.041834 -2.378807
H -1.137646 2.496837 -0.828713
I 0.357947 -1.326339 0.122568
C 1.741387 0.320464 0.067217
C 2.939990 0.106312 -0.582848
C 1.412790 1.515639 0.671243
C 3.847077 1.159773 -0.629840
C 2.337349 2.552396 0.617216
H 0.463145 1.652277 1.171451
C 3.550196 2.377284 -0.034211
H 4.791688 1.015551 -1.135878
H 2.101418 3.495757 1.090141
H 4.264531 3.188140 -0.074252
F 2.009620 -2.458688 -0.586925
H 3.158233 -0.857120 -1.018189

PhIF_SMes.log
Energy (E) = -1089.88514189 Hartree
Enthalpy (H) = -1089.597037 Hartree
Gibbs free energy (G) = -1089.670105 Hartree
Charge = 0, Spin = 1
C 3.870360 -0.475404 1.069362
C 4.510895 -0.174399 -0.125665
C 3.831747 0.597653 -1.064312
C 2.547882 1.071139 -0.832268
C 1.924618 0.759117 0.388522
C 2.583569 -0.024665 1.347023
H 4.383944 -1.079410 1.809120
H 4.315931 0.837611 -2.004868
C 1.930474 -0.376066 2.651966
H 1.683892 0.519975 3.221613
H 0.990615 -0.907694 2.492463
H 2.587298 -1.005067 3.250281
C 5.901176 -0.667189 -0.407907
H 6.588235 0.167029 -0.555941
H 6.278369 -1.275310 0.412527
H 5.924869 -1.269759 -1.316708
C 1.850939 1.897961 -1.872564
H 1.597616 2.884644 -1.484418
H 2.480994 2.015615 -2.752424
H 0.910530 1.434965 -2.178135
S 0.300780 1.362491 0.722257
I -0.702191 -0.723489 -0.301798
C -2.675314 0.016337 -0.037810
C -3.699390 -0.905240 0.068271
C -2.880522 1.380798 0.035280
C -4.988729 -0.424264 0.260902
C -4.178807 1.840057 0.226131

H -2.063559 2.081446 -0.064387
C -5.229091 0.940968 0.342487
H -5.805807 -1.127261 0.345541
H -4.360831 2.904404 0.279293
H -6.236515 1.304298 0.492010
F -1.686492 -2.413494 -1.086039
H -3.492358 -1.959822 -0.028927

PhIF_SO3Ph.log
Energy (E) = -1197.56517679 Hartree
Enthalpy (H) = -1197.348480 Hartree
Gibbs free energy (G) = -1197.414626 Hartree
Charge = 0, Spin = 1
C 4.775624 1.185565 -0.617259
C 3.786221 1.792543 0.146973
C 2.688026 1.056599 0.571804
C 2.603801 -0.282281 0.218965
C 3.590162 -0.905899 -0.531792
C 4.680957 -0.160482 -0.953775
H 5.630931 1.761417 -0.944073
H 3.874850 2.834903 0.421147
H 1.910211 1.493780 1.183110
H 3.497500 -1.958226 -0.762226
H 5.460306 -0.629392 -1.538271
S 1.207034 -1.223448 0.737324
O 1.625503 -2.576376 0.972392
O 0.522778 -0.501062 1.782840
O 0.328135 -1.174667 -0.549089
I -1.814859 -1.088192 -0.357085
C -1.618353 1.008403 -0.146598
C -2.623491 1.668995 0.528798
C -0.514531 1.637484 -0.683053
C -2.502459 3.045790 0.675737
C -0.415402 3.014203 -0.522738
H 0.251723 1.083775 -1.204521
C -1.403363 3.715214 0.155501
H -3.272601 3.589097 1.204917
H 0.440468 3.531244 -0.934844
H -1.317629 4.786091 0.277278
F -3.801926 -0.779870 -0.243827
H -3.477953 1.135544 0.915715

PhIF_StBu.log
Energy (E) = -898.319021832 Hartree
Enthalpy (H) = -898.086789 Hartree
Gibbs free energy (G) = -898.146688 Hartree
Charge = 0, Spin = 1
C 2.514277 0.911777 0.421772
C 3.605270 1.831112 -0.118262
H 4.322276 1.272756 -0.721084
H 3.178490 2.625611 -0.731161
H 4.138057 2.286656 0.719593
C 3.137337 -0.187343 1.269220
H 3.661136 0.259266 2.117969
H 2.378490 -0.862825 1.665950
H 3.850700 -0.768674 0.685156
C 1.510969 1.714386 1.235015
H 1.057811 2.502179 0.632525
H 0.713293 1.080748 1.626890
H 2.019102 2.174654 2.085978
S 1.740172 0.214399 -1.089745
I -0.018287 -1.291275 -0.225819
C -1.504488 0.251481 -0.105586
C -2.592121 0.016186 0.711767
C -1.341171 1.413889 -0.829239
C -3.557777 1.012740 0.805760
C -2.323071 2.392964 -0.727404
H -0.471214 1.567022 -1.452524
C -3.426293 2.195365 0.091313
H -4.417481 0.852809 1.441743
H -2.218894 3.307778 -1.294404
H -4.185702 2.961402 0.168442
F -1.594396 -2.515671 0.522662
H -2.687642 -0.926462 1.229185

PhIF_Thiazole.log
Energy (E) = -910.715920192 Hartree
Enthalpy (H) = -910.564501 Hartree
Gibbs free energy (G) = -910.619453 Hartree
Charge = 0, Spin = 1
C -3.530739 1.364357 0.379676
C -3.420246 0.963229 -0.915138
C -1.641324 -0.057657 -0.121239
S -2.251795 0.704962 1.291739
H -4.284992 1.992165 0.821506
H -4.100900 1.231605 -1.708303
N -2.344899 0.158255 -1.189709
I 0.100371 -1.339942 -0.088446
C 1.338989 0.392674 -0.089143
C 2.589345 0.278698 0.485162
C 0.867490 1.563980 -0.649295
C 3.396360 1.410174 0.507363
C 1.695105 2.680814 -0.623292
H -0.105271 1.615977 -1.116994
C 2.952348 2.606387 -0.040310
H 4.379130 1.347312 0.953715
H 1.350945 3.604744 -1.066977
H 3.590488 3.479177 -0.021525
F 1.987607 -2.353441 0.002220
H 2.921496 -0.676181 0.863667

PhIF_Thiophene3.log
Energy (E) = -894.679307675 Hartree
Enthalpy (H) = -894.516118 Hartree
Gibbs free energy (G) = -894.571249 Hartree
Charge = 0, Spin = 1
C -3.225101 1.073257 1.117467
C -2.026396 0.455700 1.303325
C -1.554836 -0.173903 0.116977
C -2.413209 -0.019414 -0.929225
S -3.786872 0.887895 -0.482366
H -3.808694 1.620302 1.838660
H -1.493408 0.448422 2.242789
H -2.303579 -0.385021 -1.937170
I 0.245311 -1.327732 -0.008592
C 1.418039 0.461013 -0.056059
C 2.765754 0.344028 0.224242
C 0.812819 1.669665 -0.342258
C 3.534111 1.502652 0.214115
C 1.601891 2.814736 -0.351895
H -0.242535 1.738863 -0.562883
C 2.958067 2.733238 -0.070537
H 4.591542 1.433538 0.429471
H 1.146630 3.768015 -0.582617
H 3.566165 3.627495 -0.077982
F 2.191050 -2.291121 -0.104901
H 3.186083 -0.633907 0.410190

AnthI8BA_B_Bn.log
Energy (E) = -1126.06666230 Hartree
Enthalpy (H) = -1125.650190 Hartree
Gibbs free energy (G) = -1125.733018 Hartree
Charge = 0, Spin = 1
C 7.484209 -1.009879 0.325492
C 6.618720 -0.053513 0.754796
C 5.231950 -0.122919 0.429294
C 4.766592 -1.222855 -0.361680
C 5.706662 -2.204726 -0.792441
C 7.021050 -2.103166 -0.460878
C 4.326155 0.845219 0.855973
C 3.415050 -1.303157 -0.686750
C 2.514965 -0.332859 -0.255065
C 2.974623 0.765238 0.528610
C 2.032444 1.747193 0.936592
H 2.402171 2.585678 1.516642
C 0.698874 1.715683 0.627251
C 0.275747 0.576169 -0.127485
H 4.681254 1.679658 1.450409
H 8.533855 -0.946025 0.578244
H 6.965235 0.780956 1.351848
H 5.347722 -3.032770 -1.391263
H 7.725633 -2.853147 -0.793653
H 3.060133 -2.134926 -1.285325
I -1.755517 0.172428 -0.611389
C 1.127422 -0.390903 -0.558123
H 0.772846 -1.222351 -1.153962
C -0.130682 2.905390 1.056846
H -0.156194 3.608798 0.221741
H 0.448224 3.395662 1.839611
C -1.560966 2.665860 1.568777
H -1.681916 1.614822 1.838614
H -1.676219 3.208604 2.506473
C -2.677952 3.131709 0.612174
H -3.501498 2.412202 0.615504
H -3.089412 4.083179 0.939654
C -2.213768 3.341564 -0.831922
O -1.582716 2.348299 -1.384647
O -2.411802 4.413411 -1.374062
C -1.588932 -1.846676 0.292002
C -2.977616 -2.351857 0.426070
H -1.082509 -1.687927 1.240399
H -0.979086 -2.453900 -0.369175
C -3.554901 -3.118126 -0.586180
C -3.750366 -2.012566 1.537970
C -4.869019 -3.552037 -0.481568
H -2.963219 -3.380282 -1.455587
C -5.062066 -2.447538 1.644958
H -3.310614 -1.407645 2.322971
C -5.624896 -3.219430 0.634885
H -5.302249 -4.150896 -1.271010
H -5.647096 -2.184648 2.515709
H -6.648311 -3.558329 0.717870

FuranIBCMe2S_A_Bn.log
Energy (E) = -1026.54845903 Hartree
Enthalpy (H) = -1026.276798 Hartree
Gibbs free energy (G) = -1026.343360 Hartree
Charge = 0, Spin = 1
C -2.606752 2.789673 0.001011
C -1.306857 2.429378 -0.105032
C -1.330797 1.002291 -0.099715
C -2.614269 0.596236 0.019613
O -3.402271 1.696050 0.080785
H -3.100026 3.744529 0.017391
H -0.463551 3.090335 -0.192142
I 0.229803 -0.367802 -0.153653
S -2.012696 -1.973171 -0.418533
C -3.225855 -0.756546 0.171388
C -3.558389 -0.984616 1.648522
H -4.273021 -0.237841 2.005086
H -3.991589 -1.977398 1.768703
H -2.653125 -0.931946 2.253761
C -4.498142 -0.857744 -0.669204
H -4.908792 -1.862269 -0.570648

H -5.244194 -0.136852 -0.327944
H -4.277976 -0.678112 -1.720275
C 1.726224 1.339007 0.018122
C 3.050147 0.688508 0.073013
H 1.452529 1.869065 0.926010
H 1.572719 1.948409 -0.867765
C 3.756168 0.411597 -1.099725
C 3.585046 0.265366 1.292043
C 4.975553 -0.247530 -1.053030
H 3.343336 0.725674 -2.051395
C 4.804345 -0.393113 1.339455
H 3.037498 0.464104 2.206093
C 5.504951 -0.650096 0.166972
H 5.514112 -0.446956 -1.969598
H 5.209336 -0.706506 2.292191
H 6.456220 -1.162878 0.203817

Indole_NMe_IBCONAc_A_Bn.log
Energy (E) = -1004.65961456 Hartree
Enthalpy (H) = -1004.316965 Hartree
Gibbs free energy (G) = -1004.397419 Hartree
Charge = 0, Spin = 1
C 1.866074 -1.630134 0.000261
C 3.257192 -1.356015 -0.000118
C 4.217190 -2.368168 0.000015
C 3.775722 -3.673934 0.000503
C 2.403217 -3.969370 0.000886
C 1.452407 -2.970934 0.000784
C 1.263089 -0.338786 0.000092
H 5.272205 -2.129702 -0.000253
H 4.493843 -4.482192 0.000612
H 2.085230 -5.002889 0.001291
H 0.407270 -3.238482 0.001133
N 3.457658 0.000216 -0.000528
C 2.238344 0.623162 -0.000399
C 2.006033 2.103871 -0.000834
O 2.941375 2.886334 -0.002013
I -0.716158 0.305070 0.000165
C 4.780495 0.601929 -0.000736
H 5.327932 0.282730 -0.887752
H 4.670955 1.679490 -0.001141
H 5.327951 0.283353 0.886493
N 0.668979 2.339227 -0.000024
C 0.143516 3.621847 0.000727
C -1.374439 3.641813 -0.000403
H -1.765834 3.132247 0.882461
H -1.713935 4.672962 -0.000079
H -1.764480 3.133135 -0.884370
O 0.776471 4.654095 0.002282
C -1.678113 -1.693268 0.000232
C -3.130753 -1.386604 -0.000044
H -1.343998 -2.201896 0.898845
H -1.343700 -2.202162 -0.898119
C -3.816148 -1.208995 -1.202386
C -3.816263 -1.207669 1.202046
C -5.163965 -0.881715 -1.202874
H -3.285988 -1.337773 -2.138856
C -5.164077 -0.880382 1.202062
H -3.286180 -1.335425 2.138704
C -5.840973 -0.718241 -0.000528
H -5.686639 -0.755166 -2.141045
H -5.686832 -0.752795 2.140047
H -6.891930 -0.464154 -0.000720

NaphIBMeUreaMe_Bn.log
Energy (E) = -968.742040121 Hartree
Enthalpy (H) = -968.375203 Hartree
Gibbs free energy (G) = -968.452429 Hartree
Charge = 0, Spin = 1
C 2.823265 -1.694525 -0.332449
C 3.660941 -0.777566 0.346739
C 3.156136 0.486803 0.718775
C 1.859232 0.876381 0.454777
C 1.037104 -0.088732 -0.189948
C 1.485580 -1.310943 -0.582415
H 3.826219 1.177044 1.213345
H 0.828706 -2.009575 -1.084542
I -0.993643 0.380457 -0.457708
C 0.655600 2.974665 -0.037671
O 0.623117 4.175423 0.200019
N 0.089093 2.341859 -1.076167
C -0.686275 3.202778 -1.945496
H -0.063979 3.998406 -2.357247
H -1.082105 2.612330 -2.773827
H -1.519224 3.689922 -1.426651
N 1.372721 2.118660 0.856011
C 2.078426 2.810060 1.920220
H 2.305003 2.102855 2.716788
H 3.006501 3.273974 1.574139
H 1.437173 3.600862 2.293344
C 4.999768 -1.155140 0.613709
C 5.471247 -2.377862 0.222447
C 4.630741 -3.289693 -0.454663
C 3.333238 -2.952734 -0.725631
H 5.643890 -0.454677 1.130717
H 6.496345 -2.653053 0.430957
H 5.018151 -4.253033 -0.756542
H 2.678420 -3.642510 -1.244178
C -1.620616 -1.669081 0.236615
C -3.088399 -1.573818 0.403813
H -1.075234 -1.843433 1.159983
H -1.328112 -2.380705 -0.529192
C -3.943907 -1.865792 -0.660164
C -3.642701 -1.117786 1.602024
C -5.317911 -1.728175 -0.523733
H -3.522687 -2.213318 -1.596617
C -5.015390 -0.982519 1.740301
H -2.985436 -0.875185 2.428905
C -5.858134 -1.288674 0.677945
H -5.967408 -1.966436 -1.355202
H -5.429615 -0.637267 2.677921
H -6.928838 -1.183295 0.786171

NpthIBCMe2O_C_Bn.log
Energy (E) = -859.309852393 Hartree
Enthalpy (H) = -858.956902 Hartree
Gibbs free energy (G) = -859.028606 Hartree
Charge = 0, Spin = 1
C 3.243396 0.801115 0.137113
C 1.913885 1.107179 0.013437
C 1.017230 0.038501 -0.132979
C 1.393717 -1.268113 -0.156470
H 3.972109 1.593964 0.259945
H 0.683271 -2.074724 -0.272716
O 0.122288 2.518507 -0.608750
I -1.010884 0.656103 -0.286776
C 3.228266 -2.926882 -0.076479
C 4.561846 -3.206844 0.038164
C 5.497434 -2.159397 0.190829
C 5.078737 -0.857904 0.224515
C 3.705069 -0.536283 0.108711
C 2.768789 -1.589315 -0.041859
H 2.503916 -3.723685 -0.194194
H 4.906018 -4.231838 0.012682
H 6.549707 -2.392964 0.280546
H 5.791828 -0.050824 0.339457
C 1.328638 2.522050 0.061553
C 2.254535 3.524349 -0.621488
H 1.752963 4.491411 -0.626279
H 3.207410 3.631901 -0.100325
H 2.436163 3.220509 -1.651548
C 1.142158 2.906981 1.536003
H 2.091808 2.916483 2.074520
H 0.689190 3.897705 1.581533
H 0.473614 2.198338 2.029890
C -1.766494 -1.429497 0.037353
C -3.236410 -1.260350 0.125732
H -1.318300 -1.796637 0.957215
H -1.465246 -2.032135 -0.815139
C -4.030417 -1.325519 -1.020532
C -3.845652 -0.952181 1.343932
C -5.399851 -1.114872 -0.947464
H -3.564852 -1.552347 -1.972717
C -5.214401 -0.743302 1.418702
H -3.234472 -0.883698 2.236464
C -5.996864 -0.825933 0.273030
H -6.001797 -1.177231 -1.844022
H -5.672112 -0.515077 2.371870
H -7.064354 -0.663396 0.331127

NpthISO2NMe_D_Bn.log
Energy (E) = -1309.29308509 Hartree
Enthalpy (H) = -1308.971592 Hartree
Gibbs free energy (G) = -1309.045832 Hartree
Charge = 0, Spin = 1
C 1.734448 3.498249 -0.540495
C 2.930694 3.133367 -0.001466
C 3.201564 1.786722 0.331437
C 2.225142 0.761045 0.122342
C 0.970857 1.213272 -0.378626
C 0.742908 2.517202 -0.720724
H 5.175096 2.280701 1.014512
H 1.532281 4.521874 -0.821623
H 3.709429 3.865347 0.170754
C 4.474751 1.469002 0.862928
C 2.639753 -0.573211 0.408145
H -0.208073 2.812216 -1.137781
C 3.886653 -0.842307 0.902972
C 4.813747 0.183262 1.157993
H 4.150279 -1.878236 1.066758
H 5.789051 -0.057706 1.556511
I -0.756472 -0.021052 -0.580654
S 1.643896 -2.023687 0.156403
O 0.734394 -2.107227 1.288013
O 2.534696 -3.150974 -0.022376
N 0.930897 -1.601234 -1.211297
C 0.241569 -2.706092 -1.870010
H -0.077141 -2.372962 -2.857196
H 0.907880 -3.560590 -1.995079
H -0.644399 -3.051228 -1.318035
C -2.118532 1.610467 0.122021
C -3.392576 0.911860 0.417659
H -2.224236 2.350157 -0.665135
H -1.625349 2.031705 0.993875
C -3.536193 0.167151 1.590852
C -4.444144 0.934958 -0.498359
C -4.714579 -0.512930 1.853169
H -2.712256 0.122231 2.293512
C -5.623840 0.251896 -0.237169
H -4.336196 1.503172 -1.414861
C -5.762855 -0.470461 0.940512
H -4.814542 -1.081097 2.767790
H -6.434568 0.285067 -0.952288
H -6.681756 -1.002389 1.145373

perF_CMe2O_Bn.log

Energy (E) = -1102.59505311 Hartree
Enthalpy (H) = -1102.319911 Hartree
Gibbs free energy (G) = -1102.393642 Hartree
Charge = 0, Spin = 1
C 2.682919 2.082072 0.013454
C 3.666148 1.154525 0.306077
C 3.356441 -0.197672 0.307254
C 2.082246 -0.652075 0.012096
C 1.104065 0.297207 -0.220286
C 1.391301 1.645884 -0.239260
O 0.496452 -2.220306 -0.753880
I -0.863997 -0.528224 -0.406882
F 0.480700 2.576570 -0.524559
F 2.973412 3.373886 -0.026032
F 4.897335 1.561743 0.569484
F 4.340097 -1.039723 0.623898
C 1.680223 -2.142399 -0.063320
C 2.716751 -2.956735 -0.839327
H 3.658617 -3.065433 -0.306907
H 2.281657 -3.942585 -0.998902
H 2.894628 -2.501437 -1.813020
C 1.544881 -2.668399 1.369480
H 1.248456 -3.716356 1.326257
H 2.483142 -2.582114 1.920251
H 0.770761 -2.111700 1.901975
C -1.953078 1.380026 0.033665
C -3.337168 0.888665 0.217690
H -1.835030 2.035728 -0.820106
H -1.514890 1.811785 0.927899
C -3.789497 0.472336 1.471944
C -4.188337 0.753182 -0.881050
C -5.069820 -0.034925 1.628771
H -3.128263 0.558573 2.326227
C -5.469197 0.242600 -0.725728
H -3.840539 1.065032 -1.859159
C -5.914176 -0.149432 0.530146
H -5.410857 -0.342327 2.608045
H -6.120522 0.152732 -1.584414
H -6.913033 -0.545029 0.652865

perF_NMeCO2_Bn.log
Energy (E) = -1192.64562620 Hartree
Enthalpy (H) = -1192.398245 Hartree
Gibbs free energy (G) = -1192.473314 Hartree
Charge = 0, Spin = 1
C -2.621955 -2.188984 -0.015144
C -3.565366 -1.294525 0.462769
C -3.274459 0.055900 0.564464
C -2.014786 0.577319 0.242608
C -1.074606 -0.363211 -0.203523
C -1.375489 -1.702121 -0.361221
I 0.846077 0.312338 -0.642564
F -0.472503 -2.555806 -0.842645
F -2.912606 -3.474777 -0.146485
F -4.773480 -1.728507 0.777424
F -4.265100 0.859203 0.940173
N -1.723786 1.922856 0.344891
C -2.336626 2.741034 1.385284
H -2.533736 2.124382 2.259954
H -1.634927 3.528583 1.643145
H -3.261858 3.208844 1.054107
C -1.141095 2.644635 -0.770582
O -1.215822 3.855560 -0.728575
O -0.611942 1.894127 -1.660717
C 1.863520 -1.250553 0.534273
C 3.281648 -0.816482 0.608712
H 1.720626 -2.183260 0.002676
H 1.353894 -1.261743 1.493424
C 3.708051 0.048899 1.616661
C 4.192790 -1.227657 -0.364012
C 5.025123 0.478570 1.662307
H 3.000089 0.379316 2.367663
C 5.510588 -0.796007 -0.319696
H 3.863902 -1.896450 -1.150768
C 5.928875 0.056315 0.694183
H 5.347621 1.143226 2.451798
H 6.210802 -1.126024 -1.074595
H 6.956036 0.391936 0.729885

PyIBCMe2O_D_Bn.log
Energy (E) = -721.839310202 Hartree
Enthalpy (H) = -721.547785 Hartree
Gibbs free energy (G) = -721.613345 Hartree
Charge = 0, Spin = 1
C 2.724598 2.853084 0.115233
C 3.864653 2.077333 0.268505
C 2.695895 0.121039 0.058791
C 1.518212 0.832701 -0.084456
H 2.786514 3.931522 0.132145
H 4.829269 2.548398 0.417129
O 1.460771 -1.781309 -0.594766
I -0.202790 -0.385331 -0.269216
C 1.504310 2.209955 -0.052701
H 0.592959 2.779928 -0.160124
N 3.856722 0.746094 0.248606
C 2.628655 -1.401942 0.041016
C 3.821589 -1.968058 -0.720658
H 4.761952 -1.665280 -0.262315
H 3.741983 -3.054674 -0.717076
H 3.796175 -1.620552 -1.752917
C 2.656223 -1.877020 1.499040
H 2.602560 -2.965618 1.510560
H 3.565657 -1.550564 2.005484
H 1.791818 -1.483282 2.039673
C -1.566434 1.367699 0.059142
C -2.913297 0.752448 0.117640
H -1.450717 2.046292 -0.781846
H -1.269761 1.841221 0.991888
C -3.412729 0.248934 1.320730
C -3.672738 0.588103 -1.042139
C -4.649789 -0.375537 1.368156
H -2.822370 0.357602 2.223262
C -4.909978 -0.038069 -0.996236
H -3.288142 0.964664 -1.982949
C -5.403872 -0.519358 0.209590
H -5.026590 -0.751400 2.309829
H -5.489646 -0.149782 -1.902582
H -6.368796 -1.005999 0.246233

PyrroleNMeIBCMe2O_A_Bn.log
Energy (E) = -723.031380822 Hartree
Enthalpy (H) = -722.716384 Hartree
Gibbs free energy (G) = -722.785037 Hartree
Charge = 0, Spin = 1
C 2.790099 2.470044 0.092797
C 1.438719 2.254272 -0.004226
C 1.303568 0.850667 -0.079520
C 2.524858 0.252260 -0.015972
H 3.346636 3.390732 0.149574
H 0.671603 3.008252 -0.026007
I -0.324661 -0.430685 -0.146101
O 1.464362 -1.787351 -0.371648
N 3.443019 1.261284 0.092339
C 4.884549 1.128087 0.133656
H 5.178502 0.362667 0.848797
H 5.286512 0.867751 -0.844742
H 5.308538 2.078558 0.448937
C 2.682008 -1.258372 0.010273
C 3.749937 -1.746716 -0.971513
H 4.763129 -1.473046 -0.670670
H 3.682945 -2.833710 -1.008697
H 3.546361 -1.354927 -1.967938
C 3.027958 -1.706853 1.436381
H 3.100675 -2.794759 1.448021
H 3.969111 -1.285748 1.799827
H 2.225146 -1.404569 2.110673
C -1.712884 1.296642 0.039558
C -3.083517 0.732348 0.068605
H -1.529137 1.930648 -0.823940
H -1.437538 1.807141 0.958991
C -3.648974 0.304408 1.271120
C -3.804733 0.548366 -1.111939
C -4.910491 -0.271309 1.295810
H -3.090246 0.432237 2.191116
C -5.066545 -0.027736 -1.088977
H -3.369122 0.867867 -2.051523
C -5.624583 -0.437855 0.115416
H -5.337683 -0.590404 2.237009
H -5.615575 -0.156287 -2.012116
H -6.608667 -0.885793 0.134035

AnthI8BA_B_carbazole.log
Energy (E) = -1371.76468096 Hartree
Enthalpy (H) = -1371.297044 Hartree
Gibbs free energy (G) = -1371.384871 Hartree
Charge = 0, Spin = 1
C -6.513365 -2.208629 -0.544080
C -5.362319 -2.865321 -0.240403
C -4.097718 -2.216501 -0.356491
C -4.064062 -0.855109 -0.802184
C -5.293732 -0.201493 -1.108668
C -6.479250 -0.854958 -0.985023
C -2.905919 -2.867683 -0.045207
C -2.836883 -0.209104 -0.923544
C -1.650471 -0.870637 -0.619048
C -1.678561 -2.220610 -0.163274
C -0.445595 -2.853532 0.153823
H -0.478324 -3.885974 0.483690
C 0.776125 -2.242490 0.065614
C 0.744444 -0.881943 -0.370383
H -2.936638 -3.896119 0.296958
H -7.466228 -2.712101 -0.452431
H -5.383026 -3.894666 0.095678
H -5.258231 0.827459 -1.444558
H -7.406238 -0.350743 -1.221878
H -2.800896 0.824963 -1.250670
I 2.460369 0.394356 -0.339004
C -0.389065 -0.225835 -0.715968
H -0.370802 0.804312 -1.048071
C 2.003159 -3.062783 0.384094
H 2.486802 -3.344435 -0.553061
H 1.636994 -3.992495 0.817508
C 3.028810 -2.425944 1.341791
H 2.587626 -1.540238 1.799301
H 3.201235 -3.111967 2.169534
C 4.395840 -2.090848 0.711815
H 4.768389 -1.140797 1.104006
H 5.134173 -2.847618 0.962624
C 4.372374 -2.024194 -0.807795
O 3.476203 -1.227889 -1.360067
O 5.114237 -2.709751 -1.472784
N 1.227651 1.912767 0.543936
C 0.351417 1.623740 1.586725
C 0.598572 2.867004 -0.259561
C 0.498095 0.695341 2.613308

C -0.811130 2.409592 1.479968
C 1.050602 3.447290 -1.441594
C -0.649187 3.217713 0.292406
C -0.532120 0.568495 3.529494
H 1.393174 0.092093 2.700264
C -1.833752 2.275363 2.416709
C 0.237015 4.383828 -2.058932
H 2.009552 3.182672 -1.867975
C -1.448115 4.168875 -0.338312
C -1.689642 1.350304 3.435582
H -0.440204 -0.148306 4.334254
H -2.729275 2.879544 2.342802
C -0.999920 4.746050 -1.514449
H 0.568154 4.847124 -2.978441
H -2.403812 4.450837 0.085766
H -2.475947 1.226138 4.167219
H -1.606962 5.485343 -2.018540

FuranIBCMe2S_A_carbazole.log
Energy (E) = -1272.24947648 Hartree
Enthalpy (H) = -1271.926731 Hartree
Gibbs free energy (G) = -1271.998962 Hartree
Charge = 0, Spin = 1
C 0.895219 0.665814 2.853571
C 0.118732 0.271097 1.817555
C 1.052817 -0.002812 0.779214
C 2.297335 0.253873 1.228387
O 2.212974 0.665485 2.509731
H 0.668311 0.954023 3.863542
H -0.952814 0.172206 1.799336
I 0.815510 -0.562955 -1.199215
S 3.376831 -0.870774 -0.926791
C 3.594224 0.256925 0.492755
C 3.904399 1.677615 0.019901
H 4.003473 2.348542 0.876121
H 4.836352 1.681823 -0.544697
H 3.107032 2.047692 -0.625910
C 4.725450 -0.278428 1.365026
H 5.650889 -0.302167 0.790014
H 4.868356 0.373659 2.228252
H 4.503387 -1.285879 1.712071
N -1.336152 -0.204576 -0.876899
C -1.866855 1.062090 -0.691392
C -2.176038 -1.103062 -0.236794
C -1.355286 2.295254 -1.093426
C -3.087010 0.977510 0.013927
C -2.041620 -2.483630 -0.094957
C -3.287095 -0.422074 0.307841
C -2.086950 3.432124 -0.796687
H -0.415694 2.364495 -1.627674
C -3.810994 2.134904 0.294612
C -3.035268 -3.168627 0.582844
H -1.188528 -3.008633 -0.505945
C -4.280005 -1.134067 0.978908
C -3.306954 3.357393 -0.112842
H -1.708937 4.399087 -1.101029
H -4.752400 2.076956 0.827078
C -4.148703 -2.504849 1.113305
H -2.950572 -4.240413 0.703533
H -5.139586 -0.619777 1.390887
H -3.854959 4.265336 0.098620
H -4.908769 -3.072098 1.632824

Indole_NMe_IBCONAc_A_carbazole.log
Energy (E) = -1250.35538859 Hartree
Enthalpy (H) = -1249.961775 Hartree
Gibbs free energy (G) = -1250.047290 Hartree
Charge = 0, Spin = 1
C 0.292824 1.934860 -0.000781
C 1.266380 2.967513 -0.000872
C 0.916800 4.318568 -0.001360
C -0.425047 4.629984 -0.001760
C -1.403842 3.621460 -0.001730
C -1.066669 2.285991 -0.001270
C 1.074261 0.745790 -0.000342
H 1.676644 5.088353 -0.001428
H -0.732059 5.666819 -0.002135
H -2.448301 3.900230 -0.002096
H -1.842765 1.536203 -0.001319
N 2.527160 2.421799 -0.000507
C 2.403167 1.060863 -0.000181
C 3.492146 0.052727 0.000302
O 4.667286 0.354995 0.000713
I 0.660721 -1.280769 0.000423
C 3.745654 3.211501 -0.000621
H 3.774873 3.844516 0.886138
H 4.596198 2.540415 -0.000272
H 3.775105 3.843924 -0.887797
N 2.934113 -1.208587 0.000381
C 3.708428 -2.368970 0.000061
C 2.896339 -3.649077 0.001417
H 2.260401 -3.710157 -0.884149
H 3.582108 -4.490097 0.001533
H 2.261388 -3.708992 0.887764
O 4.914595 -2.394168 -0.001251
N -1.414232 -0.924105 0.000321
C -2.213902 -0.767726 1.125585
C -2.214205 -0.769128 -1.124923
C -1.859397 -0.799603 2.471607
C -3.543412 -0.531016 0.722890
C -1.860071 -0.802771 -2.471000
C -3.543607 -0.531917 -0.722165
C -2.860426 -0.606408 3.408310
H -0.835663 -0.965445 2.782305
C -4.534455 -0.340463 1.684288
C -2.861350 -0.610772 -3.407681
H -0.836425 -0.969065 -2.781748
C -4.534907 -0.342592 -1.683543
C -4.187340 -0.381746 3.022698
H -2.610189 -0.626337 4.460377
H -5.559458 -0.160147 1.385500
C -4.188155 -0.385592 -3.021992
H -2.611407 -0.632072 -4.459791
H -5.559831 -0.161898 -1.384713
H -4.943704 -0.234953 3.781222
H -4.944725 -0.239766 -3.780497

NaphIBMeUreaMe_carbazole.log
Energy (E) = -1214.44587856 Hartree
Enthalpy (H) = -1214.027629 Hartree
Gibbs free energy (G) = -1214.110326 Hartree
Charge = 0, Spin = 1
C 0.478600 2.207158 -1.134609
C -0.596833 3.064928 -0.798542
C -1.793186 2.519532 -0.282397
C -1.950869 1.167178 -0.071632
C -0.845229 0.350932 -0.412568
C 0.317900 0.819353 -0.930689
H -2.603622 3.195702 -0.046192
H 1.140581 0.152186 -1.156658
I -1.001918 -1.677365 0.101992
C -3.782946 -0.482805 -0.027579
O -4.987413 -0.604621 0.119007
N -2.991867 -1.357536 -0.698451
C -3.668587 -2.522045 -1.239628
H -4.521001 -2.206968 -1.838926
H -2.976879 -3.071685 -1.878510
H -4.048389 -3.189332 -0.460653
N -3.113011 0.640350 0.509582
C -3.969009 1.552053 1.251803
H -3.347896 2.172239 1.895183
H -4.564324 2.190072 0.594235
H -4.655998 0.964797 1.851209
C -0.435881 4.457834 -0.996073
C 0.737035 4.959150 -1.489354
C 1.813083 4.099441 -1.806665
C 1.685893 2.749550 -1.632059
H -1.257245 5.118177 -0.746959
H 0.848378 6.025286 -1.634450
H 2.736725 4.515605 -2.184565
H 2.501729 2.074382 -1.861281
N 1.096587 -1.562126 0.744079
C 1.527637 -0.516952 1.550081
C 2.146895 -1.863704 -0.116626
C 0.810558 0.186822 2.516800
C 2.860700 -0.175416 1.245247
C 2.194295 -2.810249 -1.138796
C 3.265109 -1.051530 0.170431
C 1.450856 1.219981 3.178506
H -0.220274 -0.061558 2.740097
C 3.490637 0.862717 1.929368
C 3.369328 -2.932904 -1.861769
H 1.341342 -3.439593 -1.359411
C 4.441049 -1.197877 -0.562719
C 2.780557 1.556307 2.893136
H 0.913141 1.780600 3.931496
H 4.516463 1.126791 1.703756
C 4.486257 -2.138178 -1.577854
H 3.426630 -3.661857 -2.659106
H 5.305726 -0.584579 -0.340736
H 3.250399 2.368941 3.429952
H 5.390416 -2.264354 -2.157472

NpthIBCMe2O_C_carbazole.log
Energy (E) = -1105.01552813 Hartree
Enthalpy (H) = -1104.611200 Hartree
Gibbs free energy (G) = -1104.688875 Hartree
Charge = 0, Spin = 1
C -3.265557 1.055314 -0.089098
C -2.576992 -0.127781 -0.091024
C -1.181335 -0.056829 -0.202178
C -0.468698 1.093595 -0.295115
H -4.345625 1.056579 0.001567
H 0.610186 1.106608 -0.376323
O -2.329982 -2.445578 -0.506613
I -0.307272 -1.985960 -0.193900
C -0.508922 3.559297 -0.410554
C -1.214713 4.730399 -0.416471
C -2.623535 4.712628 -0.314307
C -3.295899 3.526353 -0.208504
C -2.594470 2.297240 -0.199464
C -1.180220 2.319020 -0.301233
H 0.571242 3.558718 -0.486572
H -0.696747 5.676167 -0.498321
H -3.170109 5.645955 -0.319839
H -4.375962 3.508367 -0.130302
C -3.191722 -1.512730 0.082026
C -4.535619 -1.631754 -0.620598
H -4.883326 -2.659371 -0.522773
H -5.281432 -0.971207 -0.176979
H -4.426860 -1.396478 -1.678001
C -3.346874 -1.791079 1.579730
H -4.030683 -1.078740 2.043083
H -3.730623 -2.802443 1.714305
H -2.382347 -1.712488 2.087175

N 1.611592 -1.056793 0.079260
C 2.012592 -0.442223 1.256184
C 2.427799 -0.581913 -0.938481
C 1.467045 -0.553684 2.534056
C 3.121344 0.393763 1.008785
C 2.389243 -0.867927 -2.302171
C 3.389243 0.303990 -0.407474
C 2.058629 0.165641 3.558010
H 0.608815 -1.186437 2.724208
C 3.704357 1.104892 2.056243
C 3.330323 -0.265254 -3.119129
H 1.649045 -1.542548 -2.712980
C 4.331597 0.893855 -1.248575
C 3.170165 0.985656 3.326869
H 1.652897 0.093070 4.558242
H 4.560714 1.742936 1.875930
C 4.297107 0.605675 -2.601227
H 3.319104 -0.473297 -4.180711
H 5.077577 1.568807 -0.847434
H 3.609031 1.531512 4.150752
H 5.019882 1.055490 -3.268039

NpthISO2NMe_D_carbazole.log
Energy (E) = -1554.99481891 Hartree
Enthalpy (H) = -1554.622071 Hartree
Gibbs free energy (G) = -1554.702355 Hartree
Charge = 0, Spin = 1
C -0.375409 2.238904 -2.113973
C 0.808211 2.912327 -2.076776
C 1.935067 2.371348 -1.419461
C 1.864697 1.102529 -0.762300
C 0.591778 0.471205 -0.802776
C -0.480984 0.998454 -1.460124
H 3.146503 4.064765 -1.934326
H -1.236641 2.640951 -2.627165
H 0.914707 3.875852 -2.558379
C 3.145959 3.105467 -1.432465
C 3.076901 0.632641 -0.178234
H -1.426919 0.476703 -1.484656
C 4.234574 1.359255 -0.227256
C 4.276490 2.620315 -0.847475
H 5.125474 0.928596 0.208839
H 5.199887 3.180975 -0.869015
I 0.163137 -1.363316 0.223267
S 3.230102 -0.906325 0.677720
O 2.673136 -0.715909 2.002154
O 4.597954 -1.356894 0.588432
N 2.280397 -1.825397 -0.264338
C 2.531401 -3.262907 -0.158046
H 1.976288 -3.765124 -0.948961
H 3.592279 -3.461785 -0.300383
H 2.232057 -3.684443 0.808775
N -1.840914 -0.655412 0.543313
C -2.104771 0.524957 1.225397
C -2.939610 -0.924625 -0.264616
C -1.279804 1.226437 2.103110
C -3.394253 0.989934 0.900281
C -3.130912 -1.968743 -1.166965
C -3.934480 0.054388 -0.060019
C -1.780728 2.386495 2.668226
H -0.281580 0.877571 2.341010
C -3.879939 2.157176 1.487001
C -4.334273 -2.025139 -1.850558
H -2.367208 -2.719395 -1.327010
C -5.141878 -0.028560 -0.750632
C -3.069102 2.848328 2.369653
H -1.162797 2.947962 3.355946
H -4.873843 2.517752 1.252680
C -5.334932 -1.068577 -1.643523
H -4.505464 -2.827858 -2.555222
H -5.915859 0.712059 -0.591959
H -3.428220 3.756913 2.833304
H -6.265681 -1.146141 -2.188478

perF_CMe2O_carbazole.log
Energy (E) = -1348.29240464 Hartree
Enthalpy (H) = -1347.966016 Hartree
Gibbs free energy (G) = -1348.045135 Hartree
Charge = 0, Spin = 1
C 1.276723 2.229164 -1.268020
C 2.630373 2.249125 -0.984417
C 3.241543 1.123159 -0.451371
C 2.521984 -0.029036 -0.193132
C 1.158254 0.000870 -0.426147
C 0.526526 1.095672 -0.981435
O 2.220178 -2.352662 0.053177
I 0.218767 -1.768314 0.290903
F -0.753738 1.117294 -1.312713
F 0.701212 3.282665 -1.821187
F 3.340144 3.333493 -1.243998
F 4.544612 1.203307 -0.185594
C 3.122713 -1.334938 0.351688
C 4.429952 -1.702288 -0.342195
H 5.244796 -1.035177 -0.073638
H 4.681852 -2.715262 -0.030945
H 4.294175 -1.699113 -1.422709
C 3.332465 -1.181209 1.858653
H 3.722198 -2.116996 2.258310
H 4.038317 -0.377627 2.072156
H 2.388697 -0.953528 2.359851
N -1.644523 -0.777474 0.460002
C -1.845883 0.316041 1.291510
C -2.699340 -0.821939 -0.444681
C -1.029597 0.811378 2.305472
C -3.064914 0.942531 0.963700
C -2.918169 -1.705823 -1.496480
C -3.616116 0.205836 -0.150964
C -1.465075 1.927834 3.002198
H -0.085536 0.339956 2.552572
C -3.485543 2.058784 1.680423
C -4.073819 -1.550227 -2.244145
H -2.210571 -2.491738 -1.728798
C -4.776345 0.338825 -0.909377
C -2.682294 2.545739 2.698440
H -0.849751 2.328979 3.796320
H -4.423684 2.543047 1.439900
C -4.999141 -0.541450 -1.954027
H -4.265195 -2.224319 -3.068277
H -5.489507 1.122801 -0.687328
H -2.992842 3.414768 3.261913
H -5.893217 -0.450650 -2.555332

perF_NMeCO2_carbazole.log
Energy (E) = -1438.33402612 Hartree
Enthalpy (H) = -1438.035646 Hartree
Gibbs free energy (G) = -1438.115967 Hartree
Charge = 0, Spin = 1
C 0.843456 2.160758 -1.627399
C 2.127715 2.433461 -1.186608
C 2.852456 1.488474 -0.477044
C 2.305659 0.250455 -0.131081
C 1.000770 0.019119 -0.573996
C 0.287743 0.929484 -1.327106
I 0.157139 -1.792118 0.009240
F -0.928764 0.653216 -1.770830
F 0.173397 3.052230 -2.337097
F 2.687627 3.590822 -1.487384
F 4.108386 1.793385 -0.166237
N 3.007801 -0.694637 0.609361
C 3.984656 -0.276880 1.611901
H 3.666477 0.669329 2.043780
H 4.015599 -1.039408 2.384285
H 4.984235 -0.171552 1.195996
C 3.109533 -2.049001 0.171890
O 4.021386 -2.724151 0.584938
O 2.166080 -2.424596 -0.645472
N -1.590969 -0.832709 0.608249
C -1.562640 0.302538 1.416283
C -2.788675 -0.818057 -0.108869
C -0.554107 0.748406 2.265330
C -2.768176 1.012168 1.266803
C -3.247643 -1.713973 -1.065606
C -3.557979 0.287459 0.294191
C -0.781738 1.914307 2.979662
H 0.376553 0.203177 2.374559
C -2.978235 2.176592 2.000096
C -4.501170 -1.488342 -1.611665
H -2.652751 -2.562410 -1.379715
C -4.820065 0.489114 -0.257278
C -1.981387 2.621683 2.852391
H -0.016850 2.282182 3.649950
H -3.904668 2.727664 1.900545
C -5.284366 -0.401691 -1.209970
H -4.881990 -2.169230 -2.360621
H -5.425128 1.332202 0.051066
H -2.127307 3.526836 3.425325
H -6.260760 -0.258428 -1.651490

PyIBCMe2O_D_carbazole.log
Energy (E) = -967.545188134 Hartree
Enthalpy (H) = -967.202279 Hartree
Gibbs free energy (G) = -967.273713 Hartree
Charge = 0, Spin = 1
C -0.784818 0.713532 2.979789
C -2.152808 0.890973 3.138741
C -2.601074 0.198414 1.011394
C -1.251220 0.009416 0.790874
H -0.106531 0.925395 3.793083
H -2.548902 1.250041 4.081045
O -2.928114 -0.930617 -1.040791
I -0.858330 -0.637510 -1.175887
C -0.303574 0.268470 1.754398
H 0.754642 0.129916 1.580360
N -3.046607 0.649235 2.180751
C -3.557650 -0.058882 -0.142355
C -4.832584 -0.722211 0.351011
H -5.330894 -0.091900 1.085599
H -5.491651 -0.887360 -0.500407
H -4.597503 -1.683584 0.805305
C -3.864868 1.285575 -0.803694
H -4.534318 1.120763 -1.647522
H -4.333974 1.966517 -0.093207
H -2.946148 1.749674 -1.172198
N 1.222001 -0.227520 -0.811071
C 1.750069 1.050614 -0.700936
C 2.116314 -1.101810 -0.207919
C 1.198518 2.265604 -1.104563
C 3.011088 0.997077 -0.071069
C 2.010957 -2.479865 -0.026257
C 3.247674 -0.391652 0.246853
C 1.934252 3.417565 -0.885519
H 0.226694 2.310192 -1.580503
C 3.736678 2.169325 0.134131
C 3.055917 -3.133412 0.603994
H 1.142253 -3.026447 -0.370352
C 4.291023 -1.072283 0.872558

C 3.194222 3.374265 -0.275773
H 1.526844 4.371500 -1.192581
H 4.708875 2.136035 0.610097
C 4.189378 -2.440764 1.047776
H 2.996039 -4.202983 0.754673
H 5.166192 -0.535738 1.217529
H 3.743061 4.293359 -0.123125
H 4.989017 -2.984104 1.532148

PyrroleNMeIBCMe2O_A_carbazole.log
Energy (E) = -968.732961037 Hartree
Enthalpy (H) = -968.366629 Hartree
Gibbs free energy (G) = -968.440956 Hartree
Charge = 0, Spin = 1
C -1.372568 0.271038 2.756258
C -0.410932 0.073683 1.797375
C -1.143538 -0.104604 0.608578
C -2.479756 0.000547 0.837438
H -1.272646 0.421908 3.818028
H 0.656834 0.051463 1.933853
I -0.664563 -0.332603 -1.382638
O -2.782536 -0.566245 -1.401490
N -2.620527 0.235556 2.175749
C -3.861671 0.369485 2.909616
H -4.526345 1.074983 2.414010
H -4.371366 -0.588605 3.004759
H -3.634628 0.748591 3.902849
C -3.471963 -0.031399 -0.303211
C -4.666993 -0.940088 -0.033897
H -5.322540 -0.537226 0.739476
H -5.237580 -1.026969 -0.957922
H -4.325313 -1.932134 0.258468
C -3.934480 1.390938 -0.625937
H -4.609711 1.358807 -1.481063
H -4.451744 1.855804 0.216255
H -3.070397 2.006370 -0.882353
N 1.388078 -0.100090 -0.824600
C 1.969870 1.116617 -0.499057
C 2.157180 -1.099726 -0.245439
C 1.539071 2.406059 -0.804115
C 3.152094 0.899994 0.239129
C 1.952046 -2.477966 -0.244703
C 3.272671 -0.529613 0.403335
C 2.314000 3.469339 -0.373397
H 0.626695 2.573617 -1.362292
C 3.920060 1.985094 0.656859
C 2.884048 -3.273225 0.400020
H 1.091672 -2.916745 -0.733578
C 4.201756 -1.350299 1.039972
C 3.496974 3.265760 0.347523
H 1.998550 4.479258 -0.599529
H 4.832541 1.826510 1.218368
C 4.002678 -2.719605 1.034704
H 2.744582 -4.346032 0.412806
H 5.064376 -0.920386 1.534054
H 4.080162 4.119136 0.665547
H 4.712839 -3.370851 1.525669

AnthI8BA_B_CCPh.log
Energy (E) = -1162.86872247 Hartree
Enthalpy (H) = -1162.469160 Hartree
Gibbs free energy (G) = -1162.554141 Hartree
Charge = 0, Spin = 1
C -6.990510 -1.056424 0.075888
C -5.957908 -1.777491 0.587344
C -4.606373 -1.427584 0.295452
C -4.357163 -0.298389 -0.550418
C -5.468238 0.431828 -1.066238
C -6.742173 0.066505 -0.764326
C -3.531945 -2.150319 0.807913
C -3.043128 0.054554 -0.844824
C -1.973835 -0.673712 -0.330106
C -2.217113 -1.798070 0.511500
C -1.102948 -2.518989 1.017402
H -1.303998 -3.368284 1.661029
C 0.204210 -2.216434 0.741618
C 0.391466 -1.072572 -0.093744
H -3.723250 -3.005225 1.446859
H -8.011198 -1.332003 0.303973
H -6.140900 -2.632658 1.226172
H -5.271394 1.283989 -1.705012
H -7.577628 0.626766 -1.161519
H -2.850054 0.909539 -1.482918
I 2.325392 -0.328305 -0.640501
C -0.622444 -0.335180 -0.609196
H -0.427316 0.529398 -1.230592
C 1.259456 -3.130476 1.322536
H 1.515892 -3.877087 0.568431
H 0.761652 -3.677705 2.122580
C 2.541789 -2.485902 1.877052
H 2.385720 -1.413608 2.004295
H 2.703127 -2.863775 2.885774
C 3.814427 -2.772953 1.055284
H 4.449997 -1.883780 1.019107
H 4.403318 -3.560842 1.517726
C 3.532004 -3.235883 -0.371919
O 2.718287 -2.488432 -1.072847
O 4.016553 -4.268401 -0.786606
C 1.663864 1.609199 -0.208190
C 1.418069 2.778341 -0.020567
C 1.110510 4.153861 0.219243
C 1.181835 5.085979 -0.820102
C 0.735573 4.580441 1.496645
C 0.885258 6.418513 -0.583330
H 1.472888 4.753559 -1.807033
C 0.436561 5.913636 1.726309
H 0.682373 3.857034 2.298465
C 0.511356 6.835090 0.688645
H 0.945127 7.133416 -1.392493
H 0.146207 6.235184 2.717115
H 0.279340 7.875501 0.870973

FuranIBCMe2S_A_CCPh.log
Energy (E) = -1063.34981189 Hartree
Enthalpy (H) = -1063.094965 Hartree
Gibbs free energy (G) = -1063.164309 Hartree
Charge = 0, Spin = 1
C -1.375152 2.978666 -0.355203
C -0.454419 1.987211 -0.373344
C -1.222515 0.801404 -0.206617
C -2.525067 1.132749 -0.085551
O -2.625858 2.477977 -0.178302
H -1.299895 4.045127 -0.464989
H 0.610453 2.071415 -0.495760
I -0.644178 -1.180908 -0.045955
S -3.306593 -1.415636 -0.213499
C -3.729998 0.304856 0.209252
C -4.074839 0.434179 1.694516
H -4.304452 1.472566 1.946501
H -4.942245 -0.186013 1.919502
H -3.241648 0.094199 2.309941
C -4.912446 0.754848 -0.645961
H -5.768794 0.112999 -0.440314
H -5.181827 1.785968 -0.409126
H -4.671331 0.680893 -1.704991
C 1.399501 -0.502592 -0.020896
C 2.592448 -0.296562 0.009829
C 3.997901 -0.031386 0.044259
C 4.754652 -0.070755 -1.130607
C 4.631889 0.269637 1.253377
C 6.115904 0.185720 -1.094334
H 4.262671 -0.304498 -2.064774
C 5.993353 0.525700 1.284095
H 4.044969 0.298762 2.161113
C 6.738381 0.484508 0.111700
H 6.692717 0.152322 -2.008556
H 6.474627 0.757385 2.224490
H 7.800966 0.684276 0.137907

Indole_NMe_IBCONAc_A_CCPh.log
Energy (E) = -1041.45962203 Hartree
Enthalpy (H) = -1041.134025 Hartree
Gibbs free energy (G) = -1041.216323 Hartree
Charge = 0, Spin = 1
C 0.423595 1.922660 -0.000289
C 1.500815 2.844536 -0.000391
C 1.297202 4.225016 -0.000516
C -0.003498 4.680028 -0.000513
C -1.084040 3.782069 -0.000396
C -0.889083 2.417874 -0.000288
C 1.070887 0.654537 -0.000227
H 2.135244 4.908858 -0.000621
H -0.197024 5.743916 -0.000607
H -2.092822 4.171422 -0.000396
H -1.732195 1.745454 -0.000205
N 2.693455 2.165084 -0.000367
C 2.428765 0.823108 -0.000291
C 3.430501 -0.283404 -0.000226
O 4.627041 -0.063648 -0.000083
I 0.407344 -1.312124 0.000001
C 3.985946 2.828331 -0.000554
H 4.076620 3.455677 0.886217
H 4.763938 2.074706 -0.000180
H 4.076786 3.454941 -0.887832
N 2.767291 -1.478338 -0.000295
C 3.437518 -2.695591 -0.000525
C 2.505689 -3.893501 -0.000022
H 1.866174 -3.886059 -0.885308
H 3.101733 -4.800562 0.000070
H 1.866507 -3.885614 0.885497
O 4.637423 -2.842373 -0.000909
C -1.619565 -0.799755 0.000293
C -2.826729 -0.732478 0.000467
C -4.250768 -0.608418 0.000686
C -4.952977 -0.545373 1.208015
C -4.953348 -0.545569 -1.206441
C -6.332901 -0.422658 1.204814
H -4.405434 -0.593359 2.139164
C -6.333268 -0.422855 -1.202839
H -4.406085 -0.593709 -2.137746
C -7.024958 -0.361419 0.001089
H -6.869752 -0.374458 2.142189
H -6.870408 -0.374808 -2.140056
H -8.102162 -0.265713 0.001245

NaphIBMeUreaMe_CCPh.log
Energy (E) = -1005.54610002 Hartree
Enthalpy (H) = -1005.195912 Hartree
Gibbs free energy (G) = -1005.275492 Hartree
Charge = 0, Spin = 1
C -0.672459 2.604207 -0.612050
C -1.861131 2.878073 0.106880
C -2.611859 1.812920 0.648770
C -2.233909 0.494015 0.510884
C -1.023563 0.269795 -0.196709

C -0.268251 1.256379 -0.742794
H -3.522429 2.049835 1.182221
H 0.659569 1.024747 -1.250846
I -0.355012 -1.719966 -0.334967
C -3.365751 -1.687922 0.352166
O -4.379175 -2.289633 0.675423
N -2.553770 -1.991539 -0.676927
C -2.941774 -3.163936 -1.436202
H -3.952056 -3.047502 -1.828773
H -2.258196 -3.287269 -2.278055
H -2.935890 -4.078169 -0.834757
N -2.967640 -0.541615 1.096015
C -3.854449 -0.202380 2.196142
H -3.331746 0.463788 2.880263
H -4.777480 0.274960 1.856621
H -4.129343 -1.119130 2.706243
C -2.270136 4.226627 0.249261
C -1.531487 5.239911 -0.296282
C -0.343815 4.961566 -1.009684
C 0.076284 3.669407 -1.163111
H -3.180279 4.439217 0.796269
H -1.855325 6.265635 -0.181954
H 0.230245 5.774793 -1.431975
H 0.985374 3.441197 -1.705440
C 1.650930 -1.027350 -0.058872
C 2.831306 -0.795948 0.080873
C 4.219966 -0.501087 0.254392
C 5.135618 -0.773735 -0.766340
C 4.679012 0.063510 1.448229
C 6.480527 -0.488023 -0.594286
H 4.779497 -1.210951 -1.689117
C 6.024598 0.349750 1.613974
H 3.969577 0.272713 2.236995
C 6.928443 0.074670 0.594799
H 7.180889 -0.704089 -1.389581
H 6.369491 0.787233 2.540960
H 7.978429 0.297675 0.726953

NpthIBCMe2O_C_CCPh.log
Energy (E) = -896.116526105 Hartree
Enthalpy (H) = -895.780401 Hartree
Gibbs free energy (G) = -895.855113 Hartree
Charge = 0, Spin = 1
C -3.298401 0.989037 0.153850
C -2.581615 -0.175476 0.070915
C -1.199301 -0.056921 -0.125285
C -0.529671 1.117150 -0.229774
H -4.369737 0.955623 0.314505
H 0.543702 1.148804 -0.369471
O -2.317133 -2.474282 -0.420322
I -0.241851 -1.949761 -0.231837
C -0.646386 3.586535 -0.273982
C -1.384914 4.734945 -0.199398
C -2.782629 4.670266 -0.004463
C -3.410094 3.460598 0.110987
C -2.673629 2.253934 0.037884
C -1.270576 2.322434 -0.155983
H 0.425507 3.624698 -0.422975
H -0.901661 5.698337 -0.288889
H -3.355972 5.585635 0.053022
H -4.481563 3.405977 0.259433
C -3.160427 -1.582669 0.226958
C -4.540181 -1.690794 -0.410737
H -4.864996 -2.727903 -0.335607
H -5.275679 -1.061060 0.091990
H -4.486677 -1.416412 -1.463247
C -3.246454 -1.901188 1.724359
H -3.910594 -1.208187 2.243666
H -3.618761 -2.918848 1.844382
H -2.257727 -1.836707 2.183880
C 1.655510 -0.987960 -0.081806
C 2.795299 -0.583894 -0.018158
C 4.135347 -0.091309 0.069557
C 4.912980 0.059164 -1.082594
C 4.683775 0.247902 1.310192
C 6.210147 0.538293 -0.993289
H 4.487733 -0.204343 -2.041297
C 5.980965 0.727697 1.393919
H 4.081135 0.129635 2.200237
C 6.747136 0.874033 0.243711
H 6.803657 0.650284 -1.890446
H 6.395832 0.987343 2.358363
H 7.759702 1.248038 0.311213

NpthISO2NMe_D_CCPh.log
Energy (E) = -1346.09589297 Hartree
Enthalpy (H) = -1345.791275 Hartree
Gibbs free energy (G) = -1345.868269 Hartree
Charge = 0, Spin = 1
C 0.010347 3.071385 -1.239888
C 1.188125 3.567758 -0.769584
C 2.162498 2.719229 -0.197896
C 1.944437 1.308262 -0.089114
C 0.674131 0.863696 -0.551845
C -0.247286 1.694451 -1.117600
H 3.484829 4.368976 0.163764
H -0.730824 3.713535 -1.693353
H 1.409870 4.625540 -0.831097
C 3.372454 3.296273 0.257997
C 3.026978 0.545488 0.439956
H -1.195528 1.304122 -1.458407
C 4.188848 1.137246 0.853740
C 4.367641 2.529699 0.784157
H 4.981381 0.496374 1.215163
H 5.292488 2.973361 1.124136
I 0.000294 -1.164517 -0.377542
S 3.019102 -1.218216 0.619984
O 2.259628 -1.520154 1.819359
O 4.386255 -1.686574 0.581308
N 2.234274 -1.599588 -0.730033
C 2.383081 -2.996418 -1.130914
H 1.955321 -3.114707 -2.125895
H 3.437528 -3.268136 -1.175856
H 1.883655 -3.698318 -0.449949
C -1.983241 -0.471934 -0.139108
C -3.166974 -0.282000 0.028271
C -4.559999 -0.036404 0.235940
C -5.020245 0.401470 1.481432
C -5.476595 -0.232000 -0.801424
C -6.370356 0.639689 1.681520
H -4.308951 0.549341 2.282206
C -6.825802 0.005997 -0.594888
H -5.118367 -0.572553 -1.763269
C -7.275504 0.442674 0.645457
H -6.717731 0.978147 2.648063
H -7.528078 -0.149307 -1.402470
H -8.329028 0.628066 0.804593

perF_CMe2O_CCPh.log
Energy (E) = -1139.39405850 Hartree
Enthalpy (H) = -1139.135847 Hartree
Gibbs free energy (G) = -1139.212339 Hartree
Charge = 0, Spin = 1
C 1.256063 2.566497 -0.266538
C 2.539477 2.500336 0.244926
C 3.137501 1.265397 0.450237
C 2.475901 0.085905 0.157658
C 1.167379 0.182579 -0.278301
C 0.556822 1.396157 -0.529102
O 2.333689 -2.170351 -0.510538
I 0.233140 -1.722419 -0.423506
F -0.649233 1.517196 -1.063030
F 0.700970 3.741760 -0.517360
F 3.199204 3.615463 0.509660
F 4.371075 1.264767 0.953765
C 3.097992 -1.323245 0.267304
C 4.515377 -1.350278 -0.301878
H 5.229912 -0.813899 0.317339
H 4.808858 -2.397696 -0.359398
H 4.520722 -0.938666 -1.310458
C 3.100824 -1.738773 1.740437
H 3.521457 -2.741171 1.818126
H 3.696149 -1.051911 2.343910
H 2.083070 -1.757327 2.135226
C -1.678086 -0.870145 -0.185200
C -2.830490 -0.550802 -0.009628
C -4.178493 -0.114002 0.178092
C -5.100596 -0.906555 0.866550
C -4.584674 1.123304 -0.330798
C -6.404251 -0.469201 1.038983
H -4.783938 -1.862295 1.261035
C -5.888113 1.557079 -0.150978
H -3.866447 1.733144 -0.861950
C -6.800977 0.762322 0.532214
H -7.112002 -1.089683 1.571622
H -6.193287 2.516410 -0.546123
H -7.818522 1.101986 0.669587

perF_NMeCO2_CCPh.log
Energy (E) = -1229.44219086 Hartree
Enthalpy (H) = -1229.211764 Hartree
Gibbs free energy (G) = -1229.288857 Hartree
Charge = 0, Spin = 1
C 1.057091 2.657387 -0.589549
C 2.184013 2.719542 0.213642
C 2.776686 1.564891 0.698411
C 2.243190 0.295332 0.449943
C 1.082649 0.279001 -0.332037
C 0.517713 1.416585 -0.876539
I 0.226446 -1.581566 -0.707320
F -0.535681 1.348769 -1.678073
F 0.521670 3.761595 -1.085724
F 2.731354 3.892839 0.476762
F 3.909688 1.705496 1.379315
N 2.829119 -0.864212 0.929995
C 3.526852 -0.870123 2.212096
H 3.066725 -0.139419 2.873373
H 3.427225 -1.863313 2.639488
H 4.587349 -0.650927 2.106270
C 3.122484 -1.964248 0.050592
O 3.941838 -2.774171 0.422549
O 2.450180 -1.955855 -1.050806
C -1.655905 -0.889642 -0.225261
C -2.797173 -0.595464 0.036259
C -4.142565 -0.225271 0.343111
C -4.750867 -0.679550 1.516604
C -4.859516 0.596342 -0.531466
C -6.056252 -0.318070 1.806865
H -4.191217 -1.313746 2.190090
C -6.163653 0.956232 -0.233785
H -4.382484 0.945571 -1.436673
C -6.763813 0.499604 0.933686
H -6.522437 -0.673202 2.715499
H -6.713223 1.593040 -0.913113
H -7.782424 0.781129 1.163154

PyIBCMe2O_D_CCPh.log
Energy (E) = -758.646318885 Hartree
Enthalpy (H) = -758.371561 Hartree
Gibbs free energy (G) = -758.439811 Hartree
Charge = 0, Spin = 1
C 1.122168 3.102073 -0.302159
C 2.494241 3.186011 -0.106204
C 2.732883 0.914620 -0.000095
C 1.371875 0.769164 -0.183110
H 0.529496 3.996874 -0.424825
H 2.980505 4.153658 -0.067770
O 2.883218 -1.420164 -0.323434
I 0.754000 -1.250496 -0.147679
C 0.529738 1.845575 -0.327668
H -0.536974 1.714621 -0.452277
N 3.284702 2.125729 0.050020
C 3.578648 -0.336379 0.199299
C 4.905136 -0.199162 -0.534968
H 5.463483 0.663045 -0.173865
H 5.483027 -1.108054 -0.370989
H 4.724518 -0.088270 -1.603468
C 3.813269 -0.499396 1.704199
H 4.410851 -1.395700 1.869744
H 4.332070 0.366557 2.116993
H 2.859815 -0.617209 2.225102
C -1.275960 -0.594763 -0.050617
C -2.464881 -0.368278 -0.006903
C -3.866433 -0.087933 0.051951
C -4.478292 0.207587 1.273888
C -4.641493 -0.105240 -1.111528
C -5.835737 0.480454 1.328090
H -3.877477 0.218694 2.172903
C -5.998761 0.167356 -1.051696
H -4.166884 -0.335318 -2.055543
C -6.598901 0.461077 0.166832
H -6.299844 0.707778 2.278111
H -6.589869 0.150617 -1.957182
H -7.658377 0.673626 0.211484

PyrroleNMeIBCMe2O_A_CCPh.log
Energy (E) = -759.835467406 Hartree
Enthalpy (H) = -759.537245 Hartree
Gibbs free energy (G) = -759.608416 Hartree
Charge = 0, Spin = 1
C 1.571718 2.786504 -0.185501
C 0.553517 1.867706 -0.223401
C 1.206190 0.622491 -0.155132
C 2.554381 0.782595 -0.064971
H 1.540101 3.862136 -0.235698
H -0.502672 2.058543 -0.293928
I 0.569643 -1.341773 -0.052675
O 2.746151 -1.531390 -0.185406
N 2.777409 2.131774 -0.082712
C 4.060844 2.802138 -0.067462
H 4.697020 2.394837 0.715895
H 4.572318 2.701713 -1.024104
H 3.894856 3.857422 0.135354
C 3.490436 -0.396402 0.113083
C 4.683268 -0.347125 -0.840931
H 5.382307 0.455503 -0.599336
H 5.208595 -1.298292 -0.759359
H 4.335531 -0.231368 -1.867071
C 3.971004 -0.455383 1.567088
H 4.605486 -1.333638 1.688775
H 4.536896 0.432493 1.859627
H 3.108563 -0.552824 2.228272
C -1.438588 -0.669601 -0.010756
C -2.608131 -0.360819 0.005852
C -3.988578 0.014400 0.029340
C -4.600996 0.387314 1.229529
C -4.742876 0.013676 -1.147710
C -5.937949 0.751333 1.249412
H -4.016196 0.386698 2.139114
C -6.079772 0.377540 -1.122476
H -4.267706 -0.274839 -2.075220
C -6.680576 0.747299 0.074808
H -6.402087 1.038150 2.183285
H -6.654444 0.372983 -2.038726
H -7.724081 1.031174 0.092478

AnthI8BA_B_naph.log
Energy (E) = -1240.28991265 Hartree
Enthalpy (H) = -1239.853391 Hartree
Gibbs free energy (G) = -1239.937646 Hartree
Charge = 0, Spin = 1
C -7.176468 0.292353 0.001449
C -6.320059 -0.649144 0.478883
C -4.920599 -0.565229 0.216646
C -4.433059 0.533460 -0.563104
C -5.364431 1.499945 -1.045069
C -6.691201 1.384821 -0.772941
C -4.023740 -1.516933 0.695322
C -3.069629 0.627664 -0.828083
C -2.178686 -0.328414 -0.347321
C -2.659577 -1.423140 0.428635
C -1.723644 -2.382222 0.898674
H -2.105621 -3.210749 1.484938
C -0.377153 -2.336939 0.649354
C 0.058354 -1.208468 -0.112533
H -4.395338 -2.348669 1.283444
H -8.235779 0.217332 0.206410
H -6.683466 -1.482608 1.067268
H -4.988196 2.327186 -1.634271
H -7.388948 2.122980 -1.144341
H -2.696569 1.458365 -1.416685
I 2.107824 -0.801305 -0.557657
C -0.781389 -0.256453 -0.593052
H -0.405411 0.583858 -1.163658
C 0.453022 -3.490564 1.165606
H 0.550657 -4.219393 0.358892
H -0.159834 -3.970943 1.928326
C 1.839595 -3.183797 1.756295
H 1.917412 -2.115388 1.969214
H 1.904958 -3.665450 2.731379
C 3.027953 -3.673472 0.904681
H 3.835428 -2.935880 0.924942
H 3.436196 -4.596133 1.309642
C 2.671282 -3.965646 -0.555763
O 2.033386 -3.026009 -1.188415
O 2.958232 -5.046119 -1.037473
C 1.823242 1.220799 0.100847
C 2.185980 2.244352 -0.725959
C 1.344082 1.475209 1.405497
C 2.091665 3.589872 -0.286668
H 2.546616 2.053269 -1.731423
C 1.236544 2.762795 1.847873
H 1.055391 0.648563 2.044508
C 1.607681 3.851945 1.019396
C 2.460002 4.673673 -1.116504
H 0.868030 2.971112 2.844861
C 1.508451 5.192587 1.457037
C 2.354241 5.961534 -0.666394
H 2.828791 4.464750 -2.113177
C 1.873331 6.223741 0.634553
H 1.138527 5.386890 2.456203
H 2.639969 6.785145 -1.306371
H 1.794844 7.245967 0.978698

FuranIBCMe2S_A_naph.log
Energy (E) = -1140.76902786 Hartree
Enthalpy (H) = -1140.477186 Hartree
Gibbs free energy (G) = -1140.546986 Hartree
Charge = 0, Spin = 1
C -1.421393 2.958302 -0.312700
C -0.513236 1.964426 -0.182791
C -1.300549 0.777721 -0.108453
C -2.607002 1.113963 -0.189349
O -2.682575 2.461250 -0.315190
H -1.325975 4.023548 -0.420074
H 0.557862 2.057155 -0.156865
I -0.697355 -1.196268 0.138720
S -3.431686 -1.435949 -0.269368
C -3.869065 0.321022 -0.116280
C -4.553211 0.597380 1.225443
H -4.803057 1.657367 1.321908
H -5.469017 0.009666 1.286413
H -3.904705 0.304028 2.050582
C -4.799897 0.723038 -1.261193
H -5.702982 0.115024 -1.212406
H -5.077969 1.776423 -1.182420
H -4.320288 0.547117 -2.222838
C 1.361260 -0.495121 0.369543
C 2.234779 -0.617877 -0.672400
C 1.780749 0.041198 1.606659
C 3.584000 -0.205045 -0.531949
H 1.915427 -1.025821 -1.625738
C 3.075421 0.452893 1.765441
H 1.075076 0.131324 2.423593
C 4.009016 0.340474 0.705986
C 4.514116 -0.317312 -1.592011
H 3.407908 0.869464 2.708688
C 5.354279 0.755508 0.845887
C 5.808623 0.092624 -1.428282
H 4.180530 -0.734326 -2.534259
C 6.233299 0.634852 -0.195167
H 5.673885 1.170213 1.794012
H 6.513803 0.003062 -2.243353
H 7.259766 0.954841 -0.078310

Indole_NMe_IBCONAc_A_naph.log
Energy (E) = -1118.88214979 Hartree
Enthalpy (H) = -1118.519583 Hartree
Gibbs free energy (G) = -1118.601497 Hartree
Charge = 0, Spin = 1
C -0.308314 1.868256 -0.002916
C -1.284093 2.895846 -0.065922
C -0.945012 4.248434 -0.108857
C 0.393372 4.576079 -0.092487
C 1.377039 3.575899 -0.039047
C 1.047354 2.237786 0.004919
C -1.085522 0.671421 0.017445
H -1.712324 5.009315 -0.155804
H 0.691069 5.615107 -0.125037
H 2.420166 3.861038 -0.034595
H 1.833907 1.500026 0.038140
N -2.539003 2.342499 -0.085867
C -2.419044 0.979927 -0.039492
C -3.550406 -0.004210 -0.069279
O -4.709908 0.373469 -0.055032
I -0.583384 -1.348032 0.119303
C -3.750078 3.140765 -0.172561
H -3.732957 3.734900 -1.086483
H -4.604594 2.475373 -0.176991
H -3.807664 3.812277 0.684250
N -3.040217 -1.261886 -0.099230

C -3.849132 -2.382588 -0.191681
C -3.069010 -3.682544 -0.098342
H -2.524674 -3.736692 0.846799
H -3.760050 -4.517248 -0.164307
H -2.344061 -3.751670 -0.912323
O -5.050342 -2.391812 -0.344977
C 1.512184 -0.981337 0.363122
C 2.342222 -1.067391 -0.718448
C 2.009146 -0.667767 1.646224
C 3.728639 -0.818382 -0.572919
H 1.956826 -1.302047 -1.704135
C 3.343736 -0.418019 1.808185
H 1.333382 -0.606814 2.489369
C 4.234597 -0.481482 0.708969
C 4.617565 -0.884098 -1.672147
H 3.739244 -0.164708 2.783981
C 5.618282 -0.222084 0.852109
C 5.949771 -0.627860 -1.503336
H 4.221544 -1.139605 -2.647040
C 6.455192 -0.293577 -0.226955
H 6.000189 0.033372 1.832651
H 6.623718 -0.679351 -2.347408
H 7.511267 -0.093558 -0.107876

NaphIBMeUreaMe_naph.log
Energy (E) = -1082.96428156 Hartree
Enthalpy (H) = -1082.577307 Hartree
Gibbs free energy (G) = -1082.656648 Hartree
Charge = 0, Spin = 1
C -0.980878 2.603660 -0.638709
C -2.230398 2.781711 0.003082
C -2.922249 1.661197 0.510204
C -2.429309 0.375774 0.412747
C -1.156105 0.248150 -0.205984
C -0.457257 1.293461 -0.720566
H -3.883482 1.824978 0.978302
H 0.515685 1.140458 -1.171799
I -0.277313 -1.663688 -0.244667
C -3.356406 -1.901308 0.192155
O -4.304544 -2.610990 0.504034
N -2.491818 -2.100405 -0.811227
C -2.708858 -3.326932 -1.549974
H -3.700437 -3.337984 -2.005171
H -1.966882 -3.401593 -2.347794
H -2.642154 -4.219479 -0.918375
N -3.111573 -0.712809 0.953216
C -4.099287 -0.444986 1.983699
H -3.685823 0.265394 2.698147
H -5.032603 -0.046355 1.575942
H -4.333436 -1.380460 2.479596
C -2.758934 4.092364 0.100565
C -2.076654 5.159769 -0.414648
C -0.828689 4.976779 -1.051750
C -0.293045 3.723172 -1.160247
H -3.715904 4.231942 0.587999
H -2.492623 6.155166 -0.335353
H -0.300754 5.831579 -1.451610
H 0.663119 3.567898 -1.644700
C 1.644686 -0.782413 0.254237
C 2.710193 -1.016664 -0.566596
C 1.803708 -0.043583 1.448329
C 4.000053 -0.527941 -0.234204
H 2.593704 -1.575644 -1.490179
C 3.033296 0.445935 1.787408
H 0.947465 0.138608 2.086916
C 4.162078 0.216772 0.960845
C 5.123454 -0.755298 -1.062325
H 3.165360 1.015854 2.699221
C 5.445223 0.709868 1.292165
C 6.353887 -0.266459 -0.716749
H 4.991024 -1.324832 -1.974120
C 6.516494 0.474266 0.473923
H 5.563021 1.277615 2.206994
H 7.208306 -0.446253 -1.355005
H 7.494049 0.855209 0.736502

NpthIBCMe2O_C_naph.log
Energy (E) = -973.533085383 Hartree
Enthalpy (H) = -973.160020 Hartree
Gibbs free energy (G) = -973.234061 Hartree
Charge = 0, Spin = 1
C -3.459089 0.894884 -0.010400
C -2.689651 -0.238712 -0.042666
C -1.300797 -0.064053 -0.108023
C -0.680508 1.143651 -0.145416
H -4.538276 0.814337 0.048870
H 0.396561 1.236105 -0.201342
O -2.306318 -2.515771 -0.555478
I -0.225960 -1.905587 -0.135957
C -0.897351 3.607595 -0.179761
C -1.688121 4.723072 -0.157662
C -3.093374 4.597091 -0.083476
C -3.675348 3.360487 -0.034004
C -2.883865 2.186986 -0.054945
C -1.474264 2.316893 -0.126804
H 0.180871 3.693120 -0.236394
H -1.240812 5.707036 -0.196715
H -3.708426 5.486719 -0.066748
H -4.752245 3.258373 0.020818
C -3.232523 -1.671346 0.019612
C -4.544481 -1.793499 -0.750625
H -4.840238 -2.841985 -0.738337
H -5.346775 -1.202846 -0.304965
H -4.397826 -1.484730 -1.784840
C -3.461825 -2.025064 1.496249
H -4.195414 -1.363945 1.961982
H -3.812681 -3.055720 1.555942
H -2.525309 -1.946273 2.053045
C 1.630216 -0.853446 0.255275
C 2.552336 -0.714573 -0.743925
C 1.915184 -0.381454 1.557647
C 3.806784 -0.101983 -0.495341
H 2.341636 -1.066638 -1.748757
C 3.113222 0.220449 1.824109
H 1.175886 -0.493956 2.341836
C 4.090112 0.374841 0.809358
C 4.778849 0.052085 -1.511465
H 3.336439 0.586176 2.819247
C 5.339467 0.989668 1.058081
C 5.978851 0.651248 -1.242648
H 4.553786 -0.314458 -2.505544
C 6.262290 1.125094 0.057146
H 5.550822 1.350603 2.057233
H 6.716822 0.764406 -2.025194
H 7.214921 1.596446 0.257494

NpthISO2NMe_D_naph.log
Energy (E) = -1423.51507453 Hartree
Enthalpy (H) = -1423.173525 Hartree
Gibbs free energy (G) = -1423.250066 Hartree
Charge = 0, Spin = 1
C 0.029466 2.824700 -1.760220
C 1.111138 3.452369 -1.220981
C 2.064435 2.740127 -0.459715
C 1.926603 1.333912 -0.223002
C 0.744880 0.750128 -0.762343
C -0.152751 1.452699 -1.514595
H 3.228811 4.514192 -0.143750
H -0.691941 3.359927 -2.360775
H 1.272697 4.511898 -1.373482
C 3.176173 3.451008 0.054276
C 2.992152 0.710624 0.492408
H -1.026376 0.964544 -1.921765
C 4.058781 1.429267 0.958101
C 4.153594 2.817880 0.759729
H 4.845041 0.887993 1.466289
H 5.004585 3.361916 1.144346
I 0.154269 -1.288730 -0.447144
S 3.078866 -1.023968 0.860780
O 2.169916 -1.252982 1.974592
O 4.465477 -1.366989 1.098285
N 2.542553 -1.594851 -0.529465
C 2.768147 -3.025858 -0.700193
H 2.521614 -3.293968 -1.727250
H 3.813684 -3.281523 -0.522300
H 2.156644 -3.643462 -0.025776
C -1.924802 -0.720685 -0.386930
C -2.426915 -0.155520 0.751695
C -2.766814 -1.019943 -1.480047
C -3.803261 0.159207 0.847431
H -1.780956 0.062515 1.595404
C -4.102689 -0.729960 -1.408450
H -2.353936 -1.475907 -2.371843
C -4.654125 -0.132844 -0.250045
C -4.353750 0.752236 2.008998
H -4.758536 -0.953673 -2.241090
C -6.031000 0.179355 -0.151739
C -5.687841 1.042948 2.074840
H -3.697011 0.969270 2.842141
C -6.535807 0.753223 0.981994
H -6.676214 -0.046555 -0.991775
H -6.101380 1.495897 2.965534
H -7.589538 0.987580 1.048215

perF_CMe2O_naph.log
Energy (E) = -1216.81380354 Hartree
Enthalpy (H) = -1216.518743 Hartree
Gibbs free energy (G) = -1216.594962 Hartree
Charge = 0, Spin = 1
C 1.320517 2.517323 -0.645681
C 2.565865 2.545972 -0.044540
C 3.160498 1.361117 0.363191
C 2.537953 0.137533 0.183772
C 1.262621 0.146753 -0.347787
C 0.659090 1.306744 -0.793081
O 2.475206 -2.199346 -0.144910
I 0.316836 -1.770953 -0.304725
F -0.516893 1.330144 -1.409598
F 0.767894 3.637622 -1.085900
F 3.191931 3.700811 0.115713
F 4.354552 1.456153 0.948677
C 3.179832 -1.230459 0.522929
C 4.626436 -1.287903 0.027735
H 5.296354 -0.638757 0.586422
H 4.952407 -2.321040 0.142373
H 4.665987 -1.034456 -1.031196
C 3.122686 -1.420349 2.042503
H 3.561905 -2.387589 2.286495
H 3.668687 -0.634489 2.567247
H 2.085127 -1.416385 2.383521
C -1.665723 -0.916897 -0.358514
C -2.195339 -0.399448 0.788911
C -2.438482 -1.001268 -1.536855
C -3.526589 0.083431 0.813264
H -1.608421 -0.344754 1.700362
C -3.729225 -0.551526 -1.536701

H -2.004725 -1.410574 -2.440590
C -4.305455 0.004856 -0.368961
C -4.101289 0.635714 1.982294
H -4.330579 -0.608181 -2.435962
C -5.636574 0.483257 -0.345445
C -5.390615 1.091507 1.975488
H -3.499669 0.691095 2.881251
C -6.166938 1.014397 0.797947
H -6.226623 0.421170 -1.251545
H -5.822067 1.513659 2.872879
H -7.185309 1.378477 0.805581

perF_NMeCO2_naph.log
Energy (E) = -1306.86629907 Hartree
Enthalpy (H) = -1306.599029 Hartree
Gibbs free energy (G) = -1306.675975 Hartree
Charge = 0, Spin = 1
C 1.255713 2.582108 -0.935897
C 2.268893 2.713906 -0.000290
C 2.762285 1.608992 0.673524
C 2.237275 0.323729 0.487353
C 1.187188 0.239375 -0.436712
C 0.728733 1.323972 -1.159608
I 0.296925 -1.621453 -0.722166
F -0.222154 1.184603 -2.077493
F 0.815555 3.634945 -1.608578
F 2.806909 3.902520 0.212688
F 3.799716 1.808289 1.481381
N 2.725377 -0.783035 1.154214
C 3.249918 -0.663439 2.509909
H 2.725679 0.135910 3.029978
H 3.074624 -1.607329 3.017327
H 4.320210 -0.466420 2.525058
C 3.121908 -1.973107 0.422720
O 3.845559 -2.750948 1.010151
O 2.637391 -2.046485 -0.758195
C -1.679363 -0.846635 -0.486643
C -2.120264 -0.551358 0.771859
C -2.517947 -0.713215 -1.612751
C -3.442382 -0.085690 0.968594
H -1.473216 -0.666734 1.634373
C -3.801904 -0.276771 -1.441727
H -2.141022 -0.938989 -2.601301
C -4.296275 0.049497 -0.155741
C -3.933730 0.240004 2.254886
H -4.458927 -0.165466 -2.295248
C -5.619416 0.509289 0.041811
C -5.217409 0.682417 2.414882
H -3.274617 0.132183 3.107251
C -6.068931 0.818461 1.295859
H -6.267895 0.611977 -0.819232
H -5.587213 0.930503 3.400230
H -7.081894 1.169688 1.437071

PyIBCMe2O_D_naph.log
Energy (E) = -836.062284955 Hartree
Enthalpy (H) = -835.750615 Hartree
Gibbs free energy (G) = -835.818631 Hartree
Charge = 0, Spin = 1
C 1.178103 3.076085 -0.550833
C 2.516746 3.204897 -0.207540
C 2.782067 0.946507 0.060084
C 1.451982 0.753489 -0.261179
H 0.585965 3.946313 -0.794227
H 2.979057 4.184553 -0.172838
O 3.029621 -1.398800 -0.075115
I 0.835013 -1.276678 -0.174060
C 0.617201 1.805211 -0.564399
H -0.427046 1.657355 -0.803577
N 3.300821 2.174148 0.098806
C 3.637468 -0.263067 0.422746
C 5.026487 -0.118841 -0.190310
H 5.523858 0.783335 0.162805
H 5.611200 -0.995545 0.086800
H 4.945857 -0.084048 -1.276372
C 3.735909 -0.304845 1.953146
H 4.341380 -1.165231 2.238365
H 4.186578 0.606803 2.348124
H 2.740580 -0.423558 2.389024
C -1.240561 -0.660672 -0.365226
C -1.984817 -0.408718 0.754054
C -1.832269 -0.572819 -1.646109
C -3.352274 -0.052866 0.652997
H -1.541046 -0.472223 1.742498
C -3.150298 -0.227763 -1.774018
H -1.236413 -0.776276 -2.527928
C -3.943713 0.040948 -0.632647
C -4.142640 0.213952 1.796231
H -3.608192 -0.156970 -2.753407
C -5.308984 0.398702 -0.734943
C -5.459682 0.558167 1.667233
H -3.683070 0.139750 2.774179
C -6.049468 0.651482 0.386720
H -5.755402 0.467487 -1.719383
H -6.056858 0.760474 2.545956
H -7.092388 0.924006 0.298417

PyrroleNMeIBCMe2O_A_naph.log
Energy (E) = -837.253493870 Hartree
Enthalpy (H) = -836.918378 Hartree
Gibbs free energy (G) = -836.989183 Hartree
Charge = 0, Spin = 1
C 1.739041 2.785310 -0.036227
C 0.714047 1.888332 -0.197814
C 1.337120 0.624668 -0.131393
C 2.676263 0.756221 0.074580
H 1.728174 3.862634 -0.036304
H -0.328586 2.112832 -0.340678
I 0.617739 -1.319283 -0.202175
O 2.857424 -1.559320 -0.067345
N 2.919892 2.102393 0.134006
C 4.207324 2.751709 0.267269
H 4.792450 2.285828 1.056890
H 4.773004 2.705554 -0.662763
H 4.041041 3.794309 0.528225
C 3.592782 -0.439935 0.268544
C 4.823956 -0.368941 -0.638933
H 5.510578 0.434916 -0.365474
H 5.350568 -1.318855 -0.548953
H 4.512570 -0.244422 -1.675948
C 4.023433 -0.524010 1.739092
H 4.637142 -1.416581 1.864240
H 4.595959 0.345913 2.070858
H 3.136240 -0.621375 2.366138
C -1.399017 -0.561047 -0.380371
C -2.237000 -0.577622 0.698522
C -1.847538 -0.063878 -1.625111
C -3.568227 -0.103792 0.589491
H -1.898828 -0.948563 1.660377
C -3.124923 0.406586 -1.756690
H -1.171843 -0.051325 -2.471819
C -4.018464 0.397346 -0.657965
C -4.457464 -0.112253 1.689968
H -3.474399 0.791439 -2.707289
C -5.346579 0.872907 -0.766444
C -5.736206 0.354191 1.556418
H -4.104725 -0.495717 2.639585
C -6.185875 0.852216 0.313453
H -5.685140 1.253536 -1.722385
H -6.409529 0.343667 2.402808
H -7.199484 1.217984 0.220155

AnthI8BA_B_OCH2CF3.log
Energy (E) = -1307.30991602 Hartree
Enthalpy (H) = -1306.964546 Hartree
Gibbs free energy (G) = -1307.044356 Hartree
Charge = 0, Spin = 1
C -6.730174 -1.446179 0.063764
C -5.610684 -2.049940 0.543718
C -4.315784 -1.538433 0.235405
C -4.217459 -0.373903 -0.593481
C -5.415853 0.230561 -1.075017
C -6.631948 -0.287322 -0.758140
C -3.154501 -2.135633 0.720739
C -2.960216 0.136359 -0.904681
C -1.803920 -0.470758 -0.422353
C -1.897010 -1.624344 0.410442
C -0.695597 -2.202575 0.902924
H -0.779648 -3.071475 1.546156
C 0.555388 -1.725510 0.620707
C 0.593897 -0.569535 -0.218731
H -3.232749 -3.013520 1.352271
H -7.706909 -1.843361 0.304577
H -5.680159 -2.930851 1.169801
H -5.332073 1.111764 -1.698714
H -7.535036 0.178190 -1.128539
H -2.878138 1.023460 -1.522240
I 2.394417 0.435635 -0.757319
C -0.510750 0.034038 -0.724092
H -0.429997 0.922843 -1.335134
C 1.740219 -2.450722 1.210798
H 2.184471 -3.088874 0.444832
H 1.333924 -3.131060 1.957883
C 2.820542 -1.566333 1.863617
H 2.436357 -0.550184 1.973069
H 2.985236 -1.915920 2.881281
C 4.183634 -1.561778 1.140867
H 4.612946 -0.556956 1.146438
H 4.892152 -2.211006 1.647722
C 4.117820 -2.070172 -0.289447
O 3.250130 -1.484252 -1.104792
O 4.789038 -3.002955 -0.657822
O 1.346936 2.216439 -0.482059
C 0.974965 2.520566 0.819258
H 1.779653 2.984080 1.400681
H 0.594533 1.652192 1.376115
C -0.167591 3.514809 0.770945
F 0.176641 4.644459 0.156690
F -1.228574 3.012454 0.127171
F -0.557775 3.825197 2.013435

FuranIBCMe2S_A_OCH2CF3.log
Energy (E) = -1207.79480305 Hartree
Enthalpy (H) = -1207.594209 Hartree
Gibbs free energy (G) = -1207.658834 Hartree
Charge = 0, Spin = 1
C -0.693768 2.951320 0.138820
C 0.194650 1.931959 0.204959
C -0.609615 0.767866 0.053975
C -1.899773 1.139969 -0.062032
O -1.964062 2.486756 -0.017250
H -0.580748 4.019255 0.176546
H 1.258388 1.985627 0.350066
I -0.161376 -1.244662 0.141940
S -2.631175 -1.371789 -0.556415

C -3.138787 0.311777 -0.063977
C -3.748184 0.308610 1.338214
H -4.022302 1.324065 1.632113
H -4.640505 -0.316758 1.349547
H -3.037551 -0.089115 2.062931
C -4.147589 0.825538 -1.087439
H -5.026810 0.181609 -1.093651
H -4.459424 1.836465 -0.819880
H -3.715181 0.837563 -2.086155
O 1.837974 -0.585788 0.571221
C 2.607893 -0.381339 -0.559408
H 2.129760 0.270183 -1.307544
H 2.910497 -1.307550 -1.064691
C 3.889189 0.319991 -0.157066
F 3.644254 1.513059 0.402470
F 4.602291 -0.391259 0.712395
F 4.655700 0.535143 -1.235703

Indole_NMe_IBCONAc_A_OCH2CF3.log
Energy (E) = -1185.89993846 Hartree
Enthalpy (H) = -1185.628459 Hartree
Gibbs free energy (G) = -1185.705728 Hartree
Charge = 0, Spin = 1
C -0.380775 1.839031 0.259065
C 0.458749 2.948354 -0.019265
C -0.020513 4.259093 0.005115
C -1.348471 4.445548 0.322624
C -2.188070 3.357658 0.622485
C -1.724002 2.061102 0.598658
C 0.486833 0.723268 0.125616
H 0.631330 5.094237 -0.213165
H -1.752331 5.448212 0.351350
H -3.220124 3.545186 0.883895
H -2.366860 1.233472 0.858740
N 1.736350 2.512405 -0.278587
C 1.746262 1.149129 -0.179056
C 2.896614 0.228337 -0.309849
O 4.012371 0.591736 -0.611228
I 0.289395 -1.316228 0.304885
C 2.851638 3.394098 -0.572606
H 3.008232 4.082516 0.257617
H 3.740925 2.793062 -0.722442
H 2.641071 3.968596 -1.474384
N 2.474004 -1.063403 -0.031519
C 3.347107 -2.155597 -0.012896
C 2.668751 -3.487623 0.229686
H 1.927771 -3.697298 -0.544481
H 3.426638 -4.263856 0.212370
H 2.170141 -3.501860 1.200896
O 4.539527 -2.073712 -0.164555
O -1.773184 -1.091246 0.499694
C -2.456614 -1.003544 -0.708780
H -2.194465 -1.796939 -1.418954
H -2.322482 -0.034363 -1.207176
C -3.938382 -1.152686 -0.430556
F -4.222464 -2.329596 0.124782
F -4.386745 -0.198485 0.389705
F -4.626875 -1.061377 -1.575058

NaphIBMeUreaMe_OCH2CF3.log
Energy (E) = -1149.98899929 Hartree
Enthalpy (H) = -1149.692940 Hartree
Gibbs free energy (G) = -1149.768720 Hartree
Charge = 0, Spin = 1
C 0.379211 2.557759 -0.312691
C -0.898132 3.085869 -0.007534
C -1.963286 2.207262 0.289694
C -1.808558 0.839260 0.295043
C -0.514036 0.356066 -0.023887
C 0.543791 1.154709 -0.310813
H -2.929512 2.634374 0.521893
H 1.520062 0.733925 -0.499297
I -0.259818 -1.722332 0.033082
C -3.228621 -1.139296 -0.075548
O -4.374917 -1.551764 -0.055546
N -2.226438 -1.687015 -0.814863
C -2.589166 -2.849997 -1.604853
H -3.475499 -2.626569 -2.195214
H -1.768405 -3.089537 -2.281353
H -2.818411 -3.724585 -0.989831
N -2.859148 -0.010208 0.677319
C -3.930643 0.555468 1.482433
H -3.494666 1.180281 2.259060
H -4.629342 1.146933 0.886212
H -4.488001 -0.259967 1.930585
C -1.067538 4.491441 -0.009667
C -0.015461 5.317142 -0.296183
C 1.260411 4.784898 -0.589340
C 1.454103 3.431386 -0.596128
H -2.043847 4.899803 0.219987
H -0.156290 6.389585 -0.295128
H 2.082202 5.453683 -0.804816
H 2.426747 3.005571 -0.809531
O 1.726861 -1.353911 0.726657
C 2.737210 -1.709069 -0.146894
H 2.516420 -1.485725 -1.202226
H 3.022185 -2.767120 -0.084842
C 3.989841 -0.922371 0.186370
F 3.798271 0.399032 0.047045
F 4.406111 -1.135754 1.430745
F 4.984006 -1.268198 -0.643893

NpthIBCMe2O_C_OCH2CF3.log
Energy (E) = -1040.55972357 Hartree
Enthalpy (H) = -1040.277539 Hartree
Gibbs free energy (G) = -1040.347783 Hartree
Charge = 0, Spin = 1
C -2.937631 0.499612 -0.156199
C -2.023798 -0.518178 -0.107807
C -0.674285 -0.161616 0.028474
C -0.217565 1.108616 0.157886
H -3.994572 0.278471 -0.250910
H 0.828695 1.322288 0.322241
O -1.229461 -2.712179 -0.536322
I 0.569951 -1.861021 0.057912
C -0.758745 3.516428 0.204398
C -1.684959 4.520431 0.140905
C -3.053960 4.216068 -0.028784
C -3.468196 2.916499 -0.130595
C -2.534696 1.854728 -0.069257
C -1.161047 2.164230 0.100844
H 0.294058 3.734953 0.333202
H -1.373021 5.552775 0.220779
H -3.776321 5.019602 -0.077074
H -4.517065 2.679440 -0.259479
C -2.355341 -2.004819 -0.086474
C -3.500762 -2.352185 -1.024536
H -3.639474 -3.432450 -1.013774
H -4.432052 -1.881687 -0.707585
H -3.263442 -2.037206 -2.039208
C -2.696499 -2.406230 1.349628
H -3.575565 -1.869530 1.708457
H -2.888805 -3.478674 1.381671
H -1.863296 -2.179480 2.018084
O 2.181383 -0.582581 0.528108
C 2.886757 -0.128170 -0.575261
H 2.246417 0.296080 -1.362535
H 3.523899 -0.893549 -1.034585
C 3.810665 0.989572 -0.137284
F 3.126476 2.024457 0.372592
F 4.681177 0.592007 0.786746
F 4.506582 1.449230 -1.185843

NpthISO2NMe_D_OCH2CF3.log
Energy (E) = -1490.53676493 Hartree
Enthalpy (H) = -1490.286264 Hartree
Gibbs free energy (G) = -1490.359979 Hartree
Charge = 0, Spin = 1
C -0.539815 3.184234 -0.099010
C 0.751631 3.608820 -0.174525
C 1.821701 2.688352 -0.135838
C 1.583106 1.281663 -0.029220
C 0.211021 0.900726 -0.008532
C -0.808174 1.806083 -0.019065
H 3.270967 4.262356 -0.276557
H -1.364957 3.881391 -0.107833
H 0.988803 4.662035 -0.252414
C 3.145560 3.190480 -0.190705
C 2.747498 0.463239 0.057503
H -1.829786 1.470579 0.062942
C 4.011993 0.978645 -0.003390
C 4.224978 2.362180 -0.135908
H 4.845268 0.291879 0.051459
H 5.231224 2.752600 -0.184091
I -0.454906 -1.145274 0.022702
S 2.676461 -1.275621 0.332668
O 2.231617 -1.480118 1.692084
O 3.919890 -1.875990 -0.082492
N 1.515073 -1.645498 -0.745744
C 1.536870 -3.014294 -1.260429
H 0.849219 -3.071491 -2.103288
H 2.538961 -3.247326 -1.614578
H 1.252316 -3.763469 -0.514101
O -2.394656 -0.483217 0.585965
C -3.322762 -0.506195 -0.444290
H -3.670852 -1.514296 -0.698523
H -2.968714 -0.028087 -1.369530
C -4.548569 0.274734 -0.015684
F -5.118696 -0.234268 1.071832
F -4.243910 1.553609 0.246754
F -5.460320 0.273369 -0.997373

perF_CMe2O_OCH2CF3.log
Energy (E) = -1283.83568492 Hartree
Enthalpy (H) = -1283.631425 Hartree
Gibbs free energy (G) = -1283.703621 Hartree
Charge = 0, Spin = 1
C 1.019614 2.511276 -0.426141
C 2.293881 2.339408 0.082444
C 2.764937 1.060818 0.346954
C 1.983944 -0.053594 0.106093
C 0.691299 0.148937 -0.350193
C 0.201125 1.407709 -0.640850
O 1.601191 -2.312918 -0.440054
I -0.370972 -1.678665 -0.464519
F -0.997187 1.635791 -1.146638
F 0.580506 3.724207 -0.712654
F 3.062829 3.391294 0.299783
F 3.990966 0.953737 0.854994
C 2.436209 -1.502481 0.333892
C 3.851892 -1.760936 -0.167795
H 4.603467 -1.285891 0.456970
H 4.005066 -2.839104 -0.149773
H 3.957314 -1.415526 -1.195052

C 2.318610 -1.827807 1.821887
H 2.609934 -2.865745 1.980257
H 2.967757 -1.179460 2.411402
H 1.291465 -1.695612 2.166938
O -2.180471 -0.667201 -0.461872
C -2.549279 -0.115497 0.754864
H -1.864916 0.672232 1.099771
H -2.652645 -0.856176 1.557802
C -3.903959 0.544293 0.593944
F -3.878434 1.518925 -0.311801
F -4.839299 -0.331538 0.227956
F -4.282861 1.081220 1.763476

perF_NMeCO2_OCH2CF3.log
Energy (E) = -1373.87782153 Hartree
Enthalpy (H) = -1373.701621 Hartree
Gibbs free energy (G) = -1373.774151 Hartree
Charge = 0, Spin = 1
C 2.099694 1.609649 -0.982285
C 2.924083 0.879789 -0.142562
C 2.491734 -0.310808 0.422786
C 1.199308 -0.789838 0.212458
C 0.388063 -0.012932 -0.615014
C 0.820776 1.141772 -1.234842
I -1.532465 -0.713969 -0.914419
F 0.049112 1.810666 -2.071354
F 2.536874 2.721121 -1.548930
F 4.155491 1.297579 0.085741
F 3.367363 -0.997743 1.149007
N 0.737203 -1.966463 0.796986
C 1.191972 -2.359107 2.128749
H 1.399599 -1.461493 2.706273
H 0.391958 -2.920026 2.602232
H 2.078903 -2.987670 2.092467
C 0.080236 -2.964579 0.043628
O 0.018661 -4.091690 0.466424
O -0.430501 -2.547814 -1.096959
O -2.248984 1.197687 -0.609018
C -2.695412 1.521620 0.666247
H -3.288718 0.734243 1.146572
H -3.314214 2.417672 0.594379
C -1.563643 1.849395 1.625607
F -0.886074 0.742258 1.986265
F -0.680855 2.692218 1.092029
F -2.046533 2.396970 2.741819

PyIBCMe2O_D_OCH2CF3.log
Energy (E) = -903.088994891 Hartree
Enthalpy (H) = -902.868294 Hartree
Gibbs free energy (G) = -902.932303 Hartree
Charge = 0, Spin = 1
C 0.526931 3.071764 0.167419
C 1.904900 3.164211 0.021236
C 2.155160 0.898963 -0.087421
C 0.788026 0.743950 0.034900
H -0.073518 3.963540 0.271158
H 2.387170 4.134111 0.005655
O 2.213549 -1.427639 -0.533095
I 0.229922 -1.276771 0.060146
C -0.062873 1.813563 0.192824
H -1.122729 1.676494 0.349823
N 2.709334 2.107357 -0.088235
C 3.017529 -0.351282 -0.122024
C 4.150347 -0.204235 -1.123222
H 4.773611 0.650138 -0.864538
H 4.749559 -1.113700 -1.111566
H 3.745450 -0.061908 -2.124020
C 3.559225 -0.584458 1.287670
H 4.171987 -1.485440 1.287518
H 4.159867 0.264827 1.613634
H 2.737200 -0.720520 1.994439
O -1.721654 -0.626550 0.534874
C -2.545875 -0.454462 -0.566239
H -2.863389 -1.396349 -1.029490
H -2.103985 0.176174 -1.352169
C -3.809006 0.252924 -0.120819
F -4.476014 -0.437376 0.799741
F -3.537965 1.459662 0.396484
F -4.625328 0.439701 -1.166308

PyrroleNMeIBCMe2O_A_OCH2CF3.log
Energy (E) = -904.276856407 Hartree
Enthalpy (H) = -904.032653 Hartree
Gibbs free energy (G) = -904.099034 Hartree
Charge = 0, Spin = 1
C 1.070552 2.737064 0.246407
C 0.026462 1.846675 0.279596
C 0.632094 0.594829 0.060611
C 1.980065 0.725300 -0.069443
H 1.070745 3.810182 0.338635
H -1.013863 2.058959 0.450070
I 0.008263 -1.364976 0.075139
O 2.035259 -1.570451 -0.466127
N 2.251230 2.059004 0.045438
C 3.545738 2.696653 -0.076574
H 4.279924 2.200668 0.556063
H 3.899309 2.681002 -1.106800
H 3.452104 3.729699 0.248444
C 2.874714 -0.489453 -0.141872
C 3.927825 -0.403523 -1.240787
H 4.688246 0.346015 -1.017695
H 4.414851 -1.374840 -1.318924
H 3.456731 -0.172040 -2.195020
C 3.525950 -0.735572 1.219614
H 4.126969 -1.643337 1.166468
H 4.166417 0.094950 1.523559
H 2.751421 -0.869340 1.976481
O -1.912987 -0.648875 0.512528
C -2.626273 -0.234662 -0.601239
H -3.005975 -1.062952 -1.212070
H -2.058506 0.441415 -1.256892
C -3.837192 0.547447 -0.137729
F -4.641351 -0.179933 0.634782
F -3.488576 1.637548 0.556031
F -4.550374 0.957537 -1.196972

AnthI8BA_B_OEt.log
Energy (E) = -1009.67588915 Hartree
Enthalpy (H) = -1009.309830 Hartree
Gibbs free energy (G) = -1009.385214 Hartree
Charge = 0, Spin = 1
C -6.855595 -0.790069 0.049706
C -5.756860 -1.489137 0.439655
C -4.445299 -0.982408 0.200924
C -4.307586 0.281100 -0.460279
C -5.485258 0.982803 -0.853260
C -6.718155 0.466117 -0.607227
C -3.304488 -1.679409 0.591610
C -3.033479 0.787961 -0.700437
C -1.896712 0.085303 -0.308628
C -2.029836 -1.172530 0.349890
C -0.849481 -1.860735 0.739726
H -0.964369 -2.809354 1.252404
C 0.417969 -1.398634 0.509365
C 0.499183 -0.133129 -0.149057
H -3.411716 -2.635649 1.091483
H -7.845096 -1.184955 0.236033
H -5.855655 -2.445106 0.938908
H -5.372451 1.936901 -1.353138
H -7.604896 1.006173 -0.909908
H -2.924144 1.743776 -1.200425
I 2.329629 0.867565 -0.582181
C -0.585626 0.581902 -0.539790
H -0.469335 1.548749 -1.011816
C 1.573601 -2.254784 0.968645
H 1.962983 -2.810115 0.113416
H 1.145027 -3.003474 1.633799
C 2.719112 -1.525551 1.696175
H 2.395791 -0.515828 1.957999
H 2.892387 -2.021610 2.649826
C 4.057813 -1.494047 0.930577
H 4.543680 -0.523574 1.058353
H 4.742183 -2.244301 1.317201
C 3.919343 -1.791360 -0.555471
O 3.057644 -1.059275 -1.237605
O 4.537793 -2.694166 -1.070140
O 1.408146 2.604277 0.012407
C 1.228290 2.705036 1.407495
H 2.198104 2.721475 1.921769
H 0.673182 1.834233 1.786772
C 0.455232 3.972640 1.697091
H -0.519350 3.933581 1.211273
H 0.305985 4.097954 2.769679
H 0.995714 4.837086 1.314377

FuranIBCMe2S_A_OEt.log
Energy (E) = -910.159067695 Hartree
Enthalpy (H) = -909.937825 Hartree
Gibbs free energy (G) = -909.997296 Hartree
Charge = 0, Spin = 1
C -0.469540 2.995619 0.126000
C 0.558346 2.118039 0.196361
C -0.062946 0.844654 0.062281
C -1.393832 1.021224 -0.053617
O -1.656117 2.346570 -0.021178
H -0.516161 4.068855 0.155490
H 1.604394 2.324186 0.333976
I 0.717805 -1.062494 0.157847
S -1.759301 -1.588408 -0.465401
C -2.505356 0.028642 -0.067413
C -3.170382 -0.004657 1.309525
H -3.595231 0.972864 1.548898
H -3.967593 -0.747727 1.312128
H -2.444956 -0.274347 2.076874
C -3.531277 0.372744 -1.144788
H -4.316024 -0.383433 -1.158032
H -3.984648 1.342104 -0.929463
H -3.063265 0.406124 -2.126938
O 2.563097 -0.106939 0.523777
C 3.248836 0.229280 -0.656332
H 2.598283 0.788952 -1.347053
H 3.581049 -0.668457 -1.194197
C 4.446207 1.078821 -0.282880
H 5.092942 0.529353 0.399589
H 5.020650 1.355823 -1.167599
H 4.119518 1.989186 0.221223

Indole_NMe_IBCONAc_A_OEt.log
Energy (E) = -888.267399799 Hartree
Enthalpy (H) = -887.975212 Hartree
Gibbs free energy (G) = -888.046620 Hartree
Charge = 0, Spin = 1
C -1.885478 -0.365568 -0.197045

C -2.179740 -1.738422 0.002772
C -3.488430 -2.223008 -0.024096
C -4.498893 -1.317080 -0.265173
C -4.221909 0.043954 -0.485609
C -2.933139 0.529894 -0.456821
C -0.470744 -0.304708 -0.090388
H -3.696596 -3.272707 0.133841
H -5.524238 -1.659436 -0.294339
H -5.040468 0.720680 -0.688589
H -2.718594 1.570151 -0.652243
N -1.013482 -2.438929 0.195370
C 0.028585 -1.554992 0.127814
C 1.479430 -1.844921 0.215061
O 1.919487 -2.952090 0.445385
I 0.929582 1.198192 -0.219120
C -0.955169 -3.874406 0.402689
H -1.395087 -4.390230 -0.450801
H 0.082235 -4.167705 0.513639
H -1.510322 -4.141229 1.301900
N 2.182581 -0.676547 -0.006321
C 3.576357 -0.636131 -0.038669
C 4.148469 0.756953 -0.207760
H 3.836598 1.409355 0.610380
H 5.231158 0.684403 -0.210643
H 3.819952 1.201820 -1.149198
O 4.294496 -1.601667 0.048049
O -0.559945 2.612617 -0.304350
C -1.055061 2.980720 0.968800
H -0.239452 3.313053 1.622951
H -1.534028 2.120660 1.456988
C -2.056405 4.098964 0.780638
H -2.886212 3.765425 0.157825
H -2.454246 4.423983 1.742068
H -1.581781 4.947560 0.290277

NaphIBMeUreaMe_OEt.log
Energy (E) = -852.353115011 Hartree
Enthalpy (H) = -852.036458 Hartree
Gibbs free energy (G) = -852.107529 Hartree
Charge = 0, Spin = 1
C -2.637271 0.347048 -0.263257
C -2.856483 -1.027562 -0.006107
C -1.756508 -1.870735 0.260722
C -0.459254 -1.407595 0.277052
C -0.283790 -0.024832 0.009274
C -1.309303 0.829128 -0.239566
H -1.950571 -2.916435 0.457150
H -1.121059 1.883838 -0.385387
I 1.674345 0.715210 0.091575
C 1.790407 -2.326956 -0.134395
O 2.449656 -3.353496 -0.143397
N 2.087611 -1.205333 -0.833084
C 3.300802 -1.265789 -1.626078
H 3.278817 -2.143545 -2.269838
H 3.360357 -0.374754 -2.252292
H 4.204115 -1.335539 -1.013079
N 0.604937 -2.250517 0.627478
C 0.297122 -3.452676 1.385073
H -0.408178 -3.201330 2.174731
H -0.122872 -4.243447 0.758549
H 1.218696 -3.829306 1.815393
C -4.184846 -1.517893 -0.026360
C -5.232624 -0.678930 -0.287820
C -5.009847 0.693772 -0.538631
C -3.737859 1.195223 -0.525087
H -4.354630 -2.569623 0.168138
H -6.242886 -1.065130 -0.301734
H -5.849820 1.344310 -0.739799
H -3.552439 2.245377 -0.713726
O 0.864416 2.499413 0.835455
C 0.954079 3.572956 -0.066686
H 0.493546 3.321932 -1.035934
H 2.000260 3.832219 -0.281477
C 0.248091 4.766317 0.542542
H 0.714030 5.027515 1.491495
H 0.292538 5.629830 -0.122088
H -0.796923 4.522699 0.735147

NpthIBCMe2O_C_OEt.log
Energy (E) = -742.924175761 Hartree
Enthalpy (H) = -742.621416 Hartree
Gibbs free energy (G) = -742.686067 Hartree
Charge = 0, Spin = 1
C 1.922589 1.559742 -0.142715
C 0.564879 1.391546 -0.096976
C 0.082221 0.080802 0.026498
C 0.866639 -1.020560 0.140909
H 2.345961 2.554049 -0.227269
H 0.437013 -2.002026 0.289726
O -1.687088 2.004910 -0.514034
I -2.022846 0.014412 0.066336
C 3.154904 -1.953598 0.178216
C 4.508228 -1.766816 0.119306
C 5.040497 -0.467347 -0.034250
C 4.208982 0.614756 -0.124300
C 2.803953 0.454173 -0.066886
C 2.272211 -0.851832 0.086663
H 2.736002 -2.945315 0.295888
H 5.177239 -2.613545 0.190692
H 6.112652 -0.332059 -0.079328
H 4.612483 1.613109 -0.240725
C -0.464157 2.516651 -0.071289
C -0.085512 3.656969 -1.005344
H -0.889494 4.391809 -0.991659
H 0.836068 4.147401 -0.689360
H 0.032879 3.284325 -2.021499
C -0.586148 3.023805 1.368134
H 0.360481 3.431921 1.725327
H -1.352024 3.798700 1.405276
H -0.880183 2.209634 2.033911
O -1.882084 -2.009143 0.516749
C -1.904466 -2.840011 -0.620524
H -1.169068 -2.505296 -1.368008
H -2.886244 -2.812018 -1.109974
C -1.584756 -4.253733 -0.182791
H -2.310470 -4.586708 0.557774
H -1.604786 -4.939838 -1.030070
H -0.594695 -4.291423 0.273841

NpthISO2NMe_D_OEt.log
Energy (E) = -1192.90680477 Hartree
Enthalpy (H) = -1192.635542 Hartree
Gibbs free energy (G) = -1192.703272 Hartree
Charge = 0, Spin = 1
C 0.614738 3.310525 0.874520
C -0.690853 3.558249 0.572863
C -1.559712 2.514320 0.185692
C -1.094370 1.164613 0.096717
C 0.278583 0.972949 0.413476
C 1.101735 1.994080 0.791345
H -3.211704 3.865787 -0.031897
H 1.280888 4.105562 1.177384
H -1.093793 4.561577 0.626285
C -2.908855 2.829224 -0.109725
C -2.065199 0.193475 -0.286238
H 2.139422 1.781913 1.011051
C -3.363958 0.529713 -0.549822
C -3.799887 1.864517 -0.470850
H -4.052730 -0.262706 -0.808385
H -4.829833 2.109834 -0.686764
I 1.272000 -0.920227 0.362285
S -1.702177 -1.522932 -0.496578
O -0.991122 -1.661955 -1.749339
O -2.918616 -2.281000 -0.326452
N -0.736606 -1.737757 0.788903
C -0.635842 -3.118649 1.259330
H -0.122960 -3.114261 2.220154
H -1.633624 -3.530130 1.401058
H -0.090923 -3.772792 0.568885
O 3.069455 0.081716 0.069899
C 3.269235 0.550179 -1.245704
H 2.493610 1.280854 -1.516701
H 3.198041 -0.272991 -1.966814
C 4.635459 1.197376 -1.319293
H 5.406173 0.476130 -1.051206
H 4.834861 1.567376 -2.325357
H 4.693465 2.033578 -0.622589

perF_CMe2O_OEt.log
Energy (E) = -986.203732263 Hartree
Enthalpy (H) = -985.978802 Hartree
Gibbs free energy (G) = -986.045008 Hartree
Charge = 0, Spin = 1
C 1.384717 2.254198 -0.299882
C 2.531425 1.573215 0.063897
C 2.490825 0.196148 0.227755
C 1.322664 -0.515284 0.027628
C 0.174401 0.195334 -0.282028
C 0.189818 1.564574 -0.468779
O 0.054415 -2.416128 -0.542337
I -1.530564 -1.065222 -0.338955
F -0.863385 2.276312 -0.835087
F 1.425229 3.562937 -0.490723
F 3.662670 2.234760 0.239458
F 3.618459 -0.407234 0.601426
C 1.199347 -2.041556 0.155234
C 2.360900 -2.777055 -0.504766
H 3.289610 -2.668469 0.049187
H 2.089503 -3.831331 -0.540378
H 2.499082 -2.423562 -1.525545
C 1.102577 -2.400846 1.638042
H 0.987300 -3.480475 1.731358
H 1.999898 -2.088183 2.173644
H 0.237904 -1.915849 2.095045
O -2.773695 0.547647 -0.128984
C -2.787903 1.116959 1.158666
H -1.771958 1.403587 1.470361
H -3.158045 0.397231 1.900752
C -3.670953 2.344787 1.121672
H -4.678832 2.069966 0.813980
H -3.719759 2.817221 2.103138
H -3.274218 3.060659 0.403347

perF_NMeCO2_OEt.log
Energy (E) = -1076.24394758 Hartree
Enthalpy (H) = -1076.047001 Hartree
Gibbs free energy (G) = -1076.115198 Hartree
Charge = 0, Spin = 1
C -1.798263 -2.036724 -0.516491
C -2.766481 -1.197563 0.008823
C -2.464685 0.113238 0.343375
C -1.170522 0.623806 0.218138
C -0.211032 -0.260174 -0.285592
C -0.512706 -1.549600 -0.682237
I 1.717190 0.476541 -0.489142

F 0.395212 -2.336156 -1.233471
F -2.105001 -3.272985 -0.873294
F -4.004340 -1.636398 0.147237
F -3.467942 0.882572 0.754127
N -0.855089 1.933427 0.564414
C -1.548939 2.602357 1.662035
H -1.842596 1.861002 2.401080
H -0.858973 3.311126 2.109906
H -2.426702 3.147841 1.322470
C -0.139033 2.783539 -0.322070
O -0.204324 3.978781 -0.165417
O 0.542319 2.157804 -1.244645
O 2.475123 -1.229920 0.291461
C 2.256359 -1.390792 1.680255
H 1.179224 -1.407532 1.895573
H 2.689566 -0.554433 2.241754
C 2.891385 -2.698314 2.095661
H 3.959756 -2.679156 1.887330
H 2.744719 -2.872613 3.161538
H 2.446774 -3.522159 1.540001

PyIBCMe2O_D_OEt.log
Energy (E) = -605.454029703 Hartree
Enthalpy (H) = -605.212716 Hartree
Gibbs free energy (G) = -605.271208 Hartree
Charge = 0, Spin = 1
C 0.352427 3.130445 0.149291
C 1.728238 3.003281 0.007277
C 1.617653 0.725728 -0.085090
C 0.243134 0.786432 0.034748
H -0.098990 4.107251 0.243964
H 2.357132 3.885335 -0.013526
O 1.311185 -1.584610 -0.505034
I -0.647510 -1.110498 0.077170
C -0.425935 1.979939 0.181071
H -1.495403 2.004565 0.334785
N 2.355989 1.832426 -0.092281
C 2.272278 -0.646083 -0.112162
C 3.412697 -0.681514 -1.116639
H 4.163226 0.066918 -0.867332
H 3.862177 -1.673653 -1.099424
H 3.030435 -0.486611 -2.117649
C 2.781516 -0.943383 1.299040
H 3.246864 -1.928769 1.306109
H 3.508305 -0.194206 1.614205
H 1.951013 -0.944531 2.009067
O -2.437475 -0.151611 0.531774
C -3.200448 0.176417 -0.605487
H -3.563085 -0.727129 -1.111698
H -2.594427 0.727237 -1.341935
C -4.371734 1.028256 -0.164333
H -4.015120 1.944158 0.308795
H -5.001822 1.296559 -1.012786
H -4.972858 0.484941 0.563176

PyrroleNMeIBCMe2O_A_OEt.log
Energy (E) = -606.641361666 Hartree
Enthalpy (H) = -606.376596 Hartree
Gibbs free energy (G) = -606.437576 Hartree
Charge = 0, Spin = 1
C 1.034645 2.703574 0.220259
C -0.179889 2.065380 0.256889
C 0.131574 0.706153 0.063328
C 1.474597 0.533780 -0.060012
H 1.273615 3.751050 0.297825
H -1.150018 2.502999 0.413260
I -0.937419 -1.050647 0.090953
O 1.023259 -1.722279 -0.422364
N 2.035477 1.776633 0.037489
C 3.438463 2.109964 -0.089990
H 4.045861 1.465916 0.543549
H 3.777630 2.011040 -1.120601
H 3.578256 3.139602 0.229859
C 2.078535 -0.850775 -0.124434
C 3.116904 -1.001959 -1.232177
H 4.023680 -0.430960 -1.026370
H 3.382197 -2.056367 -1.302215
H 2.695450 -0.685518 -2.185448
C 2.680421 -1.215217 1.234743
H 3.067029 -2.233416 1.187615
H 3.491526 -0.542243 1.521653
H 1.904119 -1.168971 2.000056
O -2.619529 0.081018 0.500744
C -3.217070 0.634752 -0.647528
H -3.686906 -0.144252 -1.262124
H -2.466072 1.131145 -1.279984
C -4.256115 1.641241 -0.200999
H -3.784243 2.432688 0.382126
H -4.759749 2.089337 -1.058249
H -4.999733 1.154529 0.428602

Anth_I_Allene_F.log
Energy (E) = -765.449042886 Hartree
Enthalpy (H) = -765.198740 Hartree
Gibbs free energy (G) = -765.262217 Hartree
Charge = 0, Spin = 1
C 1.311571 3.942686 -0.523246
C 0.002614 3.780086 -0.202319
C -0.573526 2.480546 -0.092330
C 0.247880 1.323937 -0.307848
C 1.613970 1.543556 -0.660531
C 2.121898 2.800545 -0.765907
C -1.915407 2.319691 0.228481
C -0.358575 0.072726 -0.182737
C -1.717204 -0.110358 0.085779
C -2.507416 1.065271 0.312279
C -3.889832 0.927512 0.631138
H -4.461807 1.829524 0.809862
C -4.469720 -0.297805 0.703999
C -3.697322 -1.460858 0.438147
C -2.373800 -1.376224 0.137602
H -2.521658 3.200231 0.410031
H 1.738884 4.932205 -0.607829
H -0.639717 4.634337 -0.027835
H 2.251943 0.698122 -0.872457
H 3.156917 2.934002 -1.051333
H -5.519568 -0.394845 0.944221
H -4.177026 -2.429950 0.455040
H -1.825219 -2.260406 -0.147306
I 0.873698 -1.648043 -0.241806
C 1.864927 -0.893840 1.536307
C 3.085477 -0.470640 1.482647
H 1.285887 -0.923729 2.449133
C 4.307503 -0.028943 1.358938
H 4.493344 1.019080 1.158059
H 5.159569 -0.686353 1.468021
F -0.396196 -2.101476 -1.917141

Anth_I_Bn_F.log
Energy (E) = -920.208566478 Hartree
Enthalpy (H) = -919.883211 Hartree
Gibbs free energy (G) = -919.955337 Hartree
Charge = 0, Spin = 1
C 1.642116 4.241069 0.082873
C 2.637696 3.422732 -0.344186
C 2.502022 2.004376 -0.291833
C 1.284070 1.429964 0.204813
C 0.270459 2.330191 0.656795
C 0.444374 3.677945 0.600731
C 3.527587 1.172866 -0.722925
C 1.190488 0.036633 0.234844
C 2.234368 -0.814156 -0.138352
C 3.430613 -0.211752 -0.650544
C 4.501752 -1.045679 -1.085213
H 5.389198 -0.568022 -1.481513
C 4.411014 -2.397618 -0.998801
C 3.245147 -2.993686 -0.445811
C 2.196099 -2.236112 -0.027377
H 4.434680 1.617240 -1.117323
H 1.756760 5.315328 0.042912
H 3.564986 3.827710 -0.730171
H -0.640511 1.930595 1.081120
H -0.335424 4.332120 0.966567
H 5.228519 -3.024319 -1.327981
H 3.202021 -4.068652 -0.335426
H 1.359311 -2.694462 0.476844
I -0.678256 -0.827971 0.717180
C -1.532869 0.095153 -1.105022
C -3.005122 0.047356 -0.939596
H -1.171223 -0.504762 -1.935458
H -1.142491 1.106325 -1.168829
C -3.691864 1.130908 -0.389291
C -3.720597 -1.107341 -1.258864
C -5.061172 1.069256 -0.181629
H -3.139777 2.029022 -0.134044
C -5.091062 -1.171594 -1.049309
H -3.193726 -1.955417 -1.681299
C -5.765482 -0.082972 -0.511812
H -5.581259 1.920177 0.237244
H -5.633264 -2.071135 -1.307735
H -6.833627 -0.131314 -0.350423
F 0.531732 -1.640355 2.324226

Anth_I_carbazole_F.log
Energy (E) = -1165.90909095 Hartree
Enthalpy (H) = -1165.532828 Hartree
Gibbs free energy (G) = -1165.609226 Hartree
Charge = 0, Spin = 1
C -1.537502 2.200310 2.818761
C -0.304921 2.630051 2.439915
C 0.605471 1.758061 1.776691
C 0.200028 0.414231 1.482923
C -1.080614 -0.005643 1.941110
C -1.918317 0.853080 2.582434
C 1.868367 2.192276 1.389656
C 1.098196 -0.383475 0.772085
C 2.389597 0.011855 0.422943
C 2.774327 1.353629 0.752200
C 4.063372 1.822885 0.365880
H 4.327718 2.842651 0.616480
C 4.930106 1.017031 -0.300036
C 4.558973 -0.321621 -0.601430
C 3.338536 -0.811564 -0.252785
H 2.155205 3.218134 1.591722
H -2.223403 2.871561 3.316543
H 0.016402 3.645752 2.633556
H -1.387308 -1.030382 1.791363
H -2.888054 0.502855 2.910121
H 5.905639 1.381100 -0.591200
H 5.267795 -0.967864 -1.100990
H 3.104701 -1.851872 -0.423582
I 0.330362 -2.114034 -0.179937
N -1.067623 -0.826487 -1.170576
C -0.665822 0.418872 -1.648607
C -2.376490 -0.679925 -0.716237

C 0.566714 0.787252 -2.180619
C -1.712015 1.351629 -1.520966
C -3.201891 -1.631173 -0.124153
C -2.820793 0.639522 -0.926523
C 0.739799 2.105795 -2.567440
H 1.370439 0.068504 -2.284410
C -1.522788 2.671076 -1.924692
C -4.465447 -1.233491 0.283531
H -2.866013 -2.649697 0.027982
C -4.099269 1.015811 -0.527677
C -0.292404 3.042898 -2.439516
H 1.692184 2.415437 -2.977067
H -2.323647 3.394049 -1.832374
C -4.913854 0.077498 0.085668
H -5.121460 -1.953646 0.754009
H -4.447687 2.028955 -0.685228
H -0.125019 4.064482 -2.752440
H -5.909051 0.353410 0.406505
F 1.787509 -3.142824 0.892335

Anth_I_CCPh_F.log
Energy (E) = -957.011678294 Hartree
Enthalpy (H) = -956.703340 Hartree
Gibbs free energy (G) = -956.777090 Hartree
Charge = 0, Spin = 1
C 0.584093 3.537543 -1.837299
C -0.374541 3.654404 -0.883071
C -1.039443 2.506130 -0.362321
C -0.681822 1.203097 -0.845361
C 0.322177 1.129360 -1.857382
C 0.926457 2.250826 -2.334041
C -2.021398 2.632672 0.611943
C -1.359092 0.113544 -0.296012
C -2.375362 0.216326 0.655355
C -2.698584 1.533392 1.124379
C -3.703103 1.693865 2.122745
H -3.921100 2.697565 2.465780
C -4.363973 0.620387 2.625870
C -4.066773 -0.681407 2.139436
C -3.114639 -0.880973 1.189598
H -2.270029 3.621372 0.981246
H 1.081001 4.414163 -2.229108
H -0.662584 4.623995 -0.496287
H 0.602551 0.168856 -2.262261
H 1.675772 2.162118 -3.108904
H -5.125055 0.749098 3.383060
H -4.622131 -1.527959 2.519389
H -2.963804 -1.866204 0.777894
I -0.715395 -1.838842 -0.825972
C 1.173135 -1.311658 -0.070618
C 2.290920 -0.979841 0.247893
C 3.593839 -0.535114 0.632004
C 3.991751 0.773185 0.339242
C 4.480790 -1.387897 1.293865
C 5.252487 1.216625 0.704682
H 3.298896 1.429062 -0.172492
C 5.741649 -0.939221 1.653853
H 4.170212 -2.398564 1.520279
C 6.130534 0.362379 1.361346
H 5.551636 2.230681 0.476620
H 6.422230 -1.606134 2.165455
H 7.114568 0.710248 1.644833
F -2.730095 -2.035048 -1.465071

Anth_I_Naph_F.log
Energy (E) = -1034.43102076 Hartree
Enthalpy (H) = -1034.085762 Hartree
Gibbs free energy (G) = -1034.159536 Hartree
Charge = 0, Spin = 1
C -4.729834 -0.040687 2.336680
C -4.236093 1.148136 1.905560
C -3.159252 1.198550 0.972975
C -2.581951 -0.025566 0.497043
C -3.152338 -1.252919 0.950805
C -4.183267 -1.254108 1.837798
C -2.654296 2.415404 0.531168
C -1.501284 0.073954 -0.382864
C -0.997865 1.285147 -0.861377
C -1.608519 2.491264 -0.379800
C -1.126009 3.752378 -0.836700
H -1.603581 4.645568 -0.453340
C -0.105183 3.831958 -1.728027
C 0.489075 2.640172 -2.223551
C 0.063457 1.417938 -1.806807
H -3.094746 3.333731 0.903422
H -5.548018 -0.071582 3.043086
H -4.647671 2.086281 2.256693
H -2.812669 -2.178365 0.512811
H -4.610231 -2.196143 2.153776
H 0.253085 4.792851 -2.070416
H 1.288160 2.707241 -2.949157
H 0.522666 0.530493 -2.216333
I -0.458968 -1.706445 -0.862020
C 1.262428 -0.873040 0.128063
C 2.471821 -0.893402 -0.504324
C 1.148433 -0.367735 1.441714
C 3.637095 -0.414444 0.144846
H 2.566631 -1.268344 -1.519565
C 2.254207 0.109597 2.087670
H 0.181348 -0.356373 1.931452
C 3.525039 0.098134 1.461489
C 4.903462 -0.425070 -0.484882
H 2.175396 0.501360 3.094606
C 4.685214 0.582745 2.109065
C 6.009316 0.049749 0.165709
H 4.980640 -0.818017 -1.491397
C 5.898952 0.559390 1.477892
H 4.593188 0.972647 3.115438
H 6.974226 0.036273 -0.322788
H 6.780364 0.931739 1.982134
F -2.394349 -2.205893 -1.702690

Anth_I_OCH2CF3_F.log
Energy (E) = -1101.45112177 Hartree
Enthalpy (H) = -1101.197145 Hartree
Gibbs free energy (G) = -1101.267673 Hartree
Charge = 0, Spin = 1
C 1.893796 3.052419 -2.063625
C 0.817527 3.403375 -1.315372
C -0.068910 2.414671 -0.797866
C 0.188900 1.027795 -1.058656
C 1.328704 0.706104 -1.853661
C 2.141574 1.680807 -2.339188
C -1.172352 2.777530 -0.036337
C -0.709999 0.102849 -0.521472
C -1.838692 0.444998 0.229505
C -2.060931 1.840089 0.476509
C -3.180102 2.243658 1.260326
H -3.320842 3.302920 1.435324
C -4.040225 1.326861 1.773540
C -3.831749 -0.054302 1.515491
C -2.777328 -0.483259 0.770377
H -1.346487 3.828655 0.164991
H 2.563118 3.806848 -2.452807
H 0.602881 4.441459 -1.094897
H 1.562329 -0.326460 -2.060926
H 2.998155 1.407305 -2.939596
H -4.887214 1.639623 2.368131
H -4.533827 -0.777962 1.906433
H -2.683148 -1.532051 0.535860
I -0.235330 -1.938466 -0.714624
O 1.561213 -1.476362 0.210514
C 1.482325 -1.074359 1.535793
H 0.567160 -0.512722 1.768915
H 1.566419 -1.909035 2.240279
C 2.629983 -0.130537 1.831885
F 2.594203 0.235043 3.121675
F 2.562323 0.981825 1.095333
F 3.814923 -0.695086 1.603290
F -2.073769 -2.131911 -1.611880

Anth_I_OEt_F.log
Energy (E) = -803.817129391 Hartree
Enthalpy (H) = -803.542387 Hartree
Gibbs free energy (G) = -803.607879 Hartree
Charge = 0, Spin = 1
C 1.077804 4.082064 -0.666005
C -0.206815 3.814727 -0.319112
C -0.669500 2.472201 -0.195937
C 0.236364 1.385284 -0.436244
C 1.578664 1.714210 -0.795502
C 1.977118 3.009294 -0.909787
C -1.985950 2.204567 0.157251
C -0.274276 0.089426 -0.321077
C -1.598831 -0.201466 0.017963
C -2.471971 0.908242 0.271224
C -3.824786 0.666309 0.648616
H -4.459864 1.523269 0.835622
C -4.298902 -0.599608 0.771984
C -3.440411 -1.700660 0.508680
C -2.144250 -1.514609 0.141360
H -2.656525 3.034911 0.349072
H 1.421773 5.102743 -0.760195
H -0.914013 4.613076 -0.131611
H 2.293468 0.925371 -0.967674
H 2.998713 3.227087 -1.189781
H -5.326025 -0.776718 1.059553
H -3.829904 -2.706424 0.587613
H -1.535835 -2.367199 -0.114677
I 1.069425 -1.520463 -0.548054
O 2.224480 -0.673537 0.914765
C 1.650545 -0.679945 2.200475
H 0.677092 -0.169680 2.190276
H 1.473522 -1.708177 2.542727
C 2.595663 0.036107 3.140113
H 2.747931 1.060122 2.800531
H 2.192445 0.057467 4.152889
H 3.561123 -0.467726 3.157059
F -0.258003 -2.165796 -2.007409

Me_Pyrrole_3I_Allene_F.log
Energy (E) = -475.683970591 Hartree
Enthalpy (H) = -475.521209 Hartree
Gibbs free energy (G) = -475.576464 Hartree
Charge = 0, Spin = 1
C -1.501687 0.954931 -0.078271
C -0.935538 -0.291280 -0.005416
C -1.950183 -1.263082 0.107878
C -3.127586 -0.560175 0.105251
N -2.852947 0.775484 -0.012562
H -1.063326 1.933707 -0.159432
H -1.793367 -2.322239 0.193284
H -4.145239 -0.904976 0.178093
C -3.830807 1.840377 0.035941
H -4.770041 1.485611 -0.382226

H -4.003634 2.176760 1.058107
H -3.483795 2.682135 -0.559139
I 1.098461 -0.710727 -0.103904
C 1.442332 1.324499 -0.778853
C 2.108515 2.159647 -0.047834
H 1.083517 1.569627 -1.770119
C 2.791282 2.968103 0.715638
H 3.871976 2.996054 0.672428
H 2.295379 3.633725 1.409649
F 0.420308 -2.645376 0.514245

Me_Pyrrole_3I_Bn_F.log
Energy (E) = -630.442743470 Hartree
Enthalpy (H) = -630.204954 Hartree
Gibbs free energy (G) = -630.268092 Hartree
Charge = 0, Spin = 1
C -2.291089 1.223460 -0.010986
C -1.969485 -0.111358 0.001907
C -3.155964 -0.873543 -0.010549
C -4.179001 0.039201 -0.028948
N -3.653968 1.302247 -0.034144
H -1.687607 2.113379 -0.008597
H -3.200761 -1.946832 -0.014242
H -5.245547 -0.109560 -0.042479
C -4.416365 2.529591 0.029117
H -3.883407 3.319207 -0.496150
H -5.377892 2.380697 -0.456779
H -4.586061 2.841851 1.059546
I -0.046085 -0.907518 0.007667
C 0.810338 1.130093 -0.006372
C 2.283190 0.941111 -0.005916
H 0.449904 1.636512 0.885621
H 0.448676 1.623411 -0.905185
C 2.980683 0.796771 -1.205845
C 2.985014 0.828587 1.194800
C 4.349088 0.569529 -1.206287
H 2.439913 0.868654 -2.142439
C 4.353467 0.601355 1.196150
H 2.447857 0.925488 2.131195
C 5.040190 0.473326 -0.004823
H 4.876924 0.466967 -2.144812
H 4.884780 0.523663 2.135104
H 6.107045 0.296826 -0.004392
F -1.154880 -2.760700 0.020310

Me_Pyrrole_3I_carbazole_F.log
Energy (E) = -876.145984968 Hartree
Enthalpy (H) = -875.856991 Hartree
Gibbs free energy (G) = -875.925644 Hartree
Charge = 0, Spin = 1
C 0.854715 0.011744 1.661202
C 1.822878 0.012040 0.690683
C 3.098991 0.008803 1.288107
C 2.860805 0.009139 2.639162
N 1.510172 0.006617 2.855722
H -0.220182 0.012632 1.607344
H 4.042074 -0.001474 0.774554
H 3.549706 0.007189 3.466622
C 0.865585 0.078099 4.150379
H 1.473874 -0.443868 4.885401
H 0.730647 1.111283 4.468455
H -0.106532 -0.405603 4.096490
I 1.462606 -0.010783 -1.356678
N -0.597004 -0.009656 -0.907757
C -1.334297 -1.129145 -0.545478
C -1.337569 1.114771 -0.567983
C -0.992973 -2.475862 -0.642937
C -2.576505 -0.725998 -0.015203
C -1.000233 2.460251 -0.692775
C -2.578654 0.718793 -0.029796
C -1.920042 -3.411021 -0.215973
H -0.033592 -2.783621 -1.038234
C -3.496798 -1.685752 0.403315
C -1.930210 3.401097 -0.285098
H -0.041763 2.762801 -1.094253
C -3.501934 1.684078 0.368890
C -3.163487 -3.024456 0.299359
H -1.677989 -4.463227 -0.282676
H -4.456749 -1.385834 0.804995
C -3.172634 3.021358 0.237725
H -1.691357 4.452460 -0.373246
H -4.461052 1.389531 0.776477
H -3.865377 -3.782147 0.619601
H -3.876877 3.783293 0.542350
F 3.526560 -0.007584 -1.494133

Me_Pyrrole_3I_CCPh_F.log
Energy (E) = -667.248579162 Hartree
Enthalpy (H) = -667.027795 Hartree
Gibbs free energy (G) = -667.093503 Hartree
Charge = 0, Spin = 1
C -1.221131 1.635438 -0.006815
C -2.034113 0.533612 0.002130
C -3.384334 0.937106 -0.011628
C -3.348144 2.308234 -0.026406
N -2.044029 2.722904 -0.027976
H -0.149623 1.725829 -0.002177
H -4.236575 0.283677 -0.018554
H -4.151639 3.025107 -0.039690
C -1.600835 4.098869 0.035110
H -2.310205 4.731277 -0.493925
H -1.518739 4.443454 1.065714
H -0.629927 4.189608 -0.446139
I -1.398782 -1.449234 0.003702
C 0.592446 -0.764038 0.001469
C 1.773553 -0.502902 0.000599
C 3.166159 -0.176452 -0.000753
C 3.855111 -0.019278 -1.207116
C 3.855588 -0.011055 1.204232
C 5.204252 0.296939 -1.205692
H 3.320491 -0.149900 -2.137957
C 5.204717 0.305175 1.200126
H 3.321343 -0.135313 2.136157
C 5.881984 0.460055 -0.003459
H 5.728639 0.415300 -2.144117
H 5.729463 0.429981 2.137512
H 6.935180 0.705944 -0.004508
F -3.474031 -1.836231 0.006281

Me_Pyrrole_3I_Naph_F.log
Energy (E) = -744.666783765 Hartree
Enthalpy (H) = -744.408929 Hartree
Gibbs free energy (G) = -744.474024 Hartree
Charge = 0, Spin = 1
C 1.242361 1.655364 -0.044510
C 2.056585 0.552020 -0.081526
C 3.402737 0.963118 -0.158007
C 3.363487 2.333998 -0.169918
N 2.060568 2.746306 -0.096430
H 0.172871 1.756203 0.011891
H 4.250962 0.305417 -0.201404
H 4.163076 3.053517 -0.223447
C 1.609032 4.118660 -0.165536
H 2.346828 4.765954 0.303377
H 1.462226 4.438828 -1.196999
H 0.668995 4.219233 0.372130
I 1.427167 -1.433403 0.002869
C -0.586750 -0.736443 0.320767
C -1.468011 -0.694919 -0.722886
C -0.998300 -0.346999 1.616121
C -2.804164 -0.263636 -0.528418
H -1.159855 -0.985328 -1.721912
C -2.279930 0.077982 1.831999
H -0.289634 -0.381849 2.434620
C -3.215697 0.131039 0.769755
C -3.735384 -0.211217 -1.592298
H -2.600990 0.377929 2.822376
C -4.548455 0.564320 0.963946
C -5.017591 0.212828 -1.375695
H -3.412380 -0.514695 -2.580612
C -5.428640 0.604734 -0.082442
H -4.857944 0.862171 1.958336
H -5.723496 0.248935 -2.194335
H -6.445658 0.936799 0.076515
F 3.534832 -1.789110 -0.313966

Me_Pyrrole_3I_OCH2CF3_F.log
Energy (E) = -811.688485925 Hartree
Enthalpy (H) = -811.521847 Hartree
Gibbs free energy (G) = -811.583226 Hartree
Charge = 0, Spin = 1
C -0.524862 1.572370 -0.394156
C -1.351633 0.565861 0.030199
C -2.535693 1.111767 0.564062
C -2.386345 2.470644 0.447619
N -1.176086 2.740070 -0.130877
H 0.444959 1.540407 -0.857400
H -3.377166 0.565220 0.947284
H -3.051426 3.269492 0.728275
C -0.629657 4.061778 -0.360318
H -1.412767 4.725412 -0.720301
H -0.202610 4.475734 0.552337
H 0.148654 3.998538 -1.116192
I -0.920558 -1.450511 -0.131700
O 1.032026 -0.876415 -0.526035
C 1.780195 -0.582851 0.606533
H 1.288867 0.139823 1.272150
H 2.046741 -1.469857 1.192461
C 3.080361 0.056794 0.168597
F 3.827104 0.355714 1.239280
F 2.867284 1.197303 -0.502566
F 3.795766 -0.742614 -0.618252
F -2.902762 -1.707626 0.296062

Me_Pyrrole_3I_OEt_F.log
Energy (E) = -514.053542804 Hartree
Enthalpy (H) = -513.866286 Hartree
Gibbs free energy (G) = -513.921985 Hartree
Charge = 0, Spin = 1
C -1.573199 0.474424 -0.337606
C -0.759119 -0.568440 0.015806
C -1.540786 -1.648241 0.471713
C -2.839776 -1.215774 0.381350
N -2.852993 0.061488 -0.109782
H -1.335502 1.444360 -0.737283
H -1.176903 -2.607158 0.790439
H -3.760145 -1.719545 0.623530
C -4.032370 0.882610 -0.279995
H -4.876857 0.251353 -0.546967
H -4.272202 1.428319 0.632316
H -3.864625 1.594865 -1.084339
I 1.303859 -0.543229 -0.140848
O 1.143773 1.470084 -0.497872
C 1.057361 2.248186 0.675331

H 0.259622 1.872849 1.331848
H 1.993792 2.199743 1.244675
C 0.765311 3.677102 0.272578
H -0.185194 3.730912 -0.259683
H 0.711682 4.325117 1.147732
H 1.546485 4.045747 -0.390653
F 1.134617 -2.562859 0.252192

Py_PhI_Allene_F.log
Energy (E) = -705.328778049 Hartree
Enthalpy (H) = -705.103739 Hartree
Gibbs free energy (G) = -705.166100 Hartree
Charge = 0, Spin = 1
C -0.381202 1.515011 0.060088
C 0.884732 0.951431 -0.044831
C 2.041142 1.674010 0.185007
C 1.940883 3.008249 0.554319
C 0.692682 3.597630 0.694906
C -0.450090 2.856622 0.443746
C -1.647289 0.816169 -0.270781
C -2.804910 -0.508063 -1.732792
C -3.942979 -0.494029 -0.936881
C -3.909000 0.232270 0.245410
C -2.745504 0.902130 0.583821
H 2.997744 1.195998 0.035730
H 2.841217 3.579715 0.731768
H 0.608105 4.637068 0.980595
H -1.425561 3.322542 0.503868
H -2.794939 -1.054889 -2.668476
H -4.827543 -1.035495 -1.239986
H -4.771417 0.267305 0.897080
H -2.665810 1.458702 1.507389
N -1.681992 0.128963 -1.412241
I 1.199740 -1.114995 -0.423220
C -0.469959 -1.662269 0.856491
C -0.264773 -1.825342 2.124016
H -1.424350 -1.854034 0.382938
C -0.021574 -1.970095 3.397600
H -0.162763 -1.149549 4.088650
H 0.326605 -2.913836 3.795927
F 2.883718 -0.313765 -1.517763

Py_PhI_Bn_F.log
Energy (E) = -860.089095663 Hartree
Enthalpy (H) = -859.789299 Hartree
Gibbs free energy (G) = -859.861206 Hartree
Charge = 0, Spin = 1
C 2.340898 0.337349 0.533584
C 1.389562 1.259663 0.119438
C 1.540435 2.618367 0.316373
C 2.694503 3.089430 0.933265
C 3.675481 2.194356 1.330287
C 3.497268 0.831338 1.129745
C 2.154695 -1.126554 0.377438
C 0.917621 -2.977123 0.889755
C 1.774640 -3.810571 0.186654
C 2.868895 -3.245274 -0.452993
C 3.064826 -1.878510 -0.356973
H 0.783359 3.308899 -0.030641
H 2.826131 4.151595 1.085932
H 4.579907 2.554290 1.801038
H 4.252935 0.127517 1.454865
H 0.050444 -3.384535 1.397727
H 1.581136 -4.872625 0.137296
H 3.549509 -3.858081 -1.028031
H 3.878128 -1.382173 -0.866204
N 1.095311 -1.660910 0.987864
I -0.309876 0.589833 -0.914543
C -1.503213 1.054562 0.880154
C -2.828886 0.412216 0.704420
H -1.555760 2.134186 0.990824
H -0.916281 0.610015 1.680947
C -2.954181 -0.969760 0.867339
C -3.949295 1.147954 0.322900
C -4.175518 -1.595284 0.674336
H -2.075990 -1.542369 1.147923
C -5.173176 0.521877 0.127398
H -3.858811 2.219434 0.188694
C -5.290190 -0.850178 0.304595
H -4.260901 -2.665004 0.810710
H -6.036275 1.106409 -0.161629
H -6.243635 -1.337886 0.154121
F 1.326238 0.123601 -2.281568

Py_PhI_carbazole_F.log
Energy (E) = -1105.79101423 Hartree
Enthalpy (H) = -1105.439946 Hartree
Gibbs free energy (G) = -1105.516292 Hartree
Charge = 0, Spin = 1
C -2.067297 0.149362 1.364792
C -0.808777 -0.371332 1.097827
C 0.200472 -0.422319 2.039955
C -0.049671 0.049812 3.321190
C -1.302119 0.557382 3.633776
C -2.292092 0.605338 2.665110
C -3.152991 0.296335 0.362585
C -3.792287 1.038058 -1.700967
C -5.107380 0.649400 -1.495225
C -5.437838 0.059574 -0.283537
C -4.446467 -0.117952 0.664847
H 1.169830 -0.833546 1.790096
H 0.732321 0.011092 4.066099
H -1.506323 0.926705 4.629173
H -3.259254 1.033578 2.895104
H -3.496676 1.506134 -2.632610
H -5.847867 0.803937 -2.266758
H -6.449977 -0.266248 -0.086168
H -4.650559 -0.598621 1.611210
N -2.832707 0.872270 -0.793977
I -0.359699 -1.197987 -0.797947
N 1.479226 -0.184321 -0.688701
C 1.607239 1.195776 -0.641504
C 2.707489 -0.740351 -0.360925
C 0.627435 2.167027 -0.832205
C 2.939520 1.536090 -0.335976
C 3.062067 -2.080850 -0.227264
C 3.648790 0.290274 -0.154537
C 1.017433 3.492604 -0.738089
H -0.403342 1.894692 -1.029472
C 3.307056 2.877492 -0.252420
C 4.374893 -2.374451 0.104213
H 2.337594 -2.871110 -0.378282
C 4.964797 -0.030536 0.170107
C 2.342717 3.848690 -0.458069
H 0.279263 4.270736 -0.879888
H 4.329188 3.154049 -0.025337
C 5.321596 -1.362197 0.297495
H 4.673566 -3.408456 0.213385
H 5.696342 0.752834 0.324858
H 2.609851 4.894781 -0.394722
H 6.339077 -1.626559 0.550792
F -2.222459 -2.093961 -0.573849

Py_PhI_CCPh_F.log
Energy (E) = -896.892073706 Hartree
Enthalpy (H) = -896.609239 Hartree
Gibbs free energy (G) = -896.682971 Hartree
Charge = 0, Spin = 1
C 2.090126 1.284313 0.301113
C 0.913052 1.080463 -0.405300
C 0.058096 2.106348 -0.754727
C 0.387326 3.409445 -0.404562
C 1.565759 3.656973 0.282976
C 2.399345 2.604742 0.630422
C 2.995339 0.201891 0.761248
C 3.219483 -1.791973 1.846444
C 4.597491 -1.784555 1.693309
C 5.181734 -0.709230 1.038233
C 4.369539 0.305588 0.566711
H -0.853653 1.897342 -1.297272
H -0.273077 4.220193 -0.678354
H 1.833117 4.667774 0.558502
H 3.302977 2.790074 1.196891
H 2.728026 -2.612603 2.356270
H 5.192603 -2.601927 2.074833
H 6.252107 -0.669530 0.888427
H 4.774964 1.146922 0.022739
N 2.429433 -0.819767 1.397550
I 0.405736 -0.853921 -1.101116
C -1.555585 -0.515695 -0.440455
C -2.718530 -0.419979 -0.122882
C -4.084857 -0.296617 0.279908
C -4.413795 -0.191809 1.634768
C -5.107601 -0.279001 -0.672949
C -5.737761 -0.070085 2.024866
H -3.620758 -0.207122 2.369595
C -6.430255 -0.160563 -0.277132
H -4.851711 -0.360660 -1.720436
C -6.748747 -0.054885 1.071392
H -5.981957 0.011372 3.075303
H -7.214352 -0.149701 -1.021960
H -7.781586 0.038495 1.378399
F 2.455809 -0.825185 -1.660774

Py_PhI_Naph_F.log
Energy (E) = -974.313629946 Hartree
Enthalpy (H) = -973.993689 Hartree
Gibbs free energy (G) = -974.066680 Hartree
Charge = 0, Spin = 1
C 2.221009 1.211365 0.457459
C 1.053579 1.196039 -0.291284
C 0.268877 2.317557 -0.472783
C 0.671944 3.523048 0.089028
C 1.852371 3.581337 0.814259
C 2.613360 2.434868 0.996527
C 3.025532 -0.001424 0.746603
C 3.088714 -2.096401 1.653254
C 4.443442 -2.226481 1.392799
C 5.101923 -1.172293 0.772832
C 4.382995 -0.038391 0.444053
H -0.641832 2.259086 -1.054307
H 0.069027 4.408987 -0.053370
H 2.177851 4.516934 1.247776
H 3.519599 2.468450 1.587935
H 2.541555 -2.898606 2.134556
H 4.964844 -3.133181 1.664753
H 6.155348 -1.241599 0.538145
H 4.837514 0.793859 -0.073672
N 2.386902 -1.006617 1.342984
I 0.462755 -0.567055 -1.285364
C -1.415806 -0.420637 -0.256552
C -2.587479 -0.315341 -0.949073
C -1.410858 -0.479481 1.155972
C -3.827579 -0.269705 -0.262359
H -2.595199 -0.260962 -2.033049

C -2.592333 -0.427851 1.840164
H -0.464358 -0.564264 1.679105
C -3.828490 -0.324268 1.154099
C -5.059505 -0.164025 -0.948818
H -2.602594 -0.470436 2.922714
C -5.064286 -0.272906 1.839866
C -6.240345 -0.116641 -0.259265
H -5.049558 -0.122662 -2.031262
C -6.242583 -0.171812 1.151452
H -5.058843 -0.315637 2.922218
H -7.177953 -0.037277 -0.792639
H -7.182108 -0.133868 1.685983
F 2.505056 -0.307547 -2.003434

Py_PhI_OCH2CF3_F.log
Energy (E) = -1041.33315885 Hartree
Enthalpy (H) = -1041.104467 Hartree
Gibbs free energy (G) = -1041.174039 Hartree
Charge = 0, Spin = 1
C 1.271888 1.384943 -0.016874
C 0.156523 0.887309 -0.676163
C -0.861554 1.692776 -1.149292
C -0.760721 3.067841 -0.983714
C 0.357693 3.605329 -0.363639
C 1.356551 2.770919 0.115612
C 2.313026 0.534980 0.611931
C 2.781159 -1.207871 2.010090
C 4.148845 -1.027960 1.865448
C 4.596147 -0.000750 1.046720
C 3.663710 0.799055 0.409987
H -1.723746 1.253153 -1.630001
H -1.551361 3.708648 -1.347118
H 0.446435 4.675432 -0.237095
H 2.208378 3.184412 0.640155
H 2.395133 -1.996395 2.645413
H 4.840840 -1.678889 2.380190
H 5.654331 0.167367 0.900272
H 3.963404 1.594453 -0.257800
N 1.876689 -0.443737 1.402554
I -0.030941 -1.167396 -1.099840
O -1.994622 -0.970851 -0.483757
C -2.153757 -0.766706 0.882434
H -2.150540 -1.699299 1.456576
H -1.398096 -0.094952 1.312029
C -3.495806 -0.106009 1.112000
F -3.685425 0.105067 2.421386
F -4.506514 -0.854561 0.671301
F -3.574492 1.078760 0.496146
F 1.936768 -1.015057 -1.667296

Py_PhI_OEt_F.log
Energy (E) = -743.692552406 Hartree
Enthalpy (H) = -743.443312 Hartree
Gibbs free energy (G) = -743.507551 Hartree
Charge = 0, Spin = 1
C 1.063433 1.337568 0.068095
C -0.274590 1.001524 -0.094950
C -1.279499 1.948404 -0.145880
C -0.950174 3.294404 -0.067028
C 0.378814 3.669470 0.061003
C 1.365441 2.699216 0.132421
C 2.169360 0.362913 0.249189
C 2.957061 -1.472899 1.350262
C 4.173579 -1.419735 0.685554
C 4.374448 -0.405125 -0.239175
C 3.357767 0.506745 -0.460125
H -2.310455 1.643565 -0.246740
H -1.731804 4.039646 -0.112488
H 0.647042 4.715189 0.120839
H 2.399487 2.984370 0.277656
H 2.765275 -2.248306 2.082889
H 4.937755 -2.156570 0.887624
H 5.304872 -0.332360 -0.785775
H 3.458284 1.296984 -1.190881
N 1.974323 -0.600961 1.145024
I -0.866628 -1.006180 -0.353668
O -2.536598 -0.555800 0.758240
C -3.735629 -0.619501 0.022459
H -3.698221 0.039208 -0.858389
H -3.916097 -1.633844 -0.357874
C -4.866822 -0.204340 0.937647
H -4.700830 0.810218 1.299174
H -5.823488 -0.241325 0.415863
H -4.909695 -0.868515 1.799427
F 0.842503 -1.139938 -1.518314

Anth_I_2thiazole.log
Energy (E) = -1217.70668694 Hartree
Enthalpy (H) = -1217.456428 Hartree
Gibbs free energy (G) = -1217.523543 Hartree
Charge = 0, Spin = 1
C 0.431767 4.085859 -1.000918
C -0.758755 3.788203 -0.419734
C -1.162068 2.436854 -0.214346
C -0.290131 1.371553 -0.617918
C 0.942755 1.731479 -1.239651
C 1.287705 3.034103 -1.423877
C -2.381741 2.138545 0.379823
C -0.728720 0.067500 -0.388188
C -1.963828 -0.258918 0.172496
C -2.806702 0.830530 0.574120
C -4.065674 0.553538 1.182349
H -4.678350 1.393613 1.484972
C -4.481081 -0.724223 1.374325
C -3.660824 -1.803741 0.947962
C -2.451190 -1.585466 0.367267
H -3.024379 2.952287 0.696987
H 0.728349 5.114187 -1.153834
H -1.435135 4.571331 -0.100327
H 1.605029 0.956986 -1.596912
H 2.224448 3.273152 -1.908684
H -5.437657 -0.927441 1.835757
H -4.014108 -2.818212 1.072501
H -1.882465 -2.415457 -0.020910
I 0.624944 -1.523105 -0.749307
F -0.922748 -2.158193 -2.078848
C 1.906733 -0.601347 0.742541
N 1.489011 -0.246992 1.913175
S 3.584973 -0.332562 0.478492
C 2.511774 0.272520 2.665055
C 3.726167 0.301076 2.056626
H 2.312897 0.617067 3.668142
H 4.664298 0.652363 2.449549

AnthI8BA_B_2thiazole.log
Energy (E) = -1423.56485007 Hartree
Enthalpy (H) = -1423.223363 Hartree
Gibbs free energy (G) = -1423.300712 Hartree
Charge = 0, Spin = 1
C -6.982692 -0.765040 0.062533
C -5.921790 -1.486477 0.511714
C -4.585391 -1.060728 0.252561
C -4.381742 0.145646 -0.492950
C -5.521453 0.873219 -0.946312
C -6.779657 0.434197 -0.678559
C -3.482733 -1.783129 0.701350
C -3.082906 0.573205 -0.753655
C -1.985040 -0.154122 -0.301595
C -2.182903 -1.356266 0.438298
C -1.041035 -2.078608 0.875402
H -1.208208 -2.993243 1.433534
C 0.252853 -1.703546 0.627714
C 0.395617 -0.480279 -0.093858
H -3.639352 -2.697309 1.262955
H -7.991435 -1.098540 0.264894
H -6.070042 -2.399641 1.074764
H -5.359379 1.783516 -1.510350
H -7.637005 0.993212 -1.028003
H -2.924851 1.486319 -1.316755
I 2.294415 0.397177 -0.531946
C -0.647885 0.259755 -0.544115
H -0.490675 1.180535 -1.092094
C 1.342629 -2.639296 1.099769
H 1.590824 -3.308721 0.273198
H 0.876819 -3.268264 1.858057
C 2.630164 -2.027568 1.676859
H 2.455147 -0.981998 1.935830
H 2.839067 -2.516988 2.627231
C 3.875411 -2.189457 0.780456
H 4.491315 -1.286809 0.822875
H 4.496392 -3.009602 1.131493
C 3.552354 -2.501783 -0.680768
O 2.719509 -1.692506 -1.280187
O 4.030337 -3.484805 -1.210691
C 1.556665 2.231178 0.345522
N 1.371795 2.352597 1.620090
S 1.226850 3.653067 -0.560485
C 0.937888 3.614434 1.933637
C 0.803304 4.462724 0.879544
H 0.735802 3.867595 2.962837
H 0.490694 5.492437 0.885582

FuranIBCMe2S_A_2thiazole.log
Energy (E) = -1324.04597375 Hartree
Enthalpy (H) = -1323.849175 Hartree
Gibbs free energy (G) = -1323.911382 Hartree
Charge = 0, Spin = 1
C 0.817267 2.977114 -0.203174
C -0.180891 2.064376 -0.180928
C 0.494738 0.811732 -0.112193
C 1.826025 1.034397 -0.080646
O 2.029525 2.370749 -0.139633
H 0.821960 4.049707 -0.270987
H -1.238831 2.254656 -0.215601
I -0.243770 -1.122941 0.035396
S 2.411886 -1.564359 -0.304900
C 2.987137 0.114589 0.095089
C 4.119015 0.499917 -0.855917
H 4.480274 1.505600 -0.631735
H 4.942288 -0.203672 -0.734790
H 3.781512 0.461131 -1.890290
C 3.470120 0.196230 1.545231
H 4.306833 -0.487985 1.684654
H 3.796093 1.211383 1.785839
H 2.672590 -0.094197 2.229119
C -2.262569 -0.218822 0.222963
N -2.761832 0.162002 1.354192
S -3.298527 0.029942 -1.127836
C -4.019587 0.685155 1.186475
C -4.479124 0.697438 -0.093222
H -4.564709 1.046272 2.045022
H -5.428119 1.050890 -0.457815

Indole_NMe_IBCONAc_A_2thiazole.log

Energy (E) = -1302.15738968 Hartree
Enthalpy (H) = -1301.889883 Hartree
Gibbs free energy (G) = -1301.963966 Hartree
Charge = 0, Spin = 1
C -0.978934 1.587903 0.144160
C -0.378511 2.866616 0.014391
C -1.122008 4.046910 0.044212
C -2.485055 3.942452 0.215091
C -3.097958 2.687736 0.363929
C -2.368985 1.518537 0.332308
C 0.126983 0.692786 0.060666
H -0.637747 5.008462 -0.059516
H -3.091117 4.837416 0.243965
H -4.167769 2.637754 0.513044
H -2.874969 0.575894 0.476013
N 0.980304 2.730270 -0.120470
C 1.290190 1.398848 -0.084624
C 2.660871 0.803910 -0.152714
O 3.643747 1.495030 -0.349319
I 0.313404 -1.376723 0.114069
C 1.882581 3.862482 -0.244205
H 1.788968 4.503855 0.632169
H 2.896780 3.491051 -0.325735
H 1.627515 4.438733 -1.133550
N 2.566988 -0.543506 0.028875
C 3.685765 -1.363920 0.079388
C 3.340482 -2.838542 0.176794
H 2.746949 -3.152777 -0.684505
H 4.260171 -3.414292 0.206077
H 2.763540 -3.039284 1.081981
O 4.837276 -0.994525 0.063980
C -1.823033 -1.671247 0.095367
N -2.512935 -1.896851 1.167220
S -2.735568 -1.637095 -1.356107
C -3.841871 -2.037064 0.864928
C -4.152478 -1.920499 -0.455949
H -4.551104 -2.226331 1.655544
H -5.119446 -1.995516 -0.922545

Me_Pyrrole_3I_2thiazole.log
Energy (E) = -927.943264948 Hartree
Enthalpy (H) = -927.780479 Hartree
Gibbs free energy (G) = -927.838663 Hartree
Charge = 0, Spin = 1
C -1.064132 1.406088 0.200937
C -1.328774 0.076784 -0.004045
C -2.691347 -0.091626 -0.320614
C -3.225654 1.171259 -0.297149
N -2.240834 2.069475 0.012452
H -0.162271 1.924146 0.476241
H -3.177182 -1.028520 -0.519914
H -4.233530 1.504274 -0.478491
C -2.433844 3.488719 0.217356
H -3.224406 3.843860 -0.439788
H -2.705555 3.708122 1.249605
H -1.516324 4.018955 -0.026970
I 0.054007 -1.476939 0.097594
F -1.705072 -2.670624 -0.018810
C 1.614854 0.027151 0.177578
N 2.092952 0.507882 1.283420
S 2.330762 0.681893 -1.241501
C 3.067935 1.439320 1.034693
C 3.332541 1.669005 -0.279669
H 3.562511 1.926152 1.861216
H 4.053530 2.344392 -0.706702

NaphIBMeUreaMe_2thiazole.log
Energy (E) = -1266.24062683 Hartree
Enthalpy (H) = -1265.948561 Hartree
Gibbs free energy (G) = -1266.020789 Hartree
Charge = 0, Spin = 1
C 2.443367 -1.058630 -0.508167
C 3.181867 0.001599 0.070618
C 2.511002 1.163264 0.508321
C 1.144526 1.315319 0.402142
C 0.441607 0.213801 -0.152991
C 1.040123 -0.919605 -0.600396
H 3.101182 1.965753 0.929861
H 0.456260 -1.735788 -1.008150
I -1.657089 0.358558 -0.214289
C -0.433636 3.177817 0.070304
O -0.609132 4.368947 0.283466
N -1.034302 2.438703 -0.880156
C -2.010654 3.155317 -1.676013
H -1.547461 4.005899 -2.176795
H -2.411481 2.485080 -2.438658
H -2.837780 3.550355 -1.077293
N 0.496783 2.458374 0.873806
C 1.184342 3.263022 1.869144
H 1.615888 2.604492 2.620876
H 1.971761 3.883707 1.433027
H 0.460404 3.927683 2.327635
C 4.587970 -0.132555 0.176260
C 5.216120 -1.261460 -0.271970
C 4.474064 -2.317789 -0.846830
C 3.115245 -2.216949 -0.961345
H 5.155892 0.677634 0.616471
H 6.290724 -1.350964 -0.186957
H 4.985859 -3.204730 -1.194167
H 2.533753 -3.019242 -1.398621
C -1.768852 -1.743577 0.437559
N -1.499000 -2.136892 1.640626
S -2.295207 -3.004842 -0.611863
C -1.697888 -3.486871 1.777130
C -2.133195 -4.128905 0.660552
H -1.510902 -3.958588 2.729621
H -2.362426 -5.173053 0.536735

NpthIBCMe2O_C_2thiazole.log
Energy (E) = -1156.81044195 Hartree
Enthalpy (H) = -1156.532357 Hartree
Gibbs free energy (G) = -1156.599472 Hartree
Charge = 0, Spin = 1
C -2.815938 -0.553427 0.053067
C -1.563841 -1.107786 0.005032
C -0.479336 -0.225476 -0.086631
C -0.589485 1.126730 -0.123706
H -3.687625 -1.192344 0.133158
H 0.272649 1.777657 -0.186979
O -0.038576 -2.839603 -0.513684
I 1.388471 -1.247549 -0.146317
C -2.075668 3.102418 -0.137579
C -3.335756 3.631774 -0.099017
C -4.461381 2.782653 -0.008211
C -4.301936 1.425406 0.041530
C -3.010406 0.847089 0.003442
C -1.883240 1.701816 -0.085906
H -1.206101 3.744336 -0.206100
H -3.476272 4.703451 -0.136933
H -5.453089 3.213332 0.022009
H -5.161515 0.770137 0.110413
C -1.262100 -2.605935 0.091944
C -2.317361 -3.427574 -0.639867
H -2.007388 -4.471517 -0.611796
H -3.300224 -3.346728 -0.173092
H -2.381960 -3.110041 -1.679647
C -1.225274 -2.998487 1.574235
H -2.187294 -2.823874 2.059345
H -0.968538 -4.055493 1.648472
H -0.465339 -2.418632 2.102319
C 2.420329 0.651809 0.220461
N 2.582441 1.160245 1.402636
S 3.144944 1.572430 -1.041361
C 3.300774 2.326320 1.346596
C 3.694269 2.710363 0.102411
H 3.515930 2.863417 2.257717
H 4.268312 3.576470 -0.178089

NpthISO2NMe_D_2thiazole.log
Energy (E) = -1606.78988859 Hartree
Enthalpy (H) = -1606.543367 Hartree
Gibbs free energy (G) = -1606.612788 Hartree
Charge = 0, Spin = 1
C -0.417028 3.185617 -1.102035
C 0.832624 3.557824 -0.708454
C 1.758320 2.609471 -0.217674
C 1.413202 1.224095 -0.114343
C 0.070276 0.916955 -0.467293
C -0.802413 1.840439 -0.962649
H 3.257294 4.116169 0.074715
H -1.119545 3.903400 -1.500188
H 1.151327 4.590250 -0.774234
C 3.047975 3.057073 0.156454
C 2.456668 0.342305 0.294089
H -1.807003 1.553546 -1.239247
C 3.697346 0.812087 0.629894
C 3.999626 2.184027 0.589852
H 4.456209 0.089280 0.896925
H 4.985203 2.527915 0.869458
I -0.786294 -1.019881 -0.174427
S 2.327123 -1.428296 0.379119
O 1.717533 -1.759277 1.654053
O 3.639436 -1.983434 0.130946
N 1.344836 -1.666109 -0.869509
C 1.280027 -3.060389 -1.299761
H 0.703375 -3.107926 -2.223002
H 2.280050 -3.445678 -1.499820
H 0.808214 -3.719870 -0.558697
C -2.668271 -0.067746 0.395890
N -2.844810 0.593762 1.492252
S -4.089552 -0.246830 -0.560433
C -4.148059 0.999958 1.617695
C -4.974205 0.632573 0.601694
H -4.447881 1.564820 2.486871
H -6.027263 0.826190 0.493542

perF_CMe2O_2thiazole.log
Energy (E) = -1400.09174087 Hartree
Enthalpy (H) = -1399.891511 Hartree
Gibbs free energy (G) = -1399.960167 Hartree
Charge = 0, Spin = 1
C 1.181438 2.466627 -0.293431
C 2.474420 2.102044 0.036515
C 2.795720 0.761106 0.187149
C 1.847743 -0.232704 0.015991
C 0.550162 0.165735 -0.240019
C 0.206049 1.489472 -0.428524
O 1.105710 -2.398954 -0.554607
I -0.818974 -1.467811 -0.201753
F -1.007582 1.889524 -0.789043
F 0.879730 3.740386 -0.490965
F 3.402400 3.033383 0.181807
F 4.054106 0.473580 0.517904
C 2.139955 -1.748690 0.084998

C 3.423388 -2.100486 -0.666114
H 4.318830 -1.742244 -0.164477
H 3.460490 -3.187327 -0.728807
H 3.384550 -1.701477 -1.679074
C 2.247768 -2.149401 1.558651
H 2.432499 -3.222053 1.614406
H 3.061216 -1.618271 2.055429
H 1.315529 -1.928607 2.082193
C -2.441577 -0.112183 0.305226
N -2.480975 0.504760 1.441782
S -3.799722 0.163515 -0.705341
C -3.624091 1.254286 1.549674
C -4.466221 1.187321 0.484744
H -3.799271 1.833293 2.443167
H -5.417945 1.672122 0.353450

perF_NMeCO2_2thiazole.log
Energy (E) = -1490.13961881 Hartree
Enthalpy (H) = -1489.967313 Hartree
Gibbs free energy (G) = -1490.037256 Hartree
Charge = 0, Spin = 1
C 0.945214 2.580849 -0.581828
C 2.204391 2.363618 -0.046312
C 2.609031 1.091713 0.322951
C 1.760806 -0.017887 0.226387
C 0.488534 0.249411 -0.290760
C 0.092304 1.502017 -0.717456
I -0.832393 -1.352184 -0.412672
F -1.105871 1.699512 -1.255614
F 0.576095 3.792098 -0.968603
F 3.047265 3.375059 0.065164
F 3.863835 0.952339 0.738738
N 2.159298 -1.287750 0.606104
C 3.083230 -1.474174 1.720559
H 2.946494 -0.667741 2.437434
H 2.848078 -2.424076 2.191071
H 4.121419 -1.503666 1.396096
C 2.002050 -2.413647 -0.276894
O 2.671497 -3.397207 -0.049064
O 1.139893 -2.233016 -1.217594
C -2.381052 -0.154433 0.473427
N -2.204681 0.488984 1.577694
S -3.938256 0.000639 -0.221389
C -3.348172 1.159874 1.929658
C -4.396861 1.003422 1.079172
H -3.362065 1.750795 2.831922
H -5.387213 1.417733 1.153411

Py_PhI_2thiazole.log
Energy (E) = -1157.58608424 Hartree
Enthalpy (H) = -1157.361277 Hartree
Gibbs free energy (G) = -1157.427541 Hartree
Charge = 0, Spin = 1
C 1.457681 1.308801 0.162169
C 0.162308 1.084140 -0.282248
C -0.751114 2.101791 -0.473424
C -0.368642 3.413514 -0.224767
C 0.922408 3.679281 0.204725
C 1.817194 2.637006 0.394076
C 2.452450 0.241670 0.436257
C 2.928554 -1.729199 1.480306
C 4.241704 -1.712606 1.035272
C 4.658232 -0.645452 0.251361
C 3.751205 0.353150 -0.051933
H -1.750745 1.884616 -0.825705
H -1.076597 4.216006 -0.377315
H 1.232929 4.696480 0.399507
H 2.817161 2.837502 0.756847
H 2.567419 -2.544008 2.097193
H 4.915257 -2.517088 1.294162
H 5.670801 -0.599802 -0.125999
H 4.019686 1.186730 -0.685324
N 2.048870 -0.772729 1.194664
I -0.463811 -0.867761 -0.804951
F 1.416457 -0.812248 -1.828250
C -2.262794 -0.517012 0.348226
N -2.298058 -0.439377 1.638954
S -3.802341 -0.365758 -0.409449
C -3.578985 -0.247151 2.086317
C -4.533597 -0.185899 1.119819
H -3.764794 -0.161486 3.145963
H -5.595098 -0.052697 1.236269

PyIBCMe2O_D_2thiazole.log
Energy (E) = -1019.34005686 Hartree
Enthalpy (H) = -1019.123368 Hartree
Gibbs free energy (G) = -1019.184327 Hartree
Charge = 0, Spin = 1
C 0.710850 3.127063 -0.199322
C 2.092231 3.042532 -0.091372
C 2.060027 0.755952 -0.037875
C 0.682118 0.774880 -0.131767
H 0.222546 4.088077 -0.269204
H 2.693318 3.943883 -0.068568
O 1.923668 -1.571964 -0.413796
I -0.173097 -1.162949 -0.078528
C -0.028065 1.950985 -0.204619
H -1.107402 1.969627 -0.264832
N 2.755483 1.891832 -0.005072
C 2.767469 -0.588338 0.081707
C 4.053083 -0.579761 -0.735015
H 4.729019 0.202391 -0.393094
H 4.531041 -1.553008 -0.628783
H 3.819699 -0.418696 -1.786868
C 3.077486 -0.813096 1.565423
H 3.577366 -1.775397 1.675002
H 3.718146 -0.022318 1.957660
H 2.152267 -0.834799 2.146364
C -2.142817 -0.238177 0.217765
N -2.572863 0.168300 1.372244
S -3.273081 -0.025167 -1.062910
C -3.843225 0.674864 1.278027
C -4.389228 0.652809 0.032415
H -4.334154 1.049899 2.163010
H -5.365675 0.985805 -0.274162

PyrroleNMeIBCMe2O_A_2thiazole.log
Energy (E) = -1020.53076787 Hartree
Enthalpy (H) = -1020.290612 Hartree
Gibbs free energy (G) = -1020.354392 Hartree
Charge = 0, Spin = 1
C 1.238821 2.732563 0.022640
C 0.088151 1.986387 -0.006141
C 0.537375 0.651604 -0.057569
C 1.896688 0.596909 -0.041963
H 1.374505 3.801048 0.042217
H -0.923474 2.352953 0.008519
I -0.407574 -1.190925 -0.022948
O 1.727407 -1.710587 -0.291091
N 2.328438 1.894135 0.008072
C 3.700131 2.357712 -0.018796
H 4.306351 1.803817 0.695024
H 4.136371 2.248067 -1.010973
H 3.712688 3.409032 0.258590
C 2.649112 -0.719282 0.012051
C 3.778342 -0.787442 -1.016693
H 4.603725 -0.112341 -0.783285
H 4.158164 -1.808757 -1.018829
H 3.391447 -0.560906 -2.009897
C 3.200804 -0.934750 1.426008
H 3.698391 -1.904295 1.459396
H 3.913338 -0.161892 1.724762
H 2.373935 -0.941946 2.137578
C -2.312159 -0.149919 0.194020
N -2.850954 0.122186 1.340887
S -3.219835 0.403699 -1.158365
C -4.033056 0.798673 1.182201
C -4.396686 1.044301 -0.104947
H -4.598466 1.093377 2.053000
H -5.274997 1.551581 -0.464895

Anth_I_ArF5.log
Energy (E) = -1376.92048947 Hartree
Enthalpy (H) = -1376.660305 Hartree
Gibbs free energy (G) = -1376.737265 Hartree
Charge = 0, Spin = 1
C 0.055138 4.106588 -1.224163
C -0.985012 3.891093 -0.378444
C -1.471545 2.574887 -0.128559
C -0.842528 1.456967 -0.771849
C 0.233545 1.732082 -1.667871
C 0.662479 3.004790 -1.884245
C -2.540158 2.359599 0.732494
C -1.347527 0.188869 -0.480849
C -2.442465 -0.050772 0.351060
C -3.045587 1.089034 0.979433
C -4.147576 0.899376 1.863021
H -4.579643 1.775346 2.330427
C -4.638039 -0.342157 2.108710
C -4.057587 -1.469051 1.466454
C -3.002905 -1.334110 0.619060
H -2.998196 3.211040 1.223298
H 0.416682 5.108507 -1.408546
H -1.474962 4.714998 0.125281
H 0.698825 0.922151 -2.209785
H 1.472514 3.181345 -2.578660
H -5.474769 -0.479608 2.779486
H -4.473094 -2.451428 1.644198
H -2.626823 -2.195563 0.090327
I -0.313153 -1.497939 -1.237639
F -2.162579 -1.869009 -2.192629
C 1.391314 -0.829491 -0.041217
C 2.584528 -0.449955 -0.622175
C 1.311287 -0.795241 1.338252
C 3.670691 -0.032328 0.130049
F 2.722399 -0.472528 -1.951181
C 2.374499 -0.381043 2.123823
F 0.187016 -1.160222 1.953370
C 3.558802 0.001190 1.511150
F 4.803913 0.333614 -0.450771
F 2.277157 -0.351441 3.444557
F 4.584142 0.395362 2.247102

AnthI8BA_B_ArF5.log
Energy (E) = -1582.77579394 Hartree
Enthalpy (H) = -1582.424412 Hartree
Gibbs free energy (G) = -1582.512678 Hartree
Charge = 0, Spin = 1
C -7.166123 -0.715140 0.040514
C -6.165538 -1.459149 0.581857
C -4.801517 -1.192150 0.262296
C -4.505290 -0.121703 -0.642692
C -5.583766 0.634620 -1.188961
C -6.871002 0.348582 -0.859385

C -3.759244 -1.941753 0.802098
C -3.178257 0.150565 -0.963274
C -2.141352 -0.601779 -0.417986
C -2.431497 -1.671303 0.479107
C -1.351274 -2.432180 1.000878
H -1.590839 -3.252053 1.668949
C -0.033546 -2.210468 0.700509
C 0.204056 -1.102130 -0.167211
H -3.986598 -2.754218 1.483080
H -8.196857 -0.927270 0.289957
H -6.384163 -2.269793 1.265807
H -5.351821 1.441333 -1.873262
H -7.681778 0.927735 -1.279781
H -2.950318 0.960265 -1.647481
I 2.163830 -0.451202 -0.704304
C -0.776688 -0.341731 -0.715262
H -0.544507 0.474803 -1.388073
C 0.977142 -3.185696 1.261422
H 1.153578 -3.955009 0.505997
H 0.468093 -3.691937 2.081038
C 2.322151 -2.638129 1.768996
H 2.245398 -1.561846 1.922755
H 2.498835 -3.048429 2.762191
C 3.538759 -2.997045 0.890033
H 4.230592 -2.151830 0.838173
H 4.090678 -3.828921 1.319501
C 3.170913 -3.426789 -0.527152
O 2.416774 -2.602910 -1.215253
O 3.532436 -4.502157 -0.954974
C 1.555261 1.556819 -0.093333
C 1.478308 2.594358 -1.000675
C 1.276594 1.833648 1.231923
C 1.133818 3.880469 -0.612666
C 0.925224 3.104275 1.654756
C 0.857809 4.130714 0.722540
F 1.329134 0.860842 2.141750
F 0.658337 3.351649 2.927146
F 0.527670 5.349667 1.110055
F 1.060032 4.862388 -1.497522
F 1.721874 2.376563 -2.293528

FuranIBCMe2S_A_ArF5.log
Energy (E) = -1483.25709311 Hartree
Enthalpy (H) = -1483.050498 Hartree
Gibbs free energy (G) = -1483.122920 Hartree
Charge = 0, Spin = 1
C 1.491427 2.367523 1.873014
C 0.542767 1.505477 1.438743
C 1.265289 0.556582 0.662054
C 2.570597 0.899231 0.660601
O 2.716912 2.015649 1.406313
H 1.452119 3.233975 2.507510
H -0.509085 1.531136 1.659137
I 0.665393 -1.085170 -0.453431
S 3.305707 -1.431480 -0.395784
C 3.728120 0.311455 -0.074302
C 3.932779 1.078079 -1.382348
H 4.161488 2.127197 -1.179719
H 4.759367 0.634938 -1.937046
H 3.035887 1.025155 -2.000553
C 4.994138 0.363553 0.776411
H 5.811825 -0.109439 0.233288
H 5.266465 1.399473 0.986104
H 4.849778 -0.165959 1.716531
C -1.456250 -0.356889 -0.189700
C -2.384532 -1.098897 0.508930
C -1.882597 0.803277 -0.803483
C -3.710663 -0.703373 0.607922
C -3.197107 1.233178 -0.724165
C -4.112314 0.468902 -0.013676
F -2.028143 -2.232670 1.116232
F -4.592336 -1.425032 1.284826
F -5.372523 0.861251 0.069971
F -3.591455 2.354066 -1.311395
F -1.016677 1.558766 -1.483120

Indole_NMe_IBCONAc_A_ArF5.log
Energy (E) = -1461.36483986 Hartree
Enthalpy (H) = -1461.087485 Hartree
Gibbs free energy (G) = -1461.173096 Hartree
Charge = 0, Spin = 1
C -0.217457 1.900683 -0.254037
C -1.172547 2.949219 -0.202543
C -0.814717 4.292453 -0.321681
C 0.517894 4.588714 -0.505193
C 1.478156 3.567018 -0.580542
C 1.130424 2.238966 -0.460811
C -1.008801 0.725202 -0.100541
H -1.564891 5.070156 -0.275204
H 0.829243 5.619495 -0.601629
H 2.514788 3.828381 -0.741872
H 1.896487 1.483312 -0.546697
N -2.430907 2.425599 -0.043061
C -2.329516 1.064184 0.011859
C -3.455472 0.090428 0.124684
O -4.605599 0.454890 0.274703
I -0.602889 -1.307802 0.007551
C -3.631562 3.242327 0.016310
H -3.729421 3.819245 -0.903186
H -4.488994 2.591768 0.137794
H -3.565967 3.928400 0.860474
N -2.954857 -1.178529 0.032980
C -3.772535 -2.303946 0.036451
C -3.000491 -3.609468 0.035728
H -2.356681 -3.679077 0.915191
H -3.706970 -4.433273 0.045738
H -2.374997 -3.687062 -0.856073
O -4.980460 -2.292510 0.027413
C 1.542859 -0.948548 0.079082
C 2.350773 -1.171940 -1.018858
C 2.124320 -0.483315 1.243881
C 3.713287 -0.919970 -0.975435
C 3.482442 -0.222087 1.319508
C 4.273989 -0.440179 0.200016
F 1.833497 -1.620647 -2.159088
F 4.480625 -1.133024 -2.031560
F 5.570338 -0.200057 0.257110
F 4.030252 0.233441 2.433424
F 1.376741 -0.236913 2.314741

Me_Pyrrole_3I_ArF5.log
Energy (E) = -1087.15348687 Hartree
Enthalpy (H) = -1086.980841 Hartree
Gibbs free energy (G) = -1087.050599 Hartree
Charge = 0, Spin = 1
C -1.249225 1.608678 -0.443791
C -1.976783 0.489108 -0.132605
C -3.348009 0.811338 -0.098818
C -3.411341 2.148767 -0.396549
N -2.146530 2.622575 -0.607662
H -0.193691 1.769962 -0.570003
H -4.150785 0.126358 0.100289
H -4.262726 2.802842 -0.478621
C -1.799411 4.003168 -0.870534
H -2.623098 4.486145 -1.390648
H -1.597245 4.544158 0.053332
H -0.917879 4.044145 -1.506180
I -1.225561 -1.420175 0.227226
F -3.267734 -1.929545 0.302776
C 0.790539 -0.580779 0.115023
C 1.589554 -0.771851 -0.995903
C 1.328781 0.115249 1.182294
C 2.889413 -0.290910 -1.055437
F 1.127555 -1.430518 -2.056792
C 2.621753 0.612046 1.156847
F 0.597498 0.344379 2.270624
C 3.402404 0.403596 0.028764
F 3.640418 -0.484667 -2.129247
F 3.118893 1.280943 2.186689
F 4.638907 0.869094 -0.011081

NaphIBMeUreaMe_ArF5.log
Energy (E) = -1425.45288797 Hartree
Enthalpy (H) = -1425.150957 Hartree
Gibbs free energy (G) = -1425.234216 Hartree
Charge = 0, Spin = 1
C -0.893236 2.579413 -0.873615
C -2.079651 2.819277 -0.138048
C -2.751790 1.741994 0.478153
C -2.291068 0.445067 0.404805
C -1.077042 0.258547 -0.304164
C -0.403512 1.255232 -0.934020
H -3.668554 1.950035 1.013160
H 0.517032 1.054573 -1.467973
I -0.288204 -1.687111 -0.307823
C -3.269504 -1.812337 0.352049
O -4.225908 -2.478532 0.715584
N -2.451085 -2.100049 -0.683105
C -2.746895 -3.344964 -1.366494
H -3.776446 -3.343803 -1.723253
H -2.083554 -3.447145 -2.226379
H -2.633335 -4.220130 -0.719200
N -2.952875 -0.609260 1.038797
C -3.851854 -0.283490 2.133652
H -3.369269 0.445216 2.782222
H -4.807226 0.115209 1.782942
H -4.059237 -1.193226 2.686316
C -2.571391 4.145276 -0.061513
C -1.914424 5.169112 -0.685806
C -0.730903 4.924509 -1.418243
C -0.231628 3.655044 -1.509533
H -3.479069 4.331725 0.498743
H -2.300646 6.177274 -0.620689
H -0.222961 5.745707 -1.904719
H 0.674595 3.453949 -2.067394
C 1.718303 -0.782248 0.074863
C 2.766200 -0.955624 -0.804550
C 1.965341 -0.086466 1.242273
C 4.032414 -0.451050 -0.547464
C 3.214367 0.436523 1.532859
C 4.251153 0.247738 0.629336
F 2.582026 -1.619419 -1.949879
F 0.984182 0.109045 2.124220
F 3.434719 1.105729 2.654937
F 5.451382 0.736645 0.893177
F 5.026487 -0.624046 -1.407001

NpthIBCMe2O_C_ArF5.log
Energy (E) = -1316.02195171 Hartree
Enthalpy (H) = -1315.733971 Hartree
Gibbs free energy (G) = -1315.812052 Hartree
Charge = 0, Spin = 1
C -3.493329 0.602472 0.070382
C -2.600660 -0.435824 0.044015
C -1.255863 -0.115383 -0.187672

C -0.789081 1.141970 -0.389765
H -4.543588 0.408794 0.254252
H 0.257173 1.346906 -0.575298
O -1.990427 -2.683242 -0.358887
I -0.025508 -1.847225 -0.192585
C -1.300153 3.552608 -0.594409
C -2.211933 4.571309 -0.573763
C -3.577448 4.299237 -0.335198
C -4.000854 3.016190 -0.124000
C -3.081530 1.940196 -0.139043
C -1.711460 2.217022 -0.375508
H -0.251362 3.751754 -0.776527
H -1.891008 5.590598 -0.739546
H -4.288697 5.113844 -0.321061
H -5.046843 2.802147 0.057364
C -2.946672 -1.904935 0.285140
C -4.306904 -2.263006 -0.299085
H -4.459054 -3.333587 -0.168193
H -5.118786 -1.735408 0.203337
H -4.328401 -2.033709 -1.363351
C -2.936451 -2.160628 1.796376
H -3.695288 -1.564009 2.305030
H -3.128964 -3.218833 1.974036
H -1.963055 -1.907240 2.221820
C 1.773514 -0.556893 -0.012013
C 2.693815 -0.454440 -1.035689
C 2.063318 0.085191 1.177152
C 3.873130 0.262627 -0.894260
C 3.227468 0.813702 1.355725
C 4.135663 0.897417 0.309502
F 1.199645 0.031784 2.192373
F 3.485459 1.427865 2.501339
F 5.254523 1.585606 0.462573
F 4.745120 0.349210 -1.888476
F 2.471452 -1.050618 -2.207931

NpthISO2NMe_D_ArF5.log
Energy (E) = -1766.00121909 Hartree
Enthalpy (H) = -1765.744877 Hartree
Gibbs free energy (G) = -1765.825358 Hartree
Charge = 0, Spin = 1
C 0.090206 2.654481 -2.011138
C 1.227386 3.271823 -1.583894
C 2.182934 2.586593 -0.799633
C 1.981402 1.222356 -0.419340
C 0.735881 0.668483 -0.819558
C -0.160001 1.330664 -1.606356
H 3.461455 4.307242 -0.704666
H -0.626202 3.166002 -2.637505
H 1.433753 4.300251 -1.851060
C 3.358970 3.270677 -0.409664
C 3.056129 0.590271 0.270496
H -1.077023 0.849302 -1.914561
C 4.188186 1.279773 0.611100
C 4.339085 2.641737 0.297886
H 4.985904 0.737110 1.099859
H 5.238852 3.164992 0.588134
I 0.073803 -1.243695 -0.149811
S 3.113385 -1.127584 0.720599
O 2.426615 -1.271261 1.989576
O 4.492534 -1.555695 0.675014
N 2.279970 -1.743656 -0.515736
C 2.440916 -3.188895 -0.667804
H 1.963616 -3.490143 -1.599249
H 3.498549 -3.445042 -0.724191
H 1.997759 -3.761939 0.156505
C -1.952716 -0.389757 0.082596
C -2.194274 0.613884 1.001909
C -3.029808 -0.918417 -0.598620
C -3.471933 1.094863 1.234853
C -4.323448 -0.465587 -0.386363
C -4.538958 0.545963 0.536393
F -2.847335 -1.887217 -1.499299
F -5.345848 -0.981210 -1.052384
F -5.764571 0.991447 0.751688
F -3.690032 2.062892 2.111456
F -1.185001 1.158217 1.676694

perF_CMe2O_ArF5.log
Energy (E) = -1559.30203242 Hartree
Enthalpy (H) = -1559.091962 Hartree
Gibbs free energy (G) = -1559.172238 Hartree
Charge = 0, Spin = 1
C 1.549384 2.443242 -0.801998
C 2.860292 2.334064 -0.374282
C 3.353776 1.101146 0.027560
C 2.562744 -0.034201 0.010863
C 1.238392 0.118360 -0.355551
C 0.726780 1.325966 -0.785940
O 2.161650 -2.347482 -0.194429
I 0.129837 -1.677052 -0.090074
F -0.513803 1.479835 -1.235262
F 1.083831 3.604482 -1.231059
F 3.641159 3.400095 -0.372017
F 4.617767 1.060806 0.443753
C 3.052648 -1.451966 0.370545
C 4.421364 -1.748334 -0.238026
H 5.222656 -1.187912 0.236563
H 4.601712 -2.813558 -0.099941
H 4.409499 -1.539510 -1.306977
C 3.098972 -1.570995 1.895332
H 3.415279 -2.580401 2.157244
H 3.798672 -0.852568 2.324759
H 2.110721 -1.392093 2.323915
C -1.755950 -0.561467 0.079965
C -1.967428 0.310906 1.128518
C -2.801514 -0.796555 -0.788231
C -3.181252 0.948502 1.316976
C -4.033723 -0.181481 -0.627773
C -4.218522 0.694712 0.430525
F -0.973211 0.585081 1.977482
F -3.363934 1.790841 2.322369
F -5.386883 1.289286 0.597816
F -5.028968 -0.418527 -1.468546
F -2.650277 -1.632090 -1.816435

perF_NMeCO2_ArF5.log
Energy (E) = -1649.34814525 Hartree
Enthalpy (H) = -1649.165951 Hartree
Gibbs free energy (G) = -1649.246643 Hartree
Charge = 0, Spin = 1
C 1.176440 2.565656 -1.001892
C 2.358464 2.660506 -0.284263
C 2.927844 1.541265 0.301327
C 2.318397 0.282618 0.245435
C 1.106438 0.237437 -0.451672
C 0.561117 1.331299 -1.096710
I 0.142334 -1.600916 -0.531231
F -0.552350 1.223924 -1.813372
F 0.660988 3.629417 -1.595931
F 2.977405 3.823885 -0.203671
F 4.109506 1.698935 0.887027
N 2.880828 -0.844107 0.820394
C 3.668035 -0.740503 2.045962
H 3.291328 0.086461 2.643196
H 3.550893 -1.669746 2.595129
H 4.727250 -0.595347 1.844428
C 3.055176 -2.053632 0.063979
O 3.860766 -2.859173 0.470368
O 2.302343 -2.141378 -0.984304
C -1.704688 -0.659595 0.058132
C -1.839543 -0.146046 1.334970
C -2.780721 -0.580838 -0.805311
C -3.022319 0.440900 1.753623
C -3.978402 -0.006572 -0.411557
C -4.093323 0.504429 0.873378
F -0.819682 -0.193816 2.187160
F -3.141411 0.934066 2.974559
F -5.227145 1.055466 1.260940
F -5.003117 0.065015 -1.243245
F -2.682123 -1.050359 -2.043597

Py_PhI_ArF5.log
Energy (E) = -1316.79893237 Hartree
Enthalpy (H) = -1316.564168 Hartree
Gibbs free energy (G) = -1316.640914 Hartree
Charge = 0, Spin = 1
C 2.130223 0.922163 1.001962
C 0.932210 1.196047 0.356252
C 0.088840 2.220287 0.741261
C 0.440112 3.016629 1.823713
C 1.632434 2.777937 2.489801
C 2.462066 1.745687 2.078351
C 3.044874 -0.189559 0.640952
C 3.297422 -2.415692 0.209022
C 4.668306 -2.284692 0.045428
C 5.232950 -1.025626 0.194835
C 4.409890 0.042864 0.501584
H -0.820016 2.420928 0.190580
H -0.211014 3.823854 2.128115
H 1.917674 3.393146 3.331851
H 3.382783 1.542536 2.609992
H 2.820016 -3.382842 0.102797
H 5.272961 -3.146961 -0.196660
H 6.296267 -0.878482 0.063349
H 4.799183 1.046582 0.596916
N 2.497986 -1.393898 0.504573
I 0.381752 0.119423 -1.377196
F 2.367165 0.632656 -1.911456
C -1.570904 -0.217860 -0.459699
C -1.776974 -1.294570 0.384328
C -2.655839 0.590246 -0.742744
C -3.020131 -1.561106 0.937484
F -0.776726 -2.111710 0.692022
C -3.911974 0.353200 -0.208850
F -2.515726 1.652627 -1.540415
C -4.089291 -0.731660 0.635833
F -3.199113 -2.596293 1.744496
F -4.935470 1.146975 -0.489462
F -5.280342 -0.974995 1.155410

PyIBCMe2O_D_ArF5.log
Energy (E) = -1178.55112457 Hartree
Enthalpy (H) = -1178.324564 Hartree
Gibbs free energy (G) = -1178.396584 Hartree
Charge = 0, Spin = 1
C 1.338407 2.674909 -1.671060
C 2.707085 2.773549 -1.460698
C 2.802597 0.750672 -0.399007
C 1.437918 0.603590 -0.559664
H 0.804552 3.456279 -2.191750
H 3.250635 3.643857 -1.808399
O 2.826303 -1.518570 0.249916
I 0.716633 -1.184983 0.304500
C 0.672011 1.554732 -1.191059

H -0.395196 1.439191 -1.319757
N 3.425651 1.841985 -0.837504
C 3.568505 -0.342745 0.332699
C 4.921548 -0.573695 -0.323862
H 5.519430 0.336045 -0.310418
H 5.437929 -1.362183 0.222215
H 4.781241 -0.895939 -1.354749
C 3.743504 0.106602 1.785924
H 4.275055 -0.672434 2.331951
H 4.306576 1.038825 1.840651
H 2.769919 0.259975 2.257890
C -1.360199 -0.401807 0.134844
C -1.796152 0.630532 0.943450
C -2.288498 -1.002258 -0.691842
C -3.110764 1.065951 0.939180
C -3.615396 -0.598161 -0.722112
C -4.022604 0.441046 0.099783
F -0.933218 1.257833 1.745608
F -3.507390 2.062887 1.716556
F -5.282868 0.840076 0.083622
F -4.491969 -1.187618 -1.521684
F -1.930044 -2.004550 -1.495476

PyrroleNMeIBCMe2O_A_ArF5.log
Energy (E) = -1179.74231624 Hartree
Enthalpy (H) = -1179.492234 Hartree
Gibbs free energy (G) = -1179.566617 Hartree
Charge = 0, Spin = 1
C 1.792460 2.549187 1.148480
C 0.719634 1.819072 0.703657
C 1.289227 0.643755 0.175808
C 2.643788 0.662295 0.309901
H 1.827639 3.525967 1.601068
H -0.316341 2.104653 0.748212
I 0.549330 -1.110756 -0.635142
O 2.703158 -1.333846 -0.883297
N 2.951401 1.847918 0.917063
C 4.274235 2.351110 1.226454
H 4.866303 1.589078 1.729265
H 4.797741 2.667624 0.325336
H 4.169650 3.203825 1.892723
C 3.504863 -0.519130 -0.087928
C 4.721412 -0.110061 -0.916370
H 5.463403 0.434090 -0.329891
H 5.182971 -1.021576 -1.294675
H 4.409194 0.497553 -1.765078
C 3.940965 -1.282431 1.166526
H 4.520815 -2.153586 0.861168
H 4.548870 -0.671452 1.837970
H 3.057694 -1.622835 1.709149
C -1.498257 -0.394829 -0.226554
C -2.287288 -0.985024 0.740579
C -2.047229 0.640431 -0.958864
C -3.587456 -0.567156 0.981950
C -3.340471 1.088349 -0.743124
C -4.111147 0.475419 0.233900
F -1.816040 -1.990699 1.478358
F -4.330157 -1.147664 1.913888
F -5.348913 0.887847 0.452061
F -3.847616 2.087668 -1.451217
F -1.325709 1.254643 -1.896676

Anth_I_Cy.log
Energy (E) = -884.547642933 Hartree
Enthalpy (H) = -884.181081 Hartree
Gibbs free energy (G) = -884.250973 Hartree
Charge = 0, Spin = 1
C -1.257984 4.241349 -0.421021
C -2.114904 3.463440 0.288820
C -2.002940 2.042066 0.278791
C -0.953823 1.422891 -0.481022
C -0.085801 2.281205 -1.223151
C -0.234583 3.632705 -1.197139
C -2.885613 1.250291 1.002257
C -0.874944 0.029056 -0.460220
C -1.793792 -0.784679 0.207894
C -2.817195 -0.137970 0.976402
C -3.741710 -0.932205 1.715662
H -4.498031 -0.421583 2.298840
C -3.675670 -2.288006 1.683992
C -2.690005 -2.929917 0.885814
C -1.782698 -2.211293 0.171539
H -3.657717 1.728688 1.594598
H -1.354972 5.318120 -0.410592
H -2.911399 3.903623 0.875986
H 0.680310 1.842468 -1.846457
H 0.427106 4.254034 -1.785211
H -4.381882 -2.884263 2.245545
H -2.677339 -4.009769 0.827602
H -1.105566 -2.703853 -0.509473
I 0.848630 -0.889719 -1.269825
F -0.703760 -1.814672 -2.520010
C 2.065561 0.122428 0.252138
C 1.567024 -0.178100 1.650897
C 3.485388 -0.374572 0.046503
H 2.002887 1.193101 0.046208
C 2.501885 0.445408 2.685256
H 1.538521 -1.265343 1.793987
H 0.548480 0.190490 1.786991
C 4.420205 0.246895 1.082593
H 3.502675 -1.465808 0.162242
H 3.834429 -0.151600 -0.965269
C 3.929262 -0.052960 2.493943
H 2.147100 0.223445 3.692615
H 2.482727 1.534305 2.571241
H 5.437052 -0.121075 0.939264
H 4.445828 1.331023 0.932504
H 4.594432 0.398366 3.231251
H 3.955823 -1.135402 2.659253

AnthI8BA_B_Cy.log
Energy (E) = -1090.40390198 Hartree
Enthalpy (H) = -1089.946306 Hartree
Gibbs free energy (G) = -1090.027285 Hartree
Charge = 0, Spin = 1
C -7.190260 -0.037120 0.218580
C -6.209090 -0.851529 0.689590
C -4.839598 -0.602639 0.378339
C -4.516462 0.525772 -0.443114
C -5.575218 1.355126 -0.917392
C -6.868477 1.084308 -0.598327
C -3.817170 -1.423609 0.847228
C -3.183755 0.780607 -0.754860
C -2.166442 -0.043702 -0.281204
C -2.483804 -1.169542 0.533157
C -1.423321 -1.998962 0.985478
H -1.684476 -2.859721 1.591394
C -0.100464 -1.797074 0.691472
C 0.172079 -0.634456 -0.094576
H -4.064404 -2.279953 1.464711
H -8.225246 -0.236727 0.460841
H -6.447731 -1.706684 1.309798
H -5.323436 2.205193 -1.539586
H -7.663707 1.719291 -0.964505
H -2.936187 1.633591 -1.377192
I 2.133885 0.019054 -0.600463
C -0.795539 0.192496 -0.570546
H -0.544220 1.047093 -1.187445
C 0.871419 -2.849659 1.178481
H 0.982401 -3.586625 0.380258
H 0.356331 -3.364162 1.990046
C 2.262387 -2.414484 1.667221
H 2.253817 -1.345989 1.892977
H 2.444793 -2.898634 2.626311
C 3.424441 -2.782232 0.722390
H 4.152398 -1.966540 0.685893
H 3.953325 -3.658405 1.089592
C 2.988965 -3.109748 -0.711635
O 2.220484 -2.238682 -1.285534
O 3.348346 -4.160814 -1.214314
C 1.737323 2.030073 0.216036
C 1.711964 3.056895 -0.899806
C 2.808821 2.340882 1.245307
H 0.760630 1.941539 0.694554
C 1.510433 4.457974 -0.324874
H 2.666118 3.025020 -1.439438
H 0.932138 2.821285 -1.627138
C 2.602122 3.743580 1.813704
H 2.801510 1.594506 2.042015
H 3.795186 2.291814 0.767644
C 2.592340 4.781305 0.698273
H 1.504427 5.193788 -1.129840
H 0.530404 4.505192 0.161066
H 3.379076 3.967154 2.545540
H 1.644241 3.774459 2.342881
H 3.568038 4.783752 0.200878
H 2.441863 5.780300 1.108543

FuranIBCMe2S_A_Cy.log
Energy (E) = -990.885342978 Hartree
Enthalpy (H) = -990.572368 Hartree
Gibbs free energy (G) = -990.636743 Hartree
Charge = 0, Spin = 1
C -1.420500 2.907381 0.751742
C -0.328349 2.110896 0.810146
C -0.802148 0.825119 0.410597
C -2.122207 0.922219 0.133631
O -2.503572 2.204799 0.341555
H -1.578919 3.945771 0.979754
H 0.660508 2.402764 1.115324
I 0.200920 -0.984229 0.180138
S -2.516385 -1.722040 0.069362
C -3.106777 -0.068924 -0.394941
C -4.484218 0.176076 0.218095
H -4.863827 1.162744 -0.056386
H -5.172489 -0.582665 -0.153071
H -4.439729 0.097221 1.303212
C -3.181447 0.081228 -1.917419
H -3.884932 -0.650562 -2.314009
H -3.514672 1.085325 -2.195347
H -2.204898 -0.107999 -2.365052
C 2.165896 0.071299 0.306982
C 2.413381 0.901258 -0.933965
C 3.213692 -1.010792 0.473738
H 2.112578 0.686570 1.207326
C 3.799986 1.540132 -0.865511
H 2.366055 0.243861 -1.809854
H 1.639386 1.660019 -1.063103
C 4.606315 -0.383218 0.532055
H 3.023693 -1.603935 1.370831
H 3.166643 -1.693237 -0.384793
C 4.871136 0.467456 -0.704746
H 3.982809 2.138593 -1.759033
H 3.839577 2.221950 -0.009141
H 5.363845 -1.161203 0.635599
H 4.671096 0.248302 1.423975

H 4.865245 -0.176617 -1.590541
H 5.861450 0.920702 -0.647674

Indole_NMe_IBCONAc_A_Cy.log
Energy (E) = -968.998596640 Hartree
Enthalpy (H) = -968.614699 Hartree
Gibbs free energy (G) = -968.692077 Hartree
Charge = 0, Spin = 1
C -0.187287 1.997115 0.279159
C -1.355450 2.762562 0.035603
C -1.366032 4.155435 0.107280
C -0.189124 4.793526 0.435754
C 0.977471 4.057300 0.694051
C 0.990692 2.680334 0.619731
C -0.619987 0.648505 0.104950
H -2.273573 4.711167 -0.086202
H -0.163738 5.872425 0.500684
H 1.885423 4.581083 0.959904
H 1.907512 2.152311 0.834914
N -2.402811 1.923778 -0.246018
C -1.959441 0.630629 -0.190905
C -2.816806 -0.591246 -0.371333
O -3.950318 -0.492681 -0.814988
I 0.354436 -1.187185 0.270938
C -3.752212 2.405423 -0.490781
H -3.753354 3.061908 -1.360932
H -4.395558 1.553761 -0.675294
H -4.104192 2.965296 0.375877
N -2.110814 -1.681010 0.005129
C -2.674082 -2.943968 0.049002
C -1.644839 -4.037222 0.277206
H -1.119089 -3.871913 1.219986
H -2.145025 -5.000424 0.308034
H -0.905283 -4.036541 -0.526408
O -3.852021 -3.211022 -0.048866
C 2.405571 -0.416886 0.220944
C 2.693726 0.257275 -1.104085
C 3.308878 -1.617646 0.438298
H 2.498163 0.267277 1.063005
C 4.144903 0.732594 -1.120546
H 2.542152 -0.473487 -1.906235
H 2.008354 1.084945 -1.288420
C 4.767761 -1.159326 0.410836
H 3.084393 -2.114369 1.384616
H 3.144619 -2.345521 -0.365313
C 5.090859 -0.441232 -0.894541
H 4.364238 1.230663 -2.065150
H 4.286210 1.475378 -0.327224
H 5.427805 -2.014981 0.555727
H 4.937892 -0.477692 1.250212
H 4.986922 -1.145879 -1.726155
H 6.127499 -0.103365 -0.892012

Me_Pyrrole_3I_Cy.log
Energy (E) = -594.779856147 Hartree
Enthalpy (H) = -594.500850 Hartree
Gibbs free energy (G) = -594.561833 Hartree
Charge = 0, Spin = 1
C -1.522968 1.347585 0.346556
C -1.591688 0.017203 0.015009
C -2.910632 -0.294539 -0.374942
C -3.614729 0.876288 -0.261505
N -2.773427 1.865931 0.168800
H -0.712472 1.967696 0.686367
H -3.249518 -1.269697 -0.672761
H -4.653555 1.086790 -0.452216
C -3.165333 3.220047 0.490999
H -4.000563 3.512680 -0.141199
H -3.464012 3.311951 1.535273
H -2.335156 3.896831 0.300306
I -0.024358 -1.355808 0.035809
F -1.688746 -2.745356 -0.268138
C 1.455111 0.243322 0.284274
C 2.751806 -0.440910 0.684037
C 1.633448 1.045464 -0.989886
H 1.113724 0.873149 1.109092
C 3.863621 0.592061 0.857562
H 3.042348 -1.148830 -0.103225
H 2.618766 -1.016670 1.602640
C 2.732986 2.089185 -0.809772
H 1.921117 0.358586 -1.794332
H 0.694637 1.511579 -1.293897
C 4.042345 1.418091 -0.411016
H 4.796918 0.098035 1.131095
H 3.598691 1.257827 1.685604
H 2.859707 2.667726 -1.726003
H 2.435453 2.793166 -0.024436
H 4.828390 2.161510 -0.272256
H 4.365398 0.759387 -1.224203

NaphIBMeUreaMe_Cy.log
Energy (E) = -933.077710294 Hartree
Enthalpy (H) = -932.669663 Hartree
Gibbs free energy (G) = -932.744796 Hartree
Charge = 0, Spin = 1
C 1.982103 -2.066992 -0.421451
C 3.057893 -1.441623 0.253616
C 2.933622 -0.100984 0.675415
C 1.791184 0.645404 0.465784
C 0.717356 -0.036066 -0.172148
C 0.798274 -1.319043 -0.615213
H 3.781172 0.358929 1.165507
H -0.041773 -1.789932 -1.110582
I -1.112278 0.982157 -0.367598
C 1.222345 3.014347 0.057893
O 1.507739 4.168342 0.358120
N 0.520786 2.594972 -0.999925
C 0.001804 3.667858 -1.821940
H 0.809821 4.283015 -2.222101
H -0.551477 3.242415 -2.661714
H -0.659975 4.344120 -1.267975
N 1.681803 1.957100 0.914933
C 2.573915 2.383824 1.977710
H 2.606717 1.615179 2.748776
H 3.589259 2.578733 1.619749
H 2.189191 3.310678 2.388387
C 4.245471 -2.184651 0.464783
C 4.348933 -3.475490 0.025184
C 3.271670 -4.094963 -0.647431
C 2.112534 -3.402617 -0.865295
H 5.071680 -1.708373 0.977990
H 5.262329 -4.030657 0.191242
H 3.368891 -5.116552 -0.988337
H 1.279409 -3.865128 -1.380695
C -2.279209 -0.790613 0.304209
C -2.945600 -1.463251 -0.880711
C -3.302735 -0.301073 1.312401
H -1.551242 -1.448419 0.782691
C -3.827615 -2.619653 -0.413397
H -3.570170 -0.727419 -1.402241
H -2.205922 -1.810542 -1.605219
C -4.191115 -1.454904 1.775023
H -2.808289 0.170082 2.163281
H -3.934164 0.465116 0.843377
C -4.866611 -2.129156 0.587292
H -4.308136 -3.094704 -1.269861
H -3.198001 -3.377895 0.063698
H -4.932405 -1.092765 2.488572
H -3.571511 -2.188730 2.300554
H -5.527285 -1.408702 0.093101
H -5.492726 -2.956122 0.924110

NpthIBCMe2O_C_Cy.log
Energy (E) = -823.645308879 Hartree
Enthalpy (H) = -823.251108 Hartree
Gibbs free energy (G) = -823.320781 Hartree
Charge = 0, Spin = 1
C -3.075679 -0.378573 0.067980
C -1.852624 -0.992109 -0.013028
C -0.721244 -0.164729 -0.068260
C -0.778140 1.194621 -0.046603
H -3.976429 -0.978537 0.124121
H 0.105615 1.815131 -0.091210
O -0.439331 -2.788681 -0.616103
I 1.115932 -1.245948 -0.163095
C -2.166840 3.241127 0.032805
C -3.399809 3.828165 0.102628
C -4.564382 3.030391 0.164660
C -4.469237 1.666238 0.154423
C -3.206815 1.029495 0.082875
C -2.041009 1.831859 0.023712
H -1.268585 3.844848 -0.015381
H -3.488889 4.906026 0.111068
H -5.534256 3.506034 0.219785
H -5.358590 1.049734 0.199871
C -1.639760 -2.511874 -0.006451
C -2.753837 -3.222105 -0.772914
H -2.506330 -4.282565 -0.806066
H -3.729630 -3.110219 -0.296689
H -2.802028 -2.843890 -1.793358
C -1.641719 -2.977531 1.457983
H -2.591268 -2.762482 1.952582
H -1.457002 -4.052002 1.478846
H -0.843206 -2.479465 2.012521
C 2.343450 0.547480 0.247880
C 2.648378 1.310792 -1.027587
C 3.621298 0.028423 0.886310
H 1.801110 1.160830 0.970477
C 3.594566 2.474978 -0.742377
H 3.130774 0.625416 -1.734657
H 1.734561 1.658916 -1.513262
C 4.581303 1.183096 1.168500
H 3.398936 -0.517891 1.805064
H 4.108095 -0.678355 0.200968
C 4.881796 1.966835 -0.103621
H 3.809410 3.021899 -1.661606
H 3.104752 3.176483 -0.058195
H 5.501981 0.805375 1.615544
H 4.119528 1.852140 1.902002
H 5.396646 1.311727 -0.814557
H 5.556886 2.796091 0.111506

NpthISO2NMe_D_Cy.log
Energy (E) = -1273.62963395 Hartree
Enthalpy (H) = -1273.266936 Hartree
Gibbs free energy (G) = -1273.339176 Hartree
Charge = 0, Spin = 1
C 0.229441 3.403412 -0.939984
C 1.476776 3.582529 -0.422797
C 2.237930 2.488674 0.048116
C 1.723511 1.152770 0.005867
C 0.383728 1.043045 -0.463579
C -0.321461 2.109648 -0.947473
H 3.877801 3.769846 0.572689
H -0.343425 4.232509 -1.330085

H 1.922881 4.567837 -0.376709
C 3.538303 2.742012 0.547302
C 2.619614 0.115831 0.402012
H -1.318603 1.969347 -1.339205
C 3.876637 0.401813 0.860981
C 4.341734 1.724498 0.964351
H 4.519744 -0.430538 1.112324
H 5.336775 1.918915 1.338942
I -0.755249 -0.757988 -0.408979
S 2.270309 -1.628552 0.322425
O 1.475131 -1.948296 1.499800
O 3.536348 -2.326284 0.220860
N 1.446920 -1.650467 -1.040096
C 1.201719 -2.996089 -1.544594
H 0.777332 -2.917694 -2.545222
H 2.127989 -3.569696 -1.610808
H 0.501990 -3.569905 -0.917912
C -2.596916 0.283022 0.251585
C -3.159356 -0.534915 1.400252
C -3.588392 0.393510 -0.890438
H -2.269336 1.261702 0.604878
C -4.471698 0.077022 1.887771
H -3.351735 -1.559006 1.055813
H -2.435040 -0.602822 2.212409
C -4.899033 1.005991 -0.398840
H -3.178414 0.973818 -1.719794
H -3.789133 -0.610683 -1.282931
C -5.476815 0.186231 0.748102
H -4.876542 -0.518074 2.706971
H -4.269710 1.076225 2.287049
H -5.610003 1.076793 -1.223007
H -4.707302 2.026481 -0.051185
H -5.718290 -0.818203 0.384503
H -6.408289 0.629226 1.101937

perF_CMe2O_Cy.log
Energy (E) = -1066.93027003 Hartree
Enthalpy (H) = -1066.613923 Hartree
Gibbs free energy (G) = -1066.685768 Hartree
Charge = 0, Spin = 1
C 1.713015 2.436822 -0.050586
C 2.949268 1.894365 0.247922
C 3.101280 0.516225 0.287125
C 2.045422 -0.341957 0.029033
C 0.804440 0.225456 -0.192611
C 0.633857 1.592567 -0.262105
O 1.077810 -2.364244 -0.707553
I -0.787839 -1.207512 -0.302060
F -0.530610 2.166833 -0.566895
F 1.561667 3.750332 -0.136946
F 3.980978 2.691655 0.474163
F 4.312348 0.055294 0.600090
C 2.172070 -1.885487 -0.041391
C 3.415246 -2.290484 -0.838102
H 4.347064 -2.069399 -0.323060
H 3.341882 -3.365630 -0.997459
H 3.407568 -1.801665 -1.811924
C 2.256143 -2.418121 1.393874
H 2.336753 -3.504401 1.353708
H 3.118678 -2.012717 1.925917
H 1.348537 -2.159391 1.943360
C -2.413076 0.196789 0.242085
C -3.329943 -0.633435 1.124123
C -3.141903 0.690005 -0.991836
H -1.977484 1.013288 0.815264
C -4.561494 0.180631 1.520210
H -3.657198 -1.525520 0.572192
H -2.800417 -0.983516 2.012037
C -4.362268 1.516430 -0.590036
H -2.478446 1.265907 -1.634836
H -3.479837 -0.179035 -1.570117
C -5.296708 0.694713 0.289030
H -5.222317 -0.423458 2.143274
H -4.238013 1.032370 2.127167
H -4.882123 1.873514 -1.479998
H -4.027188 2.400358 -0.037637
H -5.671450 -0.158029 -0.287265
H -6.164582 1.285260 0.584858

perF_NMeCO2_Cy.log
Energy (E) = -1156.98481903 Hartree
Enthalpy (H) = -1156.696177 Hartree
Gibbs free energy (G) = -1156.768959 Hartree
Charge = 0, Spin = 1
C 1.490397 2.577207 -0.339763
C 2.662632 2.163257 0.272029
C 2.876366 0.826722 0.564470
C 1.917036 -0.162478 0.306942
C 0.728865 0.302072 -0.276217
C 0.530847 1.624573 -0.624049
I -0.787604 -1.073682 -0.653019
F -0.595091 2.013609 -1.223475
F 1.299882 3.851604 -0.649225
F 3.607722 3.049702 0.533964
F 4.065457 0.501646 1.062400
N 2.131008 -1.493609 0.592910
C 2.927760 -1.888210 1.748663
H 2.827596 -1.137714 2.530530
H 2.545548 -2.840454 2.104005
H 3.980072 -2.018536 1.502623
C 1.938025 -2.513651 -0.430409
O 2.433421 -3.598849 -0.198597
O 1.260547 -2.119761 -1.434510
C -2.361299 0.142574 0.290212
C -2.958846 -0.651454 1.435847
C -3.397900 0.523716 -0.747064
H -1.831622 1.020091 0.662019
C -4.078106 0.159200 2.088784
H -3.375007 -1.589654 1.050256
H -2.192371 -0.913908 2.165932
C -4.514021 1.329060 -0.083306
H -2.940182 1.087653 -1.559073
H -3.826084 -0.388247 -1.179732
C -5.137667 0.544341 1.064076
H -4.516625 -0.411671 2.907560
H -3.650340 1.067704 2.524621
H -5.266031 1.598005 -0.825387
H -4.095665 2.264343 0.301925
H -5.602074 -0.364711 0.667761
H -5.929253 1.125763 1.537163

Py_PhI_Cy.log
Energy (E) = -824.428552134 Hartree
Enthalpy (H) = -824.087524 Hartree
Gibbs free energy (G) = -824.156913 Hartree
Charge = 0, Spin = 1
C 1.725412 0.997611 0.552676
C 0.564035 1.326485 -0.133408
C 0.049700 2.608836 -0.144332
C 0.713705 3.616002 0.547426
C 1.885629 3.322447 1.226182
C 2.383241 2.025898 1.223062
C 2.276795 -0.379235 0.617245
C 1.966030 -2.556589 1.227032
C 3.250560 -2.904846 0.841941
C 4.073031 -1.917413 0.314442
C 3.580306 -0.631248 0.199938
H -0.843461 2.835125 -0.711265
H 0.321282 4.623367 0.536531
H 2.414855 4.100362 1.759090
H 3.290887 1.788302 1.763338
H 1.295532 -3.301244 1.640620
H 3.592625 -3.924628 0.947390
H 5.075933 -2.152491 -0.015004
H 4.162392 0.164608 -0.241513
N 1.481543 -1.319189 1.122913
I -0.425524 -0.121347 -1.296960
F 1.626414 -0.122086 -2.120143
C -2.230358 0.146469 -0.086497
C -3.316518 -0.705445 -0.717587
C -1.972188 -0.253838 1.350912
H -2.498527 1.203746 -0.151977
C -4.605270 -0.600093 0.096408
H -2.987125 -1.752149 -0.729276
H -3.493458 -0.409195 -1.754584
C -3.259283 -0.136850 2.163172
H -1.615679 -1.289536 1.370864
H -1.174980 0.352270 1.785438
C -4.360045 -0.993963 1.548402
H -5.382512 -1.223277 -0.348180
H -4.962761 0.434228 0.059826
H -3.077635 -0.427027 3.198886
H -3.583362 0.909433 2.175705
H -5.281981 -0.907147 2.125062
H -4.056463 -2.045629 1.586771

PyIBCMe2O_D_Cy.log
Energy (E) = -686.174875362 Hartree
Enthalpy (H) = -685.842101 Hartree
Gibbs free energy (G) = -685.905713 Hartree
Charge = 0, Spin = 1
C 1.385634 3.135875 0.261482
C 2.740026 2.839944 0.308134
C 2.370836 0.593659 0.046451
C 1.005663 0.816409 0.004003
H 1.040921 4.156826 0.339497
H 3.468794 3.632502 0.432495
O 1.918476 -1.622497 -0.629208
I -0.154164 -0.955493 -0.161783
C 0.485149 2.087728 0.119589
H -0.576874 2.281787 0.098365
N 3.224227 1.604550 0.212706
C 2.890448 -0.838260 -0.048657
C 4.154664 -0.873577 -0.902529
H 4.937868 -0.244568 -0.481869
H 4.497511 -1.906648 -0.952799
H 3.924000 -0.534919 -1.912211
C 3.207491 -1.296872 1.381743
H 3.579288 -2.320816 1.341511
H 3.954306 -0.654880 1.851424
H 2.298614 -1.284088 1.988770
C -2.045567 0.113958 0.262526
C -2.990189 -0.942448 0.811053
C -2.614274 0.748690 -0.992640
H -1.843336 0.855609 1.038633
C -4.363695 -0.334655 1.091583
H -3.102680 -1.746529 0.071368
H -2.579693 -1.394661 1.716140
C -3.978716 1.372310 -0.706849
H -1.928214 1.485542 -1.415935
H -2.732508 -0.032739 -1.752638
C -4.936391 0.320284 -0.159781
H -5.042228 -1.099445 1.471947
H -4.259449 0.420978 1.877097
H -4.382884 1.829239 -1.611289
H -3.860959 2.172365 0.032189

H -5.095647 -0.447135 -0.924828
H -5.910001 0.762576 0.054734

PyrroleNMeIBCMe2O_A_Cy.log
Energy (E) = -687.368692223 Hartree
Enthalpy (H) = -687.012458 Hartree
Gibbs free energy (G) = -687.078767 Hartree
Charge = 0, Spin = 1
C 1.717229 2.685855 0.382864
C 0.502282 2.052882 0.324399
C 0.813191 0.694634 0.085758
C 2.163143 0.522834 0.021135
H 1.954130 3.727259 0.524732
H -0.461942 2.517218 0.435643
I -0.337184 -1.030744 -0.056642
O 1.807880 -1.724350 -0.467716
N 2.717181 1.761800 0.205692
C 4.122338 2.108337 0.151358
H 4.713159 1.410807 0.740558
H 4.492179 2.103469 -0.873292
H 4.245947 3.105607 0.567287
C 2.811371 -0.845007 -0.131764
C 3.869079 -0.854948 -1.240455
H 4.757897 -0.270532 -0.993727
H 4.168605 -1.892026 -1.390248
H 3.435597 -0.484757 -2.169526
C 3.444577 -1.256256 1.205743
H 3.867188 -2.254794 1.089605
H 4.233211 -0.576581 1.539416
H 2.667048 -1.295403 1.969977
C -2.177012 0.122628 0.278781
C -3.263733 -0.845191 0.709824
C -2.576594 0.875514 -0.975311
H -1.946263 0.810021 1.096326
C -4.576222 -0.095844 0.935644
H -3.412876 -1.594424 -0.078634
H -2.968734 -1.382077 1.613776
C -3.878154 1.638359 -0.740087
H -1.778880 1.546729 -1.299136
H -2.727794 0.151087 -1.784182
C -4.983451 0.679464 -0.312262
H -5.361967 -0.792915 1.230467
H -4.441618 0.605537 1.765620
H -4.166374 2.182651 -1.640591
H -3.720678 2.382601 0.048321
H -5.174558 -0.027840 -1.126505
H -5.914095 1.220413 -0.135739

Anth_I_SetBu.log
Energy (E) = -816.528600557 Hartree
Enthalpy (H) = -816.198268 Hartree
Gibbs free energy (G) = -816.272501 Hartree
Charge = 0, Spin = 1
C 2.437336 3.905851 -0.493980
C 3.065303 2.805955 -0.982556
C 2.599338 1.494759 -0.674665
C 1.440775 1.332738 0.155194
C 0.816504 2.516539 0.649284
C 1.299600 3.749904 0.341685
C 3.251052 0.373503 -1.172791
C 1.022555 0.026480 0.419990
C 1.695368 -1.114787 -0.021977
C 2.837269 -0.916685 -0.866964
C 3.531544 -2.048900 -1.384788
H 4.386622 -1.869613 -2.024595
C 3.133233 -3.310698 -1.082375
C 2.018573 -3.509442 -0.223604
C 1.328150 -2.457416 0.292067
H 4.114581 0.510229 -1.814122
H 2.798476 4.897111 -0.729936
H 3.939988 2.896085 -1.614553
H -0.042485 2.438052 1.298695
H 0.809919 4.627295 0.741242
H 3.664991 -4.165025 -1.477806
H 1.725170 -4.516462 0.039548
H 0.529887 -2.634763 0.995782
I -0.758017 -0.256709 1.523194
F 0.535095 -1.267002 2.867267
C -2.733437 -0.572111 -1.491942
C -3.506033 0.027920 -2.655887
C -3.622681 -1.493473 -0.677190
C -1.502891 -1.308012 -1.988370
H -2.875330 0.695718 -3.243356
H -3.858150 -0.776004 -3.308996
H -4.374286 0.588010 -2.306594
H -3.947006 -2.332941 -1.300054
H -3.087891 -1.907011 0.179804
H -4.505883 -0.968293 -0.314632
H -1.808306 -2.106325 -2.671790
H -0.824785 -0.636391 -2.515623
H -0.956232 -1.772530 -1.166008
Se -2.205869 1.030256 -0.416132

AnthI8BA_B_SetBu.log
Energy (E) = -1022.39117605 Hartree
Enthalpy (H) = -1021.969568 Hartree
Gibbs free energy (G) = -1022.053260 Hartree
Charge = 0, Spin = 1
C 7.244076 0.318887 0.285054
C 6.216851 1.020245 0.833732
C 4.870874 0.784996 0.425452
C 4.621155 -0.206904 -0.577633
C 5.726276 -0.920730 -1.127780
C 6.995298 -0.667516 -0.711554
C 3.801671 1.489657 0.973627
C 3.312099 -0.447154 -0.986644
C 2.248463 0.262998 -0.436119
C 2.492624 1.249646 0.563324
C 1.383554 1.956551 1.100583
H 1.584247 2.709023 1.855155
C 0.084075 1.759828 0.716096
C -0.109171 0.738701 -0.266220
H 3.993450 2.240865 1.731661
H 8.260779 0.506576 0.602593
H 6.400327 1.772037 1.591371
H 5.529188 -1.668617 -1.885847
H 7.826448 -1.214044 -1.135898
H 3.117983 -1.198770 -1.743452
I -2.052070 0.134053 -0.931420
C 0.901264 0.026285 -0.820581
H 0.706394 -0.752144 -1.546892
C -0.964377 2.649908 1.343421
H -1.176021 3.469298 0.654061
H -0.481923 3.108180 2.206072
C -2.279984 1.991151 1.796887
H -2.153980 0.907019 1.818586
H -2.462671 2.271015 2.833354
C -3.523853 2.383674 0.974352
H -4.167455 1.512168 0.824430
H -4.117180 3.126203 1.501218
C -3.198027 2.998250 -0.382414
O -2.376446 2.331510 -1.157569
O -3.648865 4.081009 -0.690082
C -1.629572 -2.745777 1.290078
C -1.410446 -4.236290 1.499836
C -3.010615 -2.338515 1.769670
C -0.548774 -1.944897 1.993316
H -0.426128 -4.544237 1.145422
H -1.474244 -4.460921 2.568431
H -2.168298 -4.824309 0.981159
H -3.089931 -2.526103 2.844658
H -3.196125 -1.274534 1.607019
H -3.788126 -2.906972 1.260179
H -0.578756 -2.162158 3.065361
H 0.442340 -2.195501 1.613936
H -0.697149 -0.870689 1.869682
Se -1.478286 -2.534303 -0.695727

FuranIBCMe2S_A_SetBu.log
Energy (E) = -922.880376539 Hartree
Enthalpy (H) = -922.603098 Hartree
Gibbs free energy (G) = -922.671508 Hartree
Charge = 0, Spin = 1
C 1.494862 2.991683 0.150876
C 0.427756 2.171910 0.289983
C 0.967675 0.866190 0.119242
C 2.297964 0.969216 -0.087996
O 2.629940 2.278950 -0.073695
H 1.600624 4.060798 0.174349
H -0.595832 2.436420 0.488397
I 0.084410 -1.007869 0.254688
S 2.545814 -1.626745 -0.649923
C 3.363489 -0.067700 -0.191831
C 4.367471 0.295866 -1.284266
H 4.871092 1.231236 -1.033036
H 5.115525 -0.492349 -1.366153
H 3.869276 0.404015 -2.246018
C 4.071613 -0.195802 1.158186
H 4.840924 -0.964956 1.093518
H 4.538403 0.752689 1.434197
H 3.362582 -0.482268 1.935041
C -3.240185 0.225398 -0.797645
C -4.631139 0.779736 -0.526911
C -3.340116 -1.146918 -1.439809
C -2.454698 1.178296 -1.680792
H -4.580063 1.757654 -0.046946
H -5.166634 0.890009 -1.475097
H -5.204502 0.108515 0.113216
H -3.875643 -1.065038 -2.390914
H -2.354027 -1.562484 -1.653005
H -3.879626 -1.841852 -0.796839
H -2.943791 1.258169 -2.656693
H -2.407617 2.174315 -1.239471
H -1.434495 0.826515 -1.849115
Se -2.410313 0.097493 1.022065

Indole_NMe_IBCONAc_A_SetBu.log
Energy (E) = -900.982243353 Hartree
Enthalpy (H) = -900.634326 Hartree
Gibbs free energy (G) = -900.715492 Hartree
Charge = 0, Spin = 1
C -0.443102 -2.038583 -0.292414
C -1.619424 -2.751408 0.051586
C -1.669311 -4.146148 0.059510
C -0.525774 -4.829390 -0.292201
C 0.645102 -4.141549 -0.653288
C 0.700541 -2.764544 -0.659688
C -0.828595 -0.672419 -0.174915
H -2.578520 -4.666607 0.328561
H -0.530126 -5.910696 -0.298336
H 1.521563 -4.706902 -0.938841
H 1.605023 -2.253357 -0.951160
N -2.629218 -1.867965 0.338316
C -2.145387 -0.597545 0.190600
C -2.912363 0.667452 0.356548

O -4.067581 0.682186 0.735008
I 0.126269 1.159162 -0.446339
C -3.972455 -2.283089 0.703492
H -3.936658 -2.882305 1.613228
H -4.577730 -1.400289 0.869728
H -4.402767 -2.882756 -0.098570
N -2.107965 1.722178 0.021028
C -2.542740 3.041944 0.021547
C -1.454967 4.045859 -0.305094
H -1.041210 3.859680 -1.297806
H -1.886660 5.041208 -0.279911
H -0.642178 3.989085 0.421607
O -3.675440 3.398675 0.245031
C 3.286820 0.234167 1.076812
C 4.736025 -0.216912 0.973217
C 2.463464 -0.782347 1.845569
C 3.203303 1.605945 1.720010
H 5.333322 0.494750 0.401899
H 5.160303 -0.286878 1.978808
H 4.813120 -1.197400 0.502162
H 2.839512 -0.847593 2.870868
H 1.410965 -0.495876 1.898153
H 2.525476 -1.771563 1.392338
H 3.603733 1.551993 2.736688
H 3.780886 2.339963 1.158726
H 2.171574 1.953204 1.792908
Se 2.697522 0.317111 -0.839074

Me_Pyrrole_3I_SetBu.log
Energy (E) = -526.764902475 Hartree
Enthalpy (H) = -526.521824 Hartree
Gibbs free energy (G) = -526.586642 Hartree
Charge = 0, Spin = 1
C -1.634167 1.284626 0.324953
C -1.696848 -0.032269 -0.045281
C -2.966574 -0.311684 -0.590385
C -3.650956 0.875567 -0.535197
N -2.842362 1.833421 0.012882
H -0.840028 1.854521 0.774848
H -3.300905 -1.261747 -0.962562
H -4.653576 1.115517 -0.846359
C -3.235224 3.194610 0.308863
H -3.948069 3.535337 -0.438585
H -3.691941 3.270225 1.295246
H -2.360840 3.840063 0.274098
I -0.183035 -1.455758 0.147253
F -1.677588 -2.764452 -0.566559
C 2.441503 0.898237 -0.713254
C 3.505858 1.911686 -0.324442
C 1.397798 1.534598 -1.611891
C 3.076206 -0.304605 -1.386882
H 4.249533 1.466804 0.337605
H 4.016640 2.264392 -1.225224
H 3.065155 2.772990 0.178935
H 1.868746 1.850170 -2.547940
H 0.597480 0.835102 -1.860236
H 0.952264 2.409892 -1.138017
H 3.578379 0.016774 -2.304494
H 3.812307 -0.775550 -0.735859
H 2.328431 -1.048638 -1.664612
Se 1.635192 0.375025 1.044373

NaphIBMeUreaMe_SetBu.log
Energy (E) = -865.072892978 Hartree
Enthalpy (H) = -864.700492 Hartree
Gibbs free energy (G) = -864.779383 Hartree
Charge = 0, Spin = 1
C 2.629846 -1.120785 -0.691728
C 3.338904 -0.330784 0.245532
C 2.710729 0.790346 0.830907
C 1.416473 1.150267 0.526759
C 0.735298 0.313363 -0.393300
C 1.293184 -0.768875 -0.989249
H 3.277541 1.387107 1.532989
H 0.713120 -1.395247 -1.655390
I -1.289970 0.778738 -0.754743
C 0.085701 3.217872 0.425754
O -0.066542 4.333204 0.898471
N -0.354919 2.809541 -0.788848
C -1.132845 3.793384 -1.516212
H -0.556275 4.710493 -1.628614
H -1.363925 3.402671 -2.507260
H -2.064783 4.059390 -1.007598
N 0.790357 2.232497 1.155904
C 1.323988 2.681502 2.431002
H 1.534377 1.812457 3.051876
H 2.234567 3.274765 2.314951
H 0.580359 3.310073 2.908723
C 4.673207 -0.686043 0.559017
C 5.261988 -1.770375 -0.031077
C 4.548901 -2.558787 -0.961686
C 3.258672 -2.239971 -1.283985
H 5.219026 -0.081501 1.272714
H 6.282450 -2.030932 0.215384
H 5.027996 -3.414971 -1.416195
H 2.699006 -2.836297 -1.993666
C -2.087498 -2.141778 1.314651
C -2.369115 -3.624433 1.502491
C -3.211216 -1.308915 1.904870
C -0.756947 -1.772158 1.947515
H -1.565227 -4.233421 1.088204
H -2.451762 -3.843345 2.571484
H -3.305790 -3.911134 1.022745
H -3.268345 -1.485374 2.983727
H -3.037871 -0.242277 1.751968
H -4.169895 -1.571769 1.458675
H -0.781558 -2.032901 3.010234
H 0.071422 -2.304320 1.477991
H -0.561668 -0.699416 1.879348
Se -2.028696 -1.907354 -0.676931

NpthIBCMe2O_C_SetBu.log
Energy (E) = -755.639150522 Hartree
Enthalpy (H) = -755.280621 Hartree
Gibbs free energy (G) = -755.354100 Hartree
Charge = 0, Spin = 1
C 3.230139 0.361493 -0.232294
C 2.028548 1.013263 -0.154675
C 0.887701 0.225050 0.059480
C 0.900183 -1.119582 0.219062
H 4.142170 0.925155 -0.391399
H -0.009866 -1.673016 0.421517
O 0.547812 2.786281 -0.652598
I -0.886424 1.405861 0.143332
C 2.228697 -3.203123 0.265724
C 3.438079 -3.834665 0.172102
C 4.614028 -3.087885 -0.061866
C 4.555849 -1.727999 -0.196308
C 3.319448 -1.045577 -0.104672
C 2.140877 -1.797934 0.129968
H 1.320766 -3.765027 0.446650
H 3.499453 -4.909181 0.277939
H 5.564544 -3.599001 -0.133472
H 5.455074 -1.151509 -0.375215
C 1.842322 2.524448 -0.209936
C 2.797497 3.179097 -1.199583
H 2.559900 4.240869 -1.249459
H 3.837357 3.072603 -0.887199
H 2.668634 2.742938 -2.188783
C 2.068013 3.093192 1.193937
H 3.083629 2.897382 1.540973
H 1.894511 4.169123 1.168836
H 1.371234 2.644823 1.905188
C -3.221813 -1.270955 -0.754631
C -4.184121 -2.393965 -0.400400
C -2.106132 -1.789181 -1.643933
C -3.967731 -0.136141 -1.434026
H -4.985293 -2.035195 0.246615
H -4.634232 -2.788153 -1.316692
H -3.668055 -3.209833 0.106980
H -2.526714 -2.126953 -2.596190
H -1.370226 -1.012201 -1.862403
H -1.592123 -2.632032 -1.180098
H -4.427213 -0.504707 -2.356640
H -4.753012 0.257443 -0.789037
H -3.296412 0.680307 -1.704043
Se -2.498990 -0.675351 1.019190

NpthISO2NMe_D_SetBu.log
Energy (E) = -1205.61851727 Hartree
Enthalpy (H) = -1205.291634 Hartree
Gibbs free energy (G) = -1205.367955 Hartree
Charge = 0, Spin = 1
C -0.340966 3.431579 0.304114
C -1.693961 3.580227 0.285510
C -2.546963 2.461172 0.162995
C -2.020505 1.133214 0.058527
C -0.598810 1.048683 0.076577
C 0.205554 2.141063 0.193626
H -4.289876 3.708685 0.224844
H 0.318612 4.282327 0.396994
H -2.148710 4.559531 0.362358
C -3.945377 2.685242 0.147273
C -2.987726 0.090068 -0.041173
H 1.280863 2.028660 0.168595
C -4.331949 0.342415 -0.040595
C -4.828800 1.654226 0.045617
H -5.005640 -0.501607 -0.093367
H -5.894814 1.830579 0.040480
I 0.489852 -0.812229 -0.099879
S -2.582622 -1.621773 -0.207969
O -2.131312 -1.832194 -1.567875
O -3.703286 -2.411869 0.246855
N -1.377164 -1.693684 0.870561
C -1.046090 -3.053240 1.292193
H -0.301896 -2.991000 2.085255
H -1.935231 -3.540895 1.689612
H -0.648394 -3.674762 0.481297
C 3.935040 0.185202 0.576306
C 5.300648 0.701648 0.149413
C 4.042606 -1.258345 1.036766
C 3.360432 1.057108 1.677674
H 5.241697 1.733337 -0.198886
H 5.983305 0.665213 1.003854
H 5.721150 0.088969 -0.648639
H 4.726134 -1.317513 1.889535
H 3.077187 -1.648628 1.363517
H 4.424790 -1.896626 0.240717
H 3.998291 0.989557 2.564155
H 3.316501 2.102694 1.370043
H 2.357855 0.733306 1.966620
Se 2.836355 0.307394 -1.098842

perF_CMe2O_SetBu.log
Energy (E) = -998.914868743 Hartree
Enthalpy (H) = -998.634453 Hartree

Gibbs free energy (G) = -998.710926 Hartree
Charge = 0, Spin = 1
C 1.914552 2.304386 -0.415480
C 2.978766 1.768185 0.287235
C 3.017126 0.406080 0.552482
C 2.003785 -0.435441 0.131409
C 0.910617 0.143118 -0.488523
C 0.866066 1.484061 -0.812464
O 1.126509 -2.463420 -0.673252
I -0.649874 -1.259808 -0.818096
F -0.104165 2.029535 -1.526021
F 1.903484 3.591434 -0.721878
F 3.970164 2.552173 0.675730
F 4.067390 -0.053129 1.231250
C 1.992385 -1.965458 0.290248
C 3.356785 -2.583275 -0.002915
H 4.088920 -2.361949 0.769833
H 3.210359 -3.661233 -0.056786
H 3.724368 -2.238382 -0.968239
C 1.546192 -2.307554 1.713487
H 1.507008 -3.391598 1.817948
H 2.243290 -1.900351 2.447254
H 0.552056 -1.902651 1.919582
C -2.884584 0.508914 1.377500
C -4.031920 1.469379 1.650799
C -1.604760 1.020321 2.016060
C -3.225397 -0.880031 1.885817
H -4.951880 1.129586 1.173631
H -4.207084 1.525027 2.729320
H -3.801679 2.471548 1.288095
H -1.747374 1.110818 3.097240
H -0.770773 0.333049 1.853299
H -1.330458 1.998471 1.619061
H -3.381736 -0.842114 2.968338
H -4.134203 -1.257500 1.418069
H -2.416361 -1.587701 1.696218
Se -2.727139 0.540469 -0.619806

perF_NMeCO2_SetBu.log
Energy (E) = -1088.96287114 Hartree
Enthalpy (H) = -1088.710334 Hartree
Gibbs free energy (G) = -1088.786864 Hartree
Charge = 0, Spin = 1
C -2.069945 -2.293643 -0.401127
C -2.970965 -1.673970 0.449659
C -2.832734 -0.334428 0.778283
C -1.759843 0.430976 0.314001
C -0.842455 -0.243223 -0.496951
C -1.006858 -1.556781 -0.892531
I 0.787843 0.851094 -1.167999
F -0.165783 -2.134093 -1.737582
F -2.236208 -3.559583 -0.748121
F -4.005438 -2.354685 0.910032
F -3.789942 0.213384 1.520128
N -1.605291 1.772341 0.631362
C -2.028108 2.280686 1.932186
H -1.911884 1.494270 2.675890
H -1.388542 3.121143 2.185262
H -3.059441 2.627638 1.927014
C -1.397187 2.753721 -0.396063
O -1.615230 3.912497 -0.118876
O -0.983298 2.271831 -1.521625
C 2.717049 -0.281757 1.590390
C 3.604215 -1.323974 2.254781
C 1.367087 -0.205495 2.281509
C 3.398939 1.072943 1.570061
H 4.562518 -1.415090 1.741608
H 3.800919 -1.018763 3.286002
H 3.122243 -2.301826 2.274399
H 1.520758 0.049170 3.333971
H 0.734565 0.573181 1.848977
H 0.838190 -1.157852 2.228439
H 3.557094 1.412397 2.597707
H 4.365337 1.020755 1.070049
H 2.785378 1.823944 1.069864
Se 2.530694 -1.013337 -0.265767

Py_PhI_SetBu.log
Energy (E) = -756.406966607 Hartree
Enthalpy (H) = -756.101870 Hartree
Gibbs free energy (G) = -756.174486 Hartree
Charge = 0, Spin = 1
C -2.063811 1.263023 -0.104913
C -0.691400 1.163853 0.084382
C 0.137371 2.265845 0.130692
C -0.409977 3.534672 -0.006011
C -1.778188 3.675283 -0.179134
C -2.586748 2.550477 -0.232072
C -2.985899 0.108622 -0.247136
C -3.438372 -1.880338 -1.270759
C -4.658281 -2.000413 -0.622700
C -5.040670 -0.994182 0.253664
C -4.193837 0.082288 0.443906
H 1.203424 2.140940 0.258488
H 0.234622 4.401522 0.030755
H -2.216072 4.658066 -0.286049
H -3.650429 2.652092 -0.405124
H -3.106811 -2.643935 -1.964749
H -5.286721 -2.861554 -0.799397
H -5.979199 -1.054660 0.787697
H -4.434055 0.873565 1.139859
N -2.617240 -0.847814 -1.094835
I 0.169817 -0.728966 0.499664
F -1.505160 -0.856117 1.792720
C 3.736558 -0.193144 0.226620
C 5.000423 -0.011831 -0.599473
C 3.790458 -1.502395 0.994068
C 3.549364 0.976034 1.174813
H 4.964655 0.913422 -1.175788
H 5.866865 0.031485 0.067099
H 5.141135 -0.844069 -1.289857
H 4.647281 -1.491048 1.674944
H 2.895004 -1.648435 1.601204
H 3.895481 -2.349905 0.317338
H 4.377252 1.001096 1.889938
H 3.538363 1.922688 0.633164
H 2.623578 0.884046 1.746643
Se 2.292661 -0.251091 -1.160432

PyIBCMe2O_D_SetBu.log
Energy (E) = -618.168920917 Hartree
Enthalpy (H) = -617.871838 Hartree
Gibbs free energy (G) = -617.939174 Hartree
Charge = 0, Spin = 1
C 1.387530 3.133739 0.219225
C 2.743135 2.929049 0.002668
C 2.492803 0.660426 -0.136781
C 1.133433 0.801233 0.073727
H 0.997091 4.131928 0.353666
H 3.423817 3.771295 -0.032027
O 2.041471 -1.597171 -0.661340
I 0.126501 -1.064247 0.140760
C 0.548285 2.027425 0.273429
H -0.511989 2.115330 0.478015
N 3.289413 1.725437 -0.160496
C 3.066195 -0.740091 -0.262905
C 4.157596 -0.779168 -1.322291
H 4.961212 -0.089944 -1.066904
H 4.547252 -1.794580 -1.381788
H 3.742990 -0.504275 -2.291063
C 3.628581 -1.137051 1.103469
H 4.040146 -2.143758 1.035279
H 4.409513 -0.443676 1.417162
H 2.836579 -1.135617 1.856187
C -3.181078 0.261212 -0.737698
C -4.551649 0.811396 -0.375521
C -3.321006 -1.078705 -1.437890
C -2.427154 1.248276 -1.610397
H -4.468201 1.764567 0.147791
H -5.131757 0.970103 -1.289710
H -5.099174 0.114304 0.259572
H -3.898691 -0.948852 -2.358428
H -2.349389 -1.490416 -1.714805
H -3.836888 -1.799477 -0.804007
H -2.953962 1.370687 -2.561761
H -2.359804 2.225262 -1.130324
H -1.416554 0.899041 -1.834496
Se -2.266307 0.037237 1.033452

PyrroleNMeIBCMe2O_A_SetBu.log
Energy (E) = -619.358043671 Hartree
Enthalpy (H) = -619.037425 Hartree
Gibbs free energy (G) = -619.107163 Hartree
Charge = 0, Spin = 1
C 1.847448 2.685029 0.379045
C 0.652855 2.016083 0.467271
C 0.975380 0.680635 0.161078
C 2.309542 0.549457 -0.078501
H 2.069341 3.731201 0.507204
H -0.311708 2.418831 0.721602
I -0.064891 -1.111663 0.155146
O 1.906702 -1.677961 -0.611796
N 2.848405 1.797830 0.057760
C 4.231150 2.175166 -0.148235
H 4.896574 1.511340 0.400825
H 4.496973 2.145792 -1.204149
H 4.369282 3.187737 0.222690
C 2.946770 -0.803828 -0.297365
C 3.924323 -0.824830 -1.470858
H 4.834359 -0.260095 -1.262046
H 4.198607 -1.863094 -1.654454
H 3.445145 -0.429018 -2.365499
C 3.643325 -1.255460 0.989113
H 4.065515 -2.247907 0.830205
H 4.442769 -0.573990 1.289229
H 2.912099 -1.310723 1.797184
C -3.215645 0.485350 -0.772089
C -4.543301 1.139048 -0.421566
C -3.448913 -0.793902 -1.555269
C -2.340931 1.447335 -1.555206
H -4.392592 2.048642 0.161073
H -5.070766 1.404747 -1.342780
H -5.176690 0.460783 0.151350
H -3.966369 -0.558548 -2.490709
H -2.507716 -1.281749 -1.813141
H -4.060435 -1.494848 -0.987475
H -2.822238 1.681711 -2.510034
H -2.192823 2.377806 -1.006700
H -1.360728 1.018220 -1.772271
Se -2.397465 0.082049 1.012080

Anth_I_SO3Ph.log
Energy (E) = -1504.56073369 Hartree
Enthalpy (H) = -1504.245156 Hartree
Gibbs free energy (G) = -1504.322445

Hartree
Charge = 0, Spin = 1
C 0.237364 4.079870 -0.342414
C -1.055355 3.913453 0.038308
C -1.650329 2.618889 0.071860
C -0.869870 1.473613 -0.295797
C 0.479496 1.696419 -0.694559
C 1.009408 2.948683 -0.719681
C -2.974067 2.451052 0.458810
C -1.500666 0.227696 -0.238783
C -2.835209 0.035775 0.131560
C -3.585236 1.203605 0.493332
C -4.946262 1.067846 0.892412
H -5.486342 1.965768 1.165104
C -5.544786 -0.150048 0.929956
C -4.808707 -1.306123 0.557240
C -3.508496 -1.220429 0.167567
H -3.551519 3.324121 0.741709
H 0.681663 5.065144 -0.364548
H -1.667578 4.759442 0.324512
H 1.099780 0.863319 -0.987251
H 2.037973 3.078855 -1.029610
H -6.577523 -0.246933 1.234223
H -5.294707 -2.271862 0.573625
H -2.994186 -2.114845 -0.148350
I -0.367893 -1.482303 -0.690493
F -1.656157 -1.823498 -2.199745
O 0.793137 -0.836186 1.053227
S 2.182672 -1.489396 1.181963
C 3.247506 -0.269326 0.477795
O 2.199652 -2.646361 0.306639
O 2.519181 -1.634495 2.566714
C 3.582818 -0.348949 -0.866190
C 3.683726 0.778490 1.276481
C 4.364354 0.654497 -1.425604
H 3.247779 -1.196142 -1.449518
C 4.468639 1.772516 0.710666
H 3.413667 0.795128 2.323264
C 4.804547 1.711815 -0.637982
H 4.639484 0.603796 -2.470059
H 4.822636 2.591874 1.321055
H 5.420857 2.486469 -1.074207

AnthI8BA_B_SO3Ph_2.log
Energy (E) = -1710.42684649 Hartree
Enthalpy (H) = -1710.020074 Hartree
Gibbs free energy (G) = -1710.105133
Hartree
Charge = 0, Spin = 1
C -6.553866 -1.631827 -0.014237
C -5.441245 -2.085342 0.623085
C -4.140314 -1.698587 0.186122
C -4.028714 -0.831608 -0.948040
C -5.218373 -0.379079 -1.589413
C -6.441901 -0.763106 -1.136801
C -2.985911 -2.128905 0.838803
C -2.766505 -0.427721 -1.373386
C -1.619943 -0.844363 -0.704828
C -1.724517 -1.715006 0.420602
C -0.532208 -2.110378 1.087609
H -0.622019 -2.791415 1.926599
C 0.717452 -1.687102 0.726668
C 0.760398 -0.781641 -0.375617
H -3.074637 -2.788146 1.695110
H -7.535286 -1.929541 0.329102
H -5.522151 -2.745830 1.477506
H -5.123055 0.279899 -2.443689
H -7.339260 -0.412612 -1.628062
H -2.676154 0.246013 -2.217927
I 2.550027 0.150504 -1.005736
C -0.329430 -0.386203 -1.079184
H -0.246042 0.316774 -1.897269
C 1.910890 -2.235189 1.469355
H 2.370778 -3.015443 0.857237
H 1.512460 -2.753014 2.340622
C 2.969983 -1.211162 1.922384
H 2.572319 -0.200234 1.831573
H 3.148370 -1.351439 2.986730
C 4.336578 -1.326537 1.209294
H 4.742853 -0.330498 1.019912
H 5.055098 -1.850059 1.833516
C 4.298768 -2.116106 -0.083252
O 3.474724 -1.697975 -1.061829
O 4.939231 -3.119360 -0.249568
O 1.356470 2.016549 -0.979469
S 1.089190 2.729919 0.362665
C -0.613878 2.379841 0.681404
O 1.218980 4.145605 0.169241
O 1.865691 2.078469 1.398513
C -1.577822 2.972087 -0.126101
C -0.960050 1.536912 1.724472
C -2.915504 2.700330 0.114694
H -1.270195 3.644229 -0.916264
C -2.305348 1.275327 1.961654
H -0.180902 1.102549 2.336121
C -3.278313 1.851110 1.157132
H -3.678504 3.151431 -0.505447
H -2.590594 0.616554 2.770733
H -4.323713 1.634585 1.335673

FuranIBCMe2S_A_SO3Ph.log
Energy (E) = -1610.91196113 Hartree
Enthalpy (H) = -1610.649609 Hartree
Gibbs free energy (G) = -1610.722990
Hartree
Charge = 0, Spin = 1
C 1.964789 3.038589 -0.164538
C 0.936635 2.163384 -0.271347
C 1.559125 0.900250 -0.090558
C 2.877803 1.079137 0.118101
O 3.145708 2.396351 0.077855
H 2.021298 4.107728 -0.254516
H -0.100151 2.356564 -0.486643
I 0.886573 -1.049150 -0.011630
S 3.317844 -1.504043 -0.287601
C 3.905928 0.057445 0.470211
C 5.258398 0.373989 -0.158010
H 5.636948 1.308587 0.257667
H 5.971144 -0.418366 0.070179
H 5.173914 0.476120 -1.238165
C 4.019016 -0.075708 1.987013
H 4.750381 -0.842183 2.241362
H 4.336008 0.875014 2.419642
H 3.058000 -0.351574 2.422481
O -1.169904 -0.001786 0.353977
S -2.116375 0.059915 -0.838622
C -3.702738 0.105862 -0.063182
O -2.019097 -1.169385 -1.597612
O -1.938987 1.306191 -1.553172
C -4.338044 -1.088295 0.244225
C -4.280898 1.331766 0.228991
C -5.576691 -1.050991 0.867908
H -3.866531 -2.023366 -0.024666
C -5.520102 1.360054 0.853427
H -3.764814 2.239677 -0.049985
C -6.164066 0.171194 1.174494
H -6.087166 -1.973567 1.108465
H -5.986039 2.308497 1.083486
H -7.131535 0.196852 1.657843

Indole_NMe_IBCONAc_A_SO3Ph.log
Energy (E) = -1589.01017442 Hartree
Enthalpy (H) = -1588.677204 Hartree
Gibbs free energy (G) = -1588.763552
Hartree
Charge = 0, Spin = 1
C 1.208852 2.070891 -0.252560
C 2.463293 2.622739 0.115571
C 2.691480 4.000400 0.119043
C 1.647245 4.817332 -0.253319
C 0.398565 4.285257 -0.624463
C 0.164652 2.929034 -0.629394
C 1.407409 0.671360 -0.120526
H 3.653772 4.404768 0.402416
H 1.788756 5.889364 -0.263325
H -0.394586 4.960179 -0.914381
H -0.791647 2.522552 -0.918248
N 3.341377 1.616681 0.437966
C 2.687546 0.425845 0.288033
C 3.210863 -0.933439 0.501034
O 4.334552 -1.188134 0.865317
I 0.317606 -1.055988 -0.391172
C 4.715144 1.836261 0.852093
H 4.736220 2.441164 1.758258
H 5.181492 0.876993 1.043856
H 5.260171 2.357306 0.065351
N 2.207174 -1.863457 0.209676
C 2.383025 -3.256769 0.303592
C 1.171745 -4.067825 -0.095806
H 0.891502 -3.876103 -1.133335
H 1.418363 -5.118528 0.013608
H 0.312131 -3.840971 0.538474
O 3.408911 -3.766516 0.667366
O -1.395585 0.301978 -0.918205
S -2.699078 -0.473380 -1.160535
C -3.702423 -0.019275 0.217100
O -2.373681 -1.886879 -1.027238
O -3.346591 -0.019105 -2.356402
C -3.435010 -0.583399 1.457348
C -4.724427 0.899481 0.041331
C -4.205935 -0.210830 2.546991
H -2.646301 -1.318371 1.553015
C -5.493313 1.265828 1.138659
H -4.909245 1.302182 -0.944544
C -5.232417 0.714450 2.386189
H -4.013690 -0.644693 3.518552
H -6.298079 1.977729 1.017829
H -5.835202 1.000494 3.237456

Me_Pyrrole_3I_SO3Ph.log
Energy (E) = -1214.79530377 Hartree
Enthalpy (H) = -1214.567100 Hartree
Gibbs free energy (G) = -1214.637645
Hartree
Charge = 0, Spin = 1
C -1.829262 1.571132 0.121321
C -2.318906 0.347667 -0.247489
C -3.643332 0.475562 -0.714269
C -3.926792 1.812831 -0.608700
N -2.829823 2.463781 -0.111588
H -0.873773 1.855965 0.527527
H -4.289393 -0.311799 -1.056080
H -4.825881 2.353363 -0.850265
C -2.766041 3.875903 0.206253
H -3.265194 4.450074 -0.571221
H -3.239437 4.084197 1.164742
H -1.724669 4.182448 0.253804
I -1.289421 -1.439760 -0.150642
F -3.092769 -2.317600 -0.131697
O 0.510217 -0.224328 -0.335053

S 1.422722 -0.064853 0.902424
C 2.981327 0.234014 0.138933
O 1.477084 -1.323322 1.606615
O 1.040763 1.118653 1.635393
C 3.761489 -0.850035 -0.236753
C 3.397421 1.540293 -0.066333
C 4.986118 -0.615380 -0.843540
H 3.412053 -1.853018 -0.034761
C 4.624737 1.764606 -0.674164
H 2.769150 2.355433 0.263559
C 5.413807 0.689267 -1.063685
H 5.609732 -1.447949 -1.138382
H 4.967748 2.776793 -0.838133
H 6.371170 0.868153 -1.534223

NaphIBMeUreaMe_SO3Ph.log
Energy (E) = -1553.10818542 Hartree
Enthalpy (H) = -1552.750208 Hartree
Gibbs free energy (G) = -1552.831705 Hartree
Charge = 0, Spin = 1
C -0.281722 2.699360 0.082540
C -1.642135 3.065291 0.228966
C -2.631253 2.060402 0.319937
C -2.314053 0.723144 0.281438
C -0.940473 0.403908 0.153817
C 0.049740 1.326232 0.048966
H -3.665661 2.362911 0.413583
H 1.087002 1.029587 -0.035510
I -0.511806 -1.641381 0.106883
C -3.406224 -1.345376 -0.475213
O -4.475884 -1.873115 -0.707141
N -2.231729 -1.723984 -1.076068
C -2.317613 -2.837988 -2.009107
H -3.126531 -2.645818 -2.709690
H -1.380632 -2.911639 -2.558977
H -2.526644 -3.787740 -1.512241
N -3.300161 -0.272005 0.417574
C -4.549163 0.093518 1.070914
H -4.321301 0.691945 1.949918
H -5.213077 0.651557 0.407416
H -5.062167 -0.816037 1.364618
C -1.973585 4.441019 0.265670
C -0.997548 5.393355 0.161562
C 0.358618 5.023300 0.017089
C 0.709622 3.702484 -0.020798
H -3.012714 4.724652 0.376365
H -1.262163 6.441653 0.190598
H 1.116160 5.790748 -0.061741
H 1.743683 3.397982 -0.130966
O 1.273412 -1.054776 1.423445
S 2.604484 -1.679487 1.011414
C 3.231002 -0.457602 -0.106337
O 2.336217 -2.877750 0.238527
O 3.492548 -1.752422 2.136352
C 2.942666 -0.555722 -1.460156
C 3.898134 0.643469 0.413956
C 3.312069 0.481768 -2.307735
H 2.446297 -1.442206 -1.831646
C 4.266920 1.673599 -0.440030
H 4.118027 0.674931 1.472404
C 3.965795 1.596535 -1.796715
H 3.095310 0.418001 -3.365232
H 4.792722 2.534741 -0.050113
H 4.253745 2.401949 -2.458781

NpthIBCMe2O_C_SO3Ph.log
Energy (E) = -1443.67142955 Hartree
Enthalpy (H) = -1443.327435 Hartree
Gibbs free energy (G) = -1443.406451 Hartree
Charge = 0, Spin = 1
C -3.722064 0.226027 -0.330674
C -2.650953 -0.622302 -0.280866
C -1.397199 -0.064632 0.026879
C -1.180005 1.247478 0.293903
H -4.709740 -0.155897 -0.561656
H -0.197521 1.619544 0.544066
O -1.435852 -2.550437 -0.930270
I 0.070737 -1.569416 0.014962
C -2.143143 3.511430 0.494921
C -3.224660 4.346177 0.435153
C -4.501979 3.834615 0.116901
C -4.670963 2.500736 -0.133998
C -3.571824 1.611713 -0.079158
C -2.290072 2.127782 0.240794
H -1.159728 3.892483 0.738881
H -3.106235 5.402730 0.632557
H -5.349187 4.505385 0.073161
H -5.648559 2.103894 -0.378186
C -2.708270 -2.129011 -0.468608
C -3.697943 -2.548320 -1.541317
H -3.623401 -3.625580 -1.682232
H -4.719176 -2.310201 -1.243628
H -3.469618 -2.050332 -2.481700
C -3.042108 -2.795175 0.864238
H -4.031231 -2.489654 1.205745
H -3.021963 -3.877538 0.738846
H -2.319498 -2.516376 1.632899
O 1.355334 0.010758 1.012043
S 2.822123 -0.397321 1.173307
C 3.645866 0.573973 -0.051257
O 2.931114 -1.790575 0.773262
O 3.318751 -0.000300 2.459949
C 3.613260 0.158408 -1.375568
C 4.291438 1.740740 0.326681
C 4.233774 0.935705 -2.341856
H 3.127804 -0.774412 -1.631200
C 4.911656 2.512516 -0.647242
H 4.310433 2.017858 1.371386
C 4.879540 2.112265 -1.977057
H 4.221941 0.621614 -3.376568
H 5.424764 3.422287 -0.367071
H 5.366997 2.714036 -2.732277

NpthISO2NMe_D_SO3Ph.log
Energy (E) = -1893.65473571 Hartree
Enthalpy (H) = -1893.342381 Hartree
Gibbs free energy (G) = -1893.423571 Hartree
Charge = 0, Spin = 1
C 0.607025 3.276707 -0.949445
C 1.837145 3.629431 -0.481391
C 2.732590 2.661738 0.027806
C 2.365036 1.281701 0.075493
C 1.049779 0.995213 -0.377085
C 0.200707 1.930154 -0.890017
H 4.233687 4.135980 0.442013
H -0.069743 4.012275 -1.358943
H 2.162787 4.661617 -0.498891
C 4.005917 3.078451 0.483511
C 3.368194 0.379812 0.537209
H -0.783060 1.654853 -1.246068
C 4.598218 0.816293 0.951018
C 4.922950 2.183256 0.947956
H 5.330233 0.080923 1.255886
H 5.896348 2.506671 1.286900
I 0.261859 -0.972285 -0.253904
S 3.196523 -1.379382 0.613783
O 2.532205 -1.728111 1.844879
O 4.469398 -1.984788 0.321093
N 2.221437 -1.612637 -0.694106
C 2.284939 -2.947185 -1.299635
H 1.733654 -2.917636 -2.237082
H 3.326443 -3.178352 -1.509908
H 1.873122 -3.730837 -0.658771
O -1.726617 0.072108 0.237823
S -2.821929 -0.214466 -0.787636
C -4.295285 0.087244 0.131032
O -2.776978 -1.617086 -1.145924
O -2.753046 0.751686 -1.864375
C -4.810622 -0.929275 0.922409
C -4.906696 1.328613 0.048693
C -5.962343 -0.689557 1.656548
H -4.316649 -1.890822 0.940804
C -6.059625 1.558860 0.786599
H -4.484591 2.084966 -0.597801
C -6.582831 0.553128 1.589865
H -6.379572 -1.471743 2.275658
H -6.552169 2.519884 0.730586
H -7.482851 0.735379 2.161580

perF_CMe2O_SO3Ph_2.log
Energy (E) = -1686.94429439 Hartree
Enthalpy (H) = -1686.678300 Hartree
Gibbs free energy (G) = -1686.759444 Hartree
Charge = 0, Spin = 1
C 2.061085 2.575082 -0.222561
C 3.326354 2.266142 0.242317
C 3.678258 0.941767 0.465001
C 2.779038 -0.081374 0.236045
C 1.497744 0.263569 -0.168428
C 1.124919 1.567225 -0.433374
O 2.154317 -2.266520 -0.394998
I 0.293439 -1.455481 -0.268273
F -0.042660 1.918241 -0.924441
F 1.743270 3.831127 -0.480115
F 4.204247 3.230888 0.449432
F 4.905899 0.697925 0.918936
C 3.087370 -1.571176 0.404463
C 4.449045 -1.965230 -0.150157
H 5.260533 -1.598248 0.472140
H 4.485713 -3.053412 -0.174940
H 4.567843 -1.591333 -1.165569
C 2.966651 -1.951666 1.876745
H 3.147524 -3.020643 1.984265
H 3.700804 -1.403784 2.468002
H 1.973077 -1.720805 2.263893
O -1.428182 -0.108896 0.101623
S -2.606627 -0.394657 -0.850622
C -3.973698 0.184388 0.095008
O -2.701400 -1.835757 -0.990900
O -2.503025 0.397712 -2.046239
C -4.576886 -0.671148 1.005291
C -4.416027 1.485103 -0.092649
C -5.648204 -0.205096 1.752682
H -4.215014 -1.685298 1.102287
C -5.487805 1.941920 0.661334
H -3.928538 2.109687 -0.827978
C -6.099019 1.099173 1.581964
H -6.135477 -0.859009 2.462701
H -5.849457 2.951914 0.526541
H -6.936687 1.457944 2.164792

perF_NMeCO2_SO3Ph.log
Energy (E) = -1776.98049427 Hartree
Enthalpy (H) = -1776.742651 Hartree
Gibbs free energy (G) = -1776.823976 Hartree

Charge = 0, Spin = 1
C 2.588929 2.394650 0.059452
C 3.409178 1.717265 0.946465
C 3.255777 0.355282 1.158984
C 2.236837 -0.366439 0.538718
C 1.405486 0.353969 -0.321262
C 1.591567 1.694399 -0.599716
I -0.049602 -0.762408 -1.275569
F 0.857723 2.323172 -1.495700
F 2.775592 3.681268 -0.171710
F 4.382892 2.364117 1.557938
F 4.139180 -0.245549 1.948530
N 2.066744 -1.737514 0.747409
C 2.370639 -2.336468 2.046846
H 2.193643 -1.598424 2.824408
H 1.706031 -3.183337 2.188975
H 3.397315 -2.690341 2.102691
C 1.941930 -2.641600 -0.321464
O 2.178581 -3.810230 -0.168091
O 1.552030 -2.099695 -1.470847
O -1.345754 0.886724 -0.717523
S -2.843370 0.598595 -0.998152
C -3.496677 0.286122 0.606746
O -2.872205 -0.645987 -1.749313
O -3.472027 1.768470 -1.529289
C -3.369445 -0.984555 1.151065
C -4.106829 1.320864 1.299225
C -3.858550 -1.219005 2.427416
H -2.919058 -1.776318 0.567458
C -4.592987 1.075294 2.575849
H -4.202886 2.289897 0.830167
C -4.465836 -0.189280 3.137591
H -3.776156 -2.204756 2.863760
H -5.076313 1.868530 3.128952
H -4.850545 -0.376882 4.130824

Py_PhI_SO3Ph.log
Energy (E) = -1444.43970111 Hartree
Enthalpy (H) = -1444.149305 Hartree
Gibbs free energy (G) = -1444.226684 Hartree
Charge = 0, Spin = 1
C 2.511861 1.240776 0.438776
C 1.269002 1.141002 -0.174106
C 0.457937 2.228428 -0.436305
C 0.901185 3.491874 -0.069043
C 2.141132 3.639653 0.534697
C 2.930983 2.527831 0.780799
C 3.389709 0.092894 0.781001
C 3.578842 -1.984886 1.708588
C 4.948693 -2.020005 1.489894
C 5.545909 -0.926406 0.880675
C 4.755726 0.151729 0.520665
H -0.499612 2.100800 -0.924346
H 0.277714 4.351862 -0.268012
H 2.493199 4.620976 0.821250
H 3.885521 2.637750 1.278810
H 3.075955 -2.820435 2.181131
H 5.525669 -2.884586 1.784894
H 6.608616 -0.917684 0.680477
H 5.174584 1.013139 0.019687
N 2.812620 -0.953291 1.366868
I 0.579970 -0.718324 -0.890669
F 2.433383 -0.990181 -1.620327
O -1.300242 -0.140202 0.067512
S -2.508653 -0.078217 -0.891241
C -3.851858 -0.153443 0.242794
O -2.506489 -1.270807 -1.707287
O -2.537819 1.202066 -1.558715
C -4.315831 -1.396140 0.648795
C -4.417047 1.026112 0.702431
C -5.370181 -1.454937 1.547737
H -3.860808 -2.290972 0.247649
C -5.471354 0.956023 1.601941
H -4.037833 1.972067 0.342616
C -5.943270 -0.281062 2.024005
H -5.748761 -2.414304 1.872236
H -5.927858 1.864908 1.968762
H -6.767347 -0.331314 2.722861

PyIBCMe2O_D_SO3Ph.log
Energy (E) = -1306.20278599 Hartree
Enthalpy (H) = -1305.920341 Hartree
Gibbs free energy (G) = -1305.992764 Hartree
Charge = 0, Spin = 1
C 1.863829 3.155909 -0.053009
C 3.224442 3.014657 0.184478
C 3.095403 0.739813 0.203414
C 1.732450 0.822357 -0.011538
H 1.428229 4.138005 -0.163577
H 3.855835 3.890078 0.274573
O 2.863161 -1.565728 -0.297515
I 0.896170 -1.096660 -0.081310
C 1.069321 2.018409 -0.147090
H 0.005588 2.066858 -0.336053
N 3.835137 1.836161 0.313449
C 3.709738 -0.638819 0.363454
C 5.063693 -0.719469 -0.314430
H 5.738929 0.013429 0.123724
H 5.469976 -1.720839 -0.179797
H 4.960714 -0.517365 -1.378993
C 3.815180 -0.959109 1.851143
H 4.235612 -1.956478 1.975104
H 4.457495 -0.230672 2.345584
H 2.832680 -0.929402 2.326979
O -1.072561 -0.069516 0.292623
S -2.045770 0.091345 -0.884138
C -3.607973 0.113267 -0.066737
O -1.977726 -1.084912 -1.720509
O -1.845353 1.382285 -1.504023
C -4.249135 -1.090087 0.189007
C -4.163684 1.328393 0.303277
C -5.472737 -1.073146 0.841956
H -3.795229 -2.014372 -0.140540
C -5.388321 1.335261 0.956229
H -3.643474 2.244669 0.062278
C -6.038233 0.137222 1.227000
H -5.988588 -2.001722 1.044154
H -5.838159 2.274405 1.247598
H -6.994286 0.146754 1.732901

PyrroleNMeIBCMe2O_A_SO3Ph.log
Energy (E) = -1307.38831183 Hartree
Enthalpy (H) = -1307.082326 Hartree
Gibbs free energy (G) = -1307.157678 Hartree
Charge = 0, Spin = 1
C 2.416981 2.205086 -1.767299
C 1.293045 1.416984 -1.766888
C 1.562286 0.430822 -0.801502
C 2.786961 0.628416 -0.238301
H 2.662466 3.062732 -2.370669
H 0.409000 1.523618 -2.367721
I 0.576732 -1.137351 0.088311
O 2.463749 -1.404001 0.858947
N 3.315991 1.733906 -0.837483
C 4.620650 2.313714 -0.593532
H 4.775838 2.476369 0.472129
H 5.415738 1.673836 -0.974008
H 4.669325 3.274529 -1.099144
C 3.246092 -0.220728 0.919530
C 4.693587 -0.679857 0.819726
H 5.385484 0.147655 0.978173
H 4.868740 -1.426043 1.593580
H 4.881361 -1.132807 -0.152350
C 2.986591 0.502261 2.238724
H 3.279106 -0.141231 3.068161
H 3.550243 1.435206 2.297389
H 1.925029 0.738178 2.329493
O -1.146009 -0.352735 -1.119974
S -2.494227 -0.998960 -0.788184
C -3.338792 0.269828 0.105177
O -2.233032 -2.087086 0.140055
O -3.237847 -1.265510 -1.986266
C -3.024928 0.474205 1.442026
C -4.282484 1.048644 -0.545167
C -3.666505 1.487213 2.138246
H -2.303988 -0.172175 1.925325
C -4.921332 2.060456 0.159863
H -4.510101 0.844654 -1.581989
C -4.611812 2.279995 1.496037
H -3.437569 1.654023 3.181969
H -5.664219 2.673083 -0.332322
H -5.114464 3.066426 2.042883

Anth_I_StBu.log
Energy (E) = -1205.31058602 Hartree
Enthalpy (H) = -1204.979588 Hartree
Gibbs free energy (G) = -1205.051596 Hartree
Charge = 0, Spin = 1
C -2.602788 3.738508 0.354917
C -3.161614 2.585445 0.802618
C -2.555685 1.322624 0.537770
C -1.327771 1.268581 -0.202599
C -0.780503 2.505835 -0.657207
C -1.396201 3.688864 -0.393480
C -3.137241 0.145865 0.992142
C -0.778651 0.006372 -0.436652
C -1.372116 -1.192258 -0.034684
C -2.586507 -1.100781 0.722720
C -3.208788 -2.292518 1.196855
H -4.121582 -2.193460 1.771069
C -2.673028 -3.511753 0.934745
C -1.484197 -3.606248 0.161829
C -0.858306 -2.494934 -0.310183
H -4.054681 0.201697 1.567437
H -3.070424 4.692110 0.556834
H -4.085745 2.594243 1.367057
H 0.130326 2.506883 -1.236958
H -0.961108 4.607981 -0.761172
H -3.150624 -4.411698 1.296807
H -1.080062 -4.581863 -0.071346
H -0.000199 -2.596621 -0.956008
I 1.121957 -0.103020 -1.368842
F 0.034202 -1.243843 -2.800542
S 2.149749 1.229091 0.454663
C 2.698285 -0.038935 1.658470
C 3.272068 0.778786 2.811621
C 3.779626 -0.926809 1.061058
C 1.526055 -0.876716 2.145336
H 2.505066 1.412332 3.257812
H 3.653930 0.100782 3.578583
H 4.092181 1.411818 2.470633
H 4.130090 -1.637846 1.813595
H 3.396244 -1.500403 0.215378
H 4.626291 -0.330429 0.721307
H 1.853877 -1.521741 2.964450
H 0.711201 -0.243396 2.499109

H 1.141884 -1.520769 1.352638

AnthI8BA_B_StBu.log
Energy (E) = -1411.17040332 Hartree
Enthalpy (H) = -1410.748137 Hartree
Gibbs free energy (G) = -1410.829747 Hartree
Charge = 0, Spin = 1
C -7.123380 -0.419199 0.297404
C -6.063884 -1.062190 0.856149
C -4.731212 -0.776329 0.436636
C -4.529617 0.203826 -0.588494
C -5.667429 0.855665 -1.148756
C -6.922421 0.555412 -0.721323
C -3.629663 -1.421219 0.994325
C -3.233931 0.492844 -1.008640
C -2.137724 -0.156889 -0.447722
C -2.334018 -1.132625 0.572784
C -1.192935 -1.779430 1.119166
H -1.358254 -2.524163 1.889865
C 0.095517 -1.534501 0.725106
C 0.238950 -0.525908 -0.278511
H -3.785098 -2.164102 1.768728
H -8.129544 -0.644997 0.623582
H -6.210902 -1.804917 1.630507
H -5.506696 1.594770 -1.923866
H -7.778611 1.055067 -1.153619
H -3.076496 1.235257 -1.782897
I 2.145900 0.172980 -0.958148
C -0.803260 0.128562 -0.844731
H -0.643127 0.899811 -1.586865
C 1.182992 -2.368324 1.364219
H 1.420276 -3.194102 0.690891
H 0.724137 -2.824745 2.240750
C 2.477621 -1.653674 1.792361
H 2.311612 -0.574746 1.799680
H 2.680873 -1.910084 2.831155
C 3.726371 -2.012714 0.961826
H 4.334581 -1.119715 0.789722
H 4.353712 -2.722354 1.494702
C 3.404381 -2.660941 -0.381979
O 2.555920 -2.026771 -1.151592
O 3.887609 -3.735077 -0.673377
S 1.483777 2.558698 -0.786732
C 1.643501 2.961941 0.994766
C 1.347006 4.458257 1.050399
C 3.053006 2.695234 1.500981
C 0.618937 2.199062 1.821350
H 0.342354 4.670892 0.683507
H 1.416036 4.798367 2.086180
H 2.063963 5.021059 0.451966
H 3.135497 3.028853 2.538129
H 3.293625 1.630259 1.477034
H 3.789088 3.231220 0.901957
H 0.654223 2.548429 2.856209
H -0.389111 2.349328 1.432942
H 0.823631 1.127168 1.825193

FuranIBCMe2S_A_StBu.log
Energy (E) = -1311.65505599 Hartree
Enthalpy (H) = -1311.377250 Hartree
Gibbs free energy (G) = -1311.444629 Hartree
Charge = 0, Spin = 1
C 1.293736 2.982990 0.238006
C 0.214260 2.187722 0.415907
C 0.711878 0.871215 0.201738
C 2.033877 0.943591 -0.065336
O 2.397704 2.245701 -0.050300
H 1.428390 4.048579 0.274213
H -0.790673 2.474339 0.667911
I -0.232825 -0.970344 0.358606
S 2.198129 -1.675906 -0.558655
C 3.071811 -0.109565 -0.248900
C 3.956482 0.214238 -1.452017
H 4.488800 1.152938 -1.286150
H 4.687121 -0.582842 -1.588171
H 3.359104 0.294970 -2.358468
C 3.922480 -0.202387 1.019672
H 4.670164 -0.985806 0.897460
H 4.429644 0.746706 1.208981
H 3.300360 -0.452290 1.878746
S -2.439898 0.130823 1.095710
C -3.371818 0.261839 -0.477641
C -4.708973 0.876580 -0.071750
C -3.609451 -1.110823 -1.091773
C -2.666049 1.174792 -1.469527
H -4.565302 1.857124 0.383211
H -5.336477 0.993519 -0.959148
H -5.230996 0.236527 0.640046
H -4.256440 -1.014270 -1.967526
H -2.675055 -1.567634 -1.421391
H -4.089418 -1.776078 -0.374117
H -3.260812 1.253775 -2.383221
H -2.538890 2.175212 -1.054996
H -1.682493 0.788009 -1.744222

Indole_NMe_IBCONAc_A_StBu.log
Energy (E) = -1289.76030131 Hartree
Enthalpy (H) = -1289.411867 Hartree
Gibbs free energy (G) = -1289.491334 Hartree
Charge = 0, Spin = 1
C 0.272794 -2.001863 -0.331494
C -0.703890 -2.956913 0.049401
C -0.442797 -4.327804 0.058540
C 0.811838 -4.743593 -0.330218
C 1.788166 -3.815390 -0.729027
C 1.536725 -2.460280 -0.736198
C -0.405353 -0.755499 -0.199519
H -1.205339 -5.034704 0.356479
H 1.047239 -5.798947 -0.336635
H 2.758693 -4.173965 -1.043495
H 2.295301 -1.764076 -1.057444
N -1.876843 -2.319420 0.366485
C -1.695305 -0.974269 0.202747
C -2.726058 0.087820 0.381307
O -3.837208 -0.157160 0.811037
I 0.116645 1.236942 -0.512005
C -3.082617 -3.022876 0.768264
H -2.888388 -3.594911 1.675472
H -3.864600 -2.296505 0.953279
H -3.389822 -3.707235 -0.022566
N -2.196330 1.287125 -0.005820
C -2.933382 2.465055 -0.012418
C -2.116925 3.691317 -0.373189
H -1.686312 3.589813 -1.371255
H -2.767582 4.559732 -0.352870
H -1.301499 3.837471 0.338358
O -4.115087 2.551991 0.226584
S 2.548397 0.912277 -0.891394
C 3.256487 1.040988 0.799252
C 4.747998 0.806526 0.575384
C 2.695189 -0.028652 1.722991
C 3.031138 2.429667 1.377780
H 5.164880 1.550008 -0.104947
H 5.268725 0.884238 1.532386
H 4.929560 -0.186463 0.162799
H 3.192727 0.036801 2.693551
H 1.624715 0.103442 1.891274
H 2.857095 -1.026910 1.316672
H 3.536676 2.508676 2.343164
H 3.427251 3.196362 0.712115
H 1.970689 2.624837 1.544992

Me_Pyrrole_3I_StBu.log
Energy (E) = -915.546546483 Hartree
Enthalpy (H) = -915.302868 Hartree
Gibbs free energy (G) = -915.365667 Hartree
Charge = 0, Spin = 1
C -1.582101 1.165169 0.414135
C -1.461943 -0.129104 -0.016421
C -2.648801 -0.524700 -0.666638
C -3.471741 0.571224 -0.611825
N -2.822755 1.586659 0.036487
H -0.895175 1.798796 0.946220
H -2.840642 -1.489111 -1.098244
H -4.471772 0.709986 -0.986485
C -3.395638 2.874573 0.363639
H -4.093770 3.168243 -0.416986
H -3.922255 2.844611 1.317165
H -2.605423 3.619496 0.420142
I 0.203040 -1.360523 0.236388
F -1.118950 -2.828786 -0.524345
S 1.584780 0.502036 1.118958
C 2.439145 1.153554 -0.369132
C 3.309289 2.284069 0.170802
C 1.446276 1.700044 -1.382993
C 3.319127 0.085605 -1.002006
H 4.020011 1.910189 0.908589
H 3.868965 2.733270 -0.653067
H 2.698398 3.057730 0.637402
H 1.988308 2.118867 -2.234720
H 0.782718 0.919011 -1.758038
H 0.832246 2.485674 -0.941640
H 3.885722 0.520866 -1.829012
H 4.020626 -0.318651 -0.272231
H 2.722615 -0.733996 -1.404641

NaphIBMeUreaMe_StBu.log
Energy (E) = -1253.84629952 Hartree
Enthalpy (H) = -1253.473230 Hartree
Gibbs free energy (G) = -1253.549876 Hartree
Charge = 0, Spin = 1
C 2.097250 -1.924085 -0.237808
C 3.226418 -1.104465 -0.000622
C 3.046647 0.273055 0.249358
C 1.803246 0.866267 0.270893
C 0.698731 0.003780 0.038763
C 0.817409 -1.325278 -0.199595
H 3.924946 0.880465 0.422036
H -0.063736 -1.941408 -0.320290
I -1.219267 0.880919 0.146252
C 0.871713 3.092481 -0.211965
O 1.101063 4.292157 -0.205219
N -0.081430 2.473492 -0.944156
C -0.920362 3.361600 -1.725095
H -0.304721 3.976037 -2.381041
H -1.594874 2.765007 -2.340815
H -1.509576 4.044858 -1.105182
N 1.652866 2.221560 0.584069
C 2.717677 2.870378 1.330660
H 3.037374 2.212271 2.136354
H 3.576092 3.118954 0.701222
H 2.330346 3.797792 1.738384
C 4.512088 -1.697102 -0.030057
C 4.656764 -3.033581 -0.281988
C 3.526181 -3.848855 -0.513386

C 2.272187 -3.303831 -0.490313
H 5.378010 -1.071864 0.148792
H 5.644068 -3.474898 -0.303378
H 3.658021 -4.904444 -0.707167
H 1.396289 -3.916923 -0.663143
S -2.234108 -1.180257 1.263983
C -3.371086 -1.815011 -0.029514
C -4.080996 -2.989171 0.638771
C -2.616189 -2.304923 -1.256344
C -4.398348 -0.761601 -0.423750
H -4.634810 -2.657762 1.517407
H -4.784705 -3.436895 -0.067765
H -3.365452 -3.751949 0.947395
H -3.323189 -2.689797 -1.995726
H -2.047590 -1.499501 -1.726759
H -1.929368 -3.109905 -0.990622
H -5.132860 -1.200146 -1.104414
H -4.918460 -0.383896 0.456368
H -3.933465 0.078846 -0.942424

NpthIBCMe2O_C_StBu.log
Energy (E) = -1144.41667004 Hartree
Enthalpy (H) = -1144.057578 Hartree
Gibbs free energy (G) = -1144.129326 Hartree
Charge = 0, Spin = 1
C 2.928046 0.617407 -0.244619
C 1.660260 1.124995 -0.141849
C 0.624931 0.209931 0.102426
C 0.800944 -1.122233 0.273525
H 3.763778 1.282842 -0.427827
H -0.032460 -1.774579 0.502496
O -0.031017 2.708954 -0.602778
I -1.277318 1.164915 0.224707
C 2.366626 -3.035036 0.302901
C 3.639358 -3.520964 0.183759
C 4.713105 -2.643348 -0.085084
C 4.491482 -1.301268 -0.227905
C 3.185717 -0.768045 -0.110454
C 2.110152 -1.651462 0.158875
H 1.536197 -3.698469 0.510583
H 3.829560 -4.579665 0.295916
H 5.715332 -3.039597 -0.176752
H 5.311849 -0.624654 -0.433325
C 1.296990 2.604860 -0.202850
C 2.145004 3.354416 -1.222653
H 1.785285 4.381381 -1.272269
H 3.199182 3.371255 -0.942006
H 2.038304 2.897496 -2.205272
C 1.497319 3.206716 1.192152
H 2.538512 3.134922 1.510603
H 1.197118 4.254493 1.168173
H 0.878869 2.684454 1.925273
S -2.452383 -0.900220 1.089209
C -3.228554 -1.612549 -0.414925
C -4.011201 -2.817385 0.098450
C -2.181314 -2.067662 -1.420356
C -4.187265 -0.620196 -1.058394
H -4.769059 -2.509539 0.819467
H -4.509685 -3.309569 -0.740521
H -3.347539 -3.537135 0.578860
H -2.675531 -2.487321 -2.300331
H -1.554678 -1.236879 -1.751664
H -1.537340 -2.836176 -0.991138
H -4.717743 -1.105334 -1.881917
H -4.918747 -0.265543 -0.332258
H -3.656684 0.239976 -1.468674

NpthISO2NMe_D_StBu.log
Energy (E) = -1594.39503426 Hartree
Enthalpy (H) = -1594.067512 Hartree
Gibbs free energy (G) = -1594.141853 Hartree
Charge = 0, Spin = 1
C -0.189498 3.466266 0.281317
C -1.546581 3.566815 0.251528
C -2.359203 2.418460 0.123374
C -1.786635 1.109044 0.026325
C -0.362054 1.076296 0.044946
C 0.402572 2.196145 0.167089
H -4.144089 3.606037 0.166889
H 0.438767 4.339584 0.381465
H -2.036116 4.529500 0.324761
C -3.764123 2.594689 0.096550
C -2.716018 0.031135 -0.063802
H 1.479558 2.118169 0.141689
C -4.068051 0.238465 -0.073890
C -4.610489 1.533033 -0.005585
H -4.712233 -0.628934 -0.116696
H -5.681932 1.672382 -0.019776
I 0.787019 -0.739845 -0.171215
S -2.256264 -1.672891 -0.186787
O -1.822502 -1.906393 -1.550240
O -3.349333 -2.482611 0.301228
N -1.035469 -1.671037 0.867929
C -0.649534 -3.003165 1.328069
H 0.086621 -2.888988 2.123138
H -1.517038 -3.522847 1.733456
H -0.218600 -3.628164 0.535900
S 2.813878 0.404533 -1.166640
C 4.041743 0.296807 0.195827
C 5.314520 0.880375 -0.411626
C 4.289163 -1.151947 0.595555
C 3.625068 1.117634 1.406610
H 5.161290 1.914839 -0.720493
H 6.113842 0.853894 0.333177
H 5.631231 0.301949 -1.279802
H 5.111503 -1.197874 1.314097
H 3.412070 -1.592040 1.072903
H 4.551162 -1.752730 -0.275108
H 4.384960 1.030260 2.186970
H 3.525047 2.172279 1.146620
H 2.677834 0.767179 1.822909

perF_CMe2O_StBu.log
Energy (E) = -1387.69412831 Hartree
Enthalpy (H) = -1387.413122 Hartree
Gibbs free energy (G) = -1387.487155 Hartree
Charge = 0, Spin = 1
C 1.773677 2.256593 -0.433417
C 2.828212 1.669592 0.241593
C 2.803258 0.308040 0.508394
C 1.740055 -0.483248 0.112748
C 0.664411 0.144252 -0.491719
C 0.676633 1.489174 -0.803784
O 0.740163 -2.476050 -0.630598
I -0.977772 -1.185645 -0.777712
F -0.286777 2.096112 -1.477017
F 1.813237 3.544971 -0.734121
F 3.865507 2.404032 0.606751
F 3.841887 -0.200875 1.169900
C 1.657137 -2.009326 0.296307
C 2.982940 -2.698791 -0.015142
H 3.743225 -2.506990 0.737877
H 2.779211 -3.768167 -0.051986
H 3.346907 -2.386140 -0.992896
C 1.229251 -2.299125 1.737795
H 1.130637 -3.377066 1.864357
H 1.965526 -1.919348 2.447734
H 0.264859 -1.833757 1.958247
S -2.767322 0.558918 -0.731238
C -3.061653 0.781411 1.065202
C -4.169464 1.829485 1.122608
C -1.814462 1.312041 1.758018
C -3.534727 -0.504998 1.725696
H -5.064587 1.477753 0.608508
H -4.424960 2.026974 2.166466
H -3.846817 2.762595 0.660152
H -2.048520 1.561592 2.795874
H -1.016020 0.565724 1.771242
H -1.445555 2.208764 1.257769
H -3.780593 -0.309633 2.772555
H -4.421972 -0.892932 1.225578
H -2.759896 -1.273253 1.706466

perF_NMeCO2_StBu.log
Energy (E) = -1477.74308526 Hartree
Enthalpy (H) = -1477.489982 Hartree
Gibbs free energy (G) = -1477.564458 Hartree
Charge = 0, Spin = 1
C 2.146859 2.082941 -0.462935
C 2.970020 1.353064 0.379363
C 2.655218 0.049212 0.729758
C 1.477252 -0.566063 0.296726
C 0.650203 0.218873 -0.512082
C 0.982019 1.494887 -0.923853
I -1.156151 -0.622197 -1.101234
F 0.209840 2.173554 -1.759301
F 2.480588 3.309526 -0.831140
F 4.098446 1.888646 0.810283
F 3.543024 -0.614798 1.463471
N 1.138044 -1.865871 0.637939
C 1.529356 -2.420879 1.929446
H 1.548375 -1.622215 2.668880
H 0.788125 -3.162905 2.210890
H 2.501474 -2.908602 1.894670
C 0.768140 -2.819522 -0.375820
O 0.842236 -3.994051 -0.089240
O 0.383317 -2.292807 -1.489200
S -2.447699 1.301779 -0.313701
C -2.760602 0.851190 1.438867
C -3.467399 2.090478 1.982408
C -1.460323 0.616868 2.194570
C -3.673544 -0.360427 1.542035
H -4.383092 2.295628 1.426593
H -3.731725 1.917584 3.027627
H -2.820681 2.966434 1.928531
H -1.679998 0.509802 3.259110
H -0.964981 -0.300893 1.869067
H -0.772365 1.453246 2.061804
H -3.904059 -0.553760 2.592142
H -4.606654 -0.190545 1.005636
H -3.196248 -1.255904 1.140860

Py_PhI_StBu.log
Energy (E) = -1145.18773290 Hartree
Enthalpy (H) = -1144.882037 Hartree
Gibbs free energy (G) = -1144.952690 Hartree
Charge = 0, Spin = 1
C -1.805905 1.261457 -0.115891
C -0.429530 1.157426 0.041155
C 0.404370 2.255533 0.066650
C -0.142813 3.527679 -0.038912
C -1.514973 3.673923 -0.170101
C -2.328243 2.551785 -0.212789
C -2.734408 0.110585 -0.247729
C -3.224831 -1.858373 -1.291298

C -4.422809 -1.988384 -0.605530
C -4.774231 -0.997055 0.300423
C -3.919421 0.074526 0.481583
H 1.472301 2.124762 0.163936
H 0.505060 4.392568 -0.012665
H -1.952891 4.659022 -0.253123
H -3.396002 2.658417 -0.355170
H -2.918107 -2.610067 -2.009386
H -5.058394 -2.845479 -0.776393
H -5.694997 -1.065435 0.863685
H -4.134855 0.854089 1.198538
N -2.396128 -0.830351 -1.124739
I 0.448688 -0.744365 0.375916
F -1.200481 -0.897537 1.711906
S 2.306209 -0.253010 -1.200690
C 3.783301 -0.278263 -0.112392
C 4.948347 -0.093181 -1.080224
C 3.916687 -1.619643 0.595865
C 3.768618 0.856593 0.899388
H 4.864013 0.853569 -1.614867
H 5.886495 -0.093948 -0.520012
H 4.979062 -0.902679 -1.809945
H 4.864281 -1.655342 1.139506
H 3.117328 -1.769239 1.324046
H 3.893597 -2.438989 -0.122491
H 4.674396 0.815708 1.509460
H 3.739788 1.823708 0.395619
H 2.911398 0.781502 1.571607

PyIBCMe2O_D_StBu.log
Energy (E) = -1006.94652897 Hartree
Enthalpy (H) = -1006.648843 Hartree
Gibbs free energy (G) = -1006.714359 Hartree
Charge = 0, Spin = 1
C 1.207195 3.131425 0.239586
C 2.549486 2.889350 -0.017571
C 2.235917 0.626600 -0.130348
C 0.886851 0.804980 0.116026
H 0.847894 4.140492 0.379717
H 3.250492 3.713230 -0.080069
O 1.709731 -1.624614 -0.606061
I -0.174683 -1.026312 0.231472
C 0.341580 2.047904 0.326950
H -0.707907 2.163712 0.563423
N 3.059134 1.670777 -0.187115
C 2.769699 -0.789932 -0.261505
C 3.822424 -0.865535 -1.357860
H 4.652605 -0.194672 -1.141844
H 4.183697 -1.891387 -1.419588
H 3.379324 -0.591940 -2.314500
C 3.372037 -1.183280 1.089839
H 3.753165 -2.201924 1.020609
H 4.182442 -0.508359 1.367250
H 2.607314 -1.150797 1.869736
S -2.277390 0.079754 1.108528
C -3.315900 0.273523 -0.393827
C -4.621319 0.870070 0.123372
C -3.594030 -1.074744 -1.043634
C -2.674874 1.223730 -1.394302
H -4.445763 1.831122 0.607964
H -5.306643 1.022760 -0.714192
H -5.095903 0.201022 0.841703
H -4.300129 -0.945066 -1.867916
H -2.684951 -1.516007 -1.454346
H -4.023924 -1.768804 -0.321501
H -3.318183 1.321456 -2.272447
H -2.539919 2.214339 -0.958568
H -1.703352 0.856219 -1.731995

PyrroleNMeIBCMe2O_A_StBu.log
Energy (E) = -1008.13599735 Hartree
Enthalpy (H) = -1007.814825 Hartree
Gibbs free energy (G) = -1007.882912 Hartree
Charge = 0, Spin = 1
C 1.664658 2.672201 0.388937
C 0.454614 2.040076 0.522969
C 0.726274 0.692765 0.217535
C 2.047476 0.518546 -0.063014
H 1.922656 3.711914 0.502008
H -0.486644 2.474221 0.808866
I -0.381520 -1.057070 0.264297
O 1.561962 -1.708206 -0.523376
N 2.627002 1.752237 0.042760
C 4.009495 2.089981 -0.224386
H 4.679162 1.402510 0.288791
H 4.225641 2.061092 -1.291702
H 4.195016 3.095113 0.146386
C 2.638273 -0.854335 -0.296443
C 3.542489 -0.910921 -1.526815
H 4.471760 -0.355645 -1.387967
H 3.789776 -1.956609 -1.707224
H 3.013420 -0.524925 -2.397499
C 3.404470 -1.301338 0.952632
H 3.785371 -2.309248 0.786483
H 4.242586 -0.641368 1.188824
H 2.724732 -1.320262 1.805884
S -2.430050 0.132043 1.089681
C -3.360883 0.501565 -0.447232
C -4.620618 1.210429 0.041033
C -3.744536 -0.778322 -1.176002
C -2.572404 1.423690 -1.364982
H -4.368905 2.125739 0.577833
H -5.244485 1.471372 -0.817544
H -5.197237 0.565549 0.705025
H -4.377587 -0.536736 -2.033830
H -2.863884 -1.301502 -1.551356
H -4.293016 -1.448878 -0.514386
H -3.167001 1.651442 -2.253643
H -2.331069 2.359619 -0.860779
H -1.639482 0.962568 -1.694541

Anth_I_3Furan.log
Energy (E) = -878.741296415 Hartree
Enthalpy (H) = -878.476097 Hartree
Gibbs free energy (G) = -878.542296 Hartree
Charge = 0, Spin = 1
C 0.623284 4.143120 -0.777134
C -0.569063 3.822225 -0.212430
C -0.981729 2.464130 -0.082247
C -0.114334 1.417150 -0.540980
C 1.121908 1.802762 -1.139805
C 1.473812 3.110940 -1.256558
C -2.205388 2.140757 0.490167
C -0.557422 0.103030 -0.388538
C -1.806390 -0.242371 0.131306
C -2.641780 0.826152 0.599169
C -3.906980 0.520457 1.180592
H -4.513371 1.343650 1.537530
C -4.337434 -0.763155 1.279422
C -3.529381 -1.818986 0.776682
C -2.312805 -1.573983 0.220783
H -2.843142 2.938599 0.854124
H 0.925639 5.176521 -0.874803
H -1.240569 4.591452 0.148349
H 1.776389 1.040741 -1.536740
H 2.410918 3.371553 -1.729453
H -5.299233 -0.987249 1.719954
H -3.899792 -2.834238 0.817319
H -1.755901 -2.375097 -0.239562
I 0.797964 -1.474800 -0.800416
F -0.746568 -1.961438 -2.229793
C 2.051035 -0.731690 0.766999
C 3.725569 0.148724 1.938707
H 4.599008 0.609444 2.363294
O 2.811786 -0.326003 2.817248
C 1.806228 -0.861619 2.092380
C 3.317431 -0.068946 0.667660
H 3.846642 0.206137 -0.228863
H 0.986784 -1.288350 2.643984

AnthI8BA_B_3Furan.log
Energy (E) = -1084.60062883 Hartree
Enthalpy (H) = -1084.244323 Hartree
Gibbs free energy (G) = -1084.321075 Hartree
Charge = 0, Spin = 1
C -6.932068 -0.794684 0.035357
C -5.857701 -1.511056 0.460097
C -4.529716 -1.047826 0.223009
C -4.349136 0.190868 -0.473884
C -5.502370 0.911966 -0.902677
C -6.751955 0.436712 -0.657279
C -3.413425 -1.764155 0.647535
C -3.058684 0.655319 -0.712687
C -1.947152 -0.066604 -0.286003
C -2.122186 -1.300383 0.405587
C -0.965939 -2.013481 0.819566
H -1.115543 -2.951225 1.343286
C 0.321070 -1.603167 0.590621
C 0.442168 -0.349073 -0.085350
H -3.552558 -2.702719 1.172376
H -7.934235 -1.156831 0.220453
H -5.988640 -2.448618 0.986146
H -5.357579 1.846944 -1.429821
H -7.619716 0.991095 -0.987866
H -2.918099 1.592998 -1.238723
I 2.321013 0.593258 -0.489297
C -0.618129 0.383738 -0.507866
H -0.477168 1.330884 -1.013360
C 1.424744 -2.534509 1.039742
H 1.703770 -3.158734 0.188832
H 0.961193 -3.206514 1.761858
C 2.687258 -1.918913 1.666738
H 2.487919 -0.882575 1.946131
H 2.878423 -2.427808 2.610894
C 3.958148 -2.031400 0.801454
H 4.553654 -1.117593 0.884497
H 4.587862 -2.847495 1.146907
C 3.680462 -2.300419 -0.679355
O 2.815735 -1.513771 -1.252051
O 4.231778 -3.231338 -1.235222
C 1.532545 2.422964 0.254346
C 0.755215 4.027642 1.576754
H 0.373684 4.692932 2.329949
O 0.963630 4.566935 0.352239
C 1.088029 2.717322 1.583038
C 1.432692 3.580270 -0.442259
H 1.649579 3.844464 -1.462327
H 1.021391 2.036726 2.414868

FuranIBCMe2S_A_3Furan.log
Energy (E) = -985.080680953 Hartree
Enthalpy (H) = -984.868921 Hartree
Gibbs free energy (G) = -984.929921 Hartree
Charge = 0, Spin = 1
C 0.766098 2.972218 0.244140

C -0.260656 2.091509 0.239065
C 0.371517 0.818392 0.135500
C 1.709203 0.997524 0.074370
O 1.955044 2.327857 0.142610
H 0.807355 4.043410 0.322652
H -1.311304 2.308810 0.310827
I -0.467901 -1.077341 0.005998
S 2.221294 -1.627148 0.196995
C 2.848228 0.051577 -0.108732
C 3.379823 0.184331 -1.538365
H 3.740610 1.199426 -1.724307
H 4.202241 -0.516507 -1.680530
H 2.598255 -0.056602 -2.258705
C 3.960948 0.374002 0.888747
H 4.769721 -0.345341 0.762251
H 4.352554 1.379147 0.718014
H 3.592080 0.299305 1.910477
C -2.432250 -0.158063 -0.059305
C -4.324223 0.802274 -0.723948
H -5.170340 1.276299 -1.187903
O -4.457212 0.492961 0.587620
C -3.107080 0.426550 -1.179940
H -2.730168 0.547220 -2.181175
C -3.300789 -0.089006 0.976898
H -3.240507 -0.396098 2.006171

Indole_NMe_IBCONAc_A_3Furan.log
Energy (E) = -963.193168883 Hartree
Enthalpy (H) = -962.910723 Hartree
Gibbs free energy (G) = -962.984562
Hartree
Charge = 0, Spin = 1
C -1.272243 1.397567 -0.014833
C -0.819903 2.742038 -0.004448
C -1.697449 3.826599 -0.018343
C -3.048844 3.559208 -0.044030
C -3.519587 2.236552 -0.055899
C -2.656816 1.161363 -0.041644
C -0.063868 0.639586 0.003187
H -1.322566 4.841119 -0.009768
H -3.756561 4.376616 -0.055738
H -4.585639 2.056286 -0.076891
H -3.055930 0.159330 -0.049639
N 0.551314 2.769816 0.017059
C 1.015323 1.482681 0.020150
C 2.453866 1.063771 0.029810
O 3.349675 1.889122 0.067129
I 0.343734 -1.402625 0.004321
C 1.315147 4.006058 0.023925
H 1.054872 4.592138 0.905423
H 2.370239 3.762270 0.040779
H 1.080676 4.586612 -0.868440
N 2.517091 -0.293897 -0.004568
C 3.725191 -0.974087 -0.026344
C 3.555428 -2.483284 -0.026132
H 2.993805 -2.806033 -0.905487
H 4.535679 -2.949535 -0.038068
H 3.012384 -2.808938 0.863646
O 4.828006 -0.475863 -0.048634
C -1.698109 -1.981579 0.033159
C -3.741242 -2.562472 0.670630
H -4.680386 -2.821075 1.124823
O -3.694804 -2.619958 -0.682981
C -2.448281 -2.271038 -1.057011
H -2.244423 -2.255982 -2.112953
C -2.543712 -2.180962 1.170243
H -2.291314 -2.043531 2.207226

Me_Pyrrole_3I_3Furan.log
Energy (E) = -588.978860357 Hartree
Enthalpy (H) = -588.801155 Hartree
Gibbs free energy (G) = -588.858014
Hartree
Charge = 0, Spin = 1
C -1.175035 1.290088 -0.079675
C -1.216175 -0.078306 -0.003036
C -2.557486 -0.506968 0.057900
C -3.306956 0.640854 0.013881
N -2.470129 1.720098 -0.073454
H -0.351295 1.979041 -0.139935
H -2.882265 -1.529767 0.105566
H -4.375214 0.776299 0.031730
C -2.884006 3.106047 -0.062738
H -3.848867 3.198572 -0.556279
H -2.968731 3.489191 0.954126
H -2.158507 3.706355 -0.606912
I 0.433567 -1.351956 0.011192
F -1.107136 -2.851021 0.000124
C 1.715047 0.360913 0.027327
C 2.964909 2.091823 0.660523
H 3.516840 2.895823 1.113000
O 3.131634 1.928951 -0.673885
C 2.367023 0.876398 -1.041603
H 2.383105 0.612484 -2.084426
C 2.111293 1.162658 1.147872
H 1.796240 1.051526 2.171384

NaphIBMeUreaMe_3Furan.log
Energy (E) = -927.275923083 Hartree
Enthalpy (H) = -926.968977 Hartree
Gibbs free energy (G) = -927.040198
Hartree
Charge = 0, Spin = 1
C -2.627110 0.264625 -0.514538
C -2.940184 -0.984508 0.073817
C -1.899770 -1.829134 0.515745
C -0.566980 -1.490027 0.403006
C -0.300808 -0.214704 -0.165397
C -1.265301 0.629721 -0.613394
H -2.167343 -2.784633 0.946209
H -1.005448 1.598034 -1.023306
I 1.707480 0.416272 -0.232434
C 1.570591 -2.679485 0.082246
O 2.156174 -3.727304 0.322481
N 1.861837 -1.793959 -0.880613
C 3.027900 -2.130823 -1.671234
H 2.895299 -3.092063 -2.169745
H 3.175041 -1.366689 -2.437312
H 3.939336 -2.210271 -1.069153
N 0.441928 -2.322948 0.885910
C 0.077853 -3.314498 1.882952
H -0.560947 -2.848830 2.631888
H -0.440017 -4.174397 1.448947
H 0.988173 -3.681187 2.344257
C -4.302468 -1.356058 0.186479
C -5.291254 -0.525533 -0.263744
C -4.973471 0.721582 -0.847379
C -3.667145 1.107307 -0.969074
H -4.545343 -2.312231 0.633202
H -6.327618 -0.822053 -0.173668
H -5.767303 1.367707 -1.196129
H -3.408892 2.060583 -1.413632
C 1.091777 2.404913 0.330955
C 0.398651 4.196937 1.451218
H 0.028244 4.960812 2.110866
O 0.842276 4.615040 0.242021
C 1.256500 3.515247 -0.427308
H 1.640272 3.675597 -1.419774
C 0.526782 2.855888 1.568120
H 0.252820 2.254985 2.418433

NpthIBCMe2O_C_3Furan.log
Energy (E) = -817.845524836 Hartree
Enthalpy (H) = -817.552552 Hartree
Gibbs free energy (G) = -817.618458
Hartree
Charge = 0, Spin = 1
C -2.583727 -0.881554 0.092178
C -1.263237 -1.241431 0.021982
C -0.327857 -0.206895 -0.114467
C -0.645233 1.111446 -0.178694
H -3.346339 -1.643181 0.206264
H 0.111190 1.878771 -0.282387
O 0.496622 -2.733087 -0.497003
I 1.682041 -0.910915 -0.193295
C -2.416013 2.838259 -0.198843
C -3.741041 3.170743 -0.137710
C -4.721113 2.162898 0.003313
C -4.354761 0.847478 0.079263
C -2.991262 0.471535 0.019108
C -2.010358 1.485207 -0.120220
H -1.656855 3.603165 -0.306749
H -4.044596 4.207139 -0.196803
H -5.766100 2.438102 0.050410
H -5.102307 0.070973 0.185650
C -0.737065 -2.677426 0.119607
C -1.673723 -3.654854 -0.584118
H -1.212219 -4.641144 -0.550023
H -2.651588 -3.714064 -0.103287
H -1.798796 -3.364223 -1.626346
C -0.627044 -3.040040 1.607250
H -1.597378 -3.000879 2.106041
H -0.216837 -4.046865 1.690172
H 0.048805 -2.348439 2.115403
C 2.420250 1.096042 0.074317
C 3.143841 3.014416 0.945701
H 3.439712 3.883363 1.505411
O 3.431072 3.029540 -0.377733
C 2.527361 1.859171 1.284015
C 2.984518 1.860743 -0.891125
H 3.134999 1.713523 -1.946316
H 2.187375 1.576214 2.265729

NpthISO2NMe_D_3Furan.log
Energy (E) = -1267.82640691 Hartree
Enthalpy (H) = -1267.564952 Hartree
Gibbs free energy (G) = -1267.633265
Hartree
Charge = 0, Spin = 1
C -0.487105 3.266005 -0.955807
C 0.782479 3.583554 -0.579177
C 1.685743 2.587997 -0.145211
C 1.299471 1.209903 -0.082515
C -0.056702 0.953197 -0.433040
C -0.907541 1.927305 -0.869646
H 3.235255 4.039958 0.159894
H -1.176318 4.020203 -1.307550
H 1.133534 4.606982 -0.614056
C 2.996478 2.985330 0.214876
C 2.320928 0.289401 0.298046
H -1.924656 1.685099 -1.140190
C 3.581237 0.710717 0.622179
C 3.929391 2.072612 0.602683
H 4.314560 -0.043902 0.871647
H 4.930911 2.378407 0.869937
I -0.987962 -0.975068 -0.283171
S 2.107047 -1.470048 0.391524
O 1.398435 -1.736654 1.633813
O 3.407089 -2.091863 0.256449
N 1.203467 -1.672961 -0.910709

C 1.094825 -3.064172 -1.337474
H 0.603947 -3.089214 -2.310000
H 2.081275 -3.517823 -1.440878
H 0.514377 -3.686360 -0.640679
C -2.860507 -0.089098 0.249857
C -4.969639 0.615232 0.242526
H -5.986261 0.926648 0.084485
O -4.516973 0.671938 1.516887
C -3.239950 0.236909 1.508800
C -4.002066 0.153816 -0.582918
H -4.083356 -0.003145 -1.645205
H -2.728947 0.213616 2.454991

perF_CMe2O_3Furan.log
Energy (E) = -1061.12557925 Hartree
Enthalpy (H) = -1060.910546 Hartree
Gibbs free energy (G) = -1060.978631 Hartree
Charge = 0, Spin = 1
C 1.200838 2.447632 -0.223458
C 2.463991 2.000171 0.118737
C 2.701193 0.639267 0.241894
C 1.701241 -0.294074 0.027571
C 0.432321 0.184744 -0.238546
C 0.173180 1.531952 -0.397470
O 0.848286 -2.405787 -0.584362
I -1.051663 -1.353462 -0.273190
F -1.010627 2.012344 -0.758081
F 0.975975 3.741737 -0.392517
F 3.442135 2.871378 0.304648
F 3.933895 0.270838 0.590350
C 1.913682 -1.826341 0.059126
C 3.180586 -2.218632 -0.702954
H 4.094508 -1.912018 -0.200061
H 3.167216 -3.304568 -0.788227
H 3.154412 -1.798327 -1.707808
C 2.008790 -2.264321 1.524270
H 2.144404 -3.345359 1.553949
H 2.844684 -1.782782 2.034418
H 1.085196 -2.017080 2.052150
C -2.657812 -0.000979 0.146764
C -4.504090 1.228976 0.032326
H -5.382017 1.811848 -0.179744
O -4.193669 1.071052 1.342339
C -3.075139 0.318202 1.394922
C -3.603647 0.593845 -0.749571
H -3.596715 0.561258 -1.825244
H -2.693689 0.089078 2.374530

perF_NMeCO2_3Furan.log
Energy (E) = -1151.17703781 Hartree
Enthalpy (H) = -1150.989803 Hartree
Gibbs free energy (G) = -1151.058322 Hartree
Charge = 0, Spin = 1
C 1.063424 2.565578 -0.466694
C 2.267495 2.212292 0.120321
C 2.535828 0.894880 0.453882
C 1.598585 -0.128815 0.267117
C 0.378336 0.276666 -0.288726
C 0.123673 1.575299 -0.683688
I -1.093027 -1.177031 -0.541730
F -1.020305 1.900591 -1.277298
F 0.827245 3.818706 -0.824044
F 3.192145 3.136153 0.315037
F 3.751073 0.625751 0.921160
N 1.861054 -1.446300 0.591192
C 2.725282 -1.778027 1.718824
H 2.634393 -1.006843 2.480975
H 2.394399 -2.730915 2.120825
H 3.768494 -1.879354 1.425895
C 1.654620 -2.500889 -0.380805
O 2.219244 -3.553542 -0.169552
O 0.878304 -2.177562 -1.347607
C -2.600208 -0.021342 0.379929
C -4.365329 1.245635 0.806701
H -5.219244 1.895667 0.862492
O -3.953266 0.696040 1.976515
C -2.886577 -0.074552 1.704354
C -3.576188 0.841438 -0.212691
H -3.658361 1.125027 -1.246709
H -2.435990 -0.596110 2.530199

Py_PhI_3Furan.log
Energy (E) = -818.623614298 Hartree
Enthalpy (H) = -818.383879 Hartree
Gibbs free energy (G) = -818.449555 Hartree
Charge = 0, Spin = 1
C 1.212859 1.275095 0.358732
C -0.037056 1.179873 -0.233810
C -0.904960 2.249411 -0.325396
C -0.507282 3.485032 0.170764
C 0.749315 3.624152 0.740544
C 1.595425 2.527879 0.833916
C 2.121271 0.116657 0.546051
C 2.440556 -1.955796 1.450143
C 3.752681 -2.006330 1.006993
C 4.251733 -0.924502 0.293536
C 3.423613 0.157363 0.058467
H -1.876034 2.129213 -0.787034
H -1.175277 4.331922 0.098316
H 1.069480 4.584061 1.121585
H 2.565866 2.624665 1.304006
H 2.017431 -2.783120 2.007973
H 4.362673 -2.874491 1.212154
H 5.265154 -0.932459 -0.083891
H 3.749261 1.007853 -0.522806
N 1.634180 -0.917392 1.231266
I -0.630847 -0.638524 -1.116024
F 1.232122 -0.219692 -2.150046
C -2.303785 -0.661938 0.204815
C -4.326060 -0.562252 1.122076
H -5.355991 -0.481364 1.419260
O -3.442897 -0.870101 2.100249
C -2.220263 -0.930970 1.528973
C -3.689297 -0.419845 -0.064035
H -4.142689 -0.177907 -1.010198
H -1.382654 -1.149790 2.168880

PyIBCMe2O_D_3Furan.log
Energy (E) = -680.374840401 Hartree
Enthalpy (H) = -680.143264 Hartree
Gibbs free energy (G) = -680.203045 Hartree
Charge = 0, Spin = 1
C 0.636534 3.132459 -0.126666
C 2.011540 3.010150 0.019830
C 1.923522 0.722881 0.002729
C 0.549380 0.778768 -0.129929
H 0.173914 4.107090 -0.183334
H 2.633454 3.895150 0.087274
O 1.743367 -1.593141 -0.420153
I -0.378778 -1.127763 -0.168286
C -0.128608 1.974709 -0.189317
H -1.205524 2.016238 -0.279877
N 2.643595 1.841207 0.091984
C 2.598831 -0.640433 0.101594
C 3.899110 -0.636361 -0.694327
H 4.585211 0.125117 -0.326462
H 4.356364 -1.621039 -0.602088
H 3.684528 -0.449745 -1.746112
C 2.886129 -0.898307 1.585839
H 3.366437 -1.871992 1.682524
H 3.534604 -0.127563 2.004709
H 1.950915 -0.916914 2.151317
C -2.318434 -0.198034 0.021420
C -4.322967 0.676483 -0.408660
H -5.248722 1.087335 -0.769253
O -4.213180 0.537156 0.933395
C -2.994582 0.005764 1.177562
C -3.197939 0.242930 -1.022779
H -3.009914 0.231118 -2.083077
H -2.747772 -0.169970 2.209930

PyrroleNMeIBCMe2O_A_3Furan.log
Energy (E) = -681.565801210 Hartree
Enthalpy (H) = -681.310788 Hartree
Gibbs free energy (G) = -681.373398 Hartree
Charge = 0, Spin = 1
C 1.191286 2.722150 0.011982
C 0.015187 2.018474 -0.046099
C 0.414727 0.666872 -0.072160
C 1.770575 0.561403 -0.020832
H 1.366776 3.784953 0.030576
H -0.983281 2.419071 -0.067828
I -0.625882 -1.124546 -0.094217
O 1.524504 -1.740599 -0.243200
N 2.248348 1.843181 0.033814
C 3.635800 2.257824 0.034402
H 4.211096 1.671575 0.747527
H 4.083122 2.147627 -0.952905
H 3.681973 3.303791 0.328487
C 2.477721 -0.780994 0.042793
C 3.603128 -0.886333 -0.989066
H 4.446000 -0.228027 -0.769106
H 3.958120 -1.916653 -0.980893
H 3.216373 -0.662889 -1.983178
C 3.032062 -1.003214 1.455976
H 3.491629 -1.991339 1.492380
H 3.776604 -0.257713 1.746618
H 2.209361 -0.975514 2.171748
C -2.478375 -0.045766 -0.002899
C -4.306613 1.108940 -0.531777
H -5.135420 1.661486 -0.936162
O -4.339687 0.871823 0.801182
C -3.193363 0.571445 -1.081017
H -2.906456 0.605204 -2.118185
C -3.223675 0.172678 1.106027
H -3.091895 -0.093560 2.139913
